# Monitoring health inequalities when the socio-economic composition changes: are the slope and relative indices of inequality appropriate? Results of a simulation study

**DOI:** 10.1186/s12889-019-6980-1

**Published:** 2019-05-30

**Authors:** Françoise Renard, Brecht Devleesschauwer, Niko Speybroeck, Patrick Deboosere

**Affiliations:** 1 Department of Epidemiology and Public Health, Sciensano, Rue Juliette Wytsmanstraat 14, 1050 Brussels, Belgium; 2 Department of Veterinary Public Health and Food Safety, Ghent University, Merelbeke, Belgium; 3 Institute of Health and Society (IRSS), Université catholique de Louvain, Louvain-la Neuve, Belgium; 4 Interface Demography, Section Social Research, Vrije Universiteit Brussels, Brussels, Belgium

**Keywords:** Health inequality, Monitoring, Inequality indices, Relative index of inequality

## Abstract

**Background:**

The slope (SII) and relative (RII) indices of inequality are commonly recommended to monitor health inequality policies. As an upwards shift of the educational level distribution (ELD) can be part of those policies, we examine how such a shift affects the SII, the RII and the population attributable fraction (PAF).

**Methods:**

We simulated 632 distributions of 4 educational levels (ELs) by varying the share (*p1* to *p4*) of each EL, with constant mortality rates (MR) and calculated the corresponding RII, SII and PAF. Second, we decomposed the effect on the three indices of a change affecting both the ELD and the MRs, into the contributions of each component.

**Results:**

RIIs and SIIs sharply increase with *p4* at fixed *p1* values and evolve as reversed U-curves for *p1* changing in complement to *p4*. The RII reaches a maximum, at much higher *p4* values than the SII. PAFs monotonically decrease when *p4* increases.

**Conclusion:**

If improving the educational attainment is part of a policy, an upwards shift of EL should be assessed as a progress; however the RII, and to a lesser extent the SII, frequently translate an increased EL4 share as a worsening. We warn against the use of SII and RII for monitoring inequality-tackling policies at changing socio-economic structures. Rather, we recommend to complement the assessment of changes in absolute and relative pairwise differentials, with changes in PAF and in the socio-economic group shares.

**Electronic supplementary material:**

The online version of this article (10.1186/s12889-019-6980-1) contains supplementary material, which is available to authorized users.

## Background

Reducing socio-economic (SE) health inequalities, is an overarching public health goal [1, 2], necessating a careful monitoring to support inequality-tackling policies [3, 4]. However, answering the simple question “Have health inequalities increased or decreased?”, has revealed to be challenging [5–9]. As pointed out by several authors, the choice of an inequality measurement affects both magnitude and direction of inequality changes [5, 10], which has clear implications for policy assessment. It is therefore essential to understand what is precisely measured when using a particular measure of inequality.

One major issue when monitoring health inequality results from potential changes in the social composition of the population. As strategies for tackling health inequalities often include addressing the social determinants of health – for instance by promoting education, increasing poor people’s income, or improving living conditions [1] – shifts in their distribution should be considered when monitoring policies.

A wide variety of inequality indices affected by the SE distribution – sometimes called “population-level” or “summary” inequality indices – has been described [8, 10, 11]. These indices are presented as good options to monitor inequality changes, since they are said to “account” for shifts in the SE composition. However, the specific meaning of the expression “account for SE shifts” differs by indicator, and is not explicit for the users. In the context of policy monitoring, it would be crucial to clarify the expectations related to this “accounting”, and to verify if the chosen indicators meet those expectations. When an upwards shift of the SE distribution (for instance the educational level, EL) is part of an inequality-reducing strategy, relevant indicators should improve when there is a progress in this distribution and worsen when there is a deterioration.

In particular, two regression-based indices, the slope index of inequality (SII) and its relative counterpart, the relative index of inequality (RII), are often recommended [4, 12–16] and used for monitoring purposes, alone or combined with other indicators. The SII measures the gradient of health across multiple SE groups that can be naturally ordered, after rescaling the SE groups in accordance to the relative position of each level, while the RII expresses the ratio between the health outcome levels at the (theoretical) bottom and top of the SE hierarchy [11]. Two properties are claimed as advantages for those indicators, namely 1) they include information available from all SE levels, and 2) they account for changes in the SE composition of the population [11, 15]. Indeed, as the SE composition intervenes in the SII and RII computation, shifts in the SE composition will impact their value; however, this exact impact has never been fully described. To our knowledge, only a single concrete case has been published that calculated the impact of a sole change in the EL distribution on the SII and RII [8], but this case was based on a unique pattern of EL shift and did not show the complexity of this impact.

In this work, we examine how upwards shifts of the SE composition of the population influence the SII and the RII. The answer to this question is of high importance, allowing to assess if they meet the expectations for policy monitoring. The behaviour of the SII and RII will be compared with another inequality indicator, namely the “population attributable fraction (PAF)” sometimes called “population attributable risk” [11].

## Methods

We first generated a large number of hypothetical populations by varying the SE compositions at fixed rates of health outcomes and examined the changes in a set of inequality indicators; secondly, we varied both the SE composition and health outcome rates and decomposed the total effect into its two components.

### Health outcomes and socioeconomic position

We used the premature mortality rates in males (defined as under 75 years mortality) as health outcome and the educational level (EL) as indicator of socioeconomic position; we grouped the EL in 4 groups ranging from EL1 to EL4 and corresponding to the International Standard Classification of Education (ISCED) 1997 (*) categories 0–1 (EL1), 2 (EL2), 3–4 (EL3), 5–6 (EL4). The share of each group is given by p1 (proportion of people with the lowest EL) to p4 (proportion of people with the highest EL).

### Generation of hypothetic populations

We generated with SAS 9.3 a series of distributions of 4 ELs in the population by varying the proportion of EL1 (p1), EL2 (p2) and EL3 (p3) from 5 to 50%, the proportion of EL4 (p4) being calculated as 100 %  − (*p*1 + *p*2 + *p*3). Only combinations where all EL proportions were comprised between 5 and 50% were retained, providing 632 different combinations. The sas code can be found at https://github.com/brechtdv/RII-SII.

### Inequality measurement

#### Simple pairwise rate differences

Pairwise rate differences compare mortality rates between two ELs either on absolute or relative scales. Those indicators are not affected by the size of the EL groups. In this paper, we present both absolute and relative rate differences, with EL4 as reference group for the comparisons; absolute rate differences correspond to the difference in rate between each EL and EL4, while rate ratios are the ratio of the rate of each level to the EL4 rate.

#### Population-level inequality indices

The SII and the RII are regression-based indicators [8, 11] relying on a regression relating health outcomes with the relative position of social groups on the SE distribution. The relative educational position corresponds to the cumulative proportion of each EL after ordering them from lowest to highest. Each educational category, represented by the midpoint of its range or “ridit” [17], is attributed its EL-specific mortality rate. Some variations exist about the type of regression to use [18]: in this paper we refer to the SII definition based on a weighted least square regression [13, 19] with weights proportional to the population size of each group and the RII definition of Kunst-Machenbach, the RII ratio [11, 19]. The slope of the regression line is the SII, a measure of absolute inequality. It is given by:

1 $$ SII=\frac{\sum \limits_{i=1}^n{w}_i\ \left({y}_i-{\overline{y}}_w\right)\left({x}_i-{\overline{x}}_w\right)}{\sum \limits_{i=1}^n{w}_i{\left({x}_i-{\overline{x}}_w\right)}^2} $$

where *x*_*i*_ is the ridit, *y*_*i*_ the mortality rate, and *w*_*i*_ the frequency of each class *i* = {1,  … , *n*}, and ${\overline{x}}_w$ and ${\overline{y}}_w$ the frequency-weighted averages of *x*_*i*_ and *y*_*i*_.

The magnitude of the slope is mainly affected by the value of the rates, and by the position of the extreme EL groups (“leverage” effect of values of *x* distant from the mean). Intermediate group values have a weaker influence on the magnitude of the slope, but will inflate the variance of the slope if they are not increasing gradually, rendering the slope not significantly different from 0.

The RII is obtained by extrapolating the regression line towards the extreme (theoretical) positions of the x axis, i.e., 0 and 1. It is calculated as the ratio of the value at the bottom of the social hierarchy (corresponding to the intercept) to the value at the top of the hierarchy (corresponding to the intercept + slope). The RII is given by:

2 $$ RII=\frac{Intercept}{Intercept+ Slope} $$

More information about these measures can be found elsewhere [8, 11, 15]. SAS code to compute those indices and their confidence interval has been published by Cheng [19].

The PAF indicates the fraction of all deaths that could have been avoided if the mortality of the whole population was equal to the one observed in the highest EL. The PAF is calculated as [11, 20]:

3 $$ PAF=\frac{Rate\ in\ the\ total\ population- Rate\ in\ the\ highest\  EL\ }{Rate\ in\ the\ total\ population} $$

### Analysis

In the first part of our analysis, we examine the effect of changes in the educational distribution on the RII, SII and PAF, while keeping the group-specific mortality rates fixed. Therefore, in each of the 632 hypothetical distributions of the Els, we attributed to each EL the EL-specific premature mortality rates in Belgian males calculated from the census 2001 as the health outcome, i.e., 733.8, 552.1, 450.1, and 313.9 per 100,000 person-years, respectively, for EL1, EL2, EL3, and EL4 (authors’ calculation).

Among all possible changes, we focused on the situations where there was a change in p4 as this is the most common evolution.

We calculated the RII, SII and PAF for each simulated EL distribution, as well as their confidence intervals [21]. Only distributions where the 95% confidence interval for the SII and RII did not include respectively 0 and 1 were analyzed, leaving 495 useable EL distributions.

Finally, since our research question was how those indices behave when the share of EL4 increases, we plotted each index against p4, for two types of evolutions in p1:

- evolutions with fixed p1 values, with p2 or p3 varying in complement to p4;
- evolutions with p1 varying in complement to p4, with p2 and p3 fixed.

In the manuscript, the behaviour of the SII, RII and PAF is described in detail for 3 examples from evolutions a) and 3 examples from evolutions b). The full set of results for all combinations of p1 to p4 values is shown in appendices.

In the second part of our work, we explore what happens both to pairwise indicators and to population-level indices when varying two dimensions, i.e., the EL distribution (ELD) and the mortality rates. We built six fictive scenarios (A to F), by combining 3 sets of EL-specific mortality rates with 2 ELDs (Table 1), and computed the pairwise relative and absolute rate differences, the RII, SII and PAF for each scenario. The three sets of rates present a classical EL gradient with mortality decreasing when the EL increases. The first set of rates (rates 1) is considered as the baseline, the second set of rates (rates 2) and the third set of rates (rates 3) having respectively lower and higher mortality rates and mortality differentials than the baseline. We also used two ELDs: the first ELD (ELD1) is left skewed, including a large p1 (40%), and a small p4 (10%). The second ELD (ELD2) represents an upward shift compared to ELD1, with a smaller p1 (20%) and a larger p4 (30%).

**Table 1:** Variation of the Absolute Rate difference, the Rate ratio, the Relative Index of Inequality (RII), the Slope index of inequality (SII) and the Population-attributable Fraction (PAF) in various sets of EL-specific rates and 2 educational distribution of the population. Decomposition of the RII, SII and PAF changes into a part due to change in mortality differentials and a part due to the EL-shift

		Rates (1)				Rates (2)				Rates (3)			
		Scenario A		Scenario B		Scenario C		Scenario D		Scenario E		Scenario F	
		Rates (1)	EL Distrib (1)	Rates (1)	EL Distrib (2)	Rates (2)	EL Distrib (1)	Rates (2)	EL Distrib (2)	Rates (3)	EL Distrib (1)	Rates (3)	EL Distrib (2)
EL specific rates and share	EL1	750	40%	750	*20%*	*630*	40%	*630*	*20%*	750	40%	750	*20%*
	EL2	550	25%	550	*25%*	*500*	25%	*500*	*25%*	570	25%	570	*25%*
	EL3	450	25%	450	*25%*	*400*	25%	*400*	*25%*	420	25%	420	*25%*
	EL4	300	10%	300	*30%*	*290*	10%	*290*	*30%*	260	10%	260	*30%*
Pairwise inequality indices	Absol. Rates Diff				*Change from A*		*Change from A*		*Change from A*		*Change from A*		*Change from A*
	EL1 vs EL4	450		450	no change	340	*− 110*	340	*−110*	490	*40*	490	*40*
	EL2 vs EL4	250		250	no change	210	*−40*	210	*−40*	310	*60*	310	*60*
	EL3 vs EL4	150		150	no change	110	*−40*	110	*−40*	160	*10*	160	*10*
	Rate ratios												
	EL1 vs EL4	2.50		2.50	no change	2.17	*−13%*	2.17	*−13%*	2.88	*15%*	2.88	*15%*
	EL2 vs EL4	1.83		1.83	no change	1.72	*−6%*	1.72	*−6%*	2.19	*20%*	2.19	*20%*
	EL3 vs EL4	1.50		1.50	no change	1.38	*−8%*	1.38	*−8%*	1.62	*8%*	1.62	*8%*
					*Change from A due to EL shift †*		*Change from A due to change in mortality differentials*		*Change from A due to EL shift †*		*Change from A due to change in mortality differentials*		*Change from A due to EL shift †*
									*Total Change from A¥*				*Total Change from A¥*
Composite inequality indices	RII	2.86		3.75	31%*†*	2.40	−16%	3.03	22% *†* 6% *¥*	3.37	18%	4.96	56%*†* 73% *¥*
	SII	− 559		− 567	1%*†*	− 424	−24%	− 441	3% *†* − 21% *¥*	− 612	9%	− 619	1% † 11% *¥*
	PAF	0.48		0.39	−19%*†*	0.43	−10%	0.34	*−*19%*†* − 29% *¥*	0.54	13%	0.44	−21%† −8% *¥*

We further decomposed, for the 3 population-level indices, the total change from baseline into both components (Table 1), i.e. the partial change due to change in mortality differentials (calculated by comparing scenarios C and E to scenario A) and the partial change due to change in ELD (by comparing scenario B to A, D to C and F to E). All changes were expressed as percentages of the baseline scenario A:

$$ {\displaystyle \begin{array}{c} Change\  due\  to\ mortality\ differentials=\frac{\ \left[ Indices\ in\ scenario\ C\  or\ E- Baseline\ in dices(A)\right]\ }{Baseline\ in dices\left(\ A\right).}\\ {} Change\  due\  to\  ELD\  shift=\frac{\ \left[ Indices\ in\ scenario s\ with\  ELD2\left(B,D,F\right)- Indices\ in\ scenario s\ with\  ELD1\left(A,C,E\right)\right]\ }{Baseline\ in dices\left(\ A\right)}\end{array}} $$

All analyses were performed using SAS 9.3

## Results

### Impact of changes in the educational distribution on inequality indices, at fixed mortality rates

**Fig. 1:**
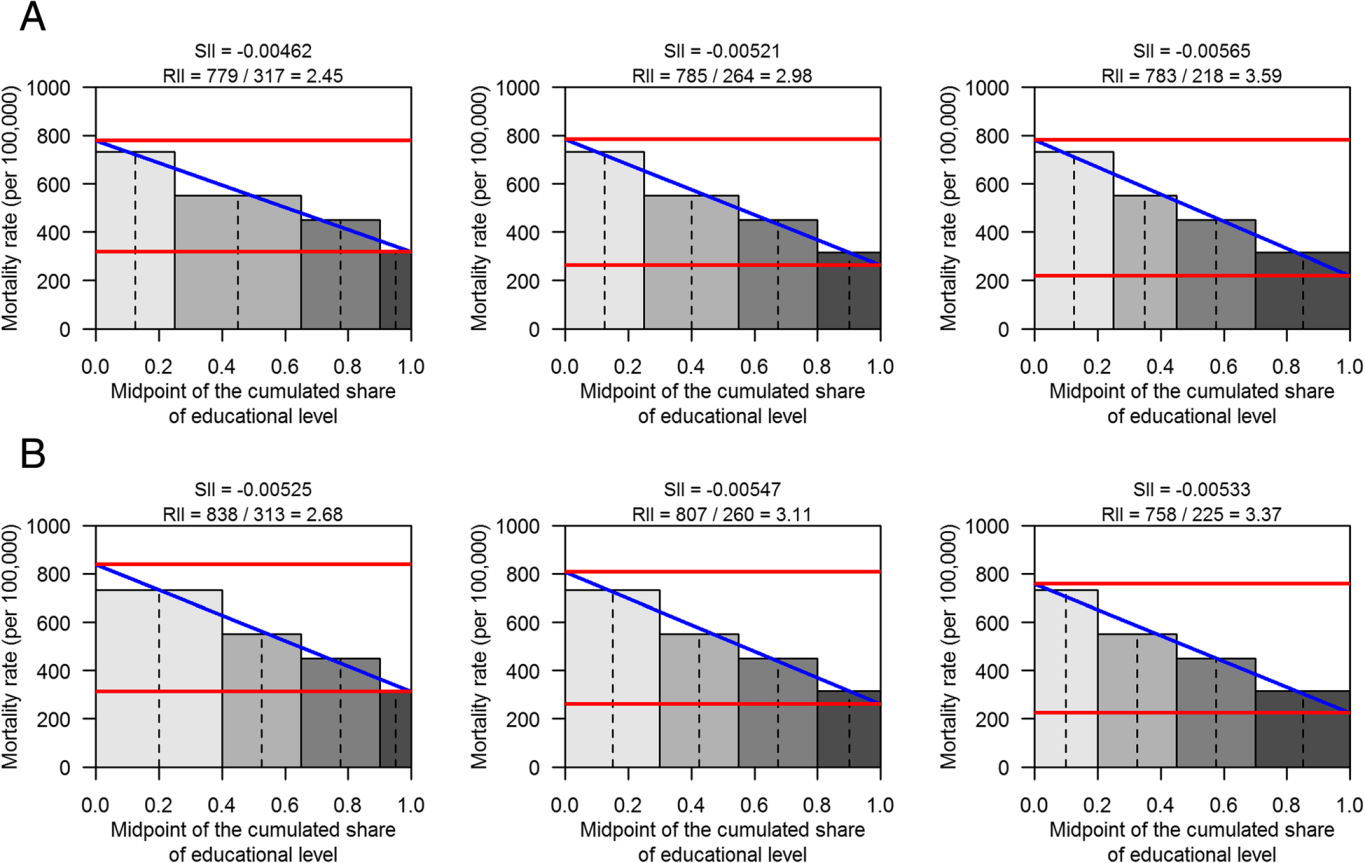
Construction of regression-based indicators when p4 varies: SII = slope index of inequality, RII = relative index of inequality; horizontal lines represent rates at intercept and at the top of the scale. Panel **a**: p1 and p3 fixed (0.25 each); p2 varying: 0.4 (left); 0.3 (mid); 0.2 (right); p4 = 1-(p1 + p2 + p3) = 0.1 (left); 0.2 (mid): 0.3 (right). Panel **b**: p2 and p3 fixed (0.25 each); p1 varying: 0.4 (left); 0.3 (mid); 0.2 (right); p4 = 1-(p1 + p2 + p3) = 0.1 (left); 0.2 (mid): 0.3 (right)

In this first part of the results, we kept the mortality rates constant and varied the share of p4, first for different fixed p1 and p3 values, then for fixed p2 and p3 values. Figure 1 illustrates the construction of the SIIs and RIIs for increasing values of p4, at given fixed values for p1 and p3 (panel A) or for p2 and p3 (panel B).

**Fig. 2:**
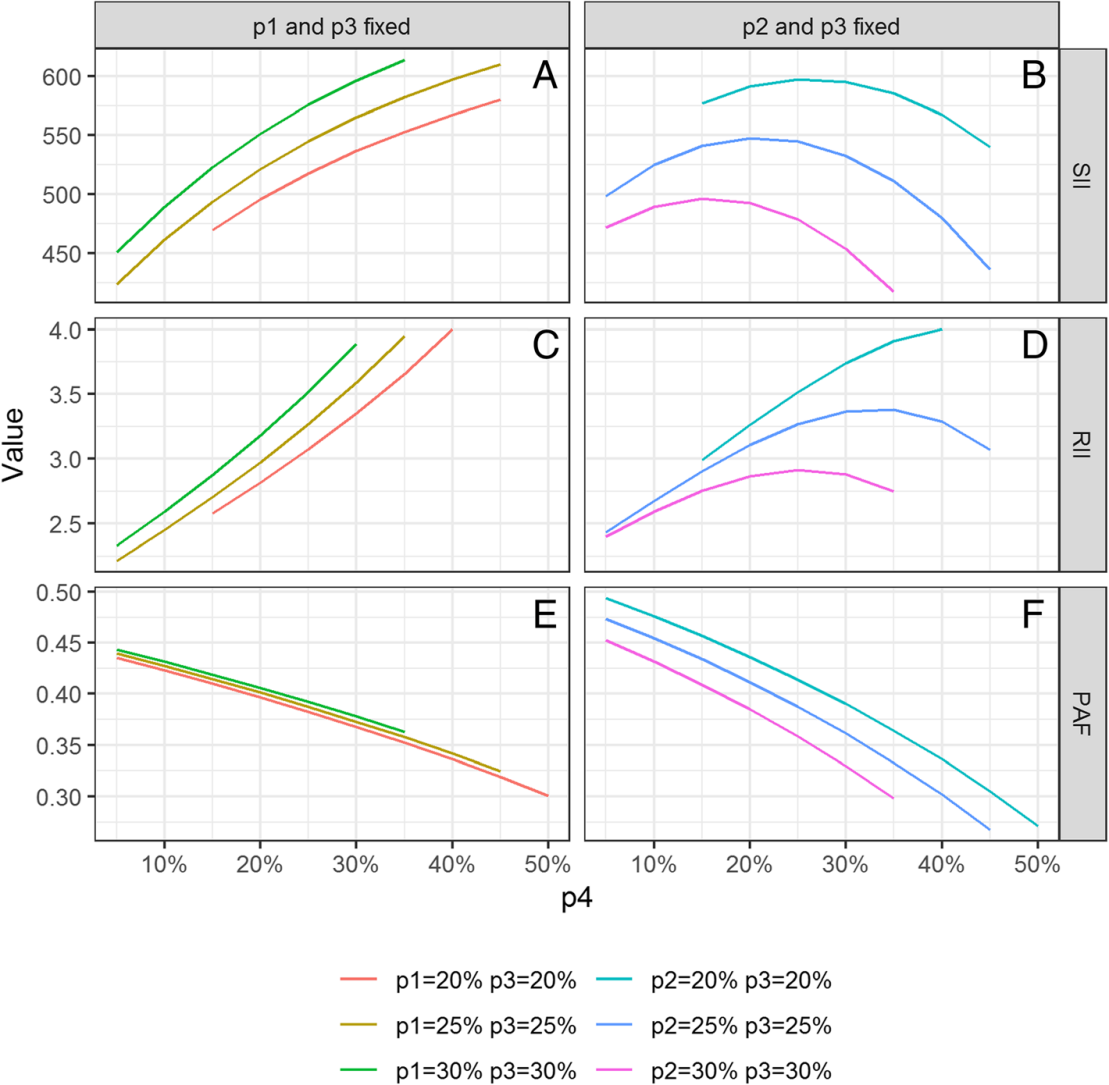
Variation of the SIIs, RIIs and PAFs, when the share of EL4 varies from 5 to 45%. Panel **a**: Variation of the SII when the share of EL4 varies from 5 to 45%, at different values of the share of EL1 and EL3. Panel **b**:Variation of the SII when the share of EL4 varies from 5 to 45%, at different values of the share of EL2 and EL3. Panel **c**: Variation of the RII when the share of EL4 varies from 5 to 45%, at different values of the share of EL1 and EL3. Panel **d**: Variation of the RII when the share of EL4 varies from 5 to 45%, at different values of the share of EL2 and EL3. Panel **e**:Variation of the PAF when the share of EL4 varies from 5 to 45%, at different values of the share of EL1 and EL3. Panel **f**: Variation of the PAF when the share of EL4 varies from 5 to 45%, at different values of the share of EL2 and EL3

Figure 2 plots the SIIs (in absolute value), RIIs and PAFs against the share of EL4 (p4) increasing from 5 to 50%. Panels A, C and E of Fig. 2 represent respectively the SIIs, RIIs and PAFs in three situations where p1 and p3 are fixed (each at 20, 25 and 30%), with p2 being the complement to 1 of (p1 + p3 + p4). Panels B, D and F of Fig. 2 represent the SIIs, RIIs and PAFs in three situations where p2 and p3 are fixed (at 20, 25 and 30%), with p1 being the complement to 1 of (p2 + p3 + p4).

When p1 is fixed, an increase of p4 results in a constant increase of the absolute value of the SIIs (Fig. 2 panel a, Additional files 1 and 2) and a still more important increase of the RIIs (Fig. 2 panel c, Additional files 3 and 4). The PAFs (Fig. 2 panel e, Additional files 5 and 6), constantly decrease.

When the value of p1 varies in complement to the one of p4, with fixed values of p2 and p3, the SIIs follow a concave curve, increasing first at low p4 values, reaching a maximum then decreasing (Fig. 2 panel b, Additional file 7). The lower the value of p2 + p3, the higher the value of p4 will be at the maximal SII. The RIIs (Fig. 2 panel d, Additional file 8) also show a concave curve, reaching a maximum at higher p4 values than the SIIs. For the set of rates used in our simulations, the maximum RII is reached when the sum of p2 + p3 + p4 = 85%. Again, the PAF, at varying values of p1 with fixed p2 and p3 (Fig. 2 panel f, Additional file 9), continuously decreases when p4 increases.

### Impact of simultaneous changes in the educational level distribution and mortality rates on inequality indices

Table 1 compares the pairwise indicators and population-level indices for six fictive scenarios combining three sets of mortality rates with two EL distributions. Scenario A (baseline scenario) reveals quite large absolute and relative pairwise mortality inequalities.

For each set of rates, when moving from ELD1 to ELD2 (that is, moving from scenario A to B, from scenario C to D, or from scenario E to F), pairwise rate differences do not change, as their calculation does not depend on the EL sizes. At the contrary, population-level indices are affected by the EL distribution, and this in a completely different way for each of them. In scenario B, where only the ELD (and not the rates) has changed from baseline, we observe a 31% increase of the RII, an almost stable SII and a 19% decrease of the PAF as compared to baseline. In scenarios C and D (second set of rates combined with respectively ELD1 and ELD2), absolute and relative pairwise rate differences are smaller than in the baseline scenario (A). The mortality decline in the low ELs exceeds the one of the high ELs in such an important way that it even results in a higher proportional decline in the low than in high ELs, leading to a decline in both types of rate differentials [22]. The simultaneous achievement of those three criteria – i.e., a decline of the mortality in each EL, and a decline in both absolute and relative rate differentials – represents a very favourable, although rarely met, evolution [23]. Population-level indices differ in scenarios C and D: in scenario C, combining smaller pairwise inequalities than in scenario (A), with a same EL distribution, all population-level indices decrease: the RII by 16%, the SII by 24% and the PAF by 10%. However, while scenario D is still more favourable than scenario C – as not only the mortality differentials decreased from A, but the ELD shifted upwards, classifying it as the best of all 6 scenarios – the regression-based inequality indices increased: the effect of an ELD improvement (upwards shift) was paradoxically translated into a 22% RII increase from (C) to (D). This effect was so strong that, when comparing scenario D to A, the partial effect due to a rates differentials decrease was not only cancelled but even exceeded: the RII of scenario D was 6% larger than in scenario A (that has worse rates and worse ELD). This 6% increase can be decomposed into a 16% decrease due to the reduction in mortality differentials, and a 22% increase due to the EL shift. The total change in SII in scenario D compared to scenario A was − 21%, decomposed into − 24% due to the decrease in mortality differentials and + 3% due to the EL shift. The total decline of the PAF from scenario A was 29% (− 10% due to change in differentials and − 19% due to the shift in EL).

Scenarios E and F (3rd set of rates) show an increase of both absolute and relative mortality differentials with respect to scenario A. In scenario E (with ELD1), the population-based indices increased compared to scenario A, consistent with the evolution of the pairwise rates differentials. When moving from scenario E to scenario F, it could be expected that the upwards shift of the EL distribution would partially compensate the increase in differentials. Yet on the contrary, the RII further increased with 56% from scenario E to F, resulting in a 73% increase from scenario A. The SII increased by 11% (9.5% due to the rates differentials, and 1.1% more due to the shift in EL), while the PAF decreased by 8% in total from scenario A: the 13% increase due to the rates differentials was exceeded by a 21% decrease due to the EL-shift, which is in line with a “compensation” of the inequalities brought by an upwards shift of EL.

## Discussion

The SII and the RII are often claimed to be good indicators for monitoring health inequalities, as they include information from all ELs, and they account for changes in the SE distribution [4, 14, 16, 24]. Although both statements are true, the exact effects of SE shifts on the SII and RII have hardly been studied. While recommending the use of those gradient-based indicators as appropriate to take into account the size of the SE groups, Regidor recognizes that changes/differences in the composition of those groups “can lead to bias” in the interpretation of disparities [16]. Only Keppel provides a single example of the way the RII and SII change when the share of the lowest EL varies [8], but other situations were not studied. Our study analyses for the first time how those indices behave when the share of the highest EL varies, in a large series of EL distributions.

### Summary of main findings

At fixed mortality rates, the SIIs and the RIIs increase with the increase of the share of EL4 (p4) when the share of EL1 (p1) is fixed. When p1 varies in complement to p4, the evolution of the SIIs and the RIIs follows a reverse U-curve with an initial increase, the reach of a maximum then a decrease; for the RIIs the increasing part of the curve is much longer than for the SIIs; the maximum is only reached when the sum of p2, p3 and p4 is large, then slowly decreases. On the contrary, the PAFs monotonically decrease when p4 increases.

When the mortality rates vary together with a change in the EL distribution, population-level indicators are affected by both components. For each given set of mortality rates, a p4 increase strongly modifies the partial effect on the RII evolution of the mortality differential component, in a paradoxical way: the effect of a decrease in the mortality differential component (resulting in a decrease of the RII) is diminished or even cancelled by the effect of the upward shift of the ELD. In other words, when a good scenario (mortality differential decreases) moves to an even better scenario (mortality differential decreases + upward shift of the ELD) the improvement is translated by the RII as a worsening. Also, an increase of the mortality differentials is not compensated by an improvement of the ELD. In our examples, the SIIs hardly change consequently to a p4 increase (1 to 3%). The PAFs substantially decrease (by about 20%).

### Mathematical and graphical explanation

When rates are maintained constant, changes in the SII or in the RII are exclusively attributable to the change in the EL distribution.

The SII, being the ratio of the weighted sum of the XY covariances (X = ridit, Y = rates) to the squared X variances (Eq. 1), will increase when the numerator (XY covariances) increases more, or decreases less than the denominator (squared X variances). There is no straightforward link between the SII and the mean EL nor the share of p4; rather, the link is very complex; there is therefore no reason for an increase of p4 to be always translated into a reduction of the slope. At the contrary, in our set of simulations, increases in p4 with fixed p1 led to SII increases, and increases in p4 with moving p1 led to various SII evolutions.

The RII (Eq. 2) is the ratio of the fitted rate at intercept to the fitted rate at the top of the scale (sum of slope and intercept). Since for adverse events, the slope will be negative, increases in the absolute value of the slope produces lower fitted values at the top of the scale, leading to RII increases. For situations where the SIIs remain constant, since the midpoint of the highest EL (ridit4) moves away from the top of the scale (moving to the left) when p4 increases, the slope has to be extended for a longer distance, resulting, for a same value of the slope, in a lower value of the mortality rate at the top of the scale. For situations where the SIIs decrease, the RIIs will start to decrease for p4 value exceeding the one producing the maximal value for the ratio intercept/(slope + intercept).

On the contrary, the PAFs express the difference between the average rate and the rate of the highest EL, related to the average rate. When the share of the highest EL increases, the average mortality rate decreases, and so does the difference between the average and highest rates, resulting in a smaller PAF.

### Strengths and limitations

We only performed simulations using EL as socio-economic indicator, and not, for instance, income or occupation. We chose for EL because a scenario in which the EL increases is a very realistic one, since developed countries’ governments have made large efforts to increase the educational attainment of their populations. The general conclusions about the behaviour of the two regression-based indicators, the SII and RII, are however independent of the nature of the socio-economic indicator. Our approach can indeed easily be extended to other socio-economic indicators, but this was beyond the scope of the current study.

Although this study is based on empirical findings, the large variety of ELDs included in the simulations allows to be quite confident to the conclusions drawn from the studied evolutions of ELDs. This study mainly focused on the change of the indicators in function of the share of the highest EL. Our conclusions can thus not be generalized to scenarios where the p4 is fixed. An example of the variation of other ELs with a fixed p4 was published by Keppler [8] and although it led to an apparently different conclusion, the divergence is easy to explain: for a fixed p4 and at fixed mortality rates, the RII will depend on the distance of p1 from the intercept axis. Even if our analyses are limited to changes in p4, our findings are sufficient to warn against using the RII in case the EL distribution changes.

### Interpretation and policy implication

Our results warn against the use of the RII in monitoring health inequalities in the context of a change in the distribution of the SE groups in the population.

Policies intended to reduce health inequalities have to address the social conditions contributing to unequal chance in health [1, 25]. In particular, increasing educational attainment levels has been recognized as a key strategy to reduce health inequalities [26]. In order to assess the implementation of this policy, it is not sufficient to look at the inequalities between ELs: the monitoring of equity-policies has to monitor both inequality between social groups and the distribution of those groups, especially when change in the distribution of the population is part of the pursuit of equity [25]. Therefore we need indicators capturing progress in a valid way, meaning indicators that can point out improvement or deterioration.

As stated by the WHO “The results of monitoring indicate whether policies, programmes and practices are accomplishing what they are designed to achieve.” [4]

However, in most situations studied here, a positive evolution consisting in an increasing proportion of the population moving to the highest educational level, translates in a deterioration of the RII. The SII, to a lesser extent than the RII, also translates many patterns of p4 increases as worsening of inequalities. Only the PAF, albeit a simpler population-level inequality indicator, translates an improvement of the EL distribution as a progress through a decrease of the indicator value. Although the PAF does not capture the gradient of the rates across the ELs, it provides policy-makers with operational information.

For monitoring purposes, we recommend to limit the use of gradient-based indicators RII and SII to situations where the EL composition does not change, for instance for comparing inequalities between populations with a same EL composition, or inequalities in different health outcomes in a given population and time. When the ELD changes, we rather recommend to compete the analysis of changes in absolute and relative rates differences and in EL-specific health outcomes, with changes in PAF and ELD, as done in recent trends analyses [20, 27]. Further research is needed about the integration of all those dimensions. Kjellson [9] and Blakely [7] paved the way towards this integration by mapping several dimensions, but the issue of the EL shift has still to be integrated.

## Conclusion

In contrast to what is commonly proposed, we warn against the use of the RII and SII for monitoring health inequalities or comparing populations with different educational distributions, because they do not translate upwards shifts of the educational distribution into a value indicating progress. The RII, and to a lesser extent the SII, increase in most patterns including an increase of the share of the highest EL. We recommend to use pairwise inequality indicators and PAFs to monitor health inequalities, in combination with a description of the shifts in the ELs.

## Additional files

Additional file 1: Full set of figures representing the evolution of the SII in function of P4 at fixed p1 and p2 (PDF 637 kb)

Additional file 2: Full set of figures representing the evolution of the SII in function of P4 at fixed p1 and p3 (PDF 616 kb)

Additional file 3: Full set of figures representing the evolution of the RII in function of P4 at fixed p1 and p2 (PDF 555 kb)

Additional file 4: Full set of figures representing the evolution of the RII in function of P4 at fixed p1 and p3 (PDF 524 kb)

Additional file 5: Full set of figures representing the evolution of the PAF in function of P4 at fixed p1 and p2 (PDF 476 kb)

Additional file 6: Full set of figures representing the evolution of the PAF in function of P4 at fixed p1 and p3 (PDF 478 kb)

Additional file 7: Full set of figures representing the evolution of the SII in function of P4 at fixed p2 and p3 (PDF 605 kb)

Additional file 8: Full set of figures representing the evolution of the RII in function of P4 at fixed p2 and p3 (PDF 517 kb)

Additional file 9: Full set of figures representing the evolution of the PAF in function of P4 at fixed p2 and p3 (PDF 484 kb)

## Abbreviations

EL: Educational level
ELD: Educational level distribution
MR: Mortality rates
P1, p2, p3 and p4: Respective proportions of educational levels 1, 2, 3 and 4
PAF: Population attributable fraction
RII: Relative index of inequality
SII: Slope index of inequality
WHO: World Health Organization
