# Supplementary material for: Monitoring health inequalities when the socio-economic composition changes: are the slope and relative indices of inequality appropriate? Results of a simulation study
Source: BMC Public Health. 2019 May 30;19:662. doi: 10.1186/s12889-019-6980-1 (PMC6543610; doi:10.1186/s12889-019-6980-1)

SII in function of the share of EL4

When EL1 and EL3 are fixed at: EL1=5% ; EL3 =15%
EL2 =1- EL4 - EL1 - EL3

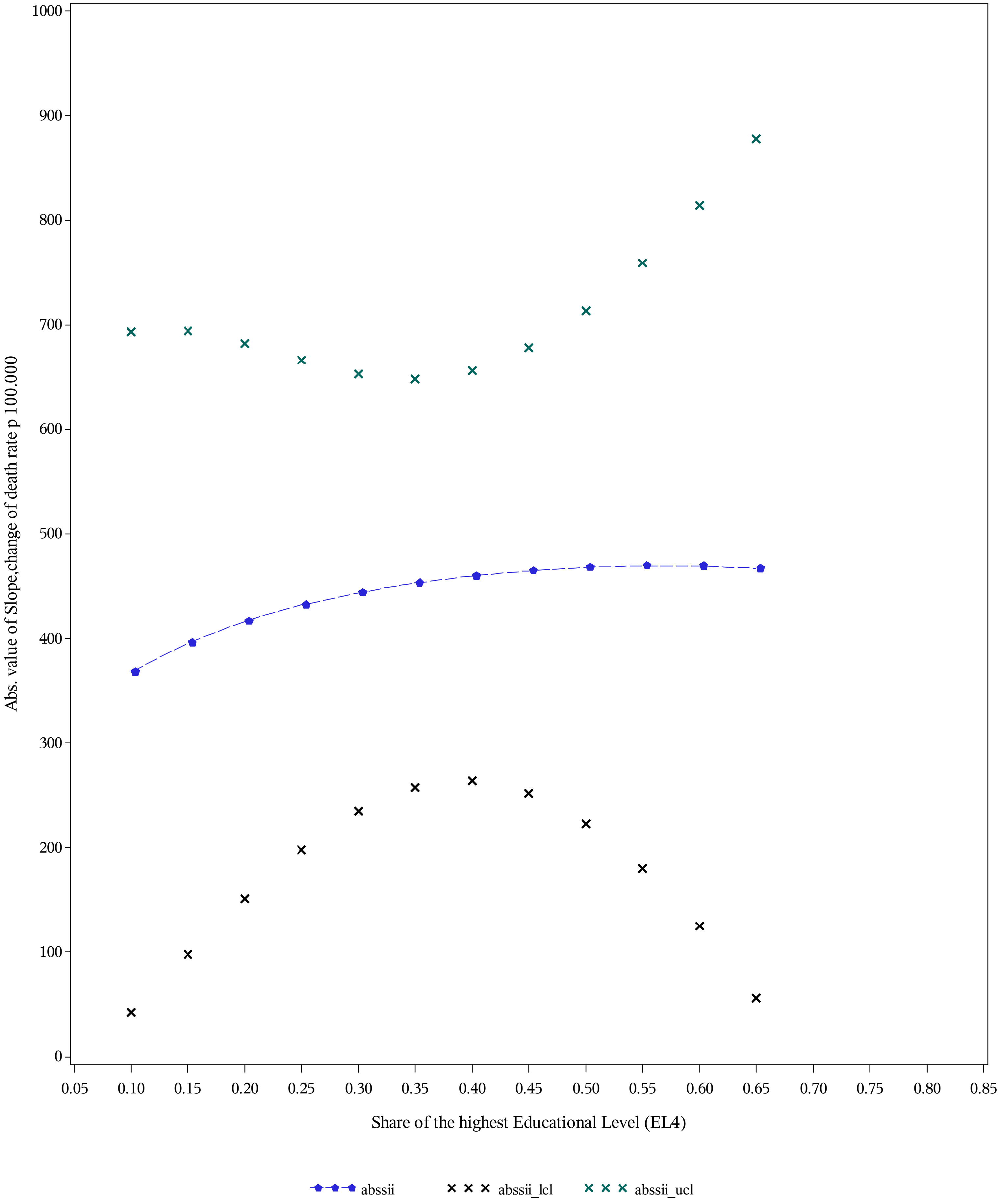

SII in function of the share of EL4

When EL1 and EL3 are fixed at: EL1=5% ; EL3 =20%
EL2 =1- EL4 - EL1 - EL3

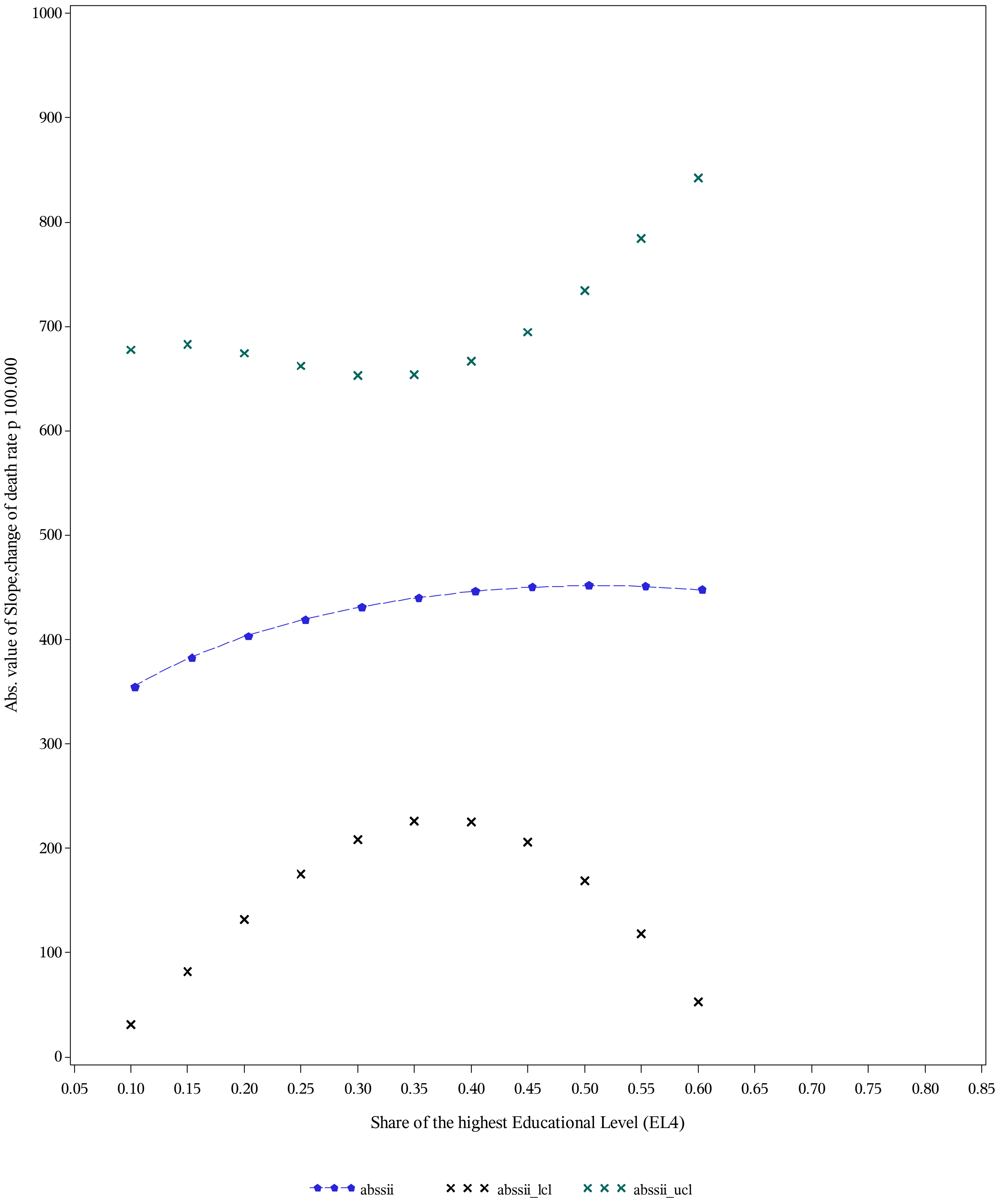

SII in function of the share of EL4

When EL1 and EL3 are fixed at: EL1=5% ; EL3 =25%
EL2 =1- EL4 - EL1 - EL3

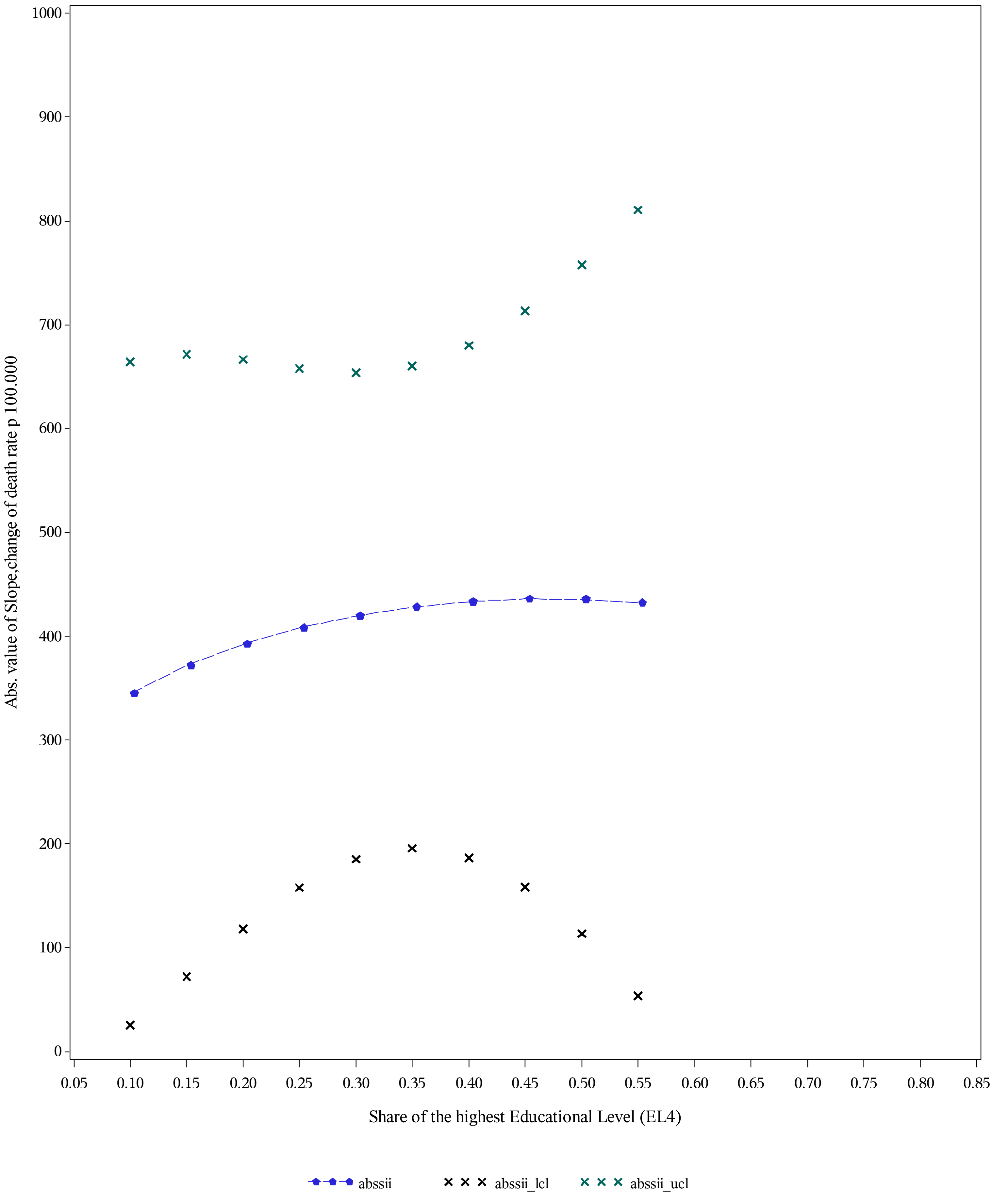

SII in function of the share of EL4

When EL1 and EL3 are fixed at: EL1=5% ; EL3 =30%
EL2 =1- EL4 - EL1 - EL3

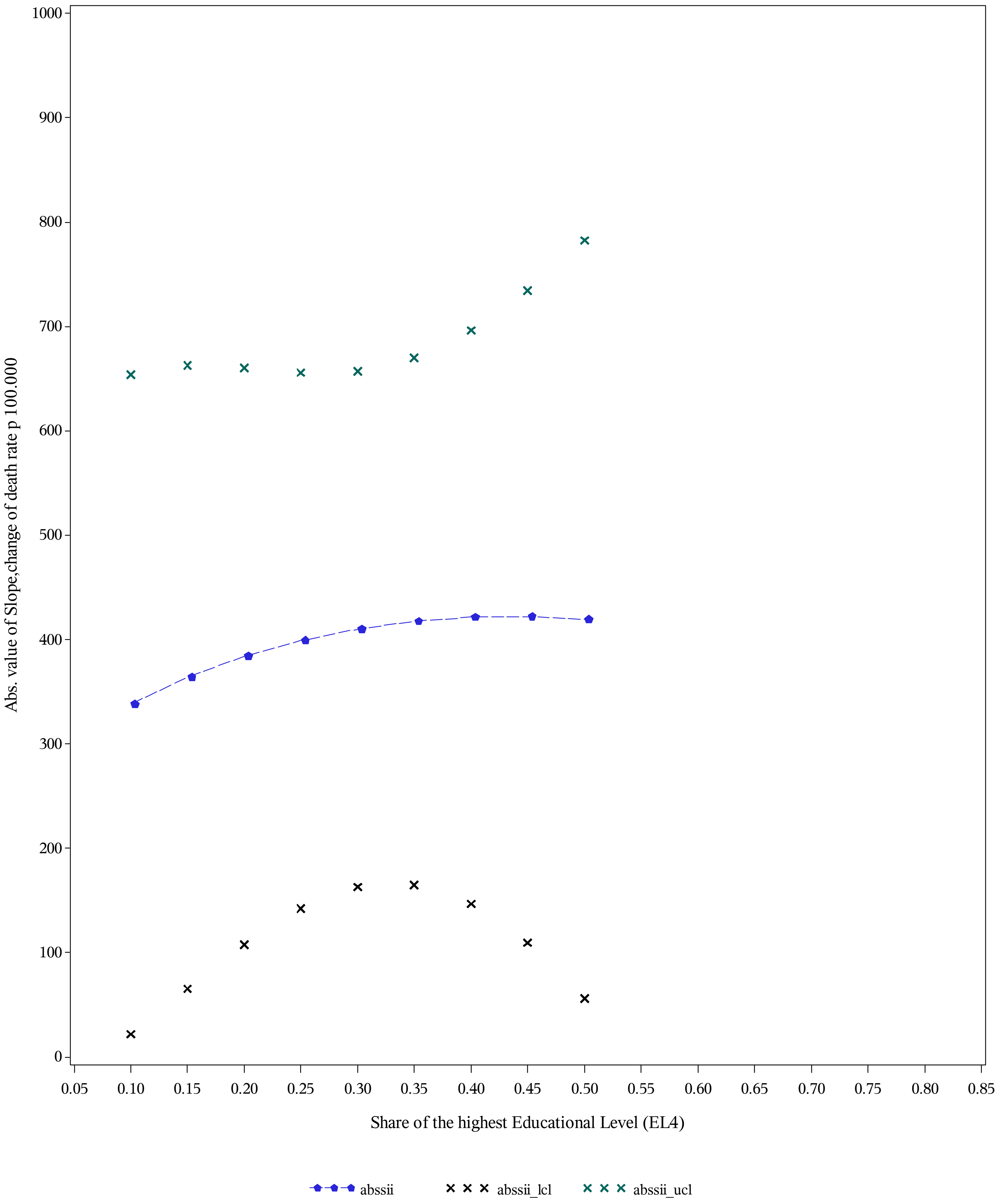

SII in function of the share of EL4

When EL1 and EL3 are fixed at: EL1=5% ; EL3 =35%
EL2 =1- EL4 - EL1 - EL3

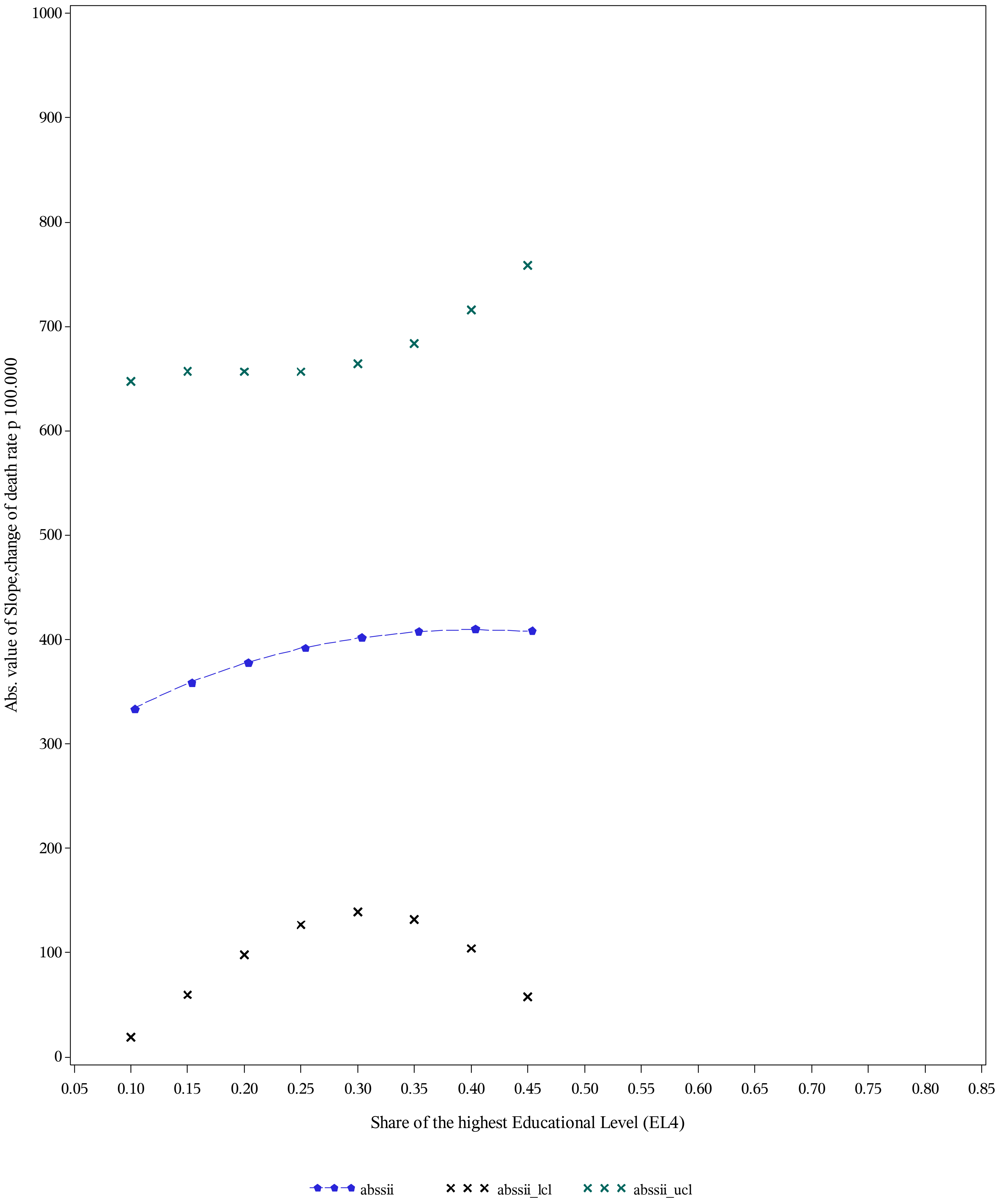

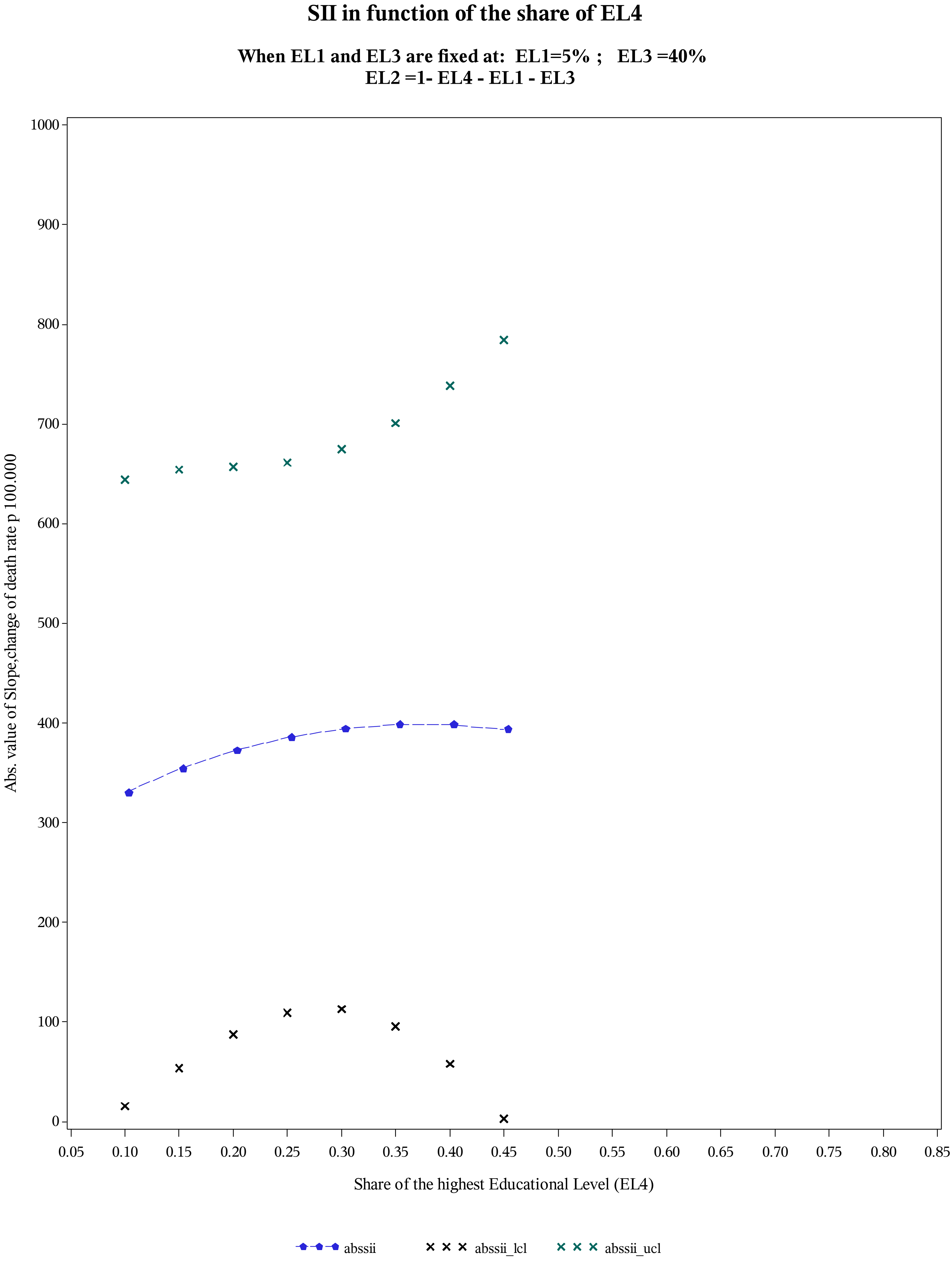

SII in function of the share of EL4
When EL1 and EL3 are fixed at: EL1=5% ; EL3 =40%
EL2 =1- EL4 - EL1 - EL3
Abs. value of Slope,change of death rate p 100.000
0
100
200
300
400
500
600
700
800
900
1000
0.05
0.10
0.15
0.20
0.25
0.30
0.35
0.40
0.45
0.50
0.55
0.60
0.65
0.70
0.75
0.80
0.85
Share of the highest Educational Level (EL4)
abssii
abssii_lcl
abssii_ucl

SII in function of the share of EL4

When EL1 and EL3 are fixed at: EL1=5% ; EL3 =45%
EL2 =1- EL4 - EL1 - EL3

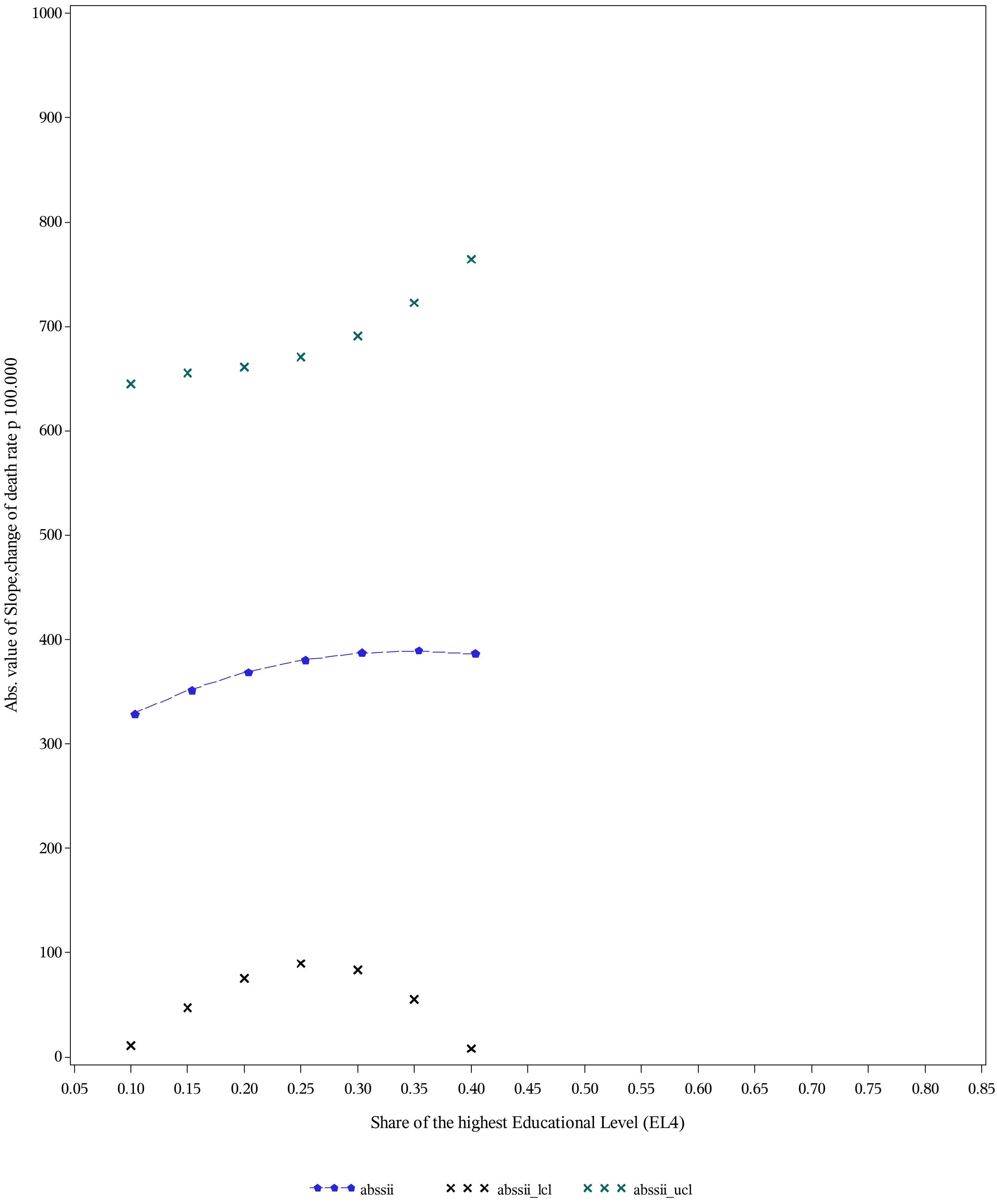

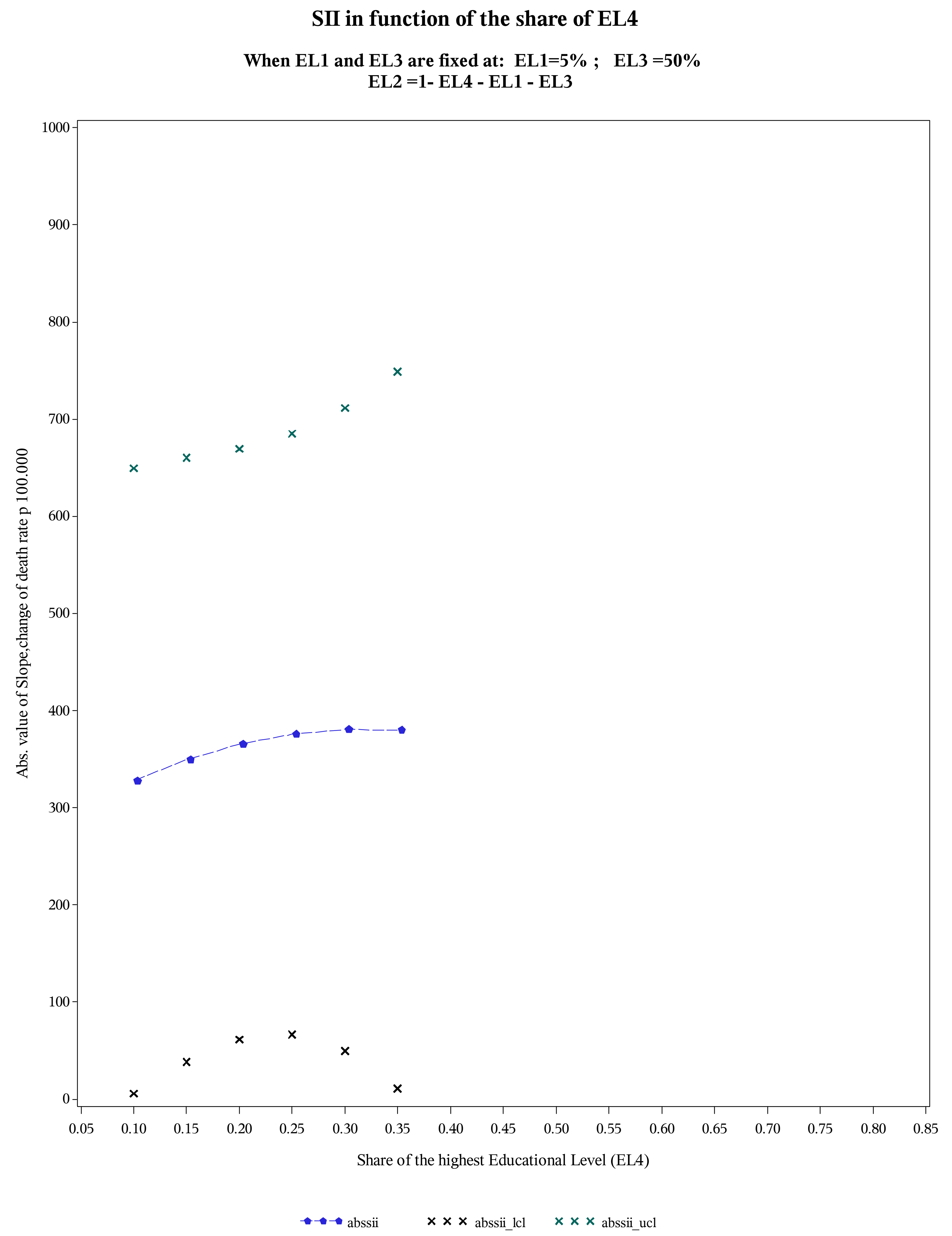
SII in function of the share of EL4
When EL1 and EL3 are fixed at: EL1=5% ; EL3 =50%
EL2 =1- EL4 - EL1 - EL3
Abs. value of Slope,change of death rate p 100.000
0
100
200
300
400
500
600
700
800
900
1000
0.05
0.10
0.15
0.20
0.25
0.30
0.35
0.40
0.45
0.50
0.55
0.60
0.65
0.70
0.75
0.80
0.85
Share of the highest Educational Level (EL4)
abssii
abssii_lcl
abssii_ucl

SII in function of the share of EL4

When EL1 and EL3 are fixed at: EL1=5% ; EL3 =55%
EL2 =1- EL4 - EL1 - EL3

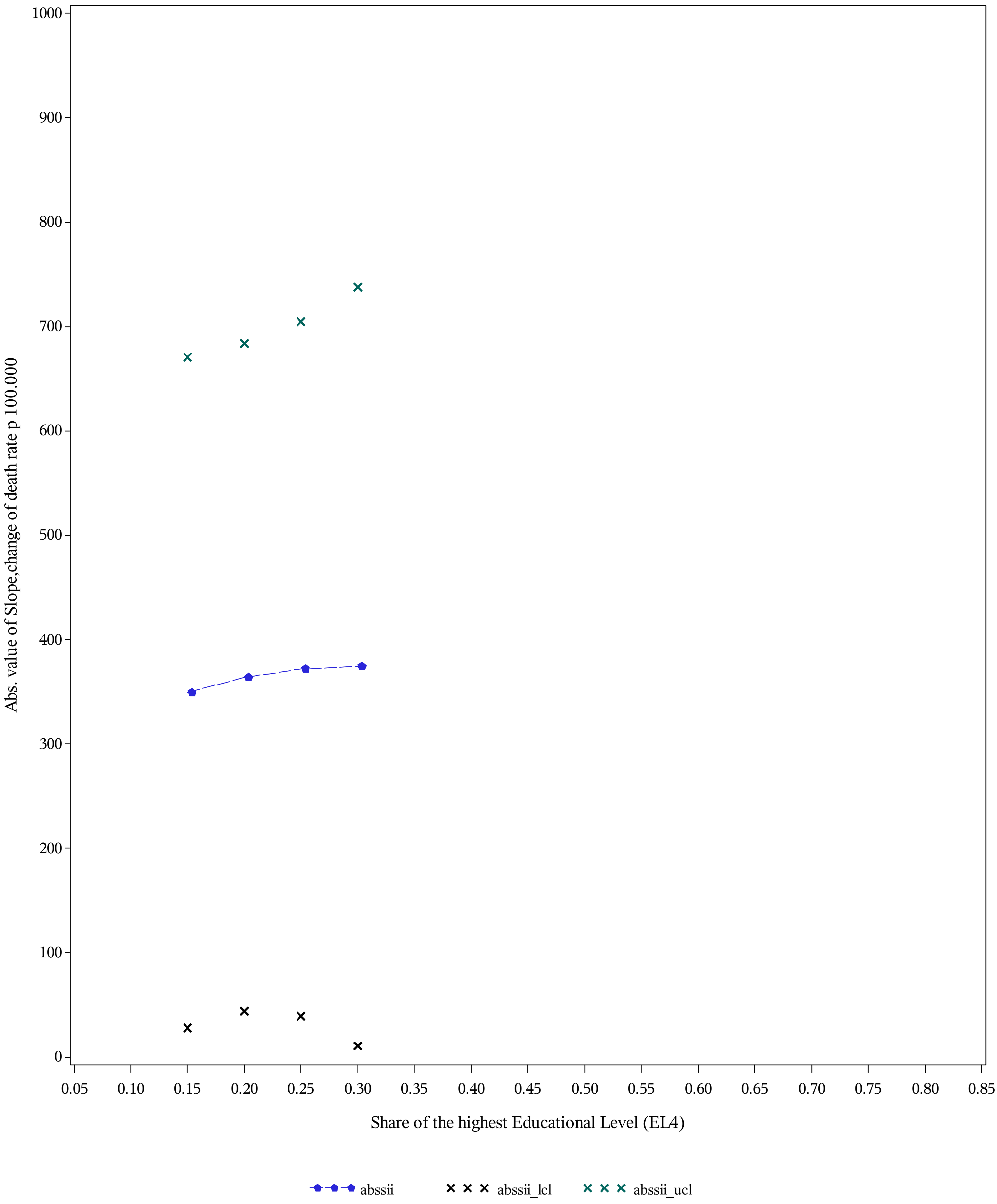

SII in function of the share of EL4

When EL1 and EL3 are fixed at: EL1=5% ; EL3 =60%
EL2 =1- EL4 - EL1 - EL3

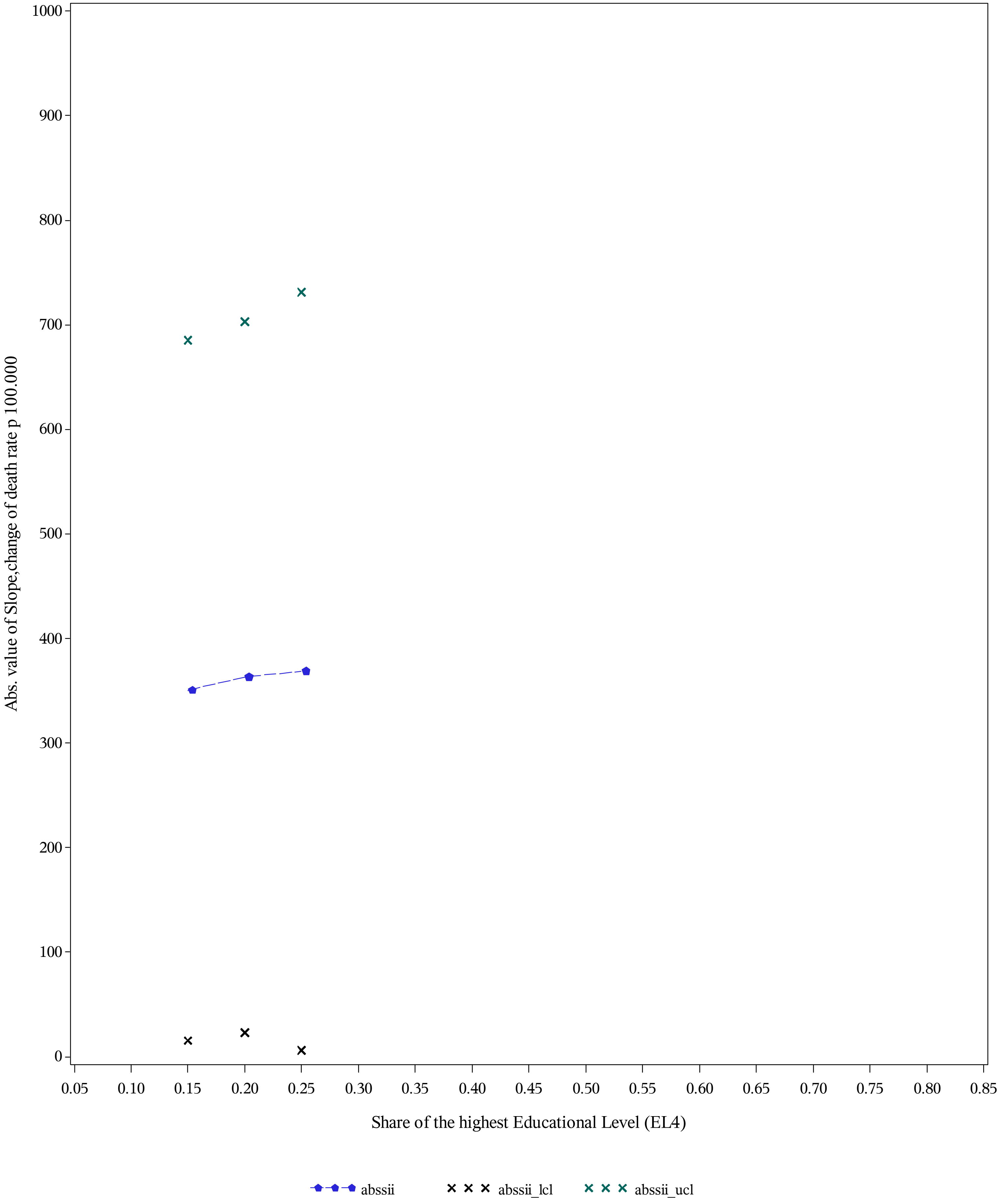

SII in function of the share of EL4

When EL1 and EL3 are fixed at: EL1=10% ; EL3 =5%
EL2 =1- EL4 - EL1 - EL3

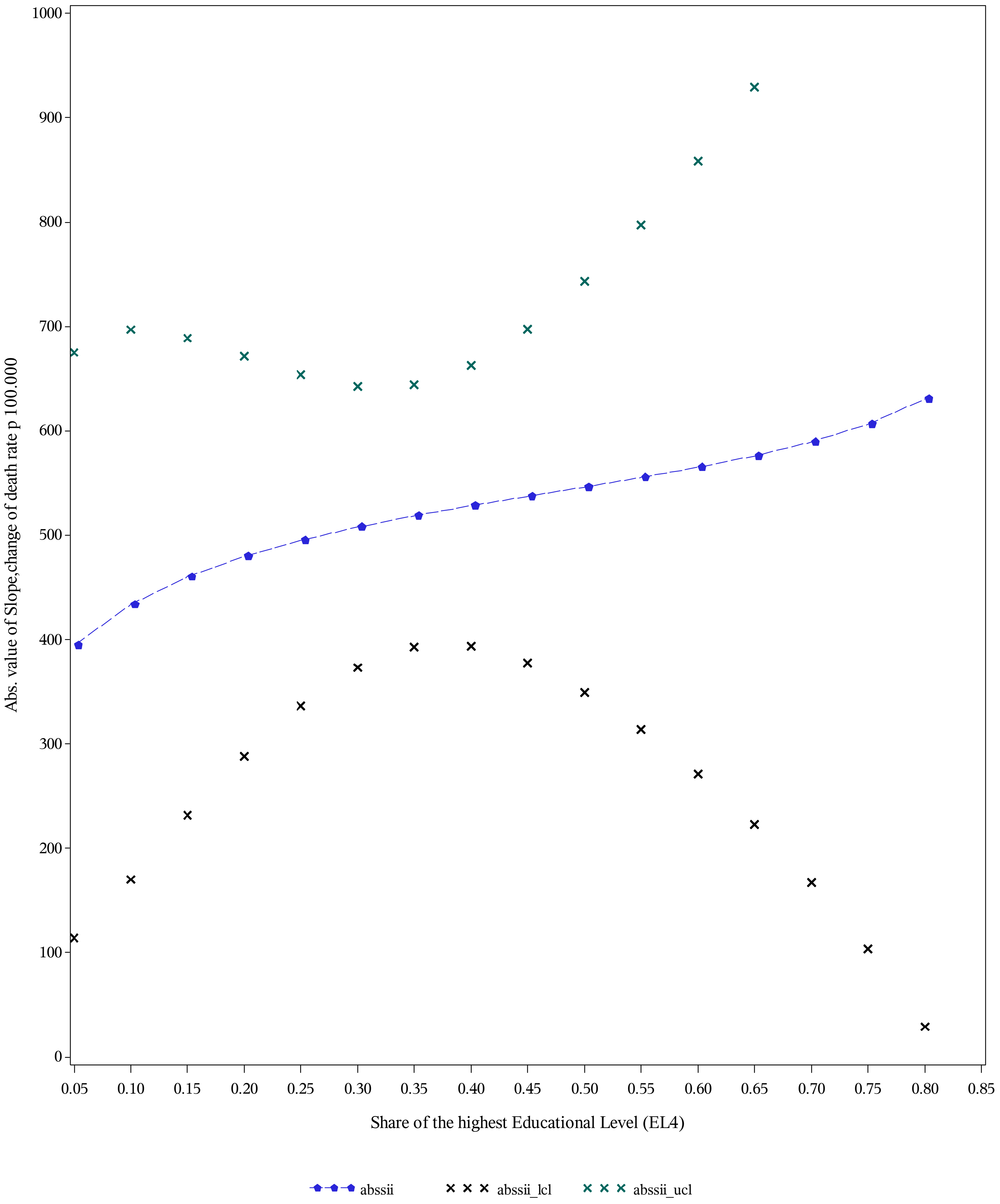

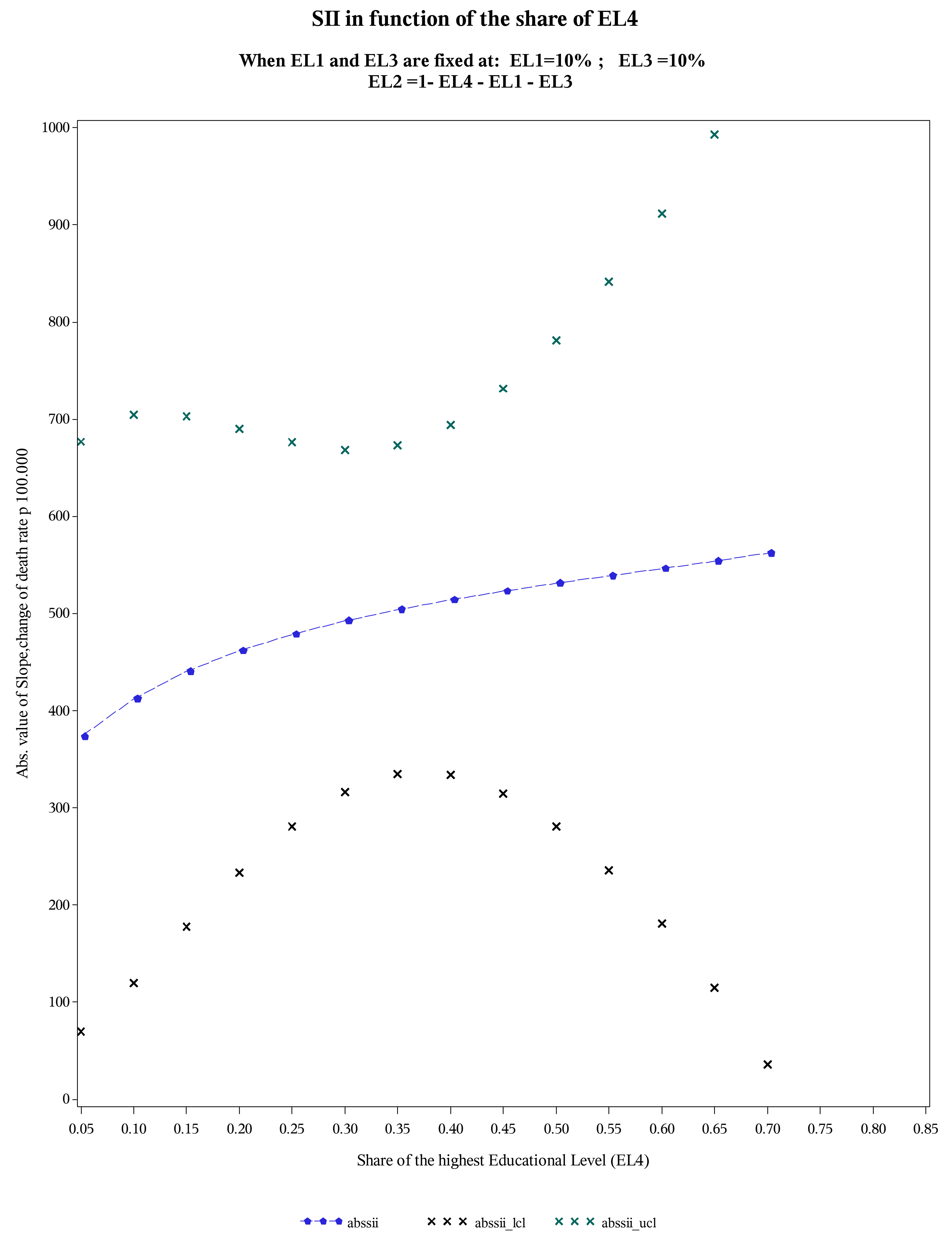
SII in function of the share of EL4
When EL1 and EL3 are fixed at: EL1=10% ; EL3 =10%
EL2 =1- EL4 - EL1 - EL3
1000
900
800
700
600
500
400
300
200
100
0
Abs. value of Slope,change of death rate p 100.000
0.05
0.10
0.15
0.20
0.25
0.30
0.35
0.40
0.45
0.50
0.55
0.60
0.65
0.70
0.75
0.80
0.85
Share of the highest Educational Level (EL4)
abssii
abssii_lcl
abssii_ucl

SII in function of the share of EL4

When EL1 and EL3 are fixed at: EL1=10% ; EL3 =15%
EL2 =1- EL4 - EL1 - EL3

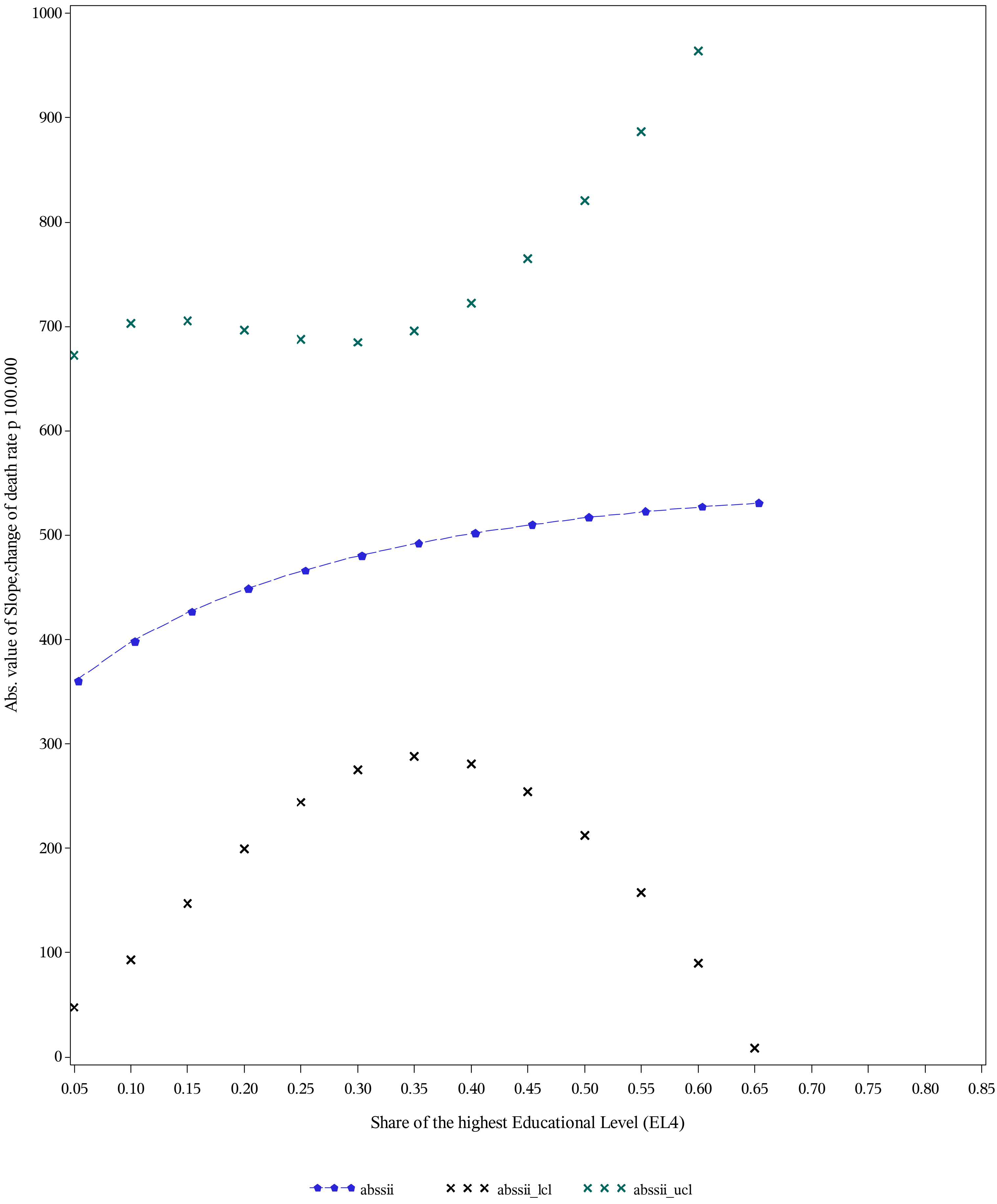

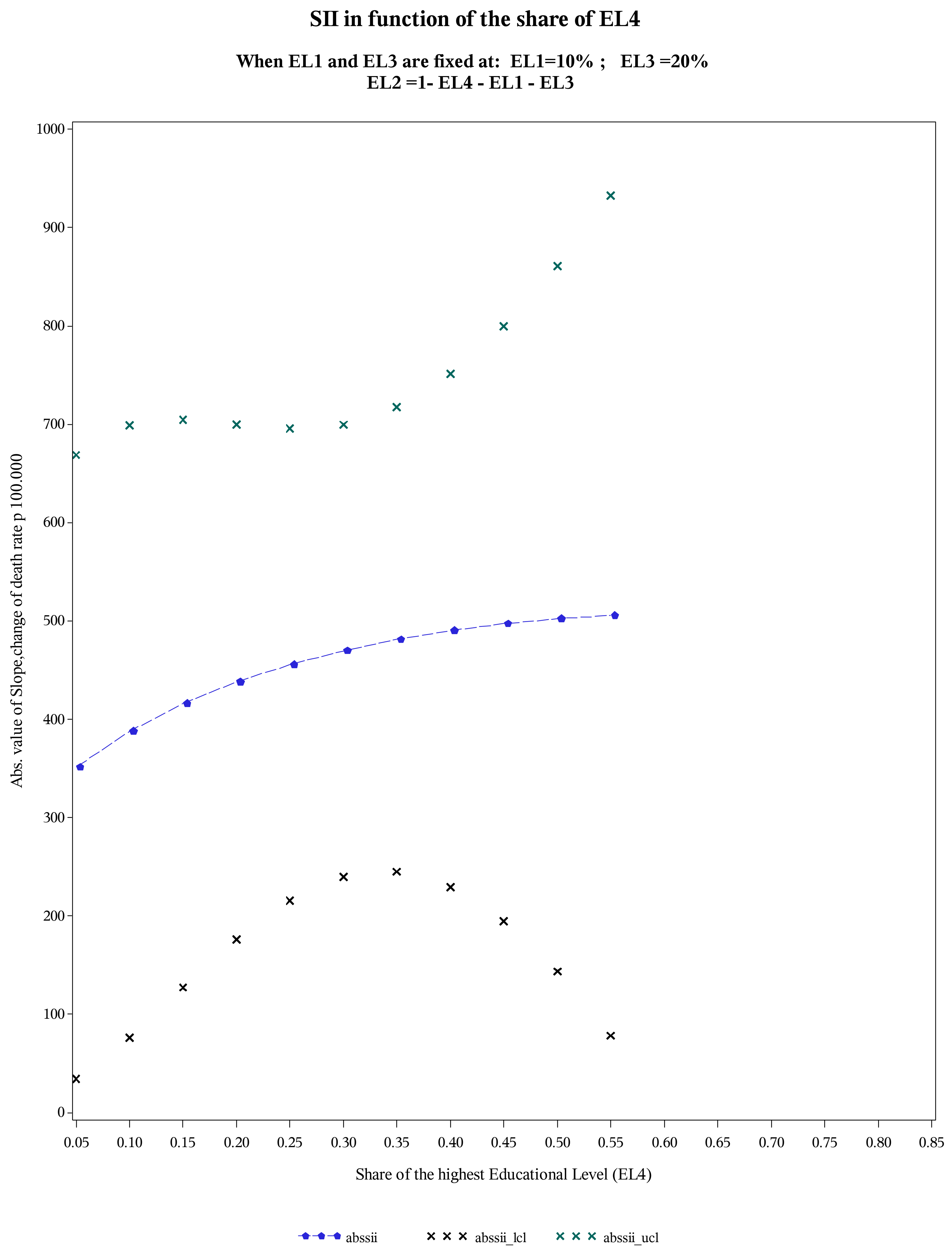

SII in function of the share of EL4
When EL1 and EL3 are fixed at: EL1=10% ; EL3 =20%
EL2 =1- EL4 - EL1 - EL3
Abs. value of Slope,change of death rate p 100.000
0
100
200
300
400
500
600
700
800
900
1000
0.05
0.10
0.15
0.20
0.25
0.30
0.35
0.40
0.45
0.50
0.55
0.60
0.65
0.70
0.75
0.80
0.85
Share of the highest Educational Level (EL4)
abssii
abssii_lcl
abssii_ucl

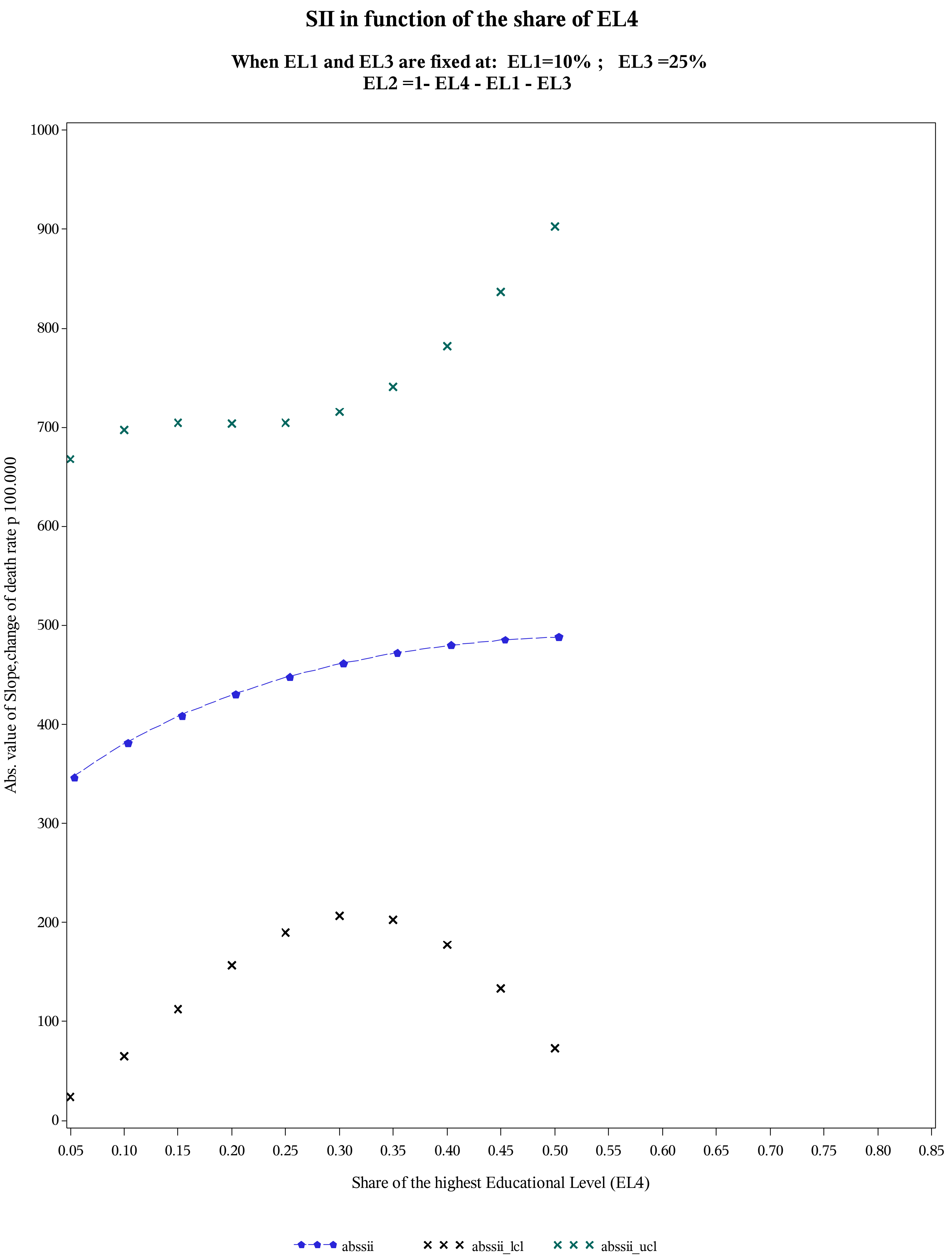
SII in function of the share of EL4
When EL1 and EL3 are fixed at: EL1=10% ; EL3 =25%
EL2 =1- EL4 - EL1 - EL3
Abs. value of Slope,change of death rate p 100.000
Share of the highest Educational Level (EL4)
abssii
abssii_lcl
abssii_ucl

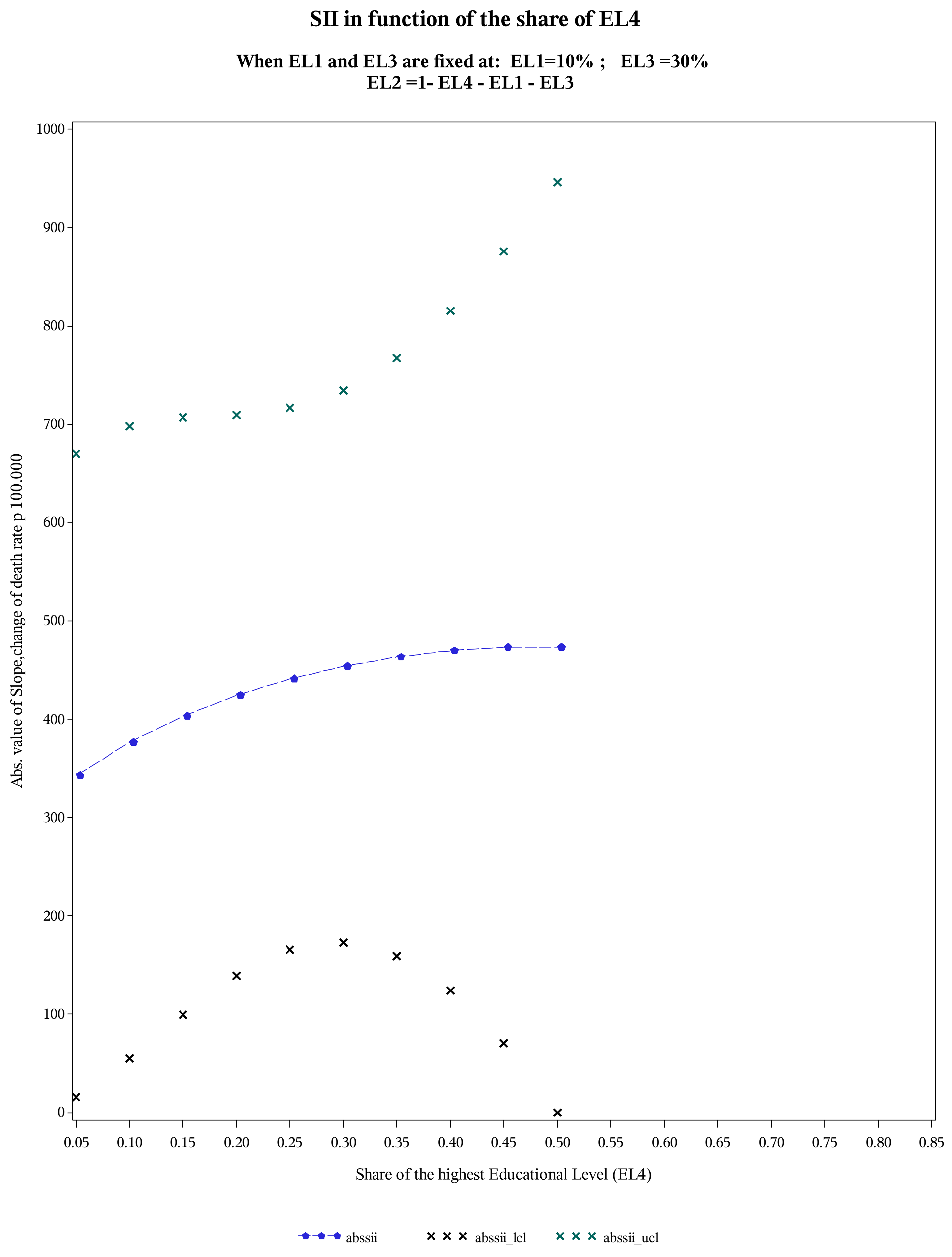

SII in function of the share of EL4
When EL1 and EL3 are fixed at: EL1=10% ; EL3 =30%
EL2 =1- EL4 - EL1 - EL3
Abs. value of Slope,change of death rate p 100.000
1000
900
800
700
600
500
400
300
200
100
0
0.05
0.10
0.15
0.20
0.25
0.30
0.35
0.40
0.45
0.50
0.55
0.60
0.65
0.70
0.75
0.80
0.85
Share of the highest Educational Level (EL4)
abssii
abssii_lcl
abssii_ucl

SII in function of the share of EL4

When EL1 and EL3 are fixed at: EL1=10% ; EL3 =35%
EL2 =1- EL4 - EL1 - EL3

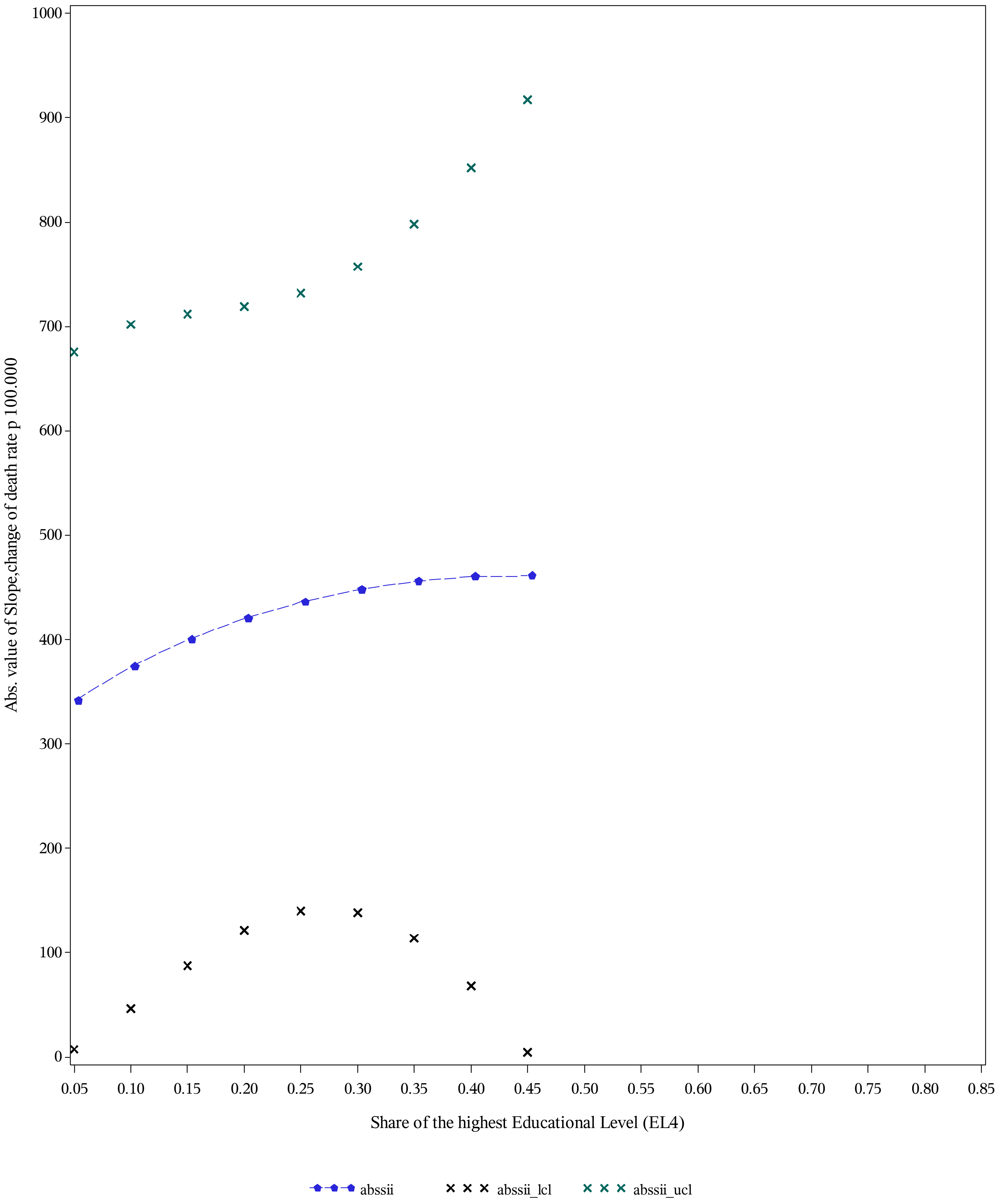

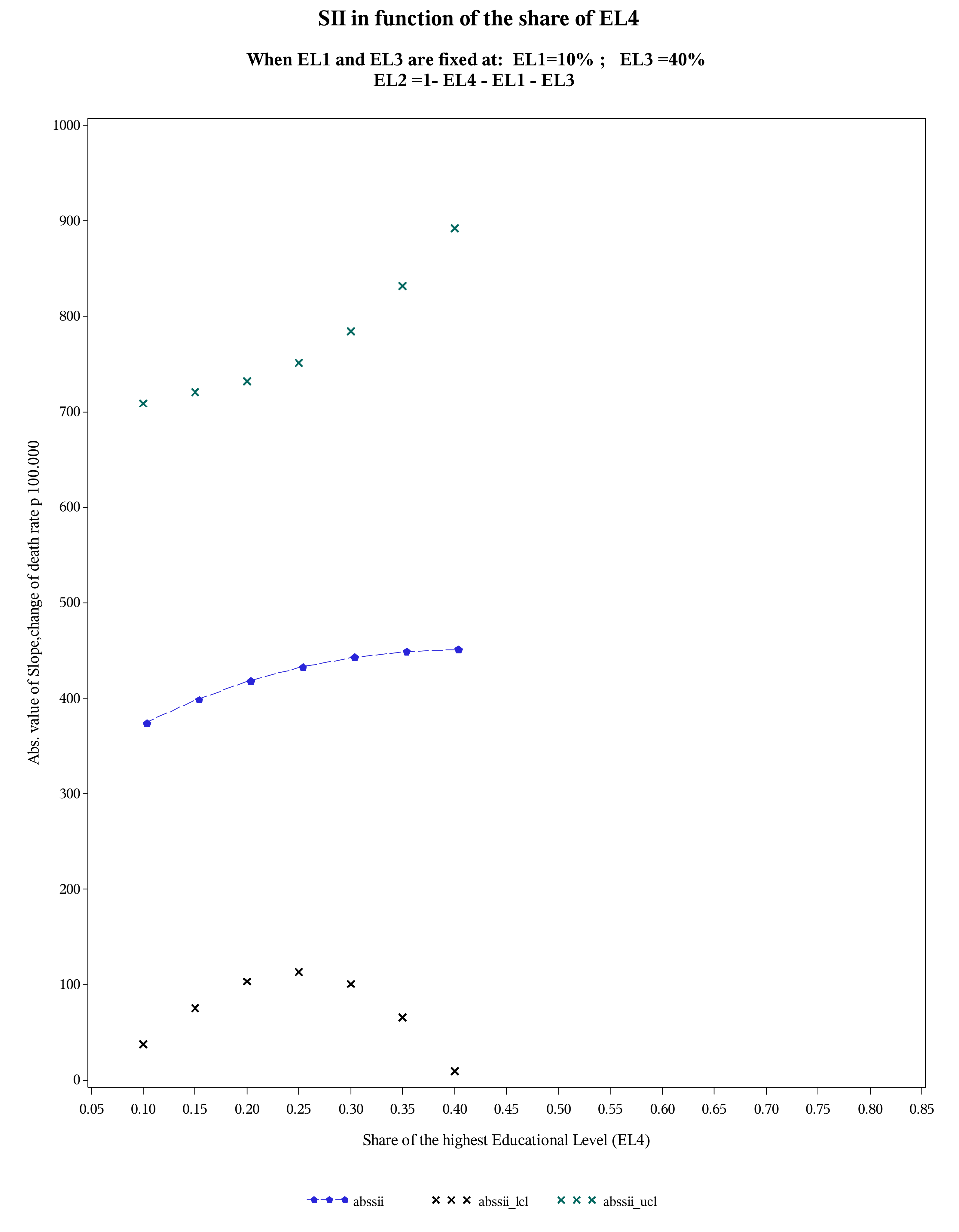
SII in function of the share of EL4
When EL1 and EL3 are fixed at: EL1=10% ; EL3 =40%
EL2 =1- EL4 - EL1 - EL3
Abs. value of Slope,change of death rate p 100.000
0
100
200
300
400
500
600
700
800
900
1000
0.05
0.10
0.15
0.20
0.25
0.30
0.35
0.40
0.45
0.50
0.55
0.60
0.65
0.70
0.75
0.80
0.85
Share of the highest Educational Level (EL4)
abssii
abssii_lcl
abssii_ucl

SII in function of the share of EL4

When EL1 and EL3 are fixed at: EL1=10% ; EL3 =45%
EL2 =1- EL4 - EL1 - EL3

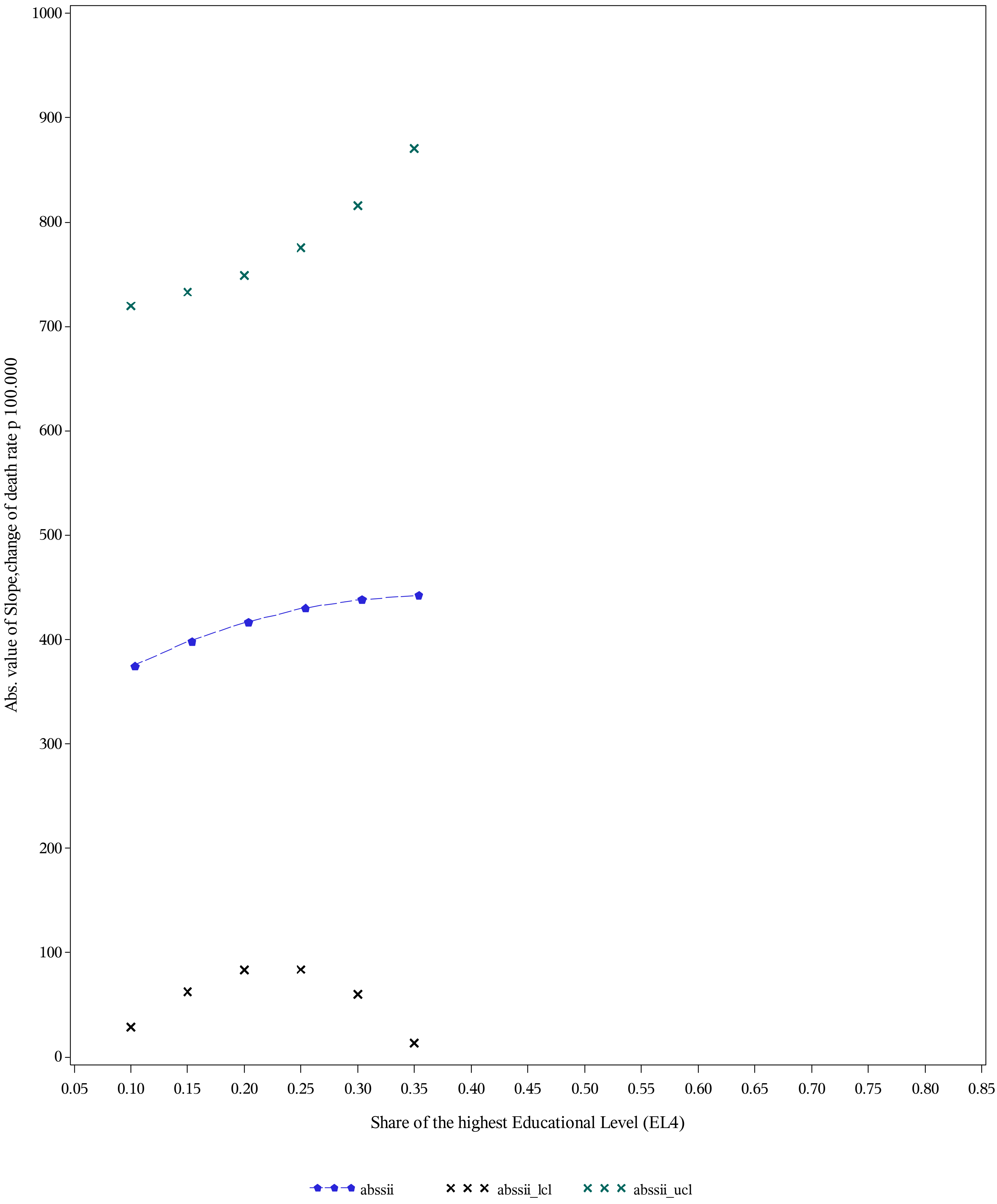

SII in function of the share of EL4

When EL1 and EL3 are fixed at: EL1=10% ; EL3 =50%
EL2 =1- EL4 - EL1 - EL3

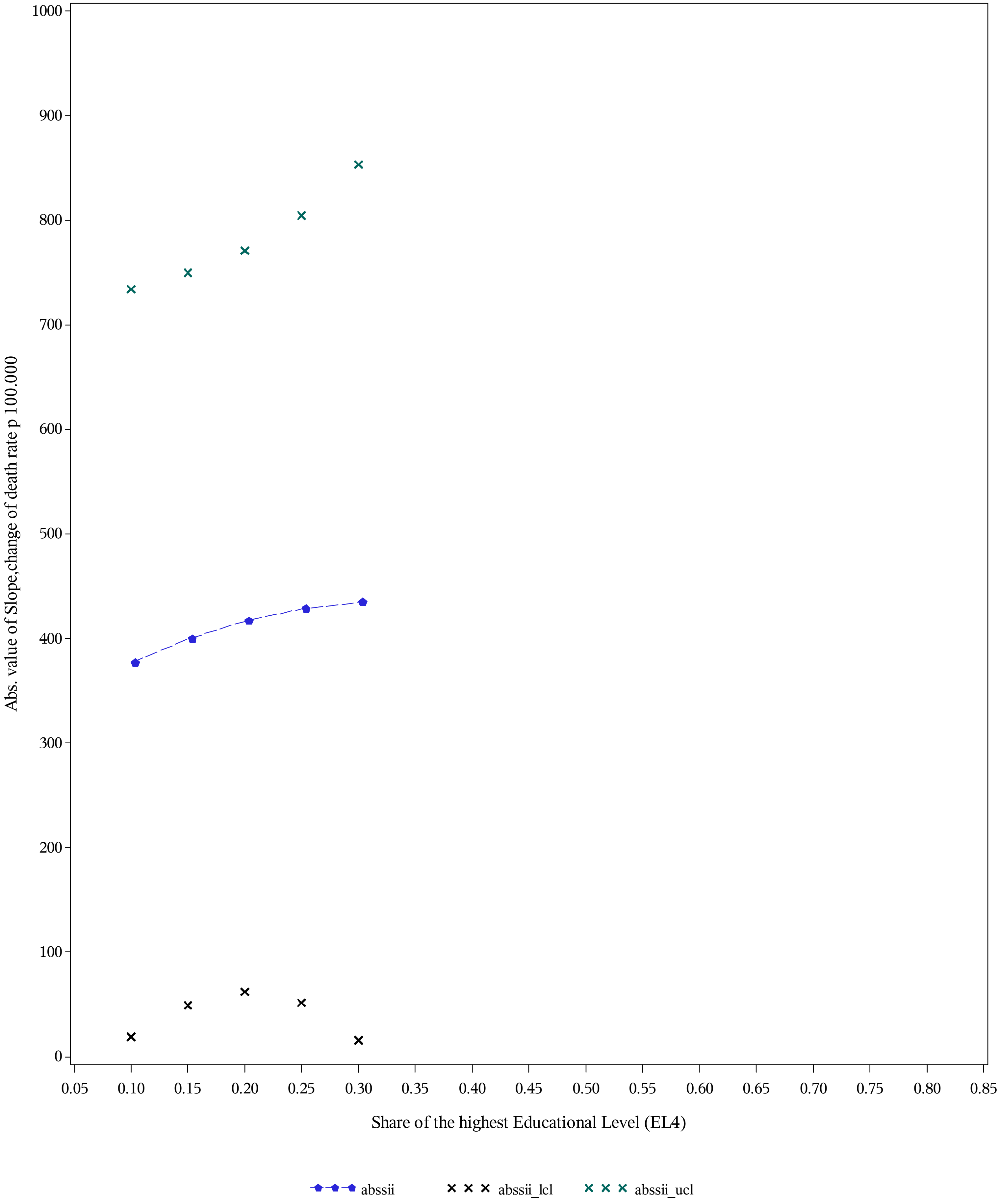

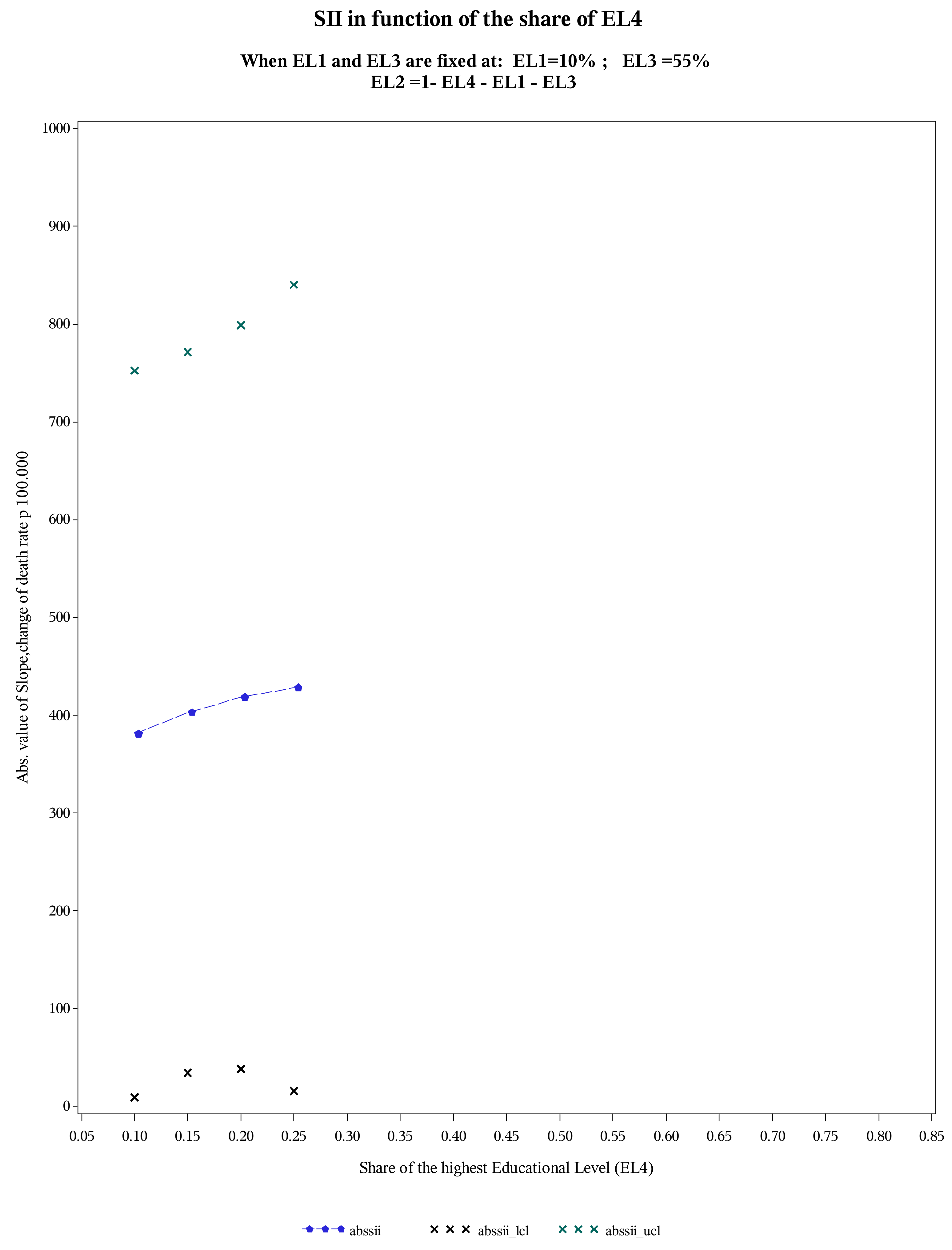
SII in function of the share of EL4
When EL1 and EL3 are fixed at: EL1=10% ; EL3 =55%
EL2 =1- EL4 - EL1 - EL3
Abs. value of Slope,change of death rate p 100.000
0
100
200
300
400
500
600
700
800
900
1000
0.05
0.10
0.15
0.20
0.25
0.30
0.35
0.40
0.45
0.50
0.55
0.60
0.65
0.70
0.75
0.80
0.85
Share of the highest Educational Level (EL4)
abssii
abssii_lcl
abssii_ucl

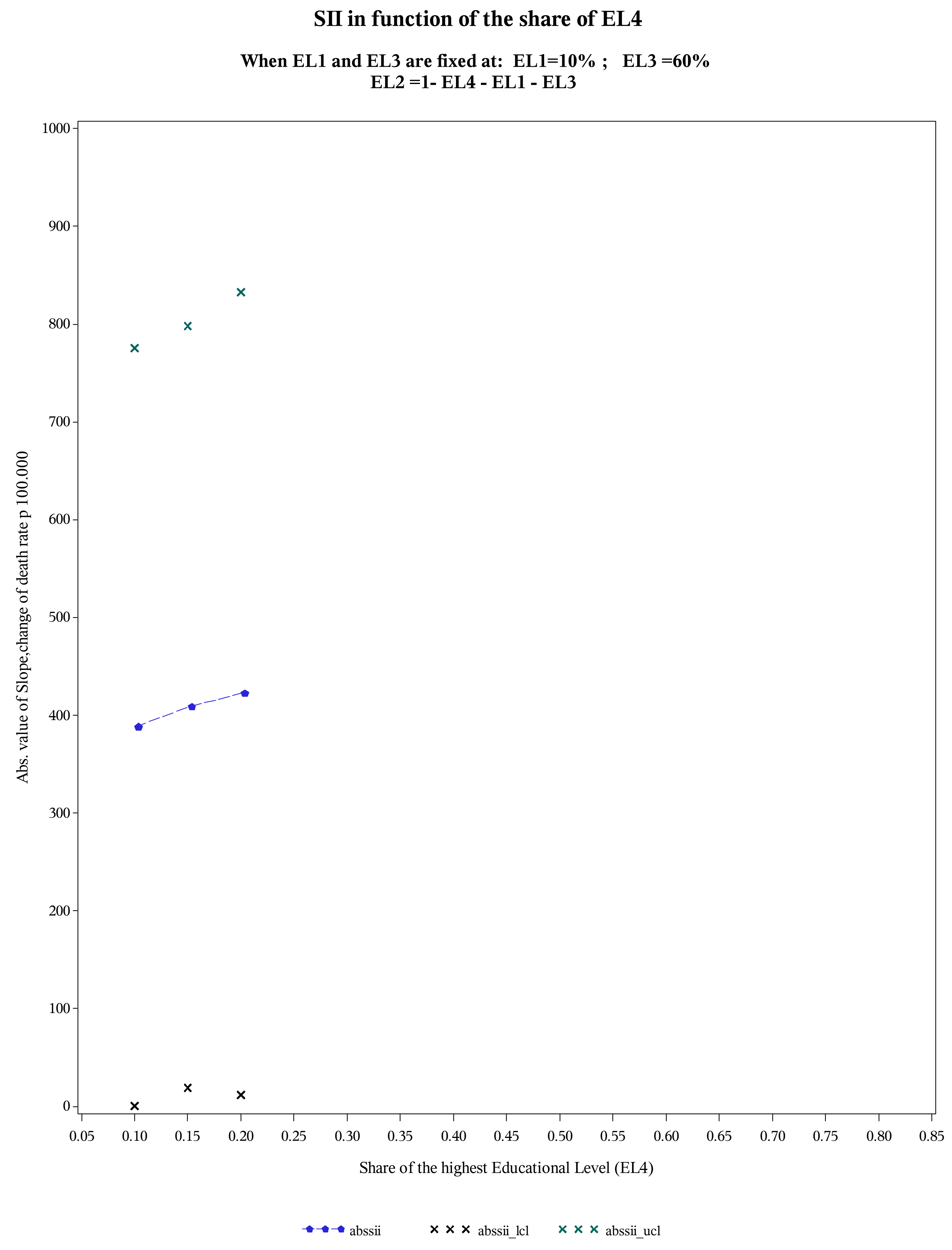
SII in function of the share of EL4
When EL1 and EL3 are fixed at: EL1=10% ; EL3 =60%
EL2 =1- EL4 - EL1 - EL3
Abs. value of Slope,change of death rate p 100.000
0
100
200
300
400
500
600
700
800
900
1000
0.05
0.10
0.15
0.20
0.25
0.30
0.35
0.40
0.45
0.50
0.55
0.60
0.65
0.70
0.75
0.80
0.85
Share of the highest Educational Level (EL4)
abssii
abssii_lcl
abssii_ucl

SII in function of the share of EL4

When EL1 and EL3 are fixed at: EL1=15% ; EL3 =5%
EL2 =1- EL4 - EL1 - EL3

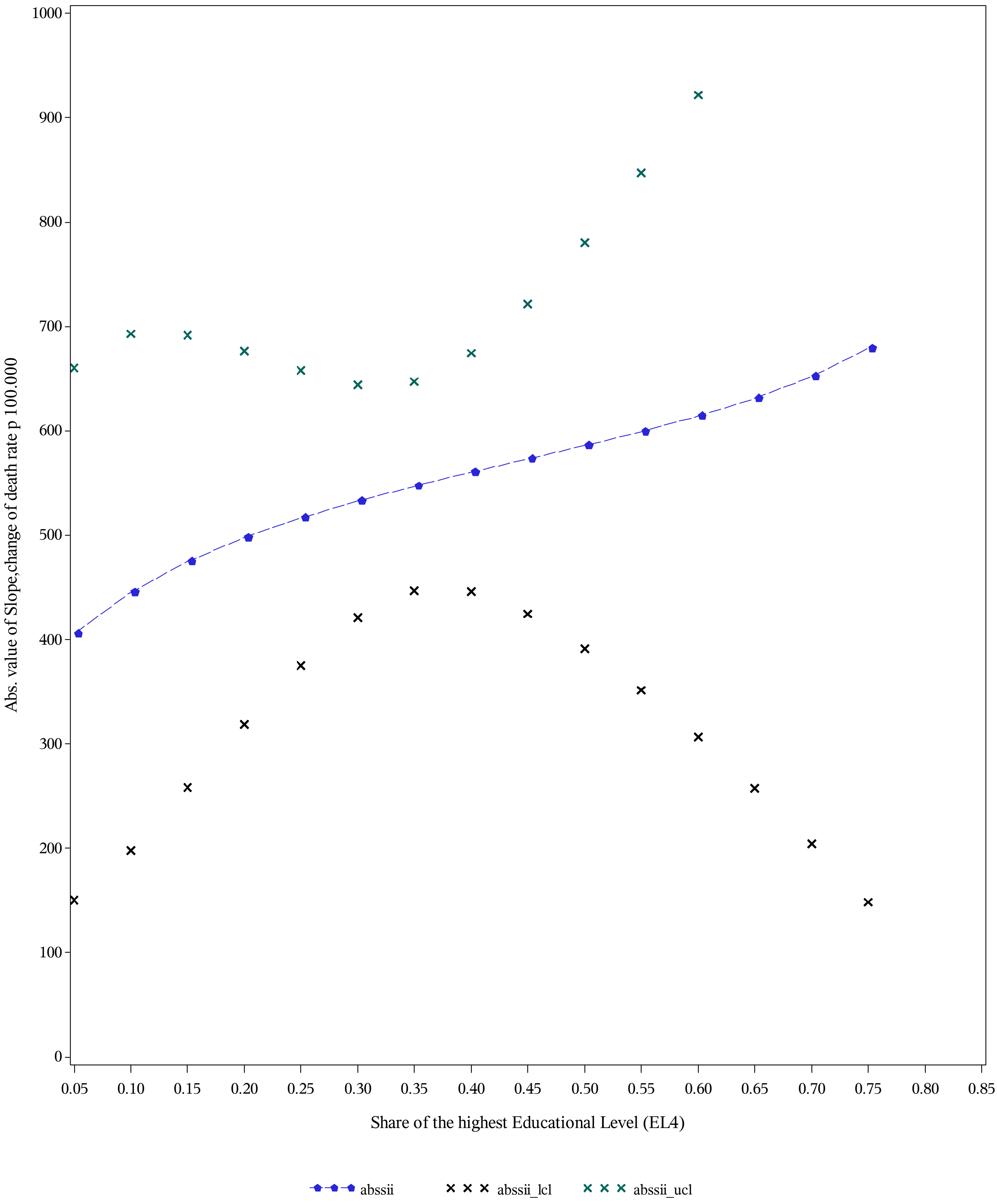

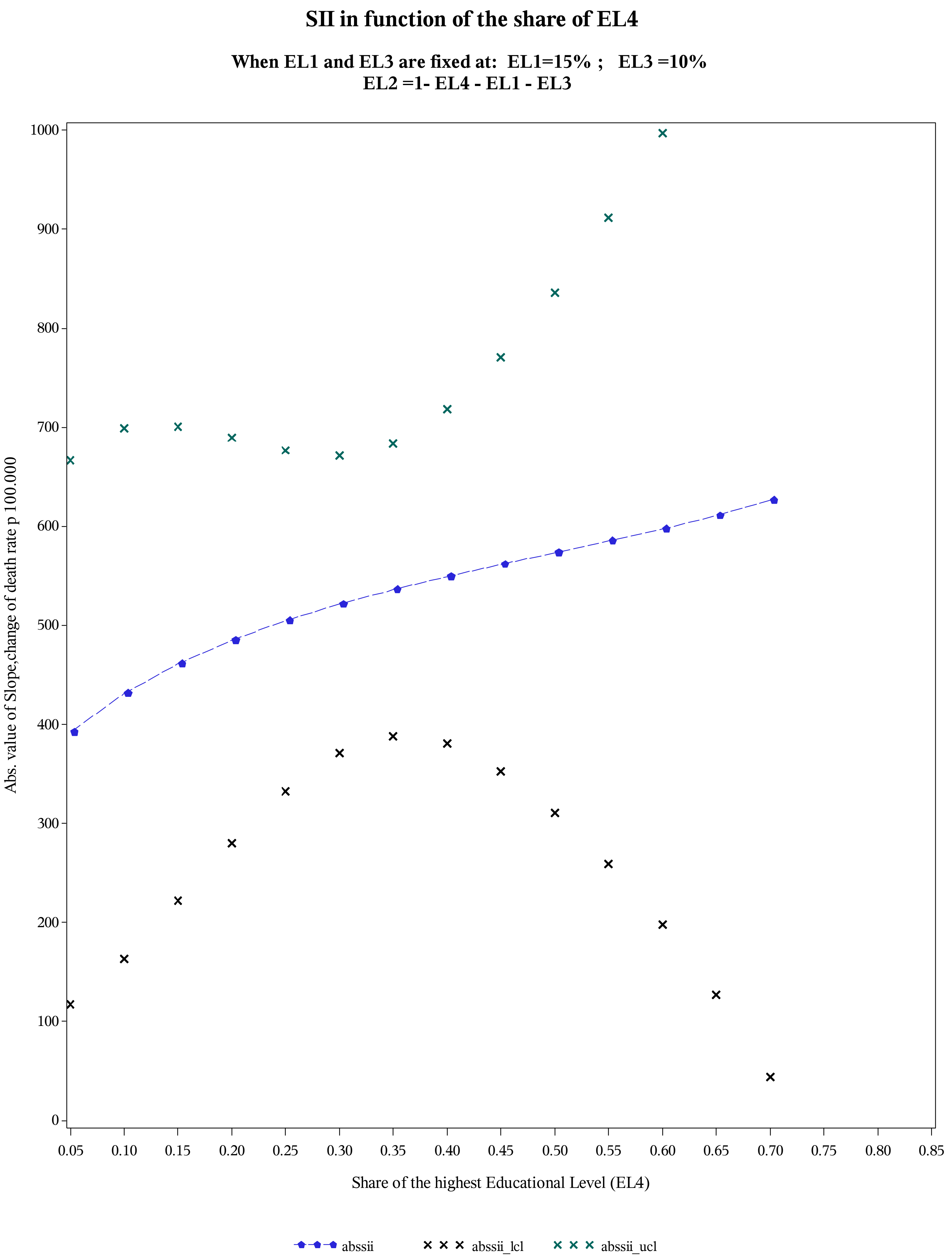
SII in function of the share of EL4
When EL1 and EL3 are fixed at: EL1=15% ; EL3 =10%
EL2 =1- EL4 - EL1 - EL3
Abs. value of Slope,change of death rate p 100.000
Share of the highest Educational Level (EL4)
abssii
abssii_lcl
abssii_ucl

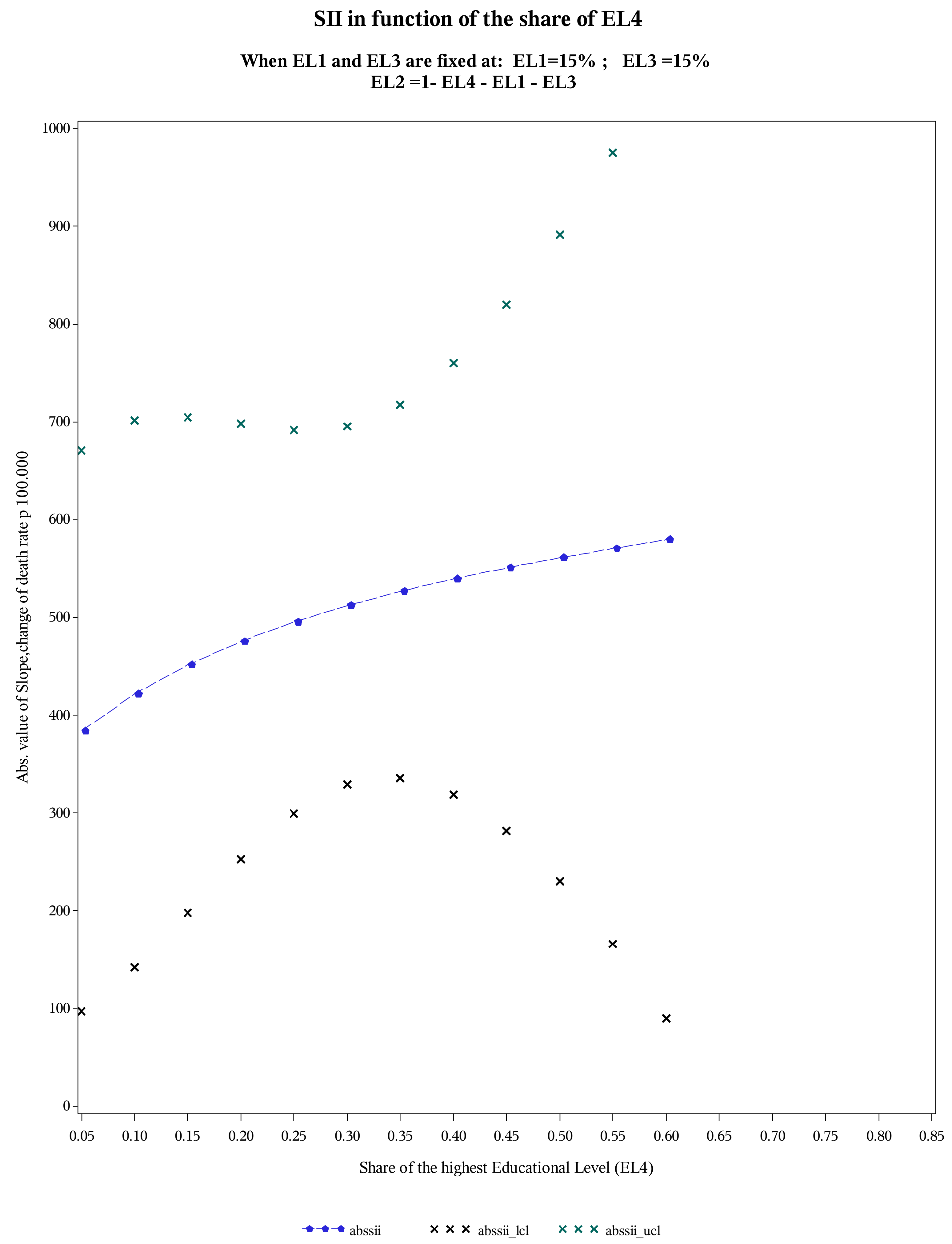
SII in function of the share of EL4
When EL1 and EL3 are fixed at: EL1=15% ; EL3 =15%
EL2 =1- EL4 - EL1 - EL3
Abs. value of Slope,change of death rate p 100.000
0
100
200
300
400
500
600
700
800
900
1000
0.05
0.10
0.15
0.20
0.25
0.30
0.35
0.40
0.45
0.50
0.55
0.60
0.65
0.70
0.75
0.80
0.85
Share of the highest Educational Level (EL4)
abssii
abssii_lcl
abssii_ucl

SII in function of the share of EL4

When EL1 and EL3 are fixed at: EL1=15% ; EL3 =20%
EL2 =1- EL4 - EL1 - EL3

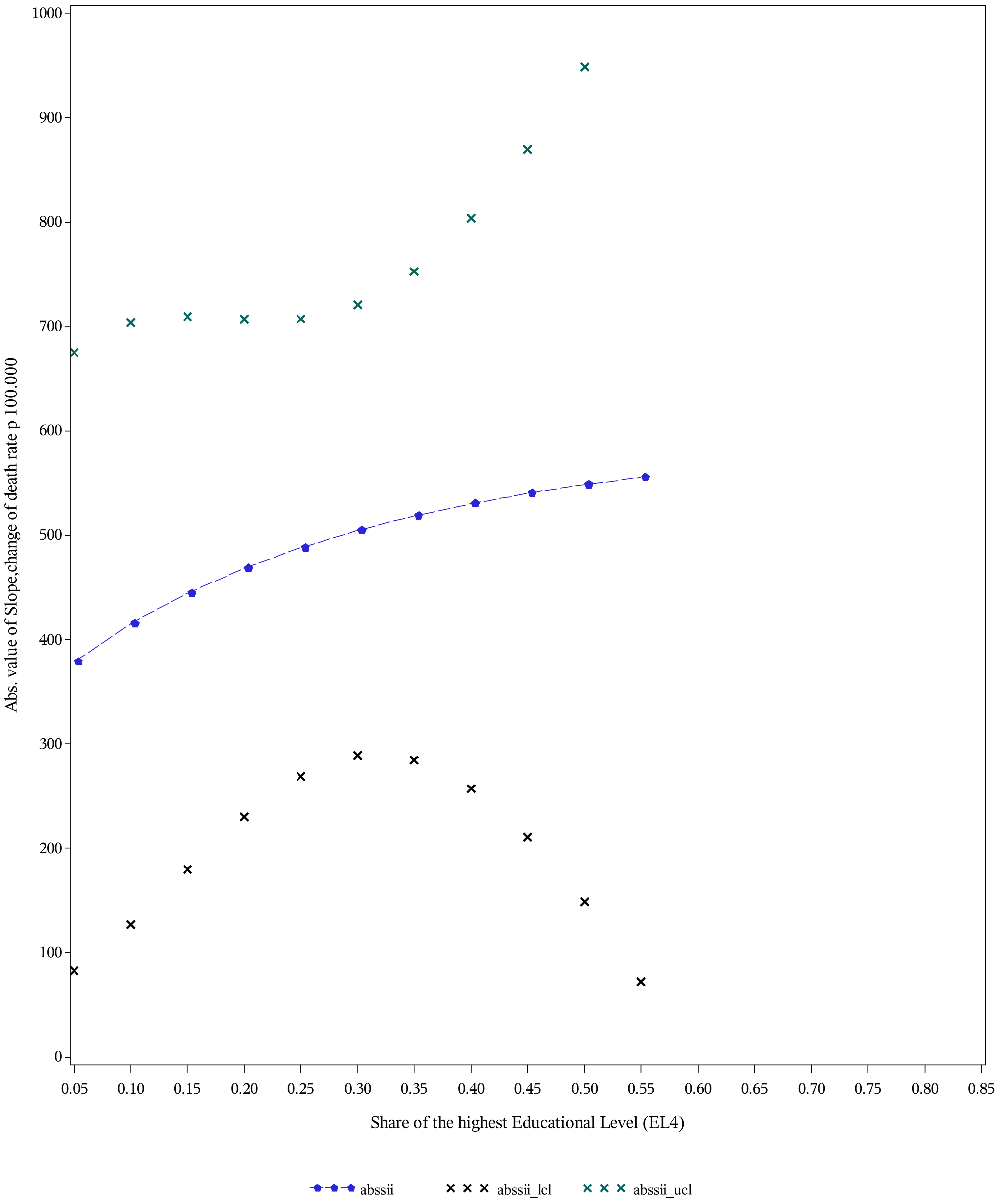

SII in function of the share of EL4

When EL1 and EL3 are fixed at: EL1=15% ; EL3 =25%
EL2 =1- EL4 - EL1 - EL3

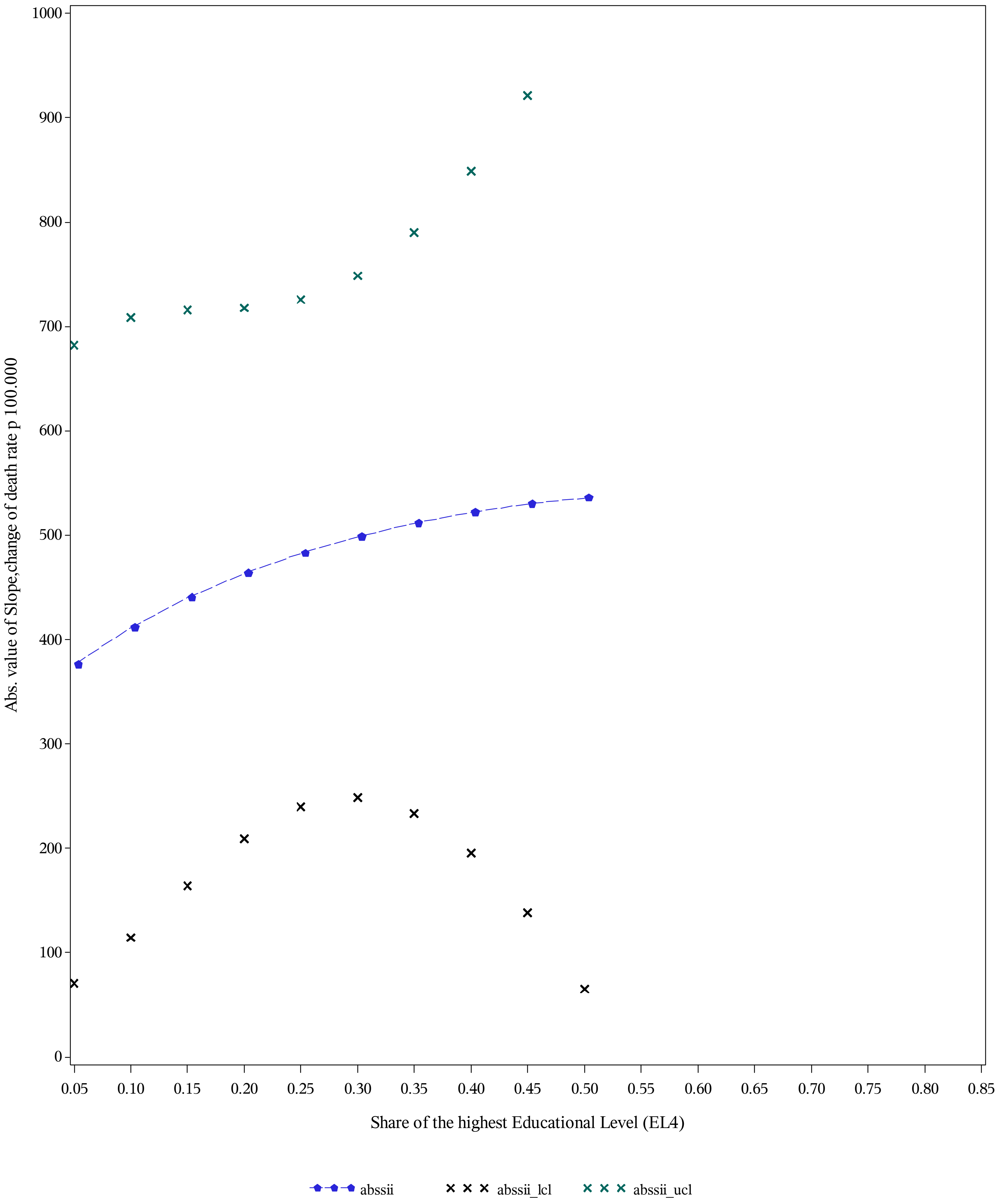

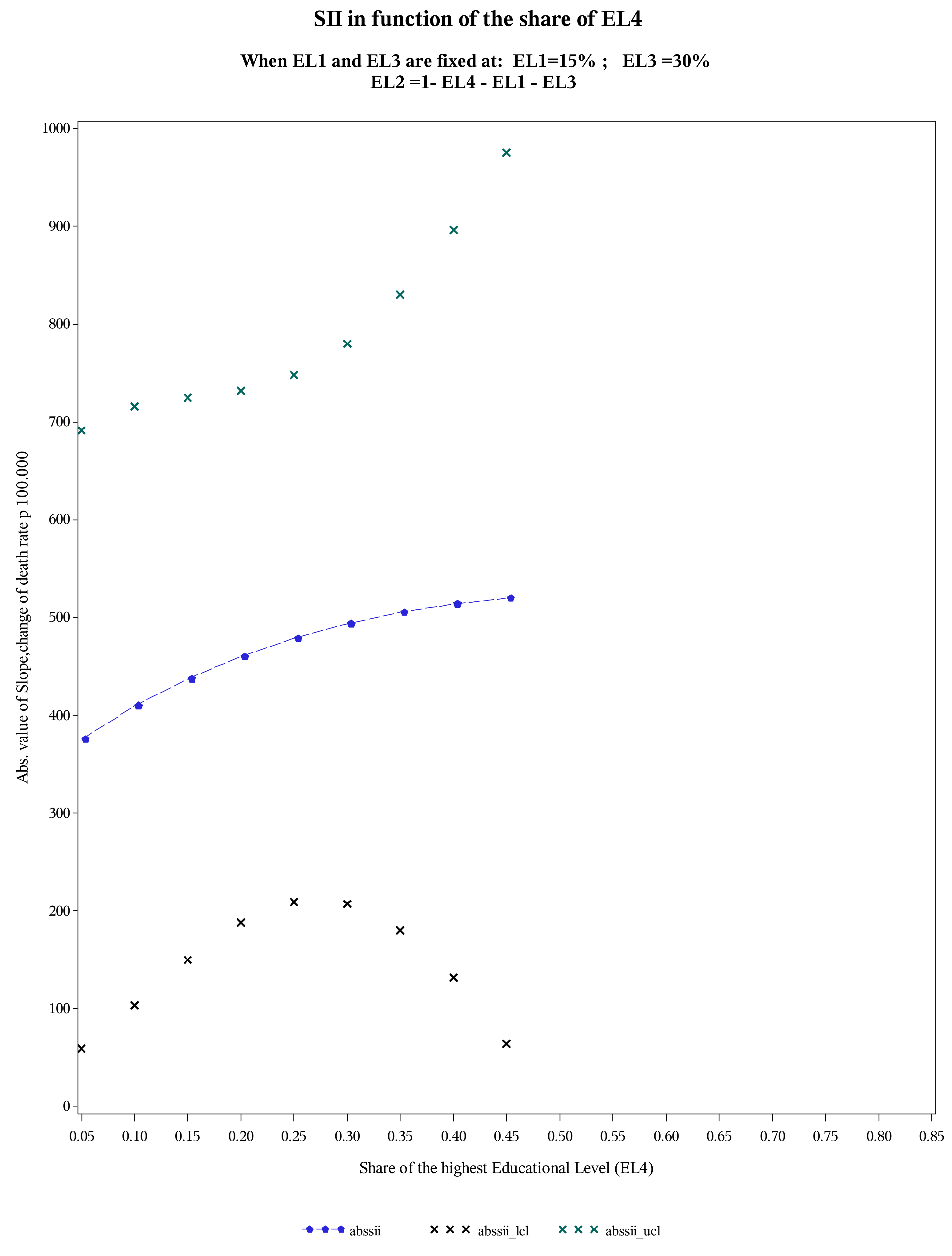
SII in function of the share of EL4
When EL1 and EL3 are fixed at: EL1=15% ; EL3 =30%
EL2 =1- EL4 - EL1 - EL3
Abs. value of Slope,change of death rate p 100.000
1000
900
800
700
600
500
400
300
200
100
0
0.05
0.10
0.15
0.20
0.25
0.30
0.35
0.40
0.45
0.50
0.55
0.60
0.65
0.70
0.75
0.80
0.85
Share of the highest Educational Level (EL4)
abssii
abssii_lcl
abssii_ucl

SII in function of the share of EL4

When EL1 and EL3 are fixed at: EL1=15% ; EL3 =35%
EL2 =1- EL4 - EL1 - EL3

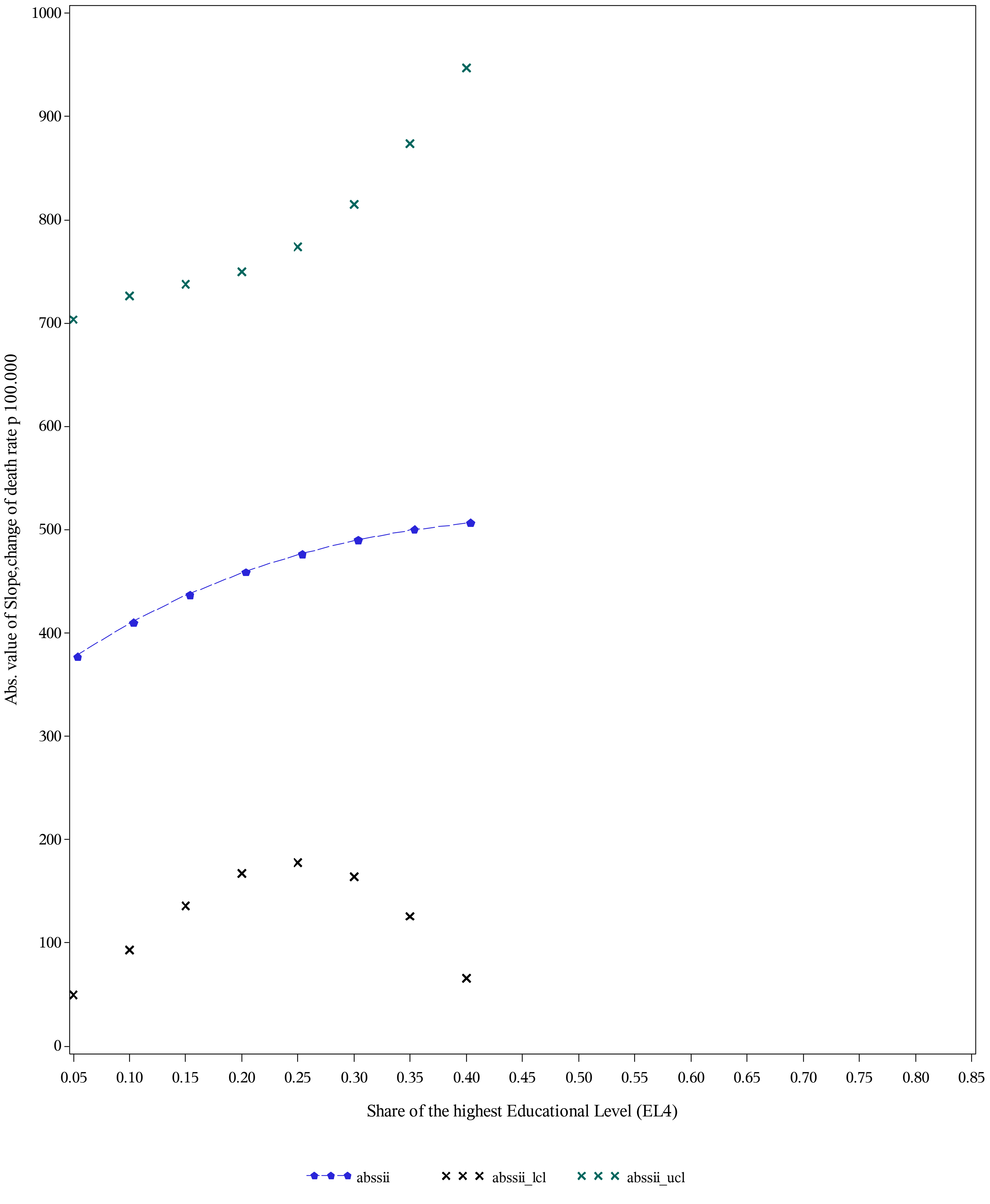

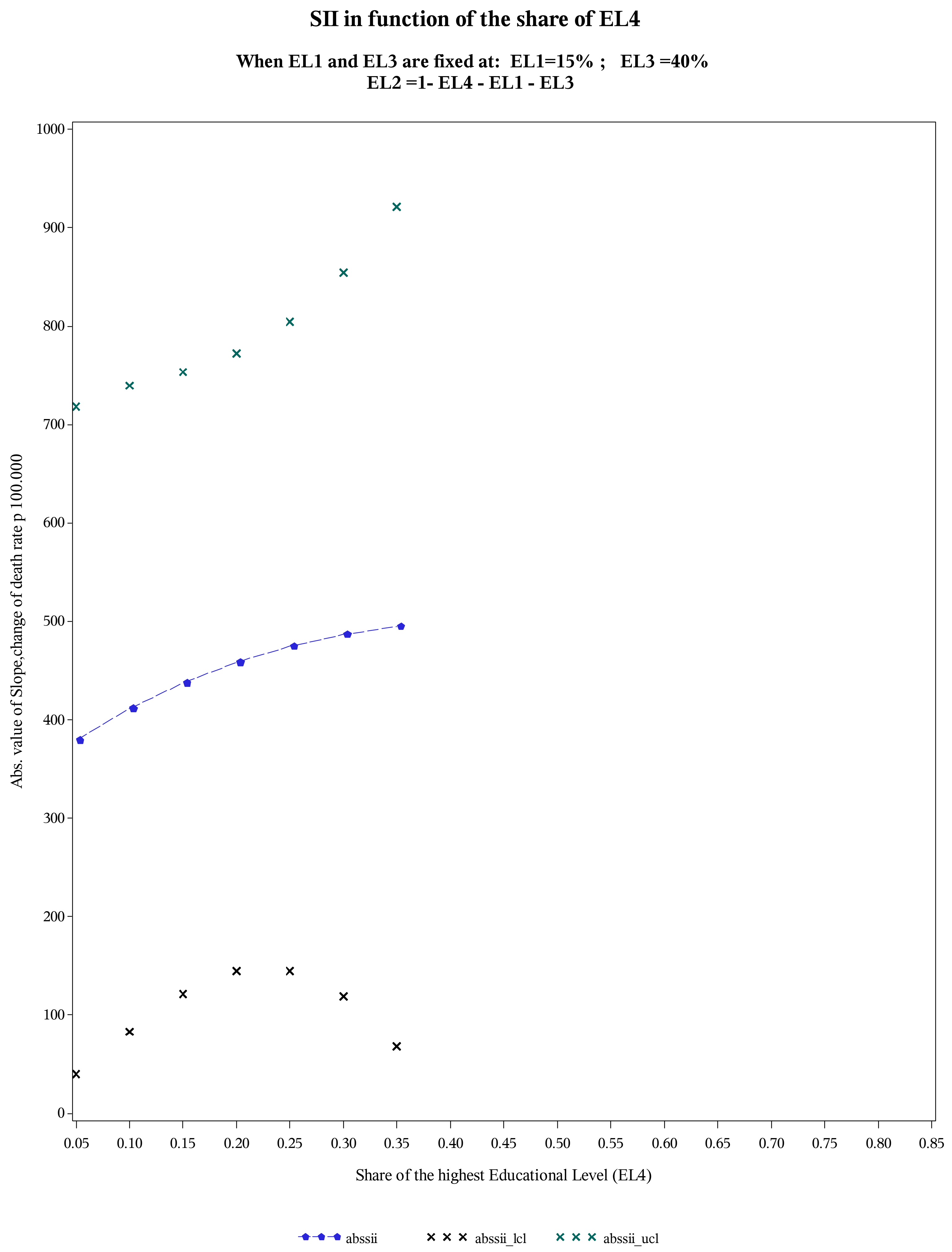
SII in function of the share of EL4
When EL1 and EL3 are fixed at: EL1=15% ; EL3 =40%
EL2 =1- EL4 - EL1 - EL3
Abs. value of Slope,change of death rate p 100.000
0
100
200
300
400
500
600
700
800
900
1000
0.05
0.10
0.15
0.20
0.25
0.30
0.35
0.40
0.45
0.50
0.55
0.60
0.65
0.70
0.75
0.80
0.85
Share of the highest Educational Level (EL4)
abssii
abssii_lcl
abssii_ucl

SII in function of the share of EL4

When EL1 and EL3 are fixed at: EL1=15% ; EL3 =45%
EL2 =1- EL4 - EL1 - EL3

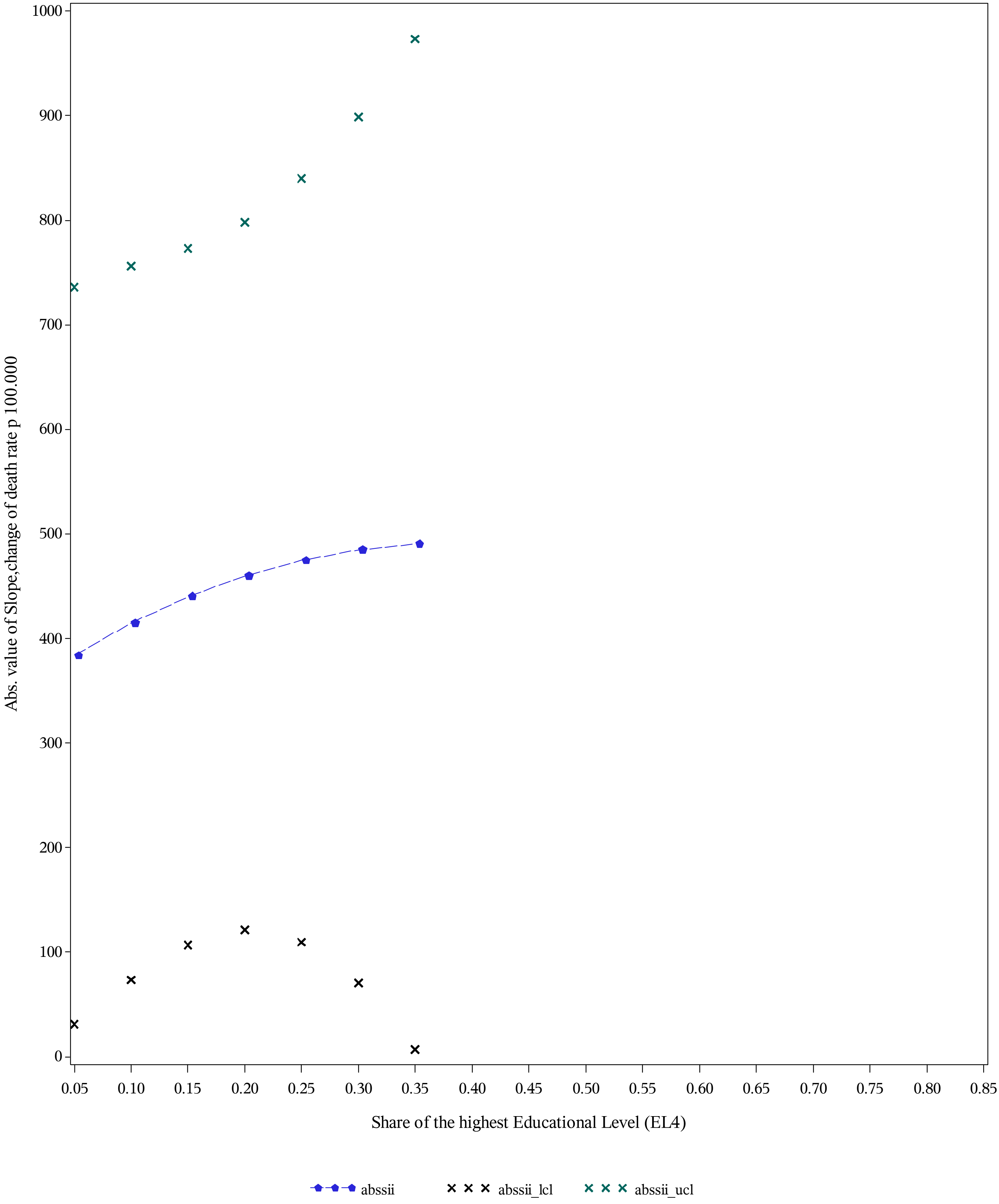

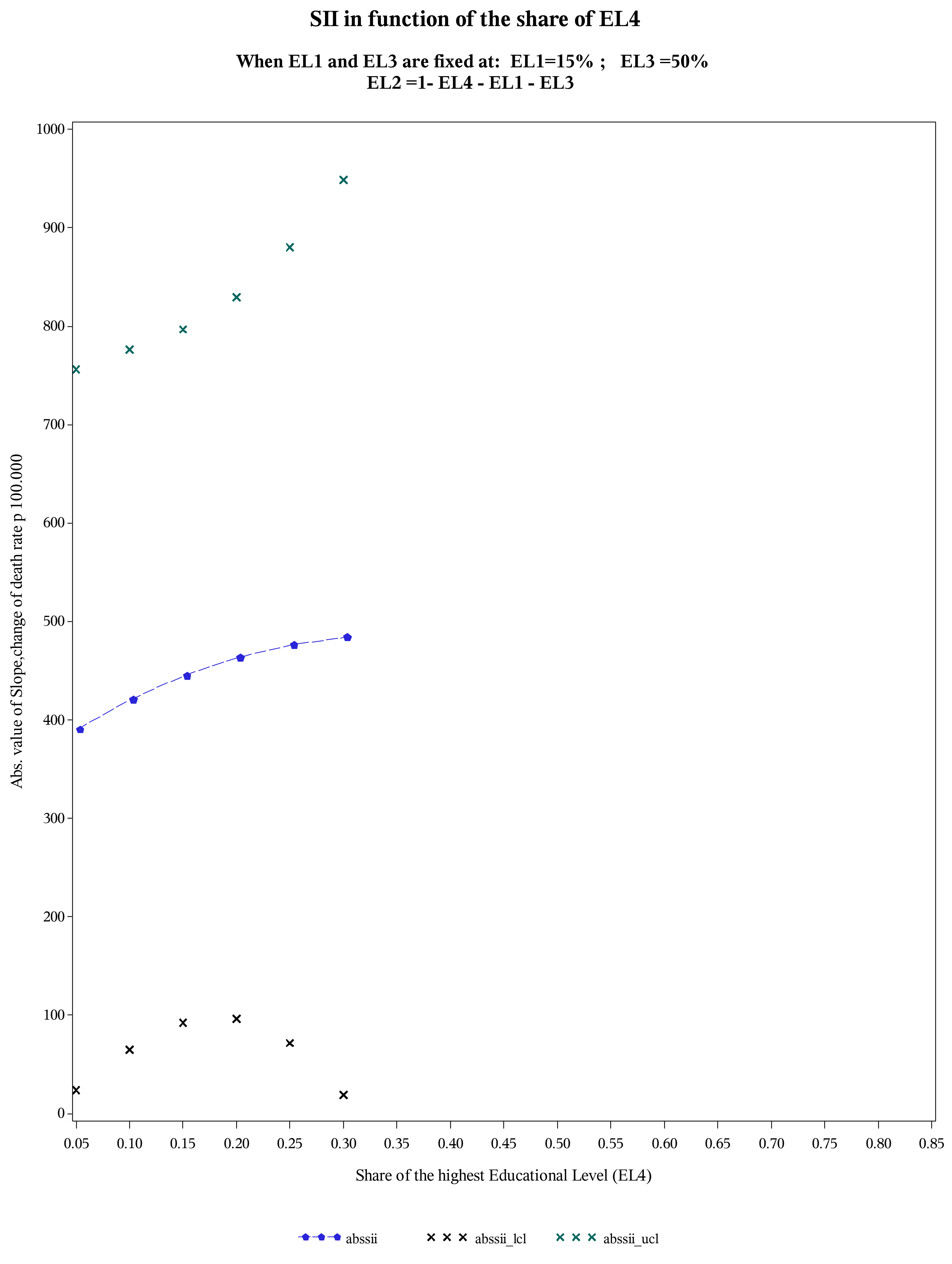

SII in function of the share of EL4
When EL1 and EL3 are fixed at: EL1=15% ; EL3 =50%
EL2 =1- EL4 - EL1 - EL3
Abs. value of Slope,change of death rate p 100.000
1000
900
800
700
600
500
400
300
200
100
0
0.05
0.10
0.15
0.20
0.25
0.30
0.35
0.40
0.45
0.50
0.55
0.60
0.65
0.70
0.75
0.80
0.85
Share of the highest Educational Level (EL4)
abssii
abssii_lcl
abssii_ucl

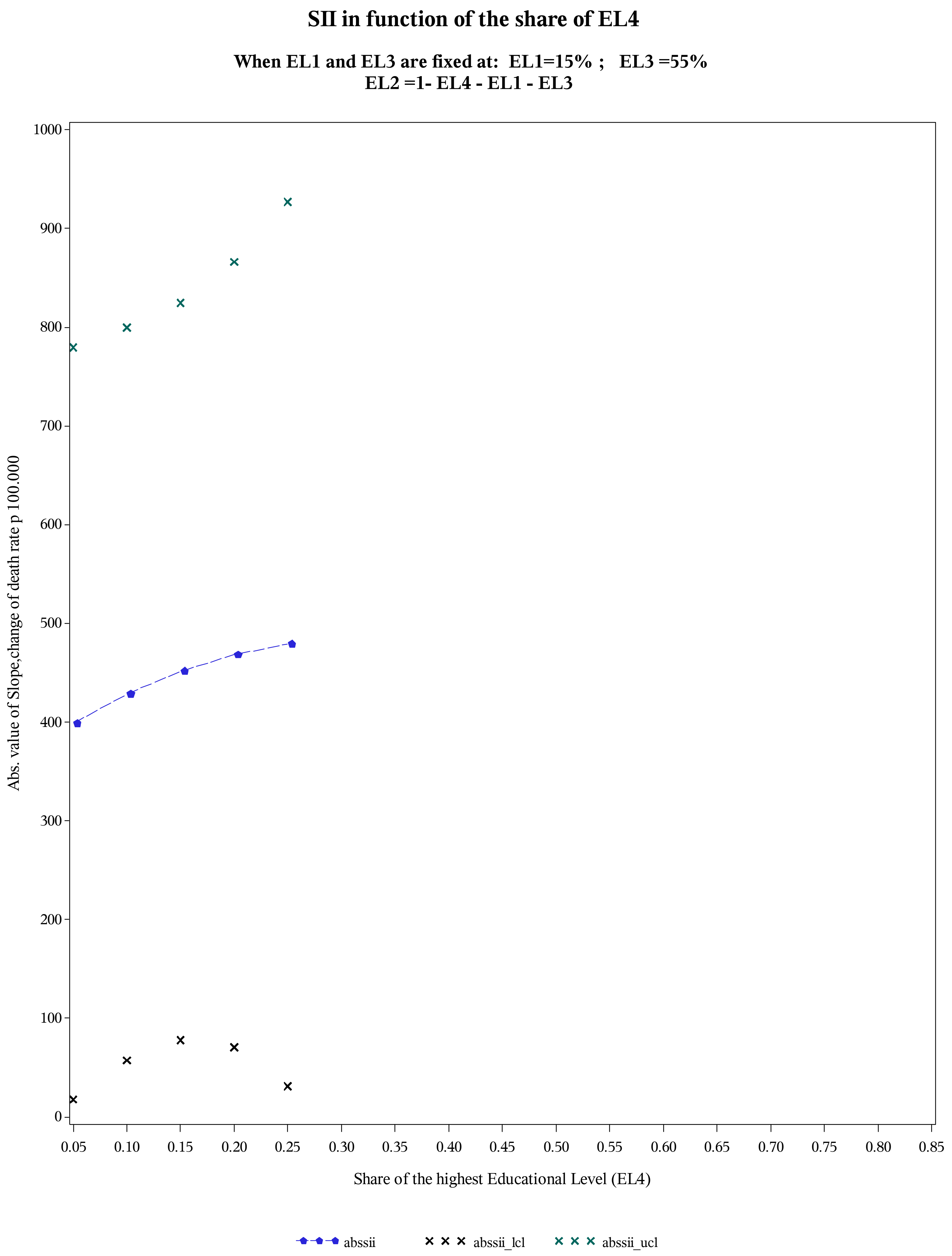
SII in function of the share of EL4
When EL1 and EL3 are fixed at: EL1=15% ; EL3 =55%
EL2 =1- EL4 - EL1 - EL3
Abs. value of Slope,change of death rate p 100.000
1000
900
800
700
600
500
400
300
200
100
0
0.05
0.10
0.15
0.20
0.25
0.30
0.35
0.40
0.45
0.50
0.55
0.60
0.65
0.70
0.75
0.80
0.85
Share of the highest Educational Level (EL4)
abssii
abssii_lcl
abssii_ucl

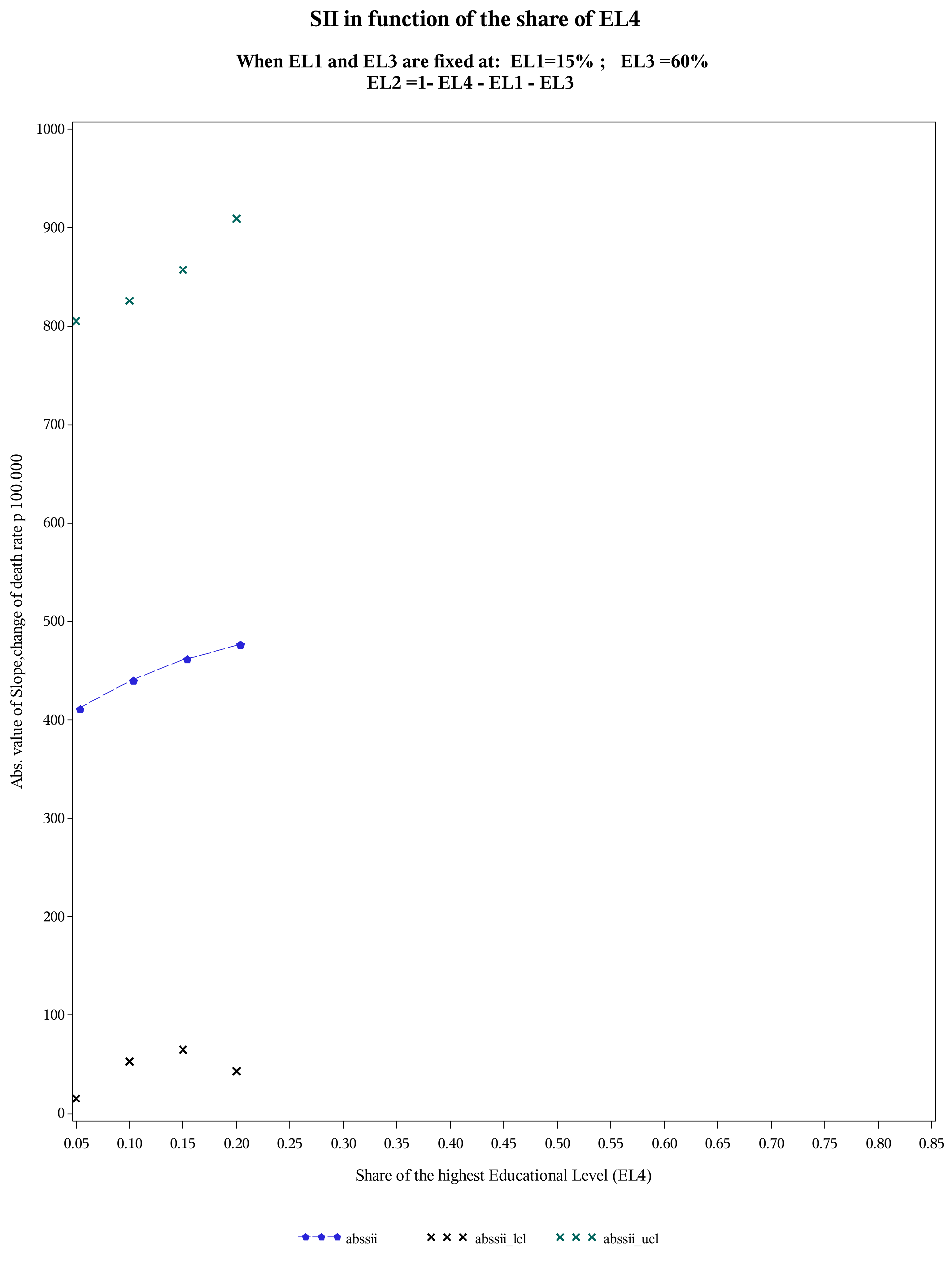
SII in function of the share of EL4
When EL1 and EL3 are fixed at: EL1=15% ; EL3 =60%
EL2 =1- EL4 - EL1 - EL3
Abs. value of Slope,change of death rate p 100.000
0
100
200
300
400
500
600
700
800
900
1000
0.05
0.10
0.15
0.20
0.25
0.30
0.35
0.40
0.45
0.50
0.55
0.60
0.65
0.70
0.75
0.80
0.85
Share of the highest Educational Level (EL4)
abssii
abssii_lcl
abssii_ucl

SII in function of the share of EL4

When EL1 and EL3 are fixed at: EL1=15% ; EL3 =65%
EL2 =1- EL4 - EL1 - EL3

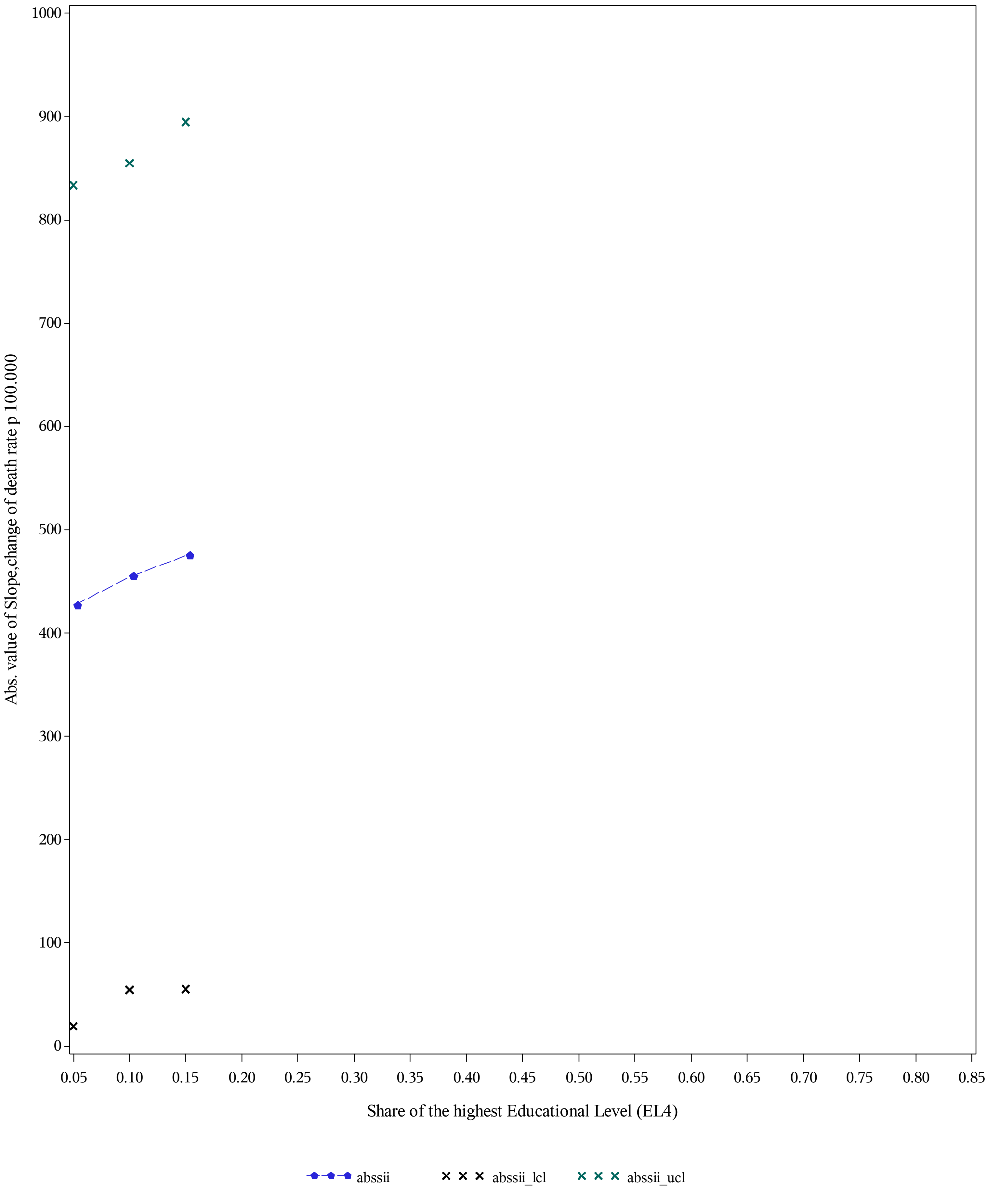

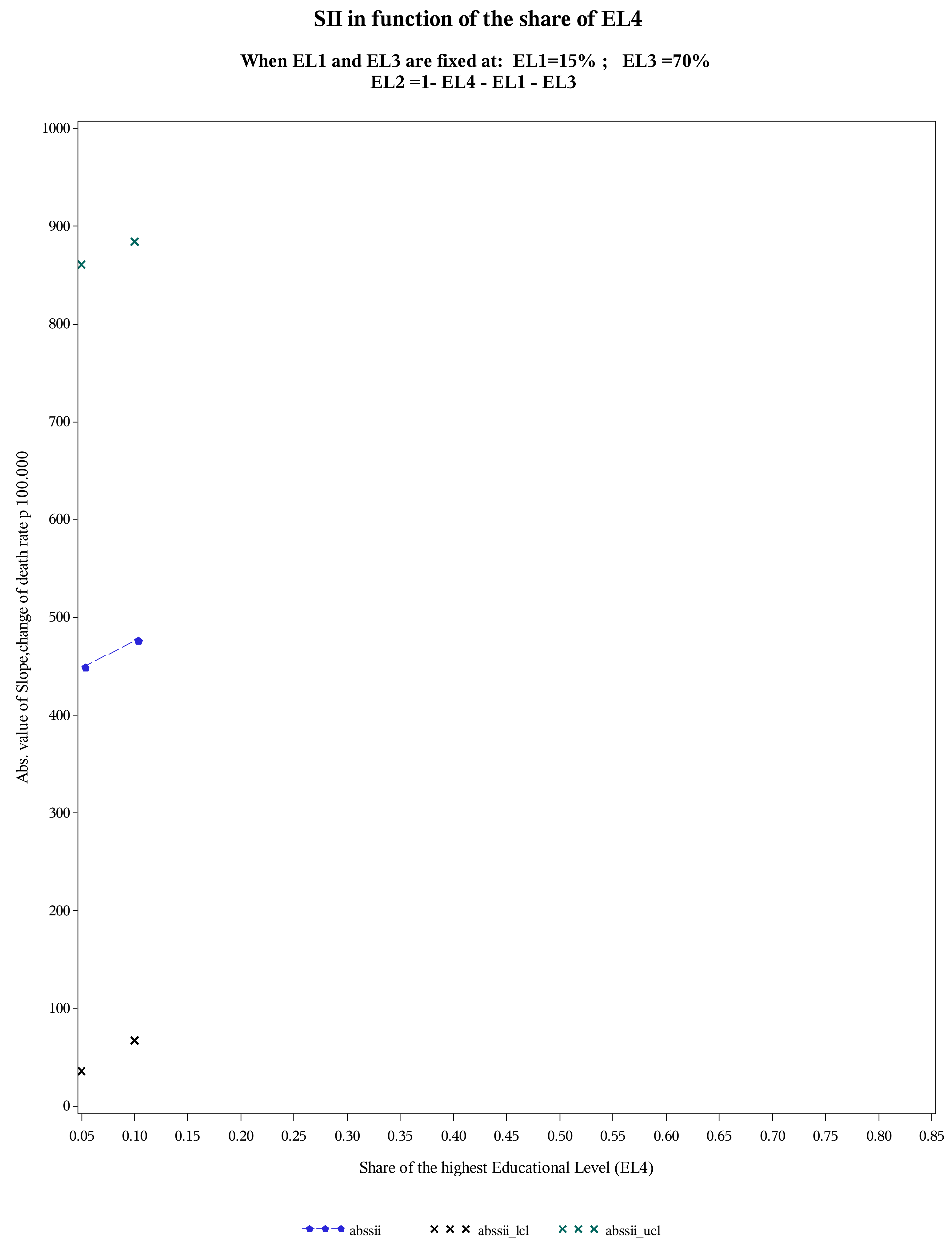
SII in function of the share of EL4
When EL1 and EL3 are fixed at: EL1=15% ; EL3 =70%
EL2 =1- EL4 - EL1 - EL3
Abs. value of Slope,change of death rate p 100.000
0
100
200
300
400
500
600
700
800
900
1000
0.05
0.10
0.15
0.20
0.25
0.30
0.35
0.40
0.45
0.50
0.55
0.60
0.65
0.70
0.75
0.80
0.85
Share of the highest Educational Level (EL4)
abssii
abssii_lcl
abssii_ucl

SII in function of the share of EL4

When EL1 and EL3 are fixed at: EL1=20% ; EL3 =5%
EL2 =1- EL4 - EL1 - EL3

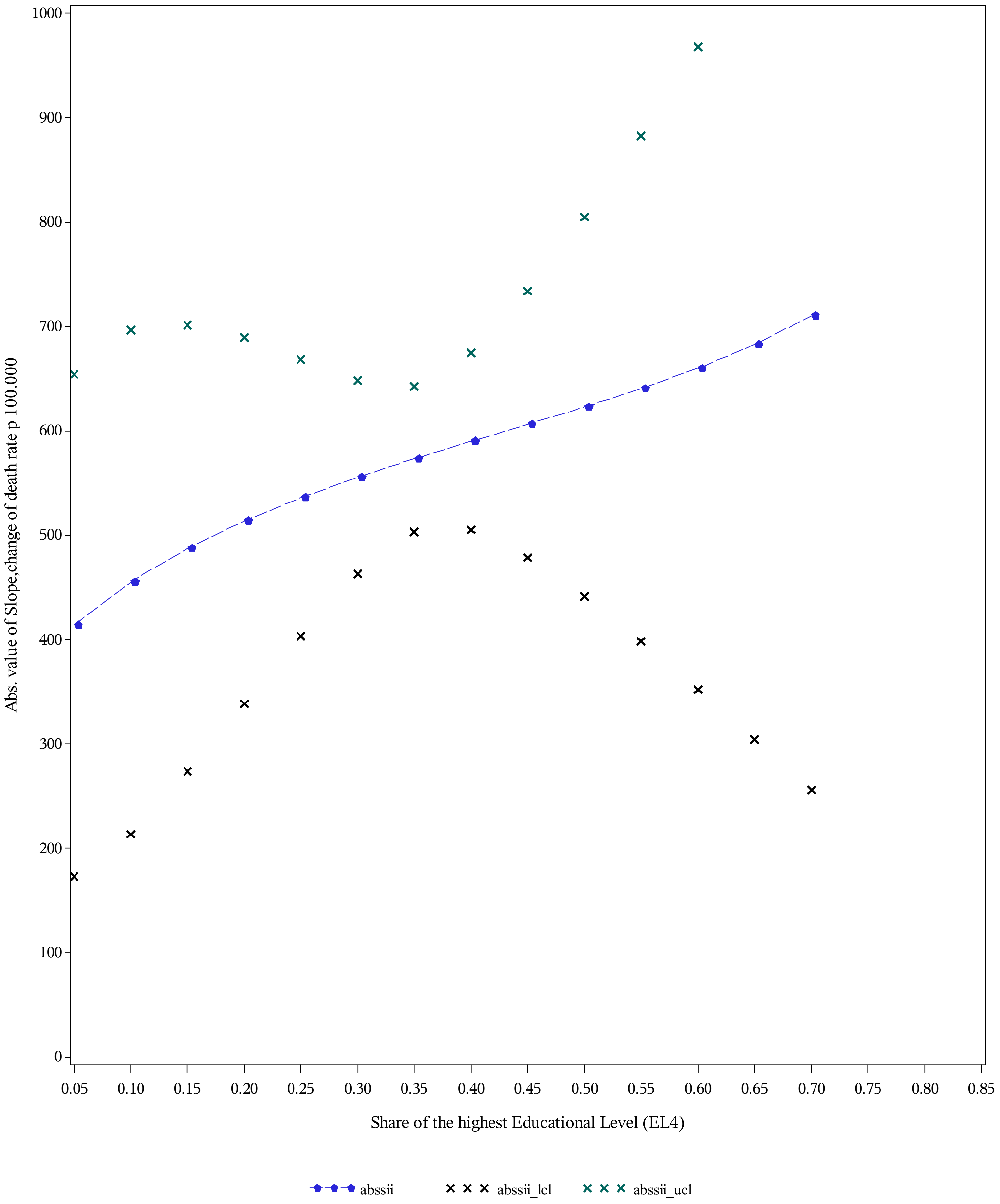

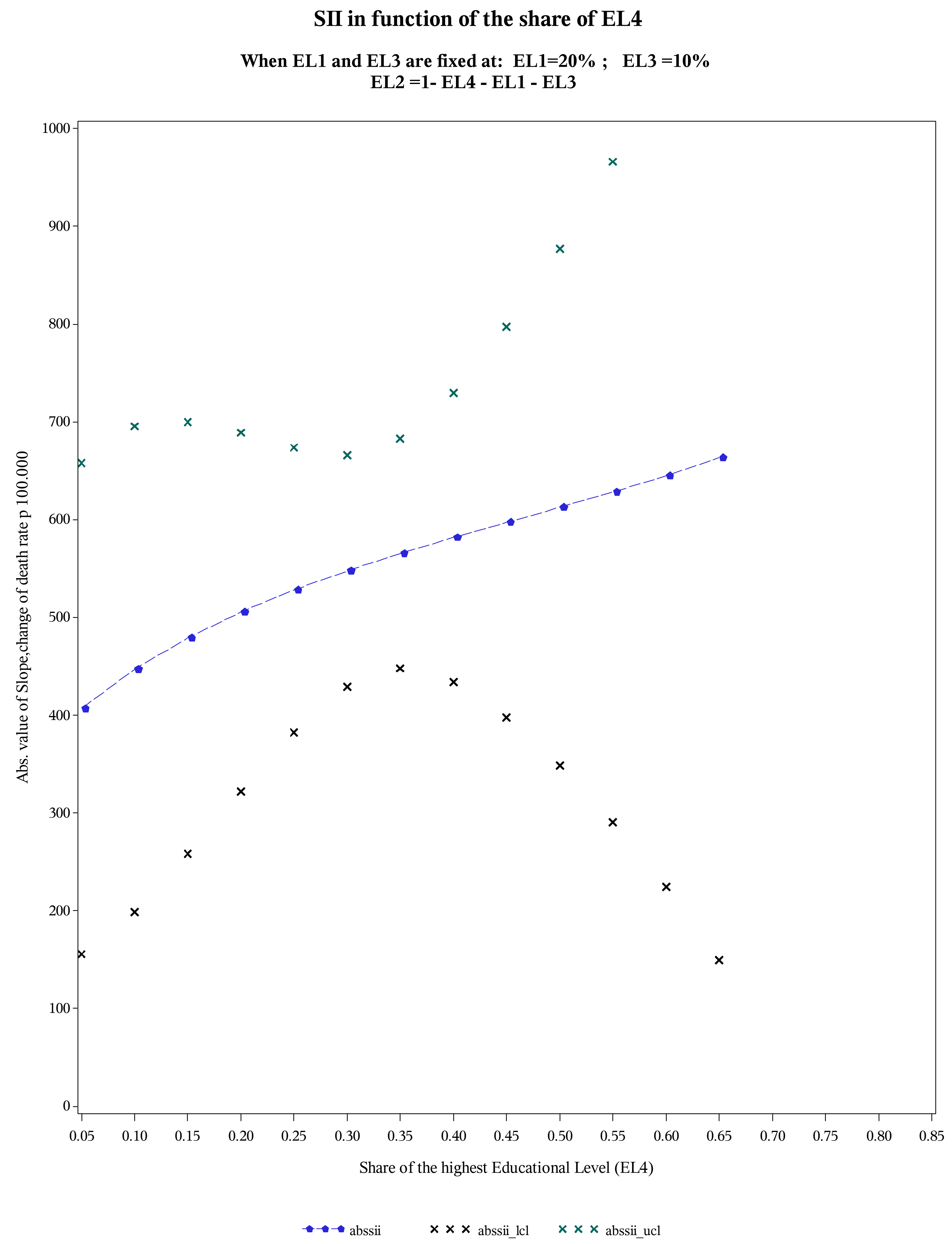

SII in function of the share of EL4
When EL1 and EL3 are fixed at: EL1=20% ; EL3 =10%
EL2 =1- EL4 - EL1 - EL3
Abs. value of Slope,change of death rate p 100.000
0
100
200
300
400
500
600
700
800
900
1000
0.05
0.10
0.15
0.20
0.25
0.30
0.35
0.40
0.45
0.50
0.55
0.60
0.65
0.70
0.75
0.80
0.85
Share of the highest Educational Level (EL4)
abssii
abssii_lcl
abssii_ucl

SII in function of the share of EL4

When EL1 and EL3 are fixed at: EL1=20% ; EL3 =15%
EL2 =1- EL4 - EL1 - EL3

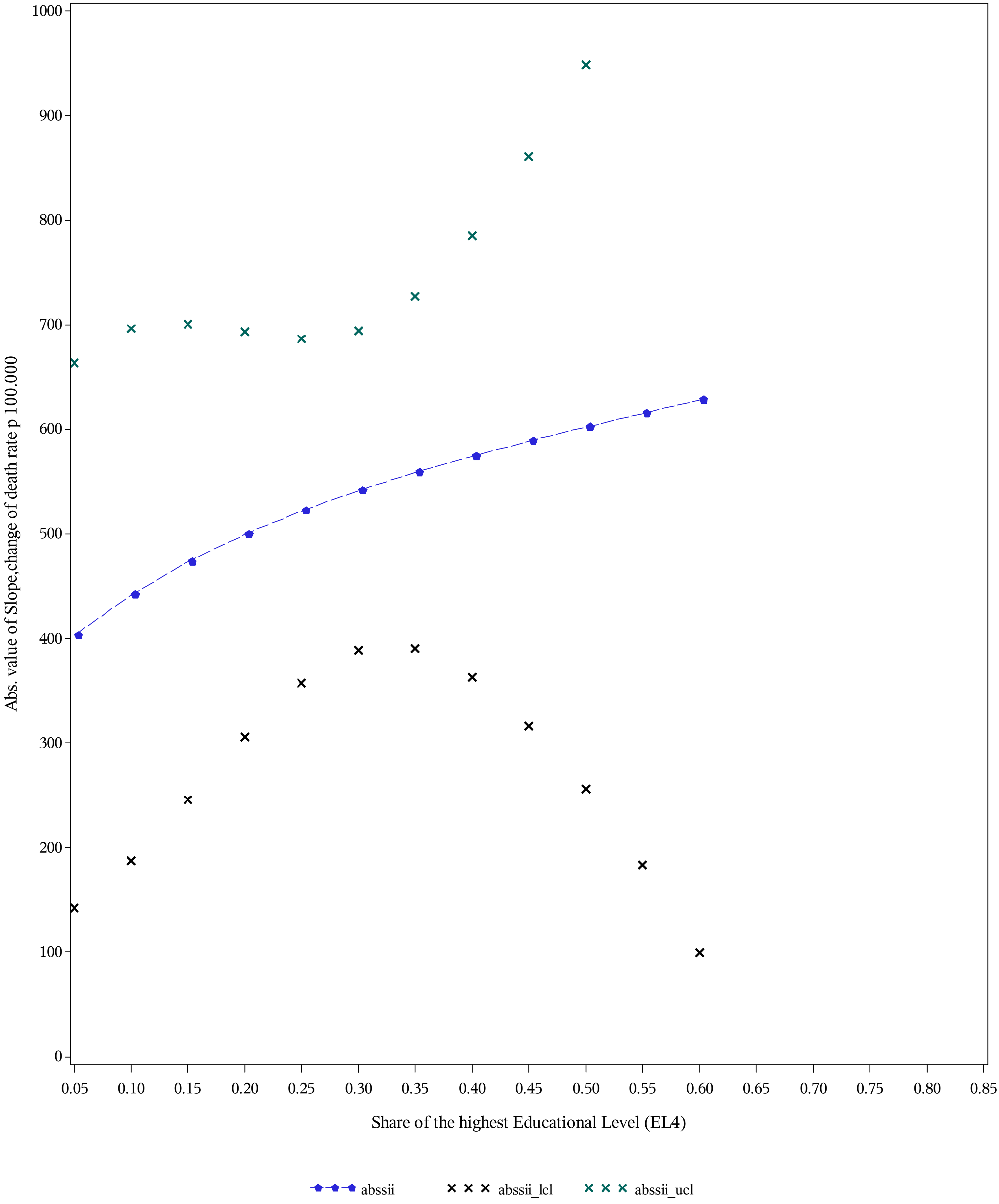

SII in function of the share of EL4

When EL1 and EL3 are fixed at: EL1=20% ; EL3 =20%
EL2 =1- EL4 - EL1 - EL3

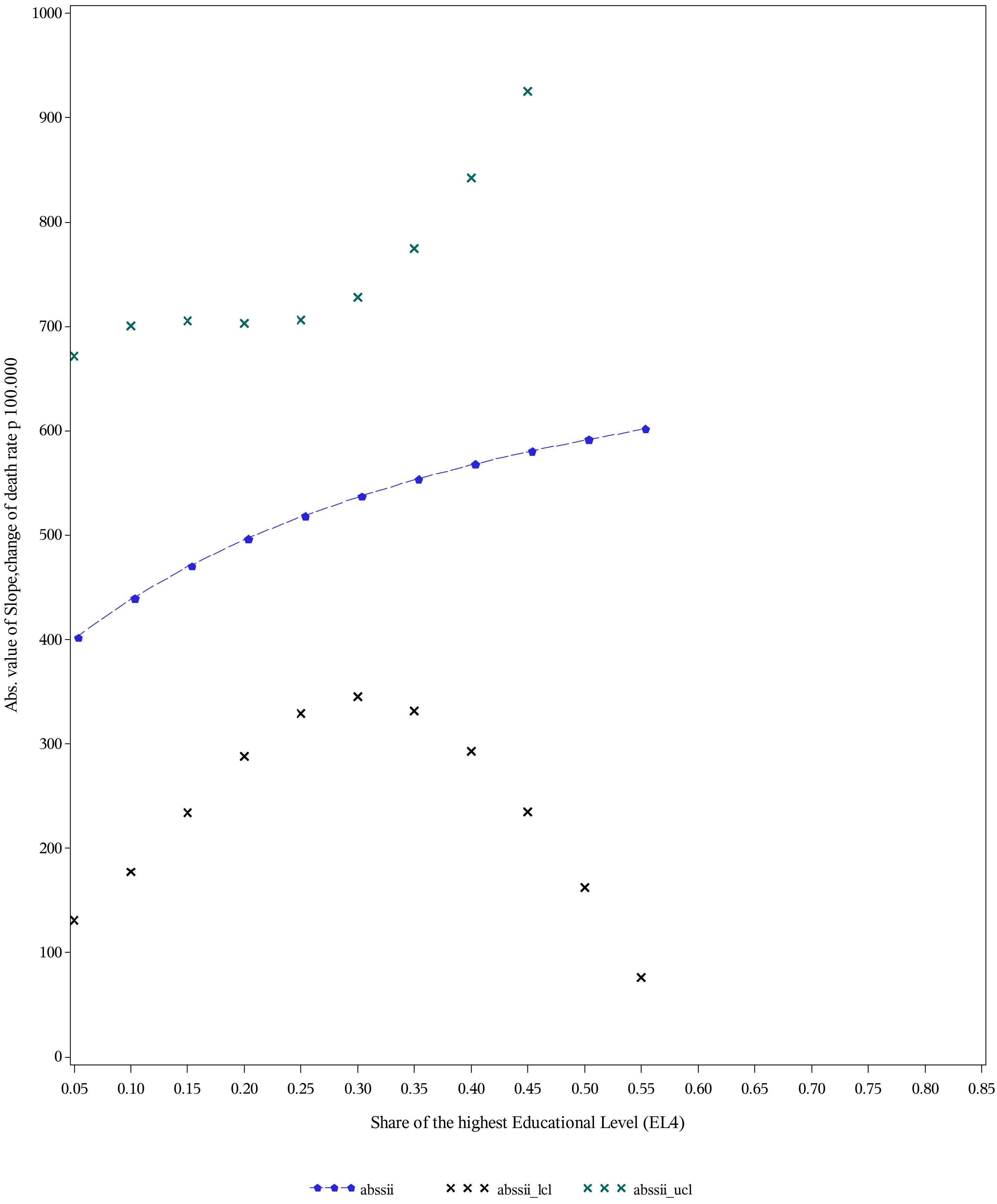

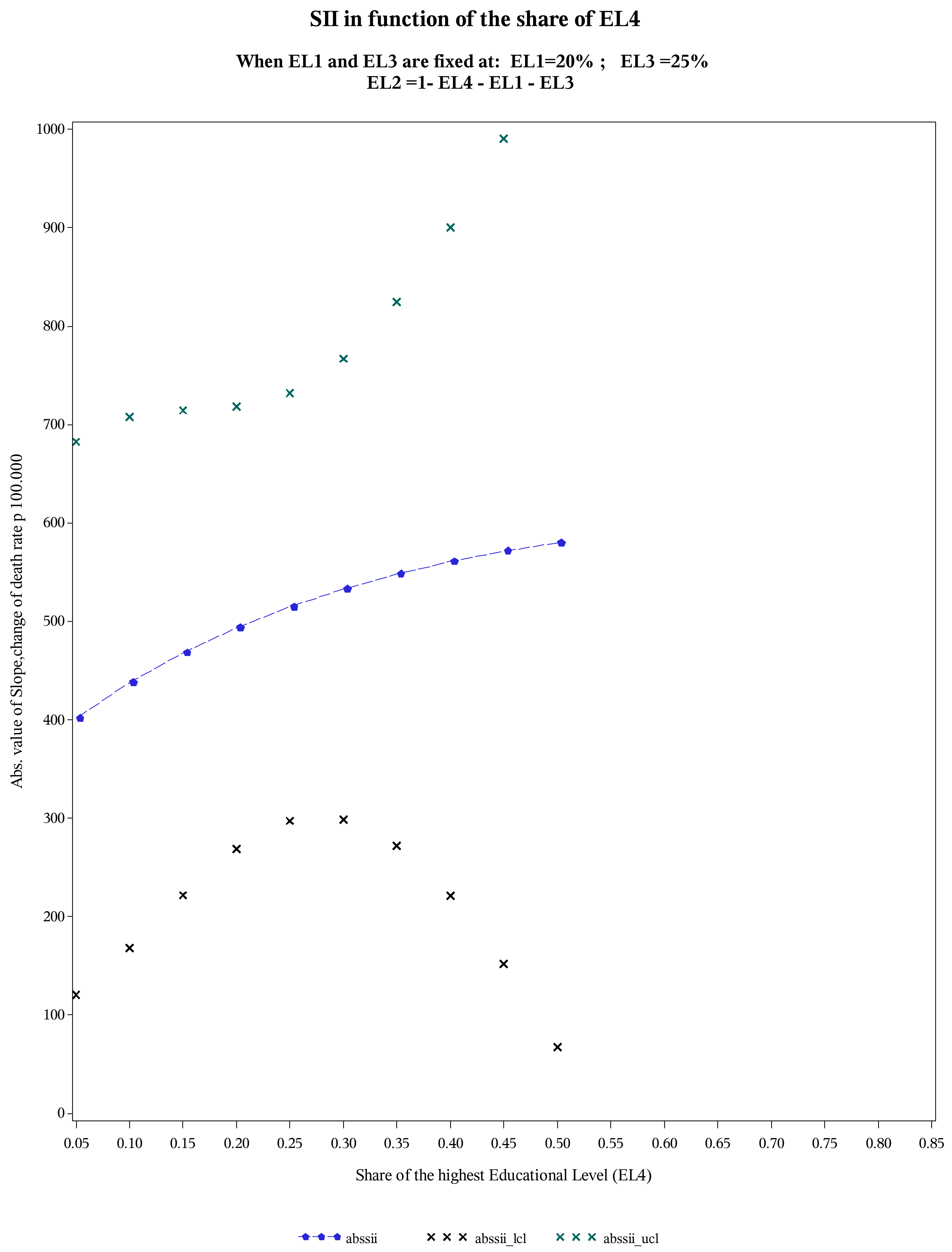

SII in function of the share of EL4
When EL1 and EL3 are fixed at: EL1=20% ; EL3 =25%
EL2 =1- EL4 - EL1 - EL3
Abs. value of Slope,change of death rate p 100.000
0
100
200
300
400
500
600
700
800
900
1000
0.05
0.10
0.15
0.20
0.25
0.30
0.35
0.40
0.45
0.50
0.55
0.60
0.65
0.70
0.75
0.80
0.85
Share of the highest Educational Level (EL4)
abssii
abssii_lcl
abssii_ucl

SII in function of the share of EL4

When EL1 and EL3 are fixed at: EL1=20% ; EL3 =30%
EL2 =1- EL4 - EL1 - EL3

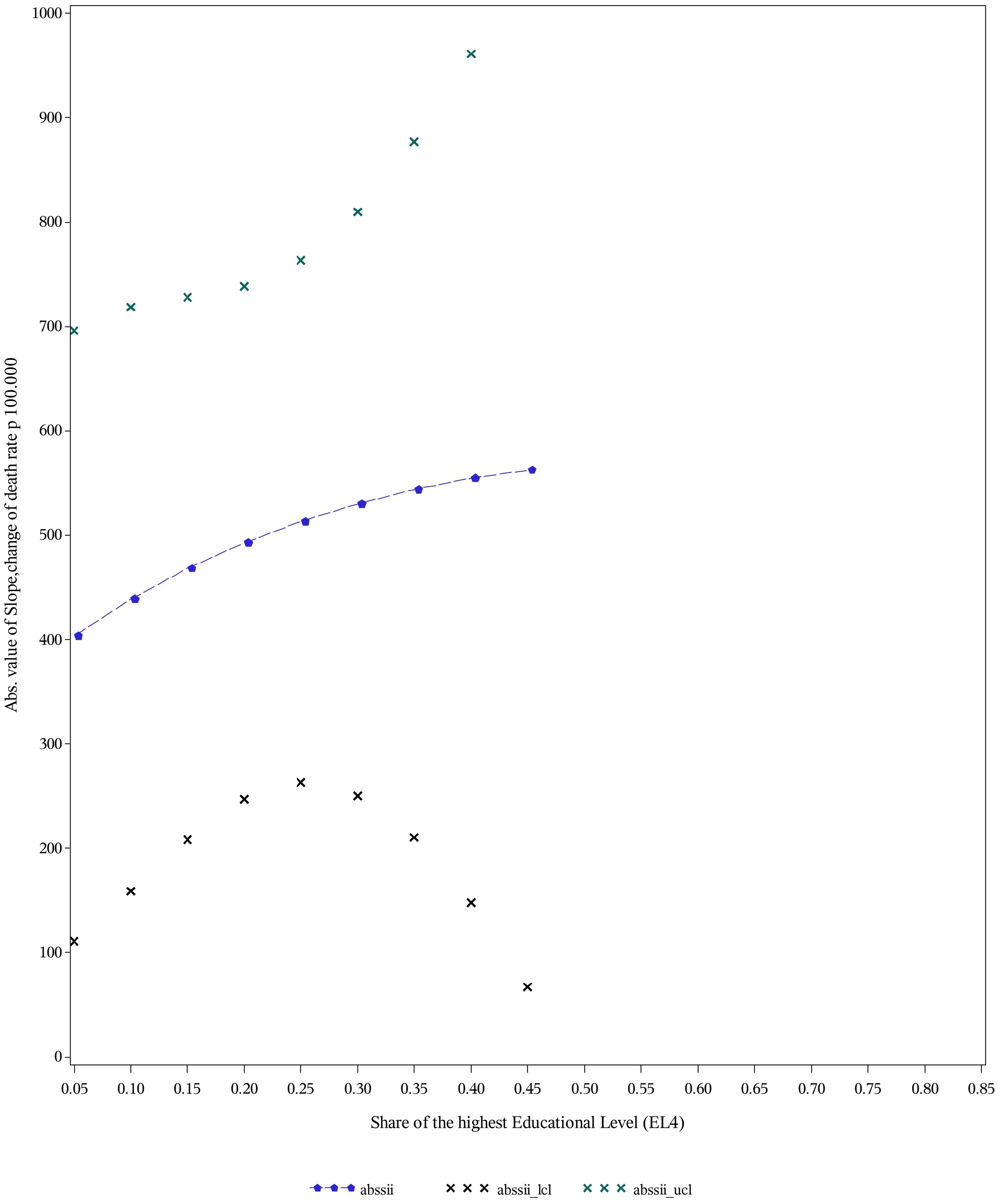

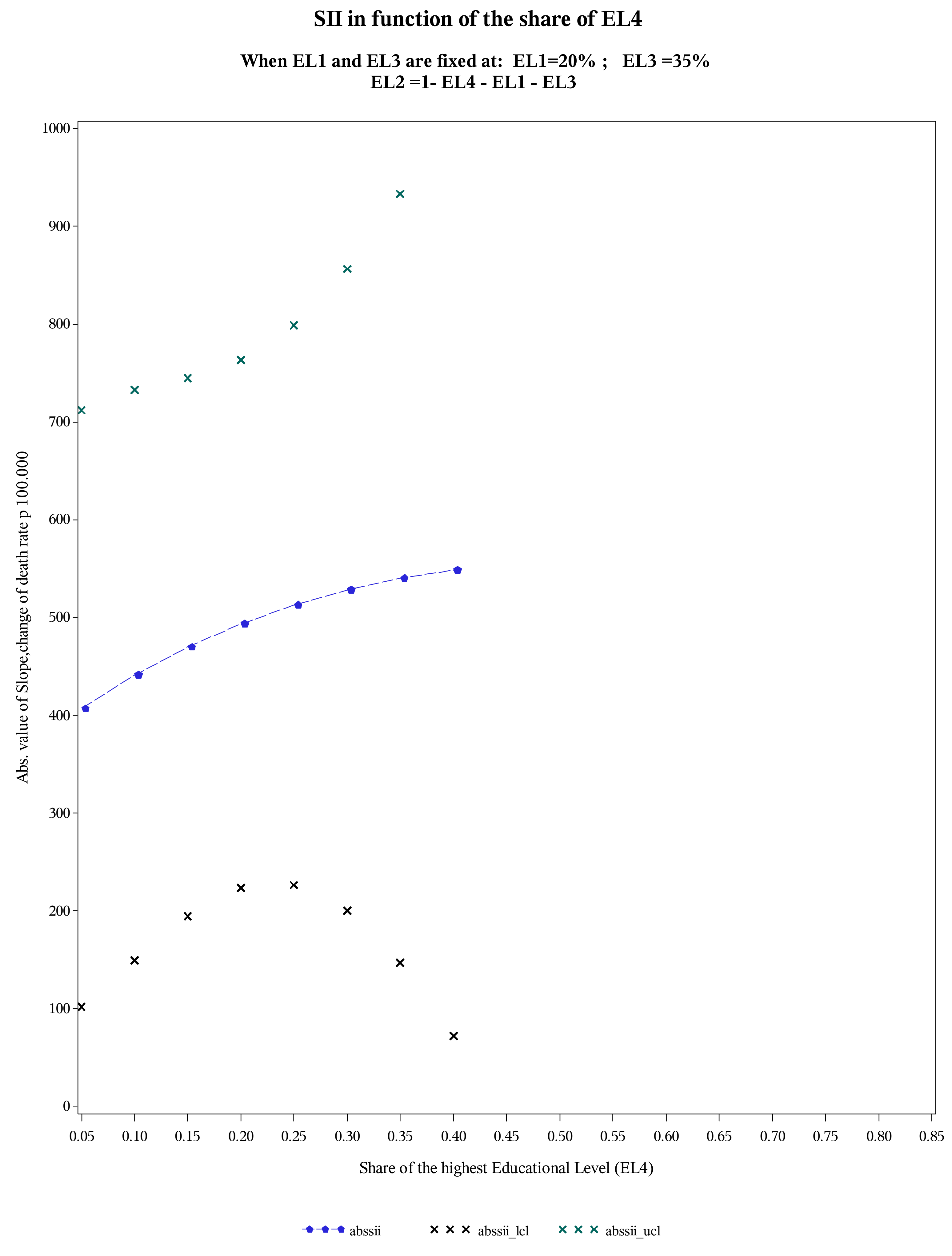
SII in function of the share of EL4
When EL1 and EL3 are fixed at: EL1=20% ; EL3 =35%
EL2 =1- EL4 - EL1 - EL3
Abs. value of Slope,change of death rate p 100.000
0
100
200
300
400
500
600
700
800
900
1000
0.05
0.10
0.15
0.20
0.25
0.30
0.35
0.40
0.45
0.50
0.55
0.60
0.65
0.70
0.75
0.80
0.85
Share of the highest Educational Level (EL4)
abssii
abssii_lcl
abssii_ucl

SII in function of the share of EL4

When EL1 and EL3 are fixed at: EL1=20% ; EL3 =40%
EL2 =1- EL4 - EL1 - EL3

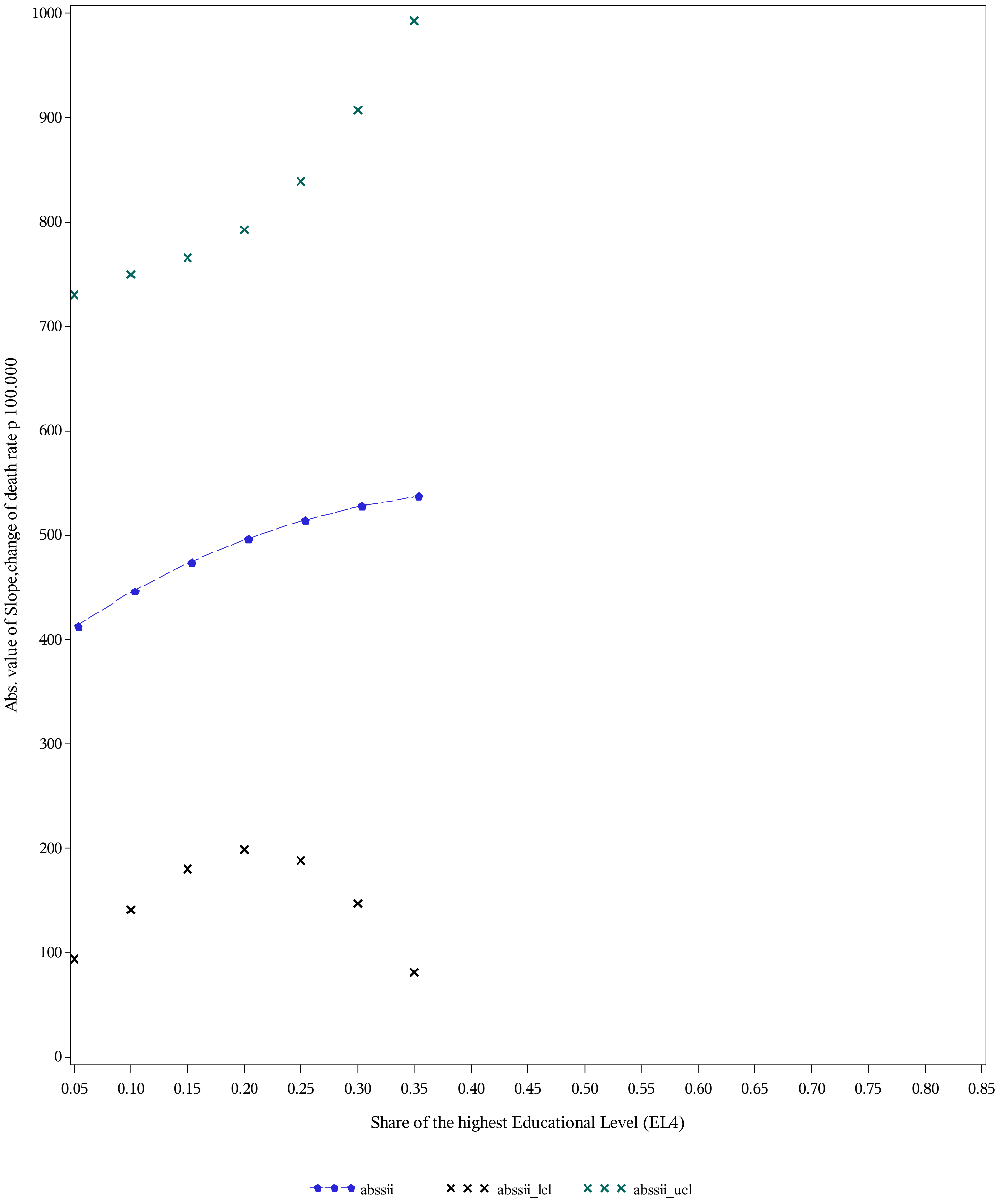

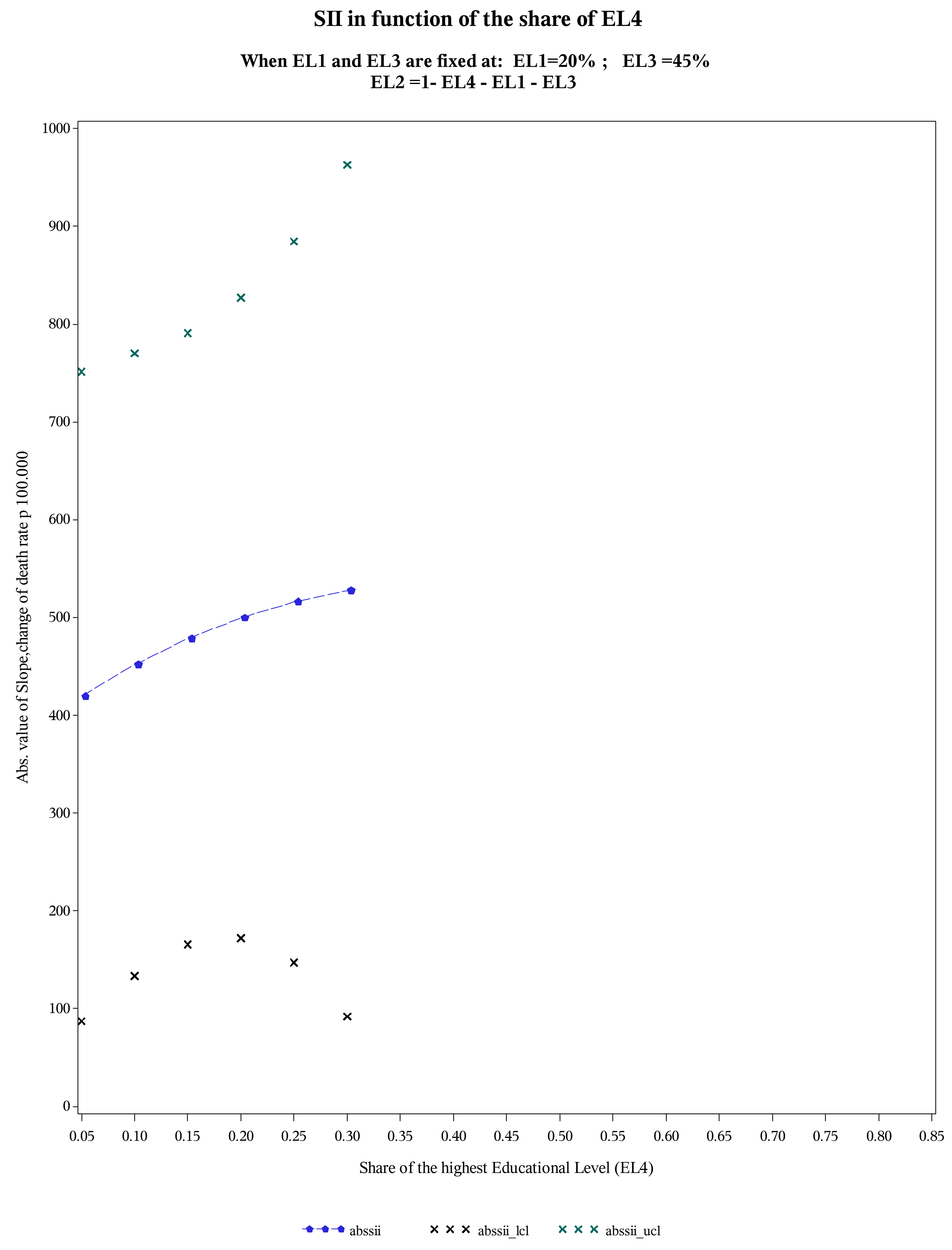

SII in function of the share of EL4
When EL1 and EL3 are fixed at: EL1=20% ; EL3 =45%
EL2 =1- EL4 - EL1 - EL3
Abs. value of Slope,change of death rate p 100.000
0
100
200
300
400
500
600
700
800
900
1000
0.05
0.10
0.15
0.20
0.25
0.30
0.35
0.40
0.45
0.50
0.55
0.60
0.65
0.70
0.75
0.80
0.85
Share of the highest Educational Level (EL4)
abssii
abssii_lcl
abssii_ucl

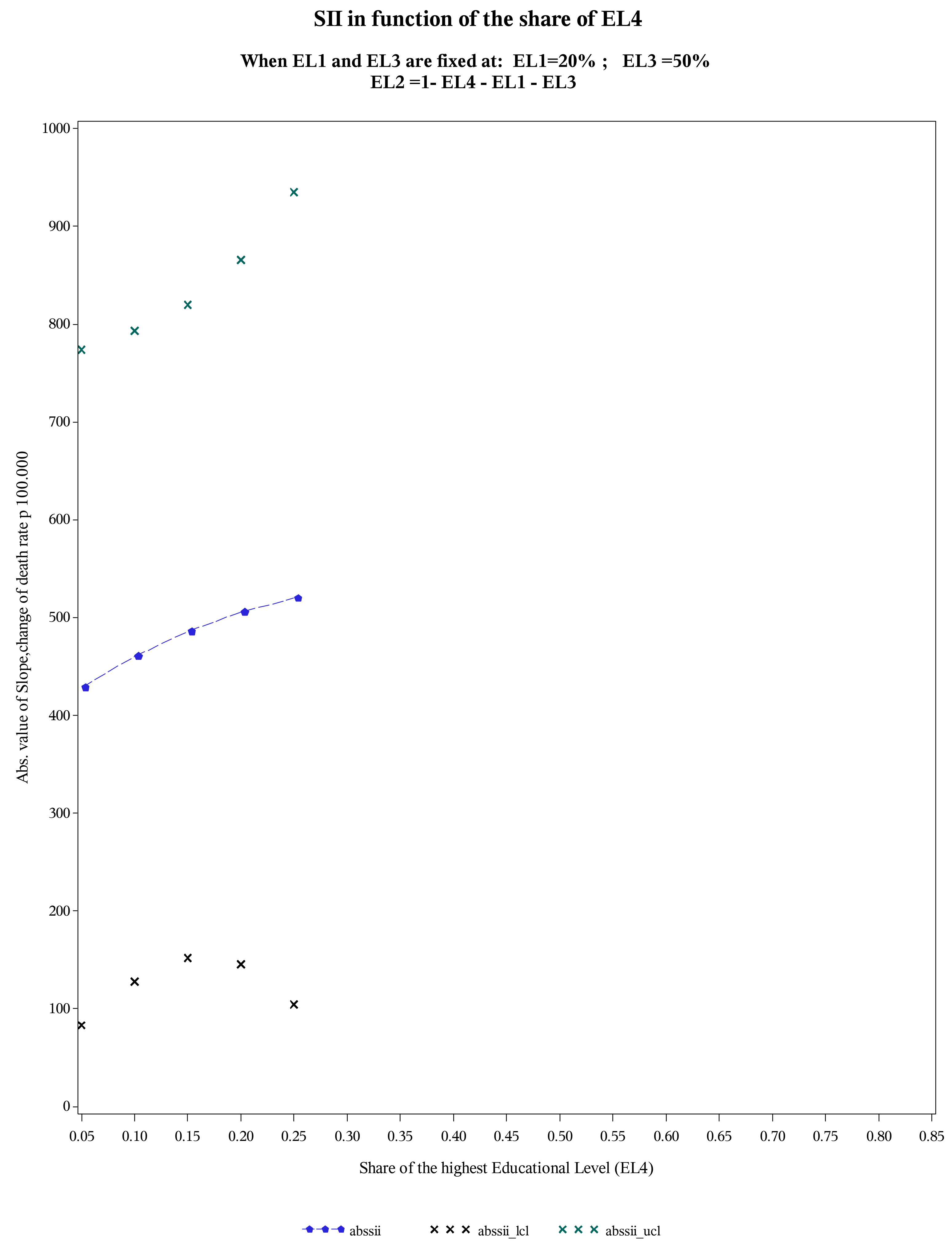
SII in function of the share of EL4
When EL1 and EL3 are fixed at: EL1=20% ; EL3 =50%
EL2 =1- EL4 - EL1 - EL3
Abs. value of Slope,change of death rate p 100.000
0
100
200
300
400
500
600
700
800
900
1000
0.05
0.10
0.15
0.20
0.25
0.30
0.35
0.40
0.45
0.50
0.55
0.60
0.65
0.70
0.75
0.80
0.85
Share of the highest Educational Level (EL4)
abssii
abssii_lcl
abssii_ucl

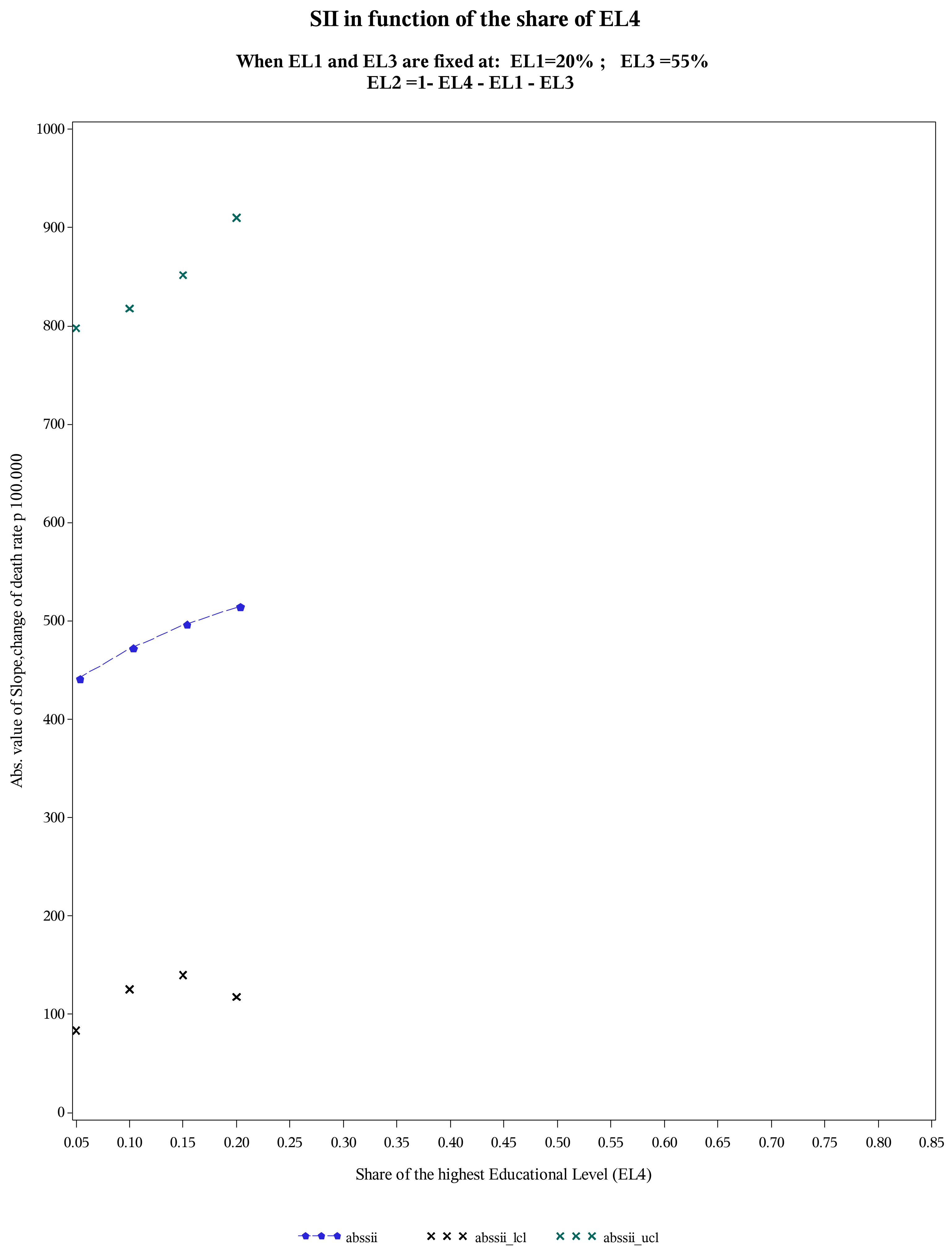
SII in function of the share of EL4
When EL1 and EL3 are fixed at: EL1=20% ; EL3 =55%
EL2 =1- EL4 - EL1 - EL3
Abs. value of Slope,change of death rate p 100.000
0
100
200
300
400
500
600
700
800
900
1000
0.05
0.10
0.15
0.20
0.25
0.30
0.35
0.40
0.45
0.50
0.55
0.60
0.65
0.70
0.75
0.80
0.85
Share of the highest Educational Level (EL4)
abssii
abssii_lcl
abssii_ucl

SII in function of the share of EL4

When EL1 and EL3 are fixed at: EL1=20% ; EL3 =60%
EL2 =1- EL4 - EL1 - EL3

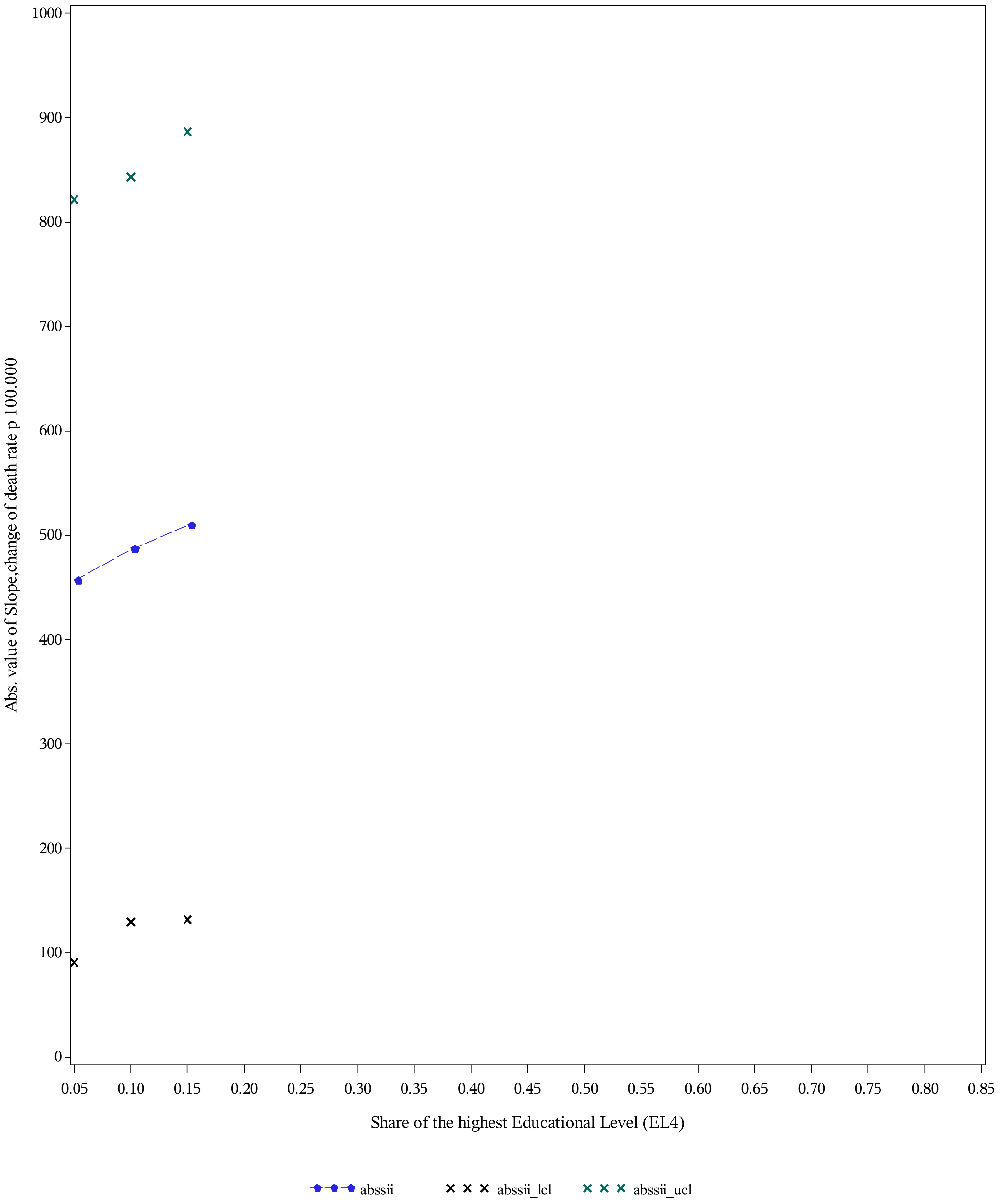

SII in function of the share of EL4

When EL1 and EL3 are fixed at: EL1=20% ; EL3 =65%
EL2 =1- EL4 - EL1 - EL3

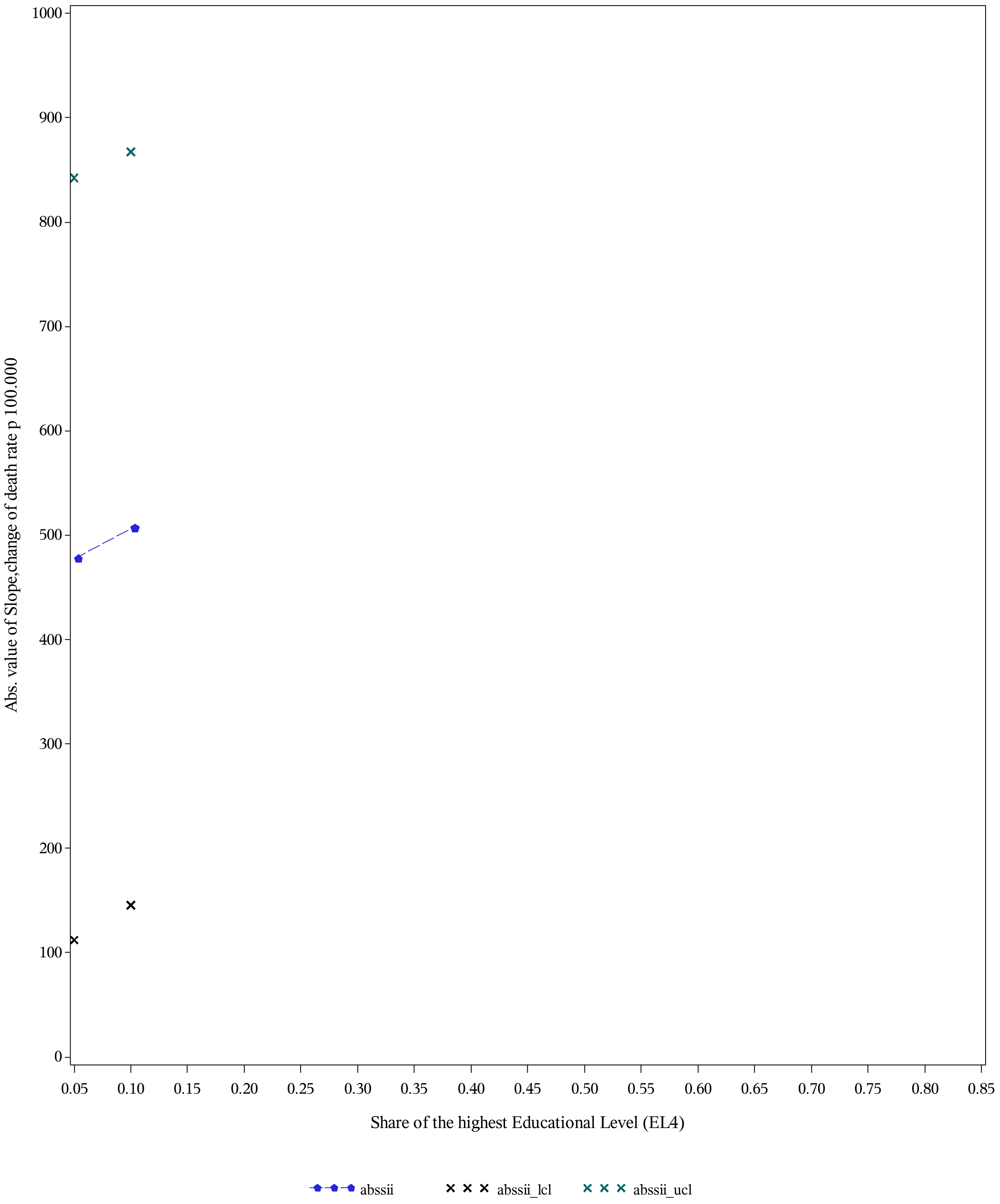

SII in function of the share of EL4

When EL1 and EL3 are fixed at: EL1=25% ; EL3 =5%
EL2 =1- EL4 - EL1 - EL3

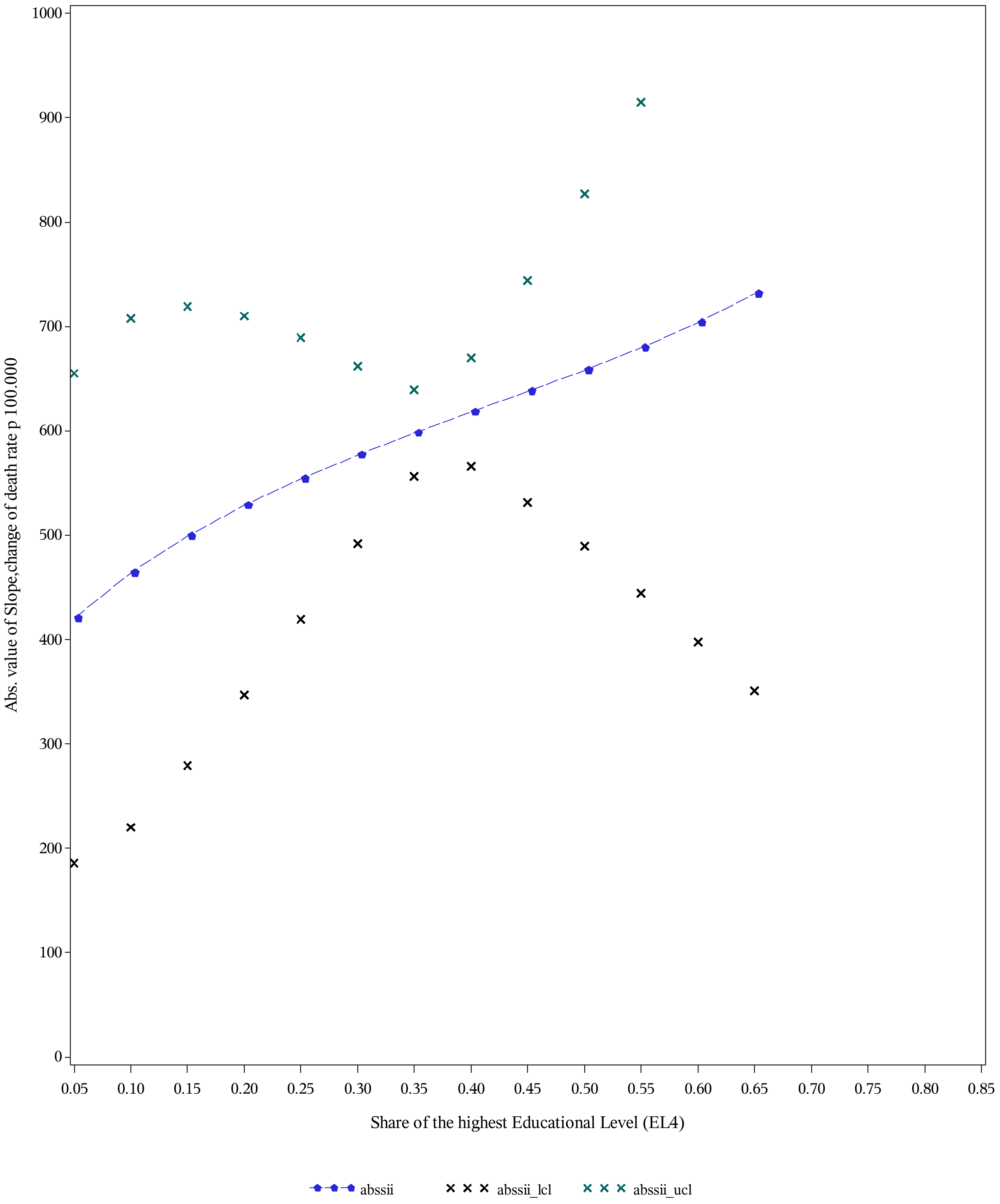

SII in function of the share of EL4

When EL1 and EL3 are fixed at: EL1=25% ; EL3 =10%
EL2 =1- EL4 - EL1 - EL3

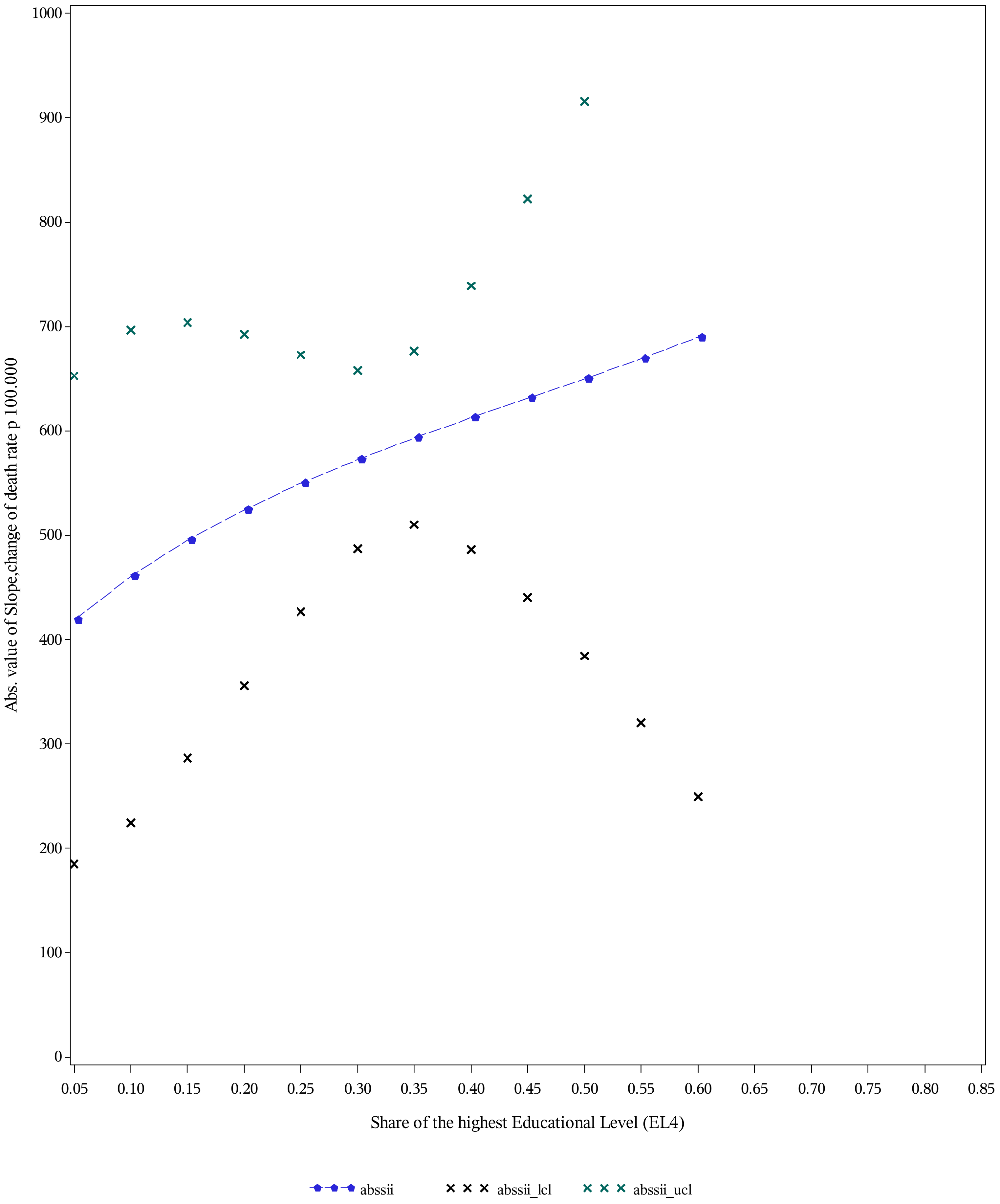

SII in function of the share of EL4

When EL1 and EL3 are fixed at: EL1=25% ; EL3 =15%
EL2 =1- EL4 - EL1 - EL3

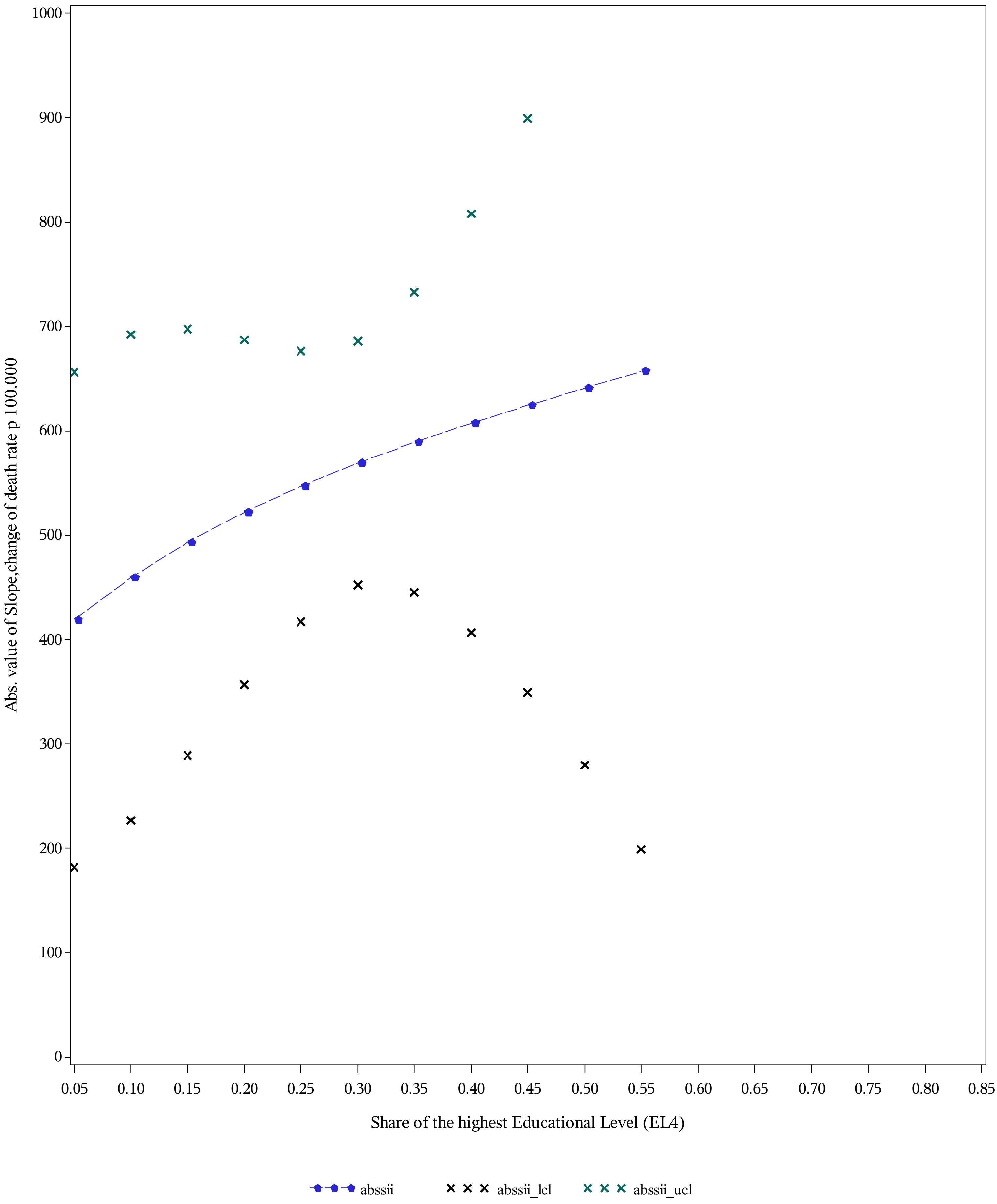

SII in function of the share of EL4

When EL1 and EL3 are fixed at: EL1=25% ; EL3 =20%
EL2 =1- EL4 - EL1 - EL3

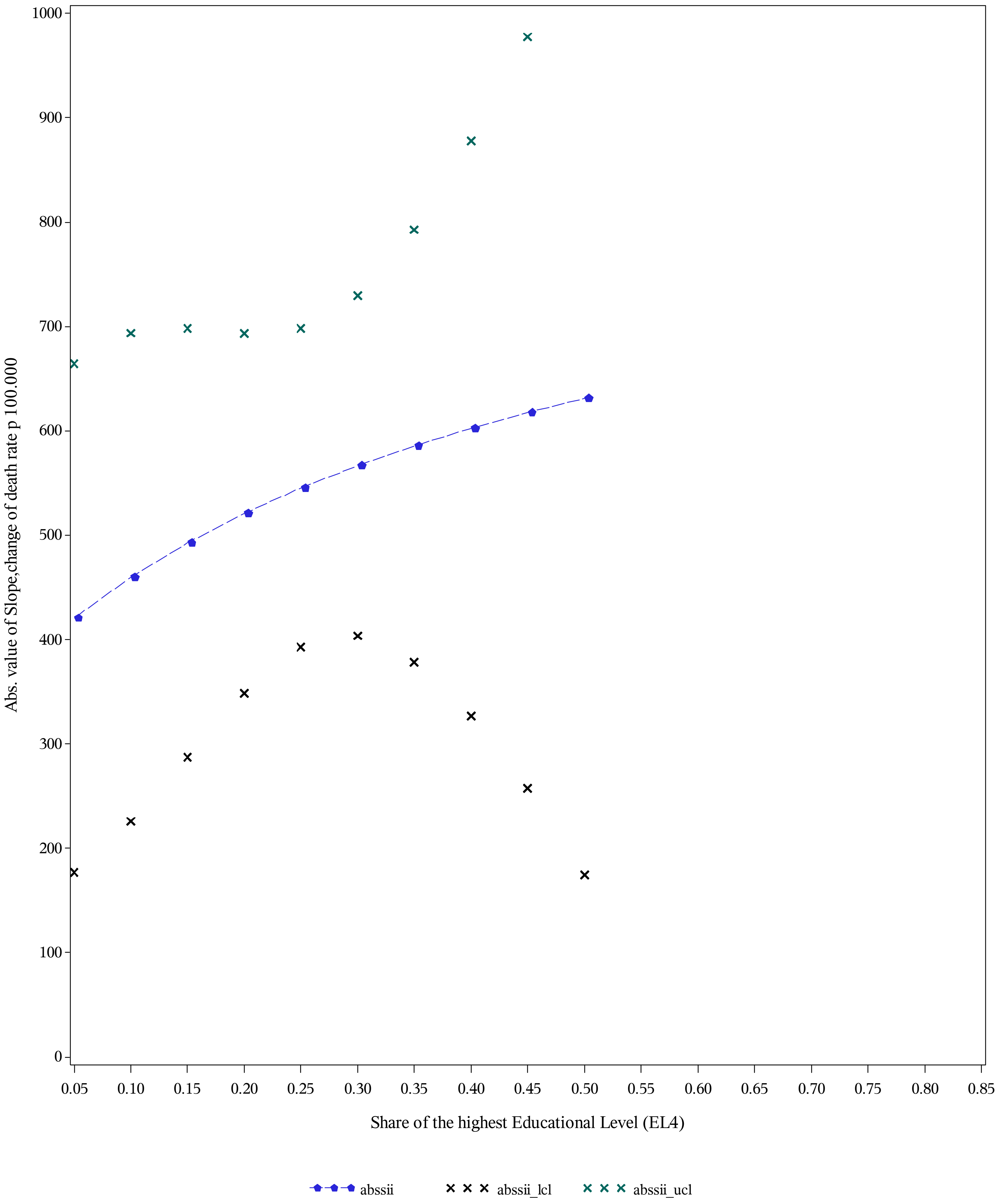

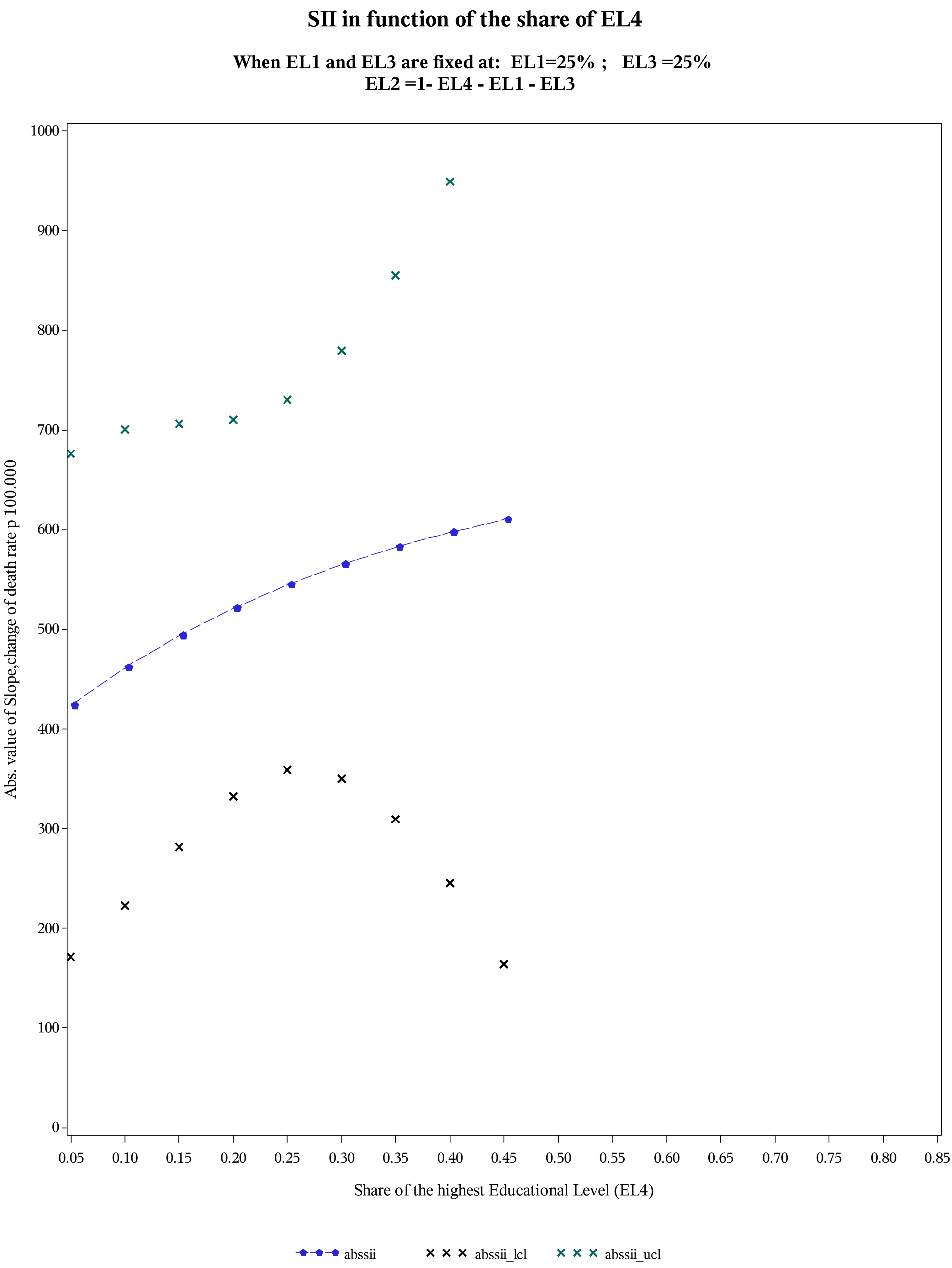
SII in function of the share of EL4
When EL1 and EL3 are fixed at: EL1=25% ; EL3 =25%
EL2 =1- EL4 - EL1 - EL3
Abs. value of Slope,change of death rate p 100.000
1000
900
800
700
600
500
400
300
200
100
0
0.05
0.10
0.15
0.20
0.25
0.30
0.35
0.40
0.45
0.50
0.55
0.60
0.65
0.70
0.75
0.80
0.85
Share of the highest Educational Level (EL4)
abssii
abssii_lcl
abssii_ucl

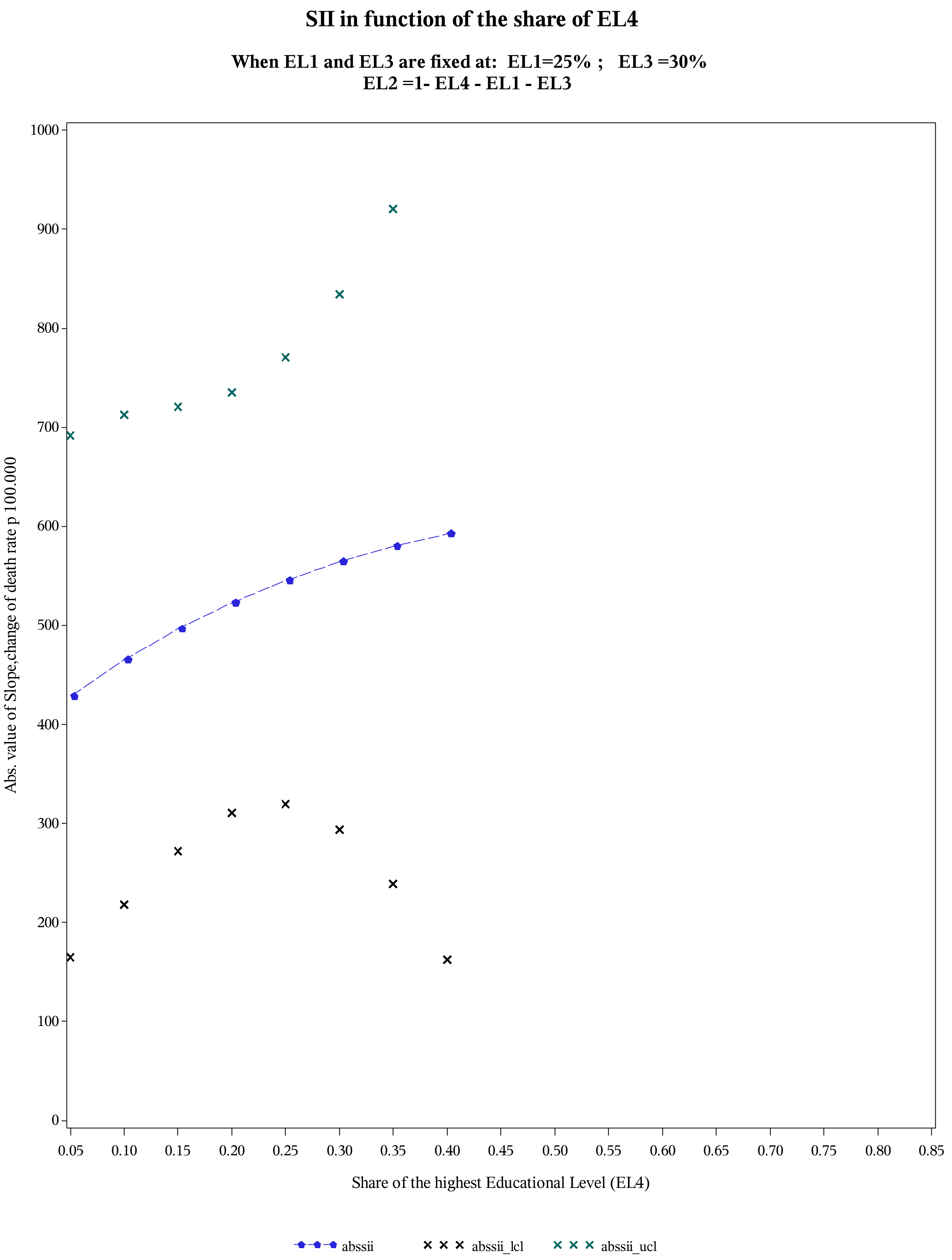
SII in function of the share of EL4
When EL1 and EL3 are fixed at: EL1=25% ; EL3 =30%
EL2 =1- EL4 - EL1 - EL3
Abs. value of Slope,change of death rate p 100.000
Share of the highest Educational Level (EL4)
0
100
200
300
400
500
600
700
800
900
1000
0.05
0.10
0.15
0.20
0.25
0.30
0.35
0.40
0.45
0.50
0.55
0.60
0.65
0.70
0.75
0.80
0.85
abssii
abssii_lcl
abssii_ucl

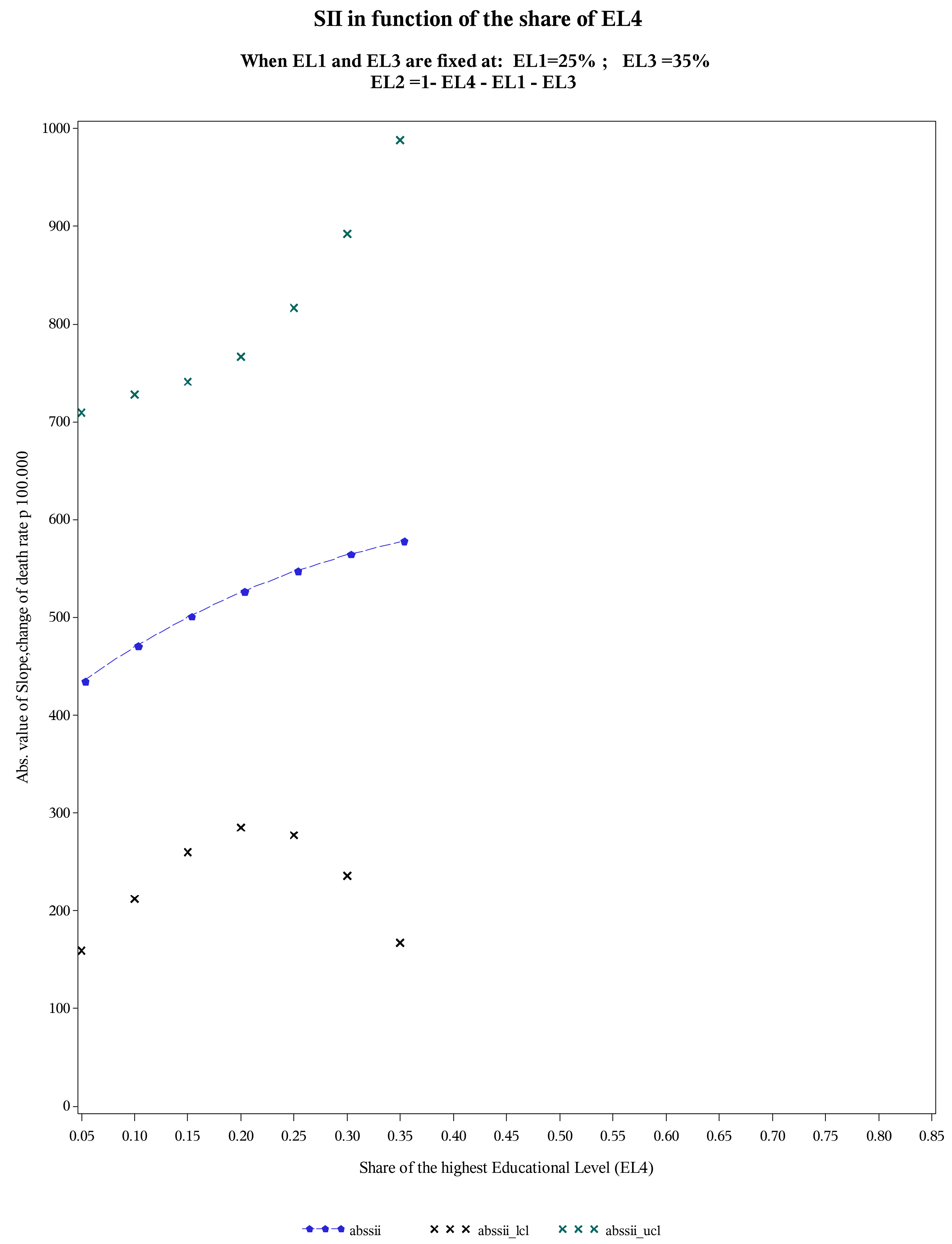

SII in function of the share of EL4
When EL1 and EL3 are fixed at: EL1=25% ; EL3 =35%
EL2 =1- EL4 - EL1 - EL3
Abs. value of Slope,change of death rate p 100.000
Share of the highest Educational Level (EL4)
0
100
200
300
400
500
600
700
800
900
1000
0.05
0.10
0.15
0.20
0.25
0.30
0.35
0.40
0.45
0.50
0.55
0.60
0.65
0.70
0.75
0.80
0.85
abssii
abssii_lcl
abssii_ucl

SII in function of the share of EL4

When EL1 and EL3 are fixed at: EL1=25% ; EL3 =40%
EL2 =1- EL4 - EL1 - EL3

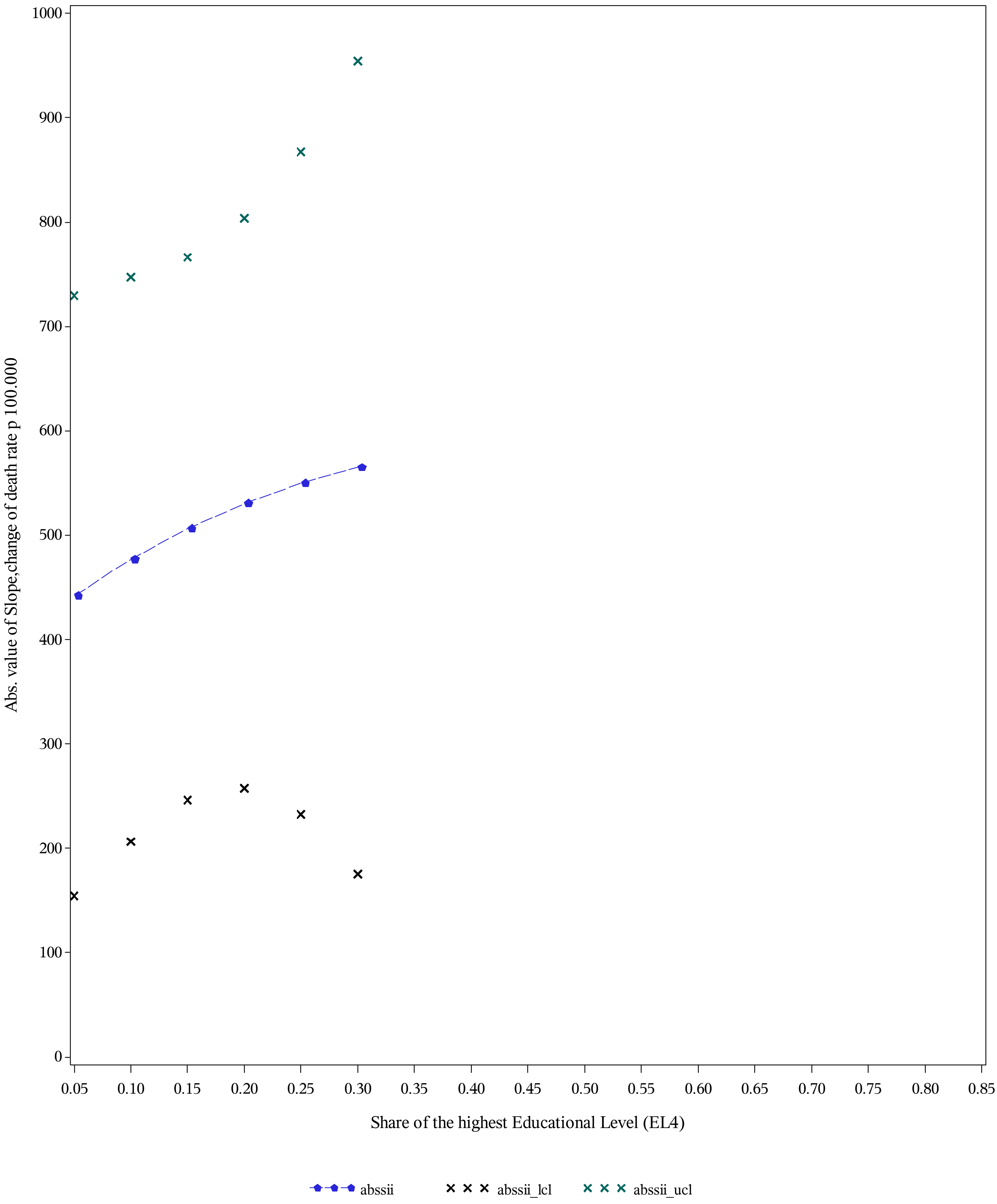

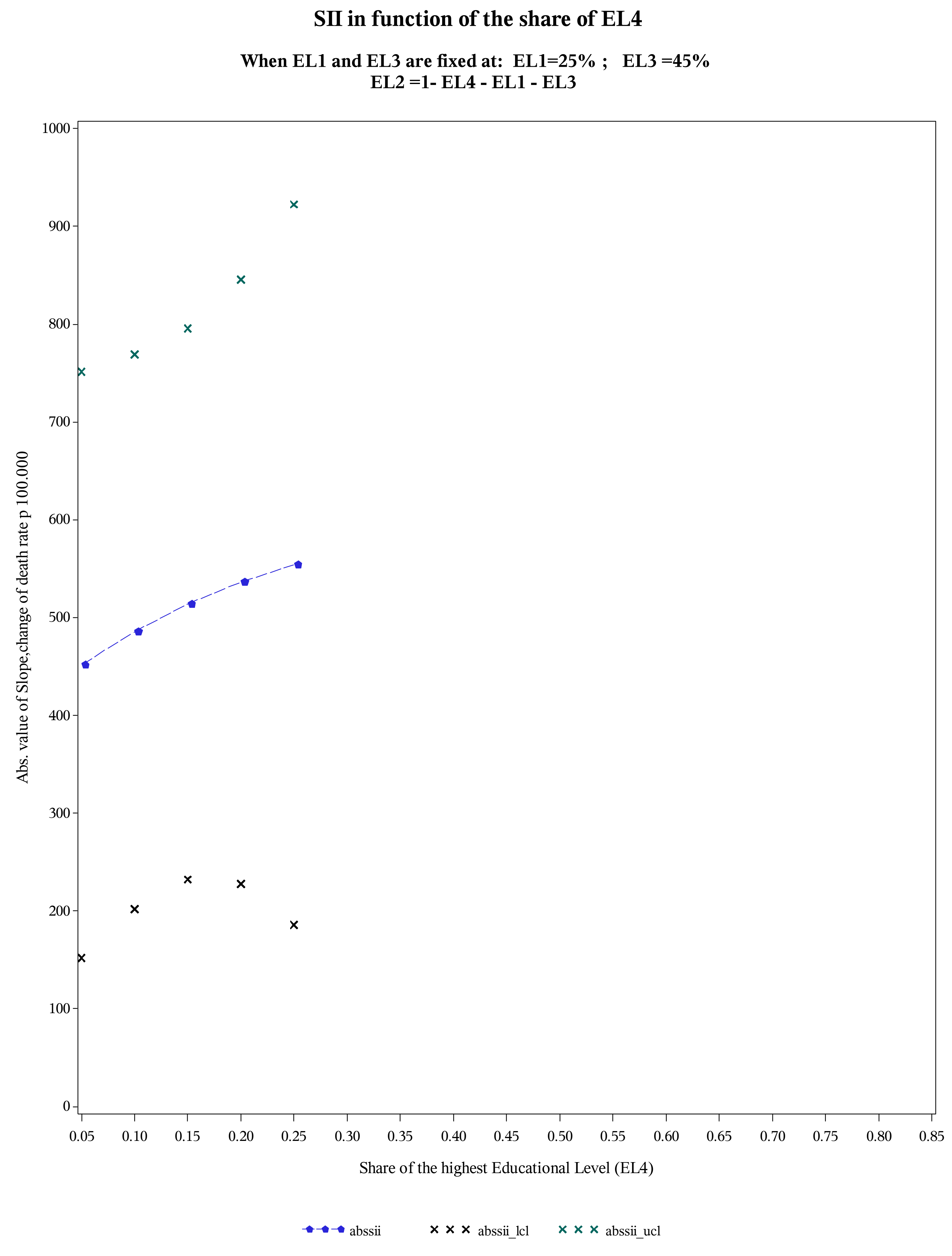
SII in function of the share of EL4
When EL1 and EL3 are fixed at: EL1=25% ; EL3 =45%
EL2 =1- EL4 - EL1 - EL3
Abs. value of Slope,change of death rate p 100.000
1000
900
800
700
600
500
400
300
200
100
0
0.05
0.10
0.15
0.20
0.25
0.30
0.35
0.40
0.45
0.50
0.55
0.60
0.65
0.70
0.75
0.80
0.85
Share of the highest Educational Level (EL4)
abssii
abssii_lcl
abssii_ucl

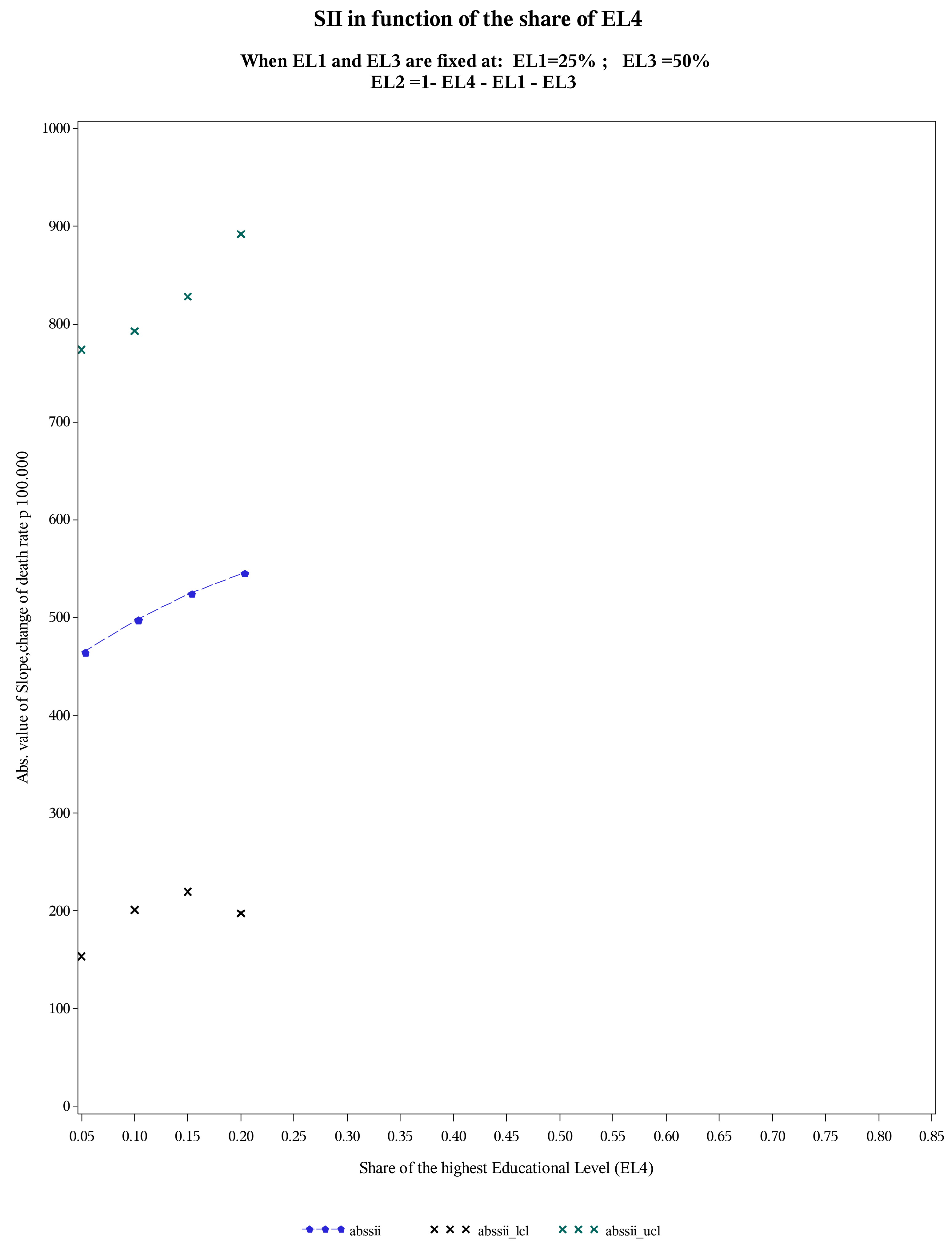

SII in function of the share of EL4
When EL1 and EL3 are fixed at: EL1=25% ; EL3 =50%
EL2 =1- EL4 - EL1 - EL3
Abs. value of Slope,change of death rate p 100.000
1000
900
800
700
600
500
400
300
200
100
0
0.05
0.10
0.15
0.20
0.25
0.30
0.35
0.40
0.45
0.50
0.55
0.60
0.65
0.70
0.75
0.80
0.85
Share of the highest Educational Level (EL4)
abssii
abssii_lcl
abssii_ucl

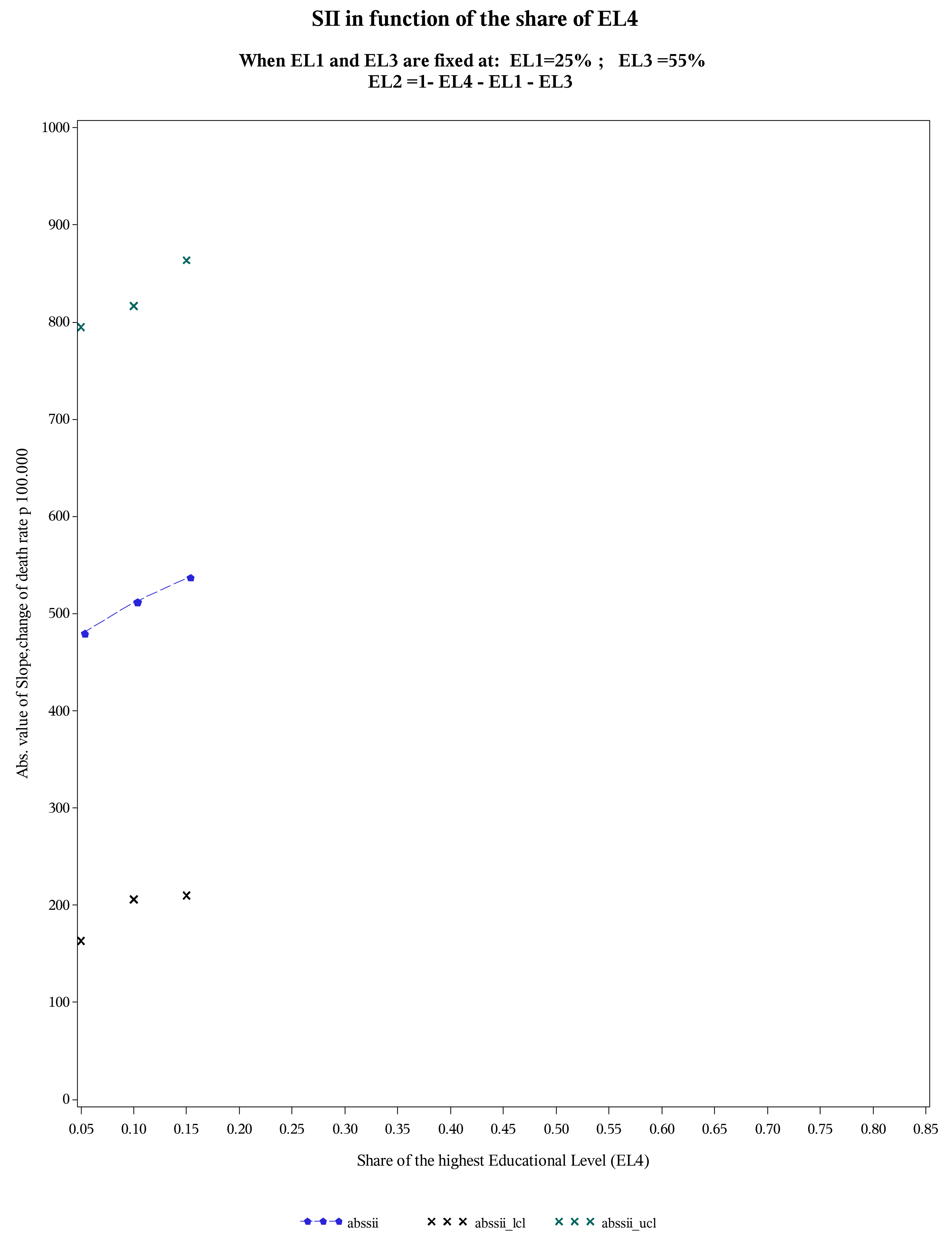

SII in function of the share of EL4
When EL1 and EL3 are fixed at: EL1=25% ; EL3 =55%
EL2 =1- EL4 - EL1 - EL3
Abs. value of Slope,change of death rate p 100.000
0
100
200
300
400
500
600
700
800
900
1000
0.05
0.10
0.15
0.20
0.25
0.30
0.35
0.40
0.45
0.50
0.55
0.60
0.65
0.70
0.75
0.80
0.85
Share of the highest Educational Level (EL4)
abssii
abssii_lcl
abssii_ucl

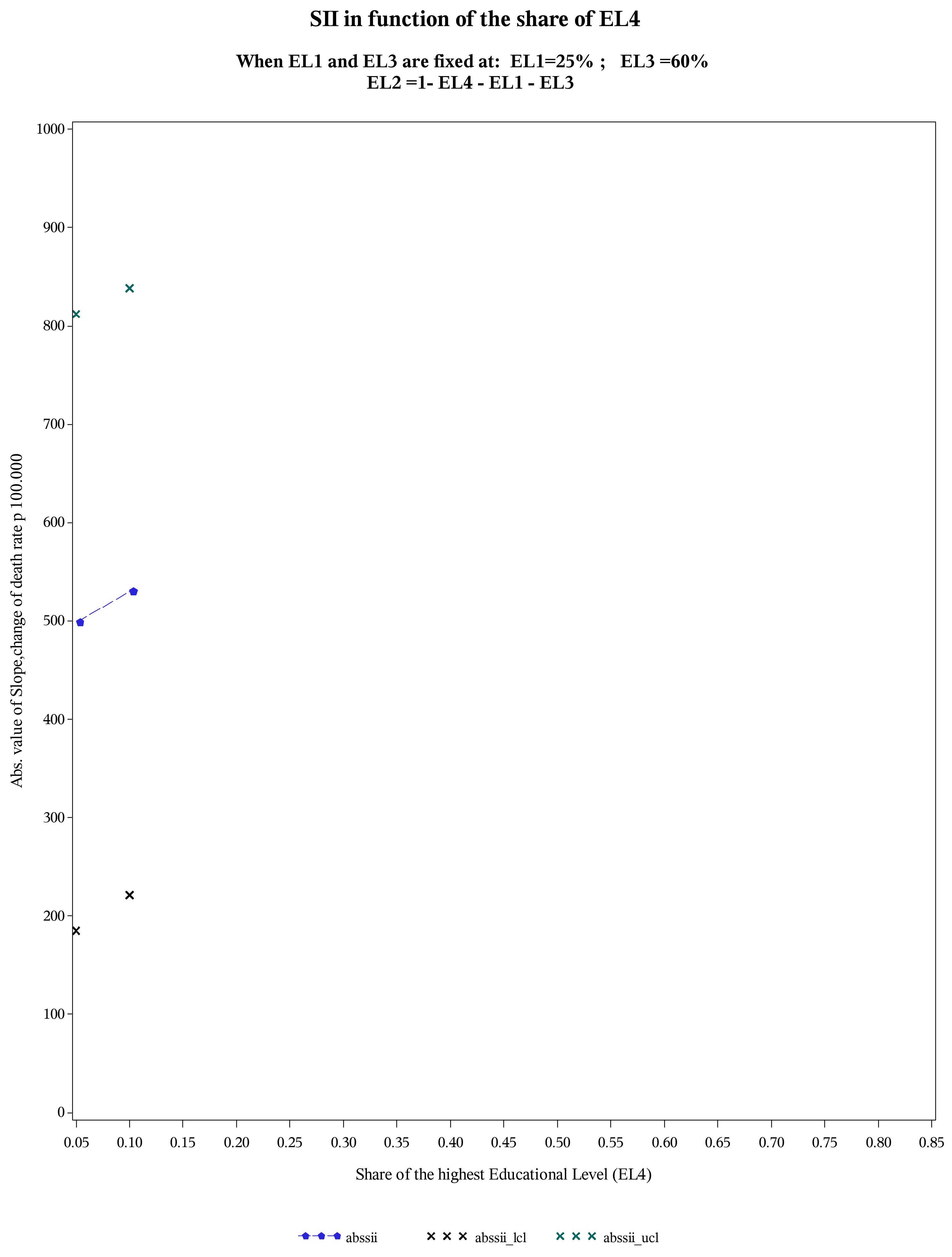

SII in function of the share of EL4
When EL1 and EL3 are fixed at: EL1=25% ; EL3 =60%
EL2 =1- EL4 - EL1 - EL3
Abs. value of Slope,change of death rate p 100.000
0
100
200
300
400
500
600
700
800
900
1000
0.05
0.10
0.15
0.20
0.25
0.30
0.35
0.40
0.45
0.50
0.55
0.60
0.65
0.70
0.75
0.80
0.85
Share of the highest Educational Level (EL4)
abssii
abssii_lcl
abssii_ucl

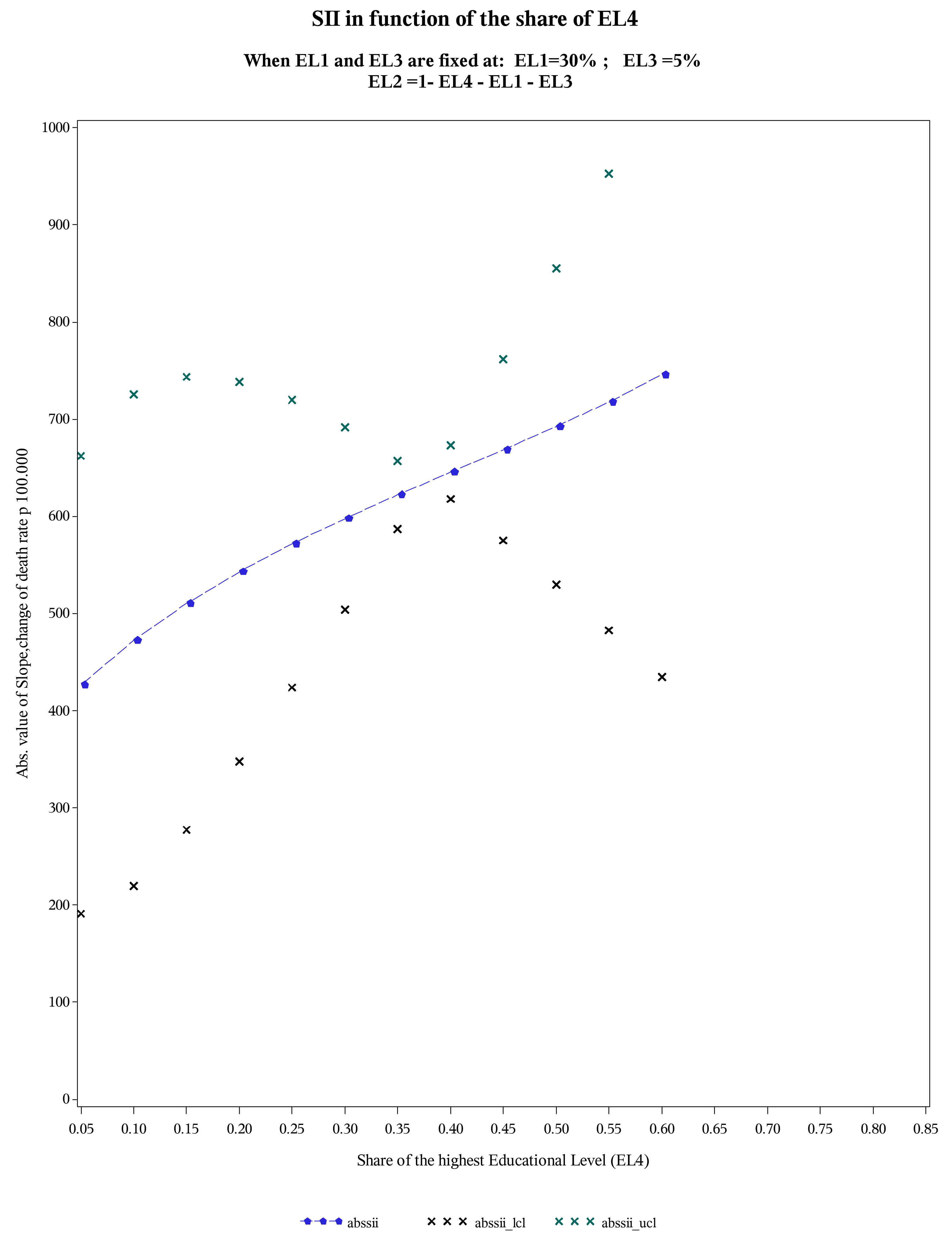
SII in function of the share of EL4
When EL1 and EL3 are fixed at: EL1=30% ; EL3 =5%
EL2 =1- EL4 - EL1 - EL3
Abs. value of Slope,change of death rate p 100.000
0
100
200
300
400
500
600
700
800
900
1000
0.05
0.10
0.15
0.20
0.25
0.30
0.35
0.40
0.45
0.50
0.55
0.60
0.65
0.70
0.75
0.80
0.85
Share of the highest Educational Level (EL4)
abssii
abssii_lcl
abssii_ucl

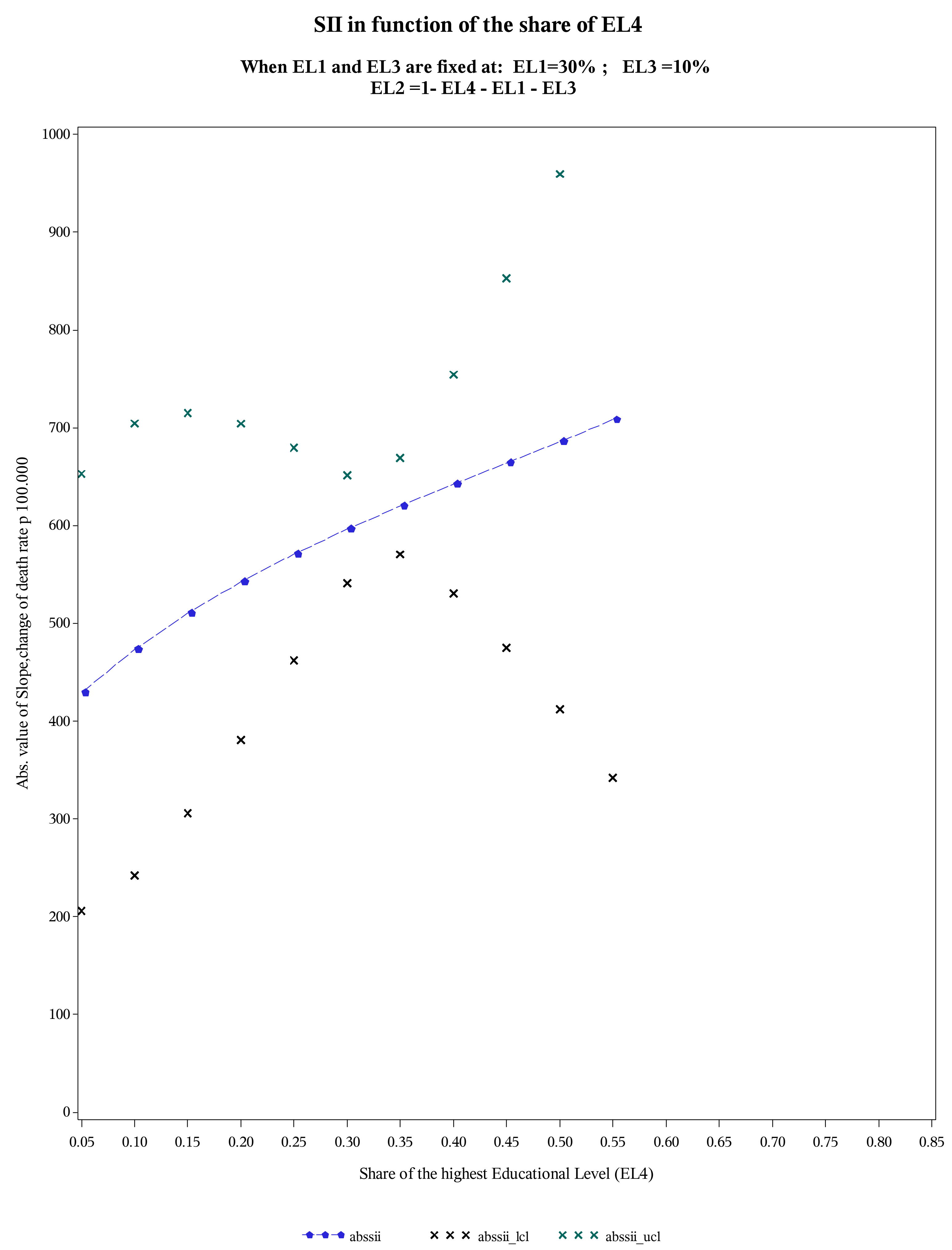
SII in function of the share of EL4
When EL1 and EL3 are fixed at: EL1=30% ; EL3 =10%
EL2 =1- EL4 - EL1 - EL3
Abs. value of Slope,change of death rate p 100.000
0
100
200
300
400
500
600
700
800
900
1000
0.05
0.10
0.15
0.20
0.25
0.30
0.35
0.40
0.45
0.50
0.55
0.60
0.65
0.70
0.75
0.80
0.85
Share of the highest Educational Level (EL4)
abssii
abssii_lcl
abssii_ucl

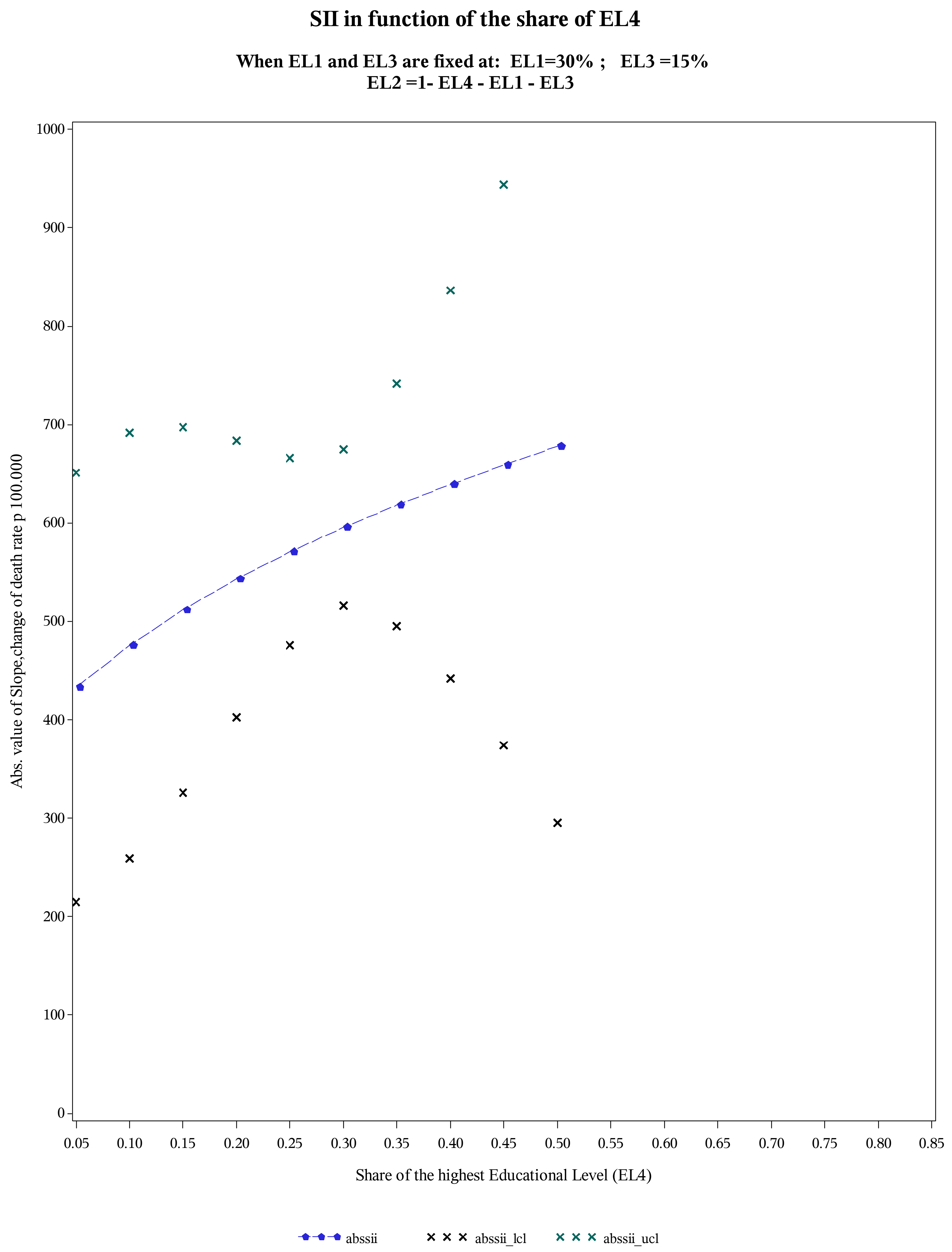
SII in function of the share of EL4
When EL1 and EL3 are fixed at: EL1=30% ; EL3 =15%
EL2 =1- EL4 - EL1 - EL3
Abs. value of Slope,change of death rate p 100.000
0
100
200
300
400
500
600
700
800
900
1000
0.05
0.10
0.15
0.20
0.25
0.30
0.35
0.40
0.45
0.50
0.55
0.60
0.65
0.70
0.75
0.80
0.85
Share of the highest Educational Level (EL4)
abssii
abssii_lcl
abssii_ucl

SII in function of the share of EL4

When EL1 and EL3 are fixed at: EL1=30% ; EL3 =20%
EL2 =1- EL4 - EL1 - EL3

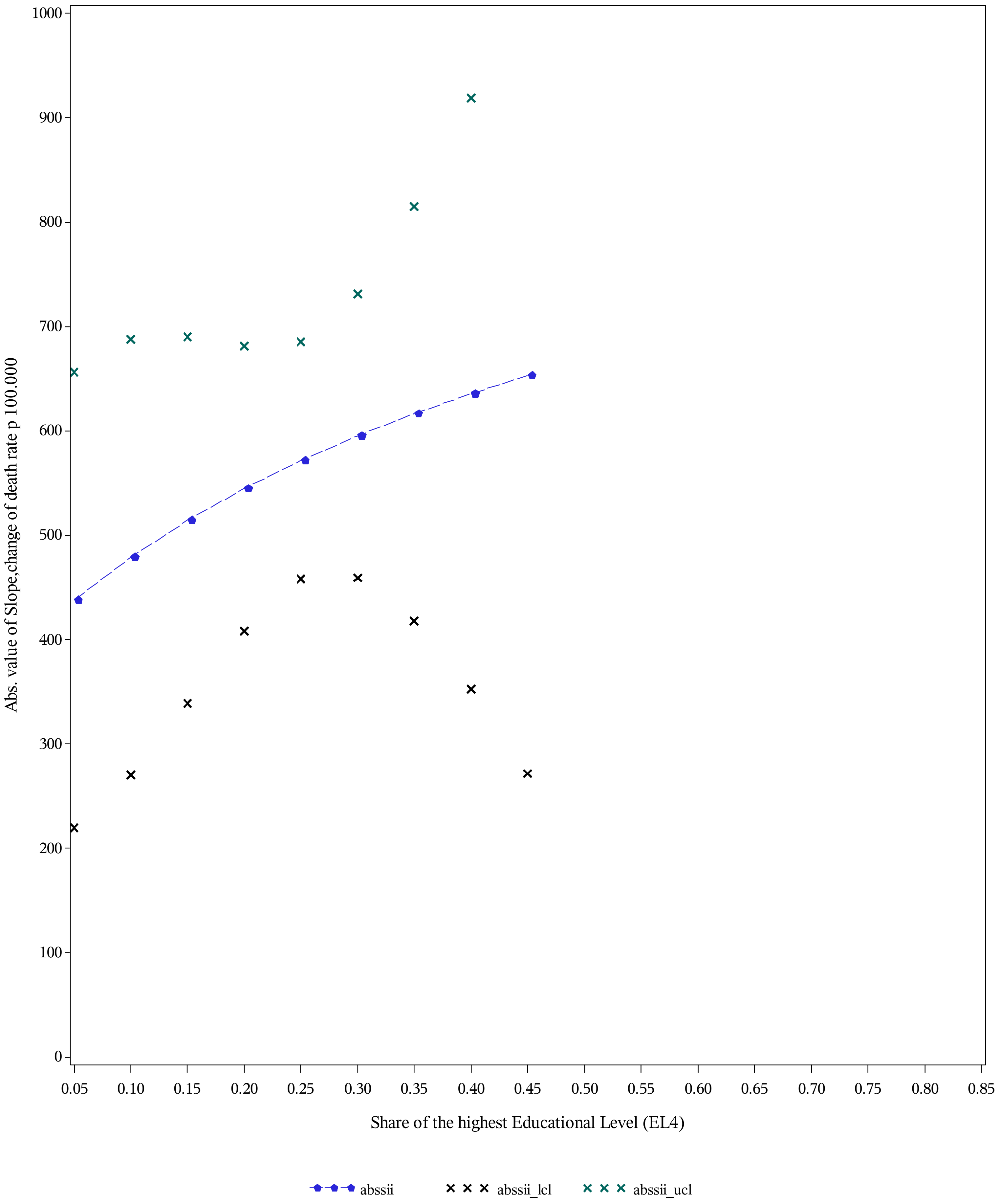

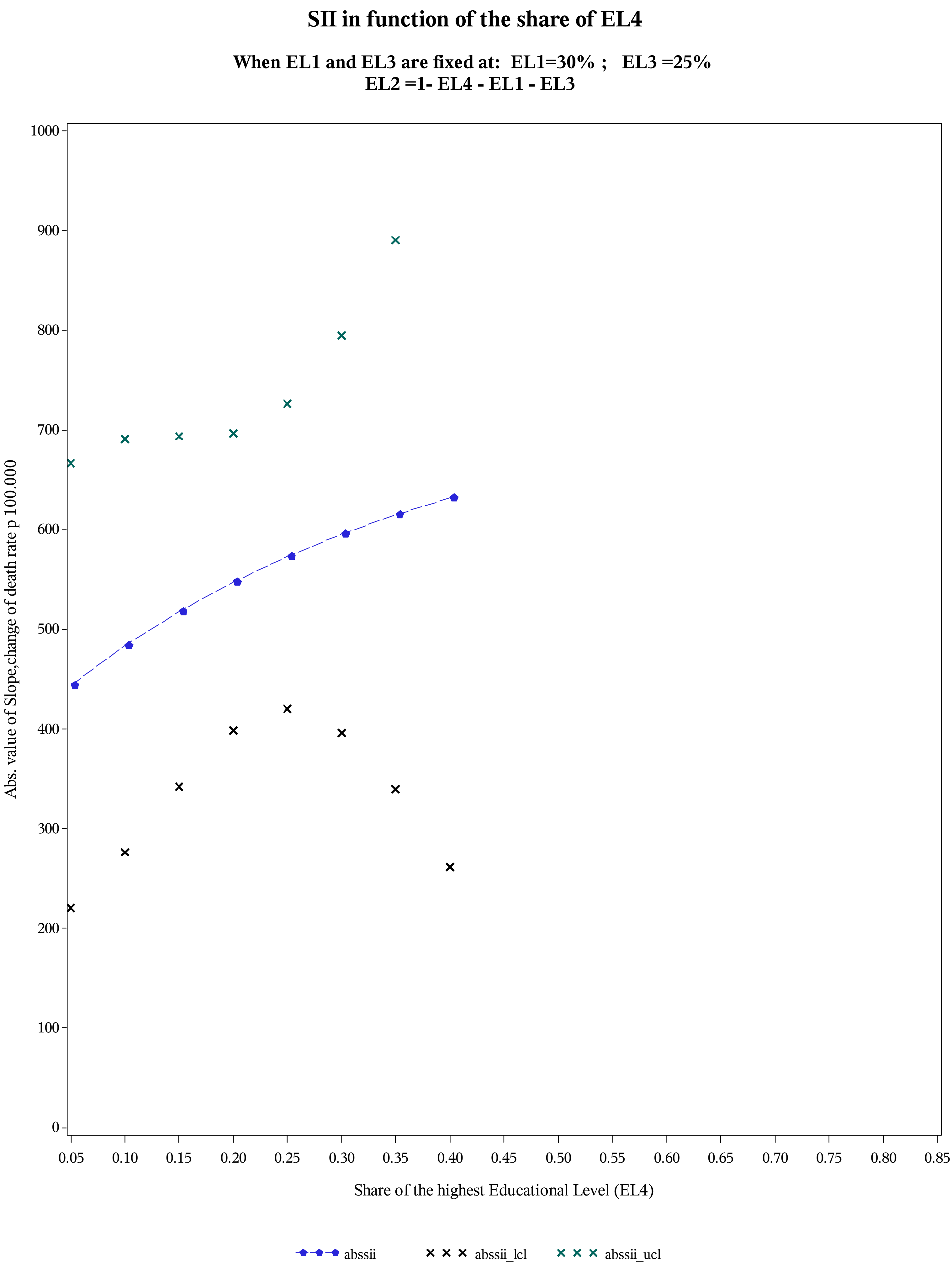
SII in function of the share of EL4
When EL1 and EL3 are fixed at: EL1=30% ; EL3 =25%
EL2 =1- EL4 - EL1 - EL3
Abs. value of Slope,change of death rate p 100.000
Share of the highest Educational Level (EL4)
abssii
abssii_lcl
abssii_ucl

SII in function of the share of EL4

When EL1 and EL3 are fixed at: EL1=30% ; EL3 =30%
EL2 =1- EL4 - EL1 - EL3

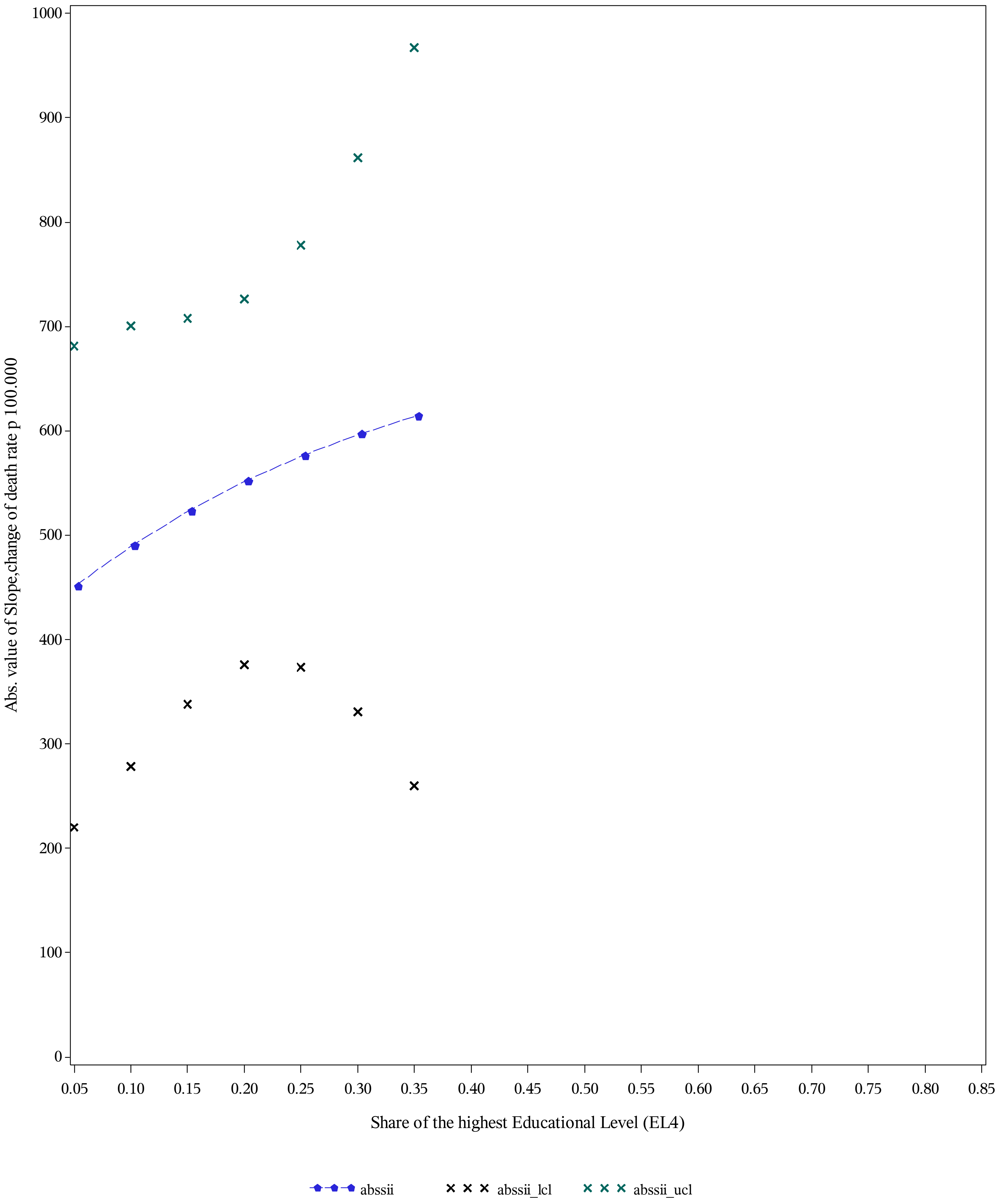

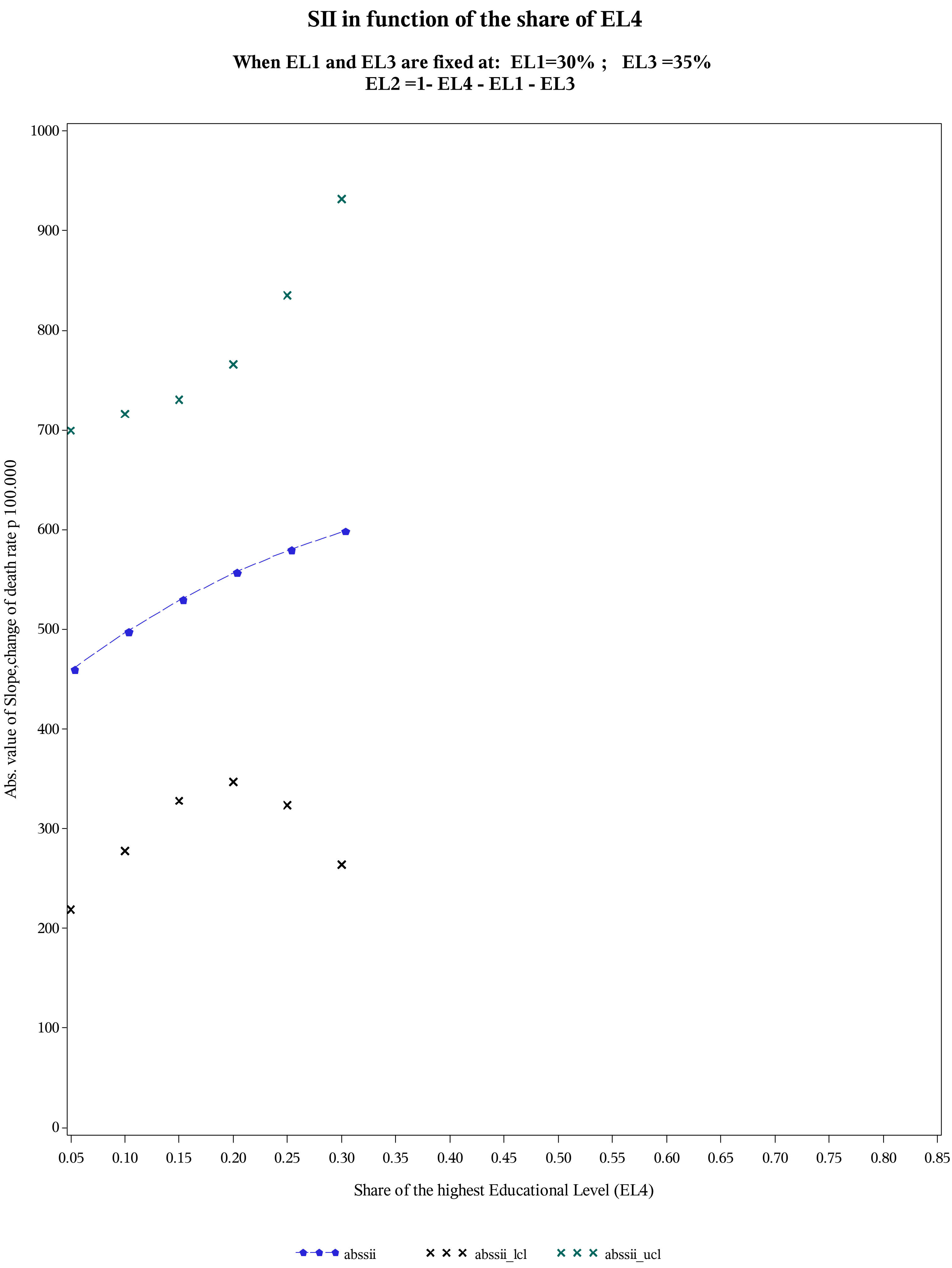

SII in function of the share of EL4
When EL1 and EL3 are fixed at: EL1=30% ; EL3 =35%
EL2 =1- EL4 - EL1 - EL3
Abs. value of Slope,change of death rate p 100.000
0
100
200
300
400
500
600
700
800
900
1000
0.05
0.10
0.15
0.20
0.25
0.30
0.35
0.40
0.45
0.50
0.55
0.60
0.65
0.70
0.75
0.80
0.85
Share of the highest Educational Level (EL4)
abssii
abssii_lcl
abssii_ucl

SII in function of the share of EL4

When EL1 and EL3 are fixed at: EL1=30% ; EL3 =40%
EL2 =1- EL4 - EL1 - EL3

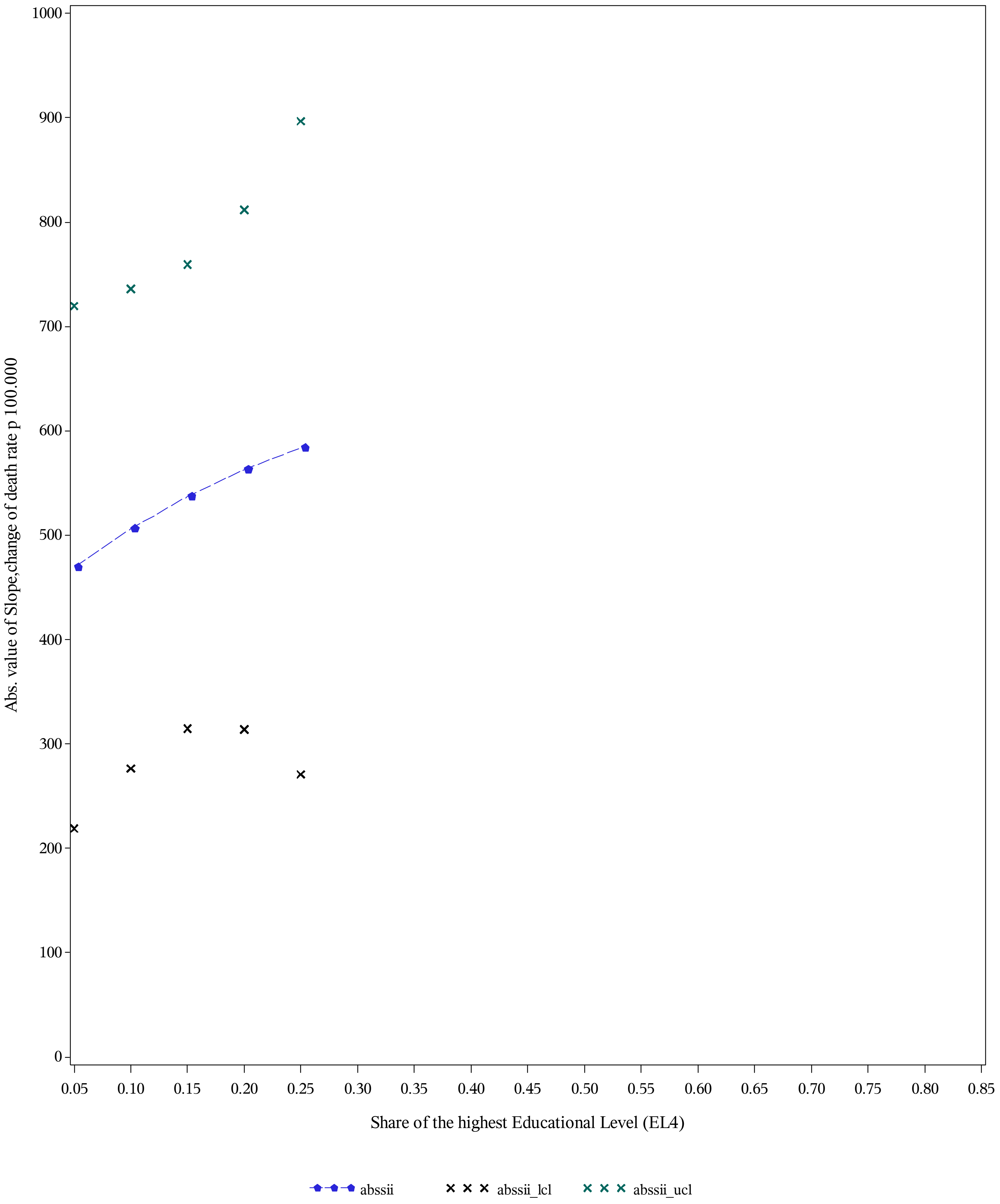

SII in function of the share of EL4

When EL1 and EL3 are fixed at: EL1=30% ; EL3 =45%
EL2 =1- EL4 - EL1 - EL3

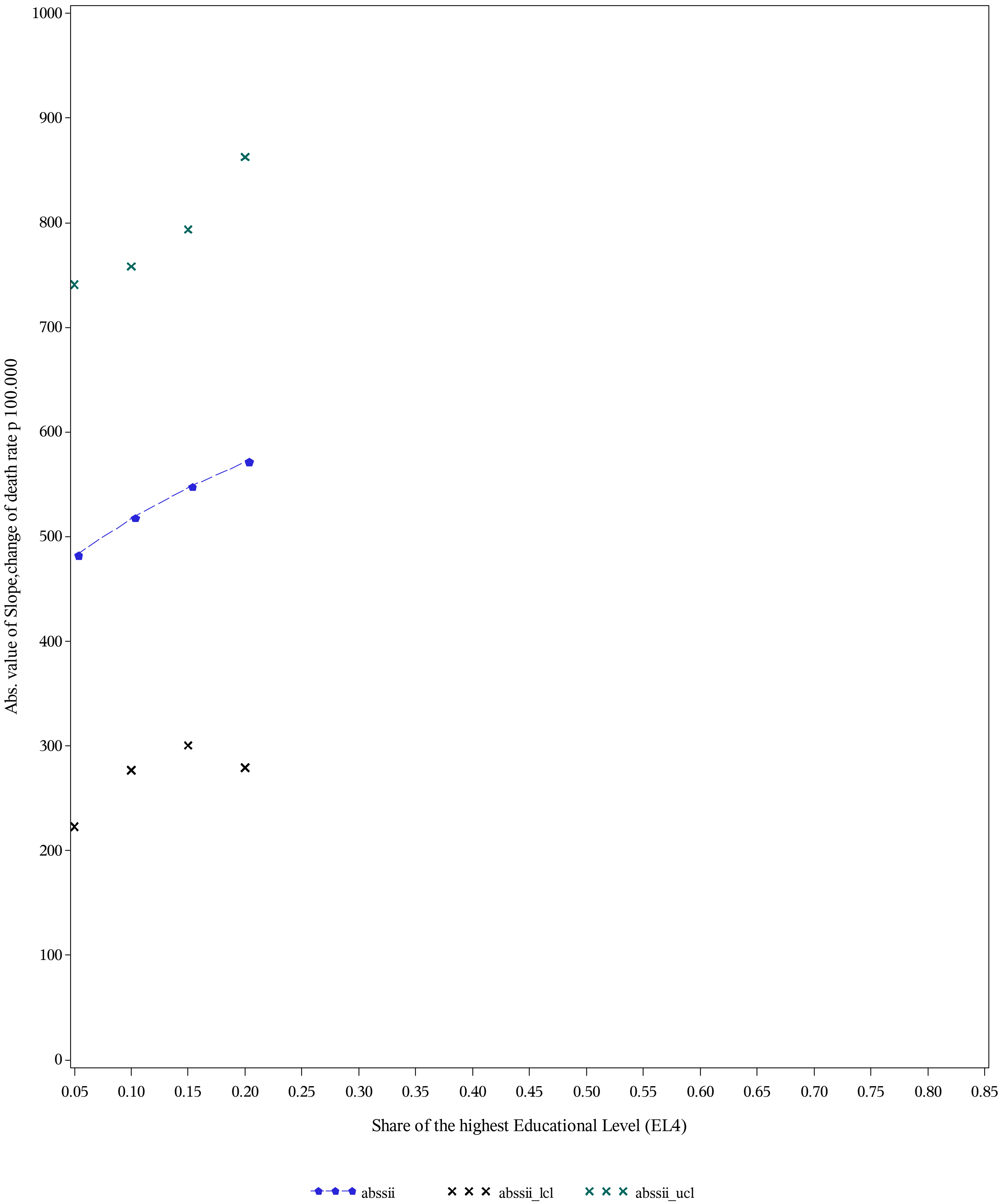

SII in function of the share of EL4

When EL1 and EL3 are fixed at: EL1=30% ; EL3 =50%
EL2 =1- EL4 - EL1 - EL3

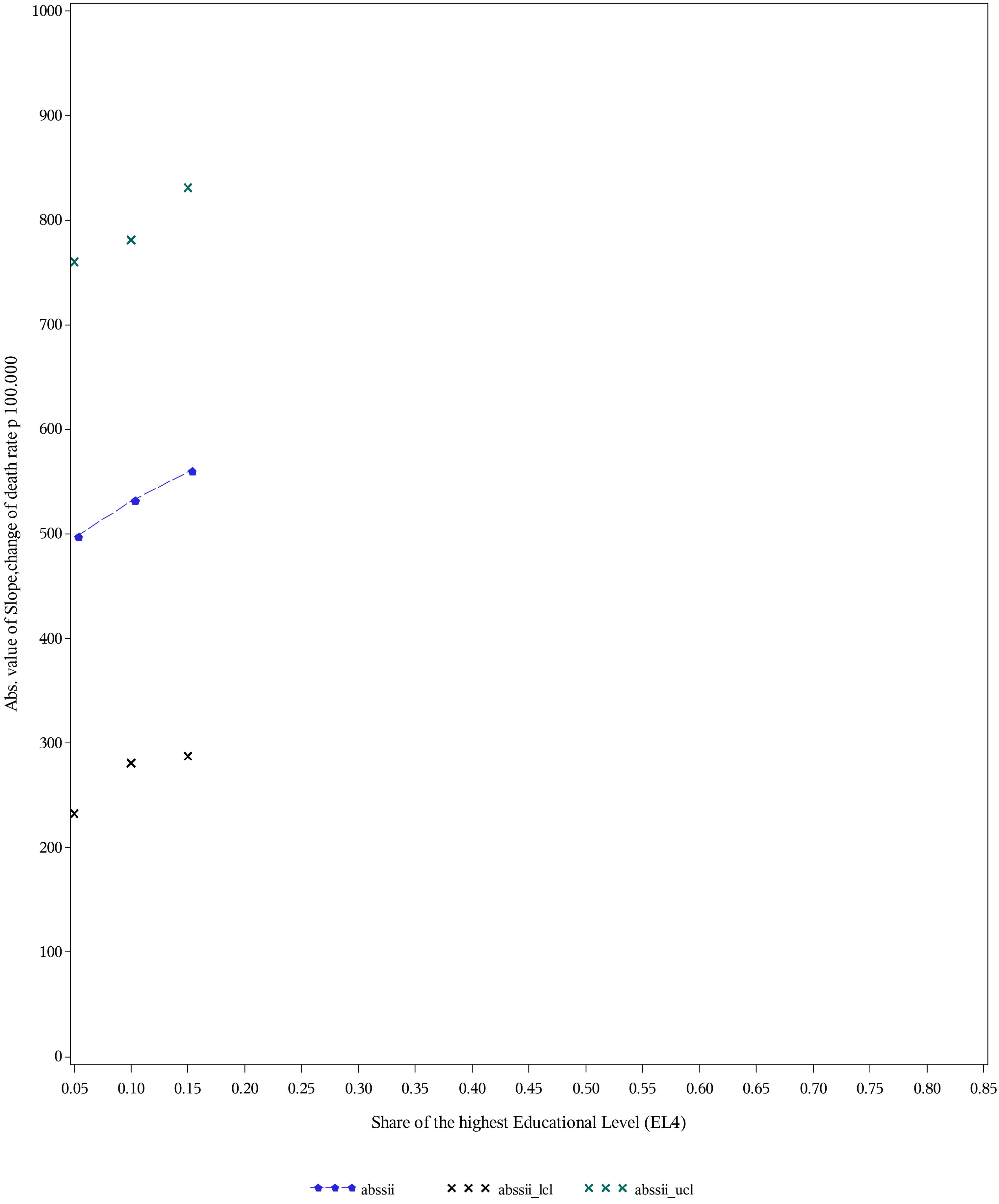

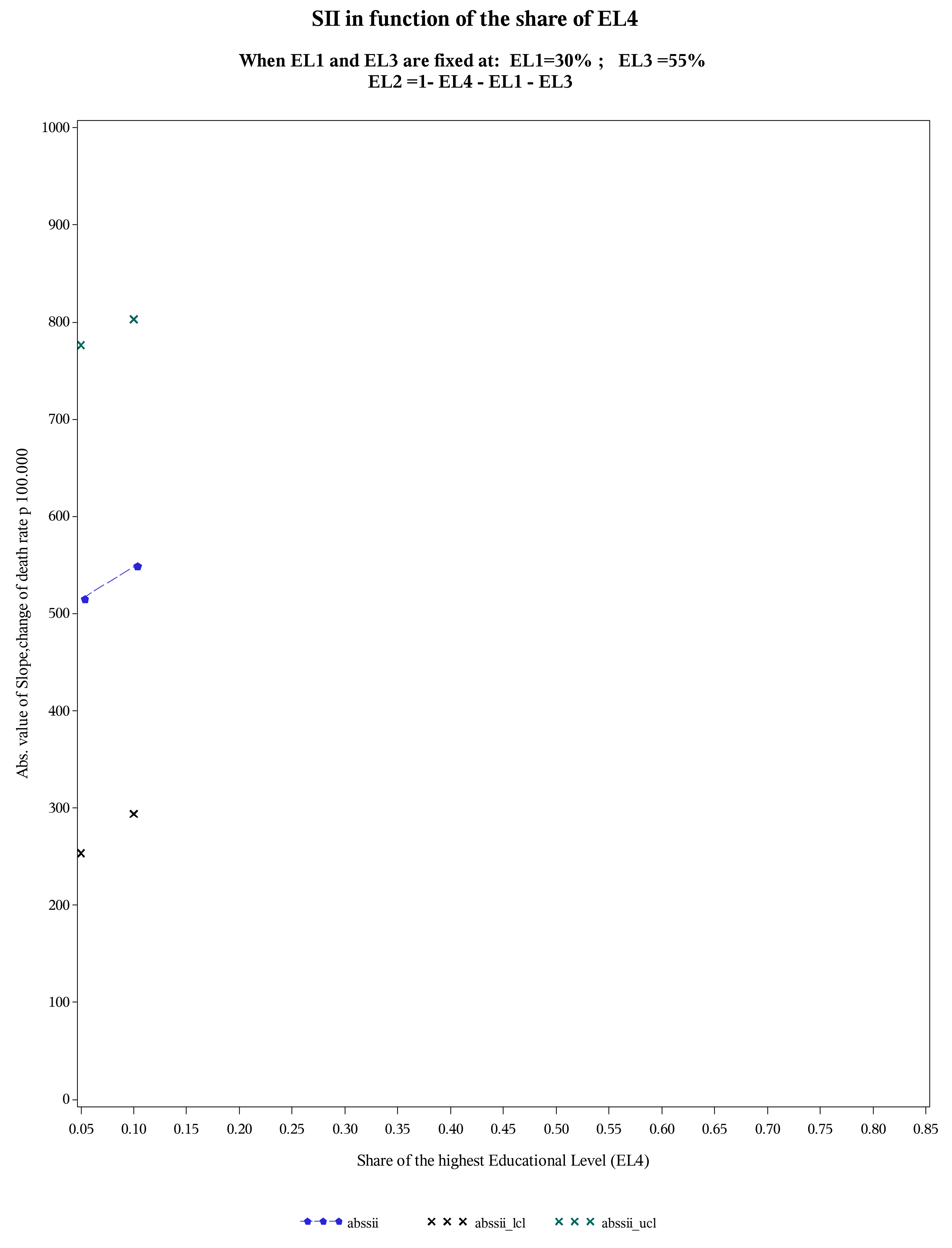
SII in function of the share of EL4
When EL1 and EL3 are fixed at: EL1=30% ; EL3 =55%
EL2 =1- EL4 - EL1 - EL3
Abs. value of Slope,change of death rate p 100.000
0
100
200
300
400
500
600
700
800
900
1000
0.05
0.10
0.15
0.20
0.25
0.30
0.35
0.40
0.45
0.50
0.55
0.60
0.65
0.70
0.75
0.80
0.85
Share of the highest Educational Level (EL4)
abssii
abssii_lcl
abssii_ucl

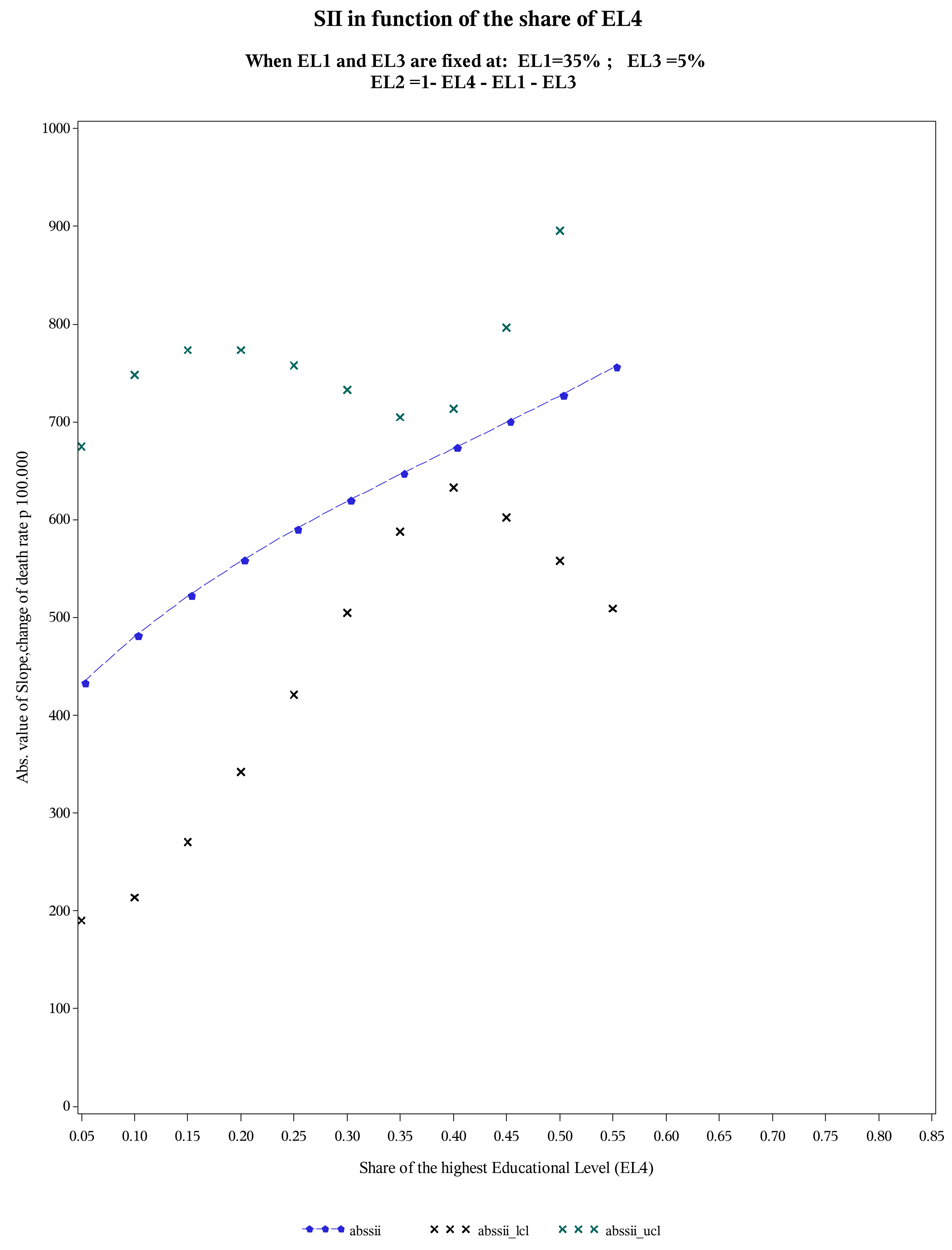
SII in function of the share of EL4
When EL1 and EL3 are fixed at: EL1=35% ; EL3 =5%
EL2 =1- EL4 - EL1 - EL3
Abs. value of Slope,change of death rate p 100.000
1000
900
800
700
600
500
400
300
200
100
0
0.05
0.10
0.15
0.20
0.25
0.30
0.35
0.40
0.45
0.50
0.55
0.60
0.65
0.70
0.75
0.80
0.85
Share of the highest Educational Level (EL4)
abssii
abssii_lcl
abssii_ucl

SII in function of the share of EL4

When EL1 and EL3 are fixed at: EL1=35% ; EL3 =10%
EL2 =1- EL4 - EL1 - EL3

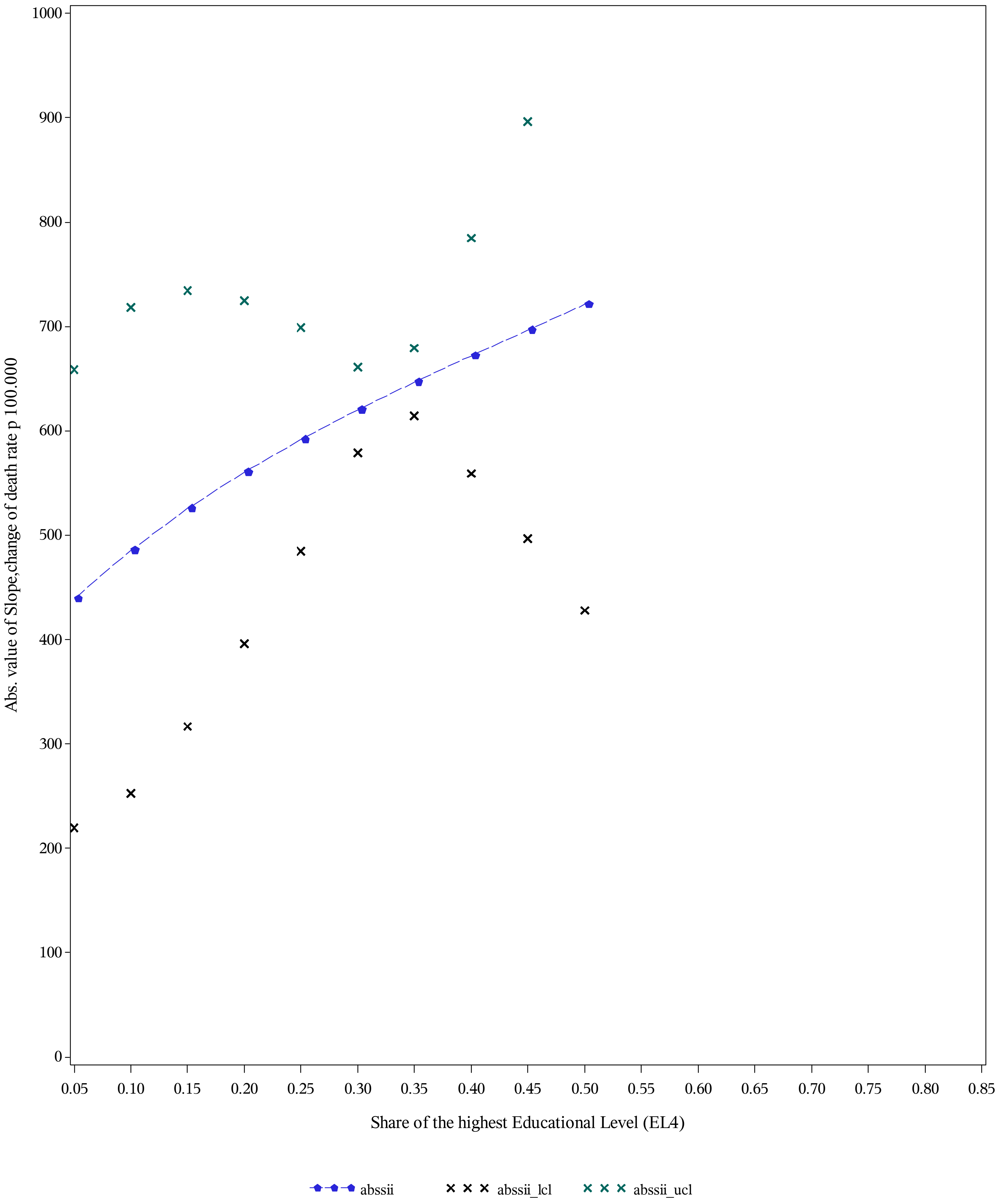

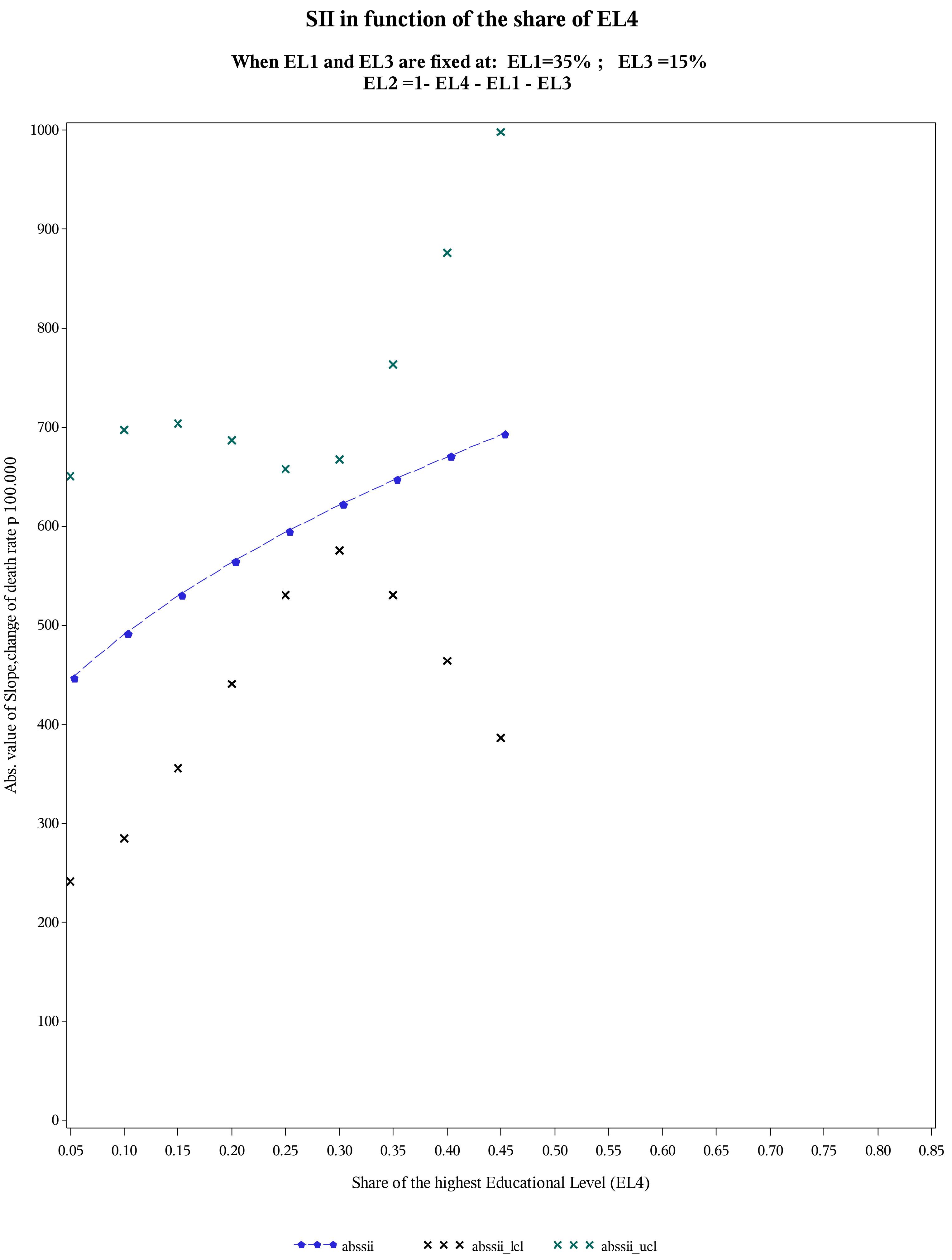

SII in function of the share of EL4
When EL1 and EL3 are fixed at: EL1=35% ; EL3 =15%
EL2 =1- EL4 - EL1 - EL3
Abs. value of Slope,change of death rate p 100.000
1000
900
800
700
600
500
400
300
200
100
0
0.05
0.10
0.15
0.20
0.25
0.30
0.35
0.40
0.45
0.50
0.55
0.60
0.65
0.70
0.75
0.80
0.85
Share of the highest Educational Level (EL4)
abssii
abssii_lcl
abssii_ucl

SII in function of the share of EL4

When EL1 and EL3 are fixed at: EL1=35% ; EL3 =20%
EL2 =1- EL4 - EL1 - EL3

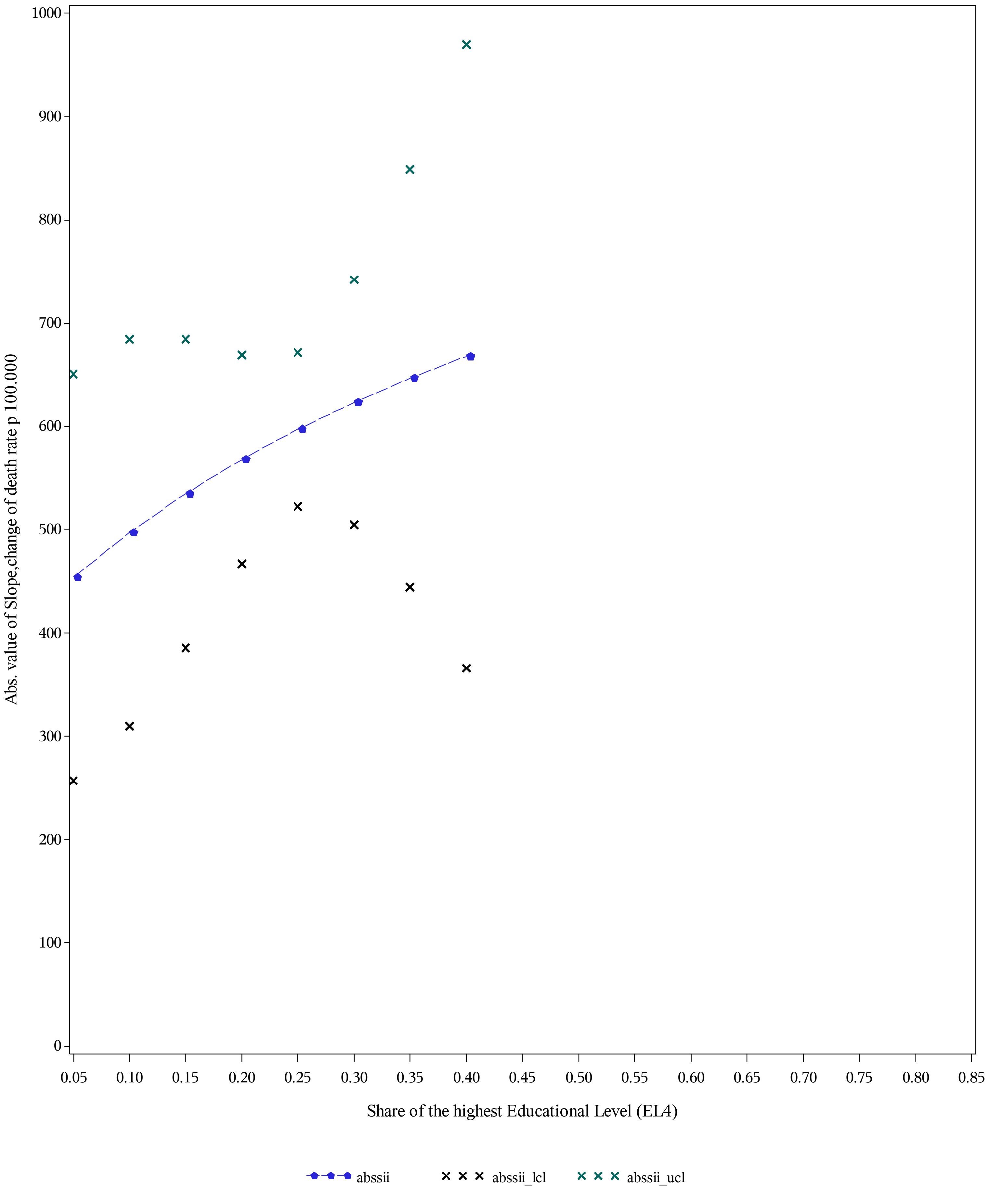

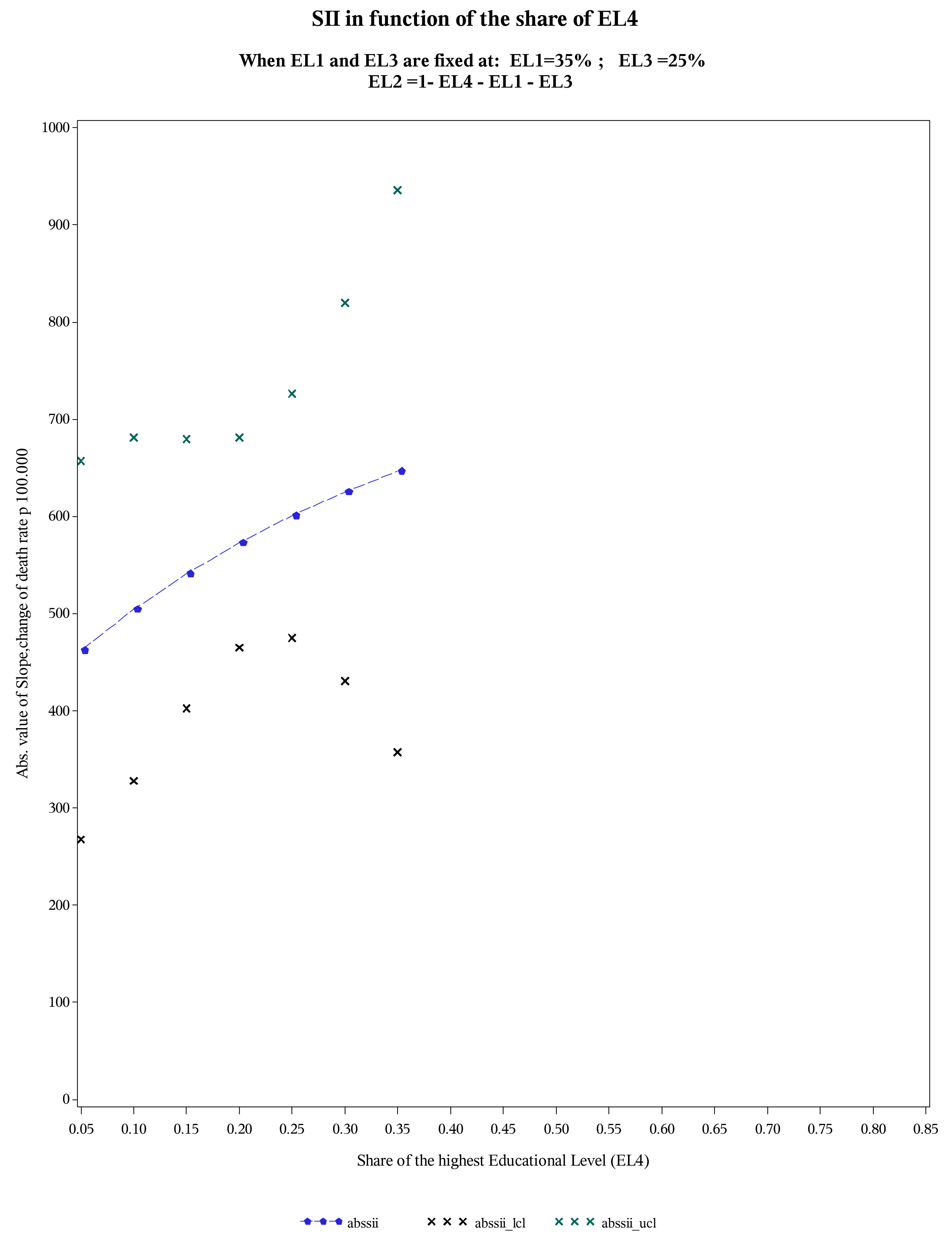
SII in function of the share of EL4
When EL1 and EL3 are fixed at: EL1=35% ; EL3 =25%
EL2 =1- EL4 - EL1 - EL3
Abs. value of Slope,change of death rate p 100.000
0
100
200
300
400
500
600
700
800
900
1000
0.05
0.10
0.15
0.20
0.25
0.30
0.35
0.40
0.45
0.50
0.55
0.60
0.65
0.70
0.75
0.80
0.85
Share of the highest Educational Level (EL4)
abssii
abssii_lcl
abssii_ucl

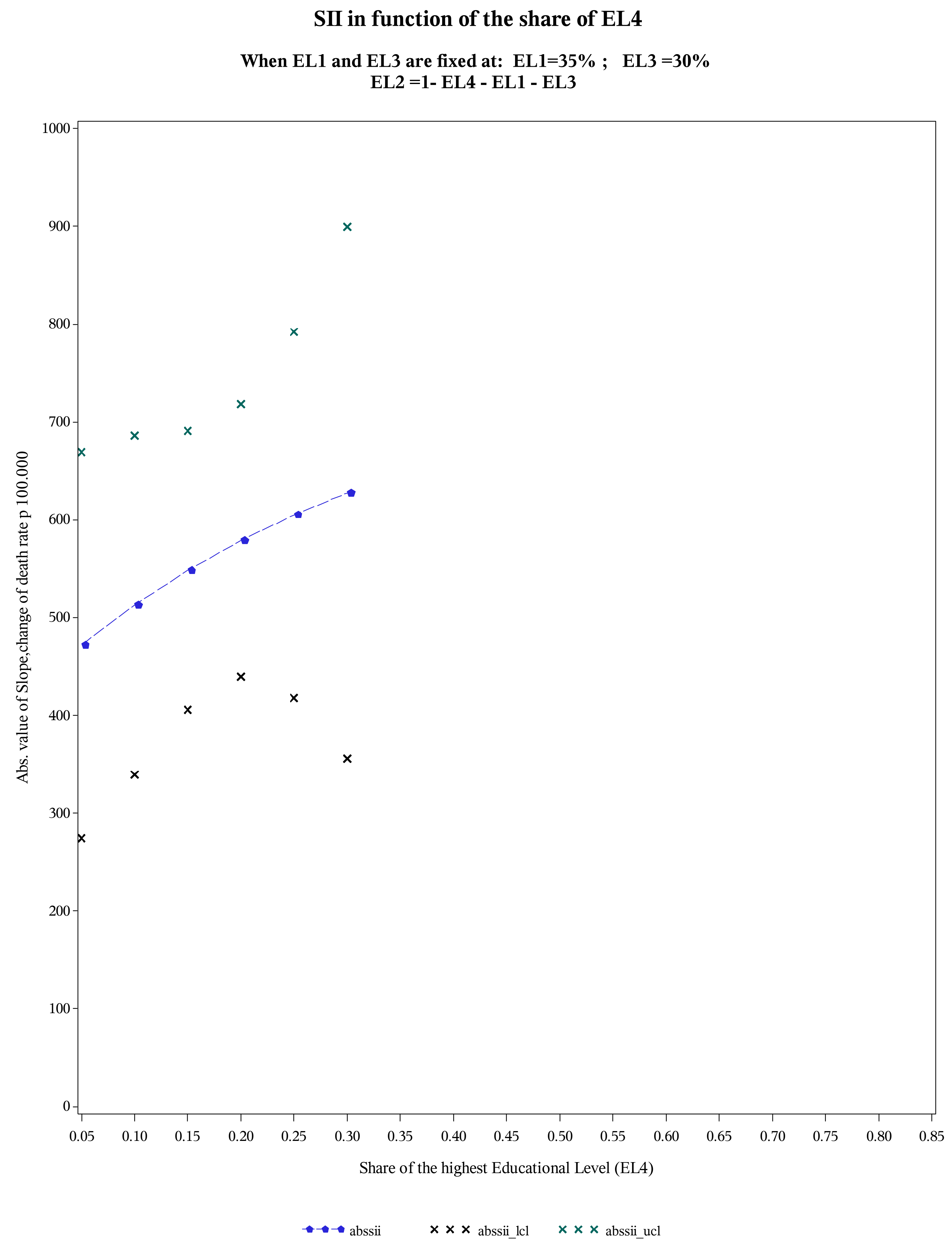
SII in function of the share of EL4
When EL1 and EL3 are fixed at: EL1=35% ; EL3 =30%
EL2 =1- EL4 - EL1 - EL3
Abs. value of Slope,change of death rate p 100.000
0
100
200
300
400
500
600
700
800
900
1000
0.05
0.10
0.15
0.20
0.25
0.30
0.35
0.40
0.45
0.50
0.55
0.60
0.65
0.70
0.75
0.80
0.85
Share of the highest Educational Level (EL4)
abssii
abssii_lcl
abssii_ucl

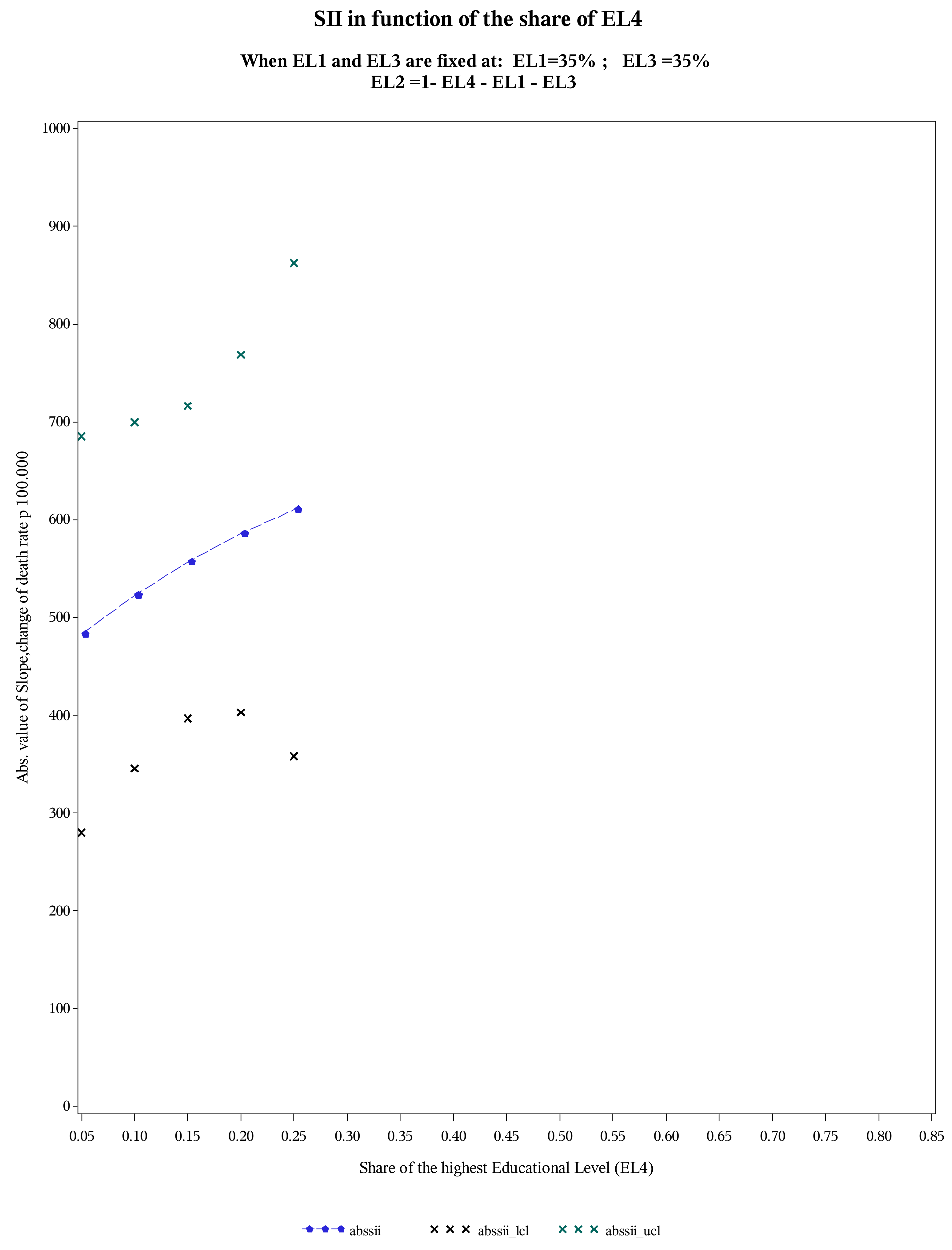
SII in function of the share of EL4
When EL1 and EL3 are fixed at: EL1=35% ; EL3 =35%
EL2 =1- EL4 - EL1 - EL3
Abs. value of Slope,change of death rate p 100.000
Share of the highest Educational Level (EL4)
0
100
200
300
400
500
600
700
800
900
1000
0.05
0.10
0.15
0.20
0.25
0.30
0.35
0.40
0.45
0.50
0.55
0.60
0.65
0.70
0.75
0.80
0.85
abssii
abssii_lcl
abssii_ucl

SII in function of the share of EL4

When EL1 and EL3 are fixed at: EL1=35% ; EL3 =40%
EL2 =1- EL4 - EL1 - EL3

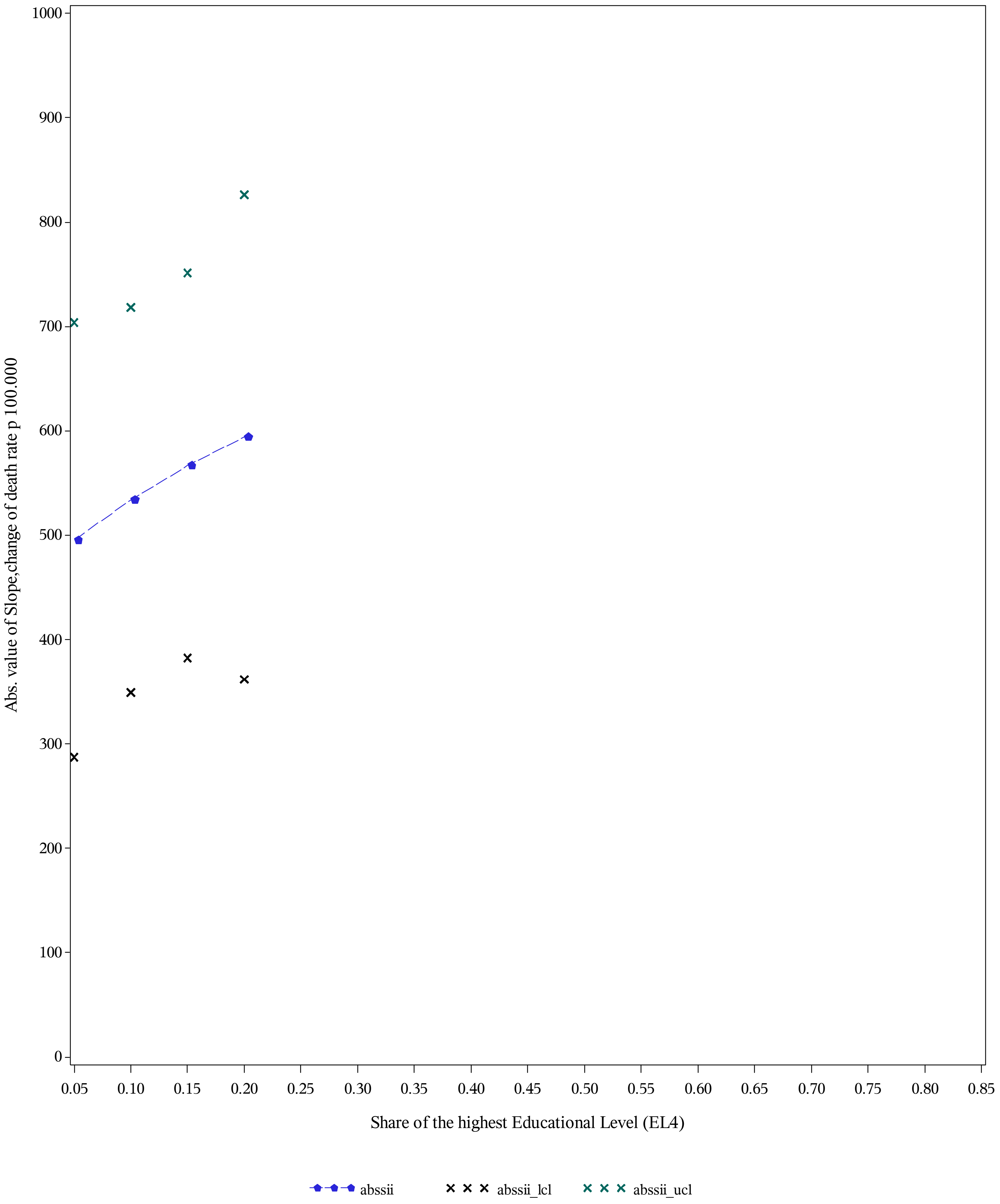

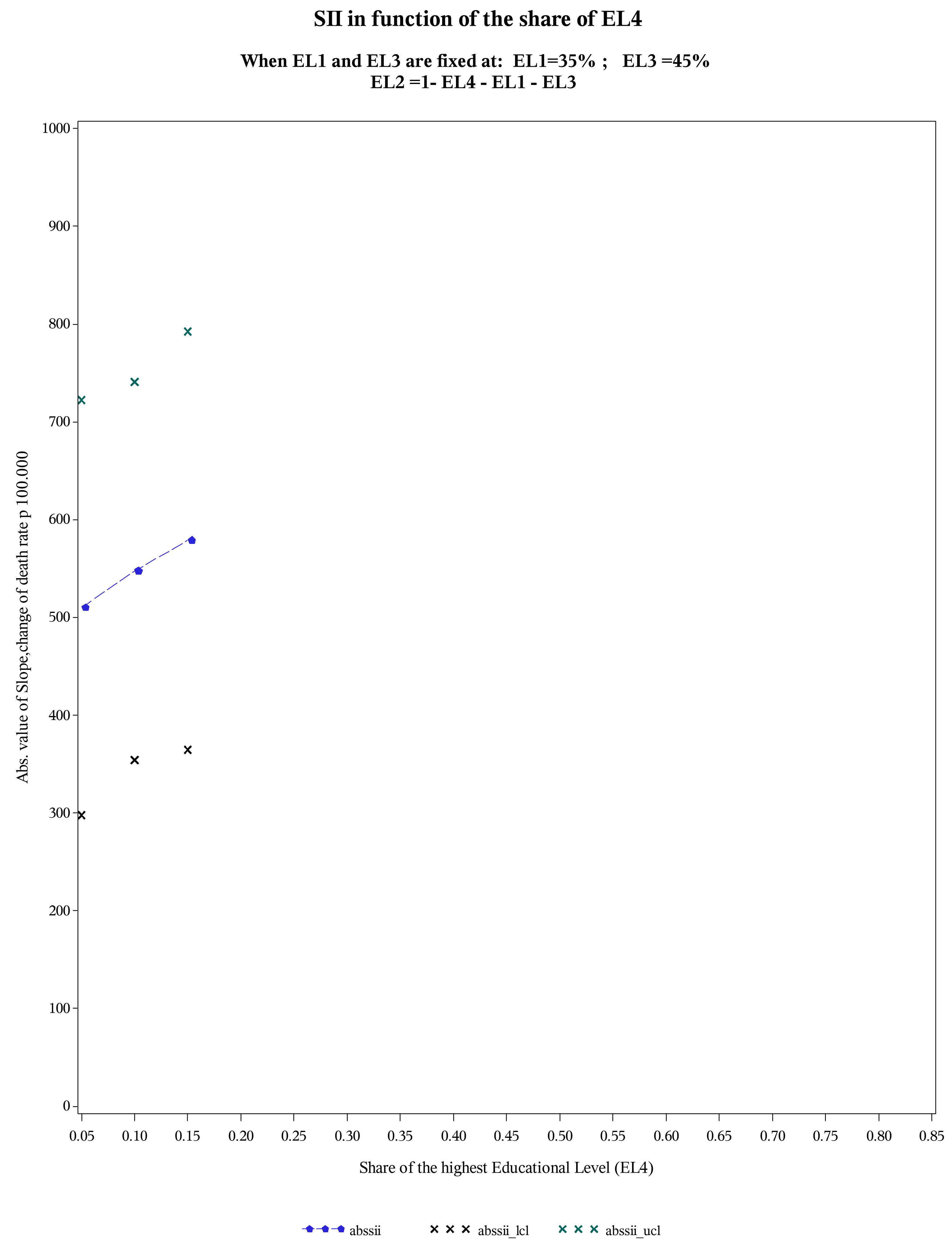
SII in function of the share of EL4
When EL1 and EL3 are fixed at: EL1=35% ; EL3 =45%
EL2 =1- EL4 - EL1 - EL3
Abs. value of Slope,change of death rate p 100.000
0
100
200
300
400
500
600
700
800
900
1000
0.05
0.10
0.15
0.20
0.25
0.30
0.35
0.40
0.45
0.50
0.55
0.60
0.65
0.70
0.75
0.80
0.85
Share of the highest Educational Level (EL4)
abssii
abssii_lcl
abssii_ucl

SII in function of the share of EL4

When EL1 and EL3 are fixed at: EL1=35% ; EL3 =50%
EL2 =1- EL4 - EL1 - EL3

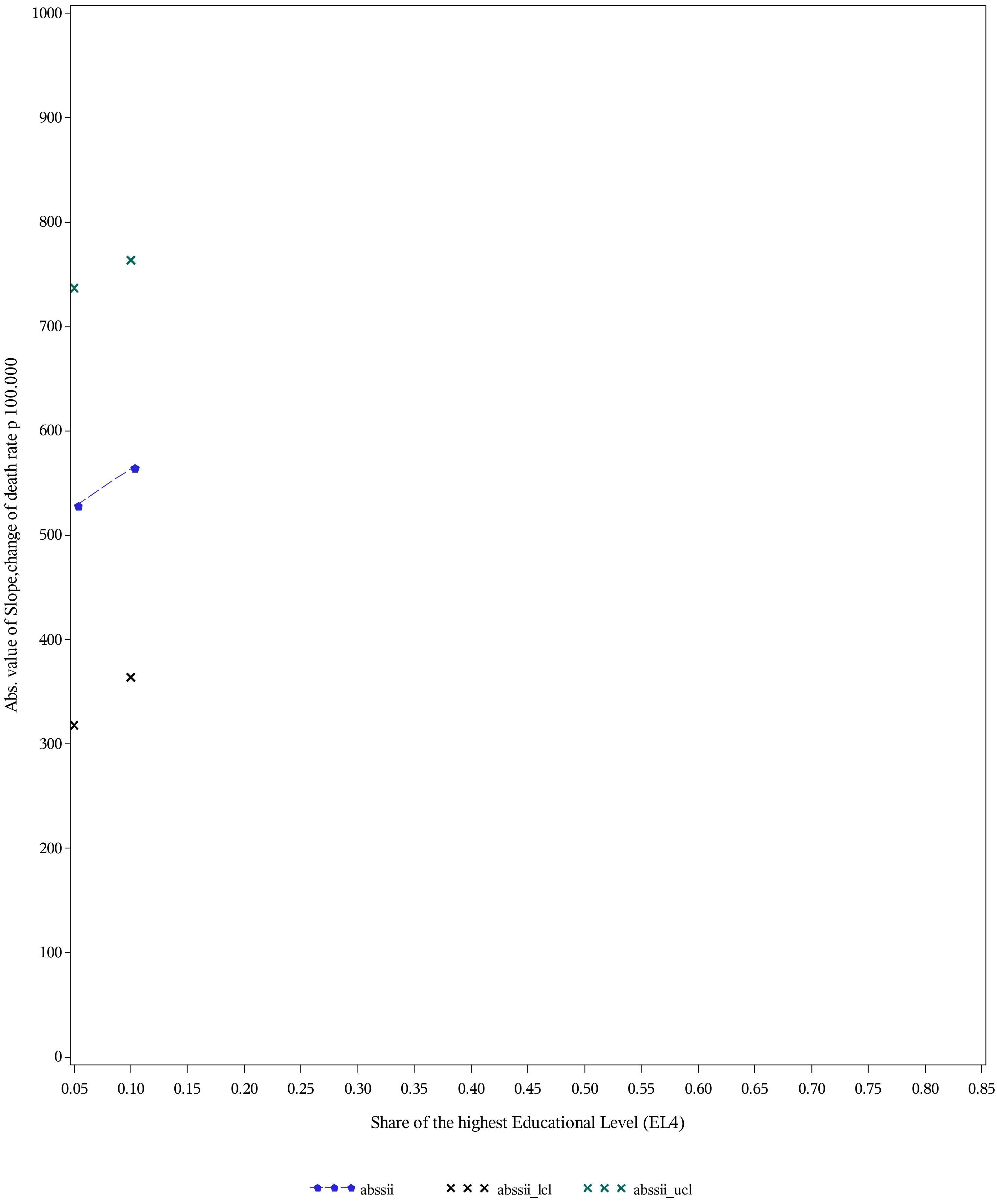

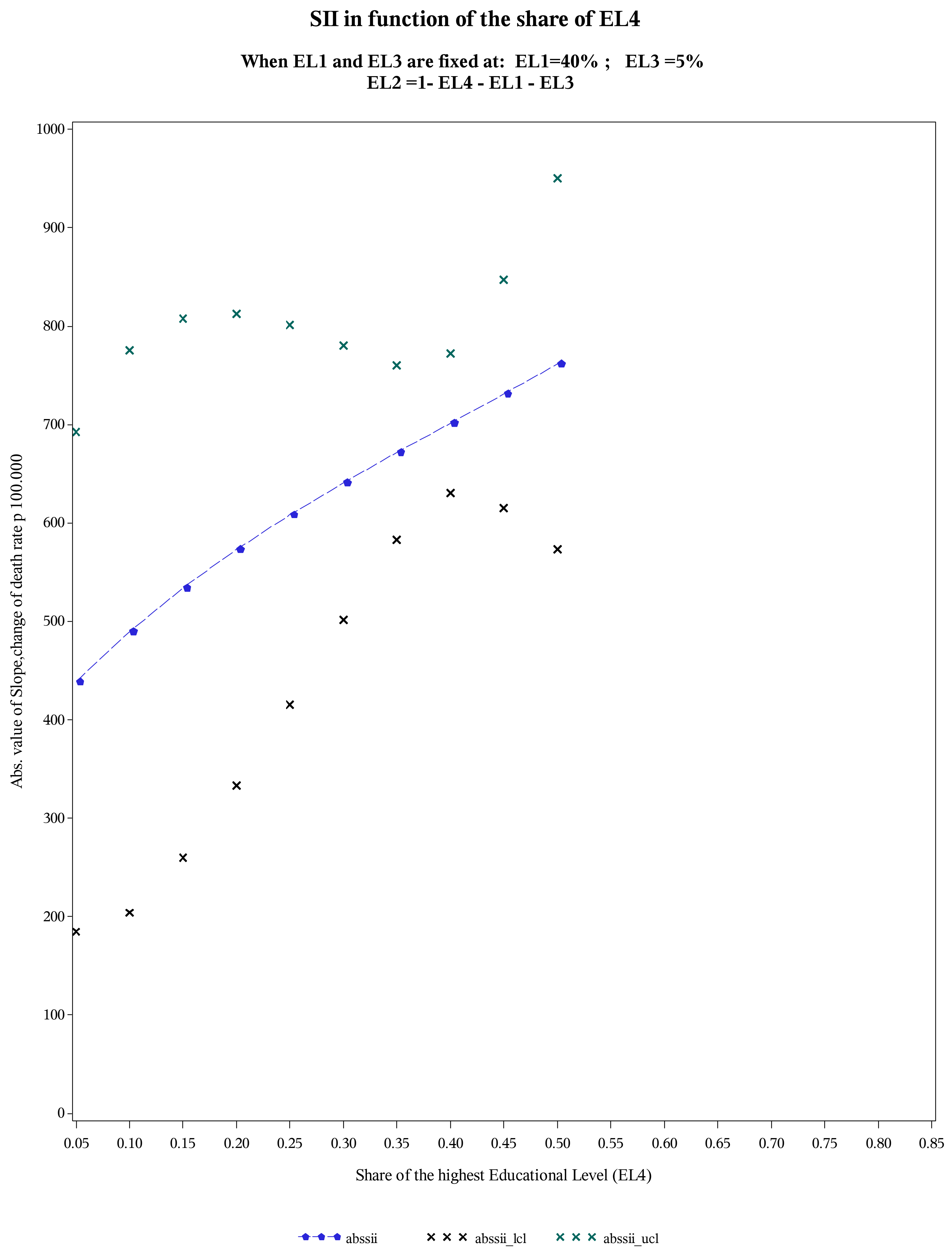

SII in function of the share of EL4
When EL1 and EL3 are fixed at: EL1=40% ; EL3 =5%
EL2 =1- EL4 - EL1 - EL3
Abs. value of Slope,change of death rate p 100.000
0
100
200
300
400
500
600
700
800
900
1000
0.05
0.10
0.15
0.20
0.25
0.30
0.35
0.40
0.45
0.50
0.55
0.60
0.65
0.70
0.75
0.80
0.85
Share of the highest Educational Level (EL4)
abssii
abssii_lcl
abssii_ucl

SII in function of the share of EL4

When EL1 and EL3 are fixed at: EL1=40% ; EL3 =10%
EL2 =1- EL4 - EL1 - EL3

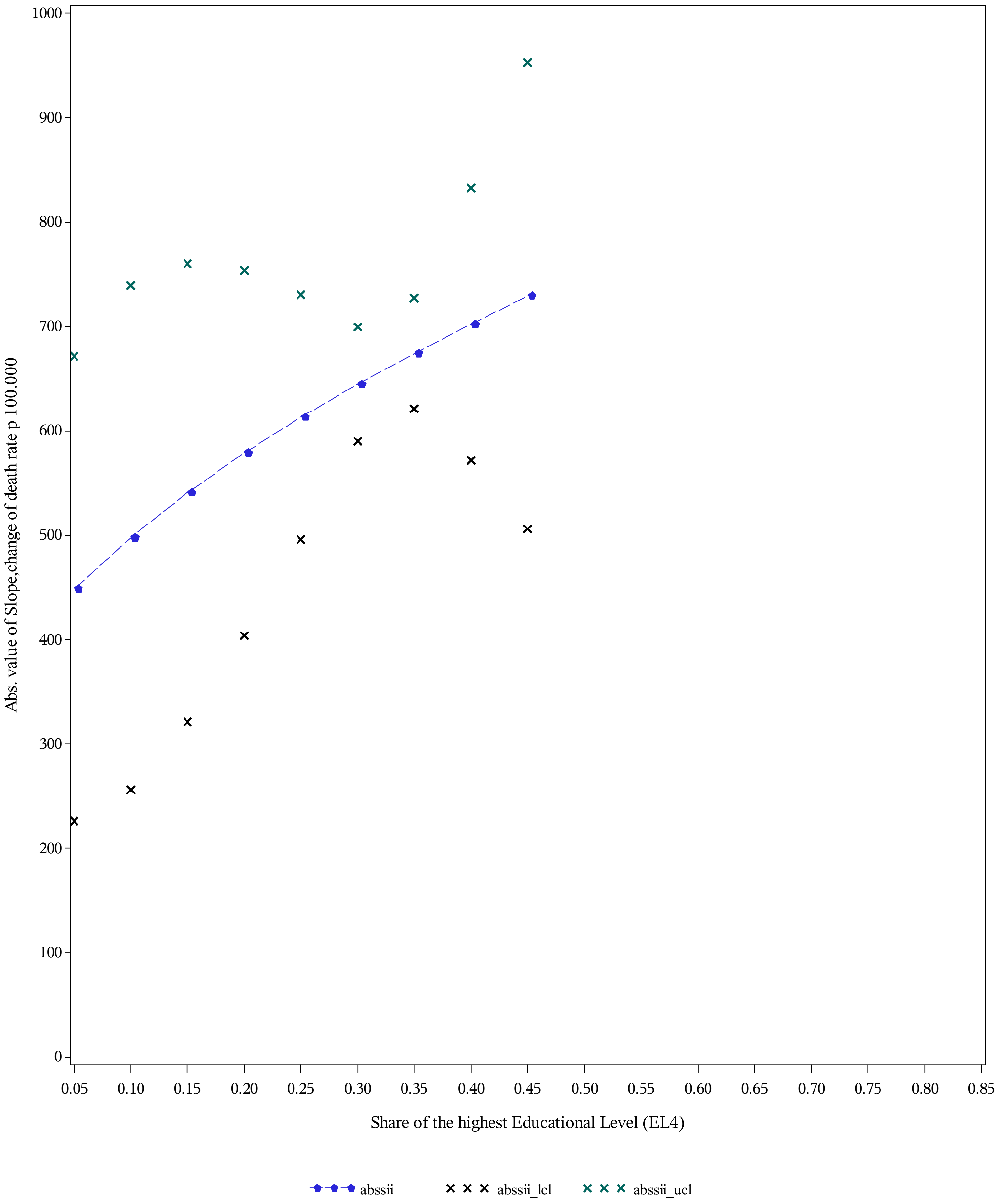

SII in function of the share of EL4

When EL1 and EL3 are fixed at: EL1=40% ; EL3 =15%
EL2 =1- EL4 - EL1 - EL3

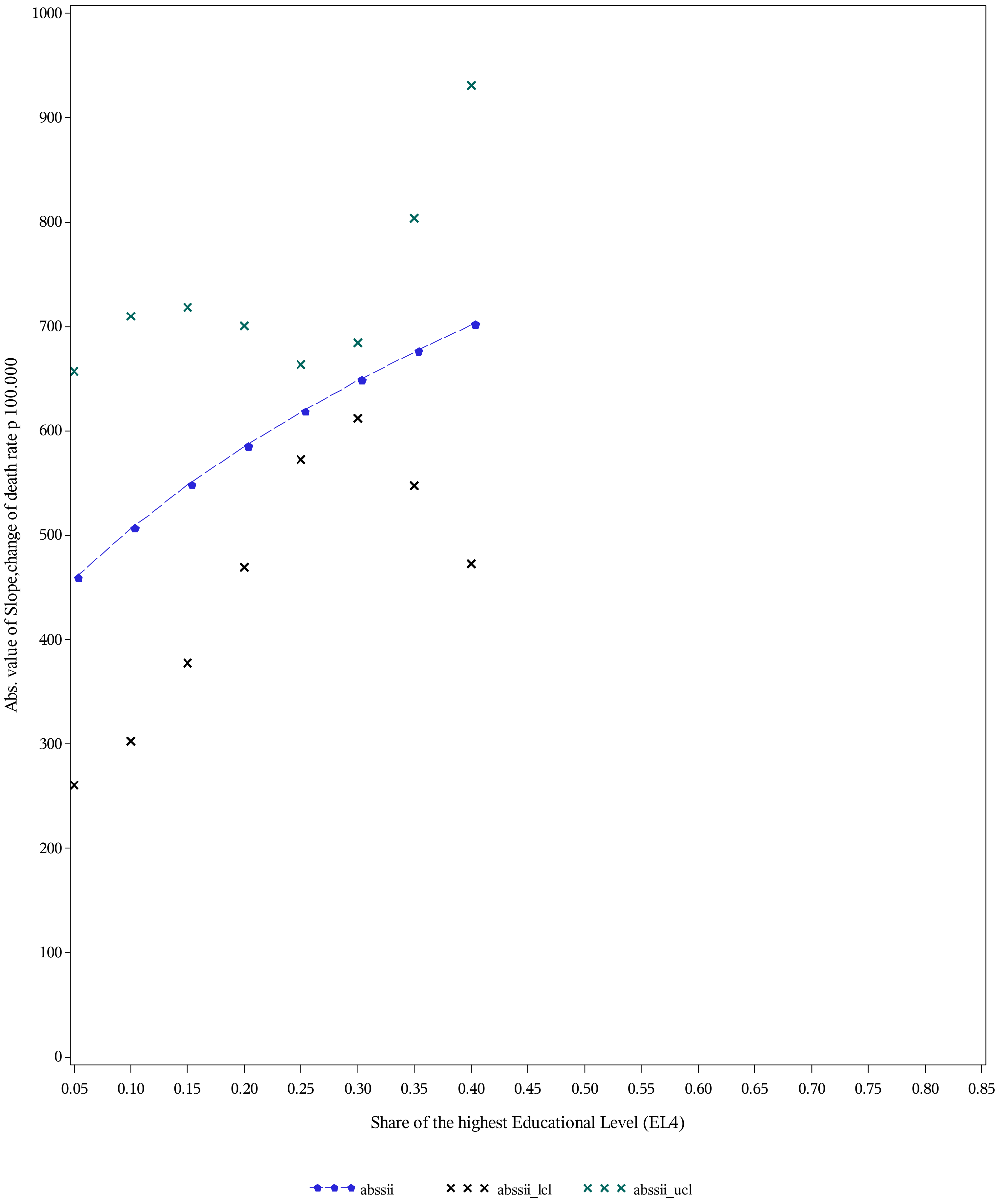

SII in function of the share of EL4

When EL1 and EL3 are fixed at: EL1=40% ; EL3 =20%
EL2 =1- EL4 - EL1 - EL3

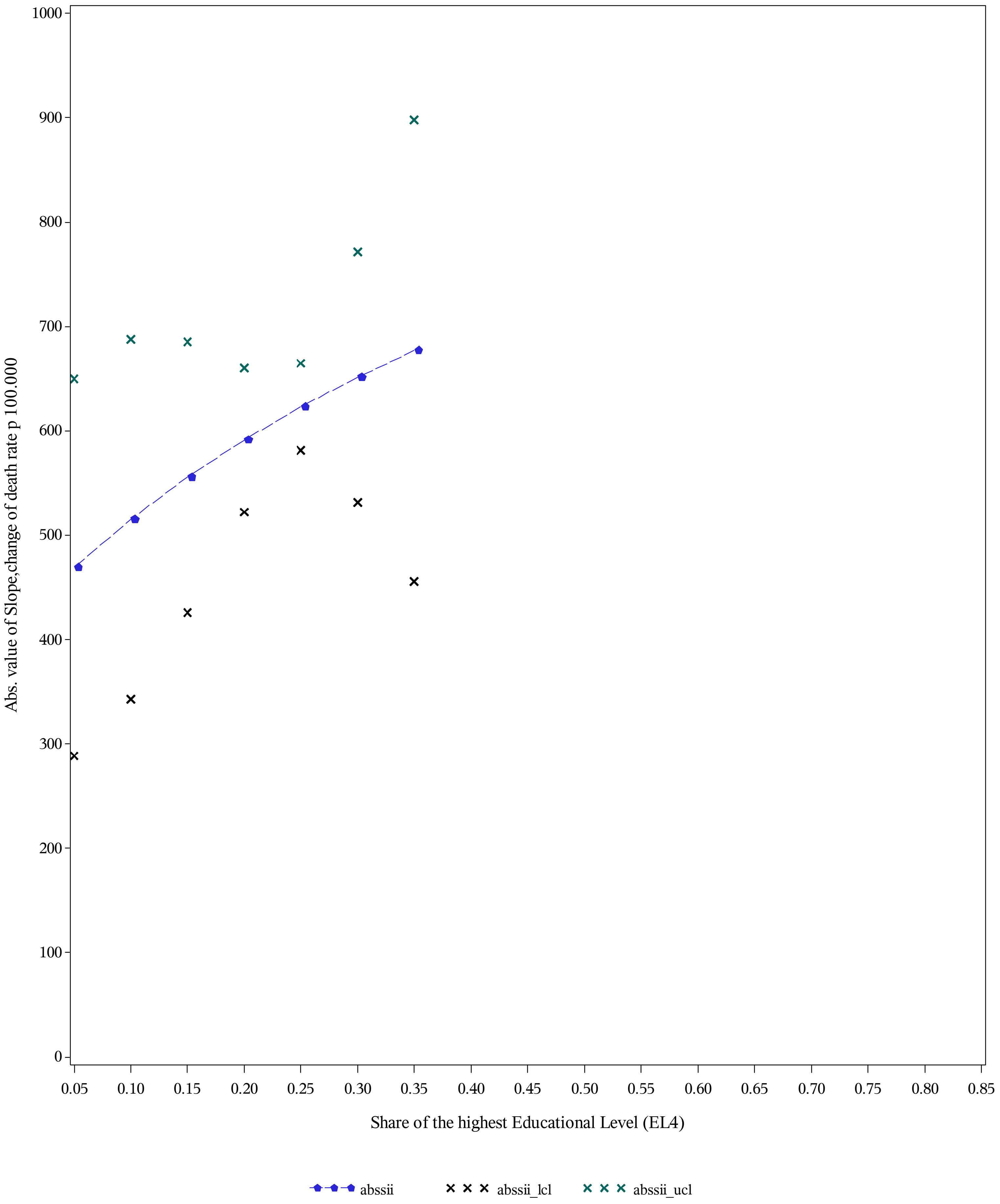

SII in function of the share of EL4

When EL1 and EL3 are fixed at: EL1=40% ; EL3 =25%
EL2 =1- EL4 - EL1 - EL3

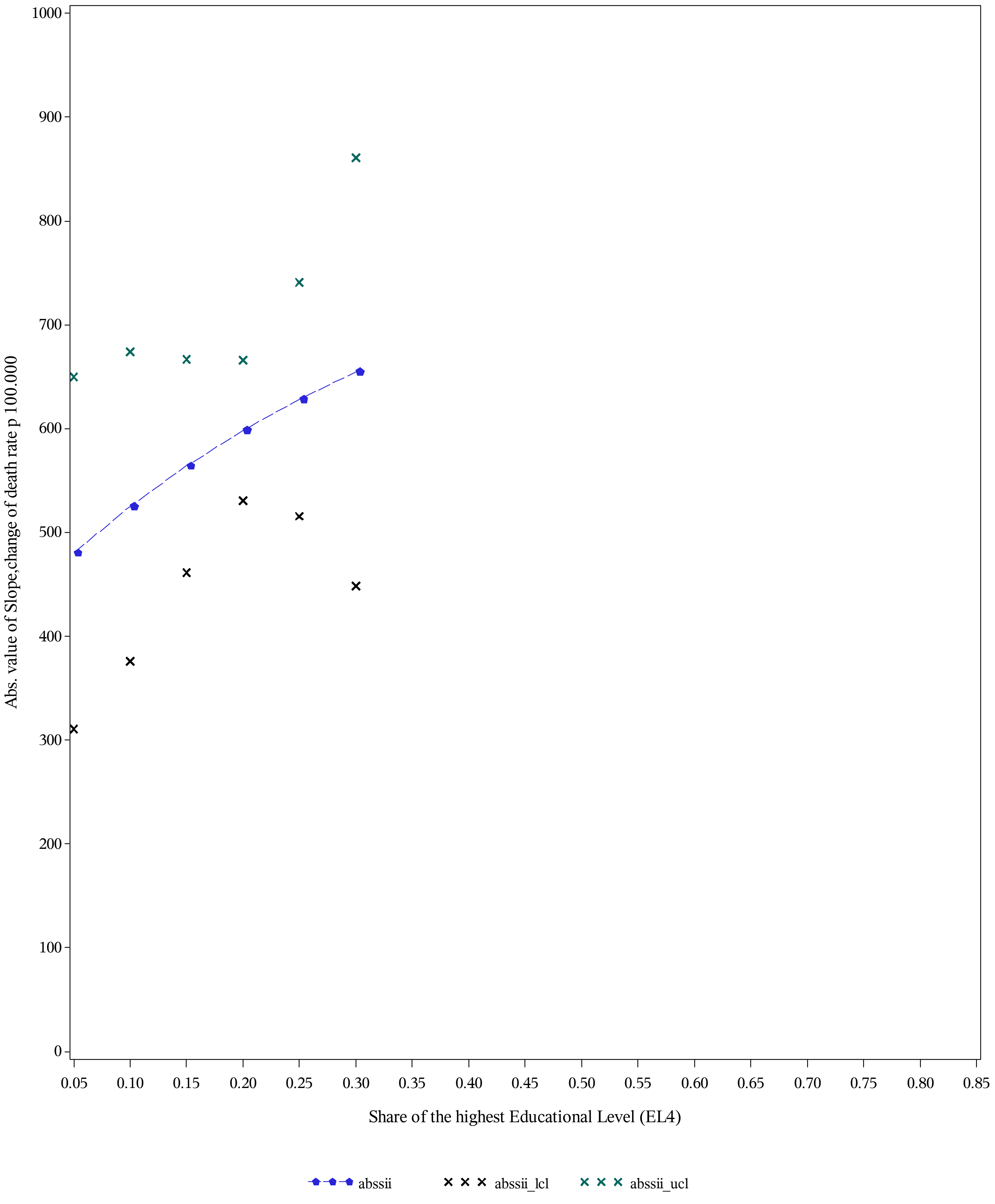

SII in function of the share of EL4

When EL1 and EL3 are fixed at: EL1=40% ; EL3 =30%
EL2 =1- EL4 - EL1 - EL3

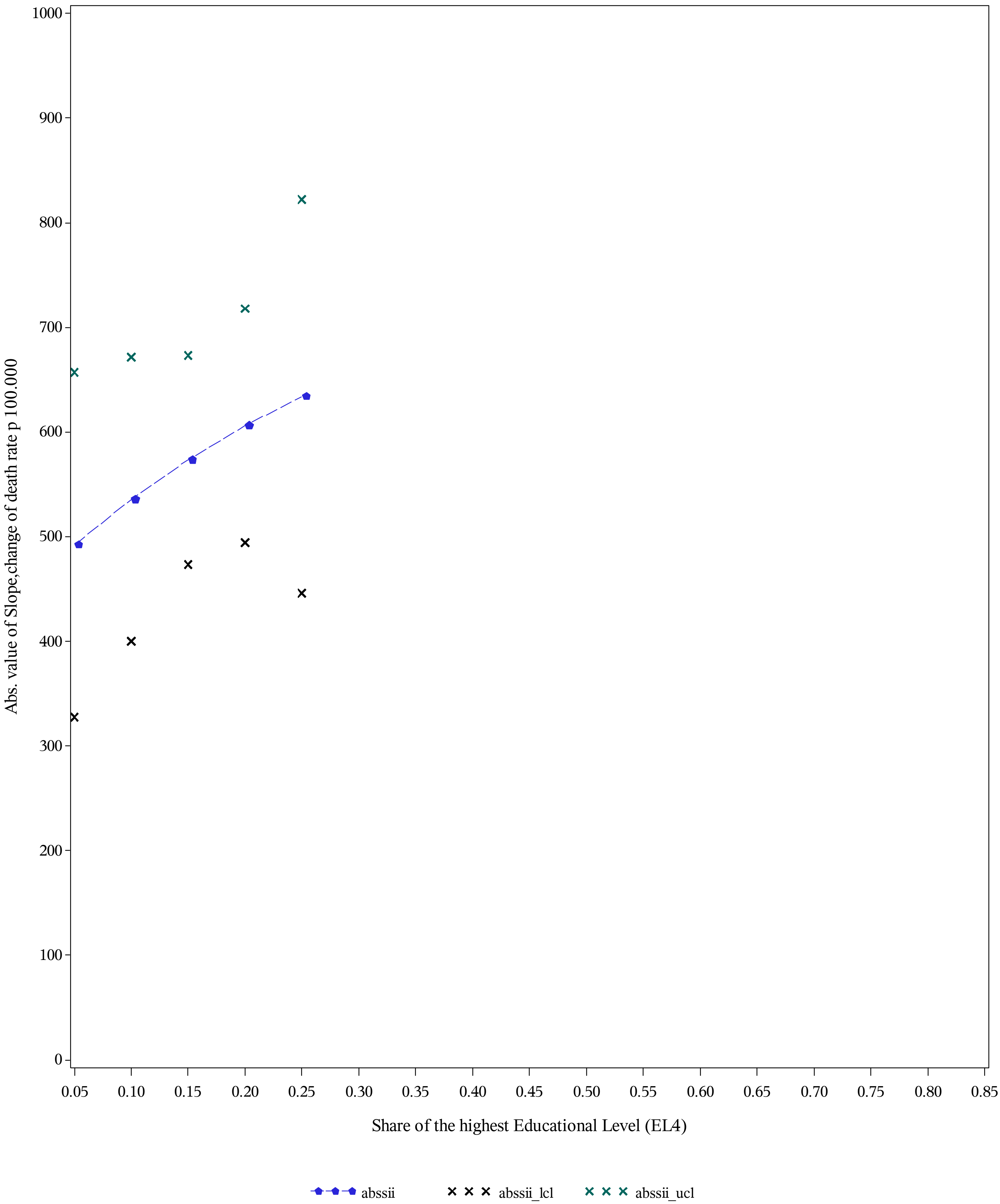

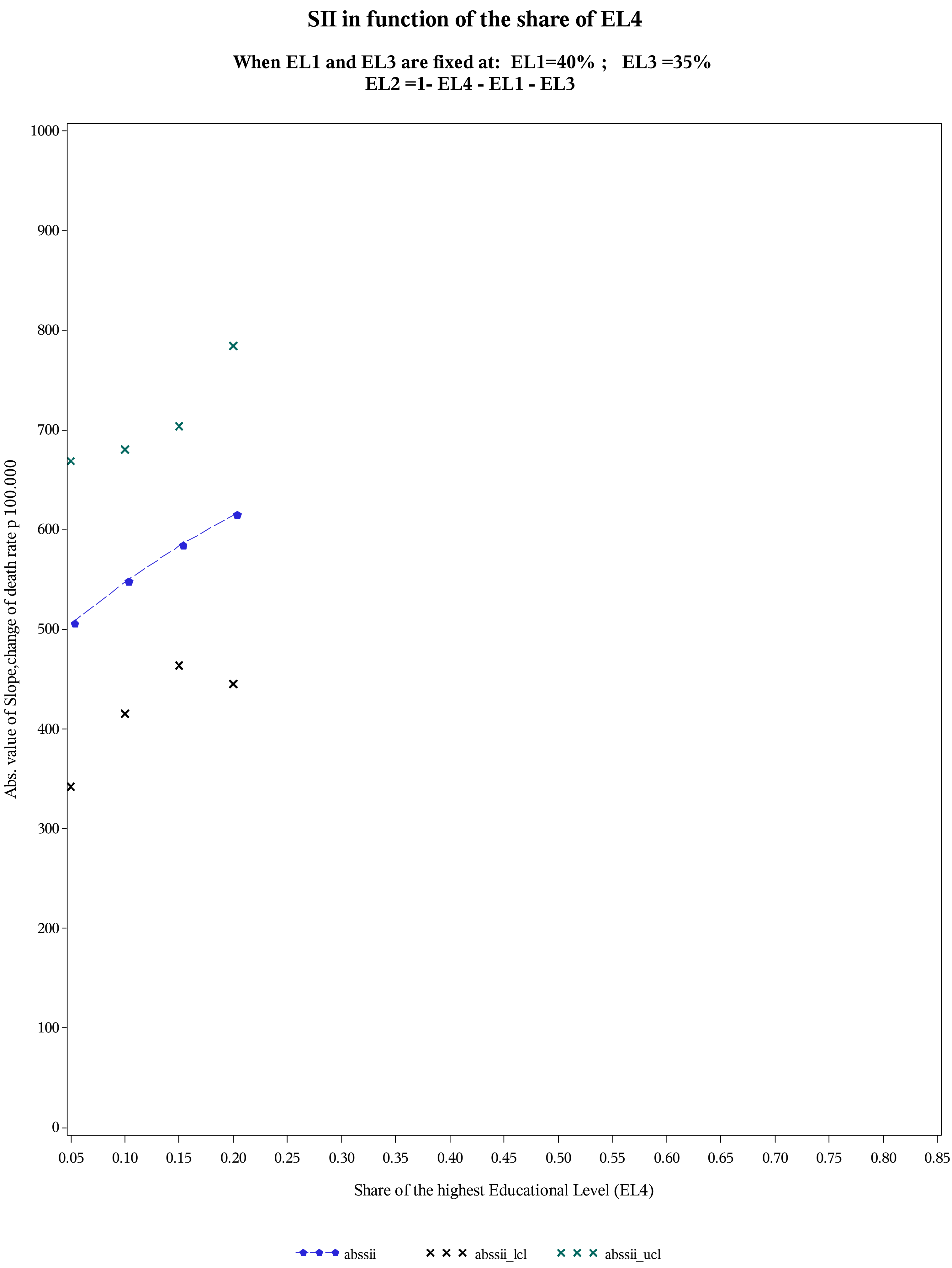

SII in function of the share of EL4
When EL1 and EL3 are fixed at: EL1=40% ; EL3 =35%
EL2 =1- EL4 - EL1 - EL3
Abs. value of Slope,change of death rate p 100.000
1000
900
800
700
600
500
400
300
200
100
0
0.05
0.10
0.15
0.20
0.25
0.30
0.35
0.40
0.45
0.50
0.55
0.60
0.65
0.70
0.75
0.80
0.85
Share of the highest Educational Level (EL4)
abssii
abssii_lcl
abssii_ucl

SII in function of the share of EL4

When EL1 and EL3 are fixed at: EL1=40% ; EL3 =40%
EL2 =1- EL4 - EL1 - EL3

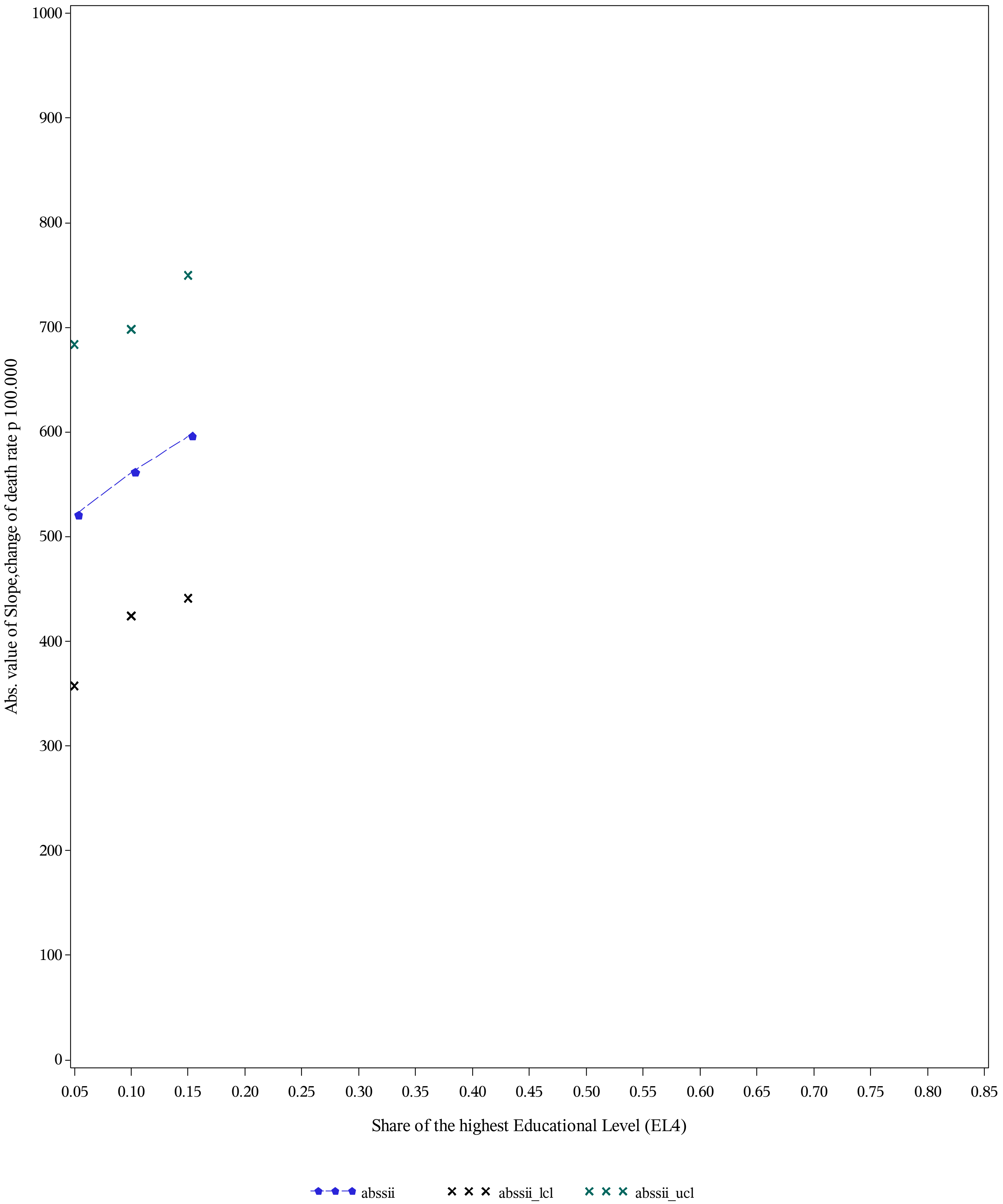

SII in function of the share of EL4

When EL1 and EL3 are fixed at: EL1=40% ; EL3 =45%
EL2 =1- EL4 - EL1 - EL3

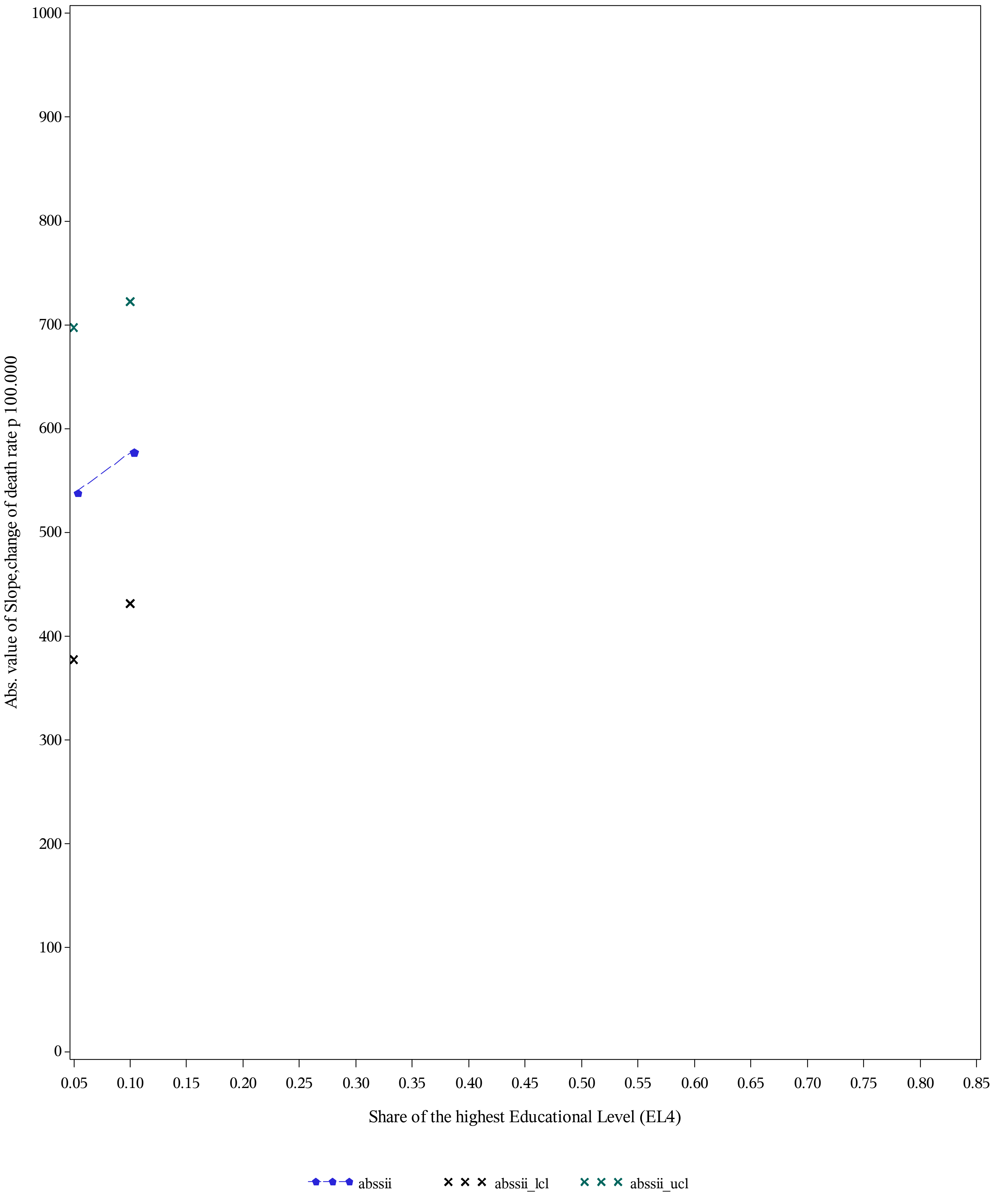

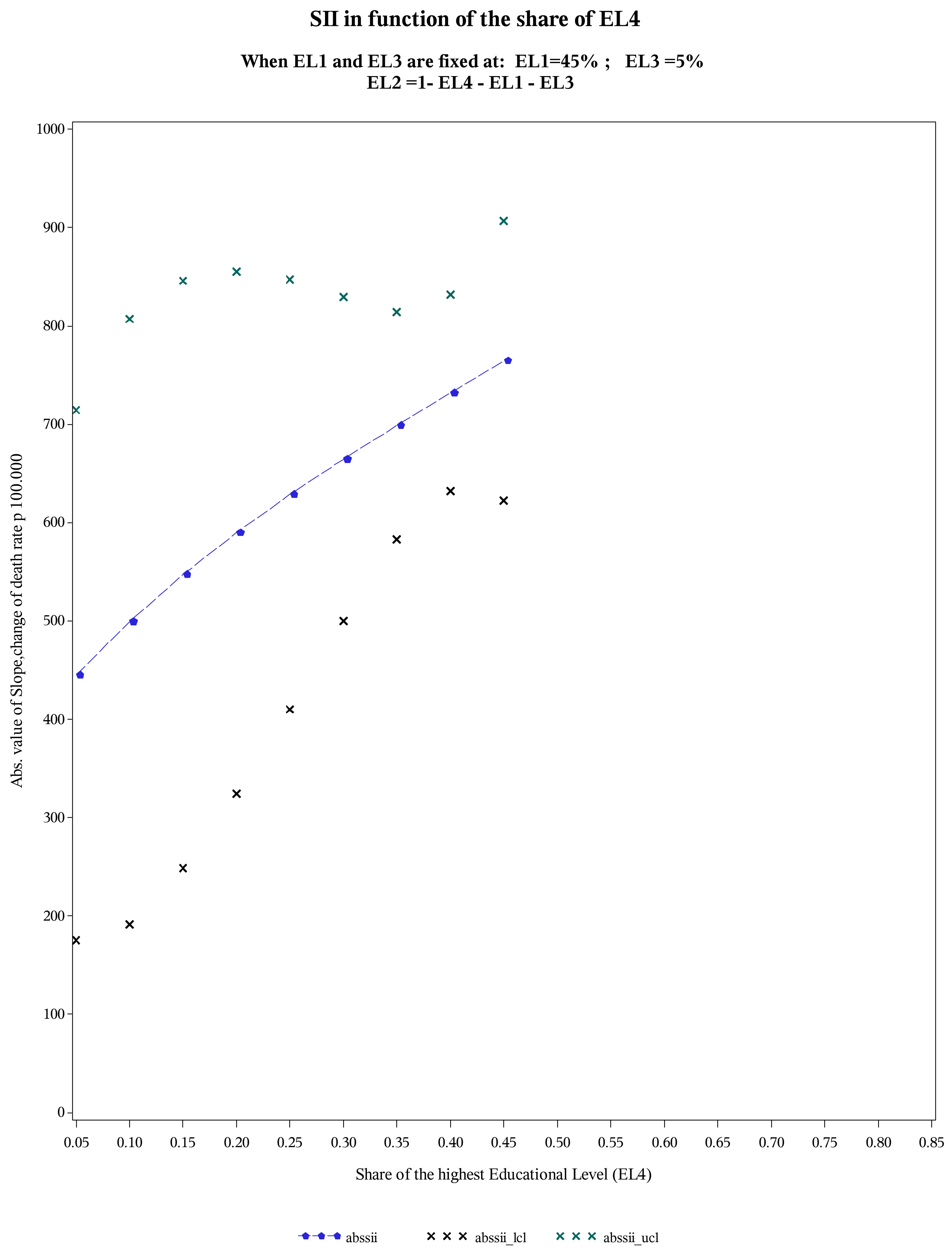
SII in function of the share of EL4
When EL1 and EL3 are fixed at: EL1=45% ; EL3 =5%
EL2 =1- EL4 - EL1 - EL3
Abs. value of Slope,change of death rate p 100.000
1000
900
800
700
600
500
400
300
200
100
0
0.05
0.10
0.15
0.20
0.25
0.30
0.35
0.40
0.45
0.50
0.55
0.60
0.65
0.70
0.75
0.80
0.85
Share of the highest Educational Level (EL4)
abssii
abssii_lcl
abssii_ucl

SII in function of the share of EL4

When EL1 and EL3 are fixed at: EL1=45% ; EL3 =10%
EL2 =1- EL4 - EL1 - EL3

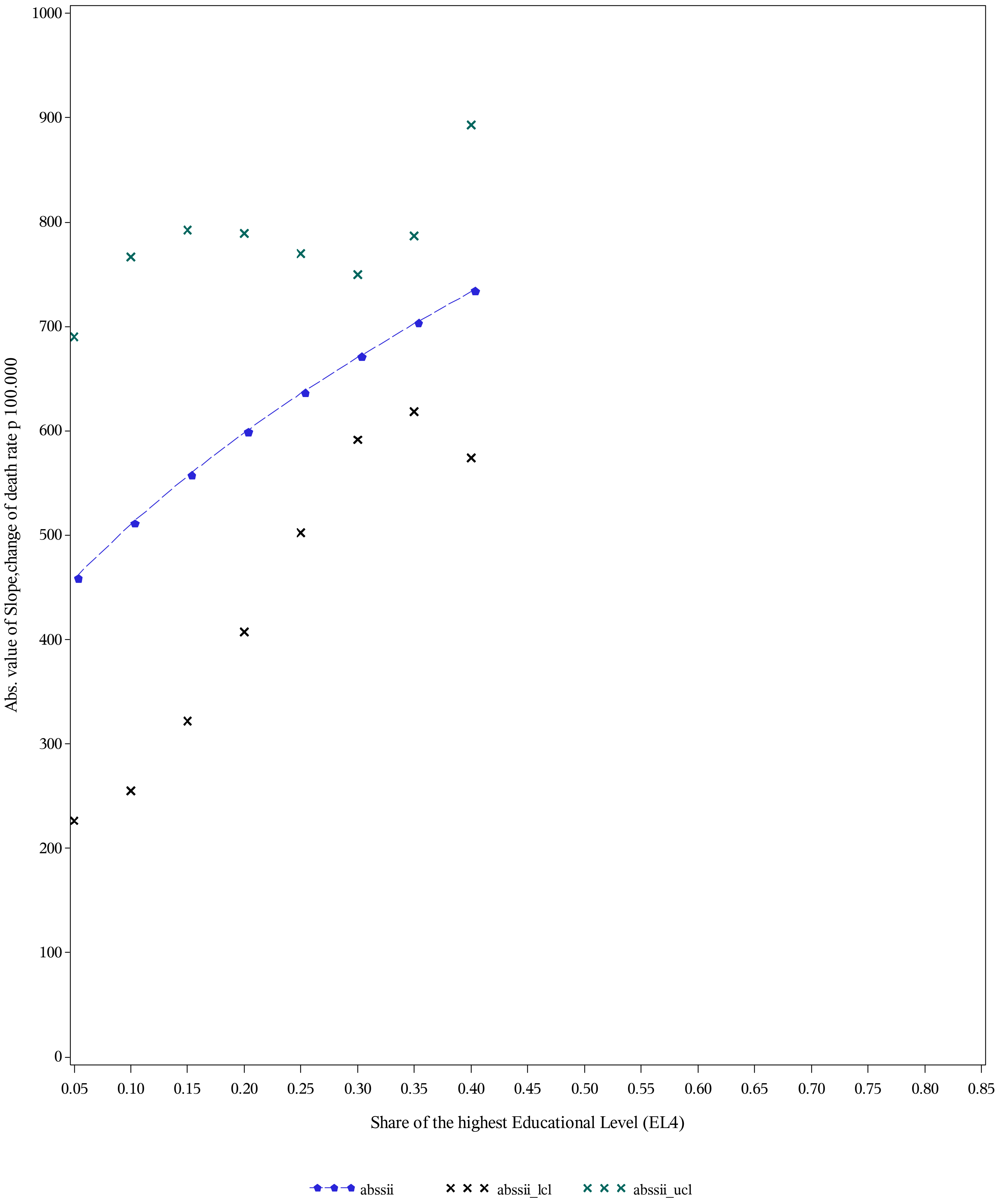

SII in function of the share of EL4

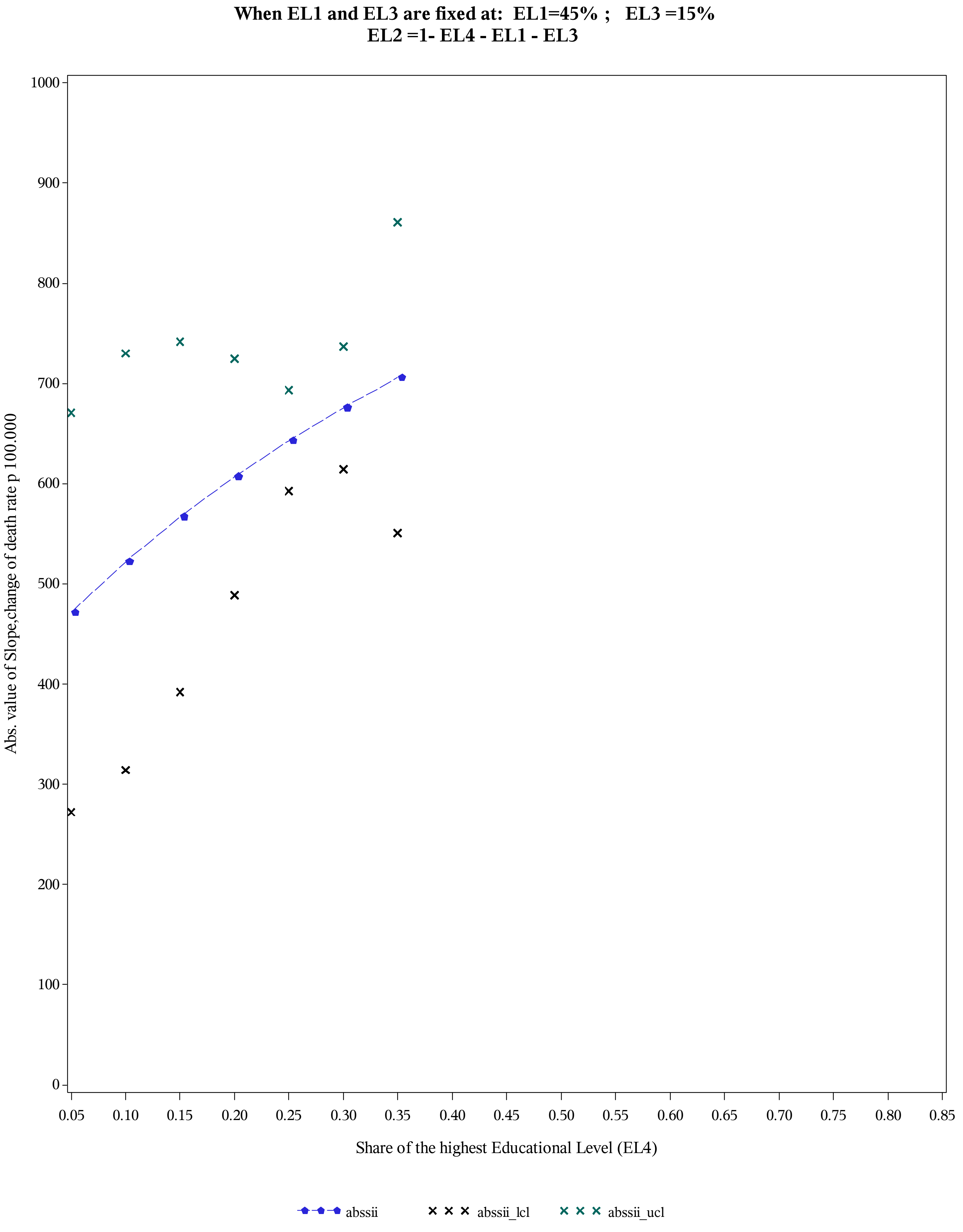

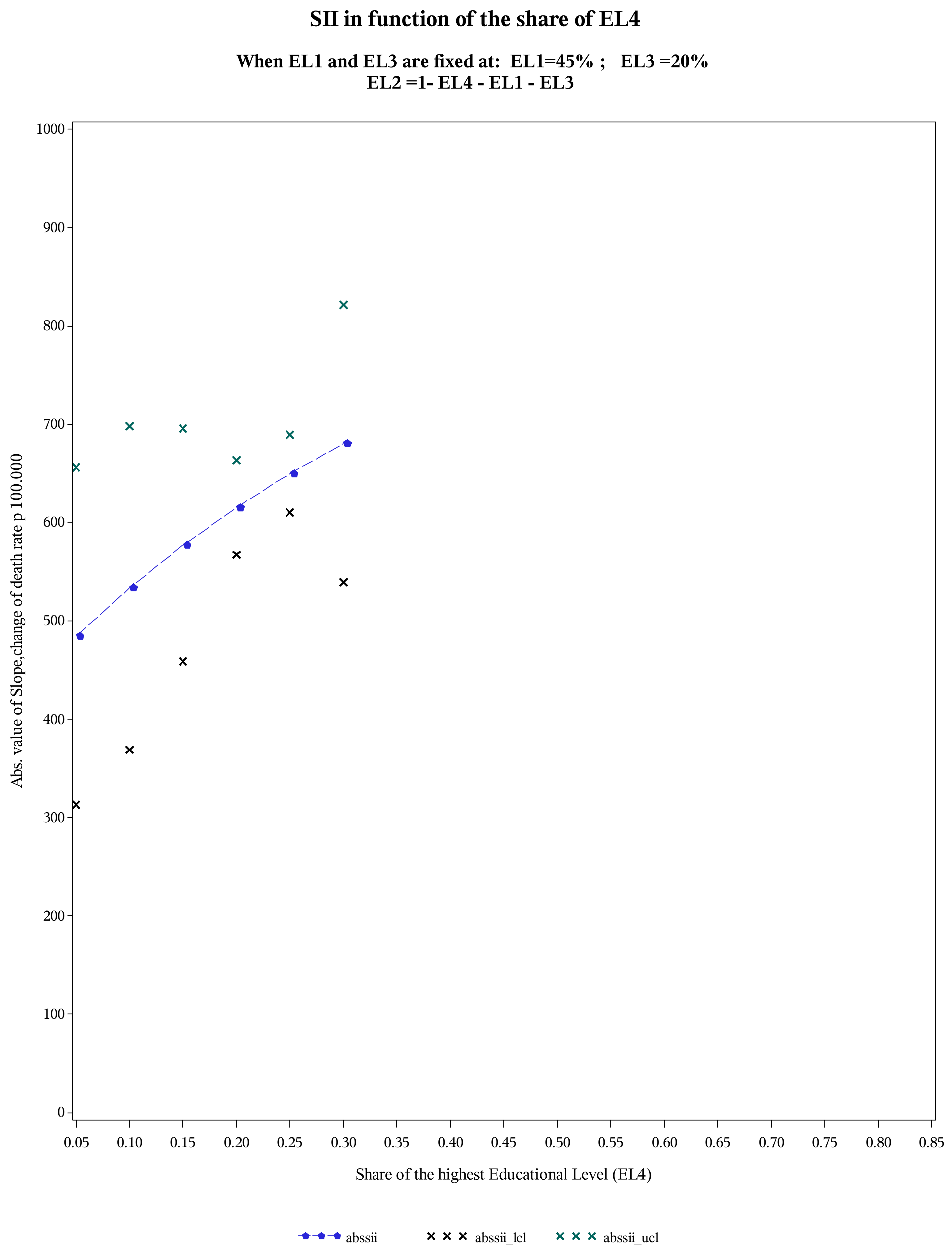

SII in function of the share of EL4
When EL1 and EL3 are fixed at: EL1=45% ; EL3 =20%
EL2 =1- EL4 - EL1 - EL3
Abs. value of Slope,change of death rate p 100.000
0
100
200
300
400
500
600
700
800
900
1000
0.05
0.10
0.15
0.20
0.25
0.30
0.35
0.40
0.45
0.50
0.55
0.60
0.65
0.70
0.75
0.80
0.85
Share of the highest Educational Level (EL4)
abssii
abssii_lcl
abssii_ucl

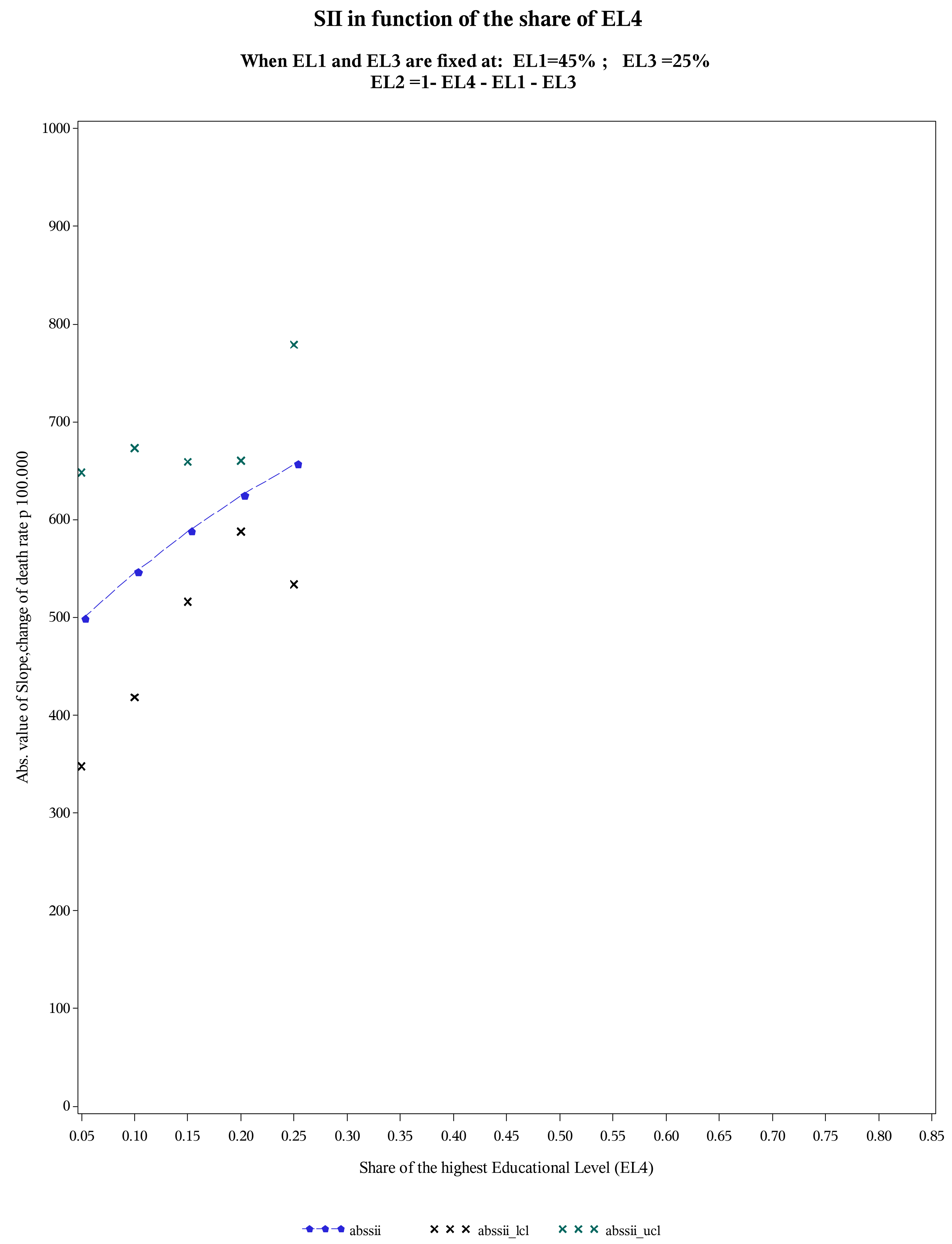
SII in function of the share of EL4
When EL1 and EL3 are fixed at: EL1=45% ; EL3 =25%
EL2 =1- EL4 - EL1 - EL3
Abs. value of Slope,change of death rate p 100.000
Share of the highest Educational Level (EL4)
abssii
abssii_lcl
abssii_ucl

SII in function of the share of EL4

When EL1 and EL3 are fixed at: EL1=45% ; EL3 =30%
EL2 =1- EL4 - EL1 - EL3

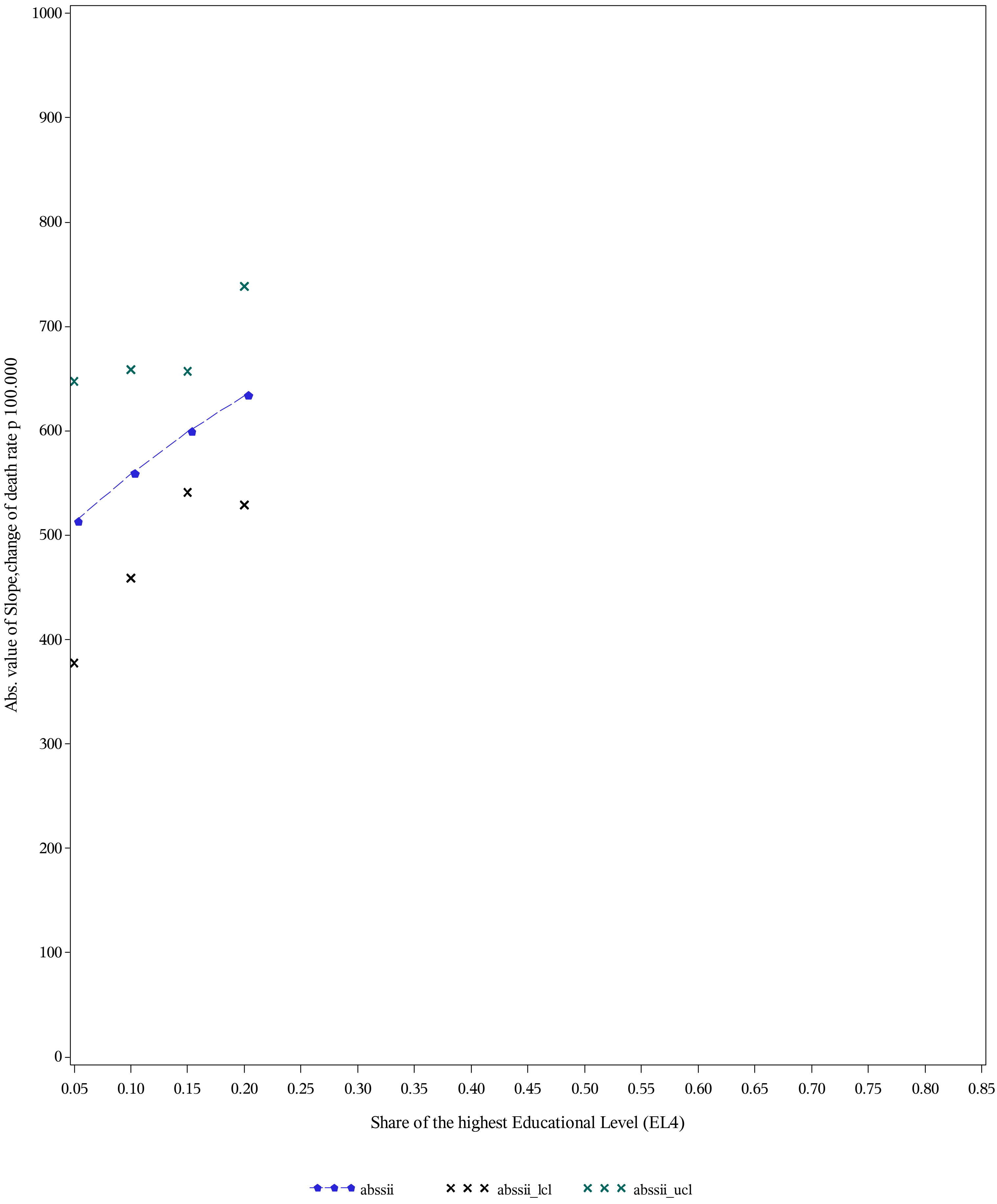

SII in function of the share of EL4

When EL1 and EL3 are fixed at: EL1=45% ; EL3 =35%
EL2 =1- EL4 - EL1 - EL3

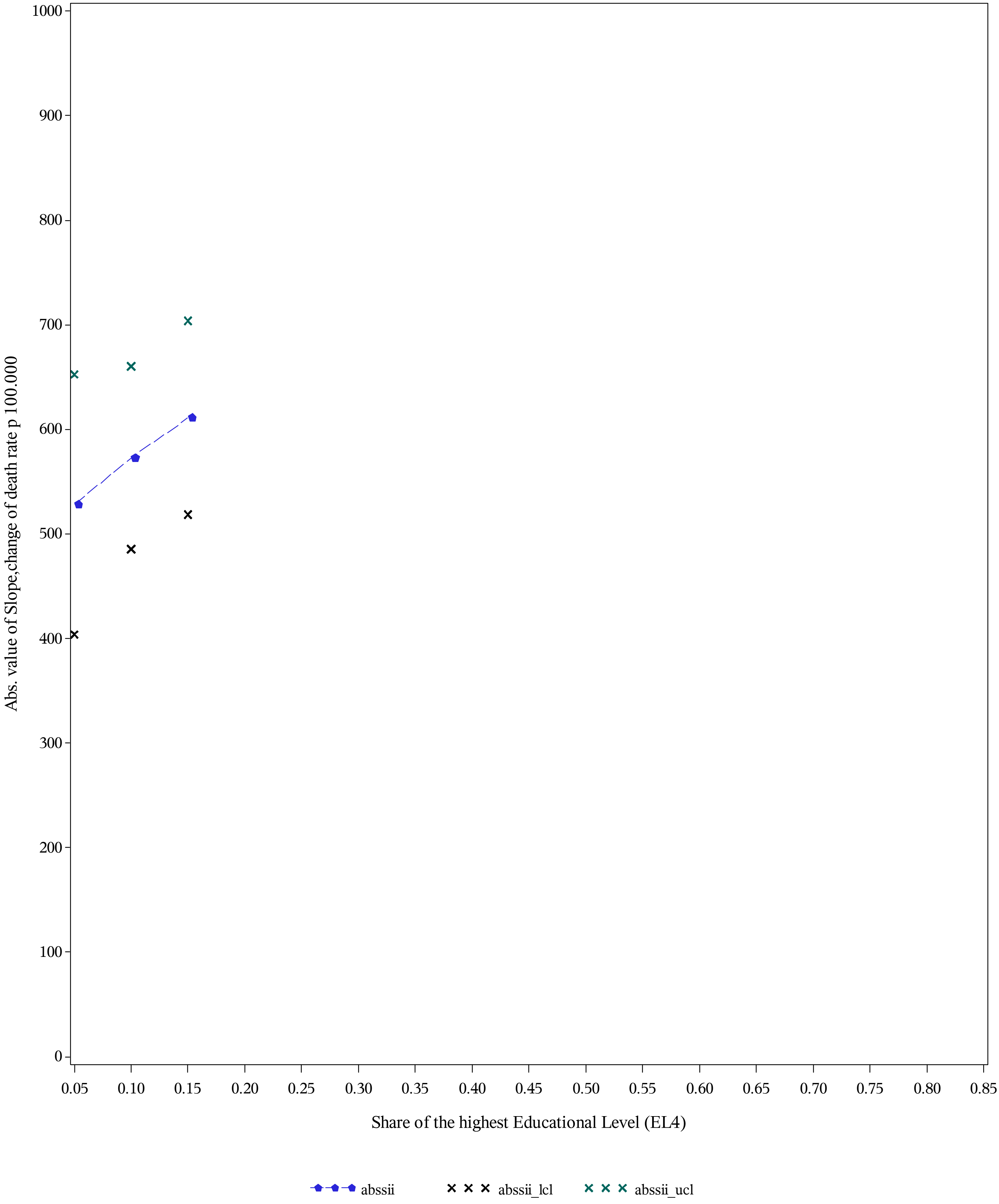

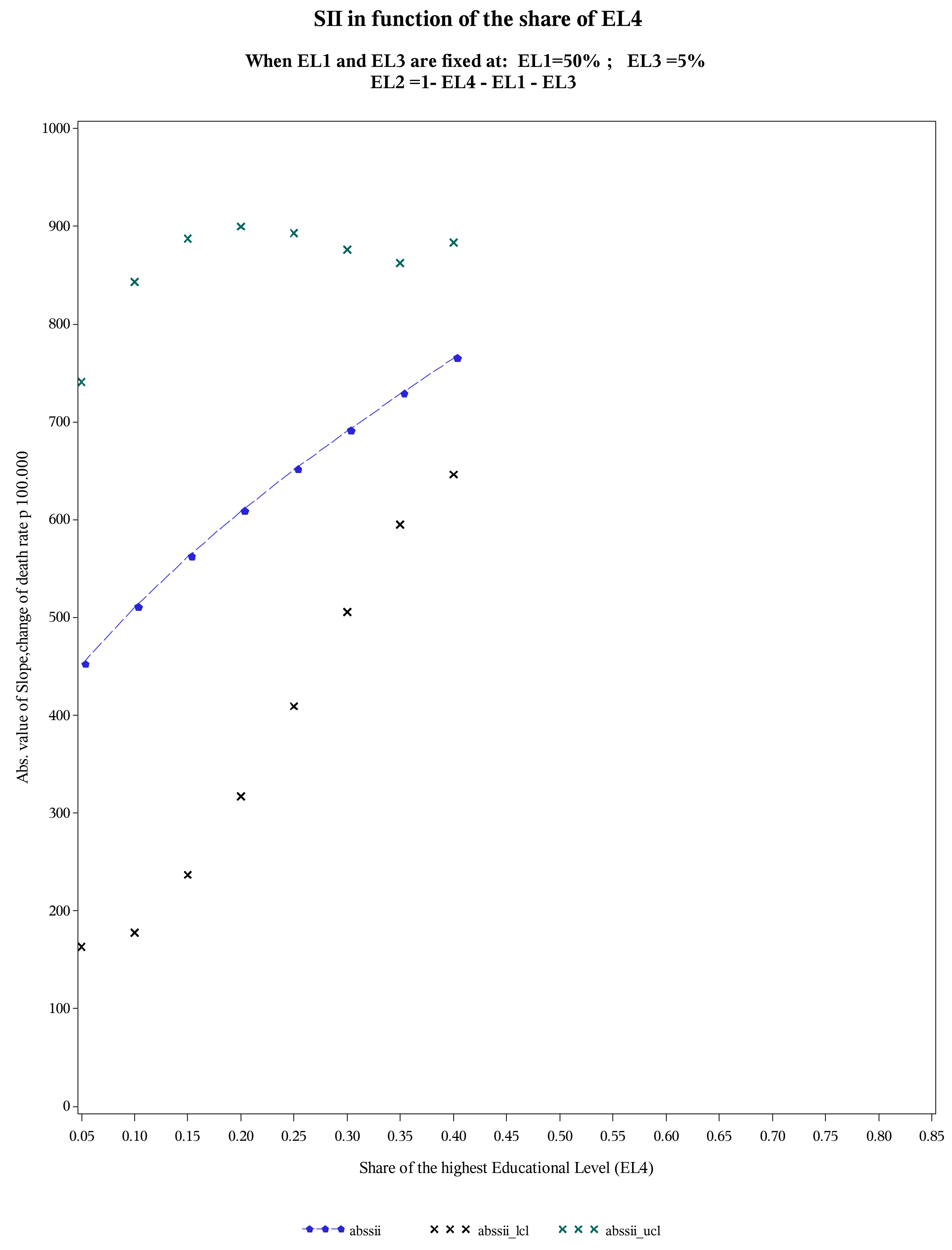

SII in function of the share of EL4
When EL1 and EL3 are fixed at: EL1=50% ; EL3 =5%
EL2 =1- EL4 - EL1 - EL3
Abs. value of Slope,change of death rate p 100.000
1000
900
800
700
600
500
400
300
200
100
0
0.05
0.10
0.15
0.20
0.25
0.30
0.35
0.40
0.45
0.50
0.55
0.60
0.65
0.70
0.75
0.80
0.85
Share of the highest Educational Level (EL4)
abssii
abssii_lcl
abssii_ucl

SII in function of the share of EL4

When EL1 and EL3 are fixed at: EL1=50% ; EL3 =10%
EL2 =1- EL4 - EL1 - EL3

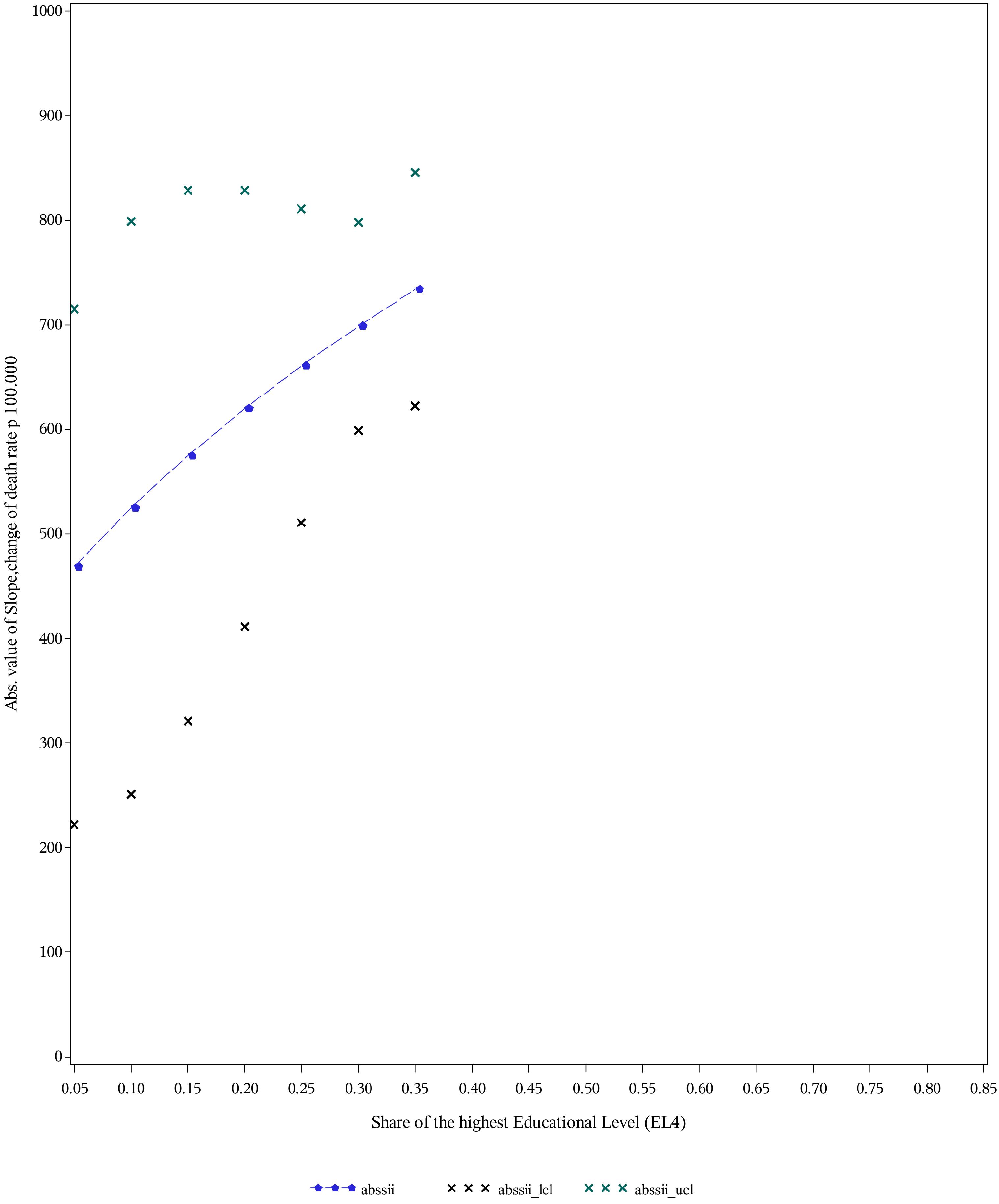

SII in function of the share of EL4

When EL1 and EL3 are fixed at: EL1=50% ; EL3 =15%
EL2 =1- EL4 - EL1 - EL3

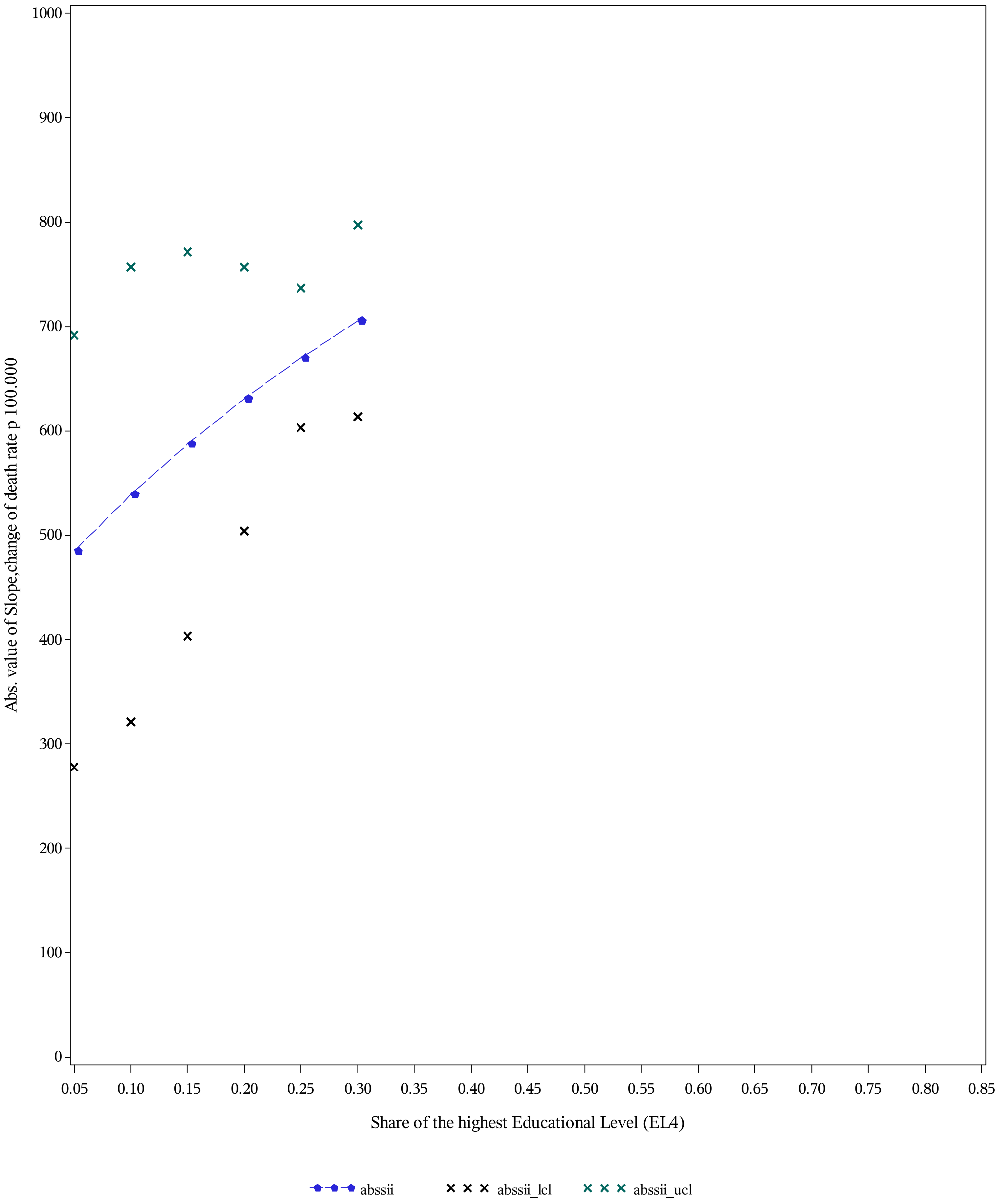

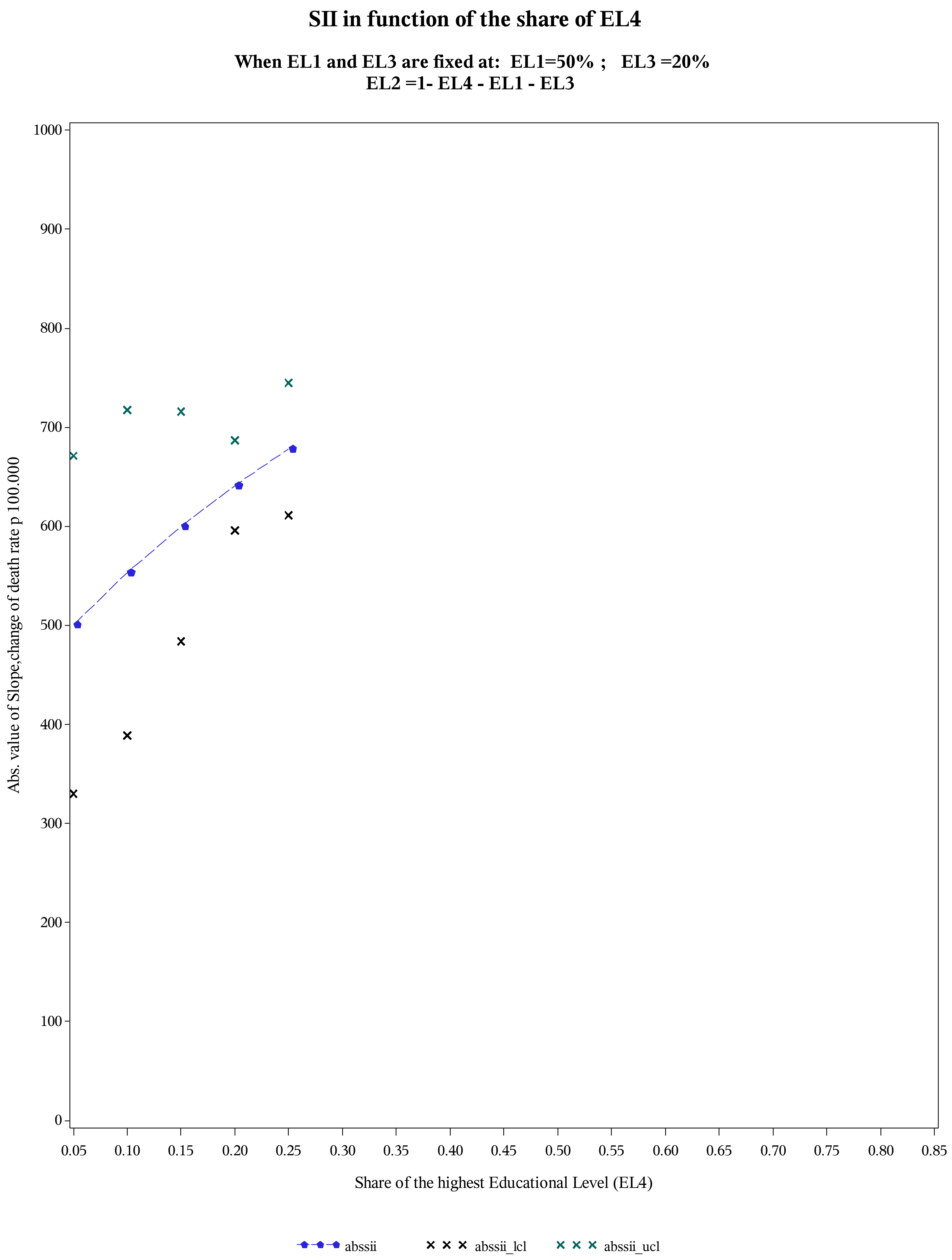
SII in function of the share of EL4
When EL1 and EL3 are fixed at: EL1=50% ; EL3 =20%
EL2 =1- EL4 - EL1 - EL3
Abs. value of Slope,change of death rate p 100.000
0
100
200
300
400
500
600
700
800
900
1000
0.05
0.10
0.15
0.20
0.25
0.30
0.35
0.40
0.45
0.50
0.55
0.60
0.65
0.70
0.75
0.80
0.85
Share of the highest Educational Level (EL4)
abssii
abssii_lcl
abssii_ucl

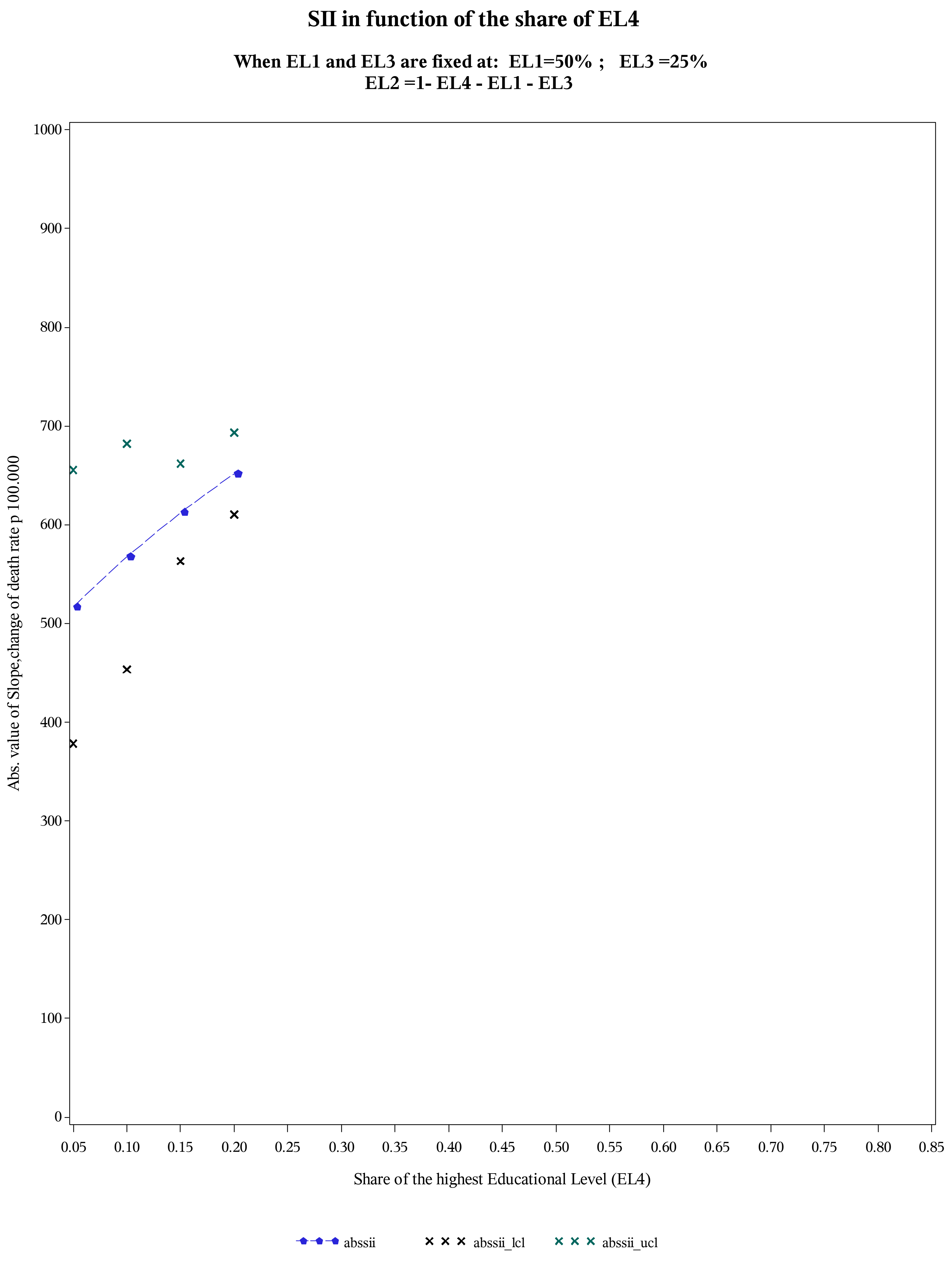
SII in function of the share of EL4
When EL1 and EL3 are fixed at: EL1=50% ; EL3 =25%
EL2 =1- EL4 - EL1 - EL3
Abs. value of Slope,change of death rate p 100.000
0
100
200
300
400
500
600
700
800
900
1000
0.05
0.10
0.15
0.20
0.25
0.30
0.35
0.40
0.45
0.50
0.55
0.60
0.65
0.70
0.75
0.80
0.85
Share of the highest Educational Level (EL4)
abssii
abssii_lcl
abssii_ucl

SII in function of the share of EL4

When EL1 and EL3 are fixed at: EL1=50% ; EL3 =30%
EL2 =1- EL4 - EL1 - EL3

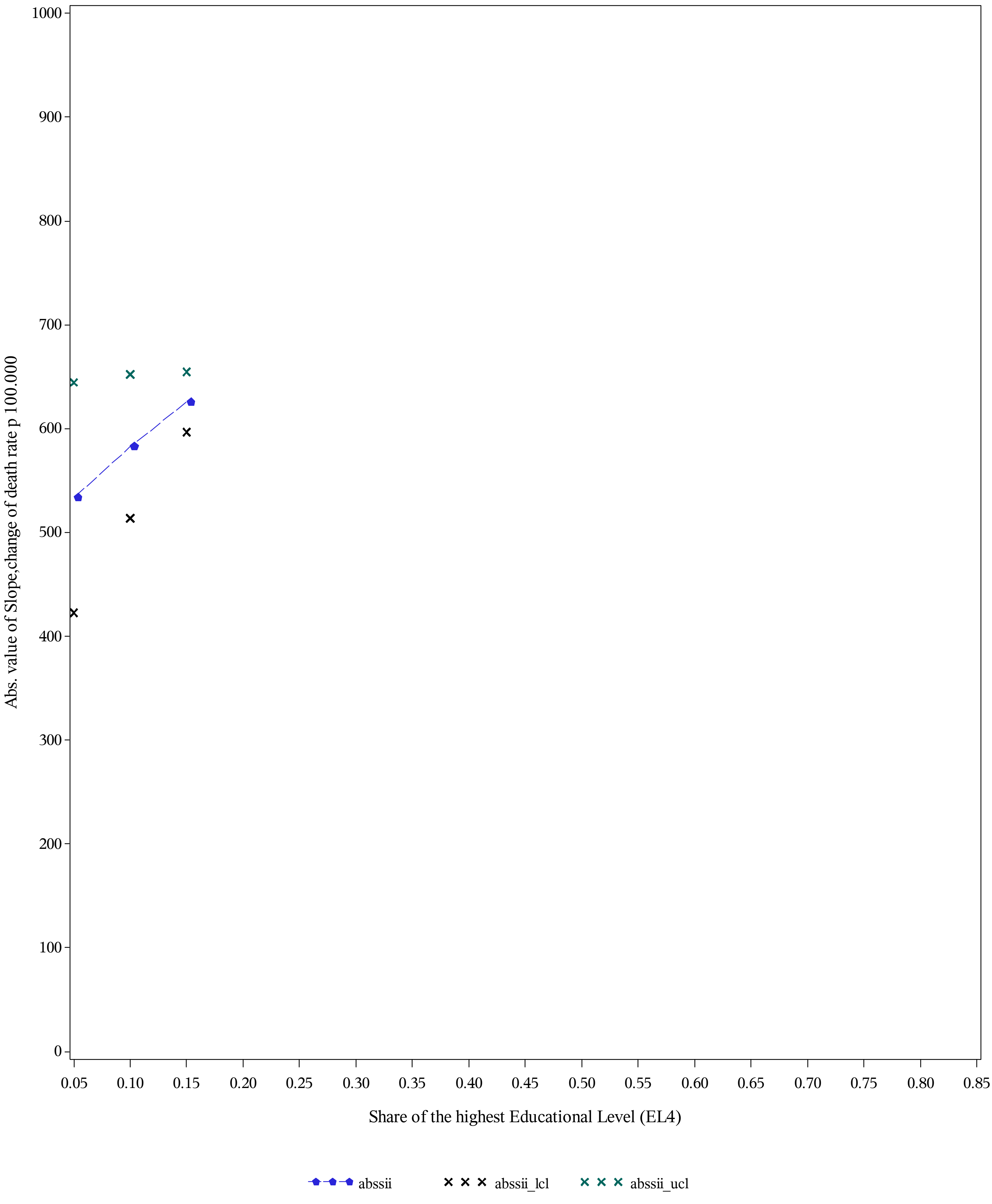

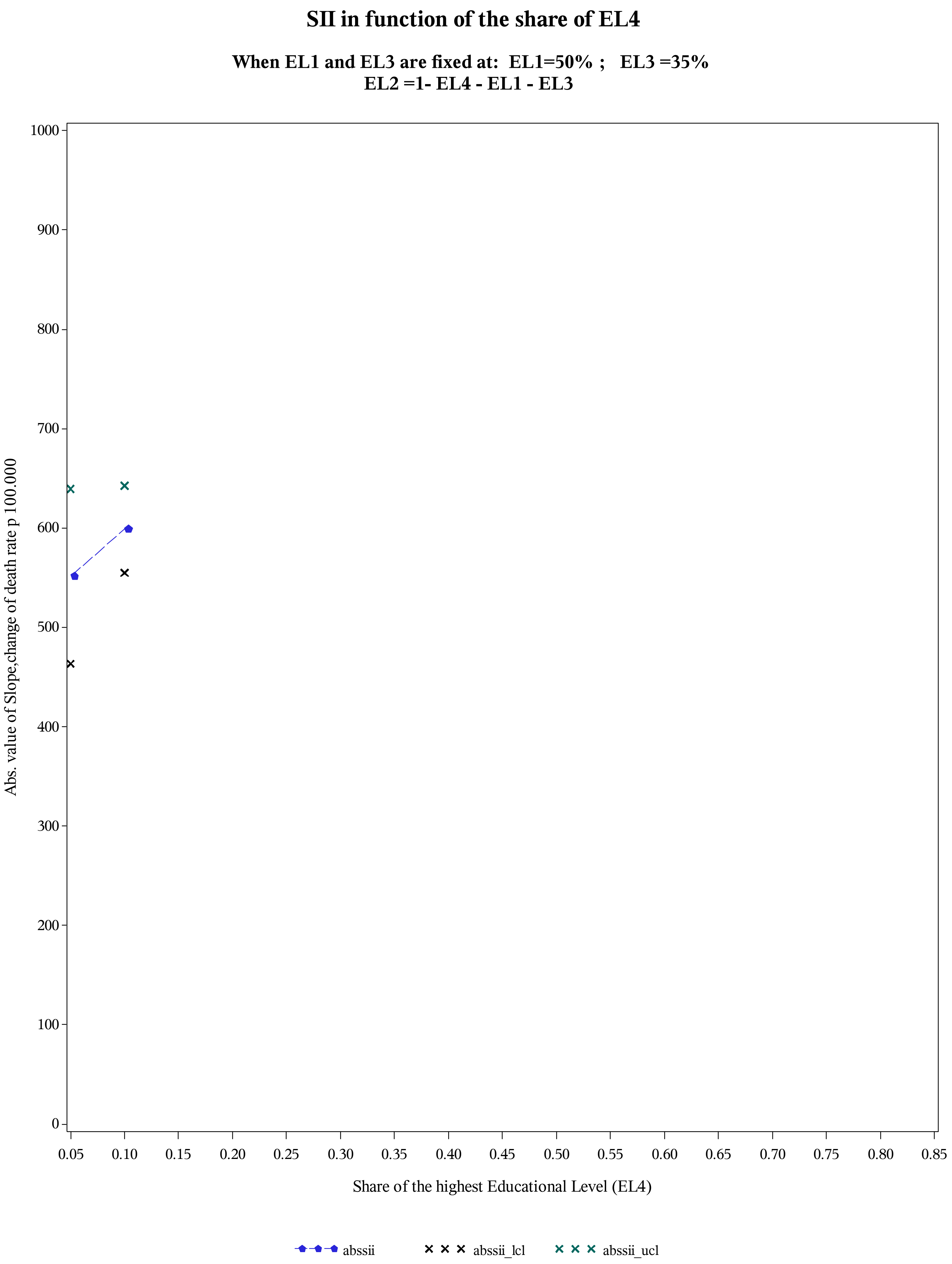
SII in function of the share of EL4
When EL1 and EL3 are fixed at: EL1=50% ; EL3 =35%
EL2 =1- EL4 - EL1 - EL3
Abs. value of Slope,change of death rate p 100.000
1000
900
800
700
600
500
400
300
200
100
0
0.05
0.10
0.15
0.20
0.25
0.30
0.35
0.40
0.45
0.50
0.55
0.60
0.65
0.70
0.75
0.80
0.85
Share of the highest Educational Level (EL4)
abssii
abssii_lcl
abssii_ucl

SII in function of the share of EL4

When EL1 and EL3 are fixed at: EL1=55% ; EL3 =5%
EL2 =1- EL4 - EL1 - EL3

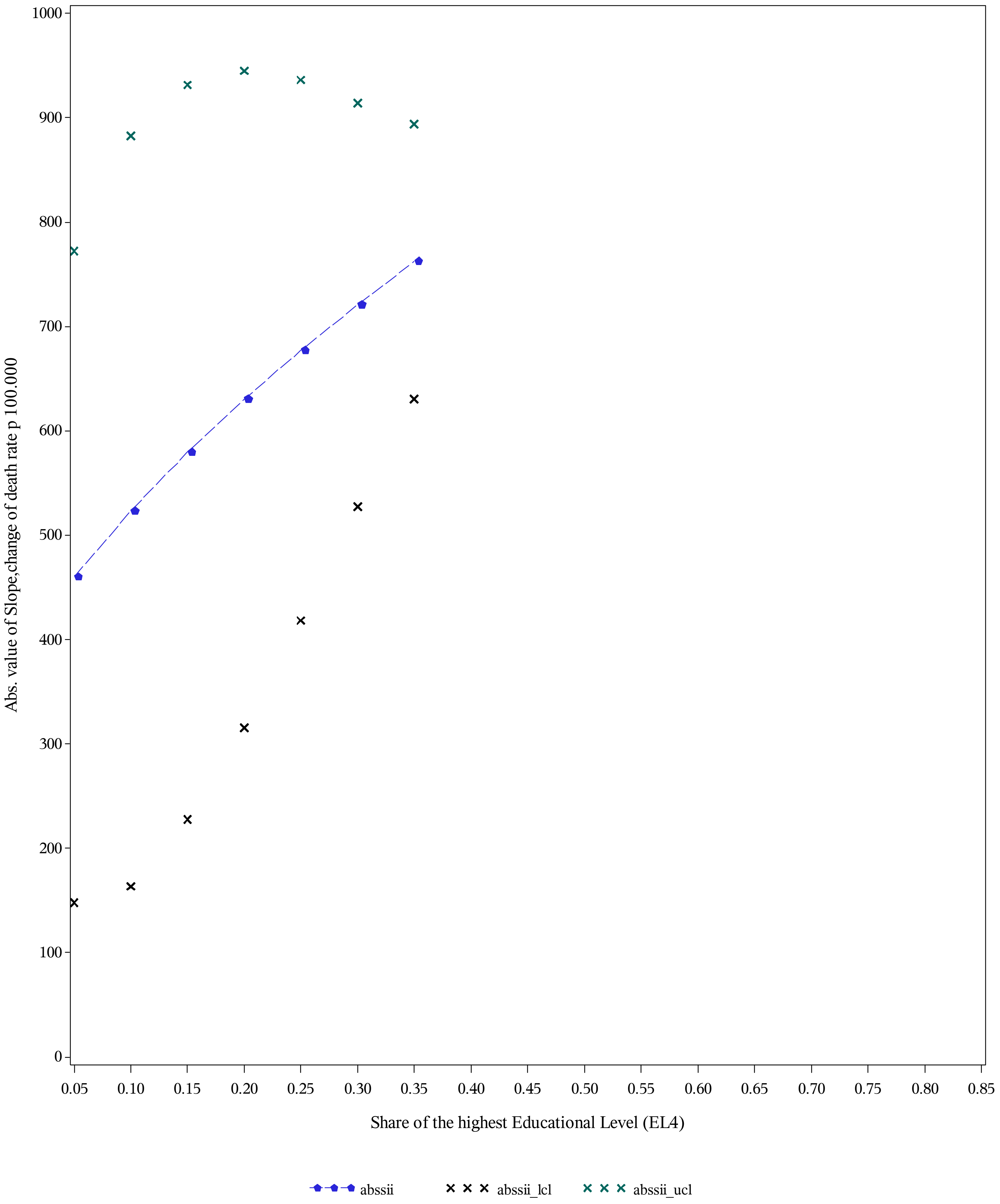

SII in function of the share of EL4

When EL1 and EL3 are fixed at: EL1=55% ; EL3 =10%
EL2 =1- EL4 - EL1 - EL3

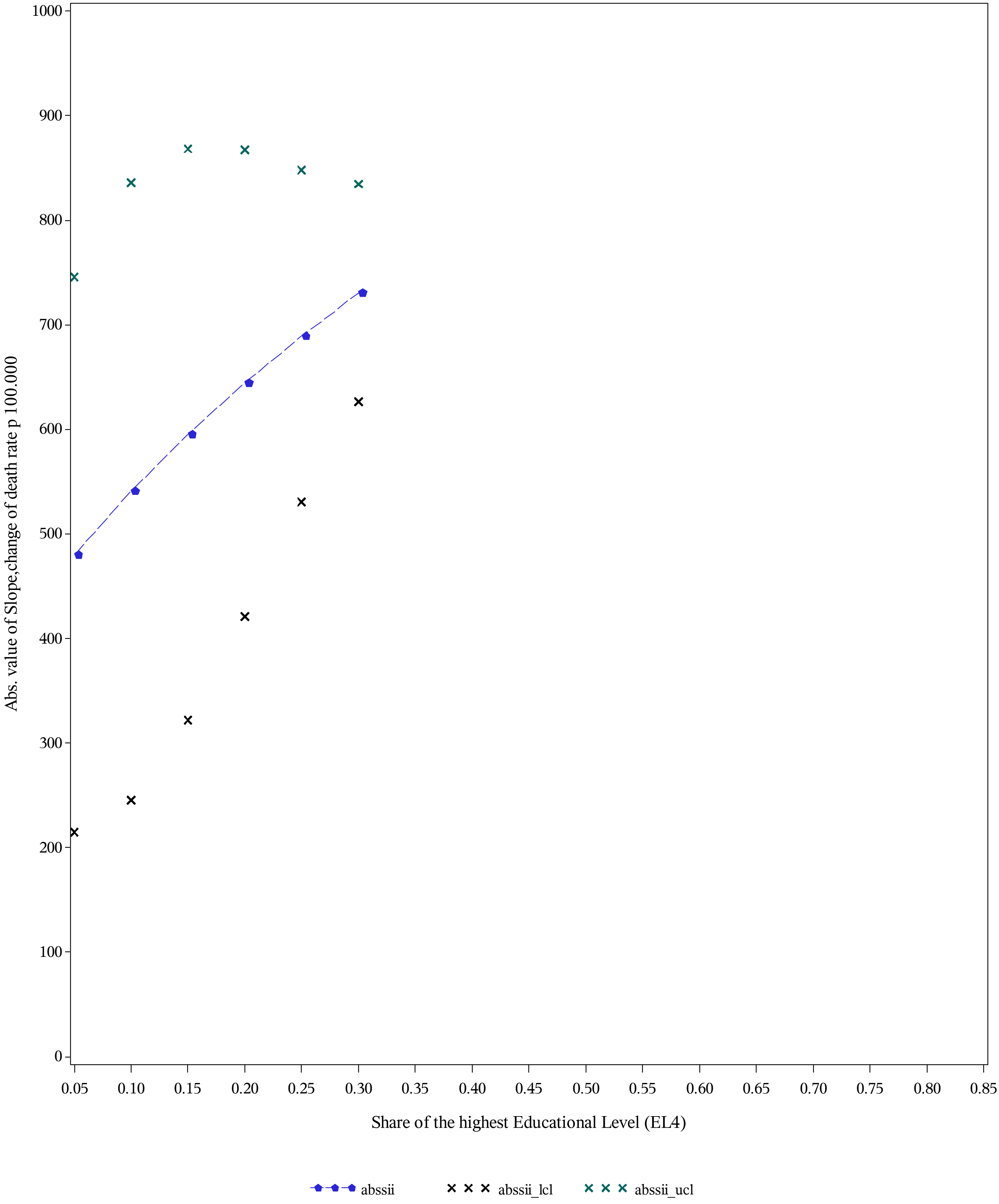

SII in function of the share of EL4

When EL1 and EL3 are fixed at: EL1=55% ; EL3 =15%
EL2 =1- EL4 - EL1 - EL3

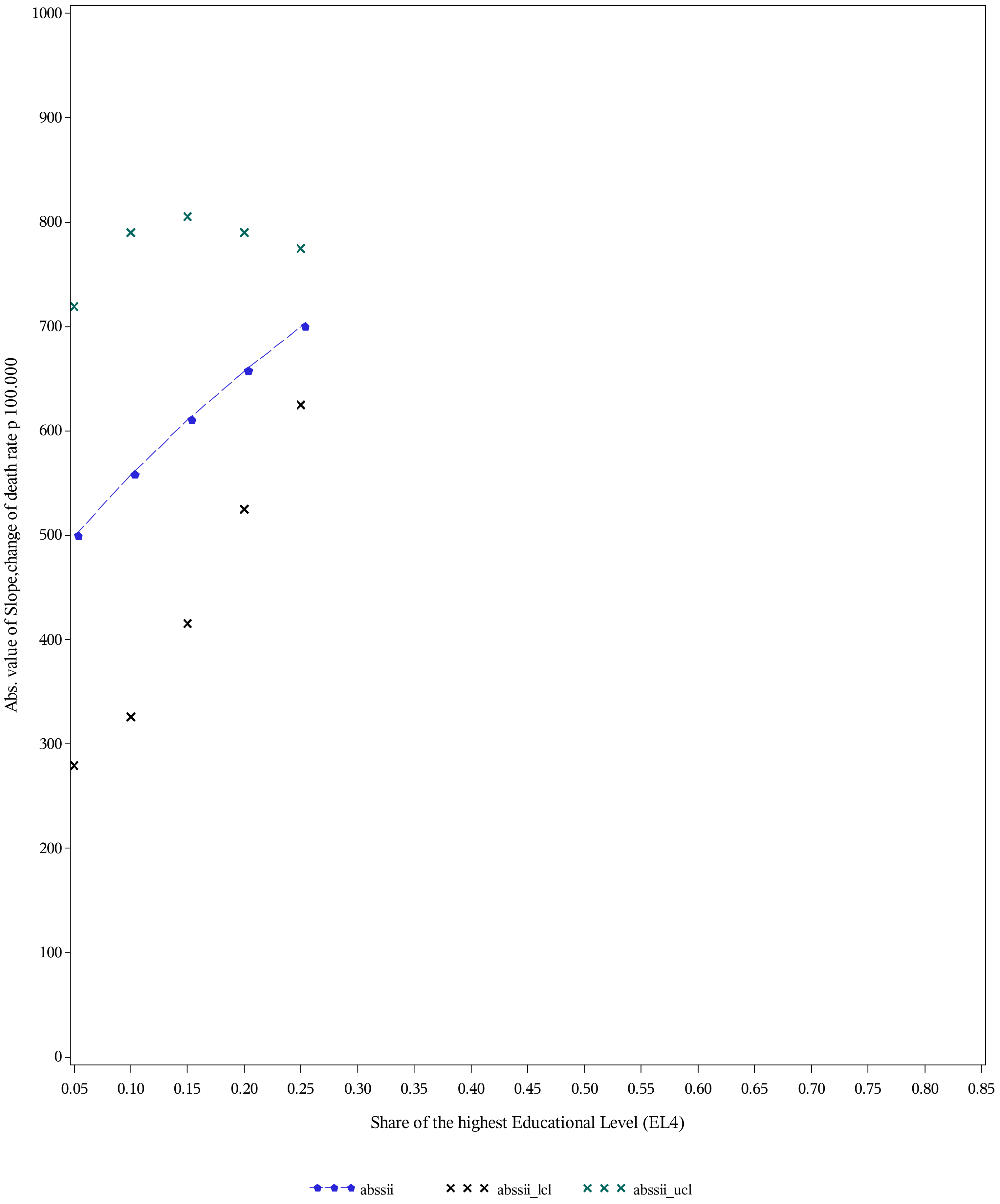

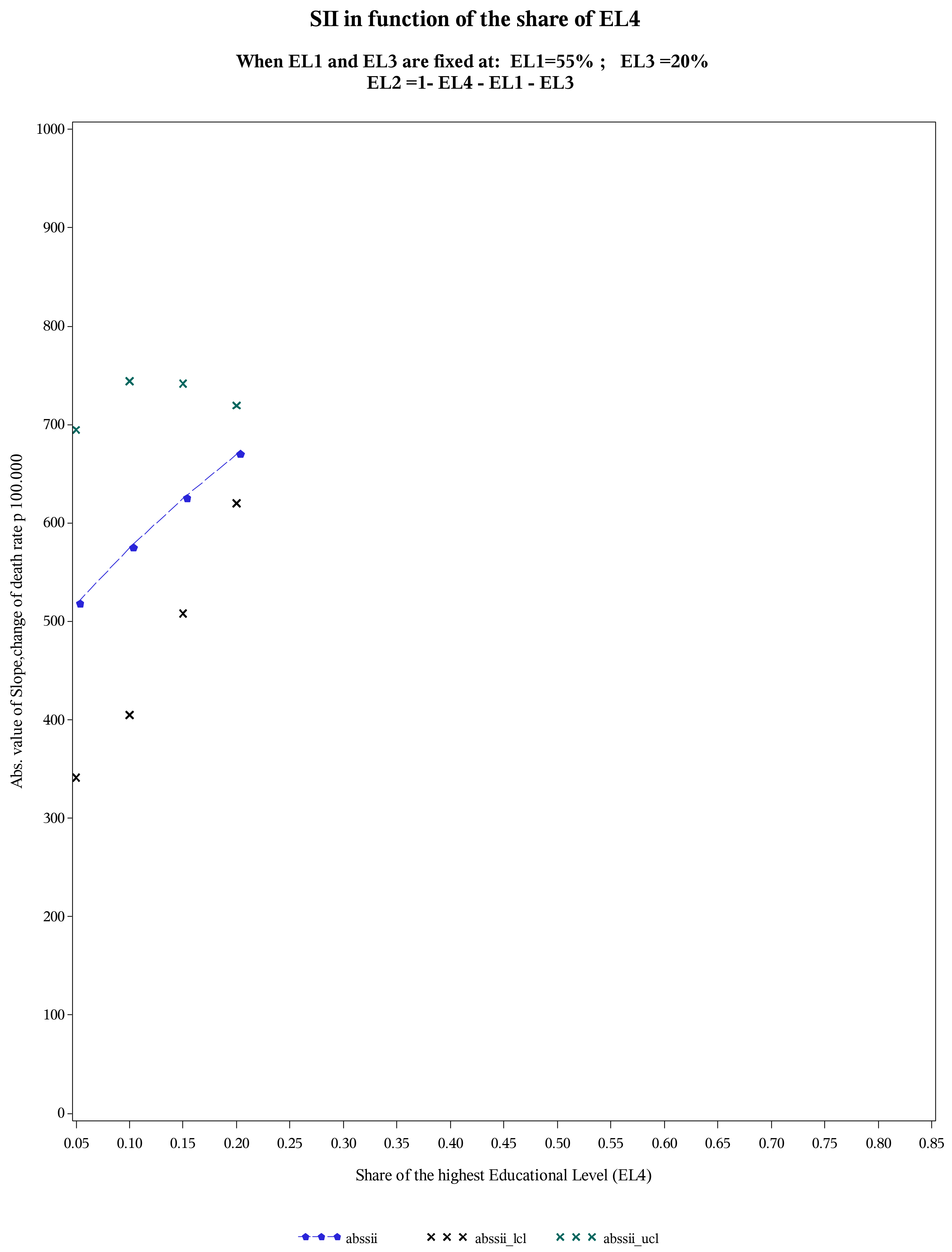
SII in function of the share of EL4
When EL1 and EL3 are fixed at: EL1=55% ; EL3 =20%
EL2 =1- EL4 - EL1 - EL3
Abs. value of Slope,change of death rate p 100.000
1000
900
800
700
600
500
400
300
200
100
0
0.05
0.10
0.15
0.20
0.25
0.30
0.35
0.40
0.45
0.50
0.55
0.60
0.65
0.70
0.75
0.80
0.85
Share of the highest Educational Level (EL4)
abssii
abssii_lcl
abssii_ucl

SII in function of the share of EL4

When EL1 and EL3 are fixed at: EL1=55% ; EL3 =25%
EL2 =1- EL4 - EL1 - EL3

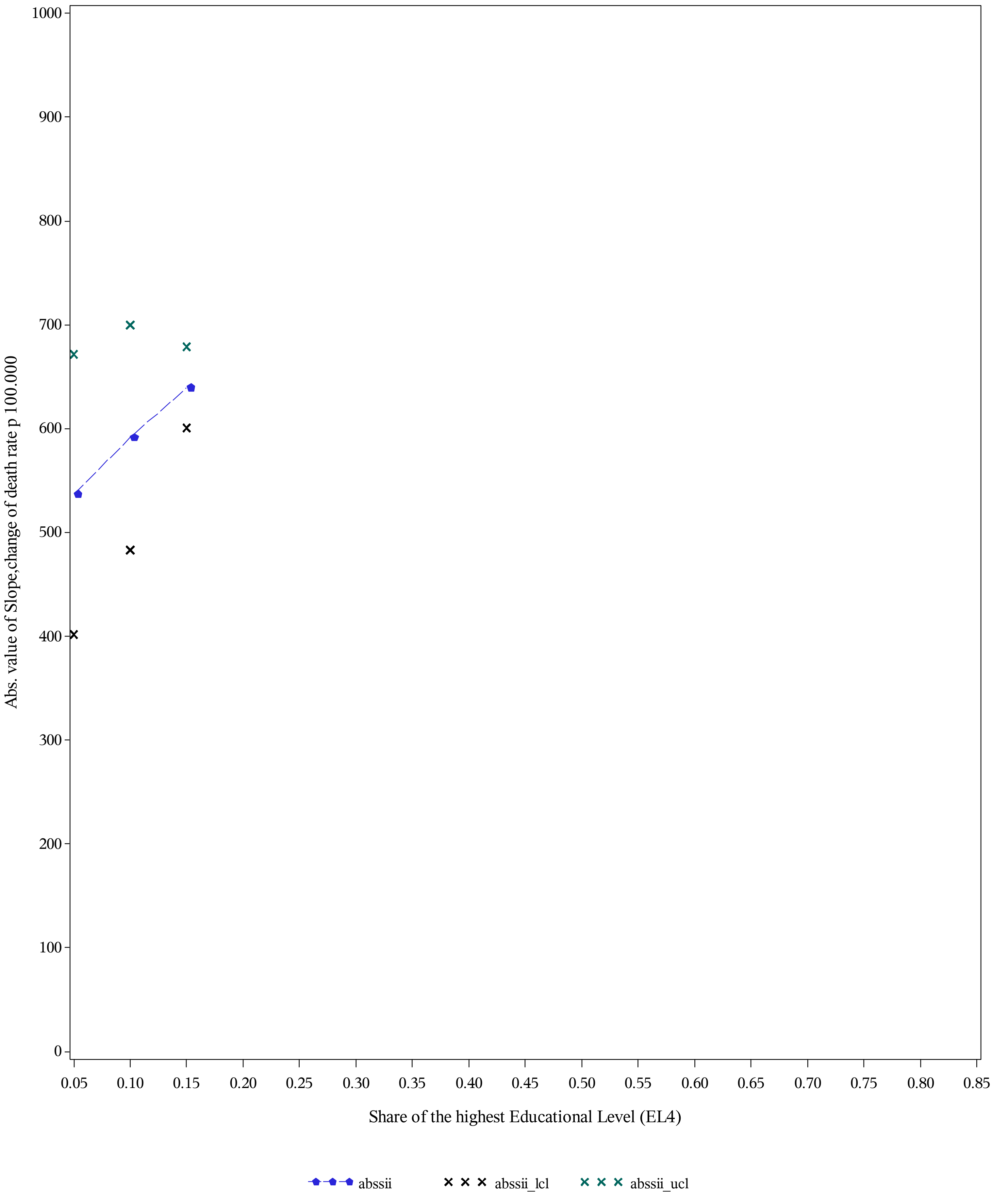

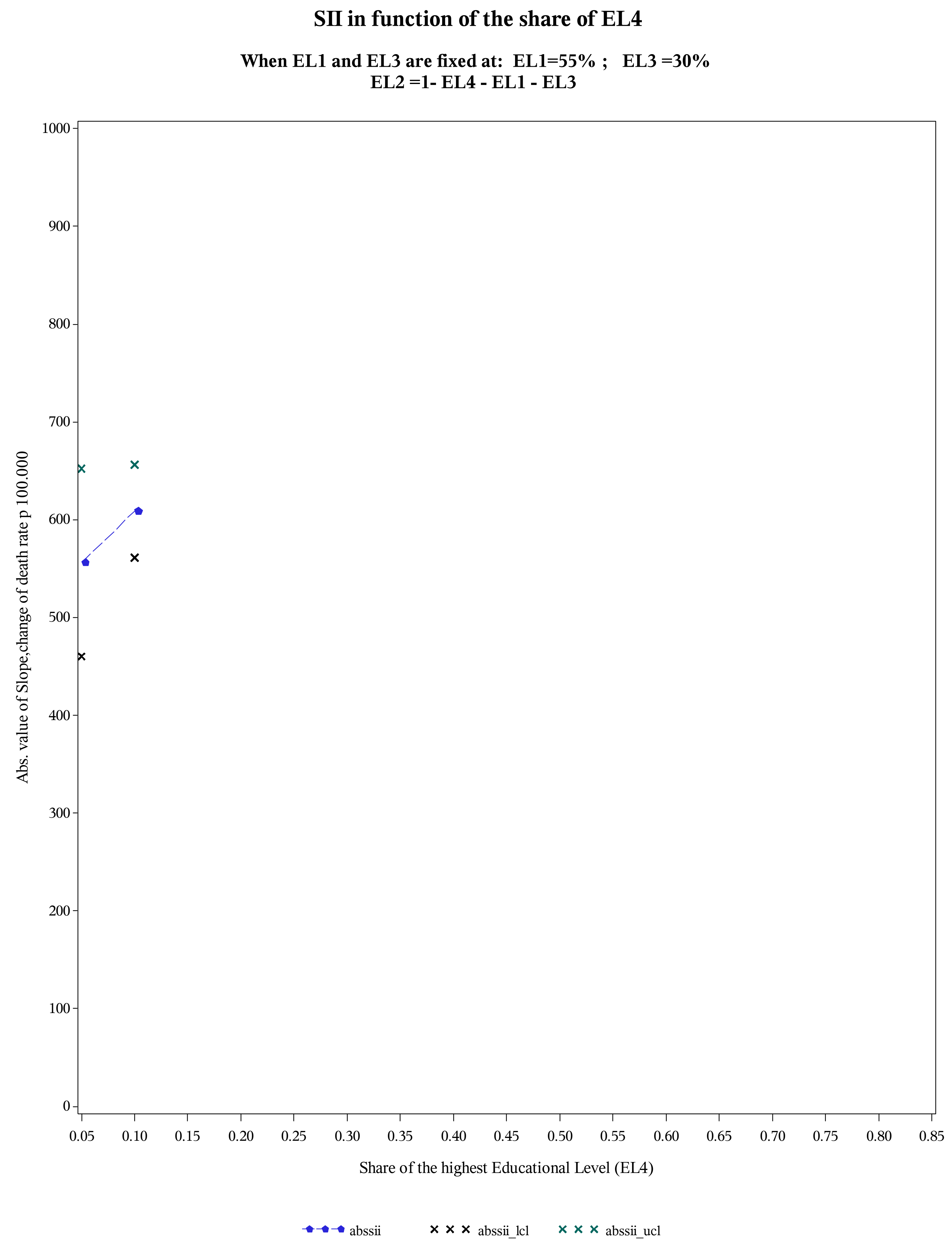

SII in function of the share of EL4
When EL1 and EL3 are fixed at: EL1=55% ; EL3 =30%
EL2 =1- EL4 - EL1 - EL3
Abs. value of Slope,change of death rate p 100.000
1000
900
800
700
600
500
400
300
200
100
0
0.05
0.10
0.15
0.20
0.25
0.30
0.35
0.40
0.45
0.50
0.55
0.60
0.65
0.70
0.75
0.80
0.85
Share of the highest Educational Level (EL4)
abssii
abssii_lcl
abssii_ucl

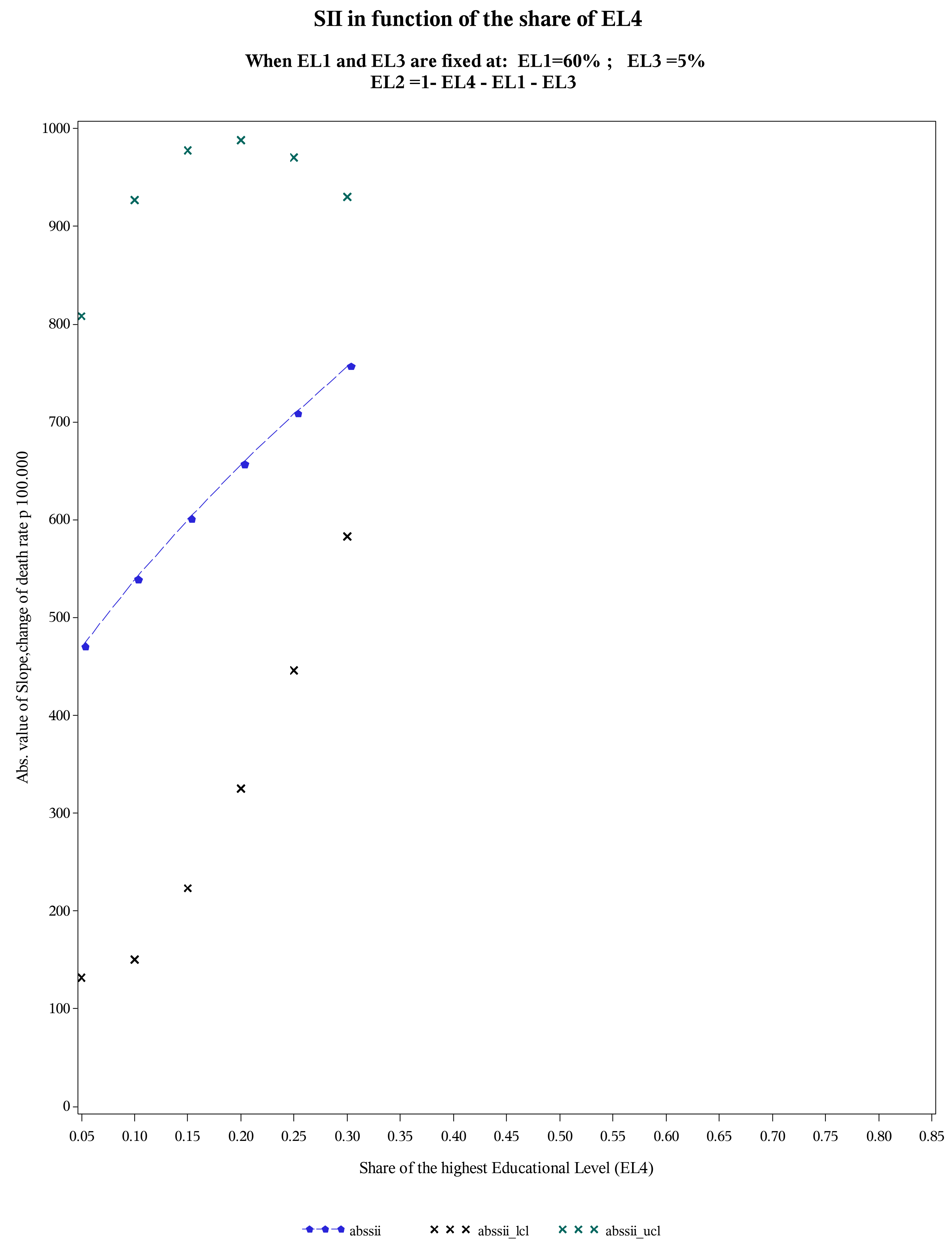

SII in function of the share of EL4
When EL1 and EL3 are fixed at: EL1=60% ; EL3 =5%
EL2 =1- EL4 - EL1 - EL3
Abs. value of Slope,change of death rate p 100.000
0
100
200
300
400
500
600
700
800
900
1000
0.05
0.10
0.15
0.20
0.25
0.30
0.35
0.40
0.45
0.50
0.55
0.60
0.65
0.70
0.75
0.80
0.85
Share of the highest Educational Level (EL4)
abssii
abssii_lcl
abssii_ucl

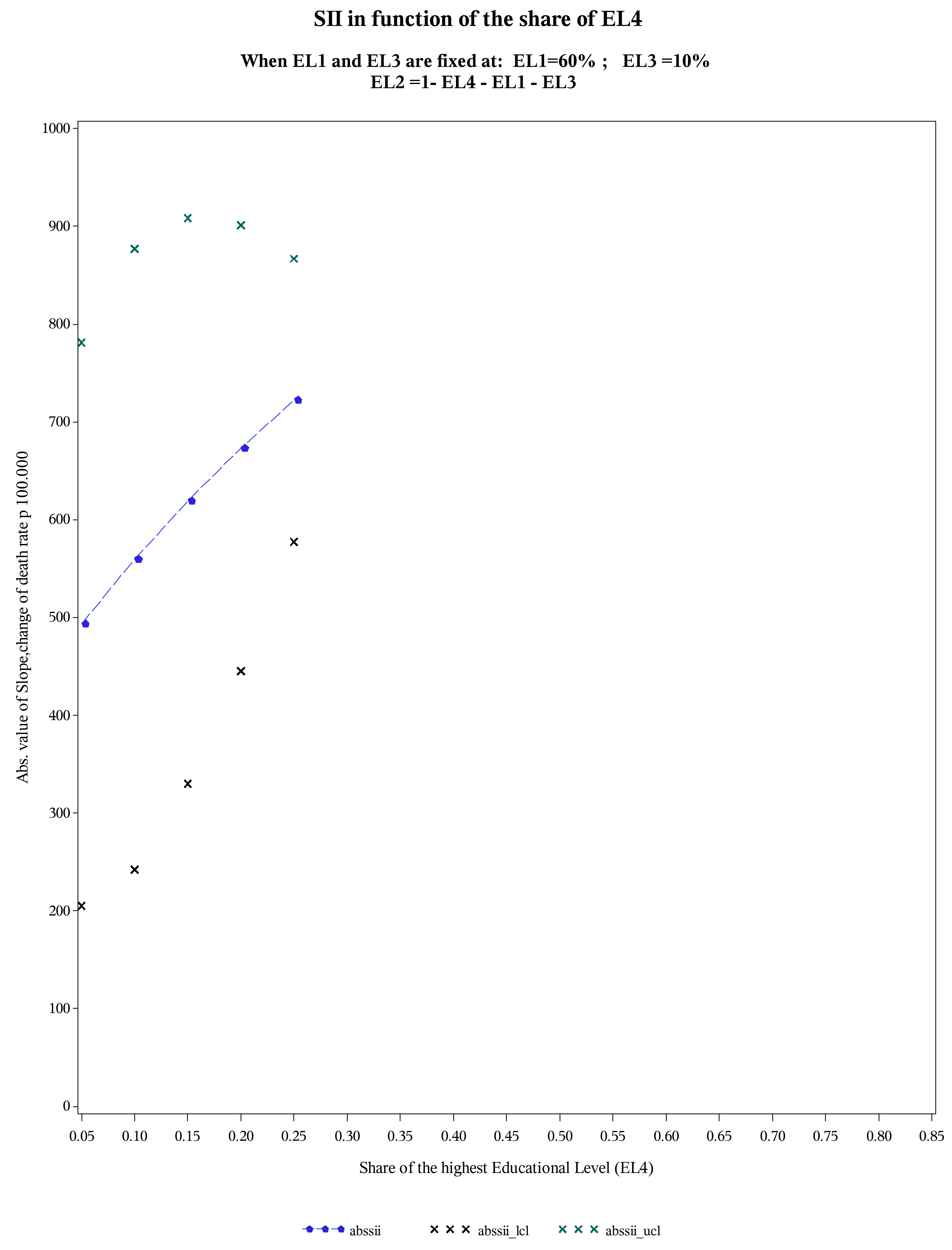

SII in function of the share of EL4
When EL1 and EL3 are fixed at: EL1=60% ; EL3 =10%
EL2 =1- EL4 - EL1 - EL3
Abs. value of Slope,change of death rate p 100.000
1000
900
800
700
600
500
400
300
200
100
0
0.05
0.10
0.15
0.20
0.25
0.30
0.35
0.40
0.45
0.50
0.55
0.60
0.65
0.70
0.75
0.80
0.85
Share of the highest Educational Level (EL4)
abssii
abssii_lcl
abssii_ucl

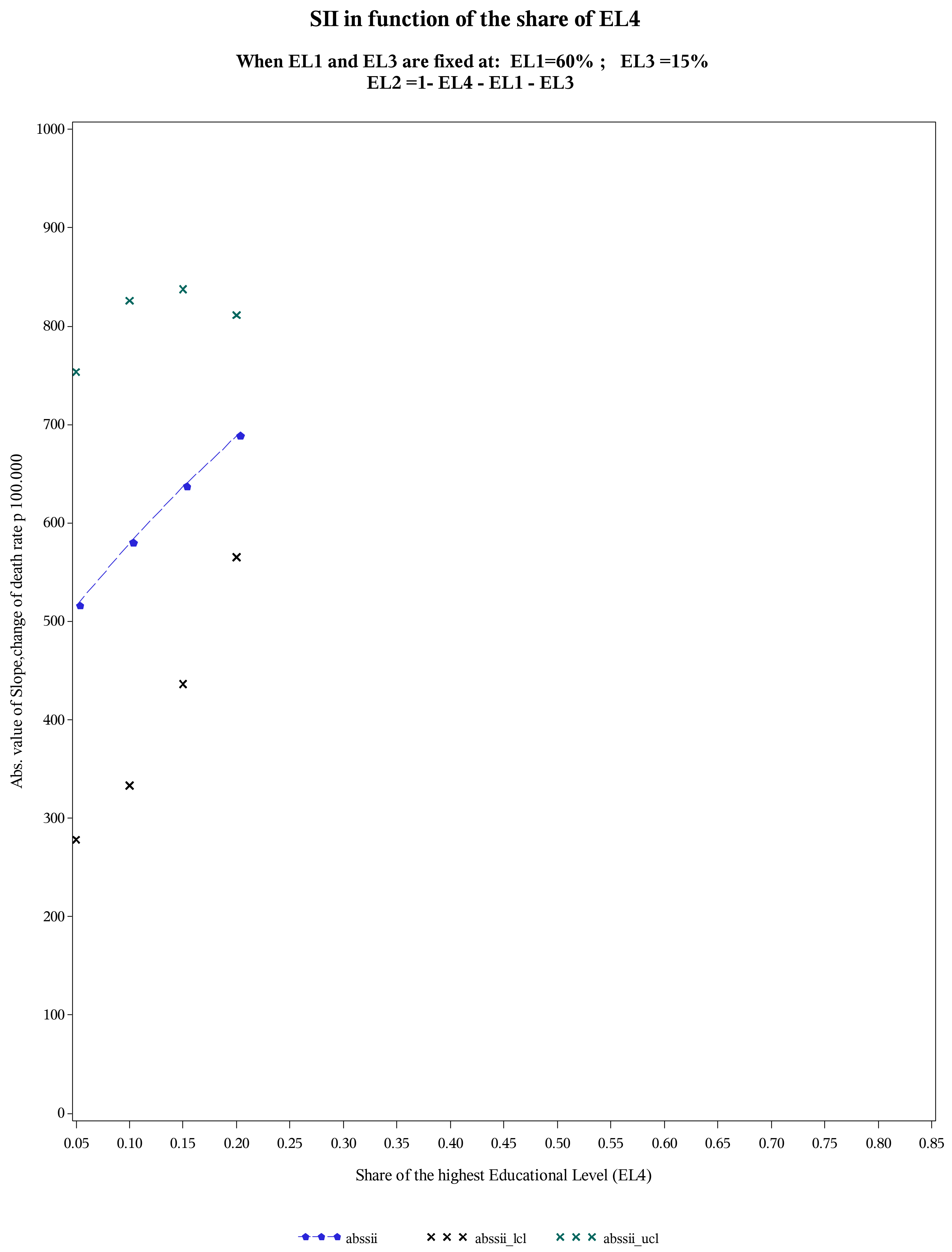

SII in function of the share of EL4
When EL1 and EL3 are fixed at: EL1=60% ; EL3 =15%
EL2 =1- EL4 - EL1 - EL3
Abs. value of Slope,change of death rate p 100.000
1000
900
800
700
600
500
400
300
200
100
0
0.05
0.10
0.15
0.20
0.25
0.30
0.35
0.40
0.45
0.50
0.55
0.60
0.65
0.70
0.75
0.80
0.85
Share of the highest Educational Level (EL4)
abssii
abssii_lcl
abssii_ucl

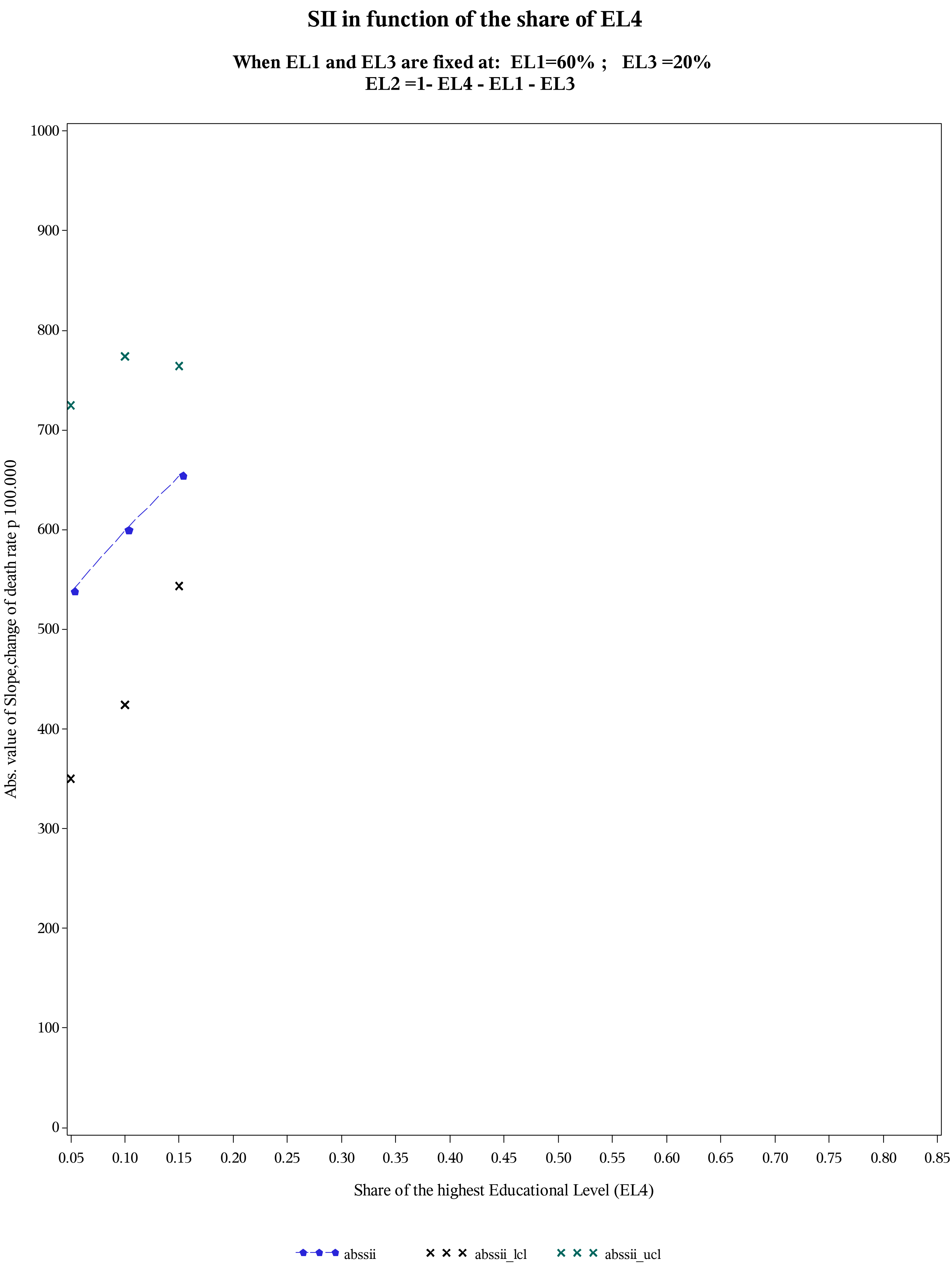

SII in function of the share of EL4
When EL1 and EL3 are fixed at: EL1=60% ; EL3 =20%
EL2 =1- EL4 - EL1 - EL3
Abs. value of Slope,change of death rate p 100.000
0
100
200
300
400
500
600
700
800
900
1000
0.05
0.10
0.15
0.20
0.25
0.30
0.35
0.40
0.45
0.50
0.55
0.60
0.65
0.70
0.75
0.80
0.85
Share of the highest Educational Level (EL4)
abssii
abssii_lcl
abssii_ucl

SII in function of the share of EL4

When EL1 and EL3 are fixed at: EL1=60% ; EL3 =25%
EL2 =1- EL4 - EL1 - EL3

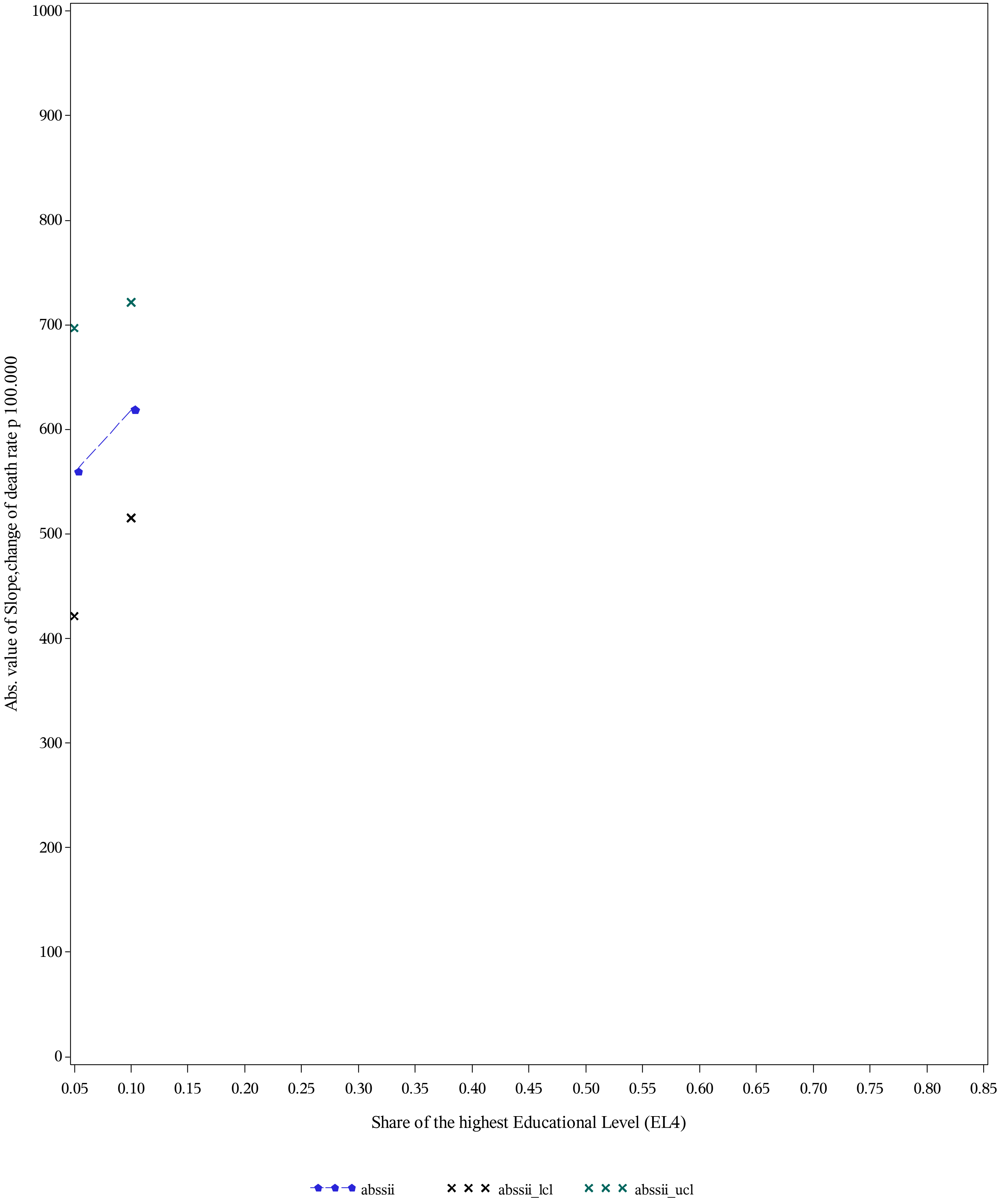

SII in function of the share of EL4

When EL1 and EL3 are fixed at: EL1=65% ; EL3 =5%
EL2 =1- EL4 - EL1 - EL3

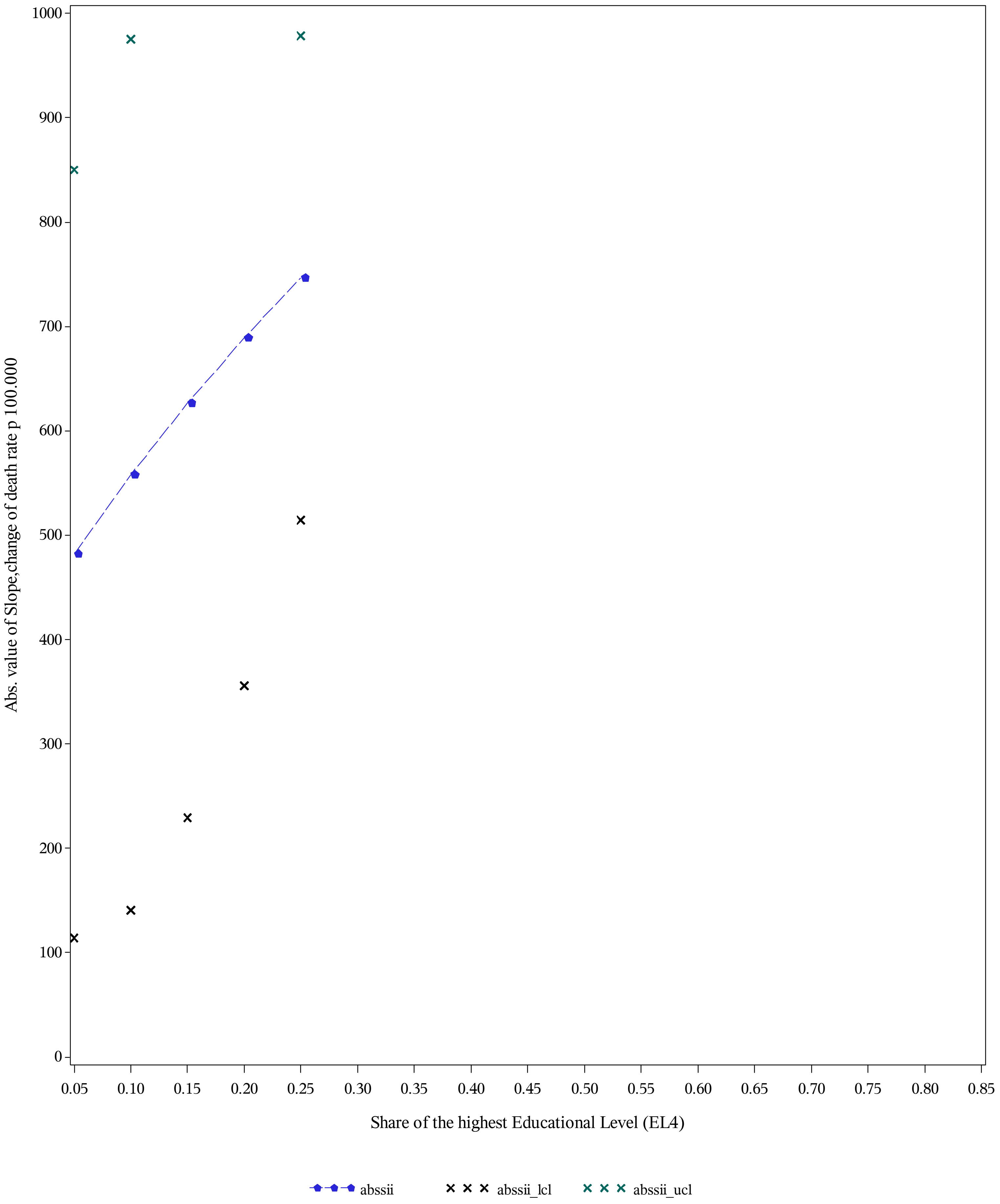

SII in function of the share of EL4

When EL1 and EL3 are fixed at: EL1=65% ; EL3 =10%
EL2 =1- EL4 - EL1 - EL3

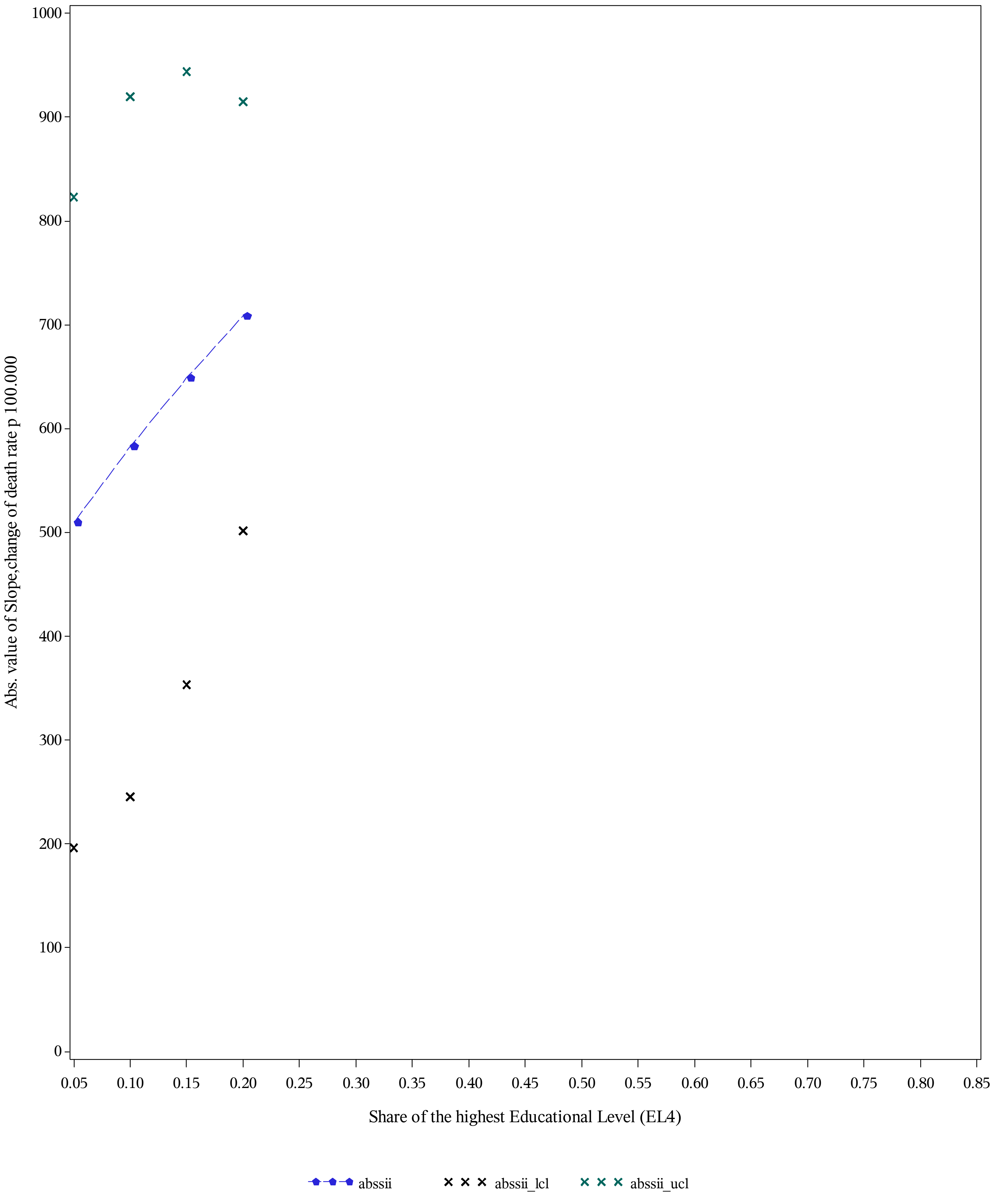

SII in function of the share of EL4

When EL1 and EL3 are fixed at: EL1=65% ; EL3 =15%
EL2 =1- EL4 - EL1 - EL3

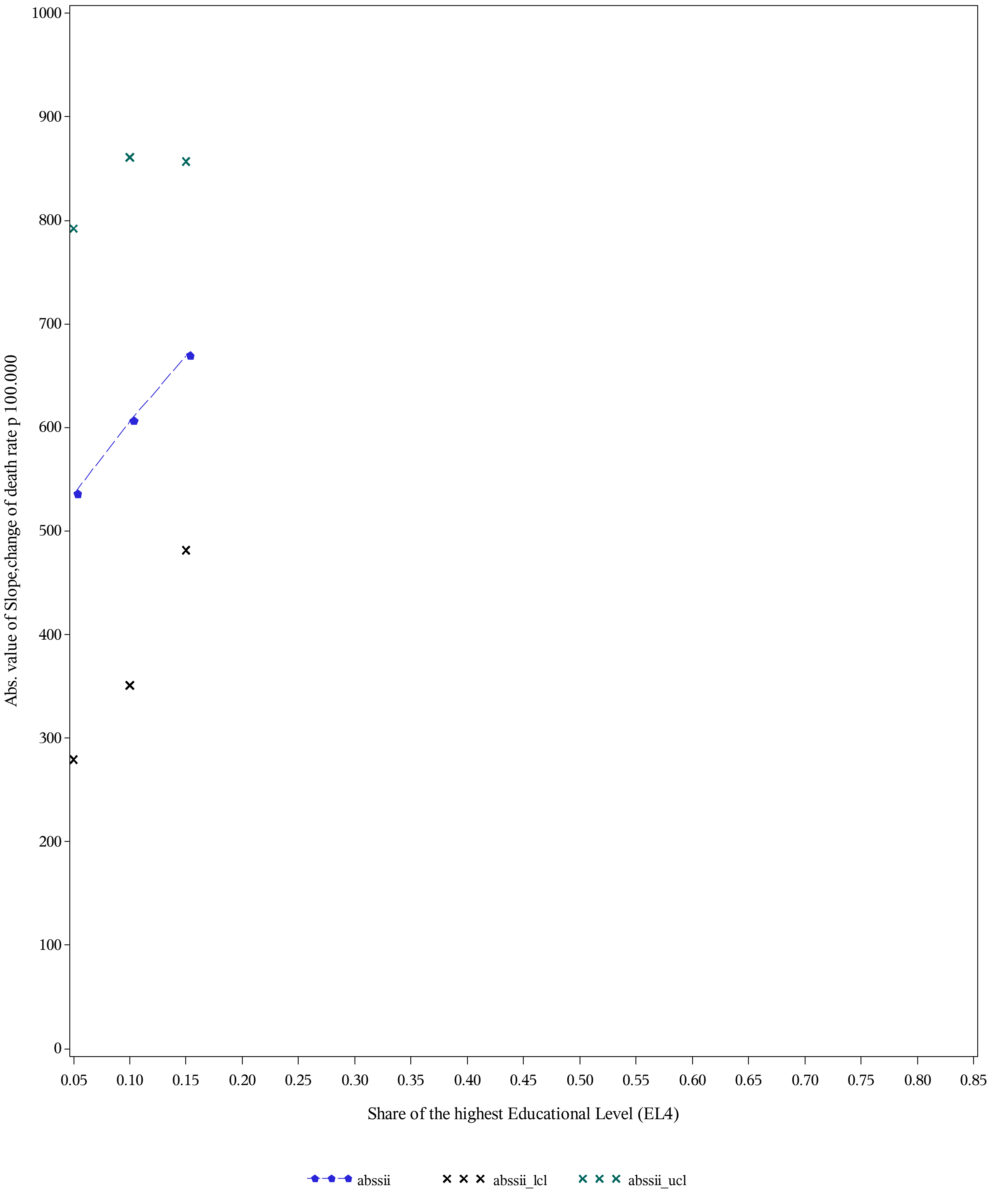

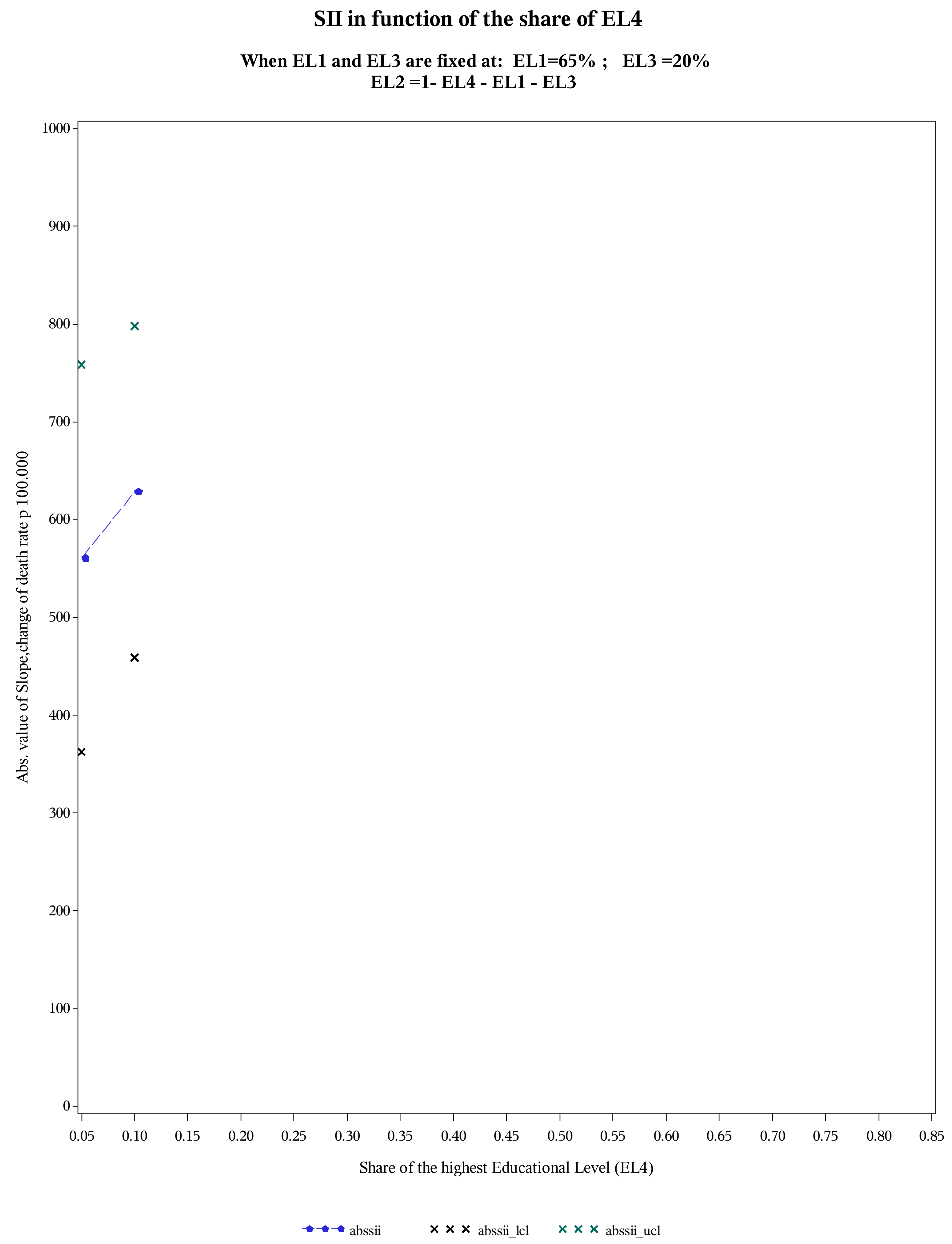
SII in function of the share of EL4
When EL1 and EL3 are fixed at: EL1=65% ; EL3 =20%
EL2 =1- EL4 - EL1 - EL3
Abs. value of Slope,change of death rate p 100.000
0
100
200
300
400
500
600
700
800
900
1000
0.05
0.10
0.15
0.20
0.25
0.30
0.35
0.40
0.45
0.50
0.55
0.60
0.65
0.70
0.75
0.80
0.85
Share of the highest Educational Level (EL4)
abssii
abssii_lcl
abssii_ucl

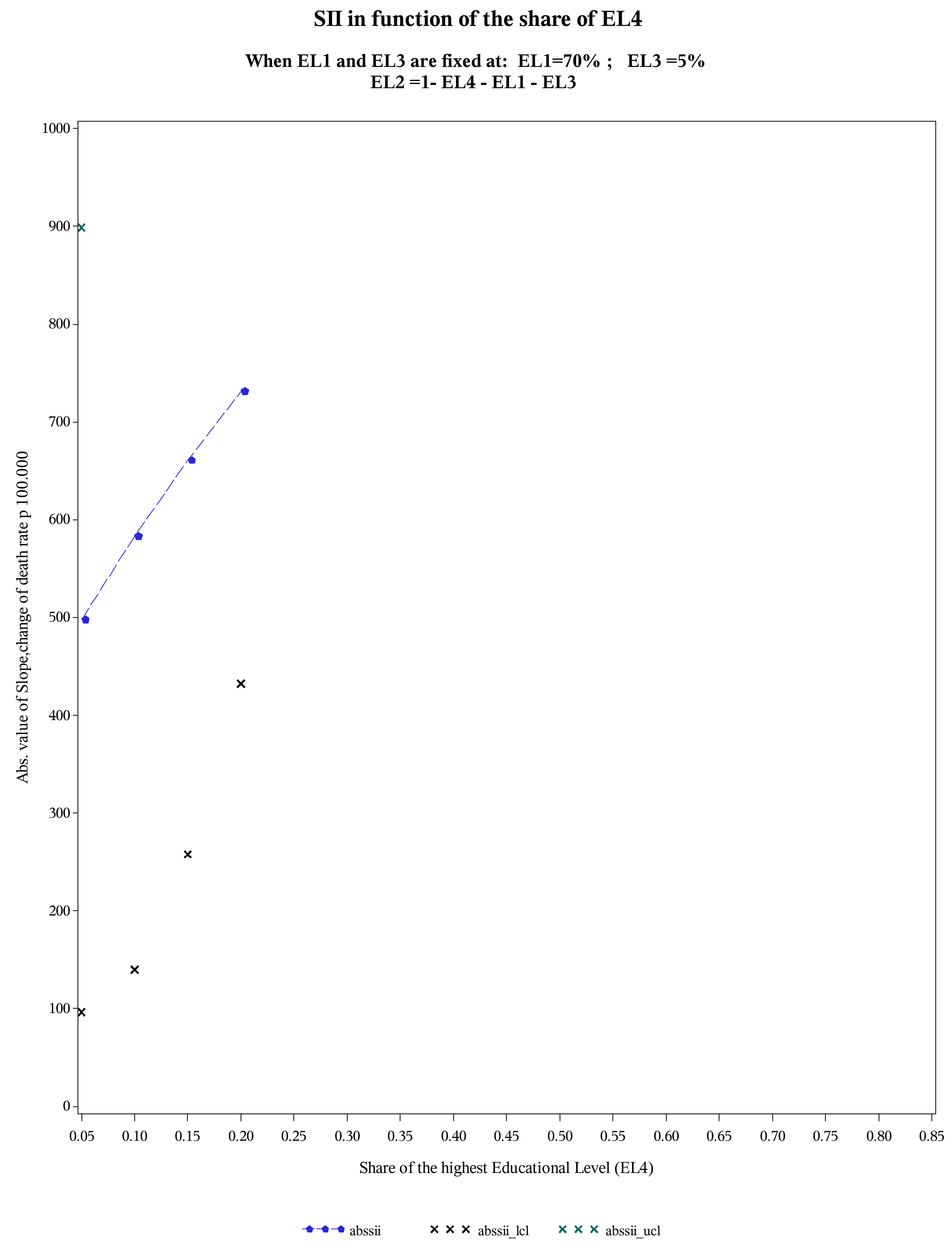

SII in function of the share of EL4
When EL1 and EL3 are fixed at: EL1=70% ; EL3 =5%
EL2 =1- EL4 - EL1 - EL3
Abs. value of Slope,change of death rate p 100.000
0
100
200
300
400
500
600
700
800
900
1000
0.05
0.10
0.15
0.20
0.25
0.30
0.35
0.40
0.45
0.50
0.55
0.60
0.65
0.70
0.75
0.80
0.85
Share of the highest Educational Level (EL4)
abssii
abssii_lcl
abssii_ucl

SII in function of the share of EL4

When EL1 and EL3 are fixed at: EL1=70% ; EL3 =10%
EL2 =1- EL4 - EL1 - EL3

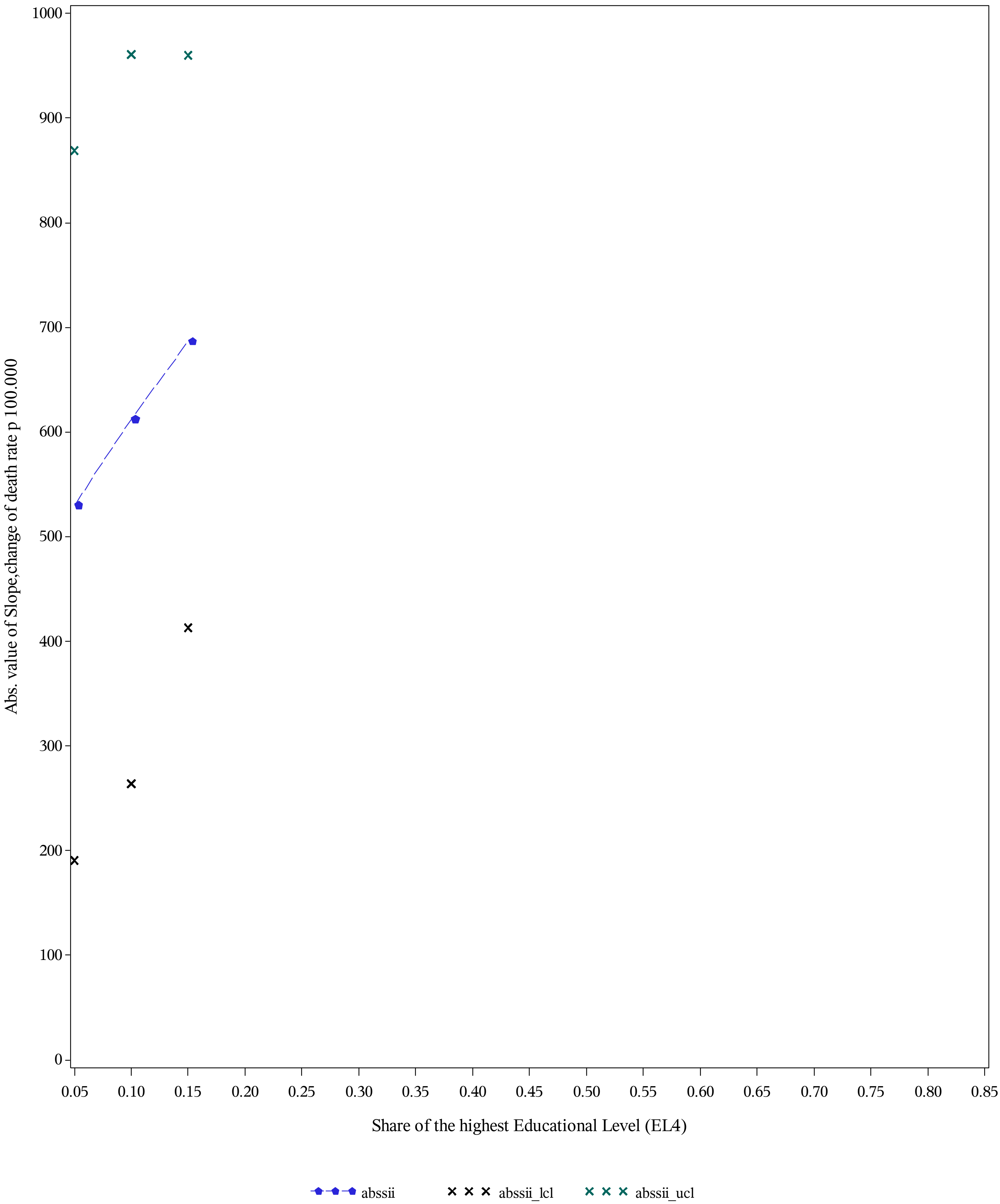

SII in function of the share of EL4

When EL1 and EL3 are fixed at: EL1=70% ; EL3 =15%
EL2 =1- EL4 - EL1 - EL3

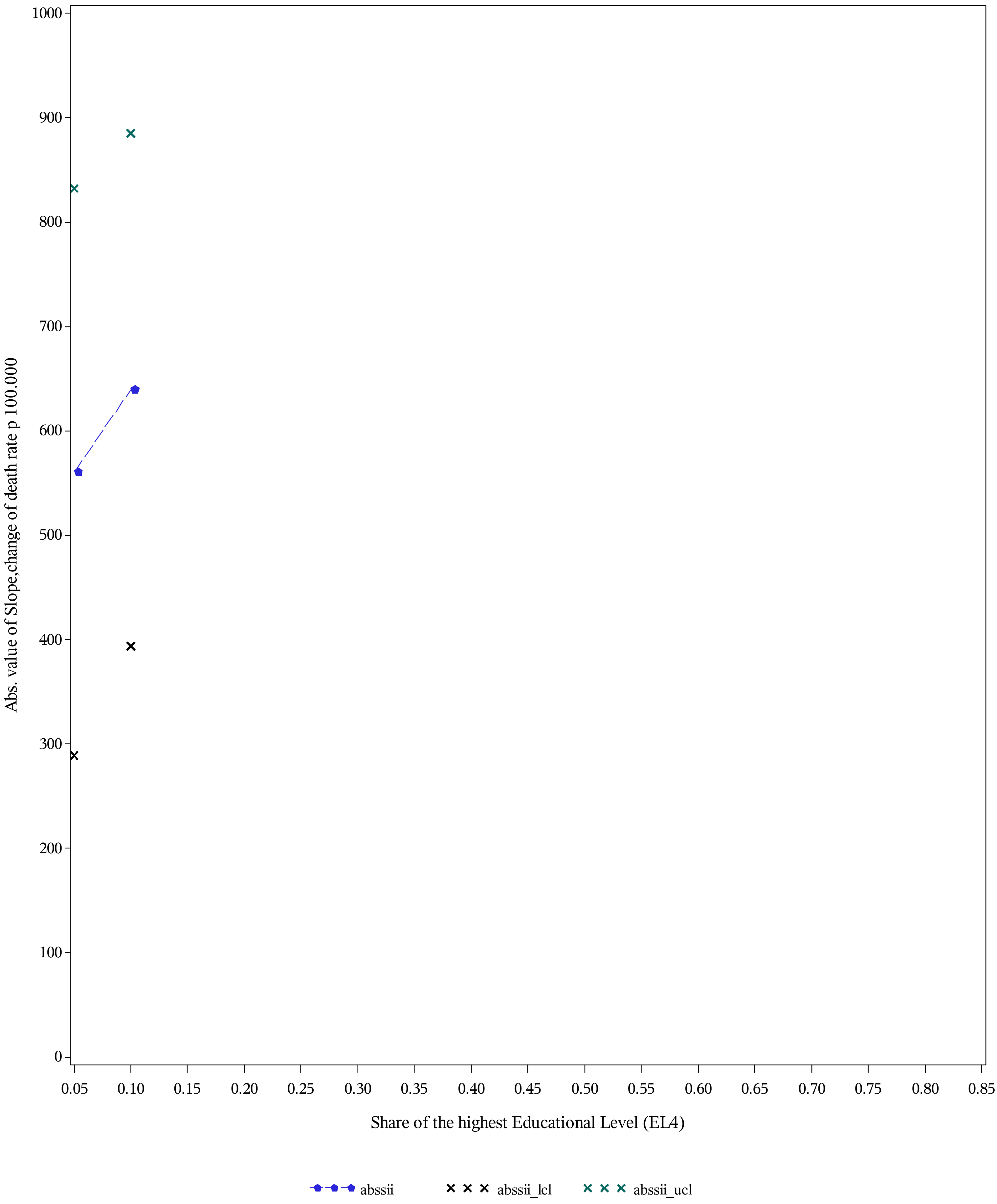

SII in function of the share of EL4

When EL1 and EL3 are fixed at: EL1=75% ; EL3 =5%
EL2 =1- EL4 - EL1 - EL3

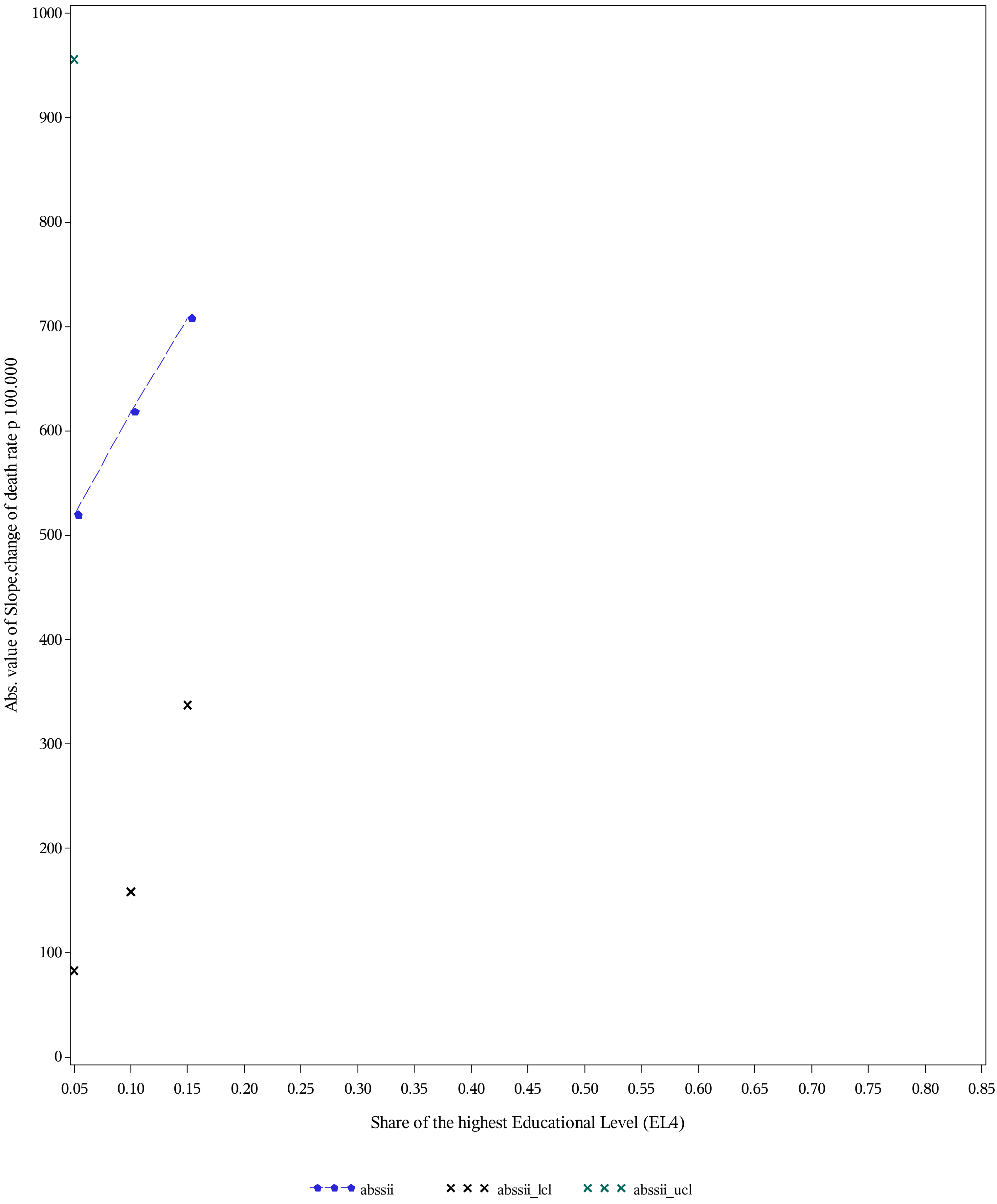

SII in function of the share of EL4

When EL1 and EL3 are fixed at: EL1=75% ; EL3 =10%
EL2 =1- EL4 - EL1 - EL3

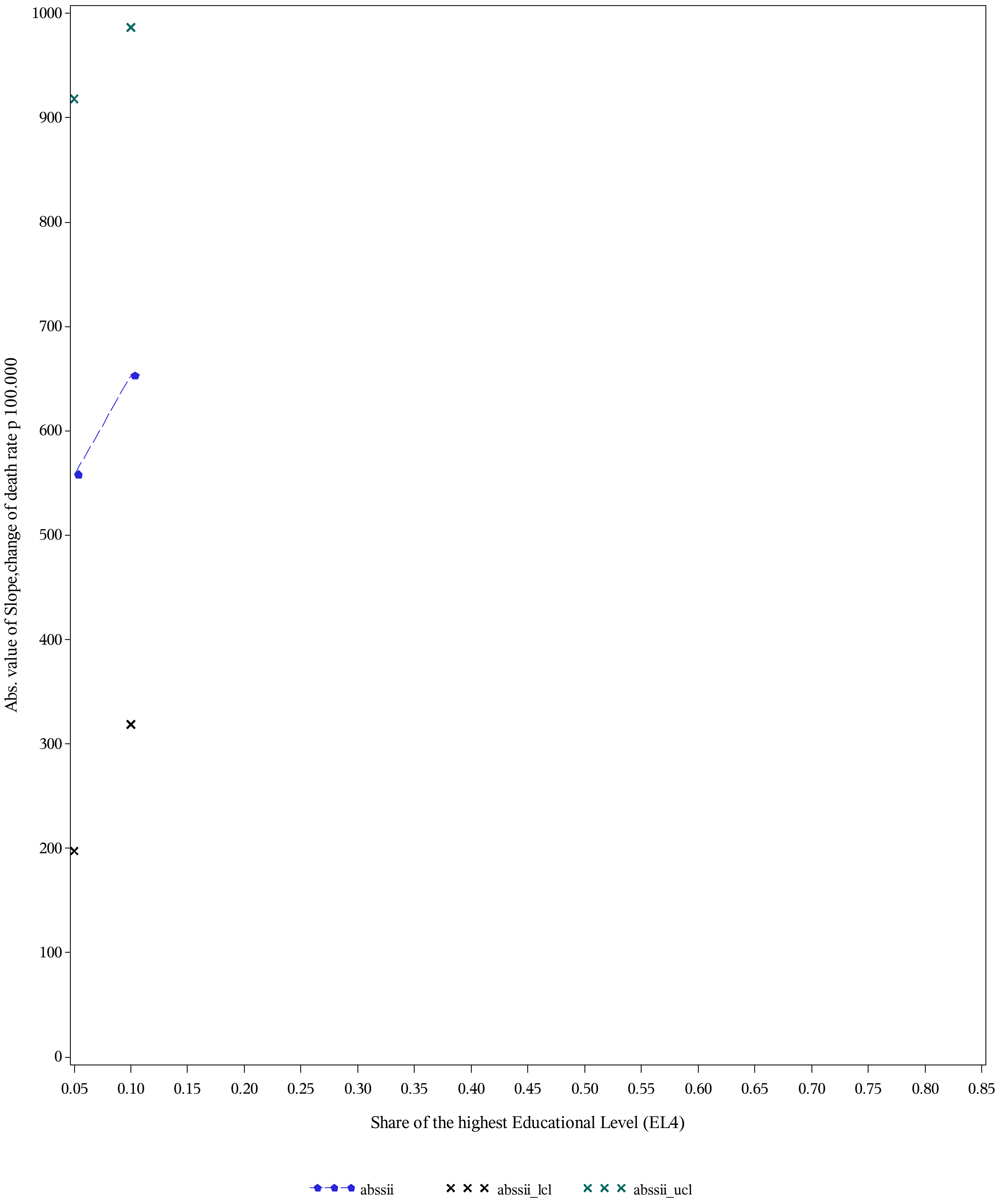

SII in function of the share of EL4

When EL1 and EL3 are fixed at: EL1=80% ; EL3 =5%
EL2 =1- EL4 - EL1 - EL3

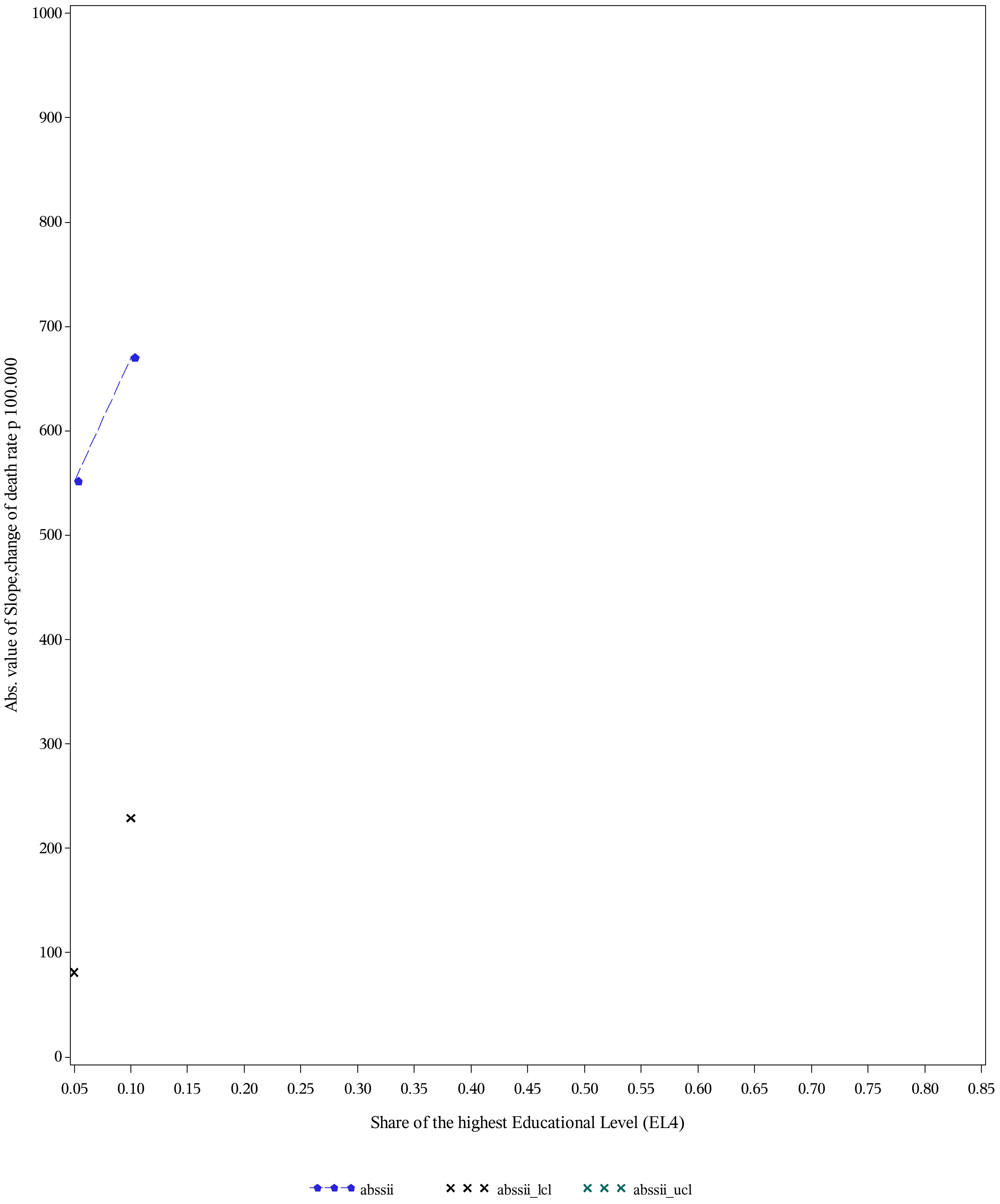

Supplement: Supplementary file 2 — Full set of figures representing the evolution of the SII in function of P4 at fixed p1 and p3 (PDF 616 kb) [file 12889_2019_6980_MOESM2_ESM.pdf]
