# Supplementary material for: Monitoring health inequalities when the socio-economic composition changes: are the slope and relative indices of inequality appropriate? Results of a simulation study
Source: BMC Public Health. 2019 May 30;19:662. doi: 10.1186/s12889-019-6980-1 (PMC6543610; doi:10.1186/s12889-019-6980-1)

PAF in function of the share of EL4

When EL1 and EL2 are fixed at: EL1=5% ; EL2=15%
EL3 =1- EL4 - EL1 - EL2

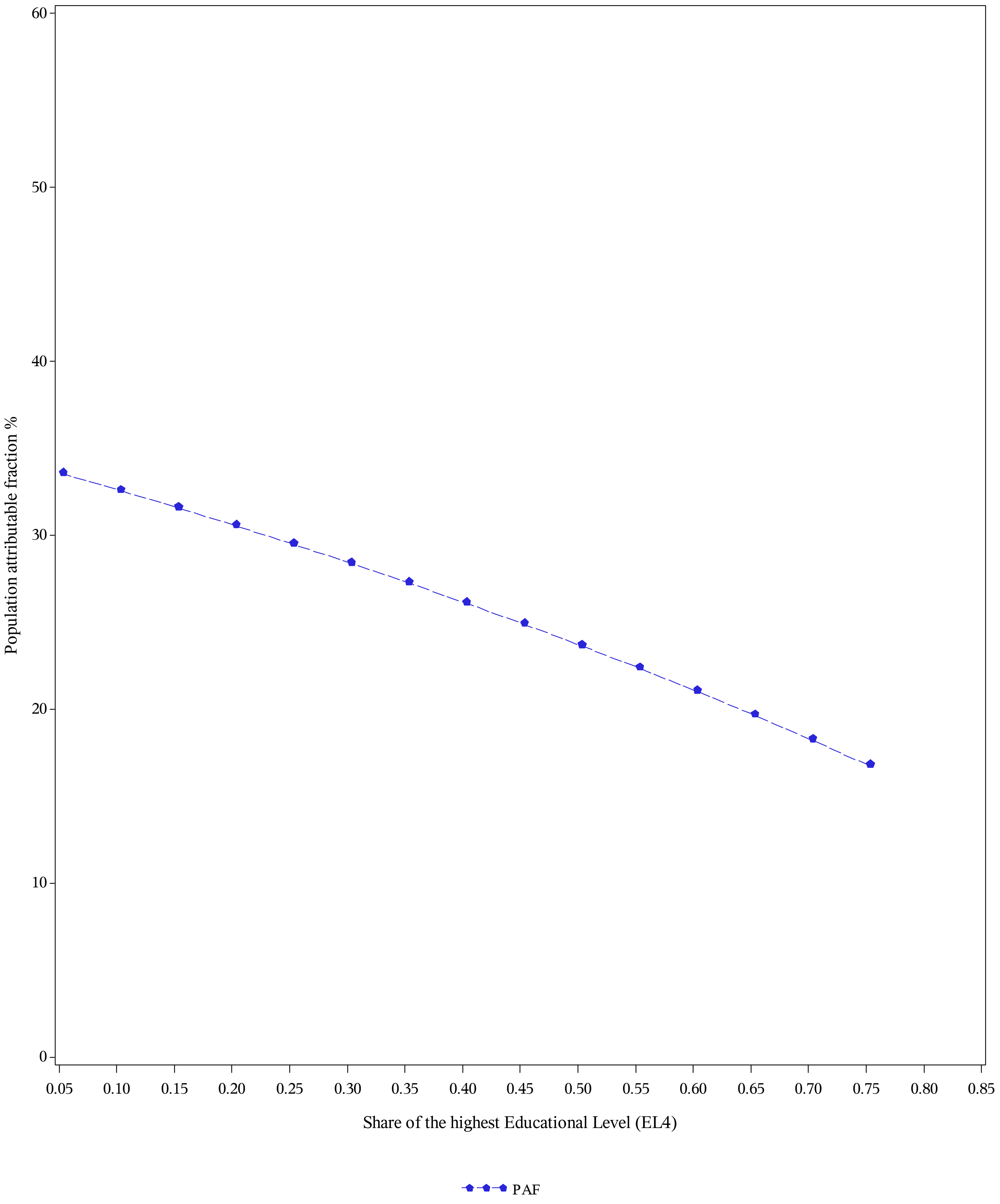

PAF in function of the share of EL4

When EL1 and EL2 are fixed at: EL1=5% ; EL2=20%
EL3 =1- EL4 - EL1 - EL2

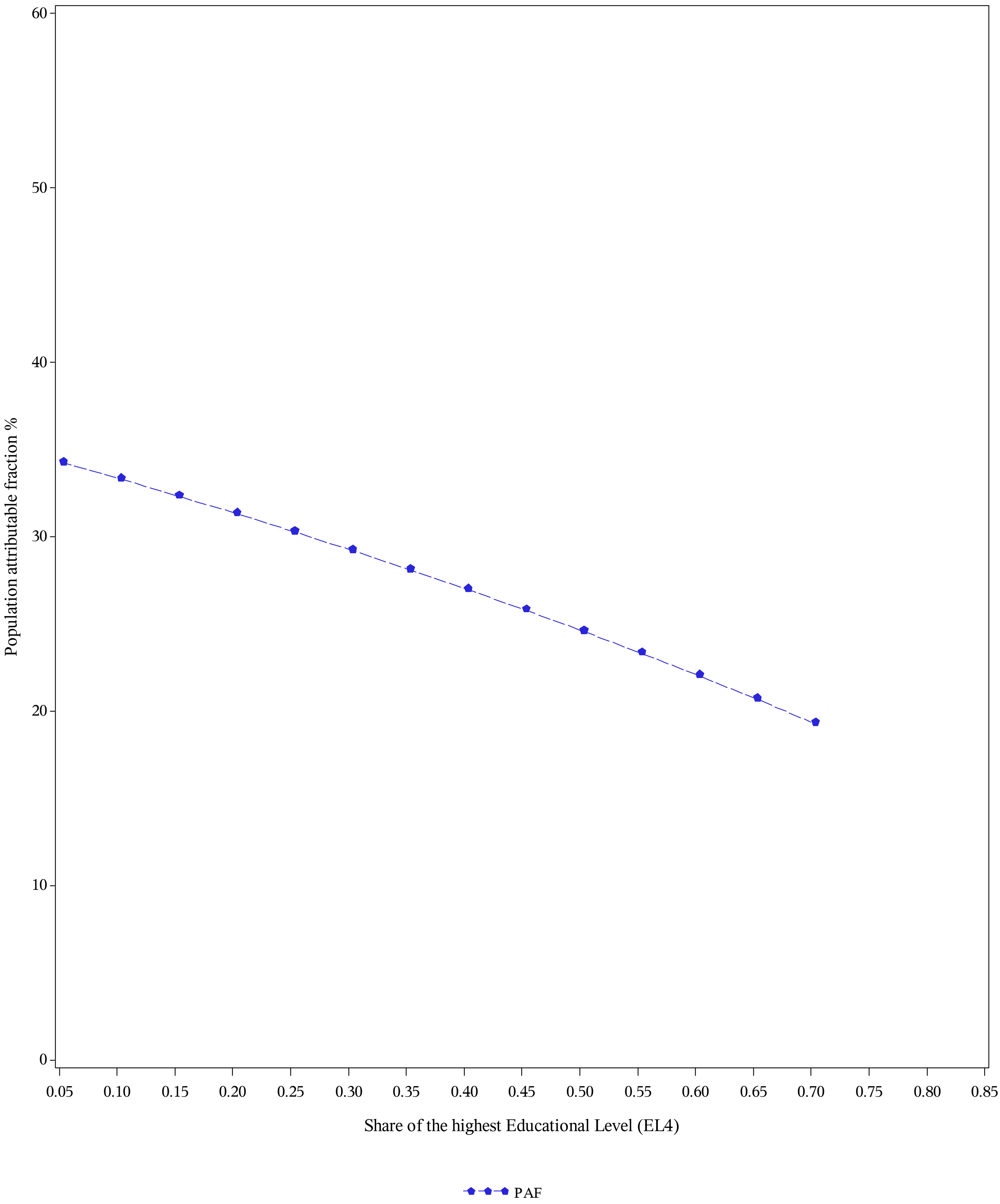

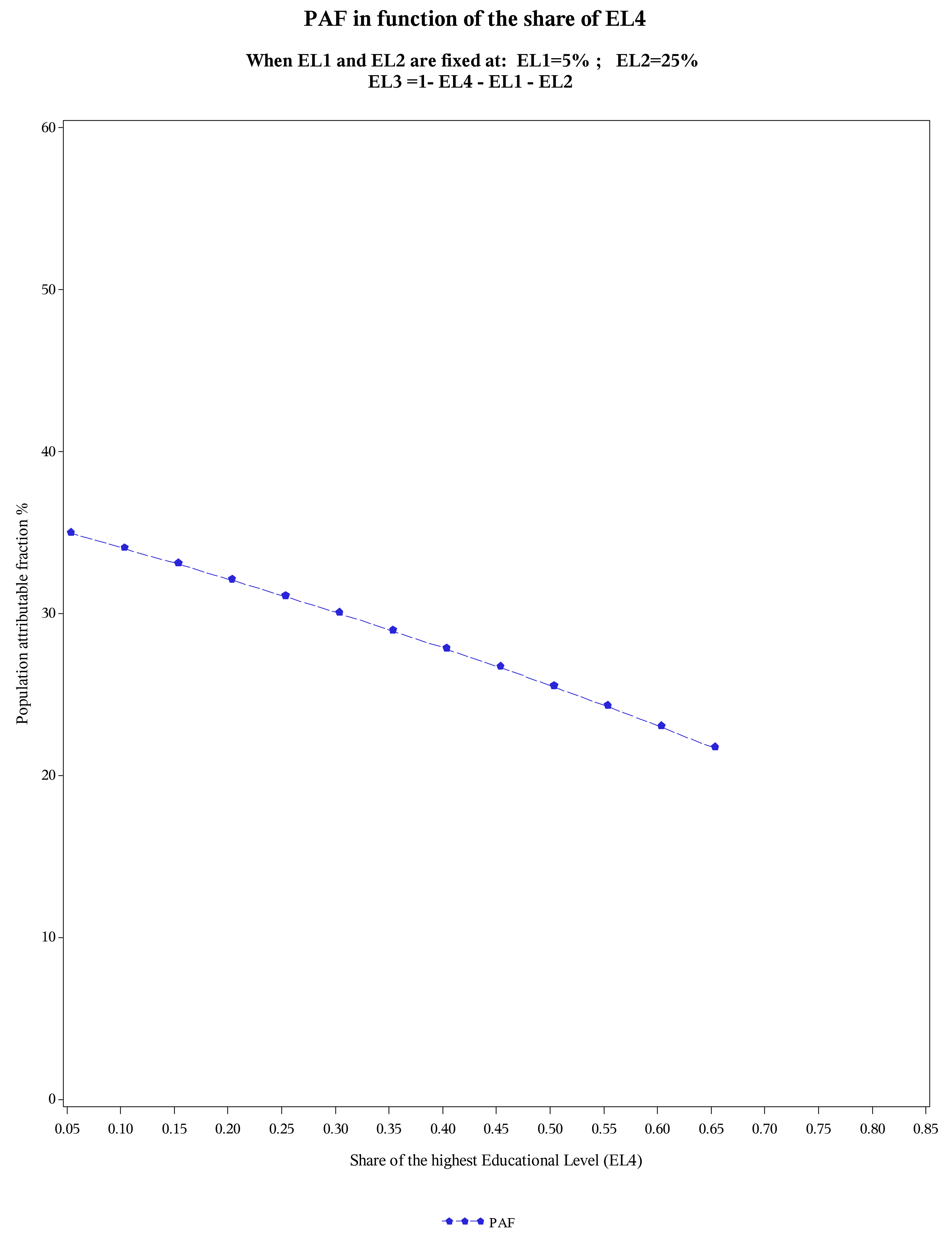
PAF in function of the share of EL4
When EL1 and EL2 are fixed at: EL1=5% ; EL2=25%
EL3 =1- EL4 - EL1 - EL2
Population attributable fraction %
0
10
20
30
40
50
60
0.05
0.10
0.15
0.20
0.25
0.30
0.35
0.40
0.45
0.50
0.55
0.60
0.65
0.70
0.75
0.80
0.85
Share of the highest Educational Level (EL4)
PAF

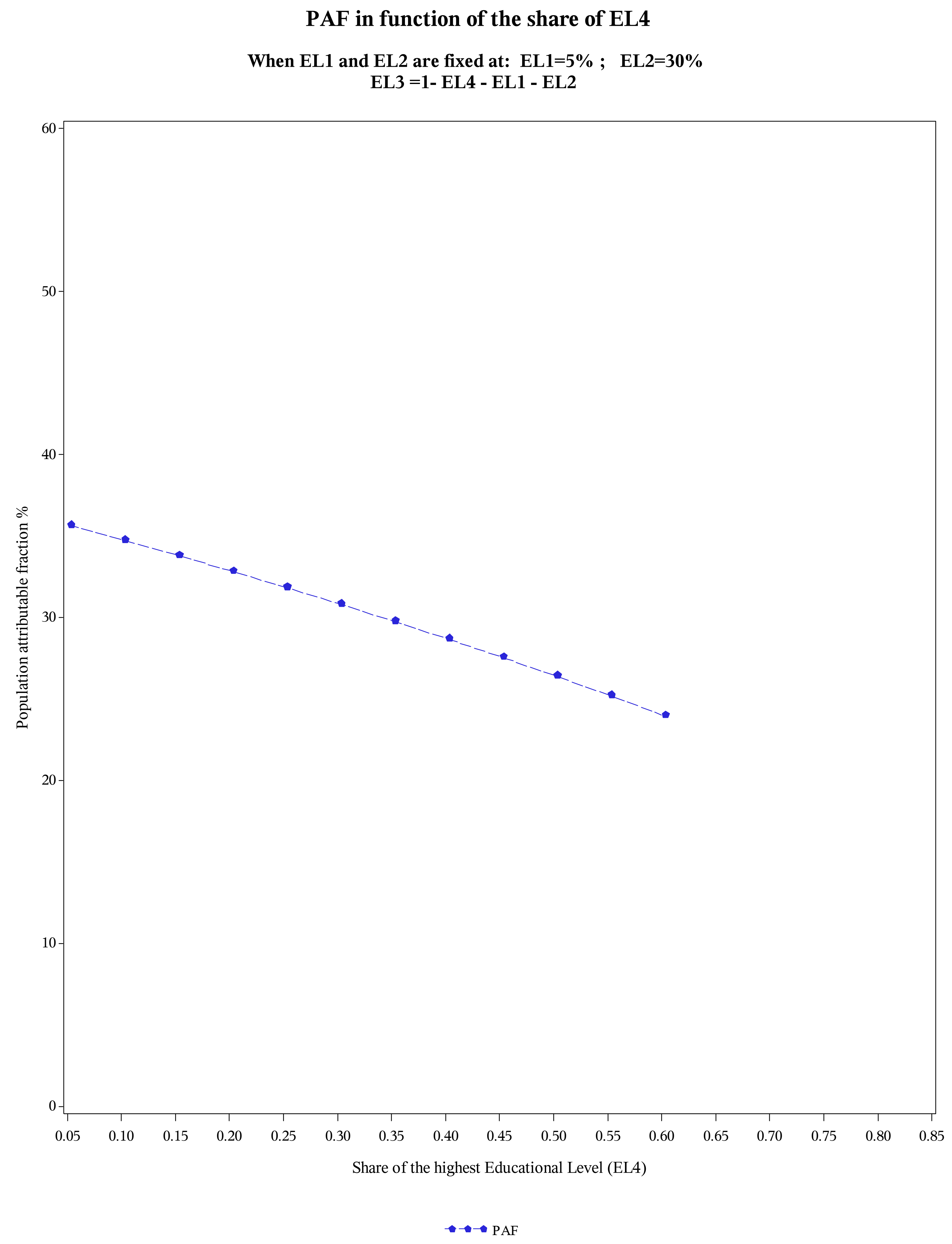

PAF in function of the share of EL4
When EL1 and EL2 are fixed at: EL1=5% ; EL2=30%
EL3 =1- EL4 - EL1 - EL2
Population attributable fraction %
0
10
20
30
40
50
60
0.05
0.10
0.15
0.20
0.25
0.30
0.35
0.40
0.45
0.50
0.55
0.60
0.65
0.70
0.75
0.80
0.85
Share of the highest Educational Level (EL4)
PAF

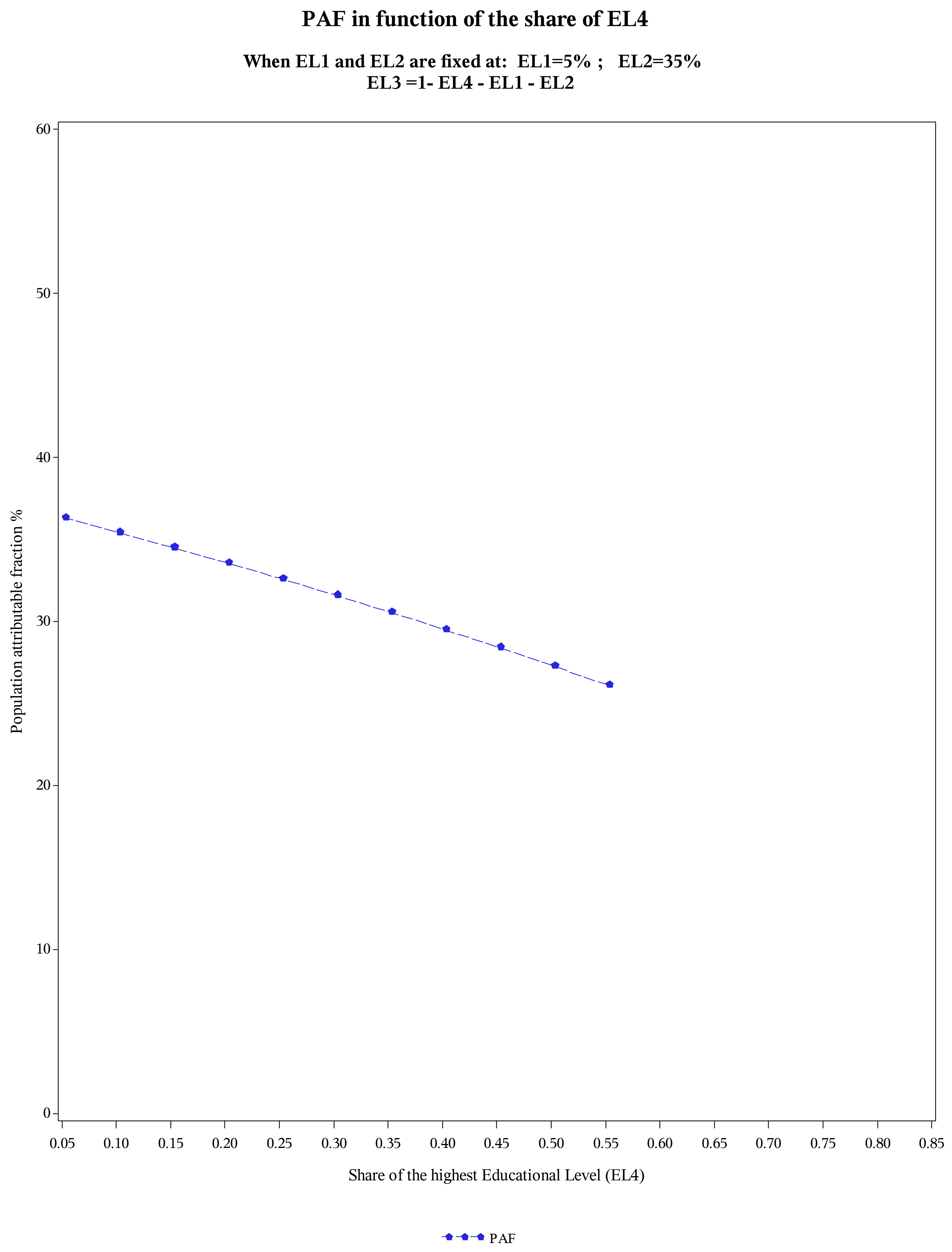
PAF in function of the share of EL4
When EL1 and EL2 are fixed at: EL1=5% ; EL2=35%
EL3 =1- EL4 - EL1 - EL2
Population attributable fraction %
0
10
20
30
40
50
60
0.05
0.10
0.15
0.20
0.25
0.30
0.35
0.40
0.45
0.50
0.55
0.60
0.65
0.70
0.75
0.80
0.85
Share of the highest Educational Level (EL4)
PAF

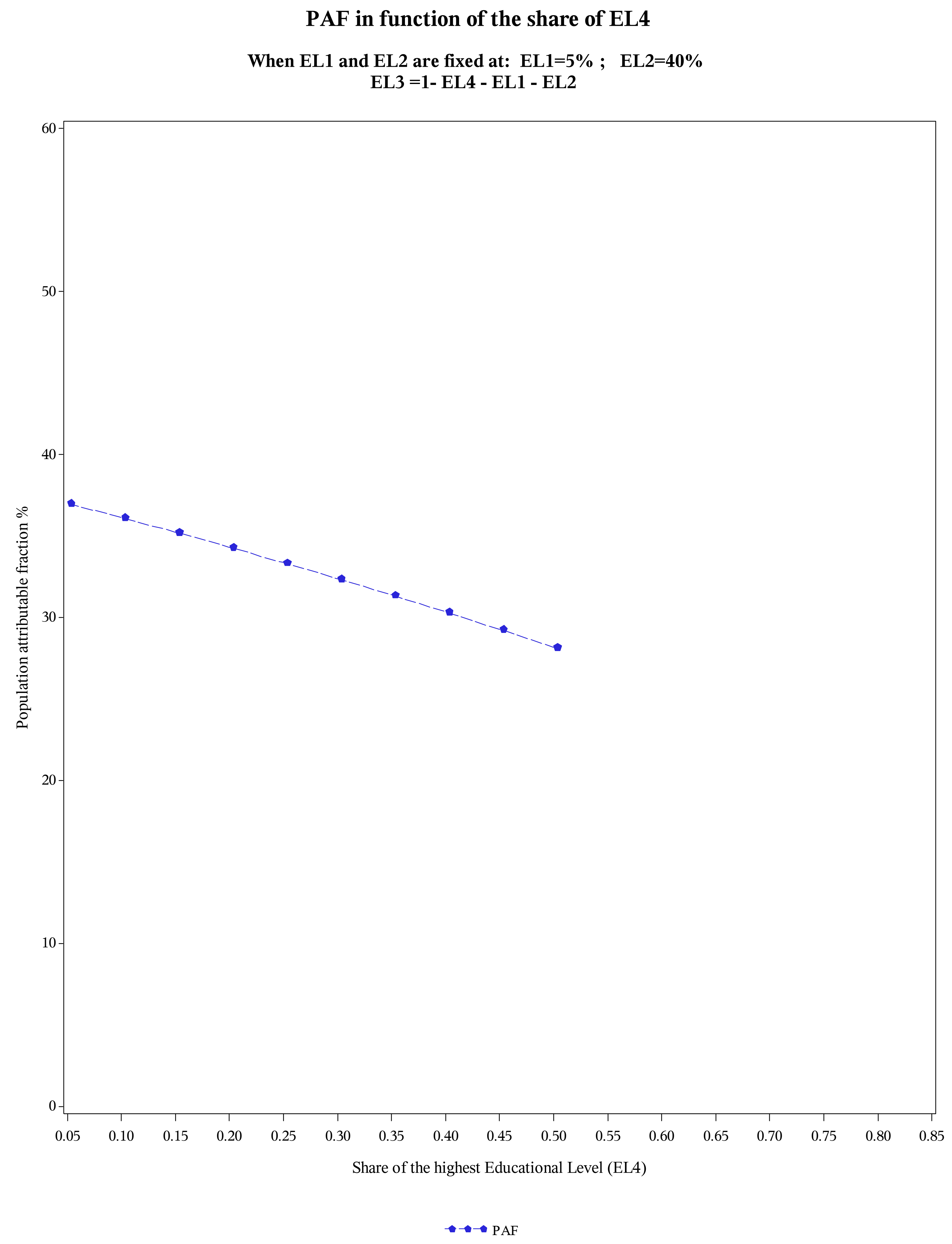

PAF in function of the share of EL4
When EL1 and EL2 are fixed at: EL1=5% ; EL2=40%
EL3 =1- EL4 - EL1 - EL2
Population attributable fraction %
0
10
20
30
40
50
60
0.05
0.10
0.15
0.20
0.25
0.30
0.35
0.40
0.45
0.50
0.55
0.60
0.65
0.70
0.75
0.80
0.85
Share of the highest Educational Level (EL4)
PAF

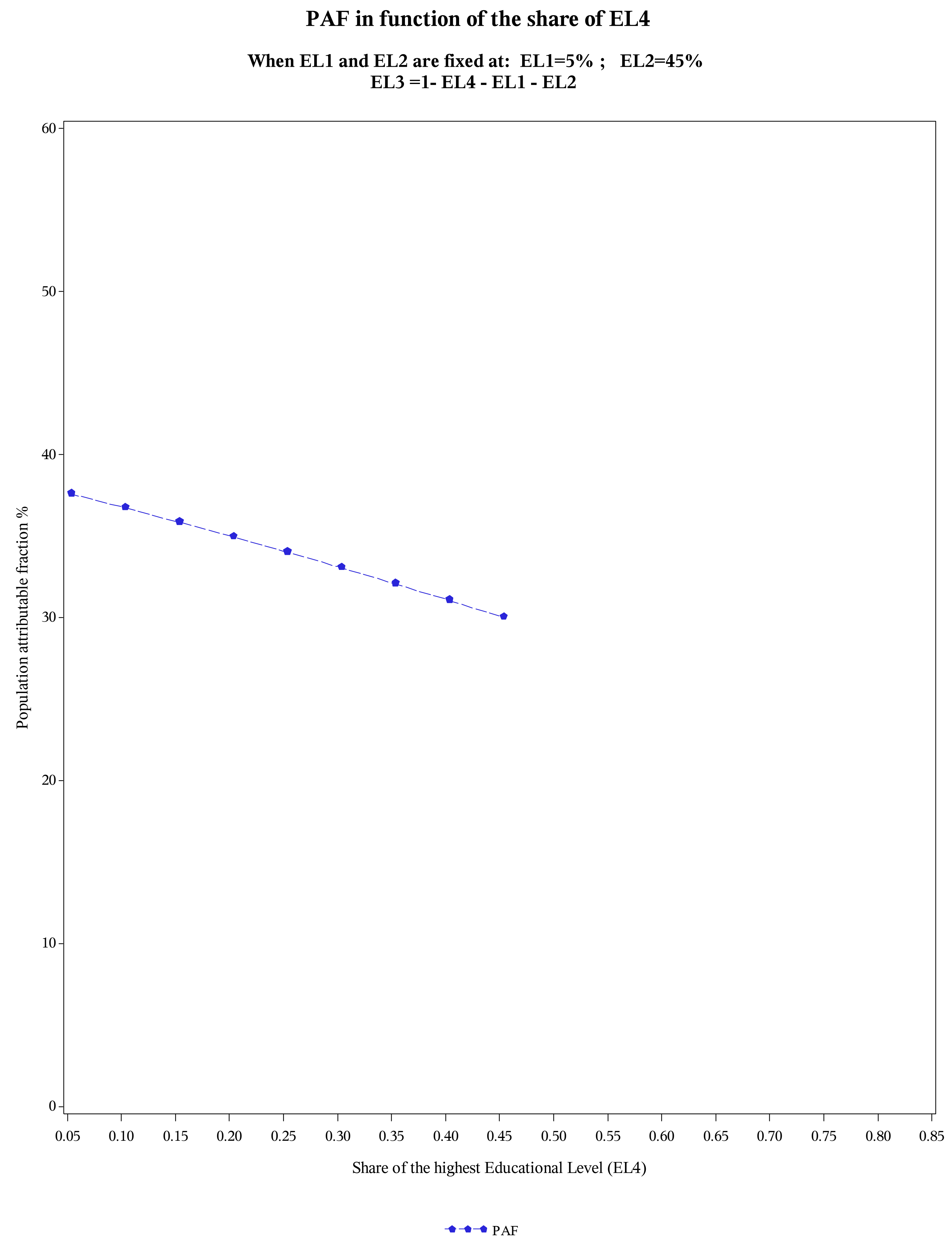

PAF in function of the share of EL4
When EL1 and EL2 are fixed at: EL1=5% ; EL2=45%
EL3 =1- EL4 - EL1 - EL2
Population attributable fraction %
0
10
20
30
40
50
60
0.05
0.10
0.15
0.20
0.25
0.30
0.35
0.40
0.45
0.50
0.55
0.60
0.65
0.70
0.75
0.80
0.85
Share of the highest Educational Level (EL4)
PAF

PAF in function of the share of EL4

When EL1 and EL2 are fixed at: EL1=5% ; EL2=50%
EL3 =1- EL4 - EL1 - EL2

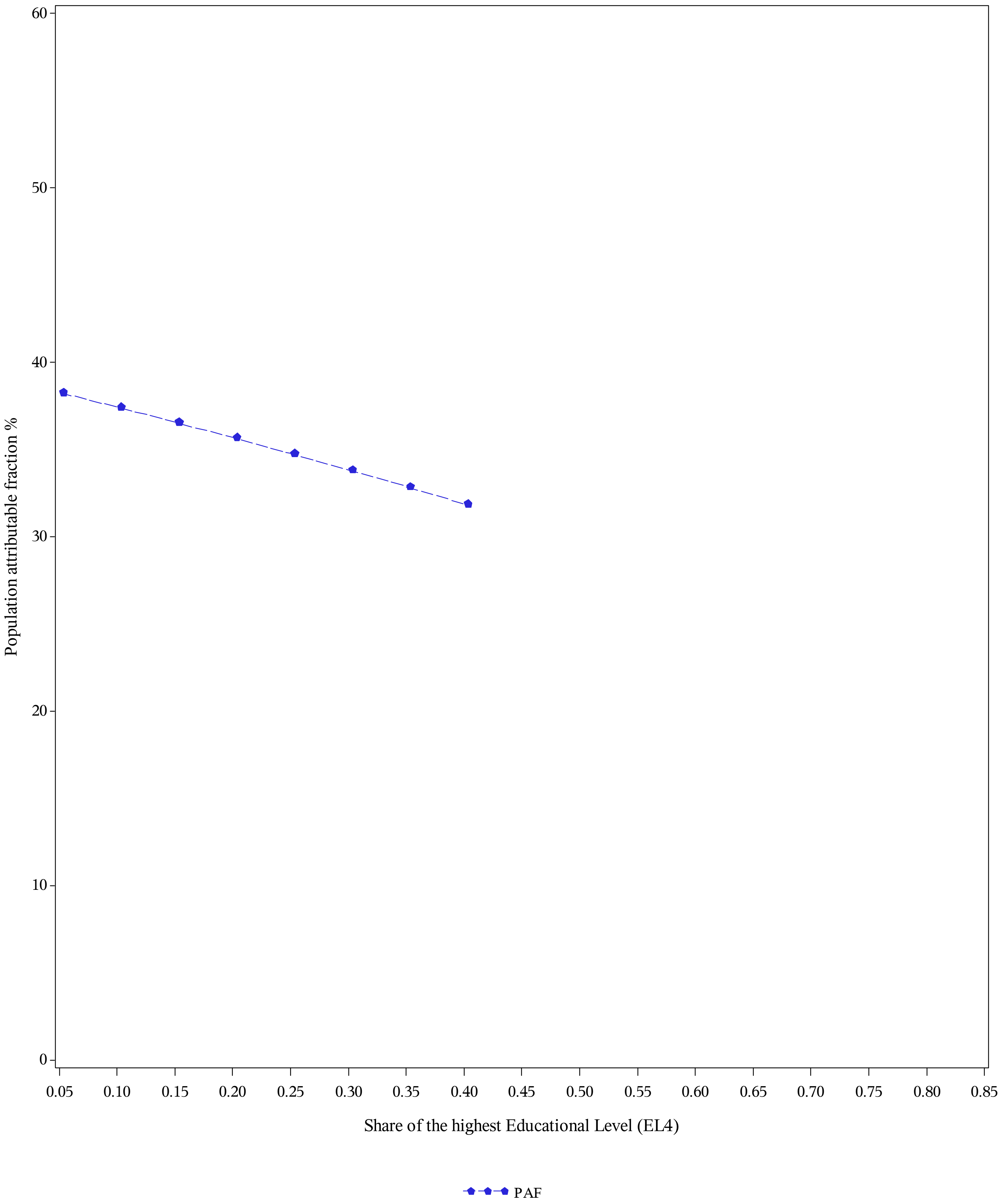

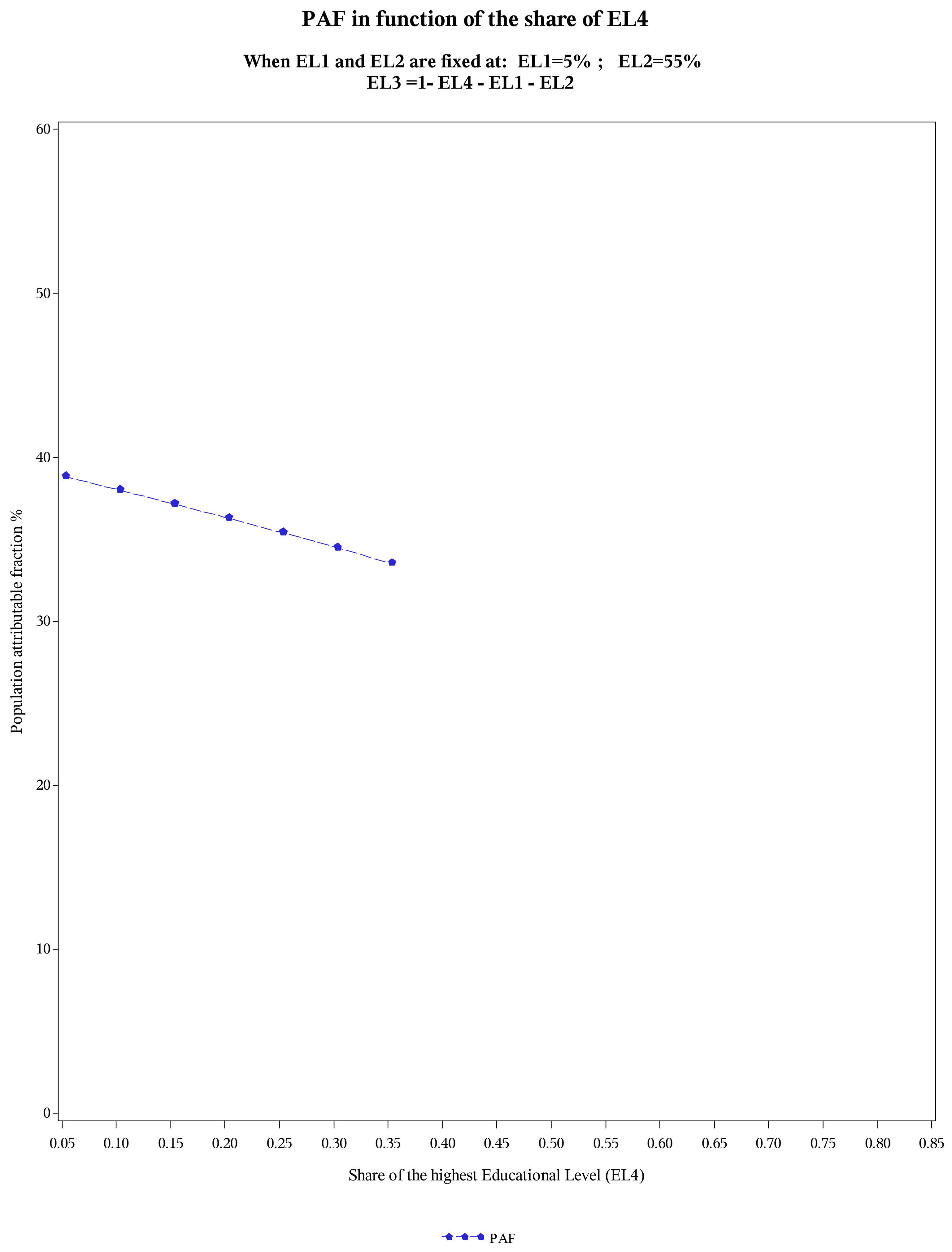

PAF in function of the share of EL4
When EL1 and EL2 are fixed at: EL1=5% ; EL2=55%
EL3 =1- EL4 - EL1 - EL2
Population attributable fraction %
0
10
20
30
40
50
60
0.05
0.10
0.15
0.20
0.25
0.30
0.35
0.40
0.45
0.50
0.55
0.60
0.65
0.70
0.75
0.80
0.85
Share of the highest Educational Level (EL4)
PAF

PAF in function of the share of EL4

When EL1 and EL2 are fixed at: EL1=5% ; EL2=60%
EL3 =1- EL4 - EL1 - EL2

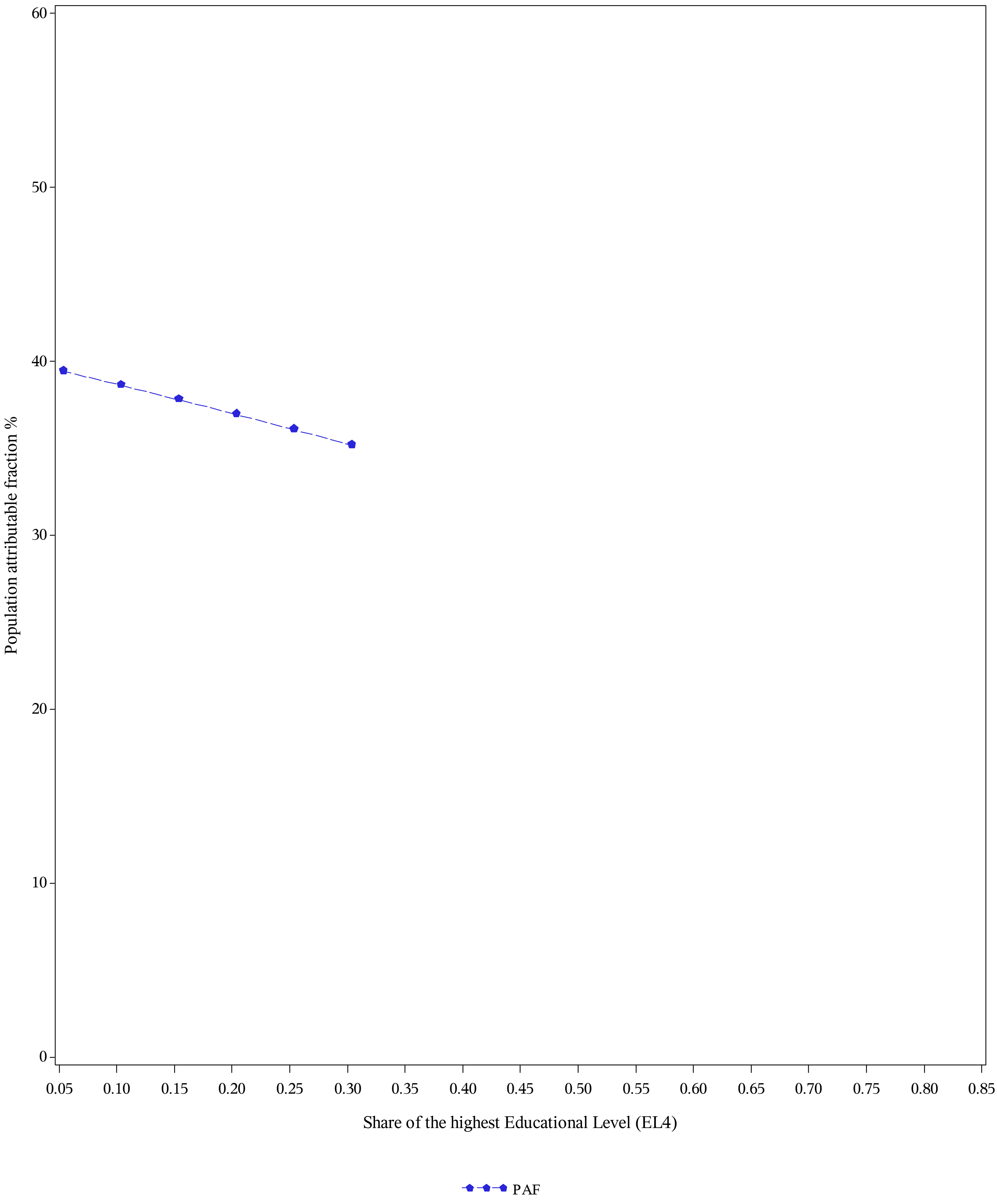

PAF in function of the share of EL4

When EL1 and EL2 are fixed at: EL1=5% ; EL2=65%
EL3 =1- EL4 - EL1 - EL2

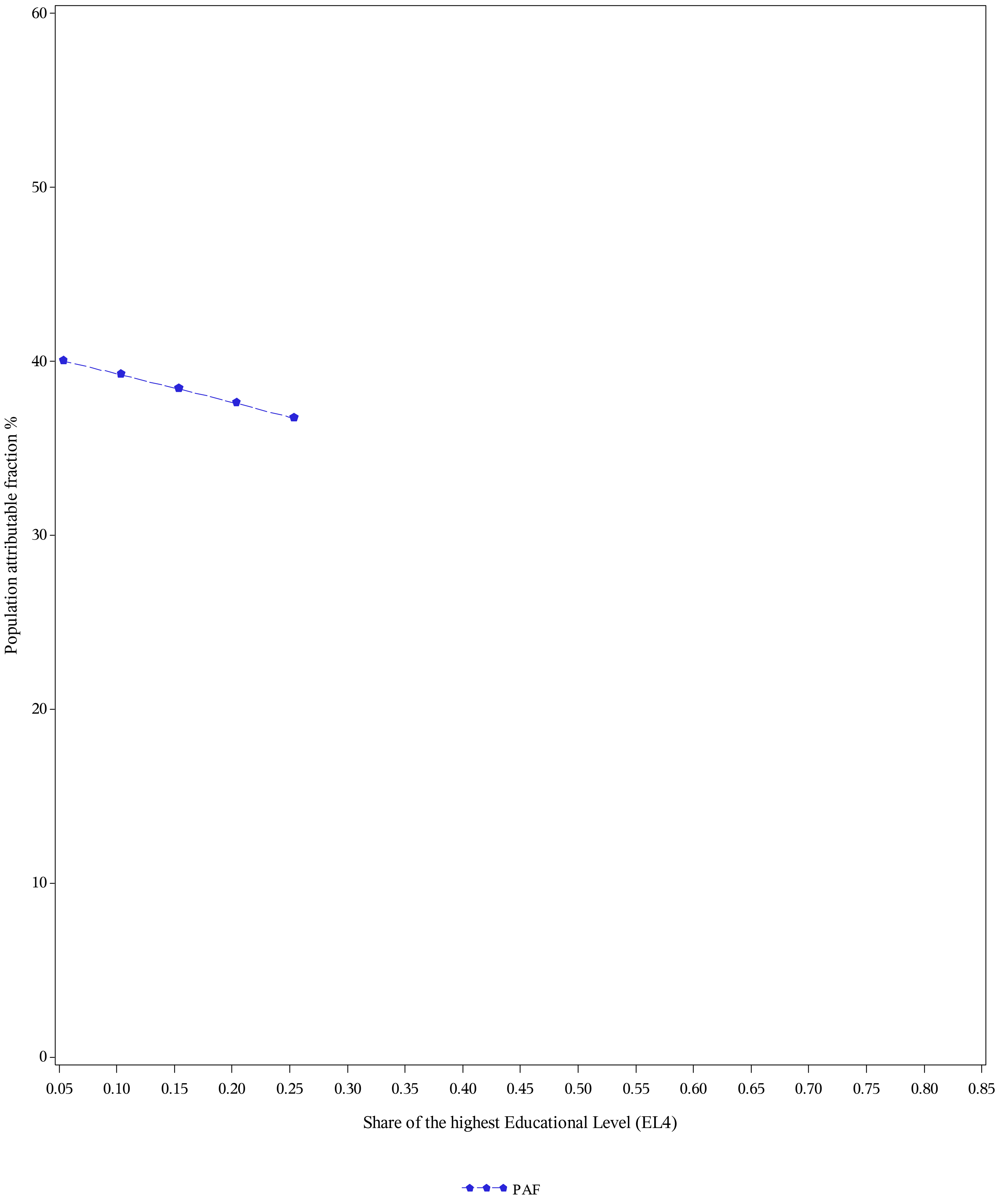

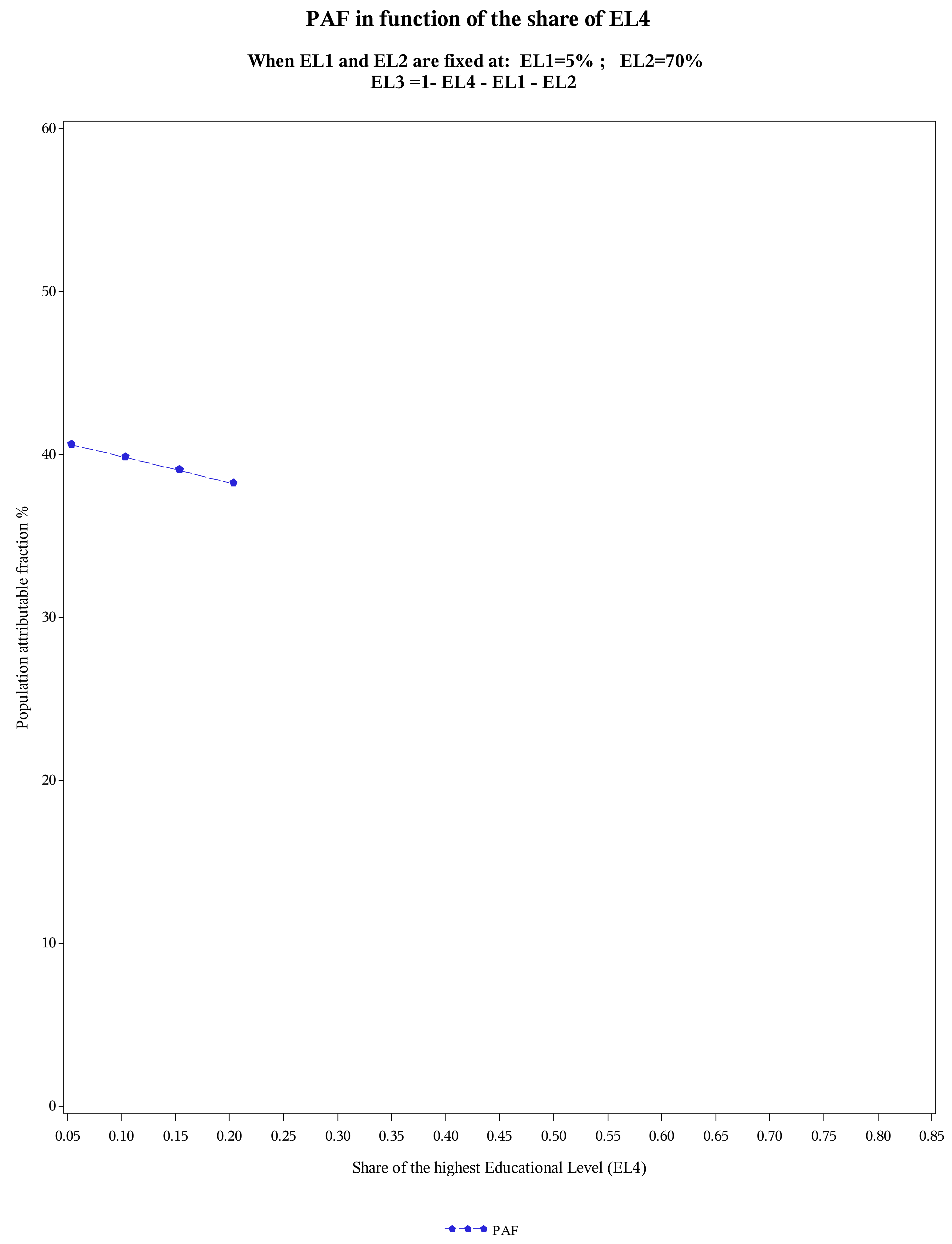
PAF in function of the share of EL4
When EL1 and EL2 are fixed at: EL1=5% ; EL2=70%
EL3 =1- EL4 - EL1 - EL2
Population attributable fraction %
0
10
20
30
40
50
60
0.05
0.10
0.15
0.20
0.25
0.30
0.35
0.40
0.45
0.50
0.55
0.60
0.65
0.70
0.75
0.80
0.85
Share of the highest Educational Level (EL4)
PAF

PAF in function of the share of EL4

When EL1 and EL2 are fixed at: EL1=5% ; EL2=75%
EL3 =1- EL4 - EL1 - EL2

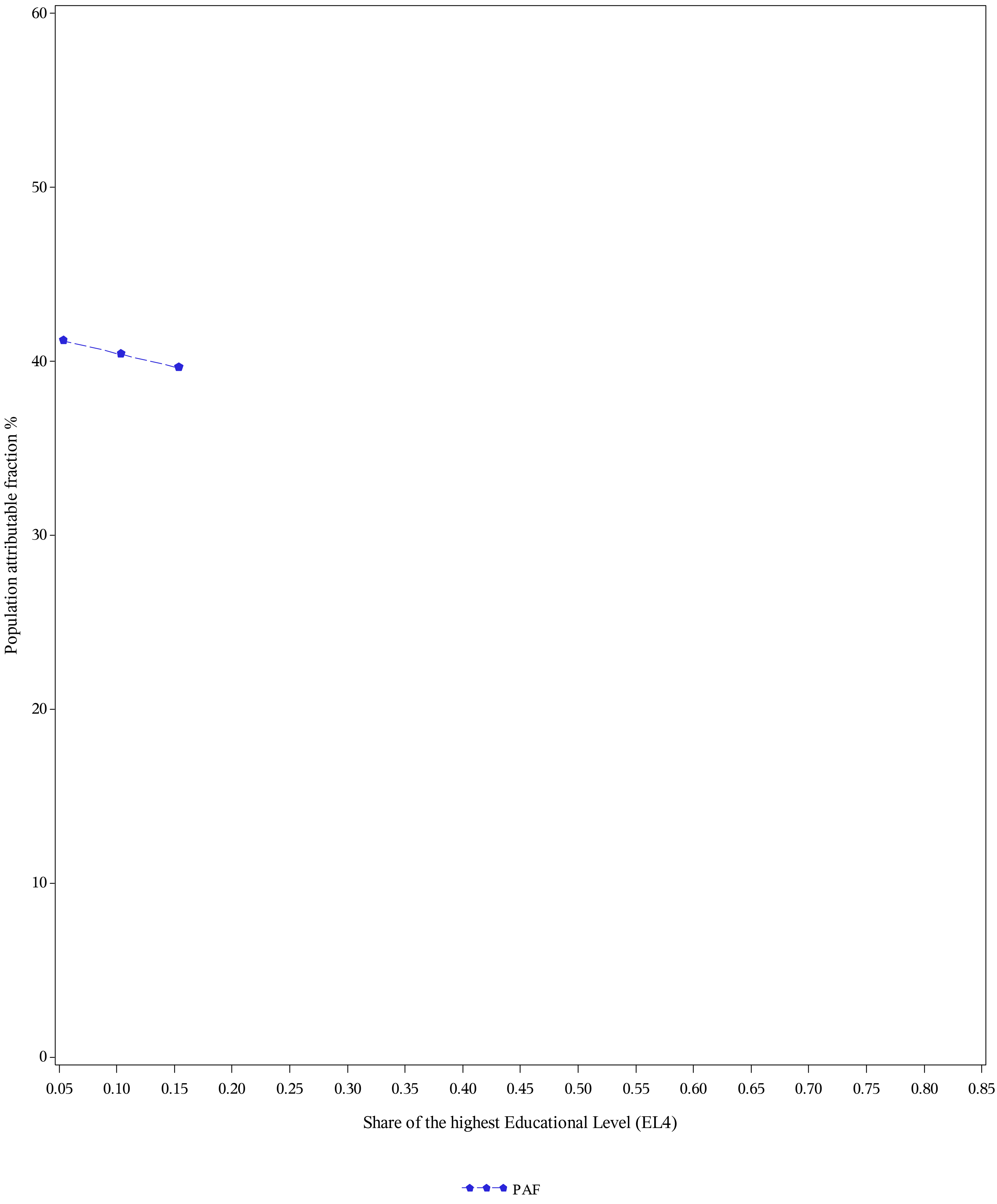

PAF in function of the share of EL4

When EL1 and EL2 are fixed at: EL1=5% ; EL2=80%
EL3 =1- EL4 - EL1 - EL2

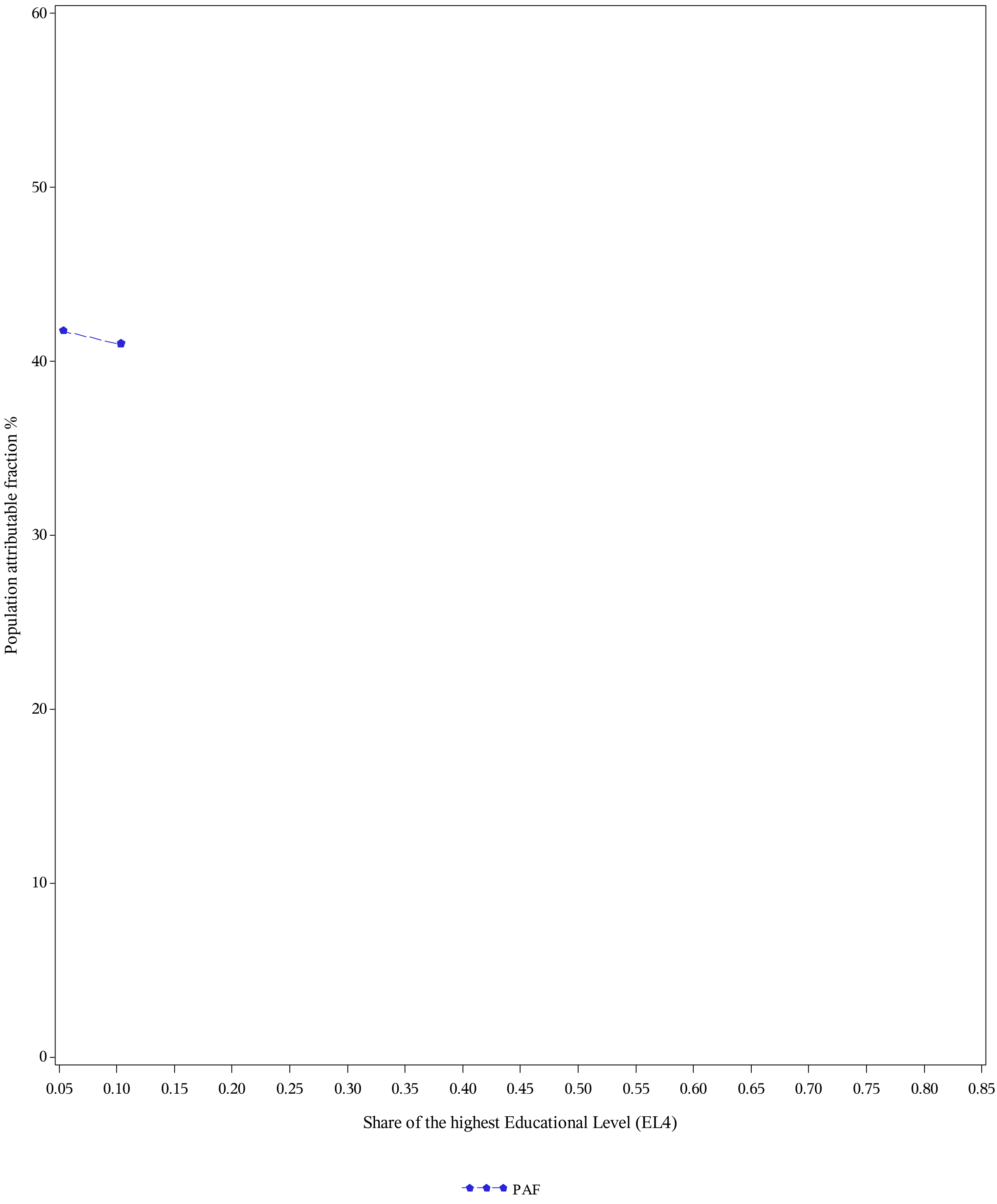

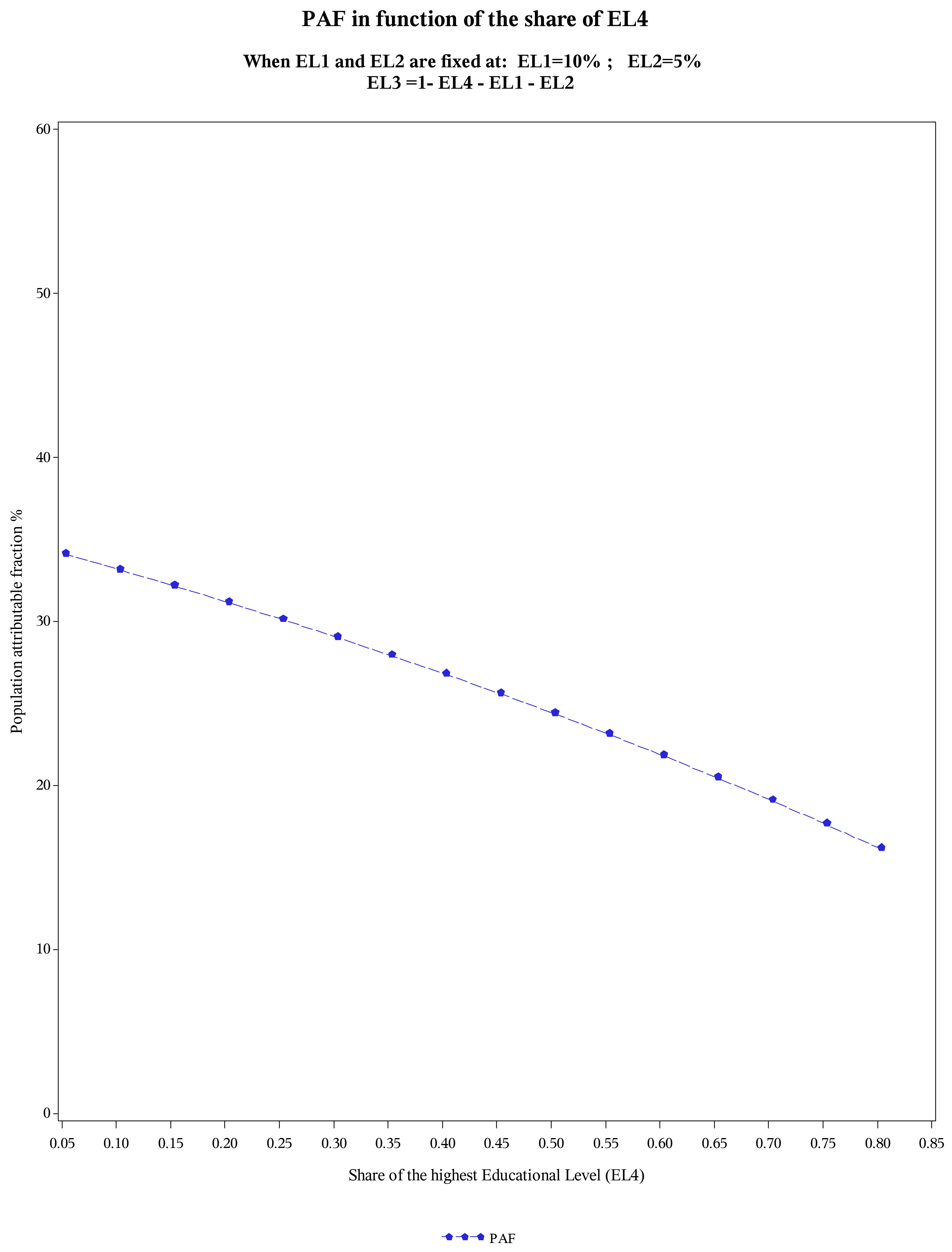
PAF in function of the share of EL4
When EL1 and EL2 are fixed at: EL1=10% ; EL2=5%
EL3 =1- EL4 - EL1 - EL2
Population attributable fraction %
0
10
20
30
40
50
60
0.05
0.10
0.15
0.20
0.25
0.30
0.35
0.40
0.45
0.50
0.55
0.60
0.65
0.70
0.75
0.80
0.85
Share of the highest Educational Level (EL4)
PAF

PAF in function of the share of EL4

When EL1 and EL2 are fixed at: EL1=10% ; EL2=10%
EL3 =1- EL4 - EL1 - EL2

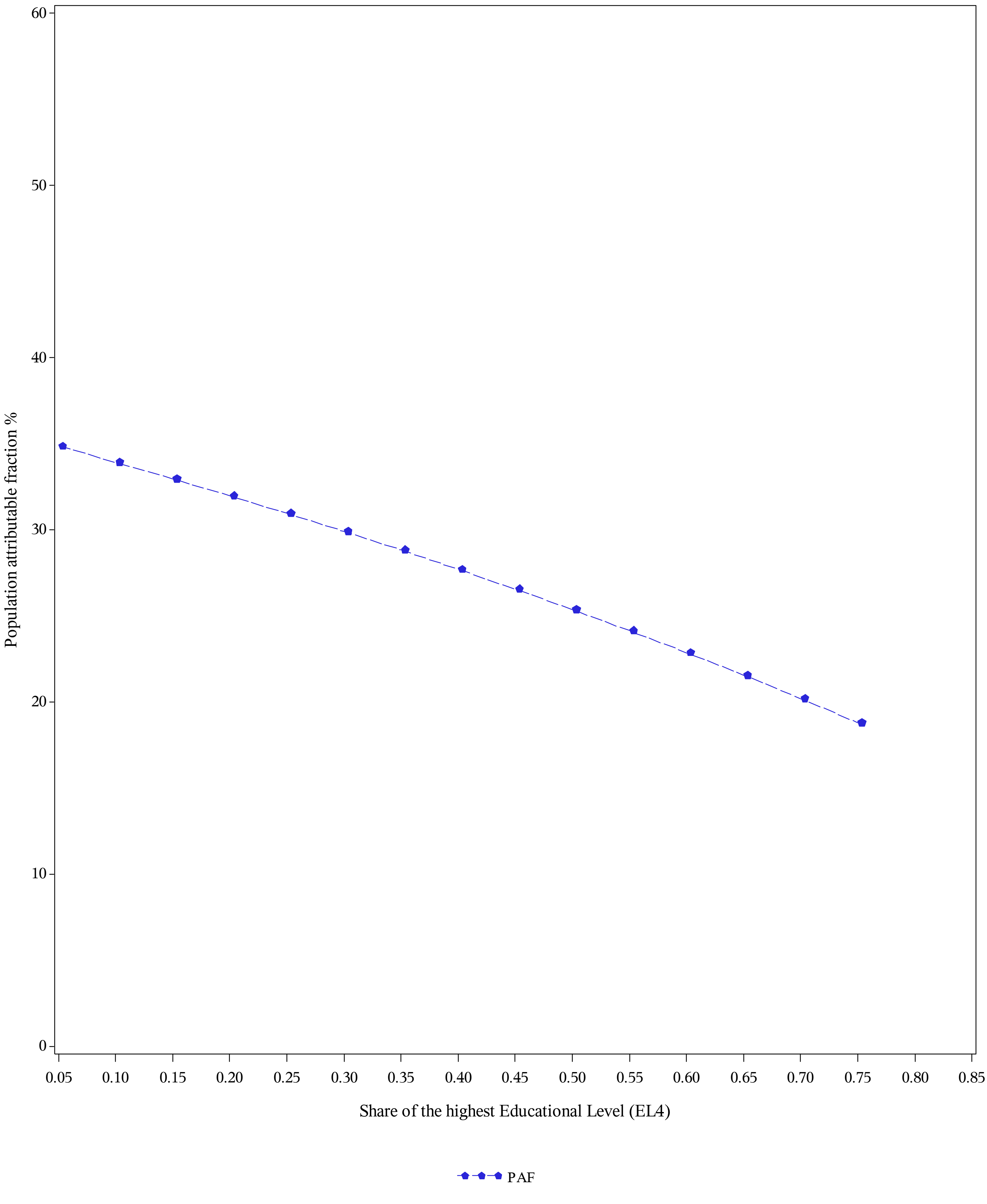

PAF in function of the share of EL4

When EL1 and EL2 are fixed at: EL1=10% ; EL2=15%
EL3 =1- EL4 - EL1 - EL2

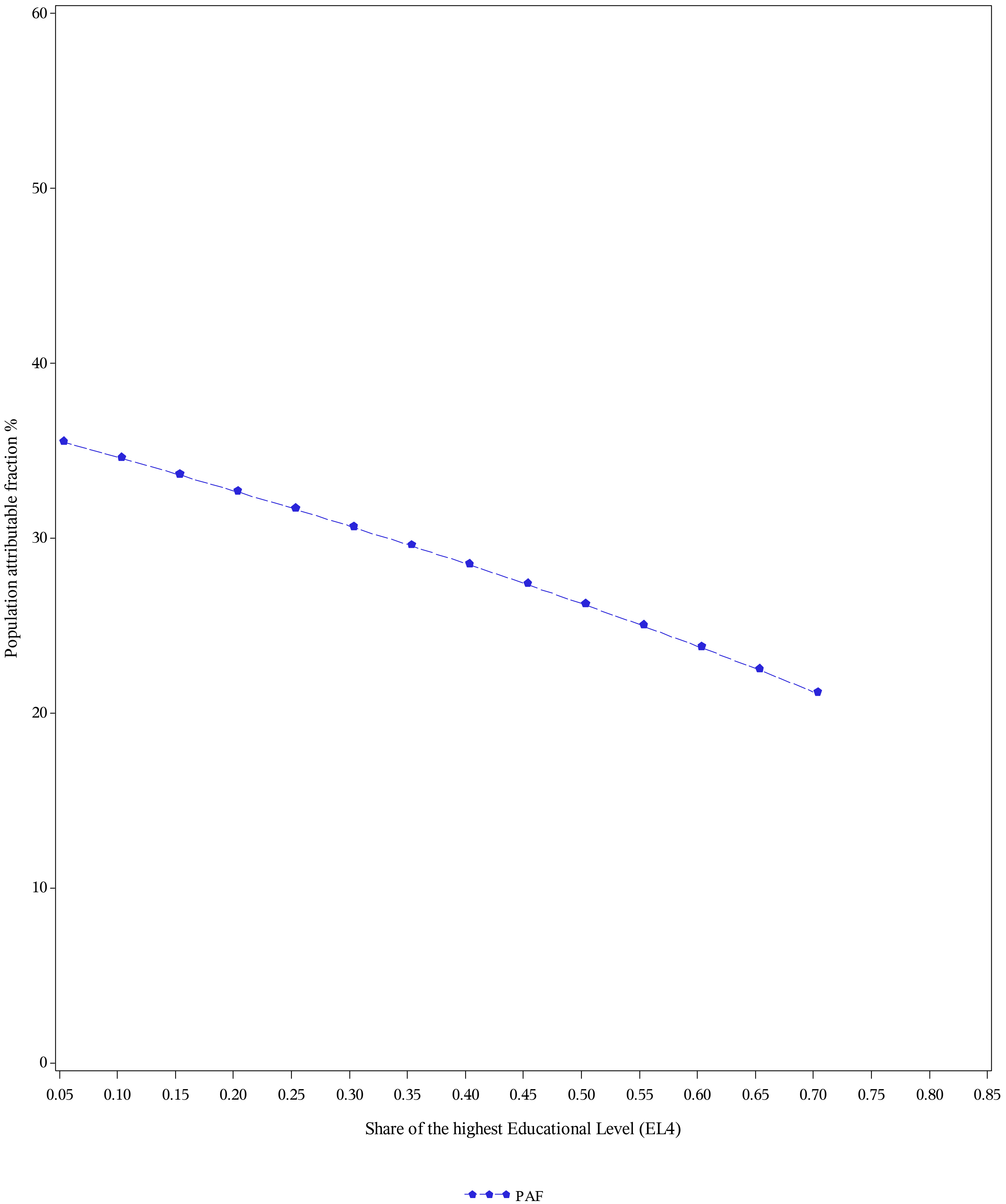

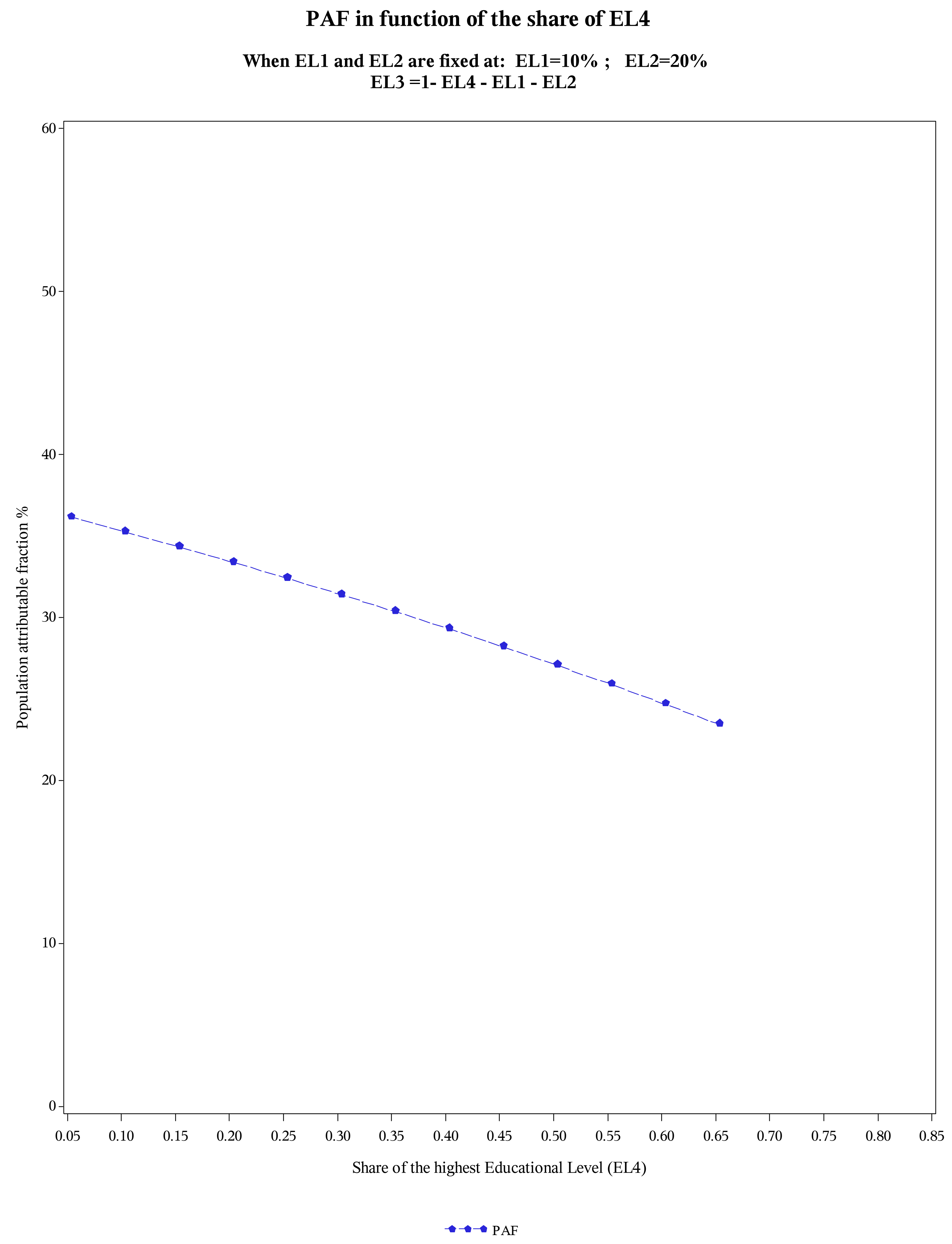
PAF in function of the share of EL4
When EL1 and EL2 are fixed at: EL1=10% ; EL2=20%
EL3 =1- EL4 - EL1 - EL2
Population attributable fraction %
0
10
20
30
40
50
60
0.05
0.10
0.15
0.20
0.25
0.30
0.35
0.40
0.45
0.50
0.55
0.60
0.65
0.70
0.75
0.80
0.85
Share of the highest Educational Level (EL4)
PAF

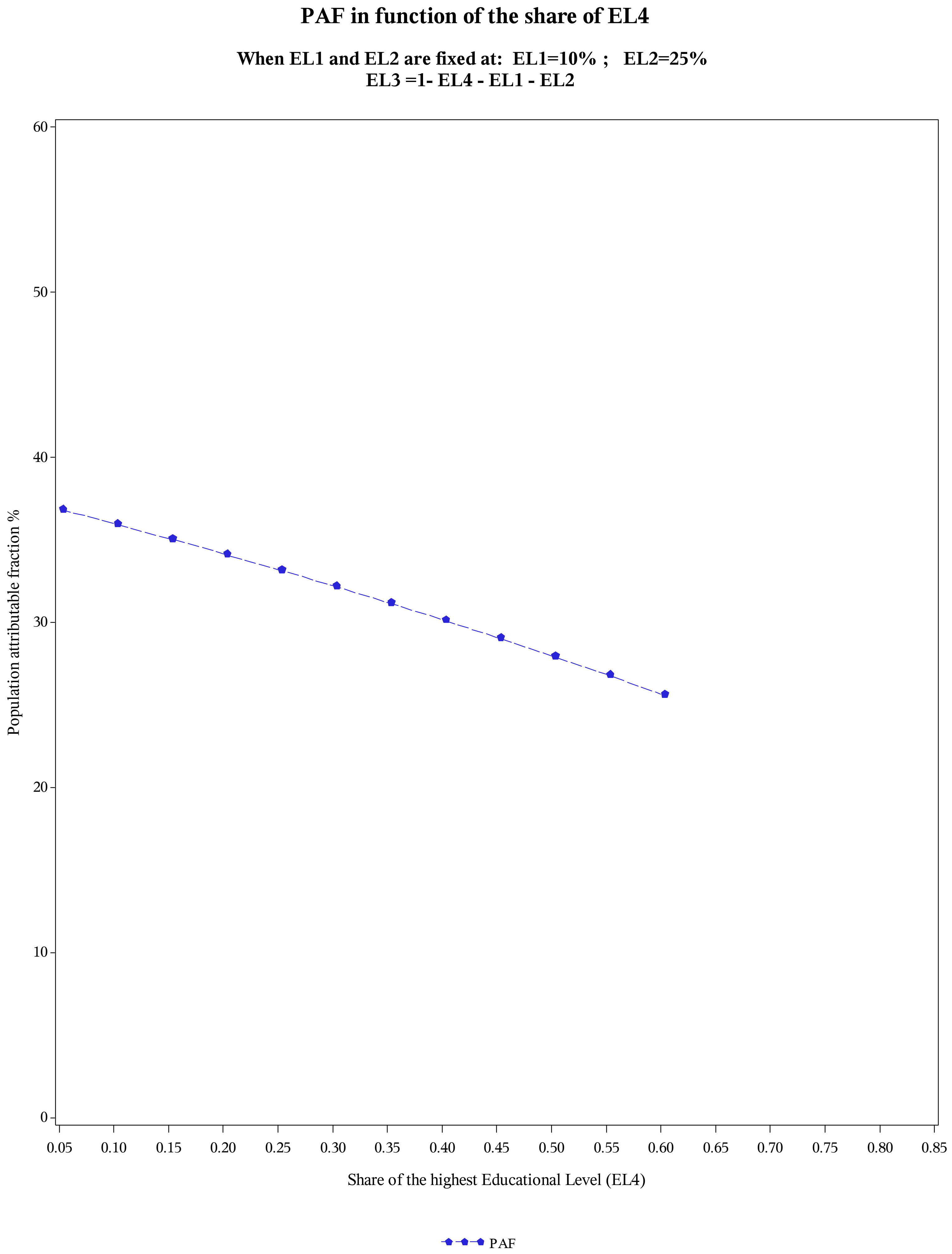

PAF in function of the share of EL4
When EL1 and EL2 are fixed at: EL1=10% ; EL2=25%
EL3 =1- EL4 - EL1 - EL2
Population attributable fraction %
0
10
20
30
40
50
60
0.05
0.10
0.15
0.20
0.25
0.30
0.35
0.40
0.45
0.50
0.55
0.60
0.65
0.70
0.75
0.80
0.85
Share of the highest Educational Level (EL4)
PAF

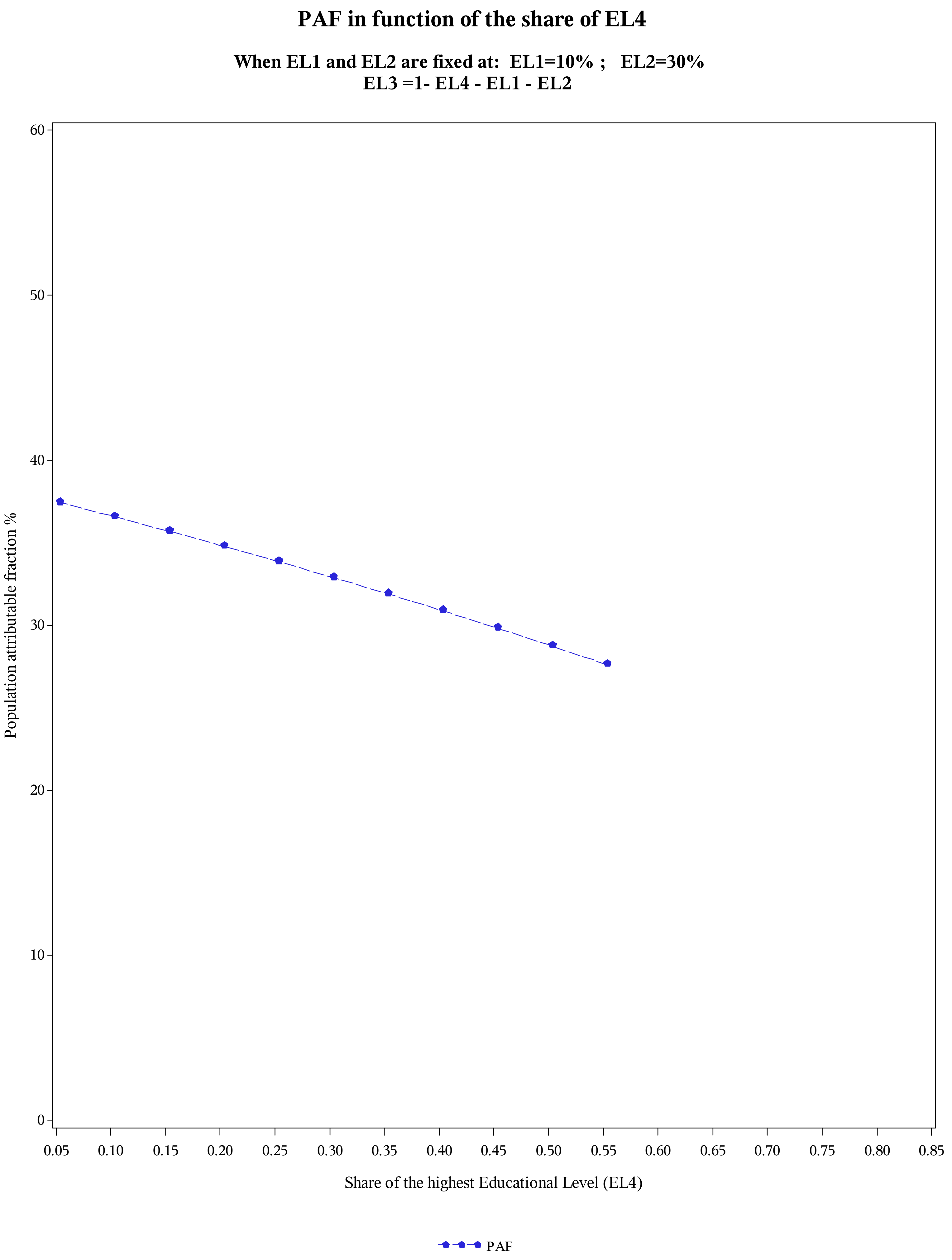
PAF in function of the share of EL4
When EL1 and EL2 are fixed at: EL1=10% ; EL2=30%
EL3 =1- EL4 - EL1 - EL2
Population attributable fraction %
0
10
20
30
40
50
60
0.05
0.10
0.15
0.20
0.25
0.30
0.35
0.40
0.45
0.50
0.55
0.60
0.65
0.70
0.75
0.80
0.85
Share of the highest Educational Level (EL4)
PAF

PAF in function of the share of EL4

When EL1 and EL2 are fixed at: EL1=10% ; EL2=35%
EL3 =1- EL4 - EL1 - EL2

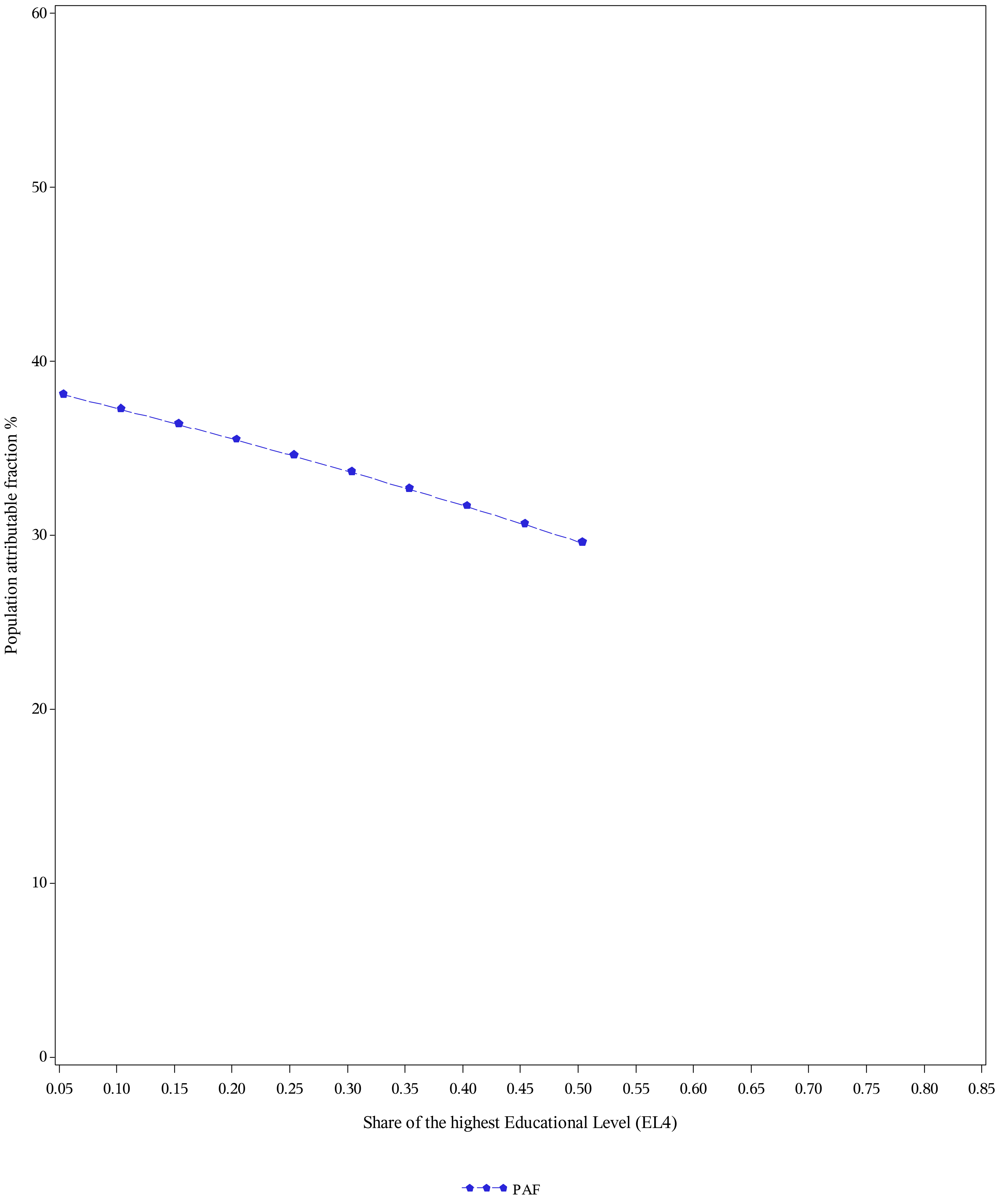

PAF in function of the share of EL4

When EL1 and EL2 are fixed at: EL1=10% ; EL2=40%
EL3 =1- EL4 - EL1 - EL2

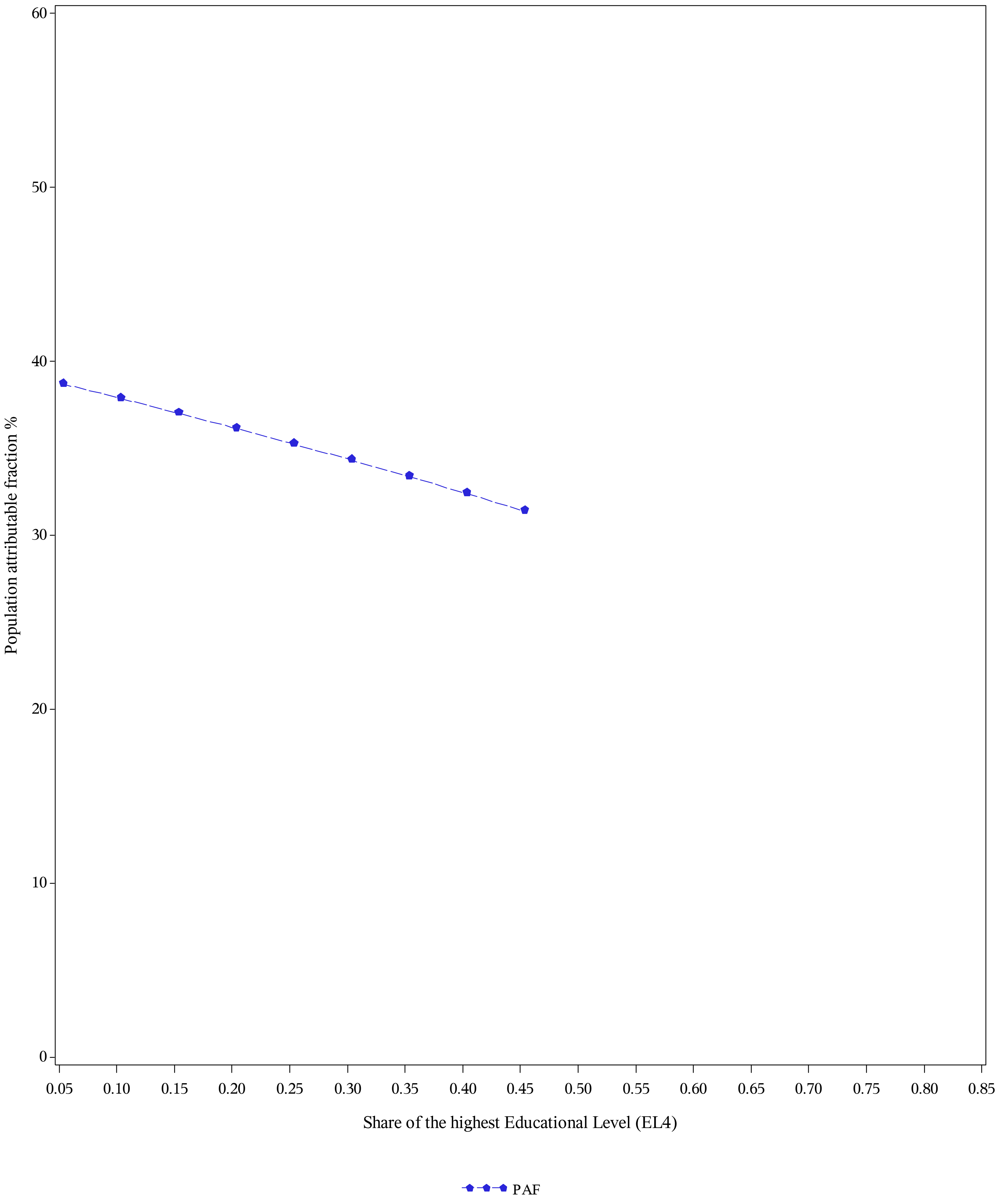

PAF in function of the share of EL4

When EL1 and EL2 are fixed at: EL1=10% ; EL2=45%
EL3 =1- EL4 - EL1 - EL2

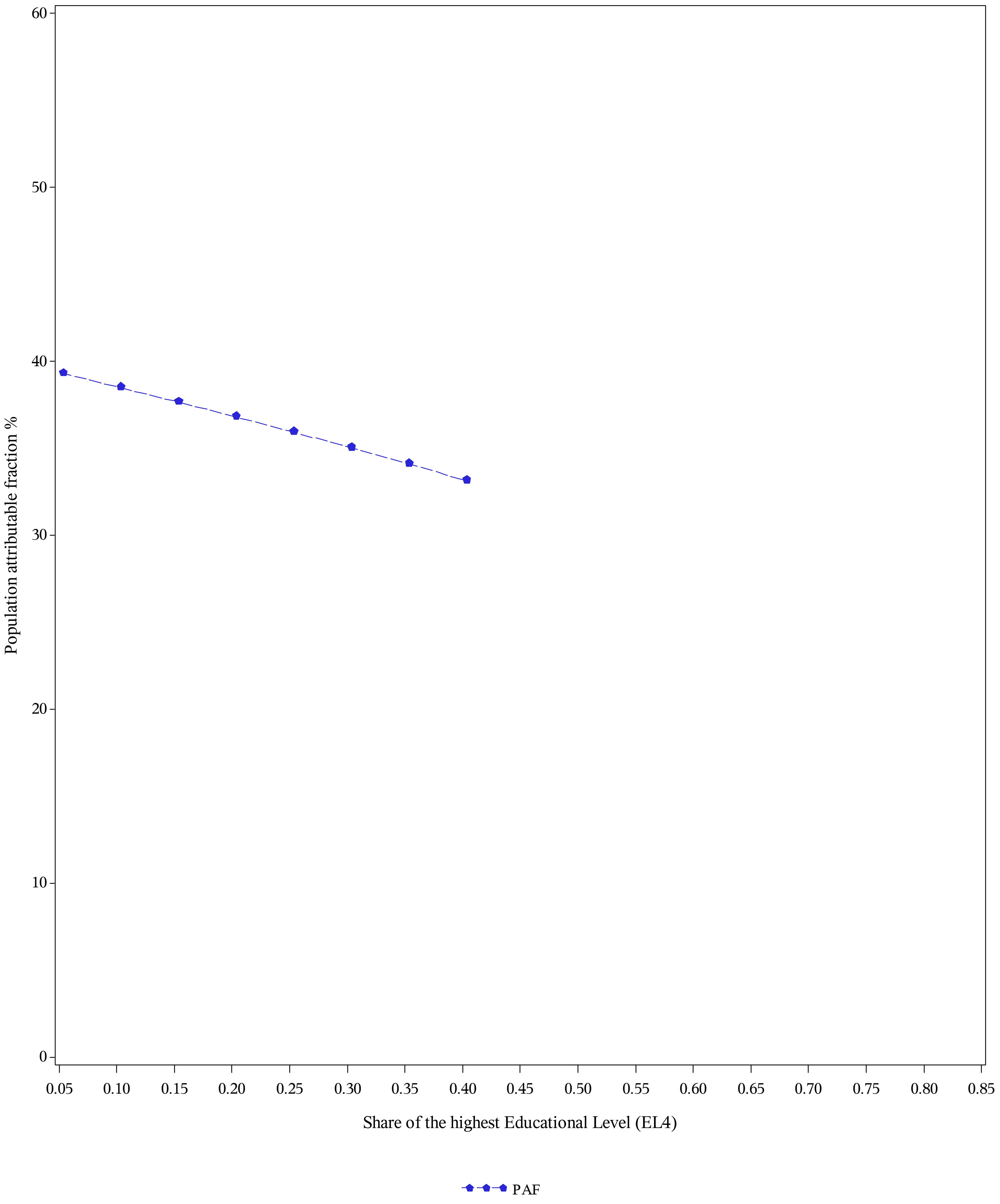

PAF in function of the share of EL4

When EL1 and EL2 are fixed at: EL1=10% ; EL2=50%
EL3 =1- EL4 - EL1 - EL2

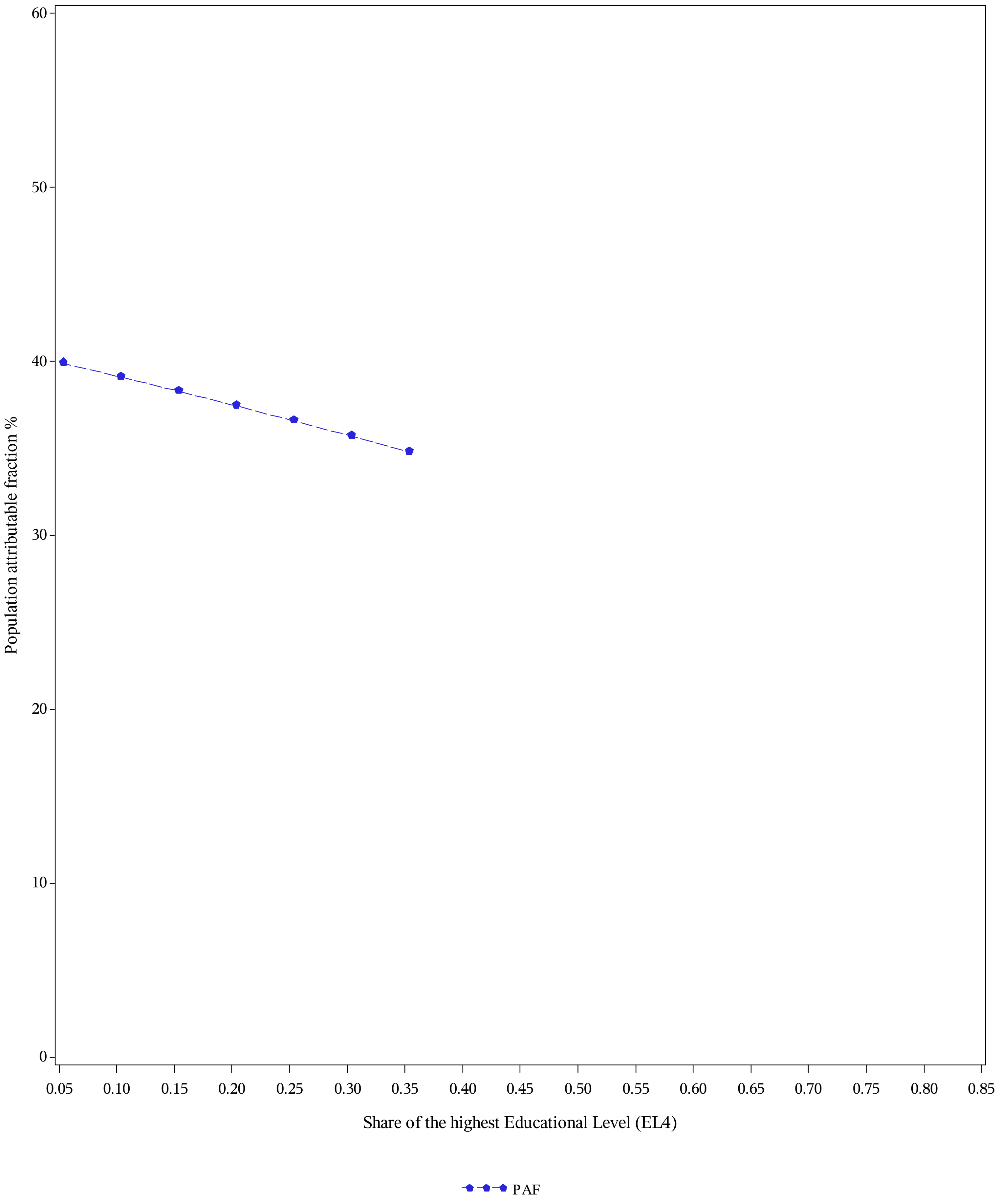

PAF in function of the share of EL4

When EL1 and EL2 are fixed at: EL1=10% ; EL2=55%
EL3 =1- EL4 - EL1 - EL2

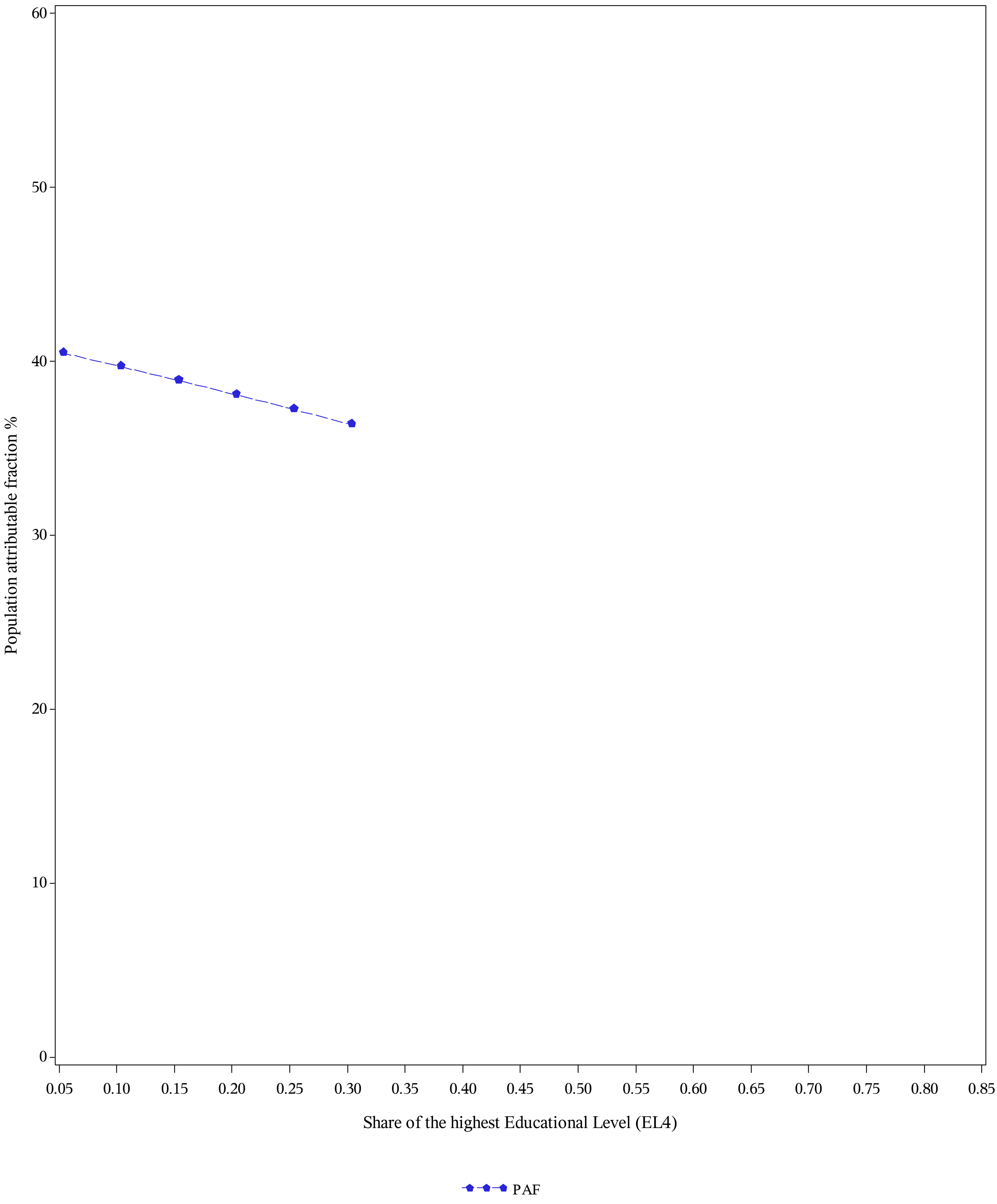

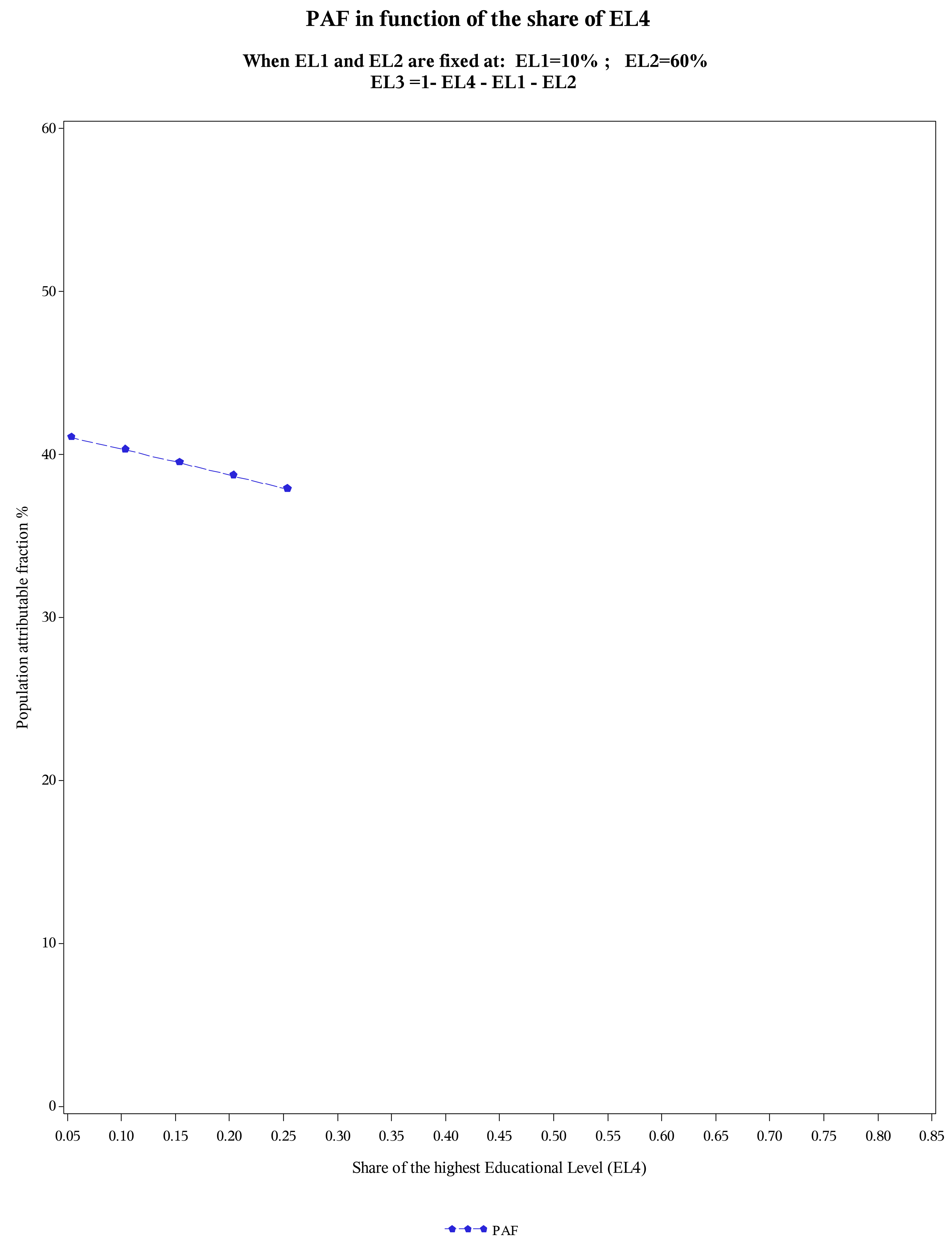
PAF in function of the share of EL4
When EL1 and EL2 are fixed at: EL1=10% ; EL2=60%
EL3 =1- EL4 - EL1 - EL2
Population attributable fraction %
0
10
20
30
40
50
60
0.05
0.10
0.15
0.20
0.25
0.30
0.35
0.40
0.45
0.50
0.55
0.60
0.65
0.70
0.75
0.80
0.85
Share of the highest Educational Level (EL4)
PAF

PAF in function of the share of EL4

When EL1 and EL2 are fixed at: EL1=10% ; EL2=65%
EL3 =1- EL4 - EL1 - EL2

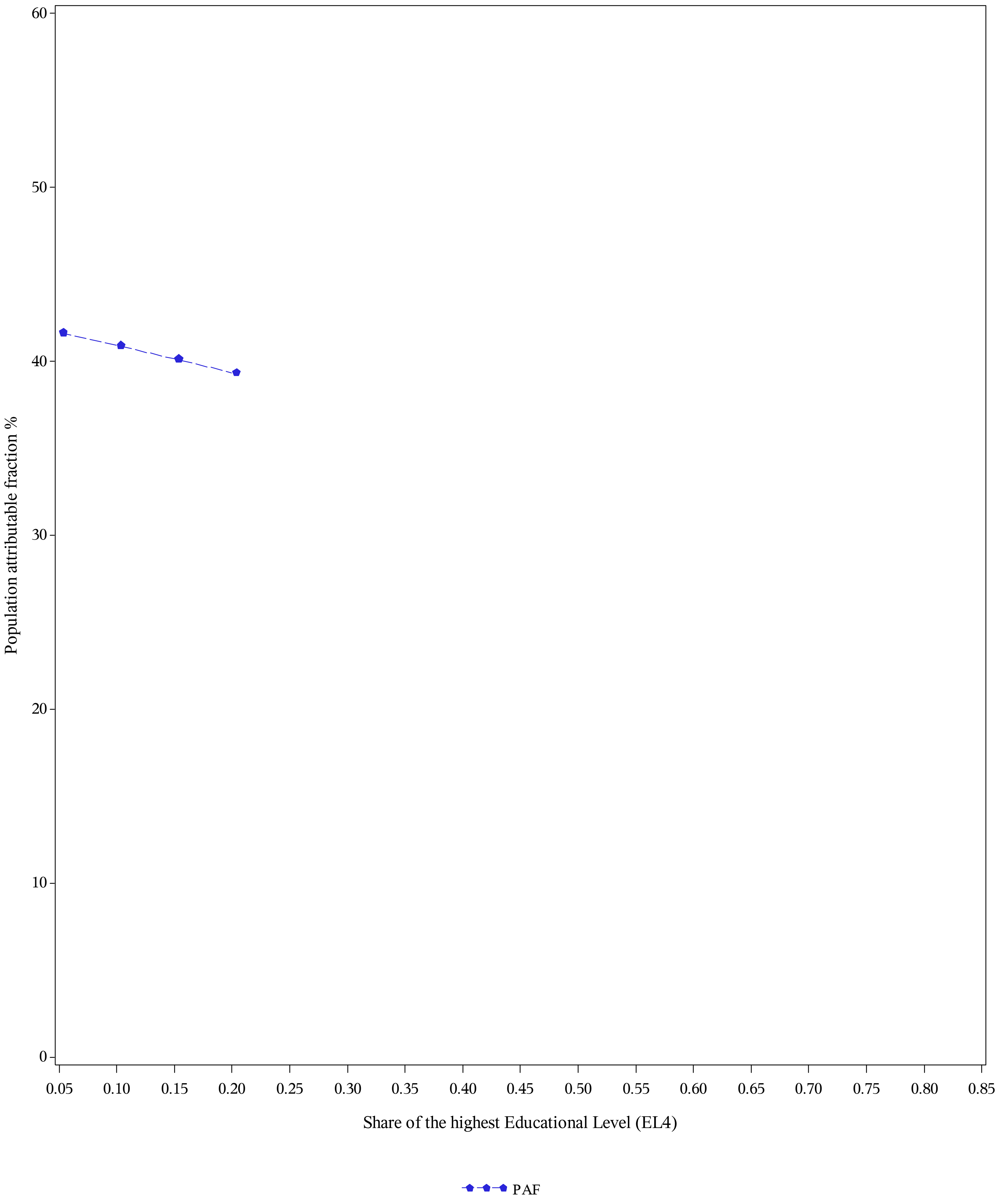

PAF in function of the share of EL4

When EL1 and EL2 are fixed at: EL1=10% ; EL2=70%
EL3 =1- EL4 - EL1 - EL2

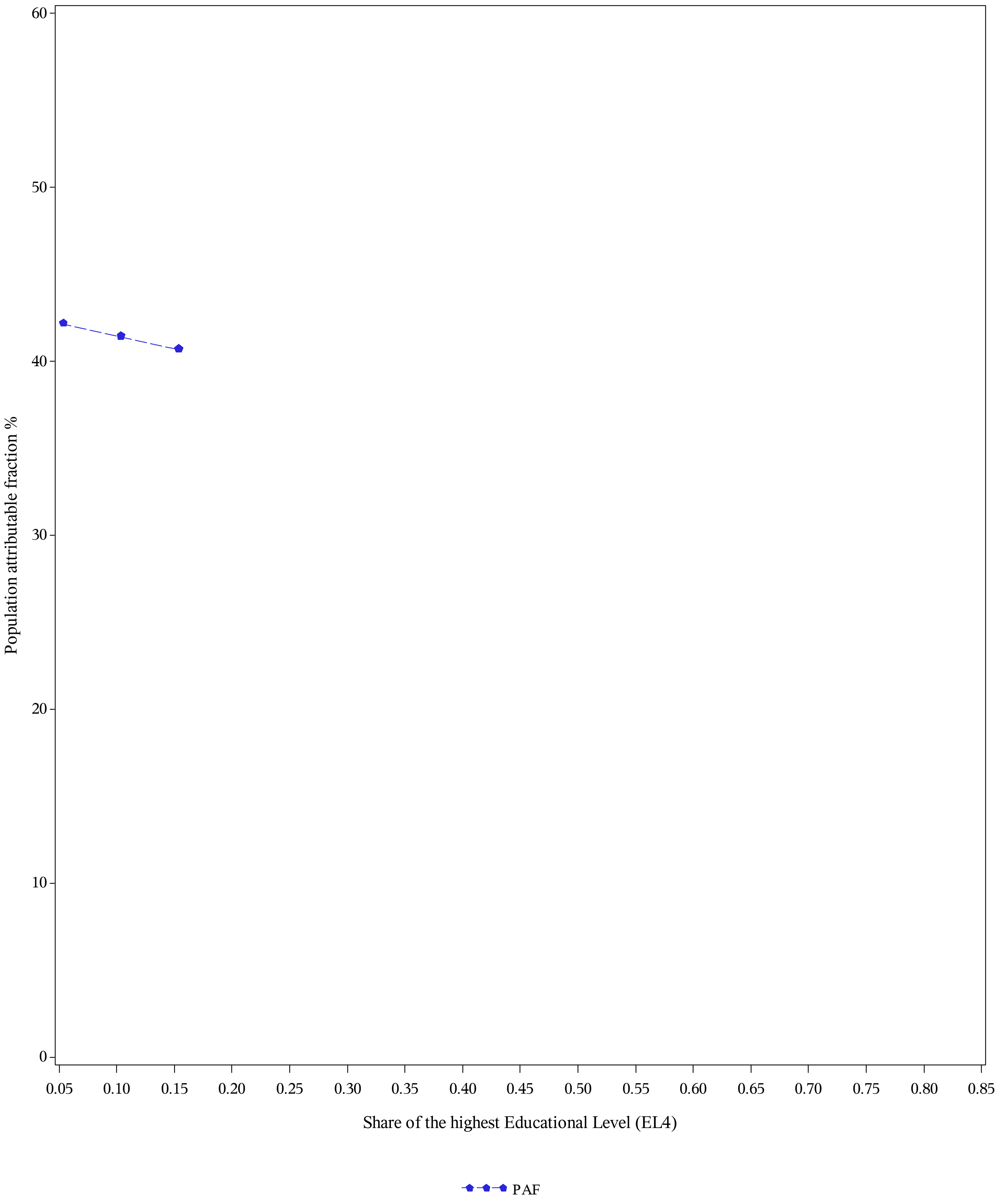

PAF in function of the share of EL4

When EL1 and EL2 are fixed at: EL1=10% ; EL2=75%
EL3 =1- EL4 - EL1 - EL2

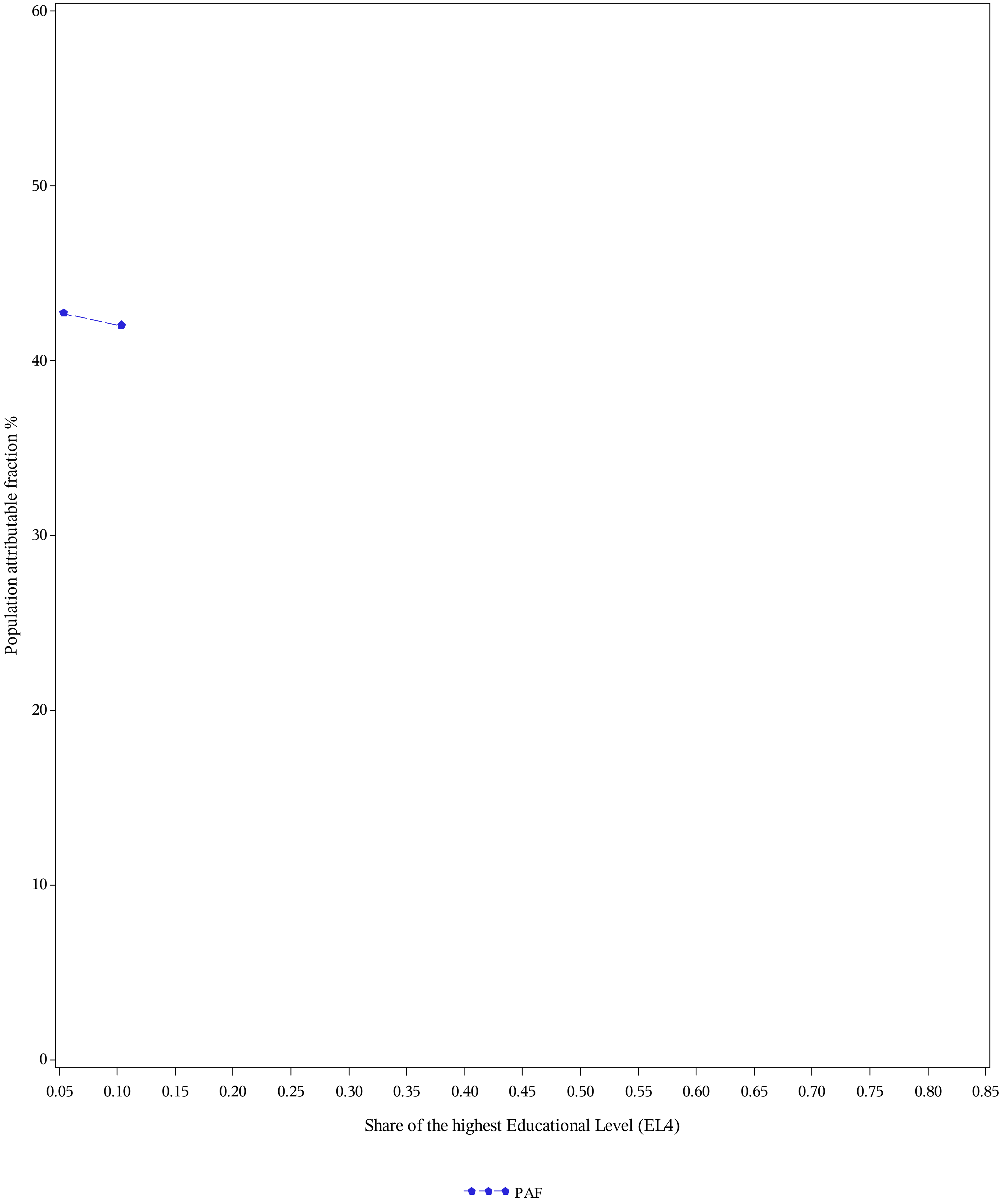

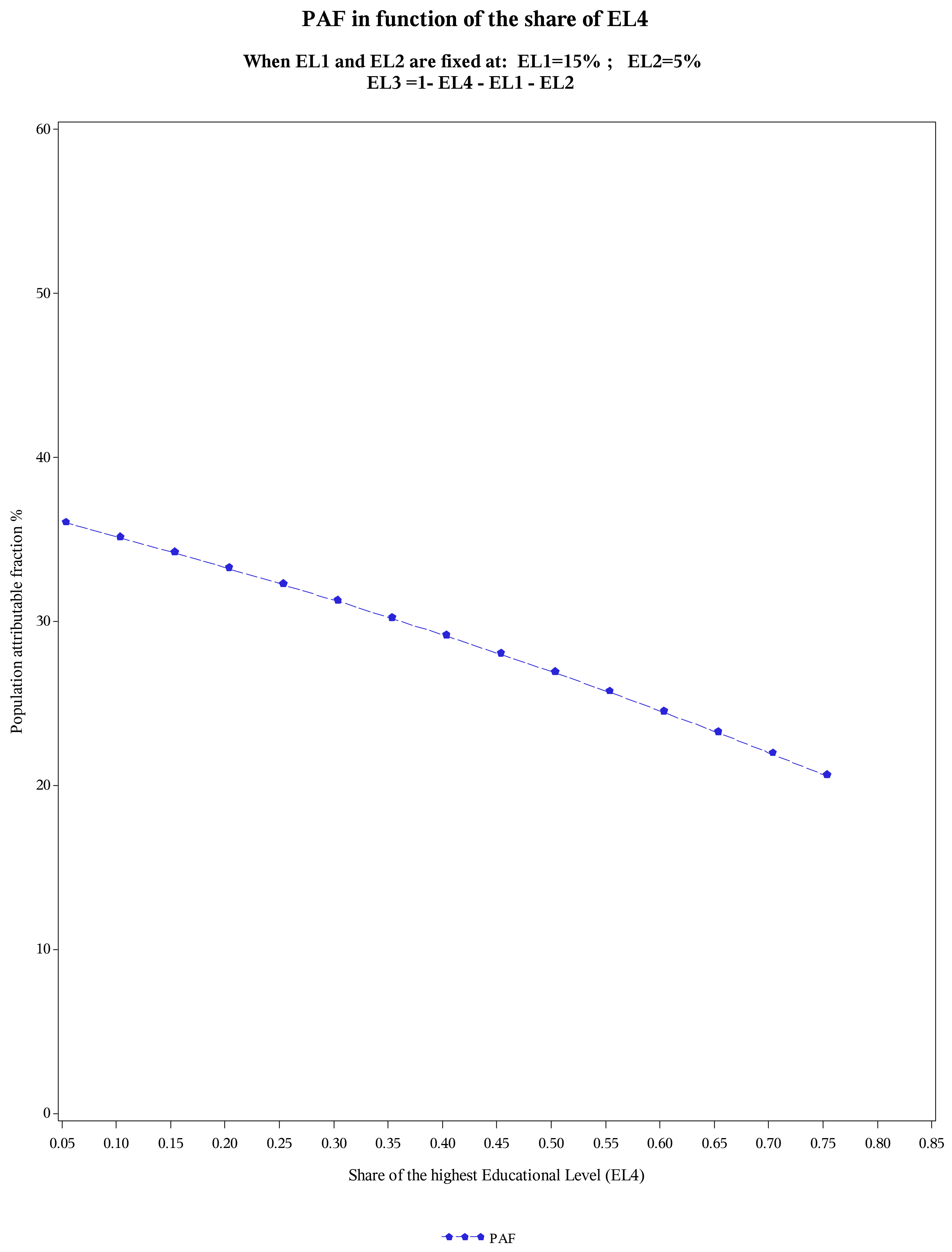
PAF in function of the share of EL4
When EL1 and EL2 are fixed at: EL1=15% ; EL2=5%
EL3 =1- EL4 - EL1 - EL2
Population attributable fraction %
0
10
20
30
40
50
60
0.05
0.10
0.15
0.20
0.25
0.30
0.35
0.40
0.45
0.50
0.55
0.60
0.65
0.70
0.75
0.80
0.85
Share of the highest Educational Level (EL4)
PAF

PAF in function of the share of EL4

When EL1 and EL2 are fixed at: EL1=15% ; EL2=10%
EL3 =1- EL4 - EL1 - EL2

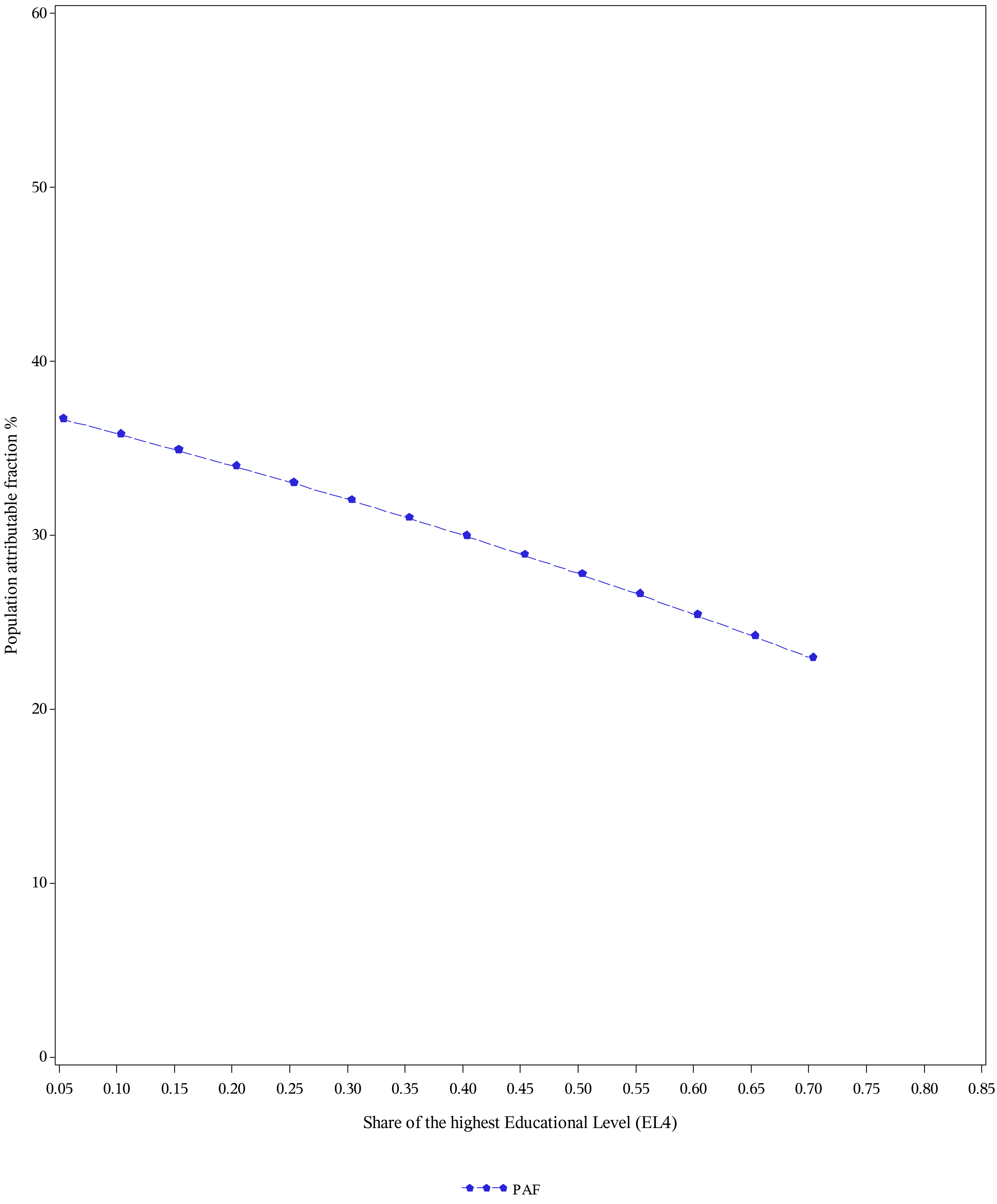

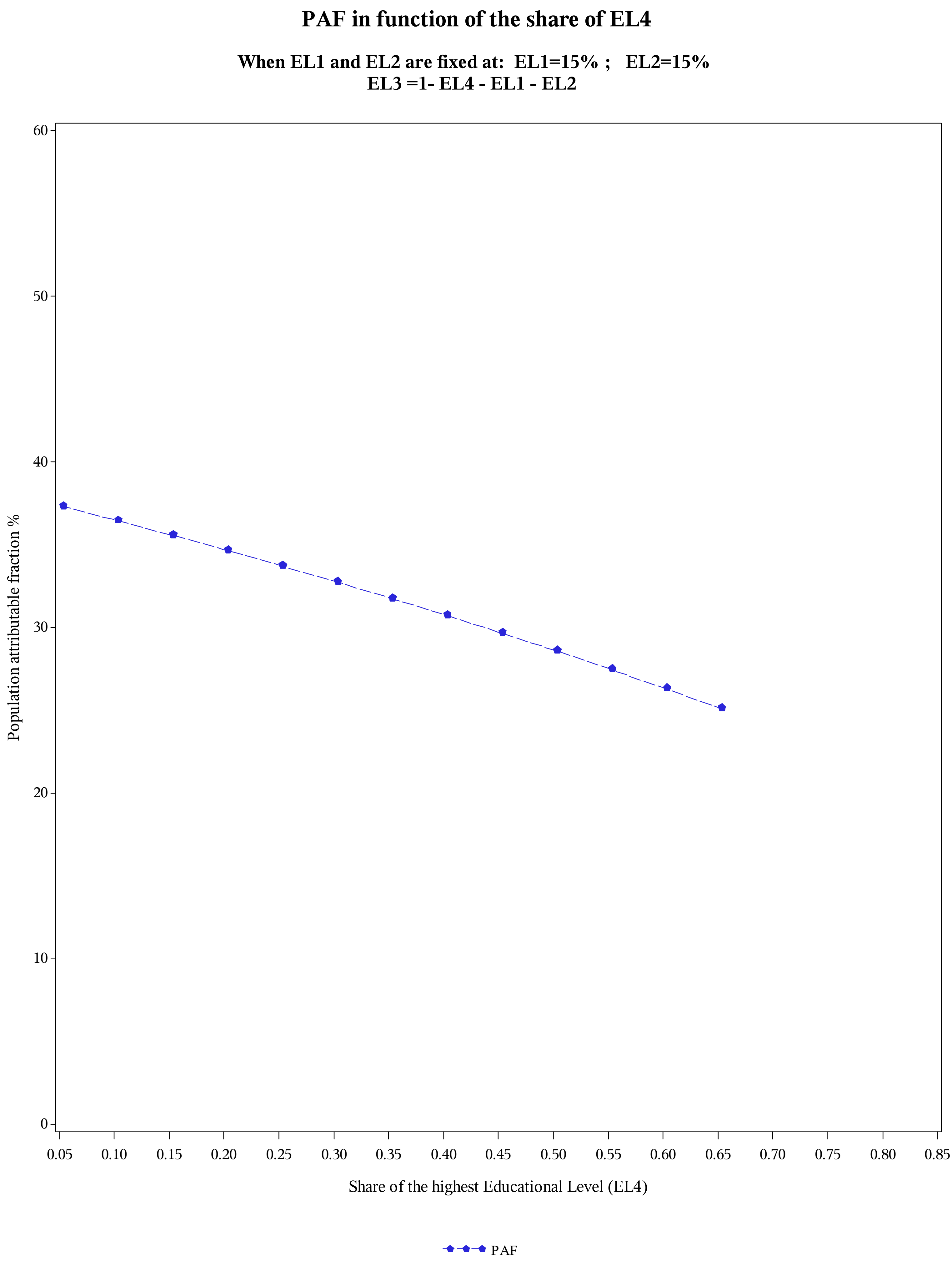

PAF in function of the share of EL4
When EL1 and EL2 are fixed at: EL1=15% ; EL2=15%
EL3 =1- EL4 - EL1 - EL2
Population attributable fraction %
0
10
20
30
40
50
60
0.05
0.10
0.15
0.20
0.25
0.30
0.35
0.40
0.45
0.50
0.55
0.60
0.65
0.70
0.75
0.80
0.85
Share of the highest Educational Level (EL4)
PAF

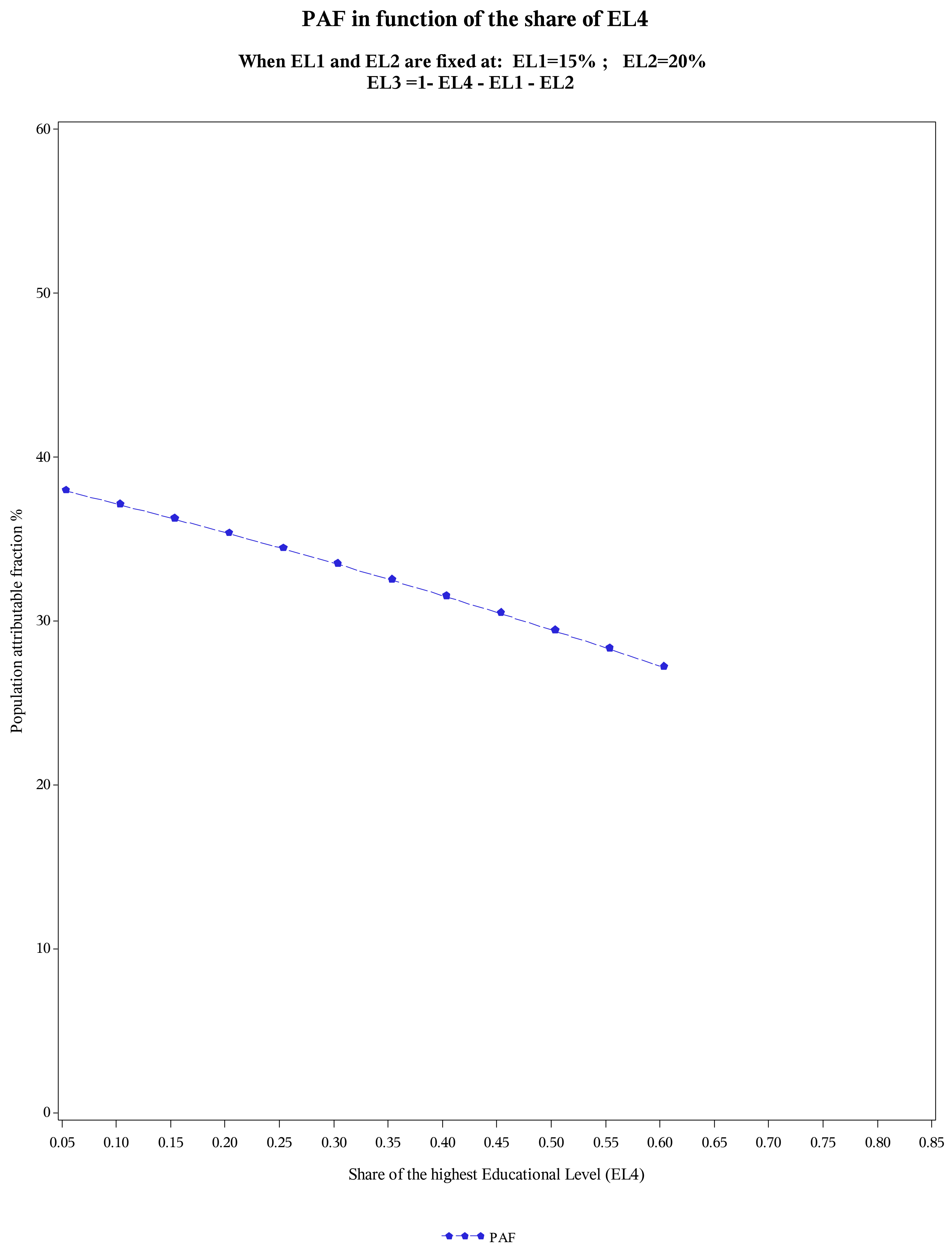
PAF in function of the share of EL4
When EL1 and EL2 are fixed at: EL1=15% ; EL2=20%
EL3 =1- EL4 - EL1 - EL2
Population attributable fraction %
0
10
20
30
40
50
60
0.05
0.10
0.15
0.20
0.25
0.30
0.35
0.40
0.45
0.50
0.55
0.60
0.65
0.70
0.75
0.80
0.85
Share of the highest Educational Level (EL4)
PAF

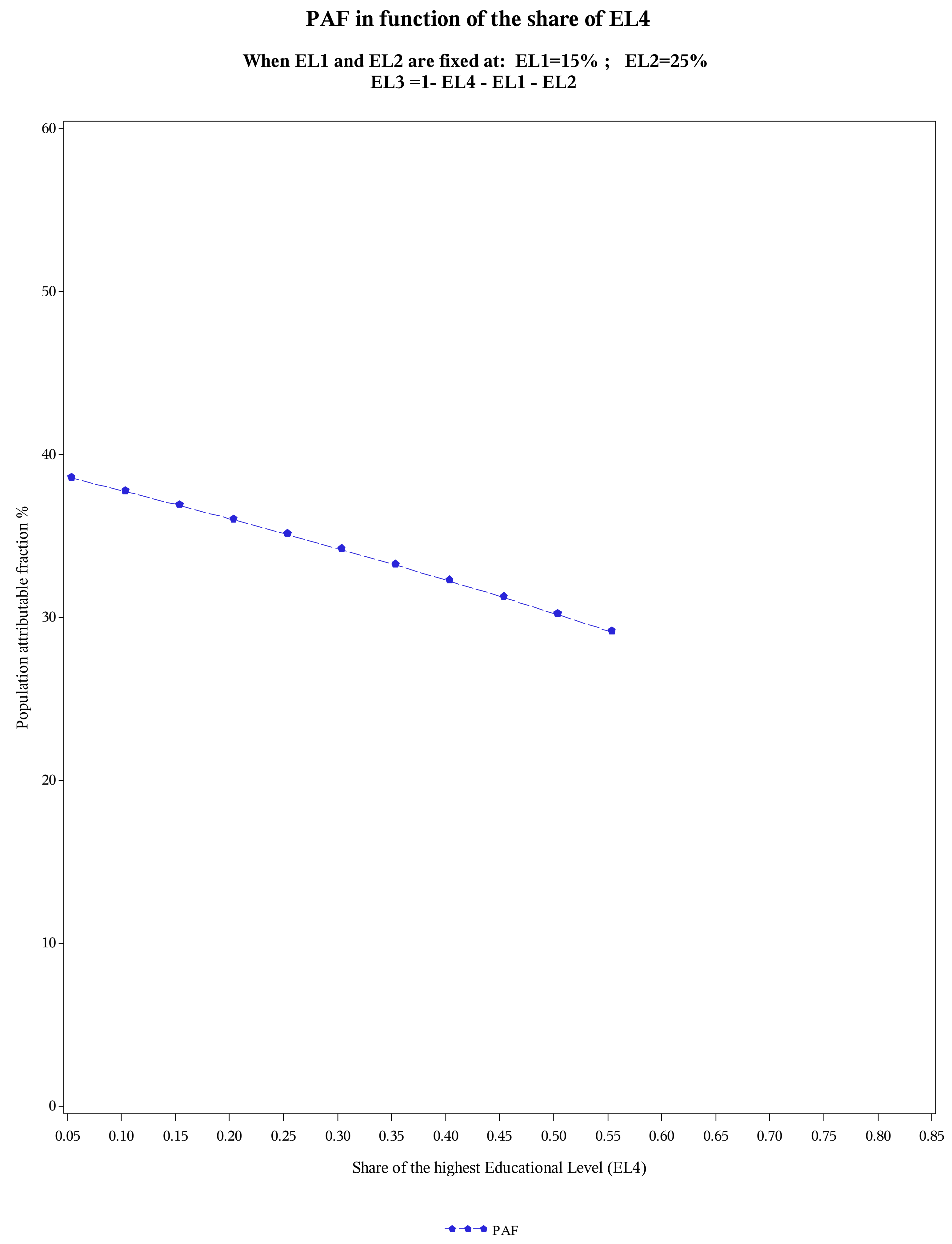
PAF in function of the share of EL4
When EL1 and EL2 are fixed at: EL1=15% ; EL2=25%
EL3 =1- EL4 - EL1 - EL2
Population attributable fraction %
0
10
20
30
40
50
60
0.05
0.10
0.15
0.20
0.25
0.30
0.35
0.40
0.45
0.50
0.55
0.60
0.65
0.70
0.75
0.80
0.85
Share of the highest Educational Level (EL4)
PAF

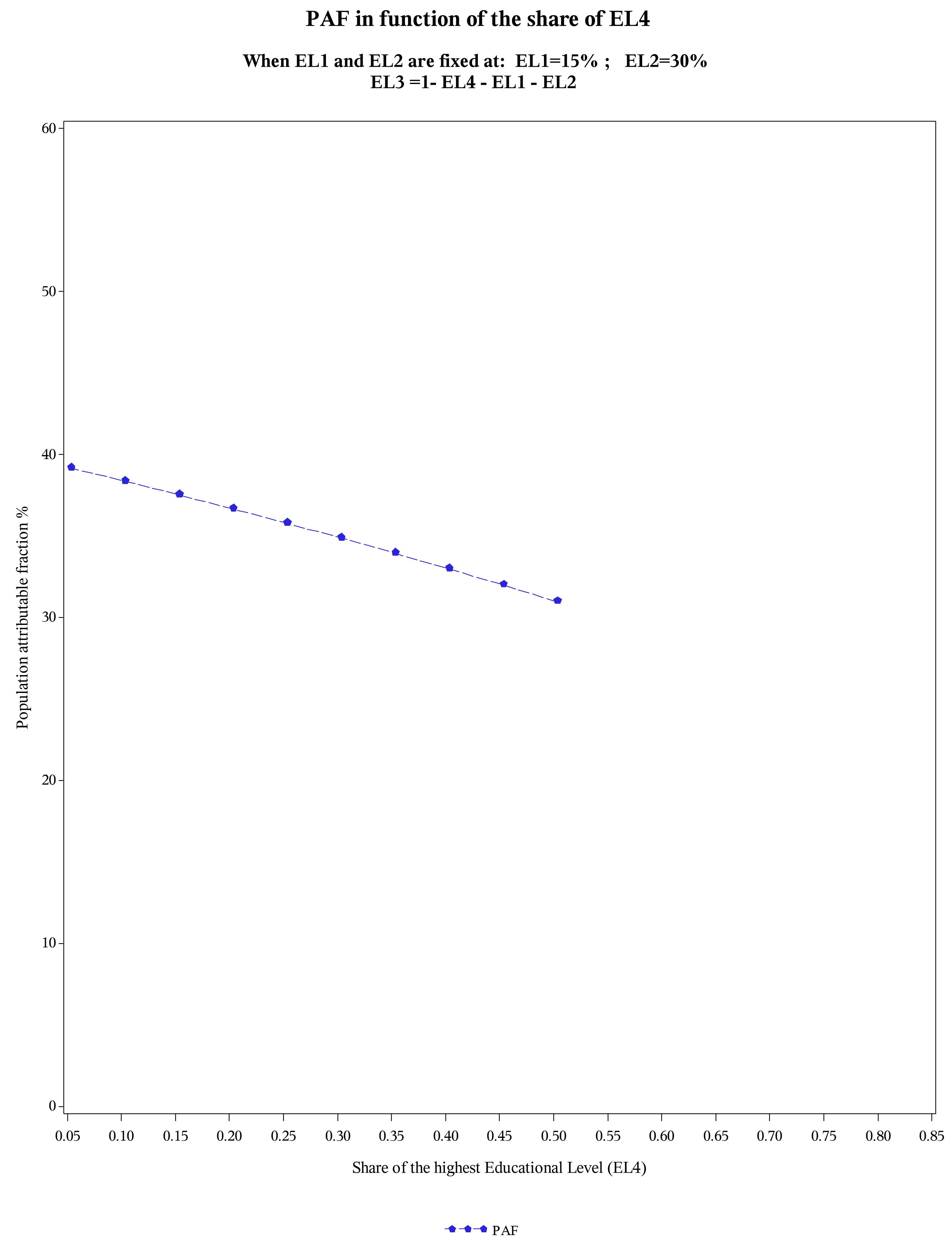
PAF in function of the share of EL4
When EL1 and EL2 are fixed at: EL1=15% ; EL2=30%
EL3 =1- EL4 - EL1 - EL2
Population attributable fraction %
0
10
20
30
40
50
60
0.05
0.10
0.15
0.20
0.25
0.30
0.35
0.40
0.45
0.50
0.55
0.60
0.65
0.70
0.75
0.80
0.85
Share of the highest Educational Level (EL4)
PAF

PAF in function of the share of EL4

When EL1 and EL2 are fixed at: EL1=15% ; EL2=35%
EL3 =1- EL4 - EL1 - EL2

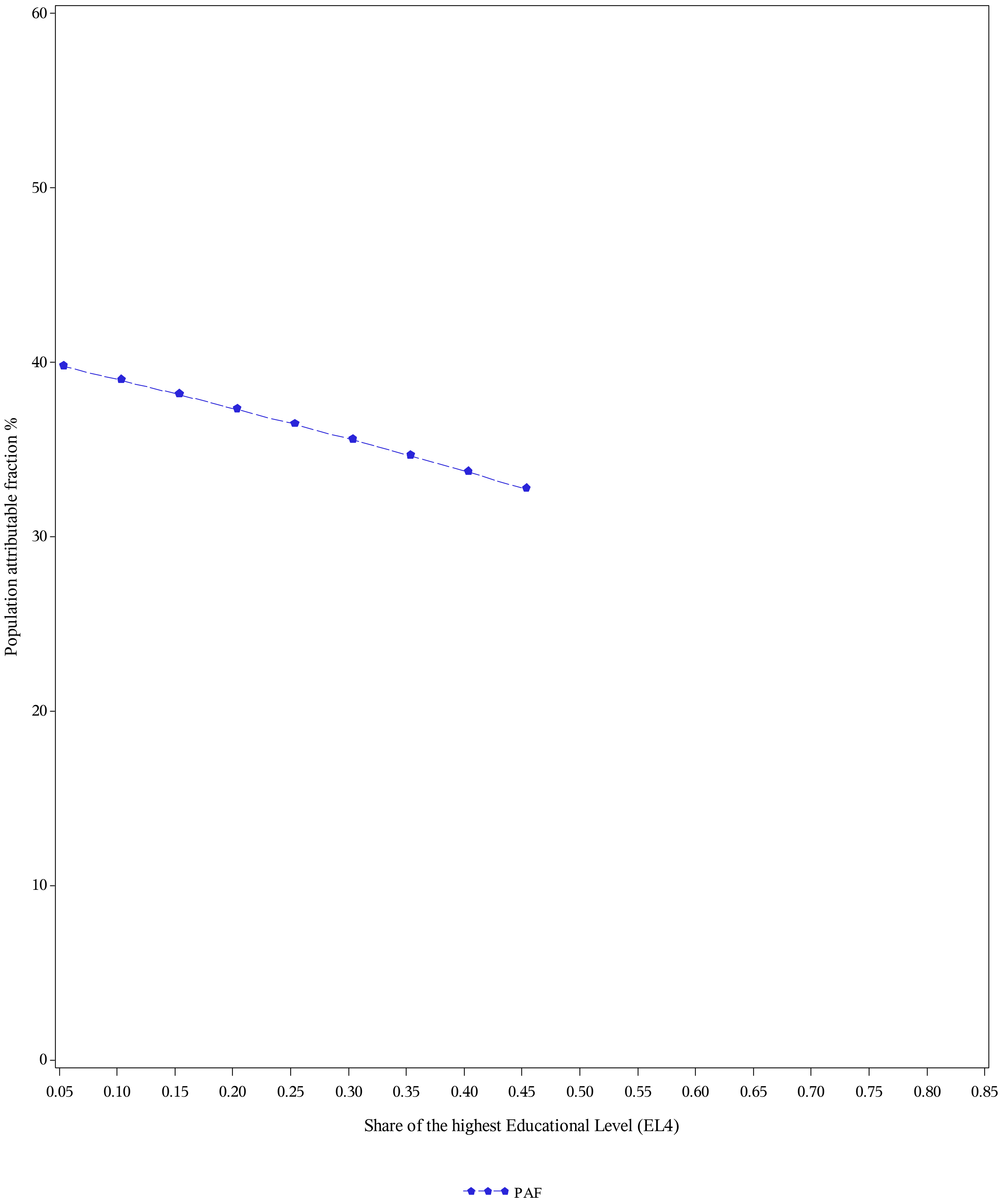

PAF in function of the share of EL4

When EL1 and EL2 are fixed at: EL1=15% ; EL2=40%
EL3 =1- EL4 - EL1 - EL2

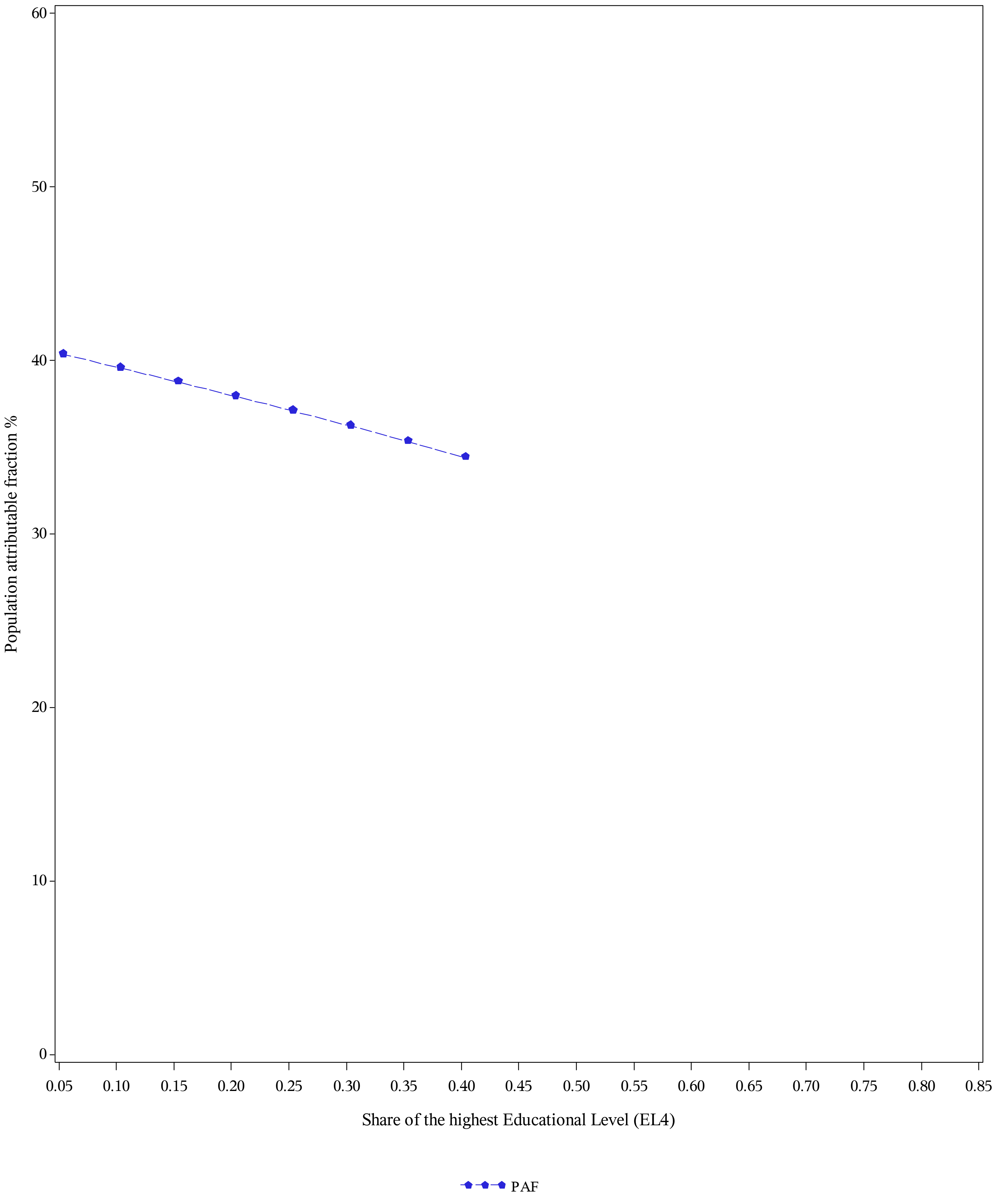

PAF in function of the share of EL4

When EL1 and EL2 are fixed at: EL1=15% ; EL2=45%
EL3 =1- EL4 - EL1 - EL2

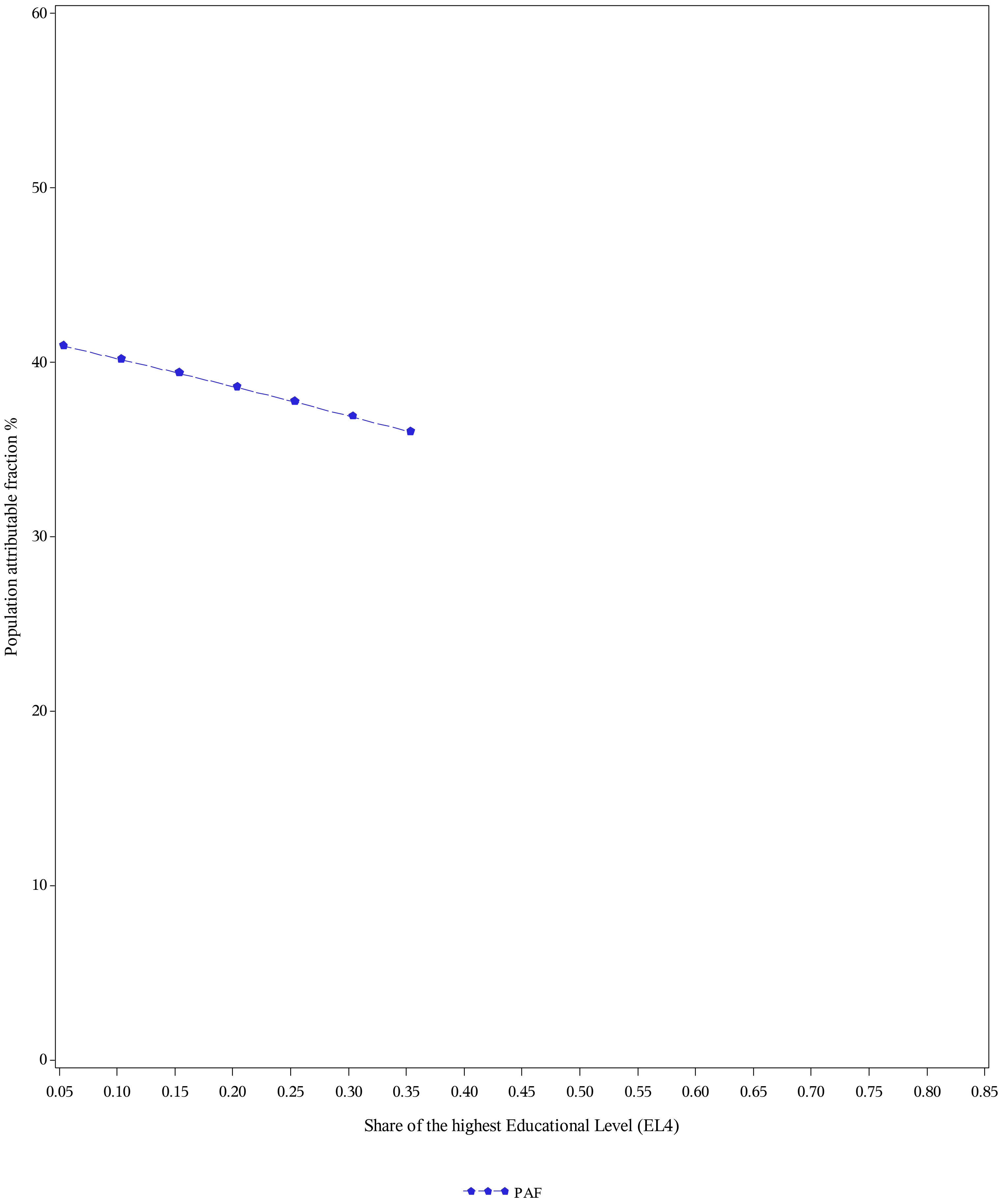

PAF in function of the share of EL4

When EL1 and EL2 are fixed at: EL1=15% ; EL2=50%
EL3 =1- EL4 - EL1 - EL2

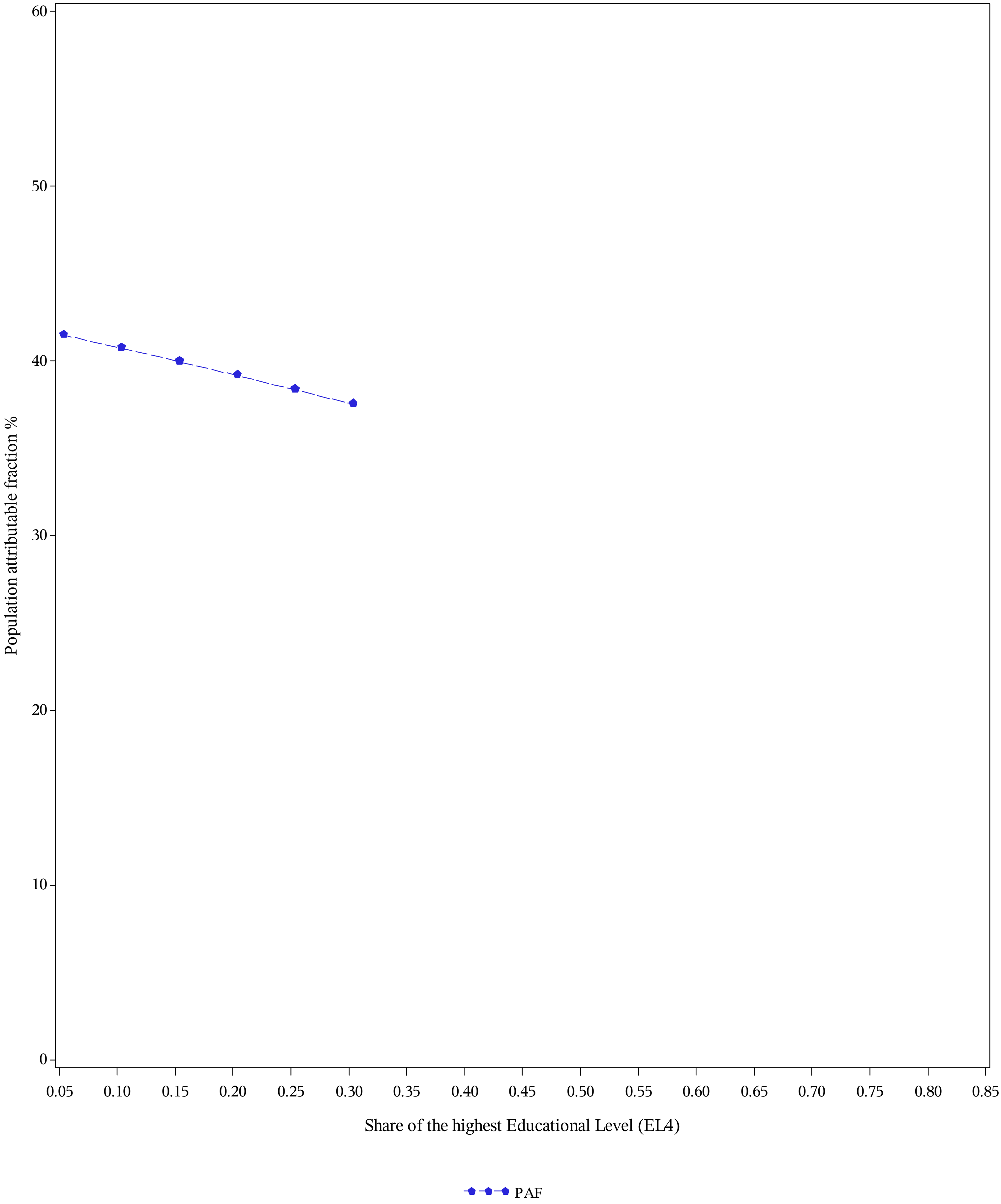

PAF in function of the share of EL4

When EL1 and EL2 are fixed at: EL1=15% ; EL2=55%
EL3 =1- EL4 - EL1 - EL2

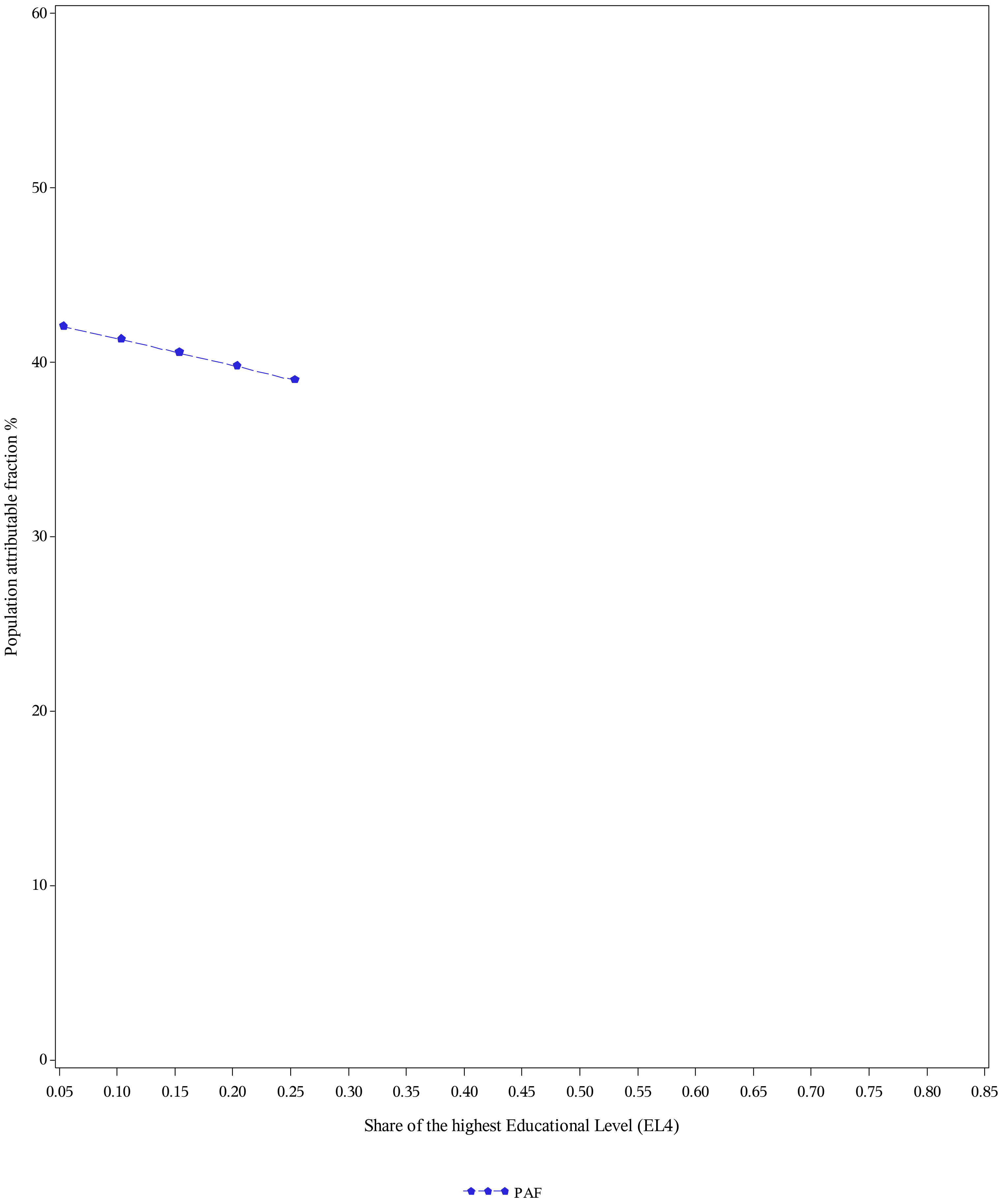

PAF in function of the share of EL4

When EL1 and EL2 are fixed at: EL1=15% ; EL2=60%
EL3 =1- EL4 - EL1 - EL2

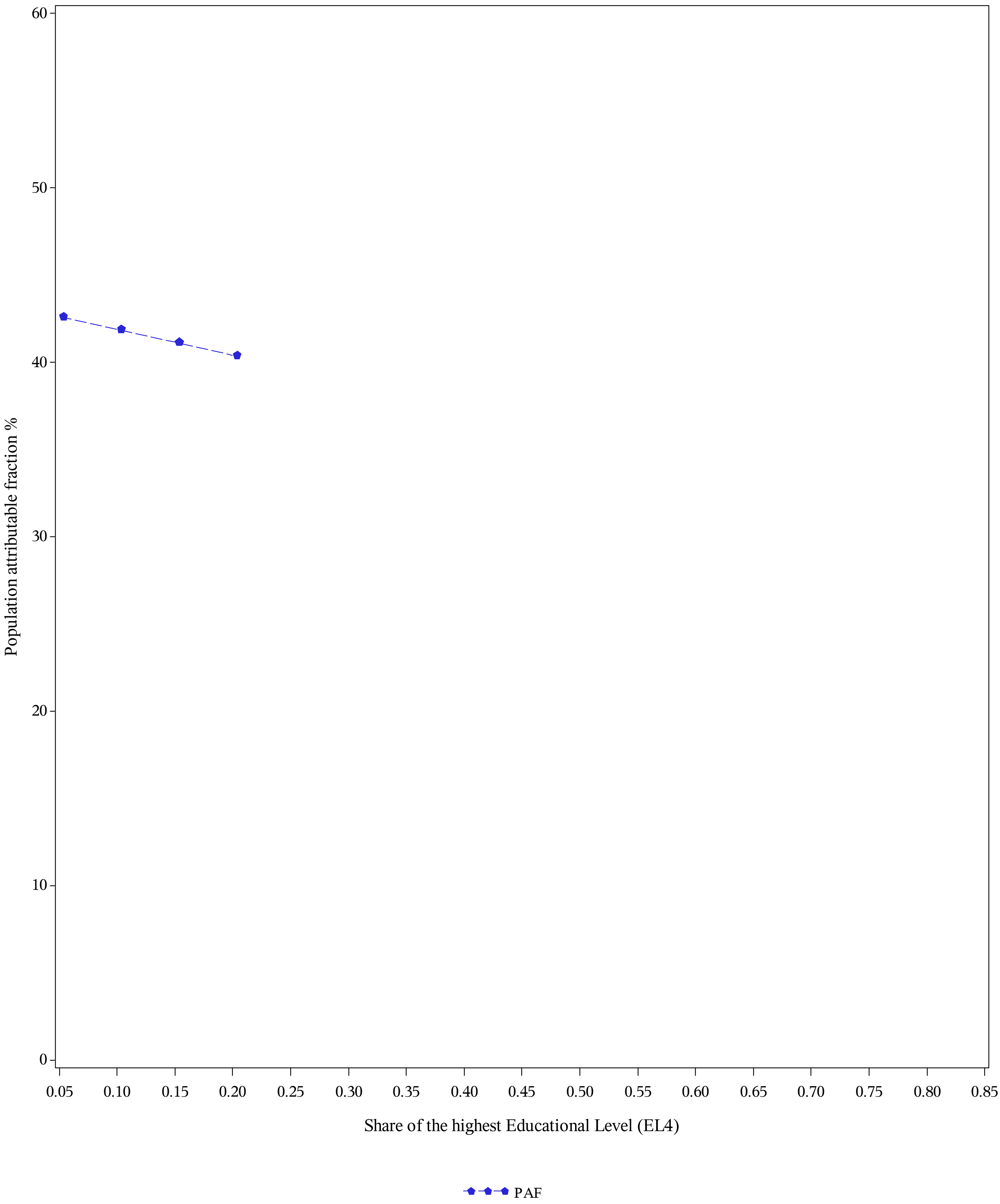

PAF in function of the share of EL4

When EL1 and EL2 are fixed at: EL1=15% ; EL2=65%
EL3 =1- EL4 - EL1 - EL2

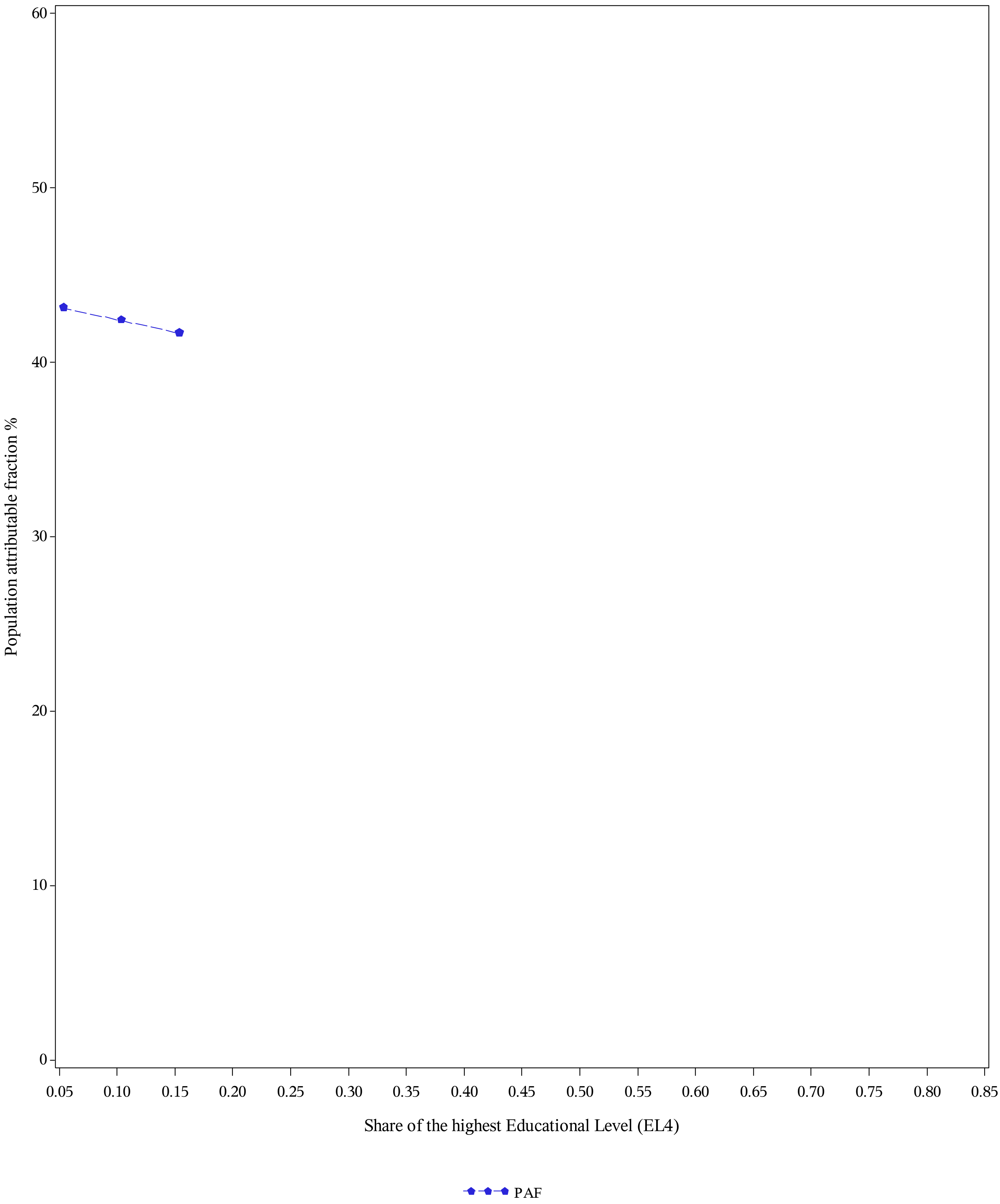

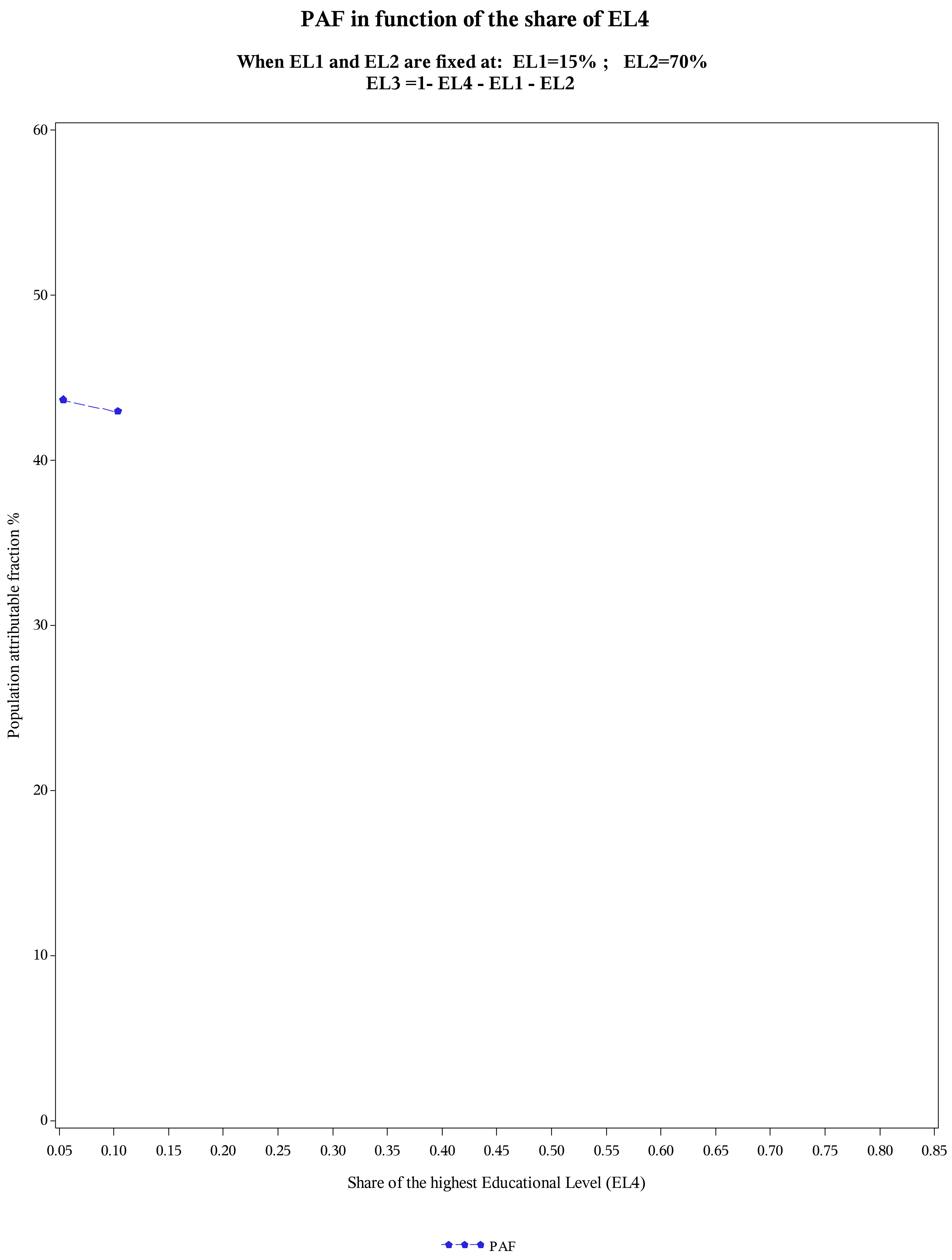
PAF in function of the share of EL4
When EL1 and EL2 are fixed at: EL1=15% ; EL2=70%
EL3 =1- EL4 - EL1 - EL2
Population attributable fraction %
0
10
20
30
40
50
60
0.05
0.10
0.15
0.20
0.25
0.30
0.35
0.40
0.45
0.50
0.55
0.60
0.65
0.70
0.75
0.80
0.85
Share of the highest Educational Level (EL4)
PAF

PAF in function of the share of EL4

When EL1 and EL2 are fixed at: EL1=20% ; EL2=5%
EL3 =1- EL4 - EL1 - EL2

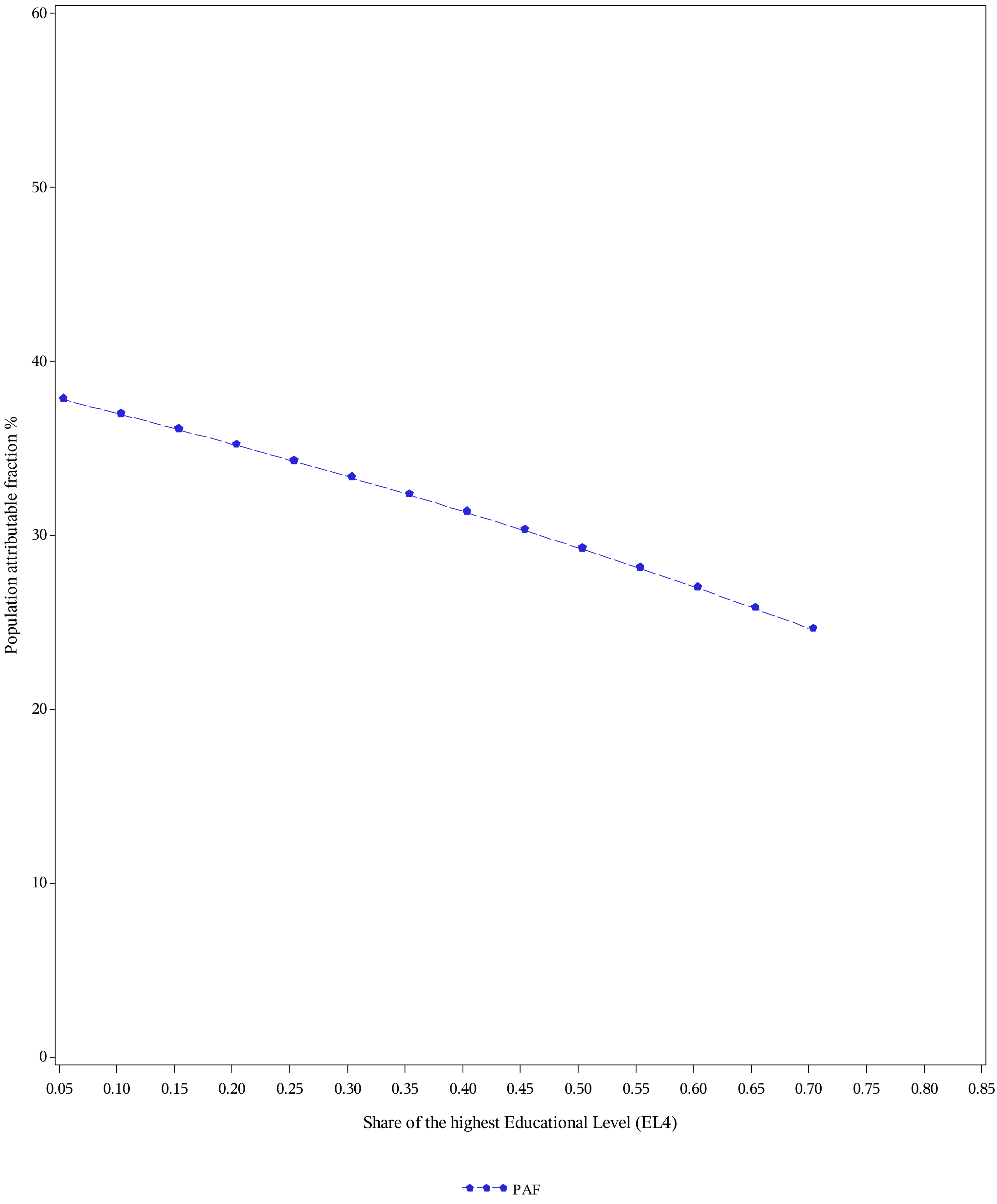

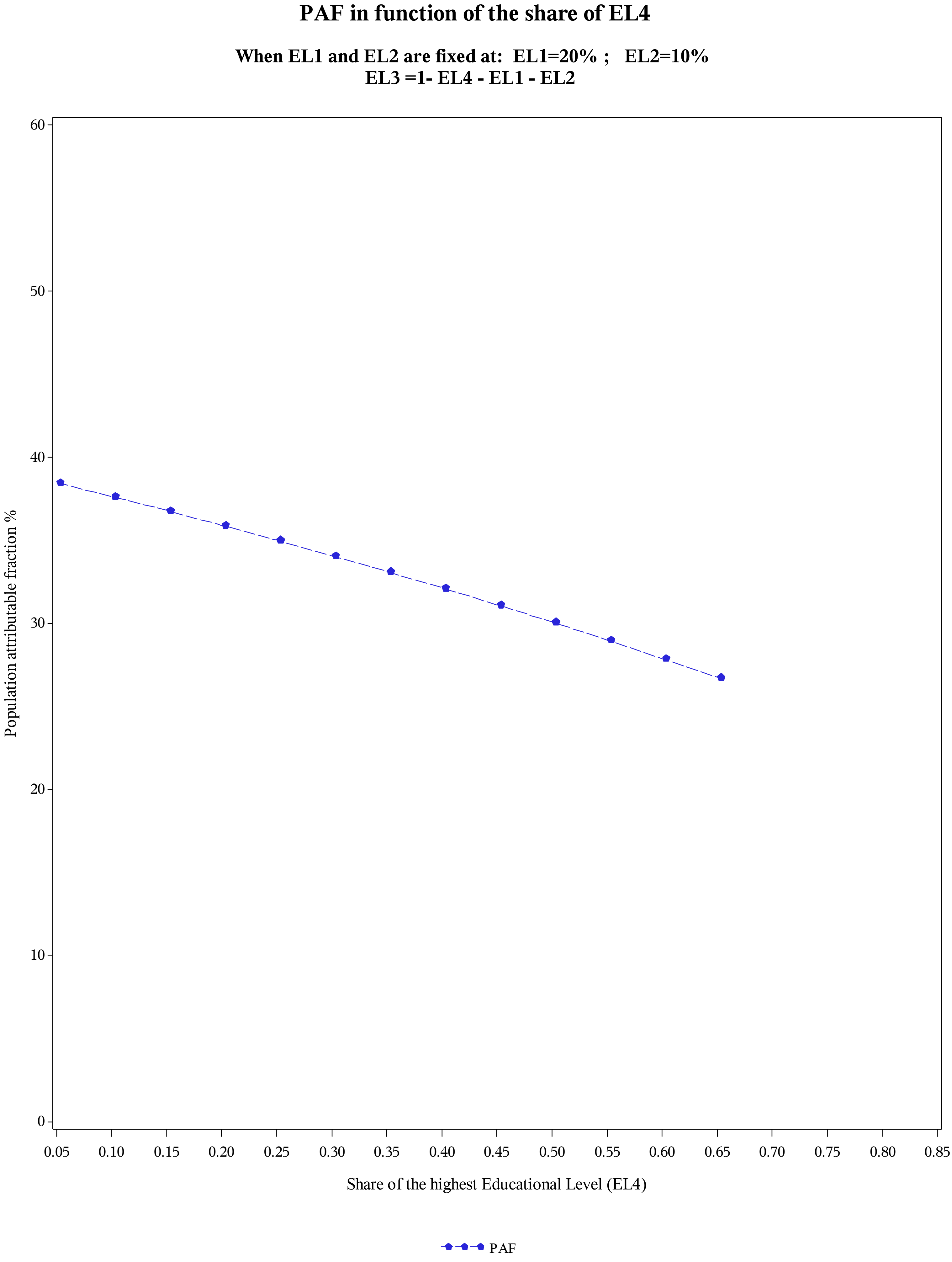

PAF in function of the share of EL4
When EL1 and EL2 are fixed at: EL1=20% ; EL2=10%
EL3 =1- EL4 - EL1 - EL2
Population attributable fraction %
0
10
20
30
40
50
60
0.05
0.10
0.15
0.20
0.25
0.30
0.35
0.40
0.45
0.50
0.55
0.60
0.65
0.70
0.75
0.80
0.85
Share of the highest Educational Level (EL4)
PAF

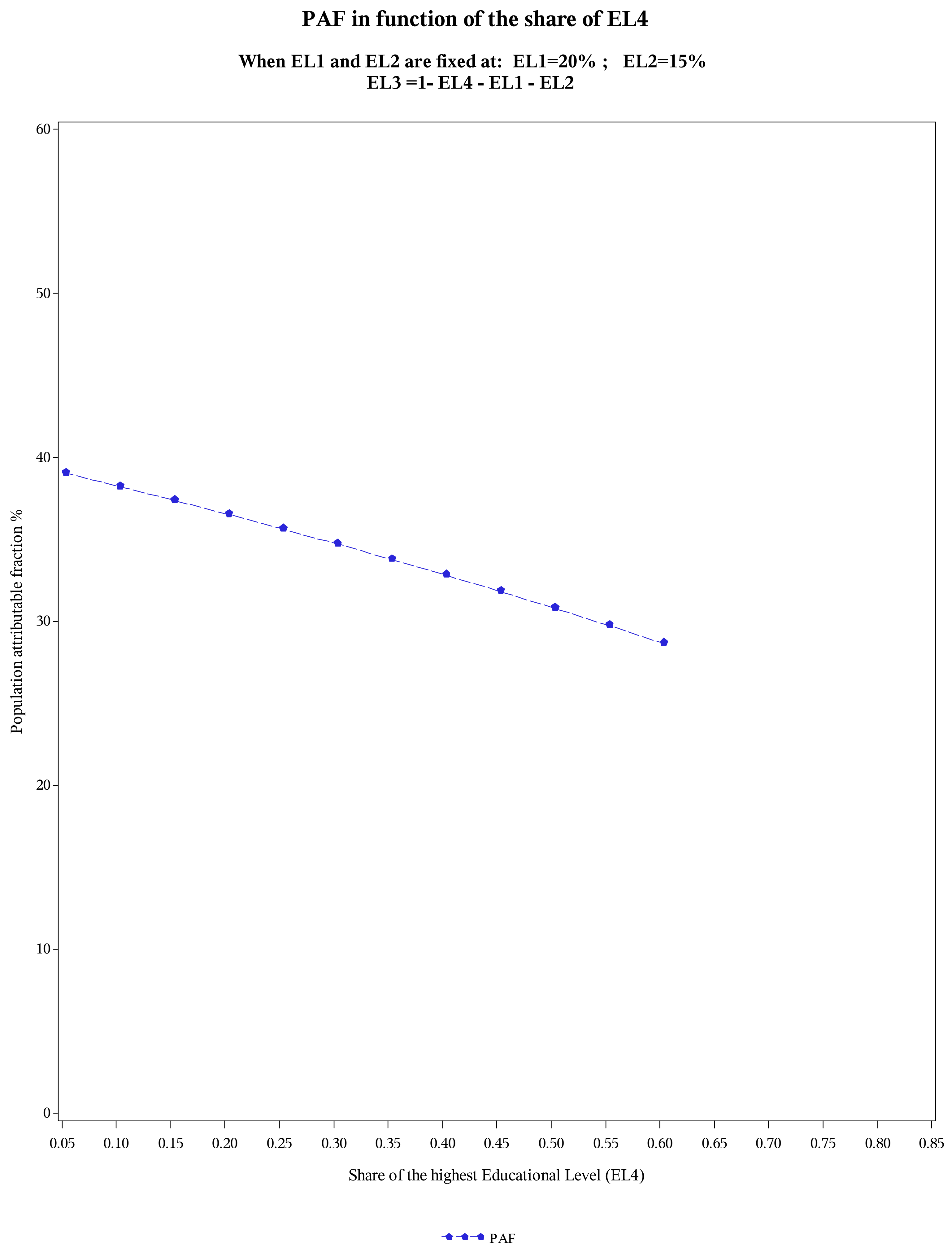

PAF in function of the share of EL4
When EL1 and EL2 are fixed at: EL1=20% ; EL2=15%
EL3 =1- EL4 - EL1 - EL2
Population attributable fraction %
0
10
20
30
40
50
60
0.05
0.10
0.15
0.20
0.25
0.30
0.35
0.40
0.45
0.50
0.55
0.60
0.65
0.70
0.75
0.80
0.85
Share of the highest Educational Level (EL4)
PAF

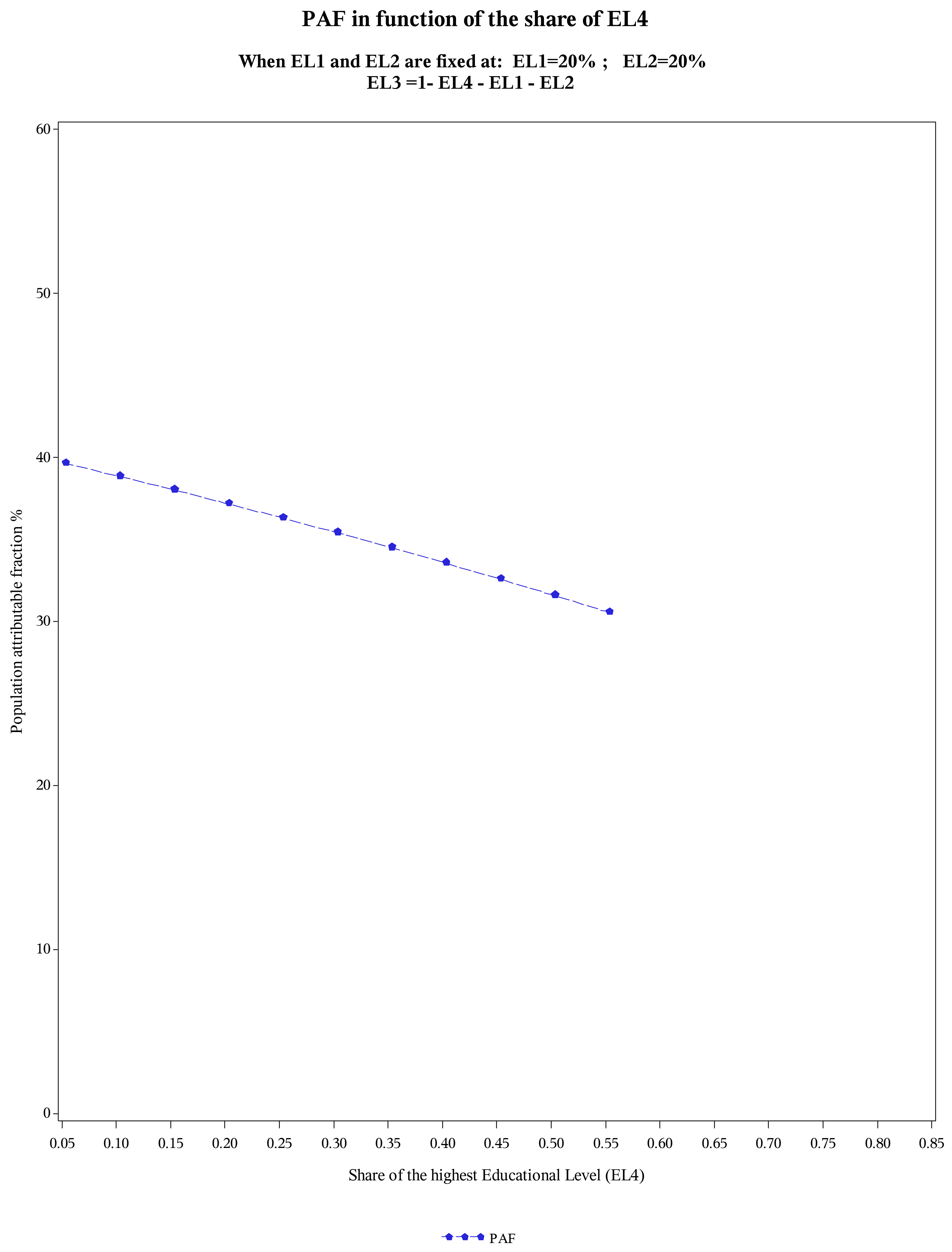
PAF in function of the share of EL4
When EL1 and EL2 are fixed at: EL1=20% ; EL2=20%
EL3 =1- EL4 - EL1 - EL2
Population attributable fraction %
0
10
20
30
40
50
60
0.05
0.10
0.15
0.20
0.25
0.30
0.35
0.40
0.45
0.50
0.55
0.60
0.65
0.70
0.75
0.80
0.85
Share of the highest Educational Level (EL4)
PAF

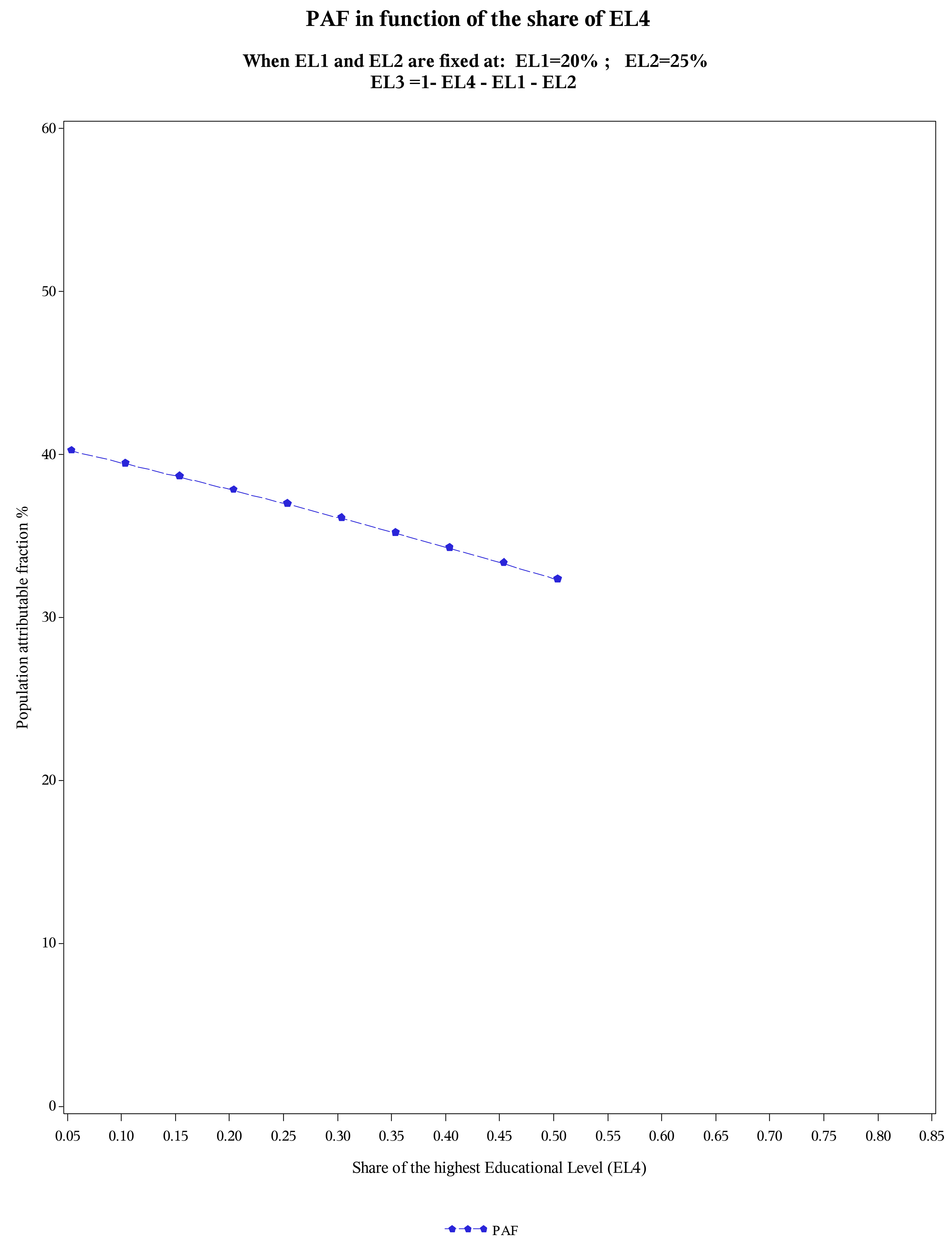
PAF in function of the share of EL4
When EL1 and EL2 are fixed at: EL1=20% ; EL2=25%
EL3 =1- EL4 - EL1 - EL2
Population attributable fraction %
0
10
20
30
40
50
60
0.05
0.10
0.15
0.20
0.25
0.30
0.35
0.40
0.45
0.50
0.55
0.60
0.65
0.70
0.75
0.80
0.85
Share of the highest Educational Level (EL4)
PAF

PAF in function of the share of EL4

When EL1 and EL2 are fixed at: EL1=20% ; EL2=30%
EL3 =1- EL4 - EL1 - EL2

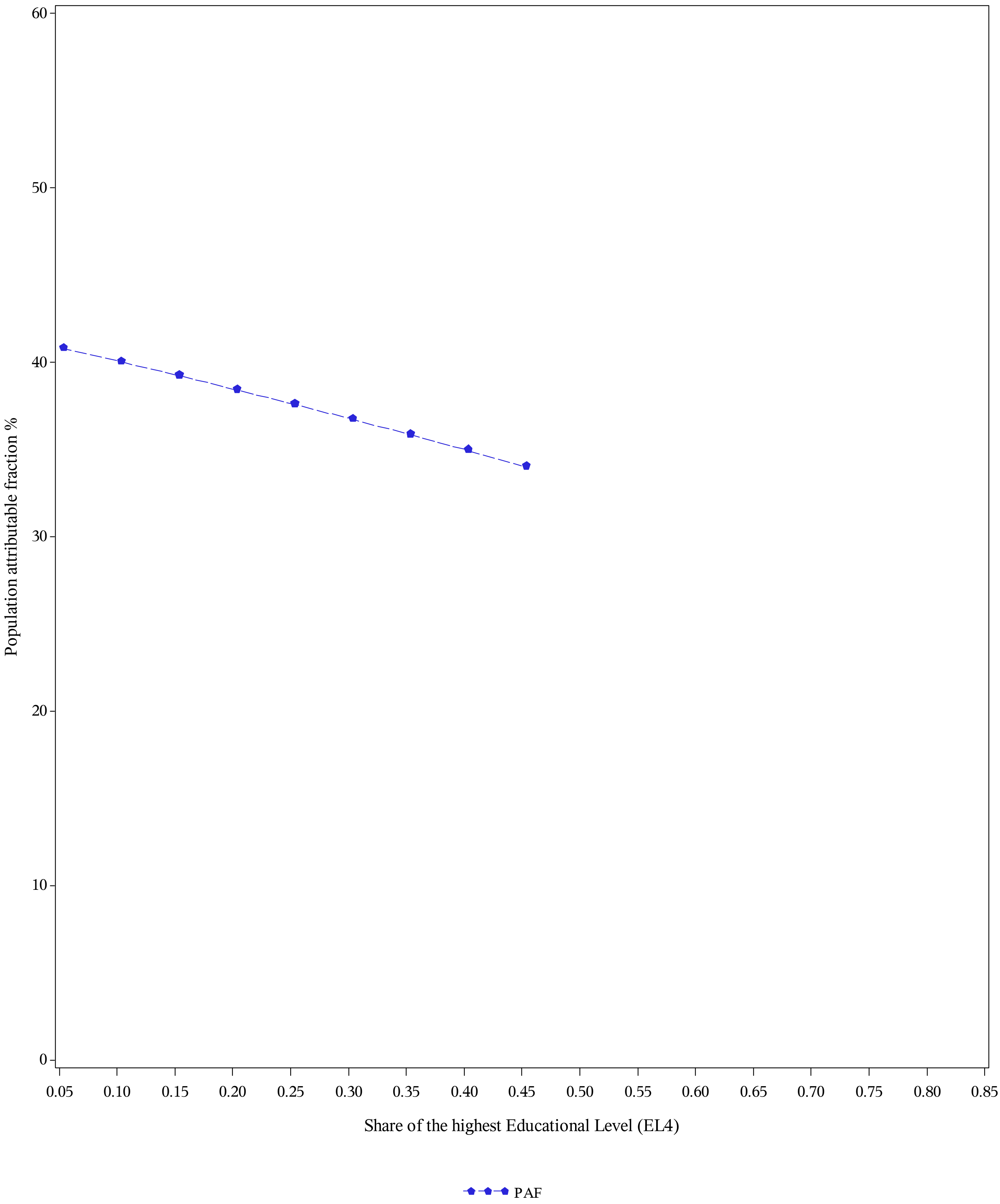

PAF in function of the share of EL4

When EL1 and EL2 are fixed at: EL1=20% ; EL2=35%
EL3 =1- EL4 - EL1 - EL2

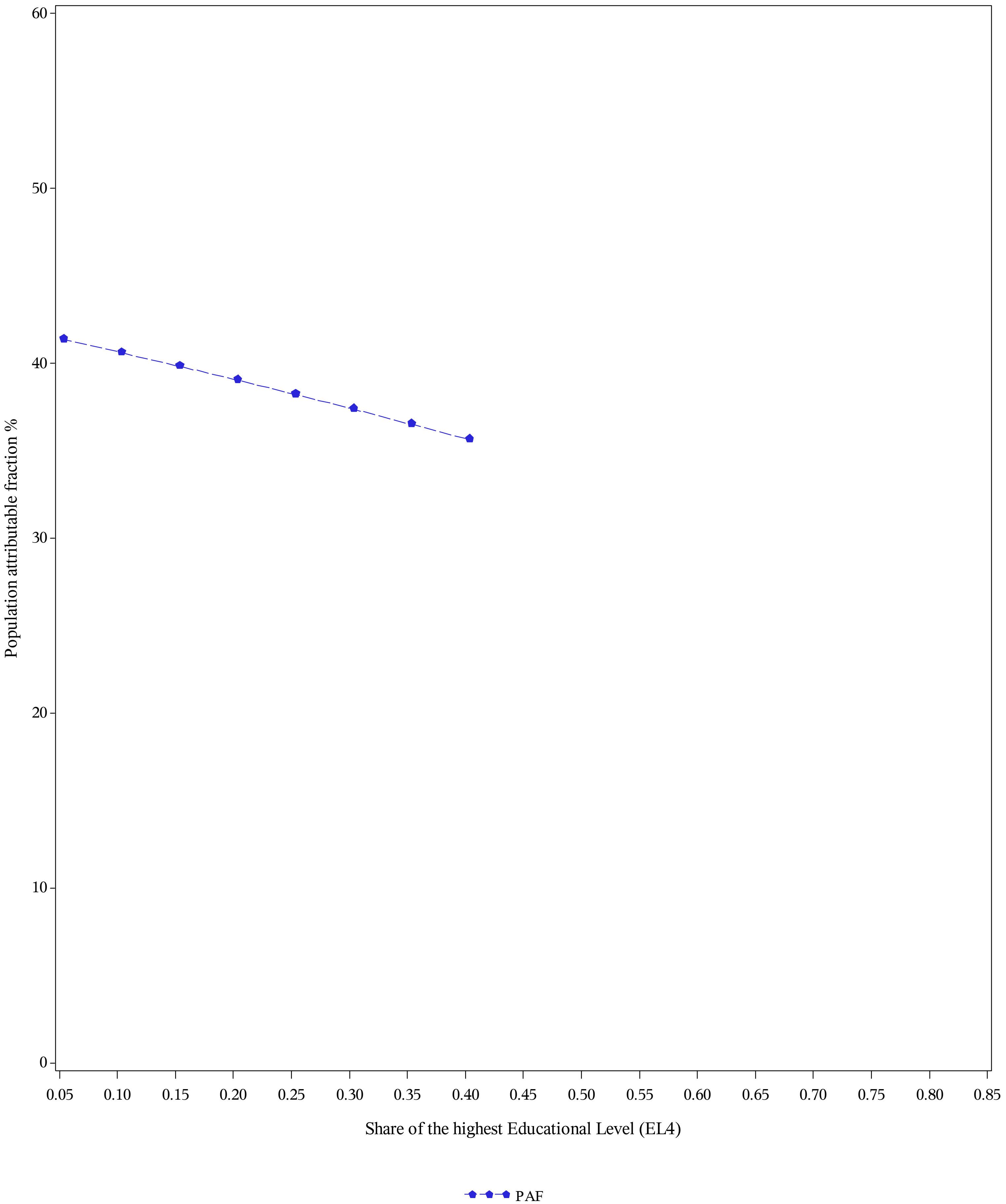

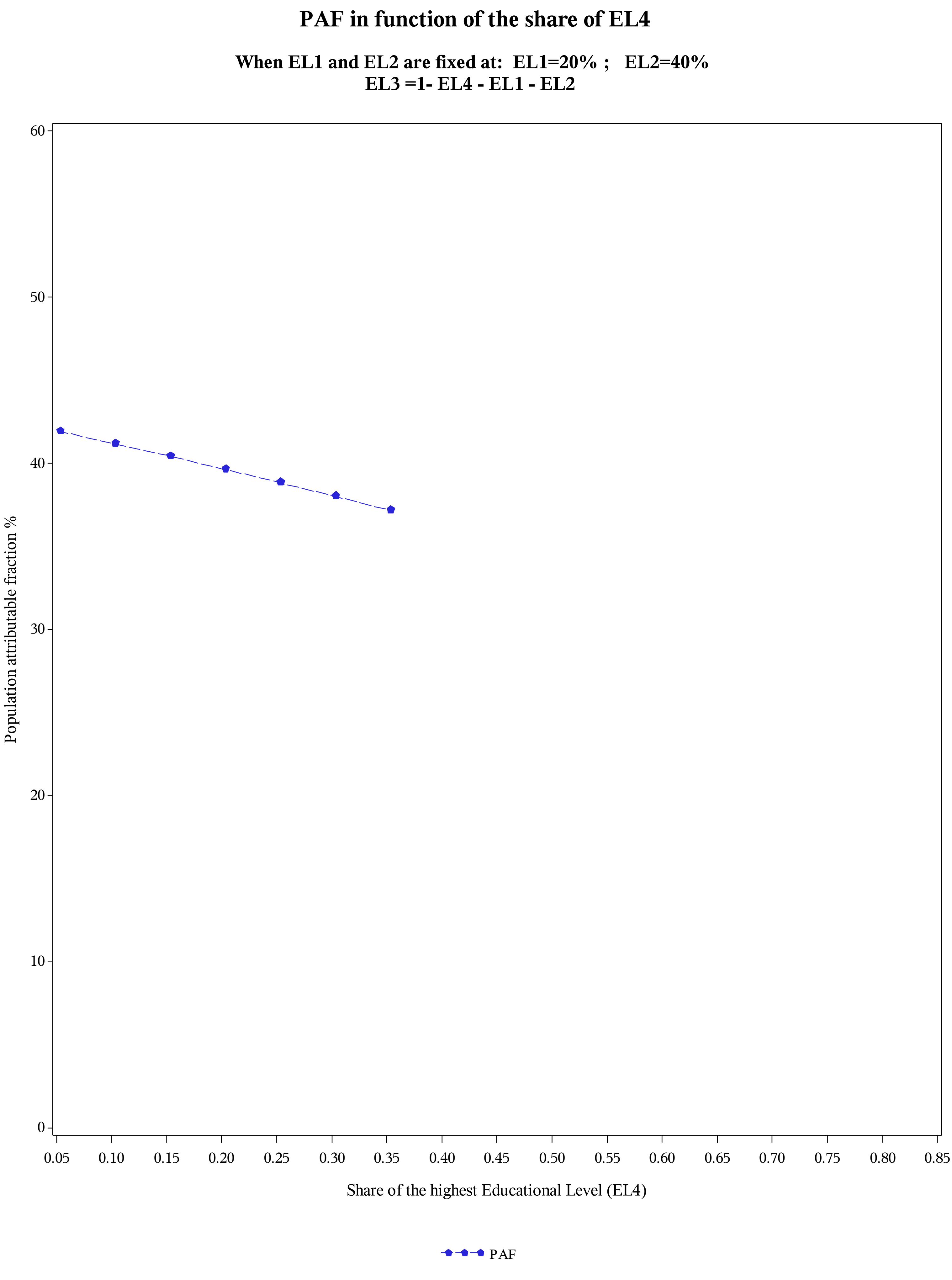
PAF in function of the share of EL4
When EL1 and EL2 are fixed at: EL1=20% ; EL2=40%
EL3 =1- EL4 - EL1 - EL2
Population attributable fraction %
0
10
20
30
40
50
60
0.05
0.10
0.15
0.20
0.25
0.30
0.35
0.40
0.45
0.50
0.55
0.60
0.65
0.70
0.75
0.80
0.85
Share of the highest Educational Level (EL4)
PAF

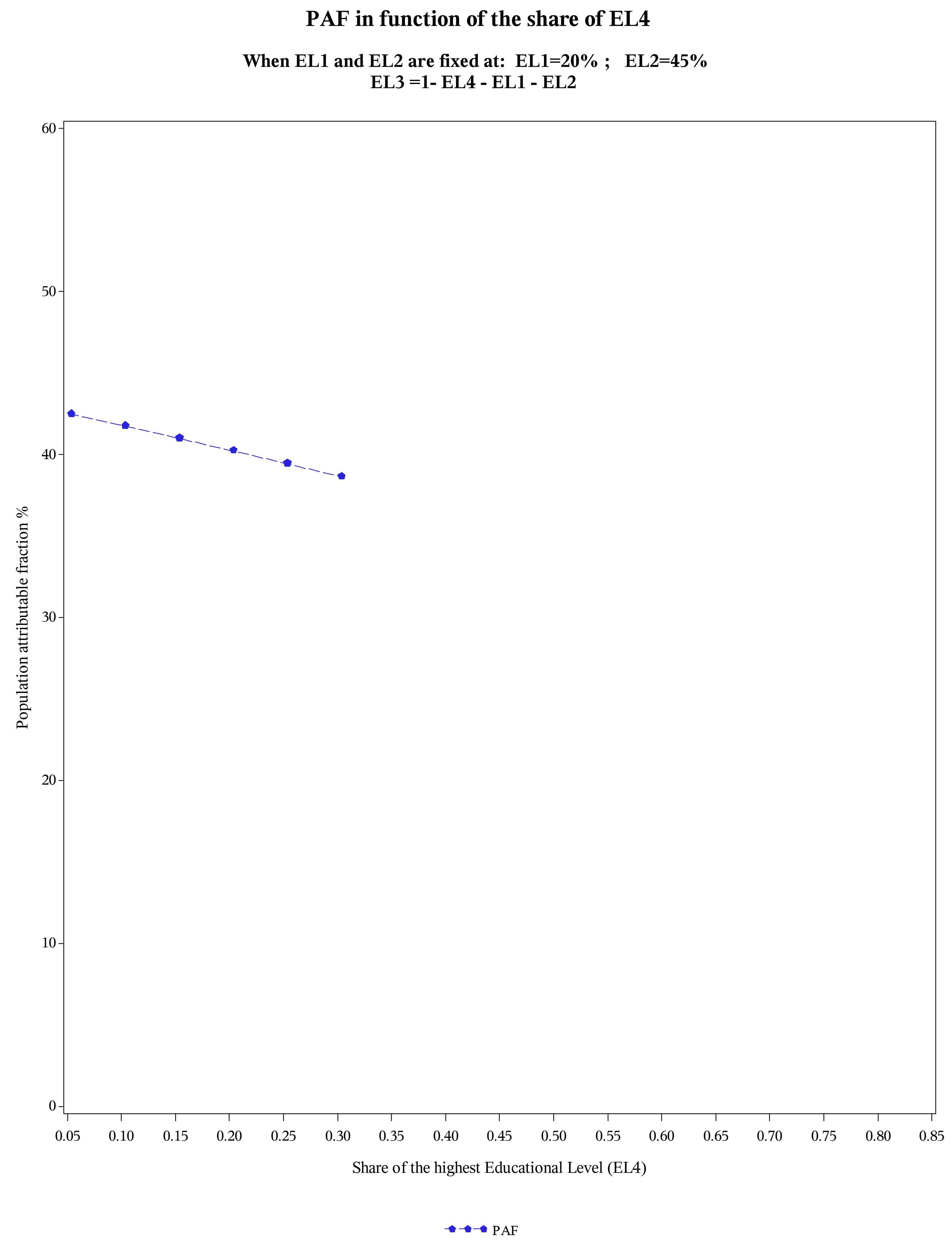
PAF in function of the share of EL4
When EL1 and EL2 are fixed at: EL1=20% ; EL2=45%
EL3 =1- EL4 - EL1 - EL2
Population attributable fraction %
0
10
20
30
40
50
60
0.05
0.10
0.15
0.20
0.25
0.30
0.35
0.40
0.45
0.50
0.55
0.60
0.65
0.70
0.75
0.80
0.85
Share of the highest Educational Level (EL4)
PAF

PAF in function of the share of EL4

When EL1 and EL2 are fixed at: EL1=20% ; EL2=50%
EL3 =1- EL4 - EL1 - EL2

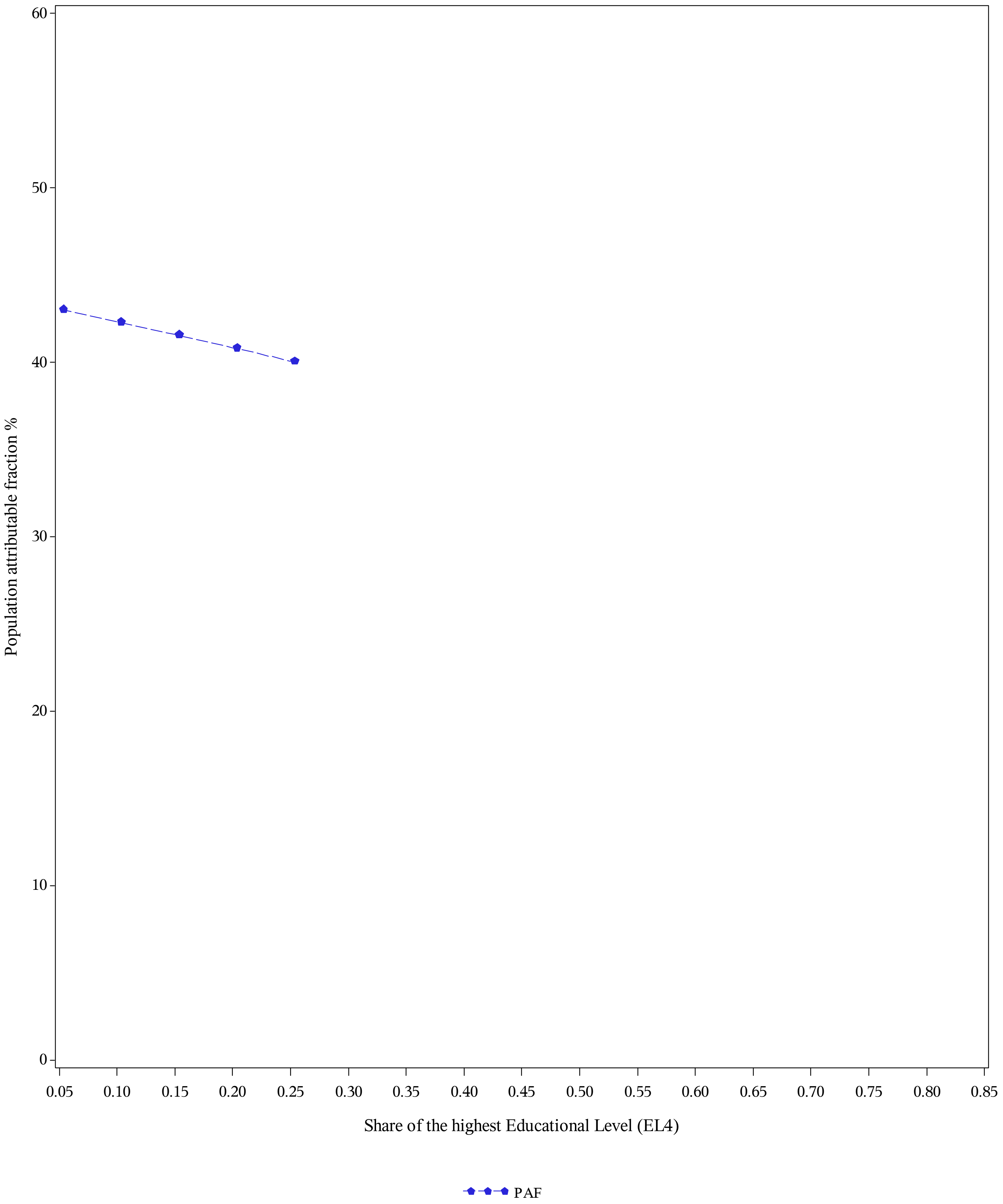

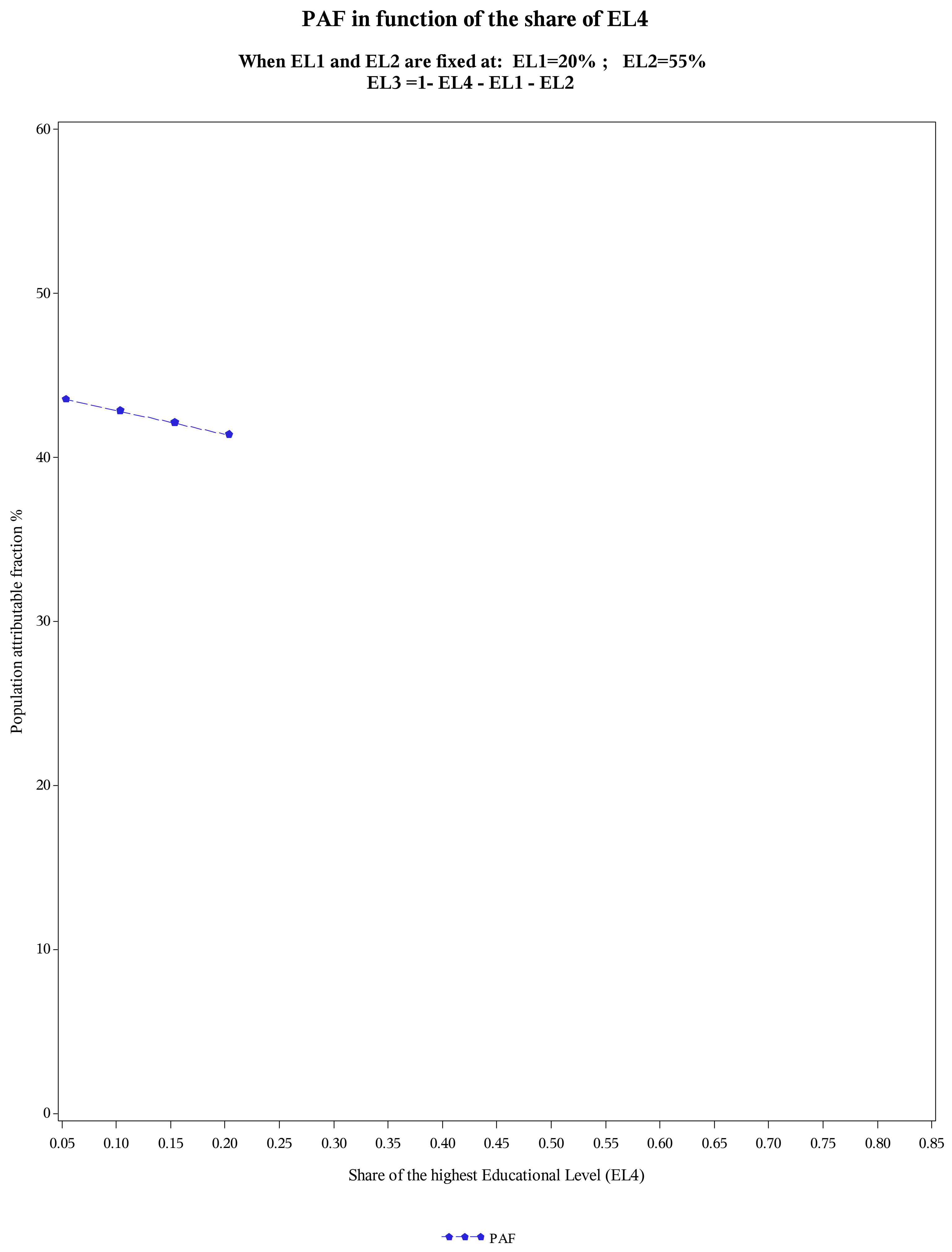
PAF in function of the share of EL4
When EL1 and EL2 are fixed at: EL1=20% ; EL2=55%
EL3 =1- EL4 - EL1 - EL2
Population attributable fraction %
0
10
20
30
40
50
60
0.05
0.10
0.15
0.20
0.25
0.30
0.35
0.40
0.45
0.50
0.55
0.60
0.65
0.70
0.75
0.80
0.85
Share of the highest Educational Level (EL4)
PAF

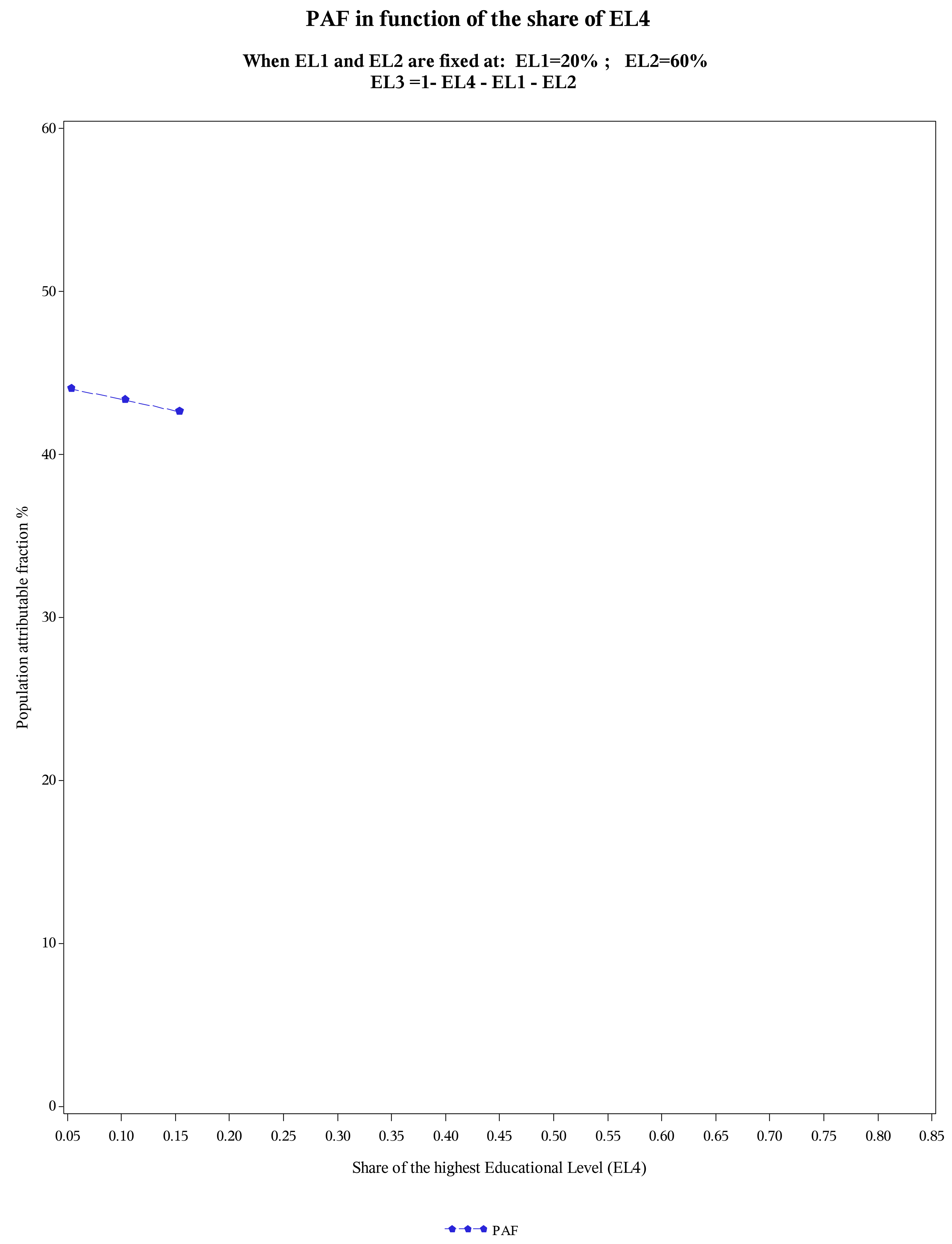
PAF in function of the share of EL4
When EL1 and EL2 are fixed at: EL1=20% ; EL2=60%
EL3 =1- EL4 - EL1 - EL2
Population attributable fraction %
0
10
20
30
40
50
60
0.05
0.10
0.15
0.20
0.25
0.30
0.35
0.40
0.45
0.50
0.55
0.60
0.65
0.70
0.75
0.80
0.85
Share of the highest Educational Level (EL4)
PAF

PAF in function of the share of EL4

When EL1 and EL2 are fixed at: EL1=20% ; EL2=65%
EL3 =1- EL4 - EL1 - EL2

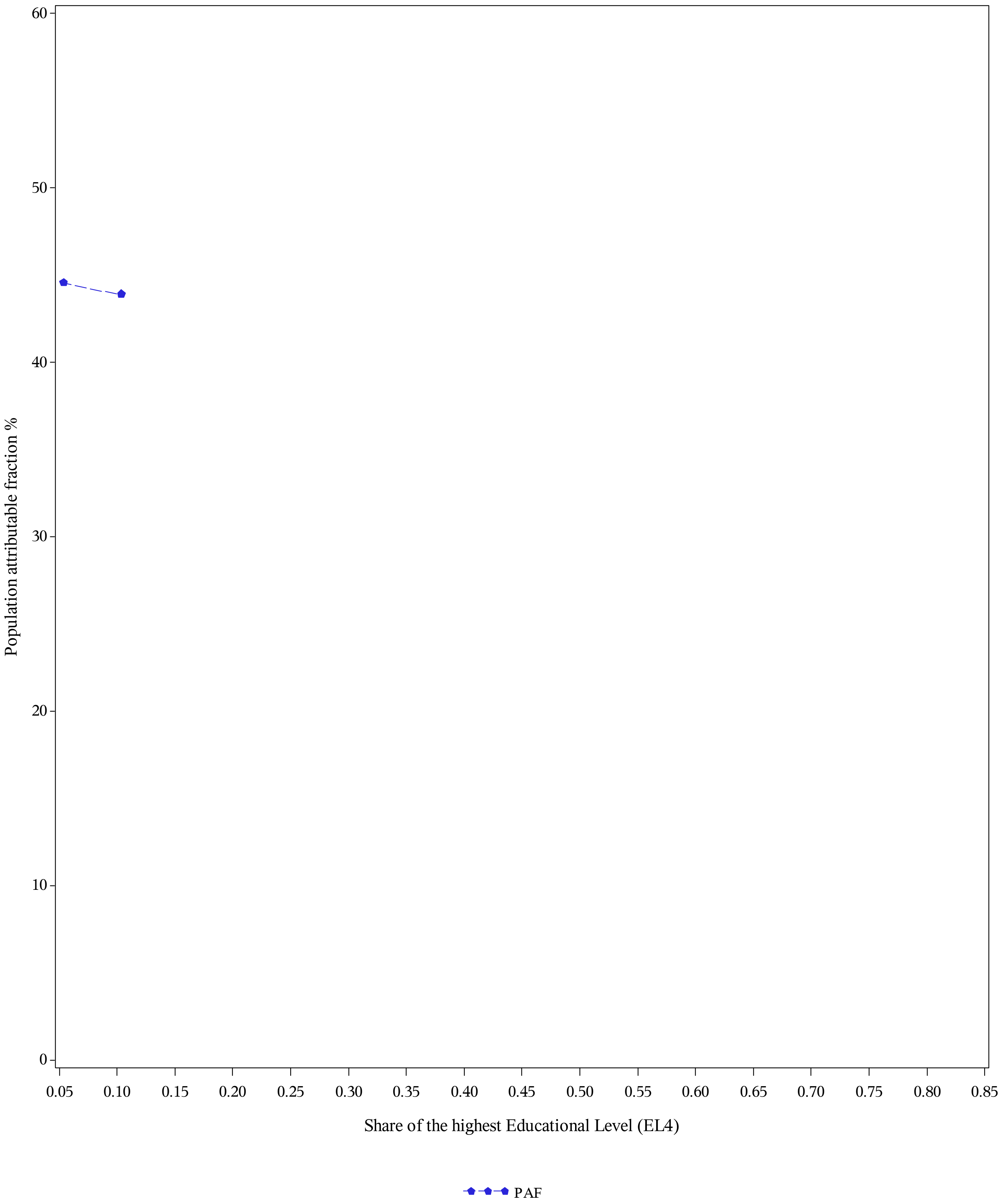

PAF in function of the share of EL4

When EL1 and EL2 are fixed at: EL1=20% ; EL2=70%
EL3 =1- EL4 - EL1 - EL2

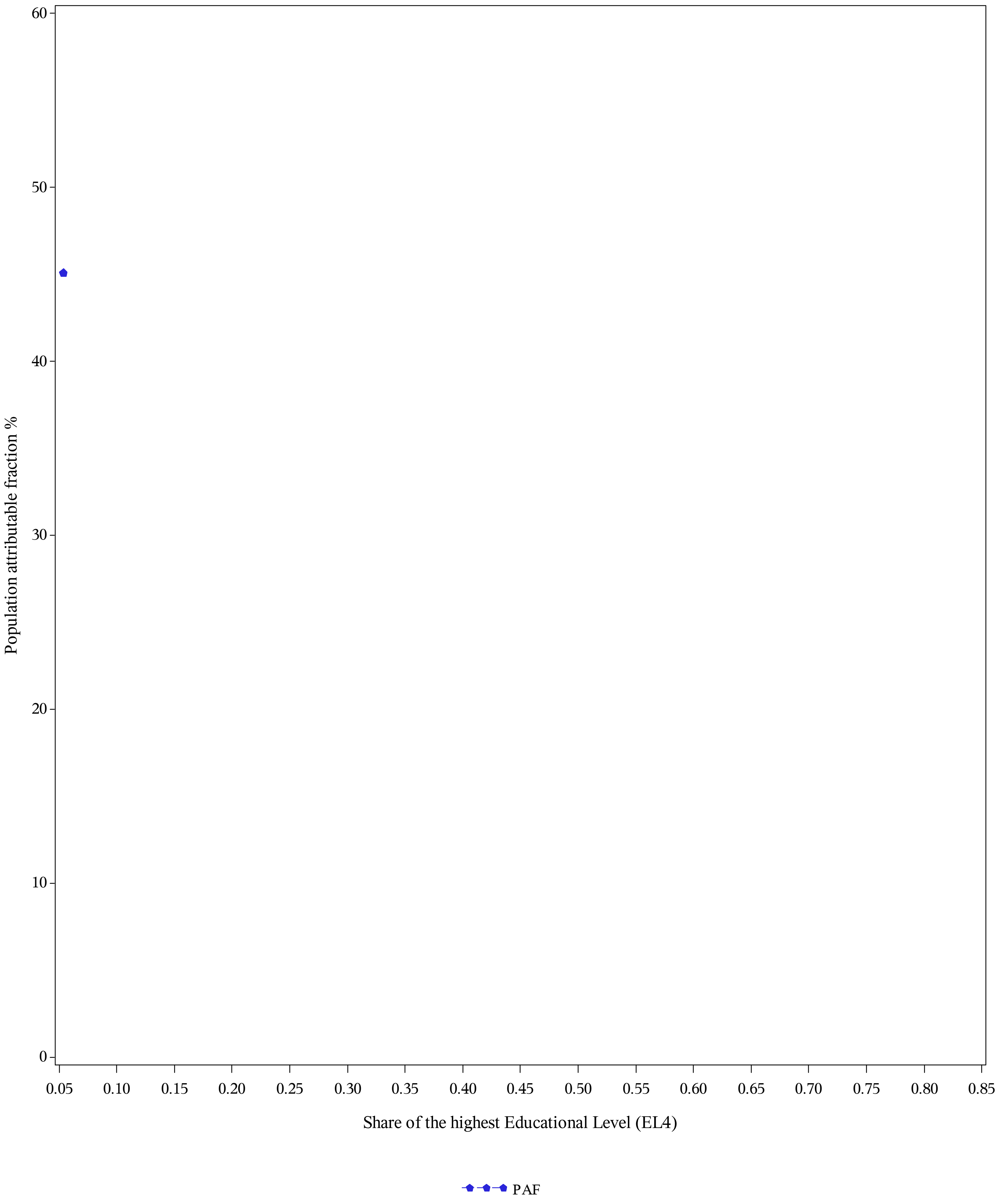

PAF in function of the share of EL4

When EL1 and EL2 are fixed at: EL1=25% ; EL2=5%
EL3 =1- EL4 - EL1 - EL2

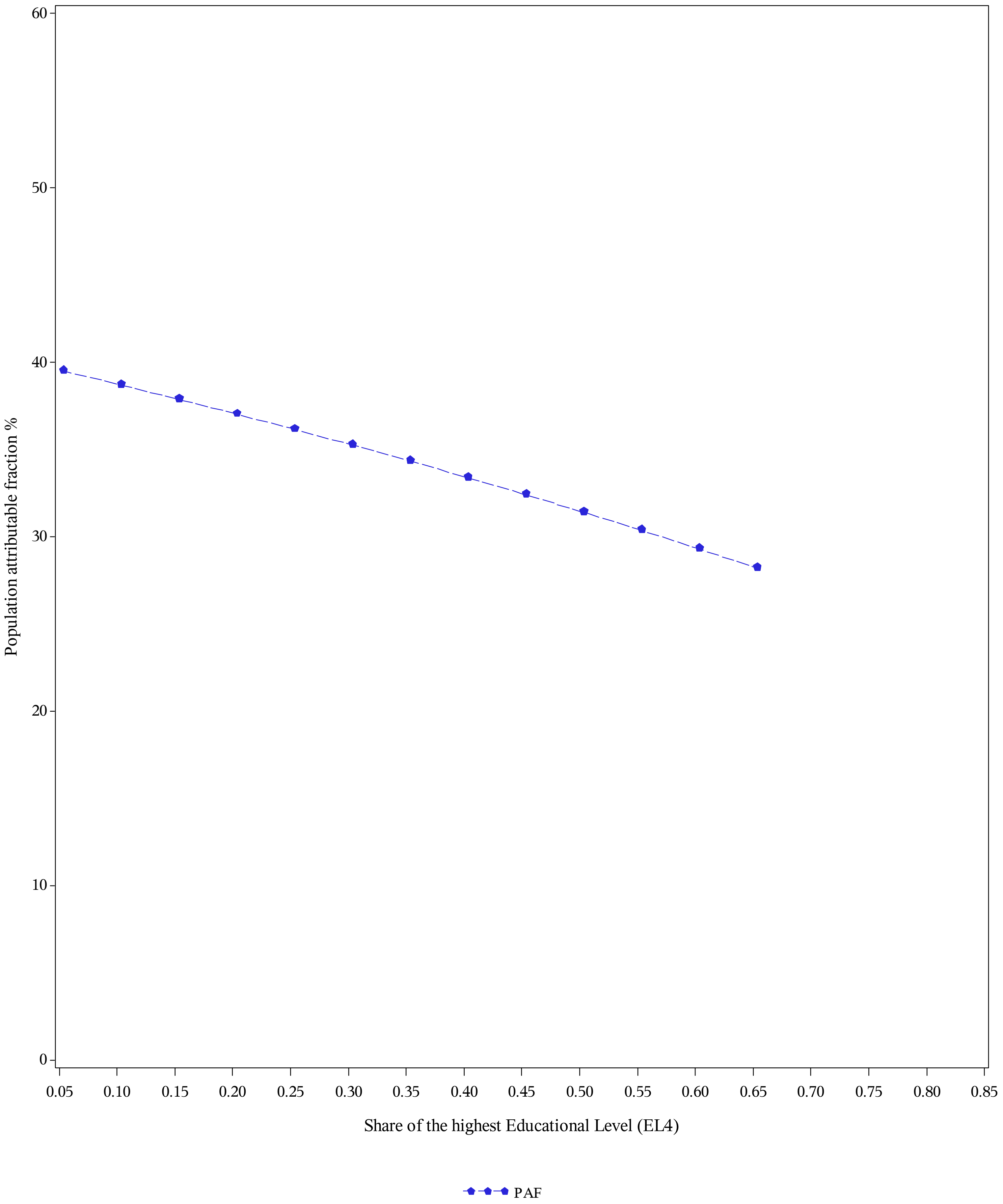

PAF in function of the share of EL4

When EL1 and EL2 are fixed at: EL1=25% ; EL2=10%
EL3 =1- EL4 - EL1 - EL2

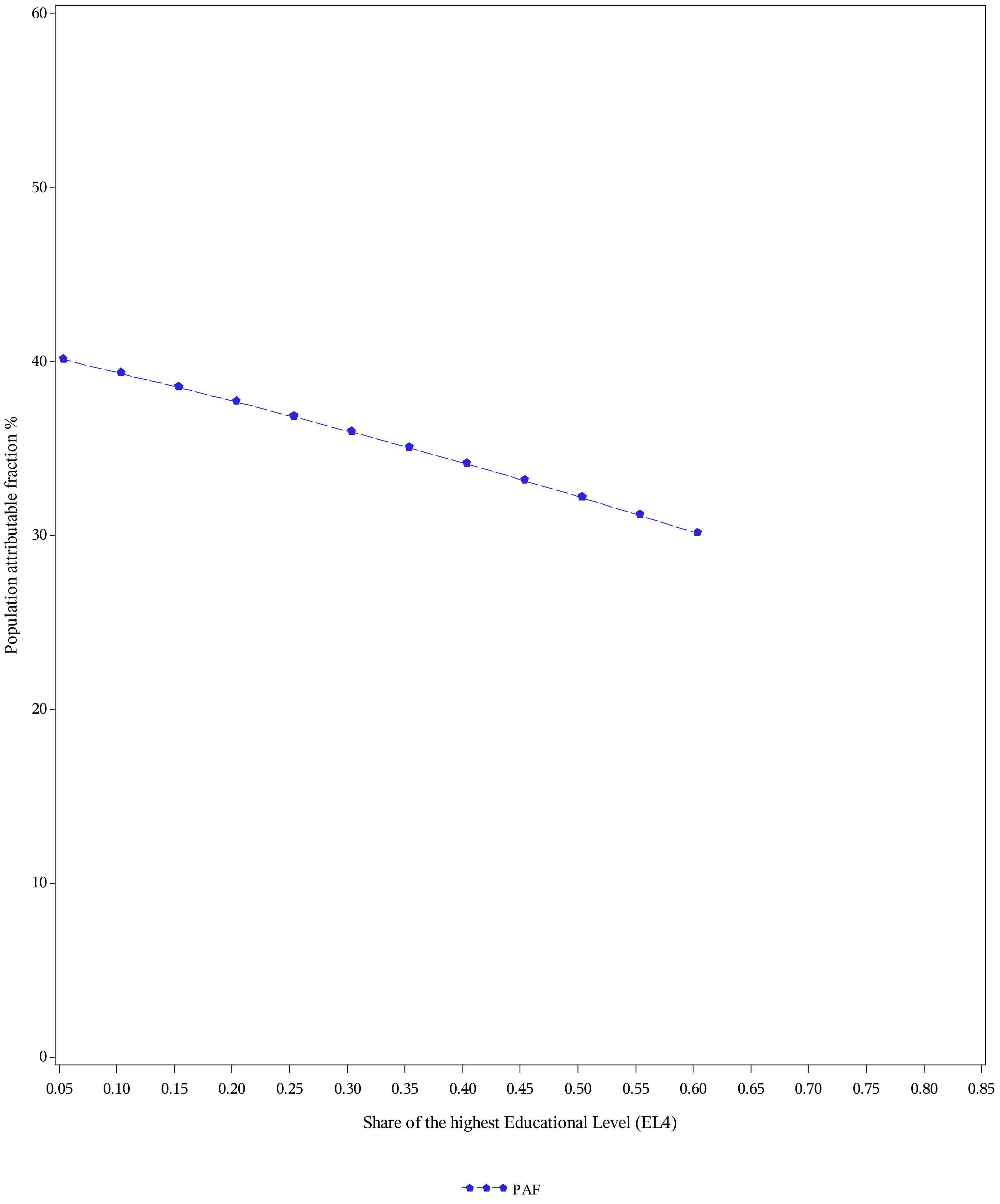

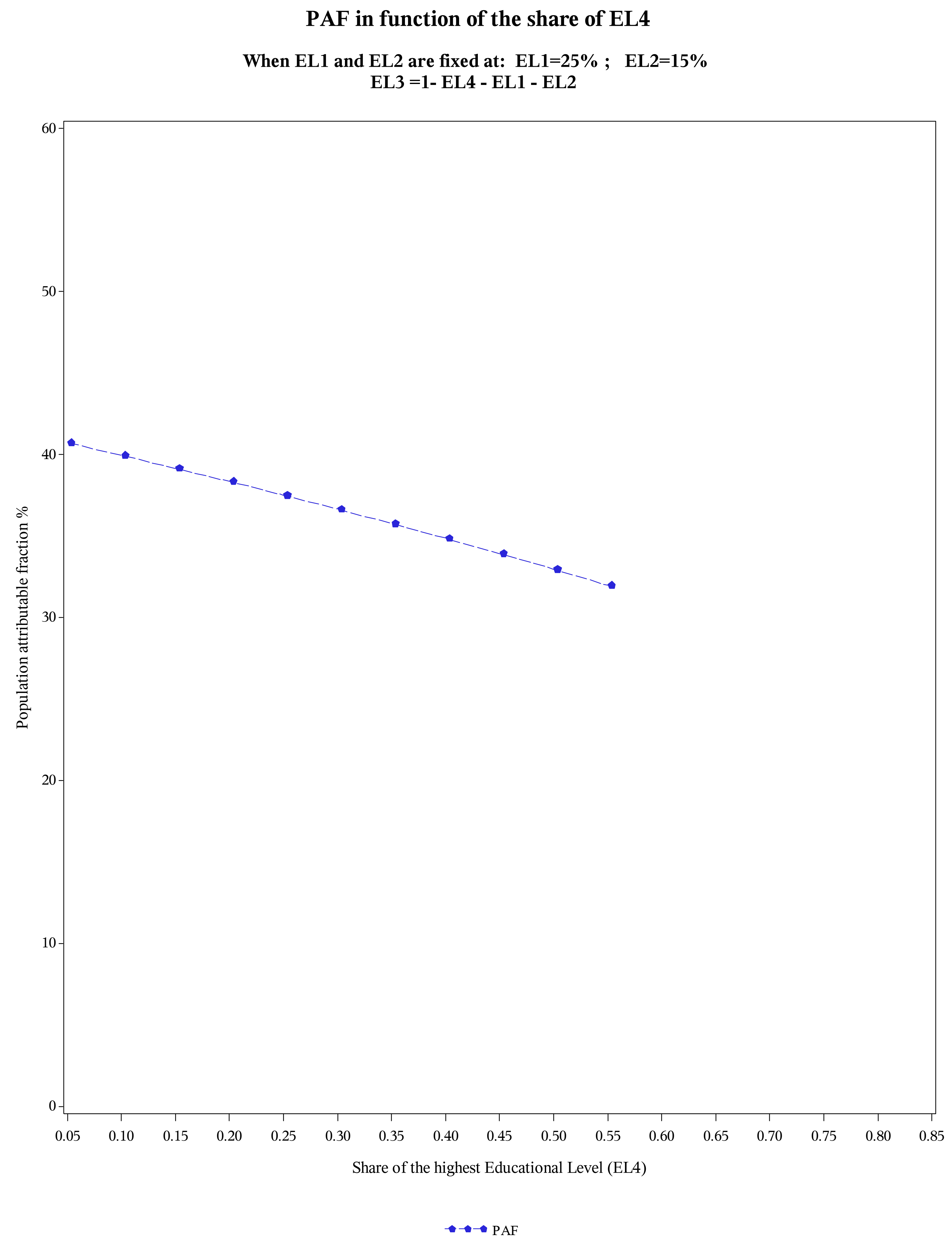

PAF in function of the share of EL4
When EL1 and EL2 are fixed at: EL1=25% ; EL2=15%
EL3 =1- EL4 - EL1 - EL2
Population attributable fraction %
0
10
20
30
40
50
60
0.05
0.10
0.15
0.20
0.25
0.30
0.35
0.40
0.45
0.50
0.55
0.60
0.65
0.70
0.75
0.80
0.85
Share of the highest Educational Level (EL4)
PAF

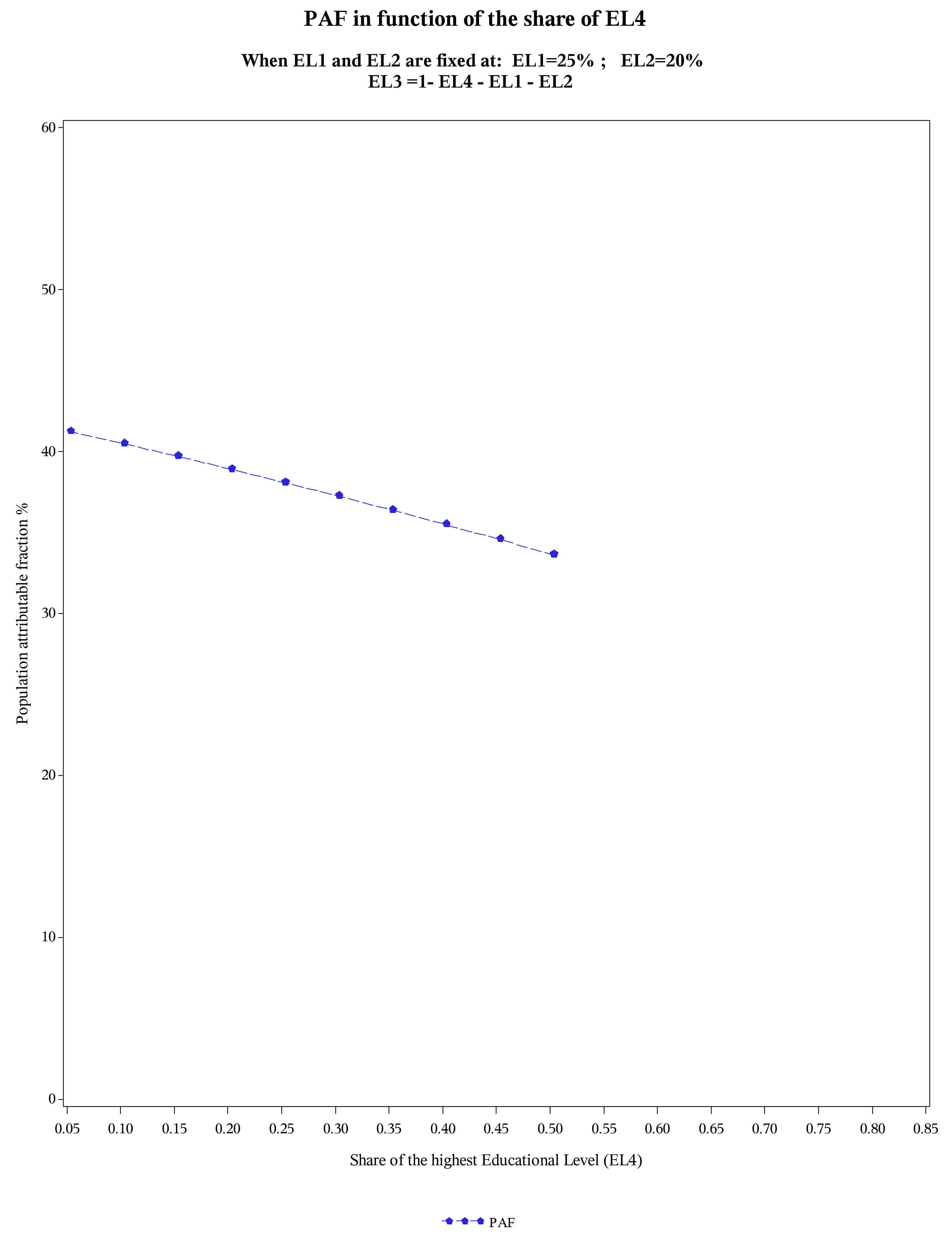

PAF in function of the share of EL4
When EL1 and EL2 are fixed at: EL1=25% ; EL2=20%
EL3 =1- EL4 - EL1 - EL2
Population attributable fraction %
0
10
20
30
40
50
60
0.05
0.10
0.15
0.20
0.25
0.30
0.35
0.40
0.45
0.50
0.55
0.60
0.65
0.70
0.75
0.80
0.85
Share of the highest Educational Level (EL4)
PAF

PAF in function of the share of EL4

When EL1 and EL2 are fixed at: EL1=25% ; EL2=25%
EL3 =1- EL4 - EL1 - EL2

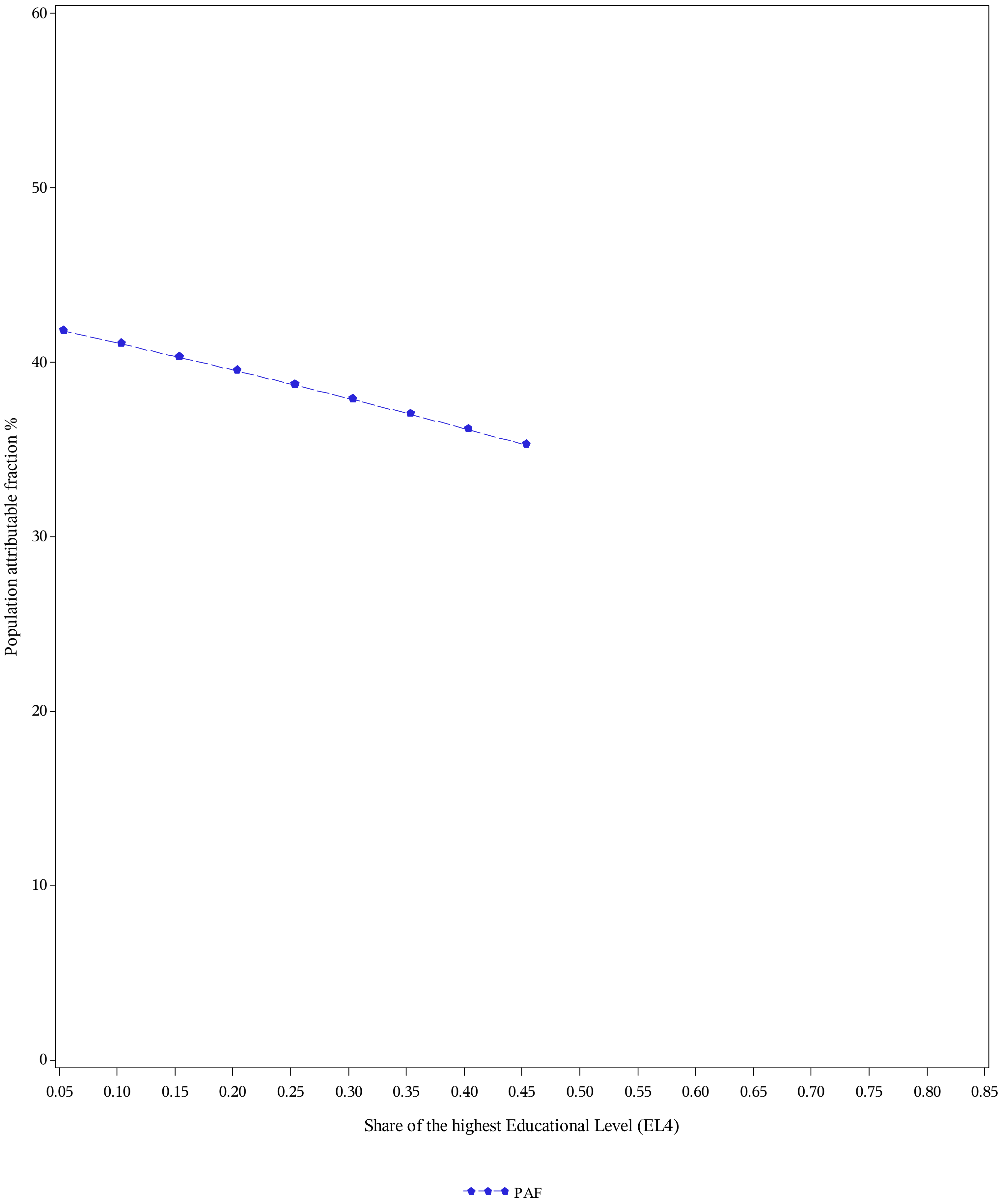

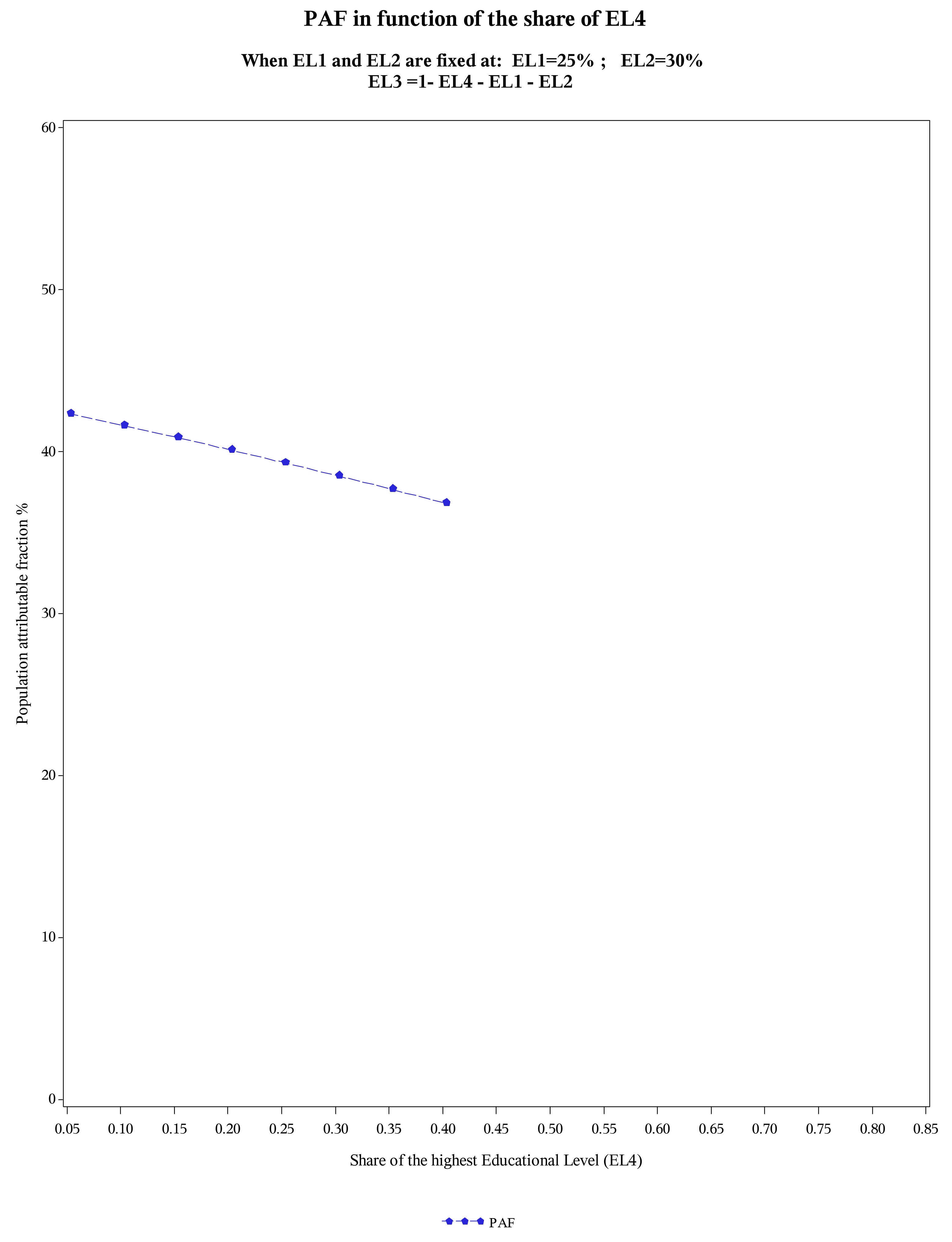
PAF in function of the share of EL4
When EL1 and EL2 are fixed at: EL1=25% ; EL2=30%
EL3 =1- EL4 - EL1 - EL2
Population attributable fraction %
0
10
20
30
40
50
60
0.05
0.10
0.15
0.20
0.25
0.30
0.35
0.40
0.45
0.50
0.55
0.60
0.65
0.70
0.75
0.80
0.85
Share of the highest Educational Level (EL4)
PAF

PAF in function of the share of EL4

When EL1 and EL2 are fixed at: EL1=25% ; EL2=35%
EL3 =1- EL4 - EL1 - EL2

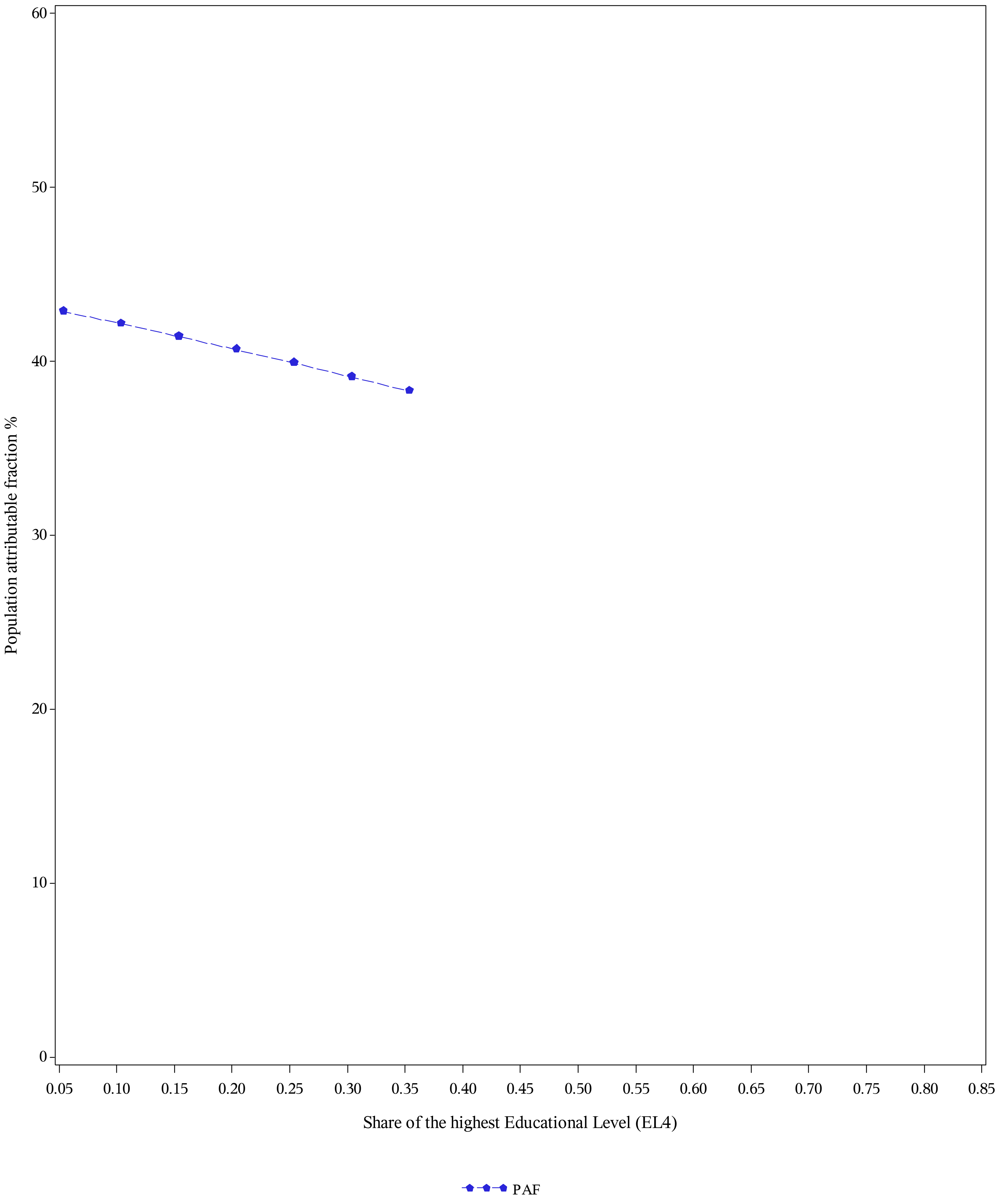

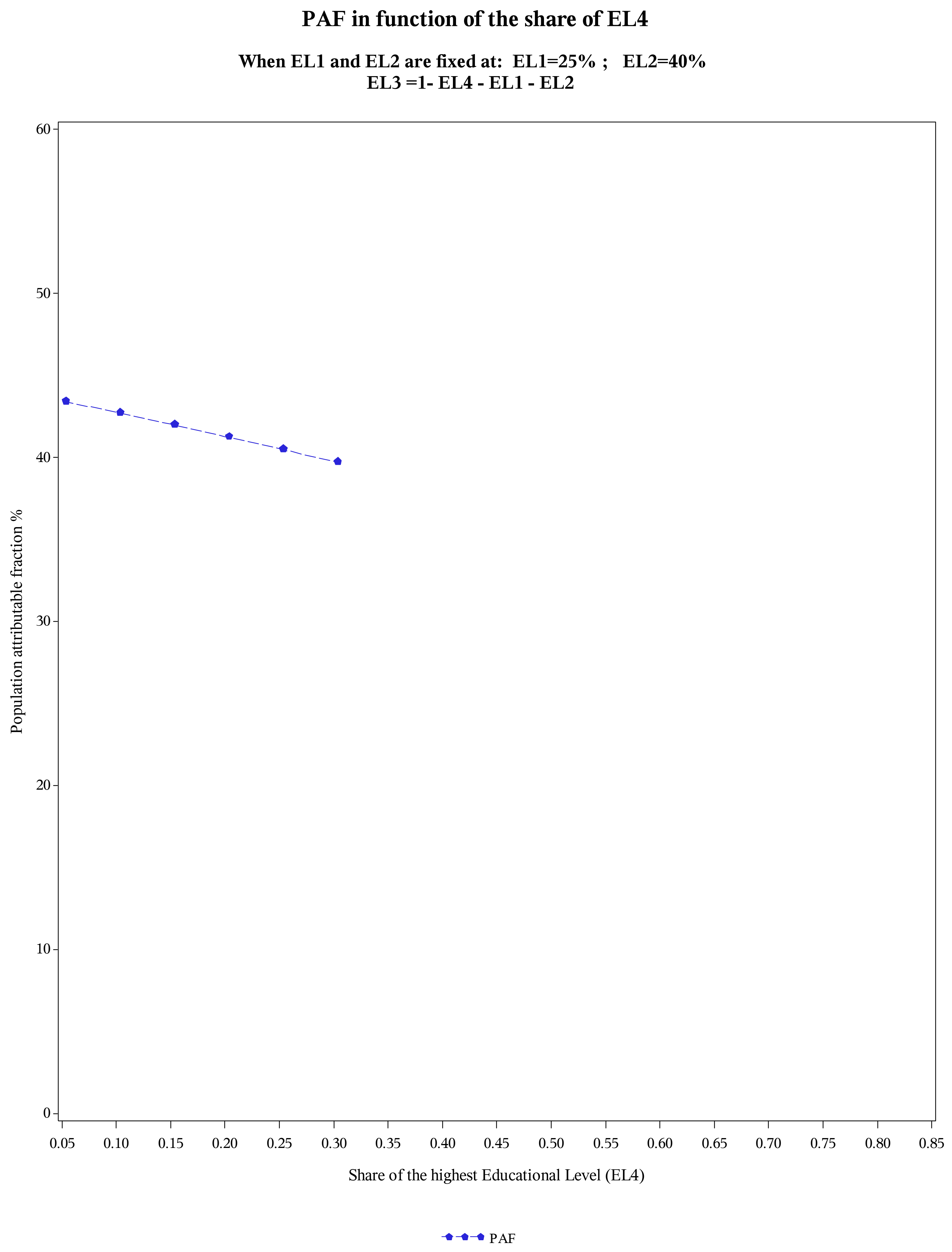
PAF in function of the share of EL4
When EL1 and EL2 are fixed at: EL1=25% ; EL2=40%
EL3 =1- EL4 - EL1 - EL2
Population attributable fraction %
0
10
20
30
40
50
60
0.05
0.10
0.15
0.20
0.25
0.30
0.35
0.40
0.45
0.50
0.55
0.60
0.65
0.70
0.75
0.80
0.85
Share of the highest Educational Level (EL4)
PAF

PAF in function of the share of EL4

When EL1 and EL2 are fixed at: EL1=25% ; EL2=45%
EL3 =1- EL4 - EL1 - EL2

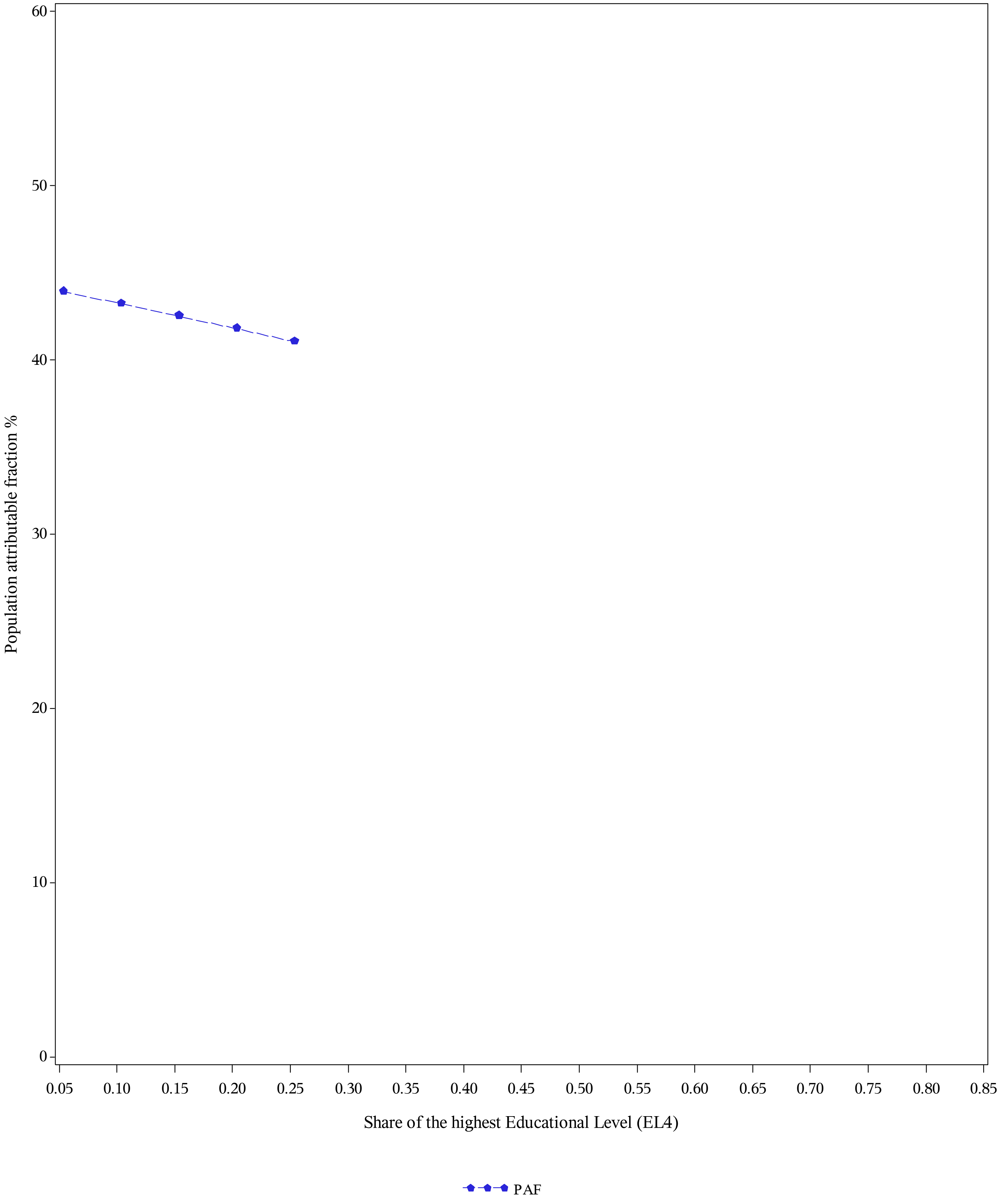

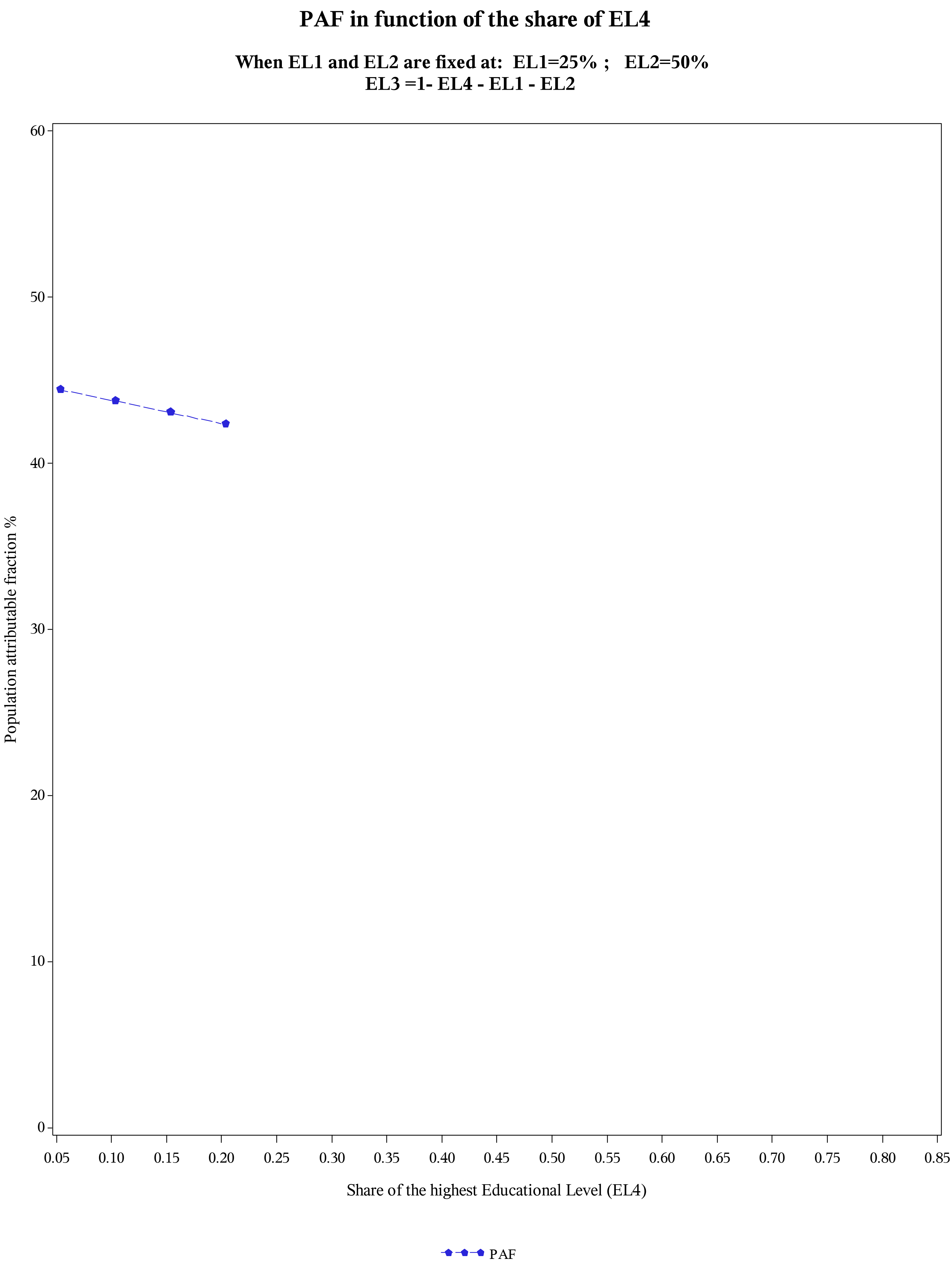
PAF in function of the share of EL4
When EL1 and EL2 are fixed at: EL1=25% ; EL2=50%
EL3 =1- EL4 - EL1 - EL2
Population attributable fraction %
0
10
20
30
40
50
60
0.05
0.10
0.15
0.20
0.25
0.30
0.35
0.40
0.45
0.50
0.55
0.60
0.65
0.70
0.75
0.80
0.85
Share of the highest Educational Level (EL4)
PAF

PAF in function of the share of EL4

When EL1 and EL2 are fixed at: EL1=25% ; EL2=55%
EL3 =1- EL4 - EL1 - EL2

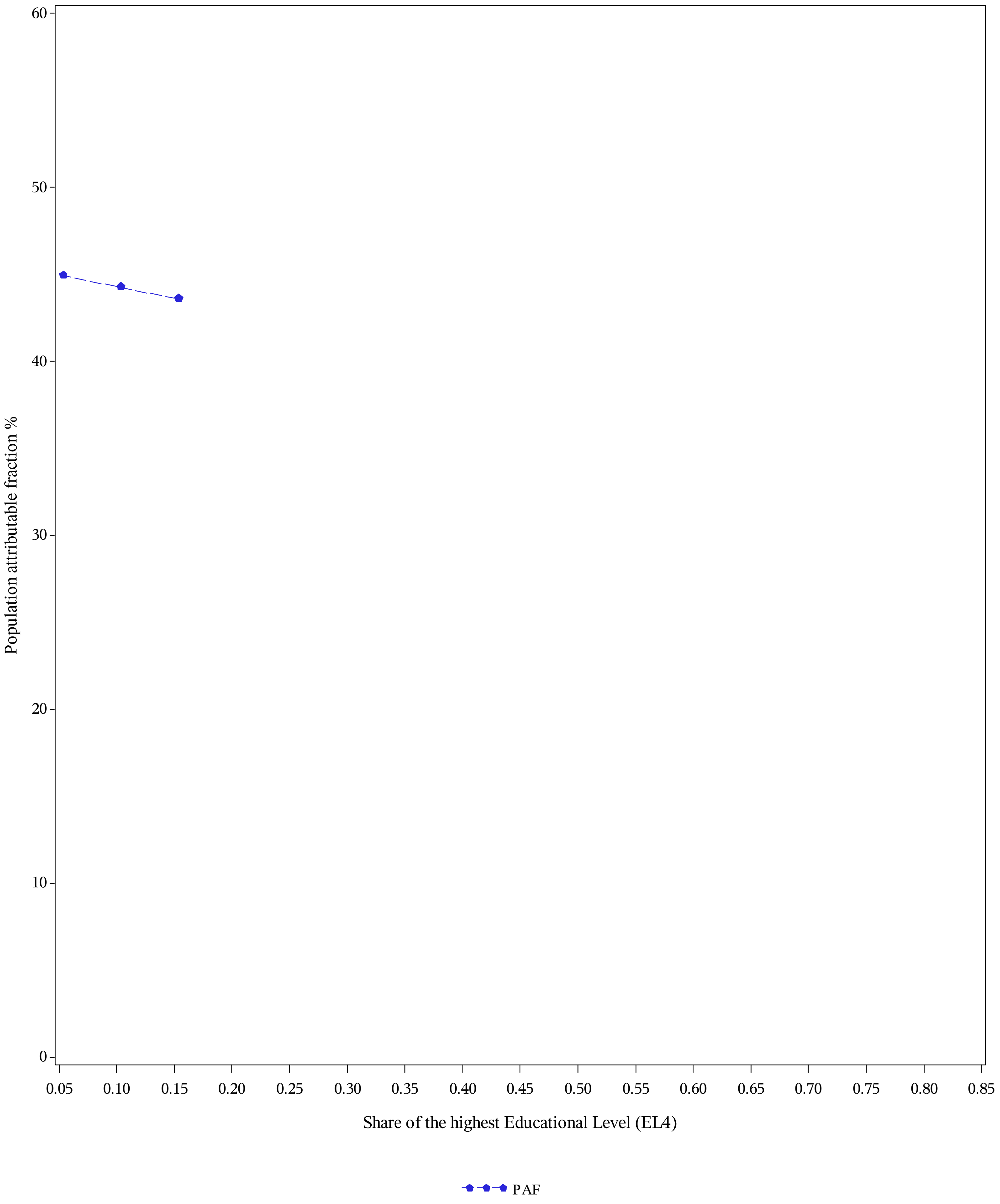

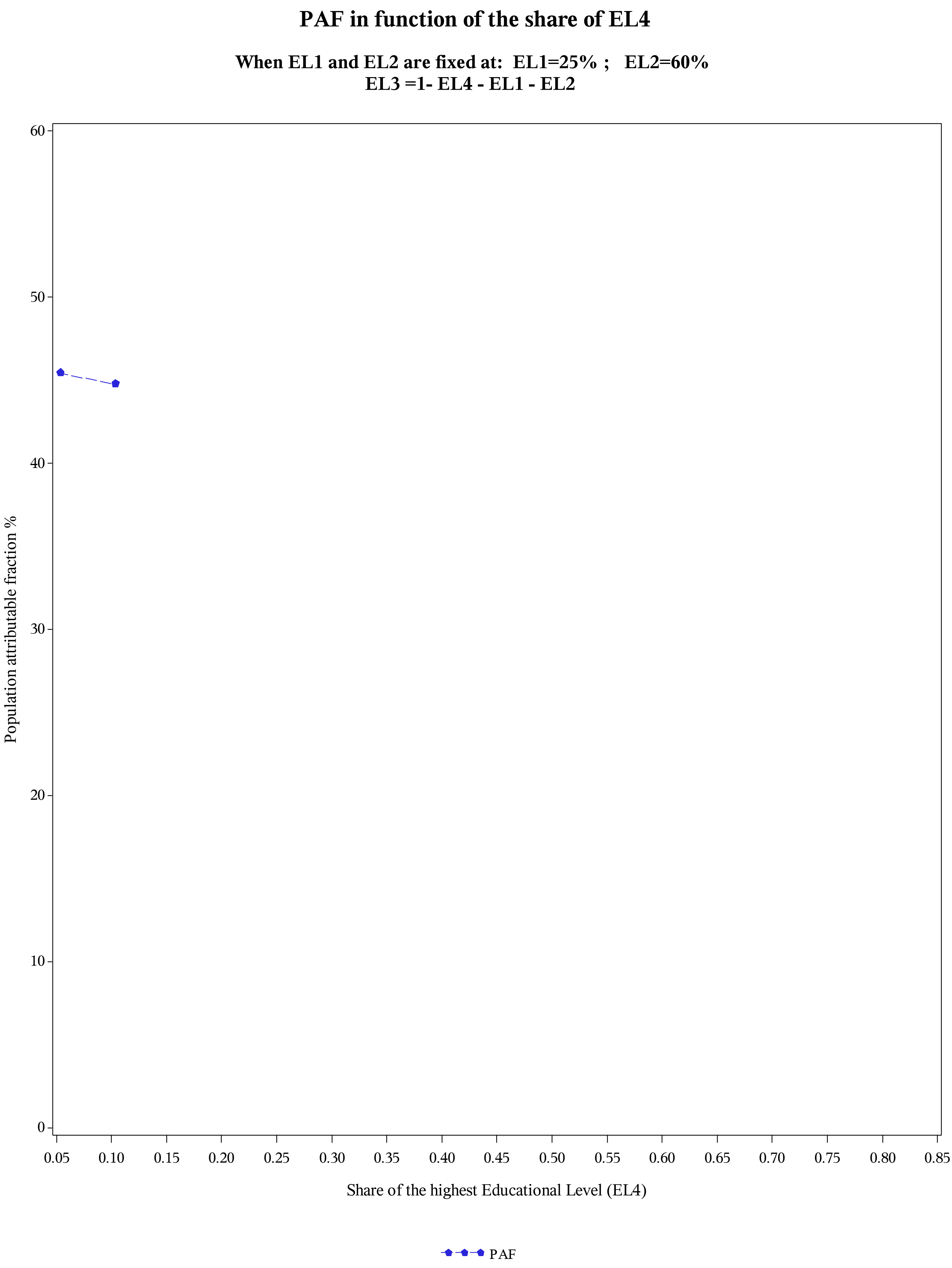
PAF in function of the share of EL4
When EL1 and EL2 are fixed at: EL1=25% ; EL2=60%
EL3 =1- EL4 - EL1 - EL2
Population attributable fraction %
0
10
20
30
40
50
60
0.05
0.10
0.15
0.20
0.25
0.30
0.35
0.40
0.45
0.50
0.55
0.60
0.65
0.70
0.75
0.80
0.85
Share of the highest Educational Level (EL4)
PAF

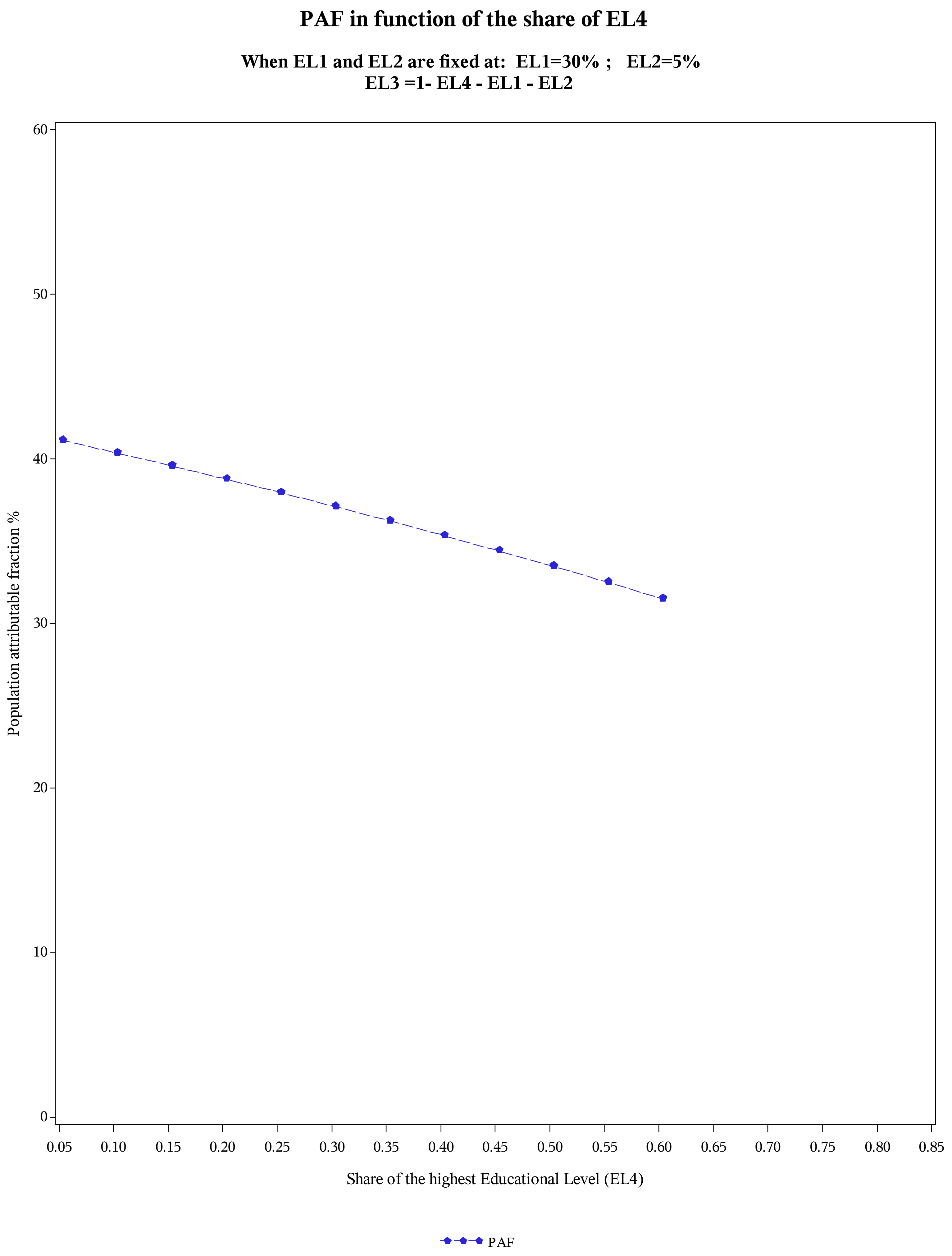
PAF in function of the share of EL4
When EL1 and EL2 are fixed at: EL1=30% ; EL2=5%
EL3 =1- EL4 - EL1 - EL2
60
50
40
30
20
10
0
Population attributable fraction %
0.05
0.10
0.15
0.20
0.25
0.30
0.35
0.40
0.45
0.50
0.55
0.60
0.65
0.70
0.75
0.80
0.85
Share of the highest Educational Level (EL4)
PAF

PAF in function of the share of EL4

When EL1 and EL2 are fixed at: EL1=30% ; EL2=10%
EL3 =1- EL4 - EL1 - EL2

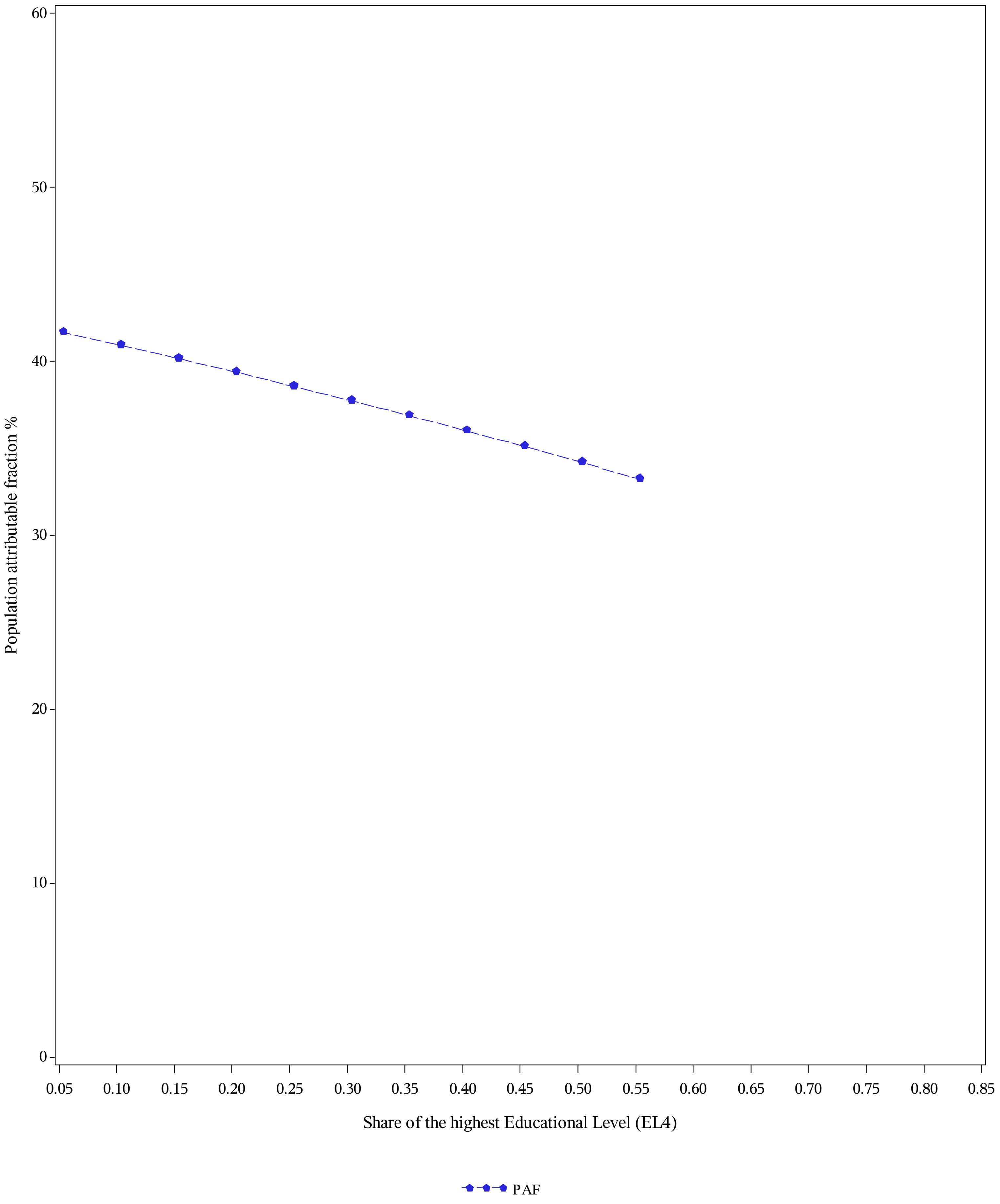

PAF in function of the share of EL4

When EL1 and EL2 are fixed at: EL1=30% ; EL2=15%
EL3 =1- EL4 - EL1 - EL2

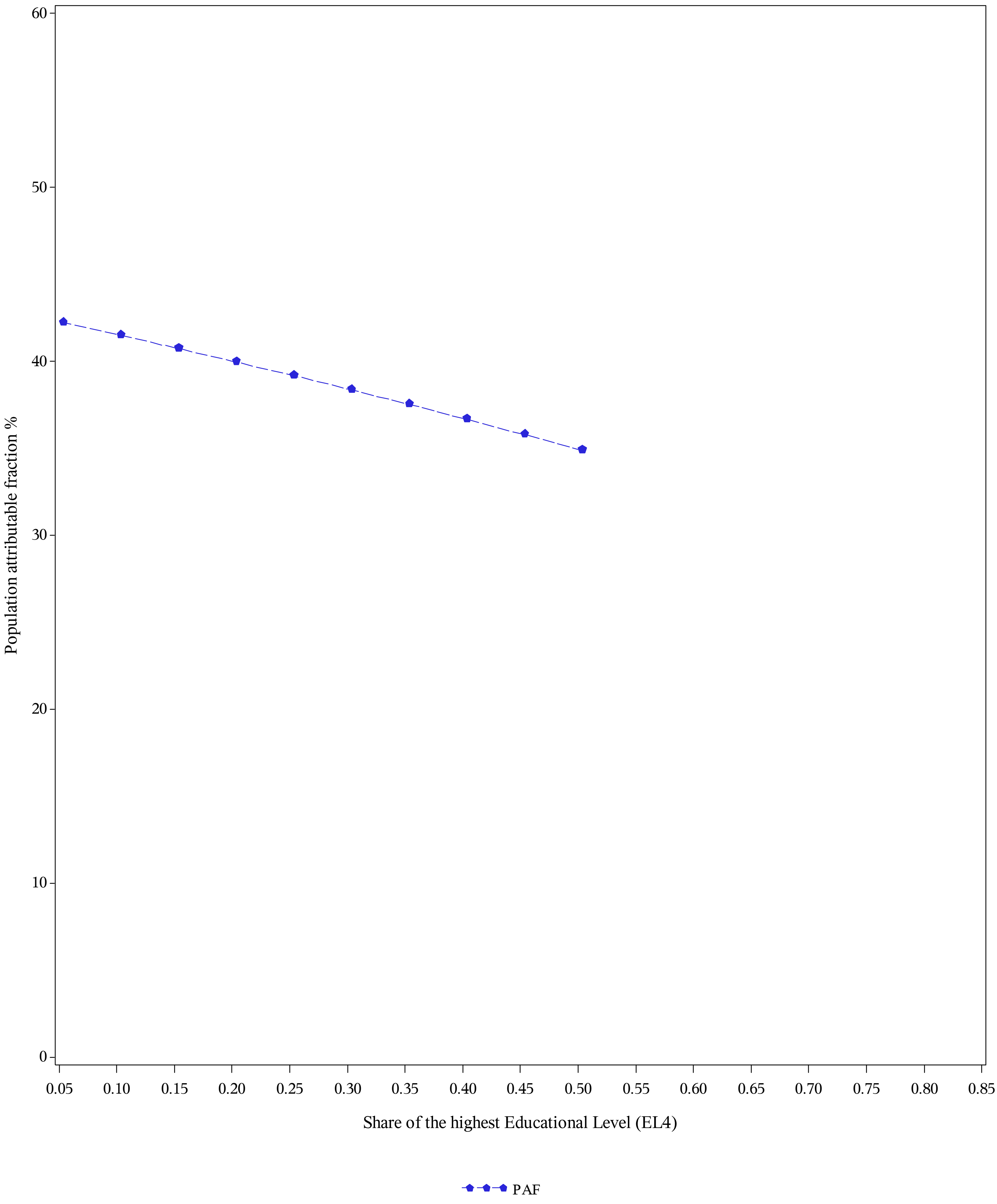

PAF in function of the share of EL4

When EL1 and EL2 are fixed at: EL1=30% ; EL2=20%
EL3 =1- EL4 - EL1 - EL2

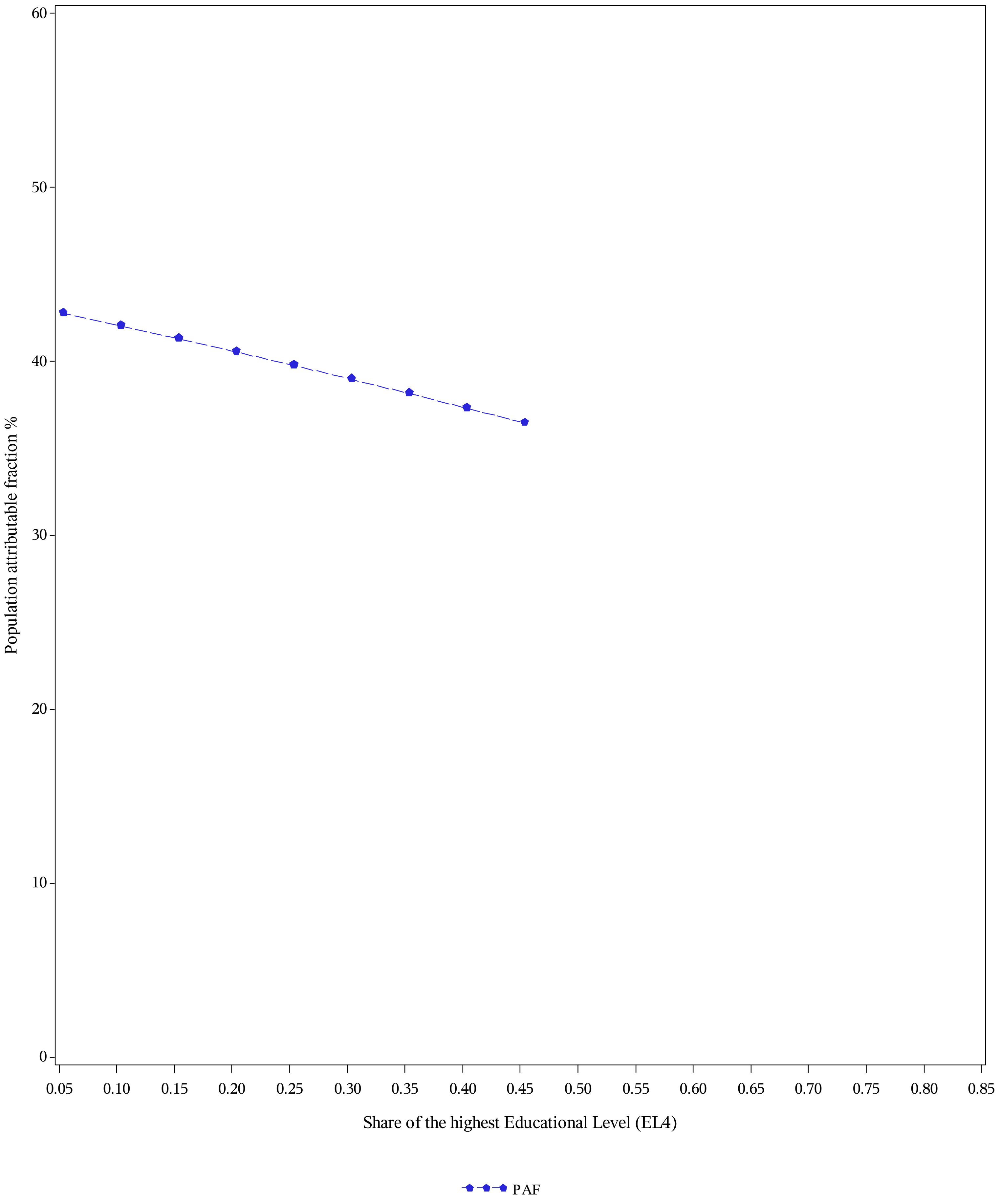

PAF in function of the share of EL4

When EL1 and EL2 are fixed at: EL1=30% ; EL2=25%
EL3 =1- EL4 - EL1 - EL2

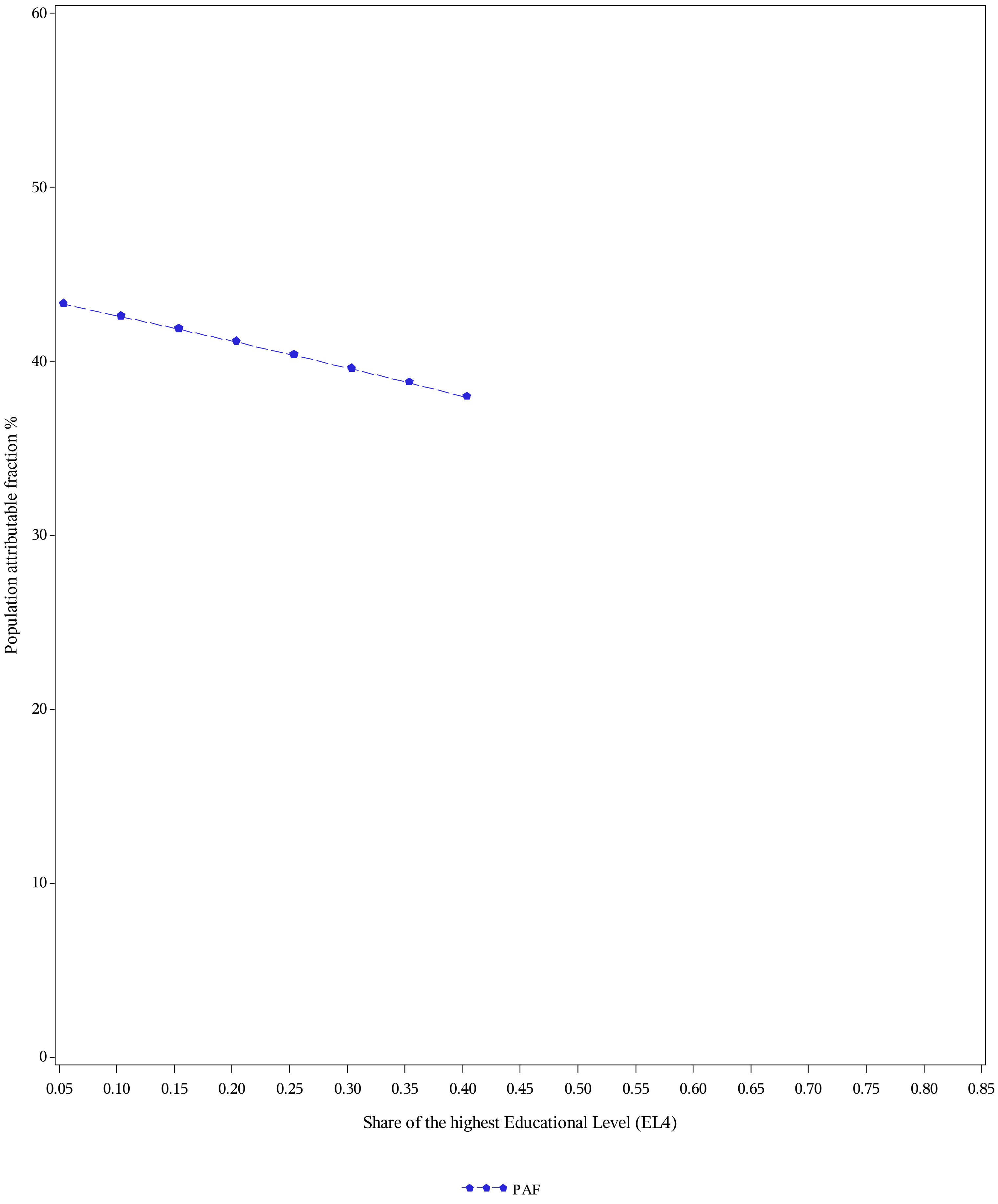

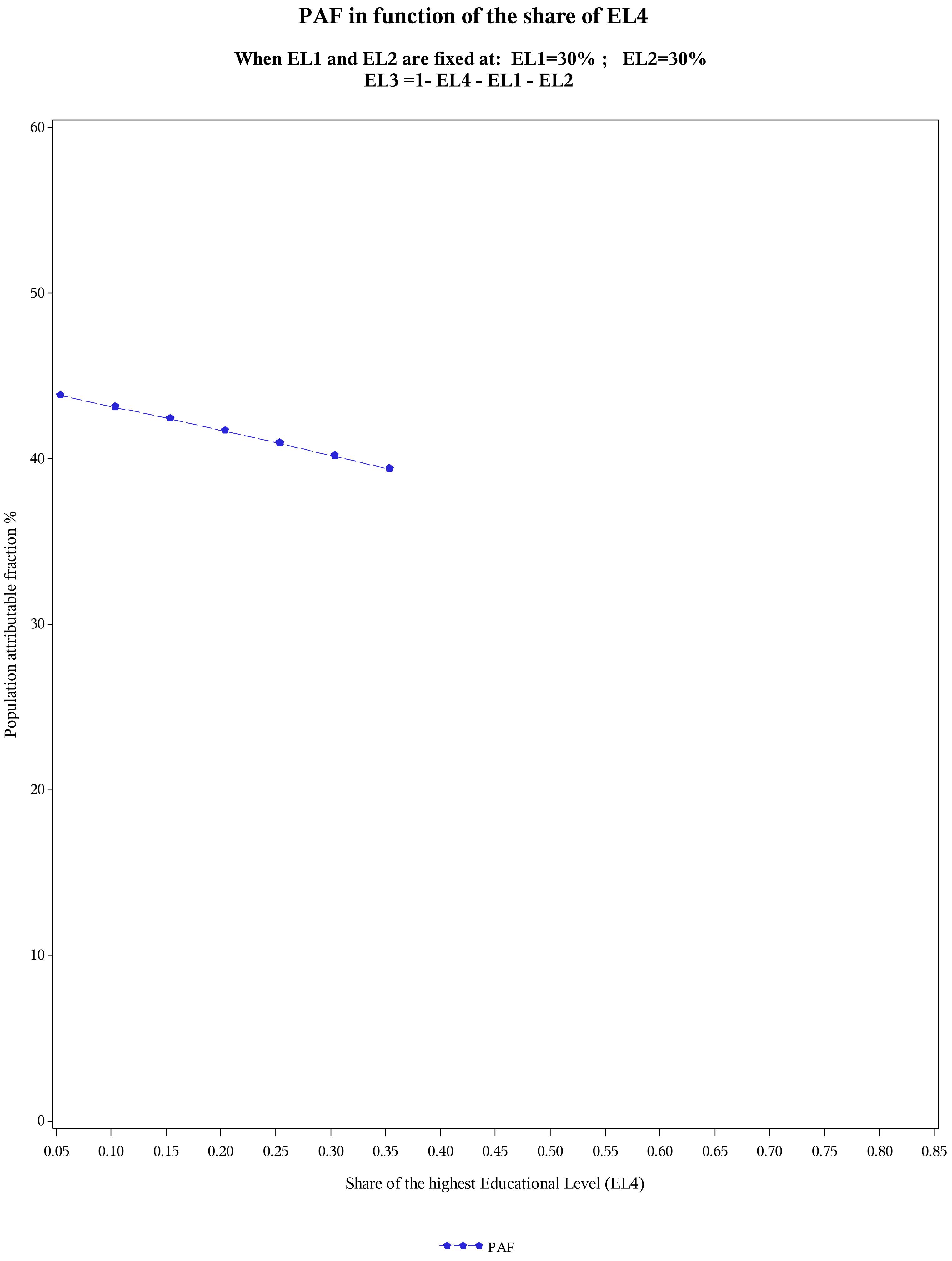
PAF in function of the share of EL4
When EL1 and EL2 are fixed at: EL1=30% ; EL2=30%
EL3 =1- EL4 - EL1 - EL2
Population attributable fraction %
0
10
20
30
40
50
60
0.05
0.10
0.15
0.20
0.25
0.30
0.35
0.40
0.45
0.50
0.55
0.60
0.65
0.70
0.75
0.80
0.85
Share of the highest Educational Level (EL4)
PAF

PAF in function of the share of EL4

When EL1 and EL2 are fixed at: EL1=30% ; EL2=35%
EL3 =1- EL4 - EL1 - EL2

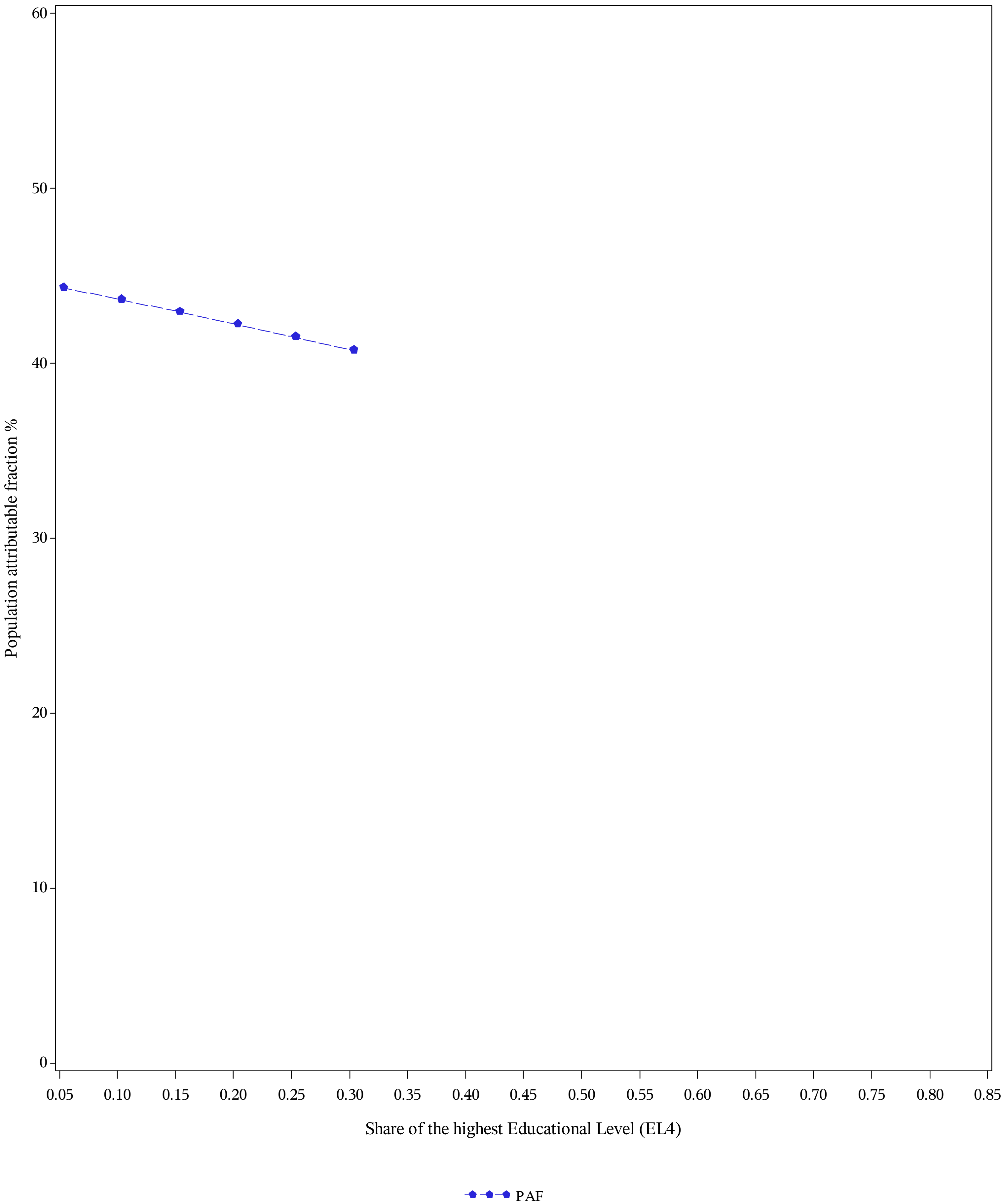

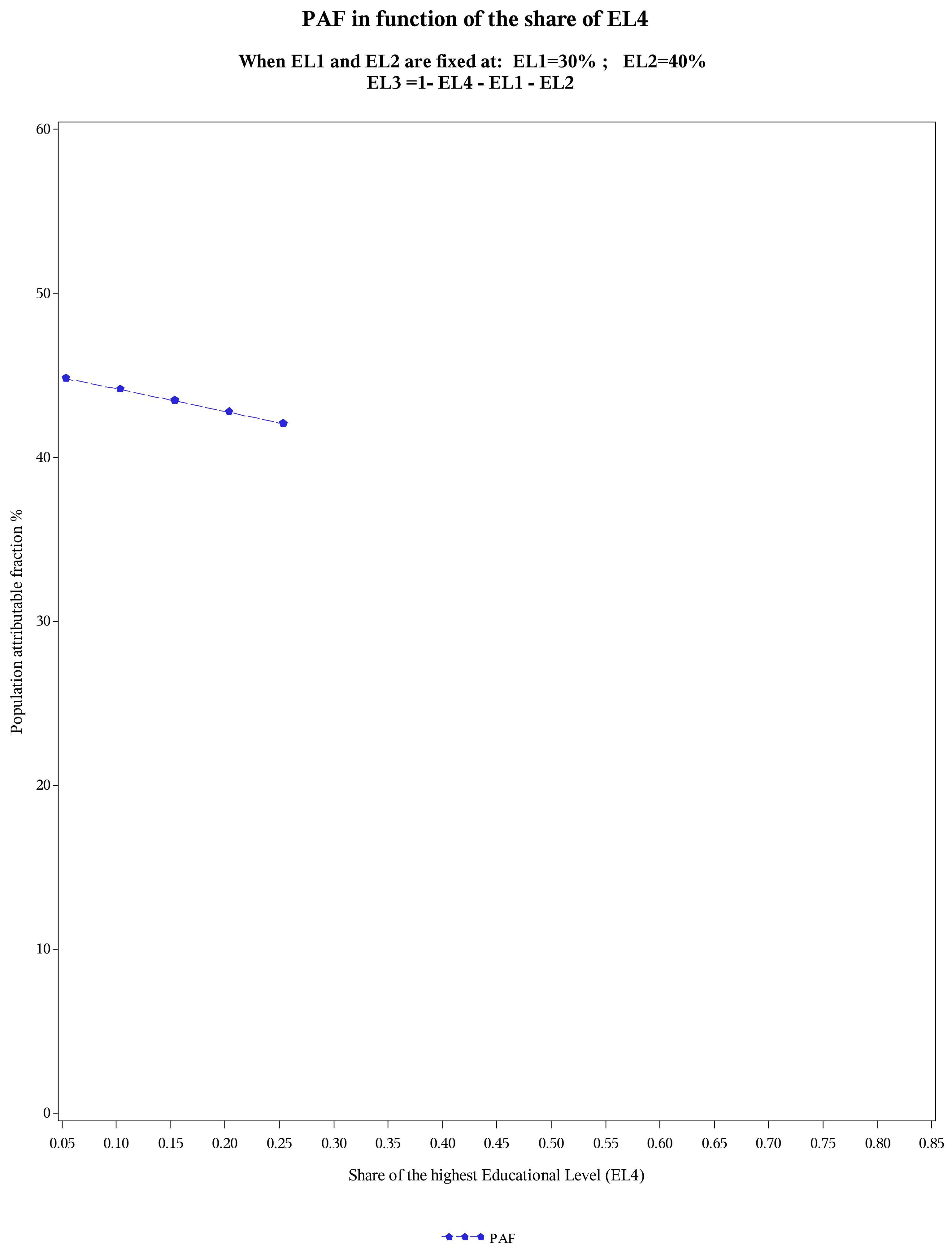

PAF in function of the share of EL4
When EL1 and EL2 are fixed at: EL1=30% ; EL2=40%
EL3 =1- EL4 - EL1 - EL2
Population attributable fraction %
0
10
20
30
40
50
60
0.05
0.10
0.15
0.20
0.25
0.30
0.35
0.40
0.45
0.50
0.55
0.60
0.65
0.70
0.75
0.80
0.85
Share of the highest Educational Level (EL4)
PAF

PAF in function of the share of EL4

When EL1 and EL2 are fixed at: EL1=30% ; EL2=45%
EL3 =1- EL4 - EL1 - EL2

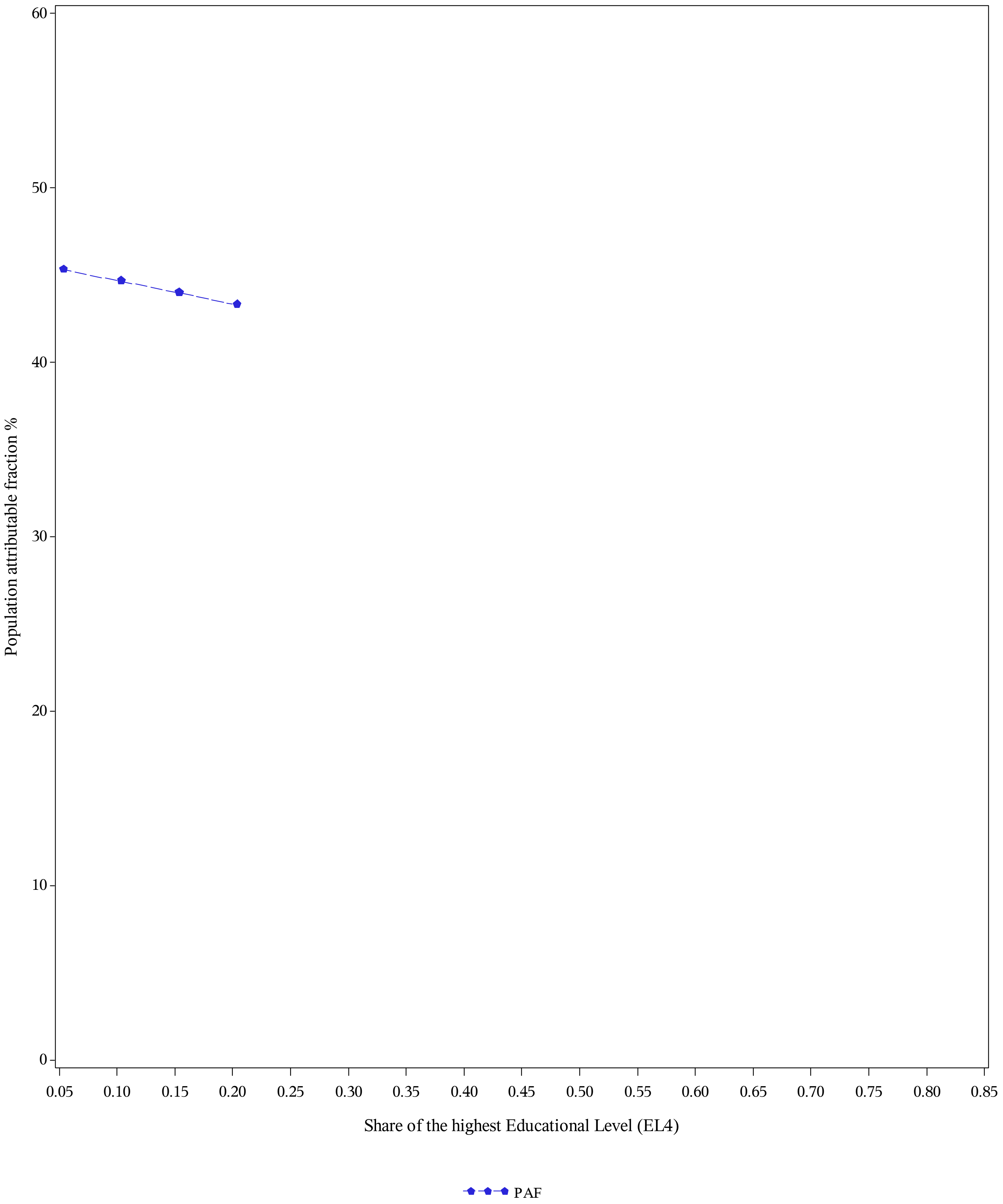

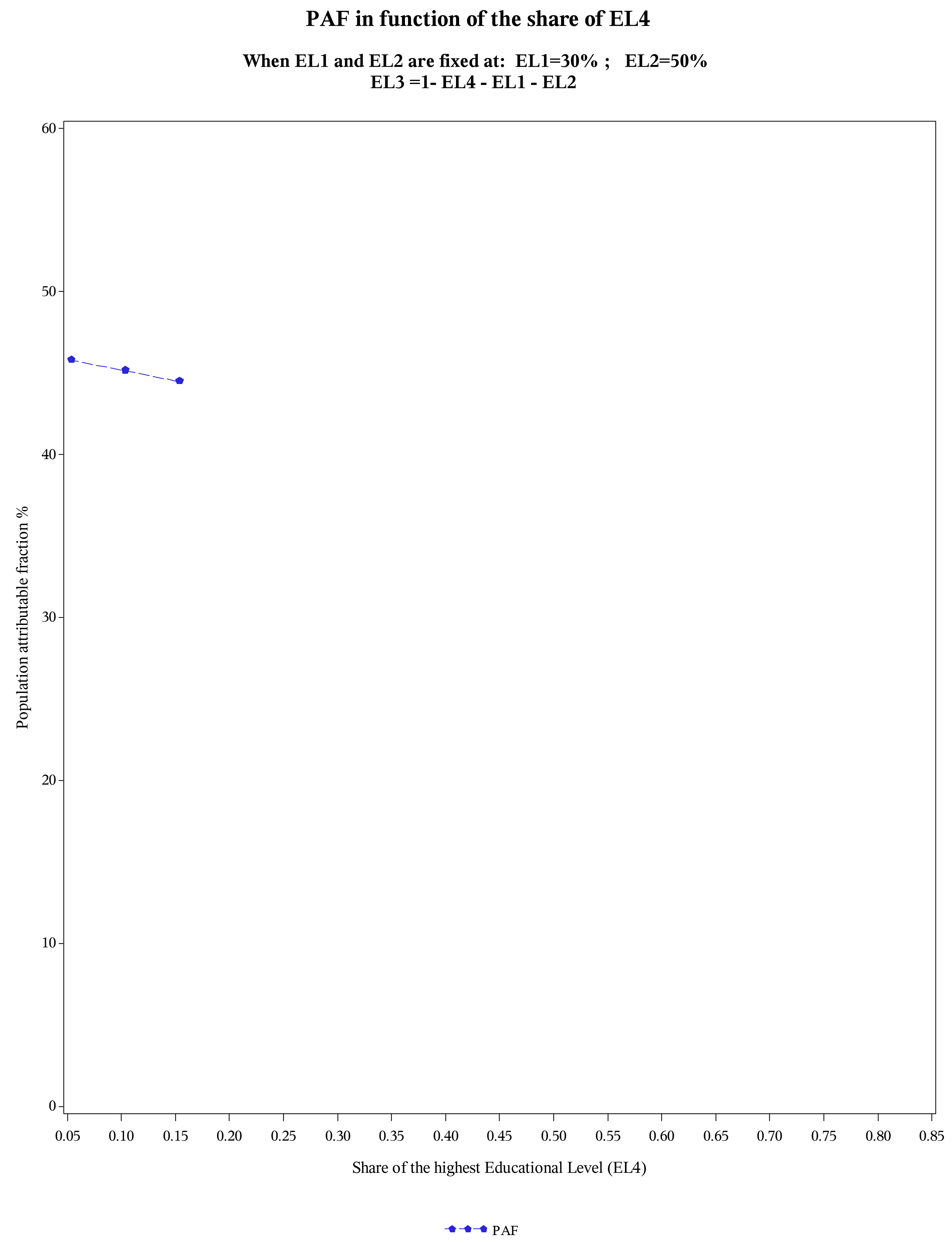
PAF in function of the share of EL4
When EL1 and EL2 are fixed at: EL1=30% ; EL2=50%
EL3 =1- EL4 - EL1 - EL2
Population attributable fraction %
0
10
20
30
40
50
60
0.05
0.10
0.15
0.20
0.25
0.30
0.35
0.40
0.45
0.50
0.55
0.60
0.65
0.70
0.75
0.80
0.85
Share of the highest Educational Level (EL4)
PAF

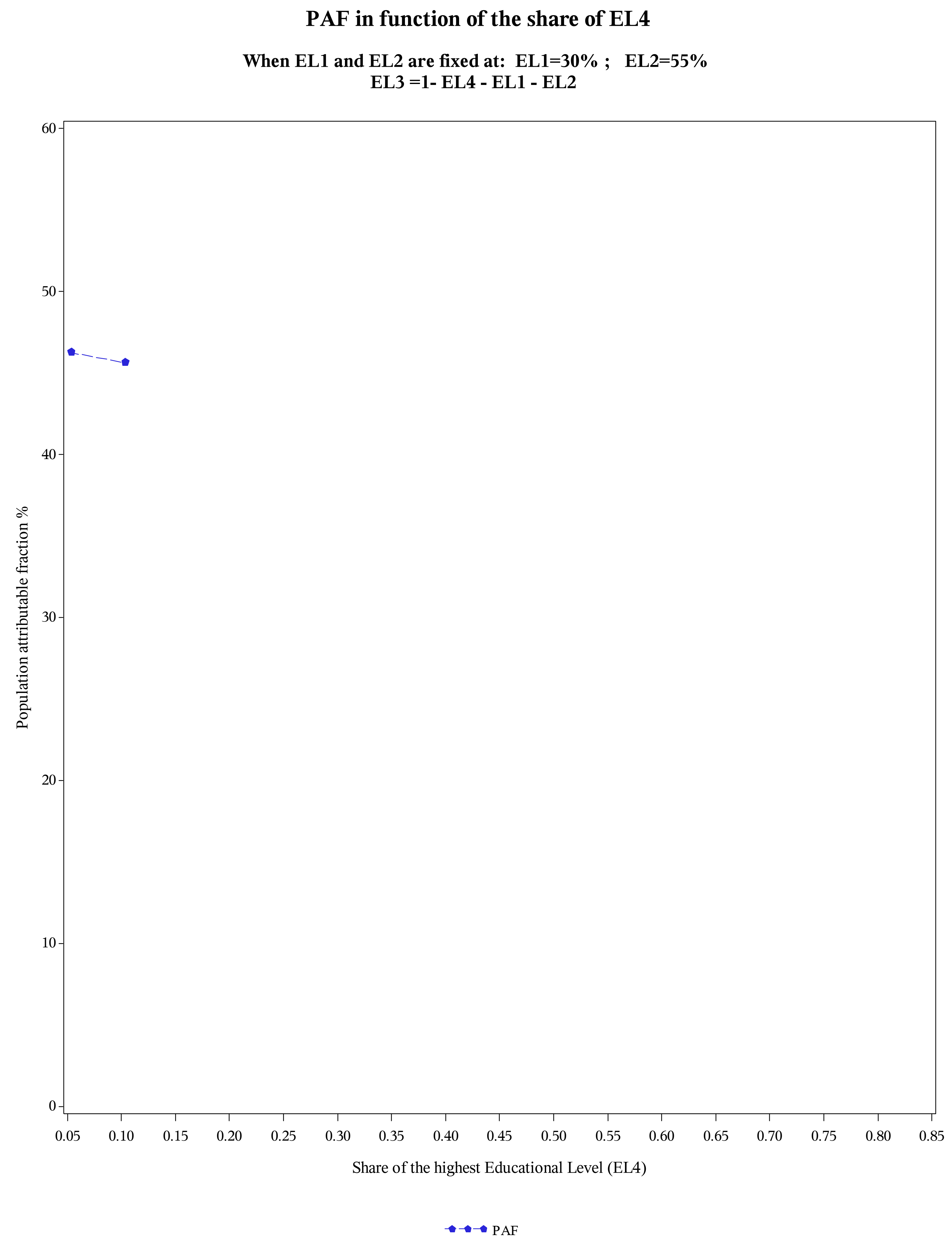

PAF in function of the share of EL4
When EL1 and EL2 are fixed at: EL1=30% ; EL2=55%
EL3 =1- EL4 - EL1 - EL2
Population attributable fraction %
0
10
20
30
40
50
60
0.05
0.10
0.15
0.20
0.25
0.30
0.35
0.40
0.45
0.50
0.55
0.60
0.65
0.70
0.75
0.80
0.85
Share of the highest Educational Level (EL4)
PAF

PAF in function of the share of EL4

When EL1 and EL2 are fixed at: EL1=35% ; EL2=5%
EL3 =1- EL4 - EL1 - EL2

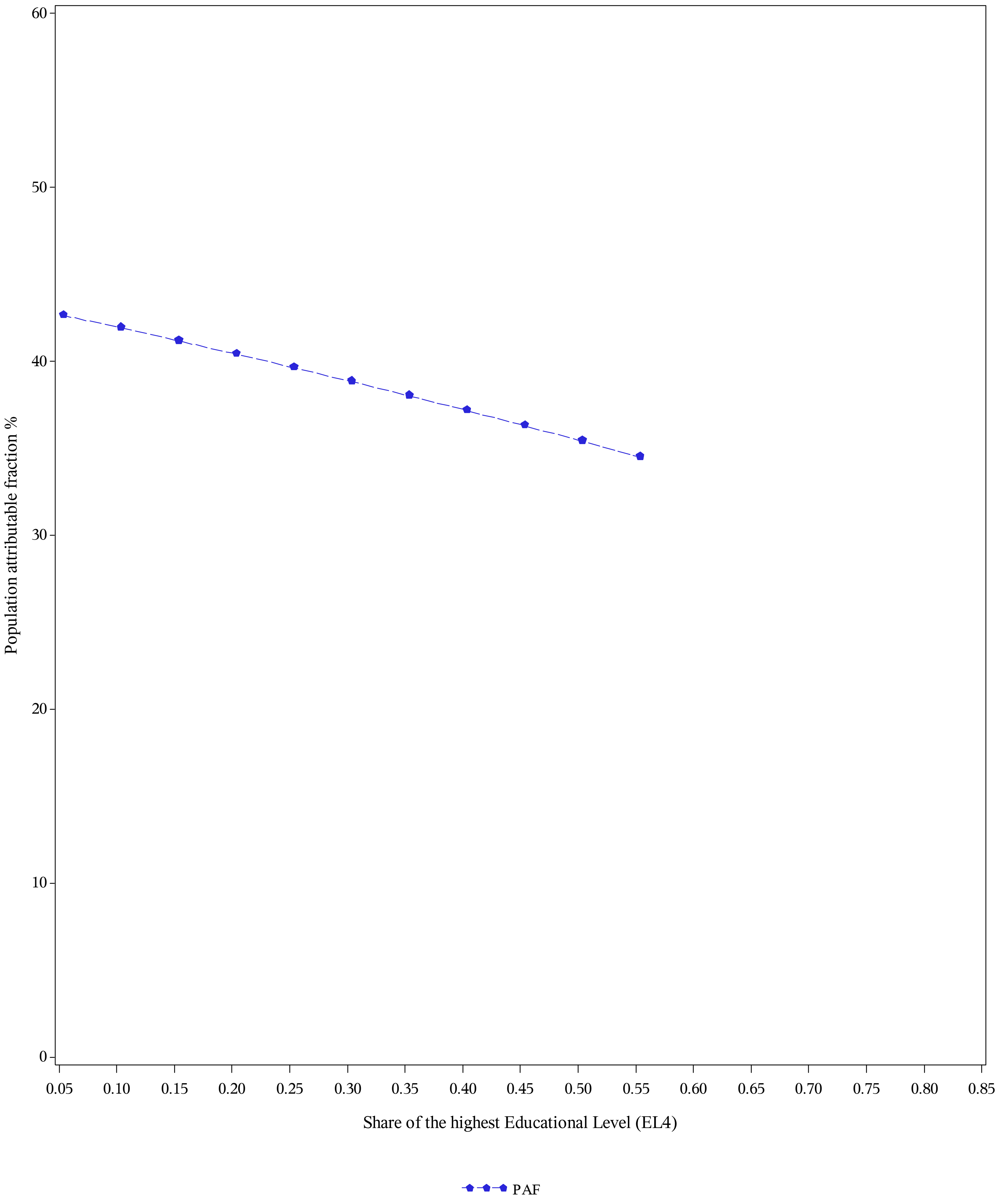

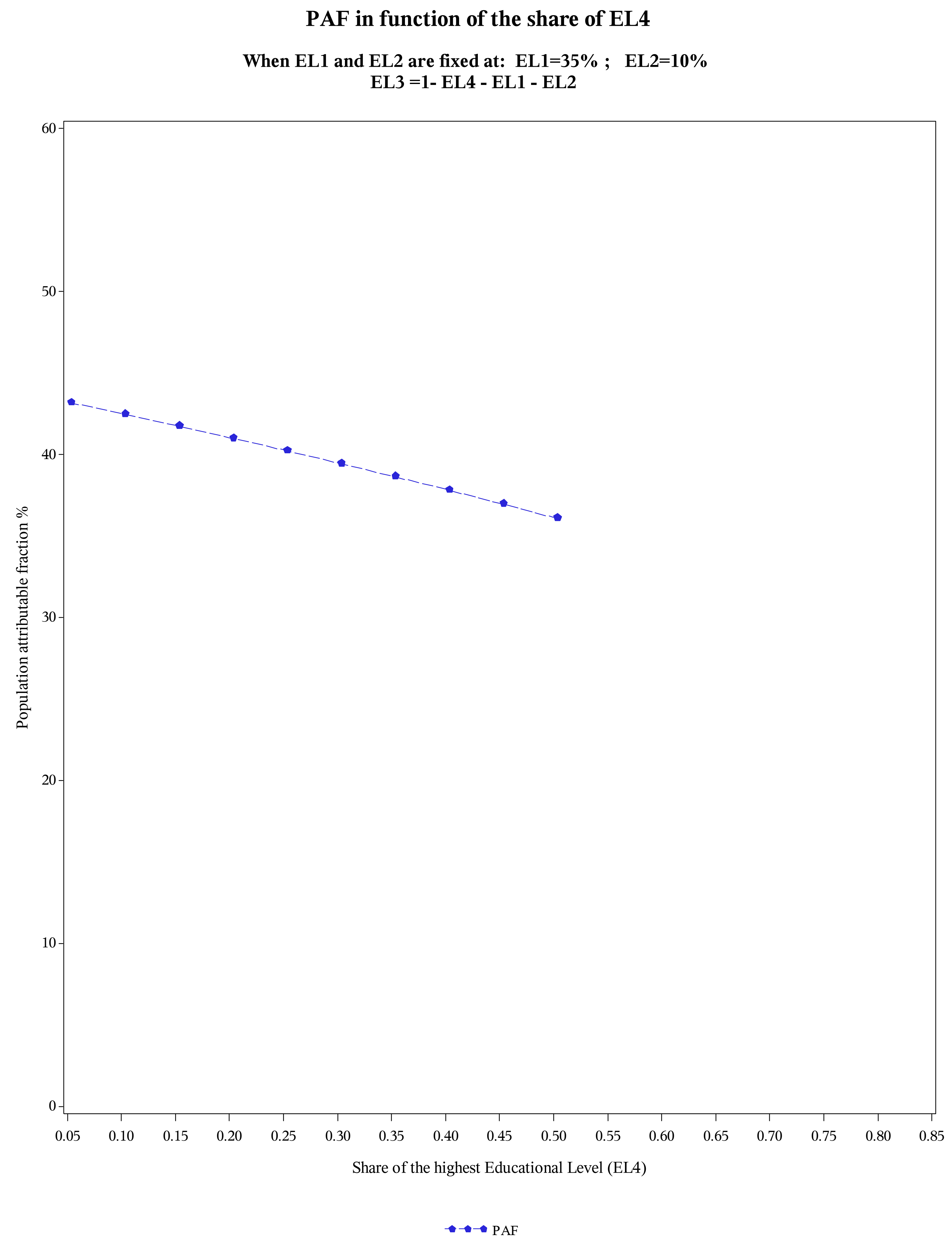

PAF in function of the share of EL4
When EL1 and EL2 are fixed at: EL1=35% ; EL2=10%
EL3 =1- EL4 - EL1 - EL2
Population attributable fraction %
0
10
20
30
40
50
60
0.05
0.10
0.15
0.20
0.25
0.30
0.35
0.40
0.45
0.50
0.55
0.60
0.65
0.70
0.75
0.80
0.85
Share of the highest Educational Level (EL4)
PAF

PAF in function of the share of EL4

When EL1 and EL2 are fixed at: EL1=35% ; EL2=15%
EL3 =1- EL4 - EL1 - EL2

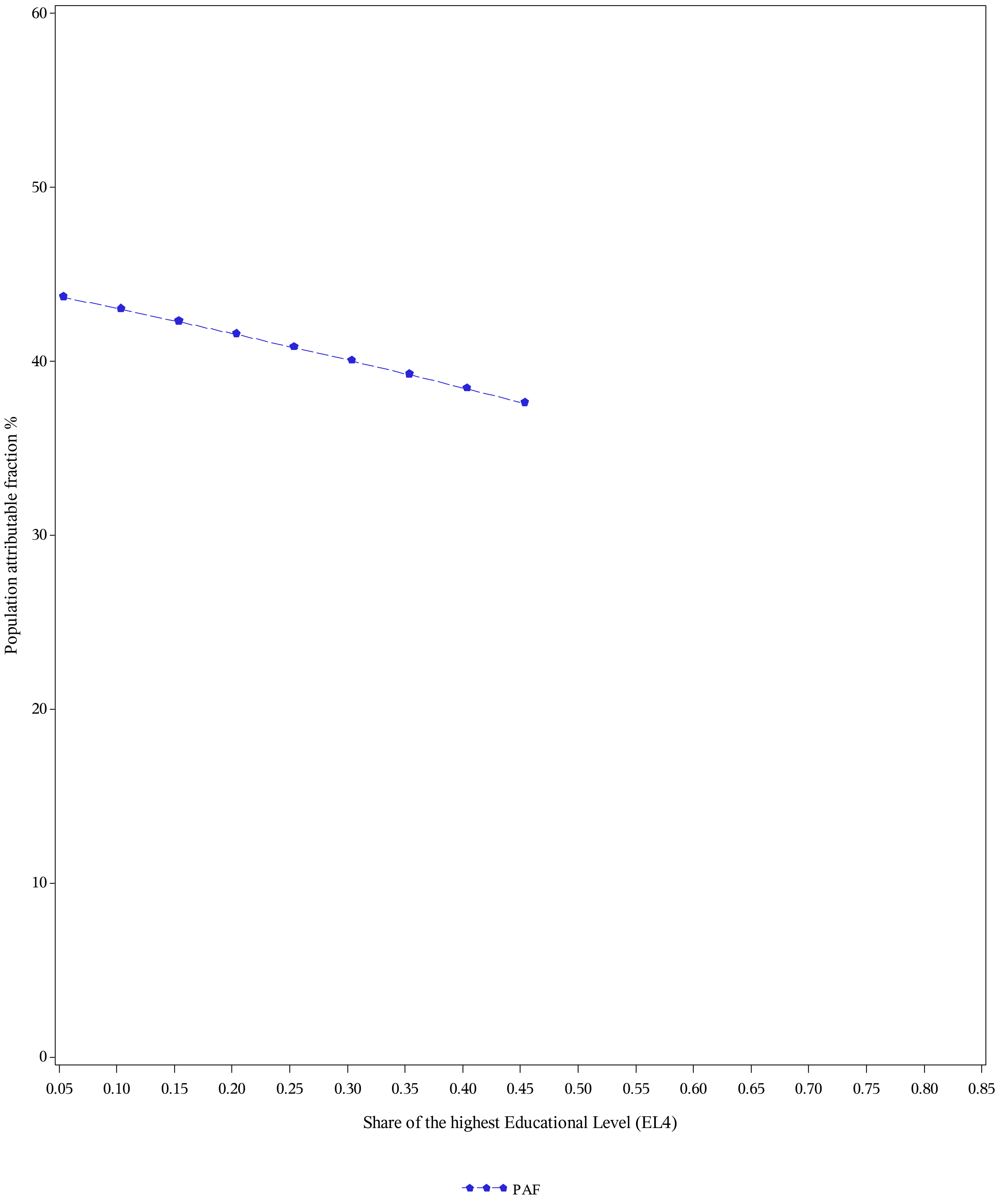

PAF in function of the share of EL4

When EL1 and EL2 are fixed at: EL1=35% ; EL2=20%
EL3 =1- EL4 - EL1 - EL2

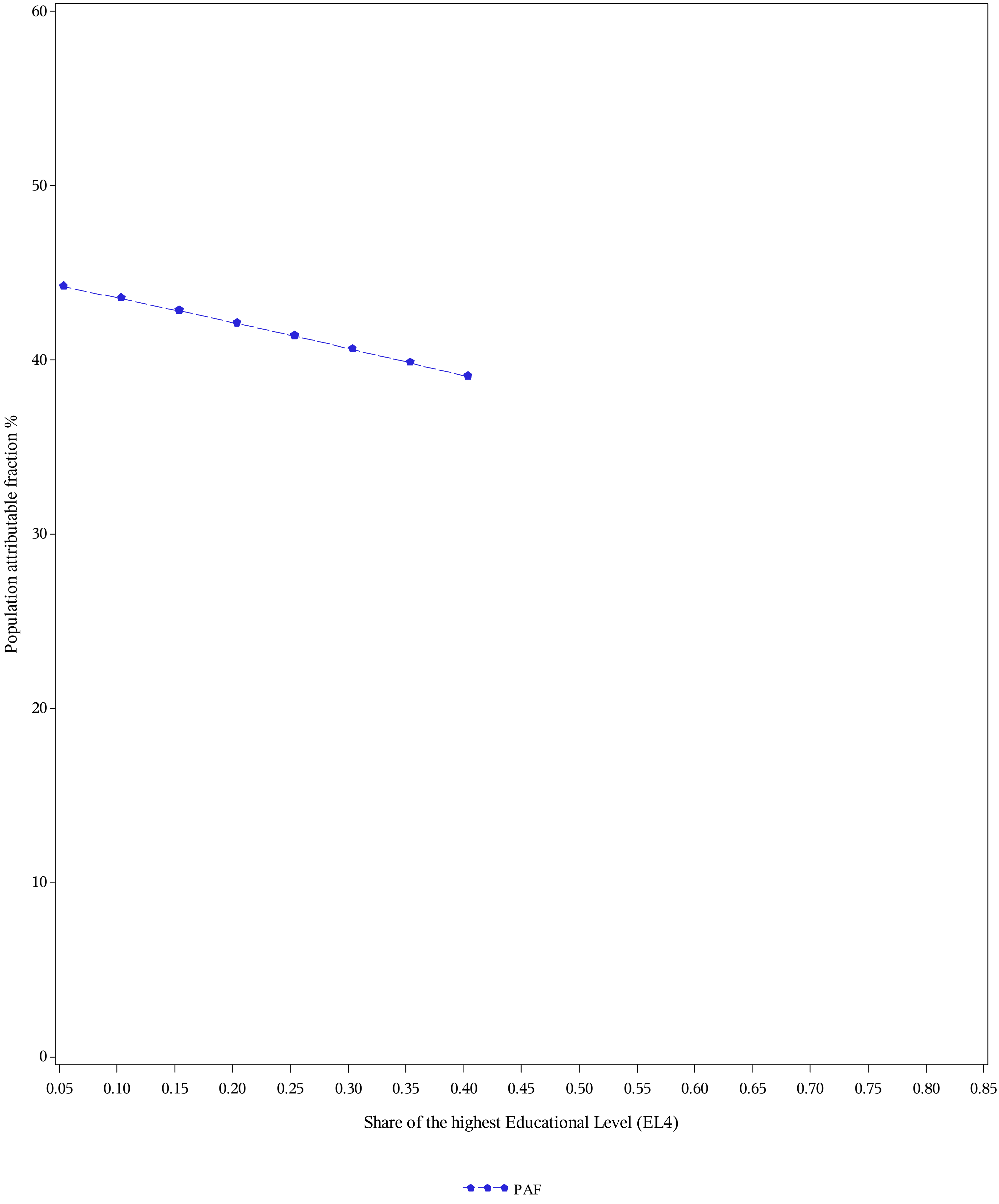

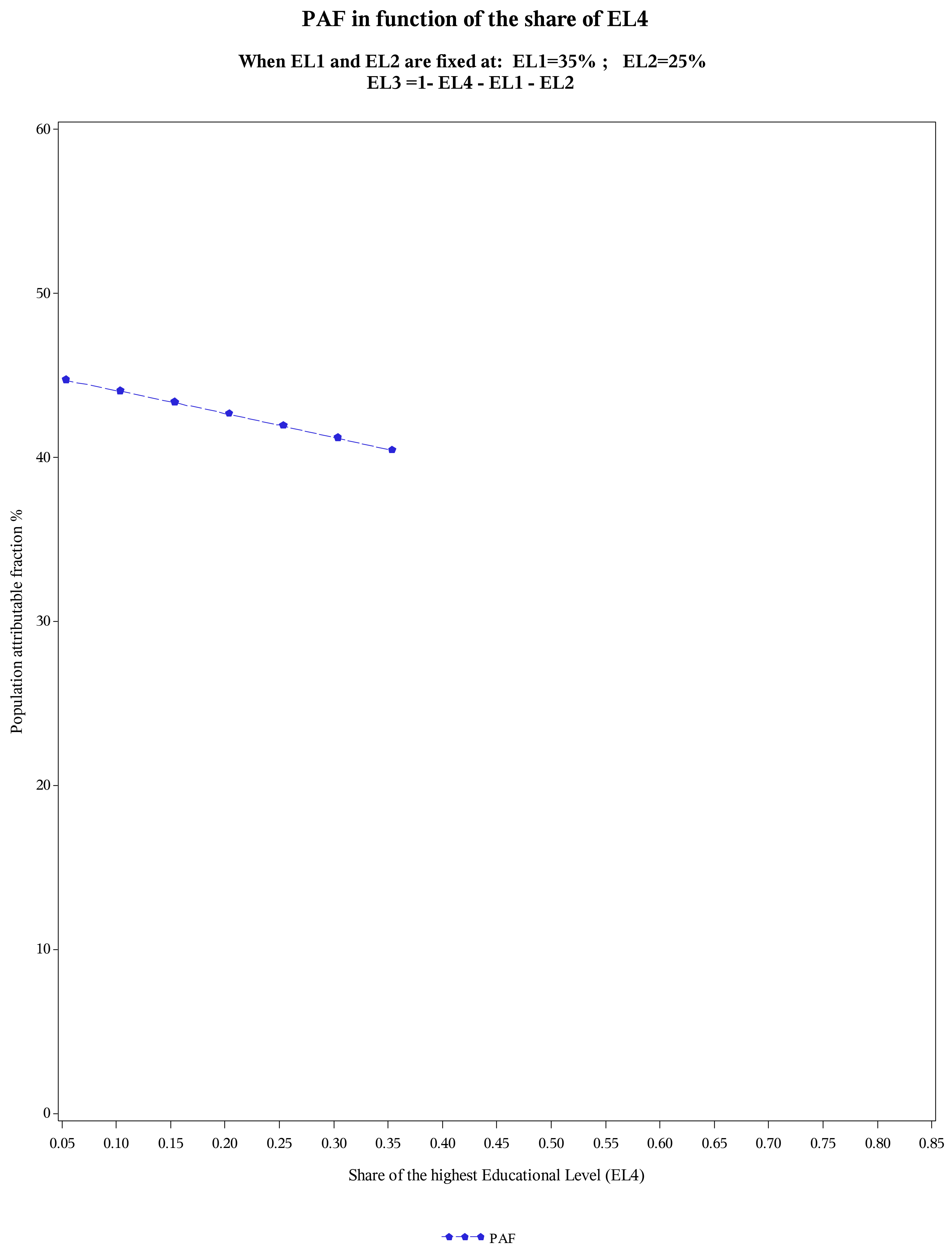

PAF in function of the share of EL4
When EL1 and EL2 are fixed at: EL1=35% ; EL2=25%
EL3 =1- EL4 - EL1 - EL2
Population attributable fraction %
0
10
20
30
40
50
60
0.05
0.10
0.15
0.20
0.25
0.30
0.35
0.40
0.45
0.50
0.55
0.60
0.65
0.70
0.75
0.80
0.85
Share of the highest Educational Level (EL4)
PAF

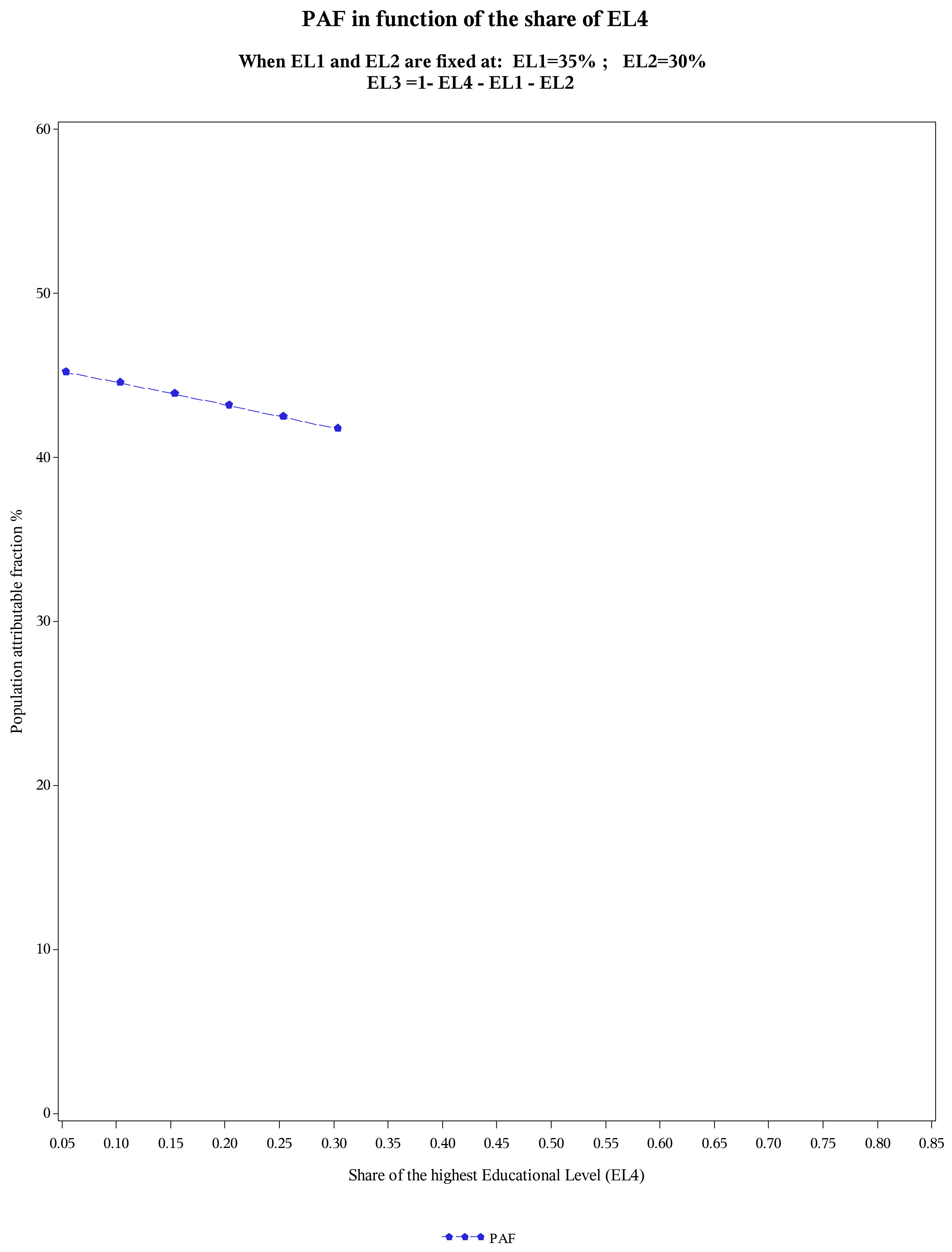
PAF in function of the share of EL4
When EL1 and EL2 are fixed at: EL1=35% ; EL2=30%
EL3 =1- EL4 - EL1 - EL2
Population attributable fraction %
0
10
20
30
40
50
60
0.05
0.10
0.15
0.20
0.25
0.30
0.35
0.40
0.45
0.50
0.55
0.60
0.65
0.70
0.75
0.80
0.85
Share of the highest Educational Level (EL4)
PAF

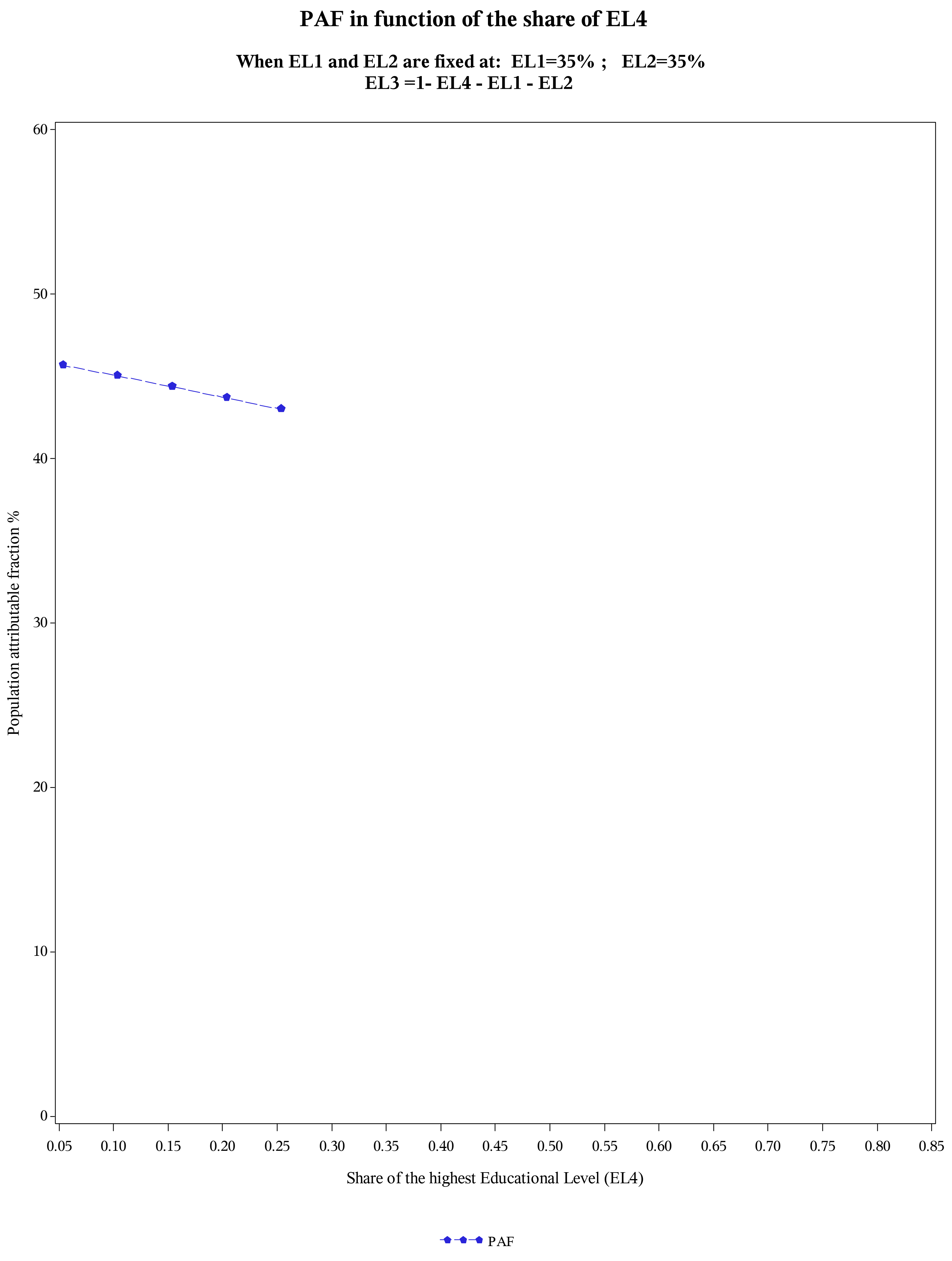
PAF in function of the share of EL4
When EL1 and EL2 are fixed at: EL1=35% ; EL2=35%
EL3 =1- EL4 - EL1 - EL2
Population attributable fraction %
0
10
20
30
40
50
60
0.05
0.10
0.15
0.20
0.25
0.30
0.35
0.40
0.45
0.50
0.55
0.60
0.65
0.70
0.75
0.80
0.85
Share of the highest Educational Level (EL4)
PAF

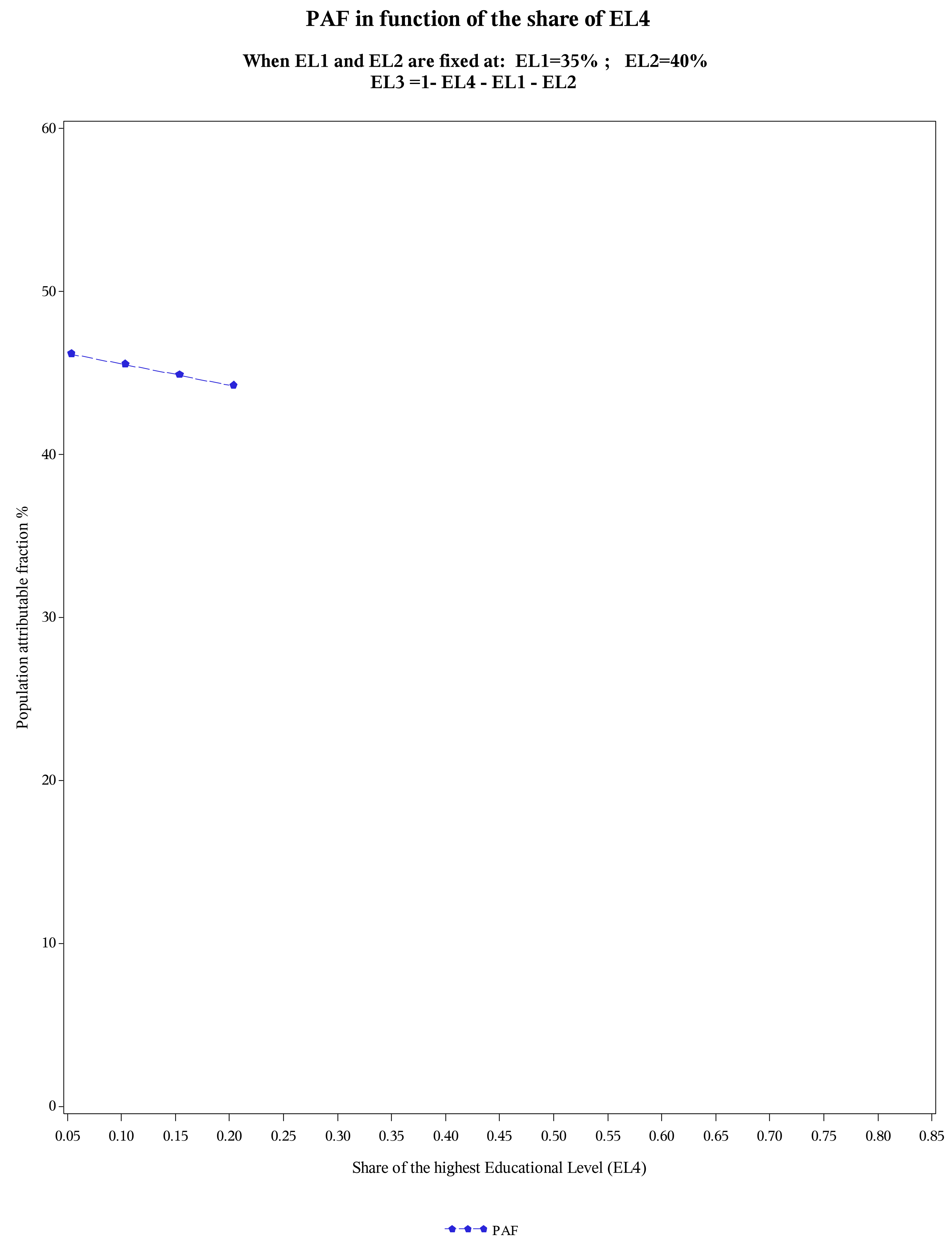
PAF in function of the share of EL4
When EL1 and EL2 are fixed at: EL1=35% ; EL2=40%
EL3 =1- EL4 - EL1 - EL2
Population attributable fraction %
0
10
20
30
40
50
60
0.05
0.10
0.15
0.20
0.25
0.30
0.35
0.40
0.45
0.50
0.55
0.60
0.65
0.70
0.75
0.80
0.85
Share of the highest Educational Level (EL4)
PAF

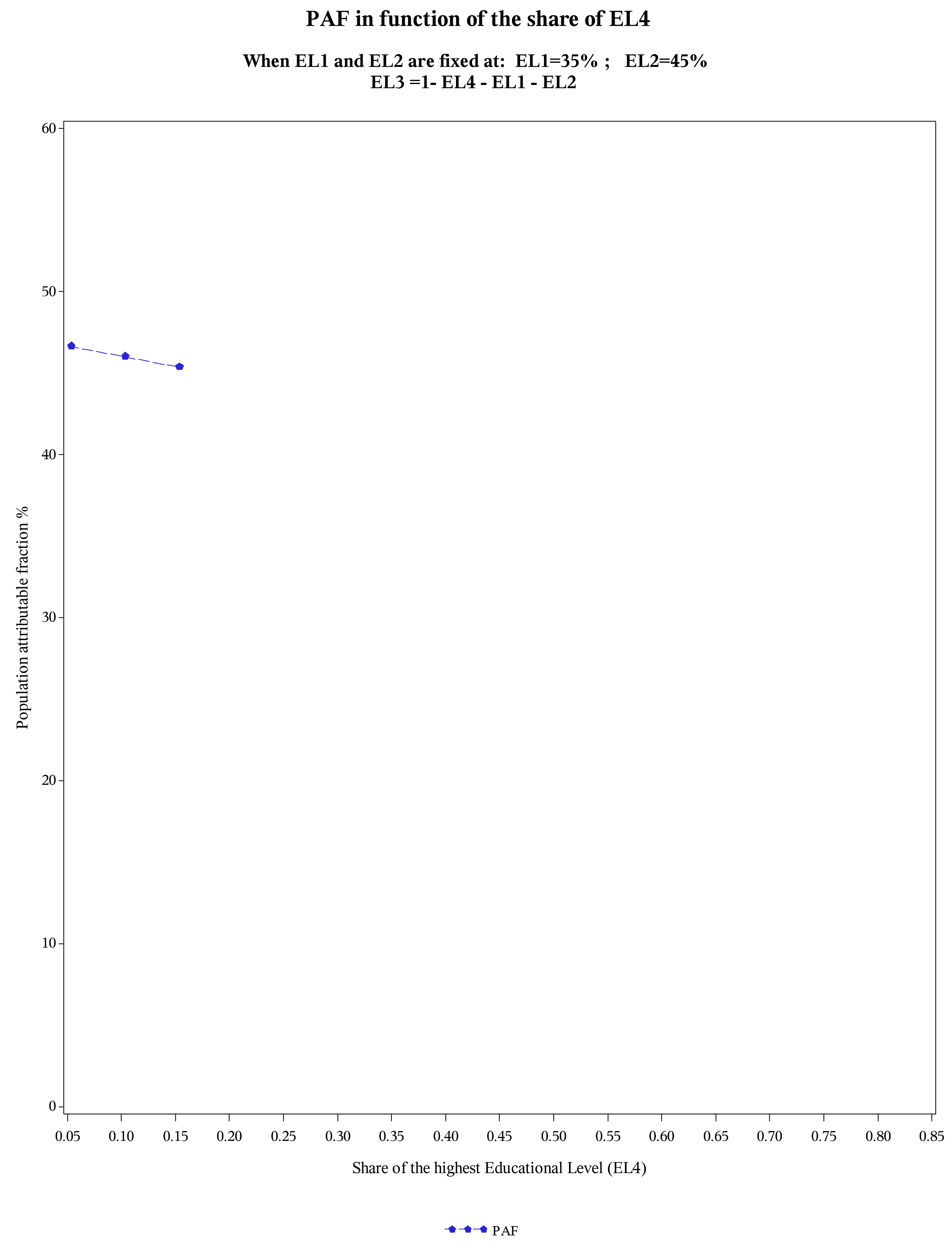
PAF in function of the share of EL4
When EL1 and EL2 are fixed at: EL1=35% ; EL2=45%
EL3 =1- EL4 - EL1 - EL2
Population attributable fraction %
0
10
20
30
40
50
60
0.05
0.10
0.15
0.20
0.25
0.30
0.35
0.40
0.45
0.50
0.55
0.60
0.65
0.70
0.75
0.80
0.85
Share of the highest Educational Level (EL4)
PAF

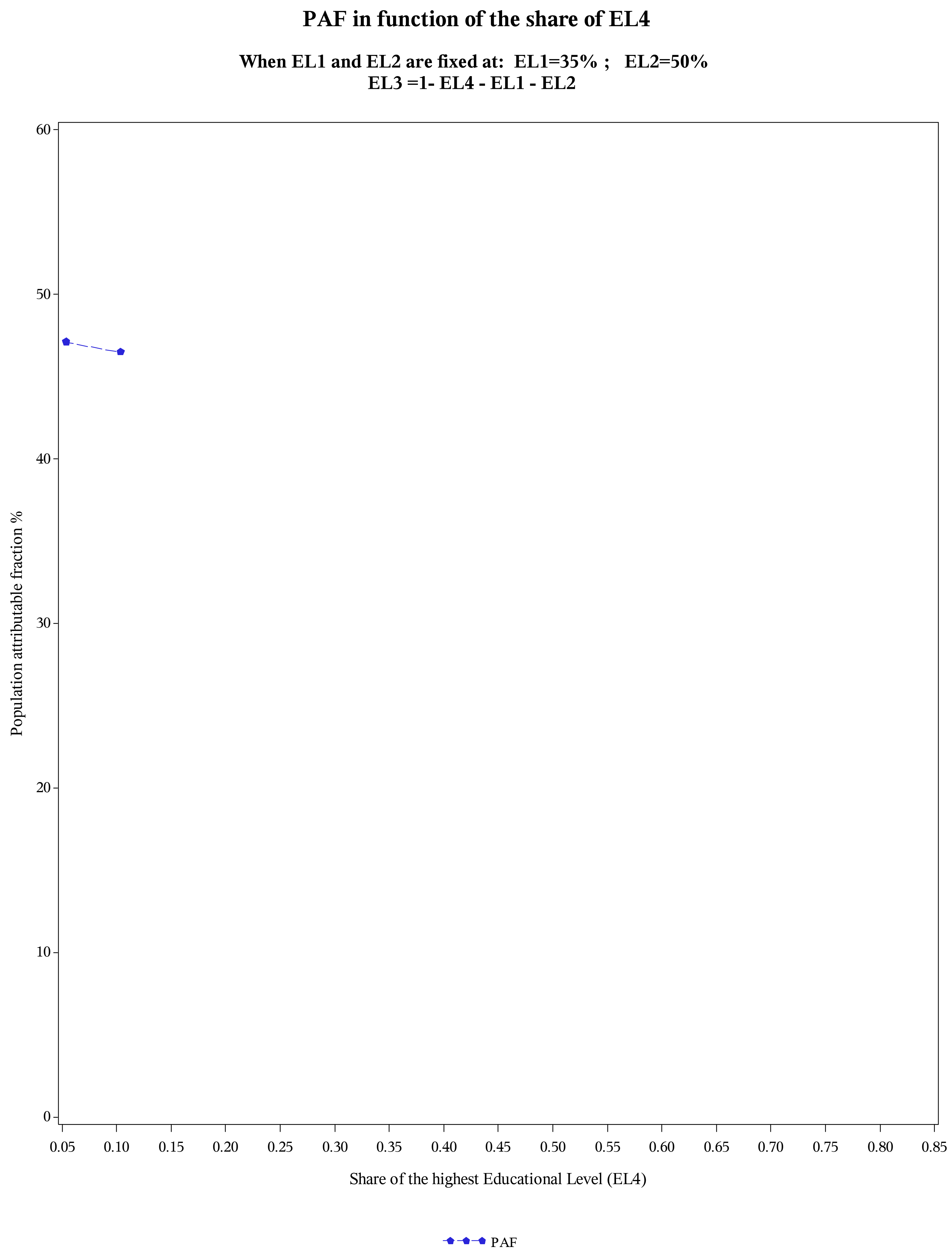
PAF in function of the share of EL4
When EL1 and EL2 are fixed at: EL1=35% ; EL2=50%
EL3 =1- EL4 - EL1 - EL2
Population attributable fraction %
0
10
20
30
40
50
60
0.05
0.10
0.15
0.20
0.25
0.30
0.35
0.40
0.45
0.50
0.55
0.60
0.65
0.70
0.75
0.80
0.85
Share of the highest Educational Level (EL4)
PAF

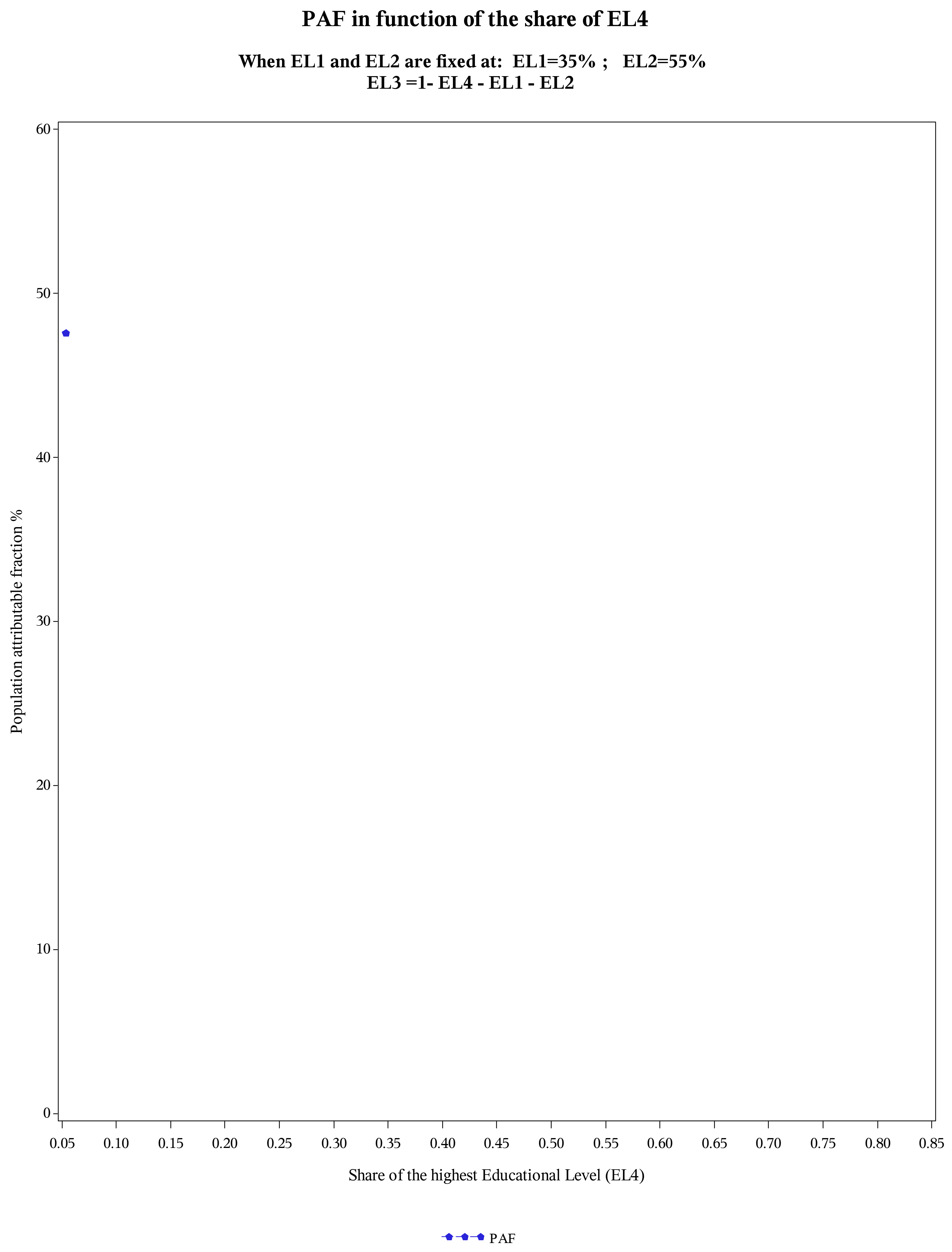
PAF in function of the share of EL4
When EL1 and EL2 are fixed at: EL1=35% ; EL2=55%
EL3 =1- EL4 - EL1 - EL2
Population attributable fraction %
0
10
20
30
40
50
60
0.05
0.10
0.15
0.20
0.25
0.30
0.35
0.40
0.45
0.50
0.55
0.60
0.65
0.70
0.75
0.80
0.85
Share of the highest Educational Level (EL4)
PAF

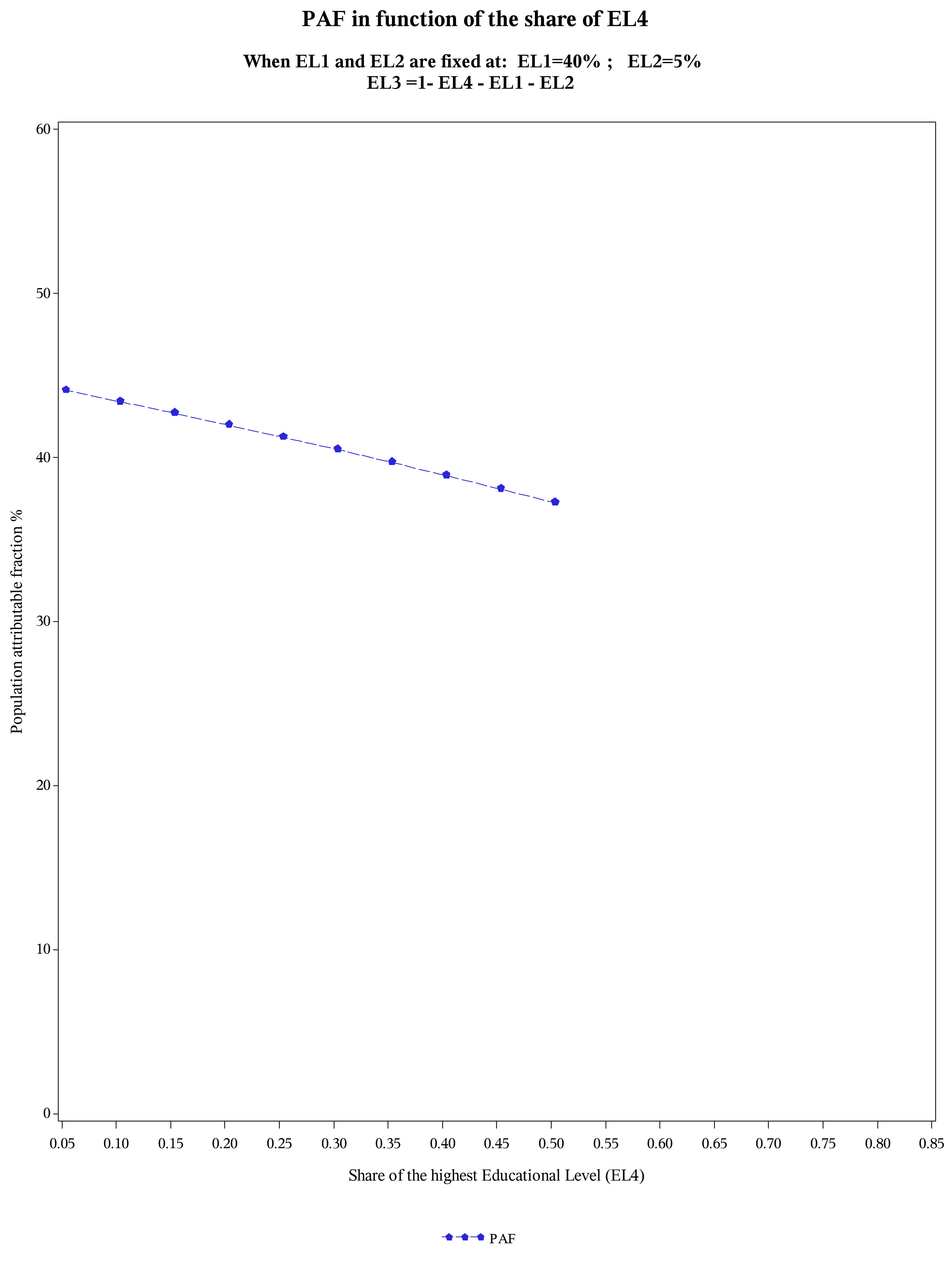
PAF in function of the share of EL4
When EL1 and EL2 are fixed at: EL1=40% ; EL2=5%
EL3 =1- EL4 - EL1 - EL2
Population attributable fraction %
0
10
20
30
40
50
60
0.05
0.10
0.15
0.20
0.25
0.30
0.35
0.40
0.45
0.50
0.55
0.60
0.65
0.70
0.75
0.80
0.85
Share of the highest Educational Level (EL4)
PAF

PAF in function of the share of EL4

When EL1 and EL2 are fixed at: EL1=40% ; EL2=10%
EL3 =1- EL4 - EL1 - EL2

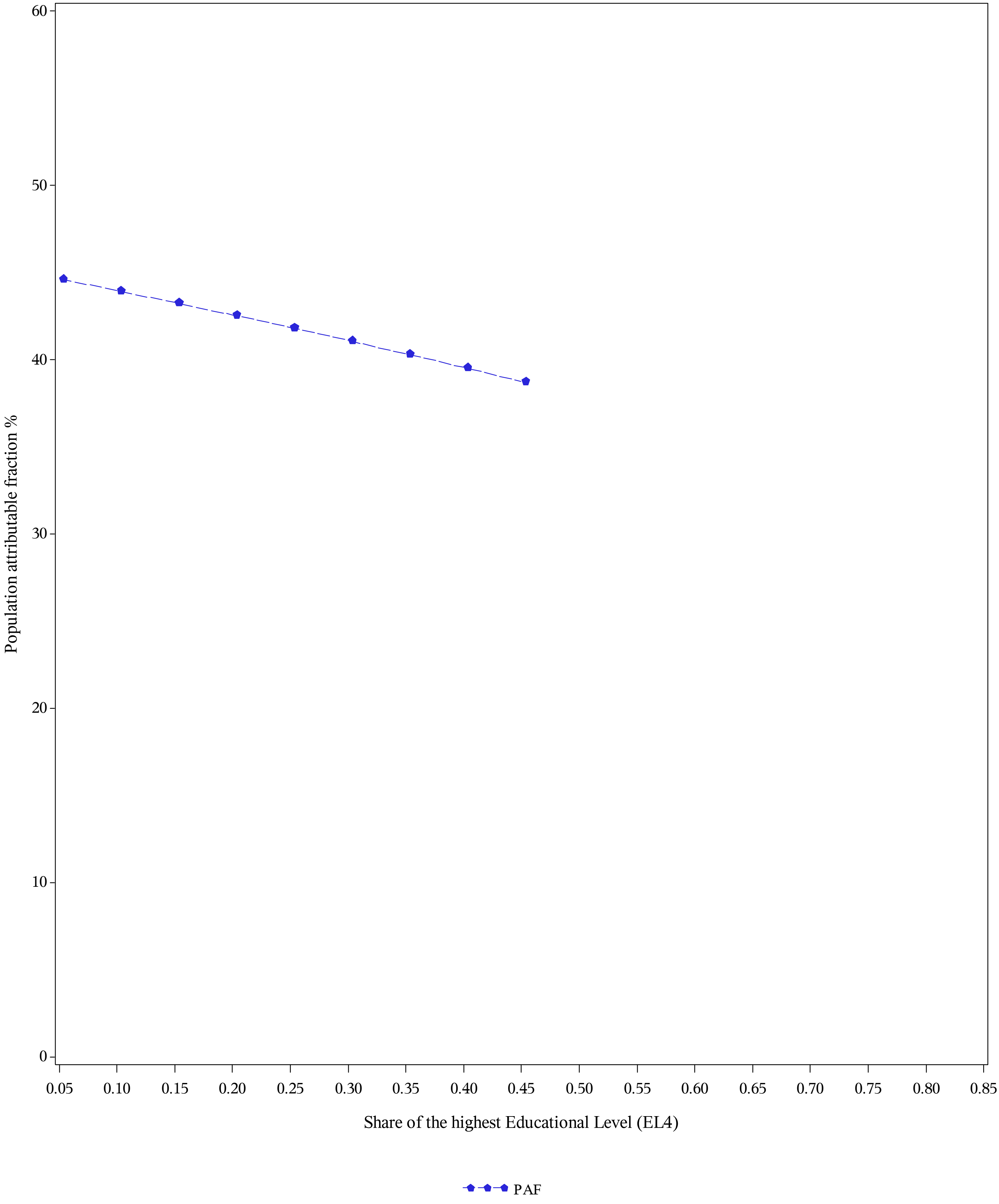

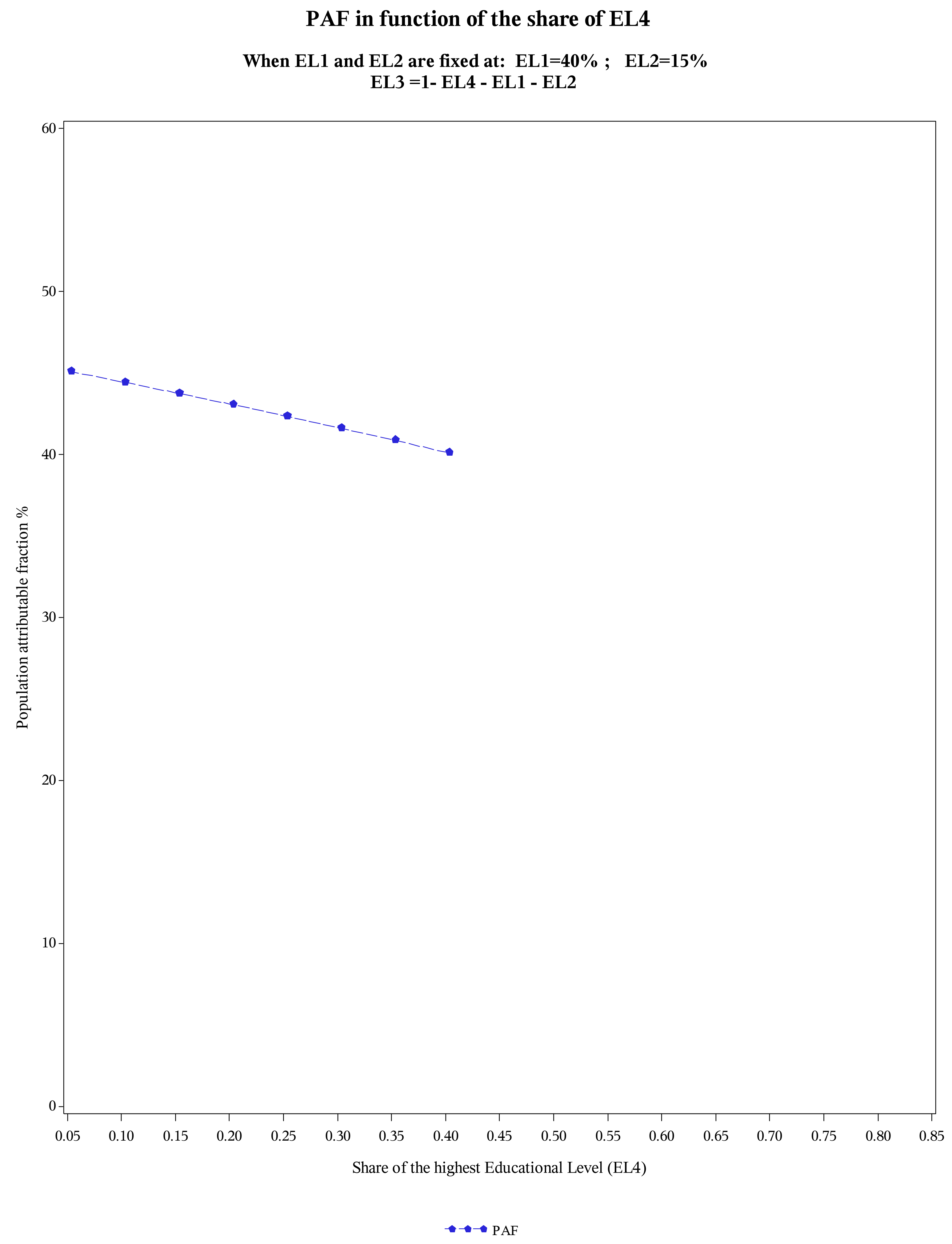

PAF in function of the share of EL4
When EL1 and EL2 are fixed at: EL1=40% ; EL2=15%
EL3 =1- EL4 - EL1 - EL2
Population attributable fraction %
0
10
20
30
40
50
60
0.05
0.10
0.15
0.20
0.25
0.30
0.35
0.40
0.45
0.50
0.55
0.60
0.65
0.70
0.75
0.80
0.85
Share of the highest Educational Level (EL4)
PAF

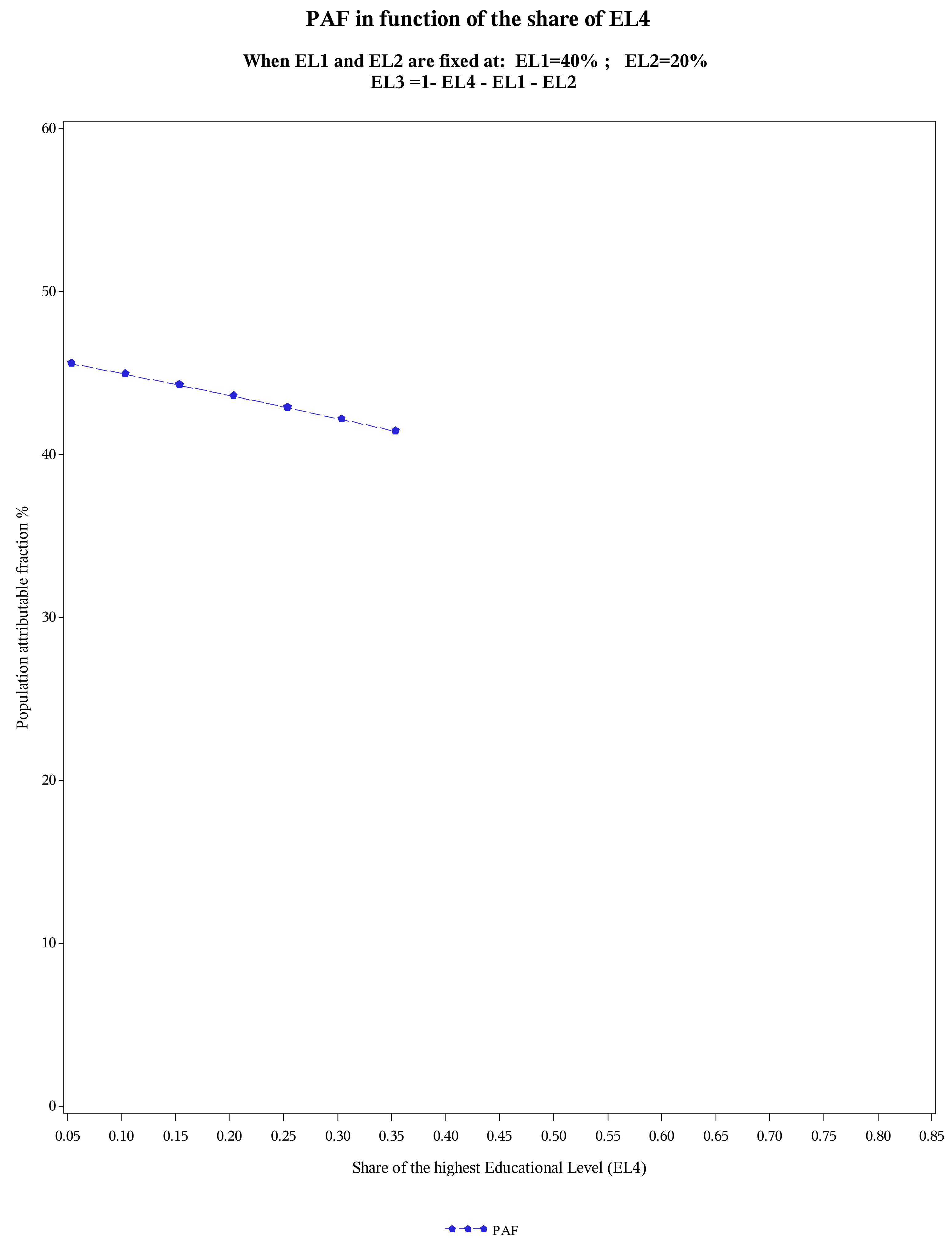
PAF in function of the share of EL4
When EL1 and EL2 are fixed at: EL1=40% ; EL2=20%
EL3 =1- EL4 - EL1 - EL2
Population attributable fraction %
0
10
20
30
40
50
60
0.05
0.10
0.15
0.20
0.25
0.30
0.35
0.40
0.45
0.50
0.55
0.60
0.65
0.70
0.75
0.80
0.85
Share of the highest Educational Level (EL4)
PAF

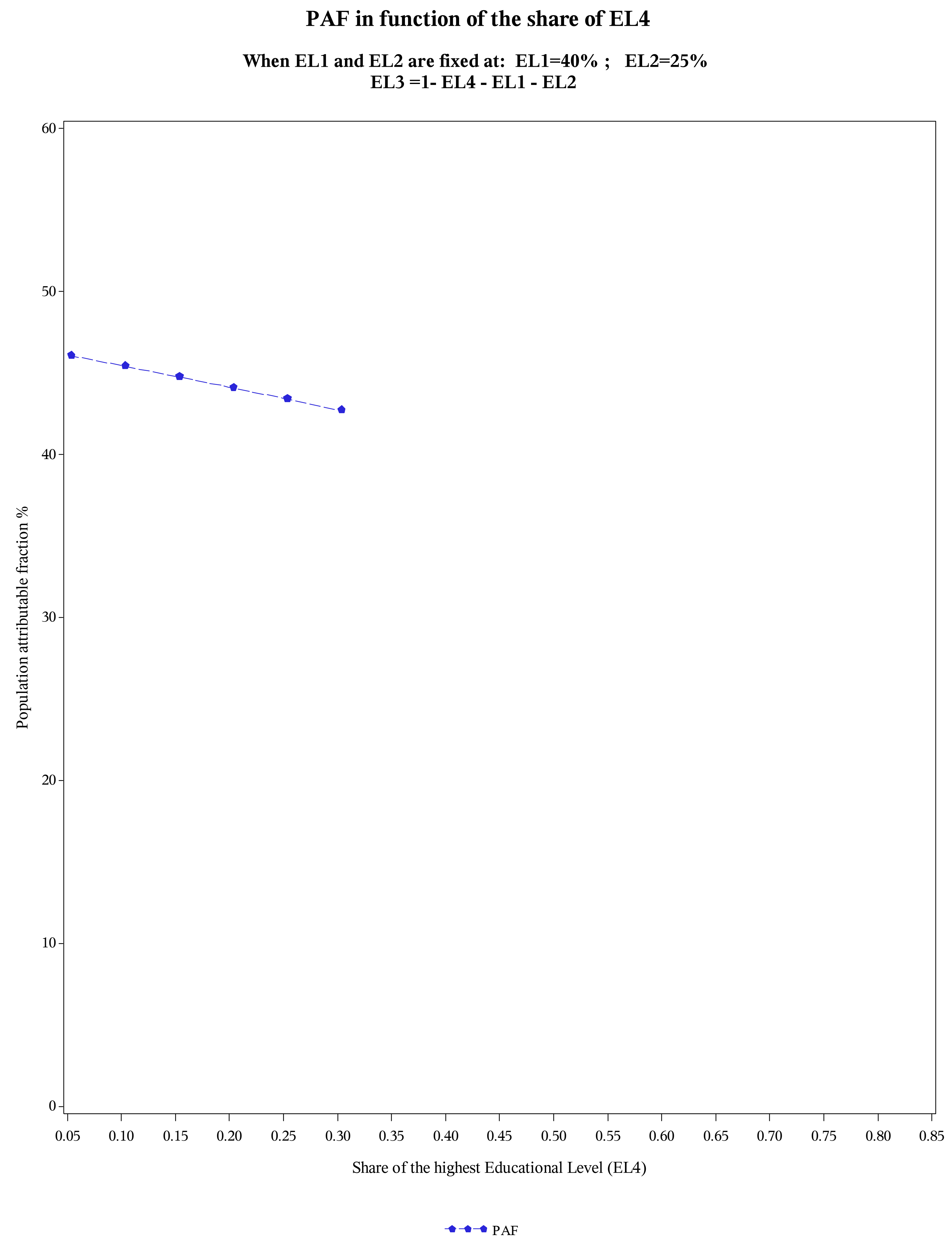
PAF in function of the share of EL4
When EL1 and EL2 are fixed at: EL1=40% ; EL2=25%
EL3 =1- EL4 - EL1 - EL2
Population attributable fraction %
0
10
20
30
40
50
60
0.05
0.10
0.15
0.20
0.25
0.30
0.35
0.40
0.45
0.50
0.55
0.60
0.65
0.70
0.75
0.80
0.85
Share of the highest Educational Level (EL4)
PAF

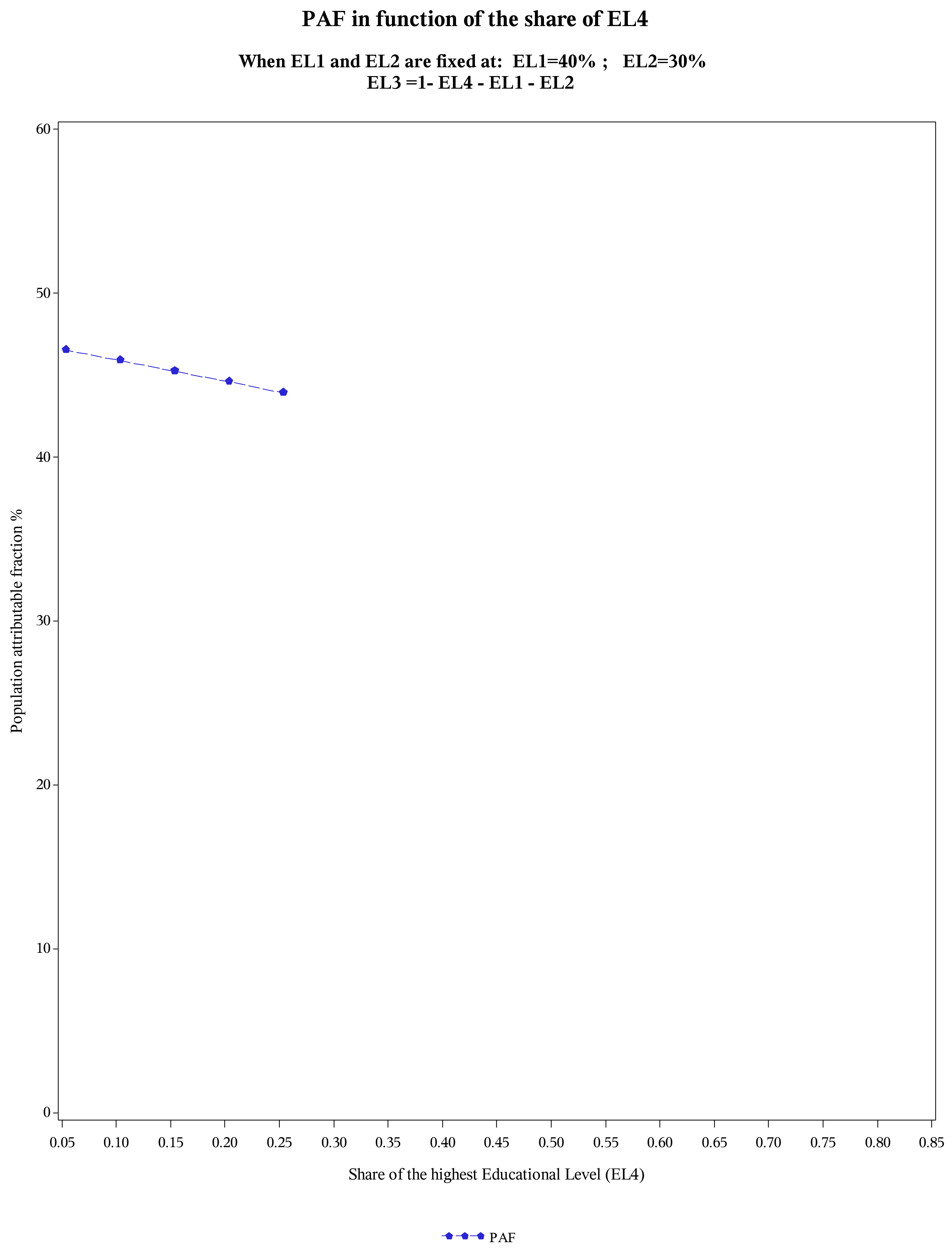

PAF in function of the share of EL4
When EL1 and EL2 are fixed at: EL1=40% ; EL2=30%
EL3 =1- EL4 - EL1 - EL2
Population attributable fraction %
0
10
20
30
40
50
60
0.05
0.10
0.15
0.20
0.25
0.30
0.35
0.40
0.45
0.50
0.55
0.60
0.65
0.70
0.75
0.80
0.85
Share of the highest Educational Level (EL4)
PAF

PAF in function of the share of EL4

When EL1 and EL2 are fixed at: EL1=40% ; EL2=35%
EL3 =1- EL4 - EL1 - EL2

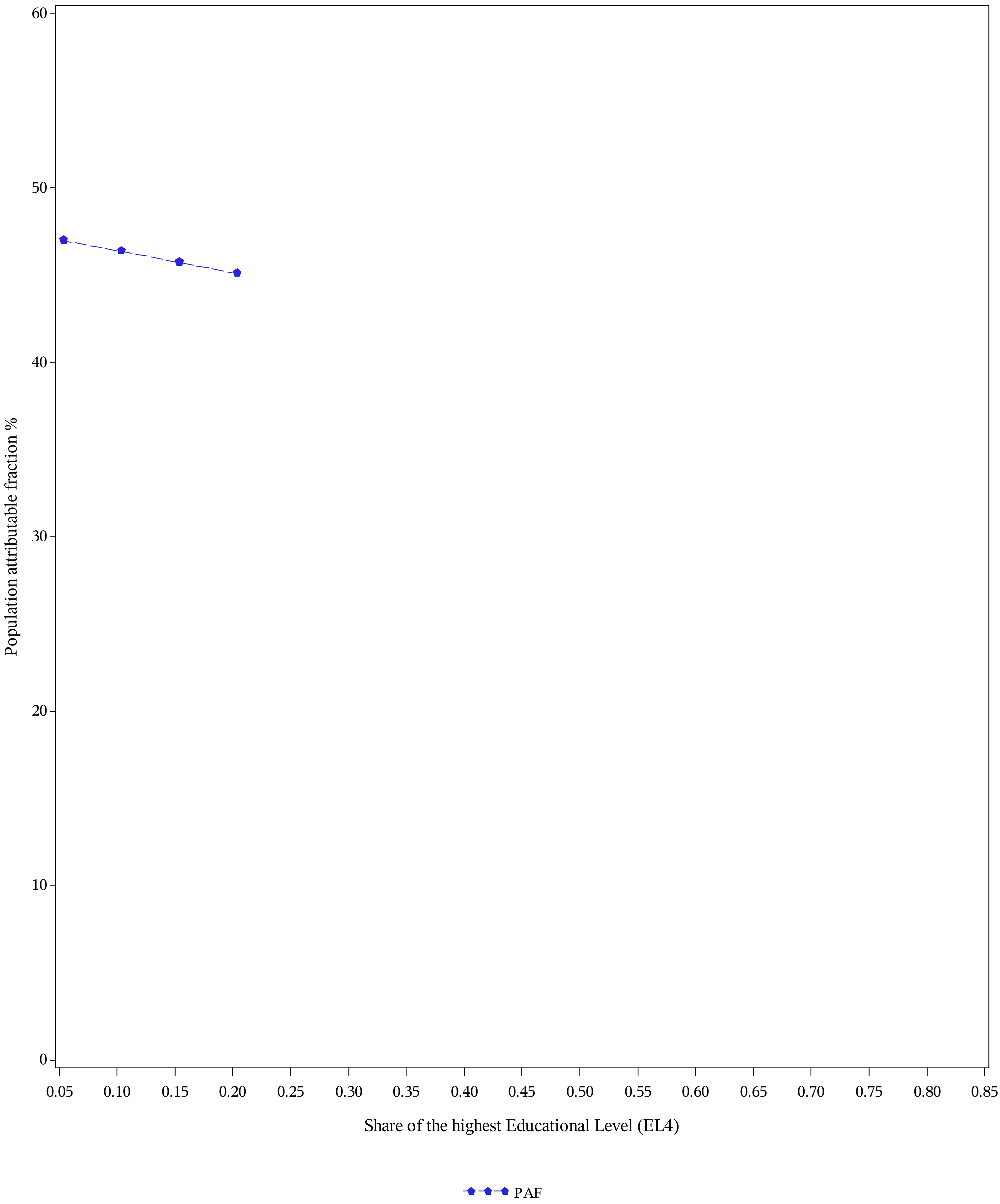

PAF in function of the share of EL4

When EL1 and EL2 are fixed at: EL1=40% ; EL2=40%
EL3 =1- EL4 - EL1 - EL2

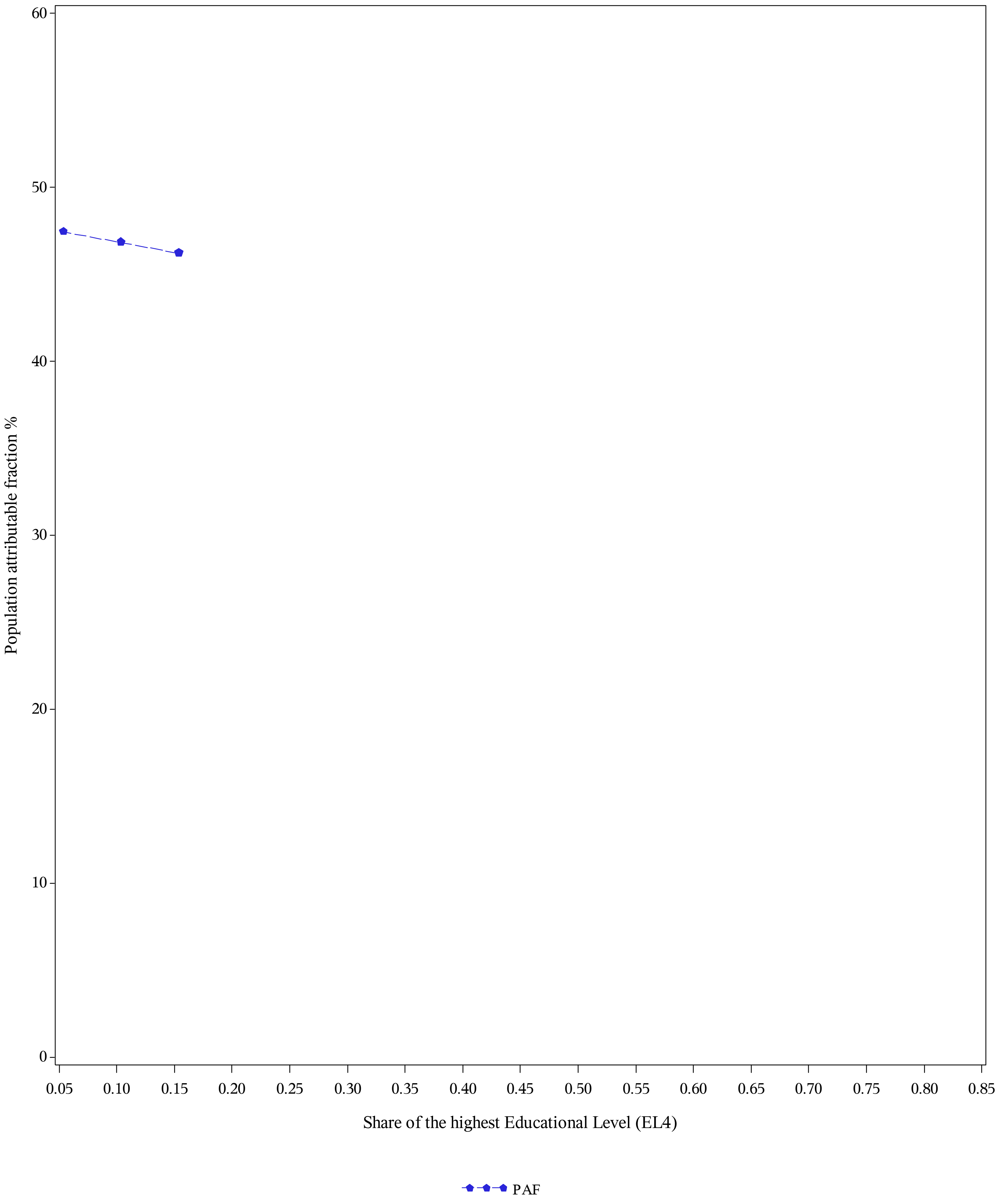

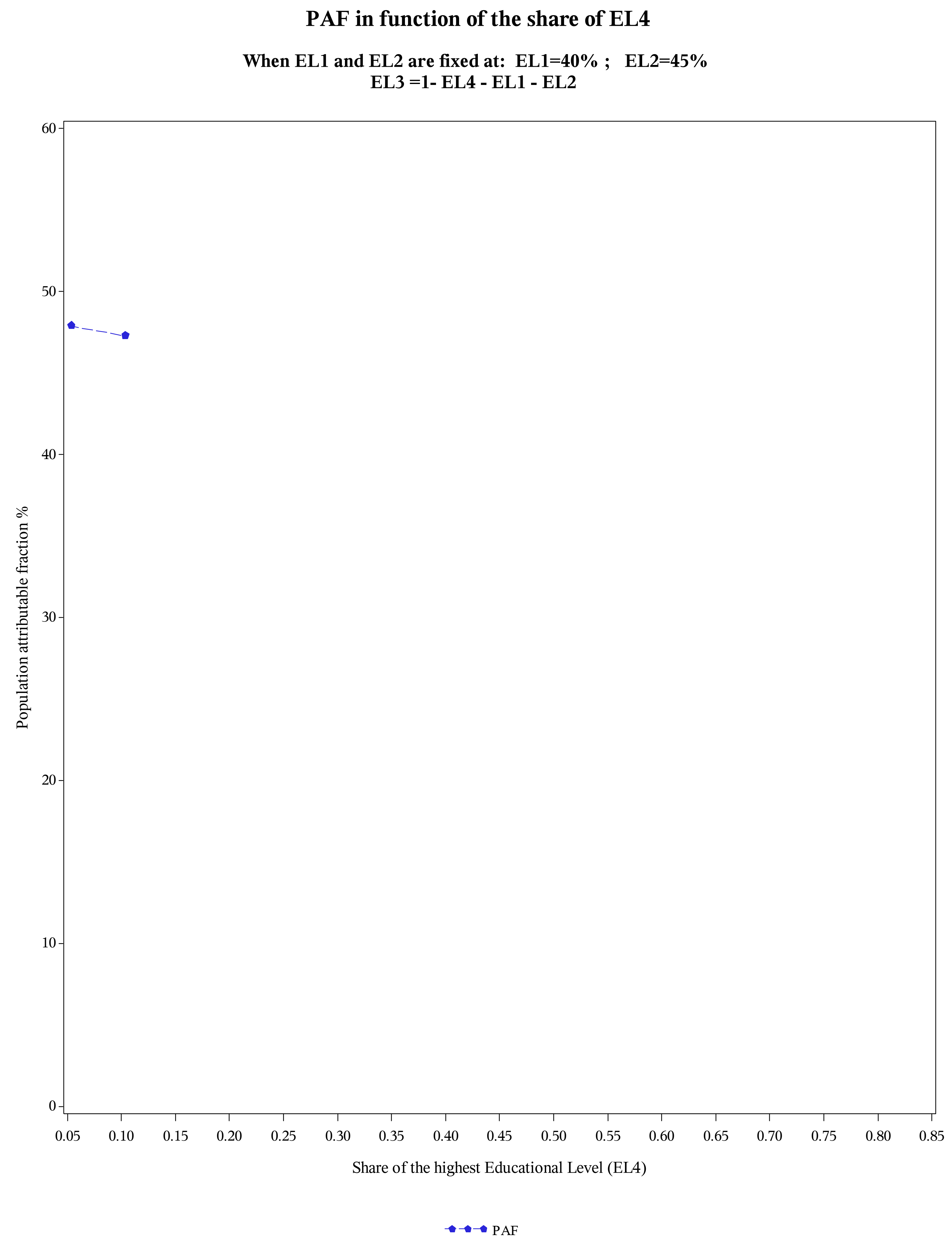

PAF in function of the share of EL4
When EL1 and EL2 are fixed at: EL1=40% ; EL2=45%
EL3 =1- EL4 - EL1 - EL2
Population attributable fraction %
0
10
20
30
40
50
60
0.05
0.10
0.15
0.20
0.25
0.30
0.35
0.40
0.45
0.50
0.55
0.60
0.65
0.70
0.75
0.80
0.85
Share of the highest Educational Level (EL4)
PAF

PAF in function of the share of EL4

When EL1 and EL2 are fixed at: EL1=45% ; EL2=5%
EL3 =1- EL4 - EL1 - EL2

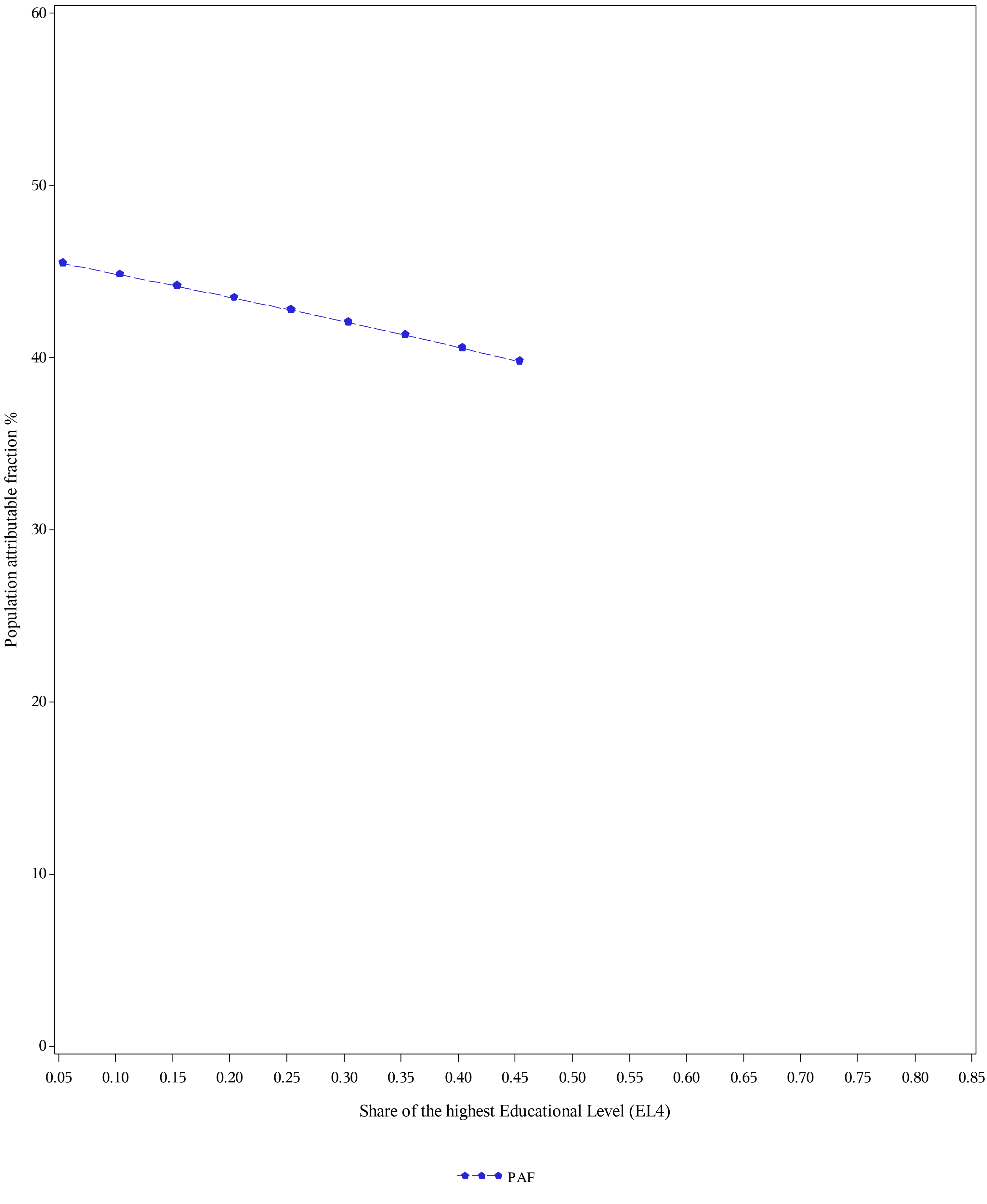

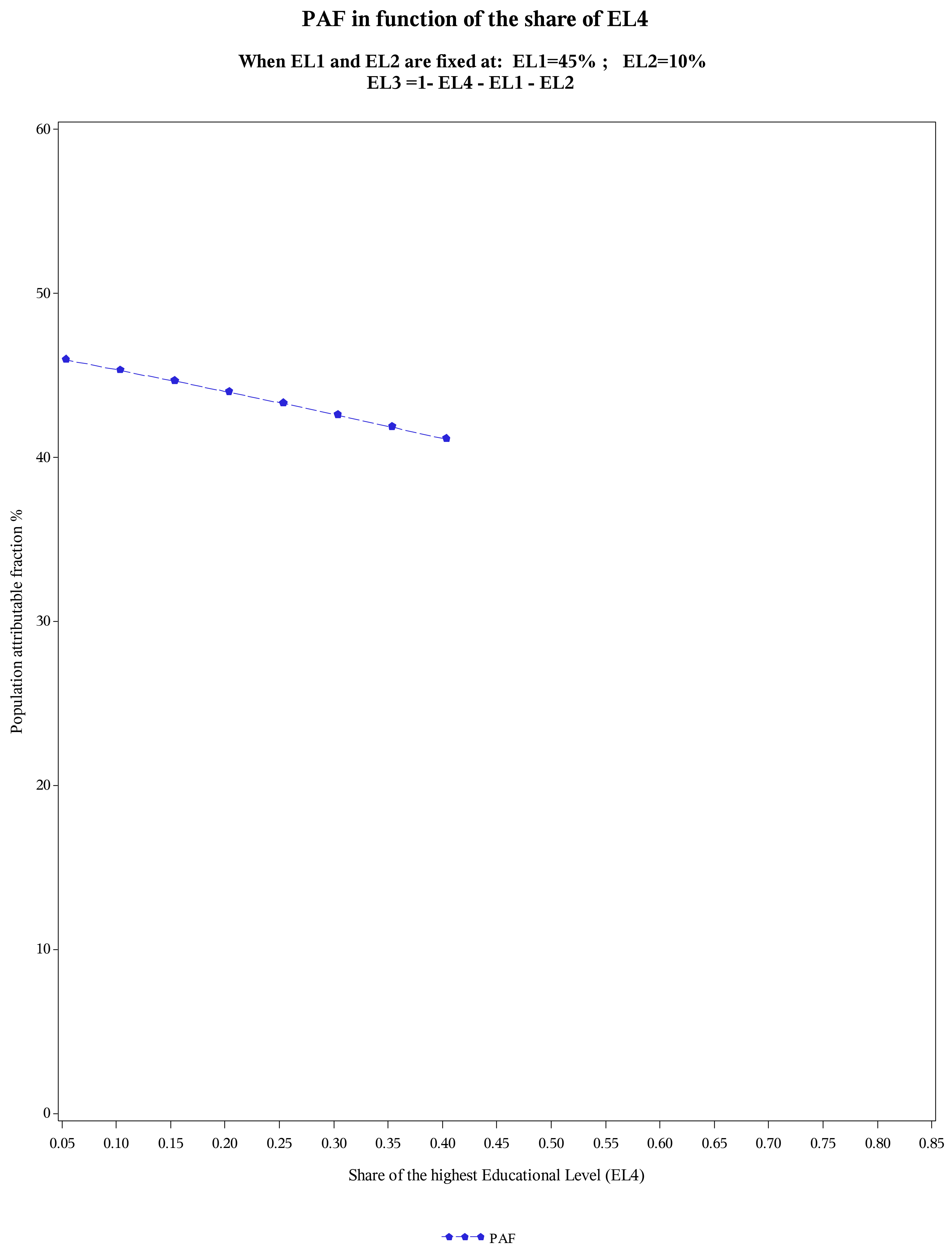

PAF in function of the share of EL4
When EL1 and EL2 are fixed at: EL1=45% ; EL2=10%
EL3 =1- EL4 - EL1 - EL2
Population attributable fraction %
0
10
20
30
40
50
60
0.05
0.10
0.15
0.20
0.25
0.30
0.35
0.40
0.45
0.50
0.55
0.60
0.65
0.70
0.75
0.80
0.85
Share of the highest Educational Level (EL4)
PAF

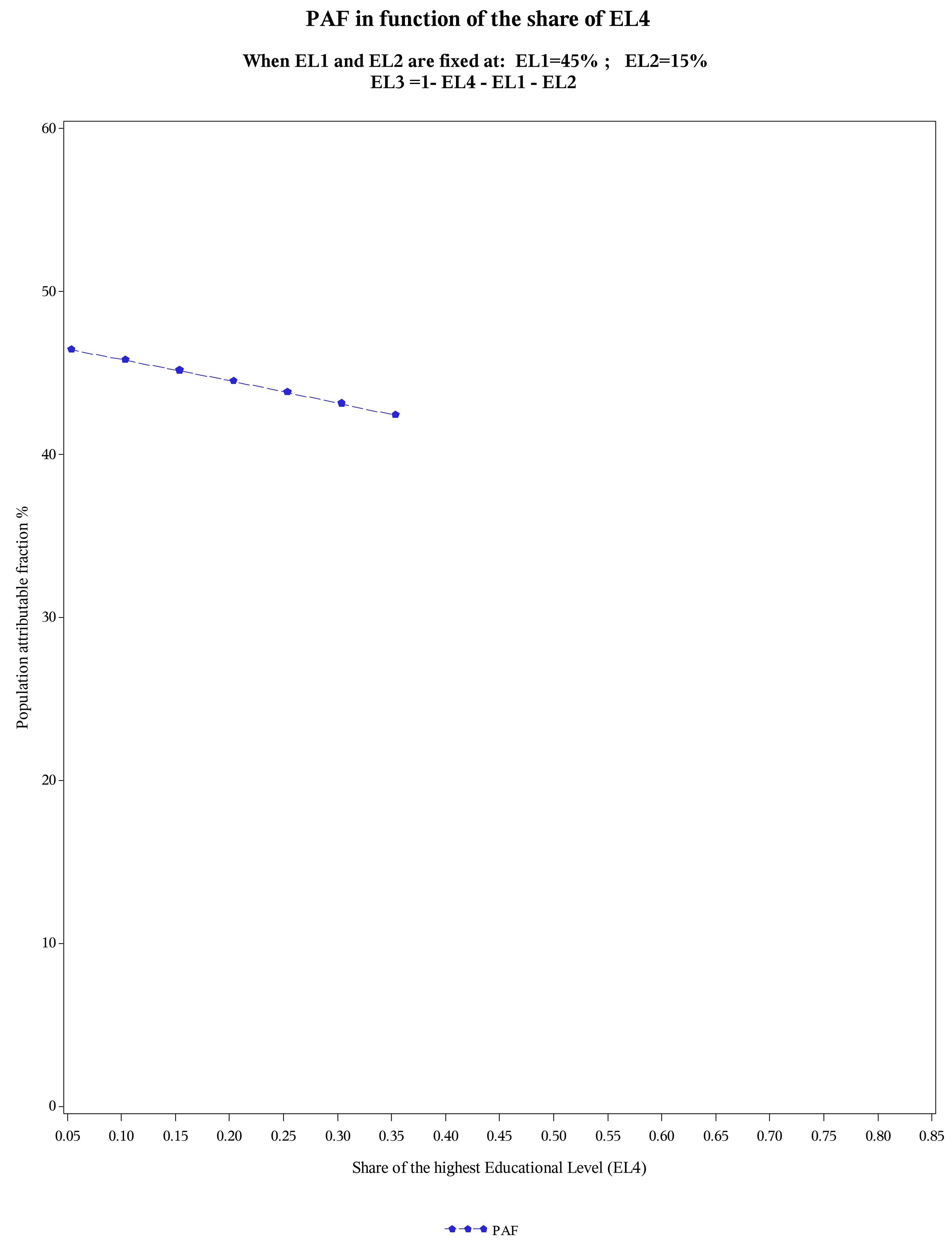
PAF in function of the share of EL4
When EL1 and EL2 are fixed at: EL1=45% ; EL2=15%
EL3 =1- EL4 - EL1 - EL2
Population attributable fraction %
0
10
20
30
40
50
60
0.05
0.10
0.15
0.20
0.25
0.30
0.35
0.40
0.45
0.50
0.55
0.60
0.65
0.70
0.75
0.80
0.85
Share of the highest Educational Level (EL4)
PAF

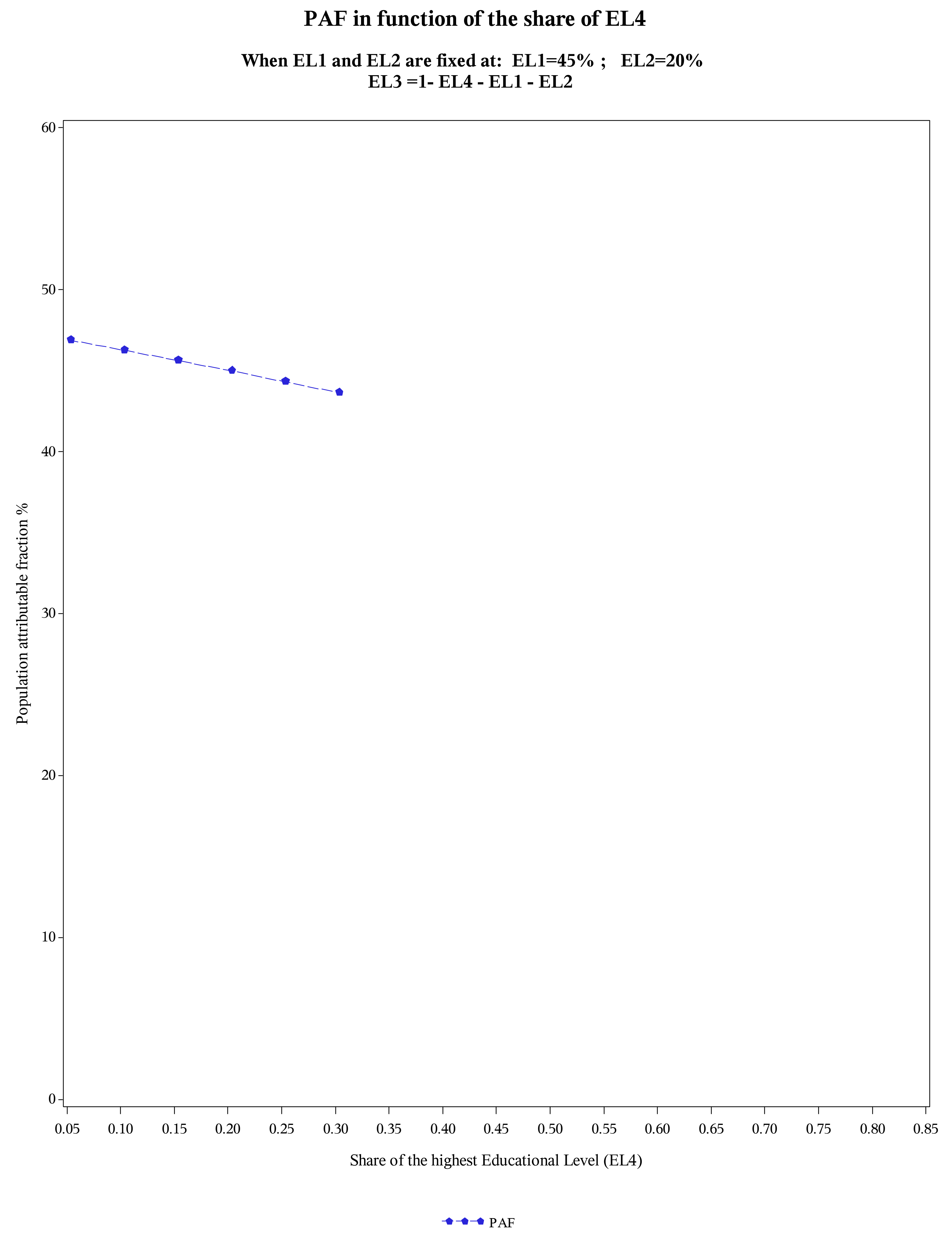
PAF in function of the share of EL4
When EL1 and EL2 are fixed at: EL1=45% ; EL2=20%
EL3 =1- EL4 - EL1 - EL2
Population attributable fraction %
0
10
20
30
40
50
60
0.05
0.10
0.15
0.20
0.25
0.30
0.35
0.40
0.45
0.50
0.55
0.60
0.65
0.70
0.75
0.80
0.85
Share of the highest Educational Level (EL4)
PAF

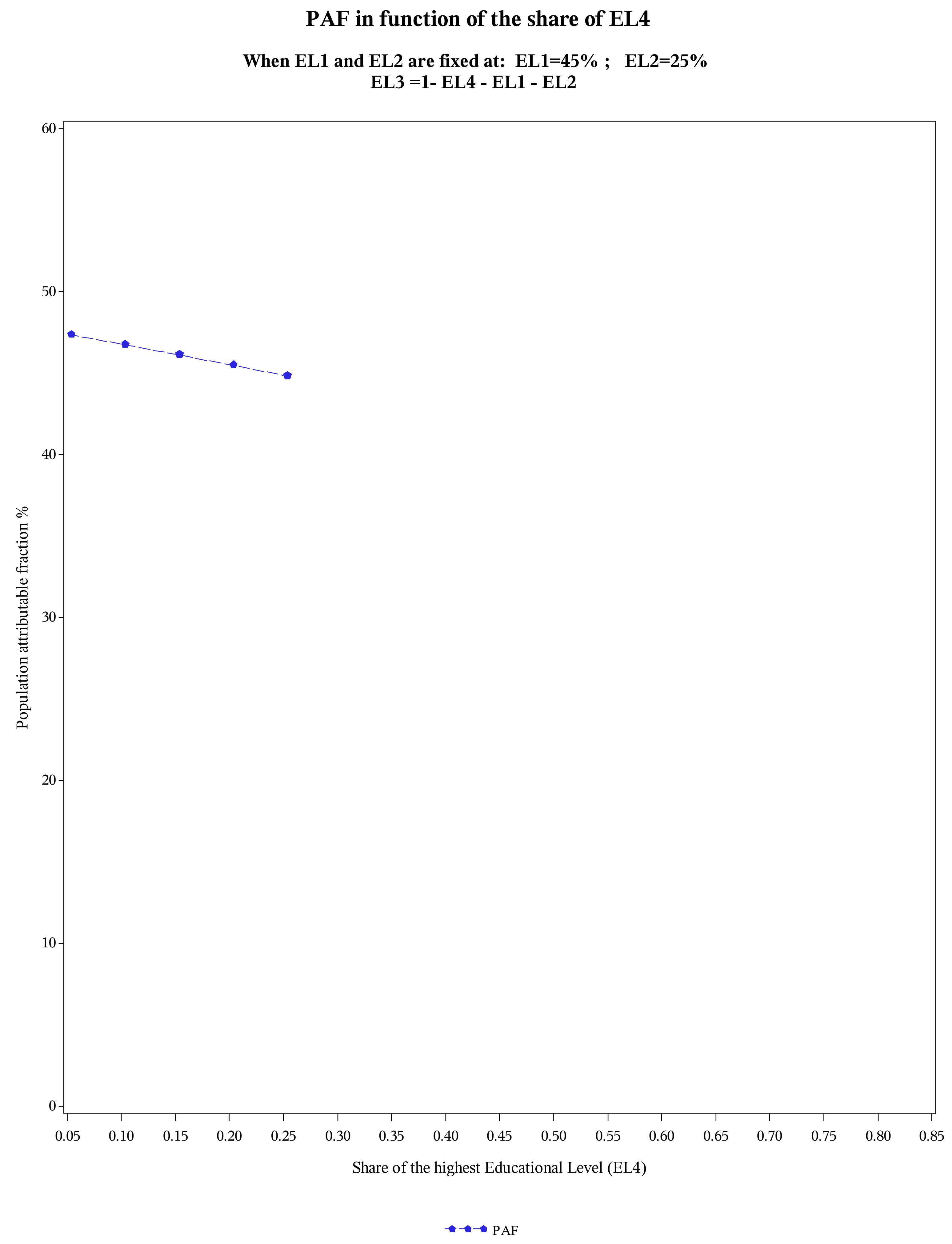
PAF in function of the share of EL4
When EL1 and EL2 are fixed at: EL1=45% ; EL2=25%
EL3 =1- EL4 - EL1 - EL2
Population attributable fraction %
0
10
20
30
40
50
60
0.05
0.10
0.15
0.20
0.25
0.30
0.35
0.40
0.45
0.50
0.55
0.60
0.65
0.70
0.75
0.80
0.85
Share of the highest Educational Level (EL4)
PAF

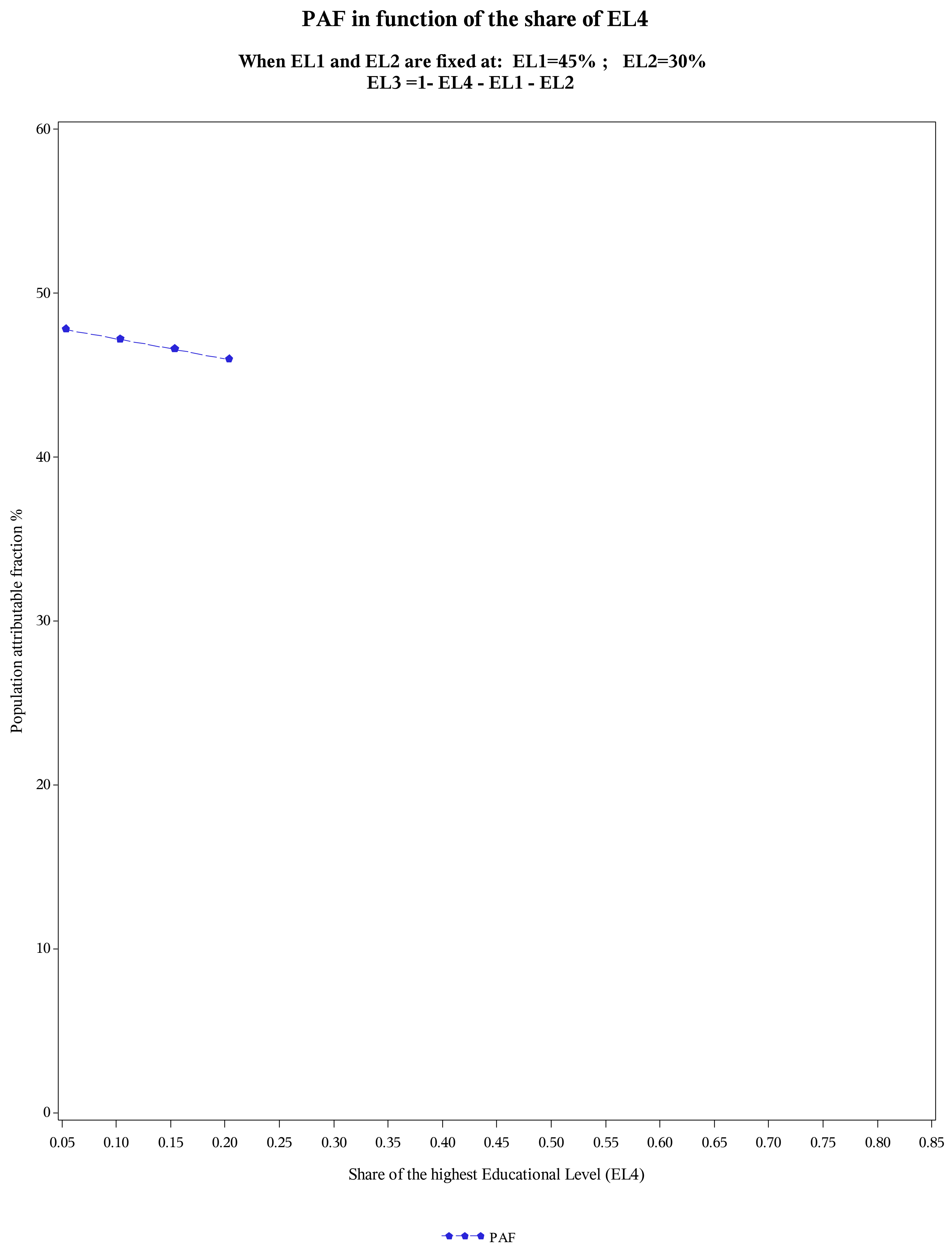
PAF in function of the share of EL4
When EL1 and EL2 are fixed at: EL1=45% ; EL2=30%
EL3 =1- EL4 - EL1 - EL2
Population attributable fraction %
0
10
20
30
40
50
60
0.05
0.10
0.15
0.20
0.25
0.30
0.35
0.40
0.45
0.50
0.55
0.60
0.65
0.70
0.75
0.80
0.85
Share of the highest Educational Level (EL4)
PAF

PAF in function of the share of EL4

When EL1 and EL2 are fixed at: EL1=45% ; EL2=35%
EL3 =1- EL4 - EL1 - EL2

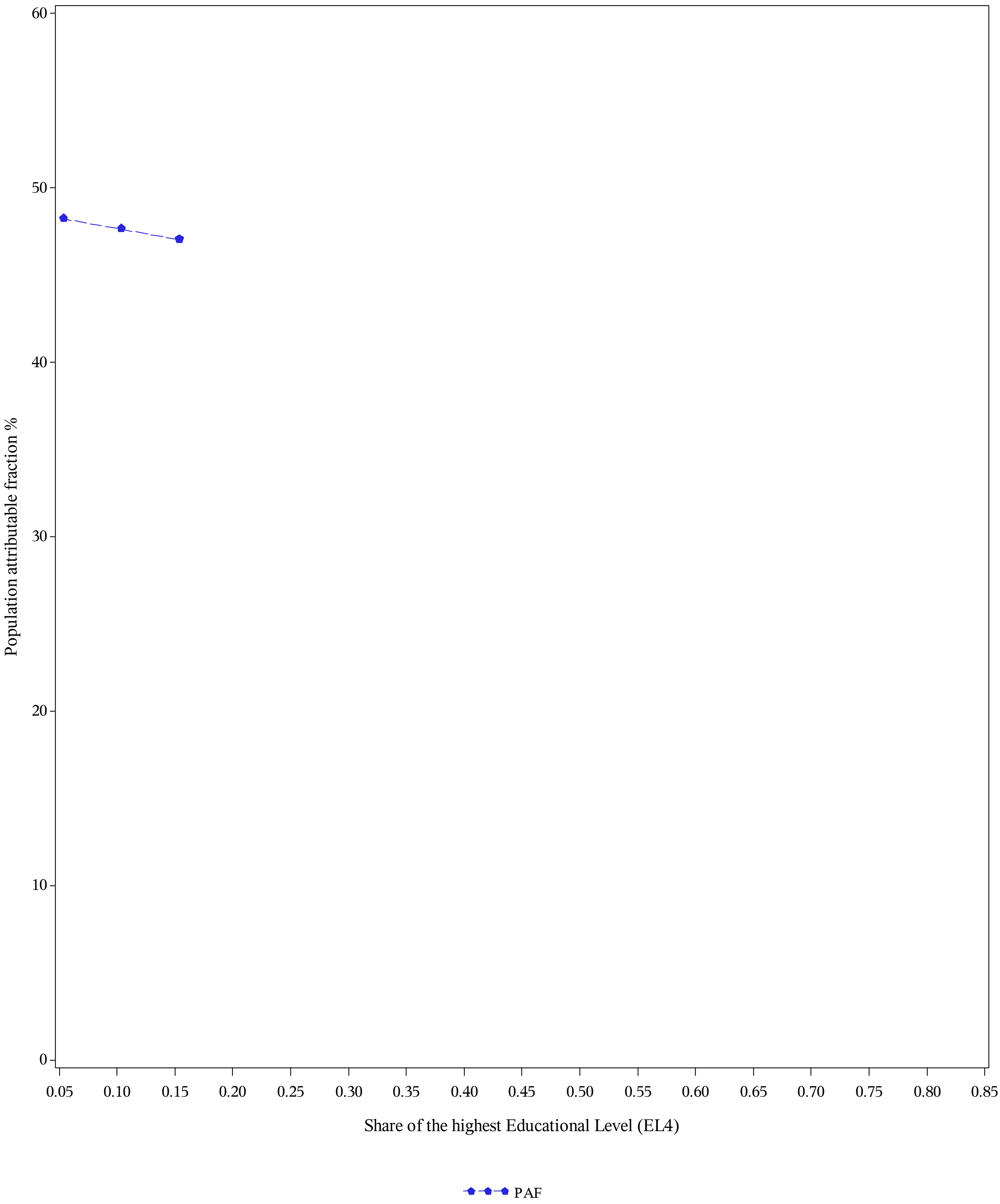

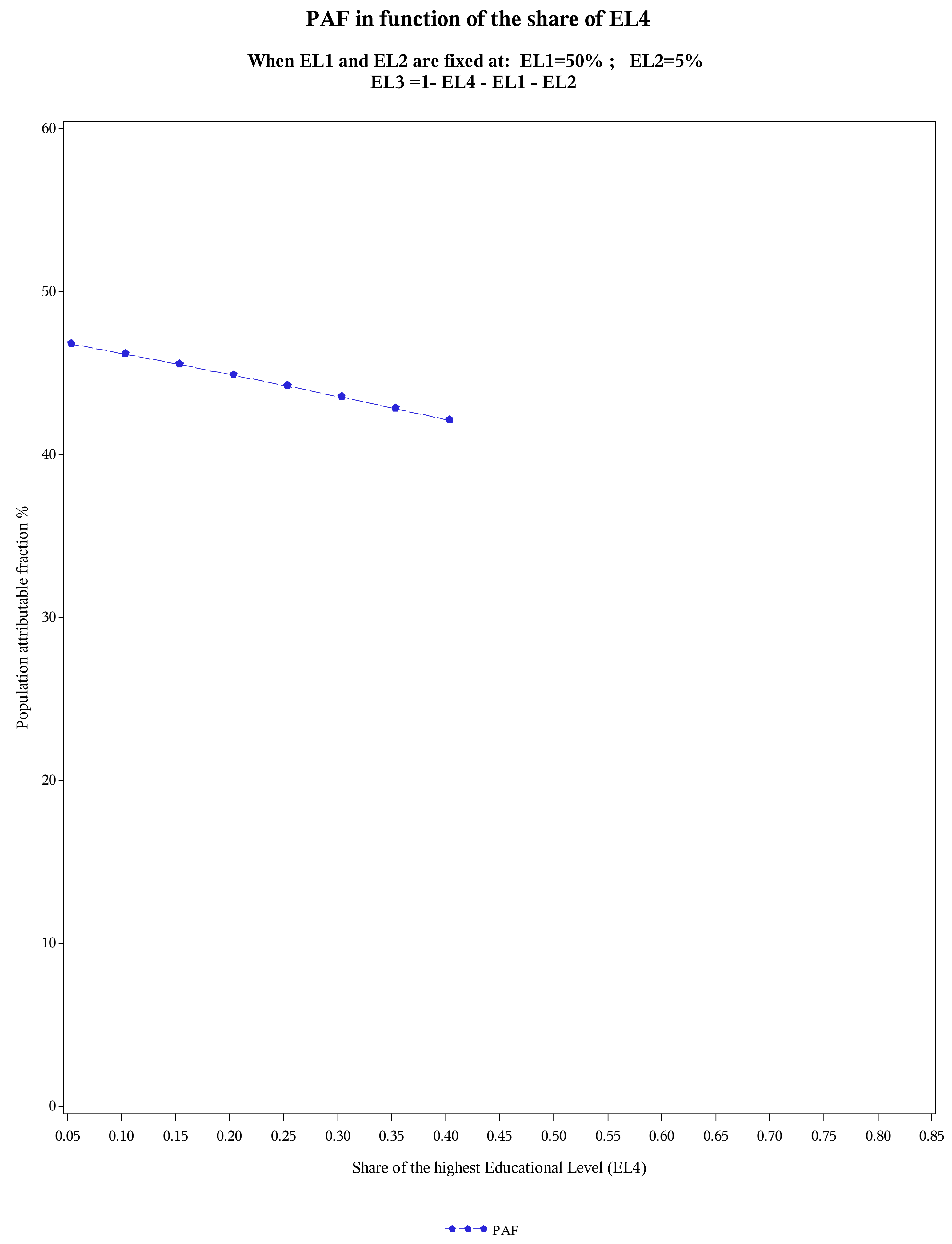
PAF in function of the share of EL4
When EL1 and EL2 are fixed at: EL1=50% ; EL2=5%
EL3 =1- EL4 - EL1 - EL2
Population attributable fraction %
0
10
20
30
40
50
60
0.05
0.10
0.15
0.20
0.25
0.30
0.35
0.40
0.45
0.50
0.55
0.60
0.65
0.70
0.75
0.80
0.85
Share of the highest Educational Level (EL4)
PAF

PAF in function of the share of EL4

When EL1 and EL2 are fixed at: EL1=50% ; EL2=10%
EL3 =1- EL4 - EL1 - EL2

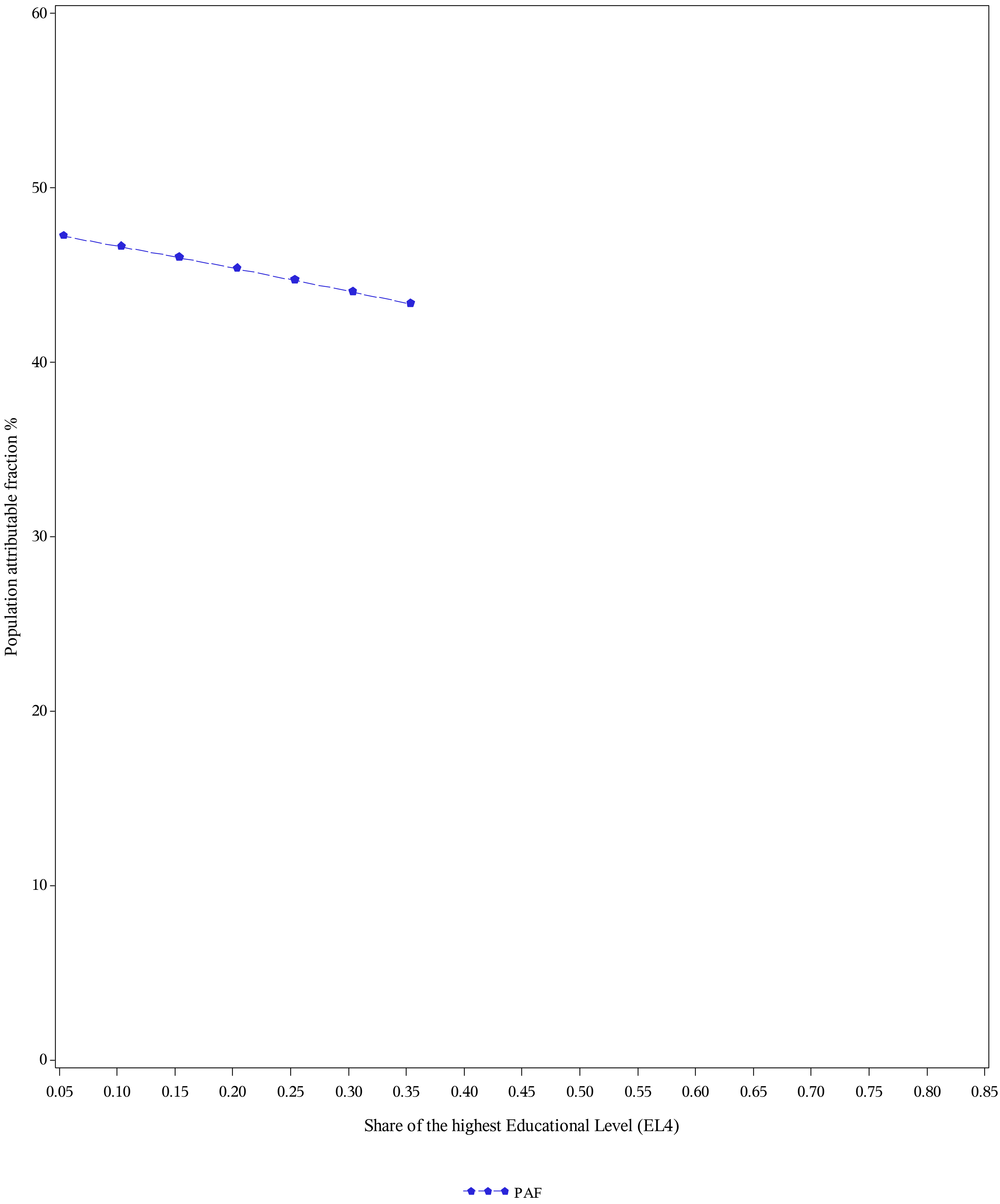

PAF in function of the share of EL4

When EL1 and EL2 are fixed at: EL1=50% ; EL2=15%
EL3 =1- EL4 - EL1 - EL2

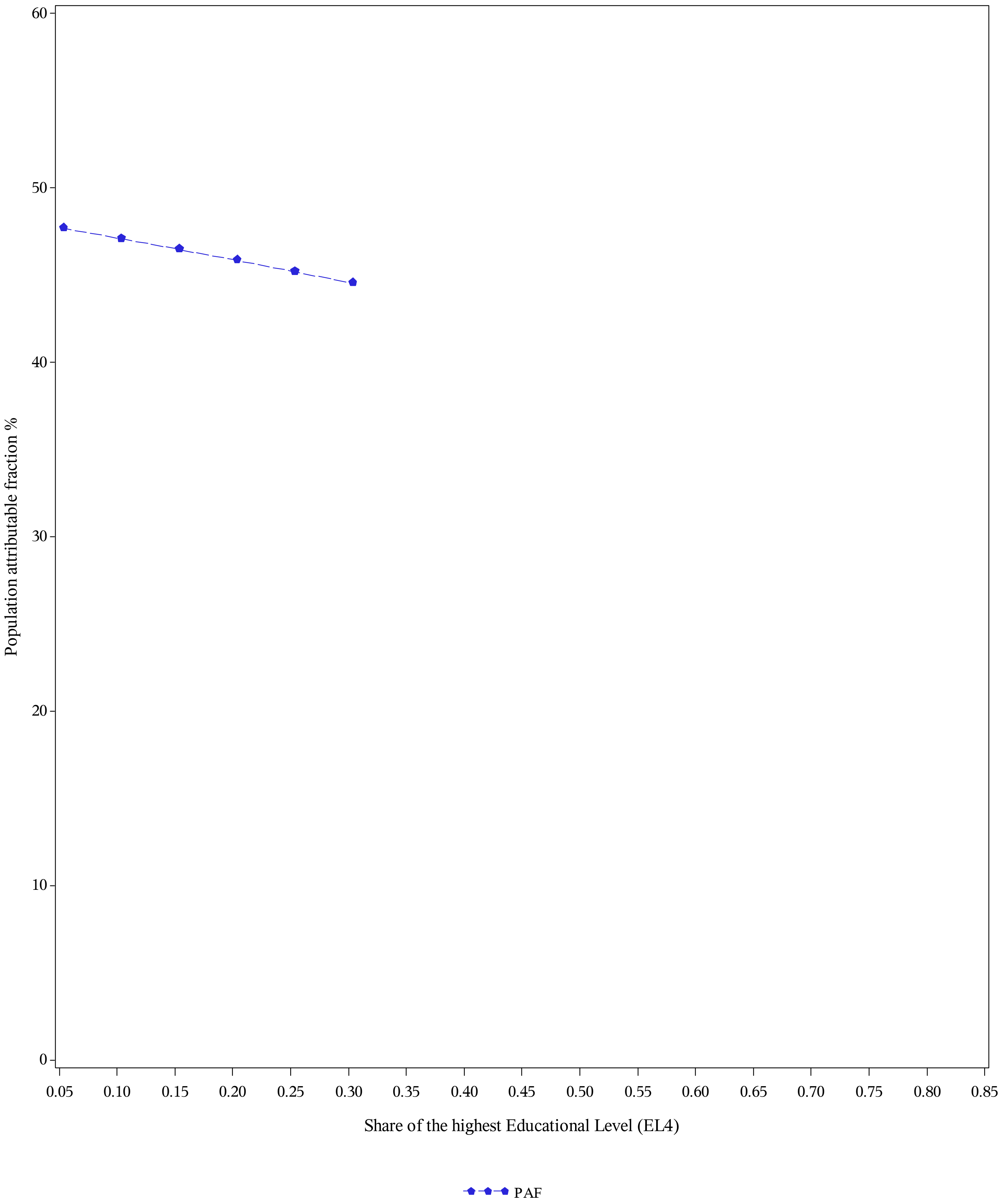

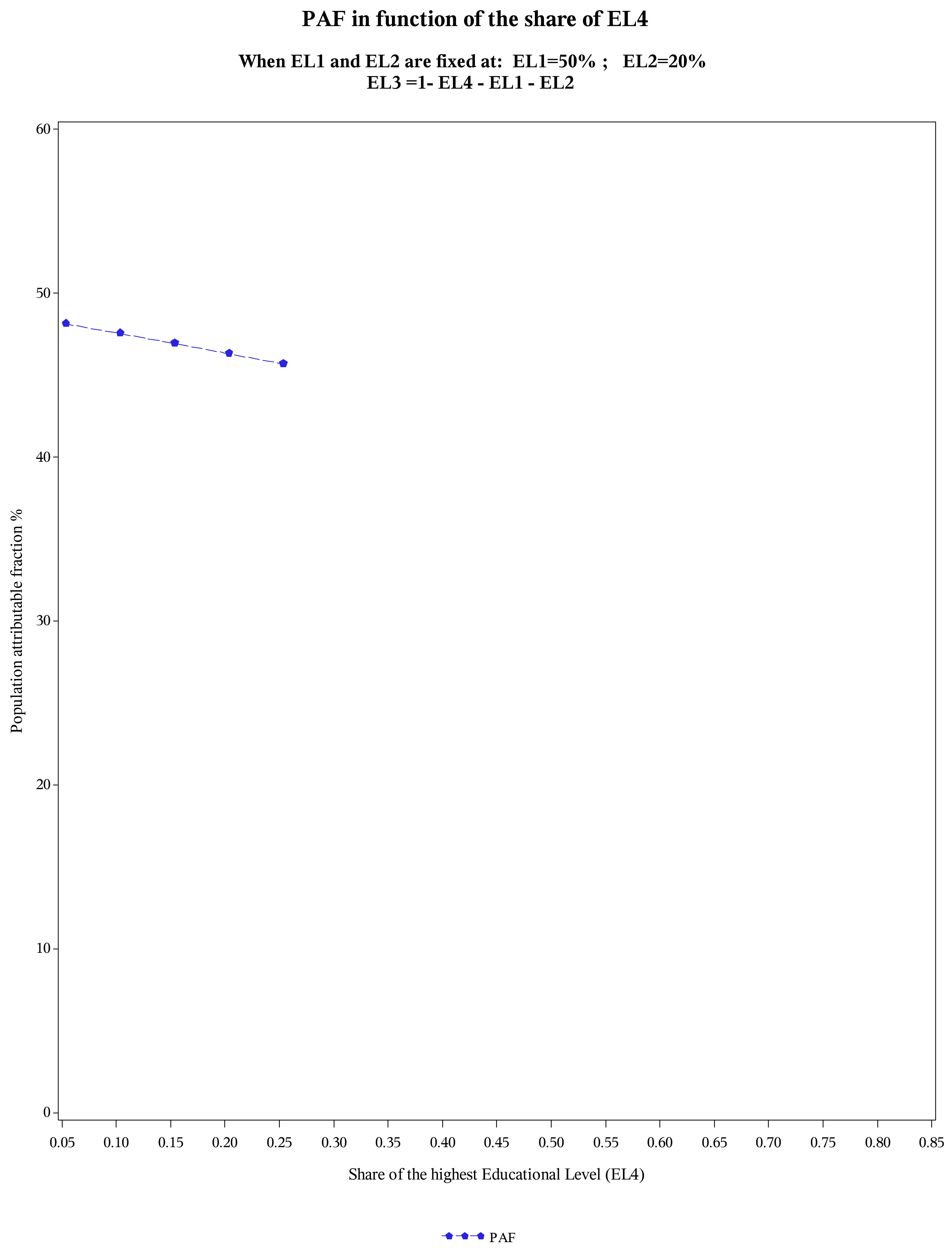

PAF in function of the share of EL4
When EL1 and EL2 are fixed at: EL1=50% ; EL2=20%
EL3 =1- EL4 - EL1 - EL2
Population attributable fraction %
0
10
20
30
40
50
60
0.05
0.10
0.15
0.20
0.25
0.30
0.35
0.40
0.45
0.50
0.55
0.60
0.65
0.70
0.75
0.80
0.85
Share of the highest Educational Level (EL4)
PAF

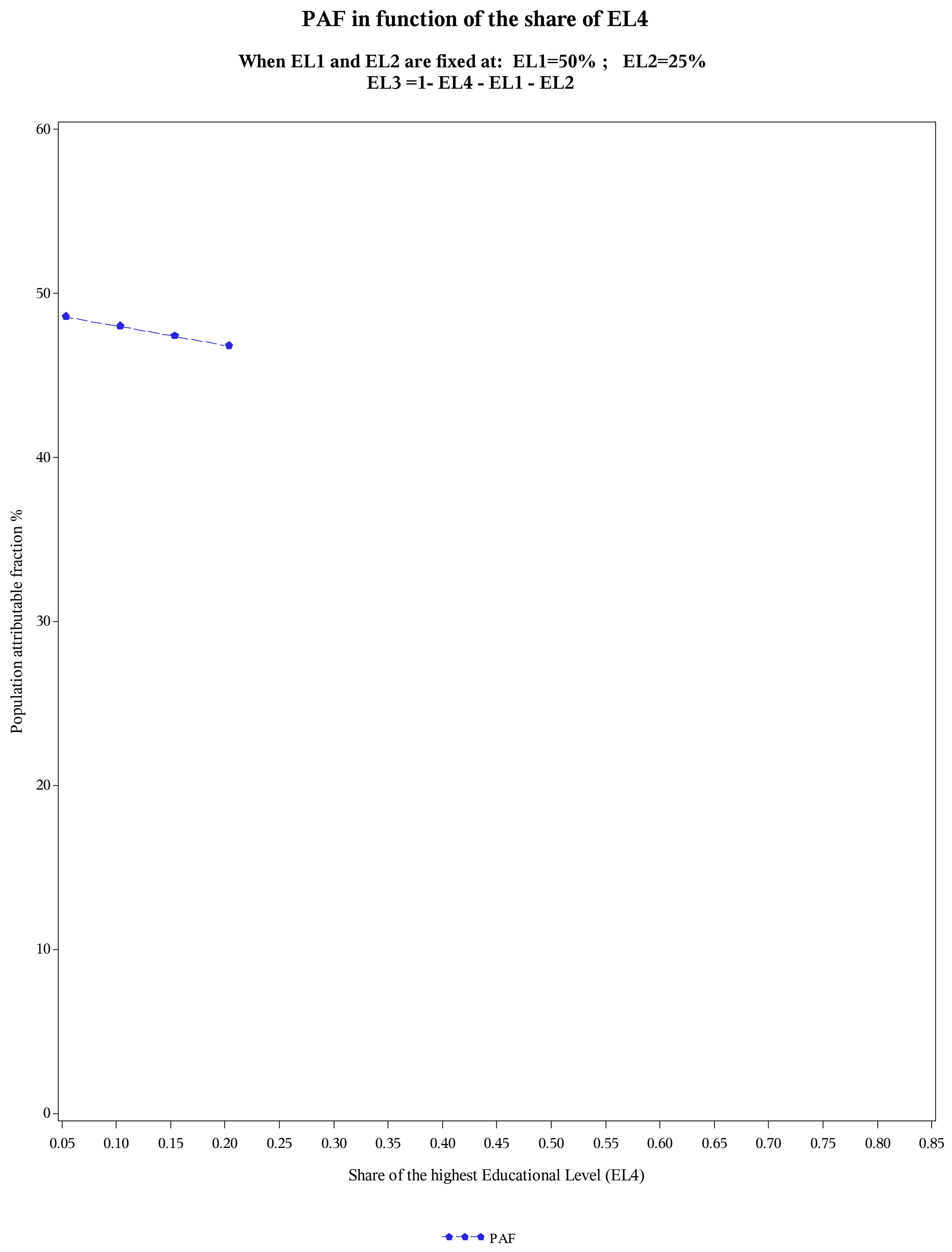
PAF in function of the share of EL4
When EL1 and EL2 are fixed at: EL1=50% ; EL2=25%
EL3 =1- EL4 - EL1 - EL2
Population attributable fraction %
0
10
20
30
40
50
60
0.05
0.10
0.15
0.20
0.25
0.30
0.35
0.40
0.45
0.50
0.55
0.60
0.65
0.70
0.75
0.80
0.85
Share of the highest Educational Level (EL4)
PAF

PAF in function of the share of EL4

When EL1 and EL2 are fixed at: EL1=50% ; EL2=30%
EL3 =1- EL4 - EL1 - EL2

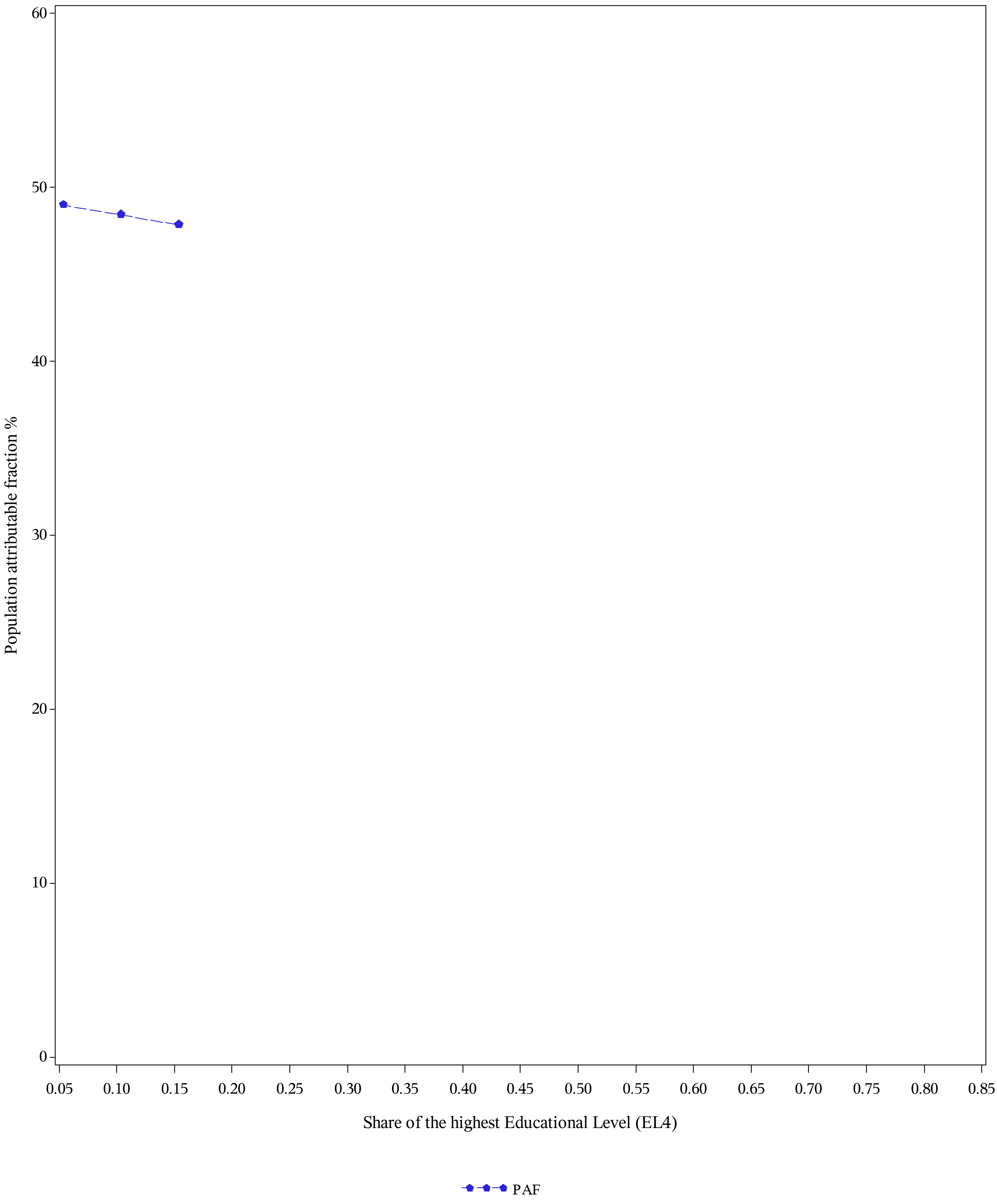

PAF in function of the share of EL4

When EL1 and EL2 are fixed at: EL1=50% ; EL2=35%
EL3 =1- EL4 - EL1 - EL2

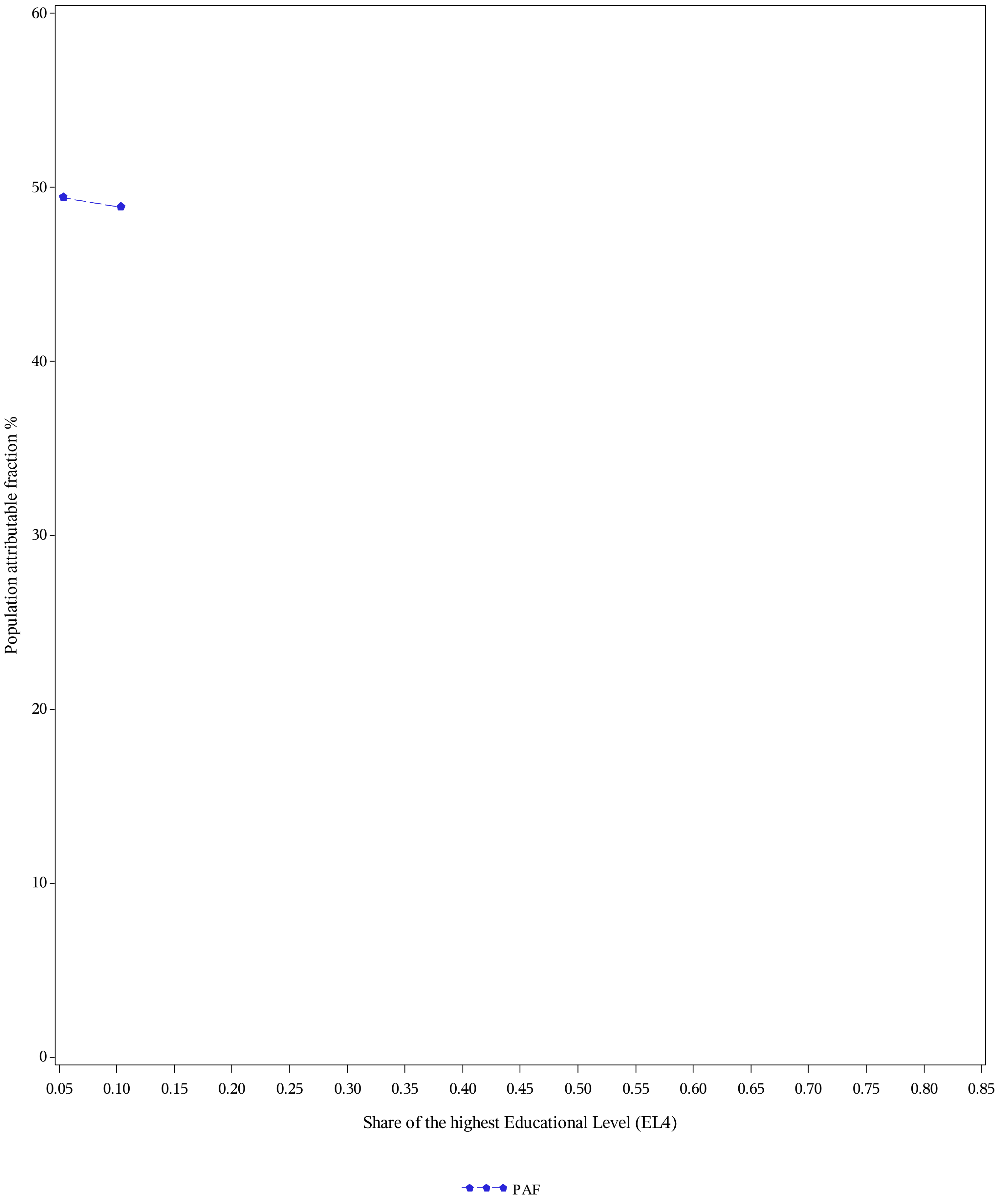

PAF in function of the share of EL4

When EL1 and EL2 are fixed at: EL1=55% ; EL2=5%
EL3 =1- EL4 - EL1 - EL2

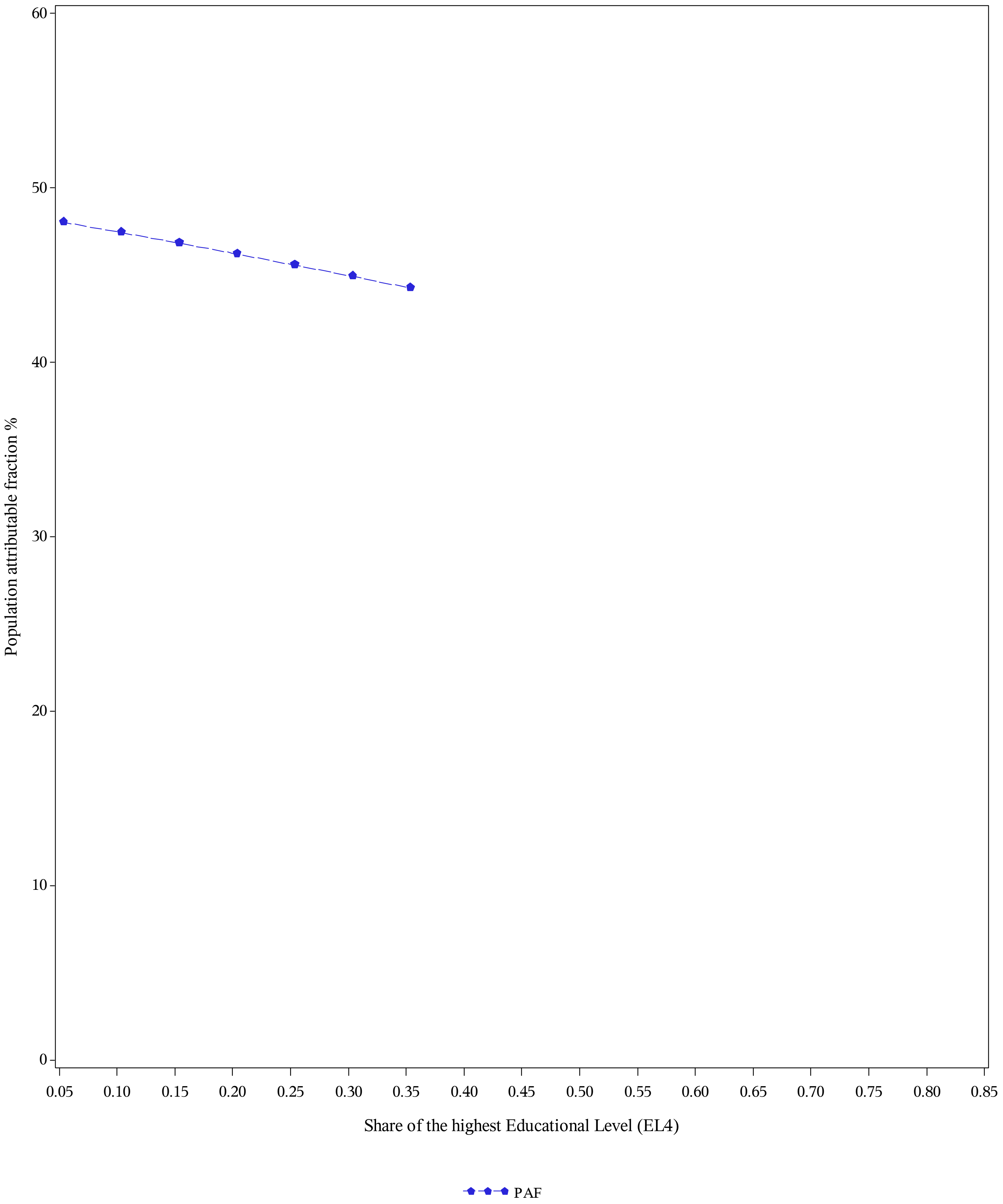

PAF in function of the share of EL4

When EL1 and EL2 are fixed at: EL1=55% ; EL2=10%
EL3 =1- EL4 - EL1 - EL2

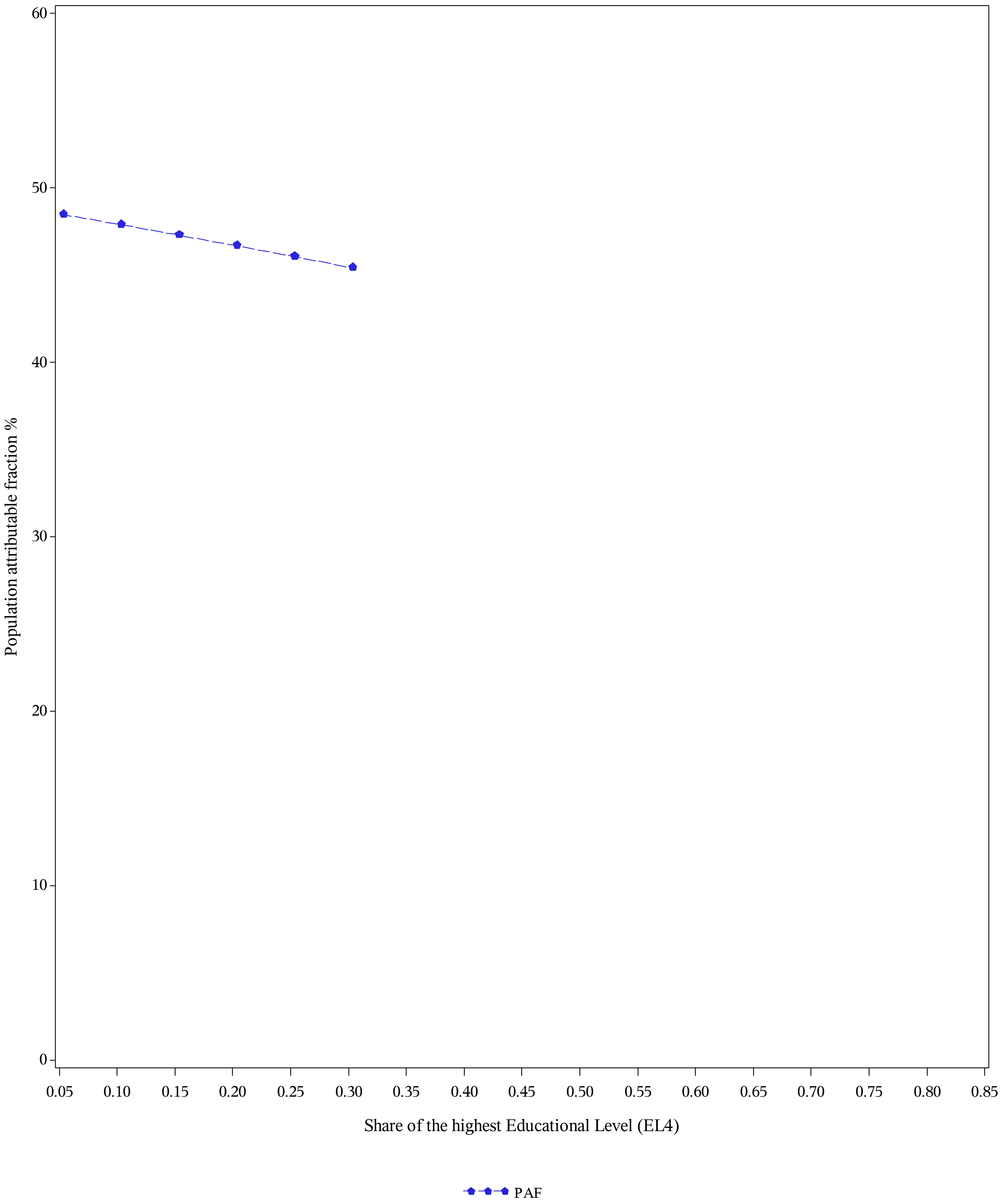

PAF in function of the share of EL4

When EL1 and EL2 are fixed at: EL1=55% ; EL2=15%
EL3 =1- EL4 - EL1 - EL2

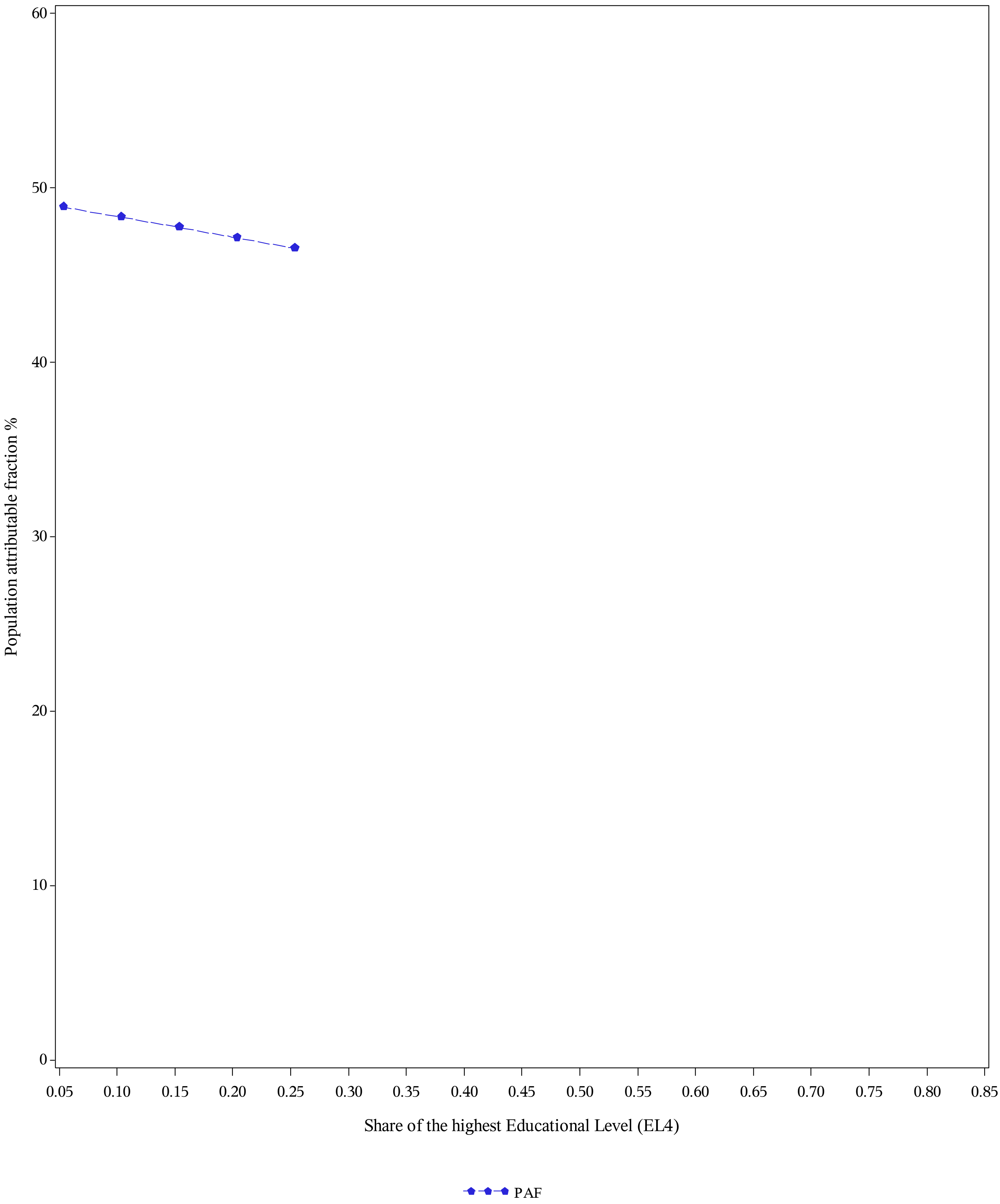

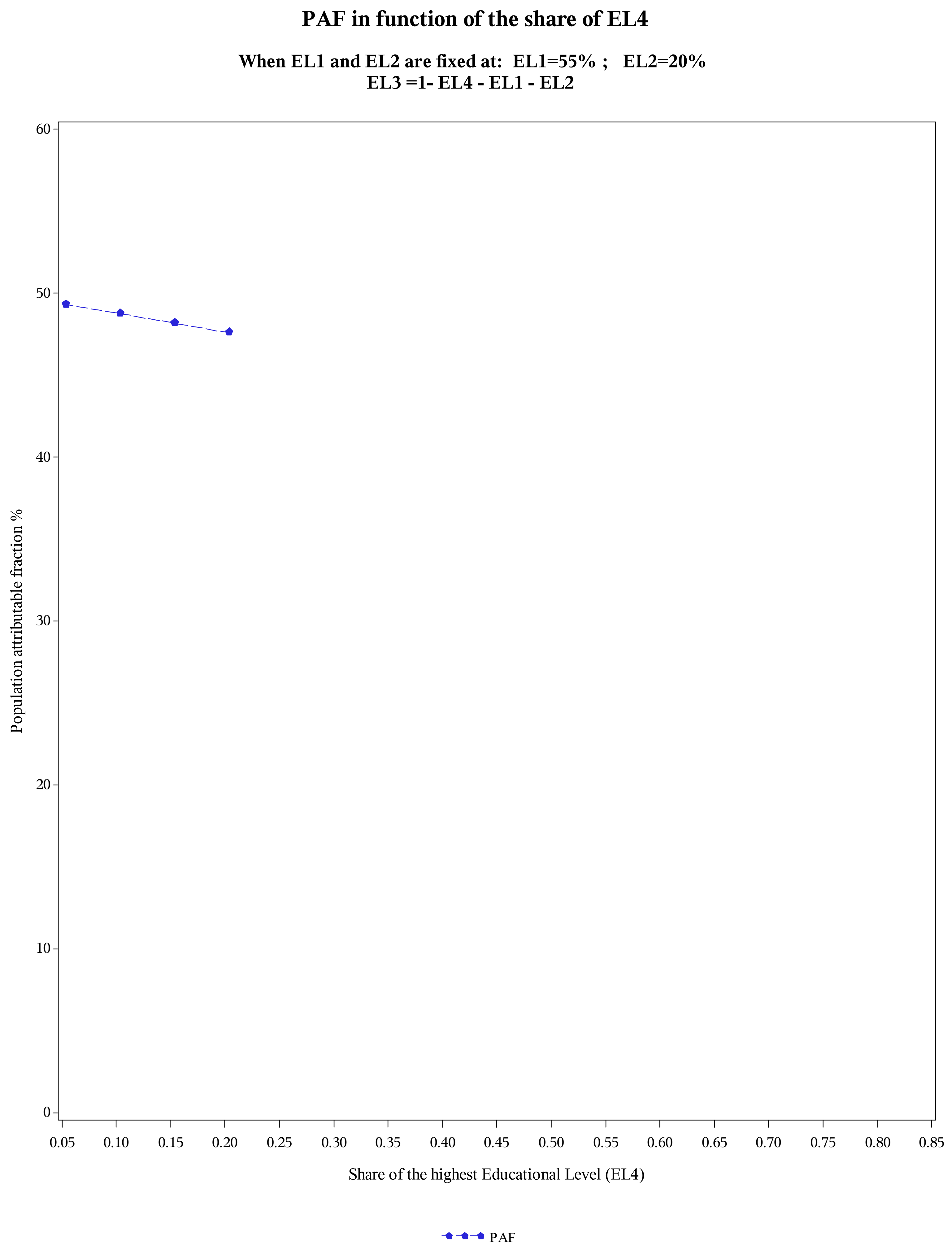
PAF in function of the share of EL4
When EL1 and EL2 are fixed at: EL1=55% ; EL2=20%
EL3 =1- EL4 - EL1 - EL2
Population attributable fraction %
0
10
20
30
40
50
60
0.05
0.10
0.15
0.20
0.25
0.30
0.35
0.40
0.45
0.50
0.55
0.60
0.65
0.70
0.75
0.80
0.85
Share of the highest Educational Level (EL4)
PAF

PAF in function of the share of EL4

When EL1 and EL2 are fixed at: EL1=55% ; EL2=25%
EL3 =1- EL4 - EL1 - EL2

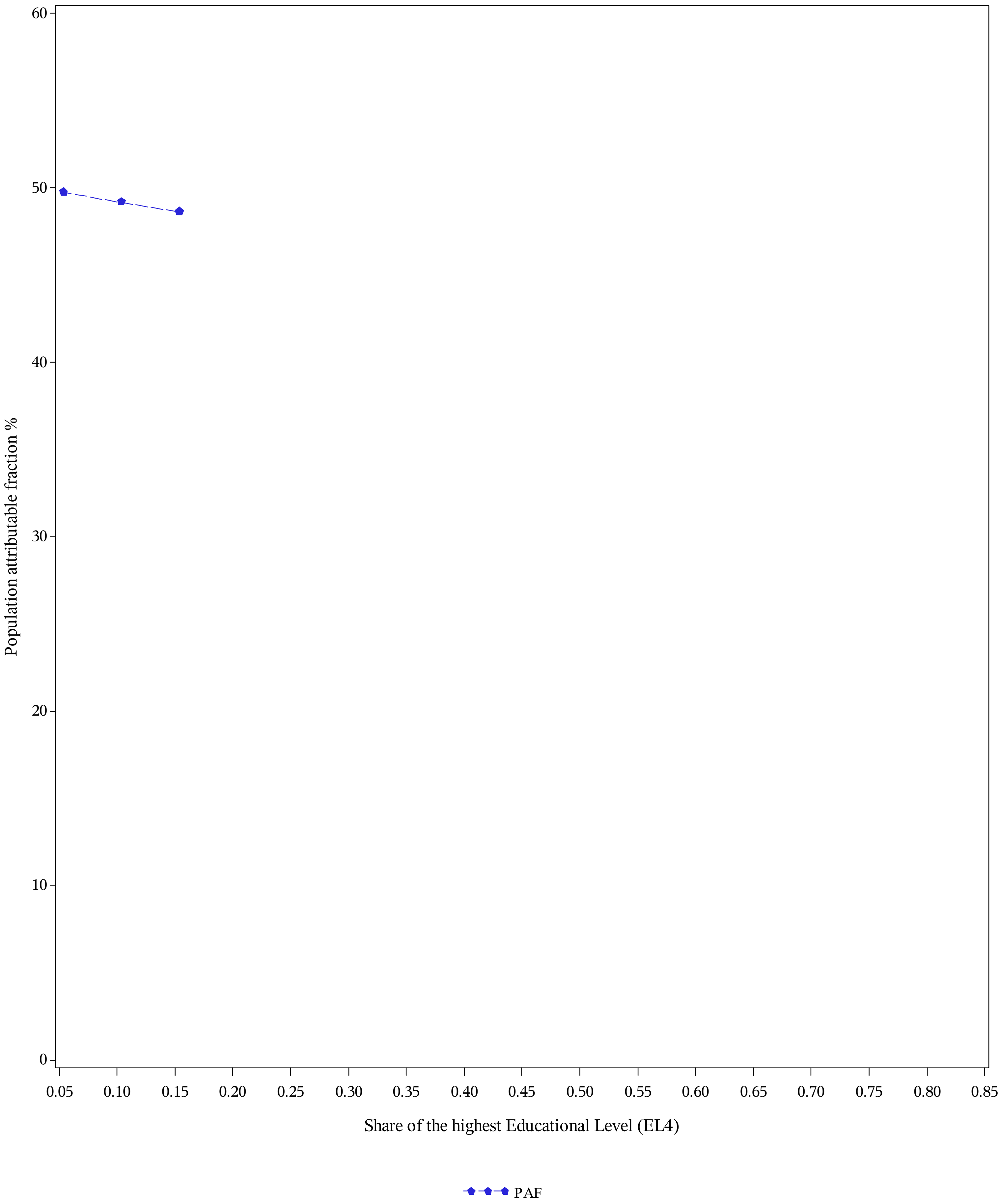

PAF in function of the share of EL4

When EL1 and EL2 are fixed at: EL1=55% ; EL2=30%
EL3 =1- EL4 - EL1 - EL2

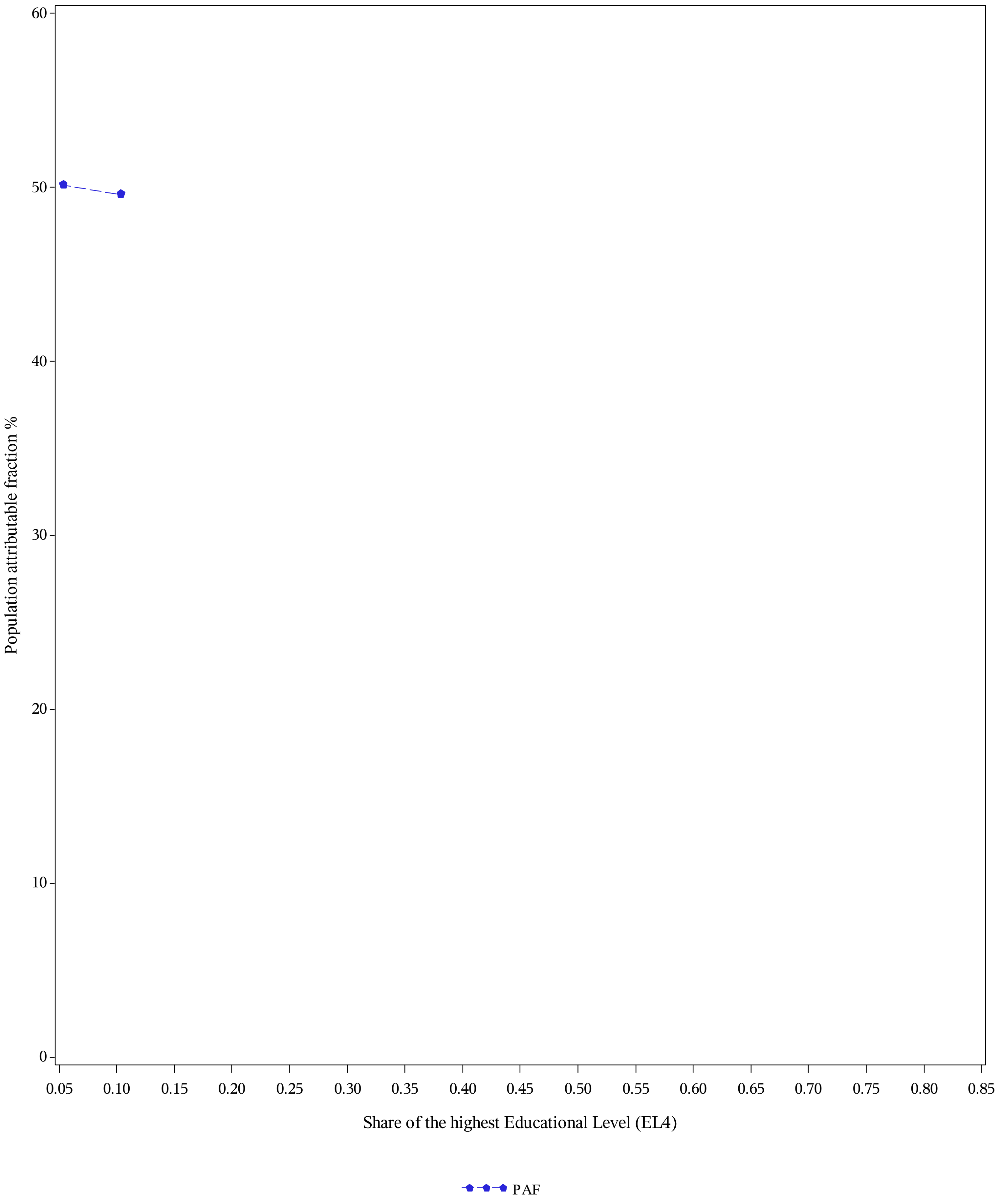

PAF in function of the share of EL4

When EL1 and EL2 are fixed at: EL1=60% ; EL2=5%
EL3 =1- EL4 - EL1 - EL2

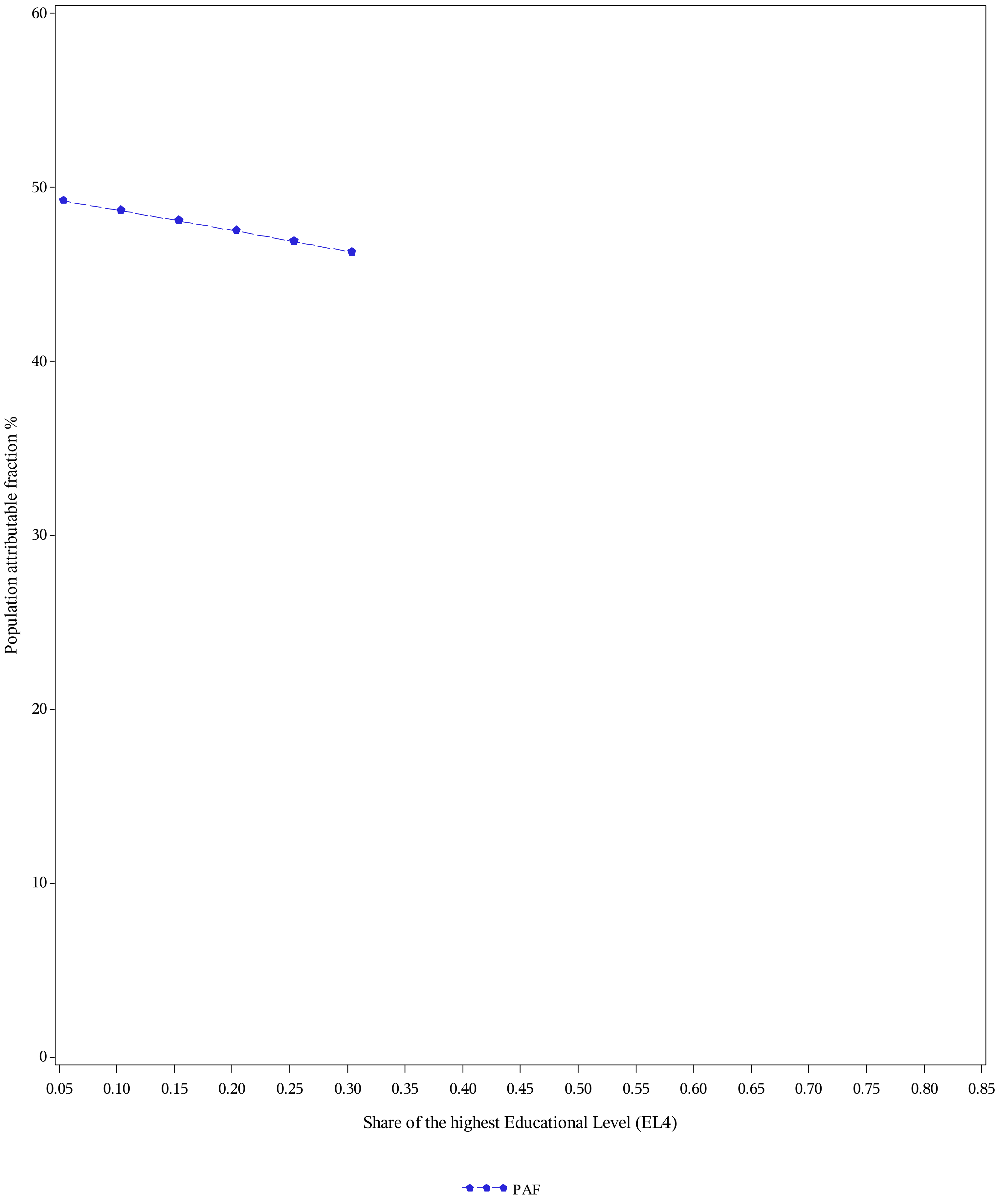

PAF in function of the share of EL4

When EL1 and EL2 are fixed at: EL1=60% ; EL2=10%
EL3 =1- EL4 - EL1 - EL2

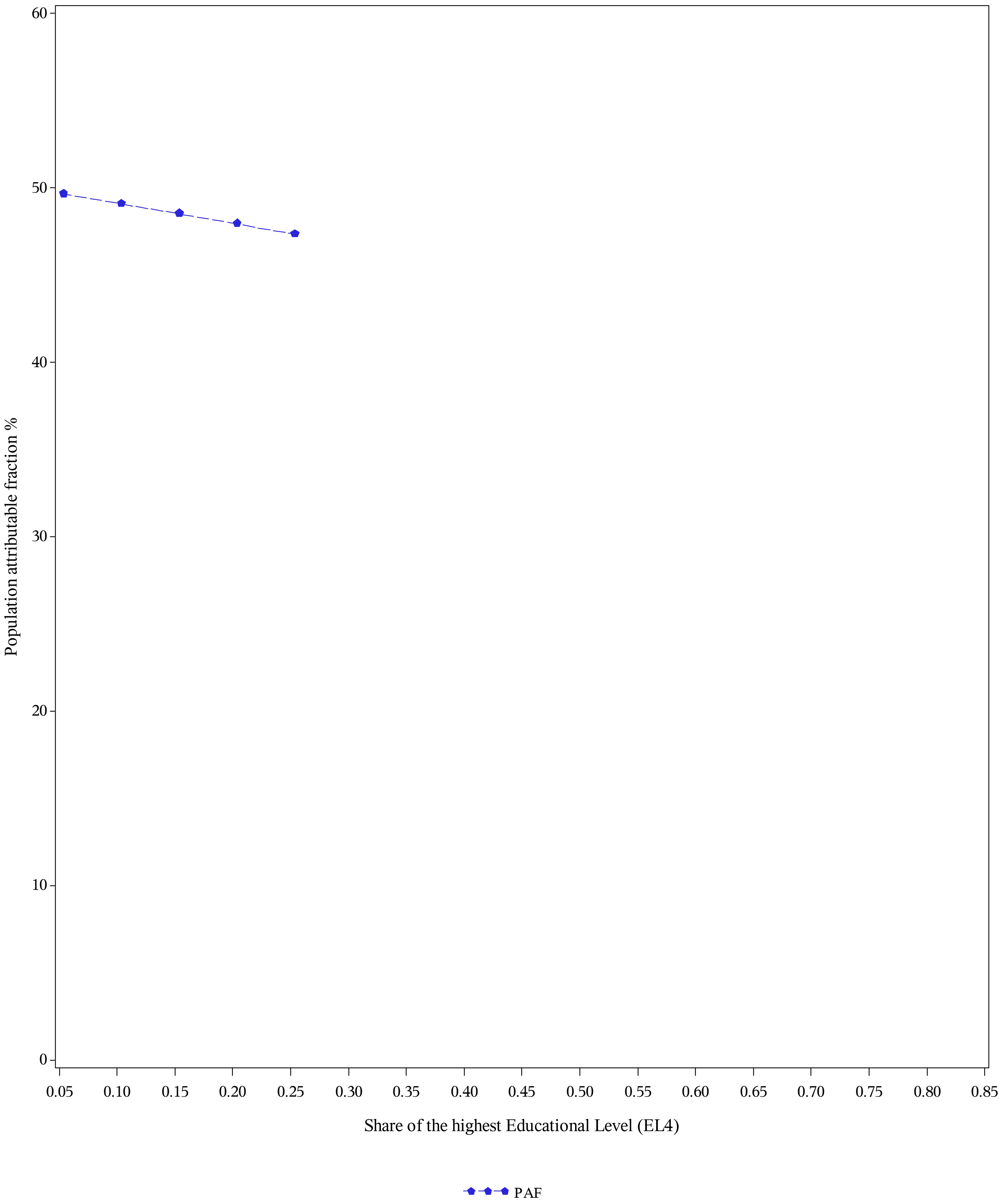

PAF in function of the share of EL4

When EL1 and EL2 are fixed at: EL1=60% ; EL2=15%
EL3 =1- EL4 - EL1 - EL2

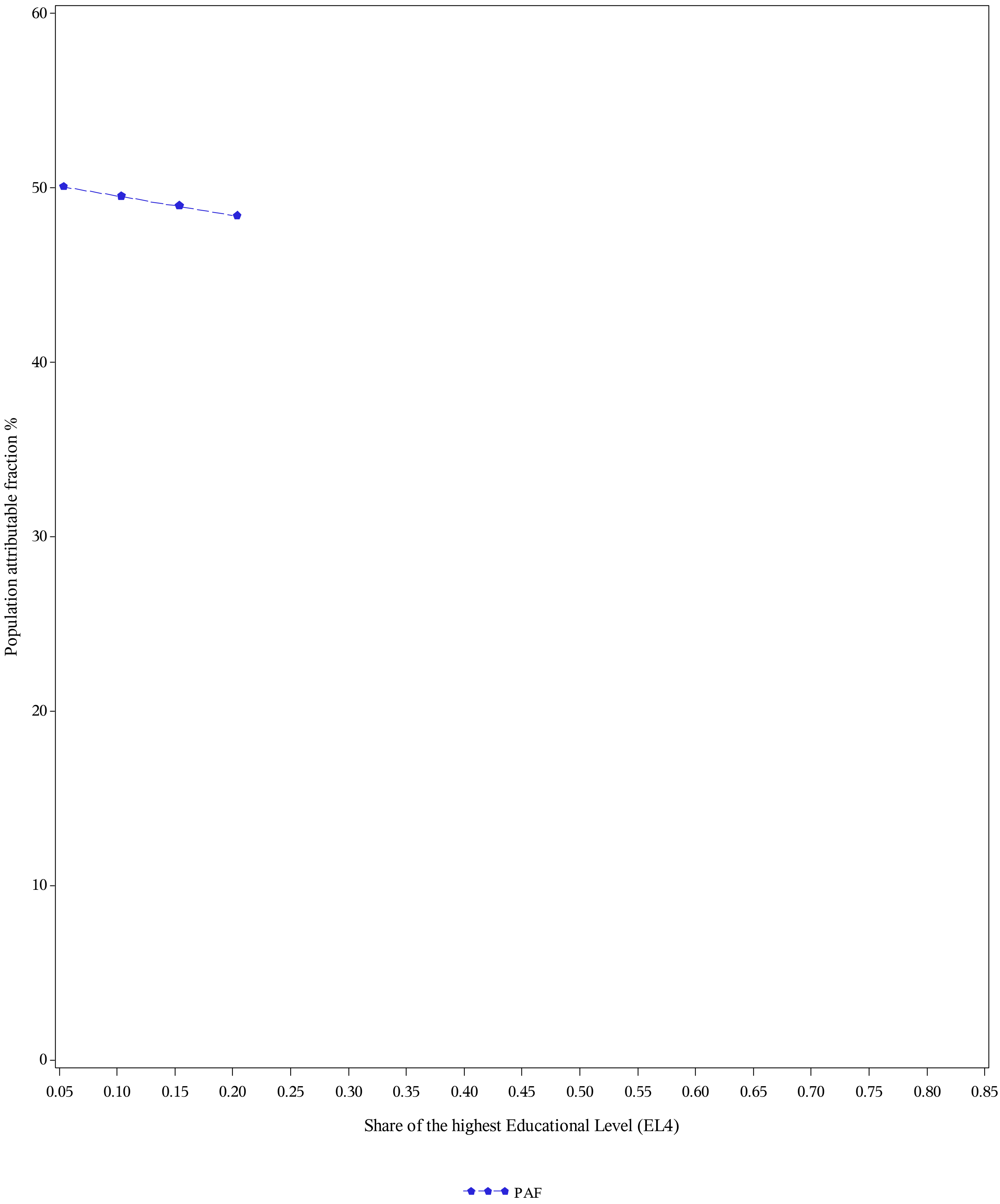

PAF in function of the share of EL4

When EL1 and EL2 are fixed at: EL1=60% ; EL2=20%
EL3 =1- EL4 - EL1 - EL2

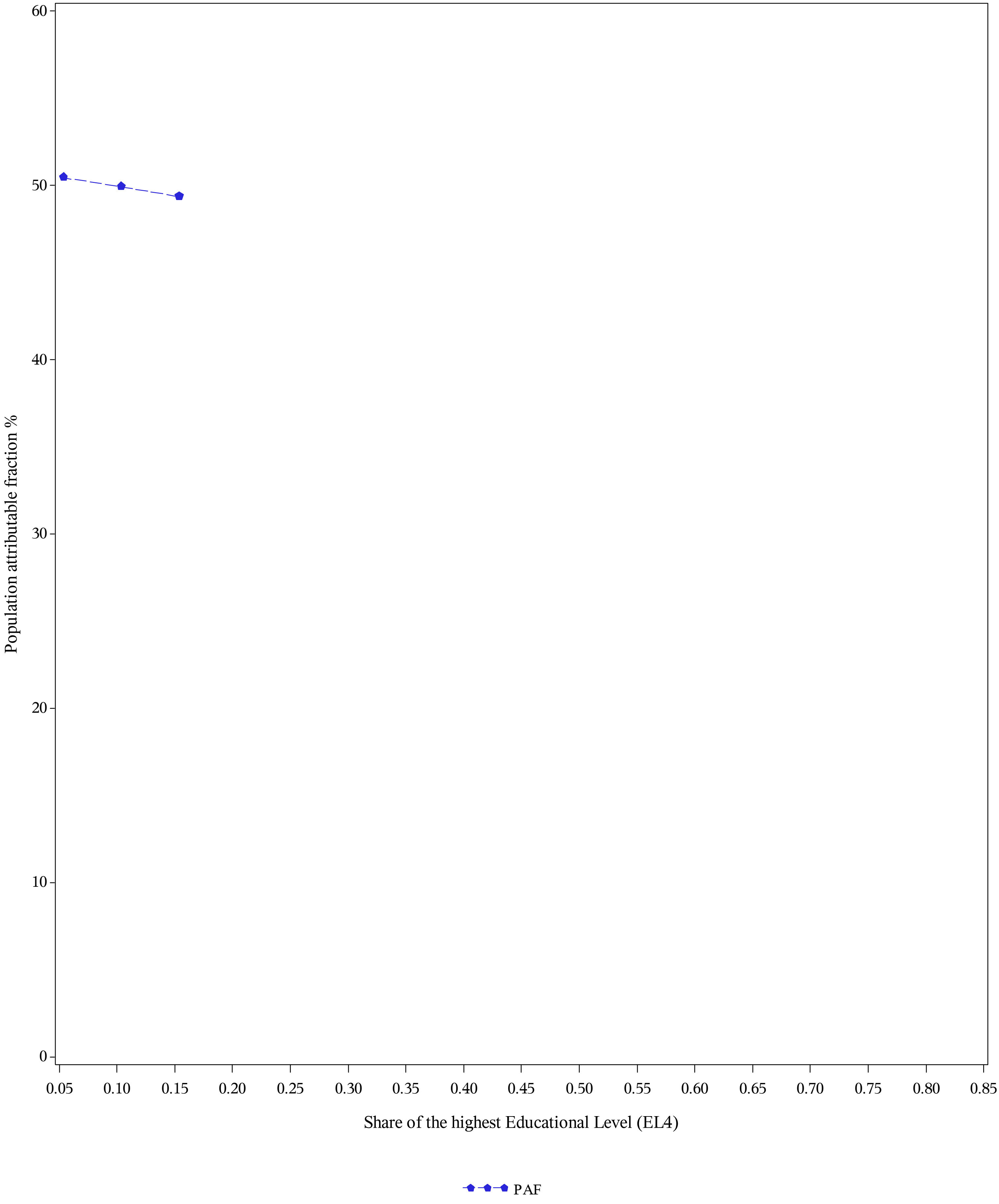

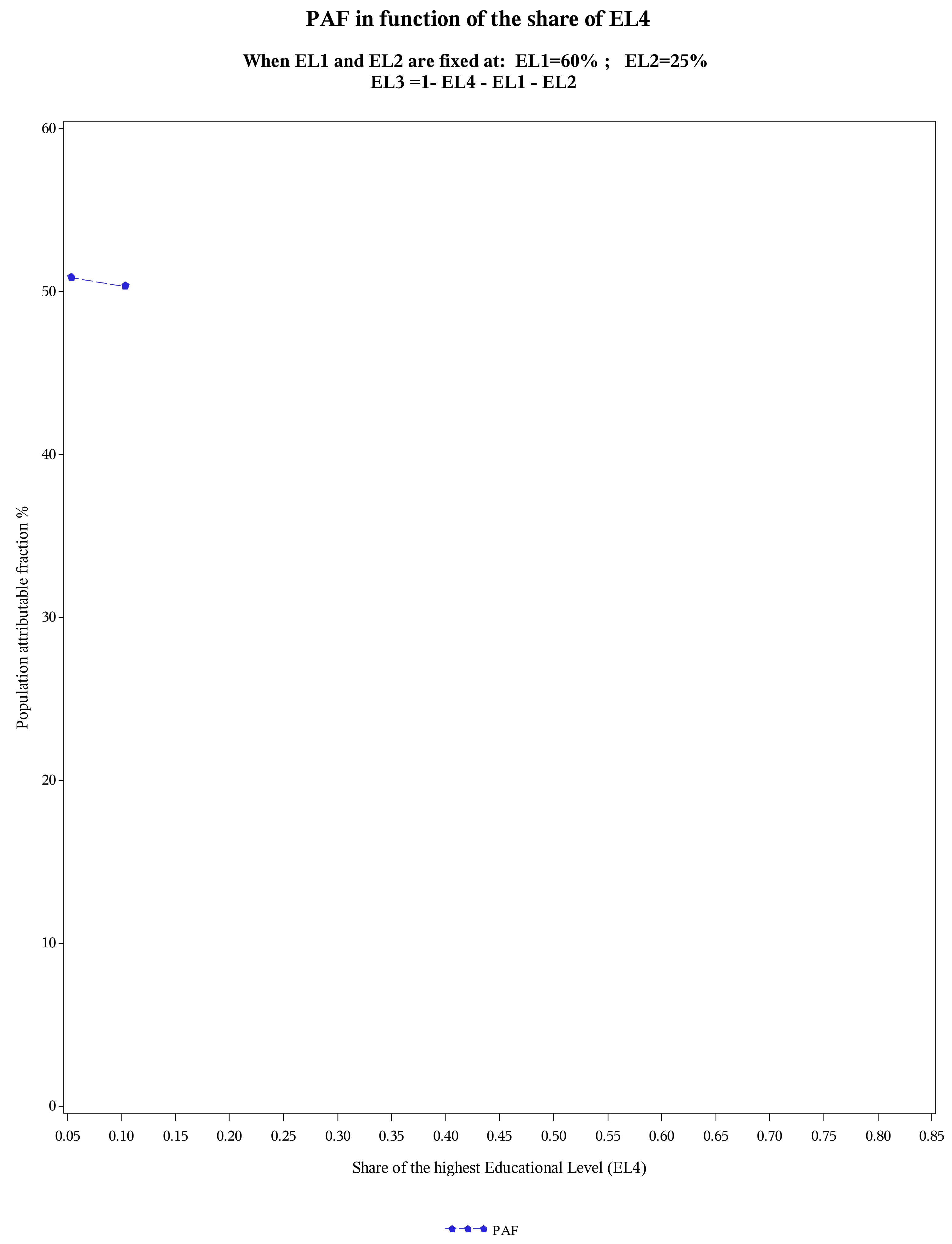
PAF in function of the share of EL4
When EL1 and EL2 are fixed at: EL1=60% ; EL2=25%
EL3 =1- EL4 - EL1 - EL2
Population attributable fraction %
60
50
40
30
20
10
0
0.05
0.10
0.15
0.20
0.25
0.30
0.35
0.40
0.45
0.50
0.55
0.60
0.65
0.70
0.75
0.80
0.85
Share of the highest Educational Level (EL4)
PAF

PAF in function of the share of EL4

When EL1 and EL2 are fixed at: EL1=65% ; EL2=5%
EL3 =1- EL4 - EL1 - EL2

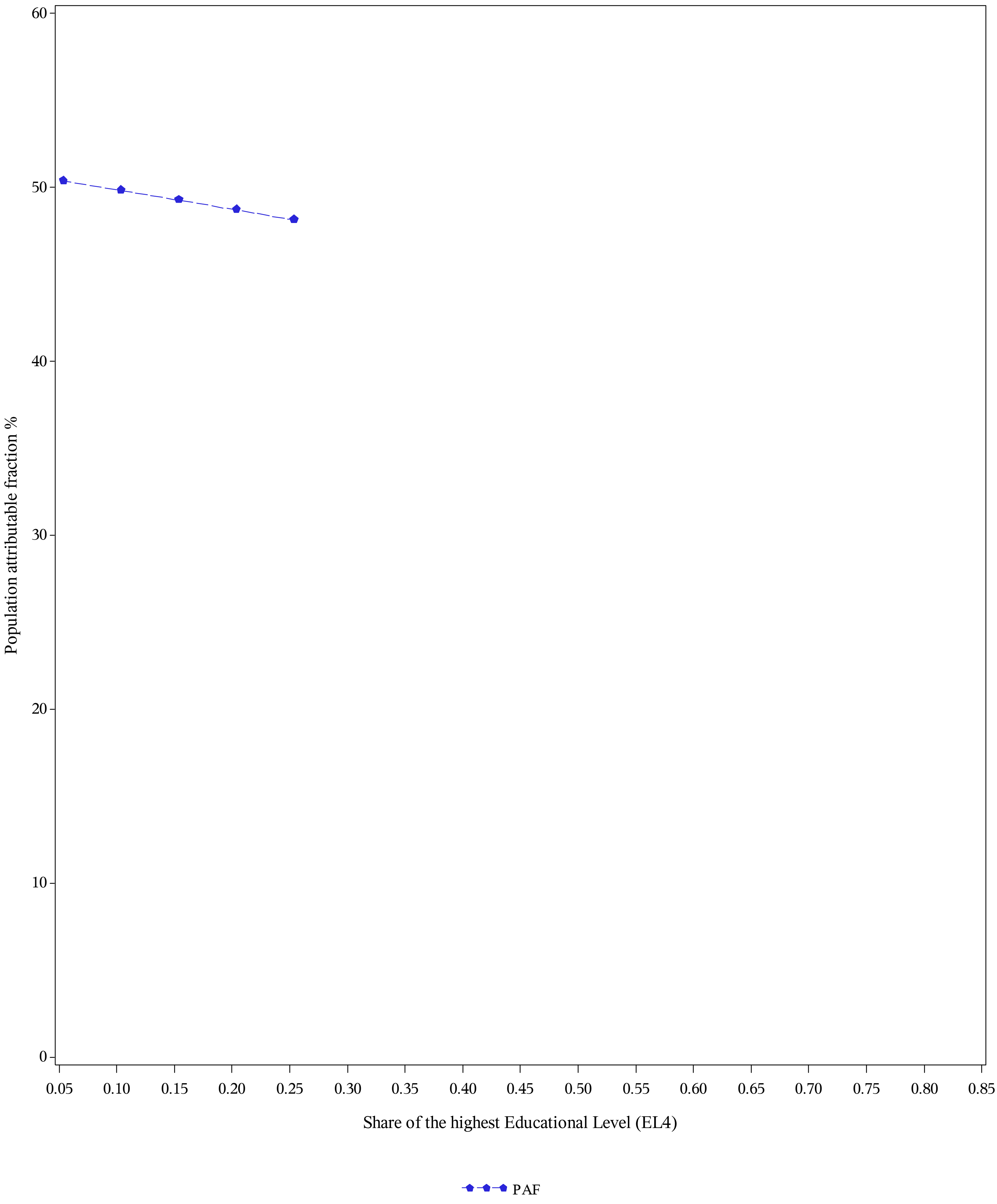

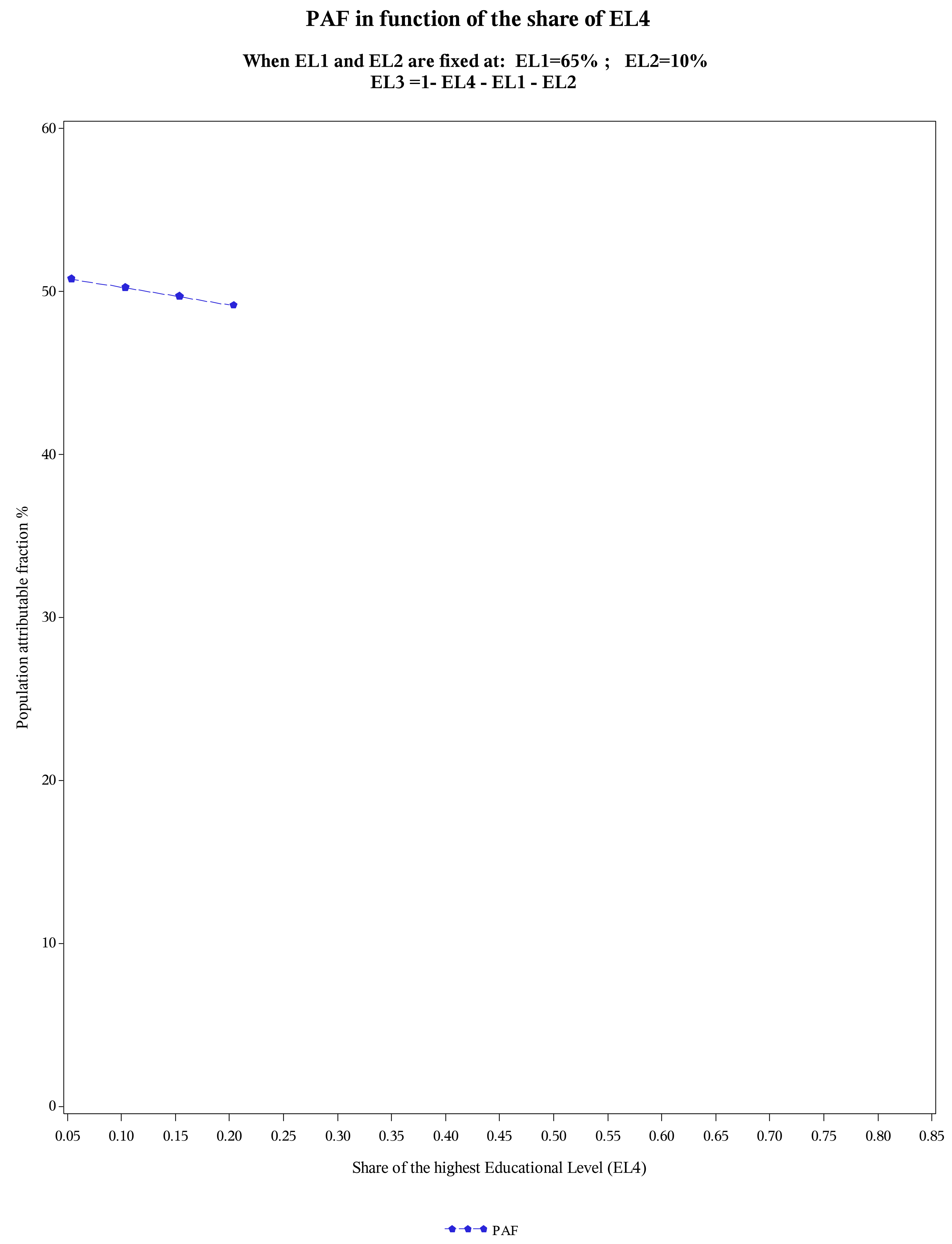

PAF in function of the share of EL4
When EL1 and EL2 are fixed at: EL1=65% ; EL2=10%
EL3 =1- EL4 - EL1 - EL2
Population attributable fraction %
0
10
20
30
40
50
60
0.05
0.10
0.15
0.20
0.25
0.30
0.35
0.40
0.45
0.50
0.55
0.60
0.65
0.70
0.75
0.80
0.85
Share of the highest Educational Level (EL4)
PAF

PAF in function of the share of EL4

When EL1 and EL2 are fixed at: EL1=65% ; EL2=15%
EL3 =1- EL4 - EL1 - EL2

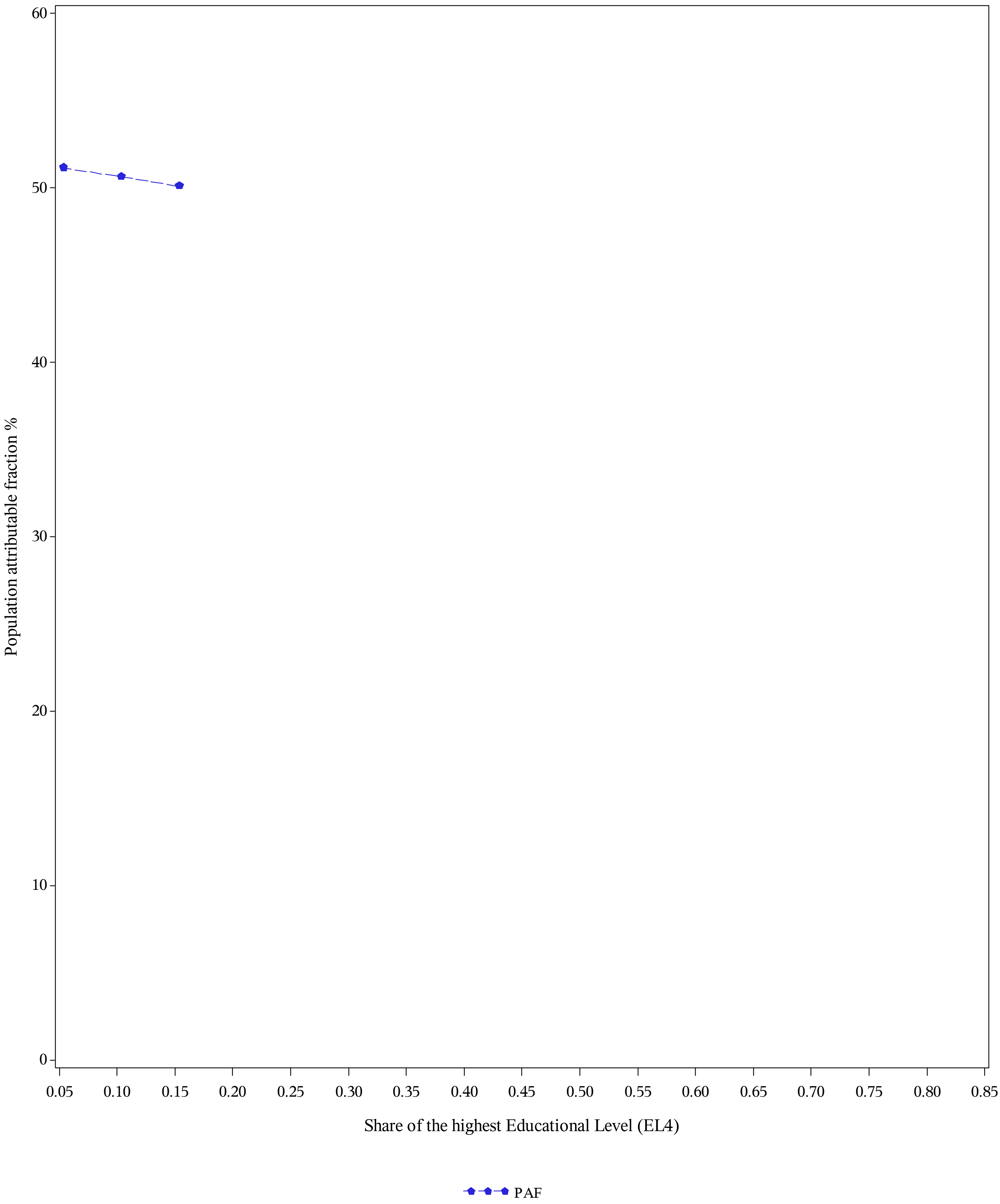

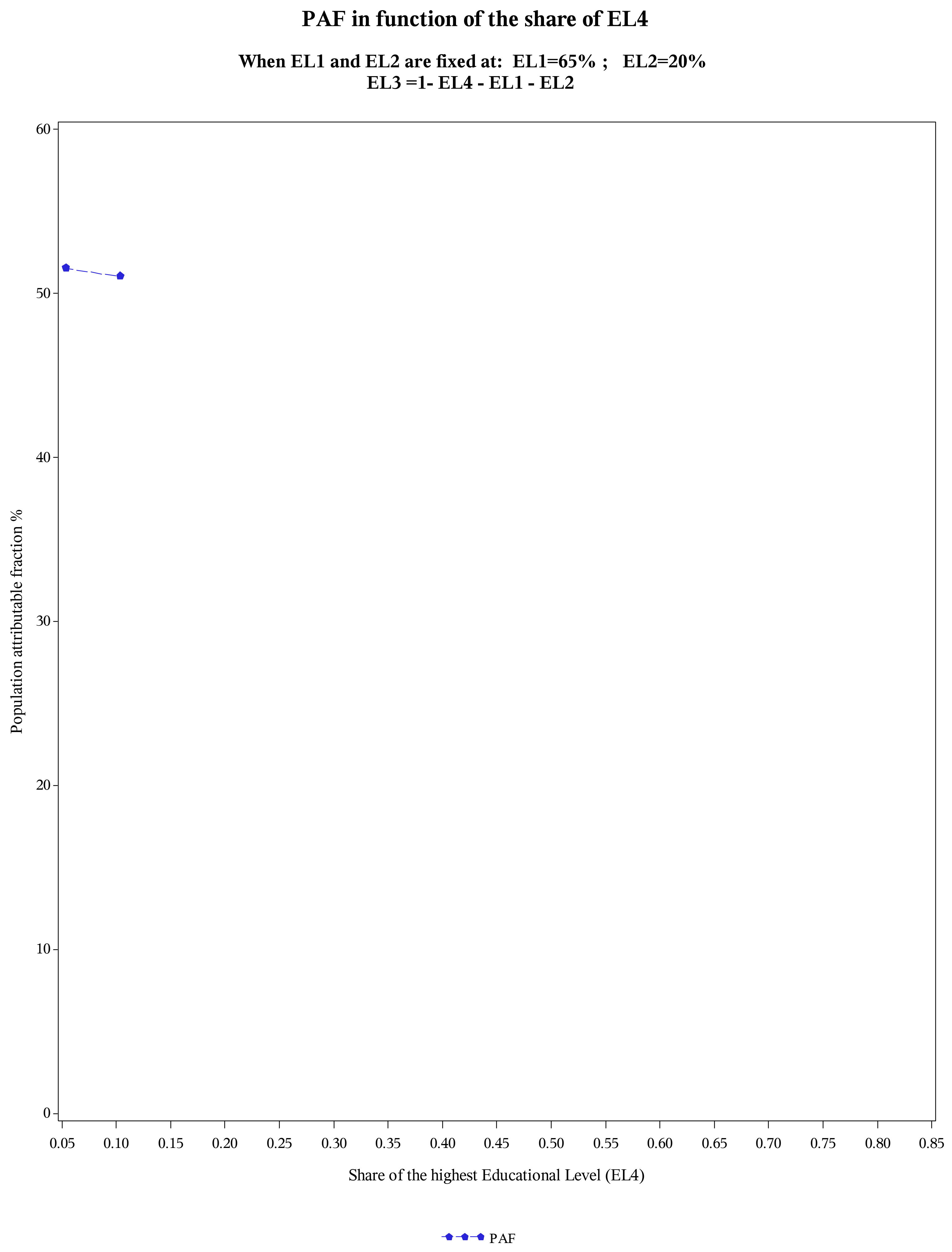
PAF in function of the share of EL4
When EL1 and EL2 are fixed at: EL1=65% ; EL2=20%
EL3 =1- EL4 - EL1 - EL2
Population attributable fraction %
0
10
20
30
40
50
60
0.05
0.10
0.15
0.20
0.25
0.30
0.35
0.40
0.45
0.50
0.55
0.60
0.65
0.70
0.75
0.80
0.85
Share of the highest Educational Level (EL4)
PAF

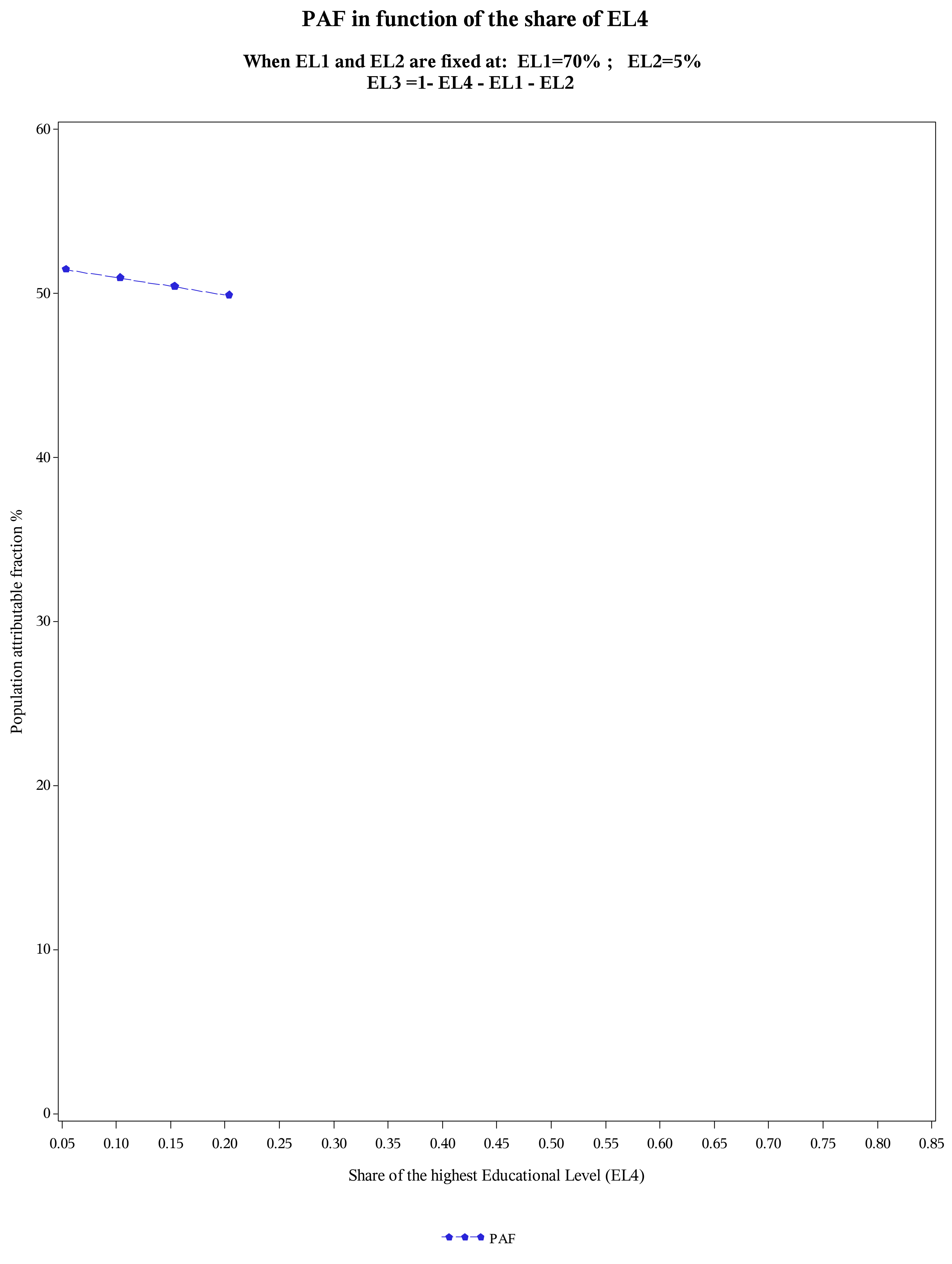
PAF in function of the share of EL4
When EL1 and EL2 are fixed at: EL1=70% ; EL2=5%
EL3 =1- EL4 - EL1 - EL2
Population attributable fraction %
0
10
20
30
40
50
60
0.05
0.10
0.15
0.20
0.25
0.30
0.35
0.40
0.45
0.50
0.55
0.60
0.65
0.70
0.75
0.80
0.85
Share of the highest Educational Level (EL4)
PAF

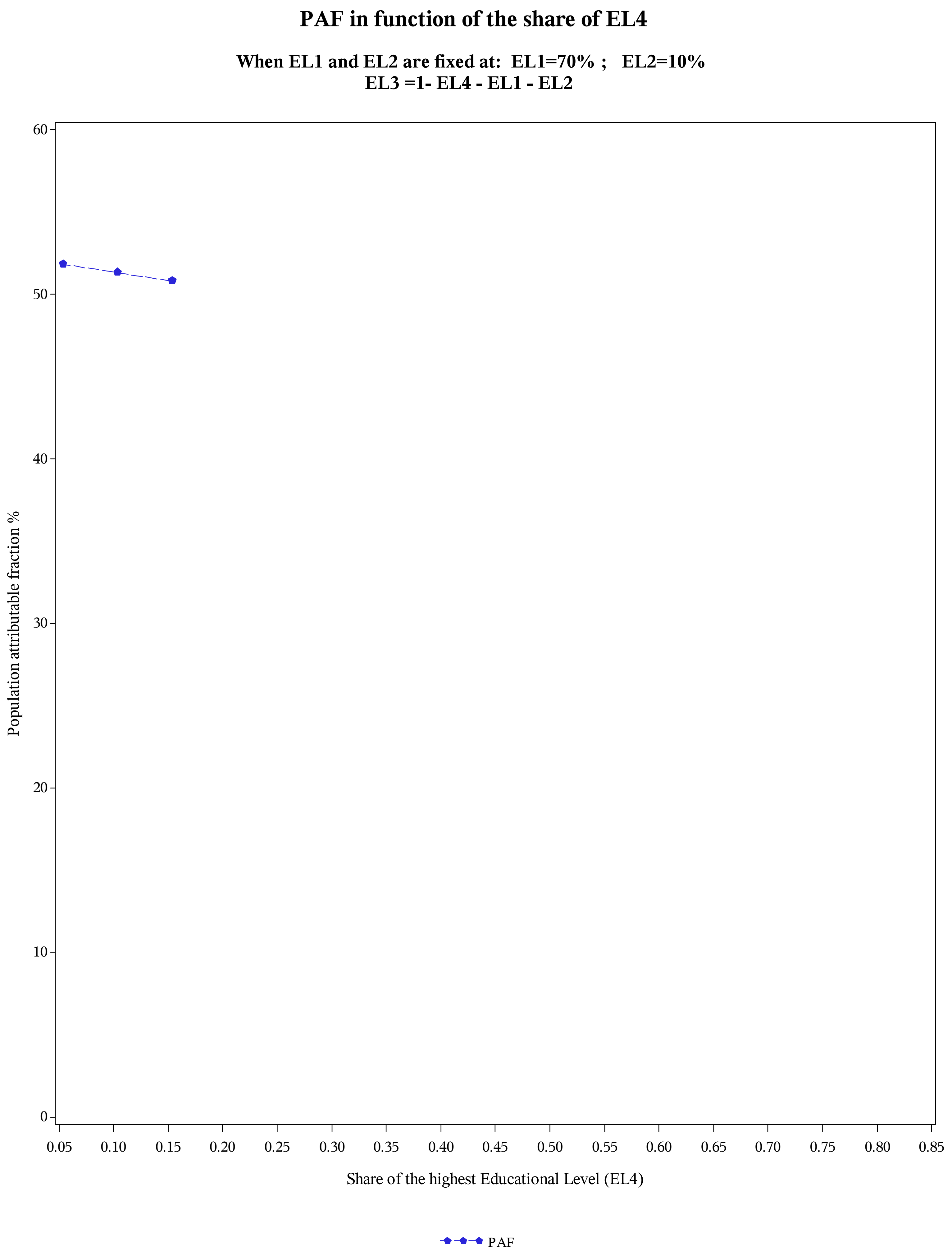
PAF in function of the share of EL4
When EL1 and EL2 are fixed at: EL1=70% ; EL2=10%
EL3 =1- EL4 - EL1 - EL2
Population attributable fraction %
0
10
20
30
40
50
60
0.05
0.10
0.15
0.20
0.25
0.30
0.35
0.40
0.45
0.50
0.55
0.60
0.65
0.70
0.75
0.80
0.85
Share of the highest Educational Level (EL4)
PAF

PAF in function of the share of EL4

When EL1 and EL2 are fixed at: EL1=70% ; EL2=15%
EL3 =1- EL4 - EL1 - EL2

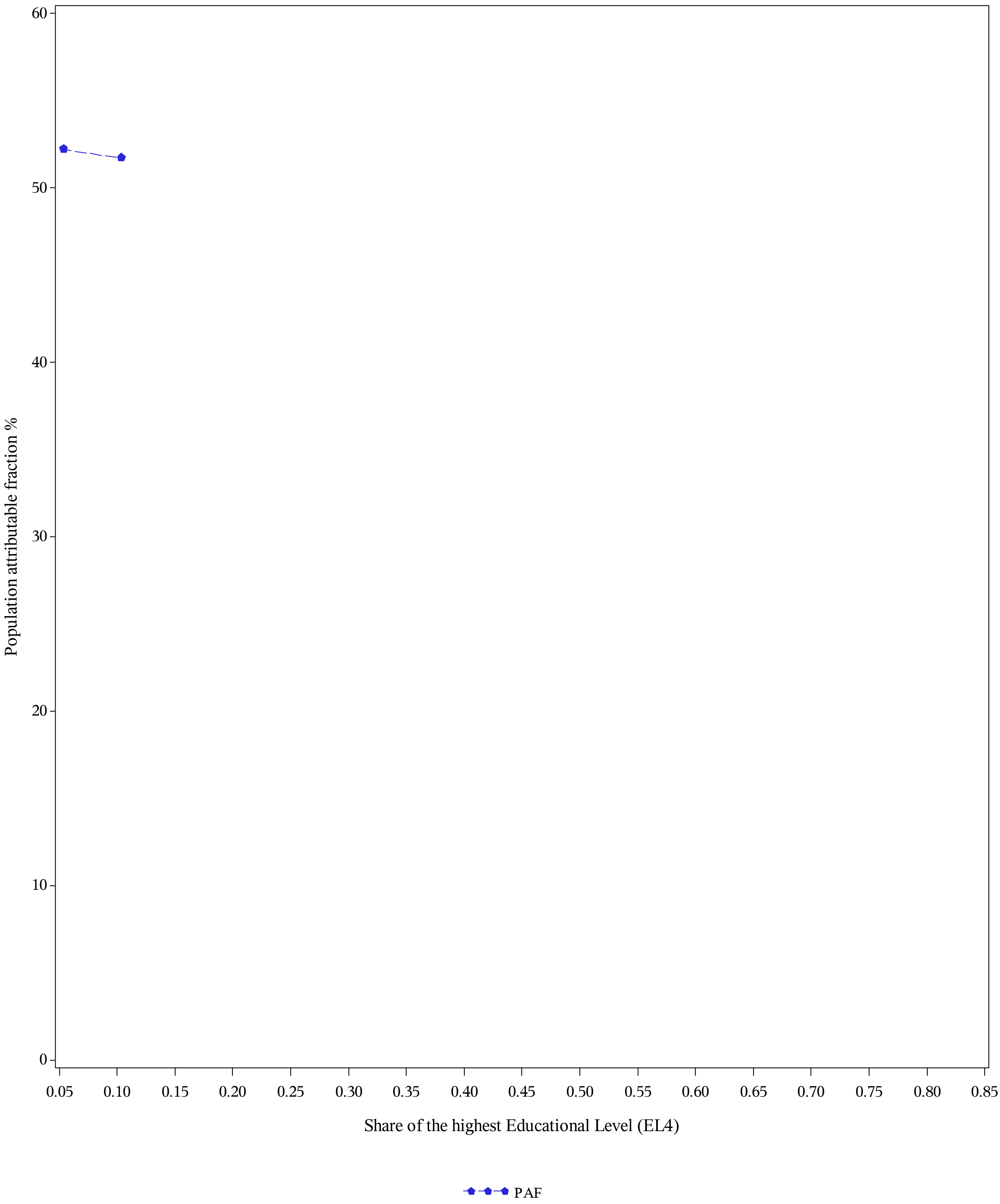

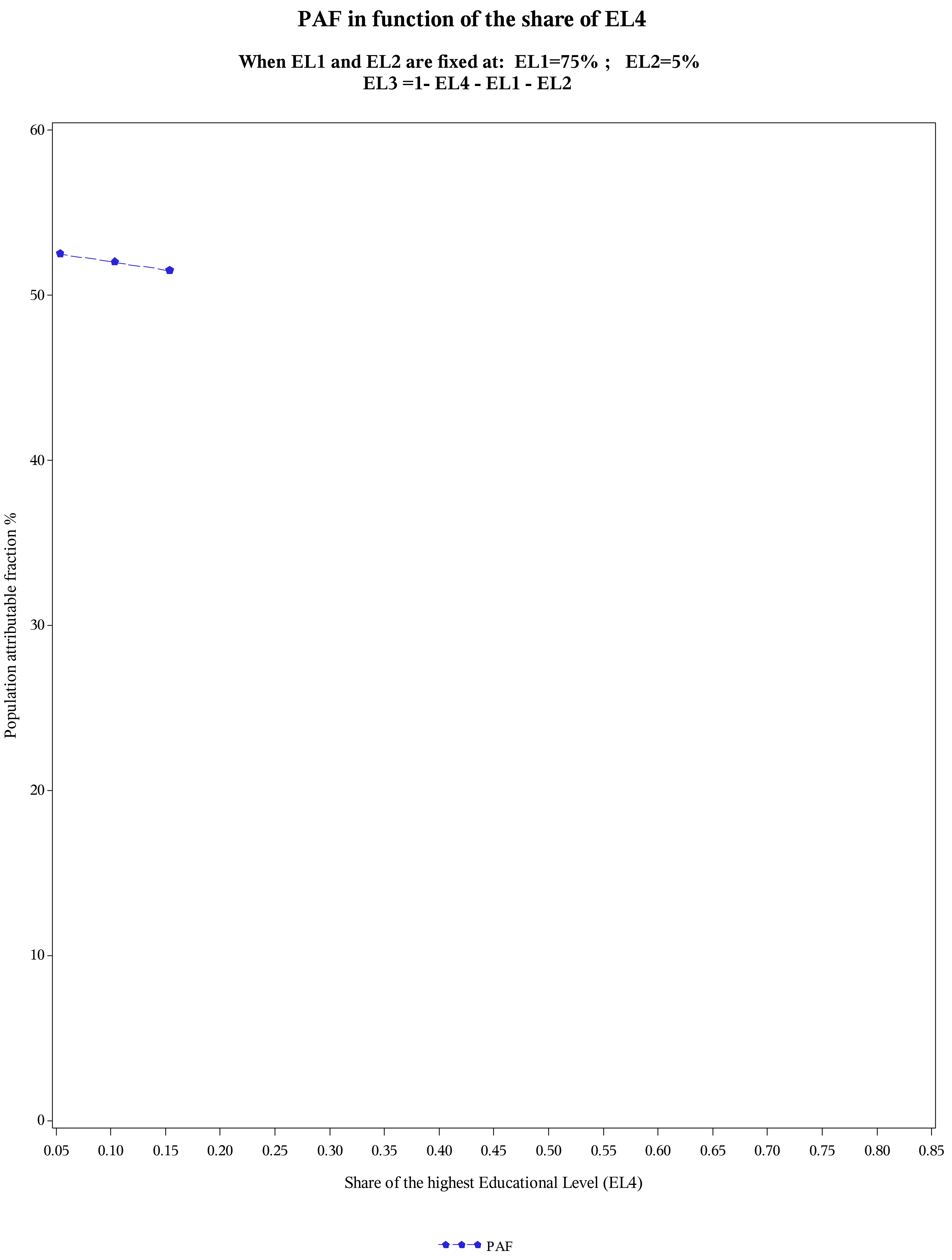
PAF in function of the share of EL4
When EL1 and EL2 are fixed at: EL1=75% ; EL2=5%
EL3 =1- EL4 - EL1 - EL2
Population attributable fraction %
0
10
20
30
40
50
60
0.05
0.10
0.15
0.20
0.25
0.30
0.35
0.40
0.45
0.50
0.55
0.60
0.65
0.70
0.75
0.80
0.85
Share of the highest Educational Level (EL4)
PAF

PAF in function of the share of EL4

When EL1 and EL2 are fixed at: EL1=75% ; EL2=10%
EL3 =1- EL4 - EL1 - EL2

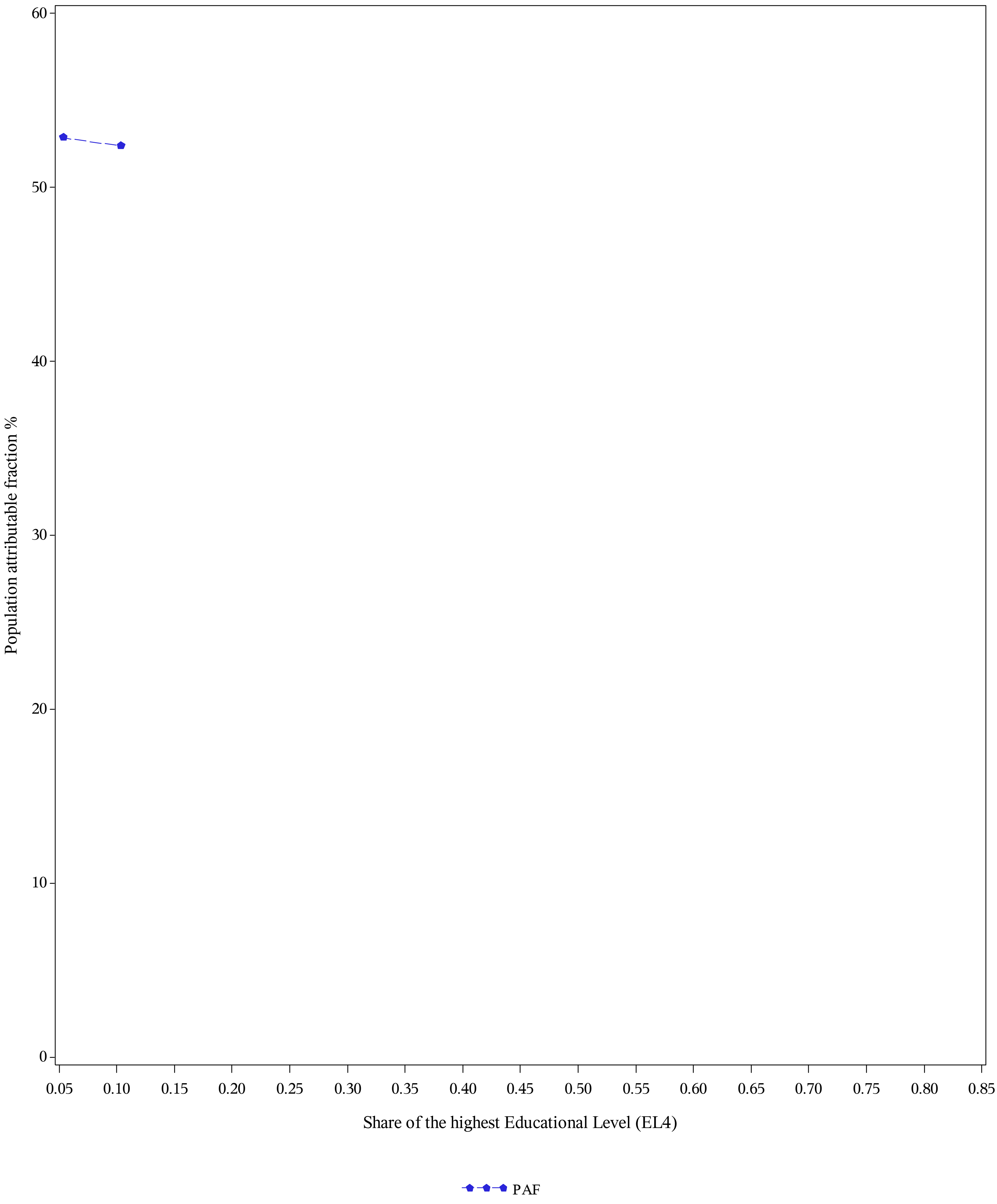

PAF in function of the share of EL4

When EL1 and EL2 are fixed at: EL1=80% ; EL2=5%
EL3 =1- EL4 - EL1 - EL2

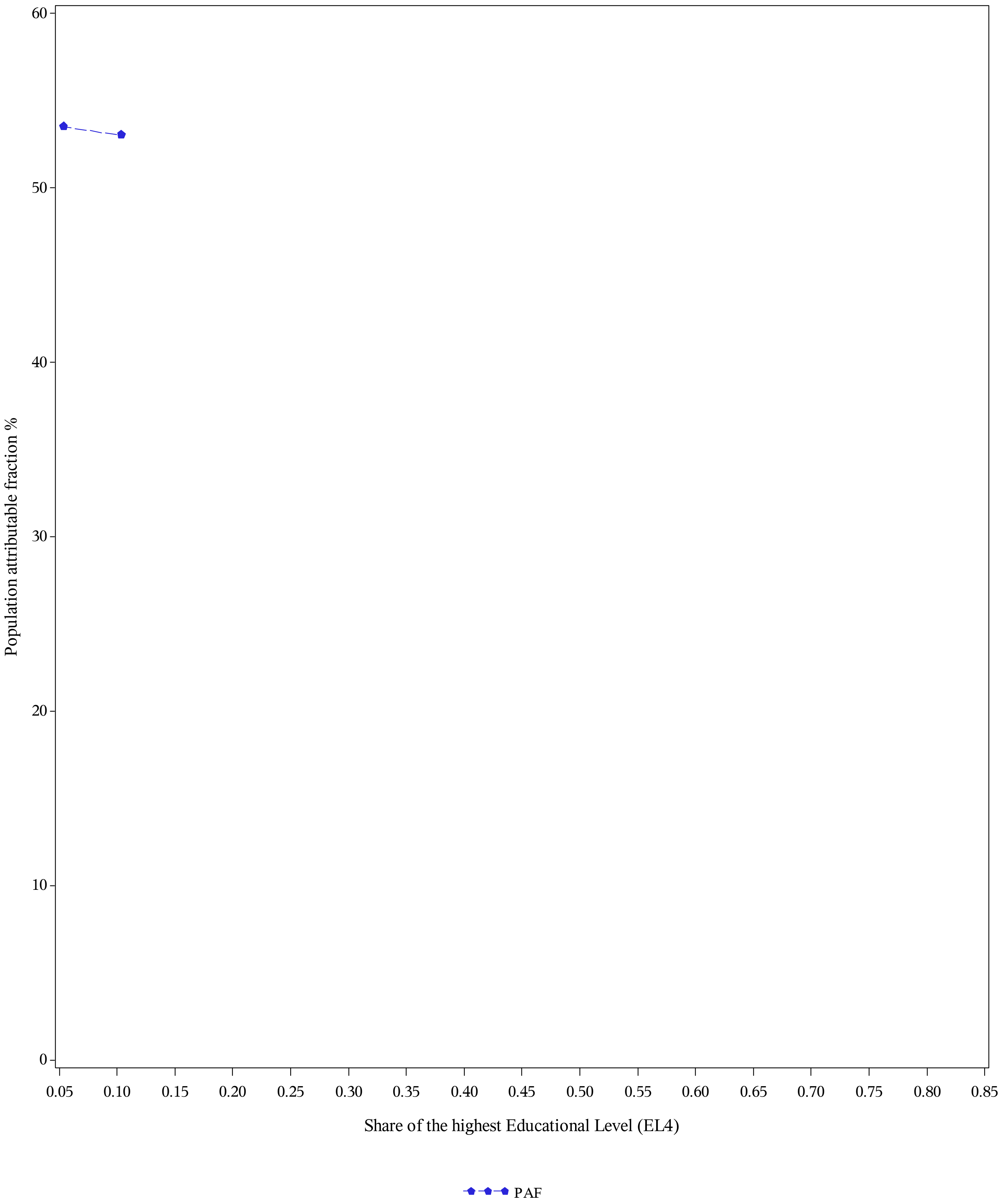

Supplement: Supplementary file 5 — Full set of figures representing the evolution of the PAF in function of P4 at fixed p1 and p2 (PDF 476 kb) [file 12889_2019_6980_MOESM5_ESM.pdf]
