# Supplementary material for: Monitoring health inequalities when the socio-economic composition changes: are the slope and relative indices of inequality appropriate? Results of a simulation study
Source: BMC Public Health. 2019 May 30;19:662. doi: 10.1186/s12889-019-6980-1 (PMC6543610; doi:10.1186/s12889-019-6980-1)

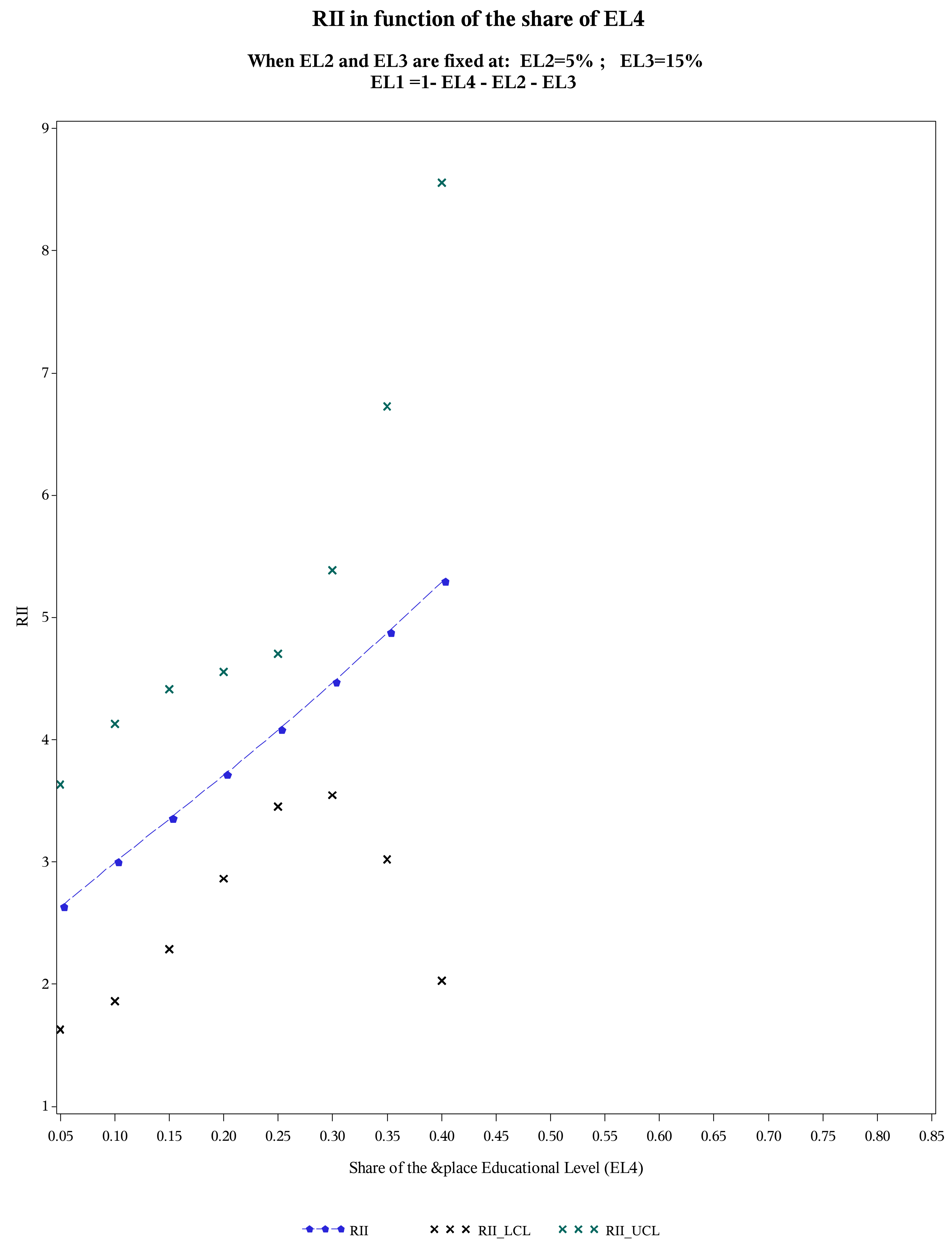
RII in function of the share of EL4
When EL2 and EL3 are fixed at: EL2=5% ; EL3=15%
EL1 =1- EL4 - EL2 - EL3
RII
1
2
3
4
5
6
7
8
9
0.05
0.10
0.15
0.20
0.25
0.30
0.35
0.40
0.45
0.50
0.55
0.60
0.65
0.70
0.75
0.80
0.85
Share of the &place Educational Level (EL4)
RII
RII_LCL
RII_UCL

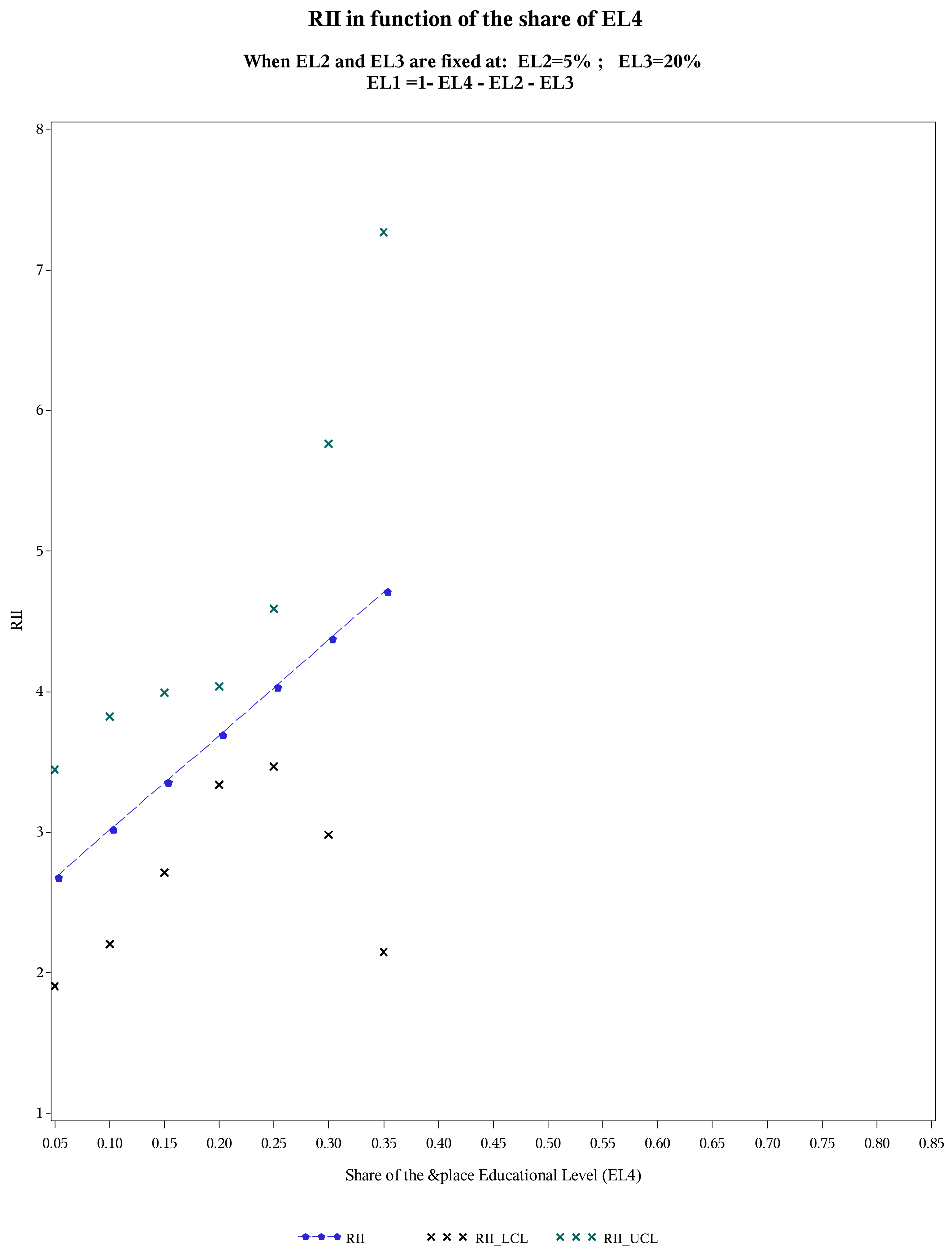

RII in function of the share of EL4
When EL2 and EL3 are fixed at: EL2=5% ; EL3=20%
EL1 =1- EL4 - EL2 - EL3
RII
1
2
3
4
5
6
7
8
0.05
0.10
0.15
0.20
0.25
0.30
0.35
0.40
0.45
0.50
0.55
0.60
0.65
0.70
0.75
0.80
0.85
Share of the &place Educational Level (EL4)
RII
RII_LCL
RII_UCL

RII in function of the share of EL4

When EL2 and EL3 are fixed at: EL2=5% ; EL3=25%
EL1 =1- EL4 - EL2 - EL3

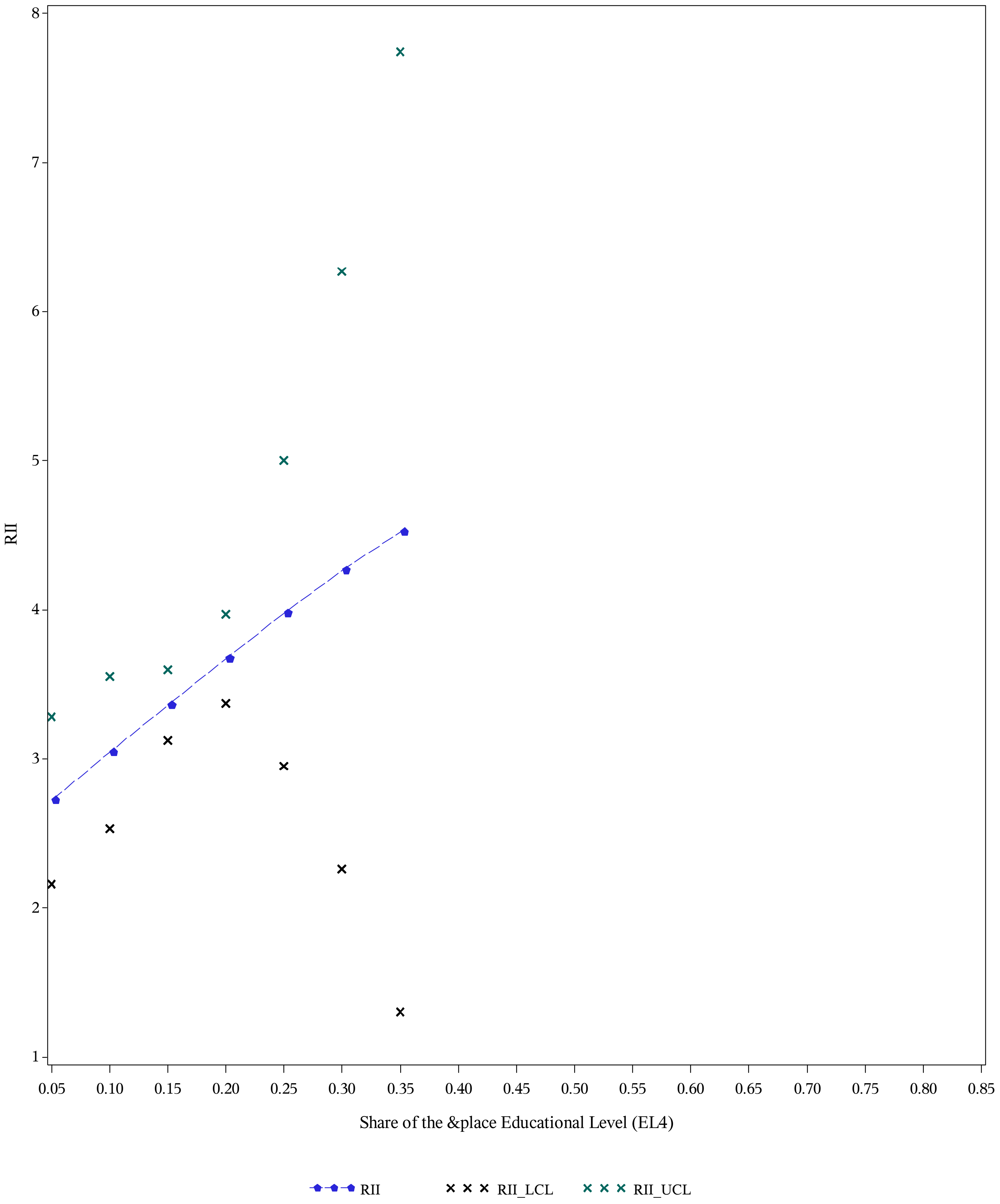

RII in function of the share of EL4

When EL2 and EL3 are fixed at: EL2=5% ; EL3=30%
EL1 =1- EL4 - EL2 - EL3

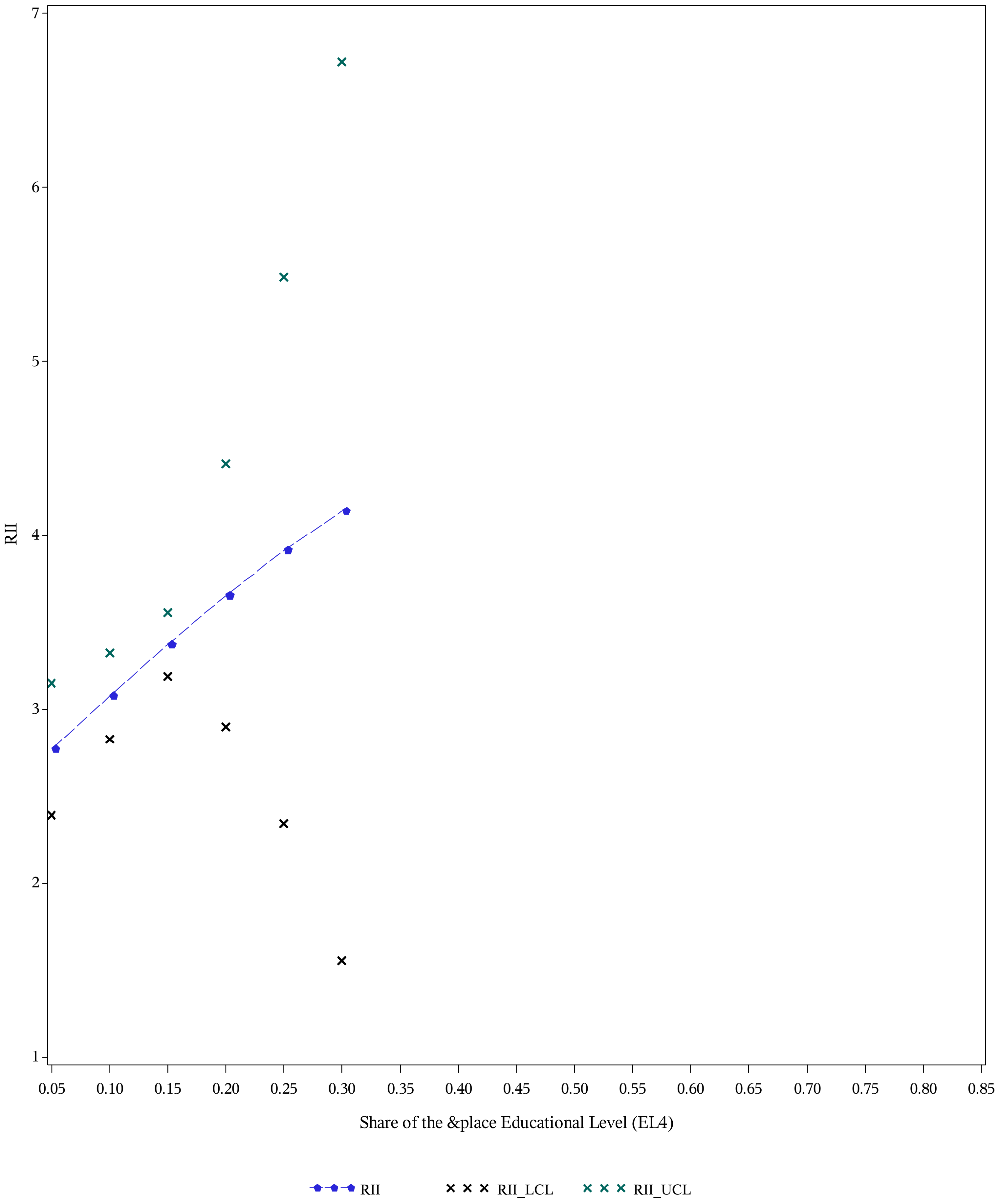

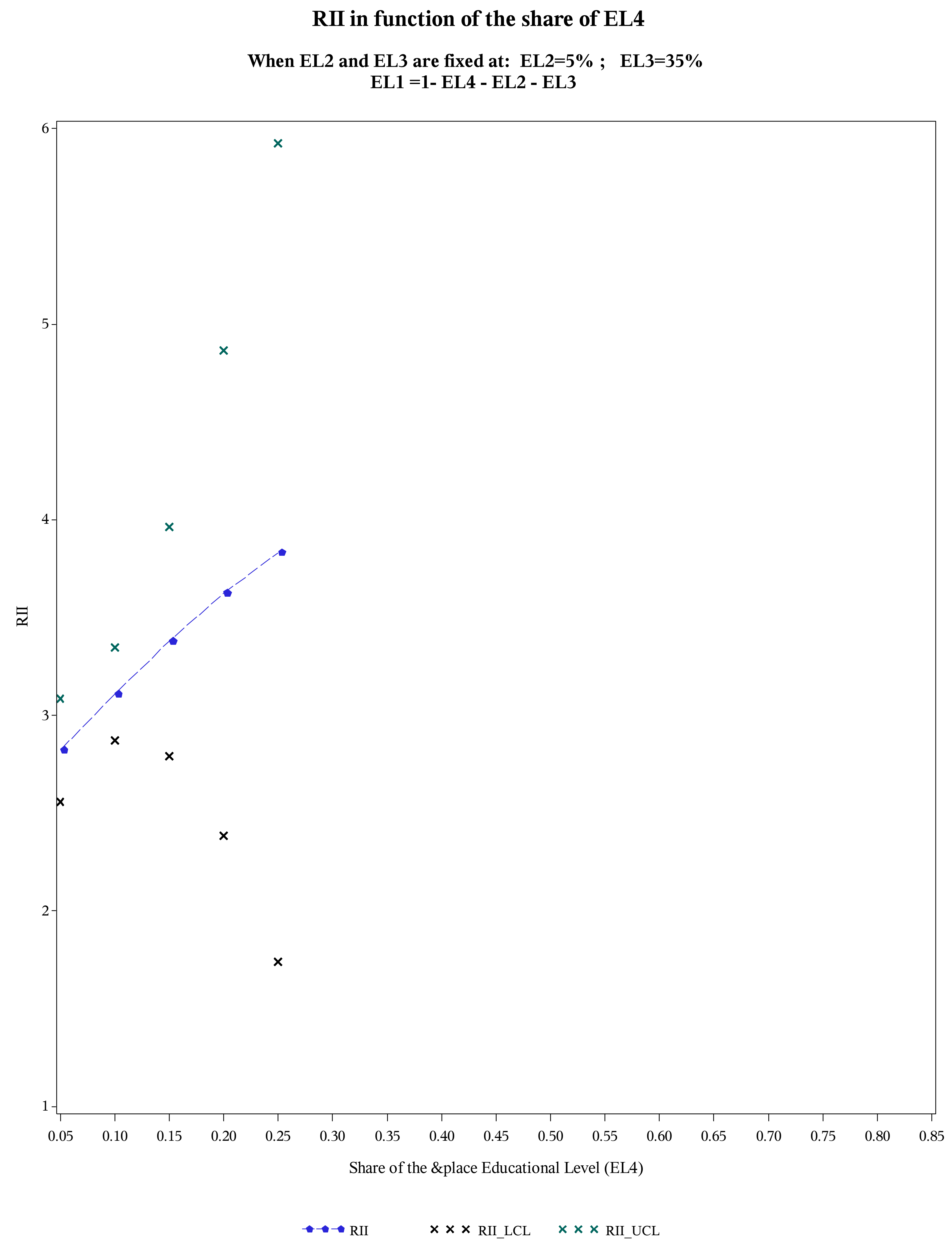
RII in function of the share of EL4
When EL2 and EL3 are fixed at: EL2=5% ; EL3=35%
EL1 =1- EL4 - EL2 - EL3
RII
6
5
4
3
2
1
0.05
0.10
0.15
0.20
0.25
0.30
0.35
0.40
0.45
0.50
0.55
0.60
0.65
0.70
0.75
0.80
0.85
Share of the &place Educational Level (EL4)
RII
RII_LCL
RII_UCL

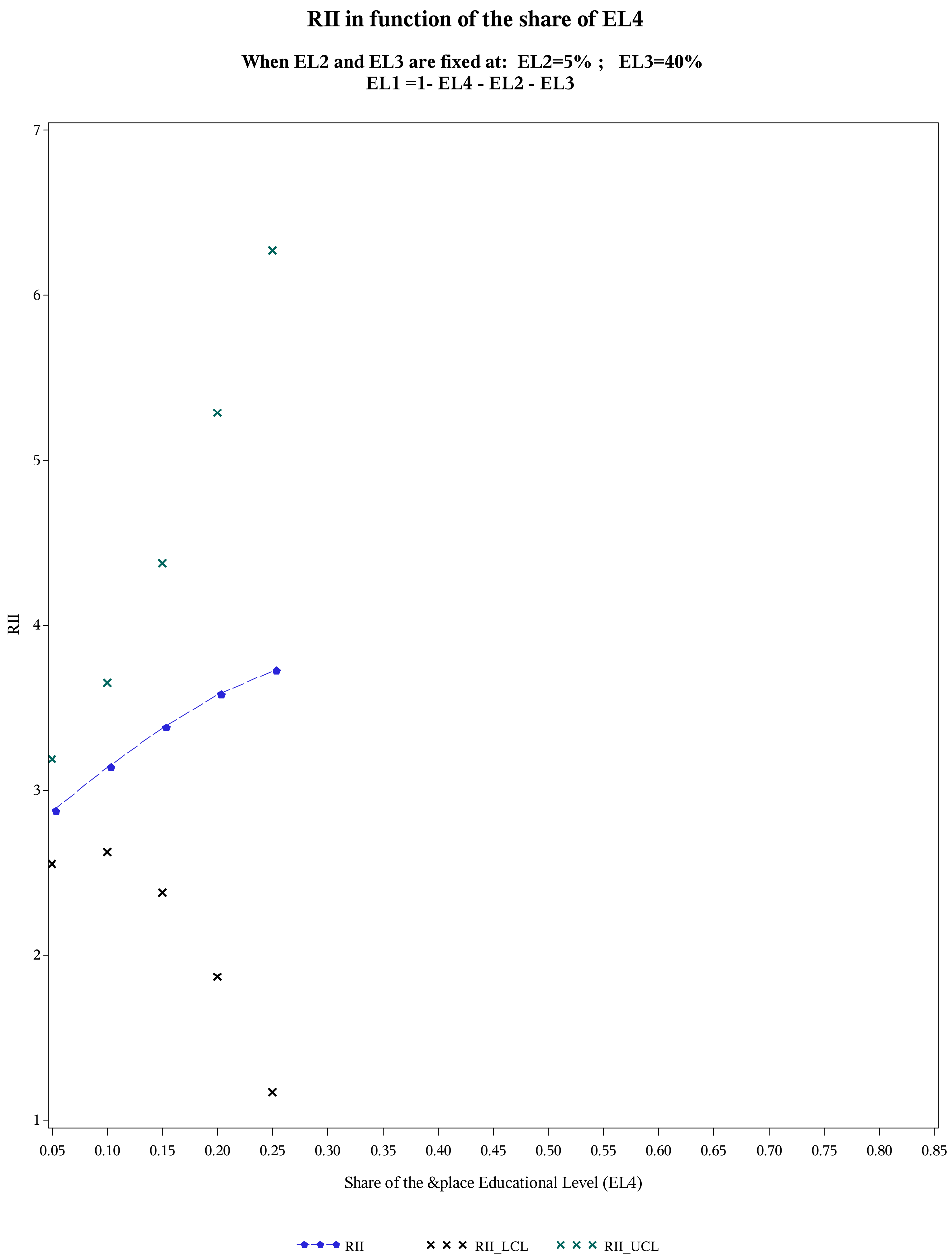
RII in function of the share of EL4
When EL2 and EL3 are fixed at: EL2=5% ; EL3=40%
EL1 =1- EL4 - EL2 - EL3
RII
7
6
5
4
3
2
1
0.05
0.10
0.15
0.20
0.25
0.30
0.35
0.40
0.45
0.50
0.55
0.60
0.65
0.70
0.75
0.80
0.85
Share of the &place Educational Level (EL4)
RII
RII_LCL
RII_UCL

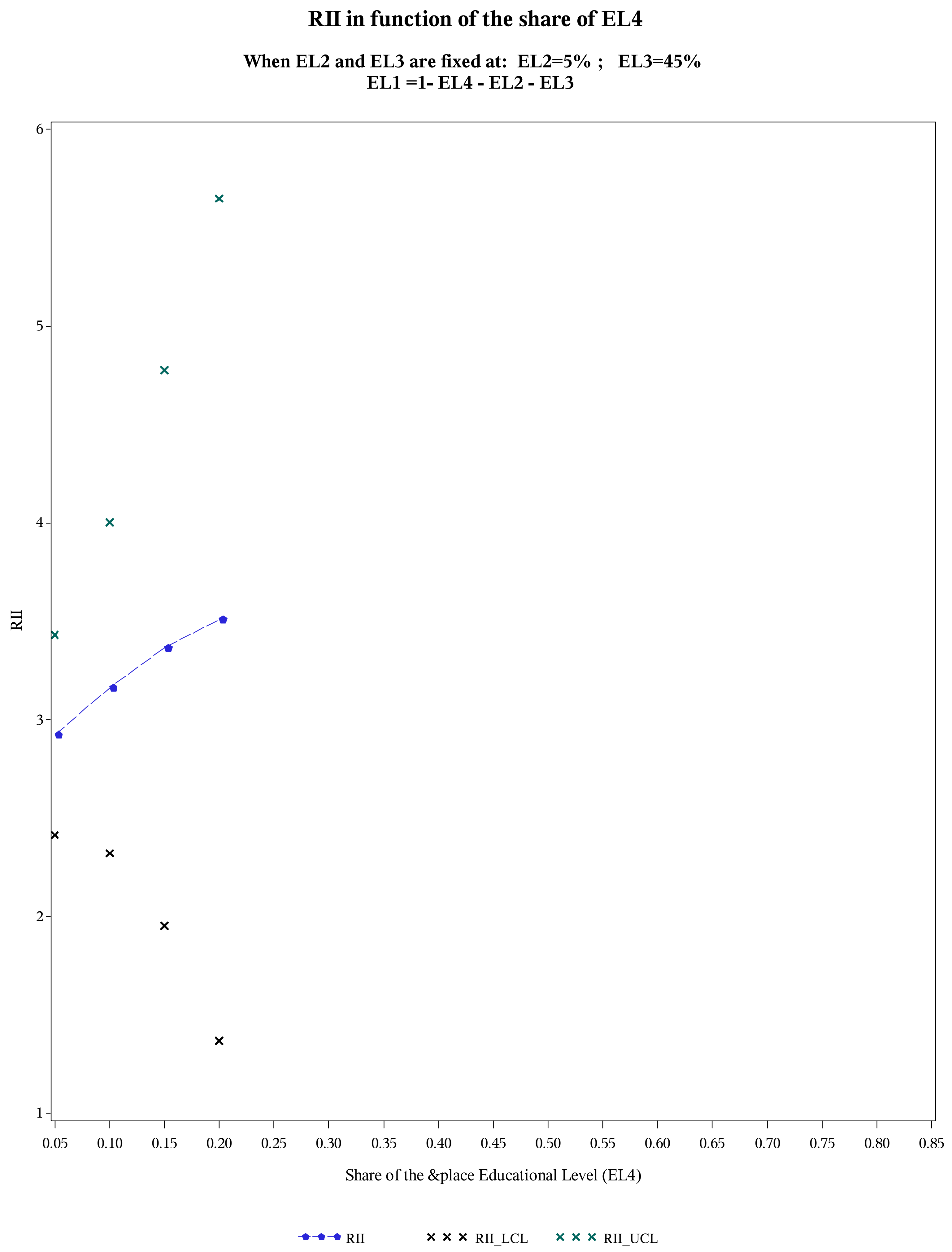
RII in function of the share of EL4
When EL2 and EL3 are fixed at: EL2=5% ; EL3=45%
EL1 =1- EL4 - EL2 - EL3
RII
1
2
3
4
5
6
0.05
0.10
0.15
0.20
0.25
0.30
0.35
0.40
0.45
0.50
0.55
0.60
0.65
0.70
0.75
0.80
0.85
Share of the &place Educational Level (EL4)
RII
RII_LCL
RII_UCL

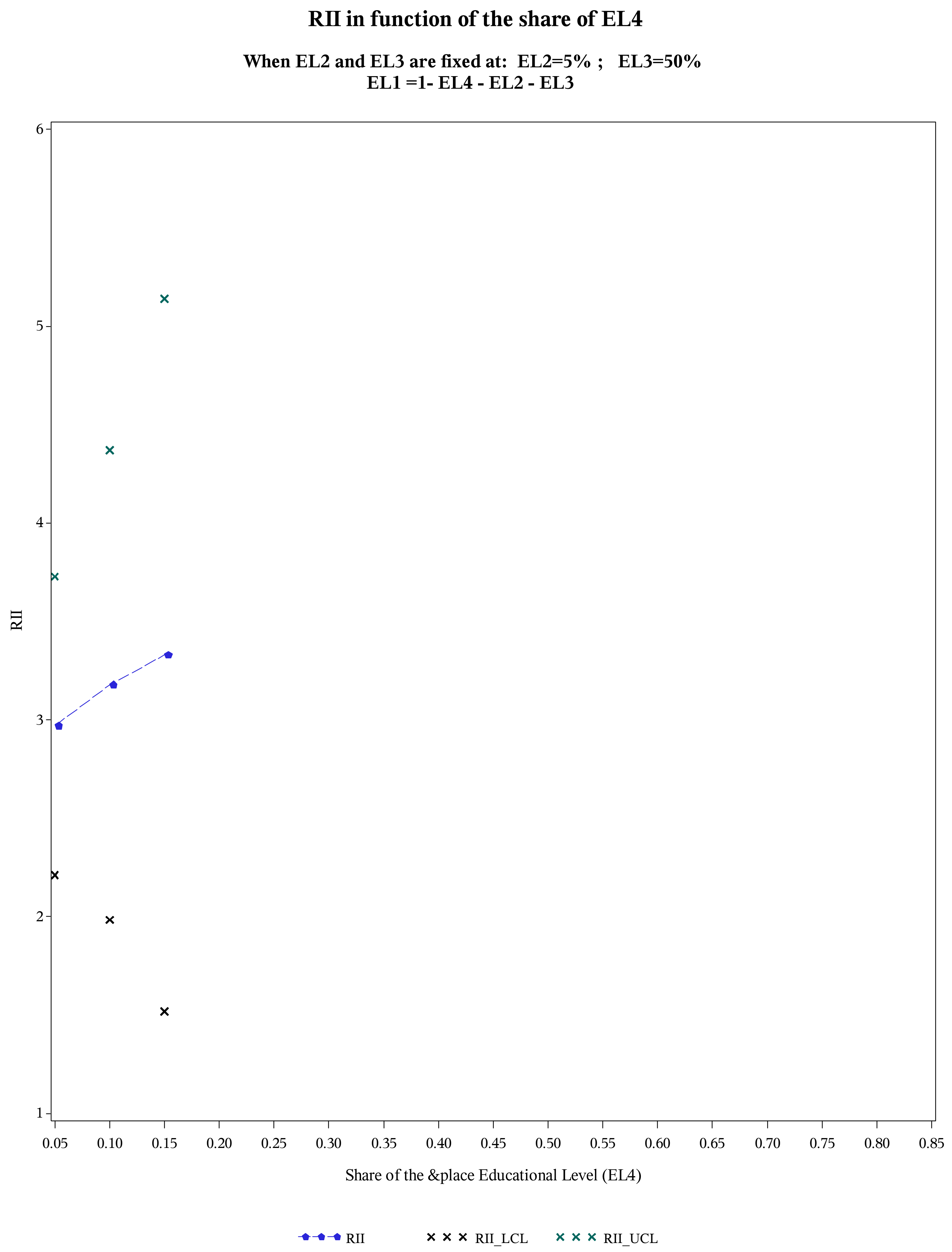
RII in function of the share of EL4
When EL2 and EL3 are fixed at: EL2=5% ; EL3=50%
EL1 =1- EL4 - EL2 - EL3
RII
1
2
3
4
5
6
0.05
0.10
0.15
0.20
0.25
0.30
0.35
0.40
0.45
0.50
0.55
0.60
0.65
0.70
0.75
0.80
0.85
Share of the &place Educational Level (EL4)
RII
RII_LCL
RII_UCL

RII in function of the share of EL4

When EL2 and EL3 are fixed at: EL2=5% ; EL3=55%
EL1 =1- EL4 - EL2 - EL3

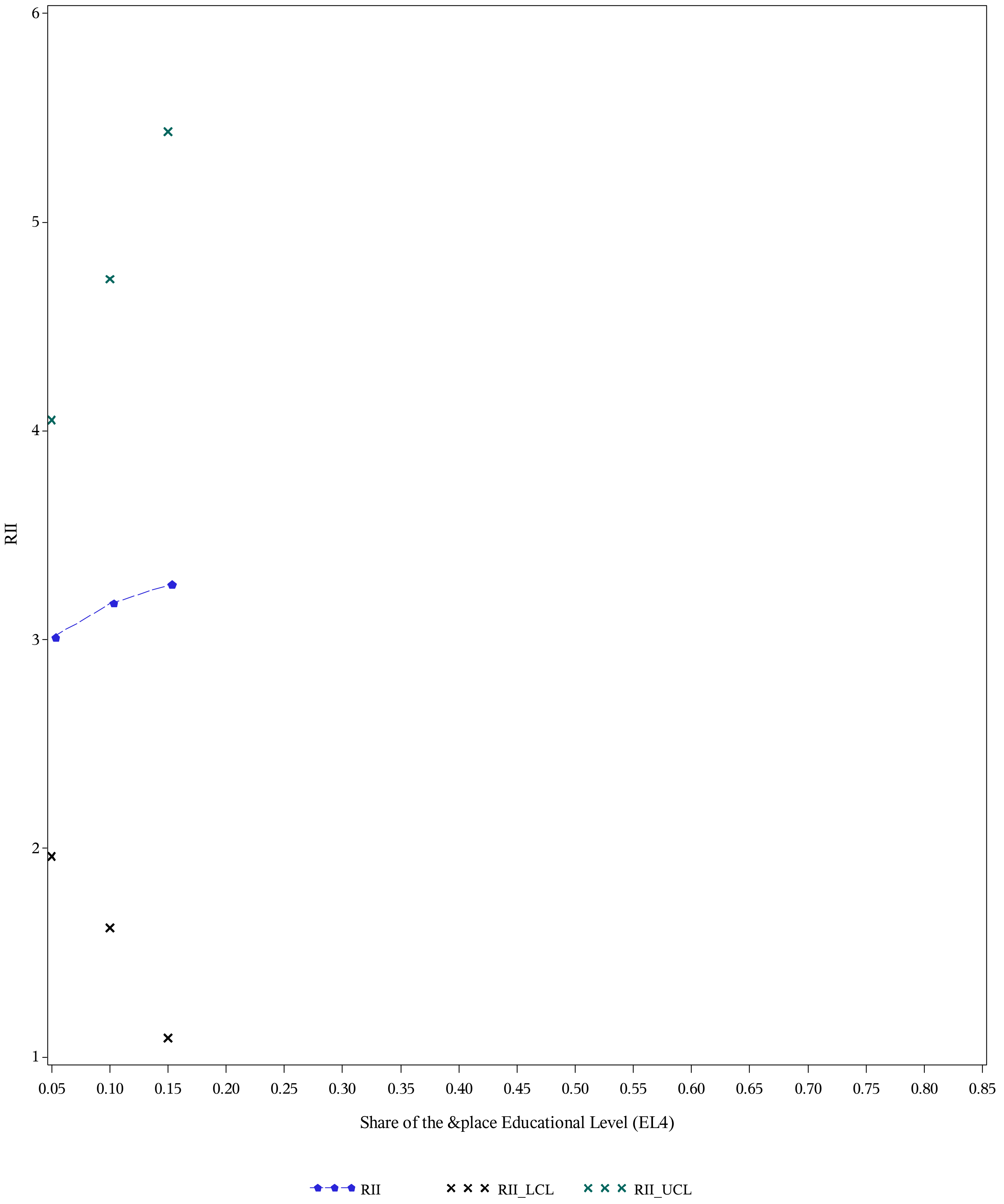

RII in function of the share of EL4

When EL2 and EL3 are fixed at: EL2=5% ; EL3=60%
EL1 =1- EL4 - EL2 - EL3

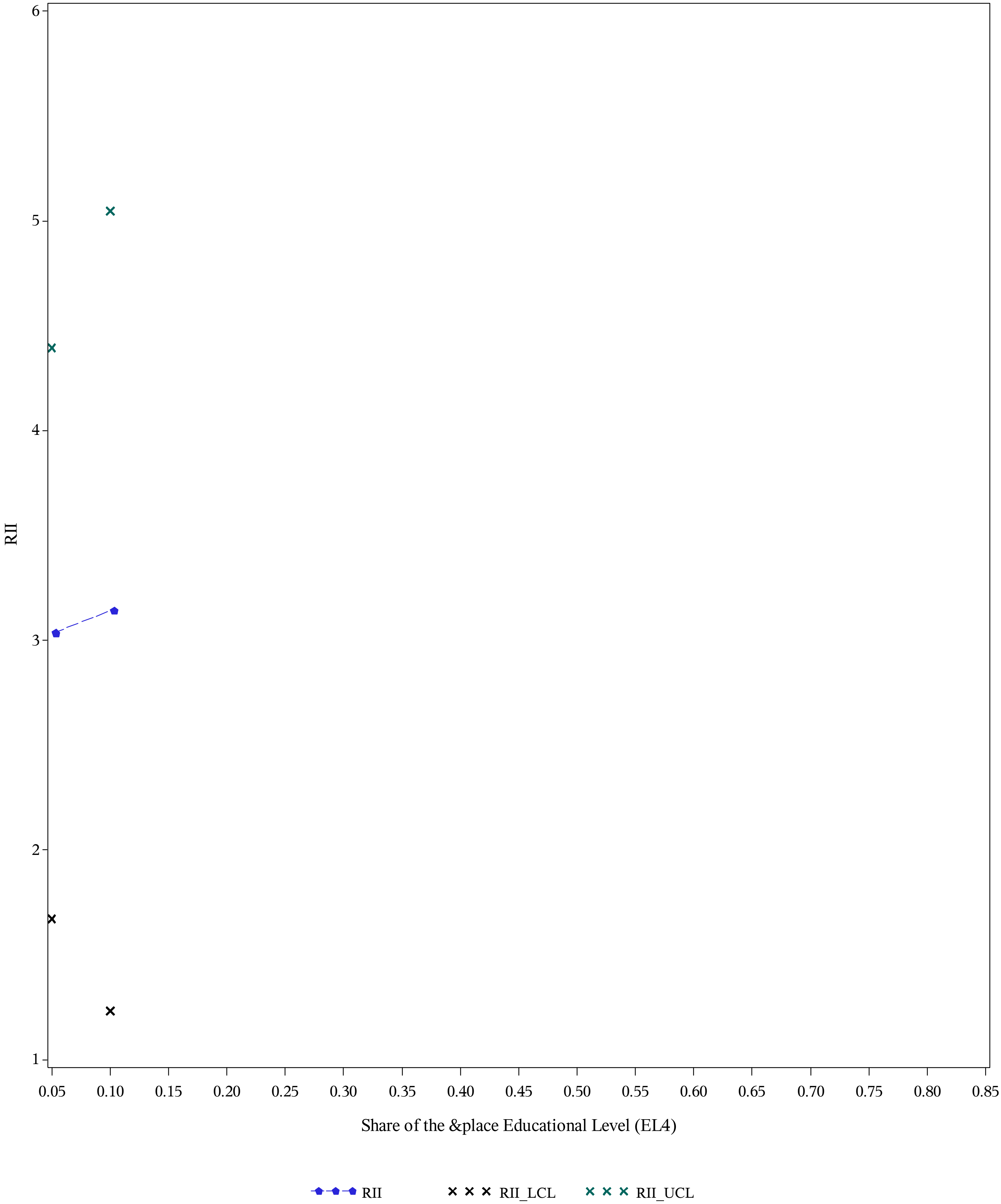

RII in function of the share of EL4

When EL2 and EL3 are fixed at: EL2=5% ; EL3=65%
EL1 =1- EL4 - EL2 - EL3

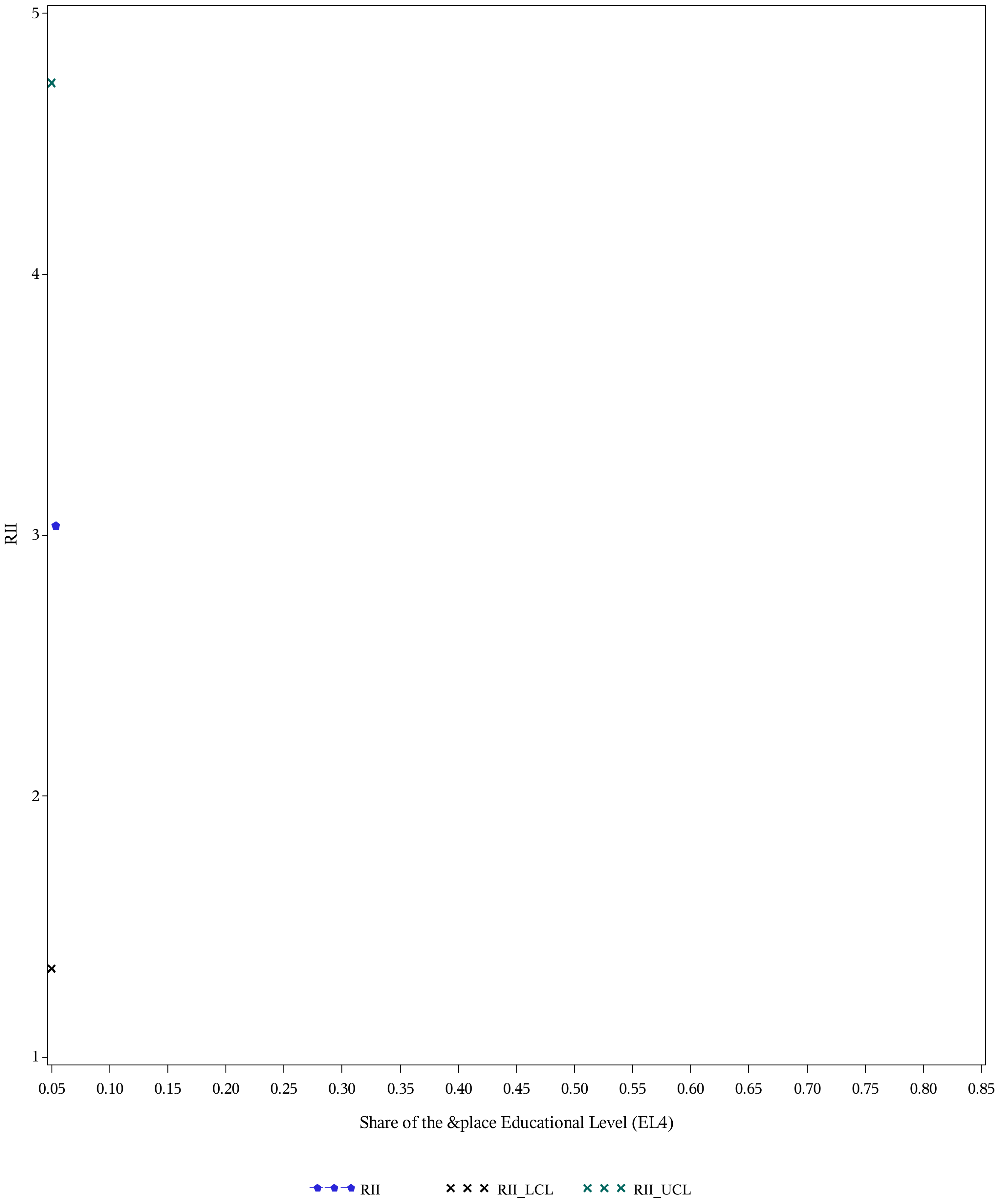

RII in function of the share of EL4

When EL2 and EL3 are fixed at: EL2=10% ; EL3=5%
EL1 =1- EL4 - EL2 - EL3

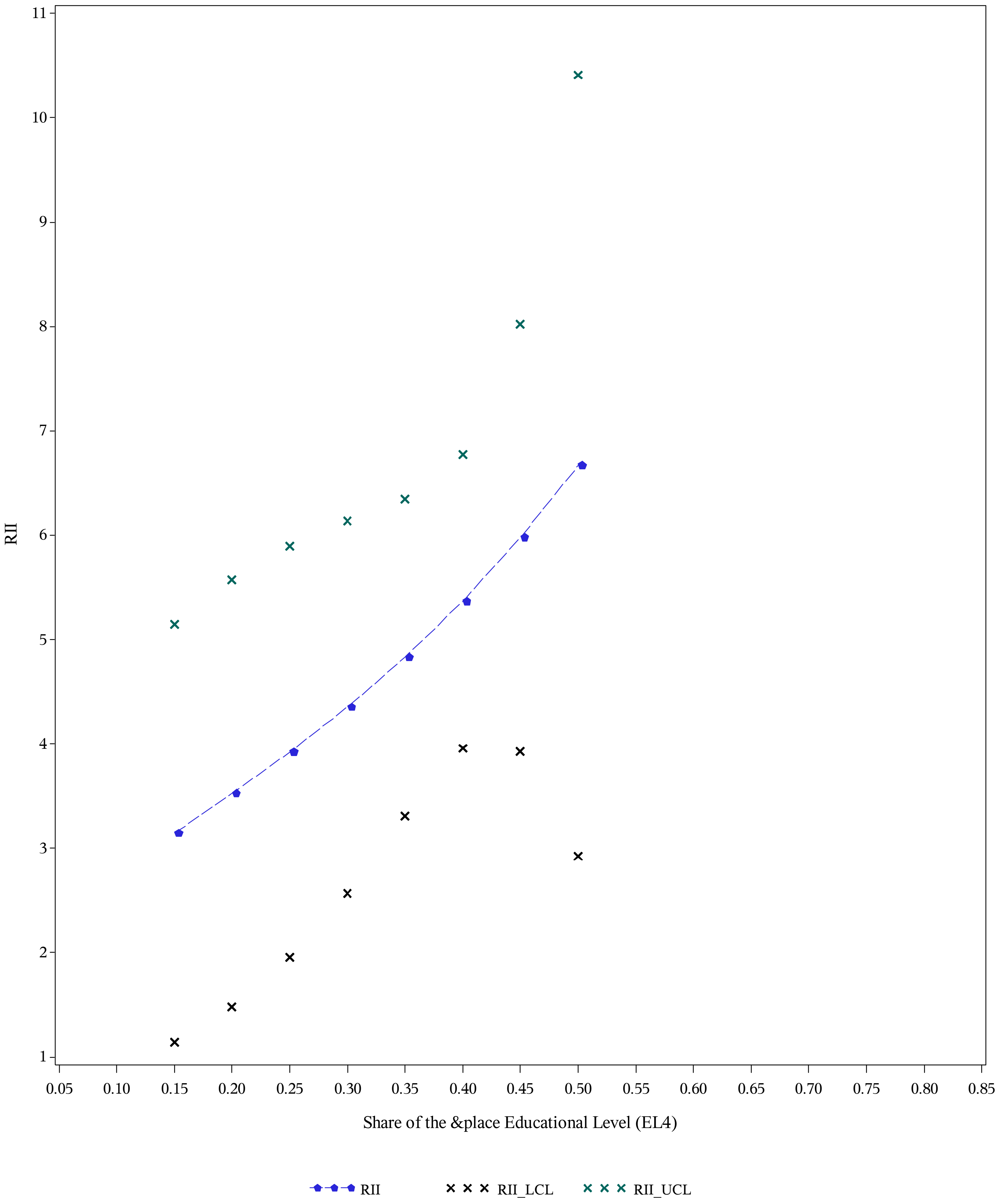

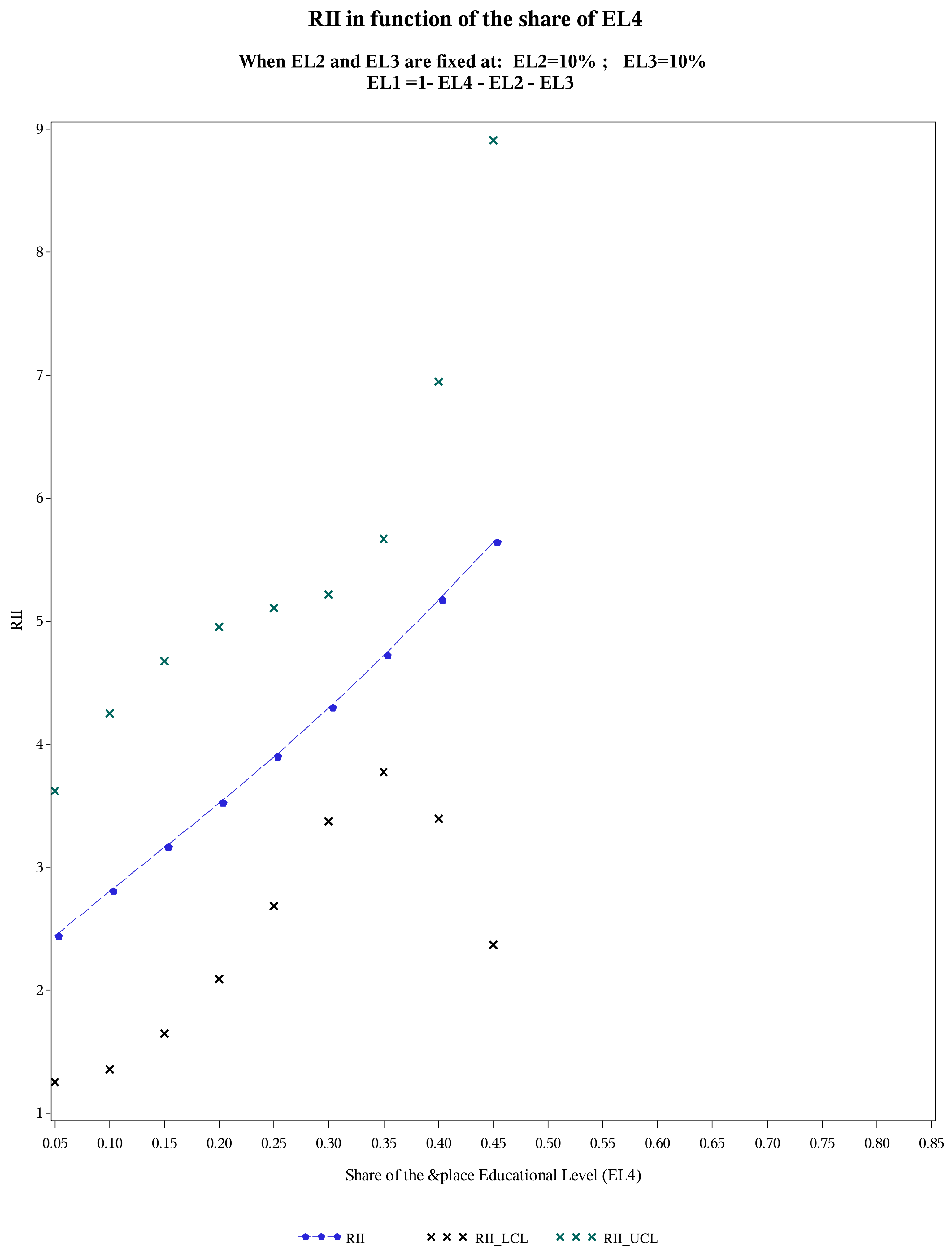

RII in function of the share of EL4
When EL2 and EL3 are fixed at: EL2=10% ; EL3=10%
EL1 =1- EL4 - EL2 - EL3
RII
1
2
3
4
5
6
7
8
9
0.05
0.10
0.15
0.20
0.25
0.30
0.35
0.40
0.45
0.50
0.55
0.60
0.65
0.70
0.75
0.80
0.85
Share of the &place Educational Level (EL4)
RII
RII_LCL
RII_UCL

RII in function of the share of EL4

When EL2 and EL3 are fixed at: EL2=10% ; EL3=15%
EL1 =1- EL4 - EL2 - EL3

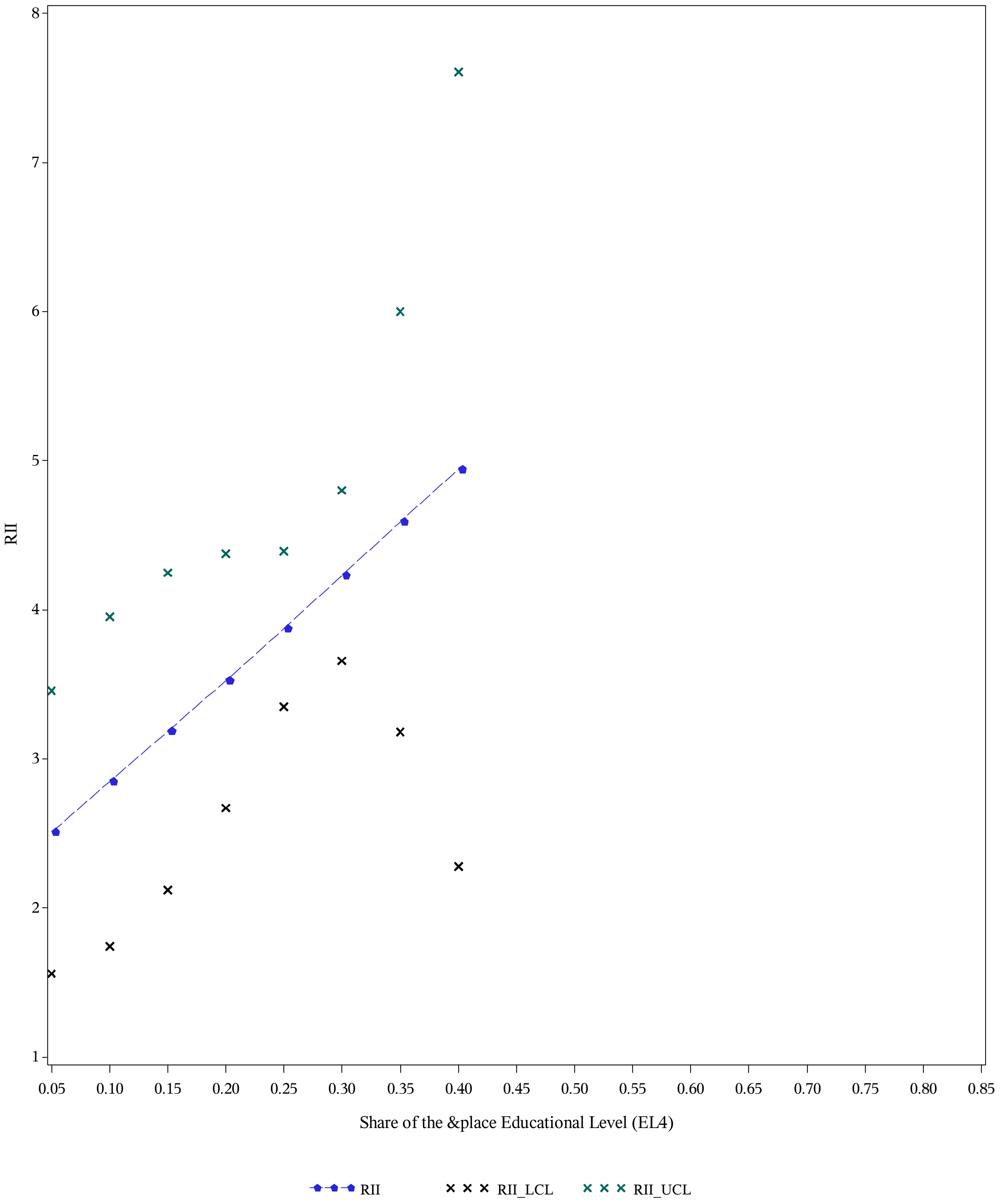

RII in function of the share of EL4

When EL2 and EL3 are fixed at: EL2=10% ; EL3=20%
EL1 =1- EL4 - EL2 - EL3

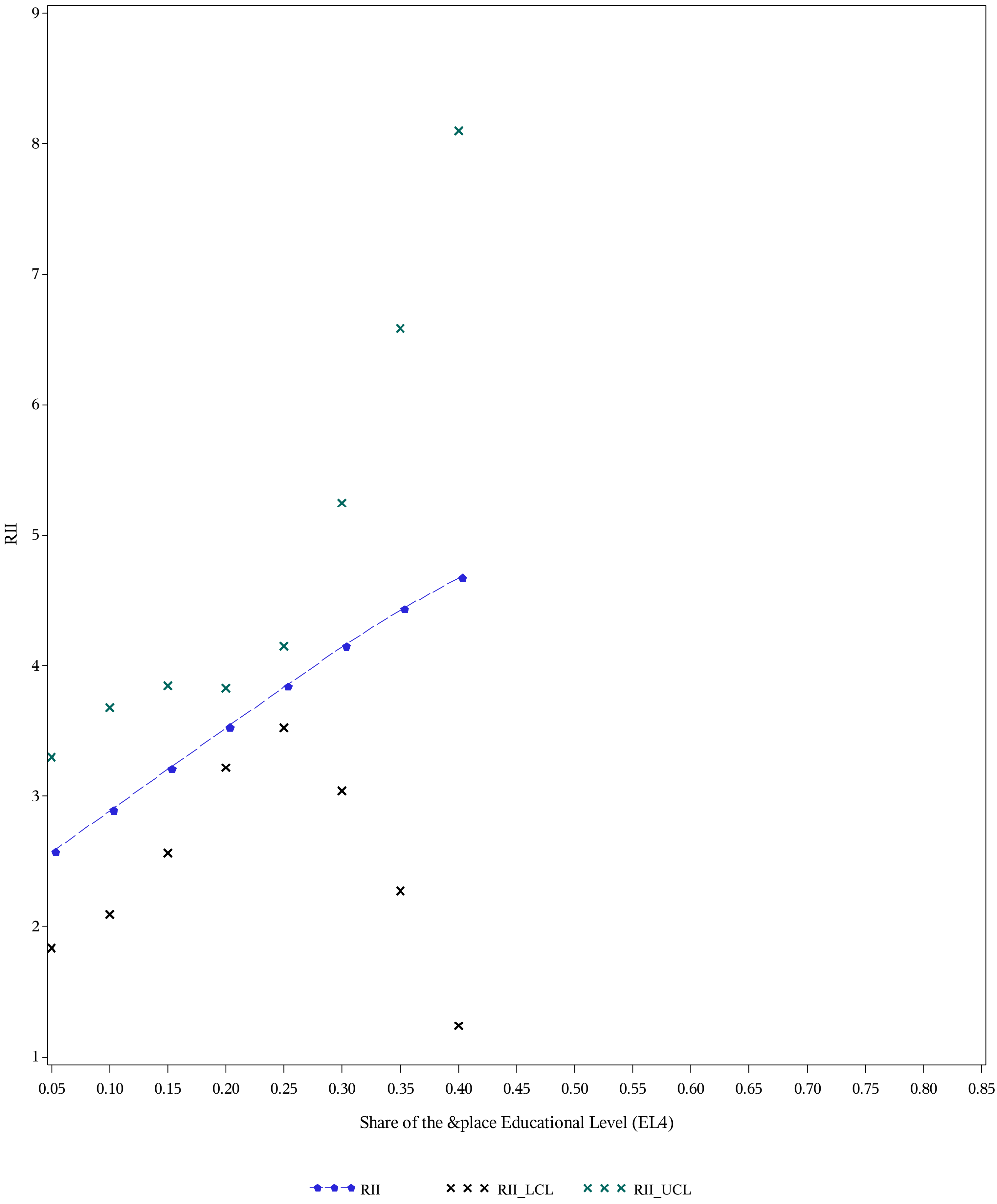

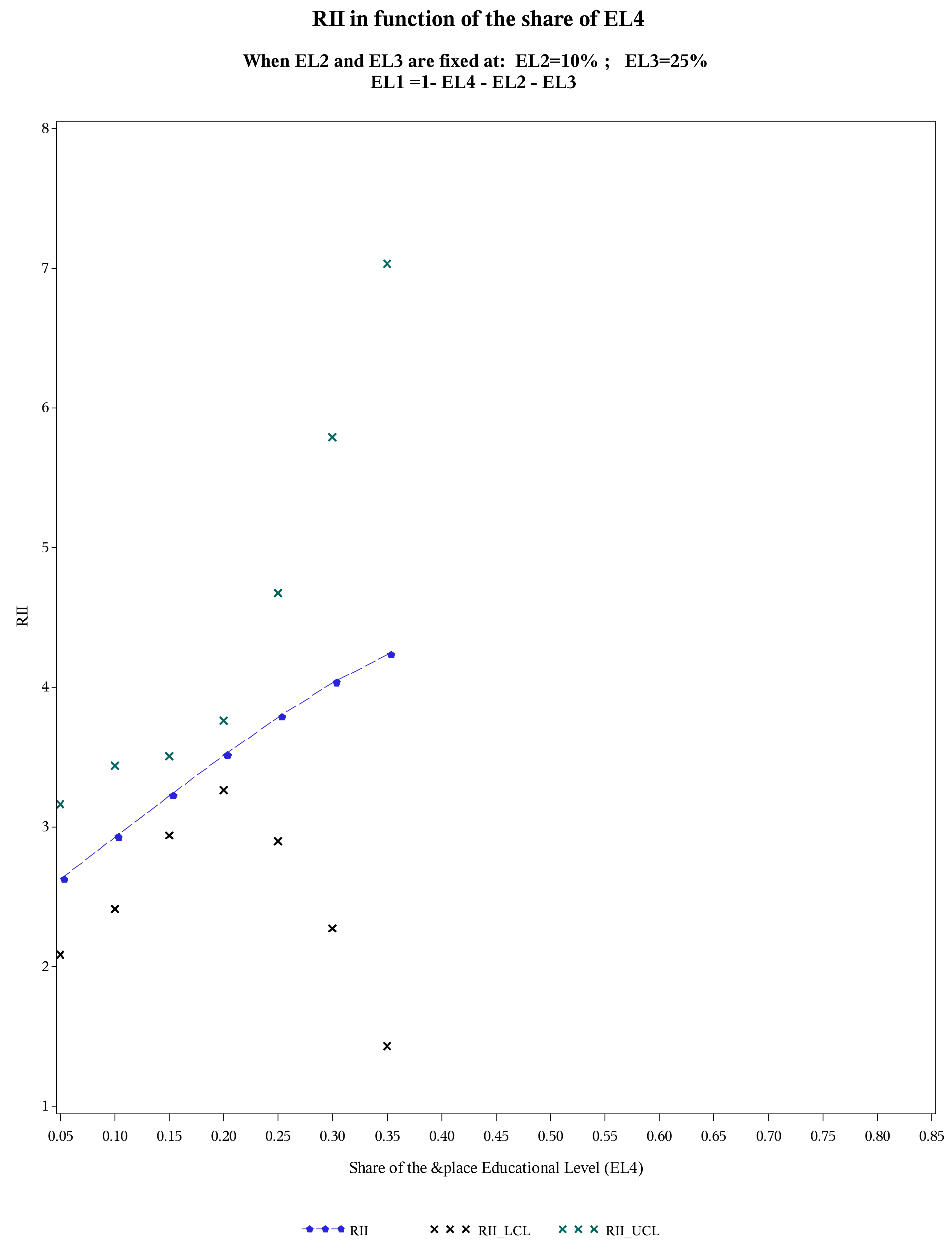
RII in function of the share of EL4
When EL2 and EL3 are fixed at: EL2=10% ; EL3=25%
EL1 =1- EL4 - EL2 - EL3
RII
1
2
3
4
5
6
7
8
0.05
0.10
0.15
0.20
0.25
0.30
0.35
0.40
0.45
0.50
0.55
0.60
0.65
0.70
0.75
0.80
0.85
Share of the &place Educational Level (EL4)
RII
RII_LCL
RII_UCL

RII in function of the share of EL4

When EL2 and EL3 are fixed at: EL2=10% ; EL3=30%
EL1 =1- EL4 - EL2 - EL3

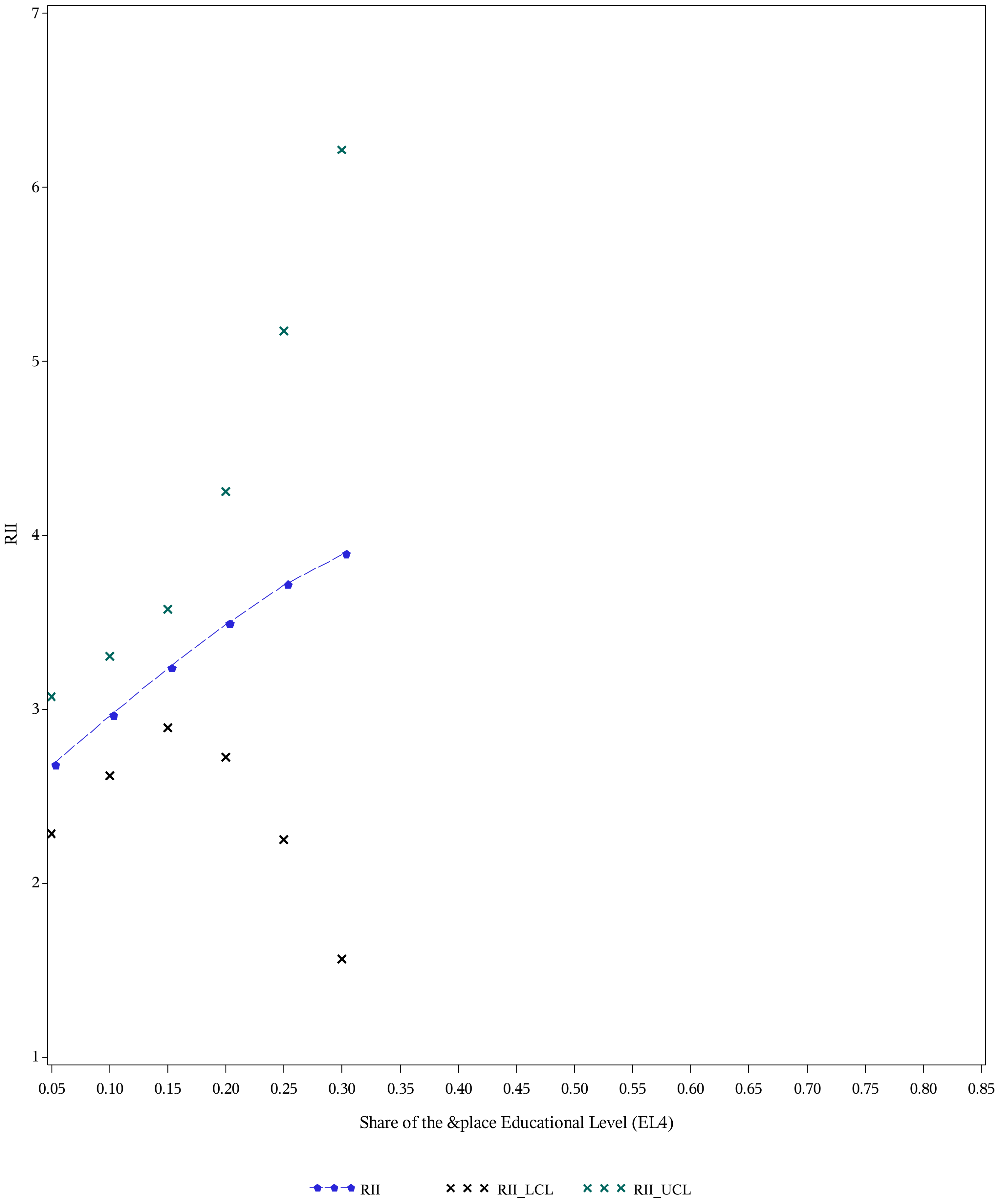

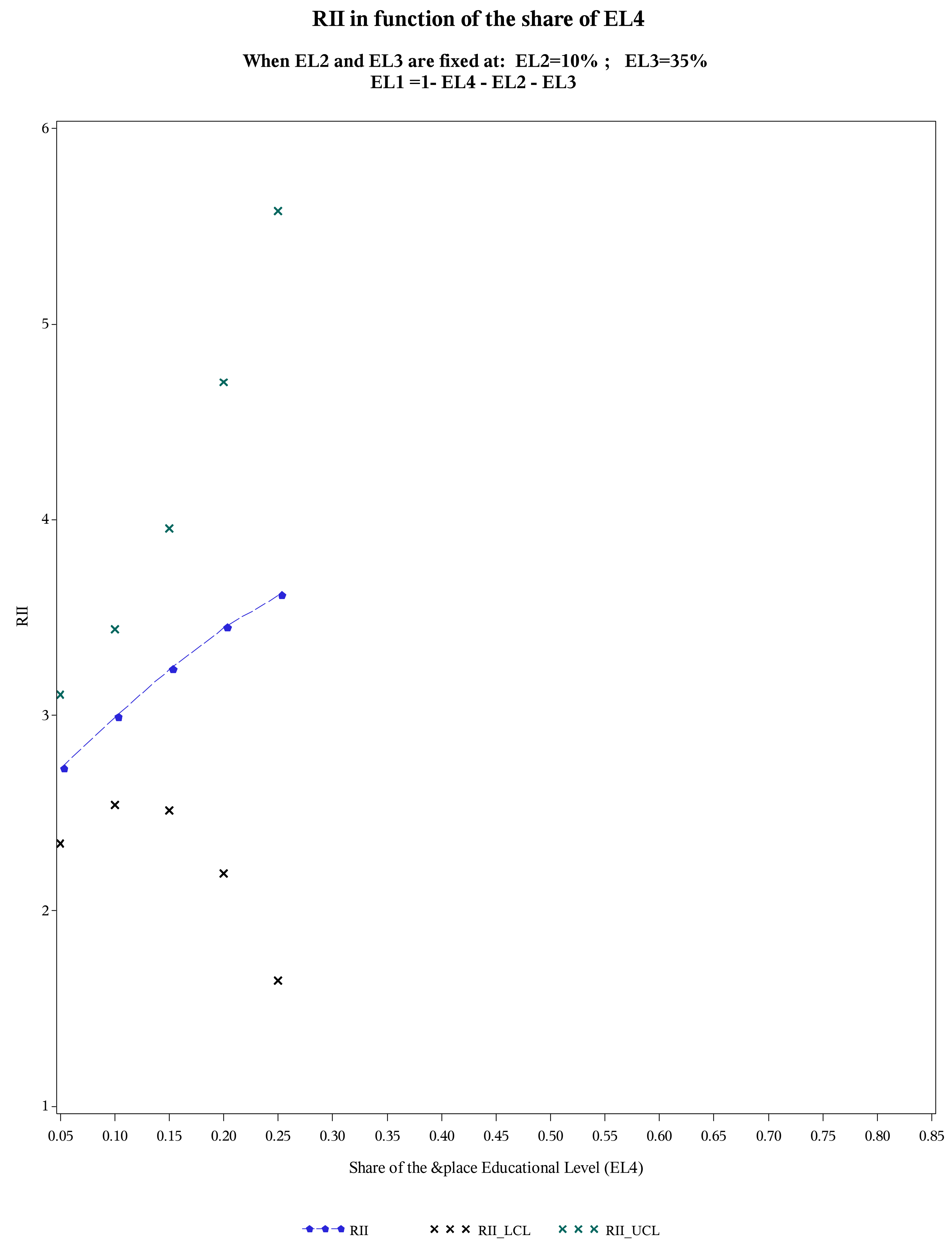

RII in function of the share of EL4
When EL2 and EL3 are fixed at: EL2=10% ; EL3=35%
EL1 =1- EL4 - EL2 - EL3
RII
1
2
3
4
5
6
0.05
0.10
0.15
0.20
0.25
0.30
0.35
0.40
0.45
0.50
0.55
0.60
0.65
0.70
0.75
0.80
0.85
Share of the &place Educational Level (EL4)
RII
RII_LCL
RII_UCL

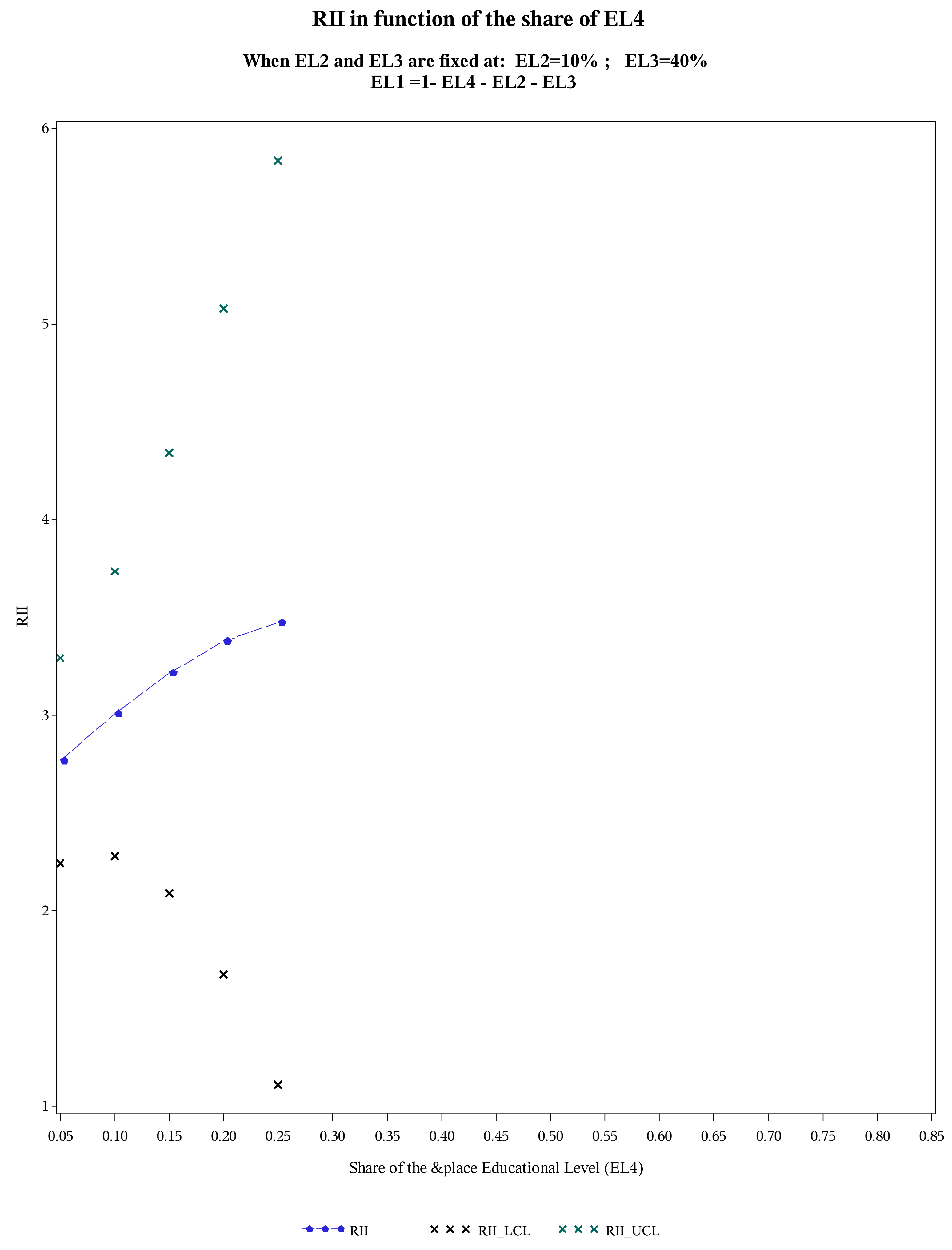
RII in function of the share of EL4
When EL2 and EL3 are fixed at: EL2=10% ; EL3=40%
EL1 =1- EL4 - EL2 - EL3
RII
6
5
4
3
2
1
0.05
0.10
0.15
0.20
0.25
0.30
0.35
0.40
0.45
0.50
0.55
0.60
0.65
0.70
0.75
0.80
0.85
Share of the &place Educational Level (EL4)
RII
RII_LCL
RII_UCL

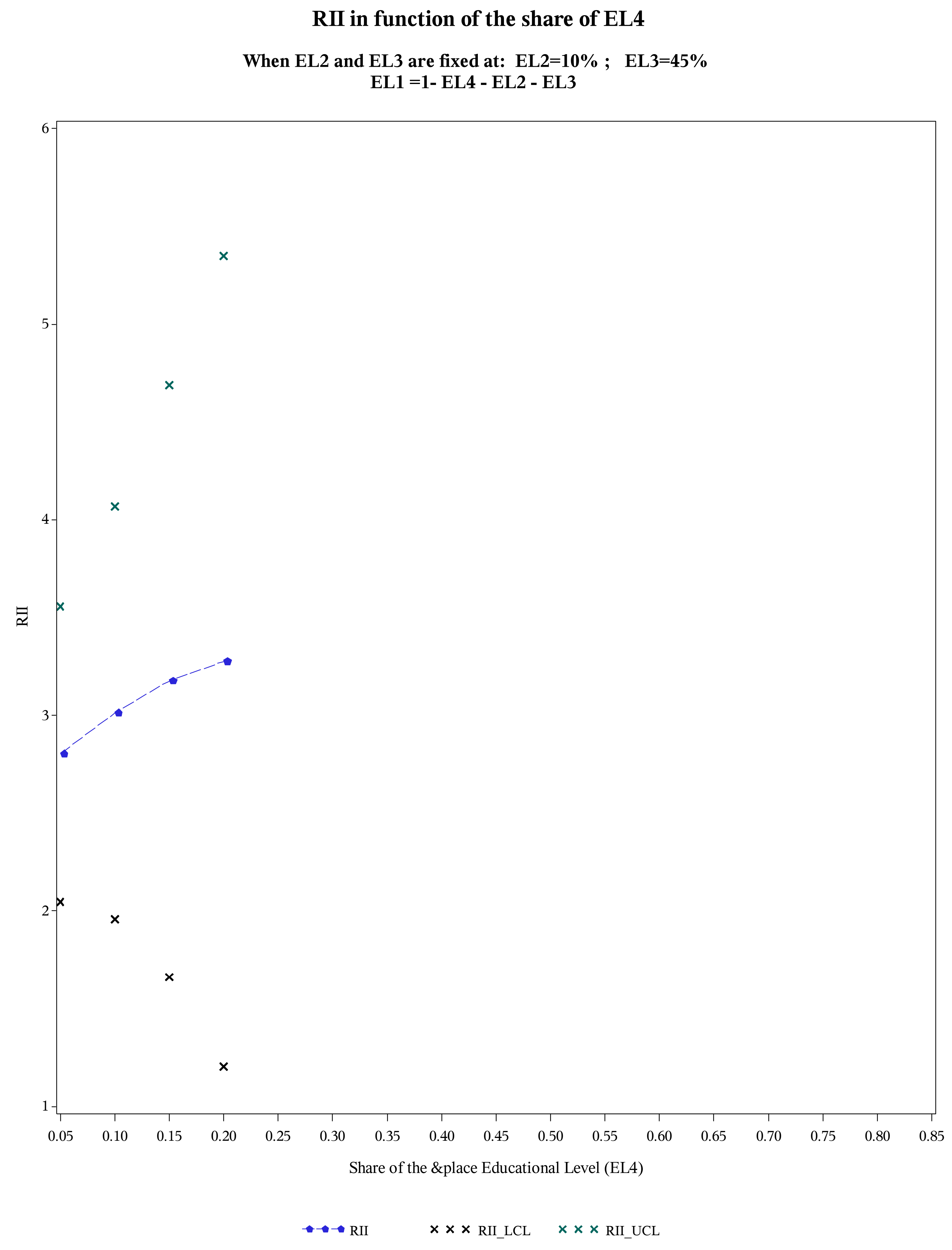

RII in function of the share of EL4
When EL2 and EL3 are fixed at: EL2=10% ; EL3=45%
EL1 =1- EL4 - EL2 - EL3
RII
1
2
3
4
5
6
0.05
0.10
0.15
0.20
0.25
0.30
0.35
0.40
0.45
0.50
0.55
0.60
0.65
0.70
0.75
0.80
0.85
Share of the &place Educational Level (EL4)
RII
RII_LCL
RII_UCL

RII in function of the share of EL4

When EL2 and EL3 are fixed at: EL2=10% ; EL3=50%
EL1 =1- EL4 - EL2 - EL3

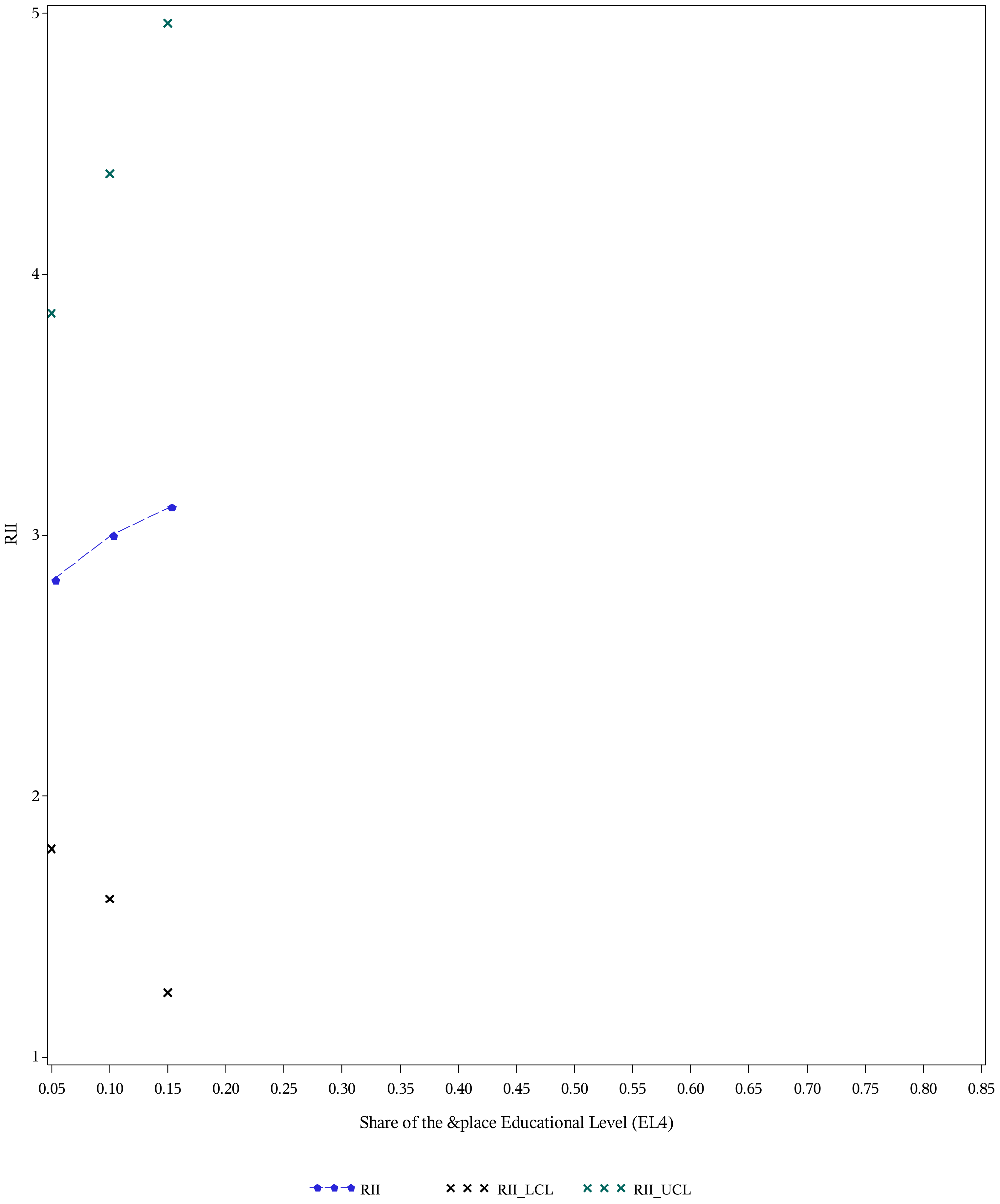

RII in function of the share of EL4

When EL2 and EL3 are fixed at: EL2=10% ; EL3=55%
EL1 =1- EL4 - EL2 - EL3

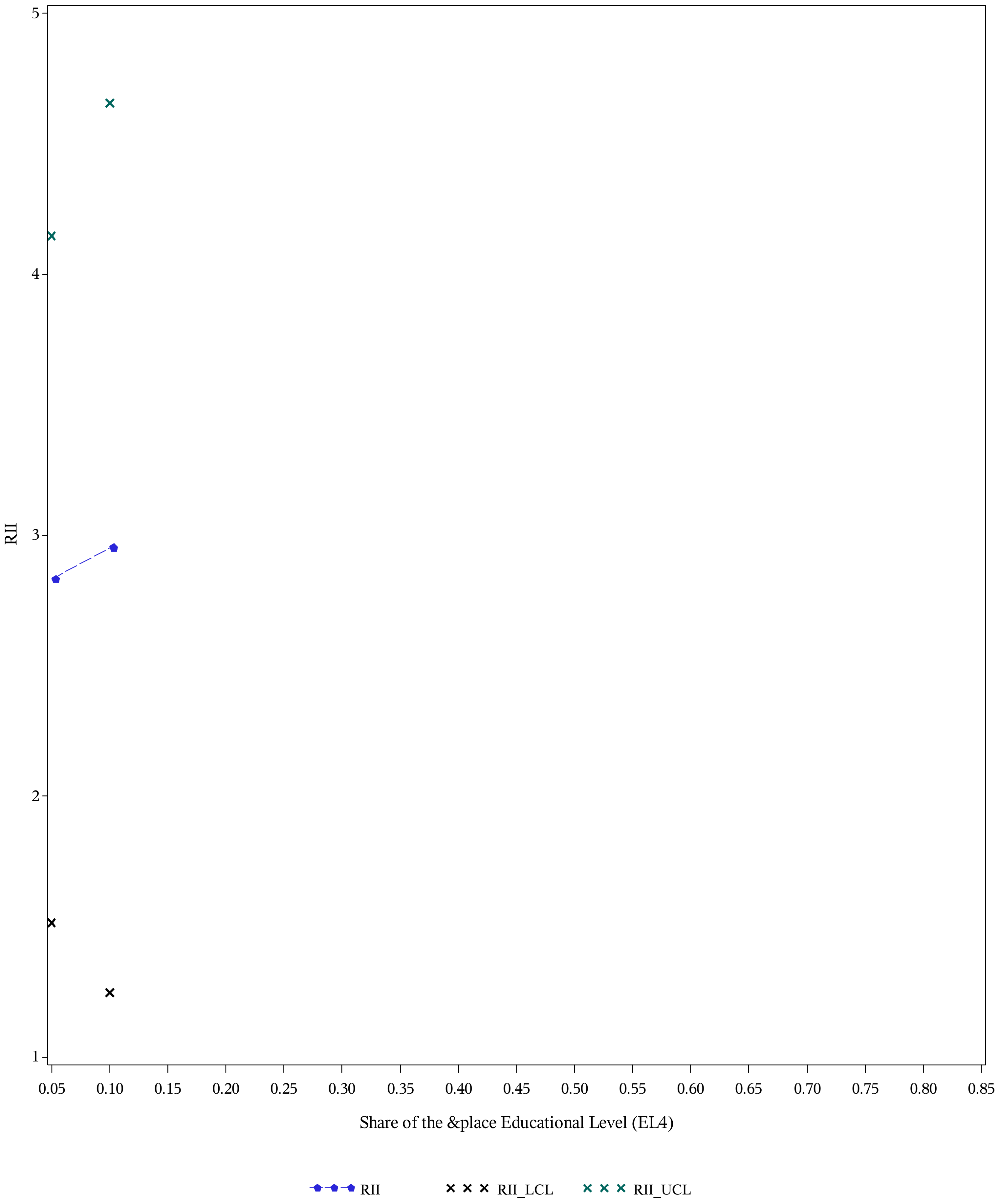

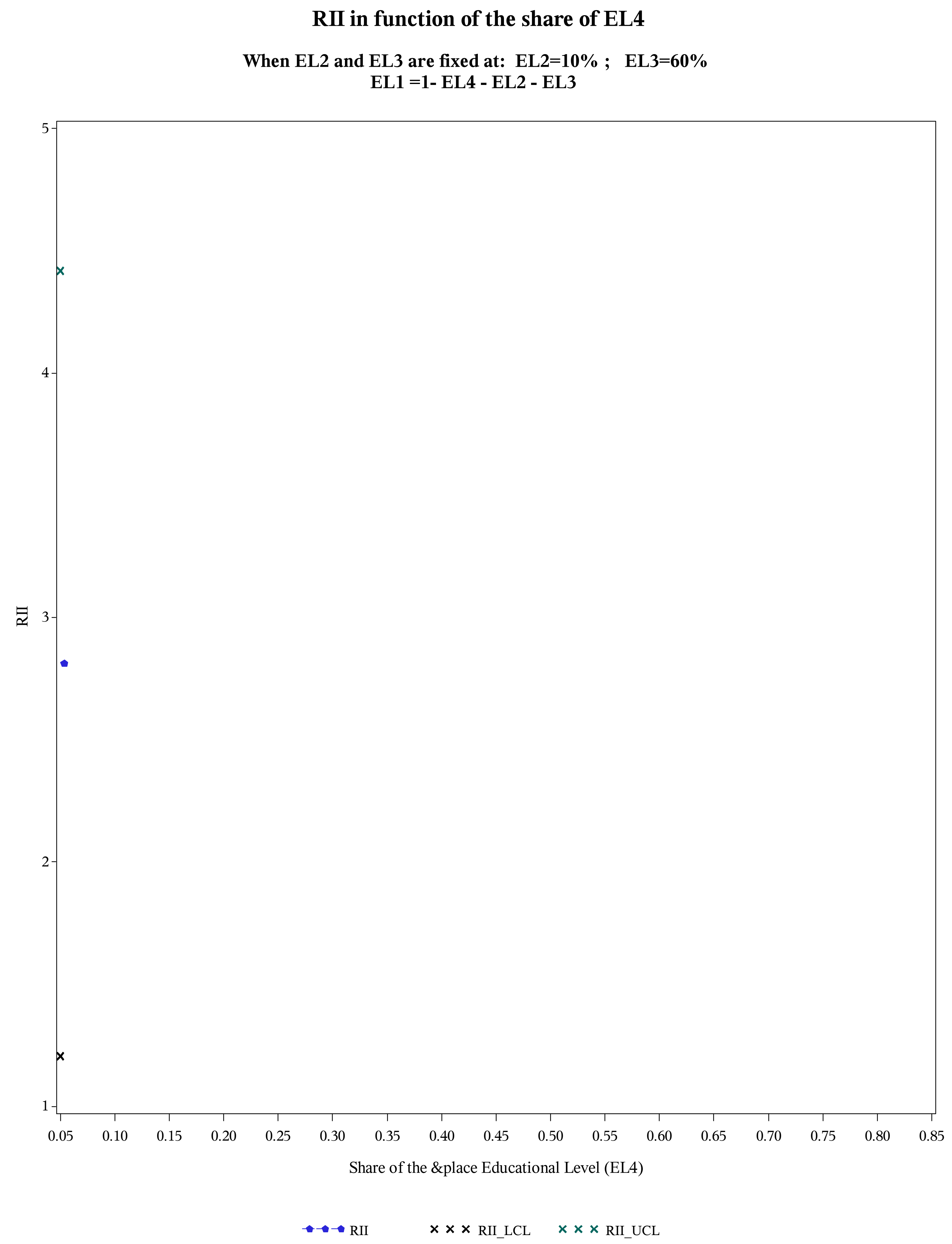

RII in function of the share of EL4
When EL2 and EL3 are fixed at: EL2=10% ; EL3=60%
EL1 =1- EL4 - EL2 - EL3
RII
5
4
3
2
1
0.05
0.10
0.15
0.20
0.25
0.30
0.35
0.40
0.45
0.50
0.55
0.60
0.65
0.70
0.75
0.80
0.85
Share of the &place Educational Level (EL4)
RII
RII_LCL
RII_UCL

RII in function of the share of EL4

When EL2 and EL3 are fixed at: EL2=15% ; EL3=5%
EL1 =1- EL4 - EL2 - EL3

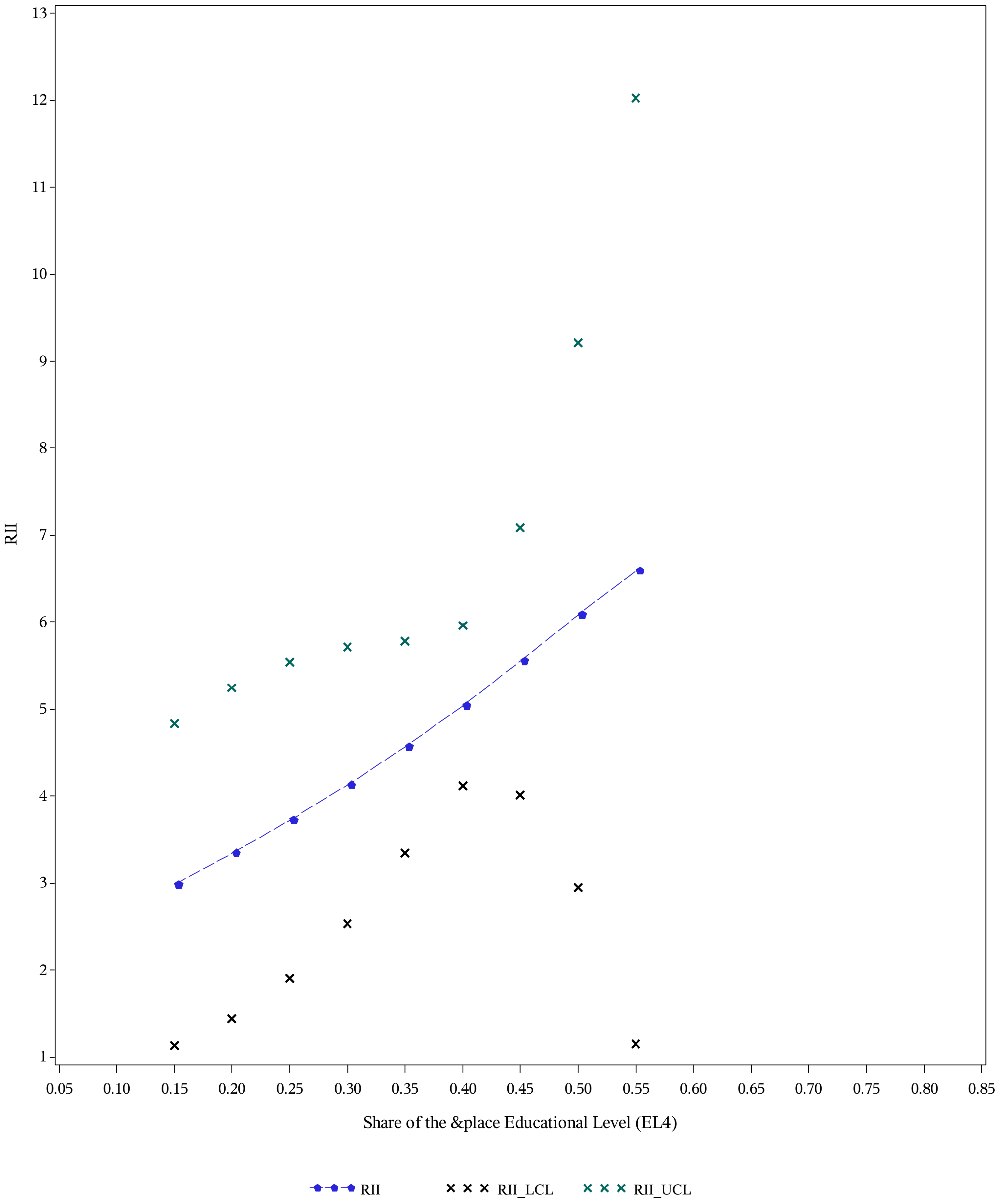

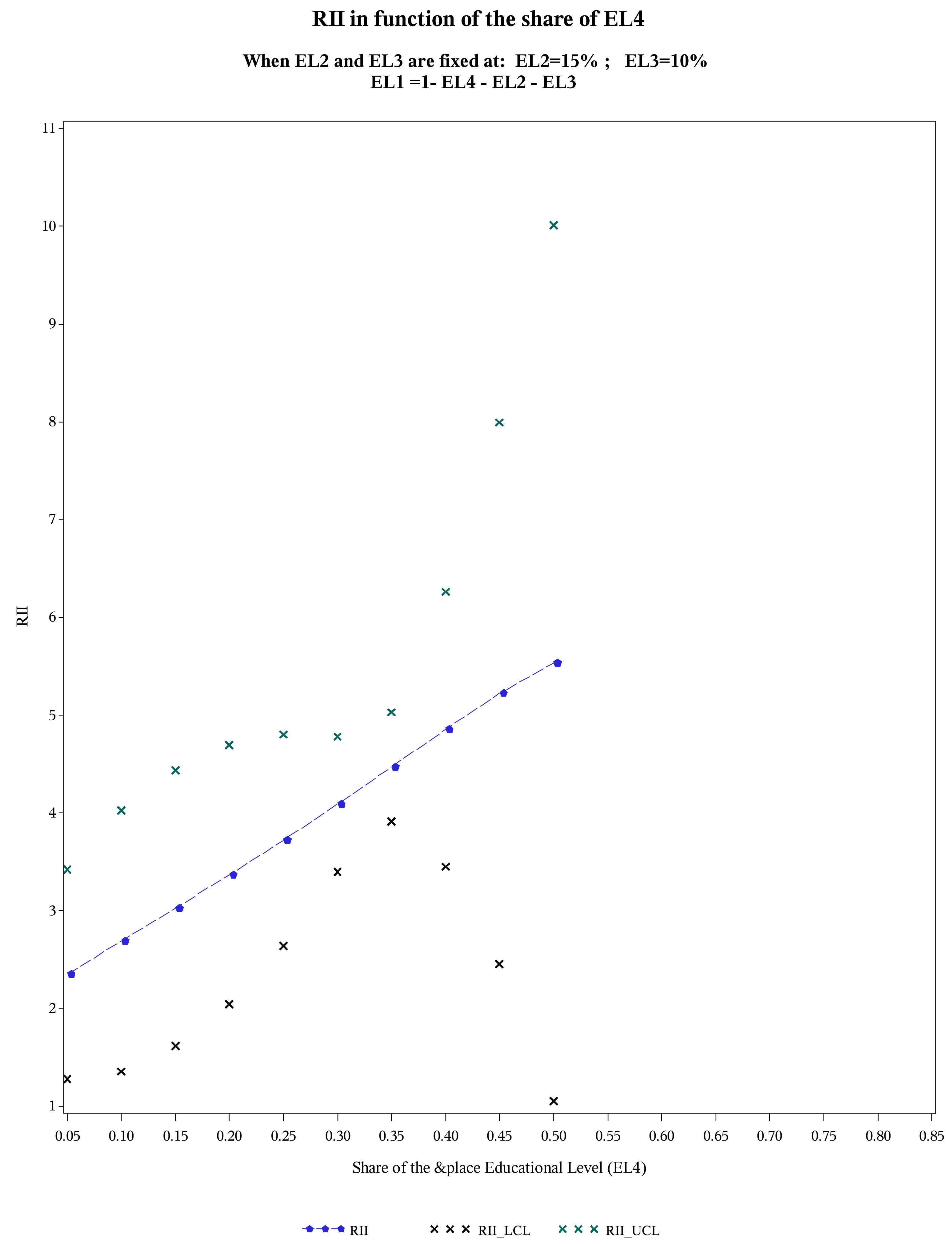
RII in function of the share of EL4
When EL2 and EL3 are fixed at: EL2=15% ; EL3=10%
EL1 =1- EL4 - EL2 - EL3
RII
1
2
3
4
5
6
7
8
9
10
11
0.05
0.10
0.15
0.20
0.25
0.30
0.35
0.40
0.45
0.50
0.55
0.60
0.65
0.70
0.75
0.80
0.85
Share of the &place Educational Level (EL4)
RII
RII_LCL
RII_UCL

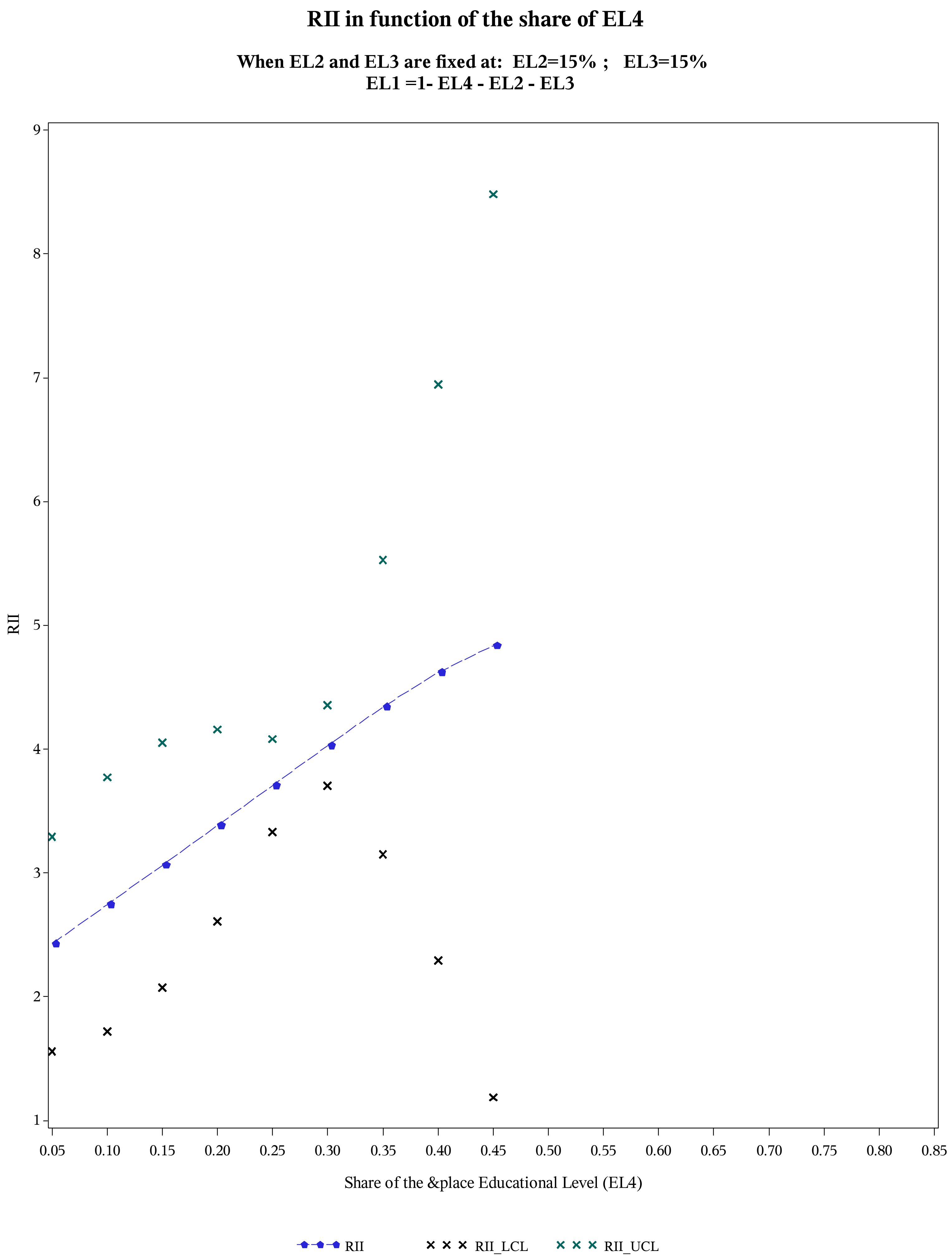
RII in function of the share of EL4
When EL2 and EL3 are fixed at: EL2=15% ; EL3=15%
EL1 =1- EL4 - EL2 - EL3
RII
1
2
3
4
5
6
7
8
9
0.05
0.10
0.15
0.20
0.25
0.30
0.35
0.40
0.45
0.50
0.55
0.60
0.65
0.70
0.75
0.80
0.85
Share of the &place Educational Level (EL4)
RII
RII_LCL
RII_UCL

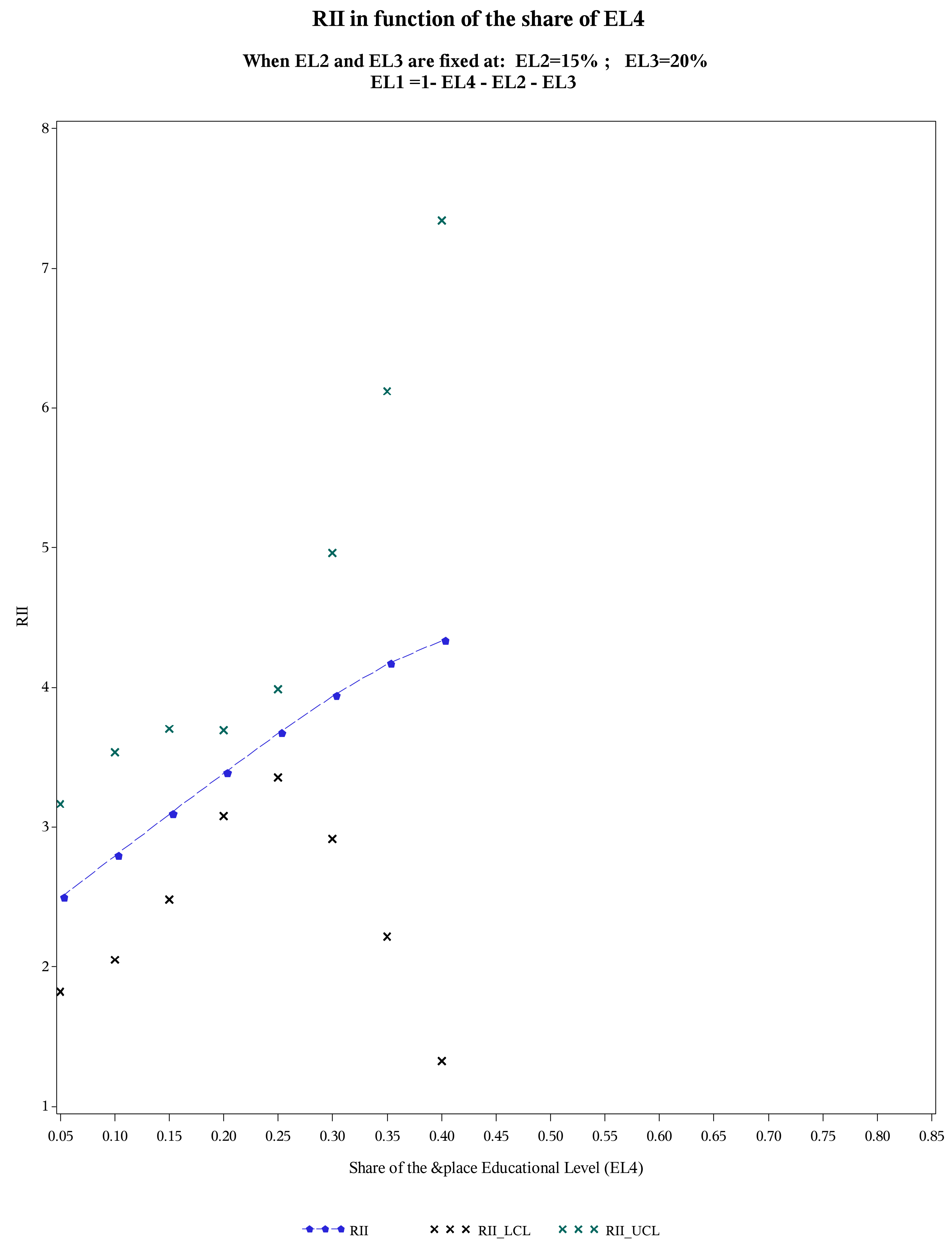
RII in function of the share of EL4
When EL2 and EL3 are fixed at: EL2=15% ; EL3=20%
EL1 =1- EL4 - EL2 - EL3
RII
Share of the &place Educational Level (EL4)
RII
RII_LCL
RII_UCL

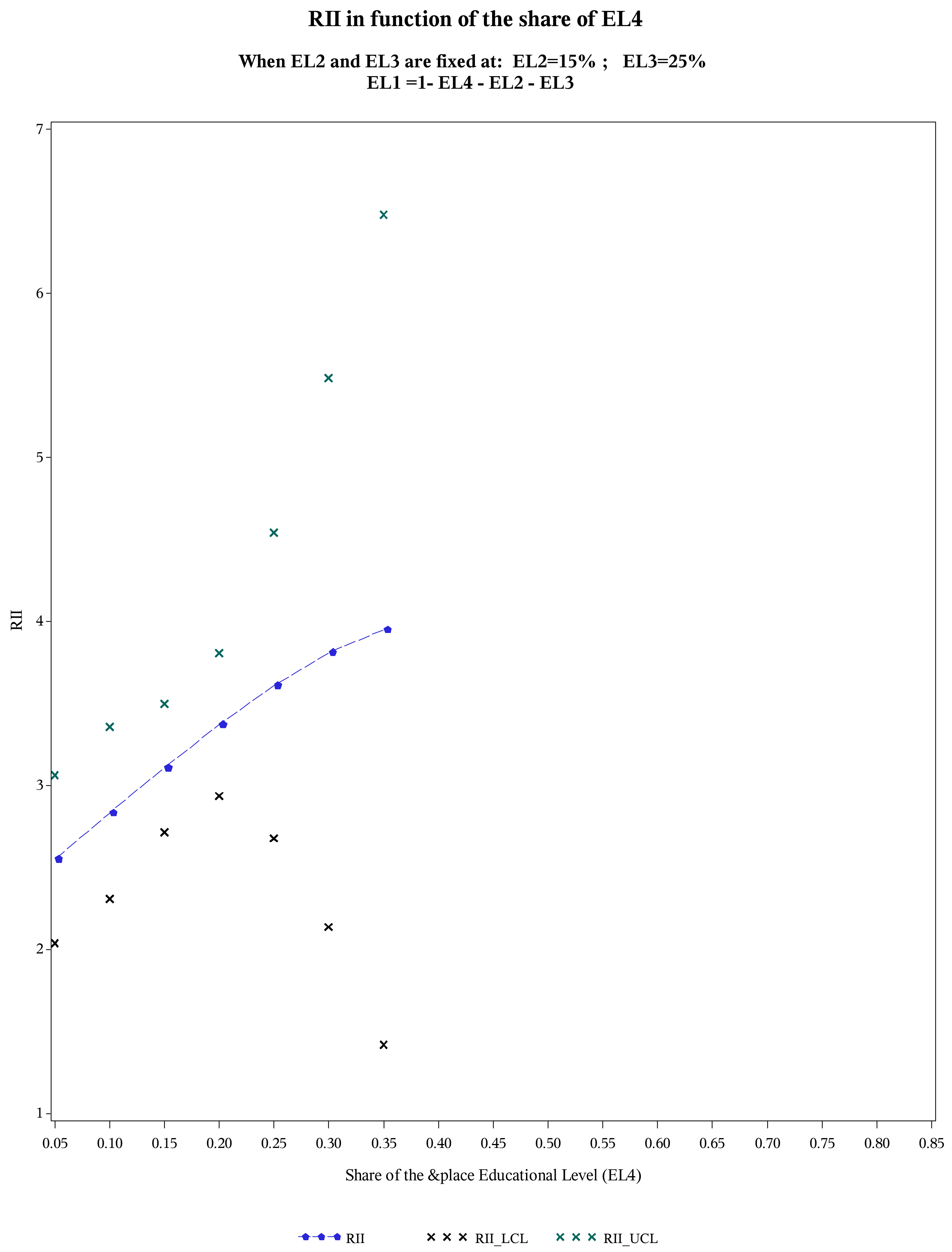
RII in function of the share of EL4
When EL2 and EL3 are fixed at: EL2=15% ; EL3=25%
EL1 =1- EL4 - EL2 - EL3
RII
1
2
3
4
5
6
7
0.05
0.10
0.15
0.20
0.25
0.30
0.35
0.40
0.45
0.50
0.55
0.60
0.65
0.70
0.75
0.80
0.85
Share of the &place Educational Level (EL4)
RII
RII_LCL
RII_UCL

RII in function of the share of EL4

When EL2 and EL3 are fixed at: EL2=15% ; EL3=30%
EL1 =1- EL4 - EL2 - EL3

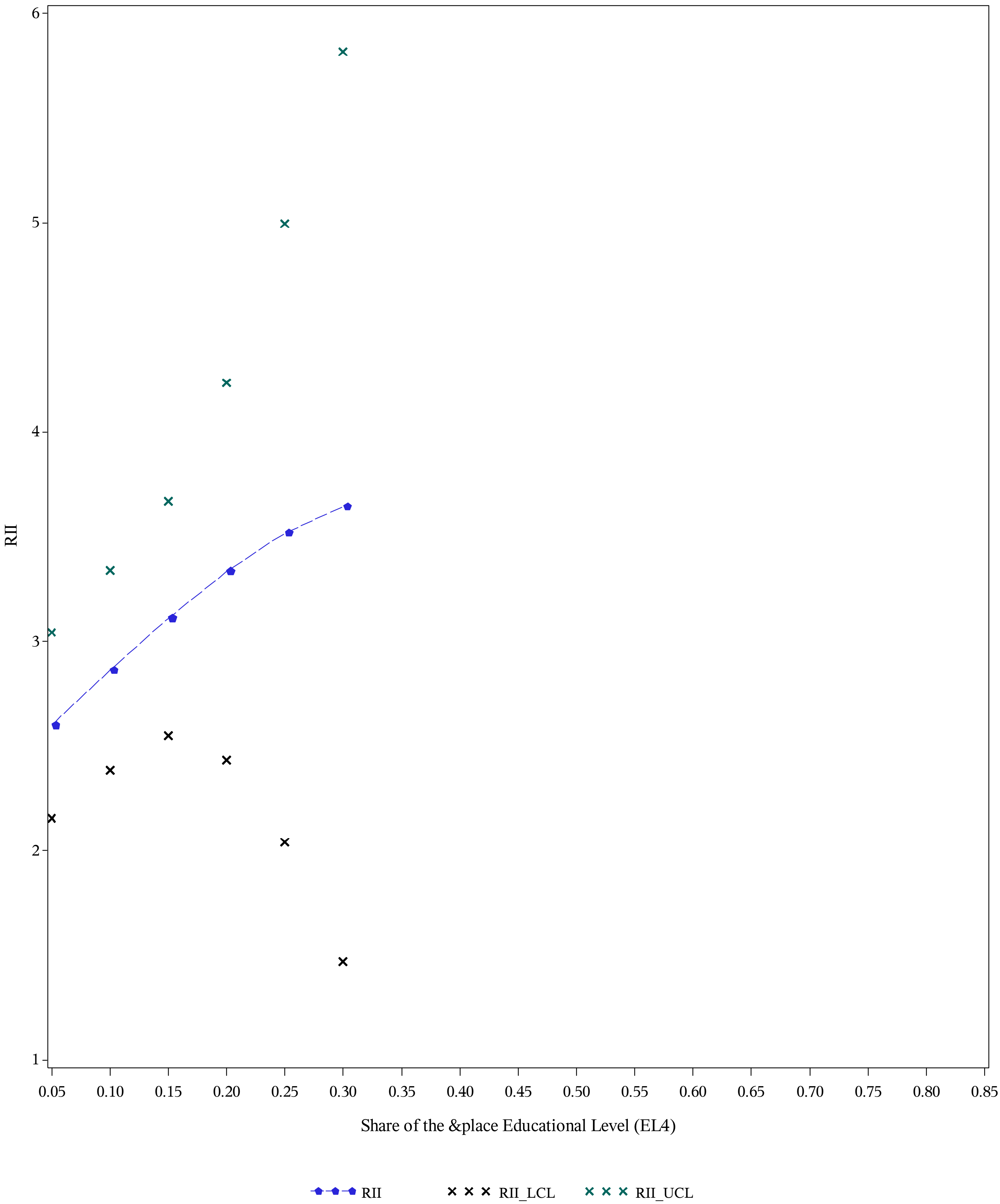

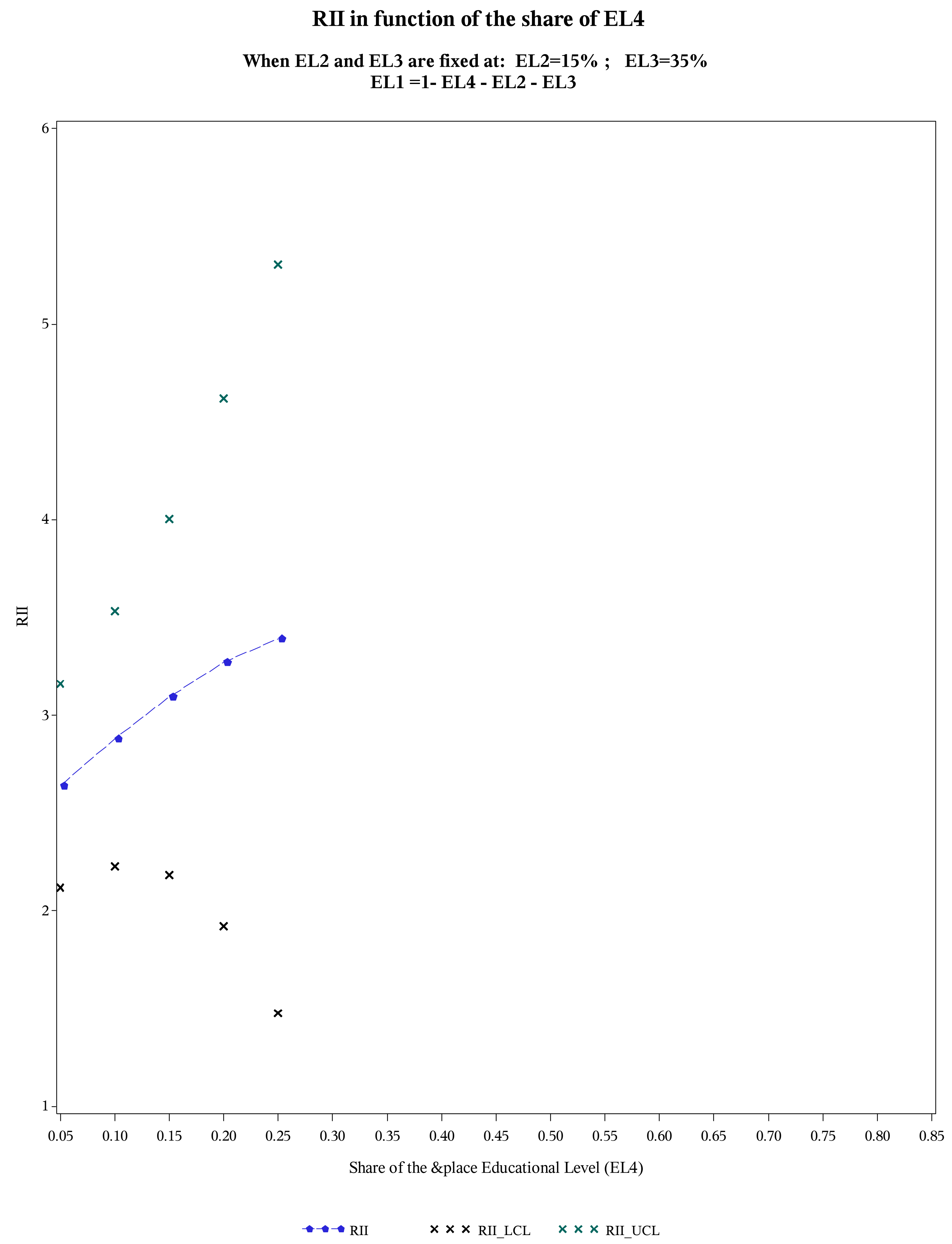
RII in function of the share of EL4
When EL2 and EL3 are fixed at: EL2=15% ; EL3=35%
EL1 =1- EL4 - EL2 - EL3
RII
1
2
3
4
5
6
0.05
0.10
0.15
0.20
0.25
0.30
0.35
0.40
0.45
0.50
0.55
0.60
0.65
0.70
0.75
0.80
0.85
Share of the &place Educational Level (EL4)
RII
RII_LCL
RII_UCL

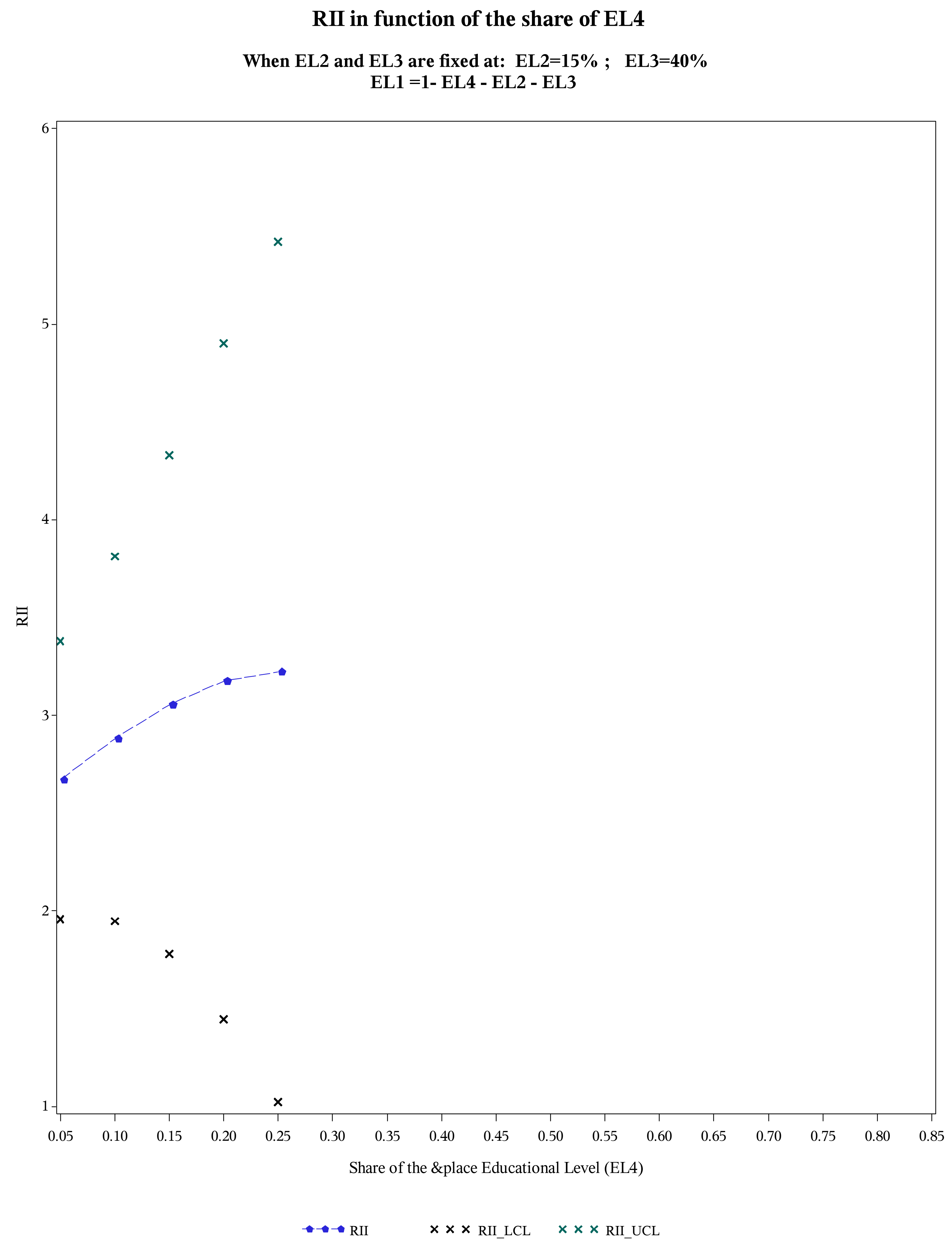
RII in function of the share of EL4
When EL2 and EL3 are fixed at: EL2=15% ; EL3=40%
EL1 =1- EL4 - EL2 - EL3
RII
1
2
3
4
5
6
0.05
0.10
0.15
0.20
0.25
0.30
0.35
0.40
0.45
0.50
0.55
0.60
0.65
0.70
0.75
0.80
0.85
Share of the &place Educational Level (EL4)
RII
RII_LCL
RII_UCL

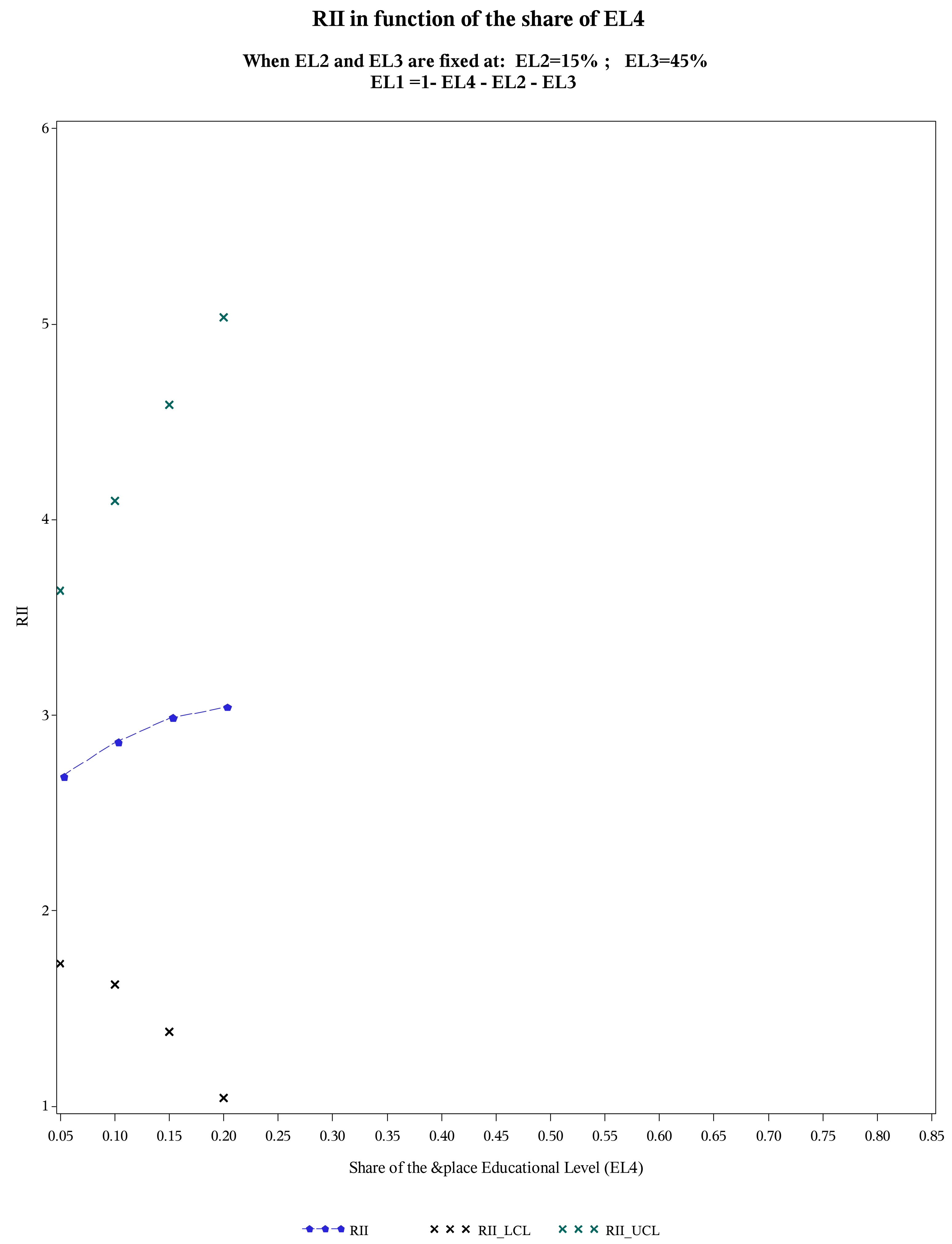

RII in function of the share of EL4
When EL2 and EL3 are fixed at: EL2=15% ; EL3=45%
EL1 =1- EL4 - EL2 - EL3
RII
1
2
3
4
5
6
0.05
0.10
0.15
0.20
0.25
0.30
0.35
0.40
0.45
0.50
0.55
0.60
0.65
0.70
0.75
0.80
0.85
Share of the &place Educational Level (EL4)
RII
RII_LCL
RII_UCL

RII in function of the share of EL4

When EL2 and EL3 are fixed at: EL2=15% ; EL3=50%
EL1 =1- EL4 - EL2 - EL3

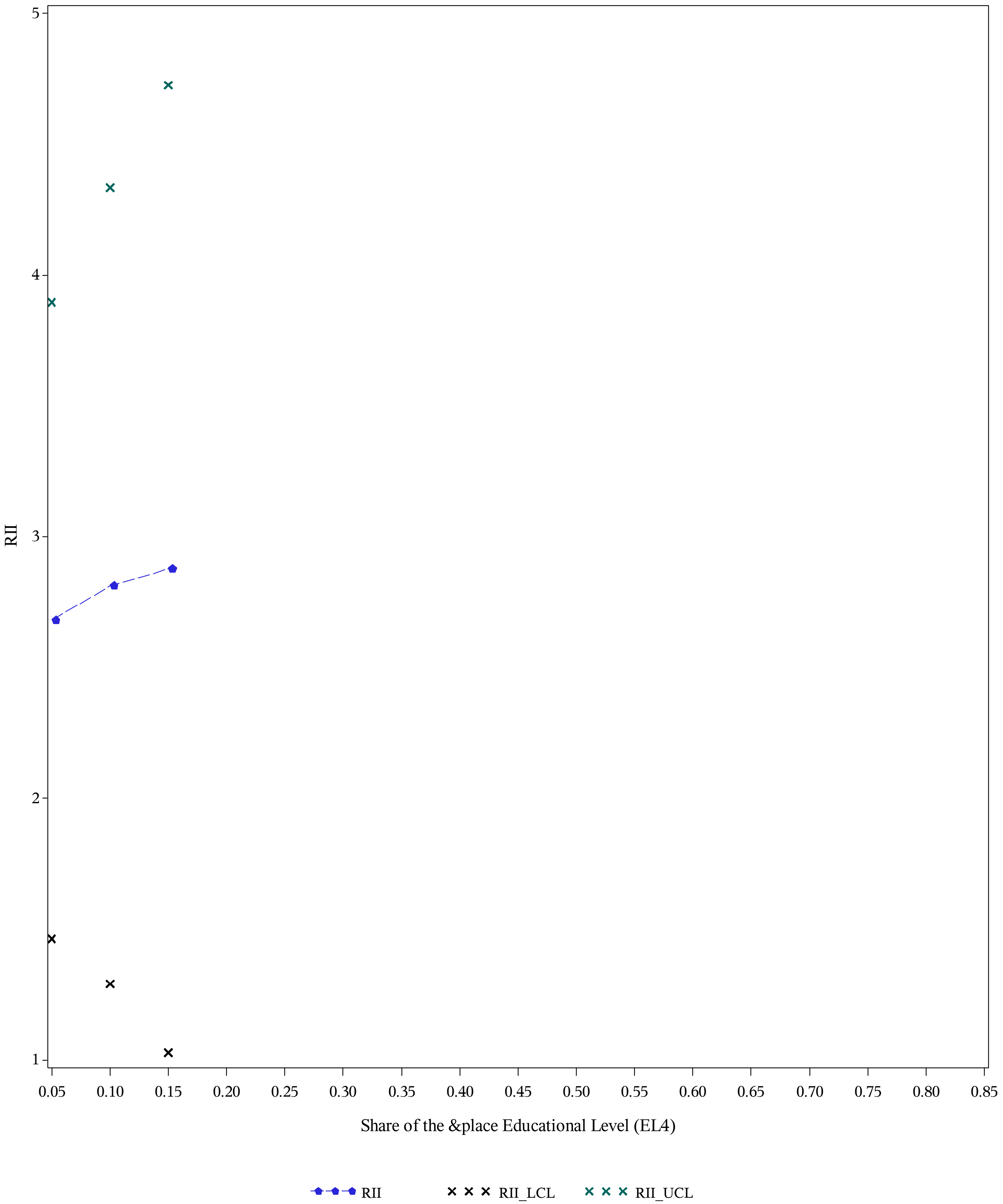

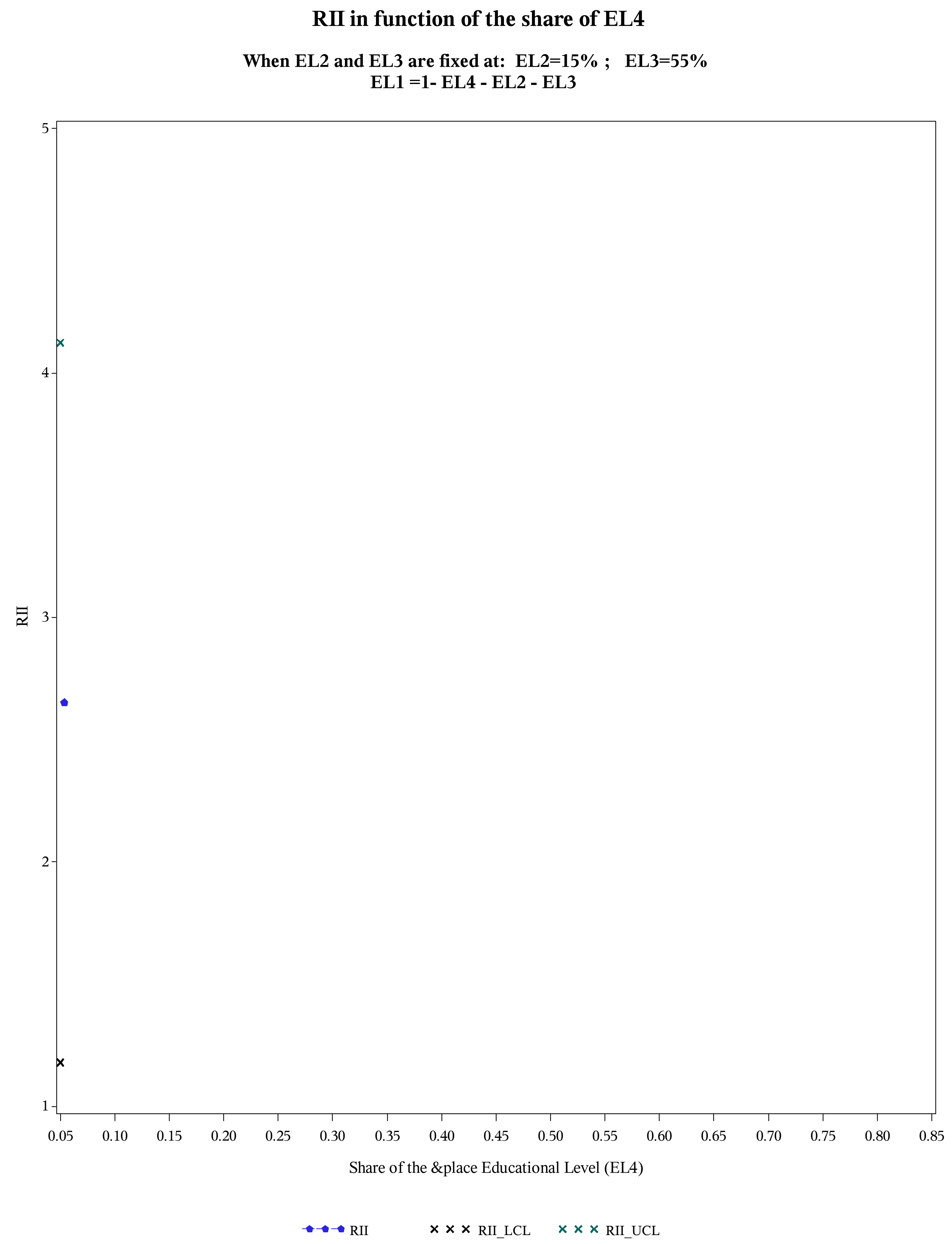

RII in function of the share of EL4
When EL2 and EL3 are fixed at: EL2=15% ; EL3=55%
EL1 =1- EL4 - EL2 - EL3
RII
5
4
3
2
1
0.05
0.10
0.15
0.20
0.25
0.30
0.35
0.40
0.45
0.50
0.55
0.60
0.65
0.70
0.75
0.80
0.85
Share of the &place Educational Level (EL4)
RII
RII_LCL
RII_UCL

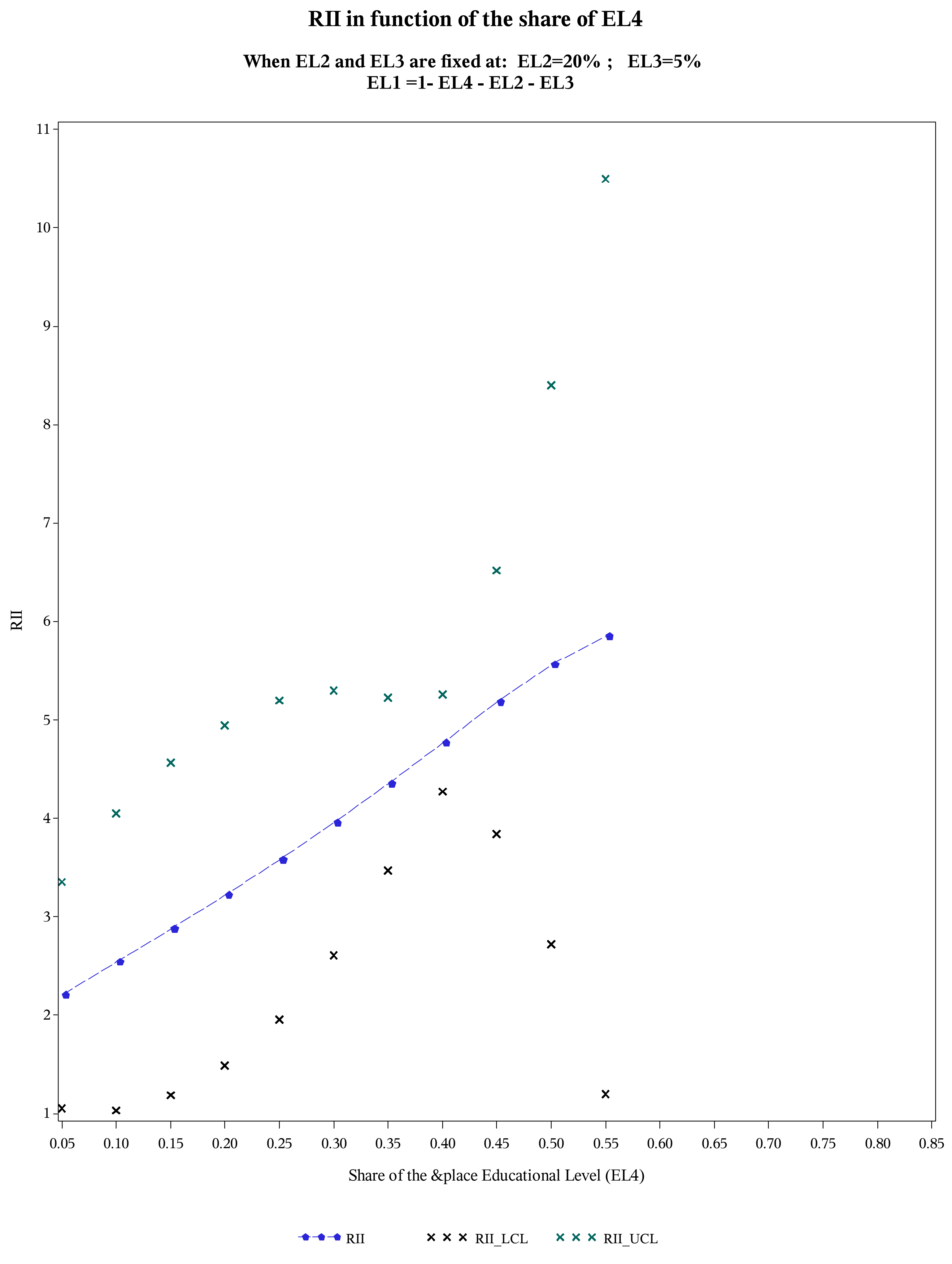
RII in function of the share of EL4
When EL2 and EL3 are fixed at: EL2=20% ; EL3=5%
EL1 =1- EL4 - EL2 - EL3
RII
Share of the &place Educational Level (EL4)
RII
RII_LCL
RII_UCL

RII in function of the share of EL4

When EL2 and EL3 are fixed at: EL2=20% ; EL3=10%
EL1 =1- EL4 - EL2 - EL3

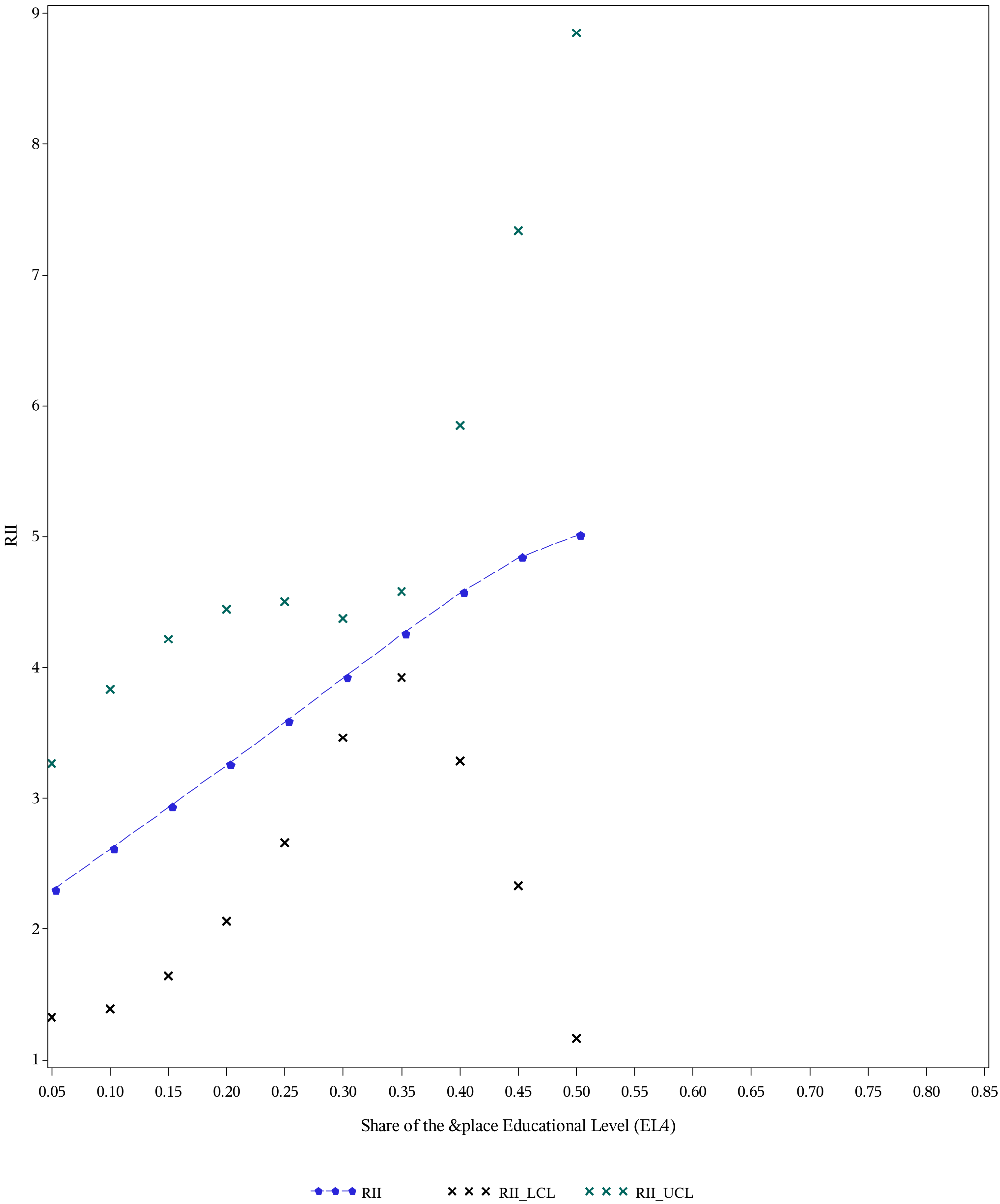

RII in function of the share of EL4

When EL2 and EL3 are fixed at: EL2=20% ; EL3=15%
EL1 =1- EL4 - EL2 - EL3

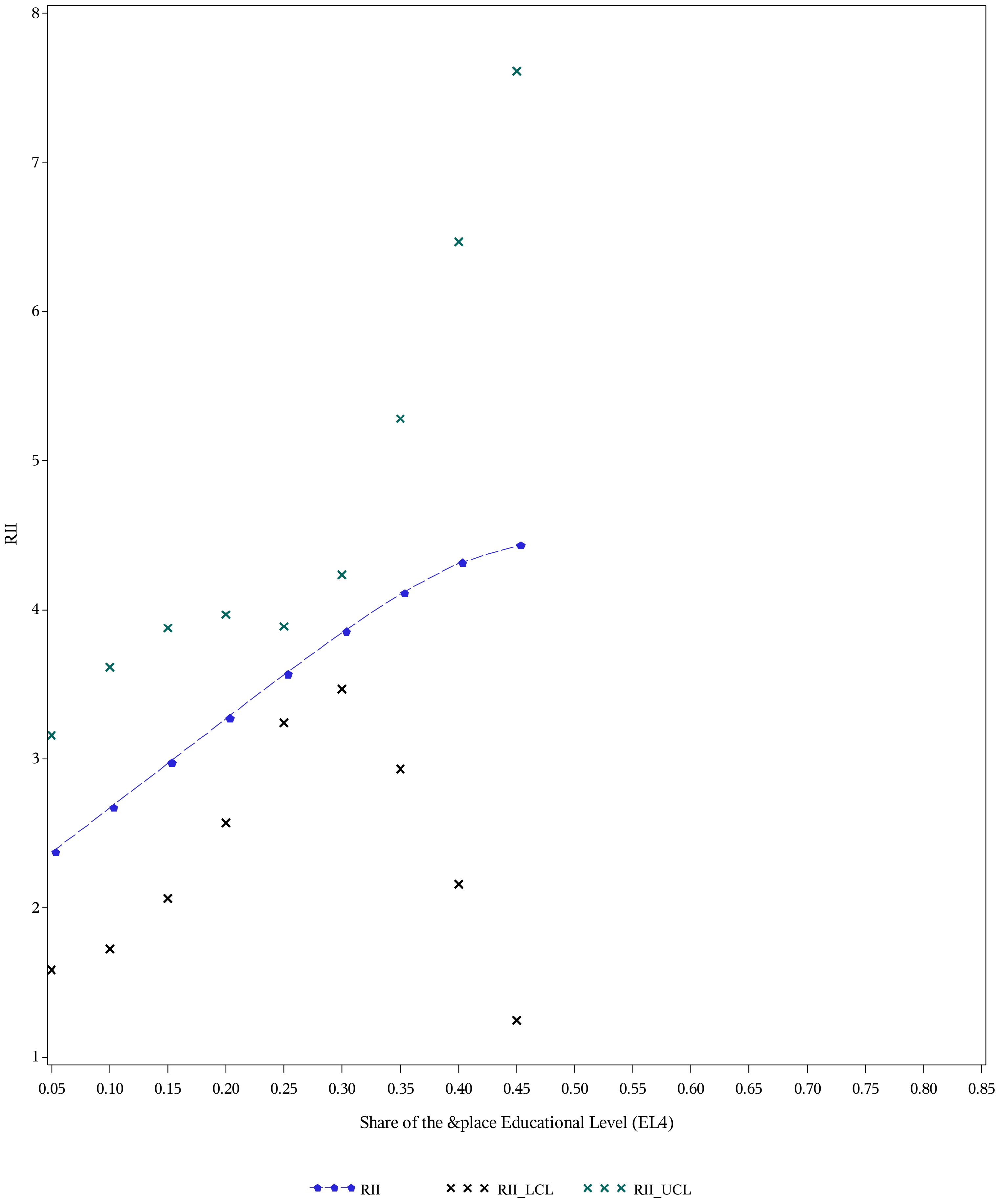

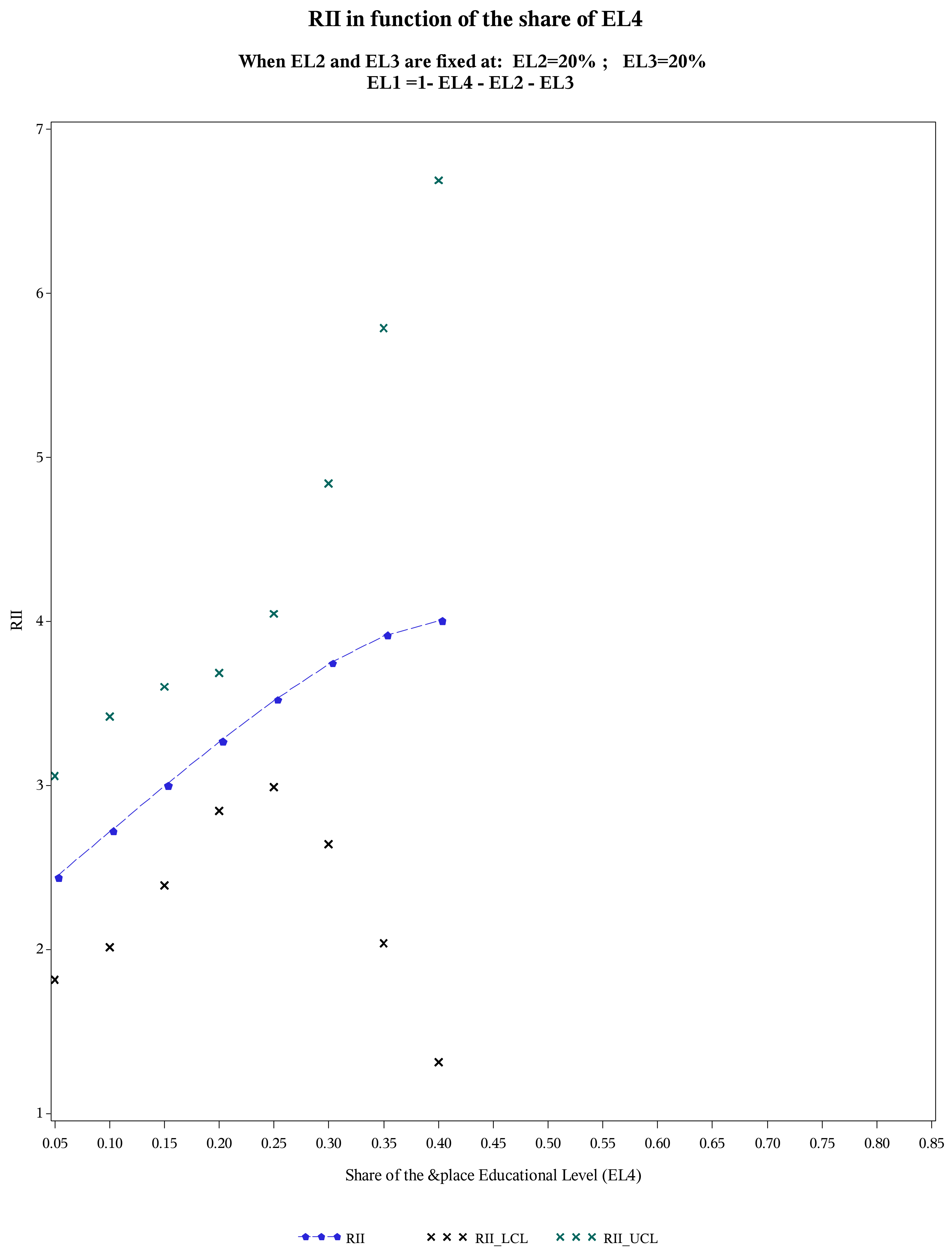

RII in function of the share of EL4
When EL2 and EL3 are fixed at: EL2=20% ; EL3=20%
EL1 =1- EL4 - EL2 - EL3
RII
1
2
3
4
5
6
7
0.05
0.10
0.15
0.20
0.25
0.30
0.35
0.40
0.45
0.50
0.55
0.60
0.65
0.70
0.75
0.80
0.85
Share of the &place Educational Level (EL4)
RII
RII_LCL
RII_UCL

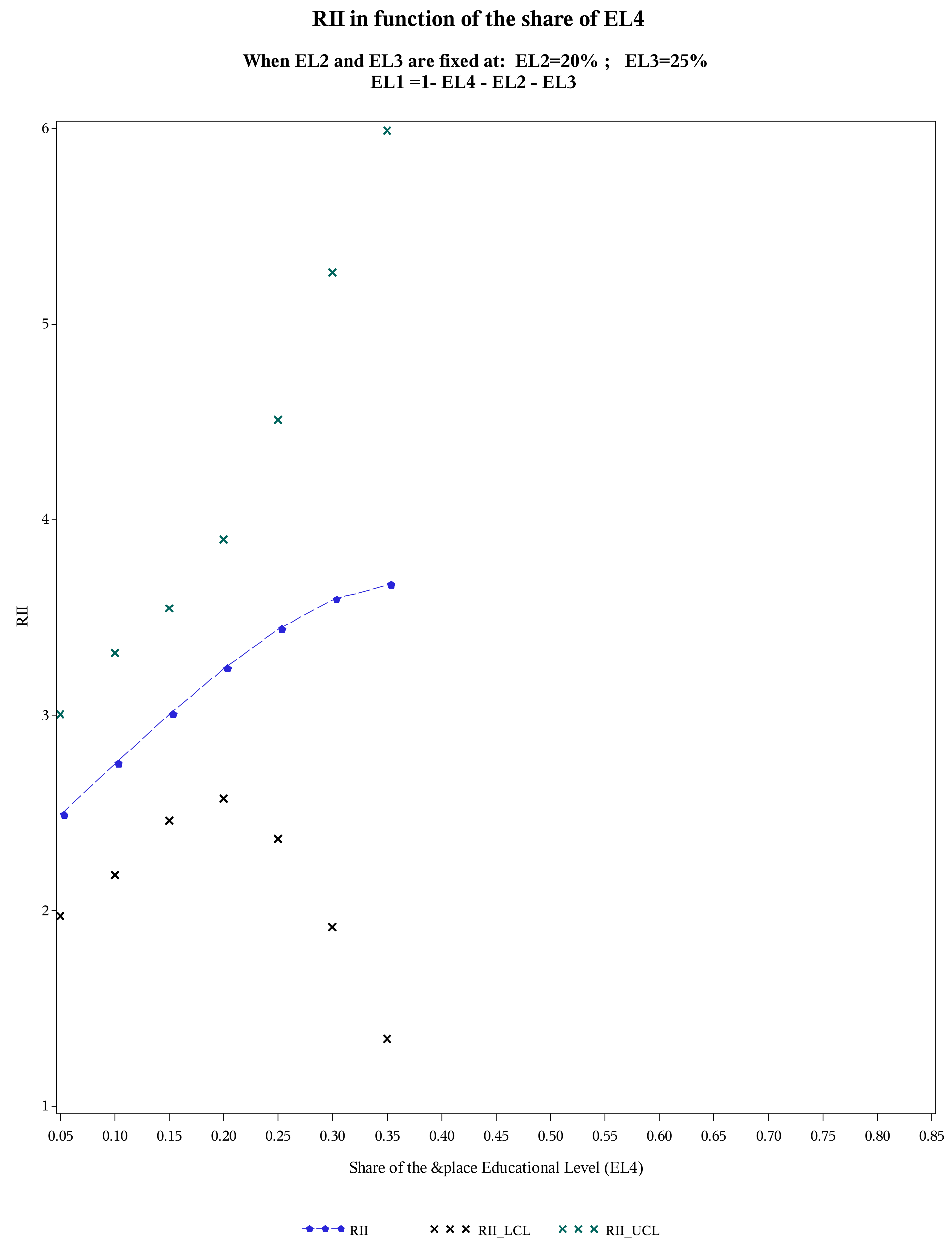

RII in function of the share of EL4
When EL2 and EL3 are fixed at: EL2=20% ; EL3=25%
EL1 =1- EL4 - EL2 - EL3
RII
1
2
3
4
5
6
0.05
0.10
0.15
0.20
0.25
0.30
0.35
0.40
0.45
0.50
0.55
0.60
0.65
0.70
0.75
0.80
0.85
Share of the &place Educational Level (EL4)
RII
RII_LCL
RII_UCL

RII in function of the share of EL4

When EL2 and EL3 are fixed at: EL2=20% ; EL3=30%
EL1 =1- EL4 - EL2 - EL3

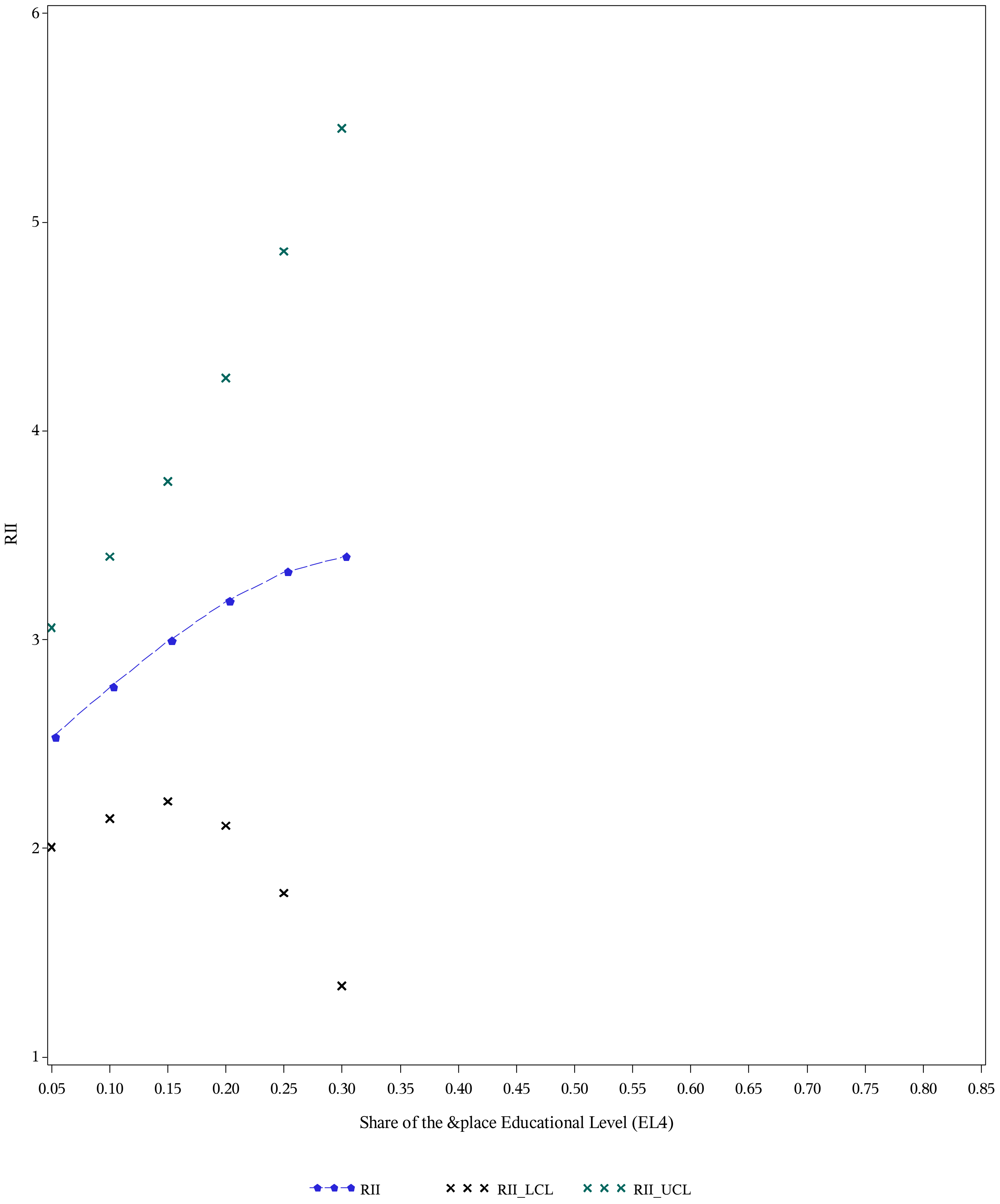

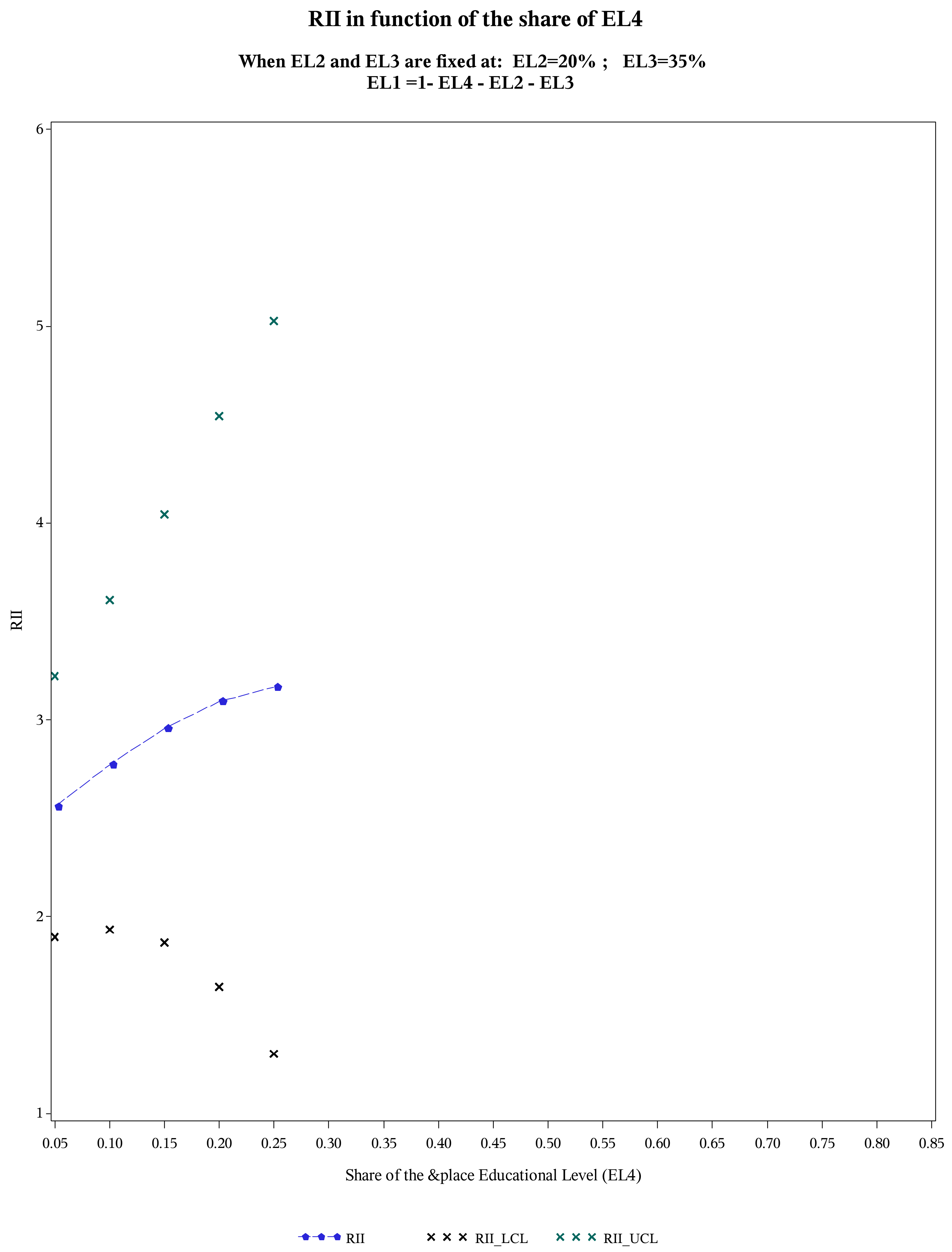
RII in function of the share of EL4
When EL2 and EL3 are fixed at: EL2=20% ; EL3=35%
EL1 =1- EL4 - EL2 - EL3
RII
Share of the &place Educational Level (EL4)
RII
RII_LCL
RII_UCL

RII in function of the share of EL4

When EL2 and EL3 are fixed at: EL2=20% ; EL3=40%
EL1 =1- EL4 - EL2 - EL3

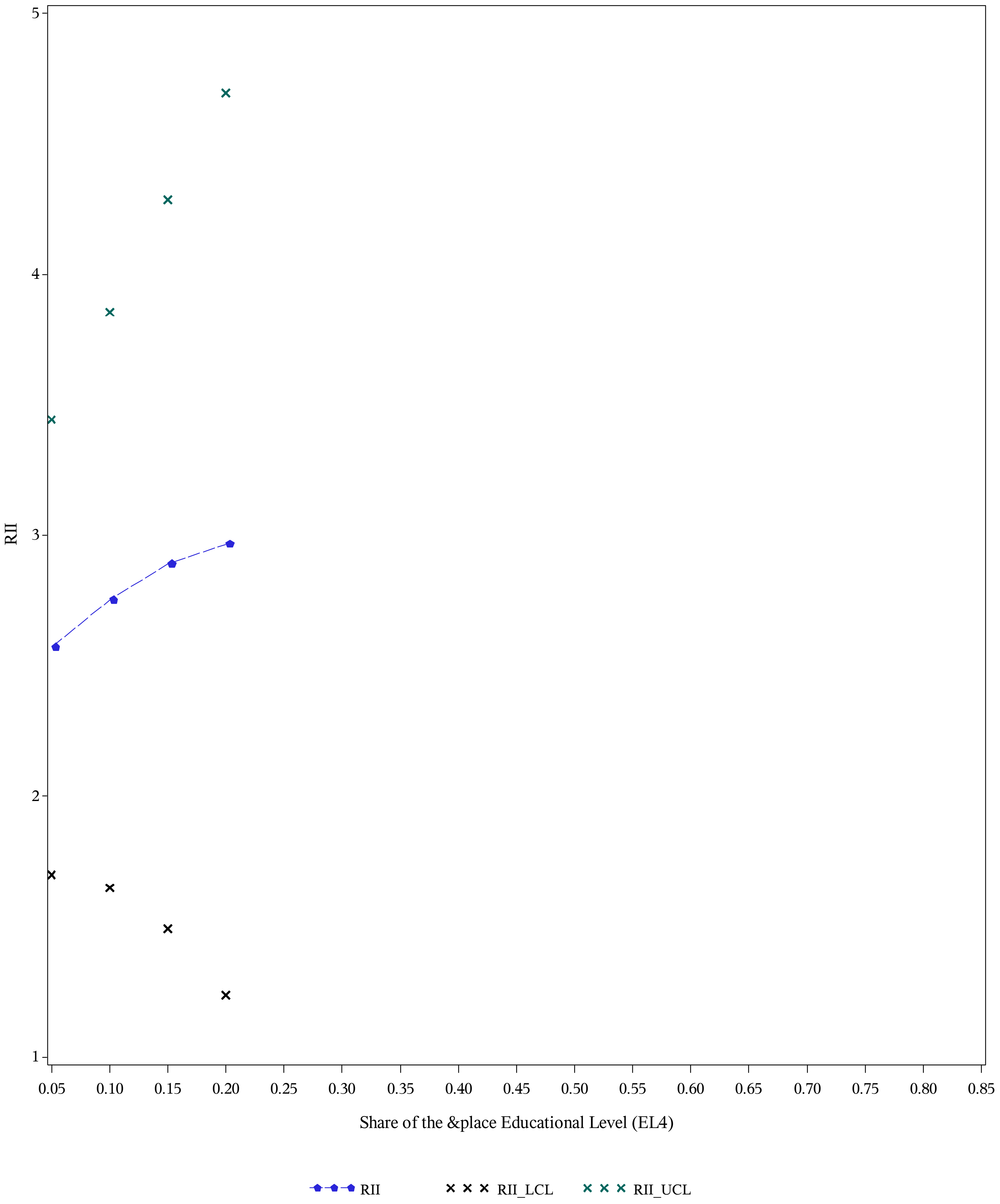

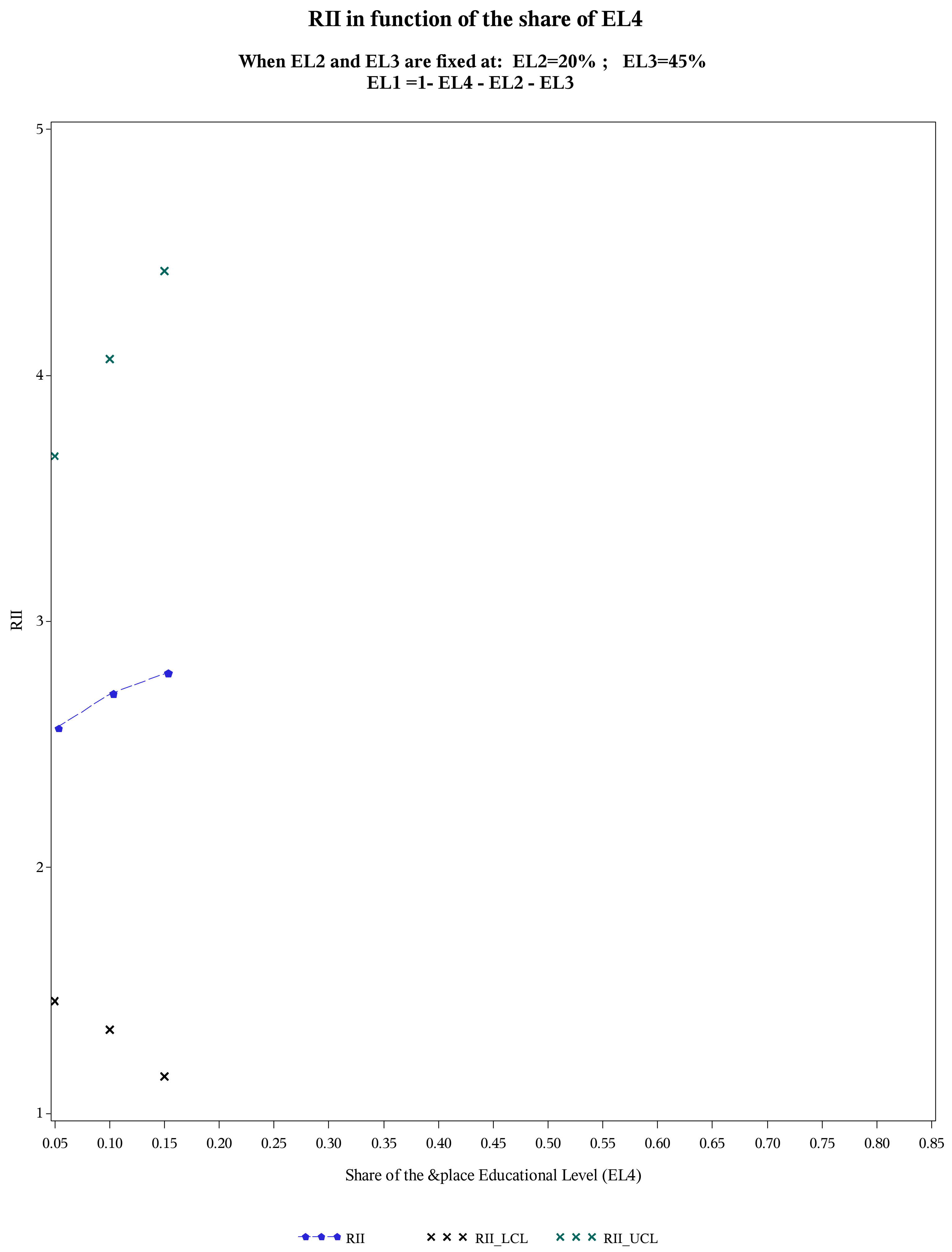
RII in function of the share of EL4
When EL2 and EL3 are fixed at: EL2=20% ; EL3=45%
EL1 =1- EL4 - EL2 - EL3
RII
5
4
3
2
1
0.05
0.10
0.15
0.20
0.25
0.30
0.35
0.40
0.45
0.50
0.55
0.60
0.65
0.70
0.75
0.80
0.85
Share of the &place Educational Level (EL4)
RII
RII_LCL
RII_UCL

RII in function of the share of EL4

When EL2 and EL3 are fixed at: EL2=20% ; EL3=50%
EL1 =1- EL4 - EL2 - EL3

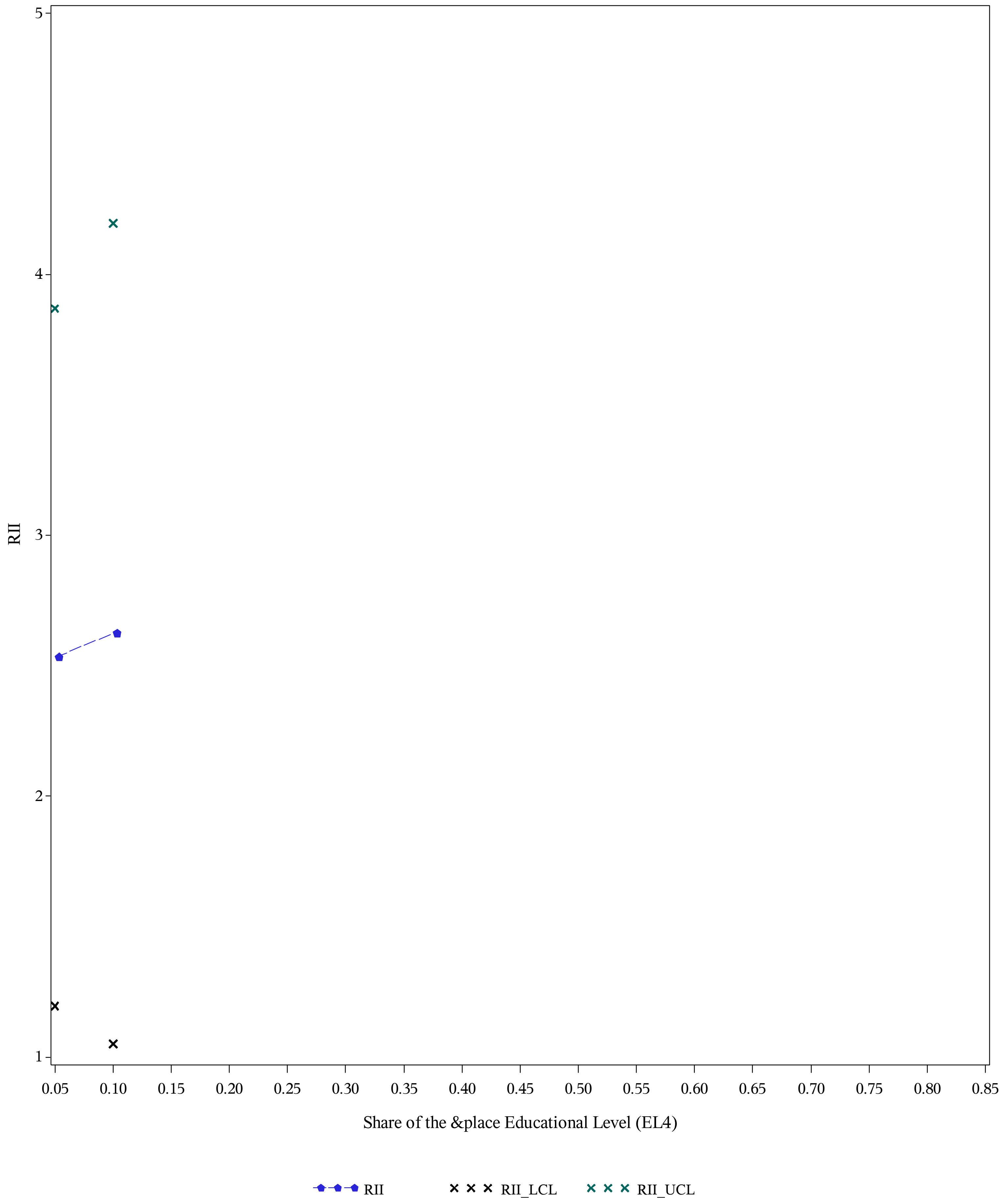

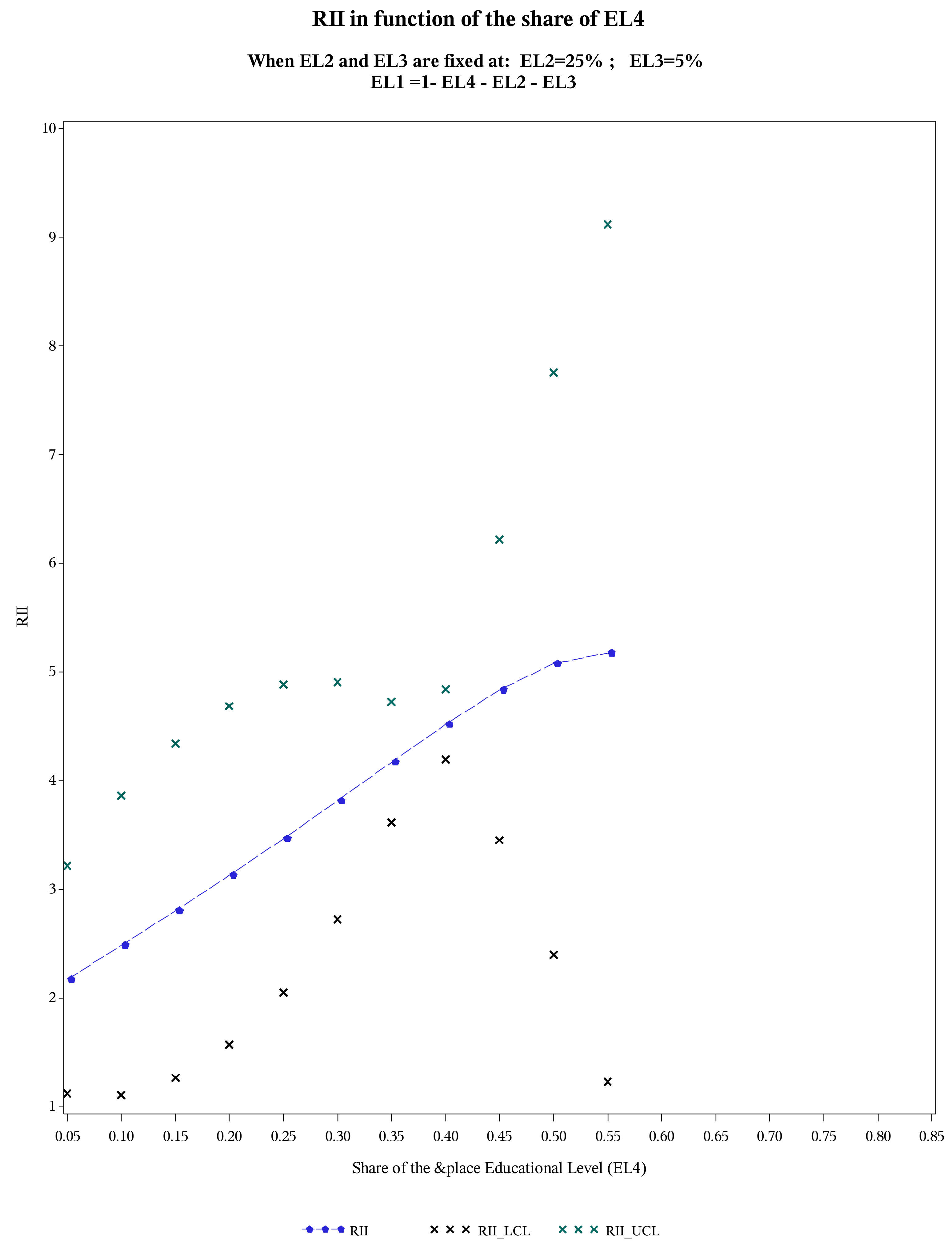
RII in function of the share of EL4
When EL2 and EL3 are fixed at: EL2=25% ; EL3=5%
EL1 =1- EL4 - EL2 - EL3
RII
1
2
3
4
5
6
7
8
9
10
0.05
0.10
0.15
0.20
0.25
0.30
0.35
0.40
0.45
0.50
0.55
0.60
0.65
0.70
0.75
0.80
0.85
Share of the &place Educational Level (EL4)
RII
RII_LCL
RII_UCL

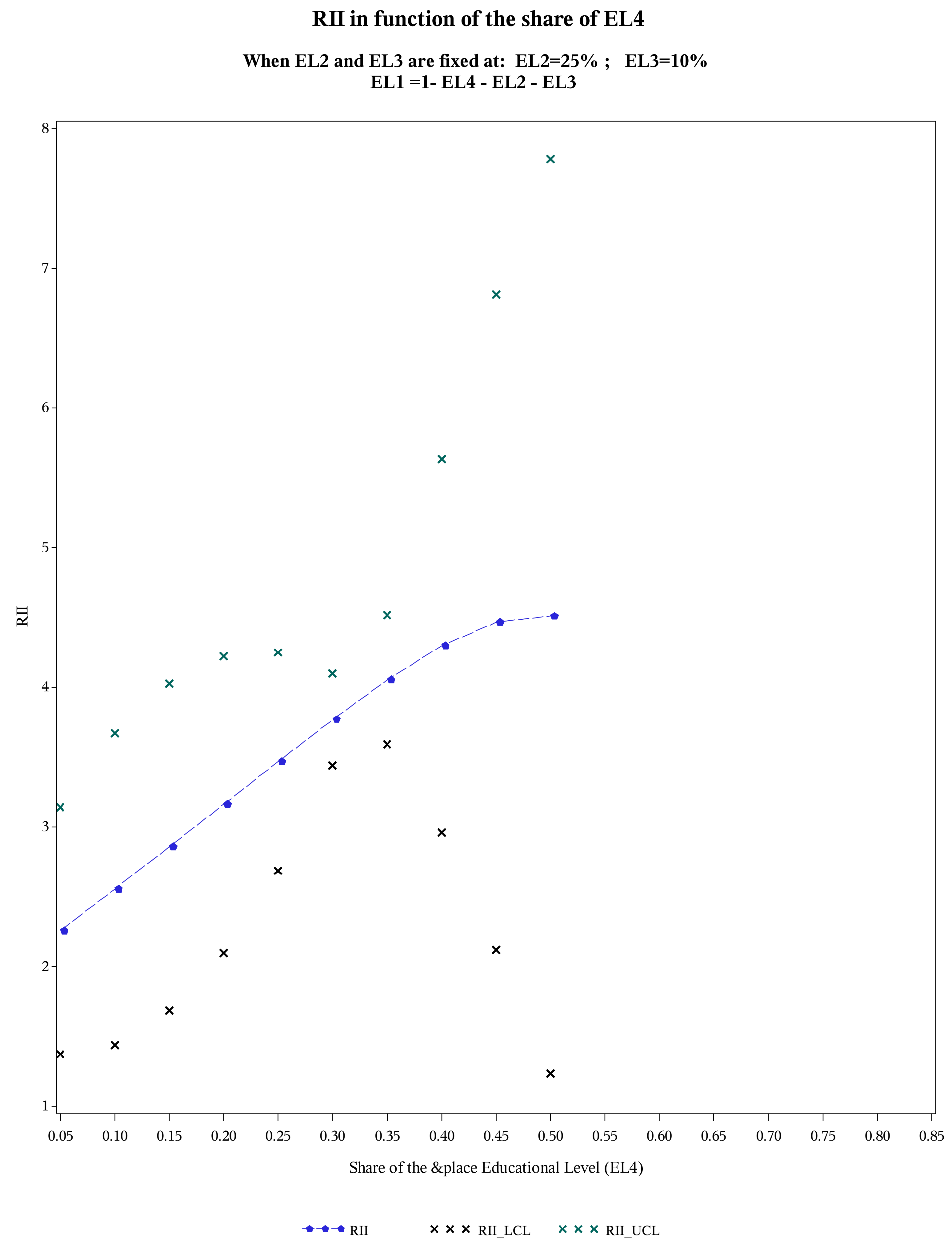

RII in function of the share of EL4
When EL2 and EL3 are fixed at: EL2=25% ; EL3=10%
EL1 =1- EL4 - EL2 - EL3
RII
1
2
3
4
5
6
7
8
0.05
0.10
0.15
0.20
0.25
0.30
0.35
0.40
0.45
0.50
0.55
0.60
0.65
0.70
0.75
0.80
0.85
Share of the &place Educational Level (EL4)
RII
RII_LCL
RII_UCL

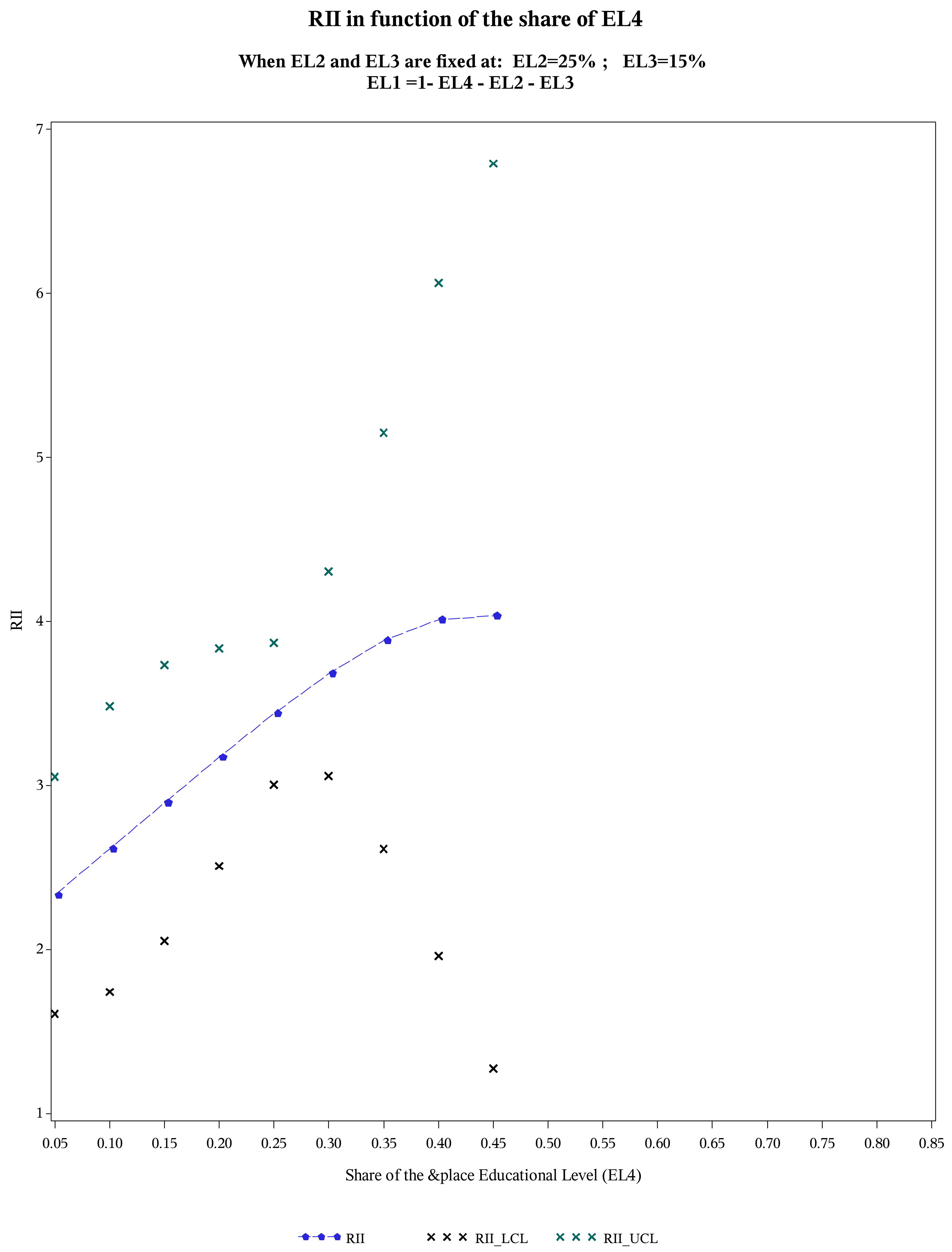
RII in function of the share of EL4
When EL2 and EL3 are fixed at: EL2=25% ; EL3=15%
EL1 =1- EL4 - EL2 - EL3
RII
Share of the &place Educational Level (EL4)
RII
RII_LCL
RII_UCL

RII in function of the share of EL4

When EL2 and EL3 are fixed at: EL2=25% ; EL3=20%
EL1 =1- EL4 - EL2 - EL3

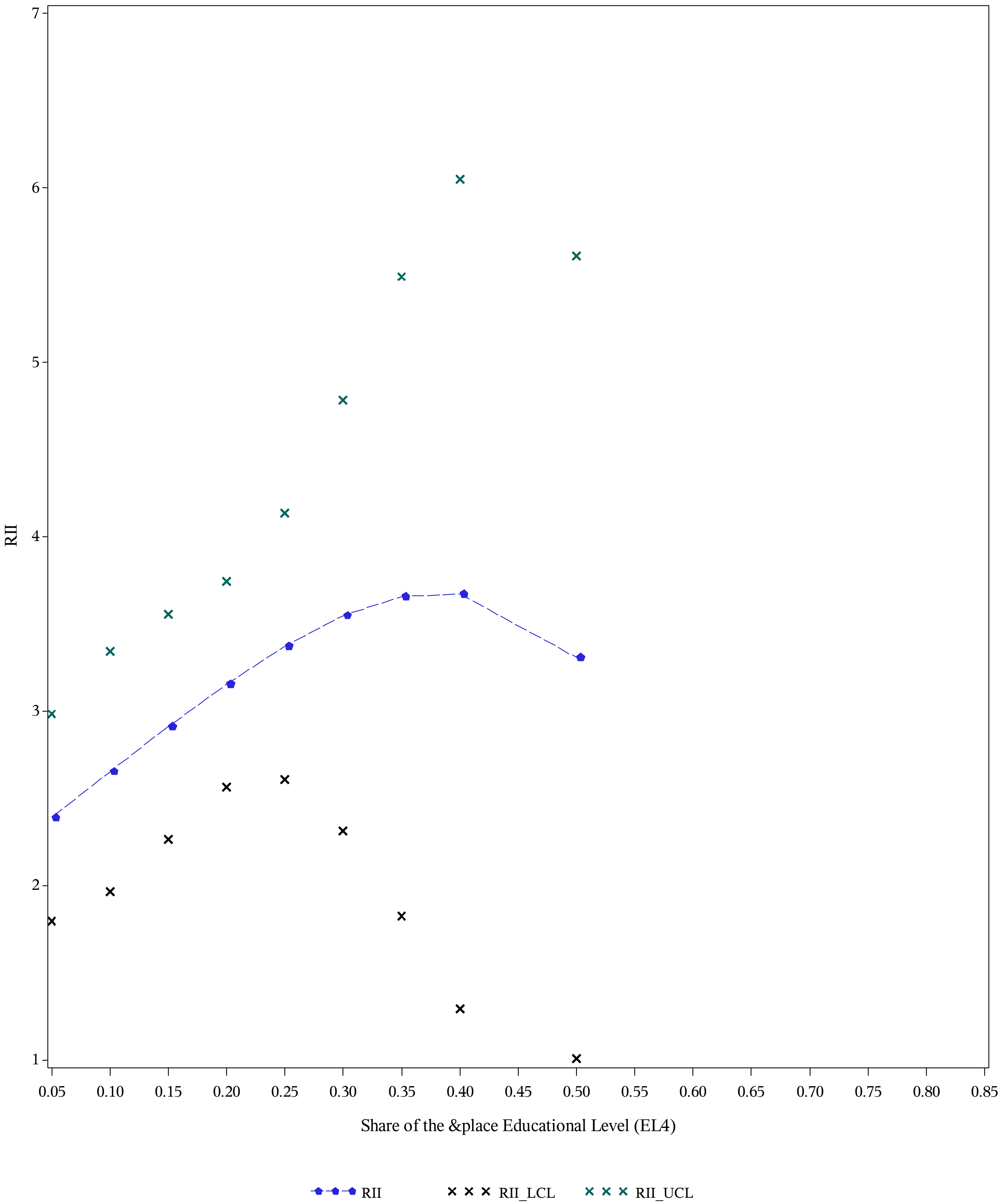

RII in function of the share of EL4

When EL2 and EL3 are fixed at: EL2=25% ; EL3=25%
EL1 =1- EL4 - EL2 - EL3

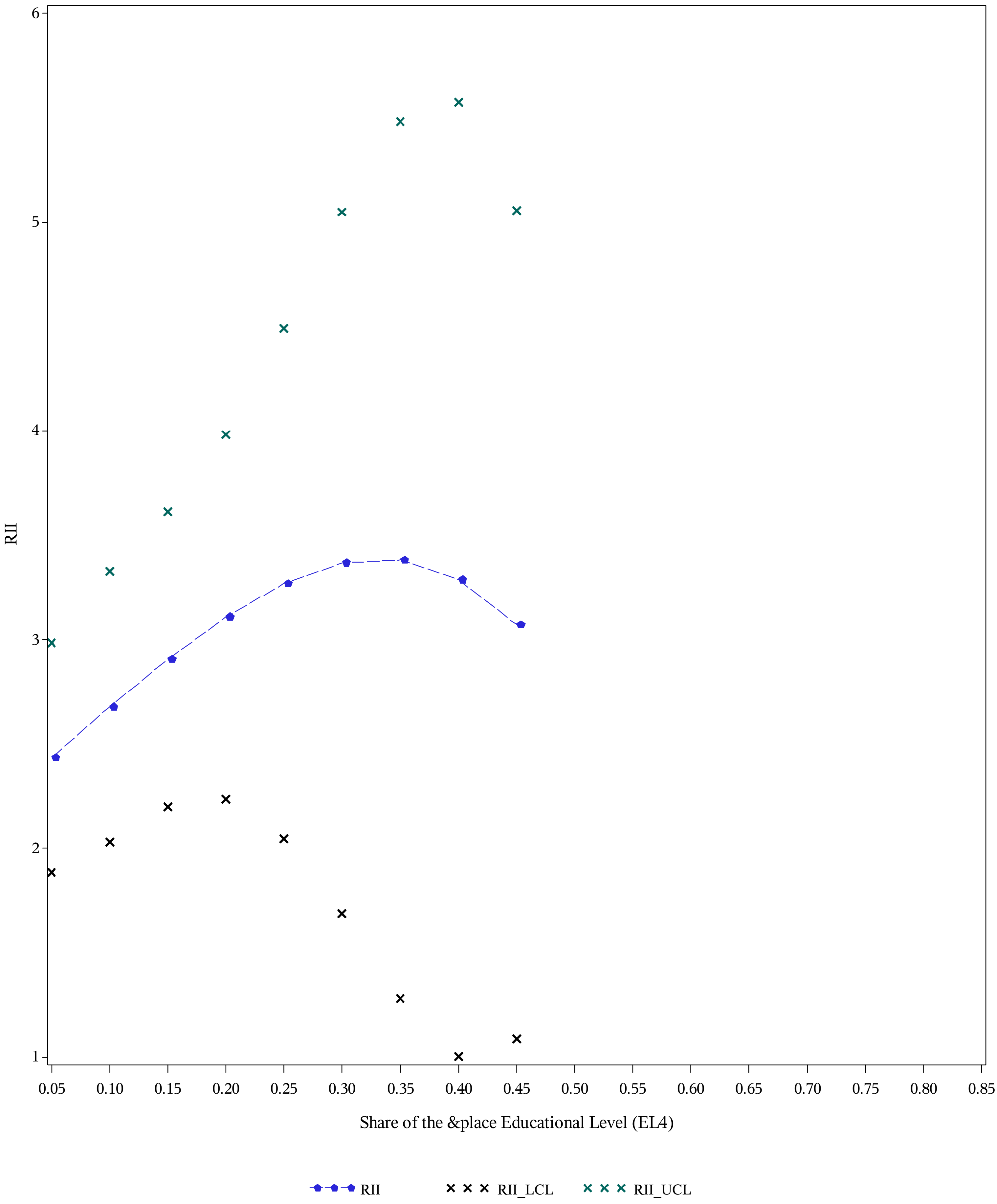

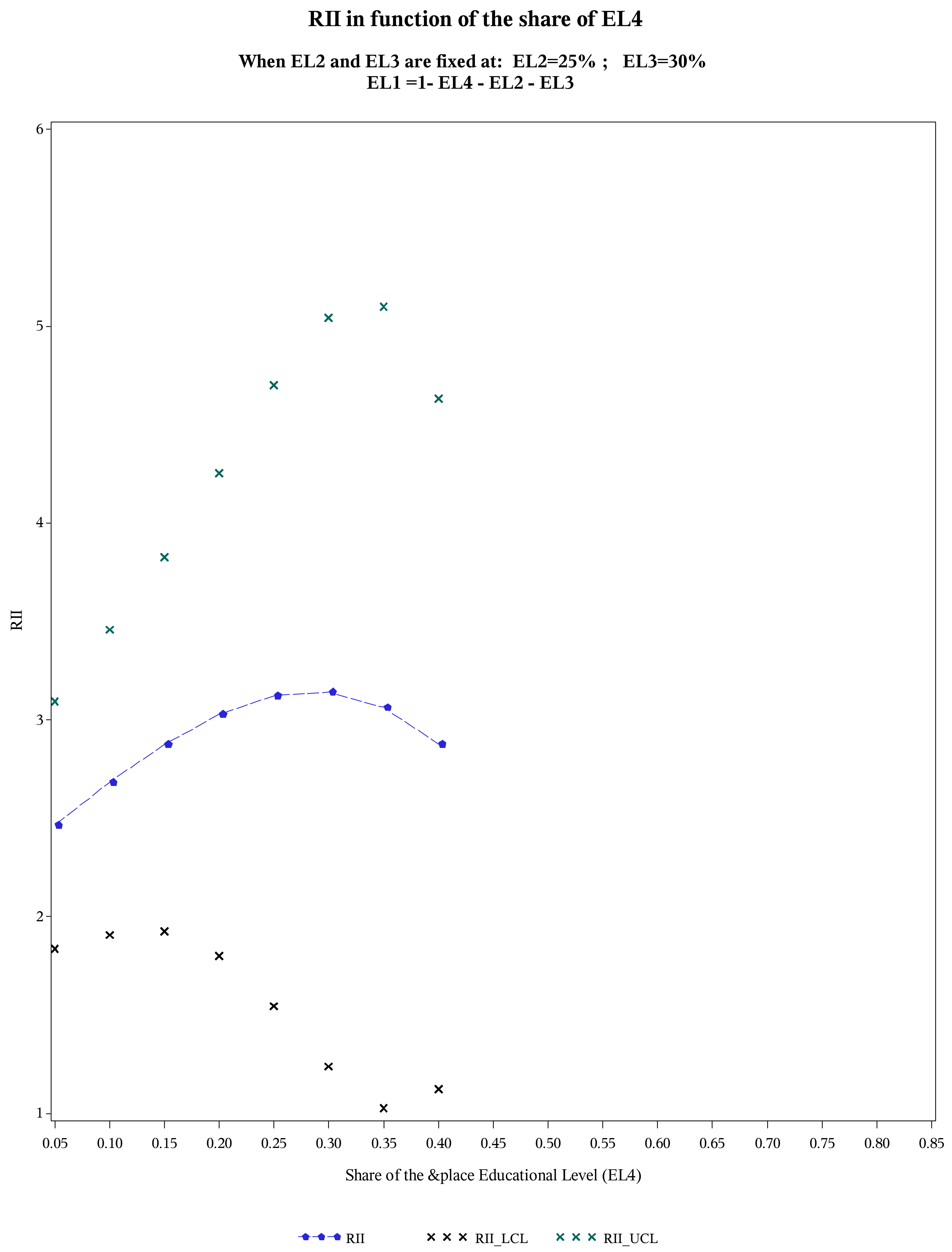

RII in function of the share of EL4
When EL2 and EL3 are fixed at: EL2=25% ; EL3=30%
EL1 =1- EL4 - EL2 - EL3
RII
1
2
3
4
5
6
0.05
0.10
0.15
0.20
0.25
0.30
0.35
0.40
0.45
0.50
0.55
0.60
0.65
0.70
0.75
0.80
0.85
Share of the &place Educational Level (EL4)
RII
RII_LCL
RII_UCL

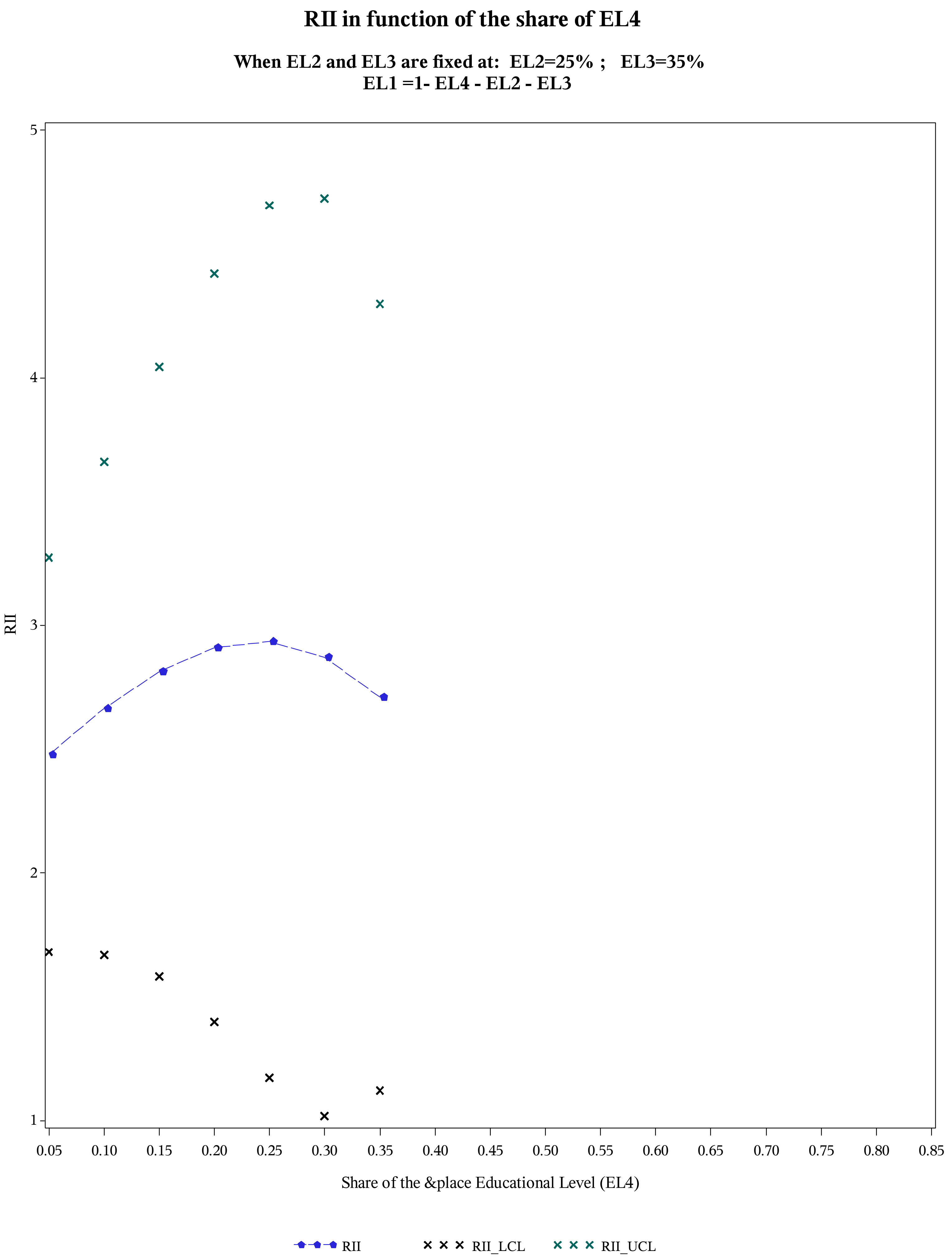

RII in function of the share of EL4
When EL2 and EL3 are fixed at: EL2=25% ; EL3=35%
EL1 =1- EL4 - EL2 - EL3
RII
1
2
3
4
5
0.05
0.10
0.15
0.20
0.25
0.30
0.35
0.40
0.45
0.50
0.55
0.60
0.65
0.70
0.75
0.80
0.85
Share of the &place Educational Level (EL4)
RII
RII_LCL
RII_UCL

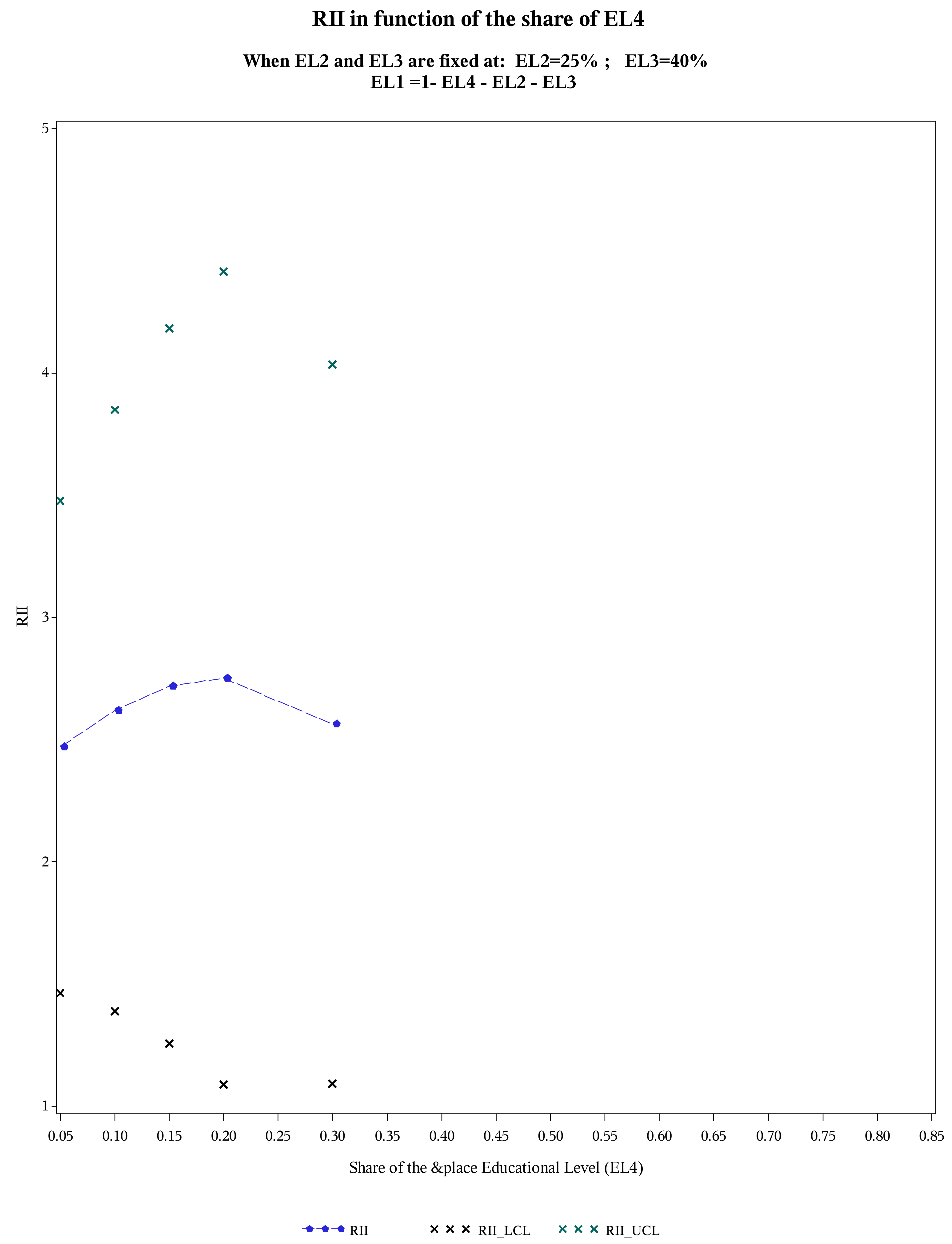
RII in function of the share of EL4
When EL2 and EL3 are fixed at: EL2=25% ; EL3=40%
EL1 =1- EL4 - EL2 - EL3
RII
1
2
3
4
5
0.05
0.10
0.15
0.20
0.25
0.30
0.35
0.40
0.45
0.50
0.55
0.60
0.65
0.70
0.75
0.80
0.85
Share of the &place Educational Level (EL4)
RII
RII_LCL
RII_UCL

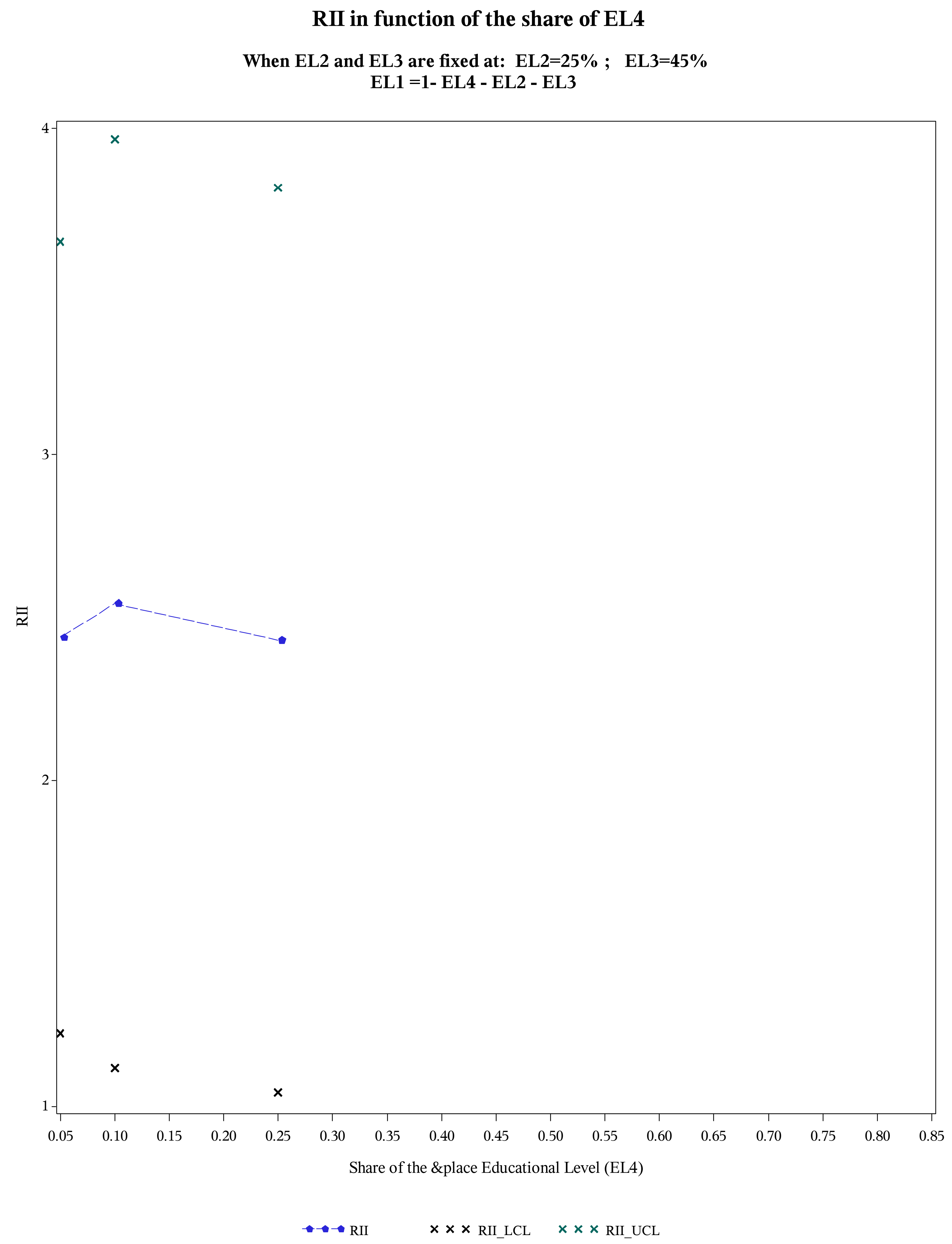
RII in function of the share of EL4
When EL2 and EL3 are fixed at: EL2=25% ; EL3=45%
EL1 =1- EL4 - EL2 - EL3
RII
4
3
2
1
0.05
0.10
0.15
0.20
0.25
0.30
0.35
0.40
0.45
0.50
0.55
0.60
0.65
0.70
0.75
0.80
0.85
Share of the &place Educational Level (EL4)
RII
RII_LCL
RII_UCL

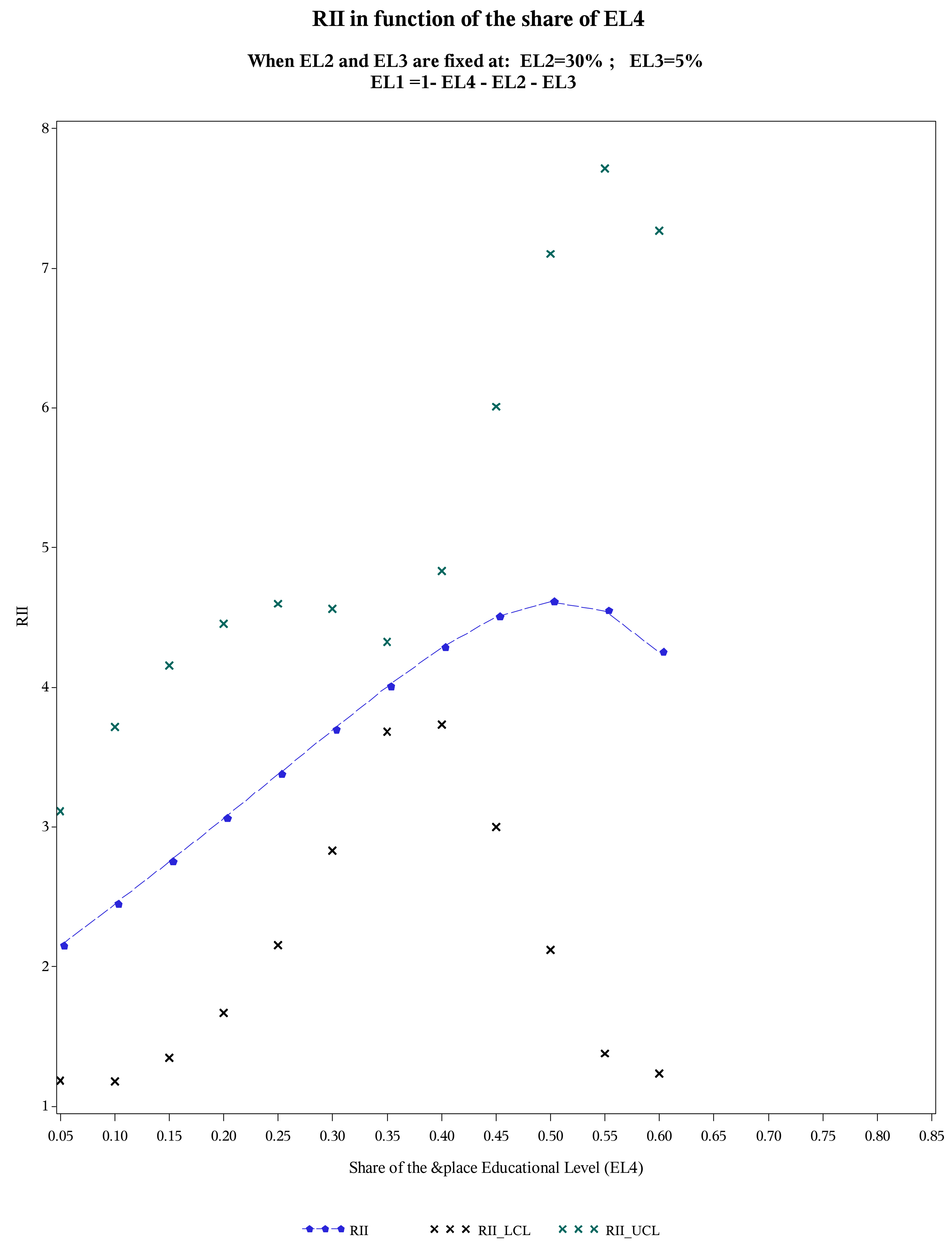
RII in function of the share of EL4
When EL2 and EL3 are fixed at: EL2=30% ; EL3=5%
EL1 =1- EL4 - EL2 - EL3
RII
Share of the &place Educational Level (EL4)
RII
RII_LCL
RII_UCL

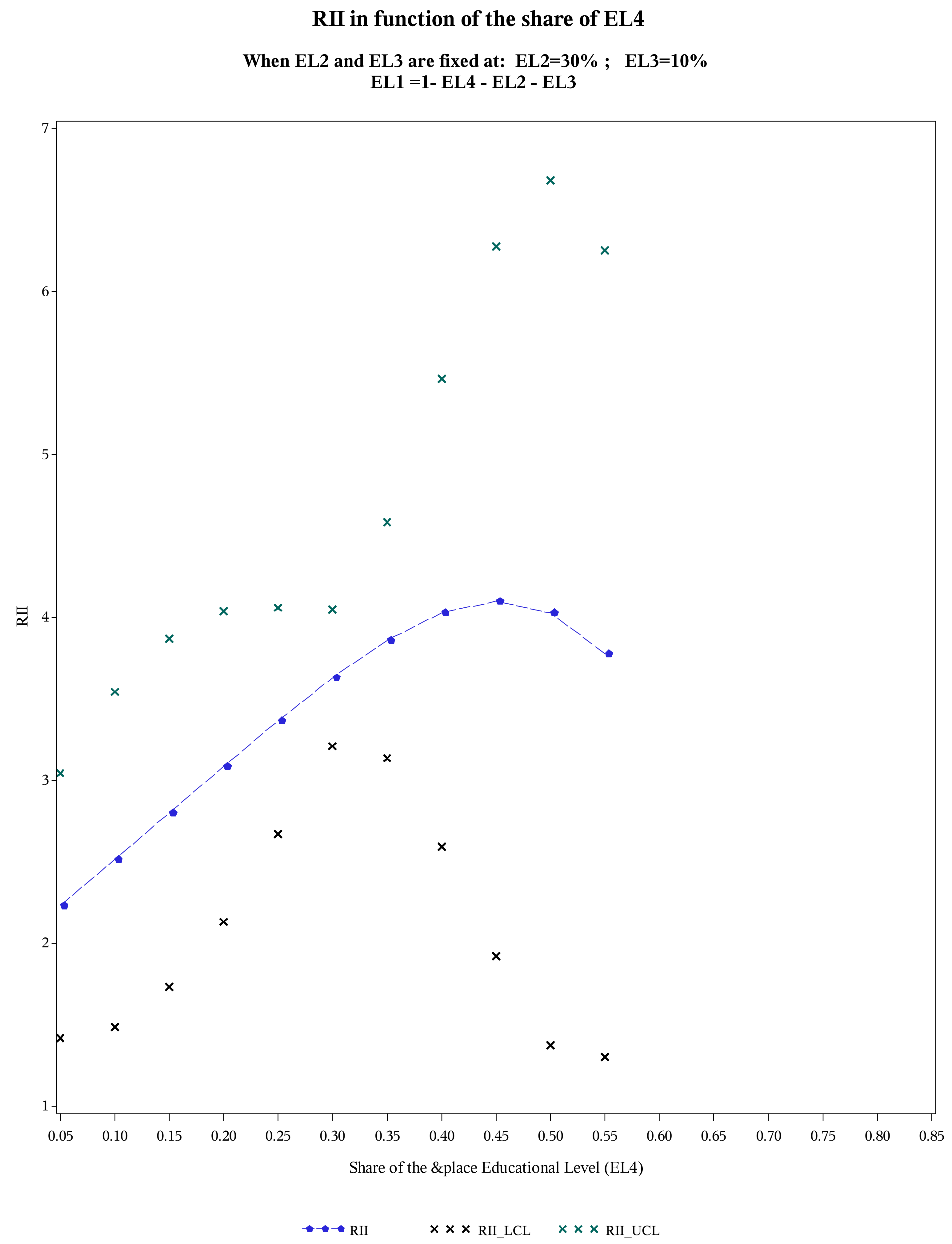
RII in function of the share of EL4
When EL2 and EL3 are fixed at: EL2=30% ; EL3=10%
EL1 =1- EL4 - EL2 - EL3
RII
1
2
3
4
5
6
7
0.05
0.10
0.15
0.20
0.25
0.30
0.35
0.40
0.45
0.50
0.55
0.60
0.65
0.70
0.75
0.80
0.85
Share of the &place Educational Level (EL4)
RII
RII_LCL
RII_UCL

RII in function of the share of EL4

When EL2 and EL3 are fixed at: EL2=30% ; EL3=15%
EL1 =1- EL4 - EL2 - EL3

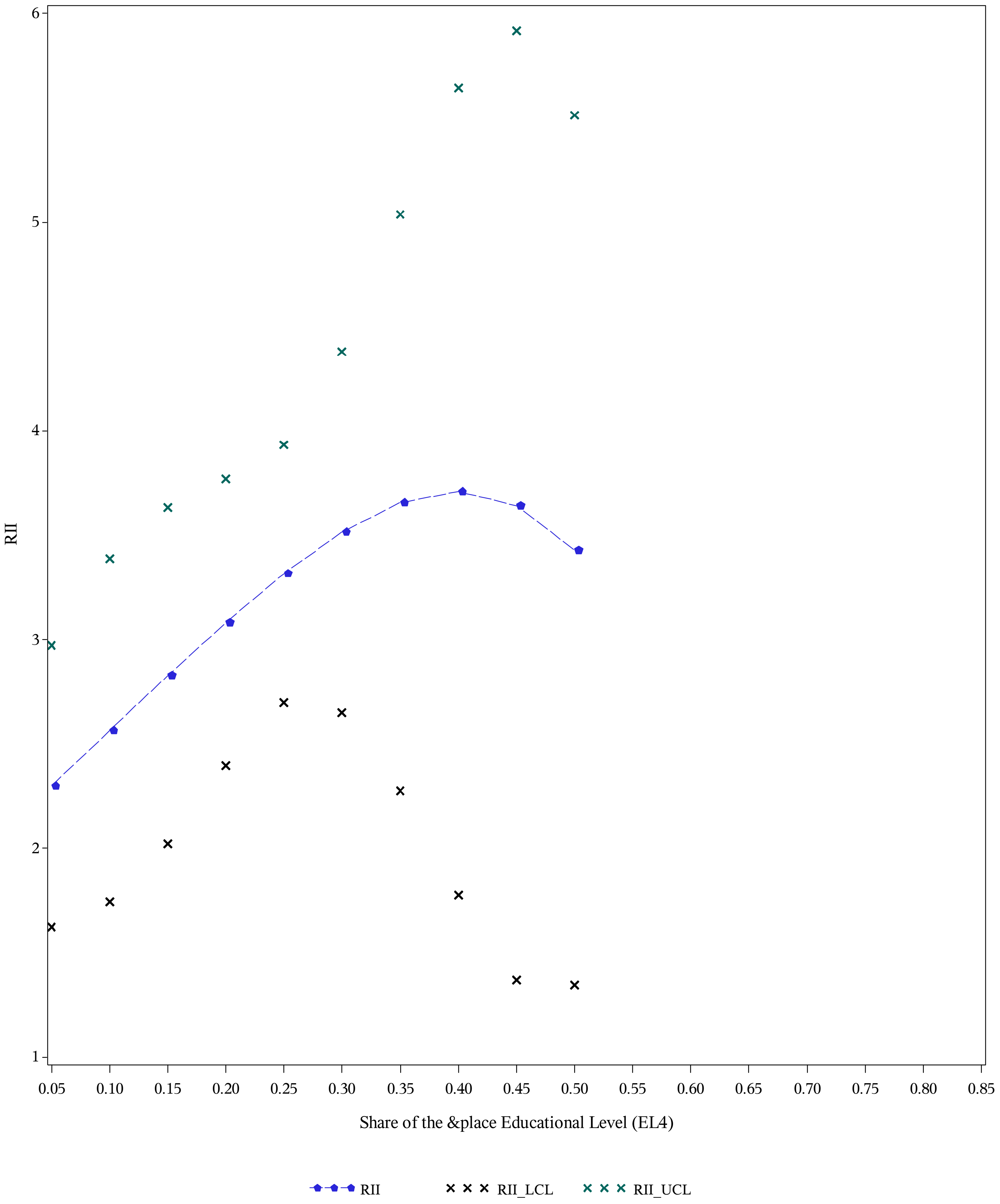

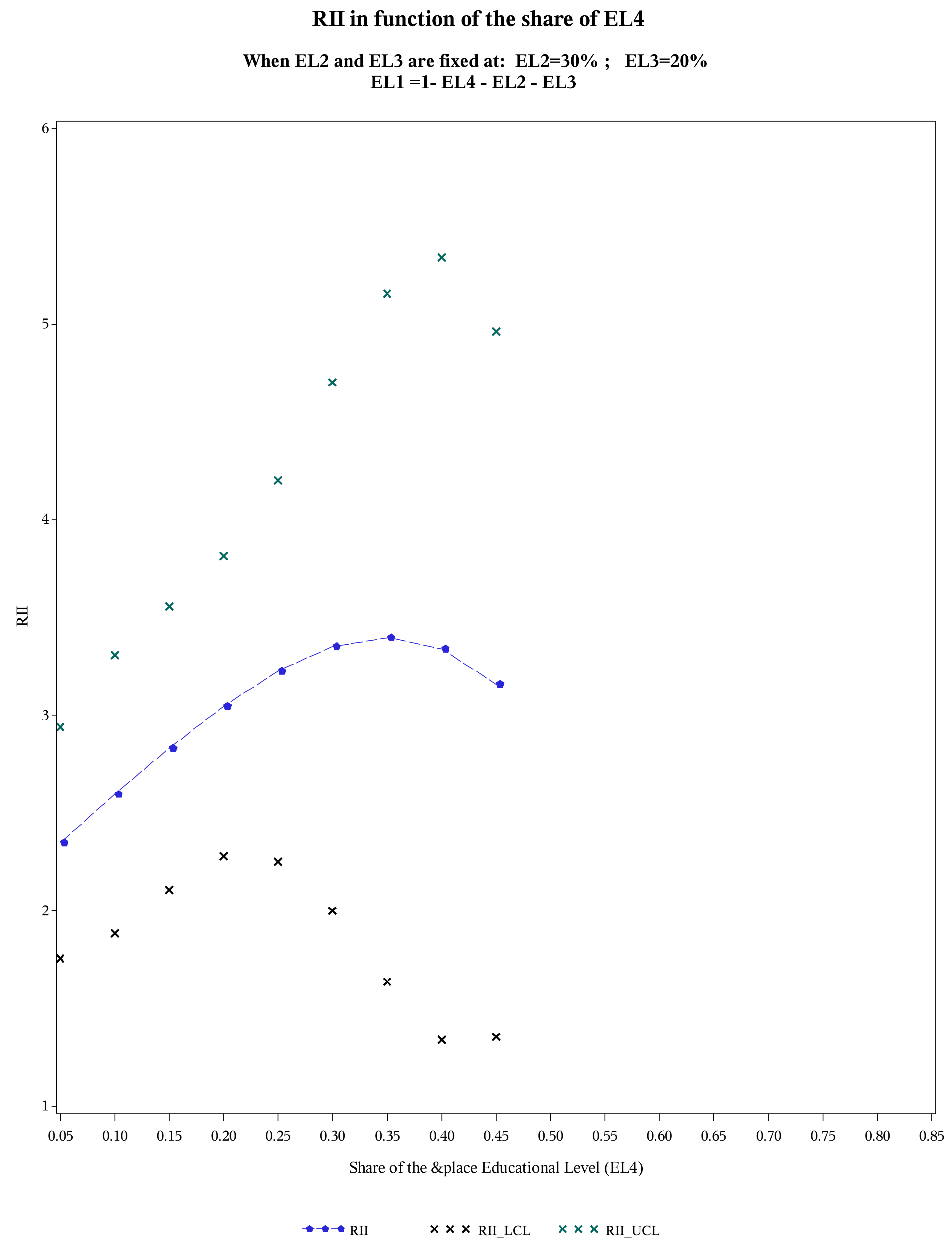

RII in function of the share of EL4
When EL2 and EL3 are fixed at: EL2=30% ; EL3=20%
EL1 =1- EL4 - EL2 - EL3
RII
6
5
4
3
2
1
0.05
0.10
0.15
0.20
0.25
0.30
0.35
0.40
0.45
0.50
0.55
0.60
0.65
0.70
0.75
0.80
0.85
Share of the &place Educational Level (EL4)
RII
RII_LCL
RII_UCL

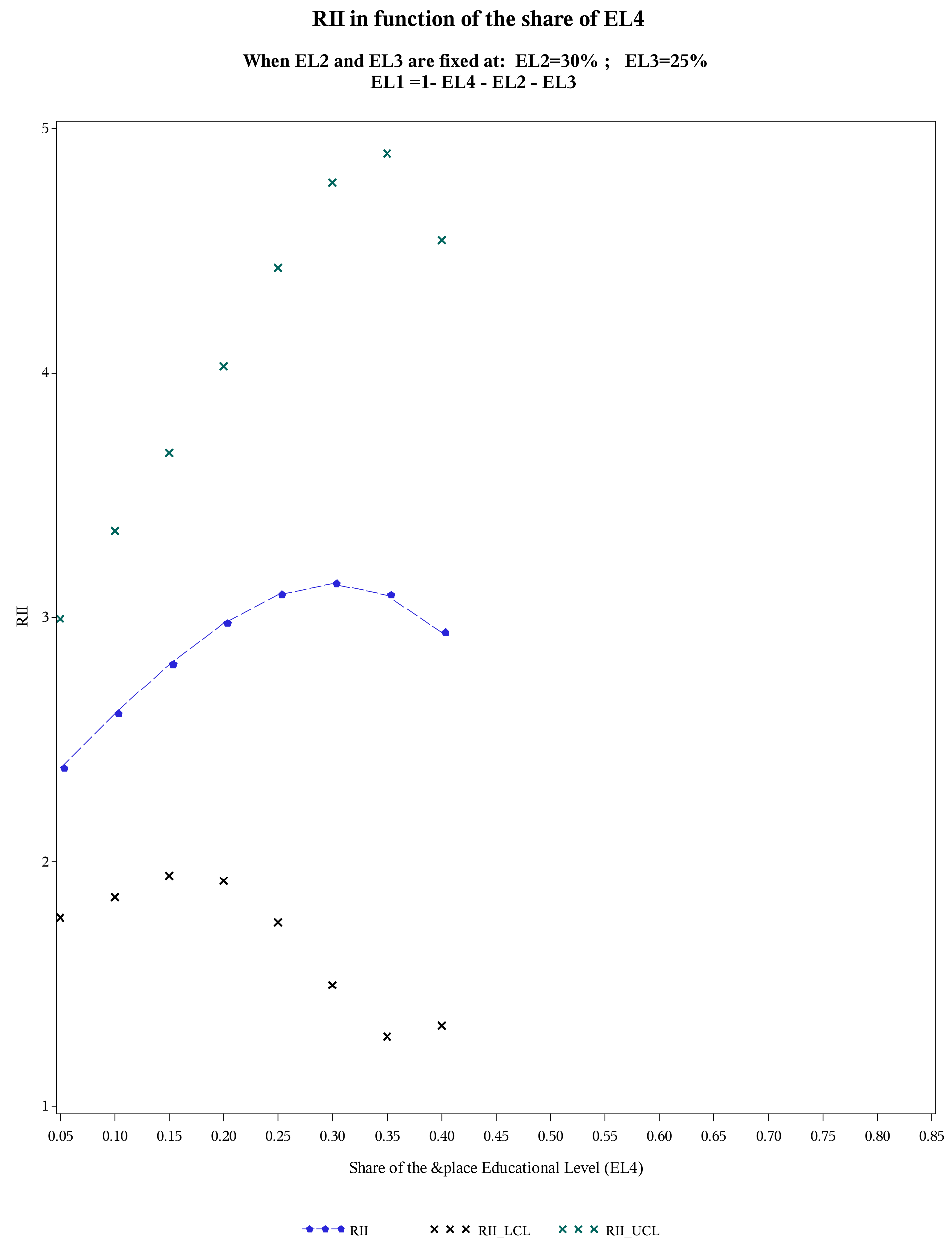
RII in function of the share of EL4
When EL2 and EL3 are fixed at: EL2=30% ; EL3=25%
EL1 =1- EL4 - EL2 - EL3
RII
1
2
3
4
5
0.05
0.10
0.15
0.20
0.25
0.30
0.35
0.40
0.45
0.50
0.55
0.60
0.65
0.70
0.75
0.80
0.85
Share of the &place Educational Level (EL4)
RII
RII_LCL
RII_UCL

RII in function of the share of EL4

When EL2 and EL3 are fixed at: EL2=30% ; EL3=30%
EL1 =1- EL4 - EL2 - EL3

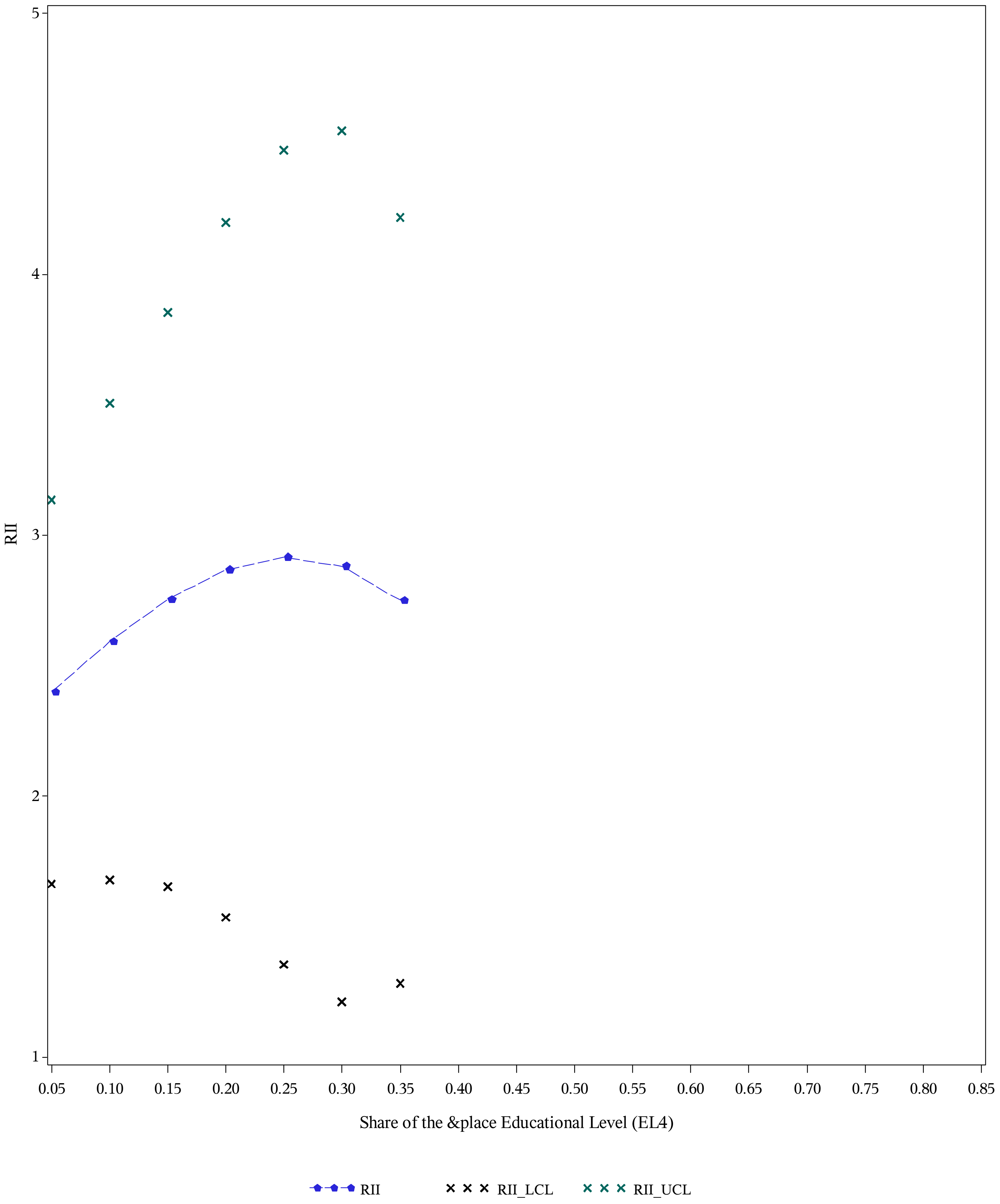

RII in function of the share of EL4

When EL2 and EL3 are fixed at: EL2=30% ; EL3=35%
EL1 =1- EL4 - EL2 - EL3

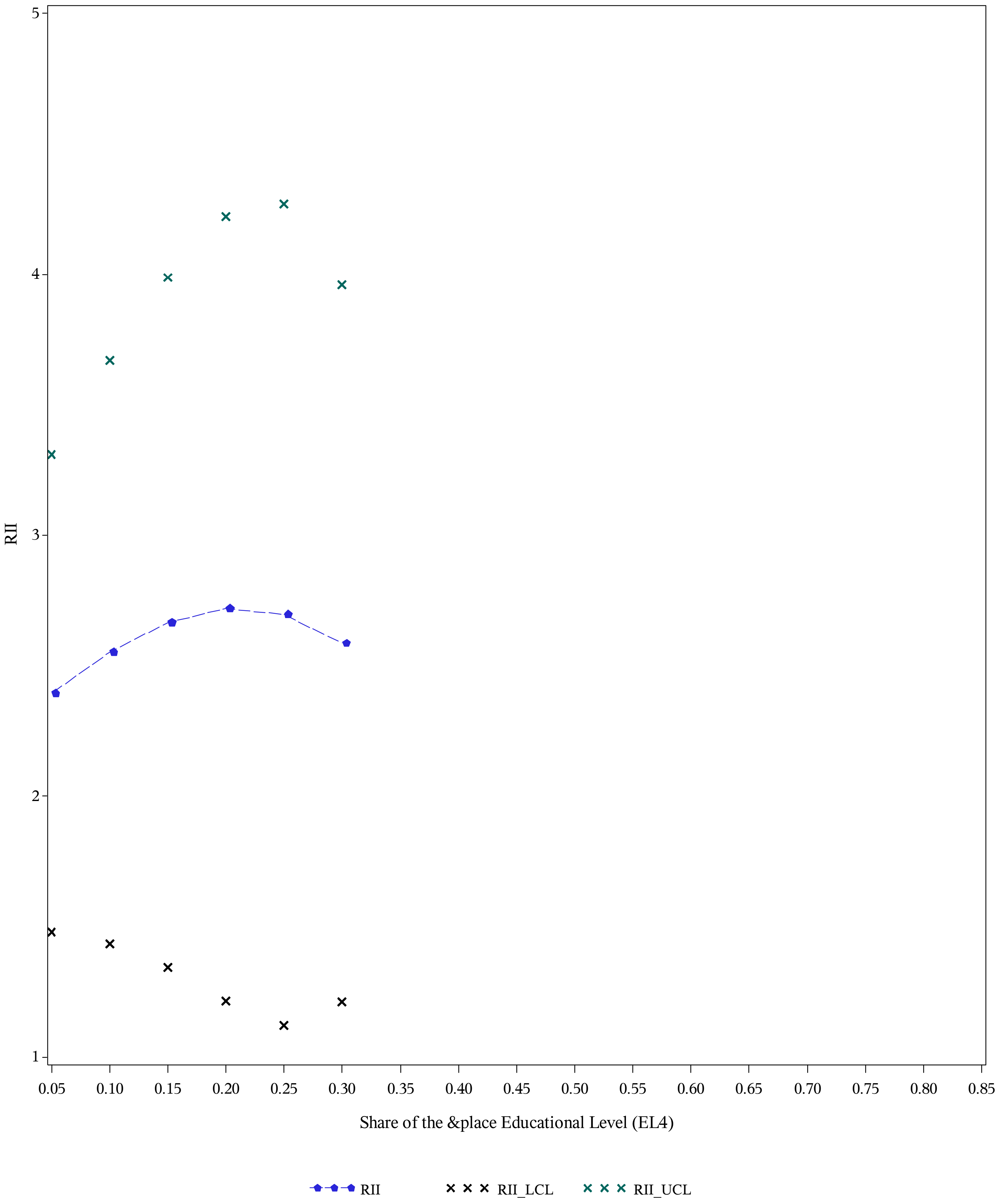

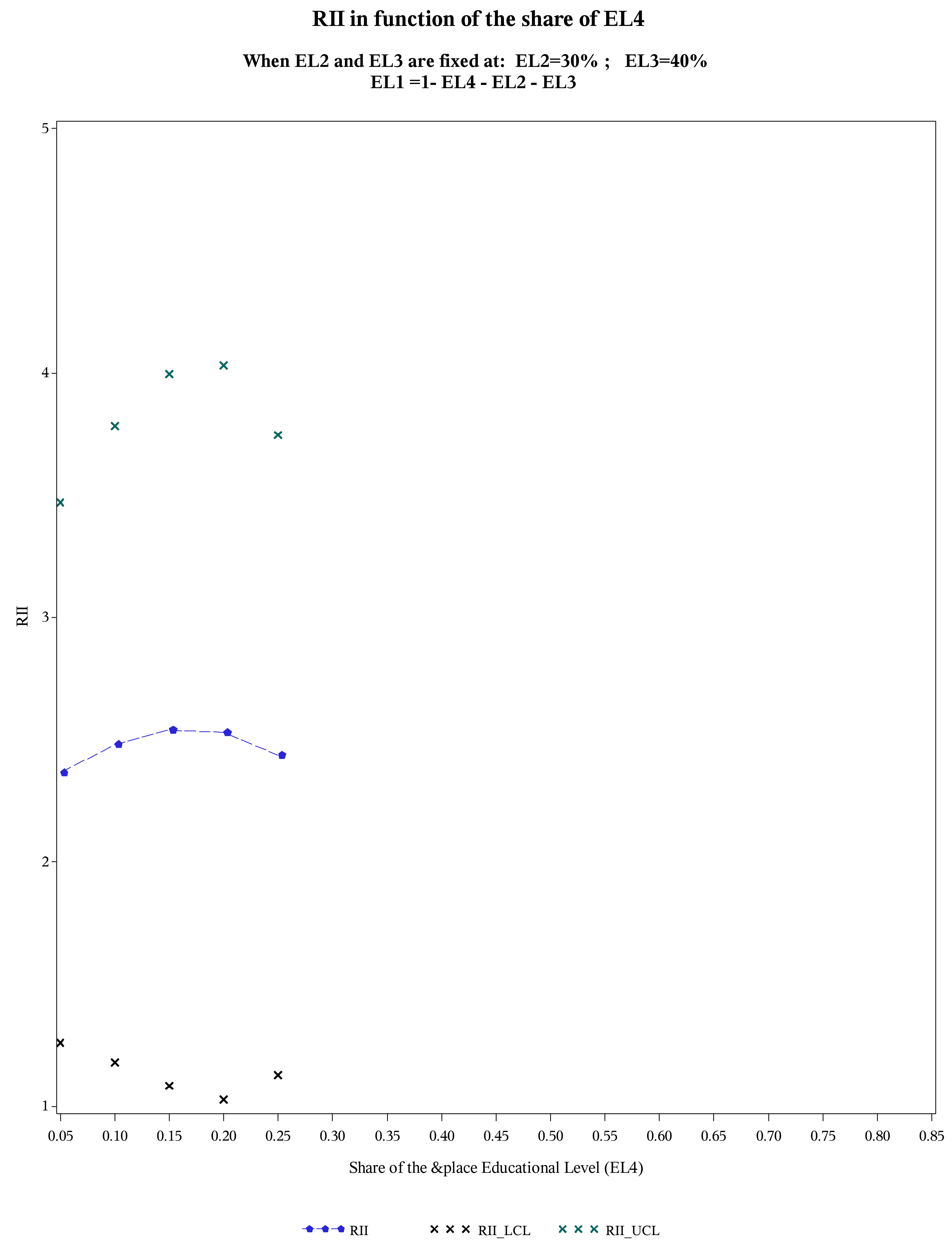
RII in function of the share of EL4
When EL2 and EL3 are fixed at: EL2=30% ; EL3=40%
EL1 =1- EL4 - EL2 - EL3
RII
5
4
3
2
1
0.05
0.10
0.15
0.20
0.25
0.30
0.35
0.40
0.45
0.50
0.55
0.60
0.65
0.70
0.75
0.80
0.85
Share of the &place Educational Level (EL4)
RII
RII_LCL
RII_UCL

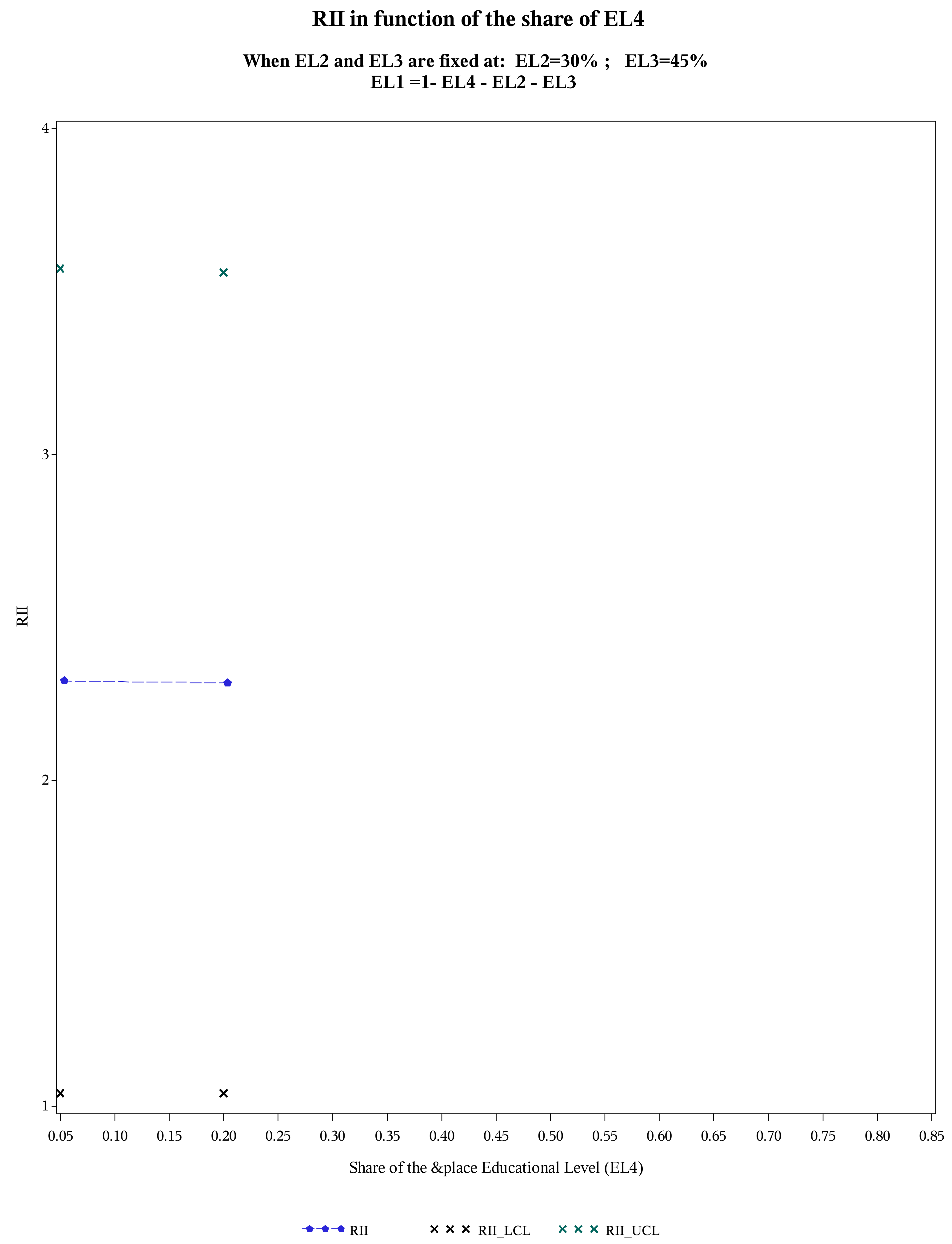
RII in function of the share of EL4
When EL2 and EL3 are fixed at: EL2=30% ; EL3=45%
EL1 =1- EL4 - EL2 - EL3
RII
1
2
3
4
0.05
0.10
0.15
0.20
0.25
0.30
0.35
0.40
0.45
0.50
0.55
0.60
0.65
0.70
0.75
0.80
0.85
Share of the &place Educational Level (EL4)
RII
RII_LCL
RII_UCL

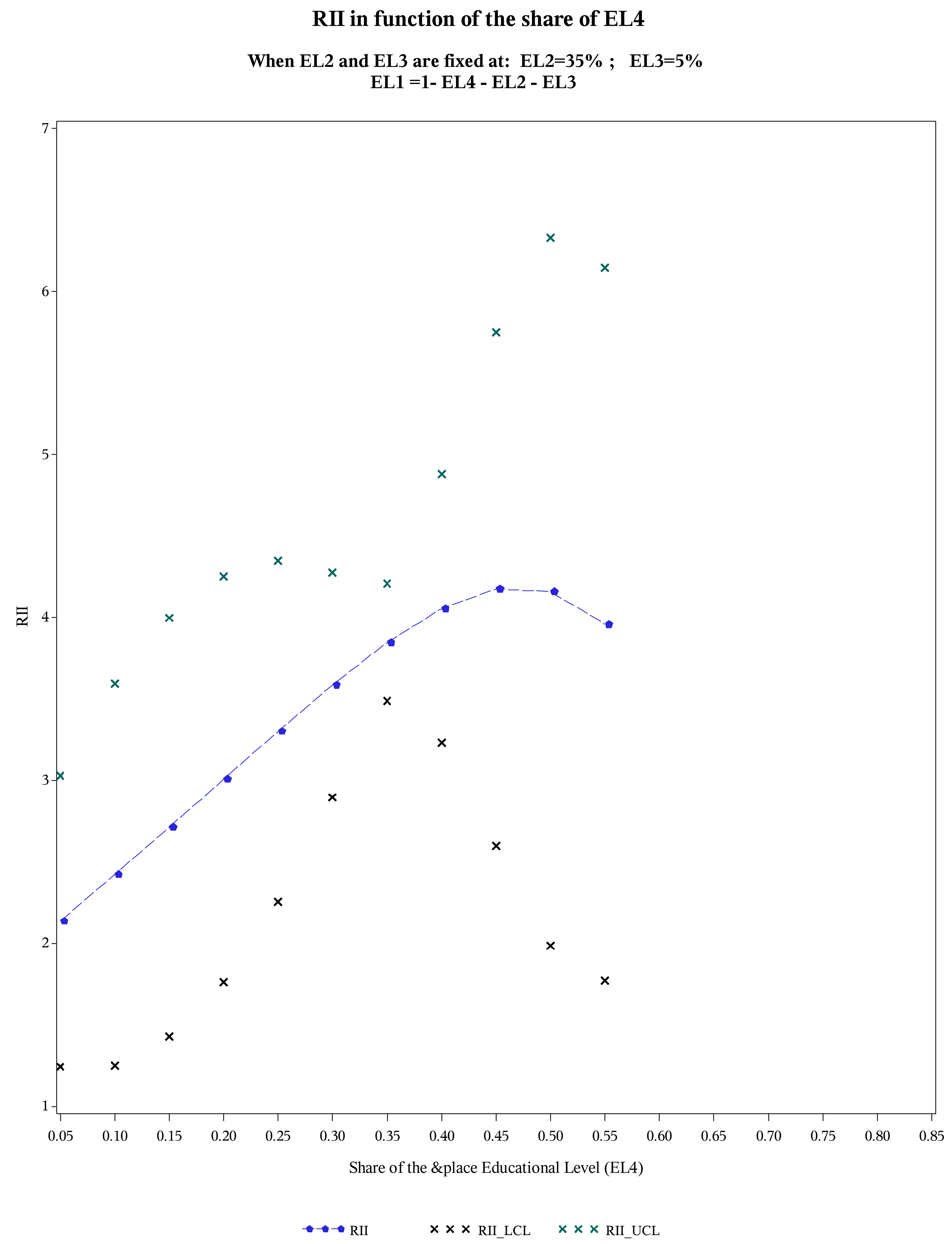
RII in function of the share of EL4
When EL2 and EL3 are fixed at: EL2=35% ; EL3=5%
EL1 =1- EL4 - EL2 - EL3
RII
1
2
3
4
5
6
7
0.05
0.10
0.15
0.20
0.25
0.30
0.35
0.40
0.45
0.50
0.55
0.60
0.65
0.70
0.75
0.80
0.85
Share of the &place Educational Level (EL4)
RII
RII_LCL
RII_UCL

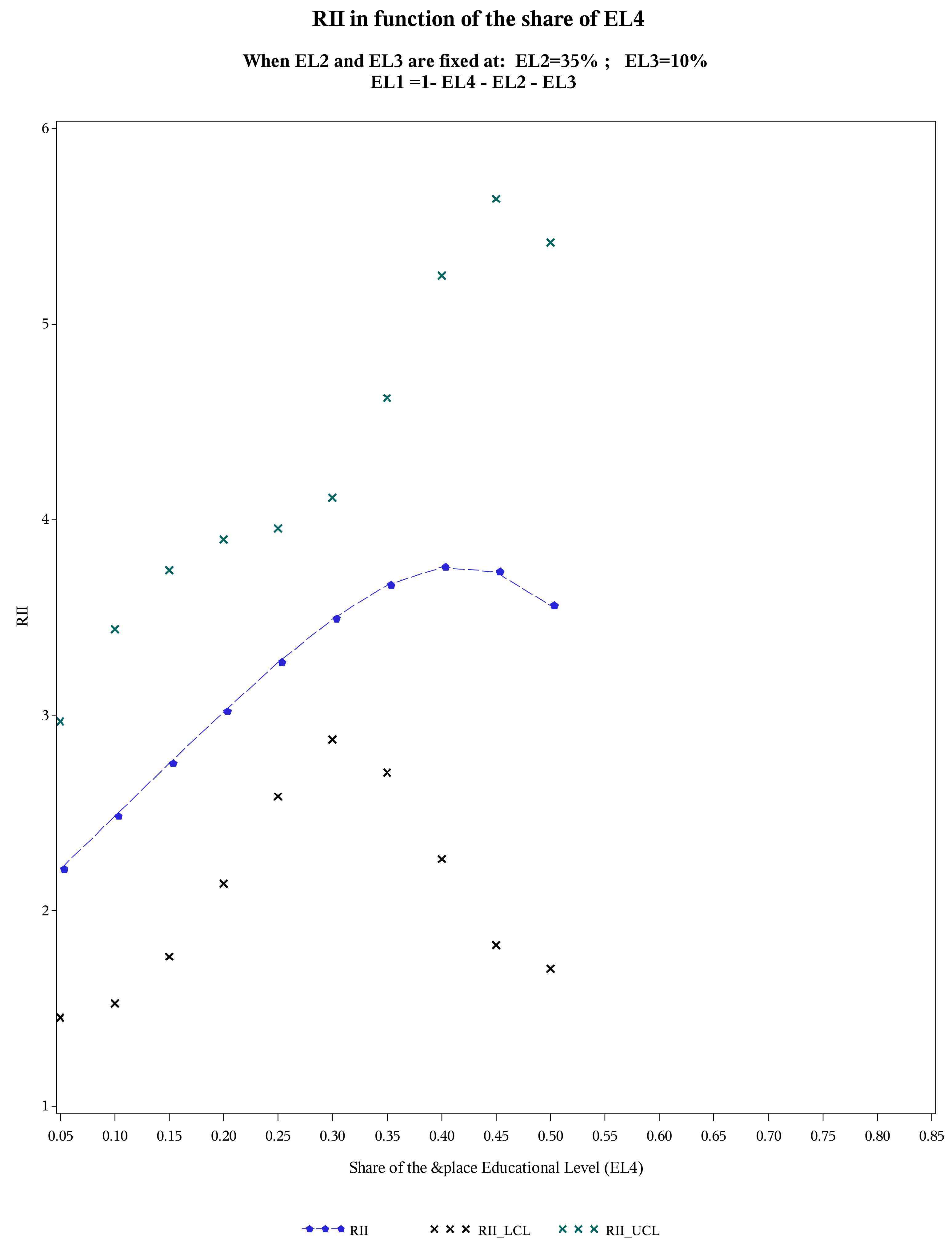
RII in function of the share of EL4
When EL2 and EL3 are fixed at: EL2=35% ; EL3=10%
EL1 =1- EL4 - EL2 - EL3
RII
6
5
4
3
2
1
0.05
0.10
0.15
0.20
0.25
0.30
0.35
0.40
0.45
0.50
0.55
0.60
0.65
0.70
0.75
0.80
0.85
Share of the &place Educational Level (EL4)
RII
RII_LCL
RII_UCL

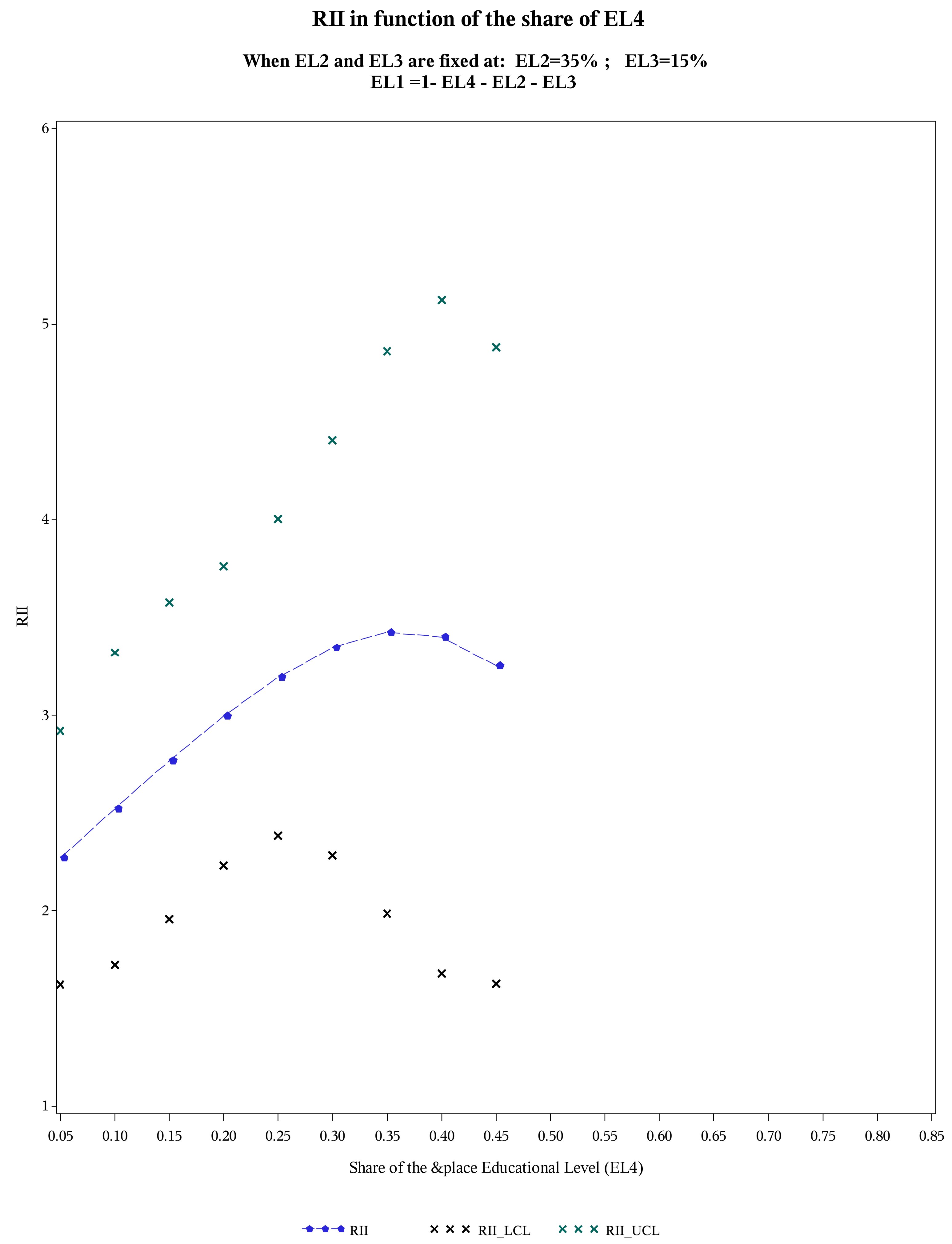

RII in function of the share of EL4
When EL2 and EL3 are fixed at: EL2=35% ; EL3=15%
EL1 =1- EL4 - EL2 - EL3
RII
1
2
3
4
5
6
0.05
0.10
0.15
0.20
0.25
0.30
0.35
0.40
0.45
0.50
0.55
0.60
0.65
0.70
0.75
0.80
0.85
Share of the &place Educational Level (EL4)
RII
RII_LCL
RII_UCL

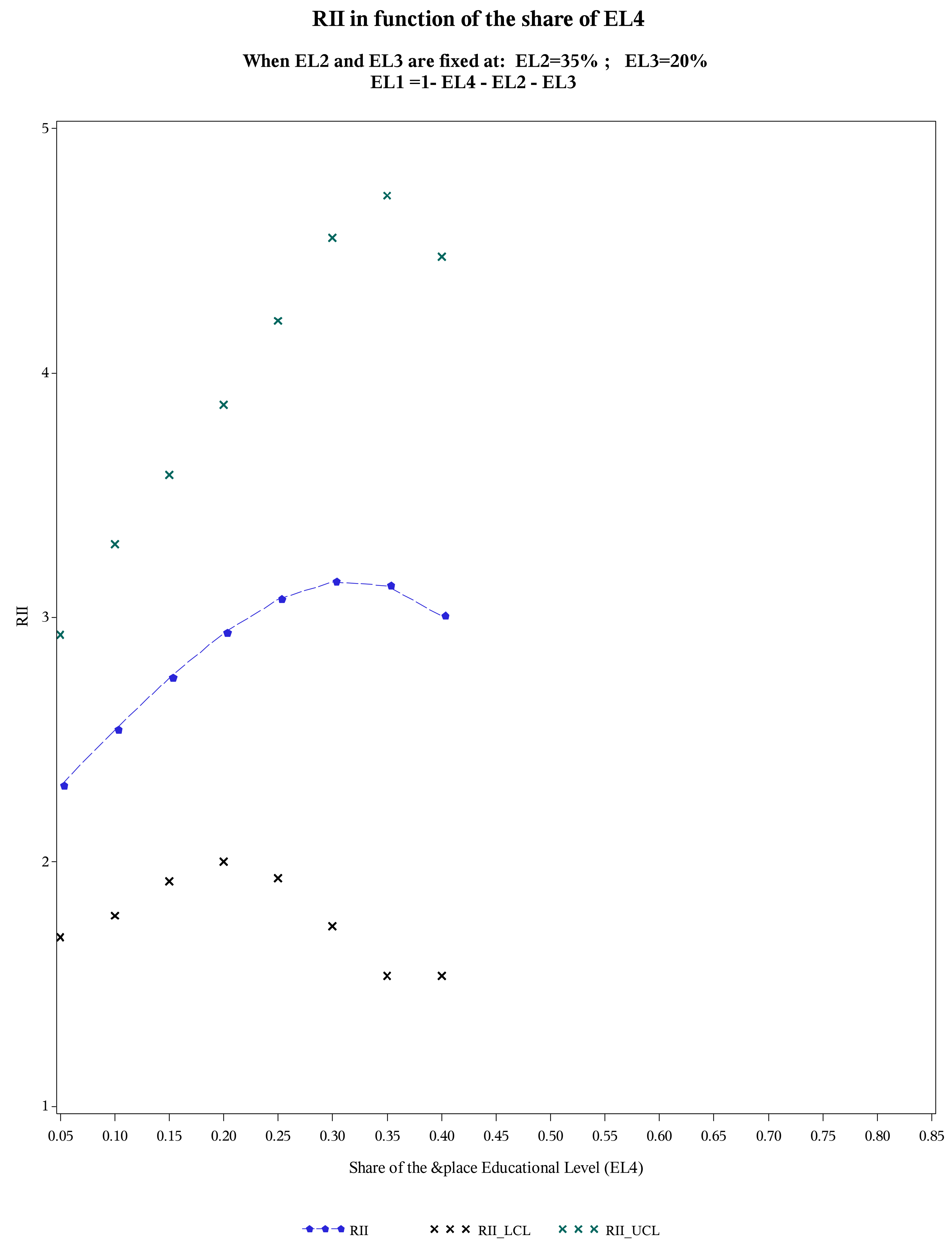
RII in function of the share of EL4
When EL2 and EL3 are fixed at: EL2=35% ; EL3=20%
EL1 =1- EL4 - EL2 - EL3
RII
1
2
3
4
5
0.05
0.10
0.15
0.20
0.25
0.30
0.35
0.40
0.45
0.50
0.55
0.60
0.65
0.70
0.75
0.80
0.85
Share of the &place Educational Level (EL4)
RII
RII_LCL
RII_UCL

RII in function of the share of EL4

When EL2 and EL3 are fixed at: EL2=35% ; EL3=25%
EL1 =1- EL4 - EL2 - EL3

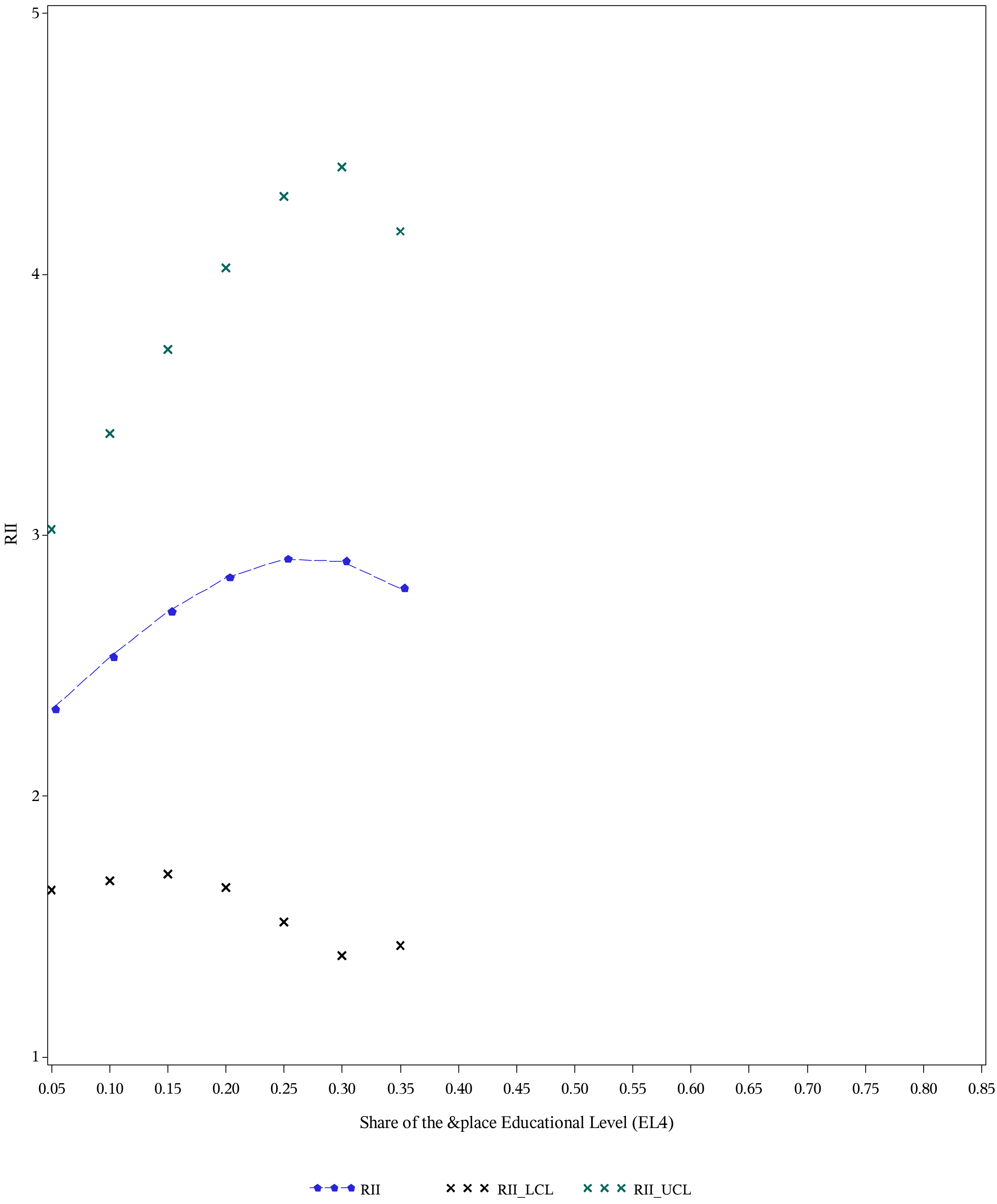

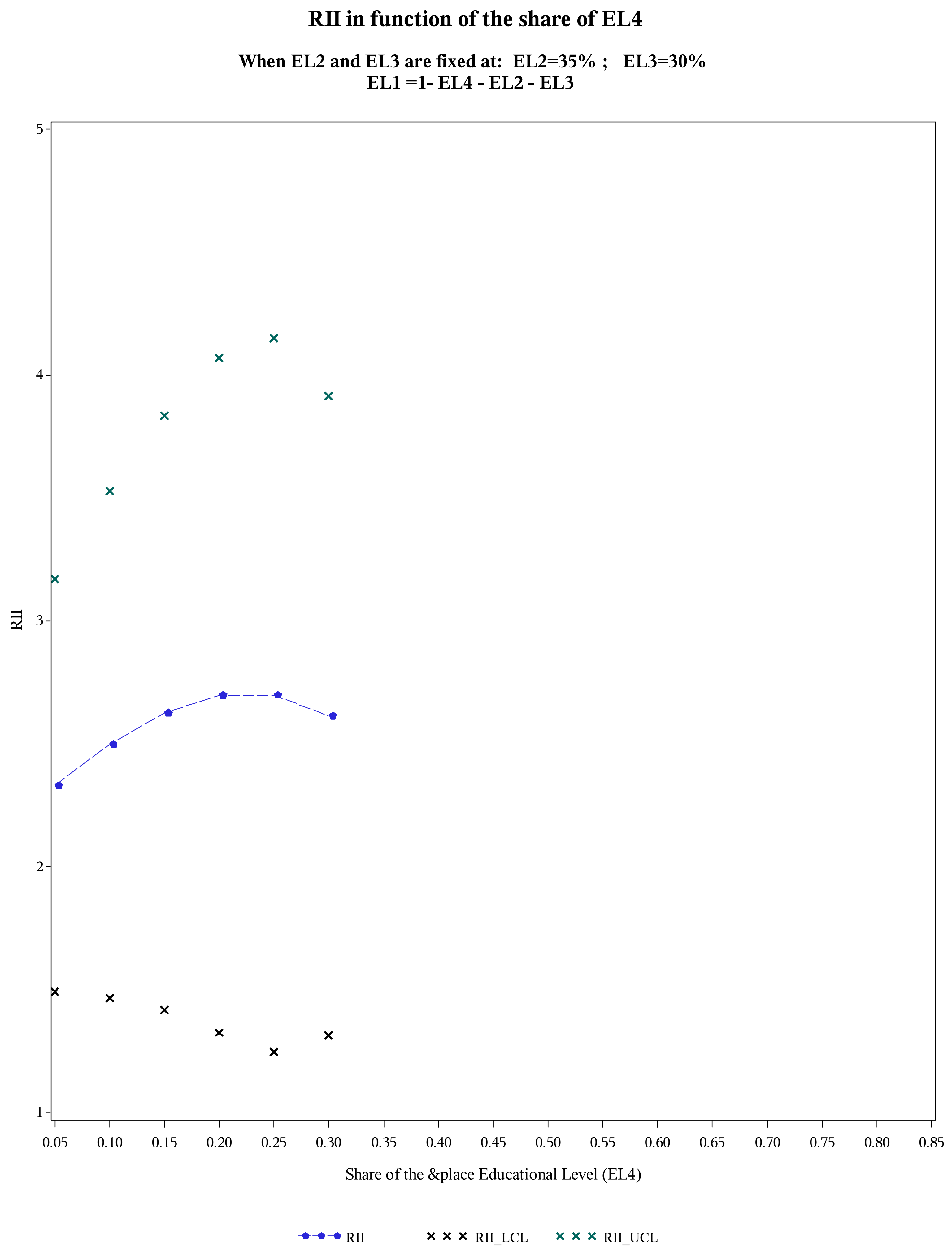
RII in function of the share of EL4
When EL2 and EL3 are fixed at: EL2=35% ; EL3=30%
EL1 =1- EL4 - EL2 - EL3
RII
1
2
3
4
5
0.05
0.10
0.15
0.20
0.25
0.30
0.35
0.40
0.45
0.50
0.55
0.60
0.65
0.70
0.75
0.80
0.85
Share of the &place Educational Level (EL4)
RII
RII_LCL
RII_UCL

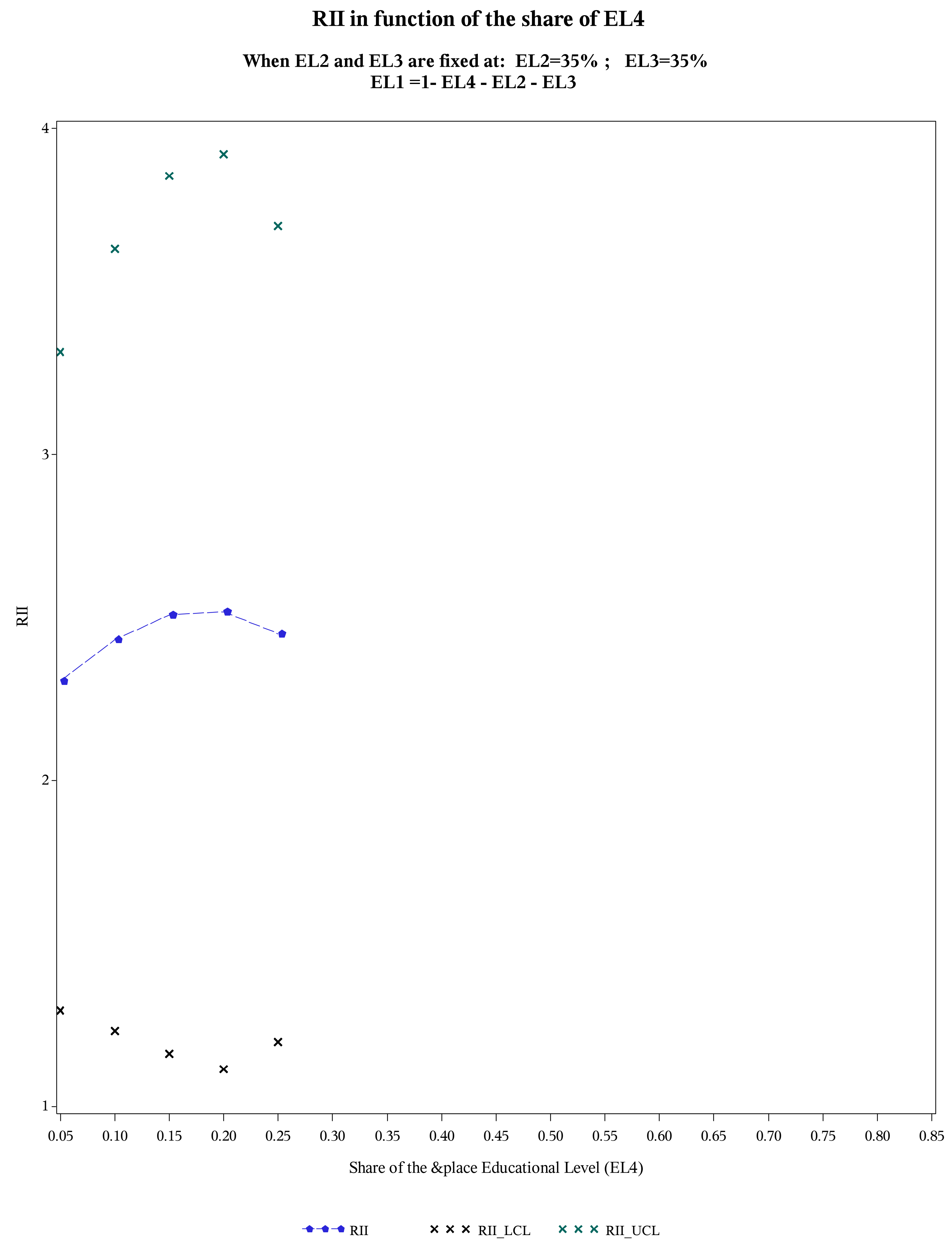
RII in function of the share of EL4
When EL2 and EL3 are fixed at: EL2=35% ; EL3=35%
EL1 =1- EL4 - EL2 - EL3
4
3
2
1
RII
0.05
0.10
0.15
0.20
0.25
0.30
0.35
0.40
0.45
0.50
0.55
0.60
0.65
0.70
0.75
0.80
0.85
Share of the &place Educational Level (EL4)
RII
RII_LCL
RII_UCL

RII in function of the share of EL4

When EL2 and EL3 are fixed at: EL2=35% ; EL3=40%
EL1 =1- EL4 - EL2 - EL3

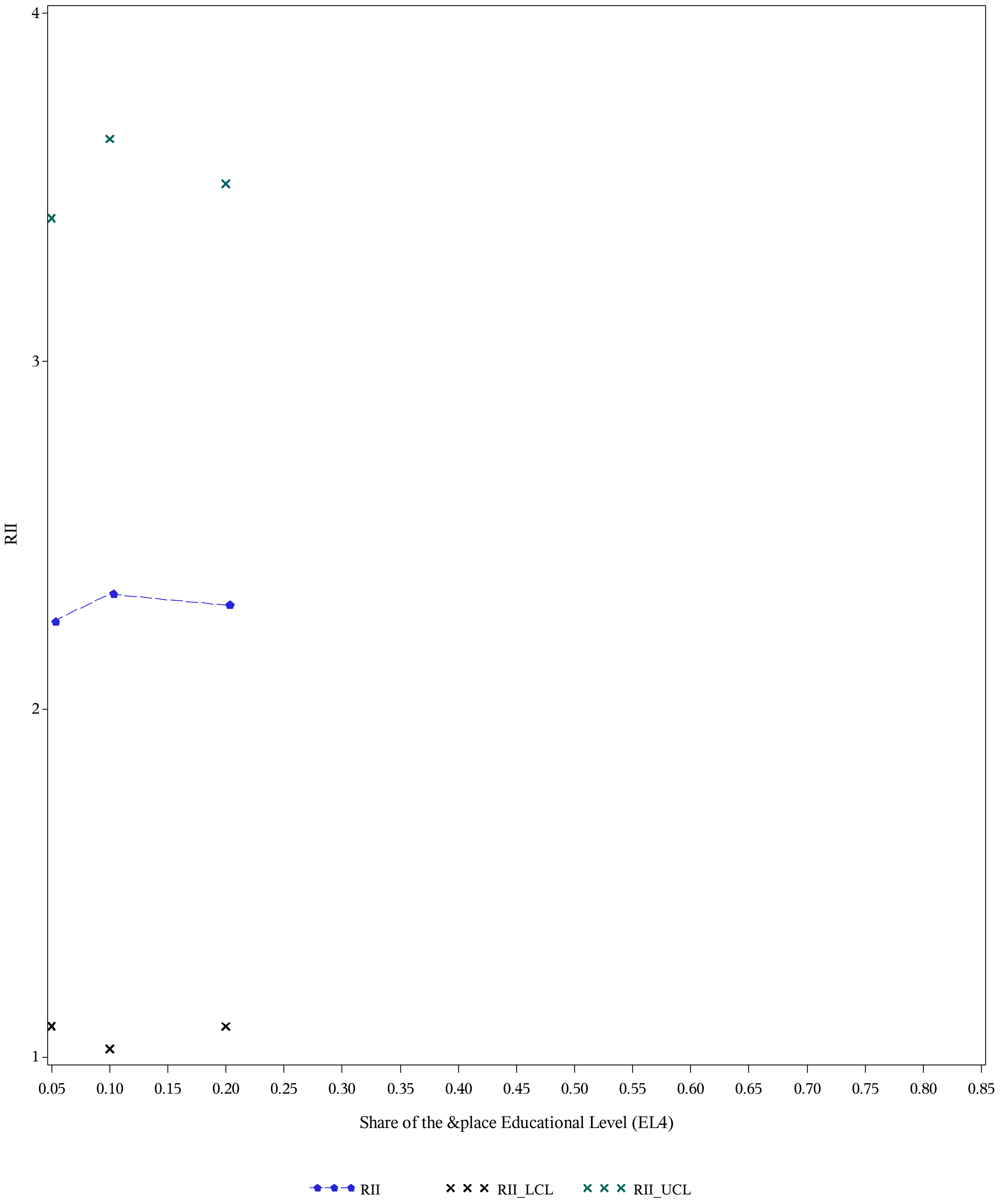

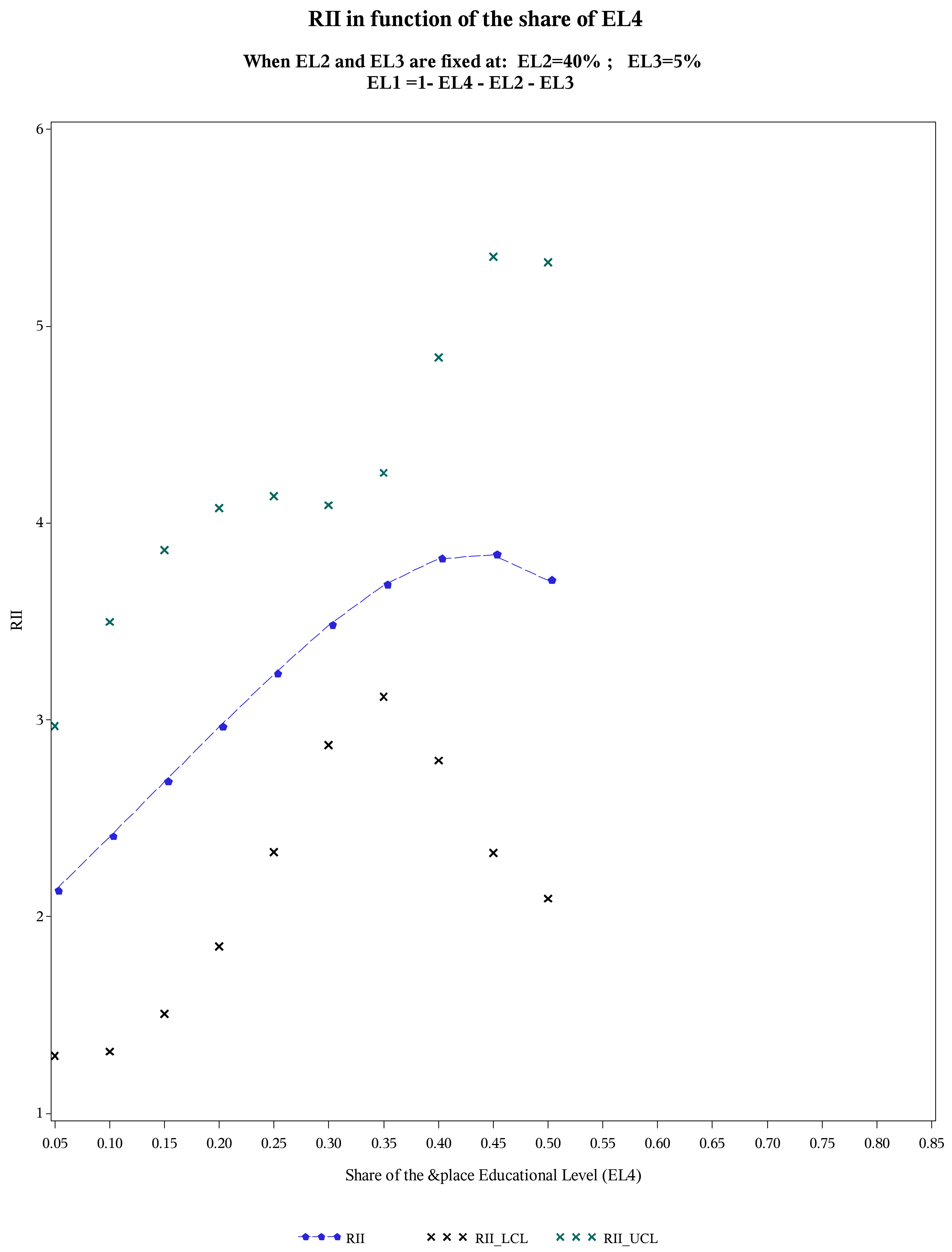
RII in function of the share of EL4
When EL2 and EL3 are fixed at: EL2=40% ; EL3=5%
EL1 =1- EL4 - EL2 - EL3
RII
6
5
4
3
2
1
0.05
0.10
0.15
0.20
0.25
0.30
0.35
0.40
0.45
0.50
0.55
0.60
0.65
0.70
0.75
0.80
0.85
Share of the &place Educational Level (EL4)
RII
RII_LCL
RII_UCL

RII in function of the share of EL4

When EL2 and EL3 are fixed at: EL2=40% ; EL3=10%
EL1 =1- EL4 - EL2 - EL3

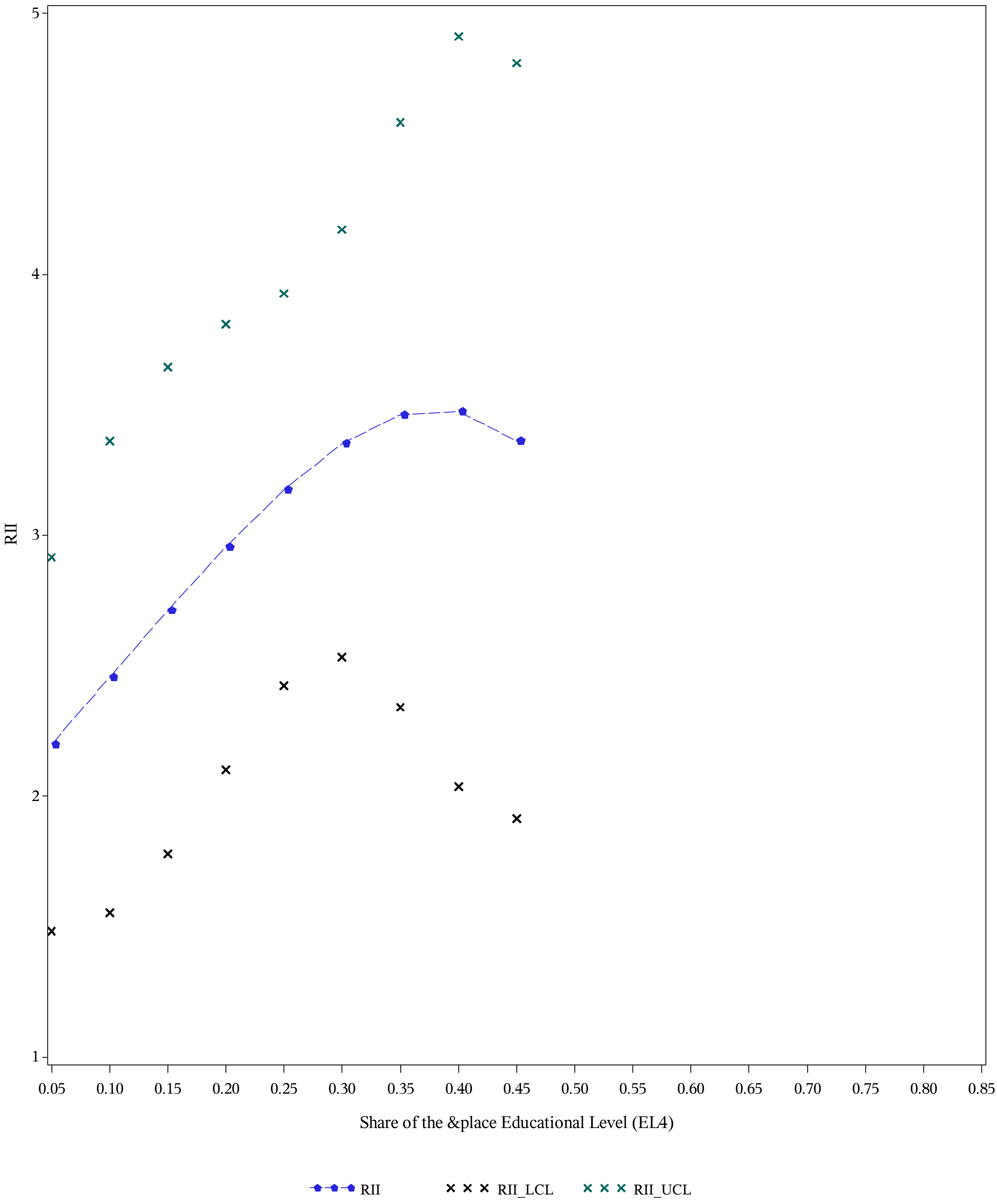

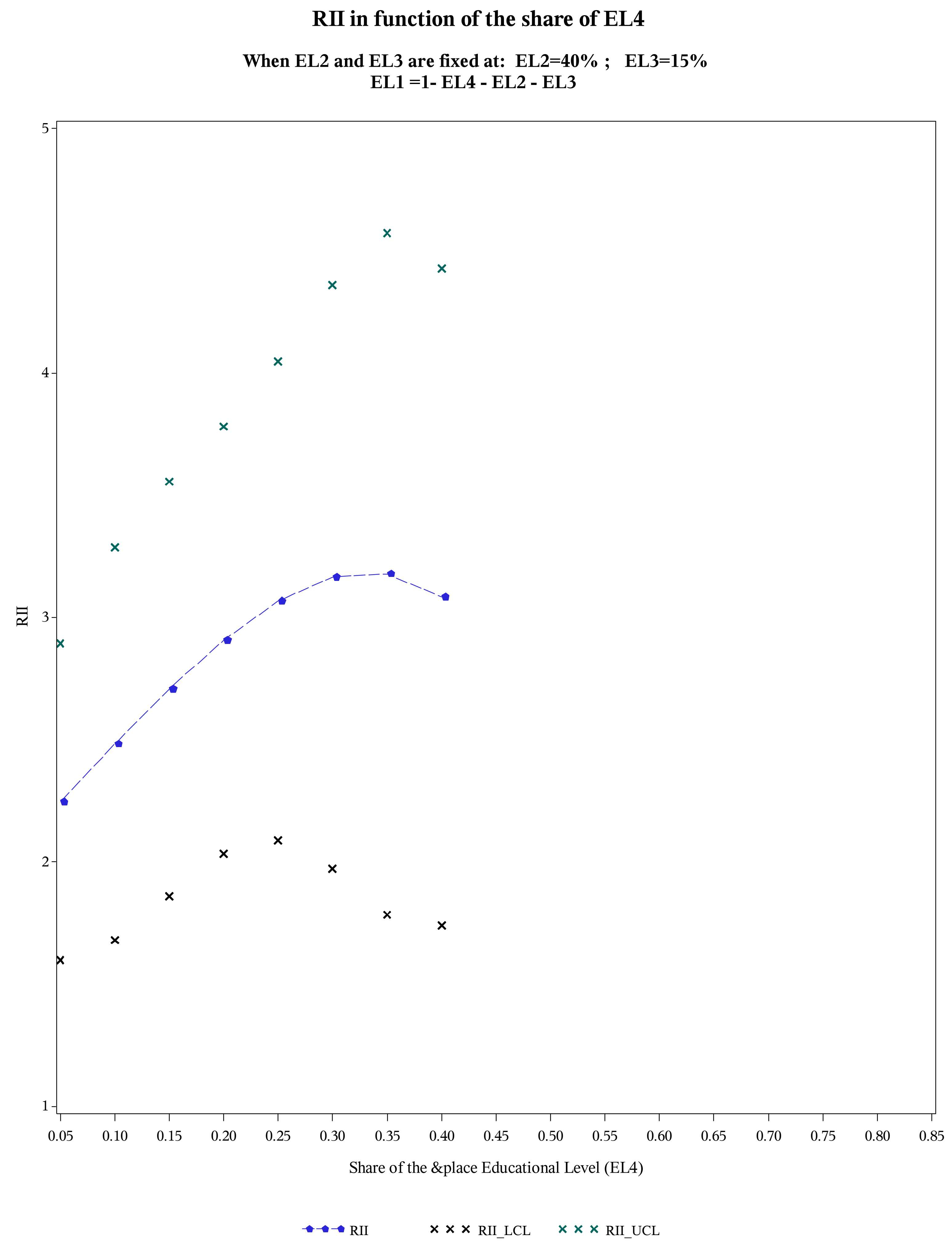
RII in function of the share of EL4
When EL2 and EL3 are fixed at: EL2=40% ; EL3=15%
EL1 =1- EL4 - EL2 - EL3
RII
5
4
3
2
1
0.05
0.10
0.15
0.20
0.25
0.30
0.35
0.40
0.45
0.50
0.55
0.60
0.65
0.70
0.75
0.80
0.85
Share of the &place Educational Level (EL4)
RII
RII_LCL
RII_UCL

RII in function of the share of EL4

When EL2 and EL3 are fixed at: EL2=40% ; EL3=20%
EL1 =1- EL4 - EL2 - EL3

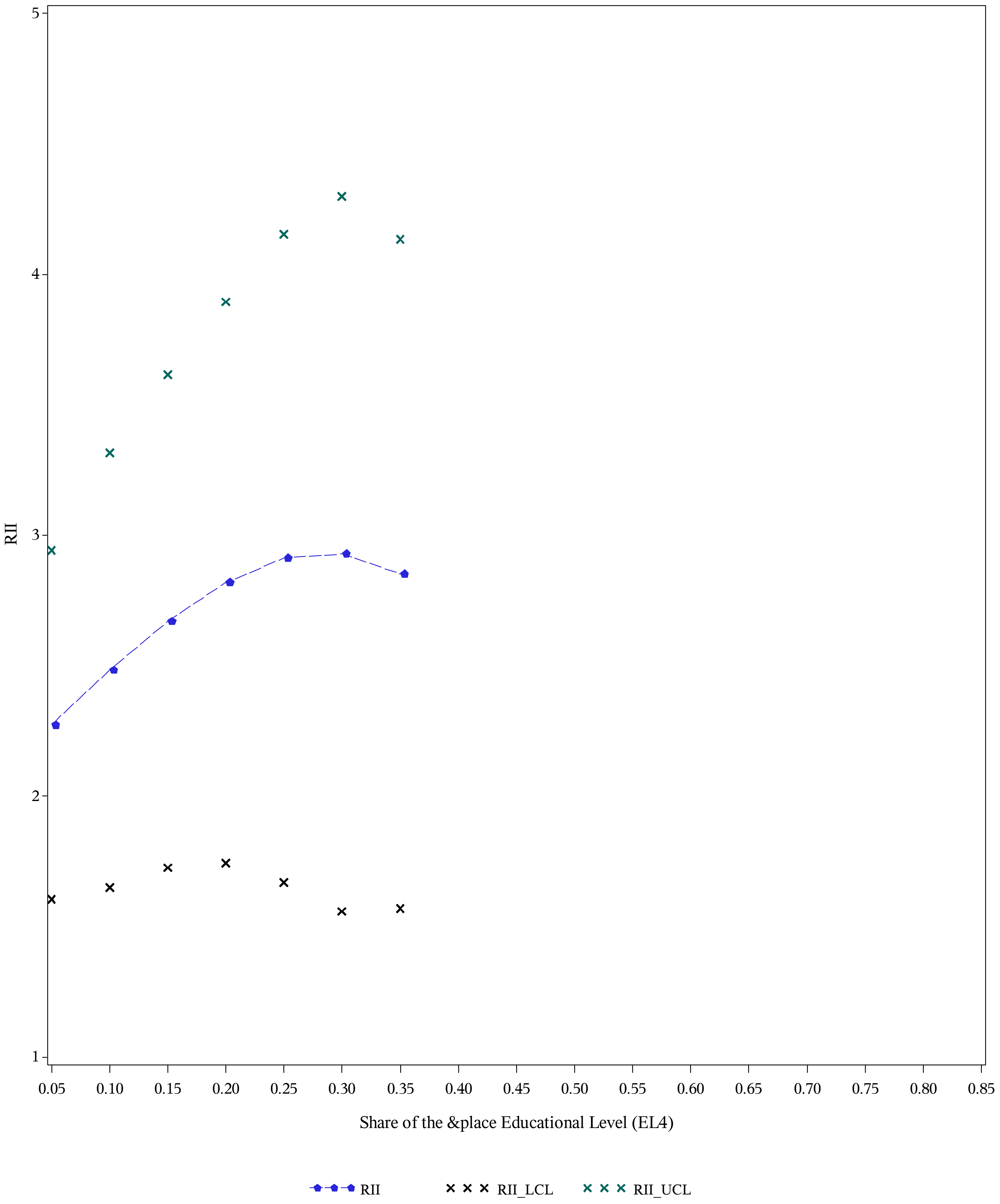

RII in function of the share of EL4

When EL2 and EL3 are fixed at: EL2=40% ; EL3=25%
EL1 =1- EL4 - EL2 - EL3

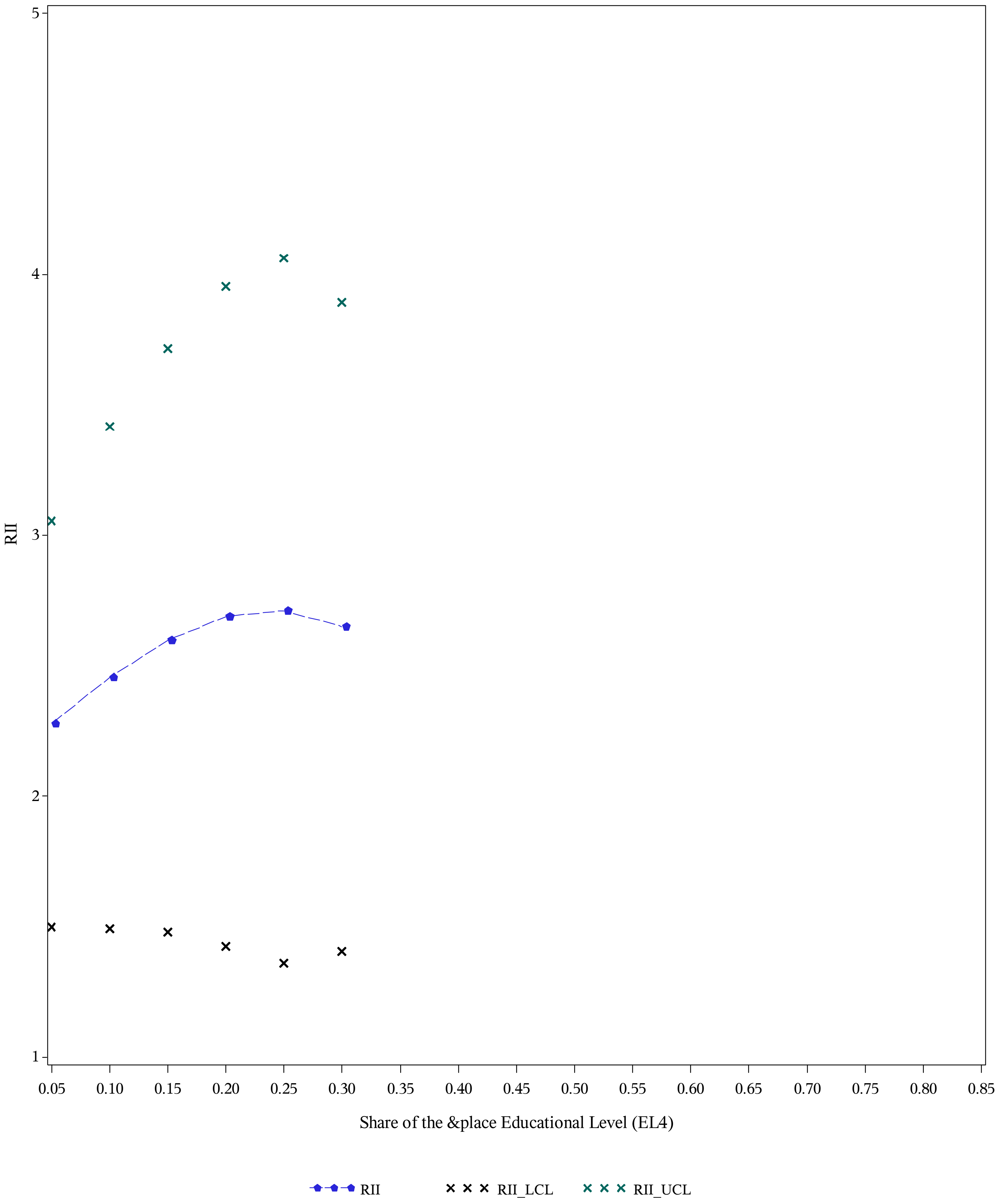

RII in function of the share of EL4

When EL2 and EL3 are fixed at: EL2=40% ; EL3=30%
EL1 =1- EL4 - EL2 - EL3

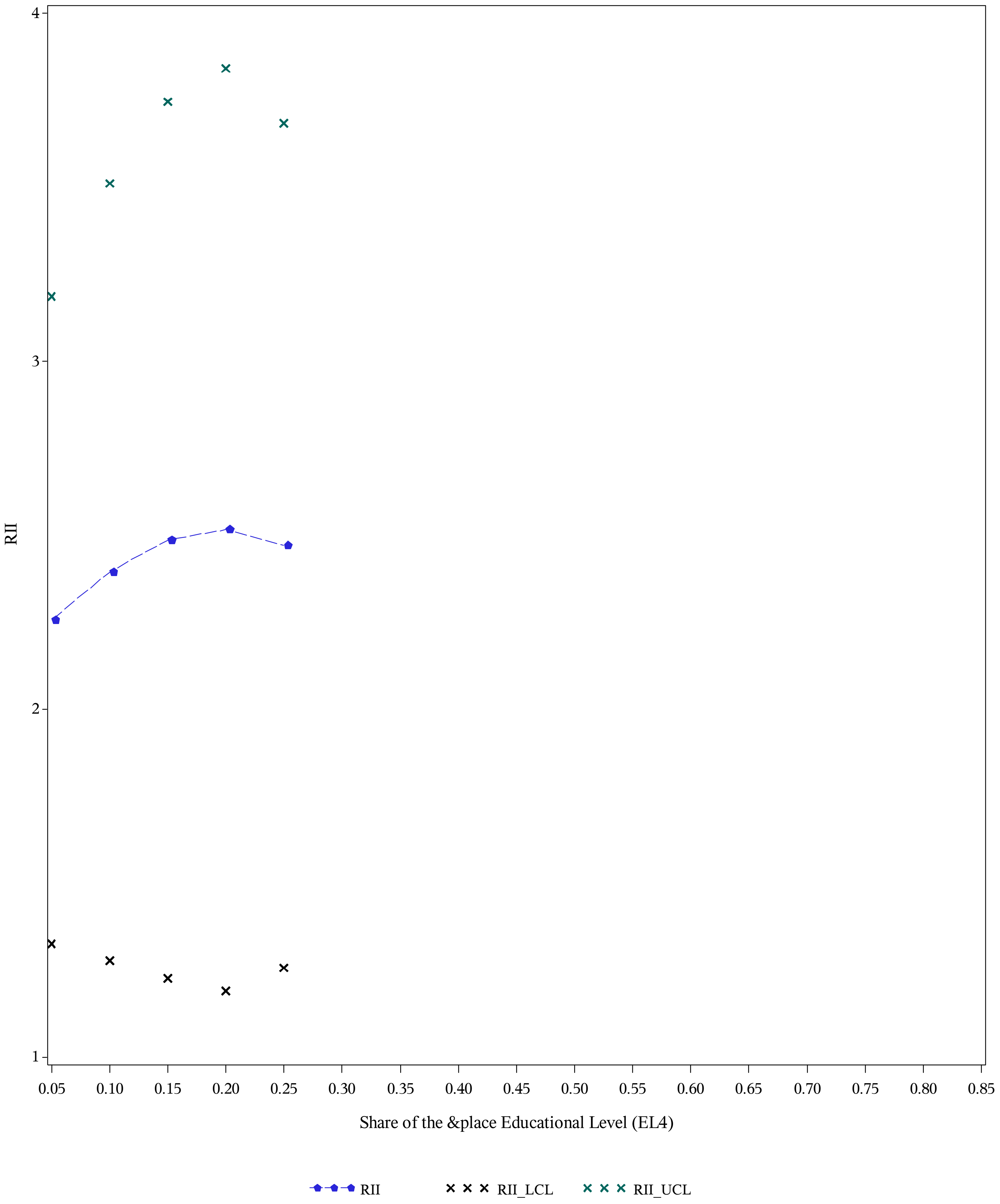

RII in function of the share of EL4

When EL2 and EL3 are fixed at: EL2=40% ; EL3=35%
EL1 =1- EL4 - EL2 - EL3

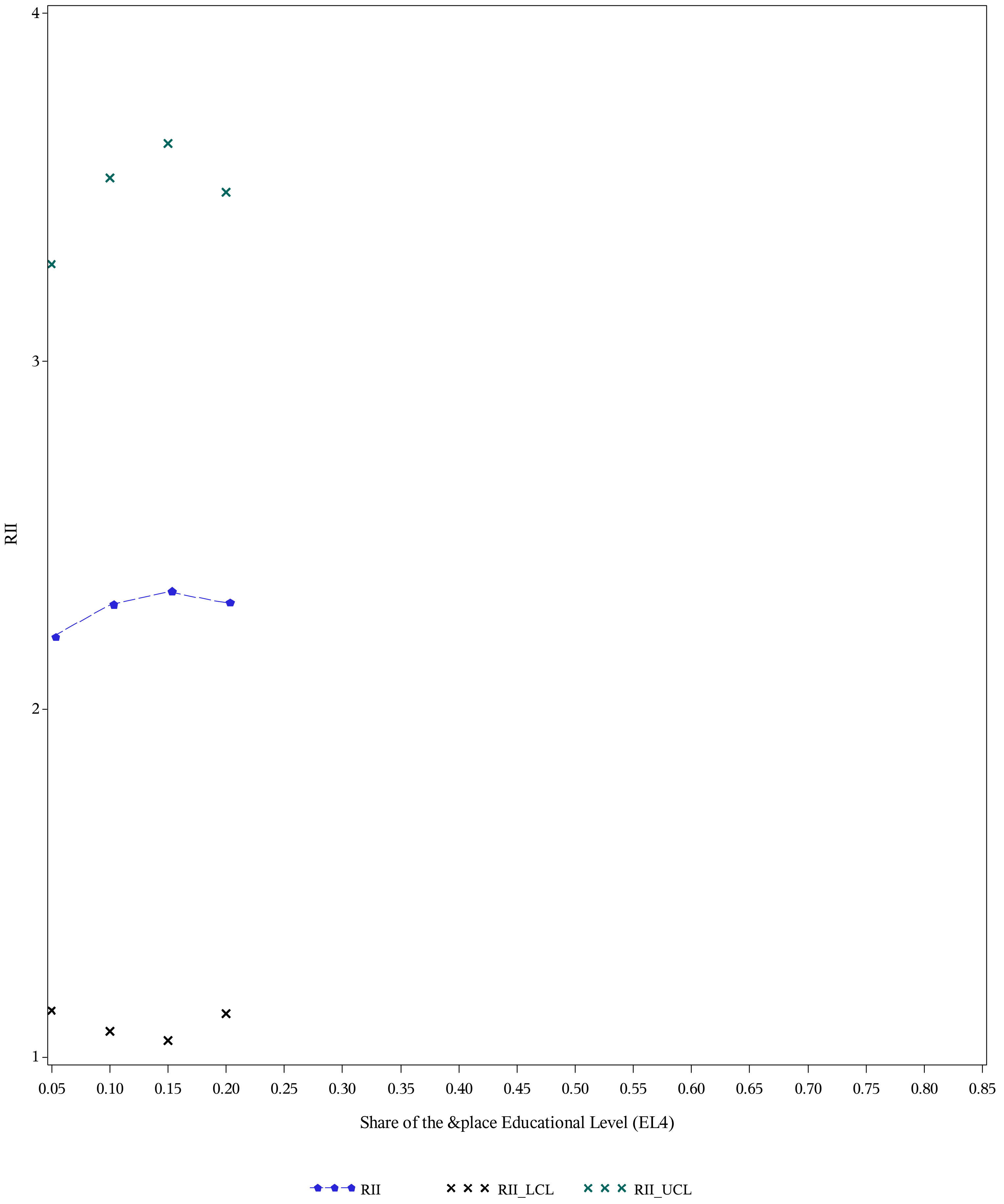

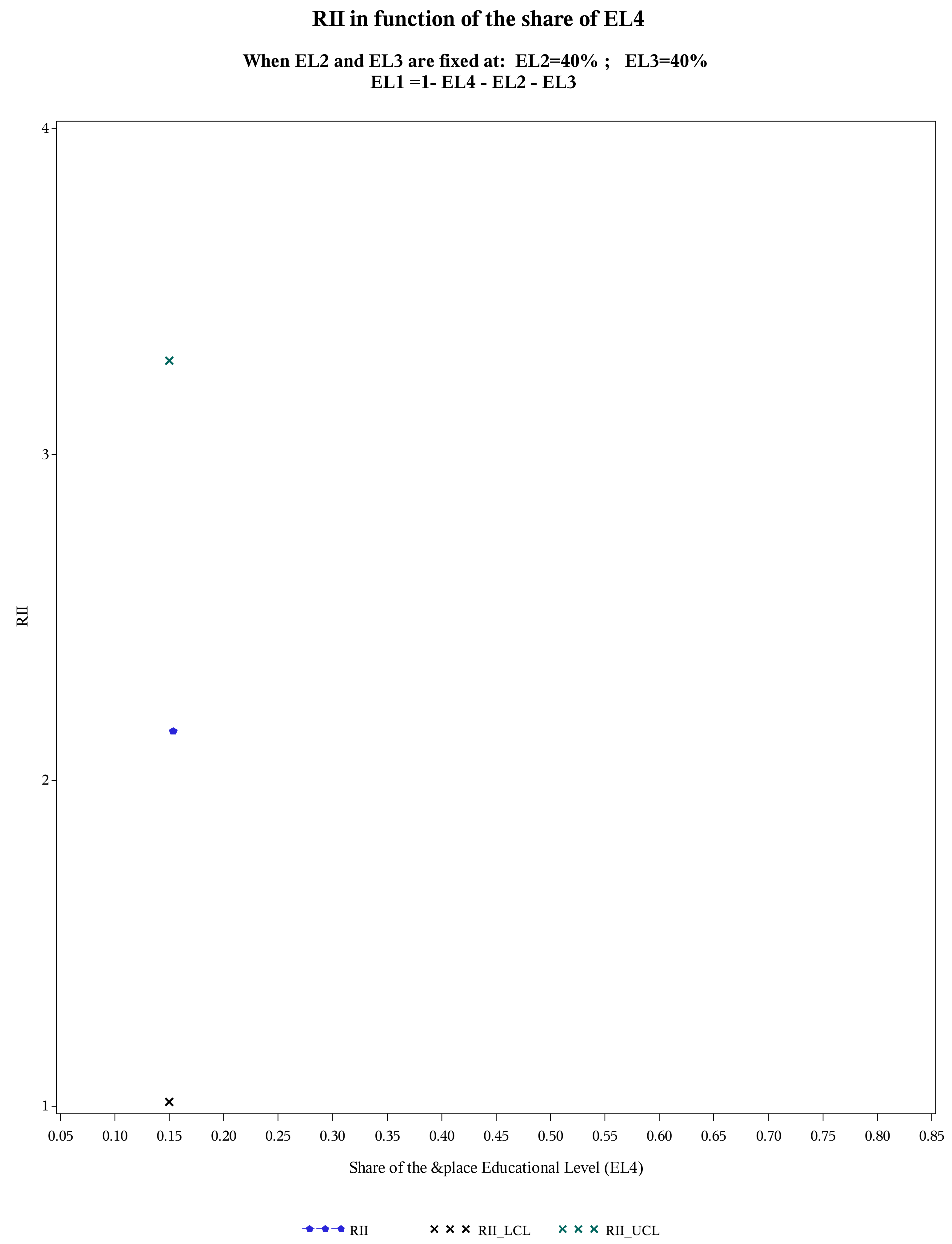

RII in function of the share of EL4
When EL2 and EL3 are fixed at: EL2=40% ; EL3=40%
EL1 =1- EL4 - EL2 - EL3
RII
1
2
3
4
0.05
0.10
0.15
0.20
0.25
0.30
0.35
0.40
0.45
0.50
0.55
0.60
0.65
0.70
0.75
0.80
0.85
Share of the &place Educational Level (EL4)
RII
RII_LCL
RII_UCL

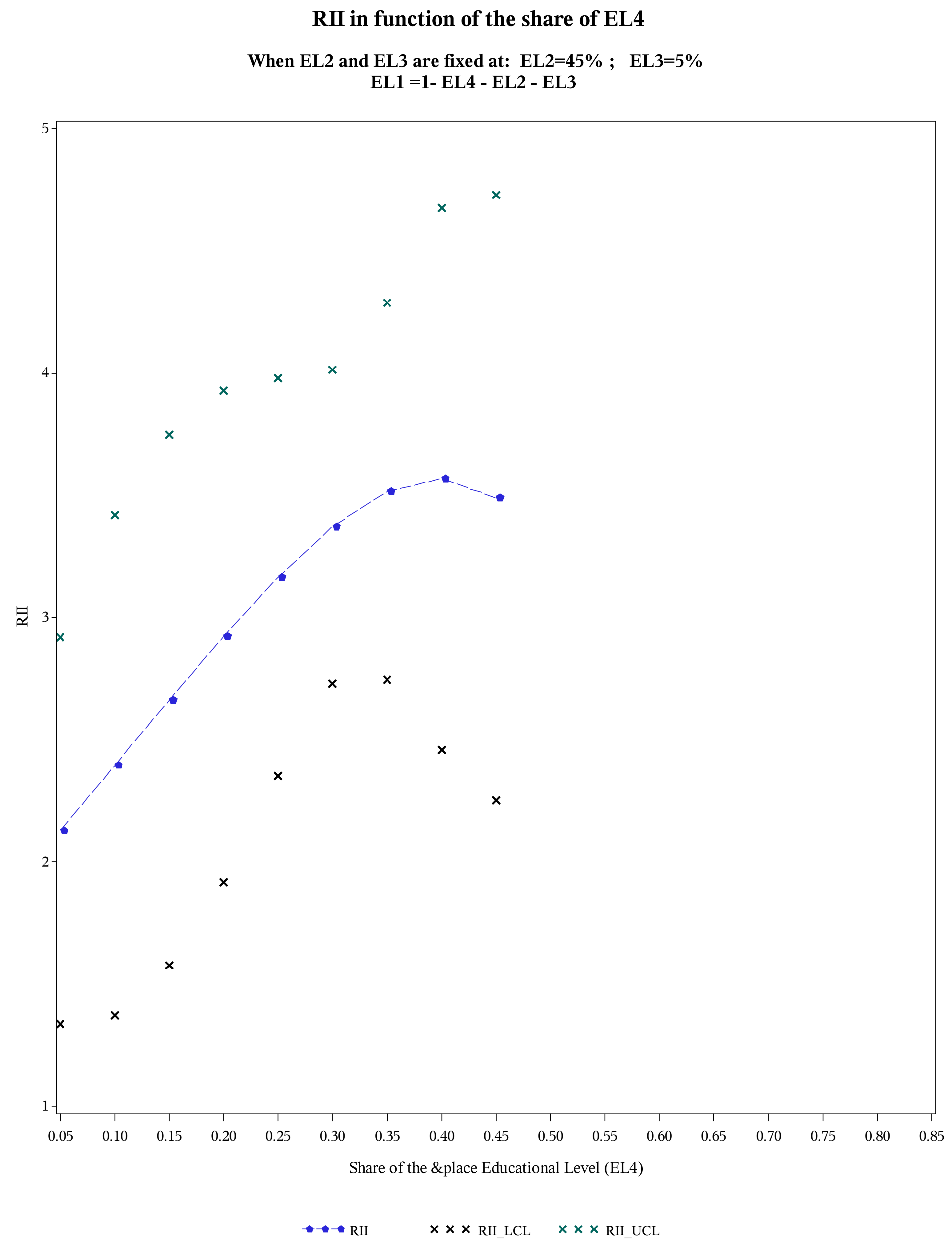

RII in function of the share of EL4
When EL2 and EL3 are fixed at: EL2=45% ; EL3=5%
EL1 =1- EL4 - EL2 - EL3
RII
1
2
3
4
5
0.05
0.10
0.15
0.20
0.25
0.30
0.35
0.40
0.45
0.50
0.55
0.60
0.65
0.70
0.75
0.80
0.85
Share of the &place Educational Level (EL4)
RII
RII_LCL
RII_UCL

RII in function of the share of EL4

When EL2 and EL3 are fixed at: EL2=45% ; EL3=10%
EL1 =1- EL4 - EL2 - EL3

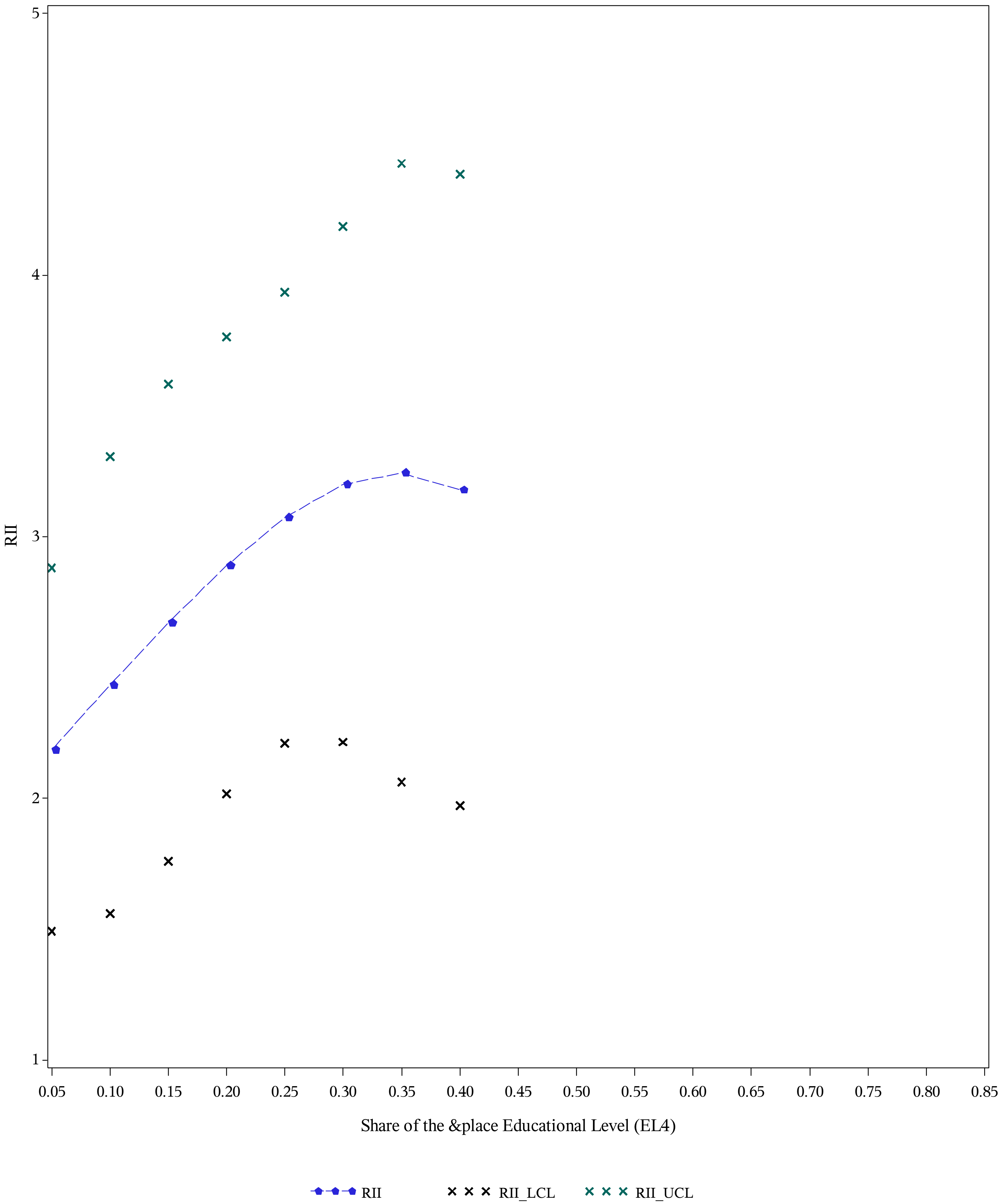

RII in function of the share of EL4

When EL2 and EL3 are fixed at: EL2=45% ; EL3=15%
EL1 =1- EL4 - EL2 - EL3

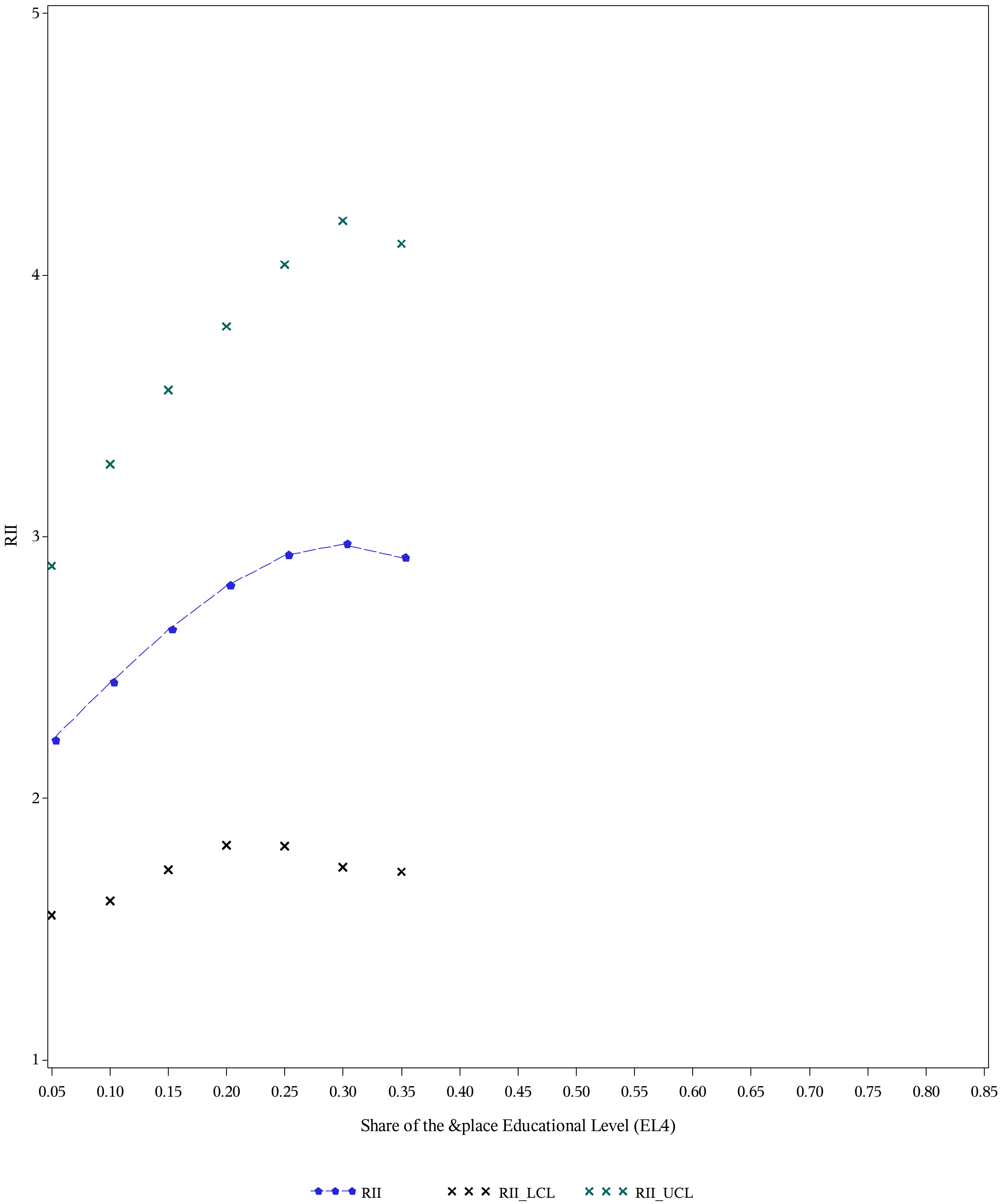

RII in function of the share of EL4

When EL2 and EL3 are fixed at: EL2=45% ; EL3=20%
EL1 =1- EL4 - EL2 - EL3

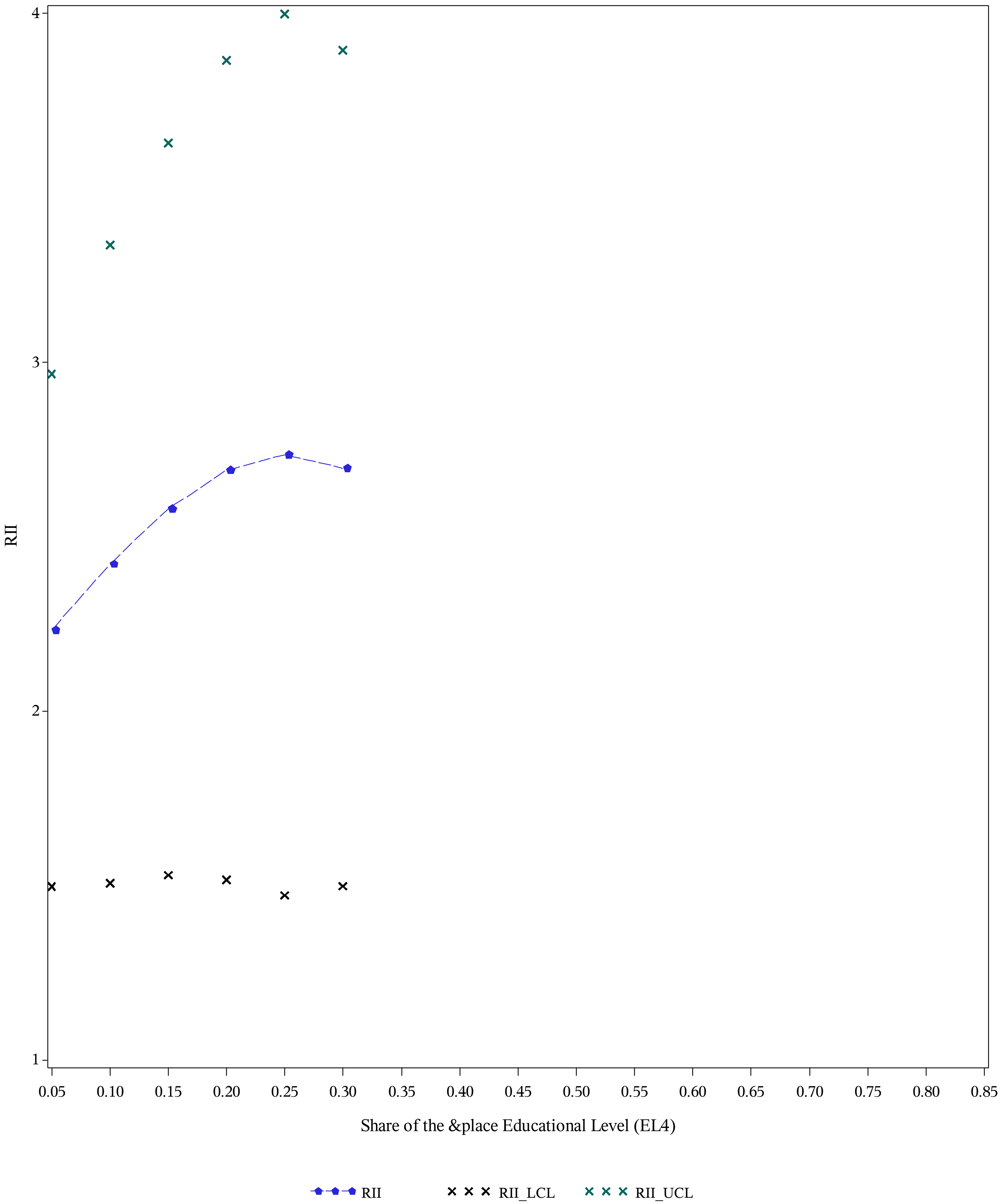

RII in function of the share of EL4

When EL2 and EL3 are fixed at: EL2=45% ; EL3=25%
EL1 =1- EL4 - EL2 - EL3

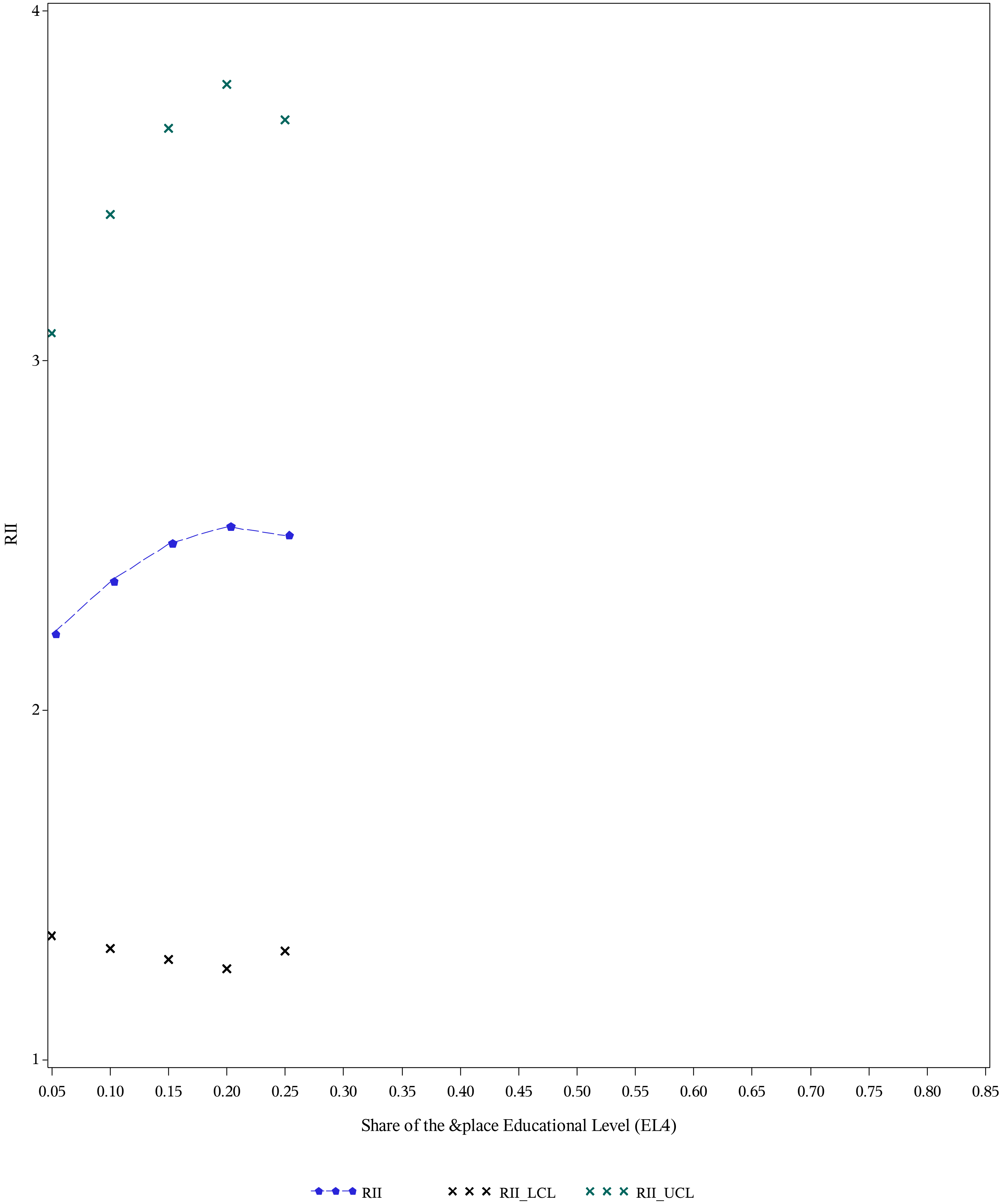

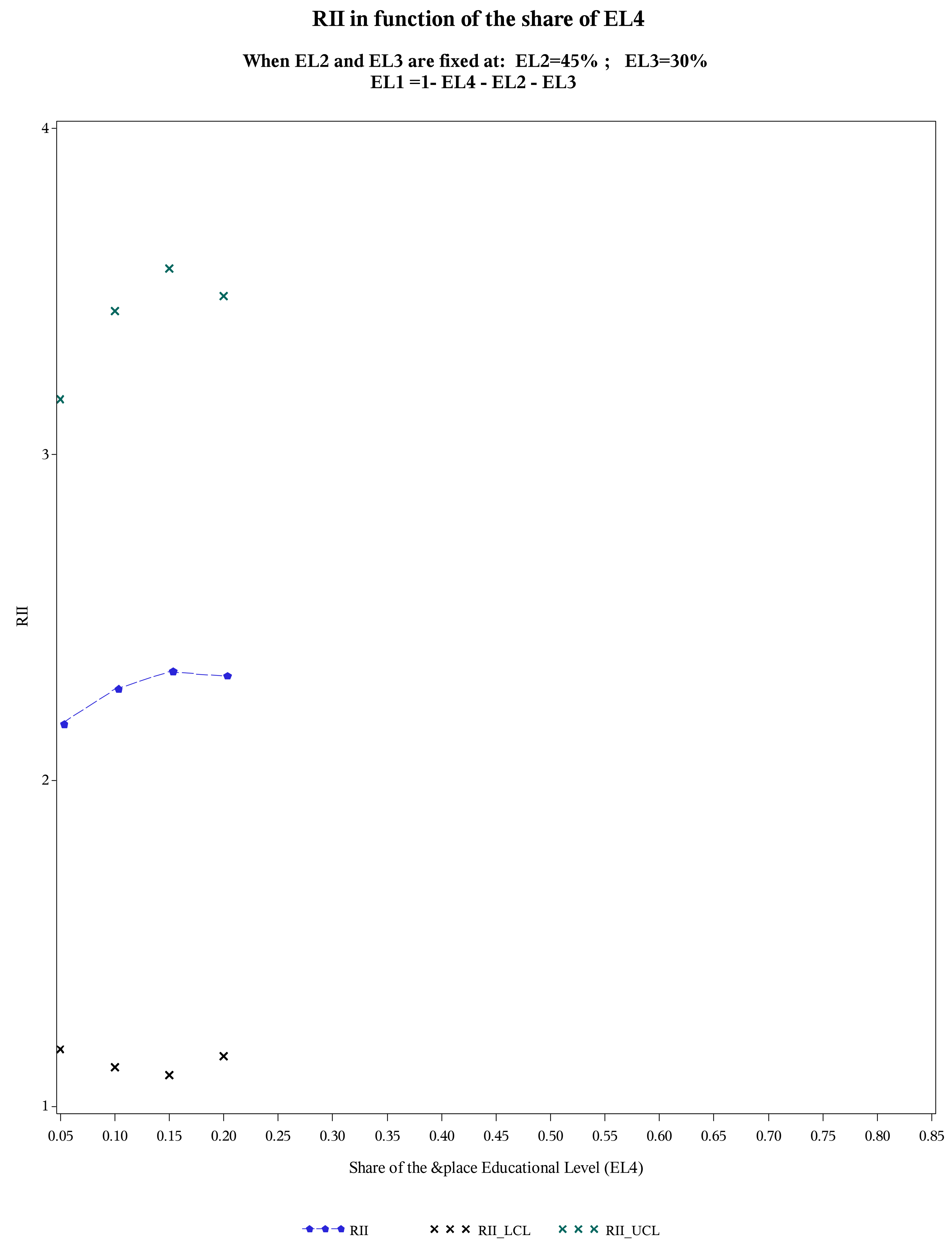

RII in function of the share of EL4
When EL2 and EL3 are fixed at: EL2=45% ; EL3=30%
EL1 =1- EL4 - EL2 - EL3
RII
1
2
3
4
0.05
0.10
0.15
0.20
0.25
0.30
0.35
0.40
0.45
0.50
0.55
0.60
0.65
0.70
0.75
0.80
0.85
Share of the &place Educational Level (EL4)
RII
RII_LCL
RII_UCL

RII in function of the share of EL4

When EL2 and EL3 are fixed at: EL2=45% ;   EL3=35%
EL1 =1- EL4 - EL2 - EL3

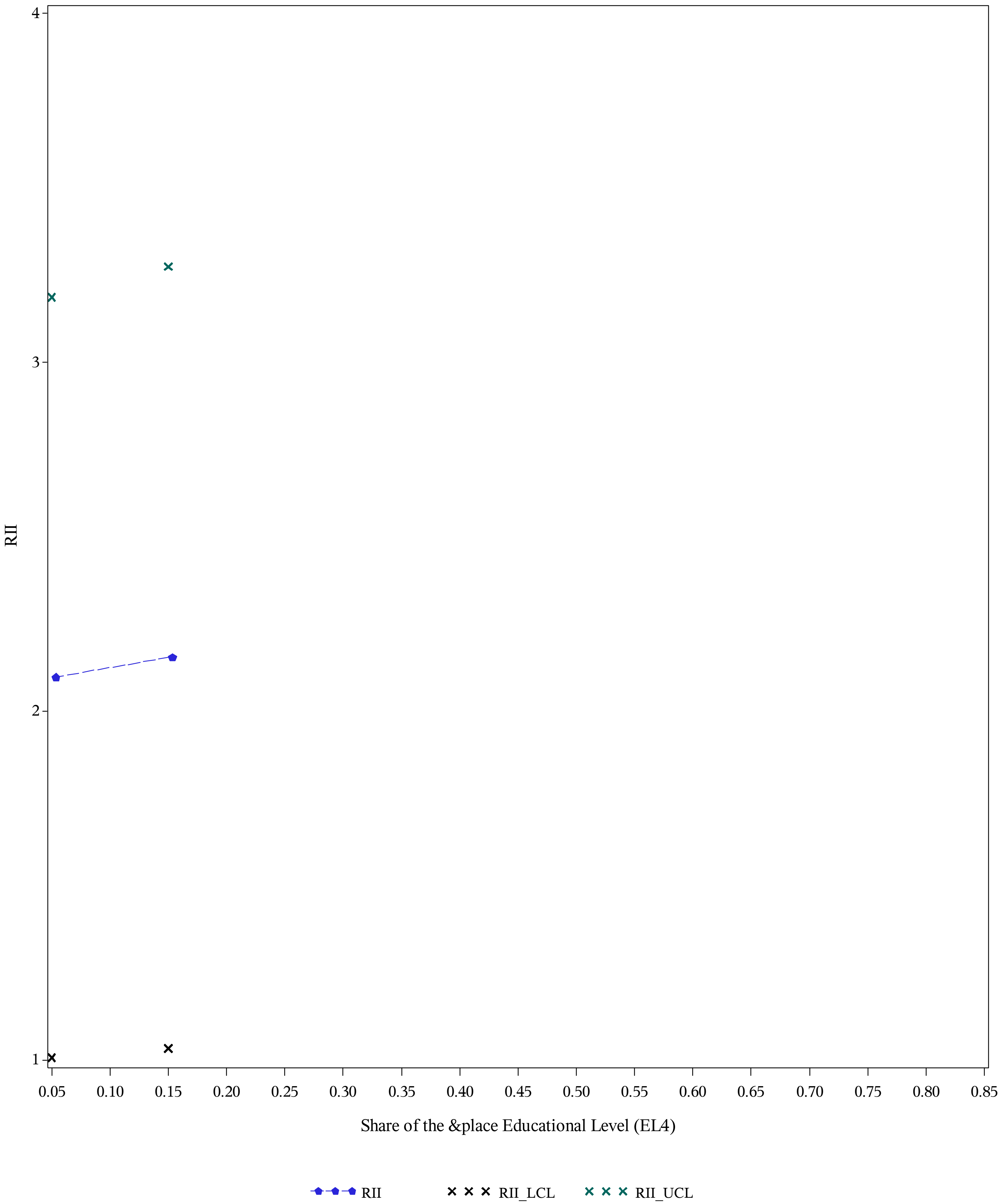

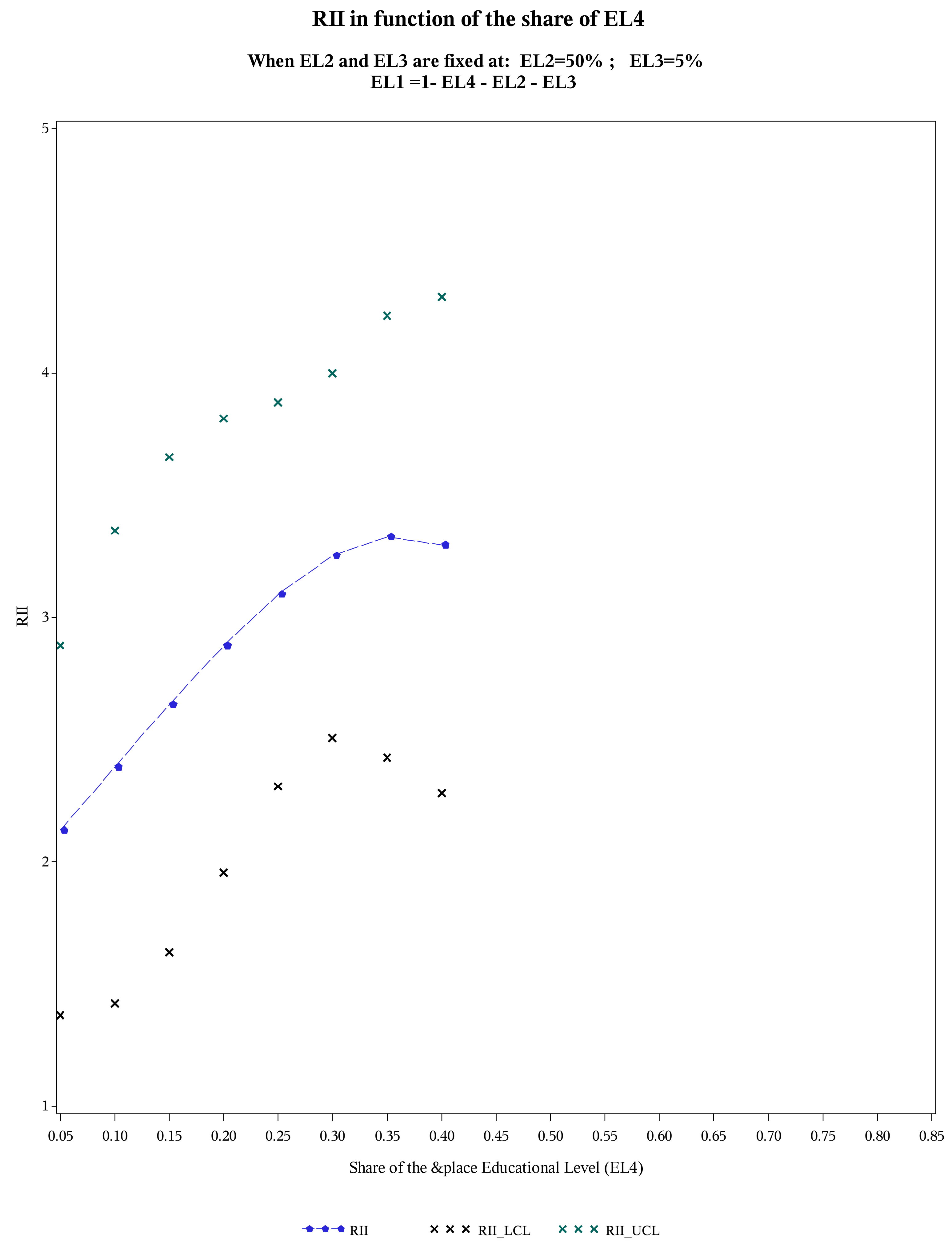
RII in function of the share of EL4
When EL2 and EL3 are fixed at: EL2=50% ; EL3=5%
EL1 =1- EL4 - EL2 - EL3
RII
1
2
3
4
5
0.05
0.10
0.15
0.20
0.25
0.30
0.35
0.40
0.45
0.50
0.55
0.60
0.65
0.70
0.75
0.80
0.85
Share of the &place Educational Level (EL4)
RII
RII_LCL
RII_UCL

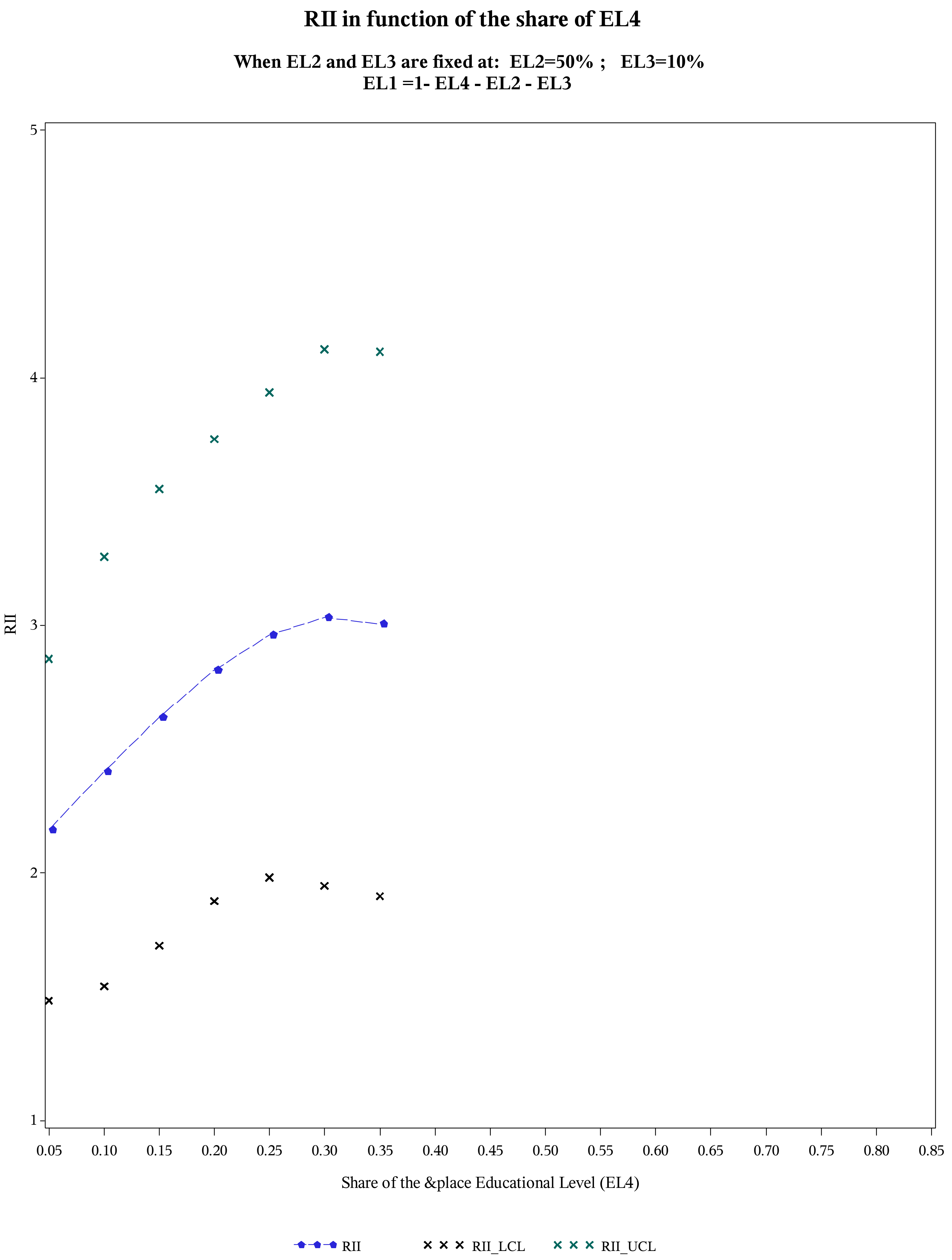
RII in function of the share of EL4
When EL2 and EL3 are fixed at: EL2=50% ; EL3=10%
EL1 =1- EL4 - EL2 - EL3
RII
1
2
3
4
5
0.05
0.10
0.15
0.20
0.25
0.30
0.35
0.40
0.45
0.50
0.55
0.60
0.65
0.70
0.75
0.80
0.85
Share of the &place Educational Level (EL4)
RII
RII_LCL
RII_UCL

RII in function of the share of EL4

When EL2 and EL3 are fixed at: EL2=50% ; EL3=15%
EL1 =1- EL4 - EL2 - EL3

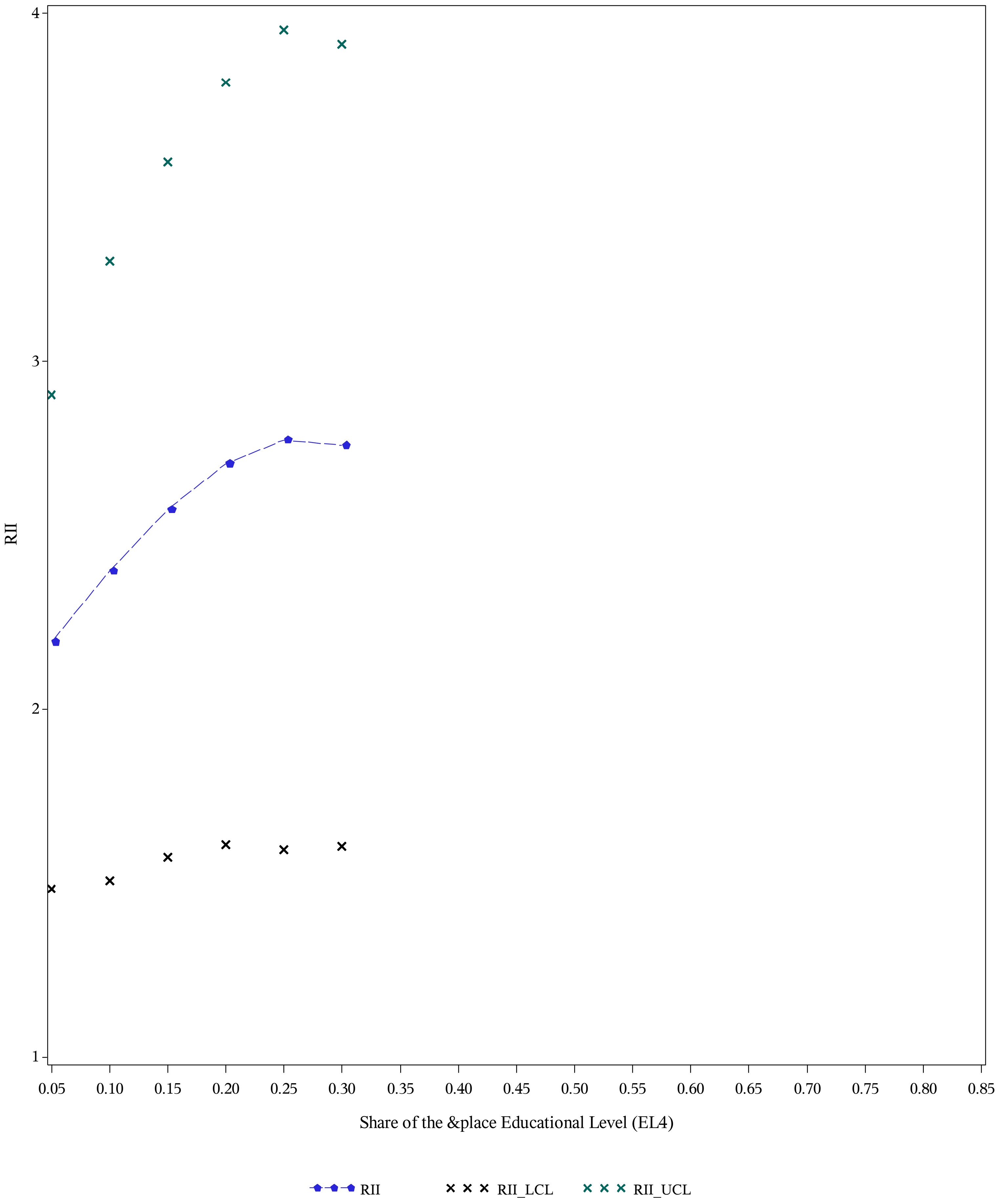

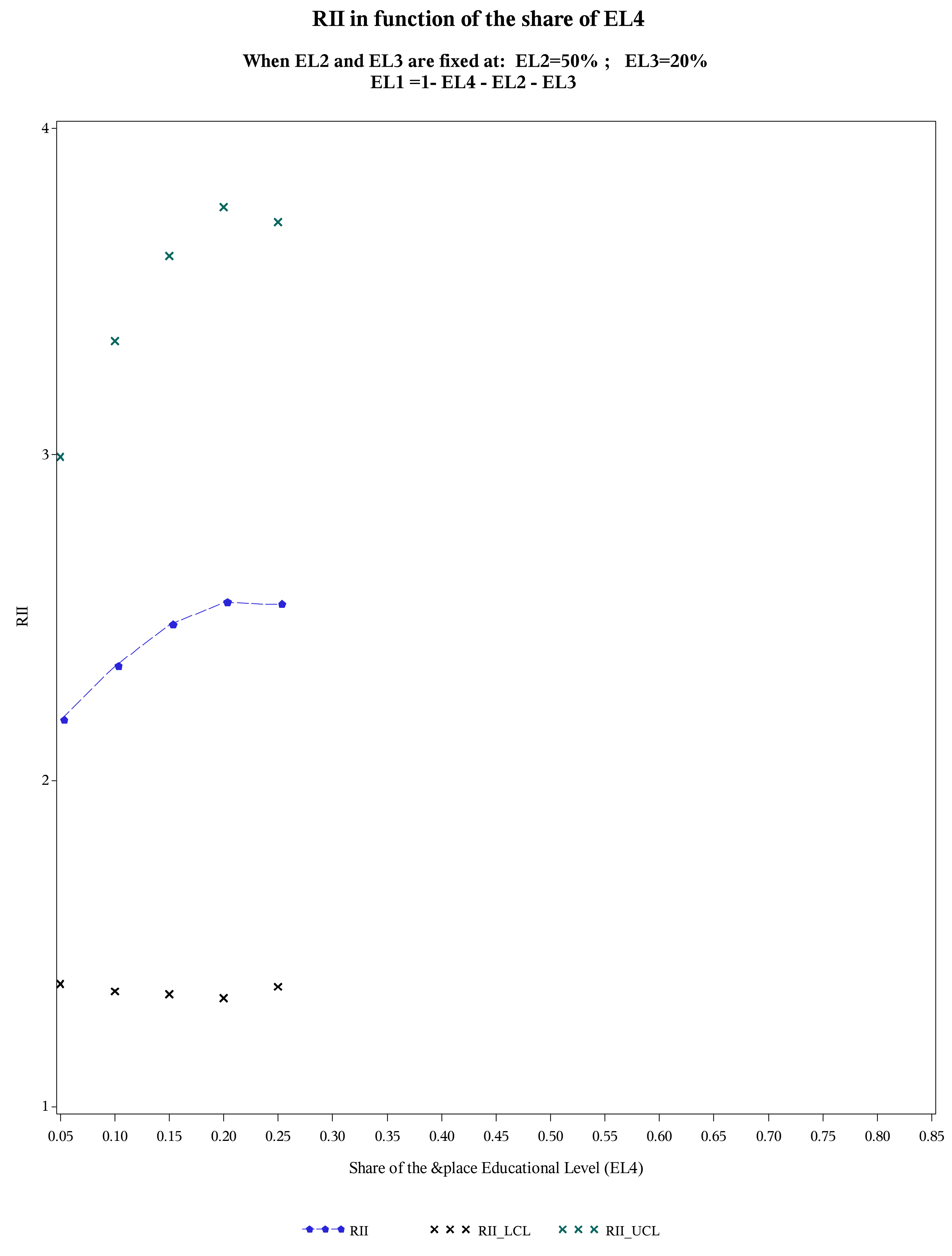
RII in function of the share of EL4
When EL2 and EL3 are fixed at: EL2=50% ; EL3=20%
EL1 =1- EL4 - EL2 - EL3
4
3
2
1
RII
0.05
0.10
0.15
0.20
0.25
0.30
0.35
0.40
0.45
0.50
0.55
0.60
0.65
0.70
0.75
0.80
0.85
Share of the &place Educational Level (EL4)
RII
RII_LCL
RII_UCL

RII in function of the share of EL4

When EL2 and EL3 are fixed at: EL2=50% ; EL3=25%
EL1 =1- EL4 - EL2 - EL3

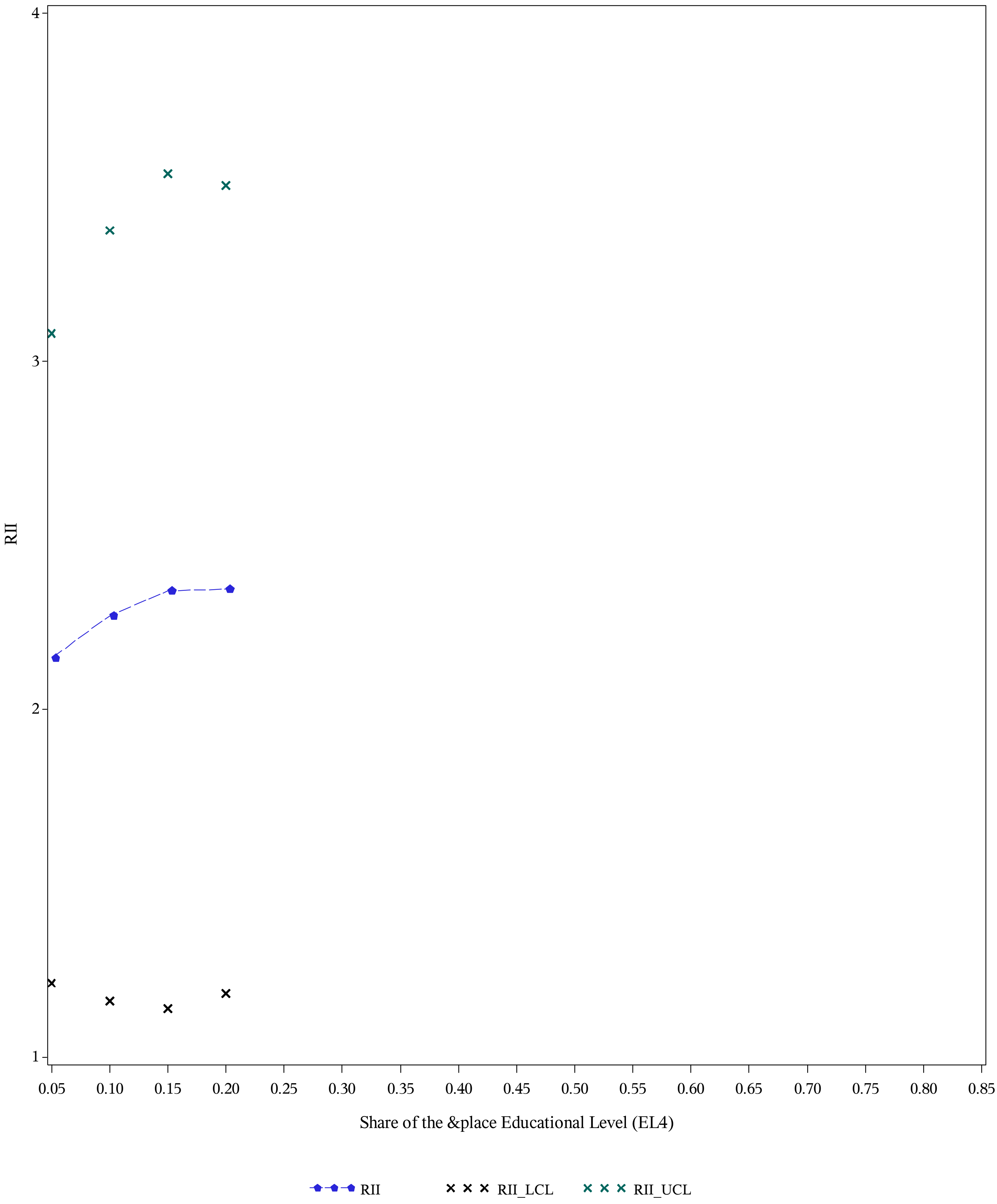

RII in function of the share of EL4

When EL2 and EL3 are fixed at: EL2=50% ; EL3=30%
EL1 =1- EL4 - EL2 - EL3

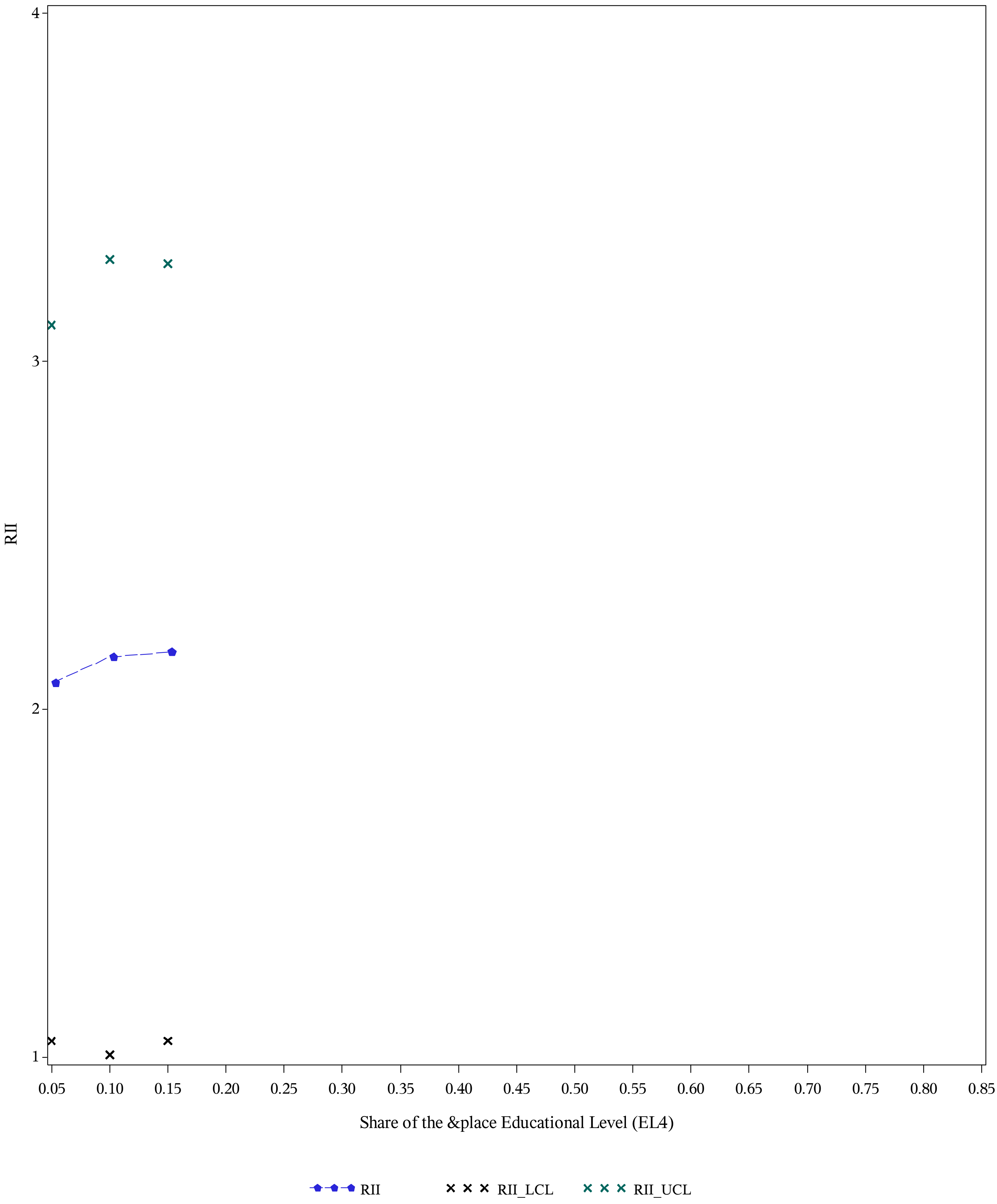

RII in function of the share of EL4

When EL2 and EL3 are fixed at: EL2=55% ; EL3=5%
EL1 =1- EL4 - EL2 - EL3

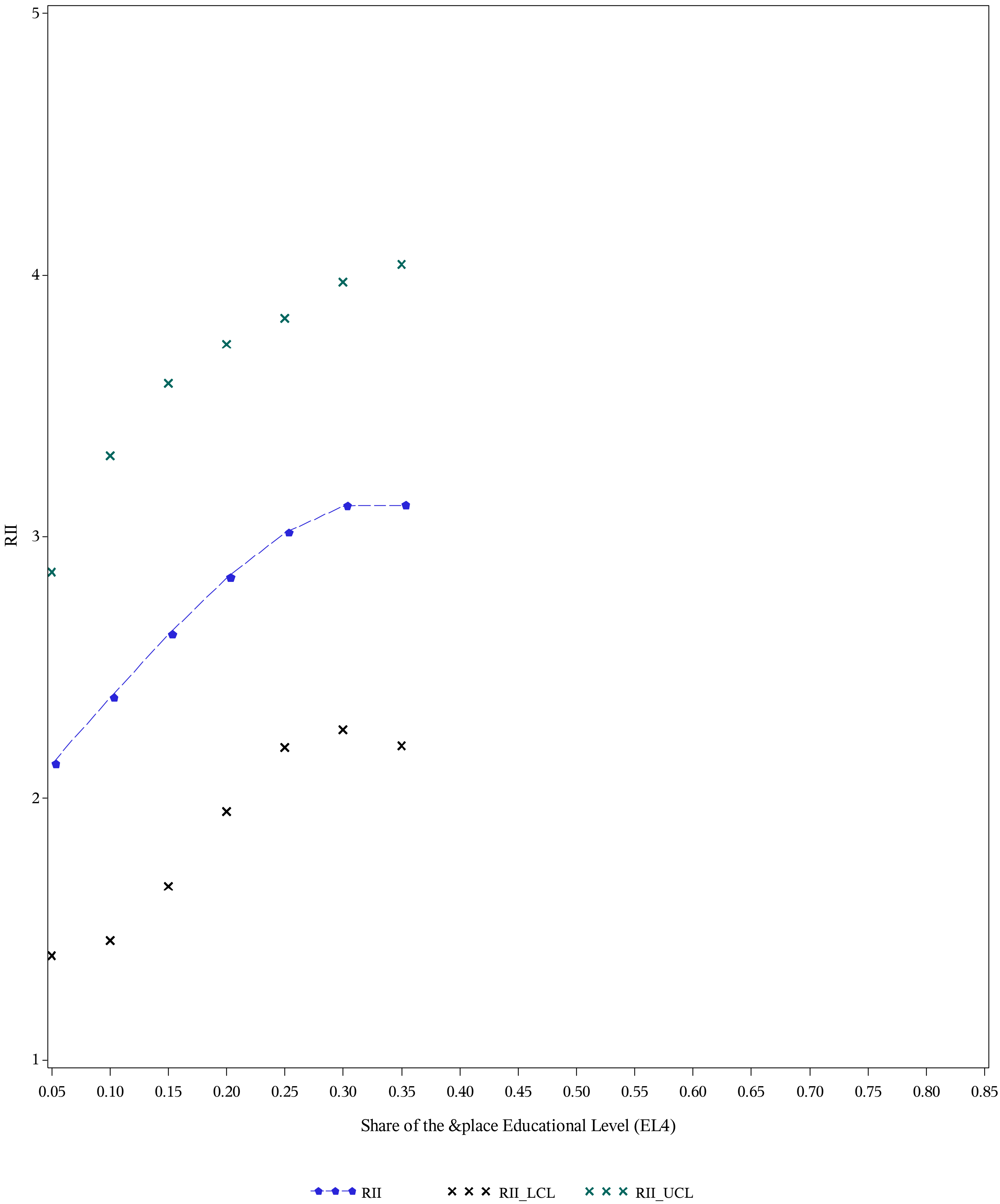

RII in function of the share of EL4

When EL2 and EL3 are fixed at: EL2=55% ; EL3=10%
EL1 =1- EL4 - EL2 - EL3

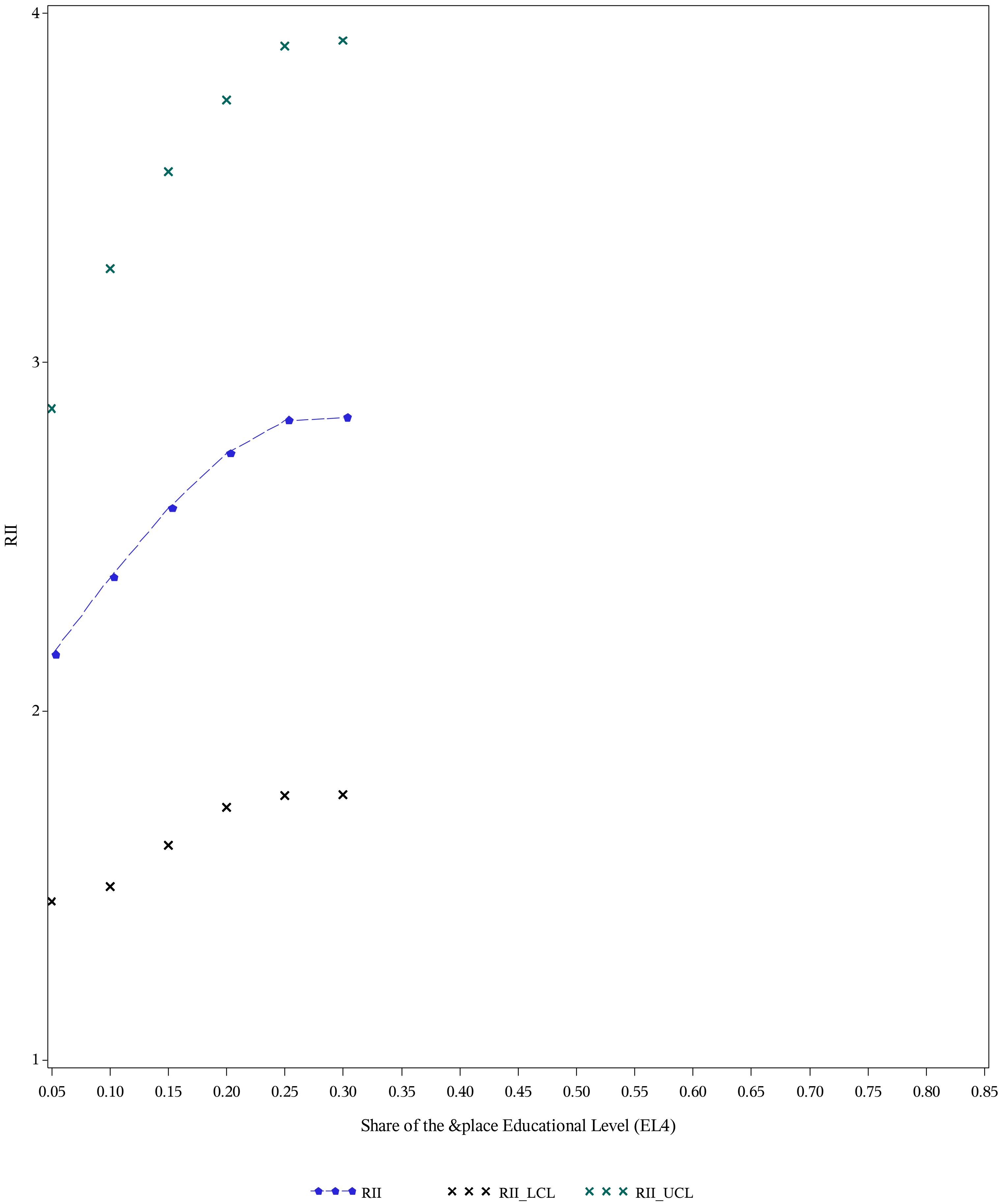

RII in function of the share of EL4

When EL2 and EL3 are fixed at: EL2=55% ; EL3=15%
EL1 =1- EL4 - EL2 - EL3

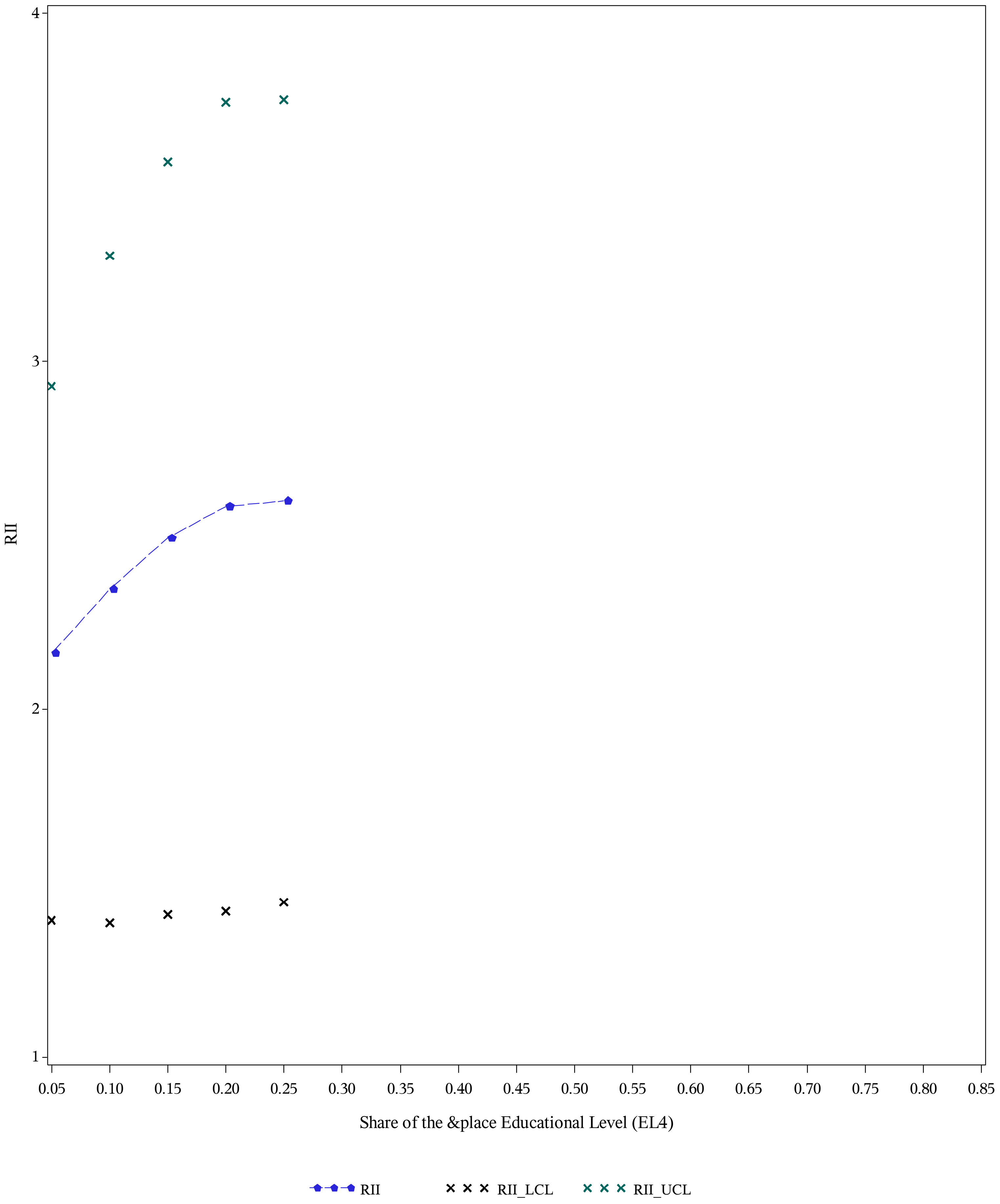

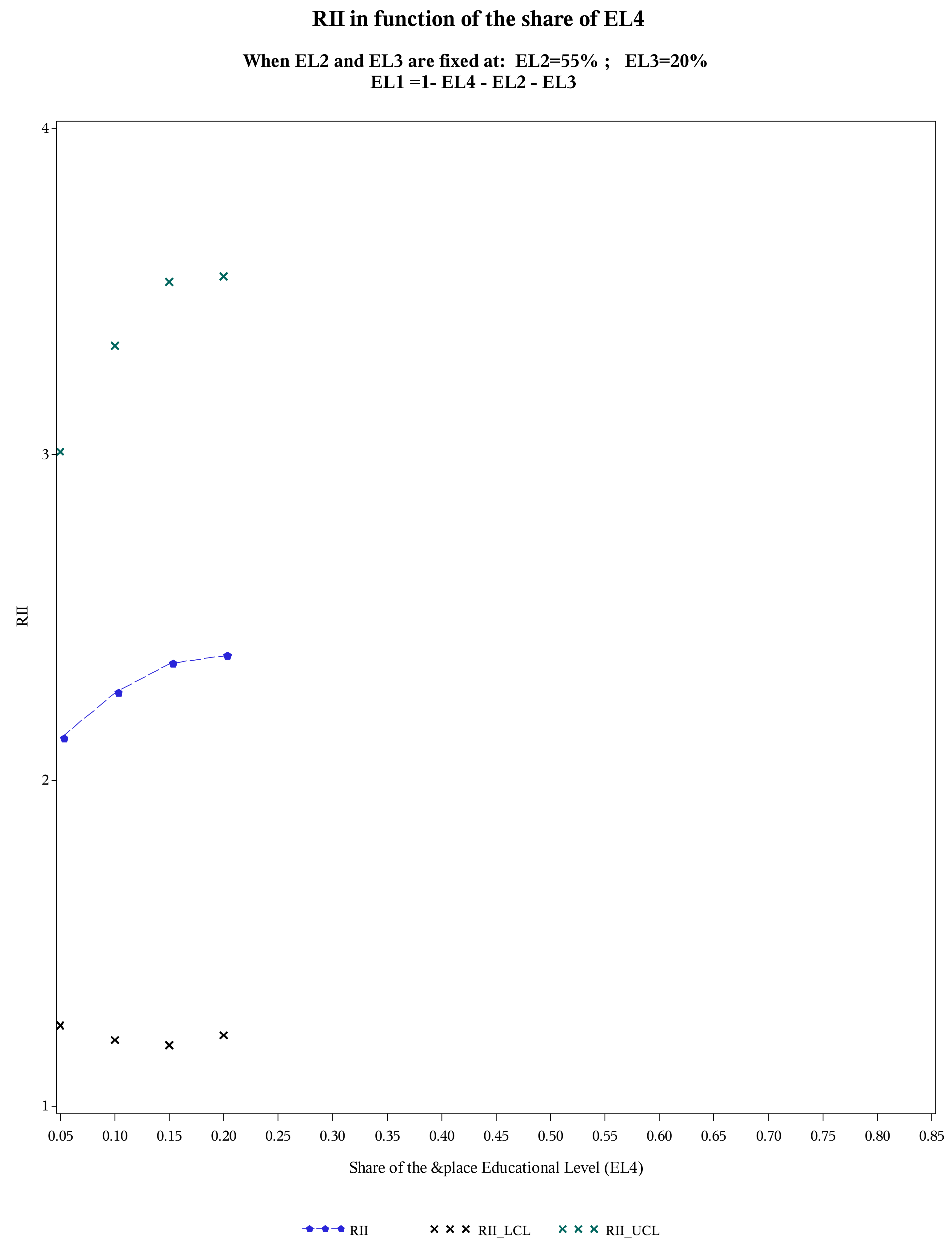
RII in function of the share of EL4
When EL2 and EL3 are fixed at: EL2=55% ; EL3=20%
EL1 =1- EL4 - EL2 - EL3
RII
4
3
2
1
0.05
0.10
0.15
0.20
0.25
0.30
0.35
0.40
0.45
0.50
0.55
0.60
0.65
0.70
0.75
0.80
0.85
Share of the &place Educational Level (EL4)
RII
RII_LCL
RII_UCL

RII in function of the share of EL4

When EL2 and EL3 are fixed at: EL2=55% ; EL3=25%
EL1 =1- EL4 - EL2 - EL3

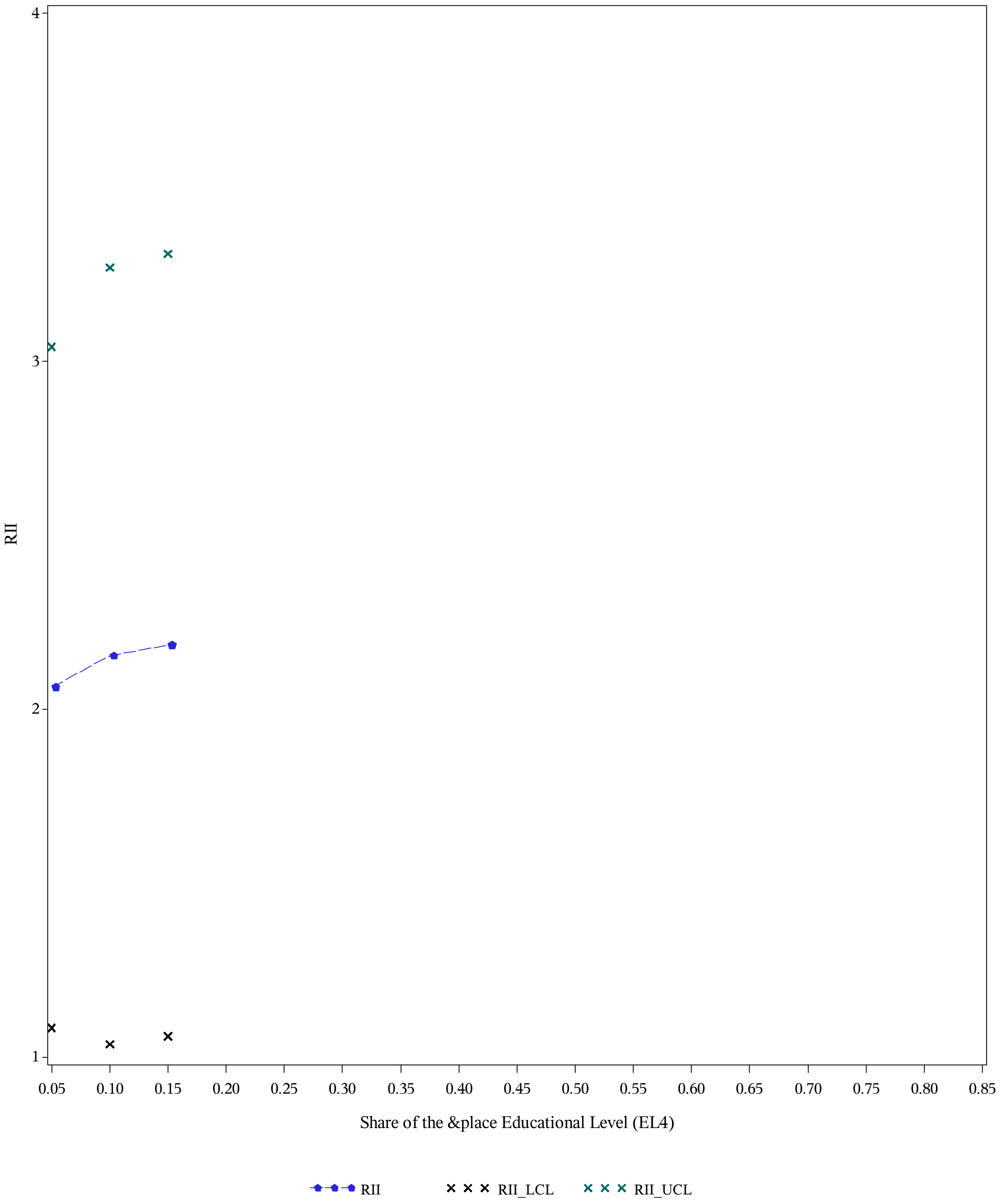

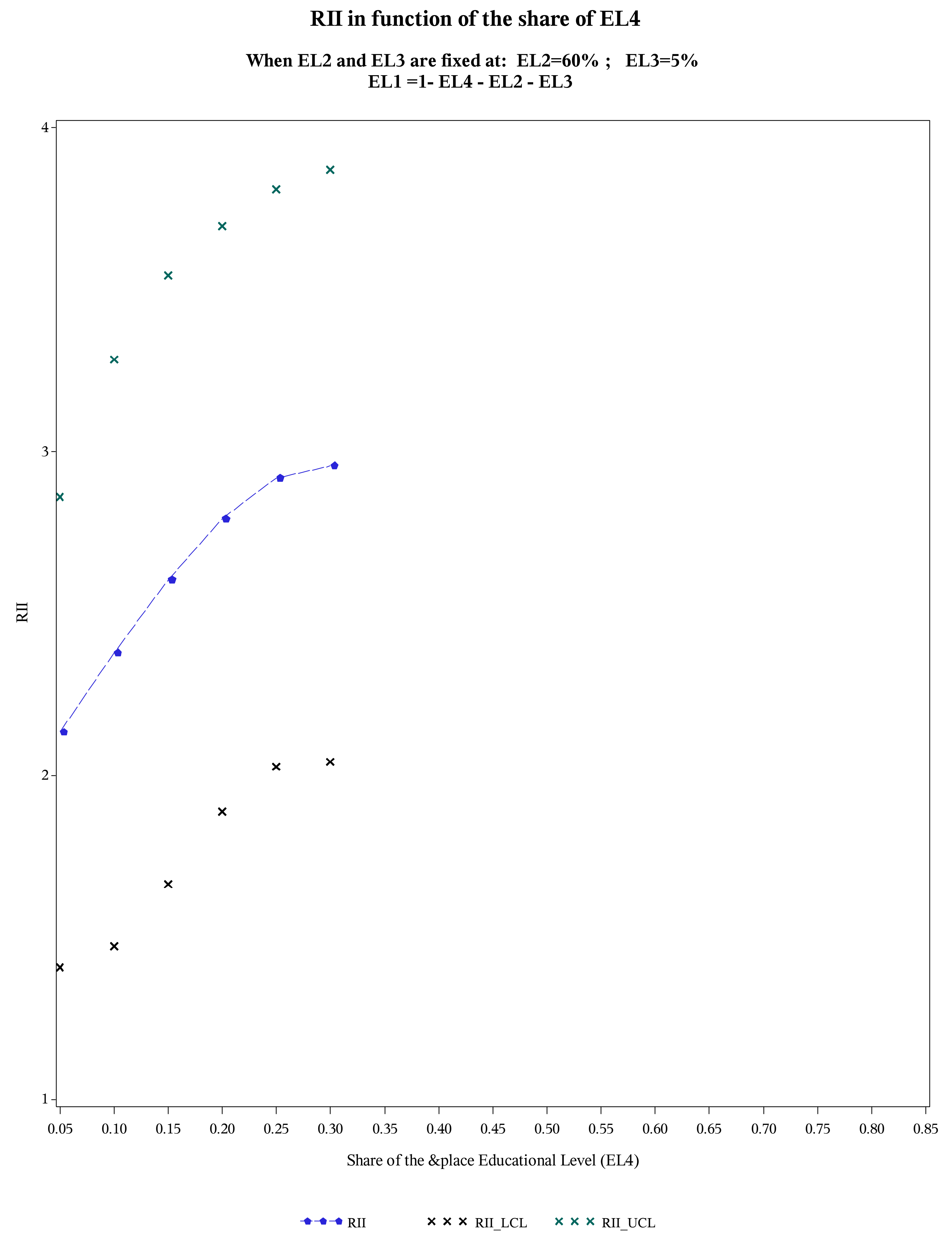
RII in function of the share of EL4
When EL2 and EL3 are fixed at: EL2=60% ; EL3=5%
EL1 =1- EL4 - EL2 - EL3
RII
1
2
3
4
0.05
0.10
0.15
0.20
0.25
0.30
0.35
0.40
0.45
0.50
0.55
0.60
0.65
0.70
0.75
0.80
0.85
Share of the &place Educational Level (EL4)
RII
RII_LCL
RII_UCL

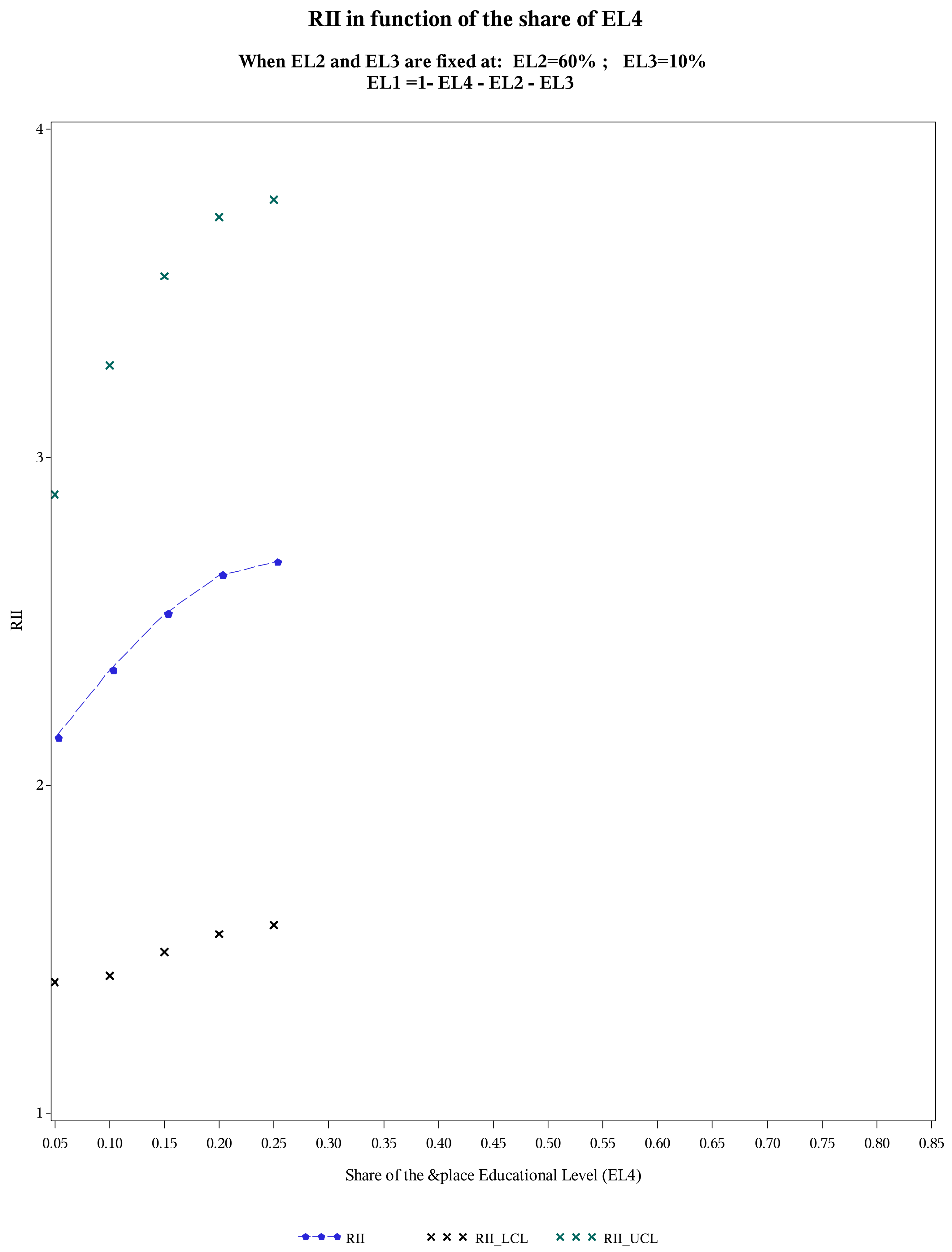
RII in function of the share of EL4
When EL2 and EL3 are fixed at: EL2=60% ; EL3=10%
EL1 =1- EL4 - EL2 - EL3
RII
4
3
2
1
0.05
0.10
0.15
0.20
0.25
0.30
0.35
0.40
0.45
0.50
0.55
0.60
0.65
0.70
0.75
0.80
0.85
Share of the &place Educational Level (EL4)
RII
RII_LCL
RII_UCL

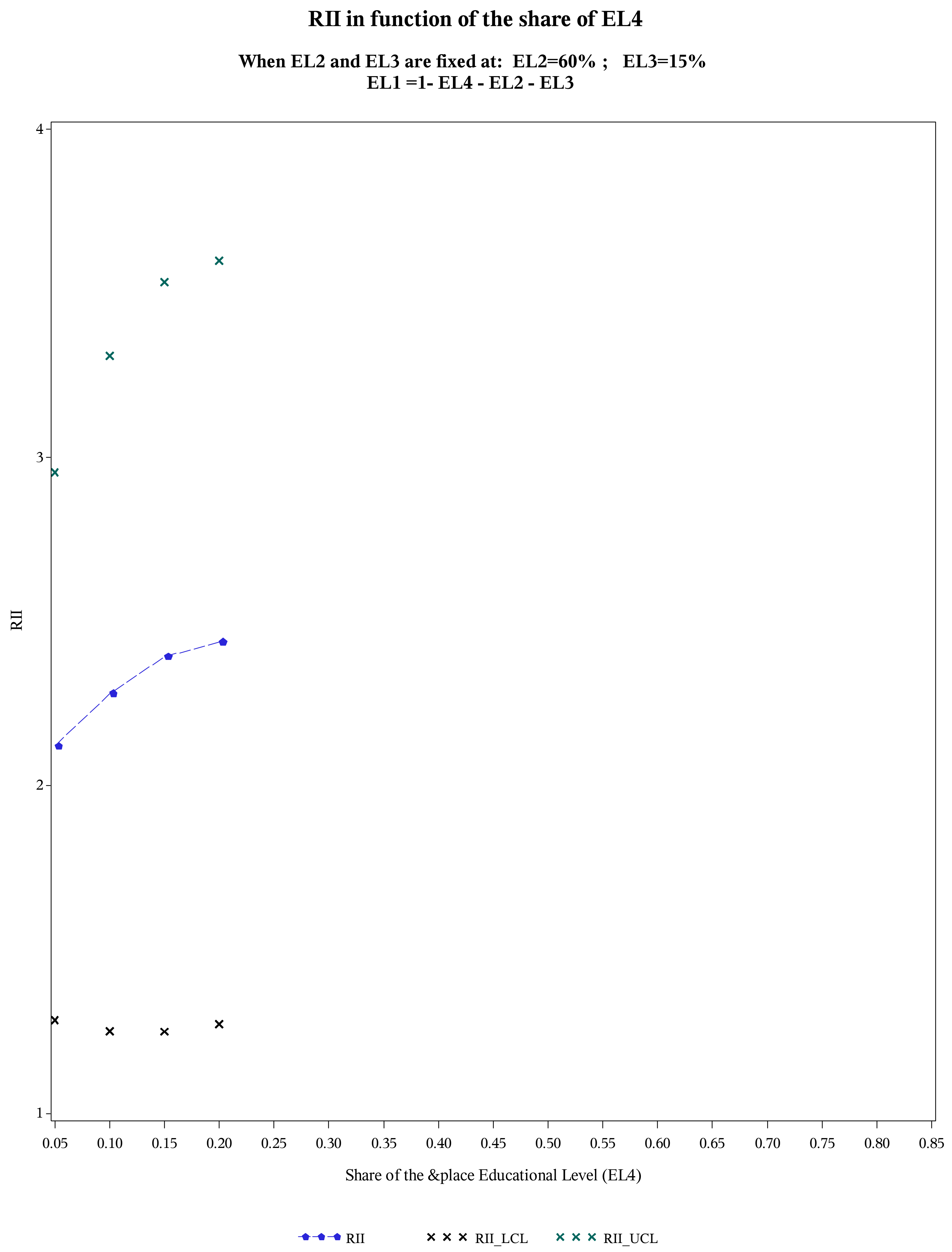

RII in function of the share of EL4
When EL2 and EL3 are fixed at: EL2=60% ; EL3=15%
EL1 =1- EL4 - EL2 - EL3
RII
4
3
2
1
0.05
0.10
0.15
0.20
0.25
0.30
0.35
0.40
0.45
0.50
0.55
0.60
0.65
0.70
0.75
0.80
0.85
Share of the &place Educational Level (EL4)
RII
RII_LCL
RII_UCL

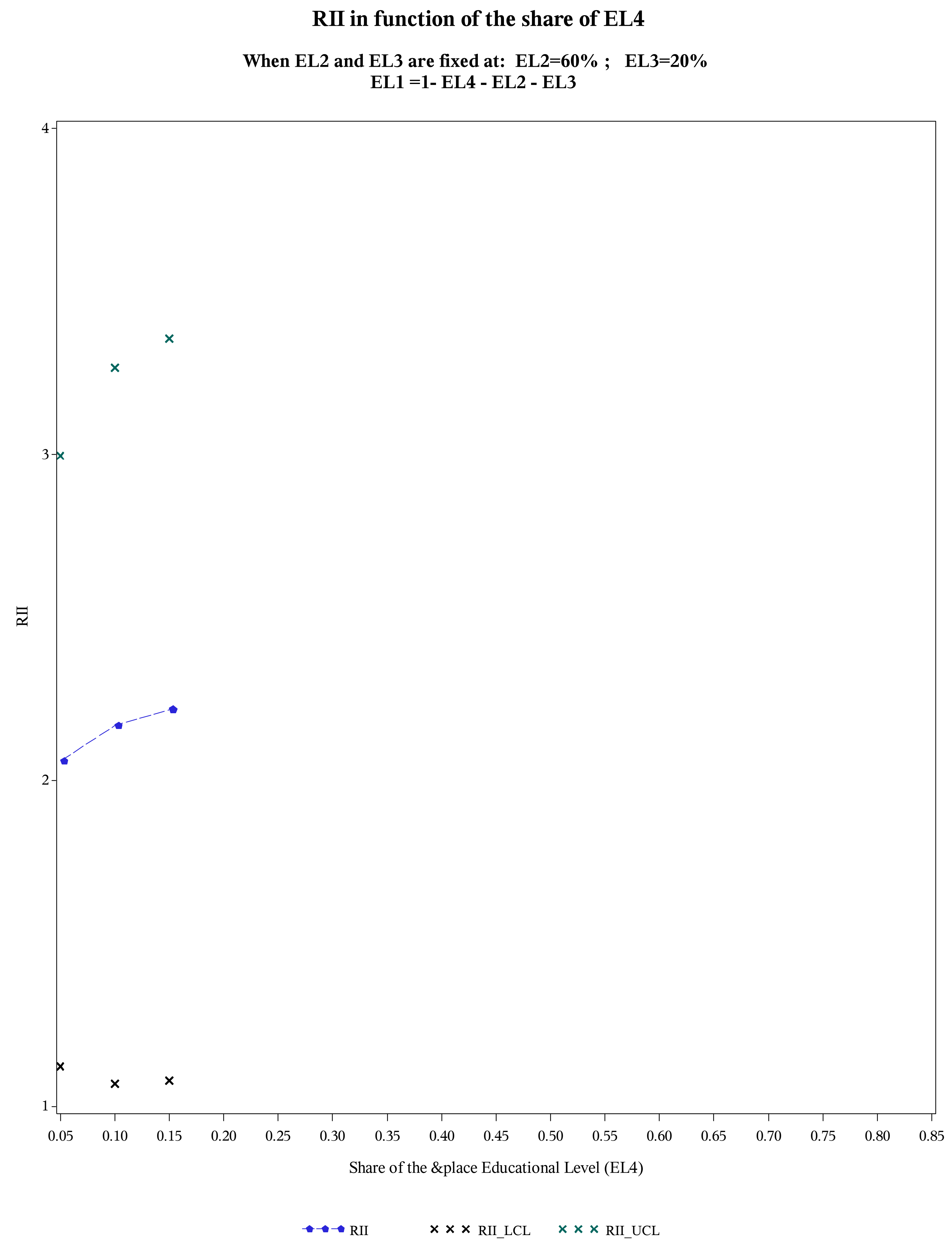

RII in function of the share of EL4
When EL2 and EL3 are fixed at: EL2=60% ; EL3=20%
EL1 =1- EL4 - EL2 - EL3
RII
4
3
2
1
0.05
0.10
0.15
0.20
0.25
0.30
0.35
0.40
0.45
0.50
0.55
0.60
0.65
0.70
0.75
0.80
0.85
Share of the &place Educational Level (EL4)
RII
RII_LCL
RII_UCL

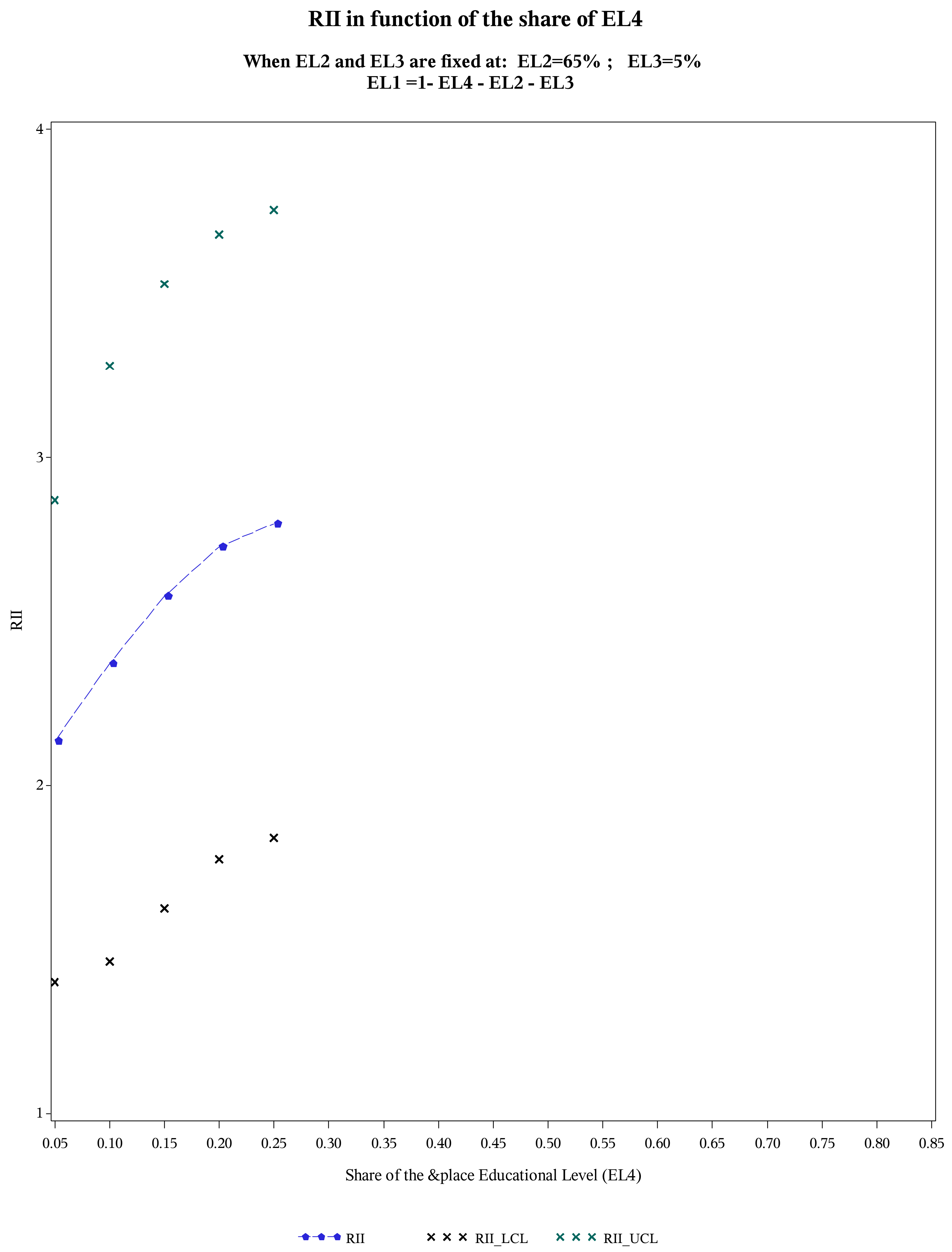
RII in function of the share of EL4
When EL2 and EL3 are fixed at: EL2=65% ; EL3=5%
EL1 =1- EL4 - EL2 - EL3
RII
1
2
3
4
0.05
0.10
0.15
0.20
0.25
0.30
0.35
0.40
0.45
0.50
0.55
0.60
0.65
0.70
0.75
0.80
0.85
Share of the &place Educational Level (EL4)
RII
RII_LCL
RII_UCL

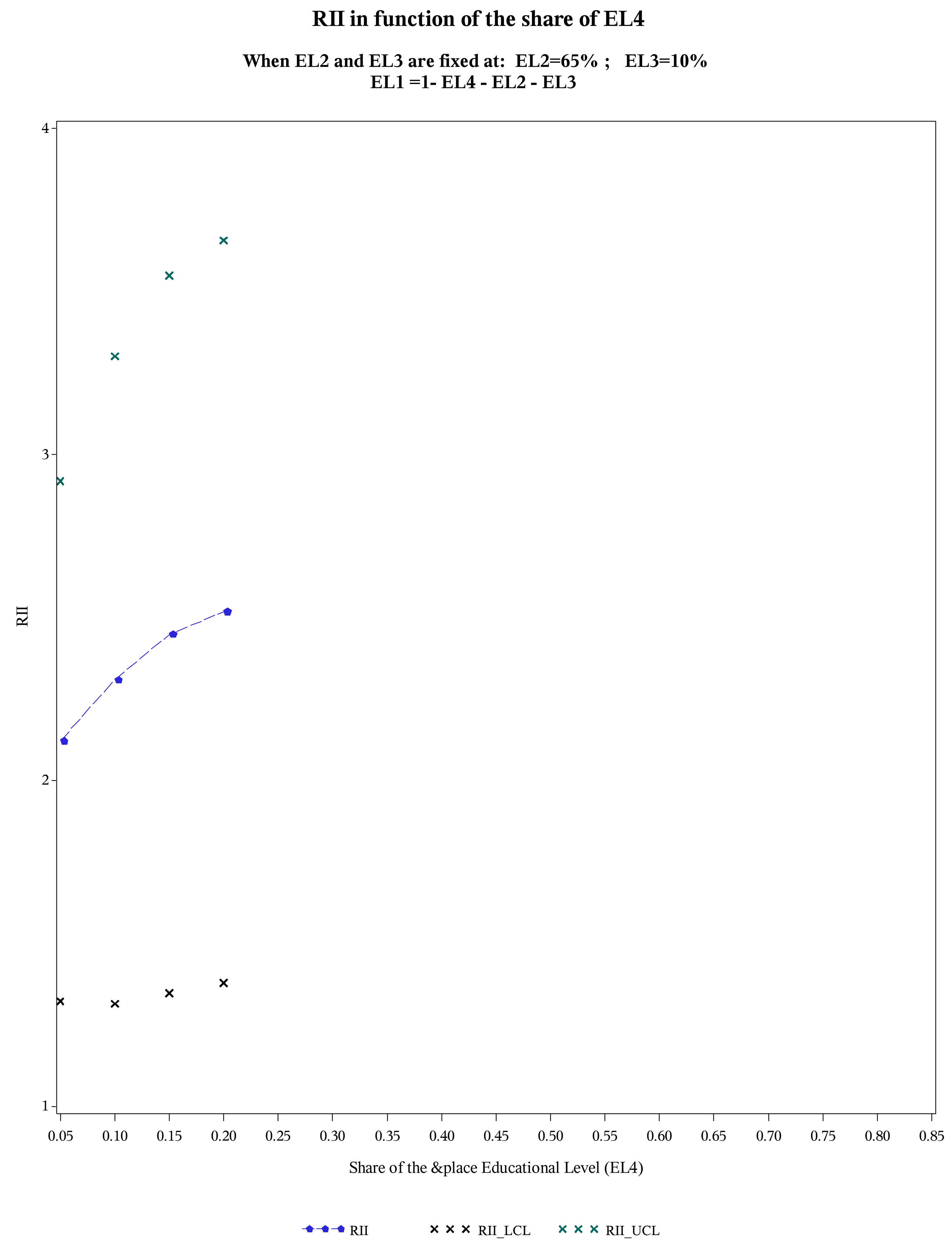

RII in function of the share of EL4
When EL2 and EL3 are fixed at: EL2=65% ; EL3=10%
EL1 =1- EL4 - EL2 - EL3
RII
1
2
3
4
0.05
0.10
0.15
0.20
0.25
0.30
0.35
0.40
0.45
0.50
0.55
0.60
0.65
0.70
0.75
0.80
0.85
Share of the &place Educational Level (EL4)
RII
RII_LCL
RII_UCL

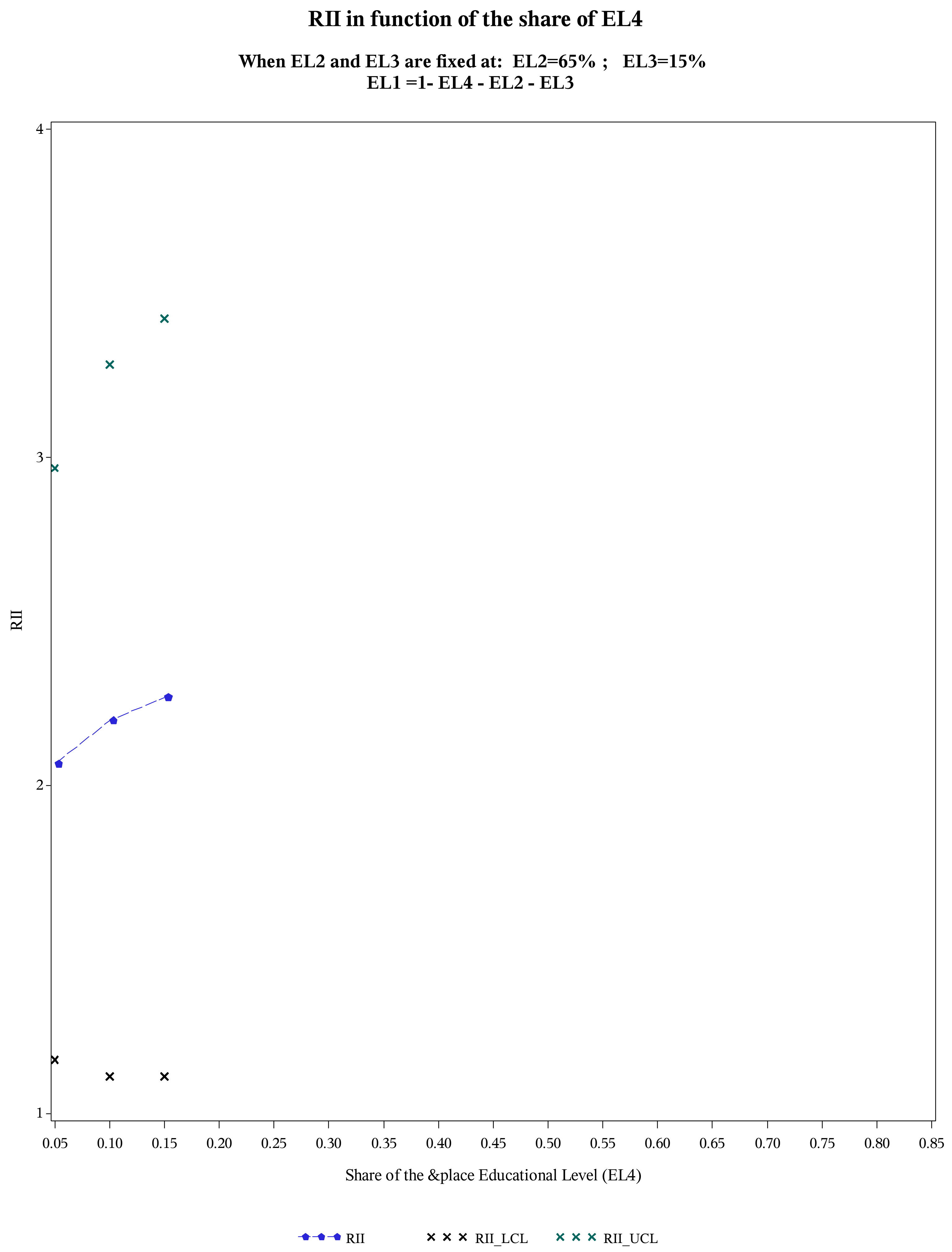

RII in function of the share of EL4
When EL2 and EL3 are fixed at: EL2=65% ; EL3=15%
EL1 =1- EL4 - EL2 - EL3
RII
1
2
3
4
0.05
0.10
0.15
0.20
0.25
0.30
0.35
0.40
0.45
0.50
0.55
0.60
0.65
0.70
0.75
0.80
0.85
Share of the &place Educational Level (EL4)
RII
RII_LCL
RII_UCL

RII in function of the share of EL4

When EL2 and EL3 are fixed at: EL2=65% ; EL3=20%
EL1 =1- EL4 - EL2 - EL3

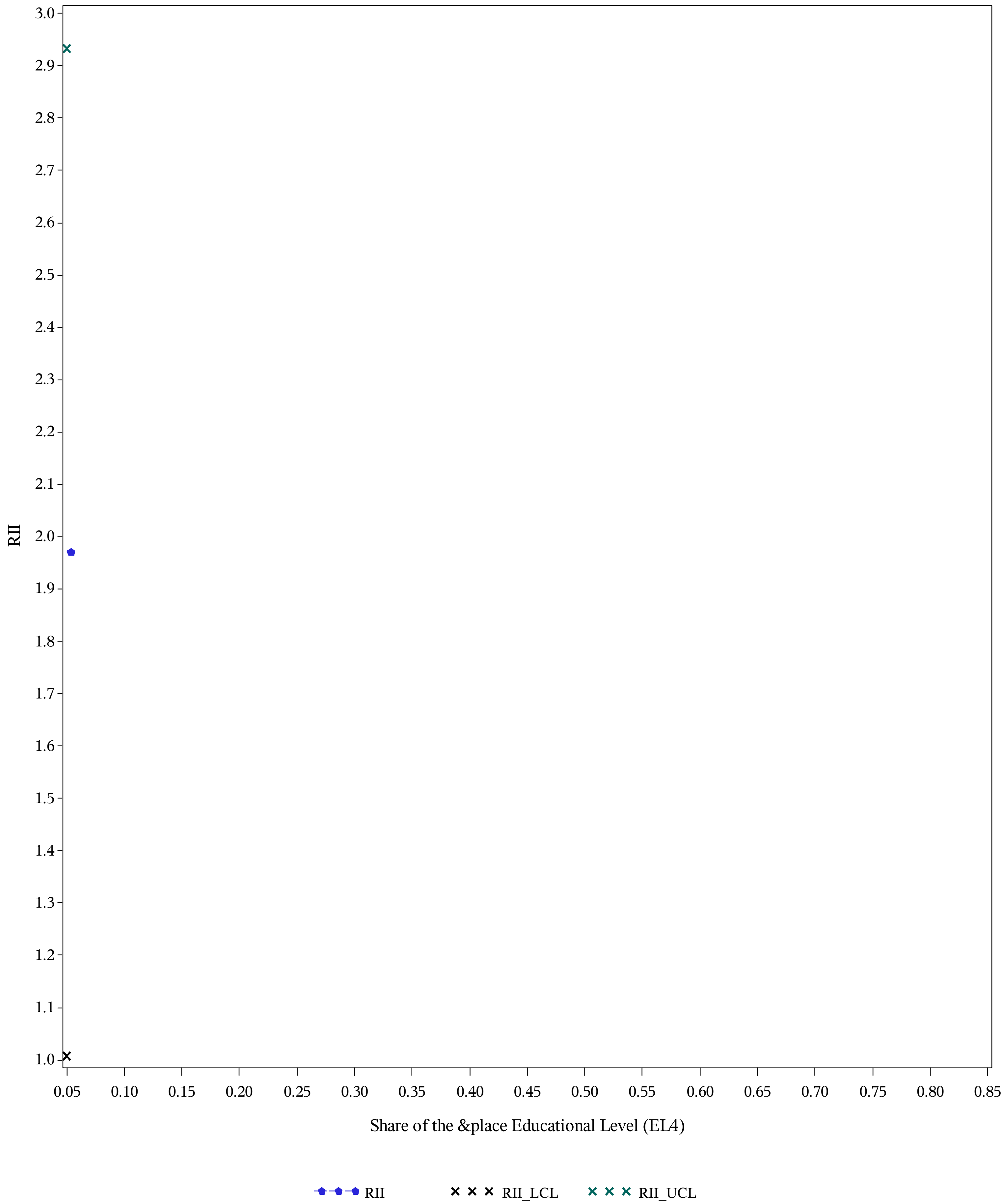

RII in function of the share of EL4

When EL2 and EL3 are fixed at: EL2=70% ; EL3=5%
EL1 =1- EL4 - EL2 - EL3

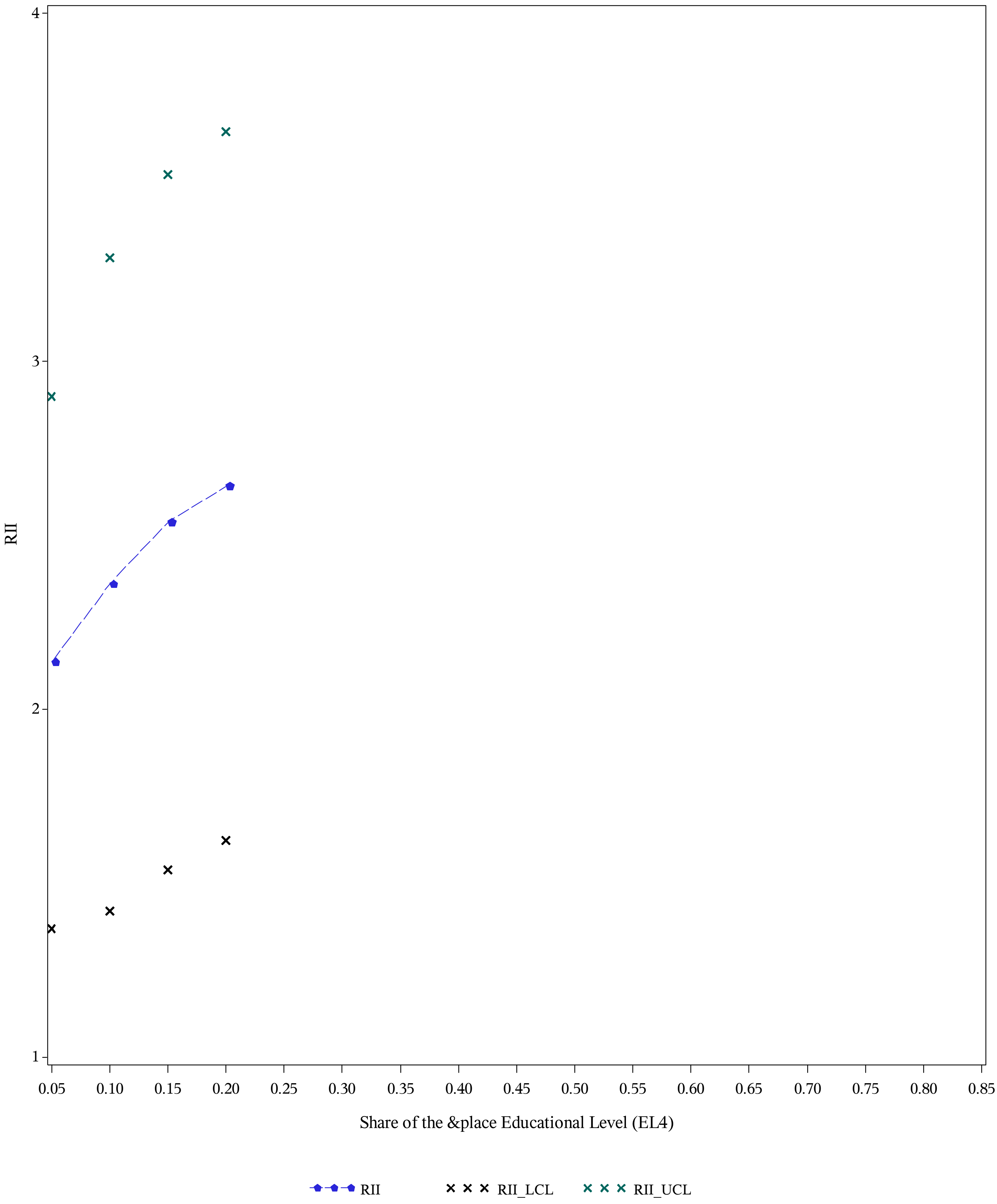

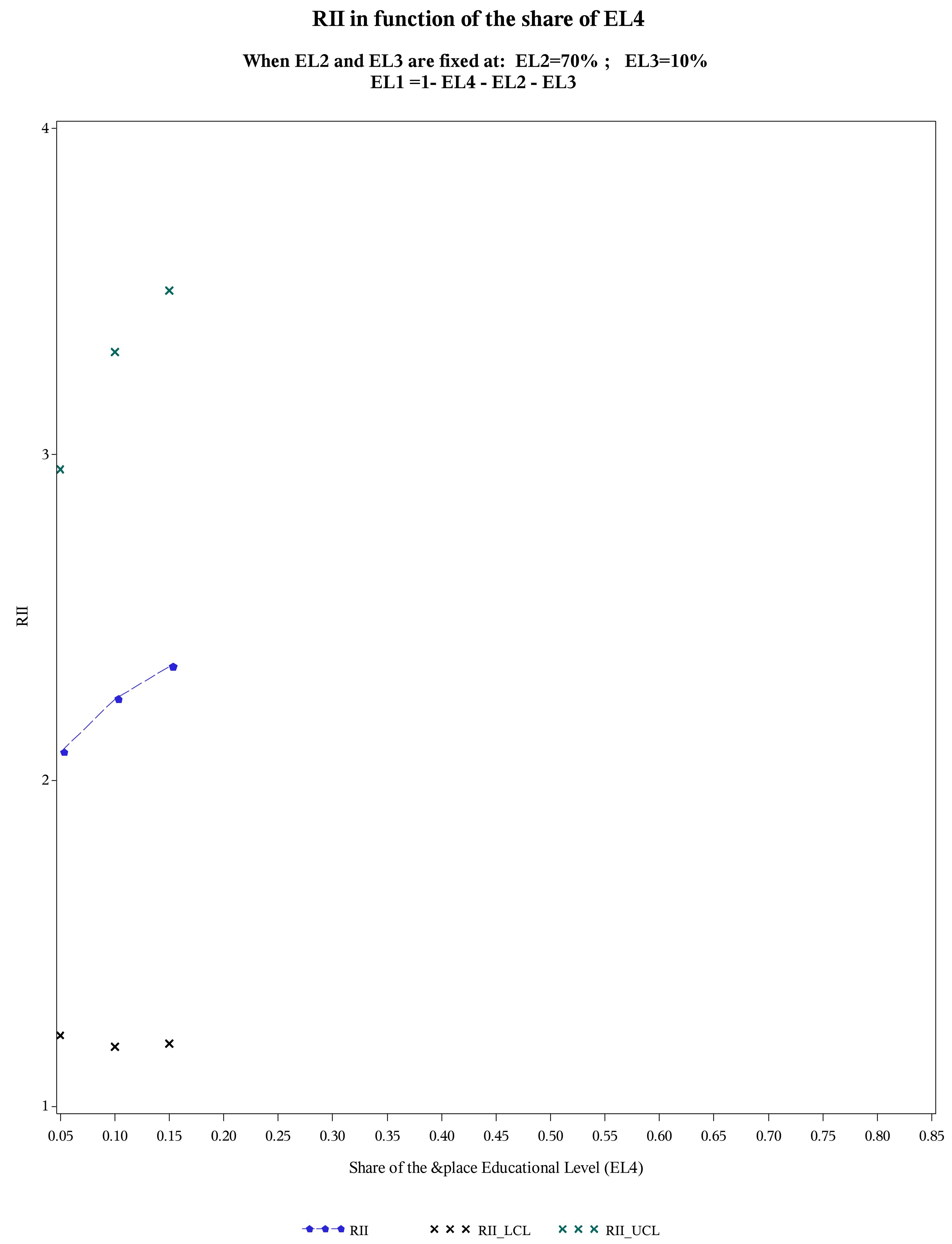
RII in function of the share of EL4
When EL2 and EL3 are fixed at: EL2=70% ; EL3=10%
EL1 =1- EL4 - EL2 - EL3
RII
1
2
3
4
0.05
0.10
0.15
0.20
0.25
0.30
0.35
0.40
0.45
0.50
0.55
0.60
0.65
0.70
0.75
0.80
0.85
Share of the &place Educational Level (EL4)
RII
RII_LCL
RII_UCL

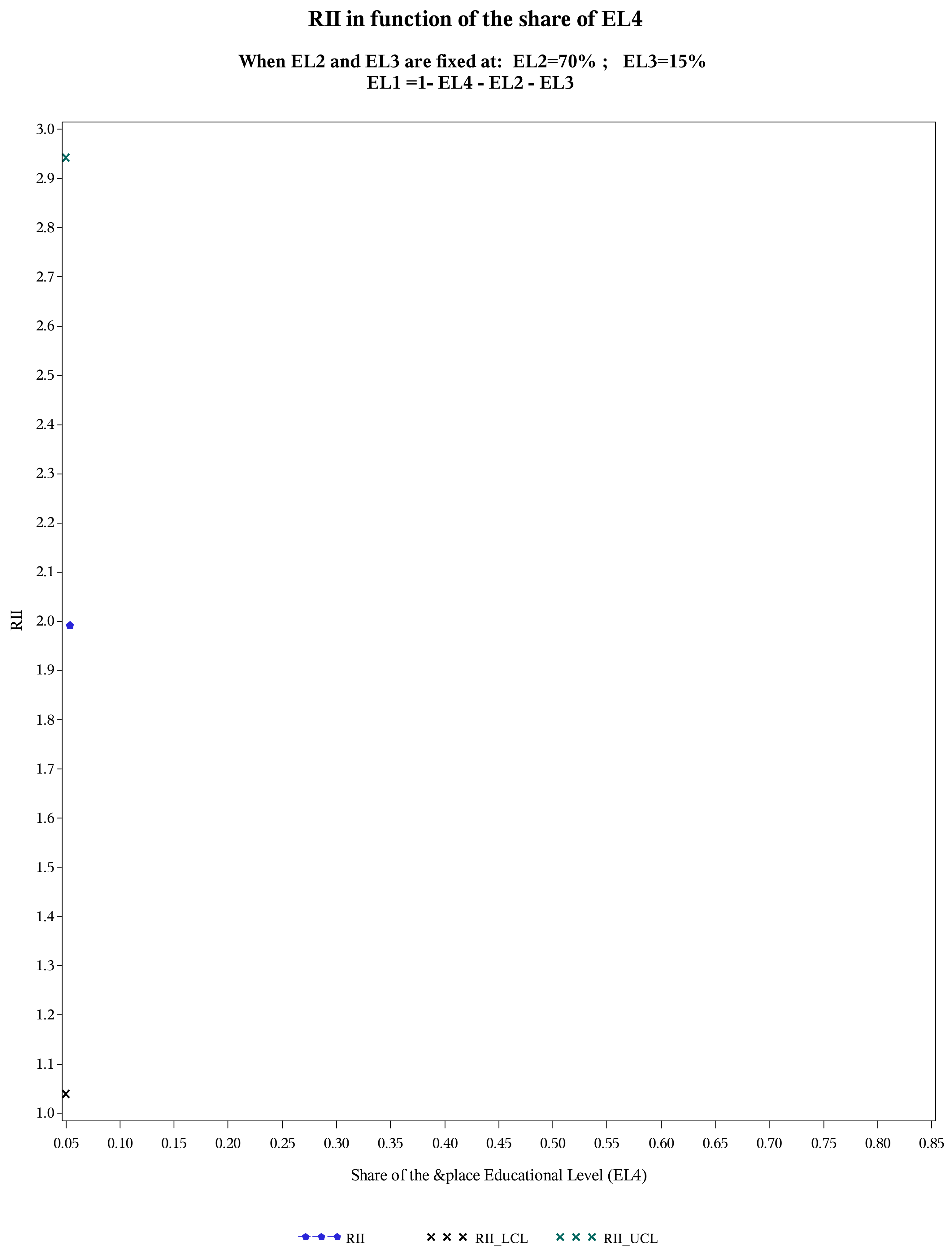
RII in function of the share of EL4
When EL2 and EL3 are fixed at: EL2=70% ; EL3=15%
EL1 =1- EL4 - EL2 - EL3
RII
3.0
2.9
2.8
2.7
2.6
2.5
2.4
2.3
2.2
2.1
2.0
1.9
1.8
1.7
1.6
1.5
1.4
1.3
1.2
1.1
1.0
0.05
0.10
0.15
0.20
0.25
0.30
0.35
0.40
0.45
0.50
0.55
0.60
0.65
0.70
0.75
0.80
0.85
Share of the &place Educational Level (EL4)
RII
RII_LCL
RII_UCL

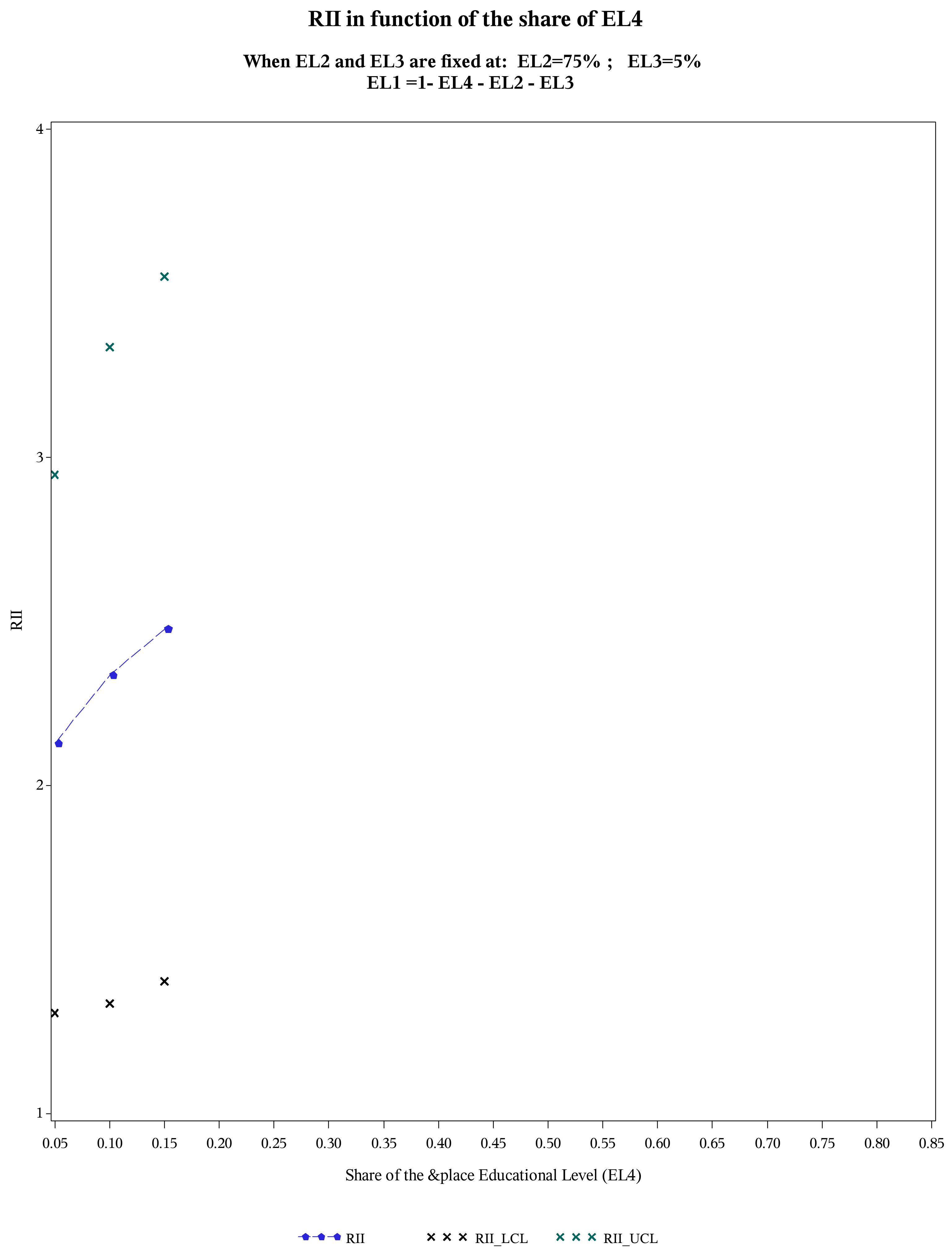
RII in function of the share of EL4
When EL2 and EL3 are fixed at: EL2=75% ; EL3=5%
EL1 =1- EL4 - EL2 - EL3
RII
1
2
3
4
0.05
0.10
0.15
0.20
0.25
0.30
0.35
0.40
0.45
0.50
0.55
0.60
0.65
0.70
0.75
0.80
0.85
Share of the &place Educational Level (EL4)
RII
RII_LCL
RII_UCL

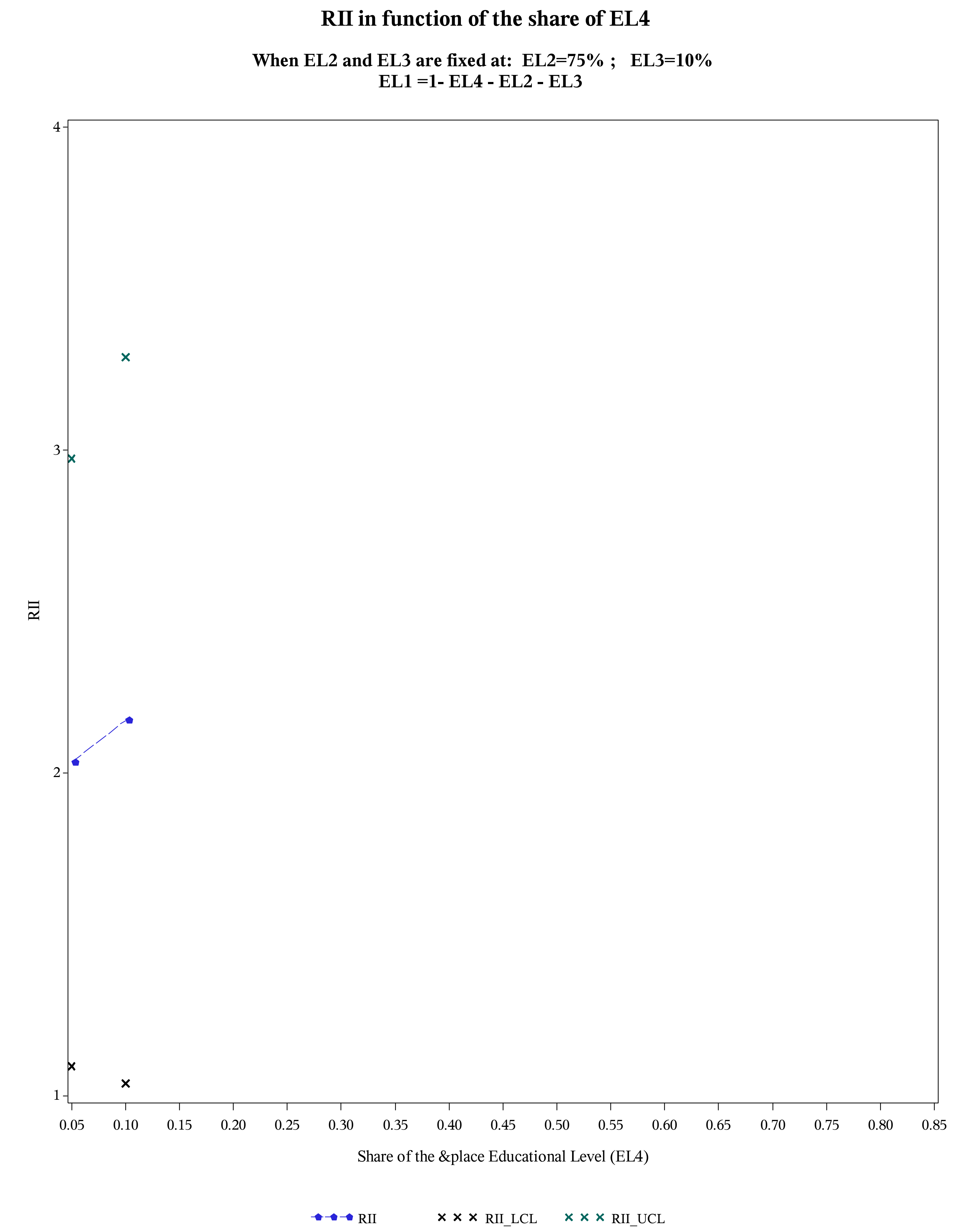

RII in function of the share of EL4
When EL2 and EL3 are fixed at: EL2=75% ; EL3=10%
EL1 =1- EL4 - EL2 - EL3
RII
1
2
3
4
0.05
0.10
0.15
0.20
0.25
0.30
0.35
0.40
0.45
0.50
0.55
0.60
0.65
0.70
0.75
0.80
0.85
Share of the &place Educational Level (EL4)
RII
RII_LCL
RII_UCL

RII in function of the share of EL4

When EL2 and EL3 are fixed at: EL2=80% ; EL3=5%
EL1 =1- EL4 - EL2 - EL3

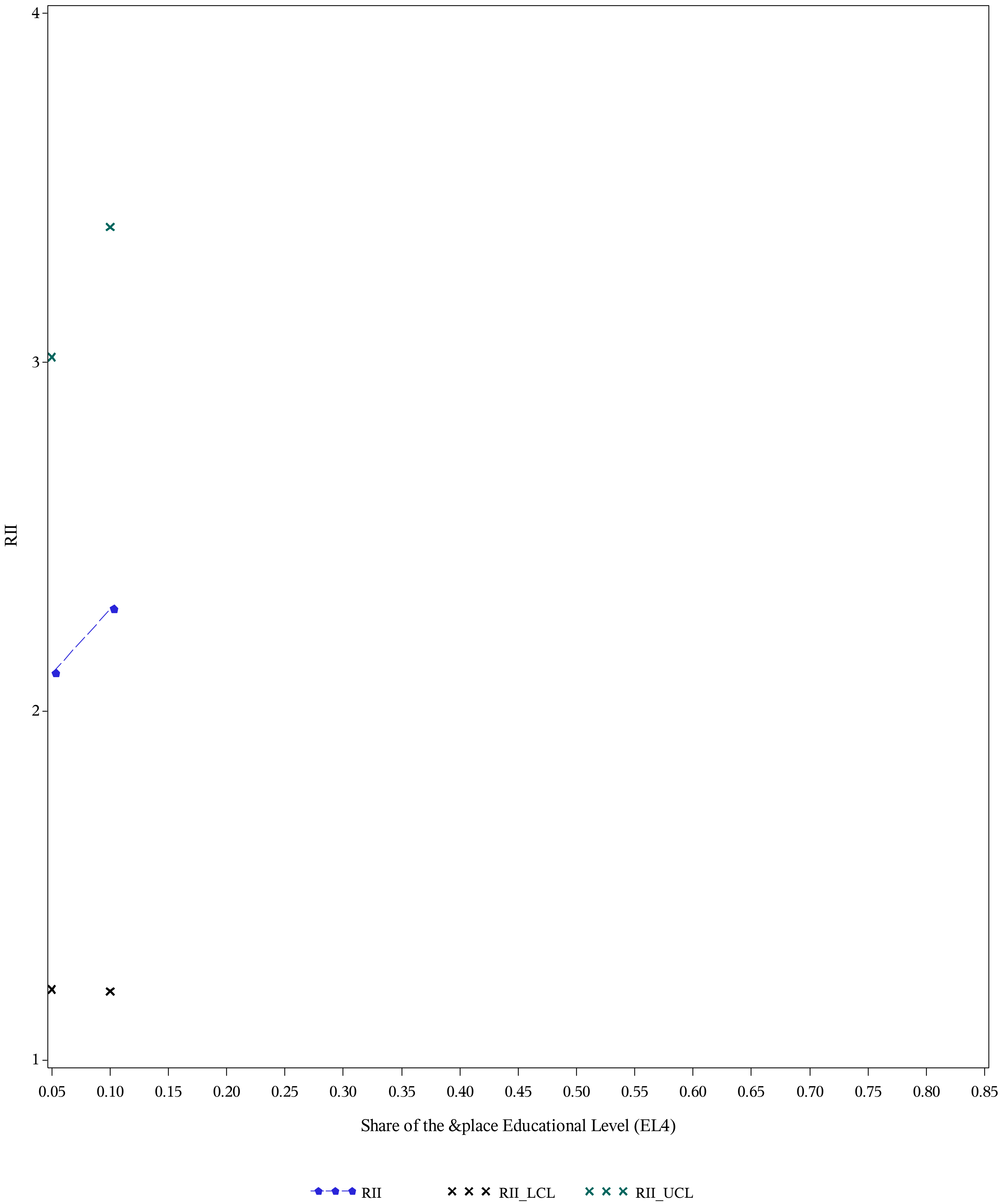

Supplement: Supplementary file 8 — Full set of figures representing the evolution of the RII in function of P4 at fixed p2 and p3 (PDF 517 kb) [file 12889_2019_6980_MOESM8_ESM.pdf]
