# Supplementary material for: Monitoring health inequalities when the socio-economic composition changes: are the slope and relative indices of inequality appropriate? Results of a simulation study
Source: BMC Public Health. 2019 May 30;19:662. doi: 10.1186/s12889-019-6980-1 (PMC6543610; doi:10.1186/s12889-019-6980-1)

PAF in function of the share of EL4

When EL2 and EL3 are fixed at: EL2=5% ; EL3=15%
EL1 =1- EL4 - EL2 - EL3

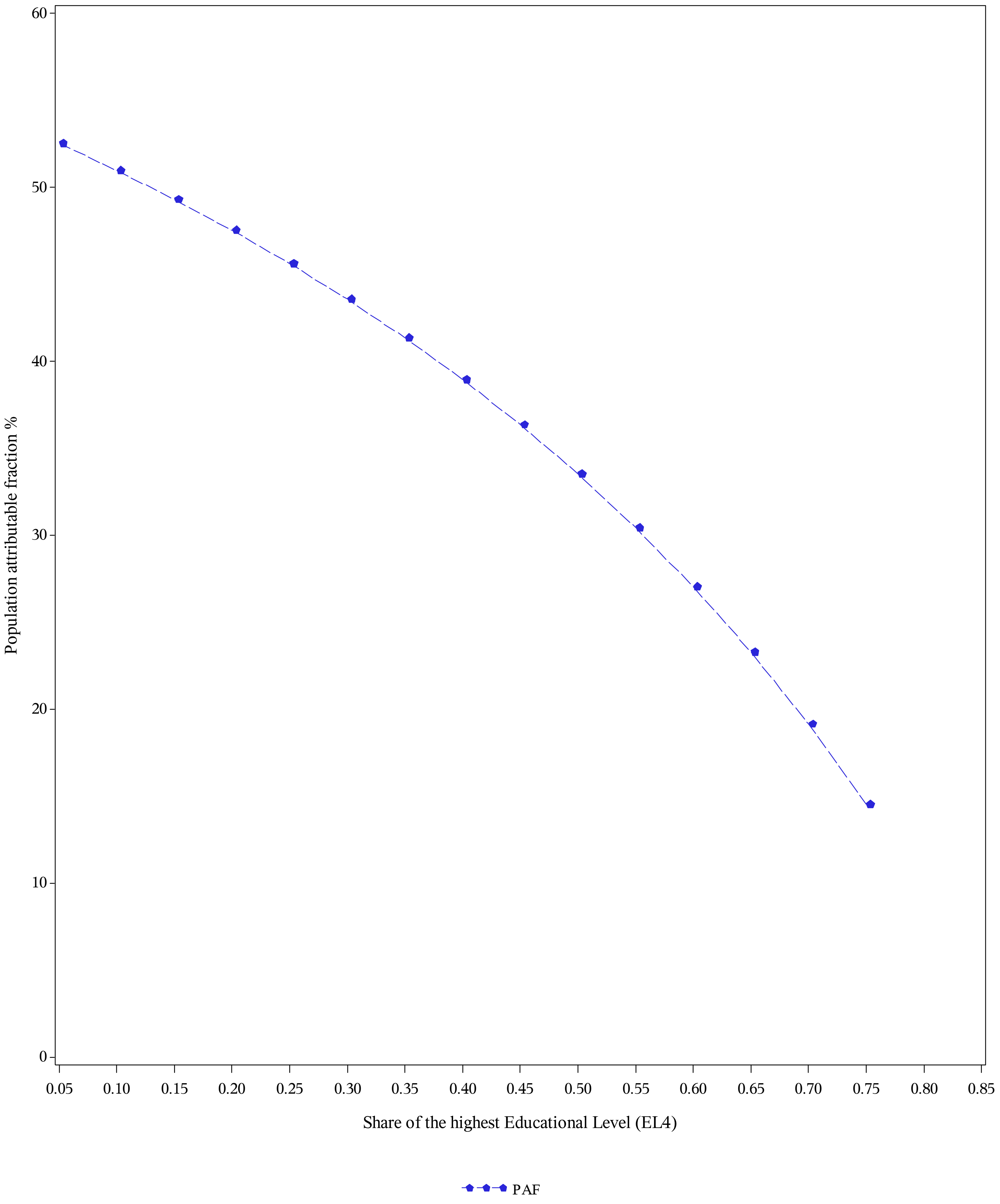

PAF in function of the share of EL4

When EL2 and EL3 are fixed at: EL2=5% ; EL3=20%
EL1 =1- EL4 - EL2 - EL3

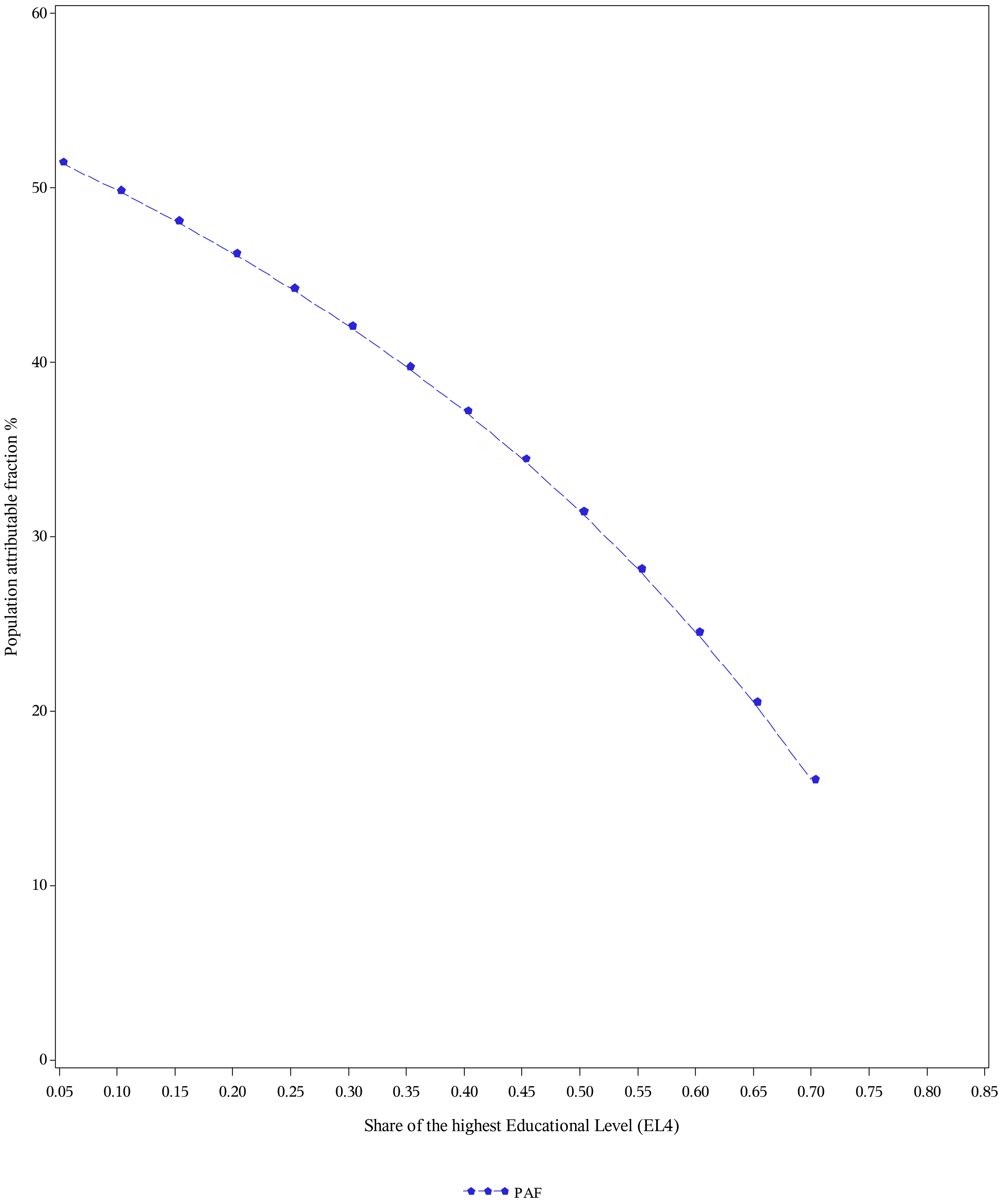

PAF in function of the share of EL4

When EL2 and EL3 are fixed at: EL2=5% ; EL3=25%
EL1 =1- EL4 - EL2 - EL3

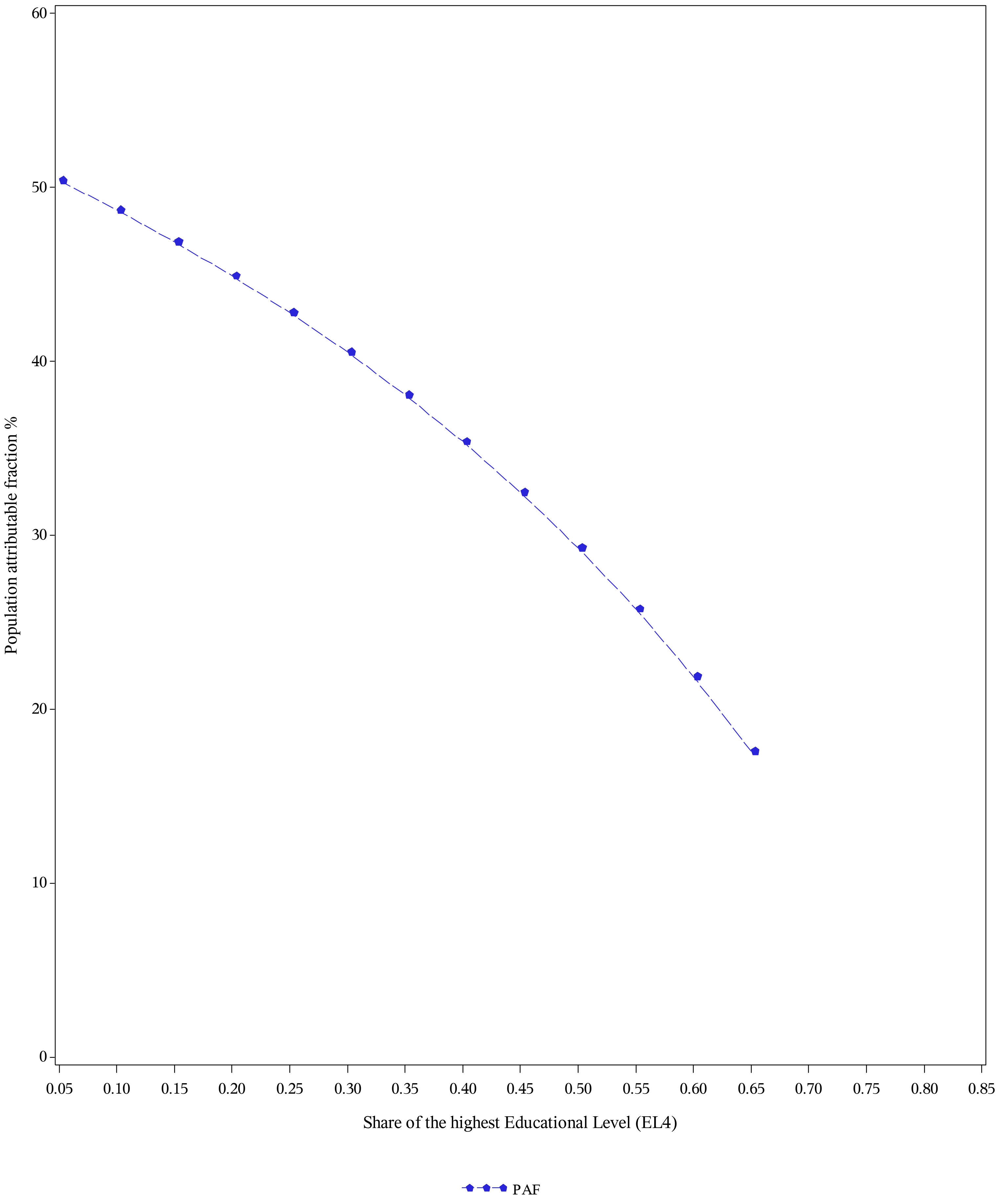

PAF in function of the share of EL4

When EL2 and EL3 are fixed at: EL2=5% ; EL3=30%
EL1 =1- EL4 - EL2 - EL3

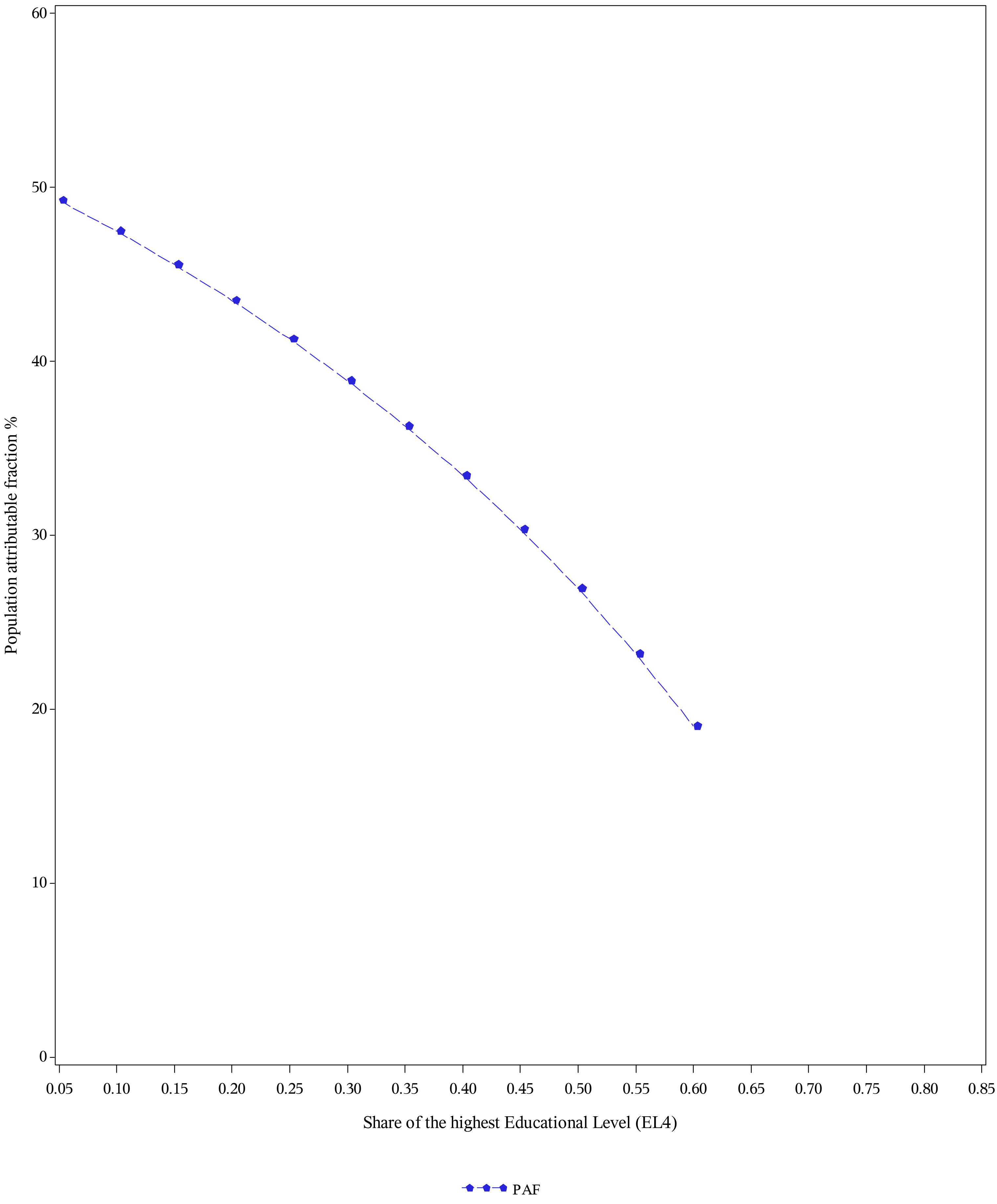

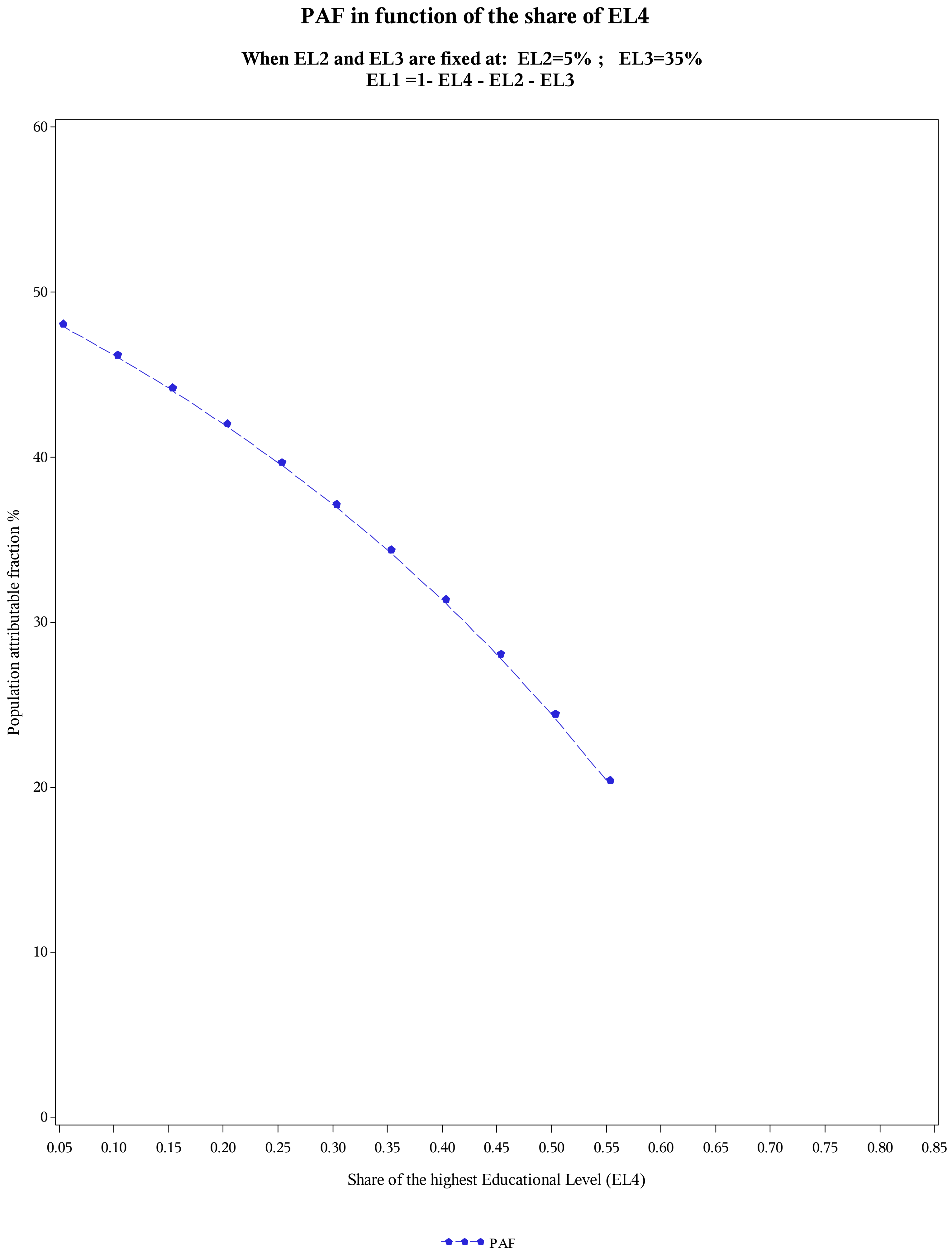

PAF in function of the share of EL4
When EL2 and EL3 are fixed at: EL2=5% ; EL3=35%
EL1 =1- EL4 - EL2 - EL3
Population attributable fraction %
0
10
20
30
40
50
60
0.05
0.10
0.15
0.20
0.25
0.30
0.35
0.40
0.45
0.50
0.55
0.60
0.65
0.70
0.75
0.80
0.85
Share of the highest Educational Level (EL4)
PAF

PAF in function of the share of EL4

When EL2 and EL3 are fixed at: EL2=5% ; EL3=40%
EL1 =1- EL4 - EL2 - EL3

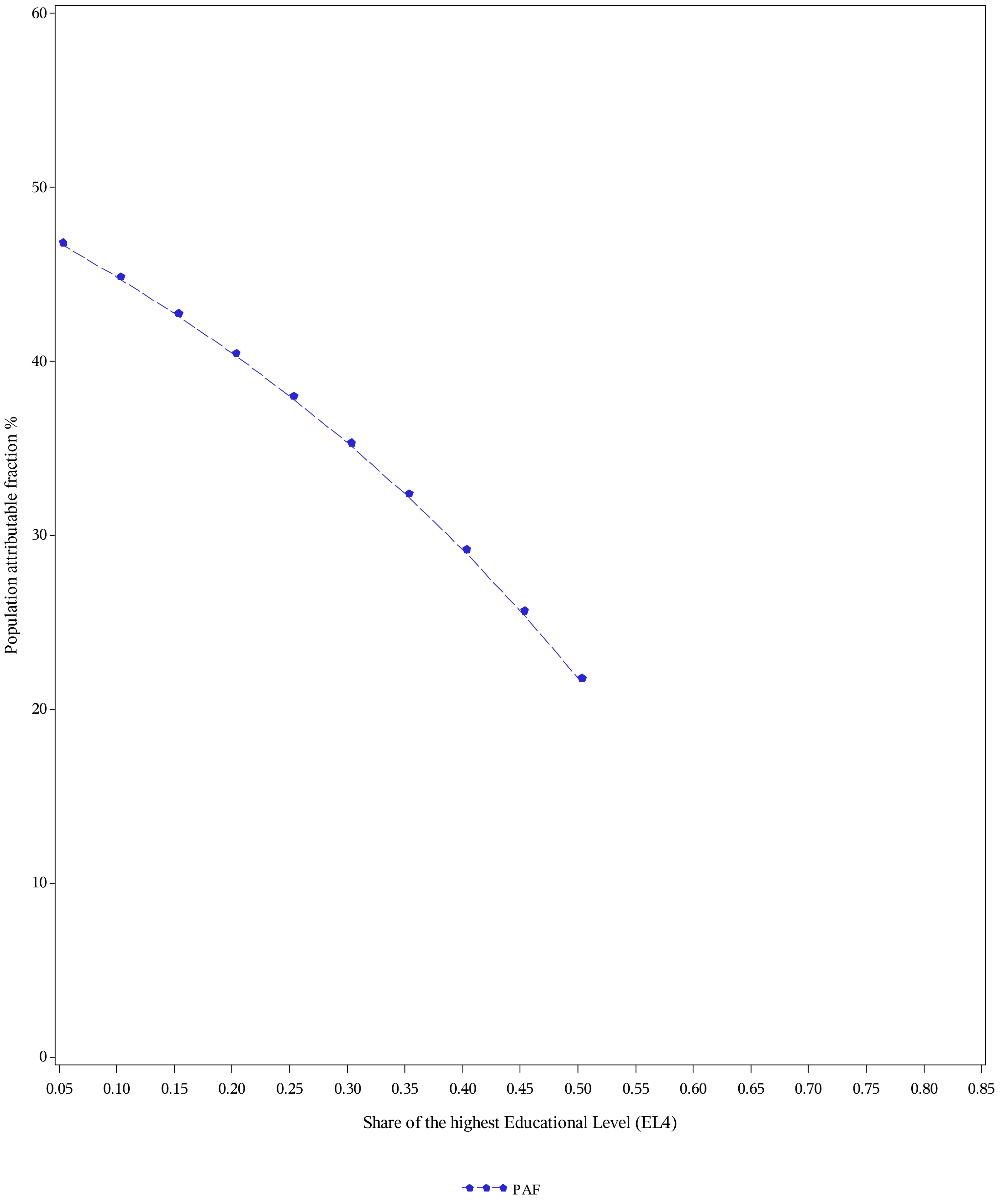

PAF in function of the share of EL4

When EL2 and EL3 are fixed at: EL2=5% ; EL3=45%
EL1 =1- EL4 - EL2 - EL3

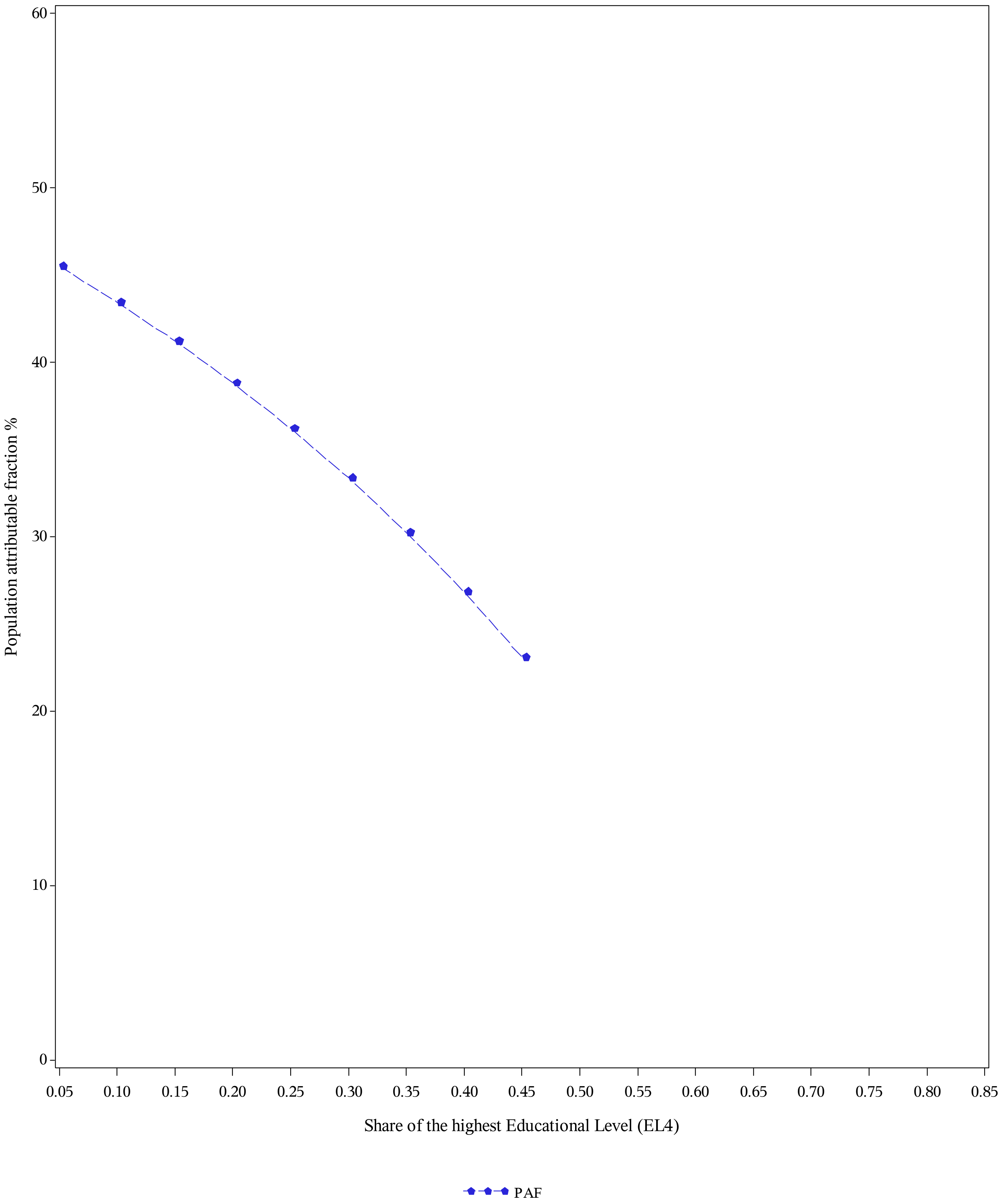

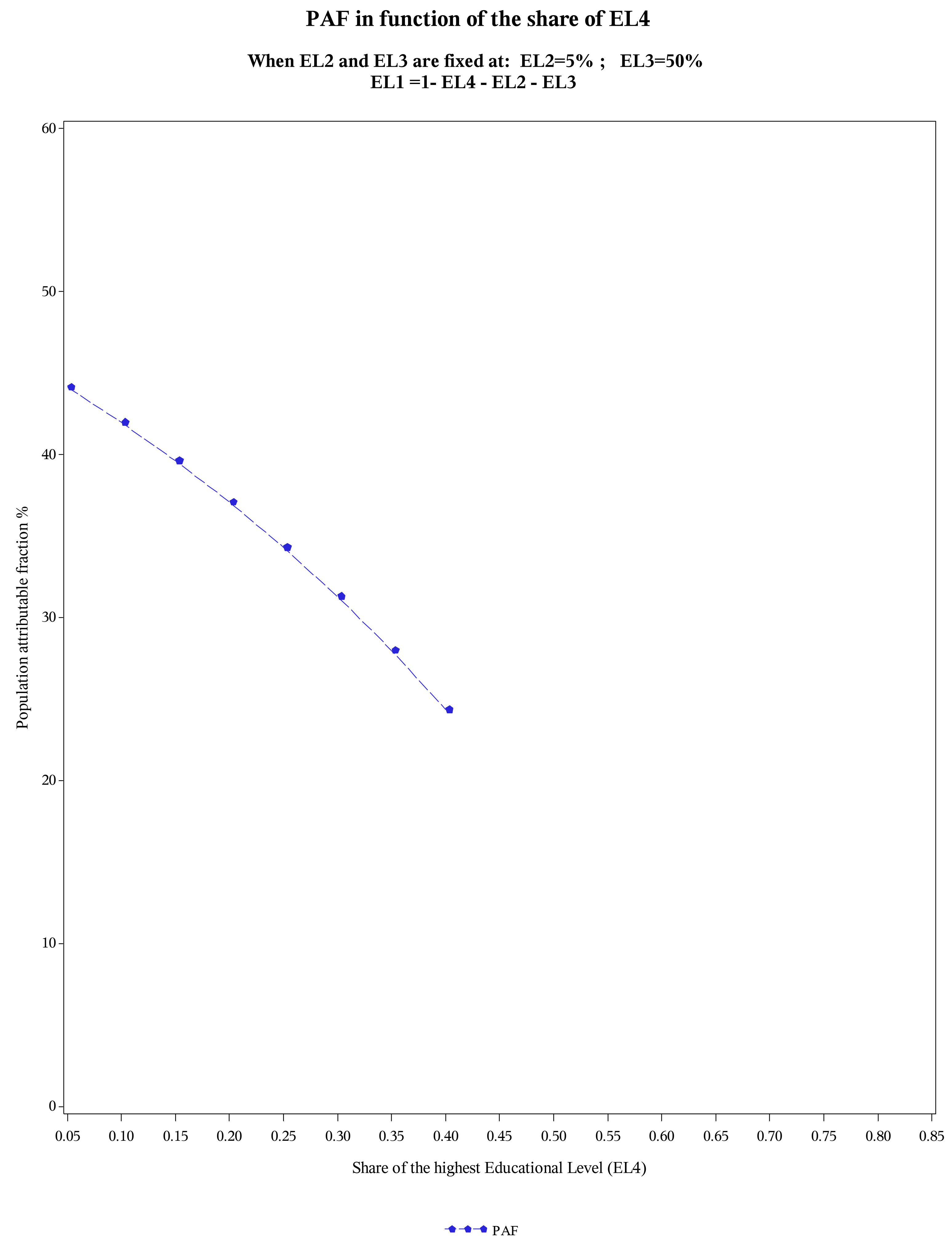
PAF in function of the share of EL4
When EL2 and EL3 are fixed at: EL2=5% ; EL3=50%
EL1 =1- EL4 - EL2 - EL3
Population attributable fraction %
0
10
20
30
40
50
60
0.05
0.10
0.15
0.20
0.25
0.30
0.35
0.40
0.45
0.50
0.55
0.60
0.65
0.70
0.75
0.80
0.85
Share of the highest Educational Level (EL4)
PAF

PAF in function of the share of EL4

When EL2 and EL3 are fixed at: EL2=5% ; EL3=55%
EL1 =1- EL4 - EL2 - EL3

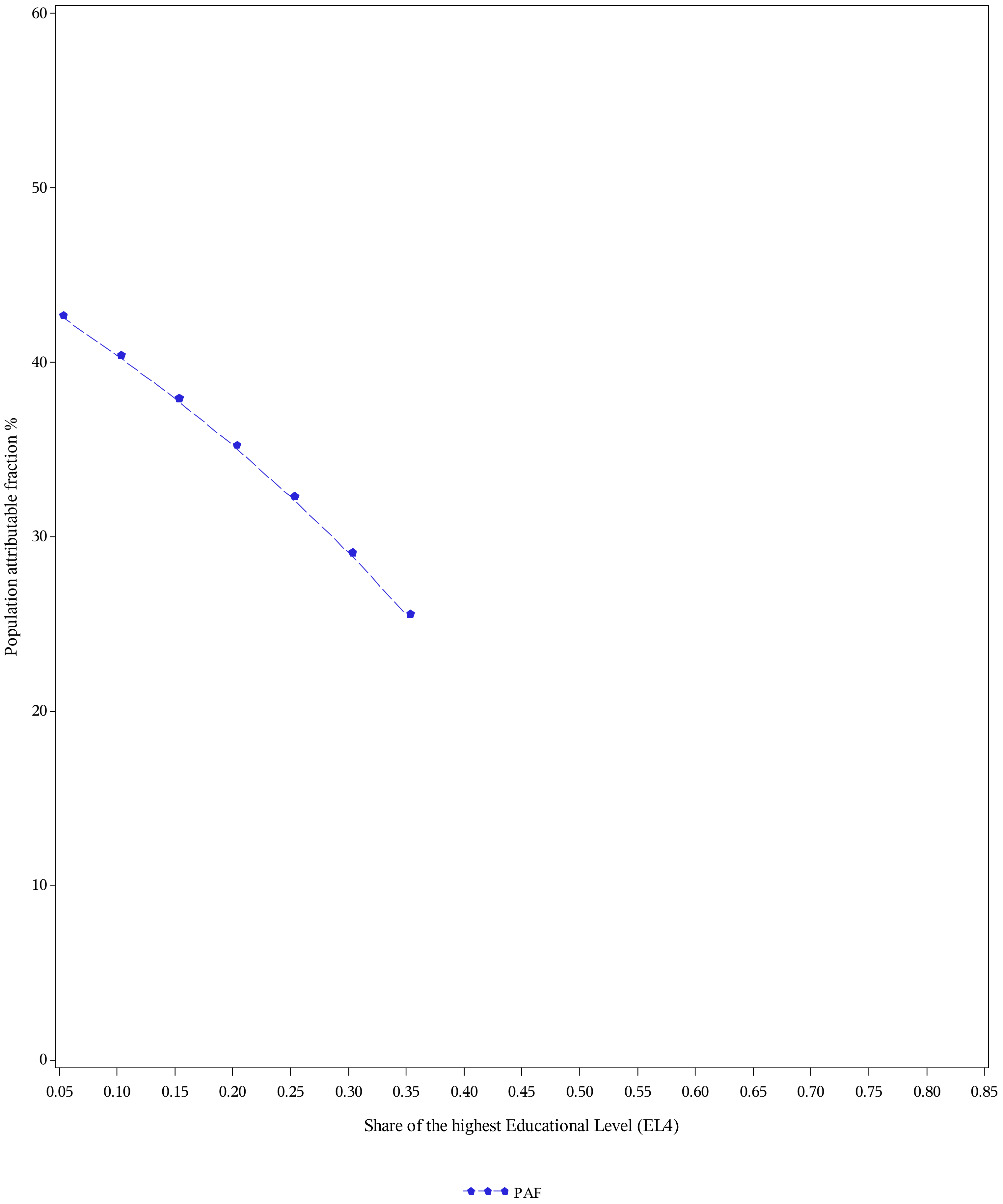

PAF in function of the share of EL4

When EL2 and EL3 are fixed at: EL2=5% ; EL3=60%
EL1 =1- EL4 - EL2 - EL3

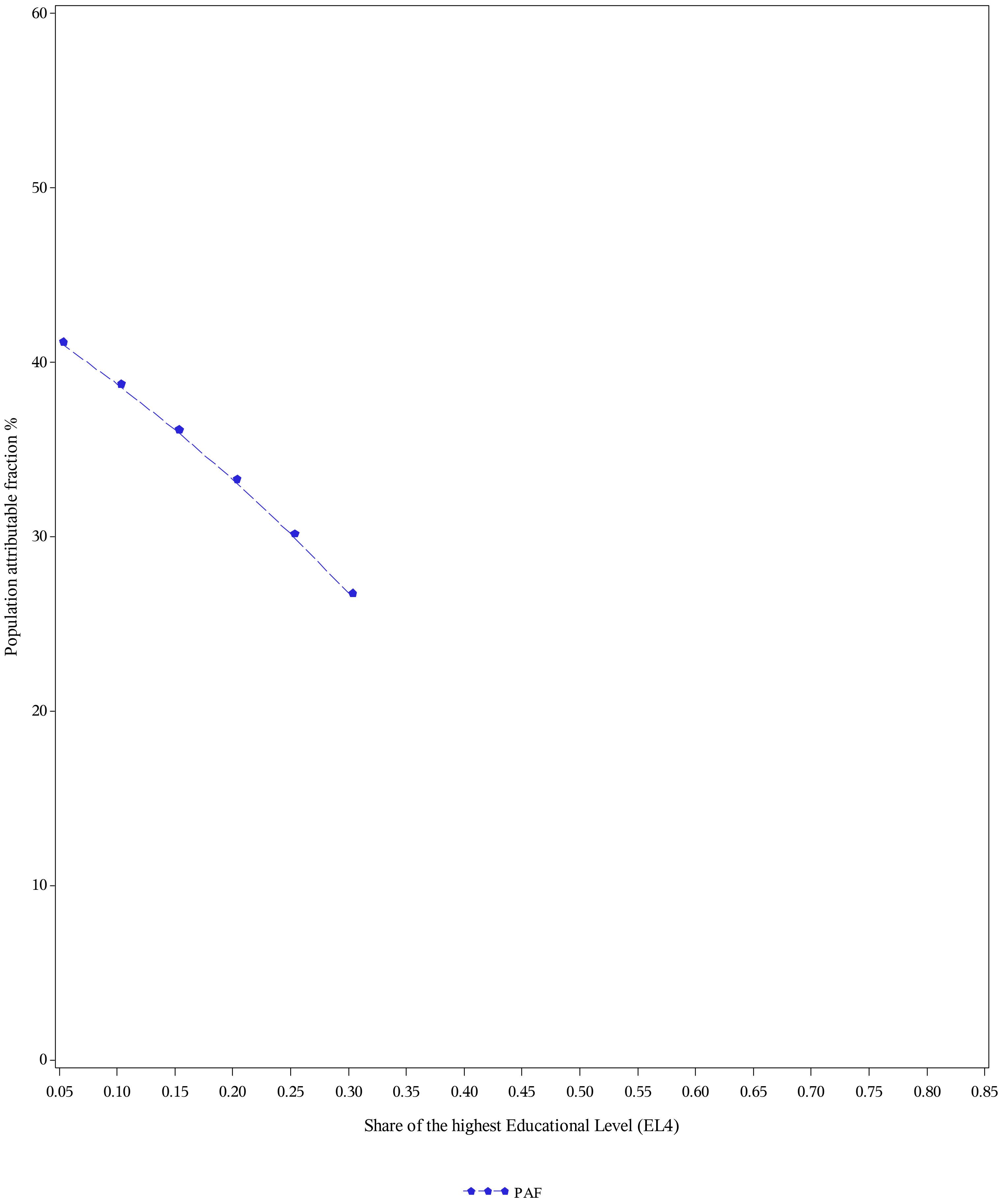

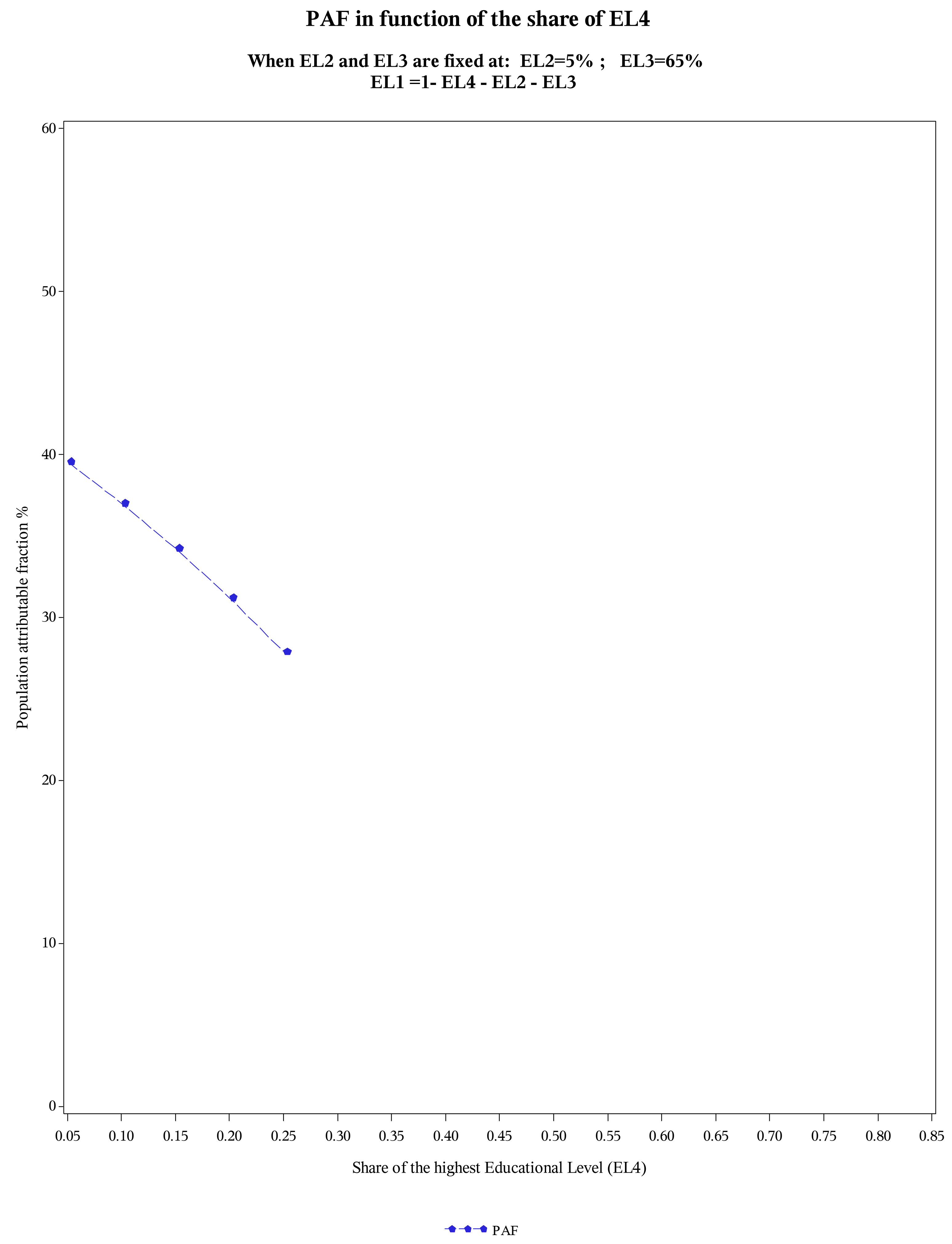

PAF in function of the share of EL4
When EL2 and EL3 are fixed at: EL2=5% ; EL3=65%
EL1 =1- EL4 - EL2 - EL3
Population attributable fraction %
0
10
20
30
40
50
60
0.05
0.10
0.15
0.20
0.25
0.30
0.35
0.40
0.45
0.50
0.55
0.60
0.65
0.70
0.75
0.80
0.85
Share of the highest Educational Level (EL4)
PAF

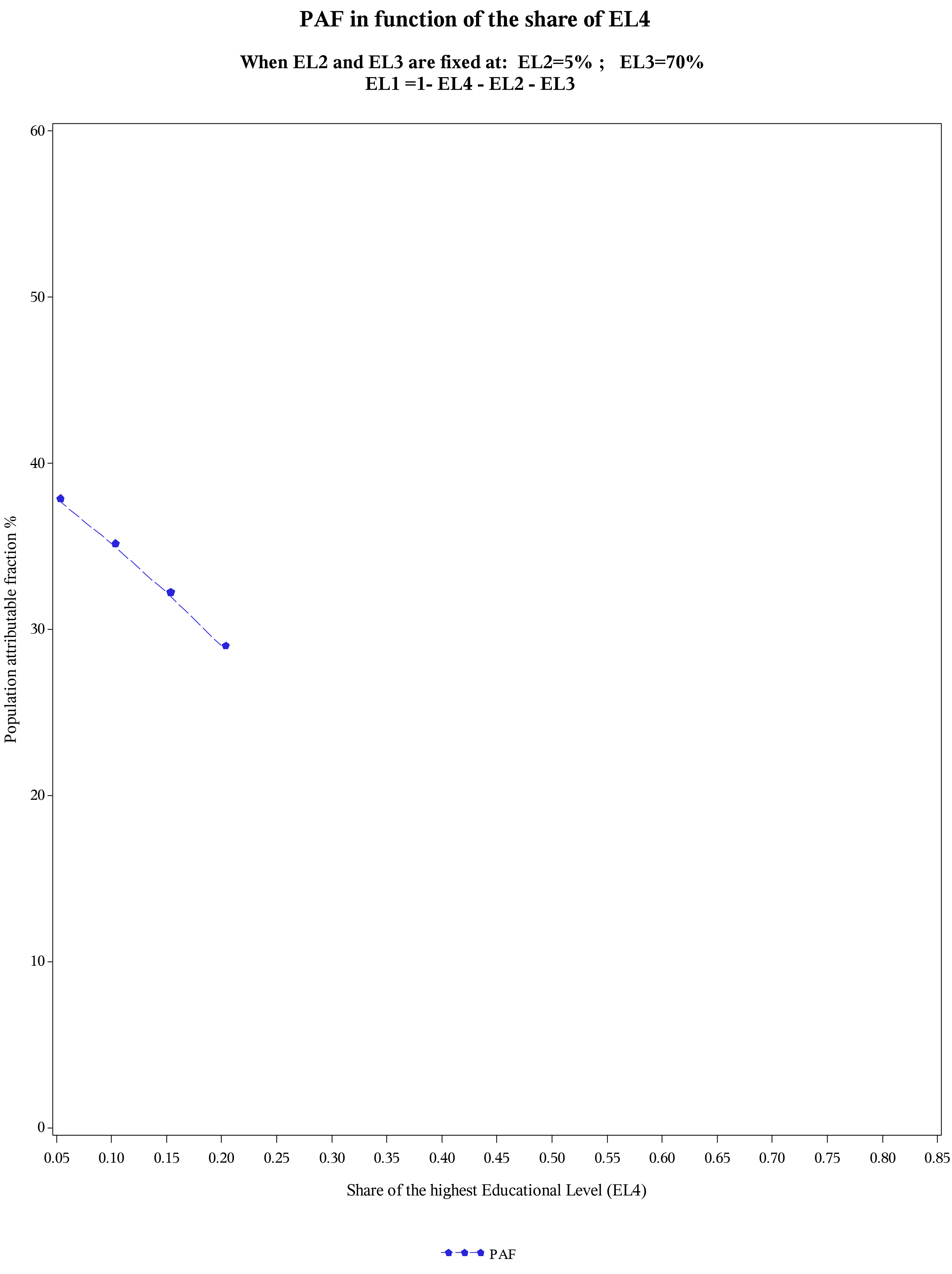
PAF in function of the share of EL4
When EL2 and EL3 are fixed at: EL2=5% ; EL3=70%
EL1 =1- EL4 - EL2 - EL3
Population attributable fraction %
0
10
20
30
40
50
60
0.05
0.10
0.15
0.20
0.25
0.30
0.35
0.40
0.45
0.50
0.55
0.60
0.65
0.70
0.75
0.80
0.85
Share of the highest Educational Level (EL4)
PAF

PAF in function of the share of EL4

When EL2 and EL3 are fixed at: EL2=5% ; EL3=75%
EL1 =1- EL4 - EL2 - EL3

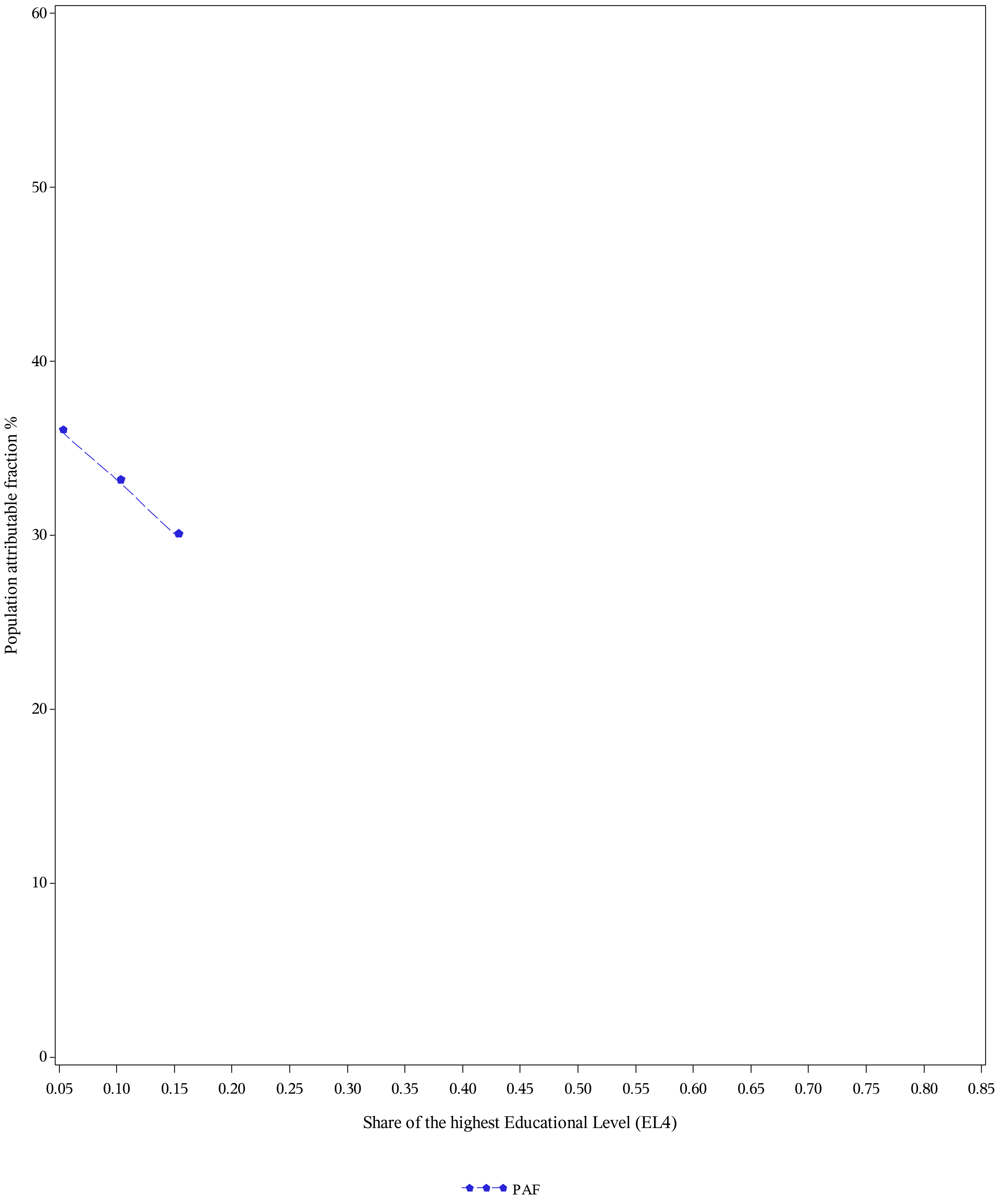

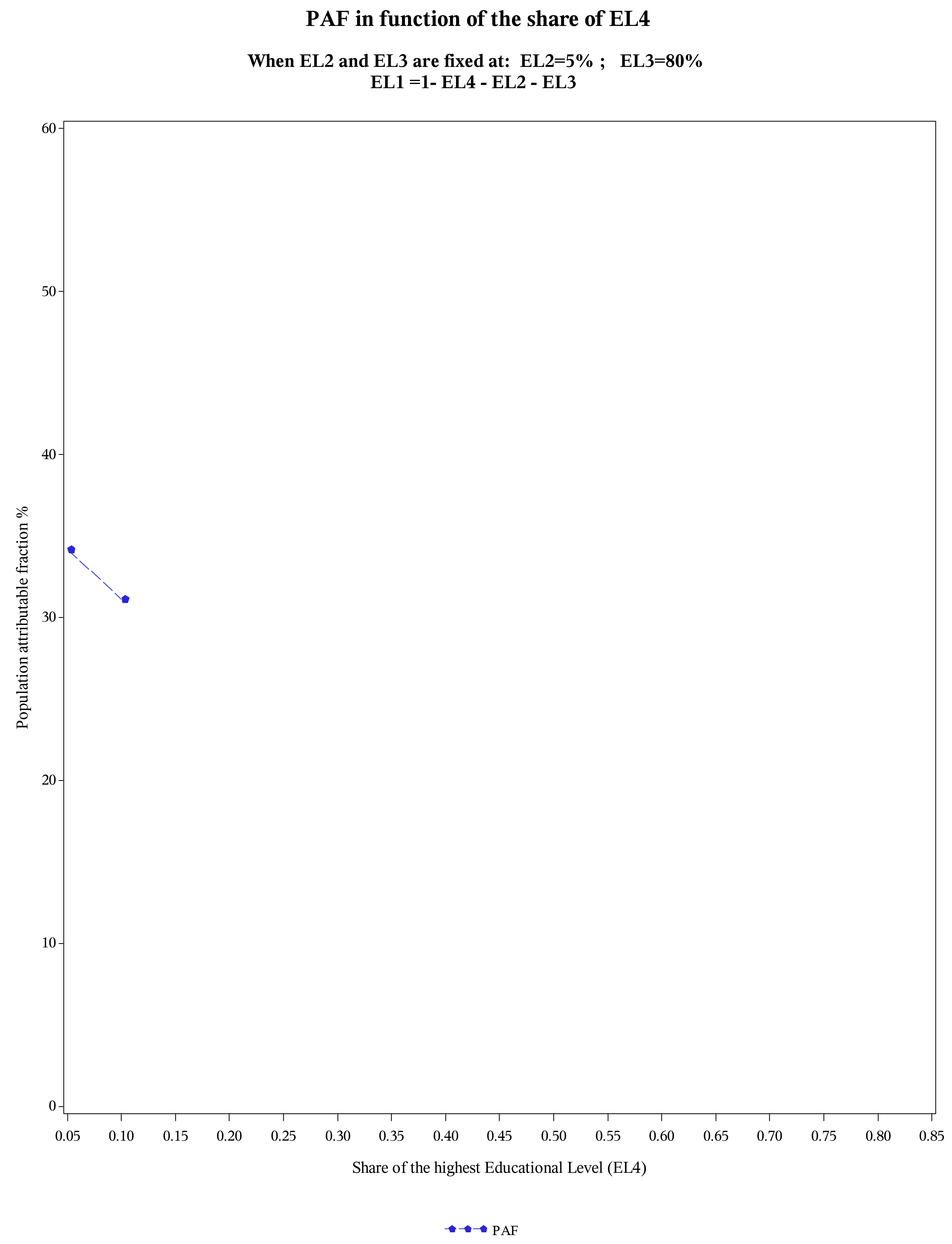
PAF in function of the share of EL4
When EL2 and EL3 are fixed at: EL2=5% ; EL3=80%
EL1 =1- EL4 - EL2 - EL3
Population attributable fraction %
0
10
20
30
40
50
60
0.05
0.10
0.15
0.20
0.25
0.30
0.35
0.40
0.45
0.50
0.55
0.60
0.65
0.70
0.75
0.80
0.85
Share of the highest Educational Level (EL4)
PAF

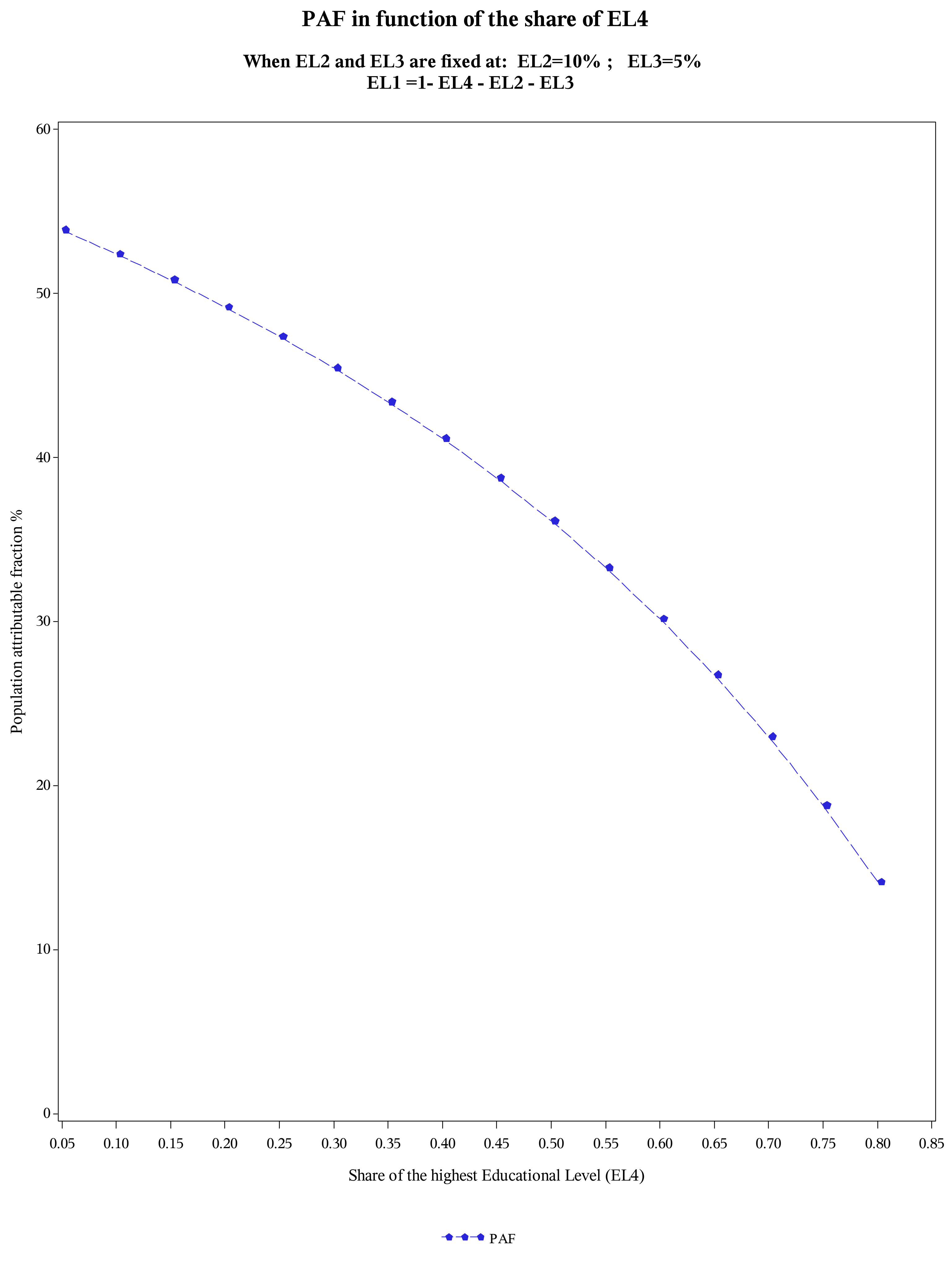
PAF in function of the share of EL4
When EL2 and EL3 are fixed at: EL2=10% ; EL3=5%
EL1 =1- EL4 - EL2 - EL3
Population attributable fraction %
0
10
20
30
40
50
60
0.05
0.10
0.15
0.20
0.25
0.30
0.35
0.40
0.45
0.50
0.55
0.60
0.65
0.70
0.75
0.80
0.85
Share of the highest Educational Level (EL4)
PAF

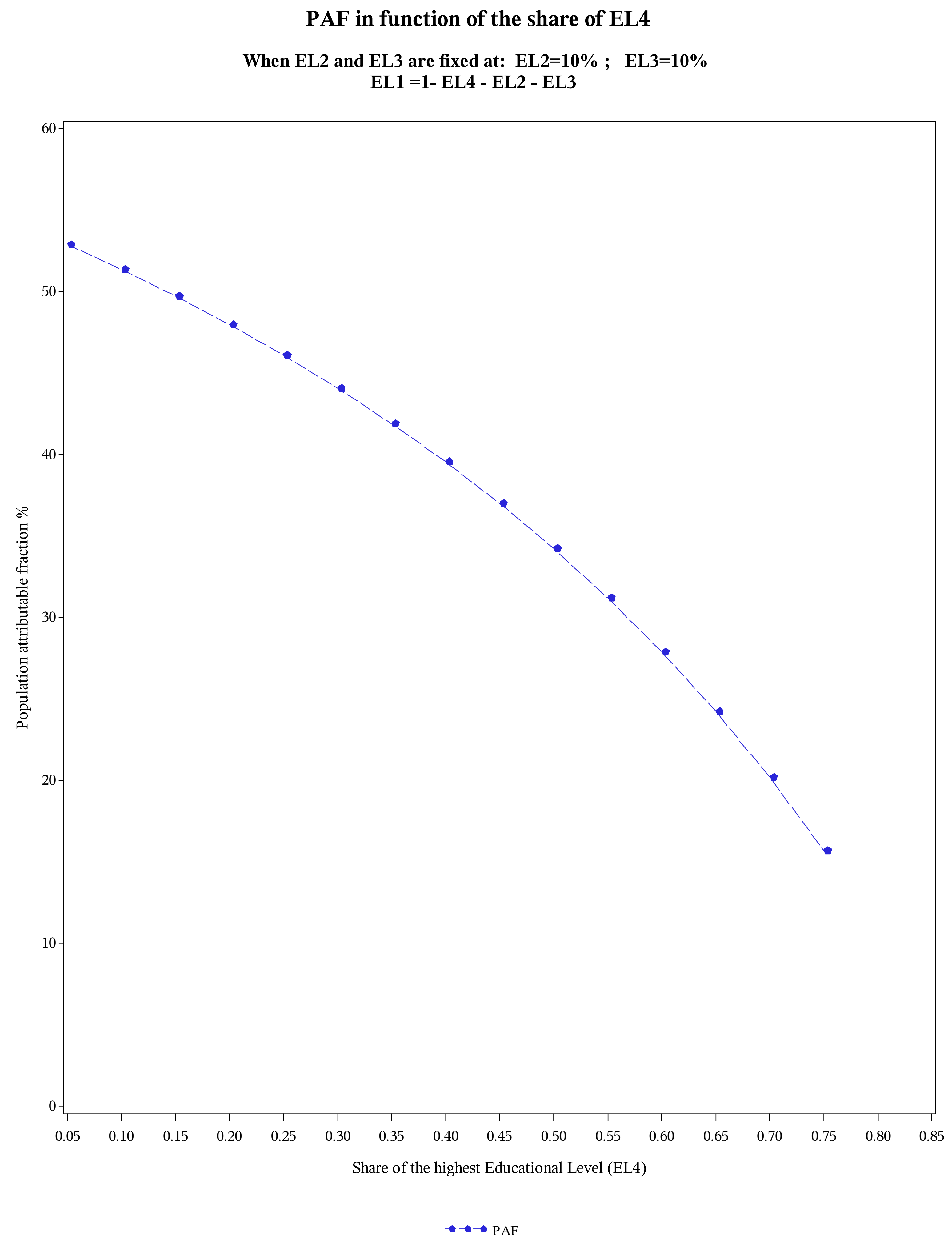

PAF in function of the share of EL4
When EL2 and EL3 are fixed at: EL2=10% ; EL3=10%
EL1 =1- EL4 - EL2 - EL3
Population attributable fraction %
0
10
20
30
40
50
60
0.05
0.10
0.15
0.20
0.25
0.30
0.35
0.40
0.45
0.50
0.55
0.60
0.65
0.70
0.75
0.80
0.85
Share of the highest Educational Level (EL4)
PAF

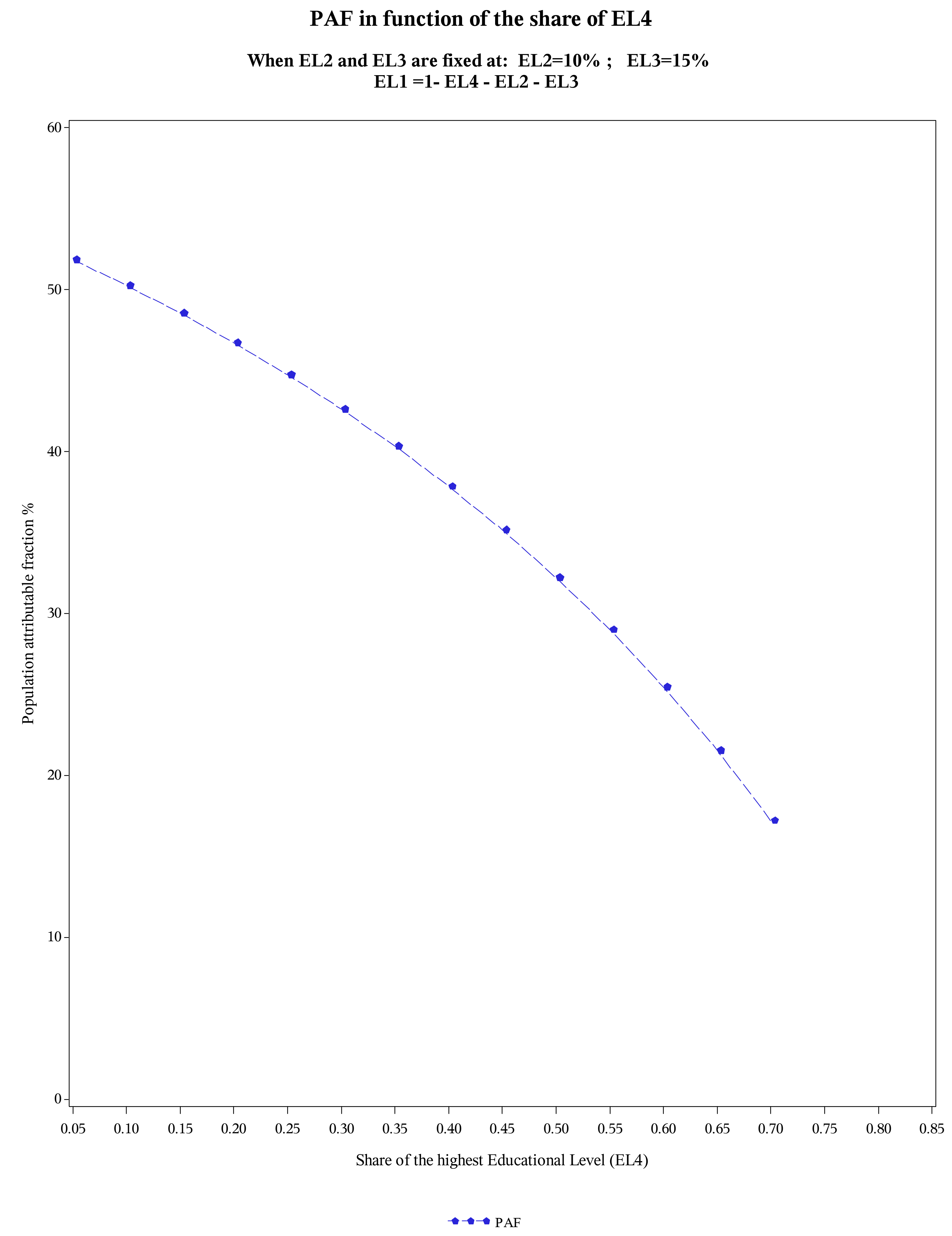
PAF in function of the share of EL4
When EL2 and EL3 are fixed at: EL2=10% ; EL3=15%
EL1 =1- EL4 - EL2 - EL3
Population attributable fraction %
0
10
20
30
40
50
60
0.05
0.10
0.15
0.20
0.25
0.30
0.35
0.40
0.45
0.50
0.55
0.60
0.65
0.70
0.75
0.80
0.85
Share of the highest Educational Level (EL4)
PAF

PAF in function of the share of EL4

When EL2 and EL3 are fixed at: EL2=10% ; EL3=20%
EL1 =1- EL4 - EL2 - EL3

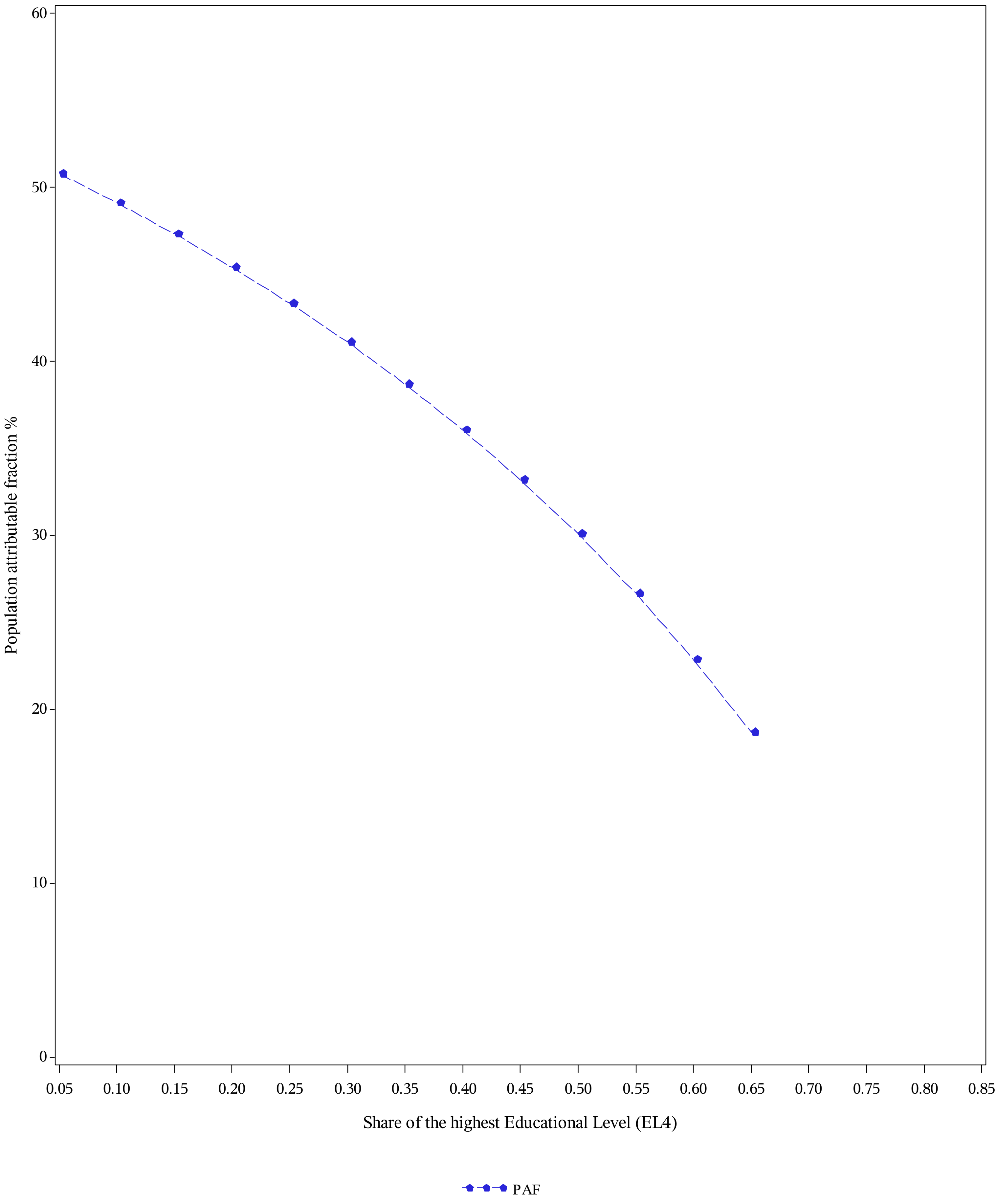

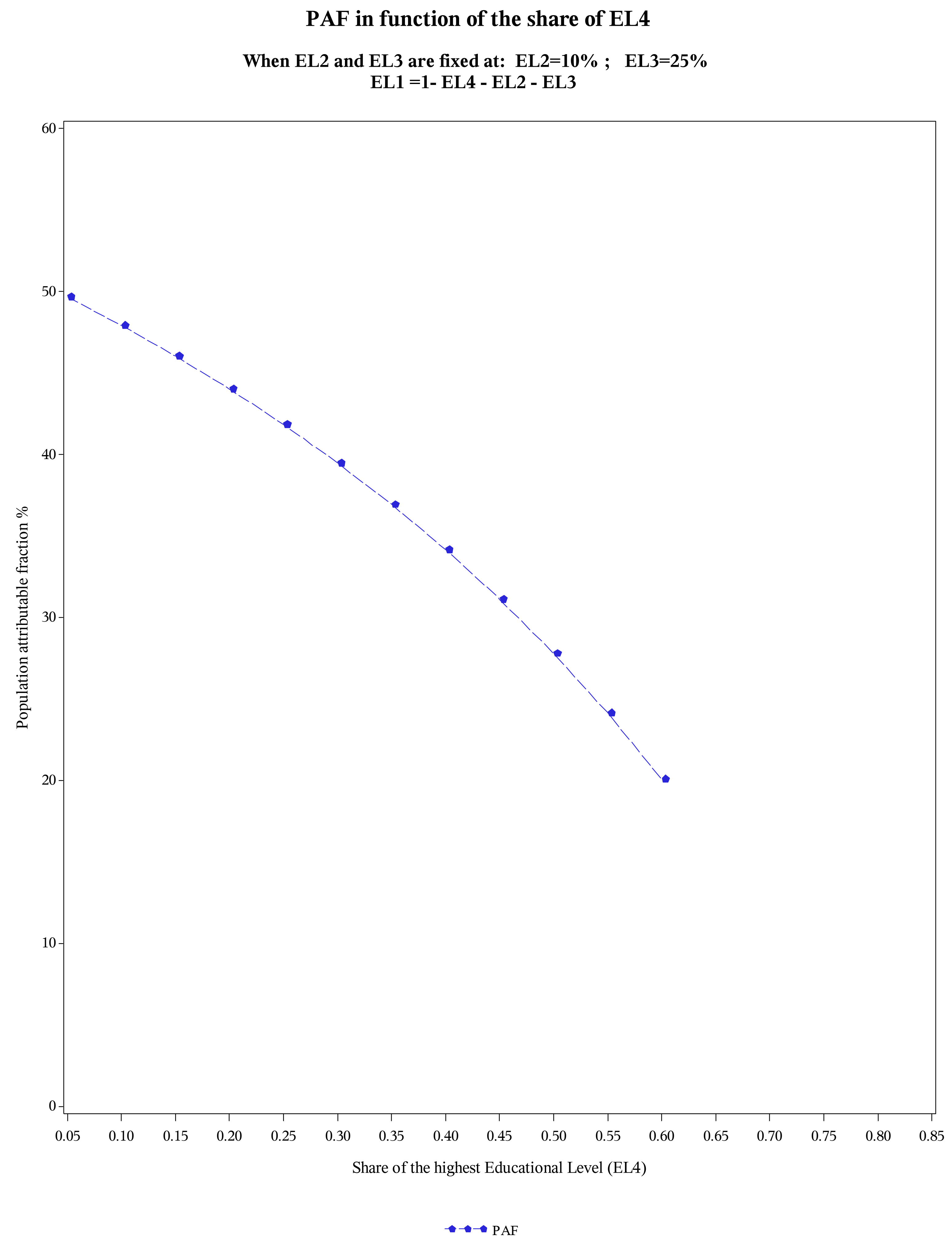
PAF in function of the share of EL4
When EL2 and EL3 are fixed at: EL2=10% ; EL3=25%
EL1 =1- EL4 - EL2 - EL3
Population attributable fraction %
0
10
20
30
40
50
60
0.05
0.10
0.15
0.20
0.25
0.30
0.35
0.40
0.45
0.50
0.55
0.60
0.65
0.70
0.75
0.80
0.85
Share of the highest Educational Level (EL4)
PAF

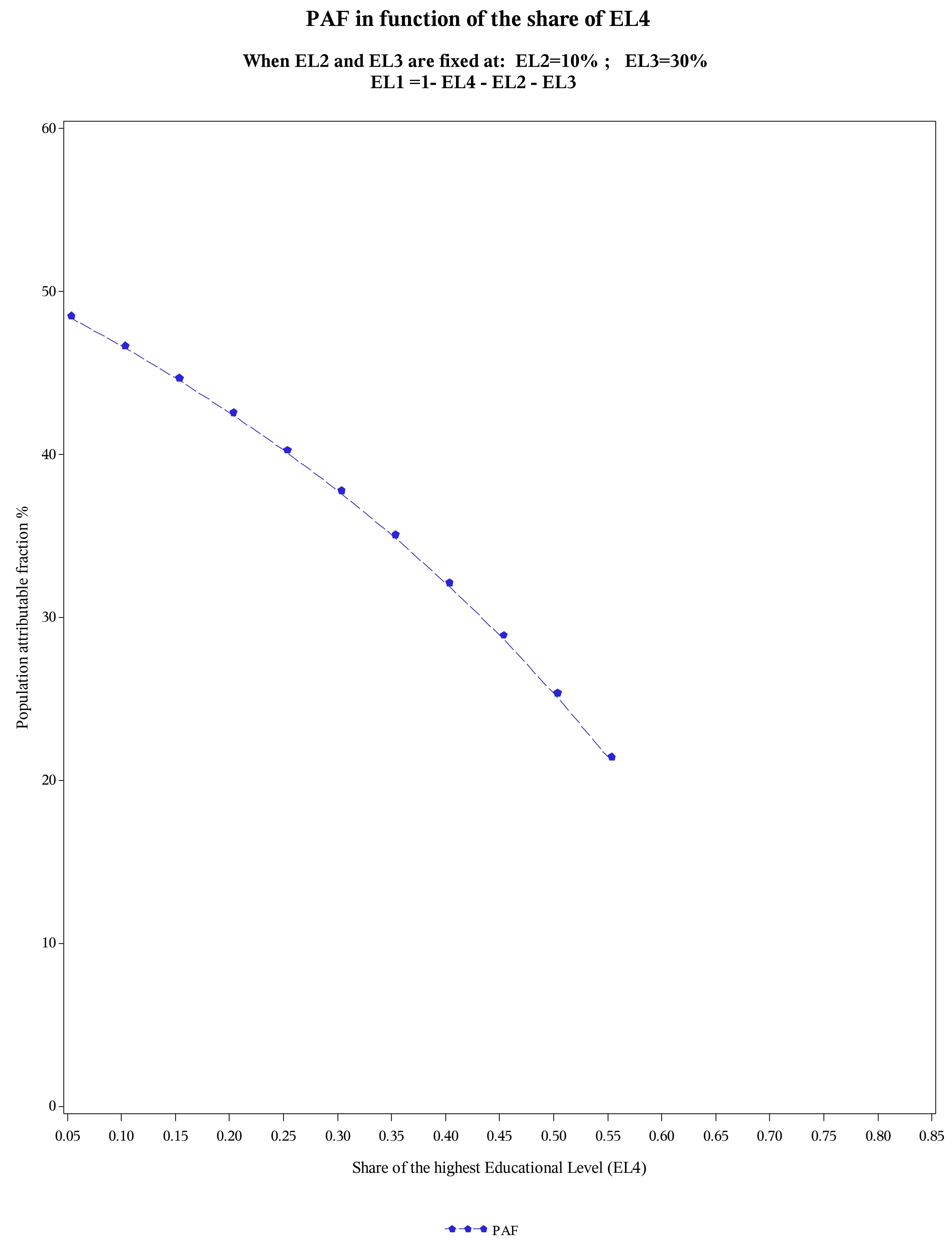
PAF in function of the share of EL4
When EL2 and EL3 are fixed at: EL2=10% ; EL3=30%
EL1 =1- EL4 - EL2 - EL3
Population attributable fraction %
0
10
20
30
40
50
60
0.05
0.10
0.15
0.20
0.25
0.30
0.35
0.40
0.45
0.50
0.55
0.60
0.65
0.70
0.75
0.80
0.85
Share of the highest Educational Level (EL4)
PAF

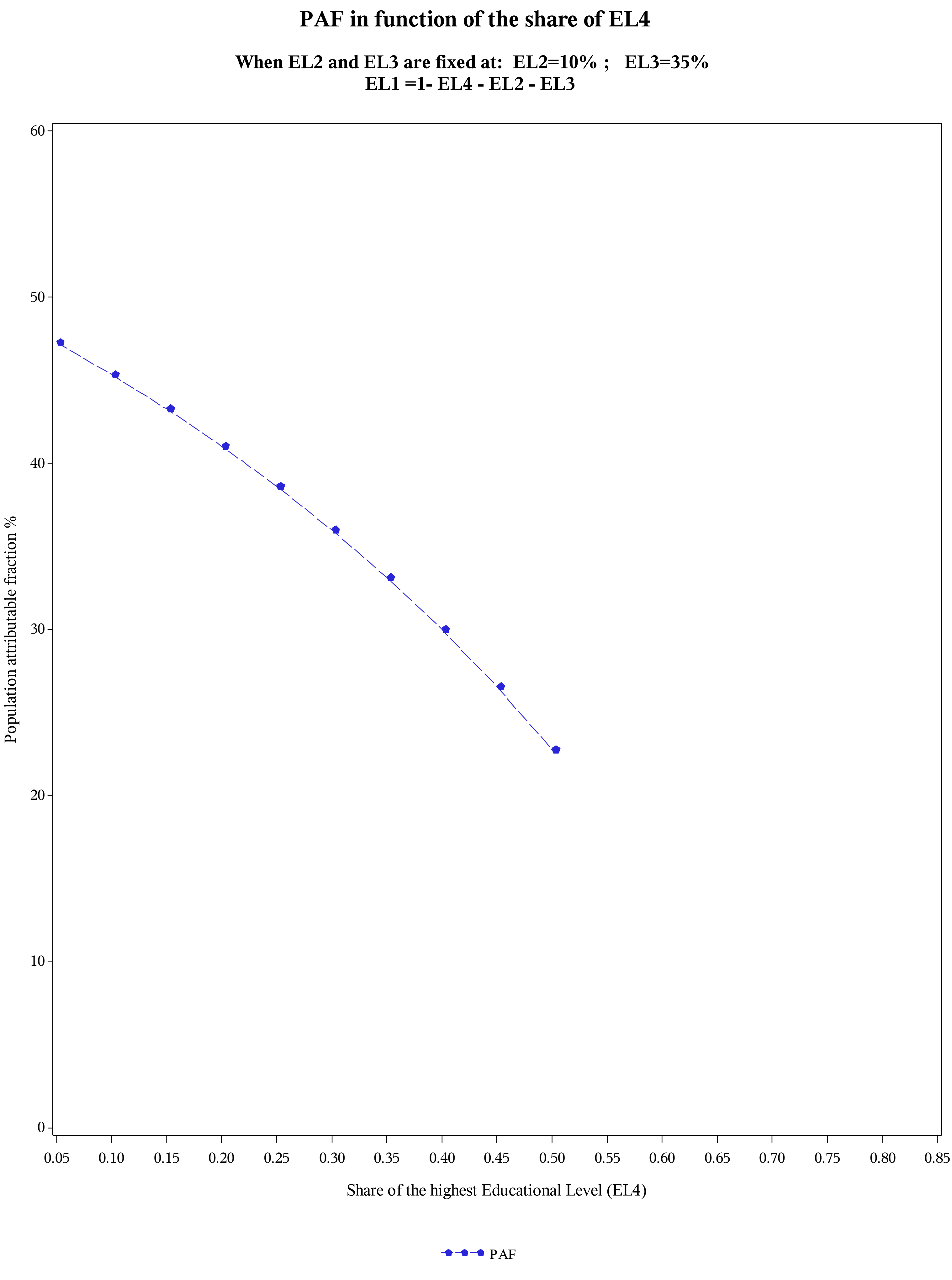
PAF in function of the share of EL4
When EL2 and EL3 are fixed at: EL2=10% ; EL3=35%
EL1 =1- EL4 - EL2 - EL3
60
50
40
30
20
10
0
Population attributable fraction %
0.05
0.10
0.15
0.20
0.25
0.30
0.35
0.40
0.45
0.50
0.55
0.60
0.65
0.70
0.75
0.80
0.85
Share of the highest Educational Level (EL4)
PAF

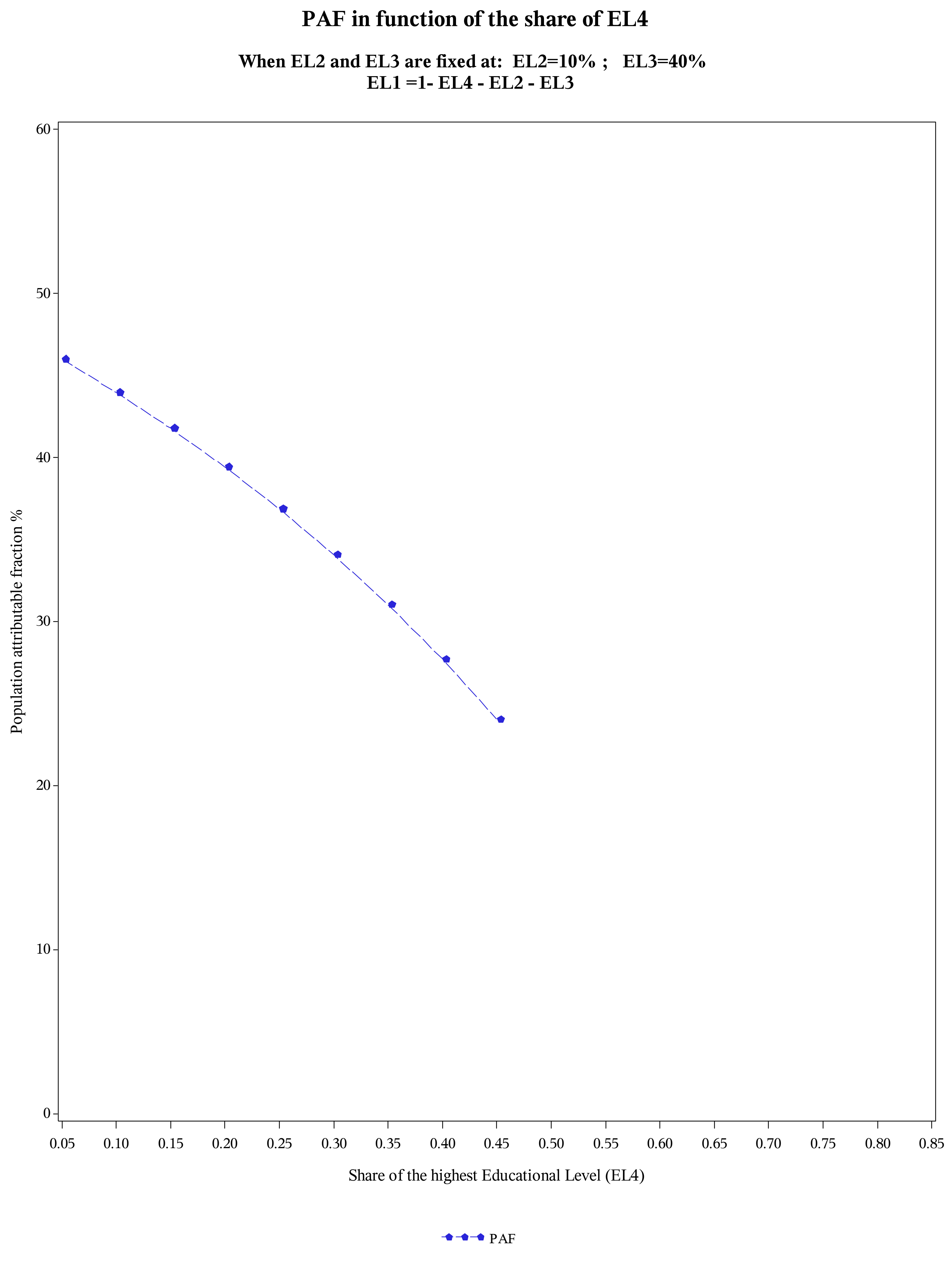
PAF in function of the share of EL4
When EL2 and EL3 are fixed at: EL2=10% ; EL3=40%
EL1 =1- EL4 - EL2 - EL3
Population attributable fraction %
0
10
20
30
40
50
60
0.05
0.10
0.15
0.20
0.25
0.30
0.35
0.40
0.45
0.50
0.55
0.60
0.65
0.70
0.75
0.80
0.85
Share of the highest Educational Level (EL4)
PAF

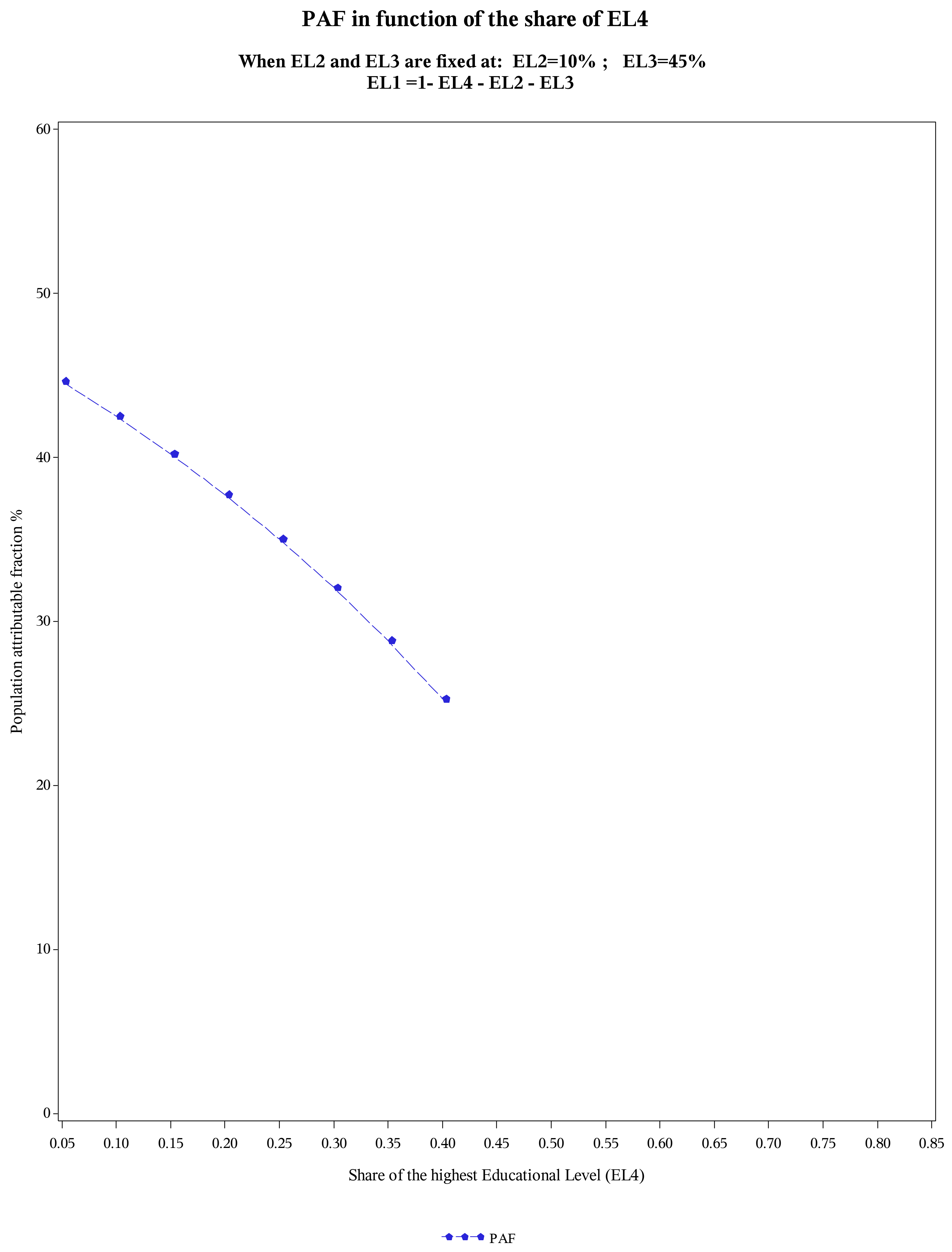
PAF in function of the share of EL4
When EL2 and EL3 are fixed at: EL2=10% ; EL3=45%
EL1 =1- EL4 - EL2 - EL3
Population attributable fraction %
0
10
20
30
40
50
60
0.05
0.10
0.15
0.20
0.25
0.30
0.35
0.40
0.45
0.50
0.55
0.60
0.65
0.70
0.75
0.80
0.85
Share of the highest Educational Level (EL4)
PAF

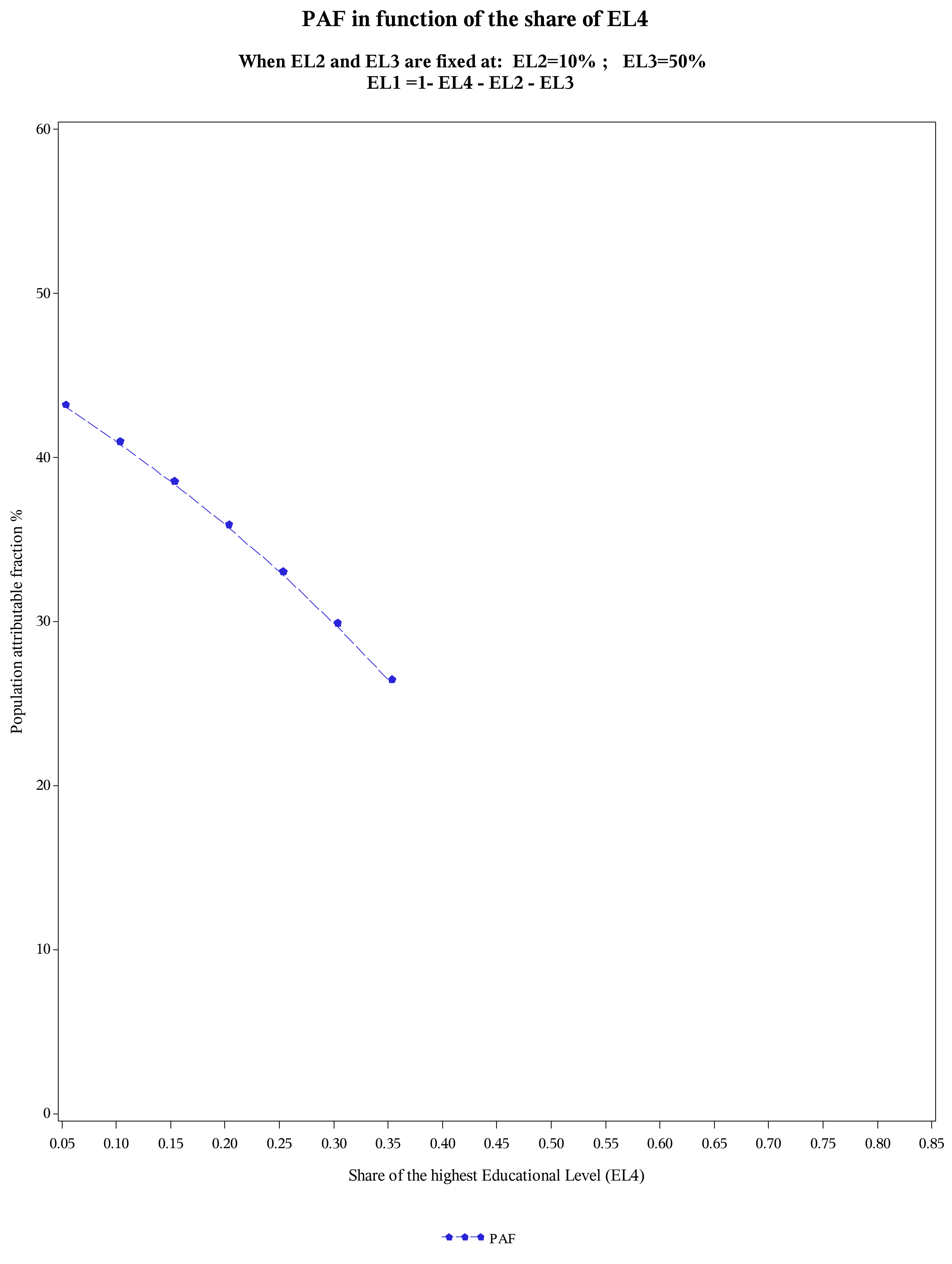

PAF in function of the share of EL4
When EL2 and EL3 are fixed at: EL2=10% ; EL3=50%
EL1 =1- EL4 - EL2 - EL3
Population attributable fraction %
0
10
20
30
40
50
60
0.05
0.10
0.15
0.20
0.25
0.30
0.35
0.40
0.45
0.50
0.55
0.60
0.65
0.70
0.75
0.80
0.85
Share of the highest Educational Level (EL4)
PAF

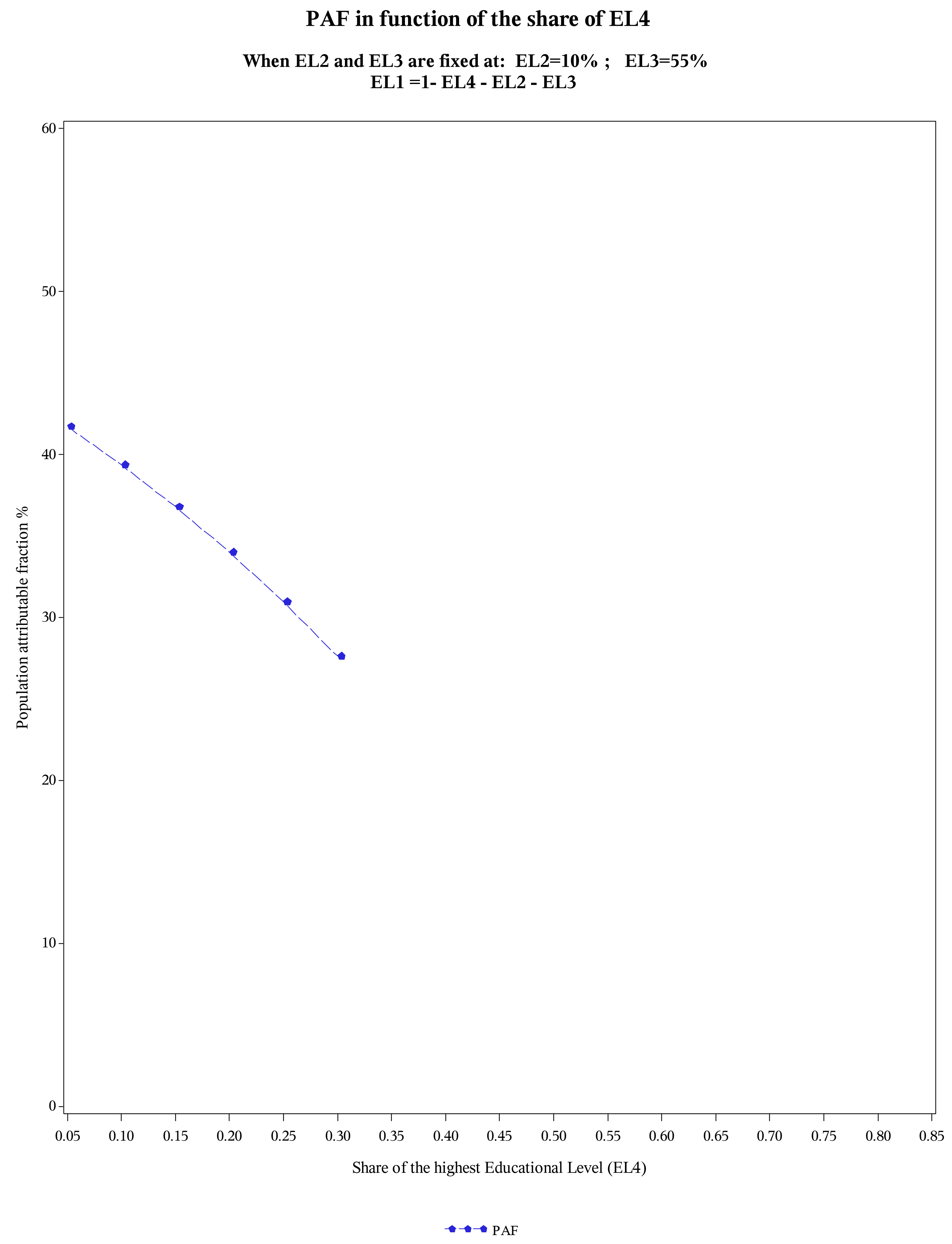
PAF in function of the share of EL4
When EL2 and EL3 are fixed at: EL2=10% ; EL3=55%
EL1 =1- EL4 - EL2 - EL3
Population attributable fraction %
0
10
20
30
40
50
60
0.05
0.10
0.15
0.20
0.25
0.30
0.35
0.40
0.45
0.50
0.55
0.60
0.65
0.70
0.75
0.80
0.85
Share of the highest Educational Level (EL4)
PAF

PAF in function of the share of EL4

When EL2 and EL3 are fixed at: EL2=10% ; EL3=60%
EL1 =1- EL4 - EL2 - EL3

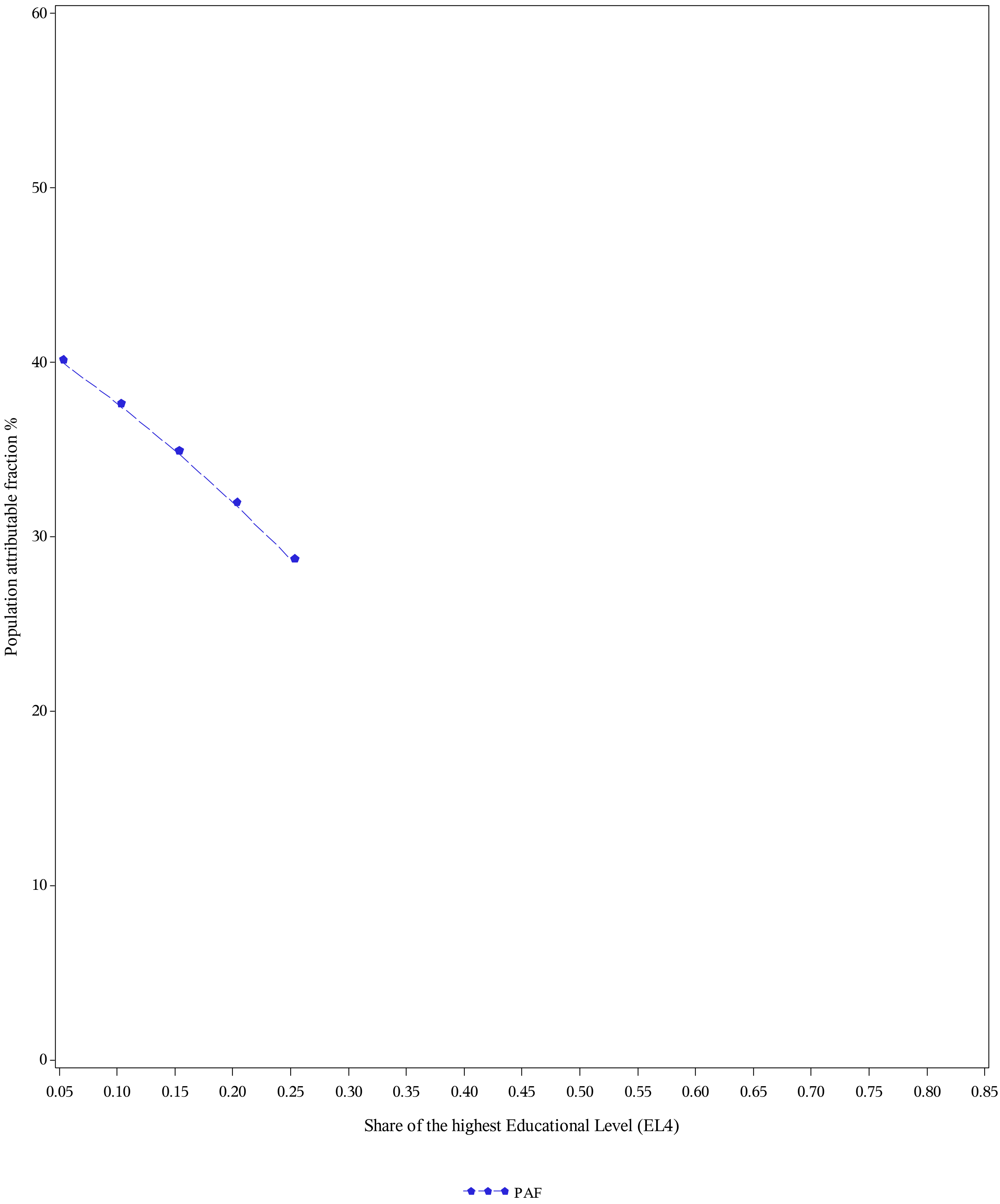

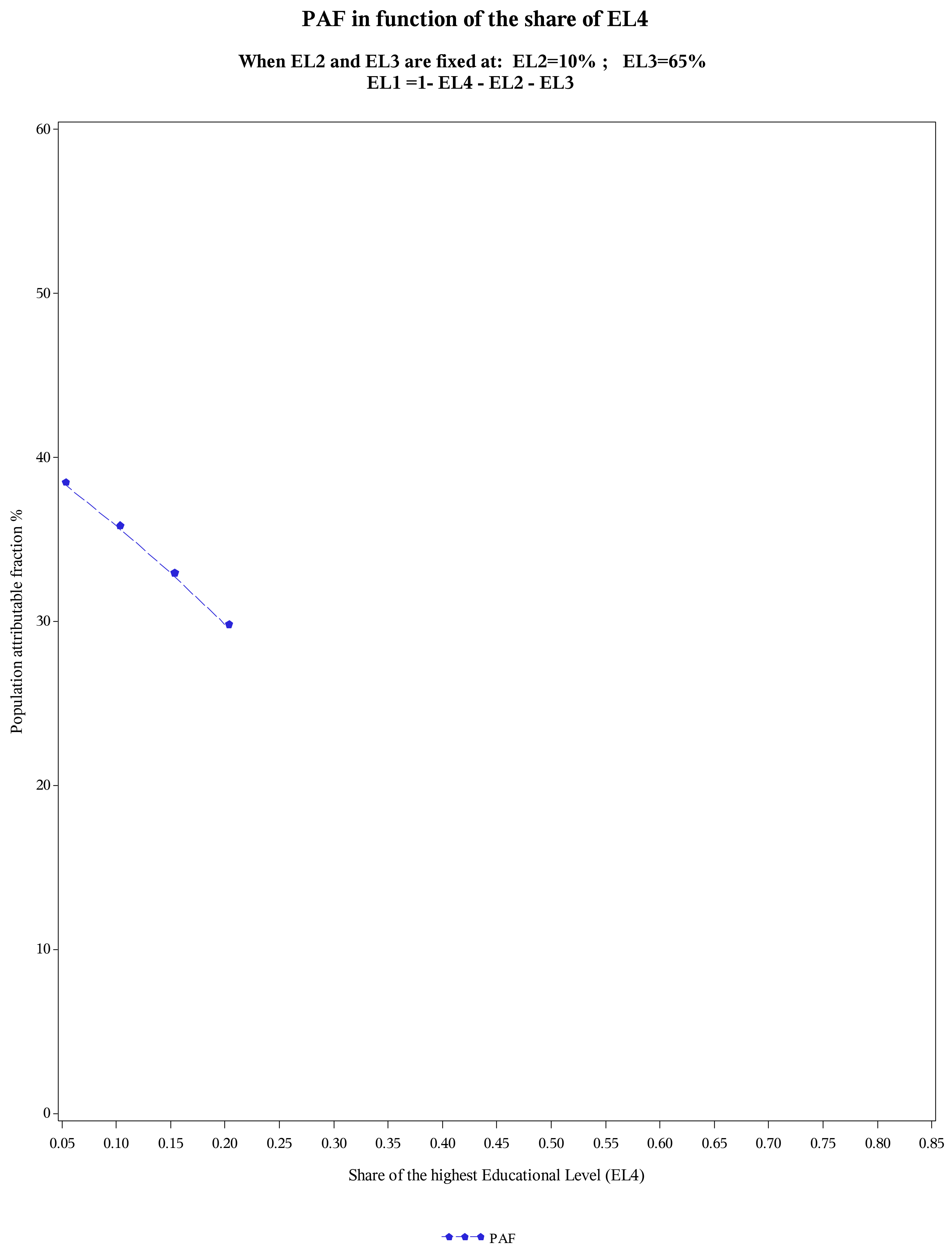
PAF in function of the share of EL4
When EL2 and EL3 are fixed at: EL2=10% ; EL3=65%
EL1 =1- EL4 - EL2 - EL3
Population attributable fraction %
0
10
20
30
40
50
60
0.05
0.10
0.15
0.20
0.25
0.30
0.35
0.40
0.45
0.50
0.55
0.60
0.65
0.70
0.75
0.80
0.85
Share of the highest Educational Level (EL4)
PAF

PAF in function of the share of EL4

When EL2 and EL3 are fixed at: EL2=10% ; EL3=70%
EL1 =1- EL4 - EL2 - EL3

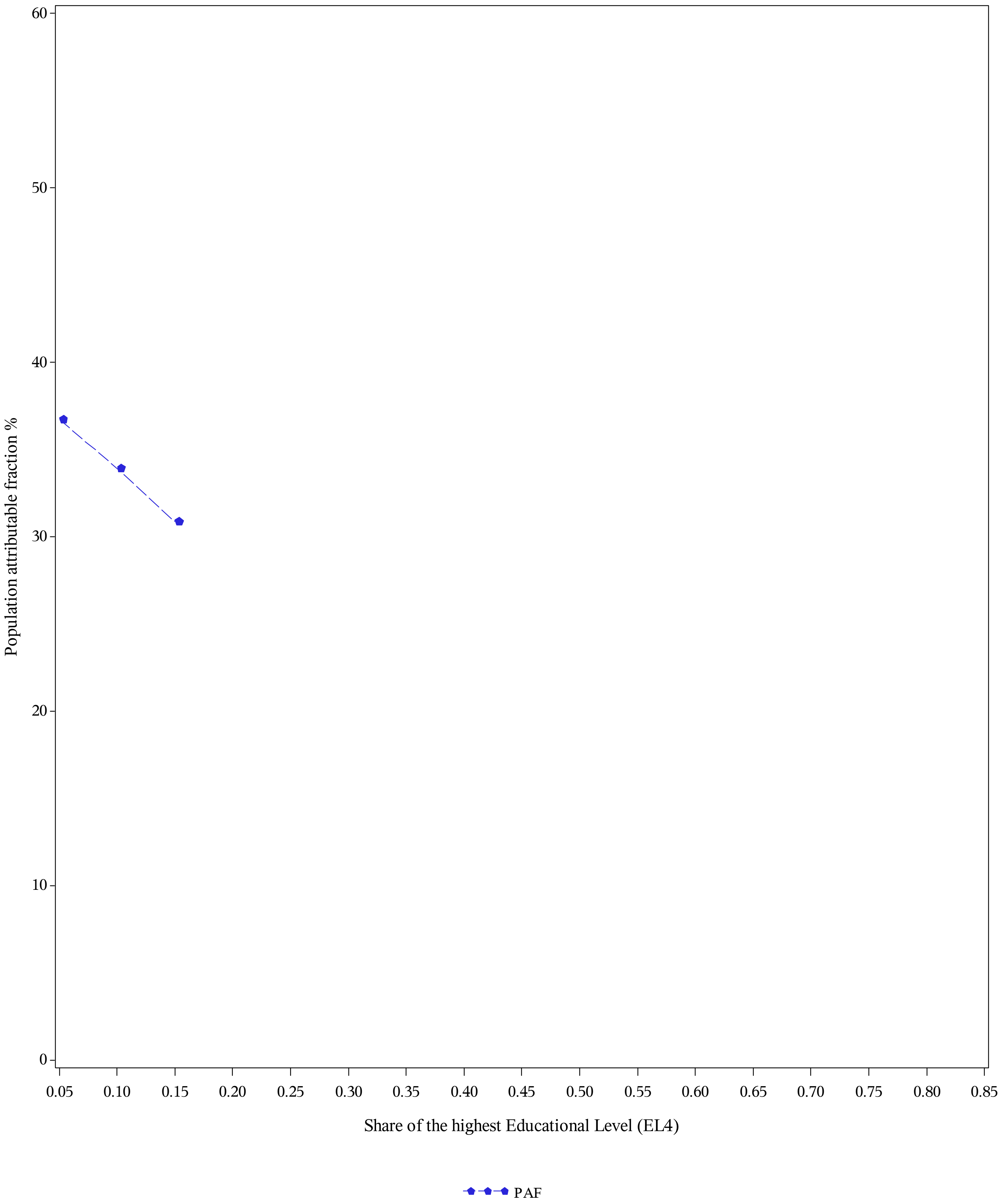

PAF in function of the share of EL4

When EL2 and EL3 are fixed at: EL2=10% ; EL3=75%
EL1 =1- EL4 - EL2 - EL3

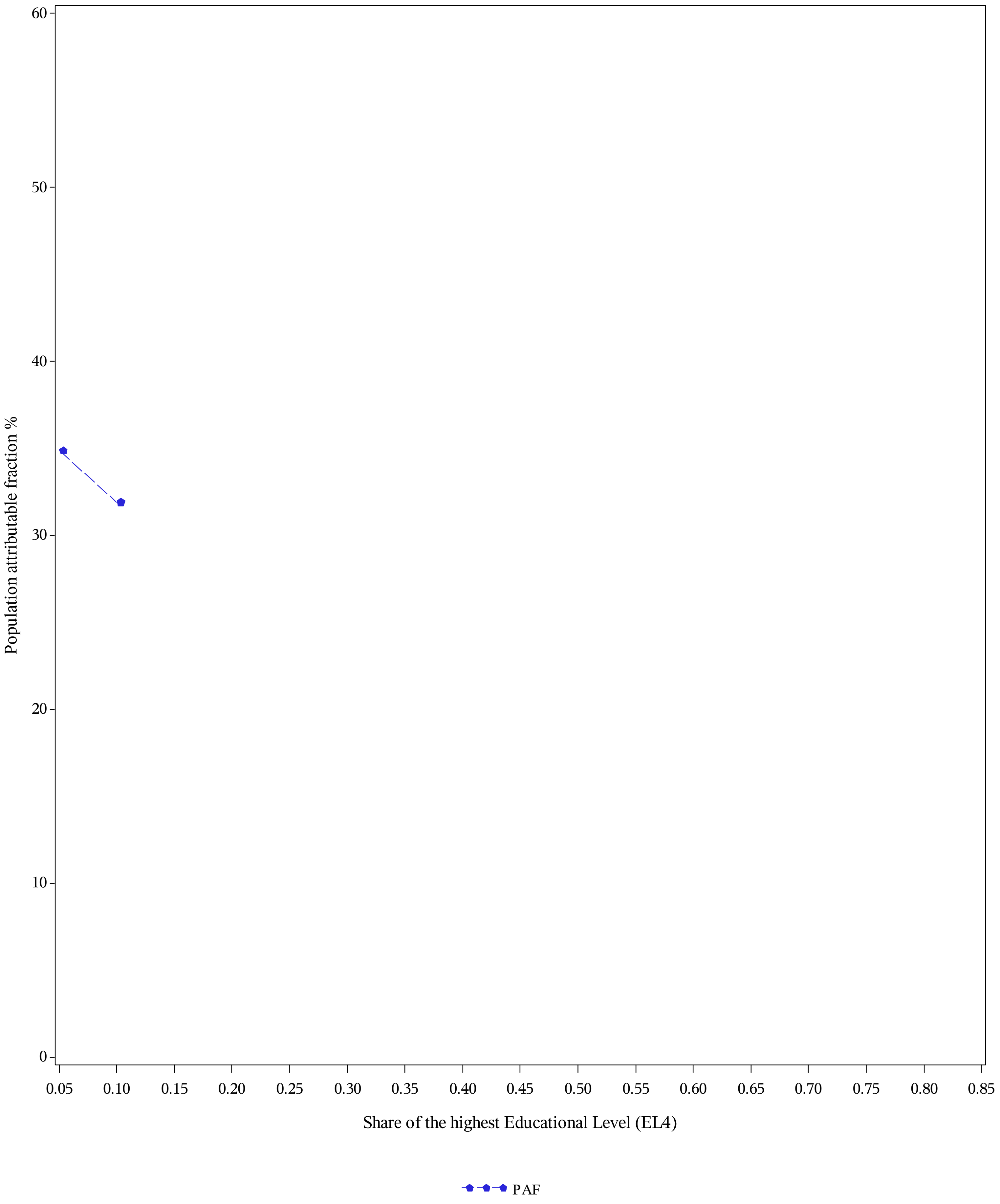

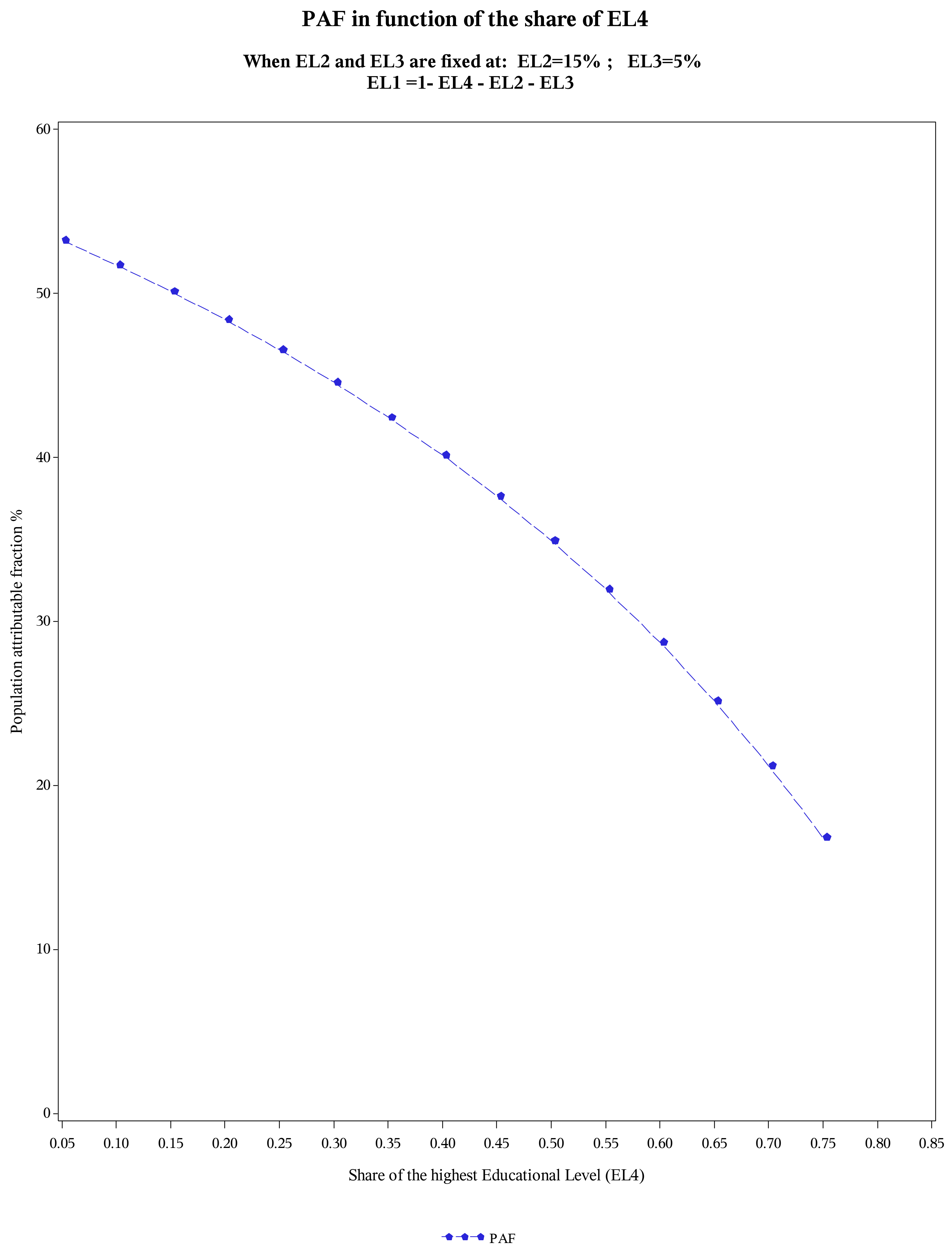
PAF in function of the share of EL4
When EL2 and EL3 are fixed at: EL2=15% ; EL3=5%
EL1 =1- EL4 - EL2 - EL3
Population attributable fraction %
0
10
20
30
40
50
60
0.05
0.10
0.15
0.20
0.25
0.30
0.35
0.40
0.45
0.50
0.55
0.60
0.65
0.70
0.75
0.80
0.85
Share of the highest Educational Level (EL4)
PAF

PAF in function of the share of EL4

When EL2 and EL3 are fixed at: EL2=15% ; EL3=10%
EL1 =1- EL4 - EL2 - EL3

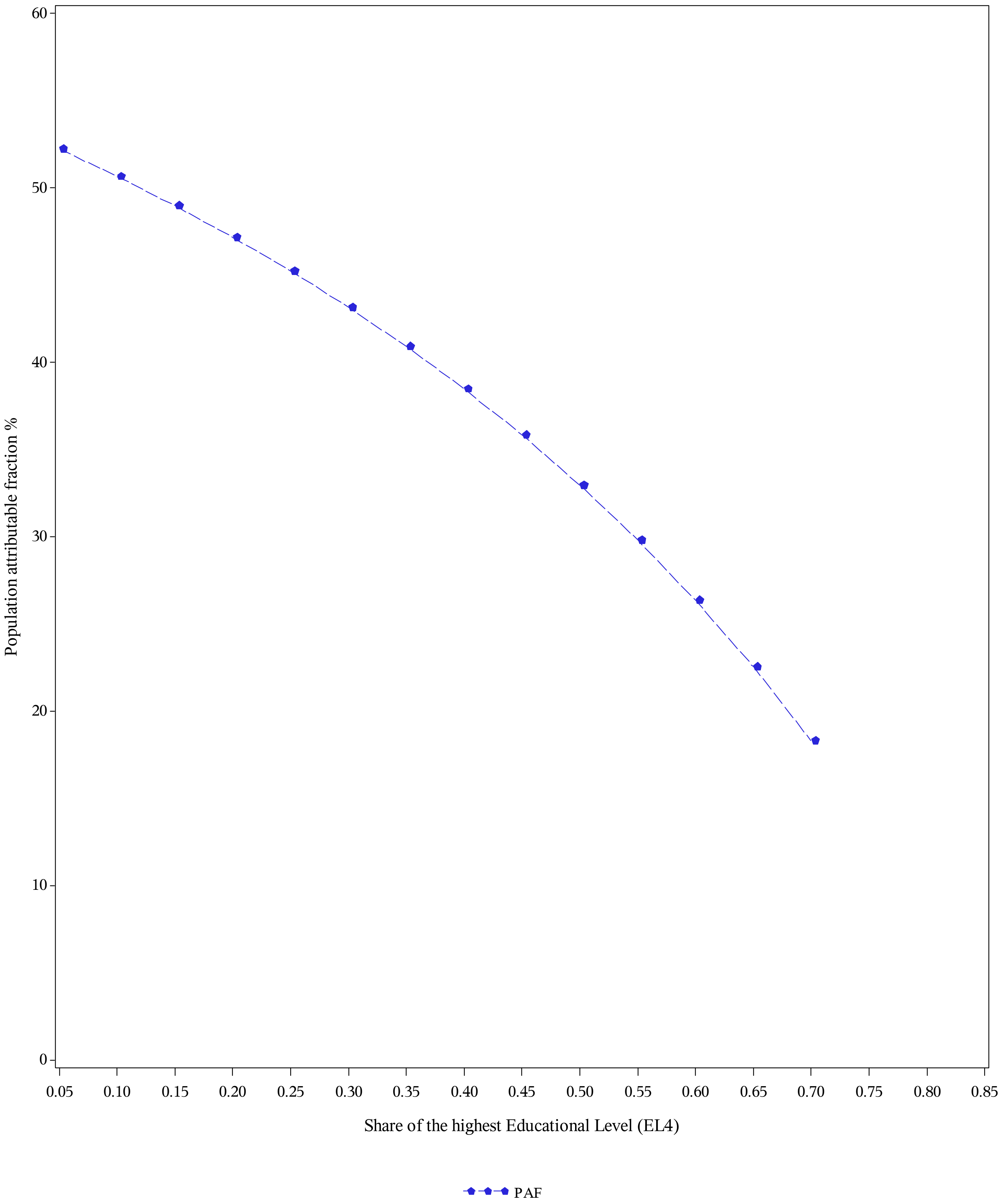

PAF in function of the share of EL4

When EL2 and EL3 are fixed at: EL2=15% ; EL3=15%
EL1 =1- EL4 - EL2 - EL3

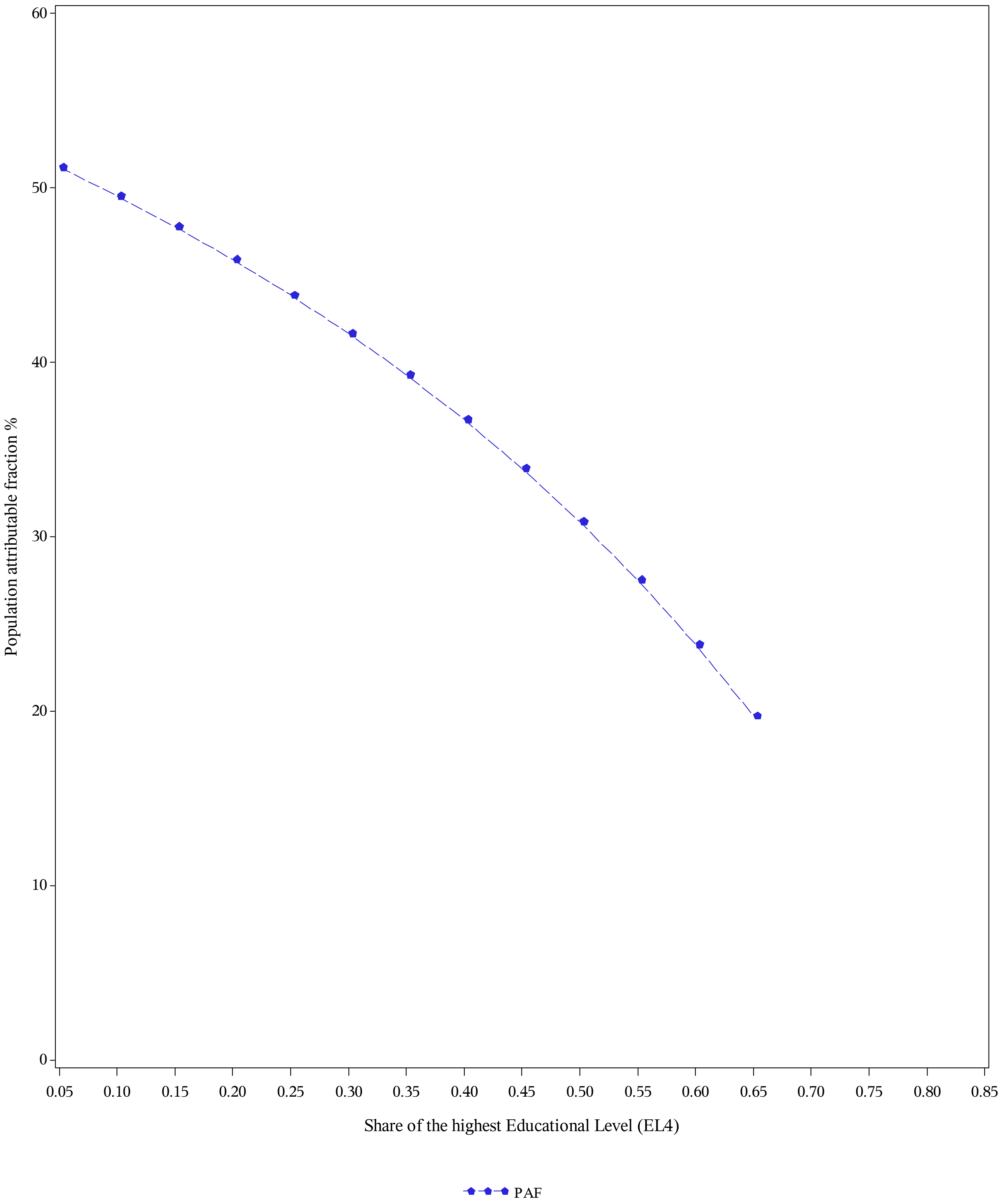

PAF in function of the share of EL4

When EL2 and EL3 are fixed at: EL2=15% ; EL3=20%
EL1 =1- EL4 - EL2 - EL3

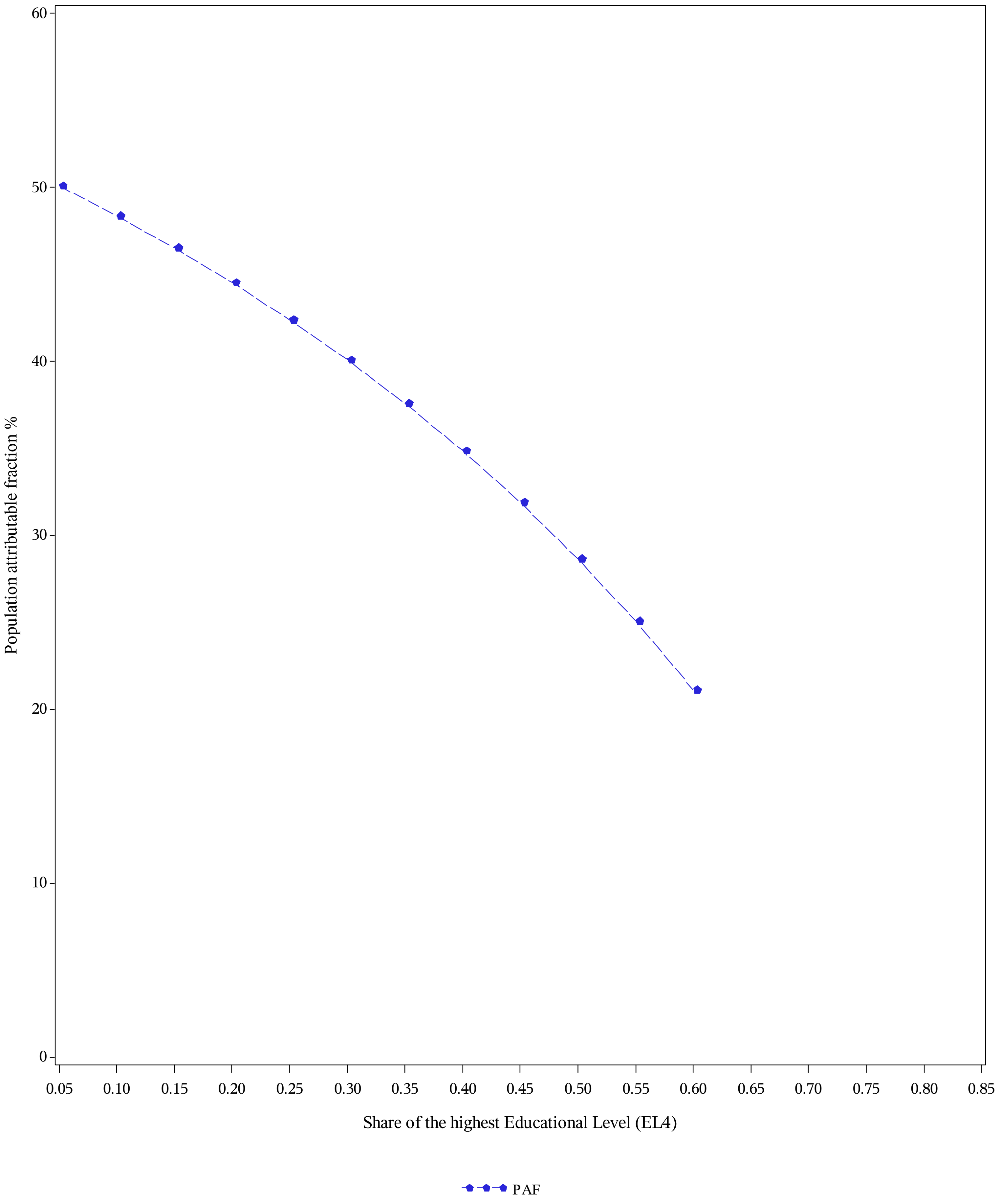

PAF in function of the share of EL4

When EL2 and EL3 are fixed at: EL2=15% ; EL3=25%
EL1 =1- EL4 - EL2 - EL3

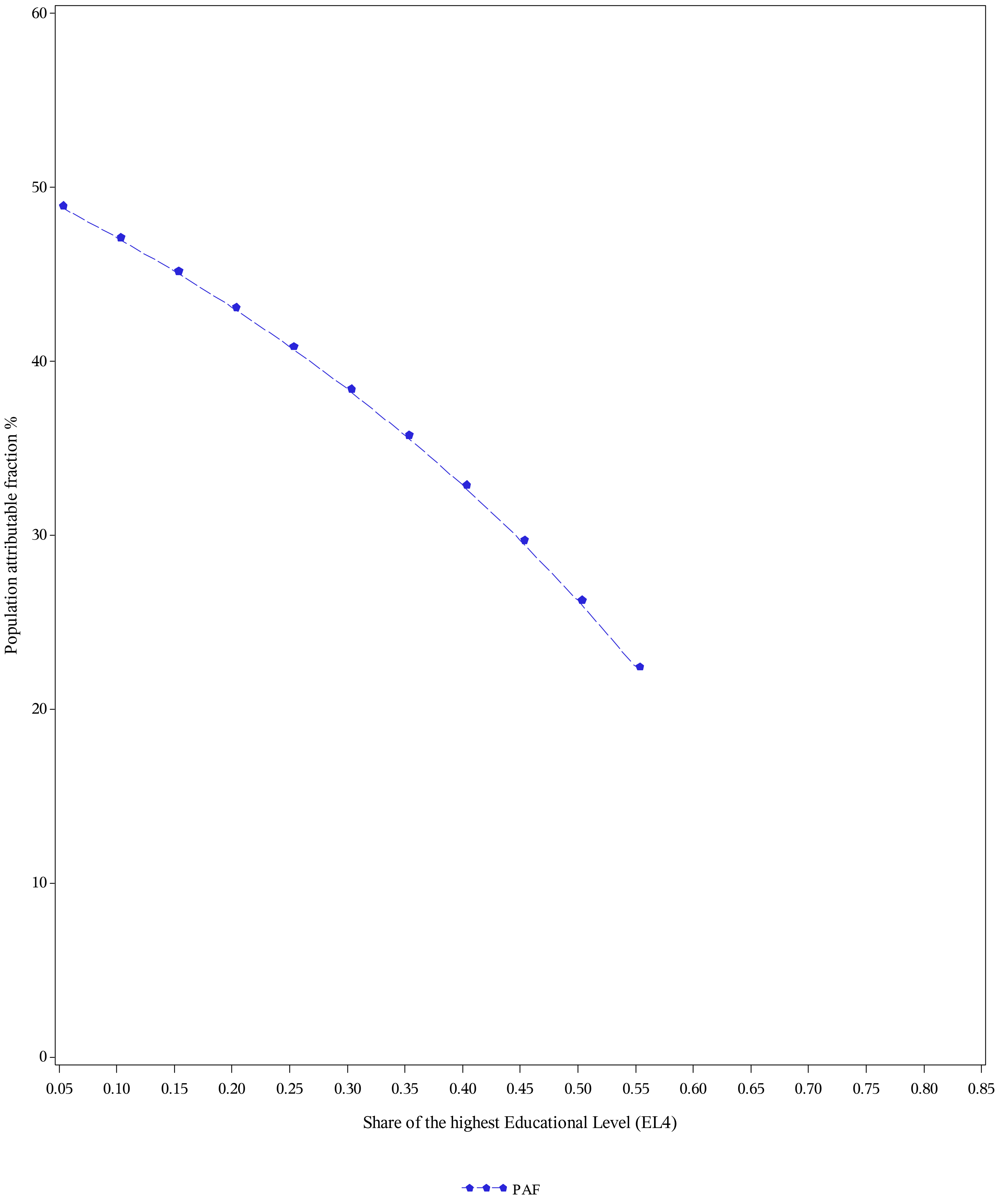

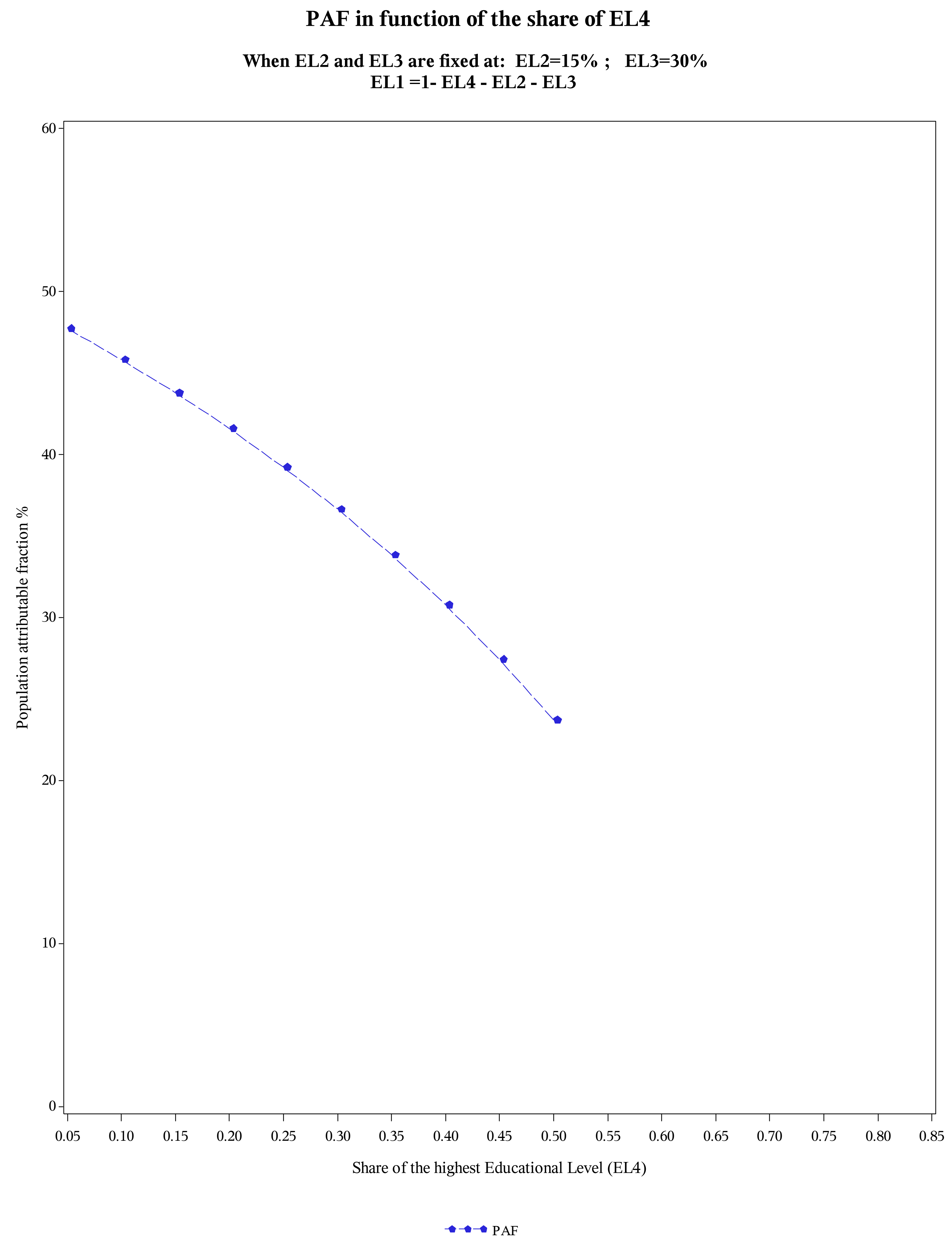
PAF in function of the share of EL4
When EL2 and EL3 are fixed at: EL2=15% ; EL3=30%
EL1 =1- EL4 - EL2 - EL3
Population attributable fraction %
0
10
20
30
40
50
60
0.05
0.10
0.15
0.20
0.25
0.30
0.35
0.40
0.45
0.50
0.55
0.60
0.65
0.70
0.75
0.80
0.85
Share of the highest Educational Level (EL4)
PAF

PAF in function of the share of EL4

When EL2 and EL3 are fixed at: EL2=15% ; EL3=35%
EL1 =1- EL4 - EL2 - EL3

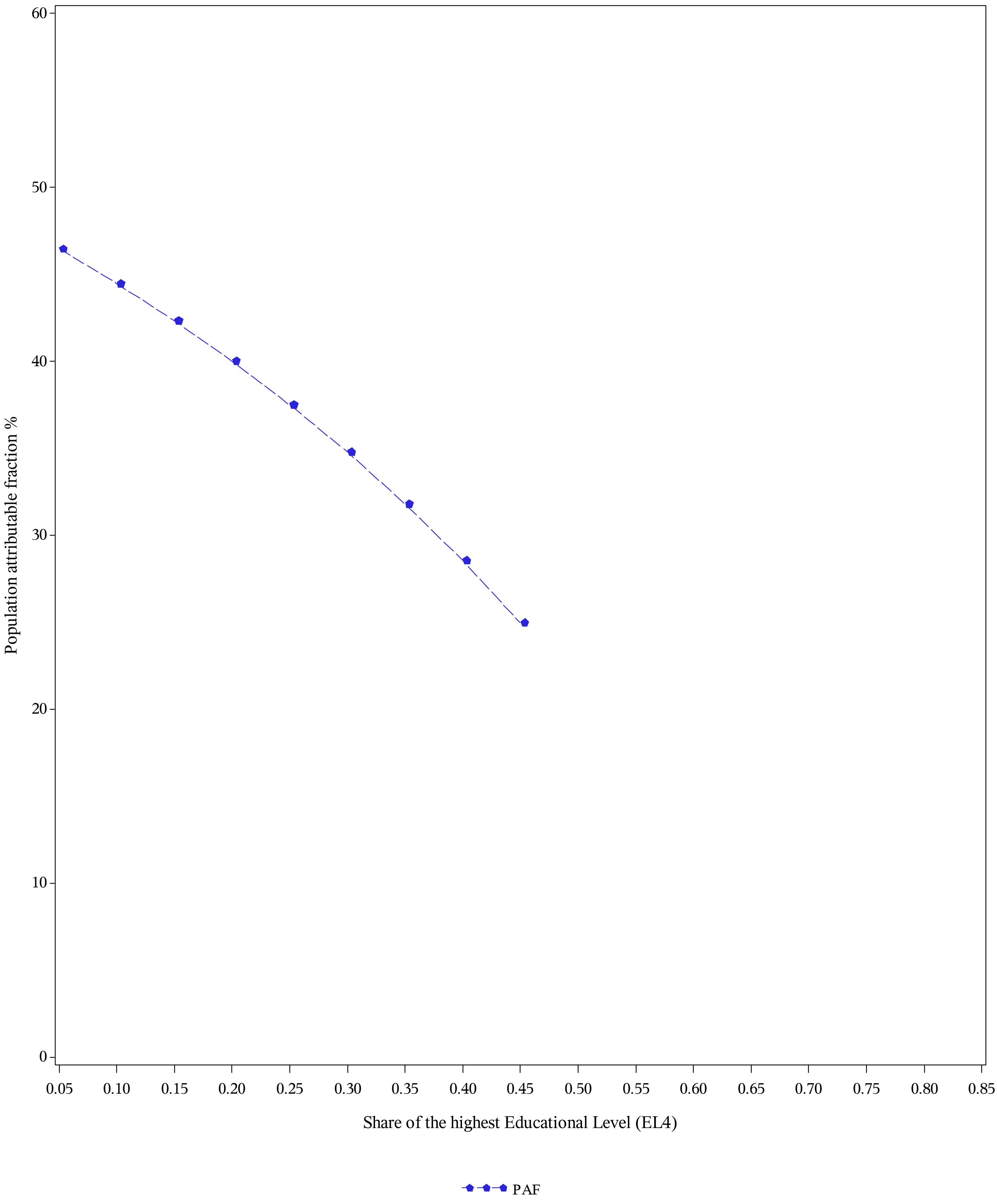

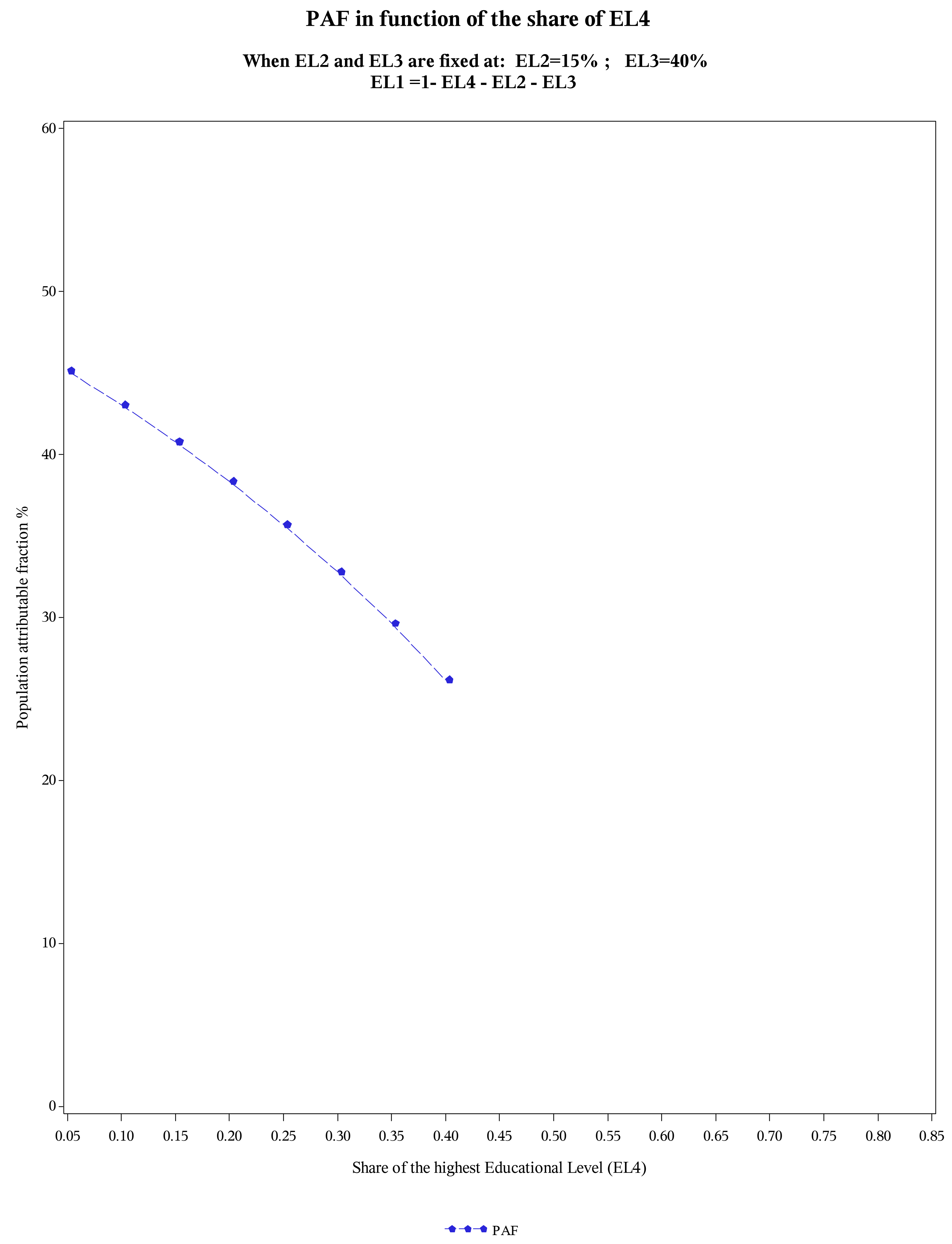
PAF in function of the share of EL4
When EL2 and EL3 are fixed at: EL2=15% ; EL3=40%
EL1 =1- EL4 - EL2 - EL3
Population attributable fraction %
0
10
20
30
40
50
60
0.05
0.10
0.15
0.20
0.25
0.30
0.35
0.40
0.45
0.50
0.55
0.60
0.65
0.70
0.75
0.80
0.85
Share of the highest Educational Level (EL4)
PAF

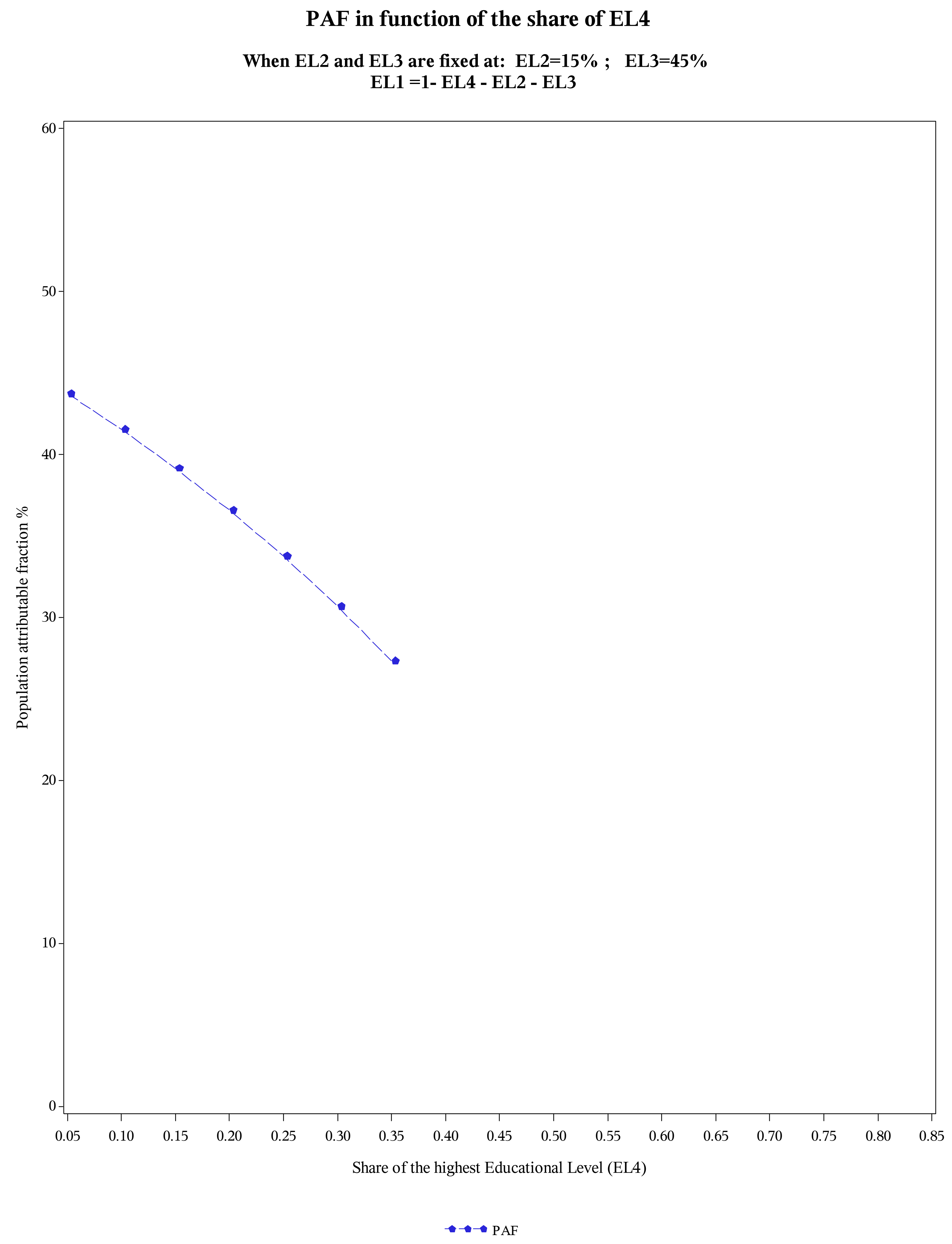
PAF in function of the share of EL4
When EL2 and EL3 are fixed at: EL2=15% ; EL3=45%
EL1 =1- EL4 - EL2 - EL3
Population attributable fraction %
0
10
20
30
40
50
60
0.05
0.10
0.15
0.20
0.25
0.30
0.35
0.40
0.45
0.50
0.55
0.60
0.65
0.70
0.75
0.80
0.85
Share of the highest Educational Level (EL4)
PAF

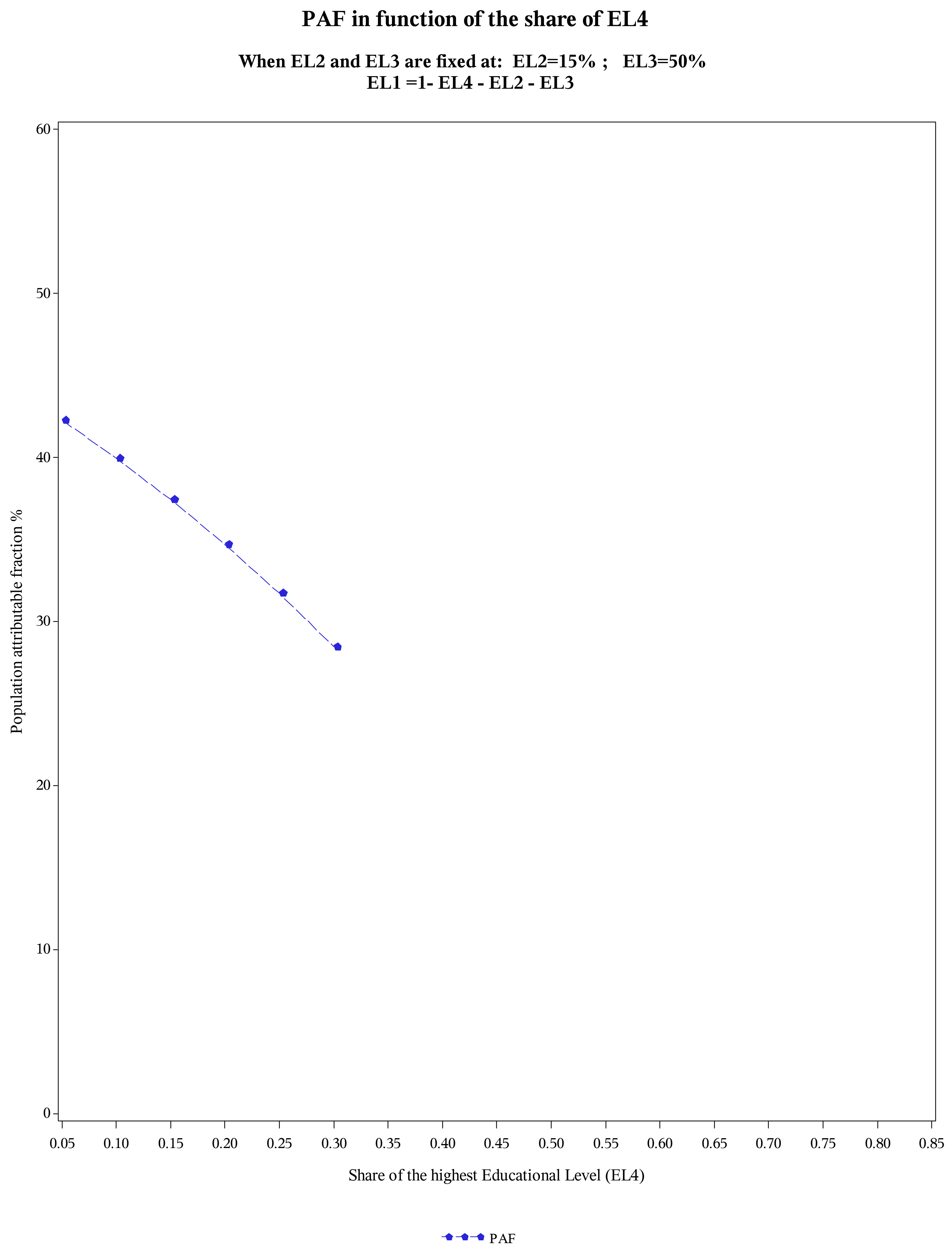
PAF in function of the share of EL4
When EL2 and EL3 are fixed at: EL2=15% ; EL3=50%
EL1 =1- EL4 - EL2 - EL3
Population attributable fraction %
0
10
20
30
40
50
60
0.05
0.10
0.15
0.20
0.25
0.30
0.35
0.40
0.45
0.50
0.55
0.60
0.65
0.70
0.75
0.80
0.85
Share of the highest Educational Level (EL4)
PAF

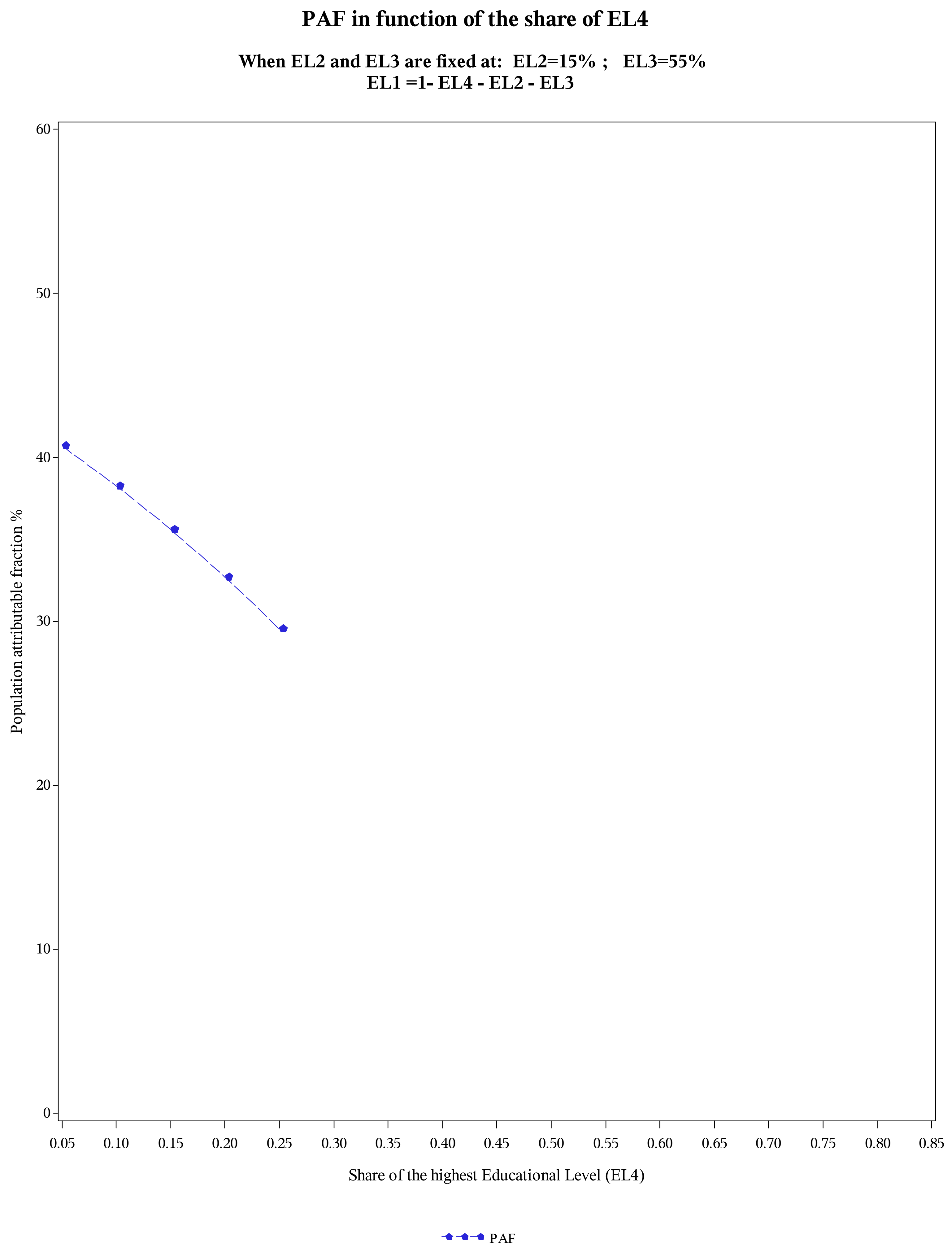
PAF in function of the share of EL4
When EL2 and EL3 are fixed at: EL2=15% ; EL3=55%
EL1 =1- EL4 - EL2 - EL3
Population attributable fraction %
0
10
20
30
40
50
60
0.05
0.10
0.15
0.20
0.25
0.30
0.35
0.40
0.45
0.50
0.55
0.60
0.65
0.70
0.75
0.80
0.85
Share of the highest Educational Level (EL4)
PAF

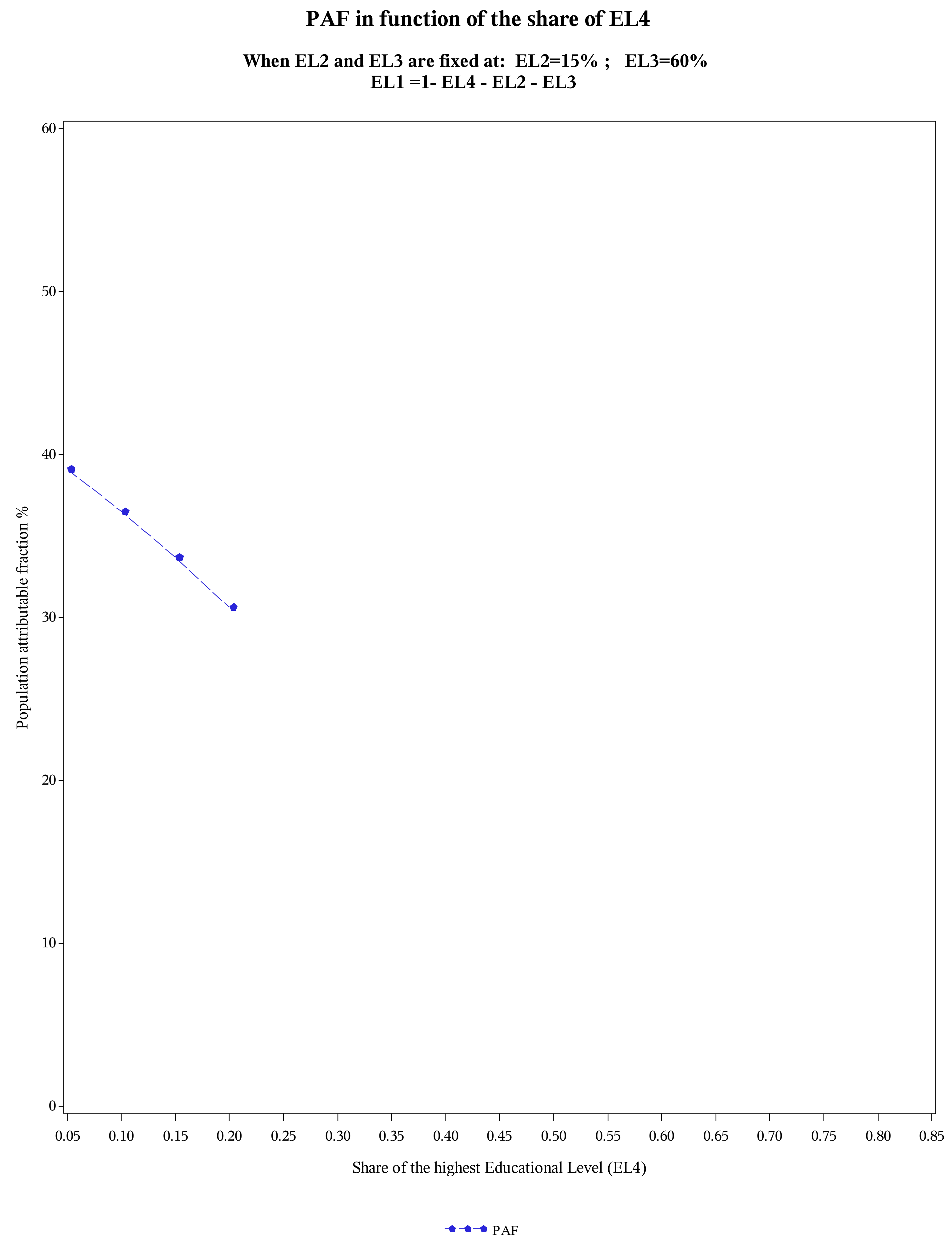
PAF in function of the share of EL4
When EL2 and EL3 are fixed at: EL2=15% ; EL3=60%
EL1 =1- EL4 - EL2 - EL3
Population attributable fraction %
0
10
20
30
40
50
60
0.05
0.10
0.15
0.20
0.25
0.30
0.35
0.40
0.45
0.50
0.55
0.60
0.65
0.70
0.75
0.80
0.85
Share of the highest Educational Level (EL4)
PAF

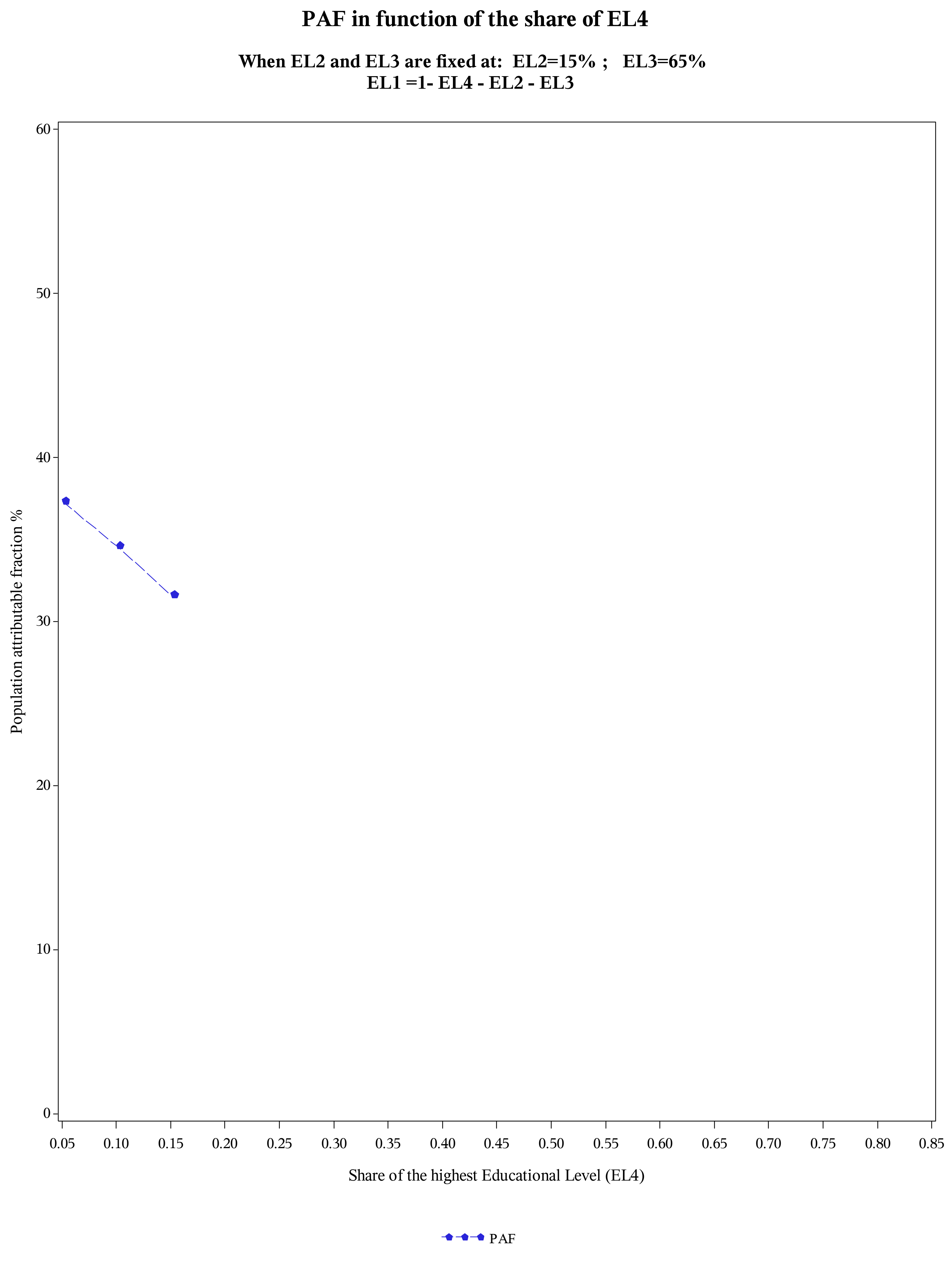
PAF in function of the share of EL4
When EL2 and EL3 are fixed at: EL2=15% ; EL3=65%
EL1 =1- EL4 - EL2 - EL3
Population attributable fraction %
0
10
20
30
40
50
60
0.05
0.10
0.15
0.20
0.25
0.30
0.35
0.40
0.45
0.50
0.55
0.60
0.65
0.70
0.75
0.80
0.85
Share of the highest Educational Level (EL4)
PAF

PAF in function of the share of EL4

When EL2 and EL3 are fixed at: EL2=15% ;   EL3=70%
EL1 =1- EL4 - EL2 - EL3

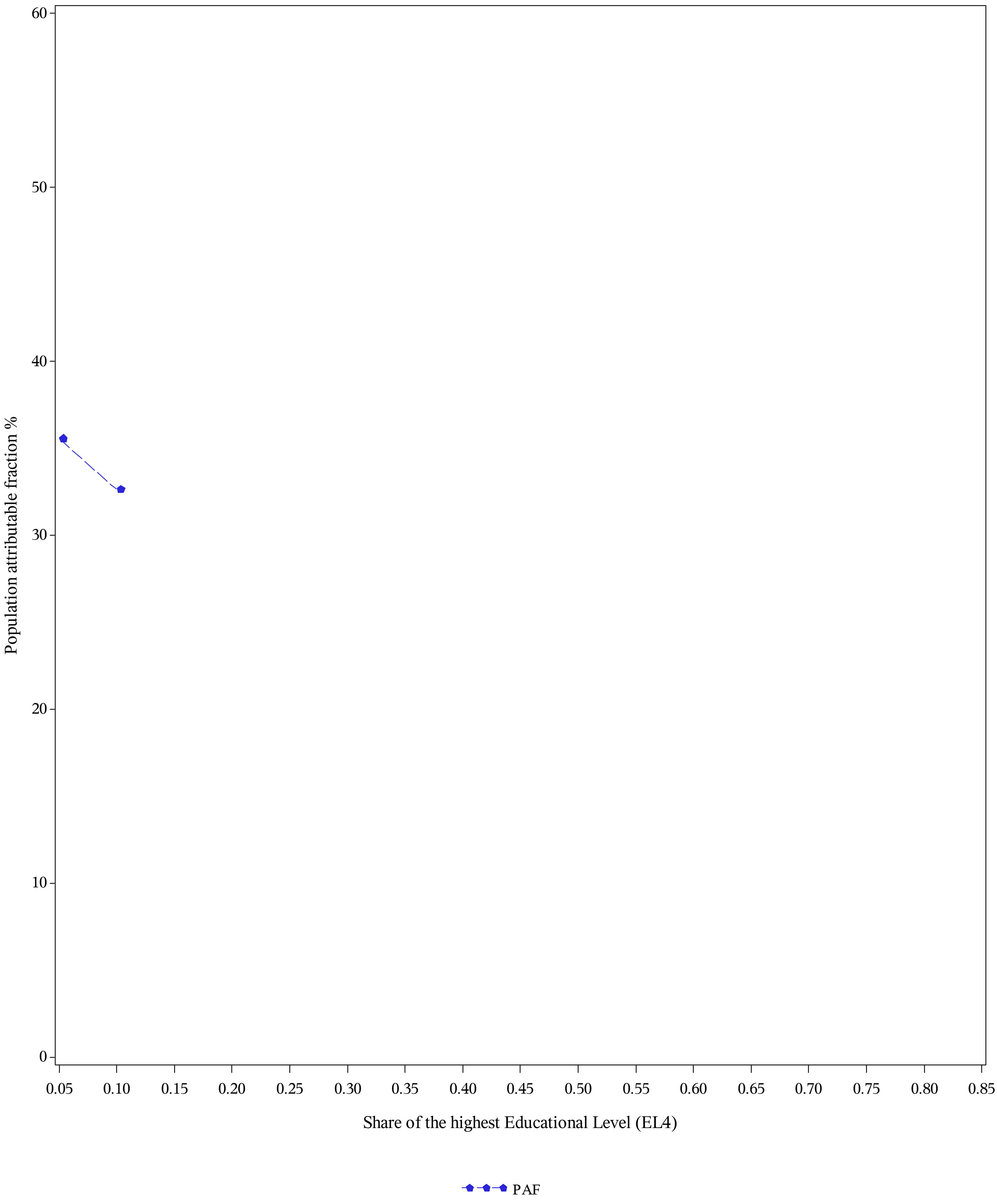

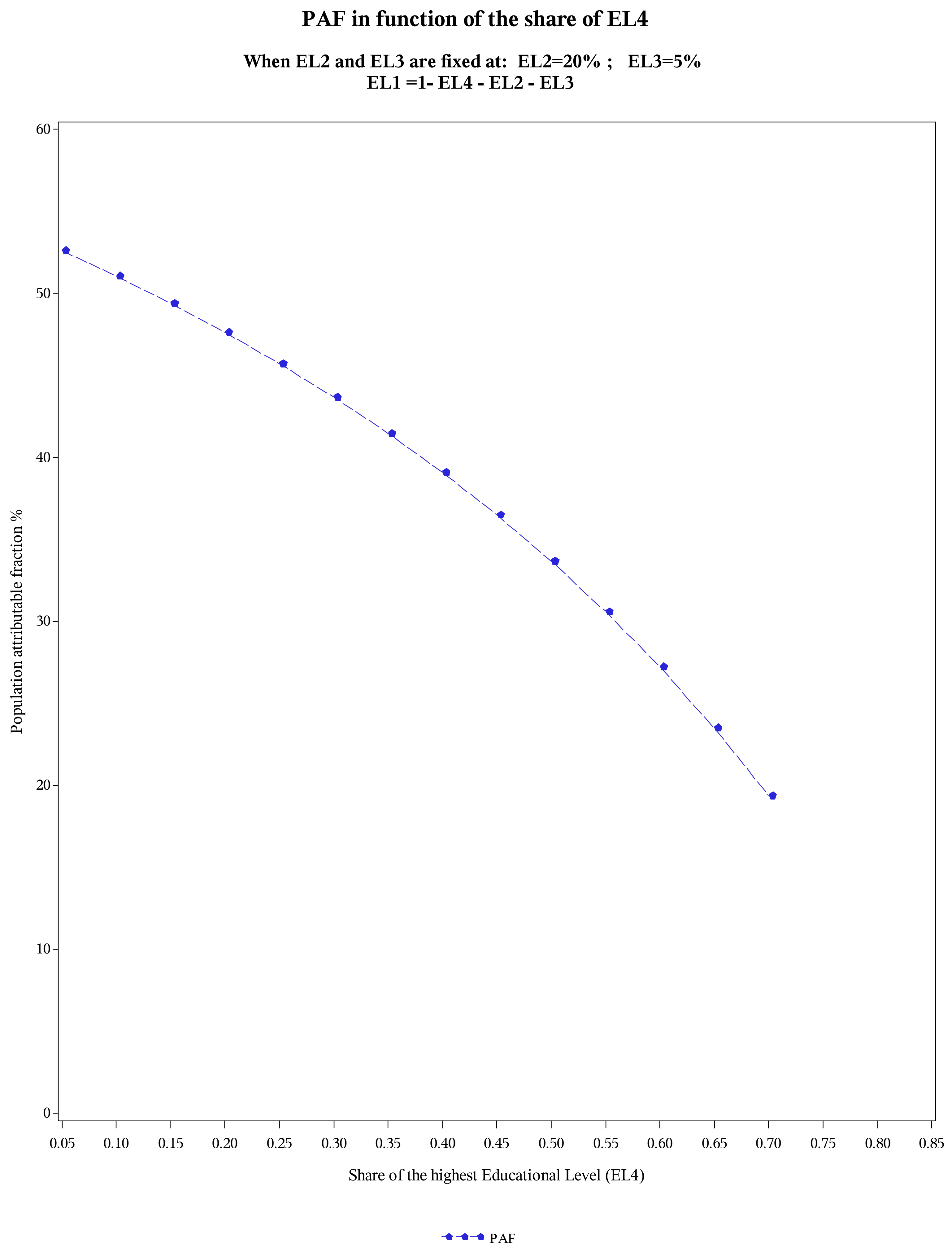
PAF in function of the share of EL4
When EL2 and EL3 are fixed at: EL2=20% ; EL3=5%
EL1 =1- EL4 - EL2 - EL3
Population attributable fraction %
0
10
20
30
40
50
60
0.05
0.10
0.15
0.20
0.25
0.30
0.35
0.40
0.45
0.50
0.55
0.60
0.65
0.70
0.75
0.80
0.85
Share of the highest Educational Level (EL4)
PAF

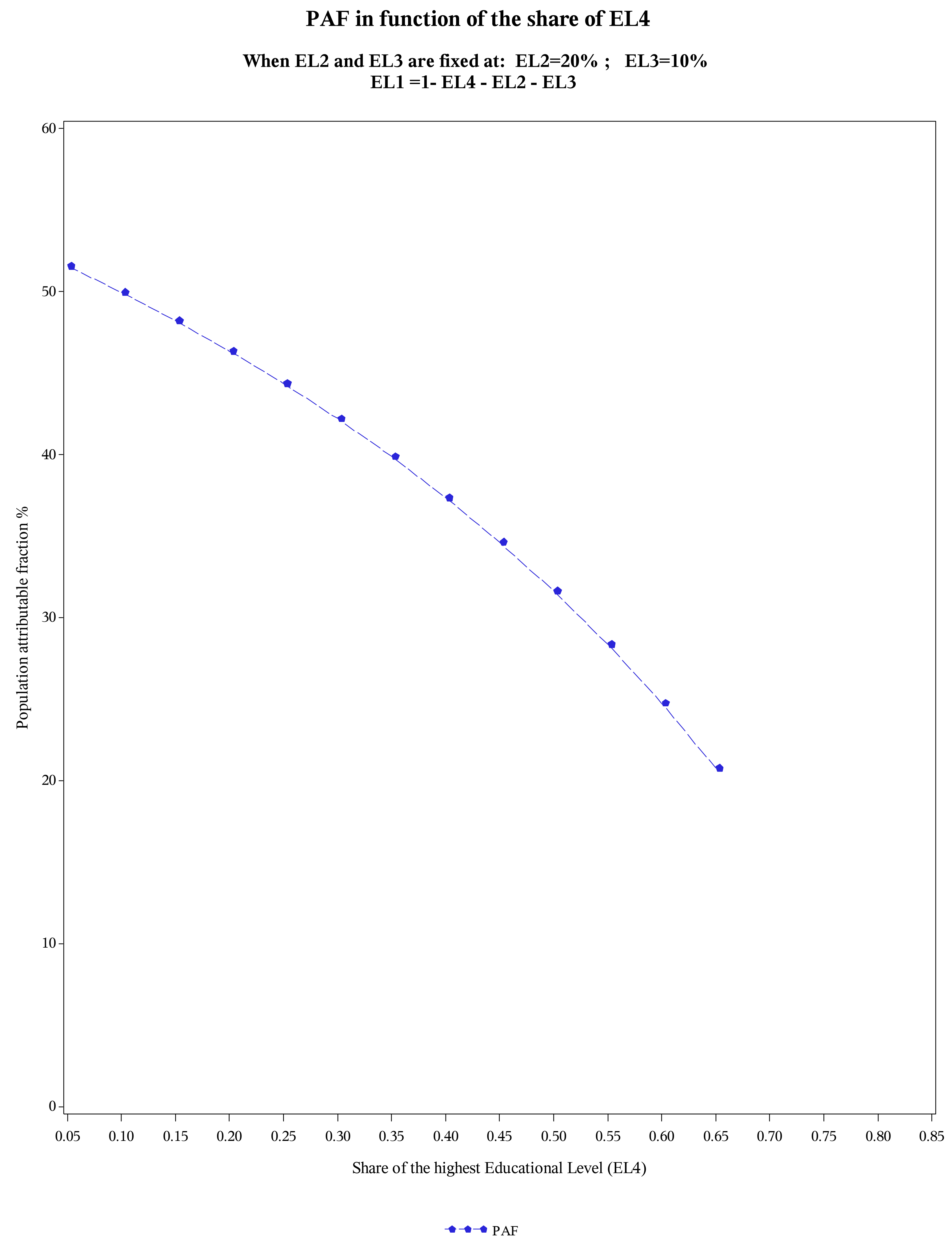
PAF in function of the share of EL4
When EL2 and EL3 are fixed at: EL2=20% ; EL3=10%
EL1 =1- EL4 - EL2 - EL3
Population attributable fraction %
0
10
20
30
40
50
60
0.05
0.10
0.15
0.20
0.25
0.30
0.35
0.40
0.45
0.50
0.55
0.60
0.65
0.70
0.75
0.80
0.85
Share of the highest Educational Level (EL4)
PAF

PAF in function of the share of EL4

When EL2 and EL3 are fixed at: EL2=20% ; EL3=15%
EL1 =1- EL4 - EL2 - EL3

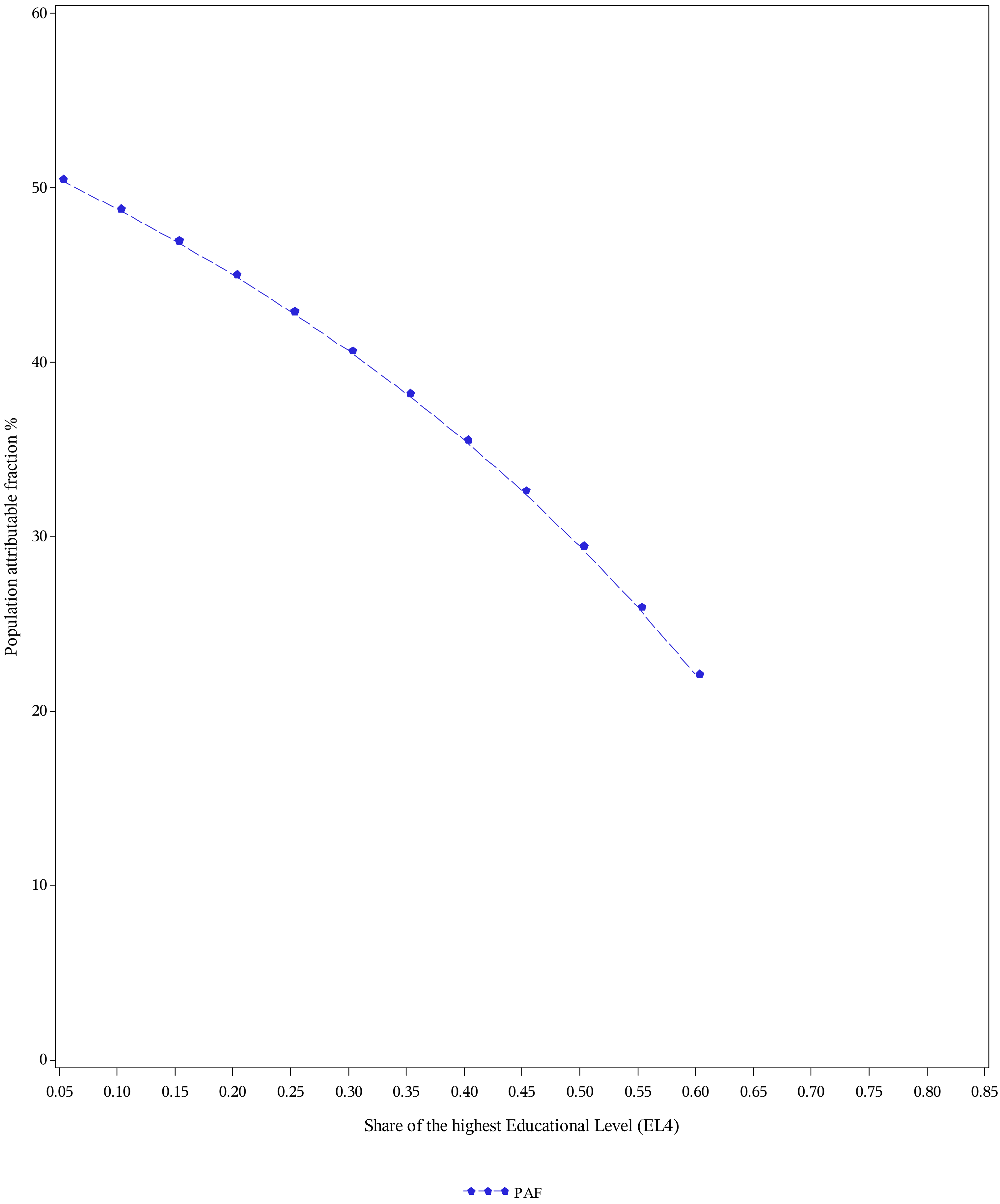

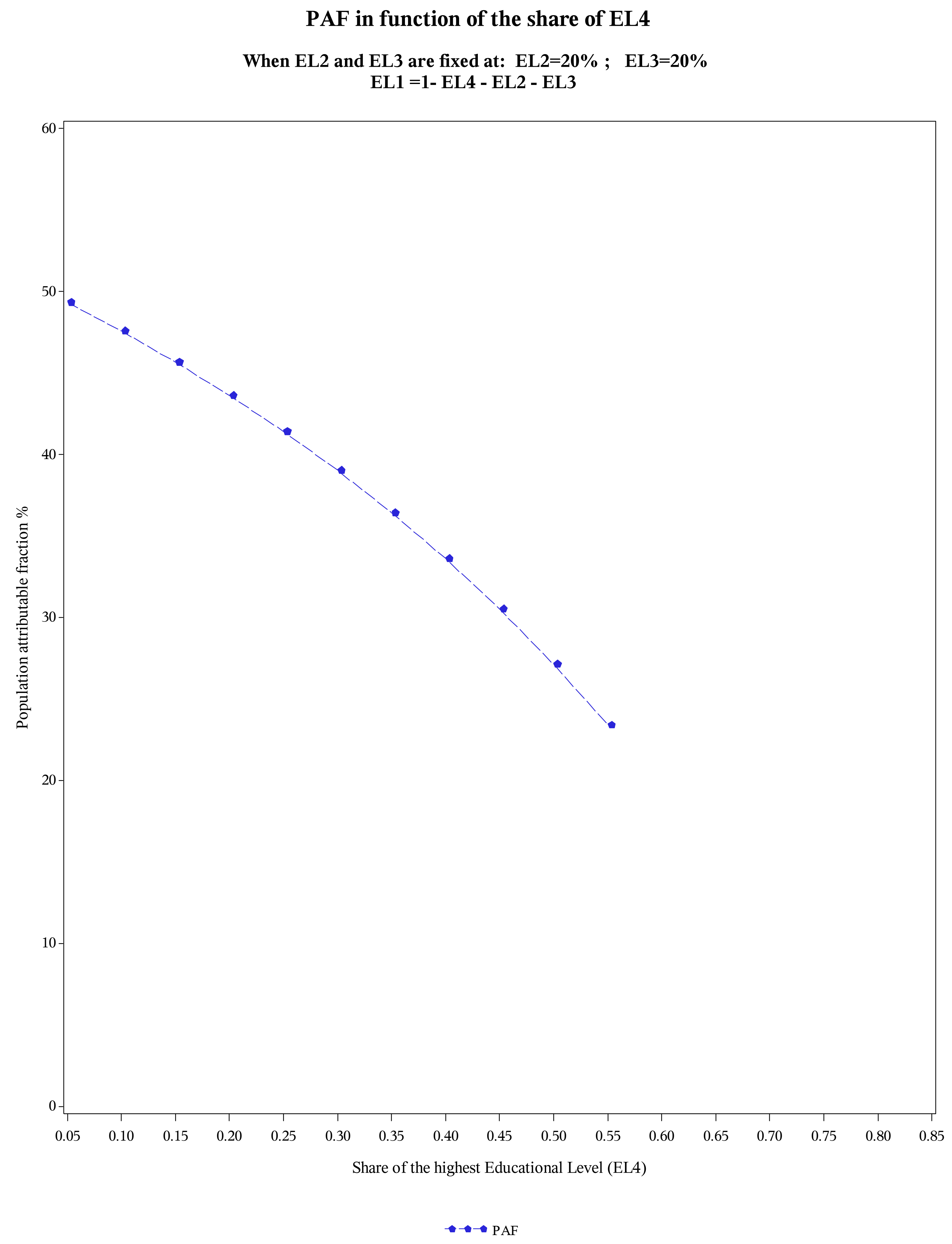
PAF in function of the share of EL4
When EL2 and EL3 are fixed at: EL2=20% ; EL3=20%
EL1 =1- EL4 - EL2 - EL3
Population attributable fraction %
0
10
20
30
40
50
60
0.05
0.10
0.15
0.20
0.25
0.30
0.35
0.40
0.45
0.50
0.55
0.60
0.65
0.70
0.75
0.80
0.85
Share of the highest Educational Level (EL4)
PAF

PAF in function of the share of EL4

When EL2 and EL3 are fixed at: EL2=20% ; EL3=25%
EL1 =1- EL4 - EL2 - EL3

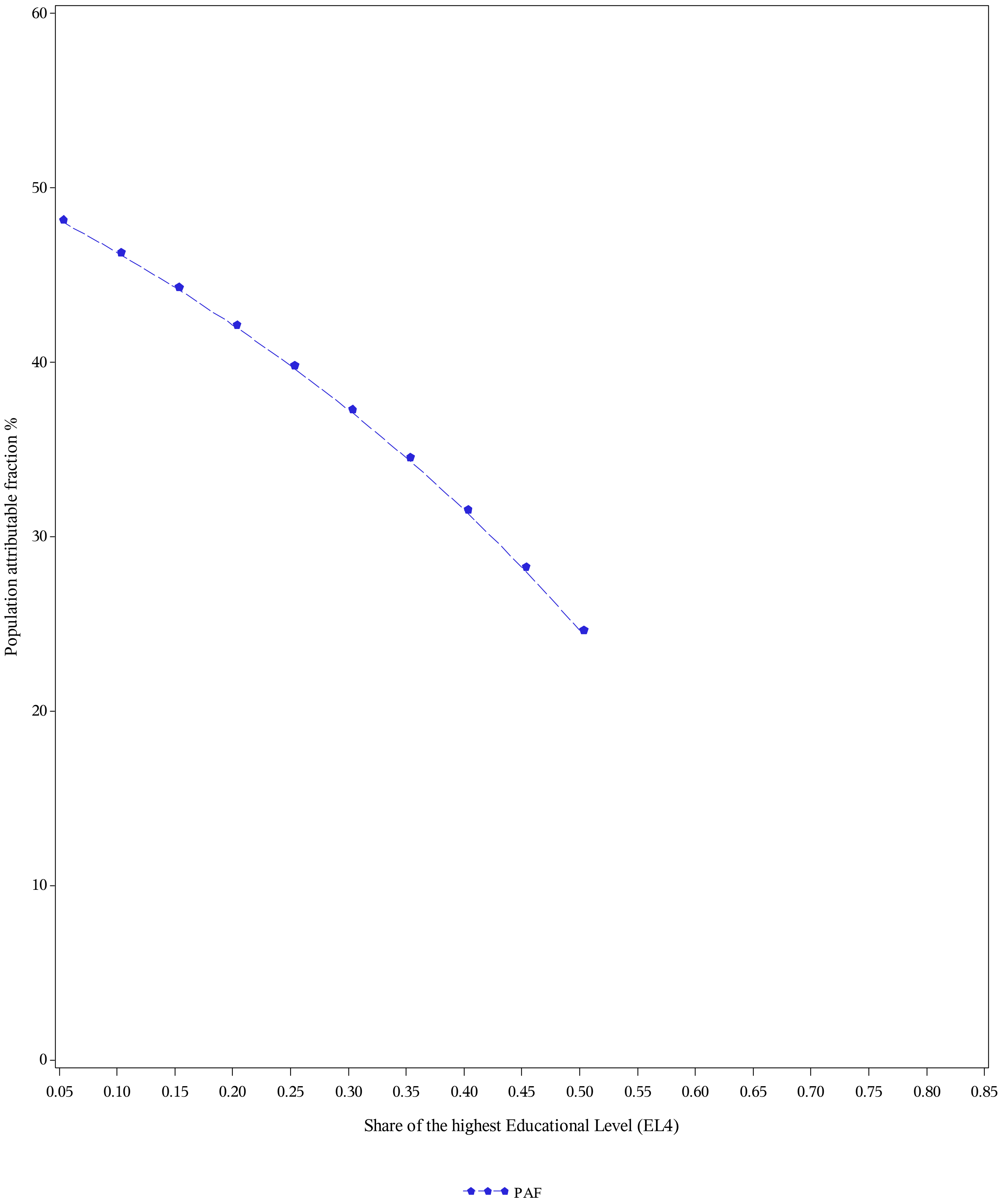

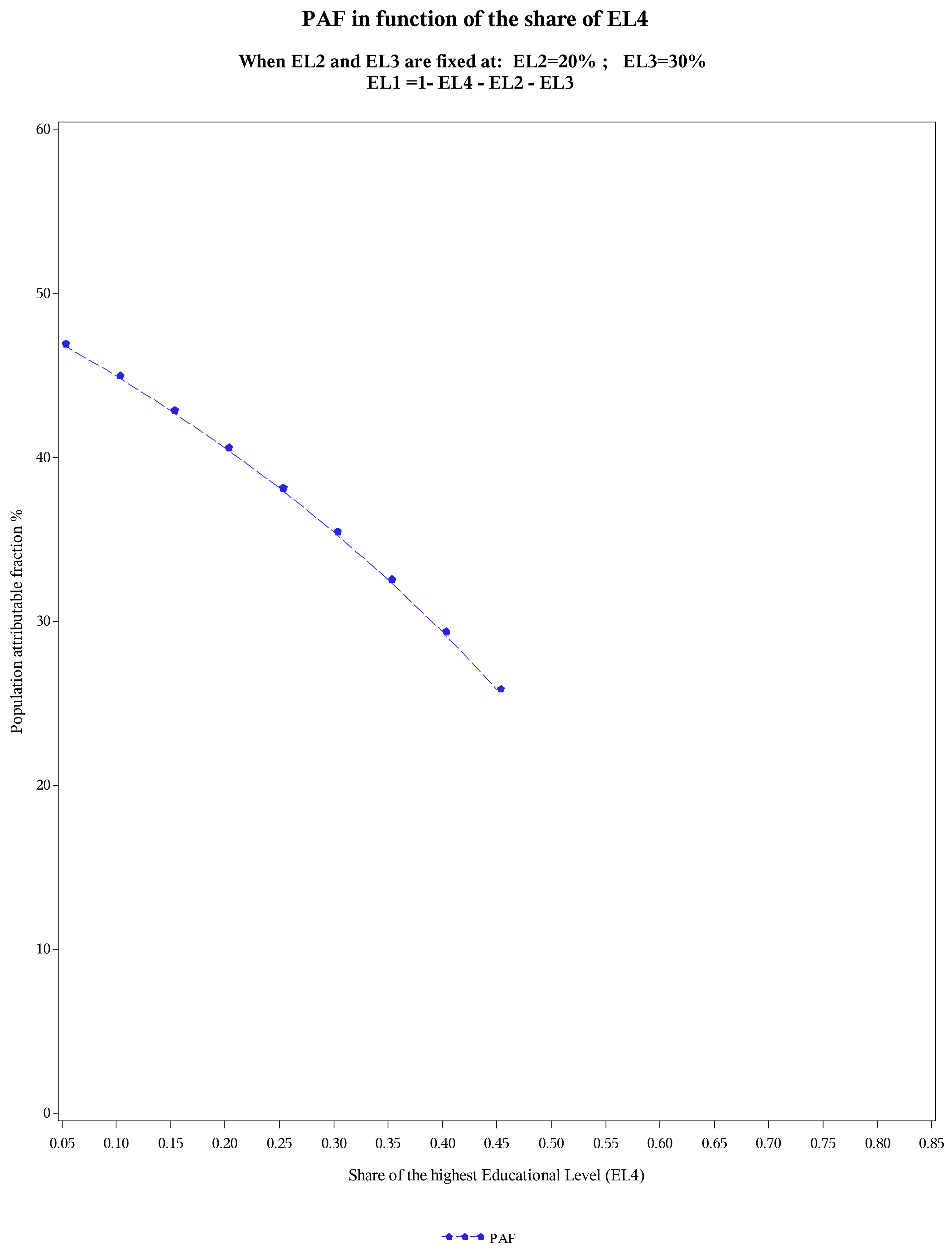
PAF in function of the share of EL4
When EL2 and EL3 are fixed at: EL2=20% ; EL3=30%
EL1 =1- EL4 - EL2 - EL3
Population attributable fraction %
0
10
20
30
40
50
60
0.05
0.10
0.15
0.20
0.25
0.30
0.35
0.40
0.45
0.50
0.55
0.60
0.65
0.70
0.75
0.80
0.85
Share of the highest Educational Level (EL4)
PAF

PAF in function of the share of EL4

When EL2 and EL3 are fixed at: EL2=20% ; EL3=35%
EL1 =1- EL4 - EL2 - EL3

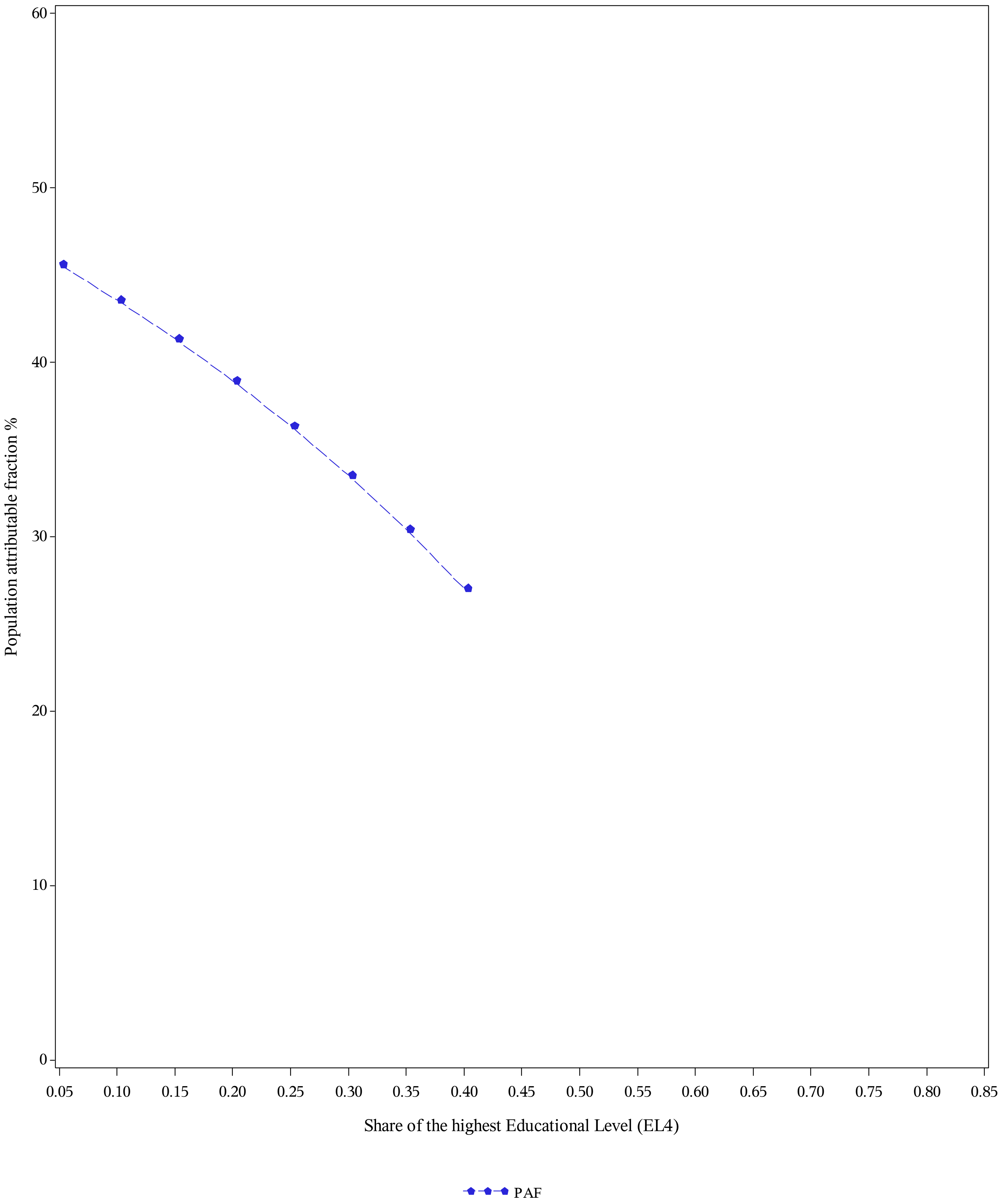

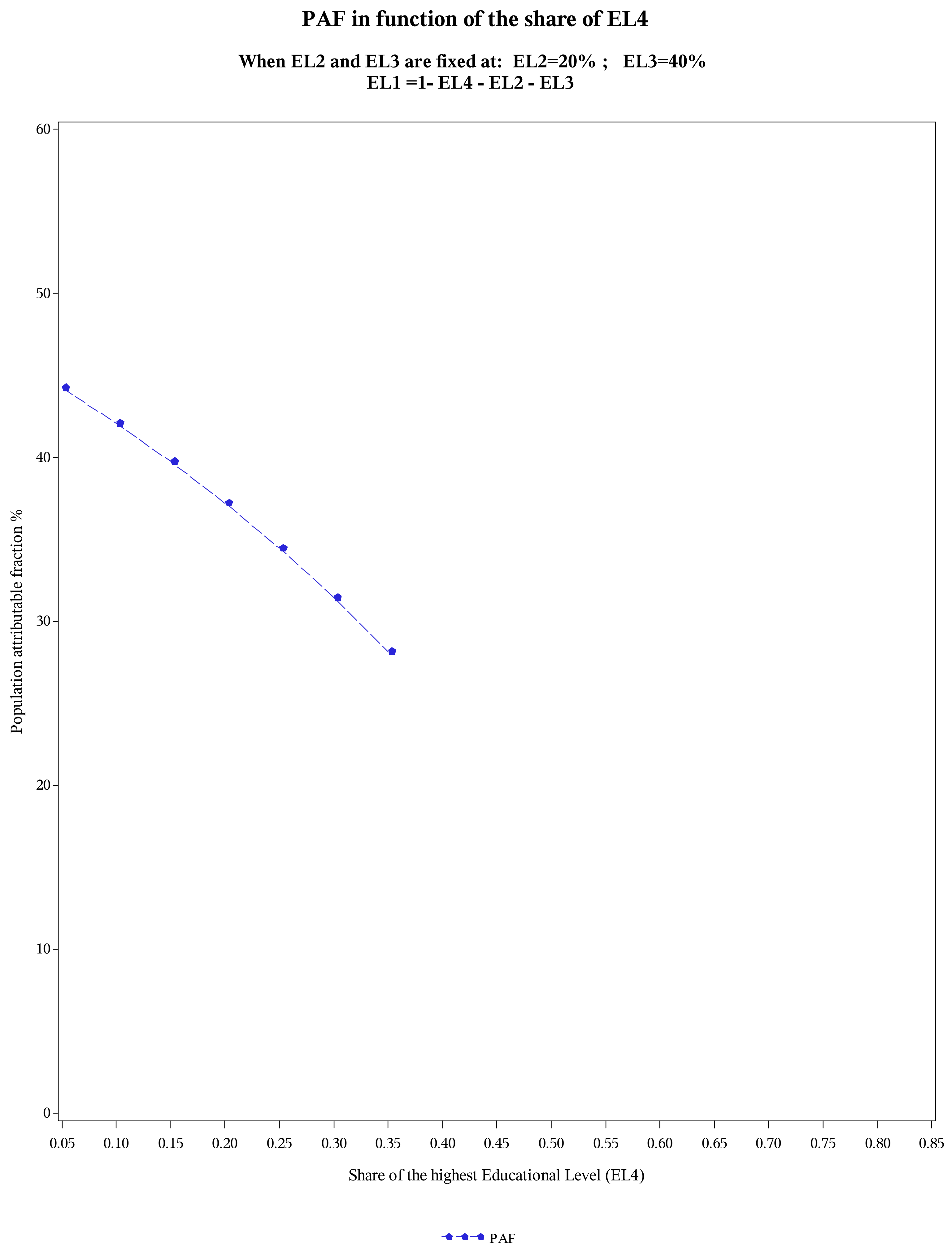
PAF in function of the share of EL4
When EL2 and EL3 are fixed at: EL2=20% ; EL3=40%
EL1 =1- EL4 - EL2 - EL3
Population attributable fraction %
0
10
20
30
40
50
60
0.05
0.10
0.15
0.20
0.25
0.30
0.35
0.40
0.45
0.50
0.55
0.60
0.65
0.70
0.75
0.80
0.85
Share of the highest Educational Level (EL4)
PAF

PAF in function of the share of EL4

When EL2 and EL3 are fixed at: EL2=20% ; EL3=45%
EL1 =1- EL4 - EL2 - EL3

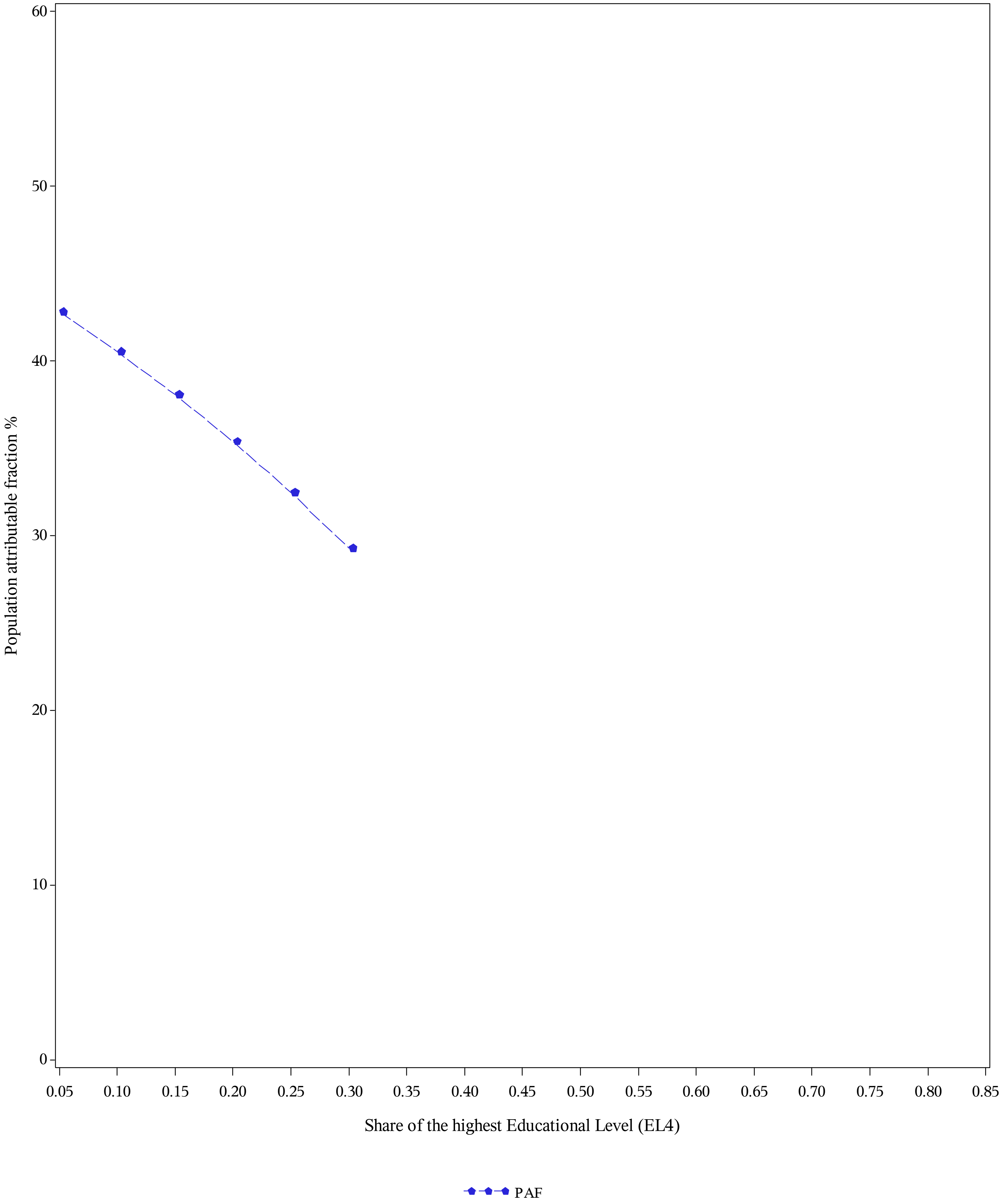

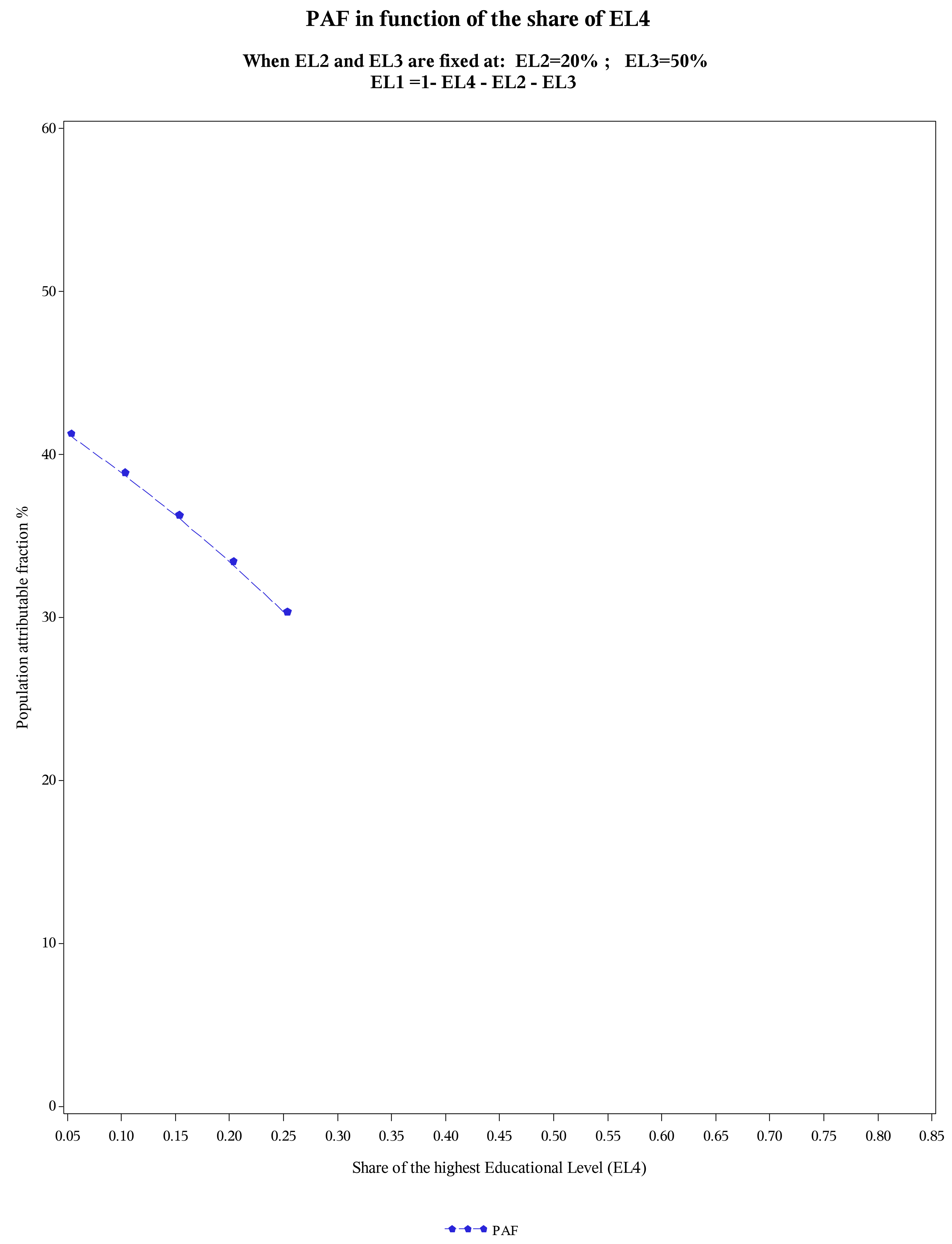
PAF in function of the share of EL4
When EL2 and EL3 are fixed at: EL2=20% ; EL3=50%
EL1 =1- EL4 - EL2 - EL3
Population attributable fraction %
0
10
20
30
40
50
60
0.05
0.10
0.15
0.20
0.25
0.30
0.35
0.40
0.45
0.50
0.55
0.60
0.65
0.70
0.75
0.80
0.85
Share of the highest Educational Level (EL4)
PAF

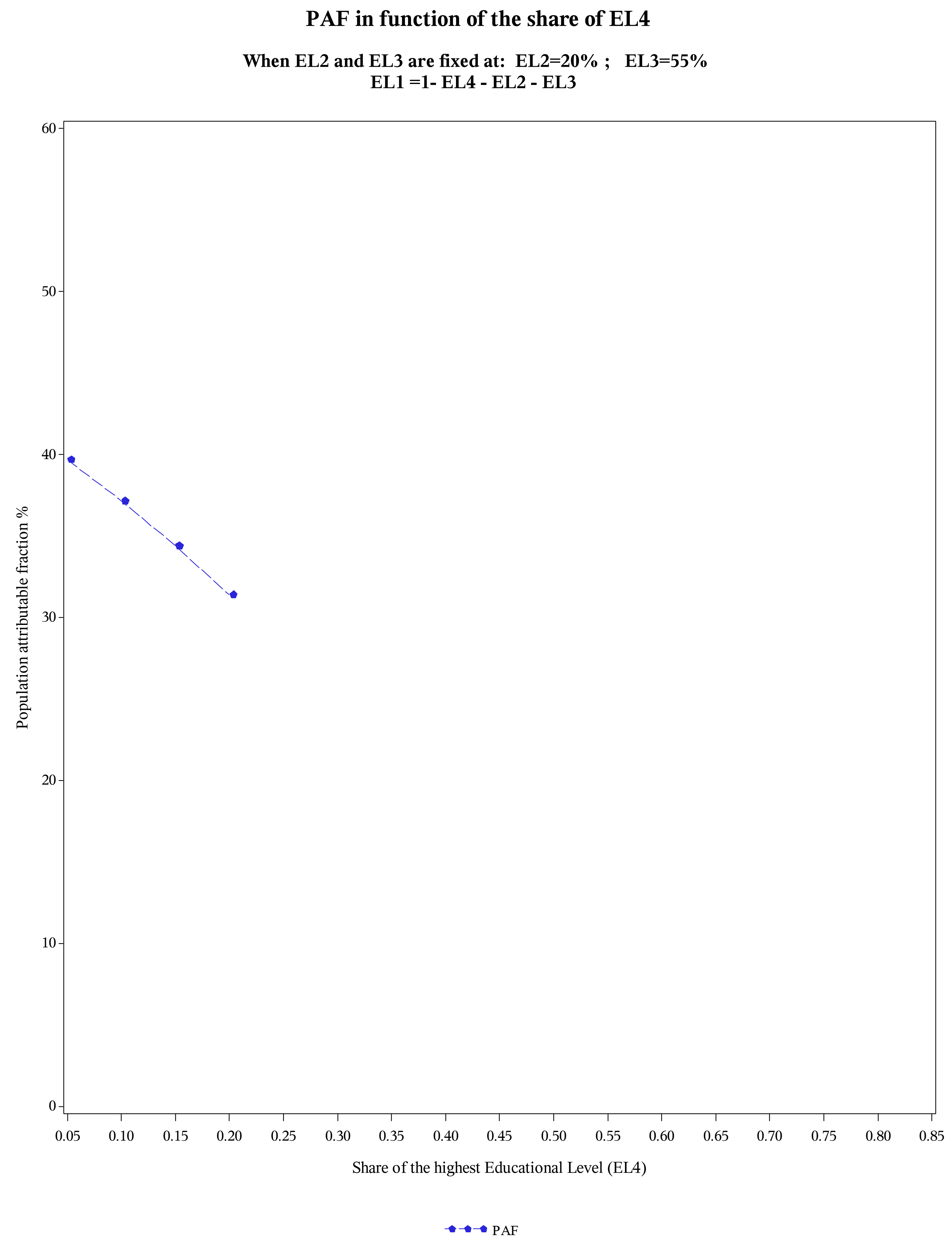
PAF in function of the share of EL4
When EL2 and EL3 are fixed at: EL2=20% ; EL3=55%
EL1 =1- EL4 - EL2 - EL3
Population attributable fraction %
0
10
20
30
40
50
60
0.05
0.10
0.15
0.20
0.25
0.30
0.35
0.40
0.45
0.50
0.55
0.60
0.65
0.70
0.75
0.80
0.85
Share of the highest Educational Level (EL4)
PAF

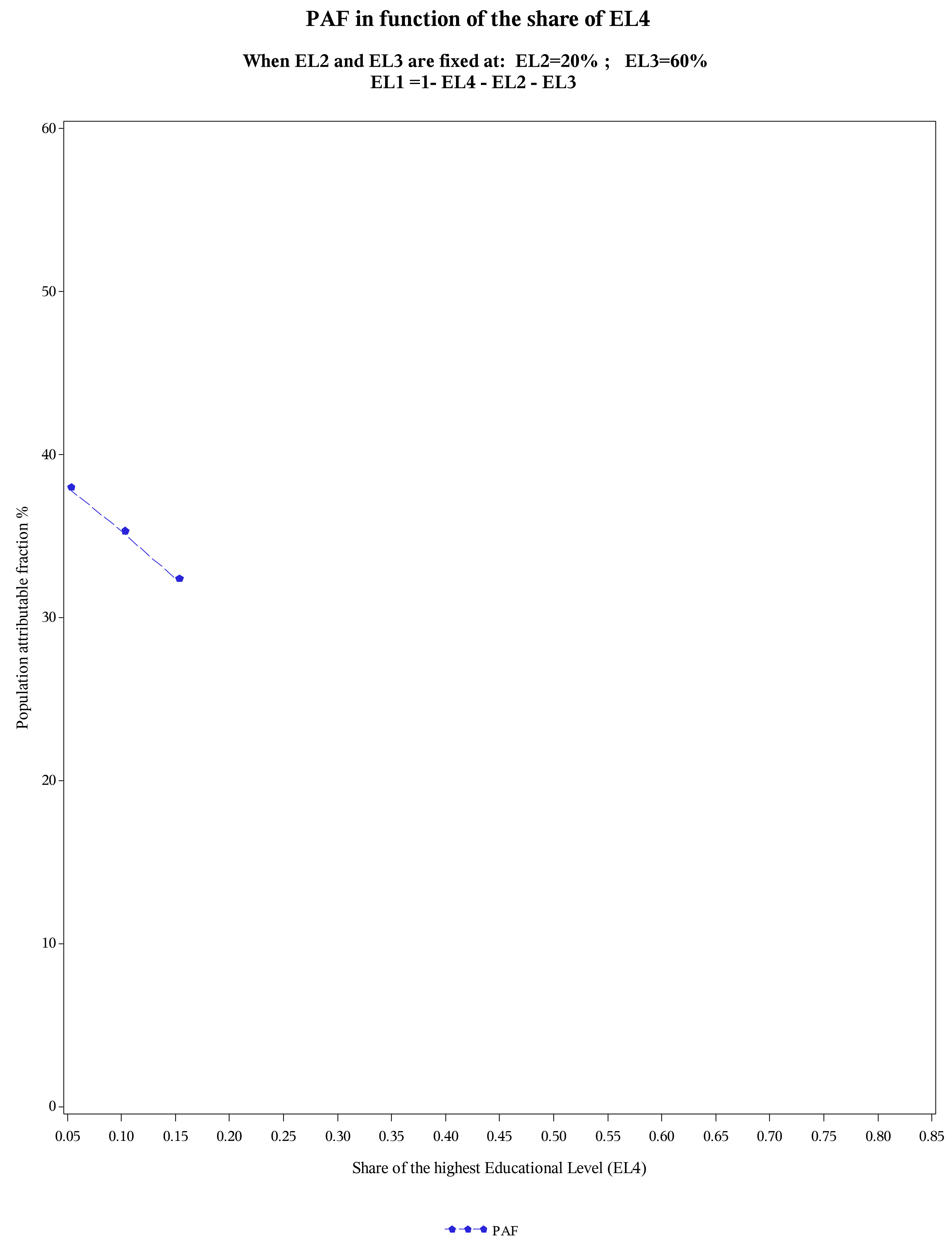
PAF in function of the share of EL4
When EL2 and EL3 are fixed at: EL2=20% ; EL3=60%
EL1 =1- EL4 - EL2 - EL3
Population attributable fraction %
0
10
20
30
40
50
60
0.05
0.10
0.15
0.20
0.25
0.30
0.35
0.40
0.45
0.50
0.55
0.60
0.65
0.70
0.75
0.80
0.85
Share of the highest Educational Level (EL4)
PAF

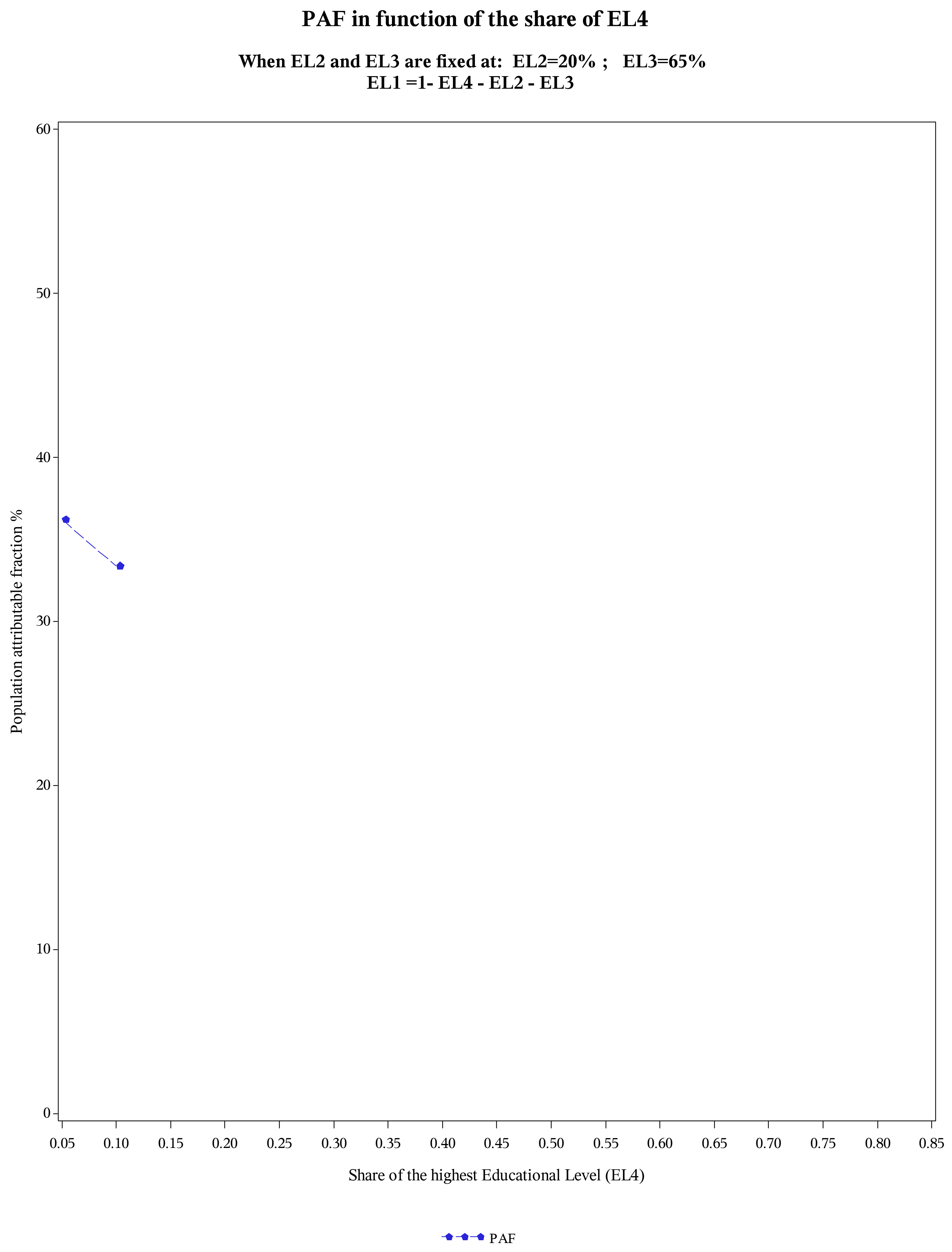

PAF in function of the share of EL4
When EL2 and EL3 are fixed at: EL2=20% ; EL3=65%
EL1 =1- EL4 - EL2 - EL3
Population attributable fraction %
0
10
20
30
40
50
60
0.05
0.10
0.15
0.20
0.25
0.30
0.35
0.40
0.45
0.50
0.55
0.60
0.65
0.70
0.75
0.80
0.85
Share of the highest Educational Level (EL4)
PAF

PAF in function of the share of EL4

When EL2 and EL3 are fixed at: EL2=20% ; EL3=70%
EL1 =1- EL4 - EL2 - EL3

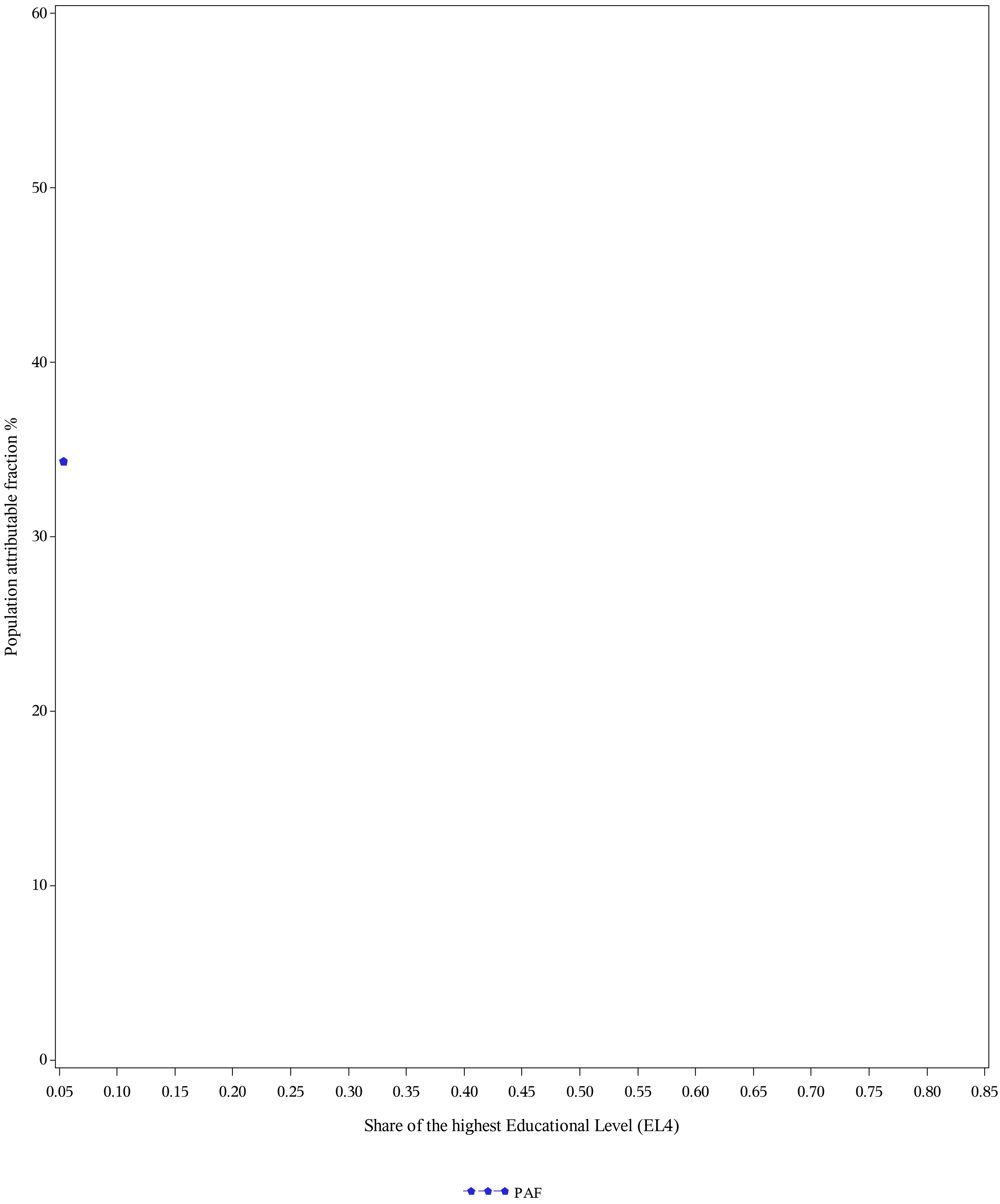

PAF in function of the share of EL4

When EL2 and EL3 are fixed at: EL2=25% ; EL3=5%
EL1 =1- EL4 - EL2 - EL3

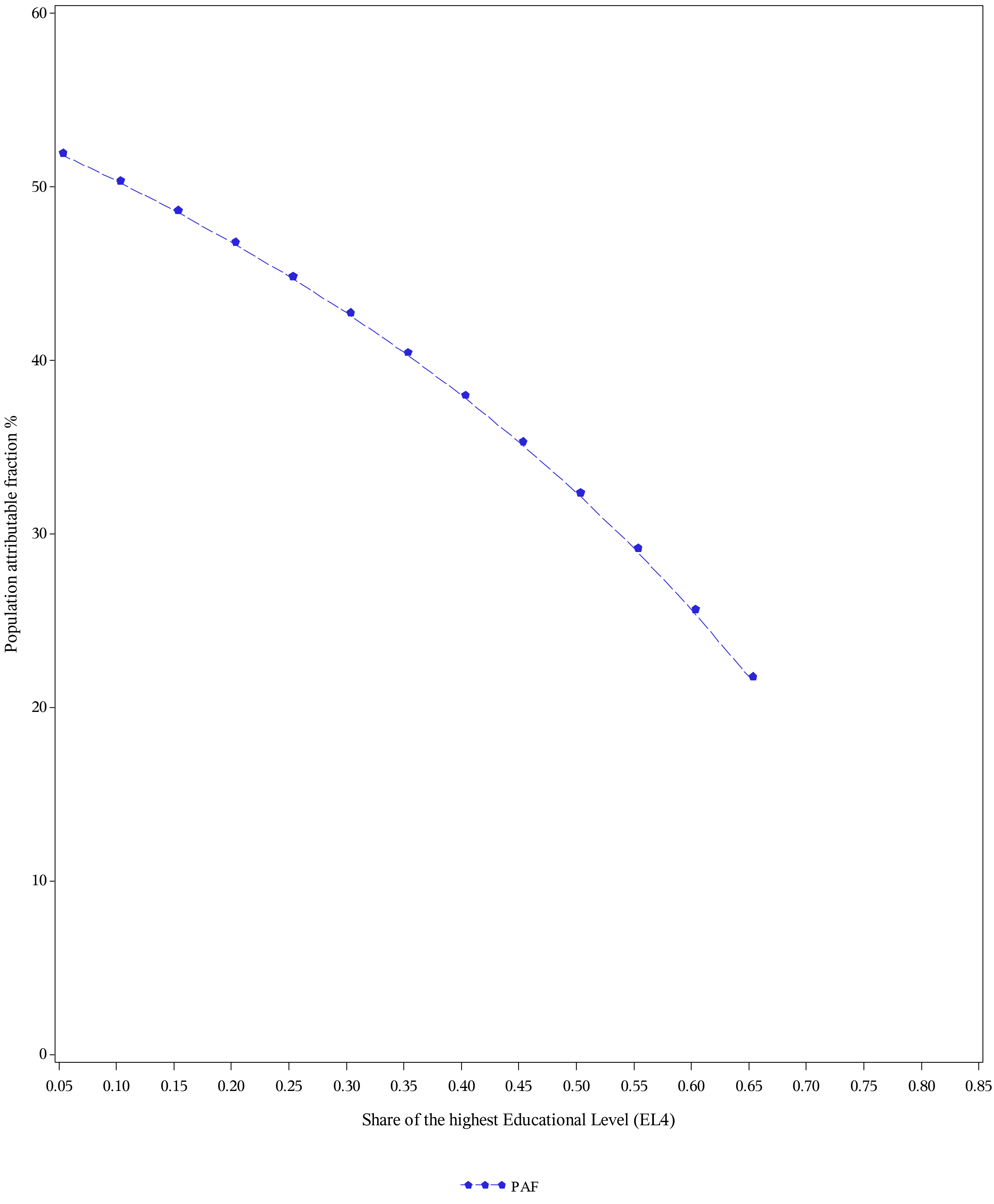

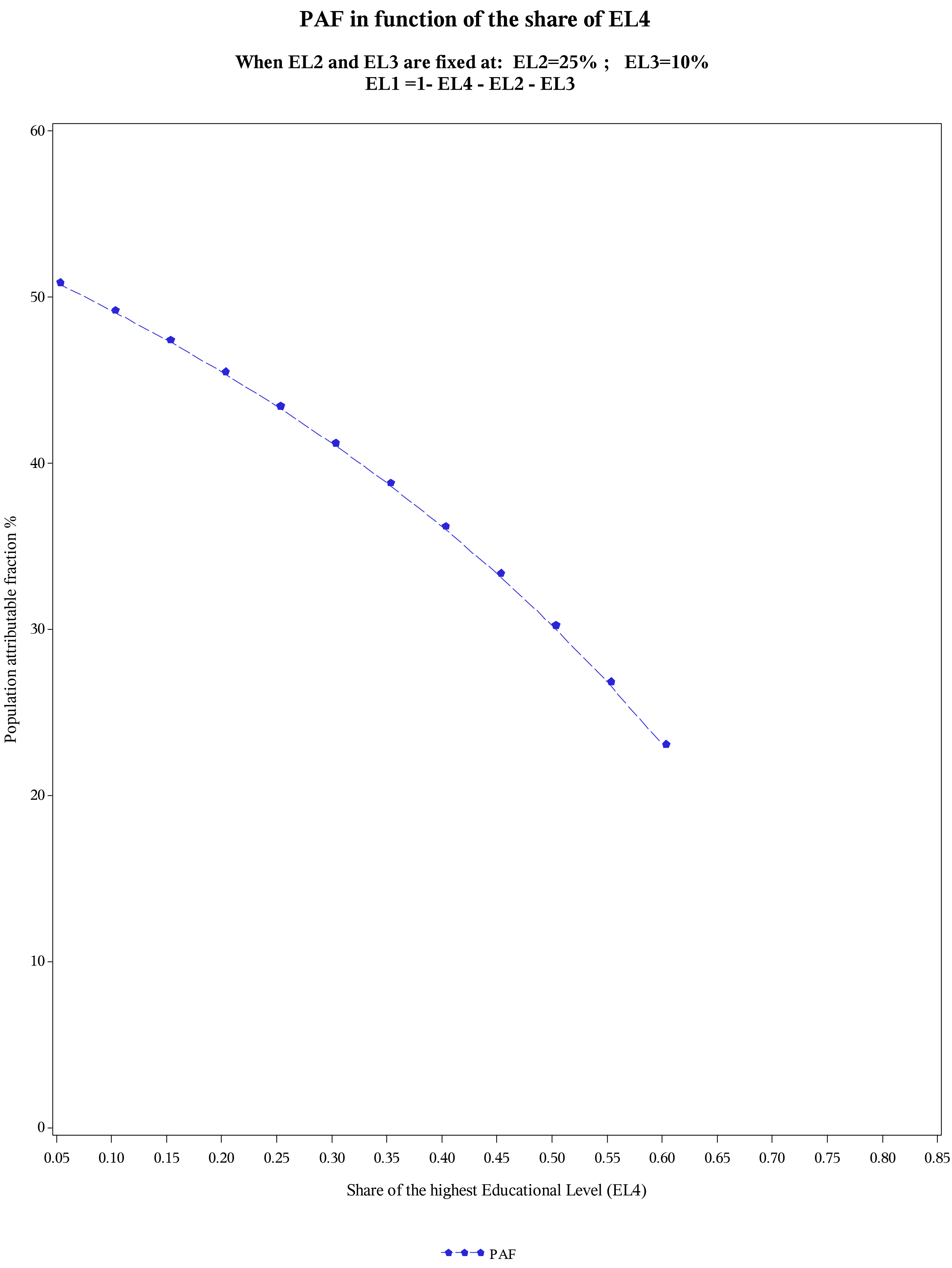
PAF in function of the share of EL4
When EL2 and EL3 are fixed at: EL2=25% ; EL3=10%
EL1 =1- EL4 - EL2 - EL3
Population attributable fraction %
0
10
20
30
40
50
60
0.05
0.10
0.15
0.20
0.25
0.30
0.35
0.40
0.45
0.50
0.55
0.60
0.65
0.70
0.75
0.80
0.85
Share of the highest Educational Level (EL4)
PAF

PAF in function of the share of EL4

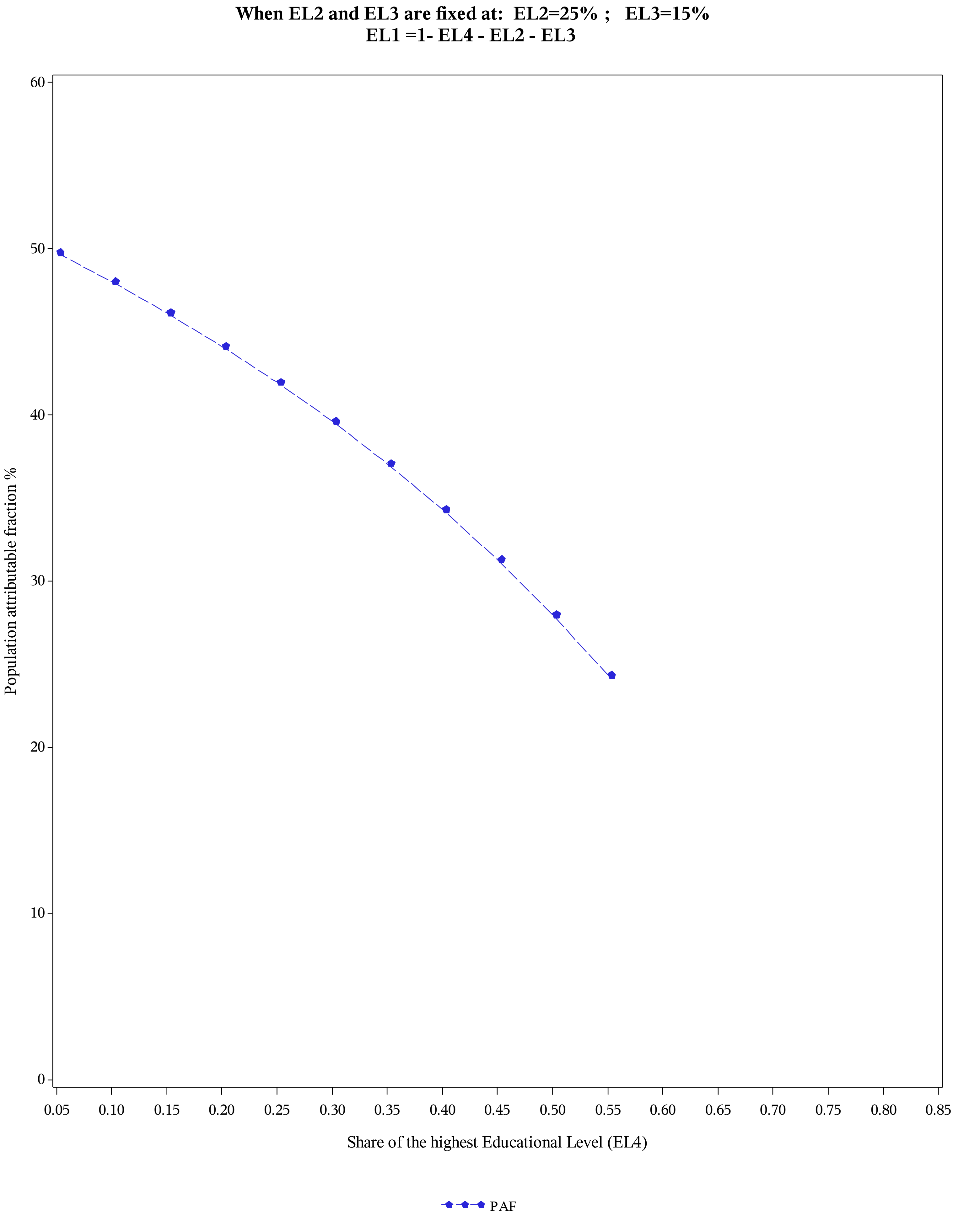

PAF in function of the share of EL4

When EL2 and EL3 are fixed at: EL2=25% ; EL3=20%
EL1 =1- EL4 - EL2 - EL3

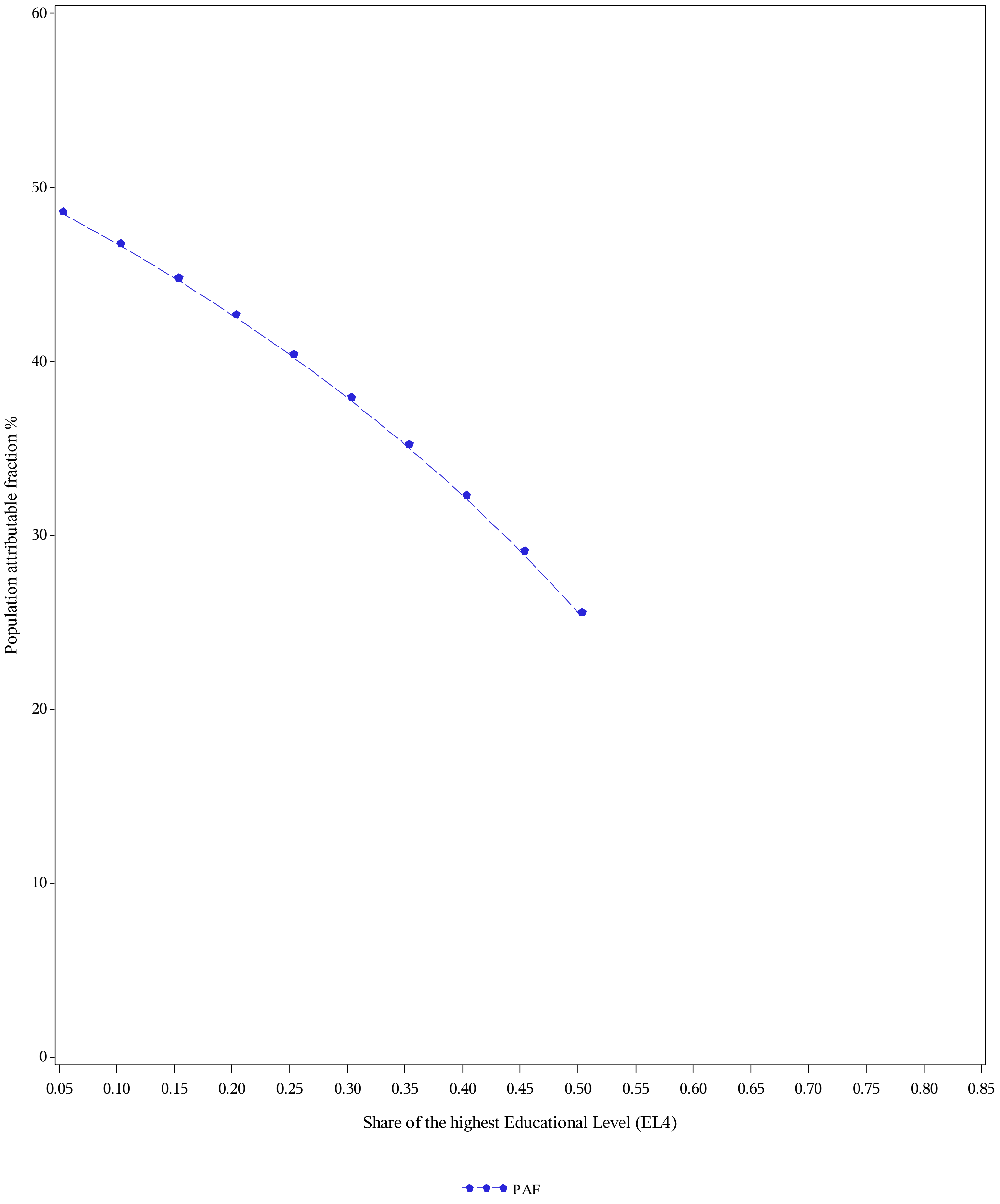

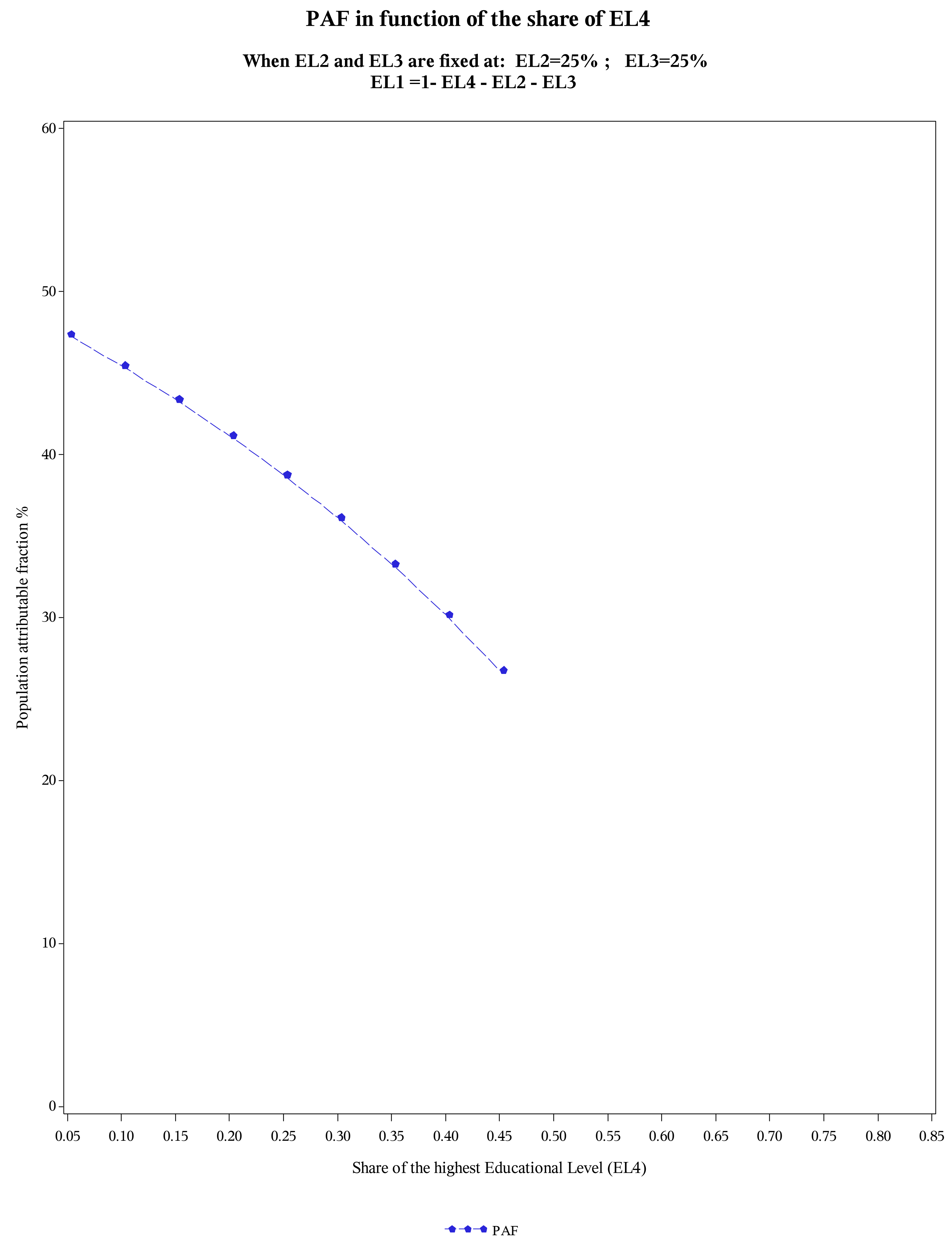
PAF in function of the share of EL4
When EL2 and EL3 are fixed at: EL2=25% ; EL3=25%
EL1 =1- EL4 - EL2 - EL3
Population attributable fraction %
0
10
20
30
40
50
60
0.05
0.10
0.15
0.20
0.25
0.30
0.35
0.40
0.45
0.50
0.55
0.60
0.65
0.70
0.75
0.80
0.85
Share of the highest Educational Level (EL4)
PAF

PAF in function of the share of EL4

When EL2 and EL3 are fixed at: EL2=25% ; EL3=30%
EL1 =1- EL4 - EL2 - EL3

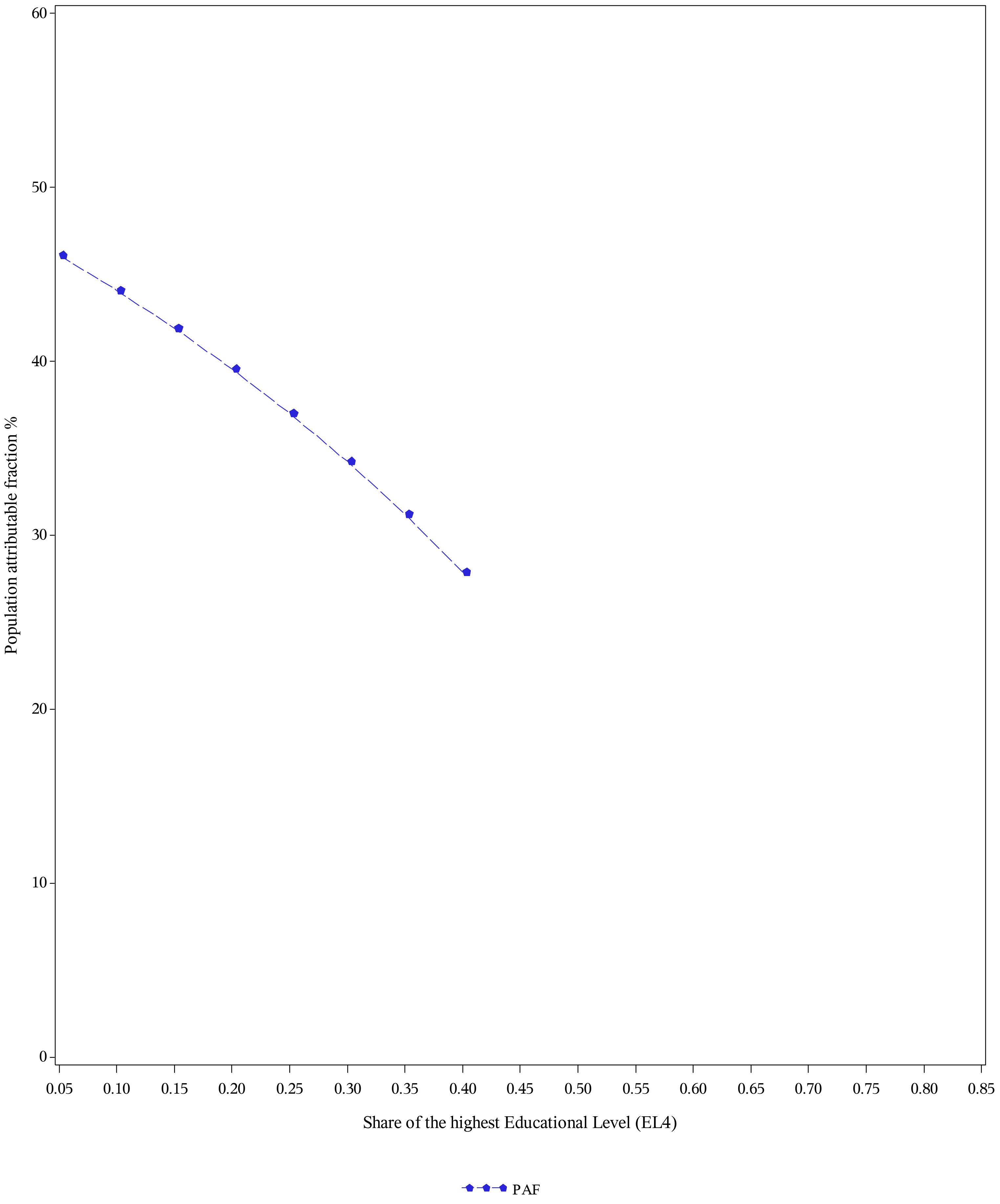

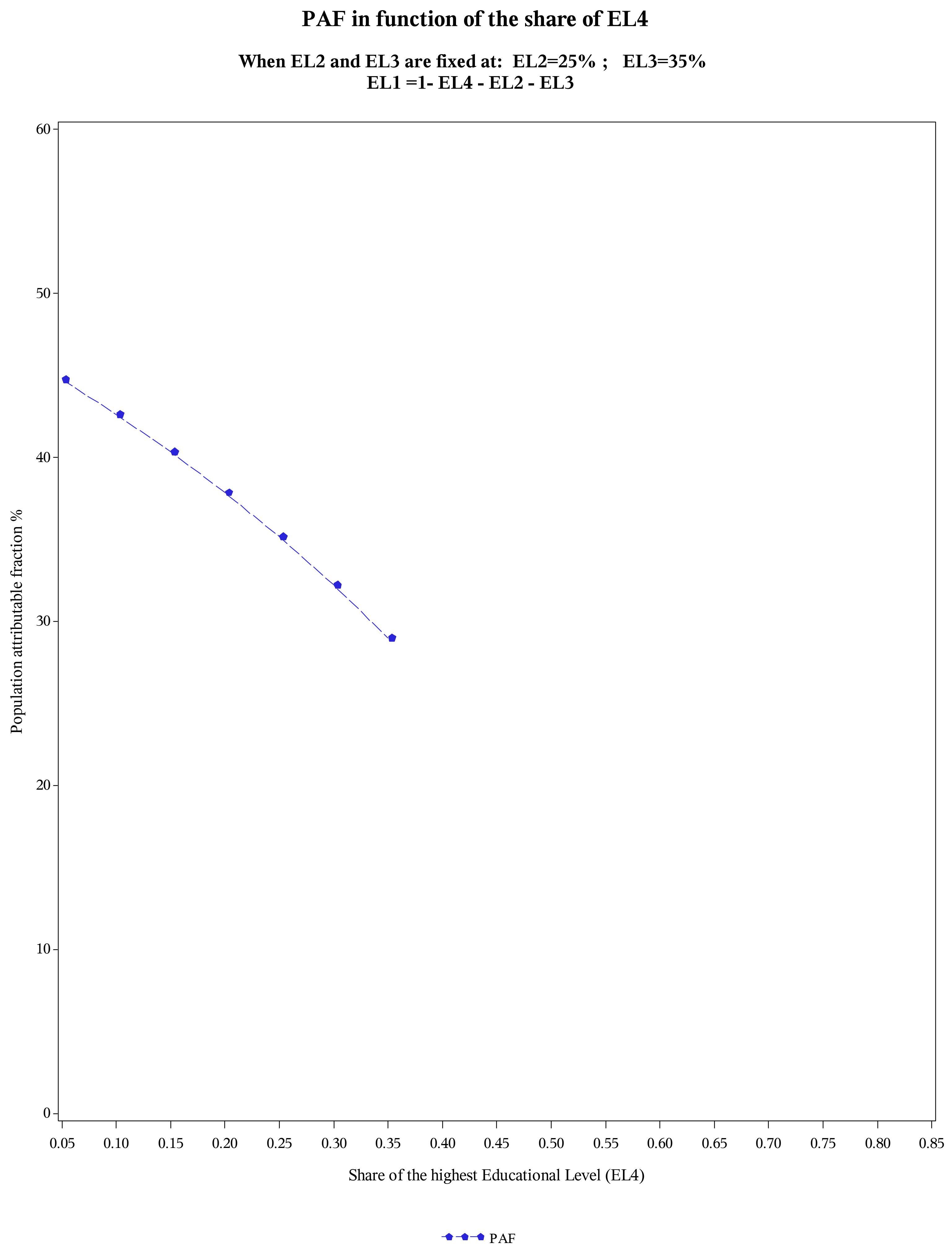

PAF in function of the share of EL4
When EL2 and EL3 are fixed at: EL2=25% ; EL3=35%
EL1 =1- EL4 - EL2 - EL3
Population attributable fraction %
0
10
20
30
40
50
60
0.05
0.10
0.15
0.20
0.25
0.30
0.35
0.40
0.45
0.50
0.55
0.60
0.65
0.70
0.75
0.80
0.85
Share of the highest Educational Level (EL4)
PAF

PAF in function of the share of EL4

When EL2 and EL3 are fixed at: EL2=25% ; EL3=40%
EL1 =1- EL4 - EL2 - EL3

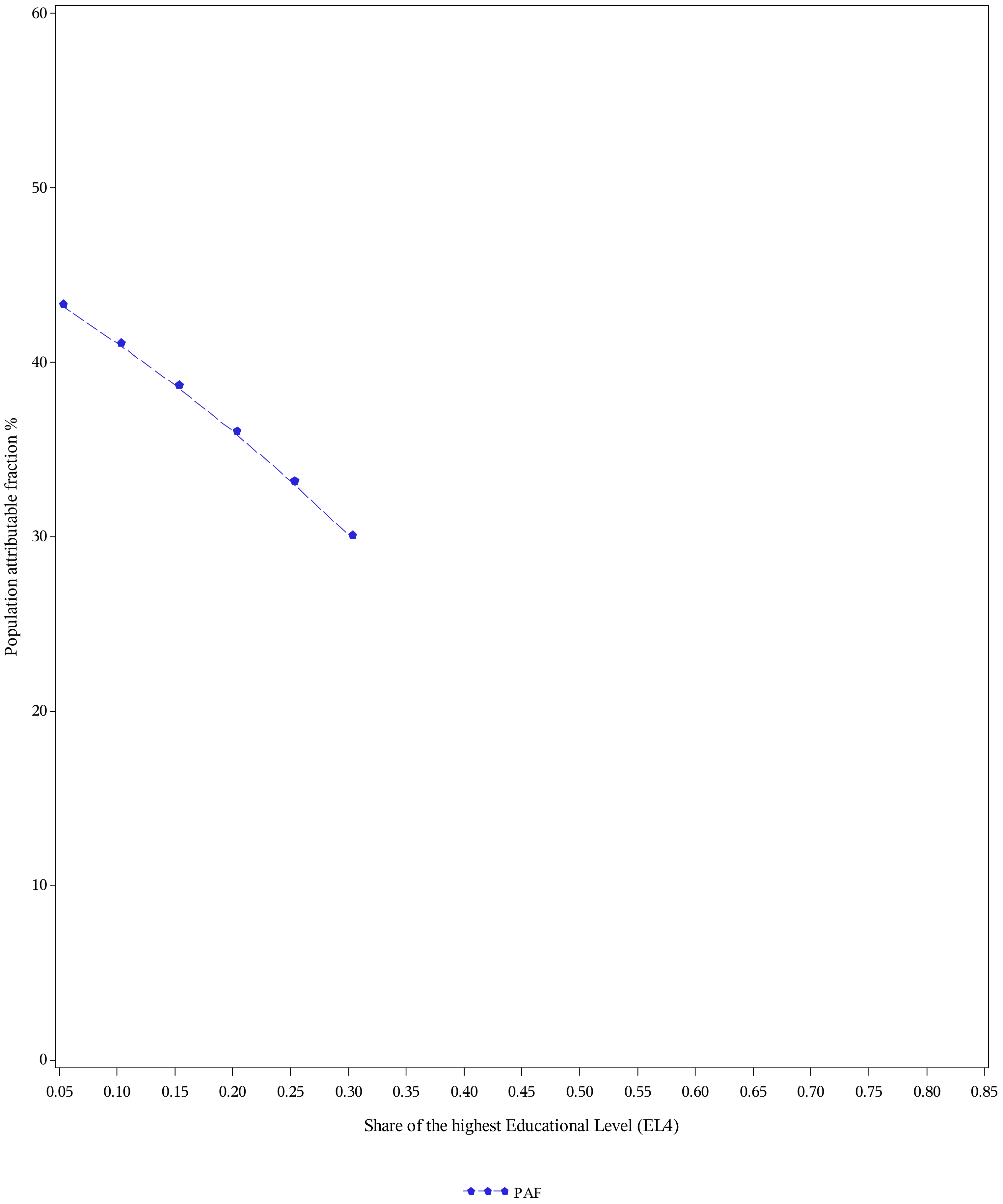

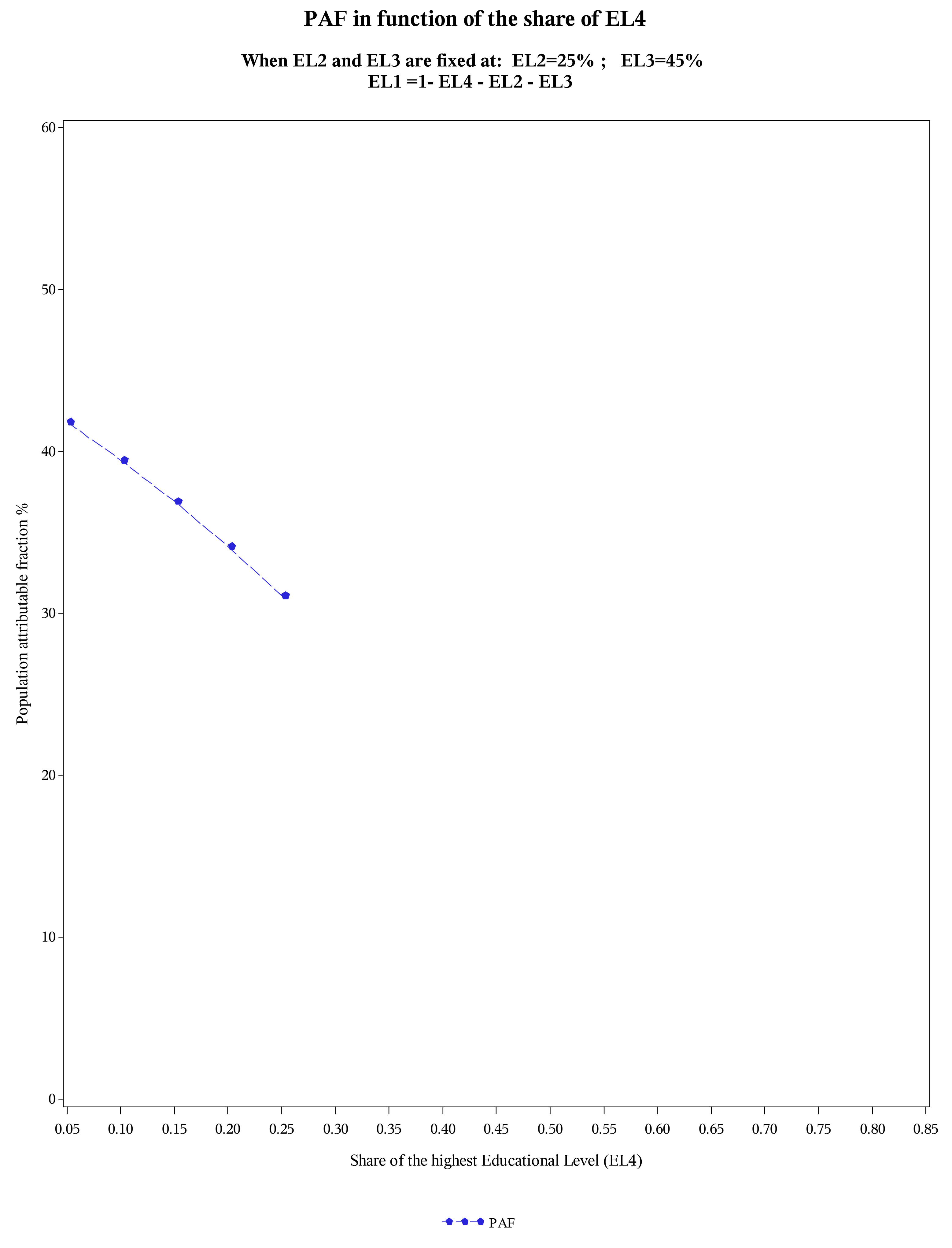
PAF in function of the share of EL4
When EL2 and EL3 are fixed at: EL2=25% ; EL3=45%
EL1 =1- EL4 - EL2 - EL3
Population attributable fraction %
0
10
20
30
40
50
60
0.05
0.10
0.15
0.20
0.25
0.30
0.35
0.40
0.45
0.50
0.55
0.60
0.65
0.70
0.75
0.80
0.85
Share of the highest Educational Level (EL4)
PAF

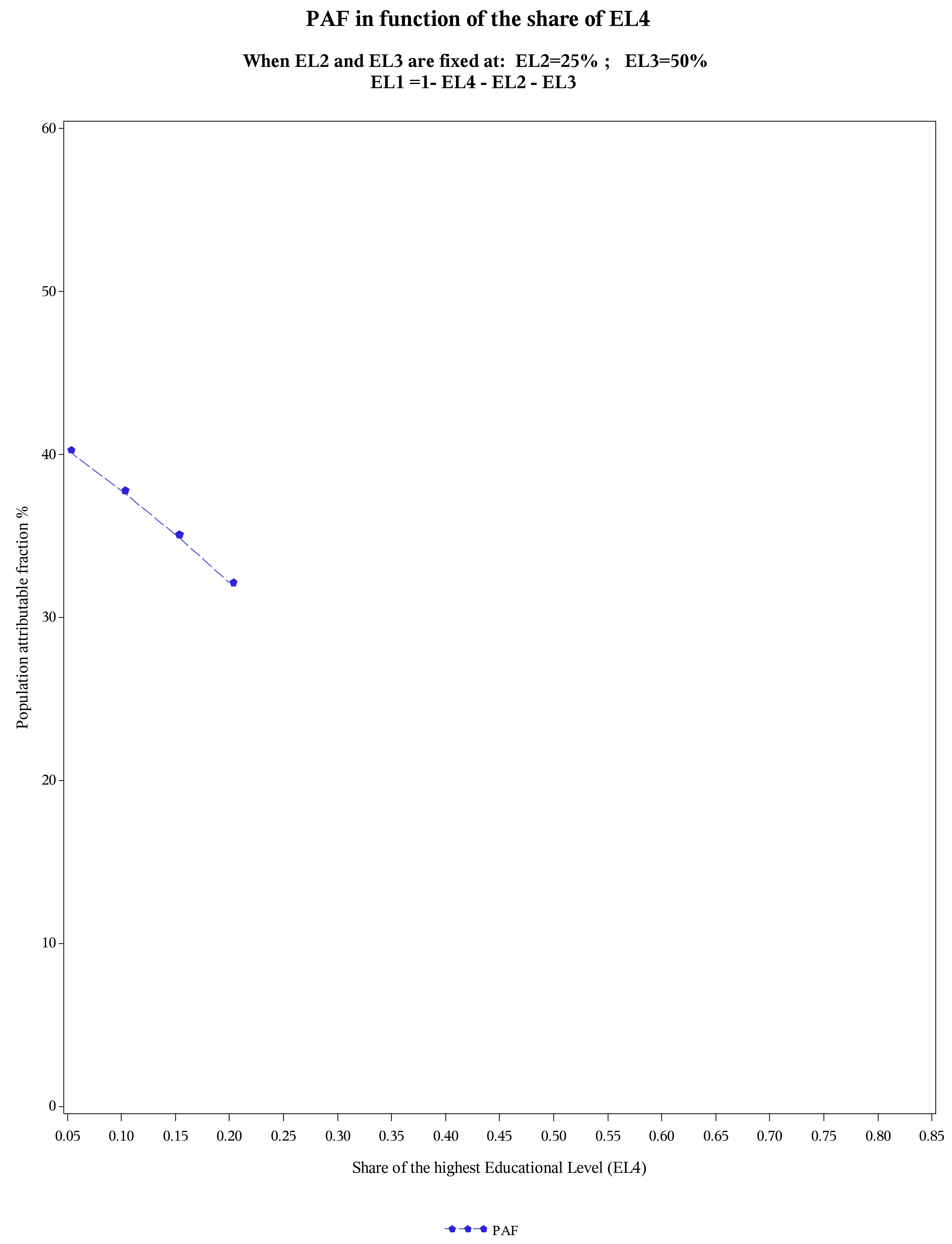
PAF in function of the share of EL4
When EL2 and EL3 are fixed at: EL2=25% ; EL3=50%
EL1 =1- EL4 - EL2 - EL3
Population attributable fraction %
0
10
20
30
40
50
60
0.05
0.10
0.15
0.20
0.25
0.30
0.35
0.40
0.45
0.50
0.55
0.60
0.65
0.70
0.75
0.80
0.85
Share of the highest Educational Level (EL4)
PAF

PAF in function of the share of EL4

When EL2 and EL3 are fixed at: EL2=25% ; EL3=55%
EL1 =1- EL4 - EL2 - EL3

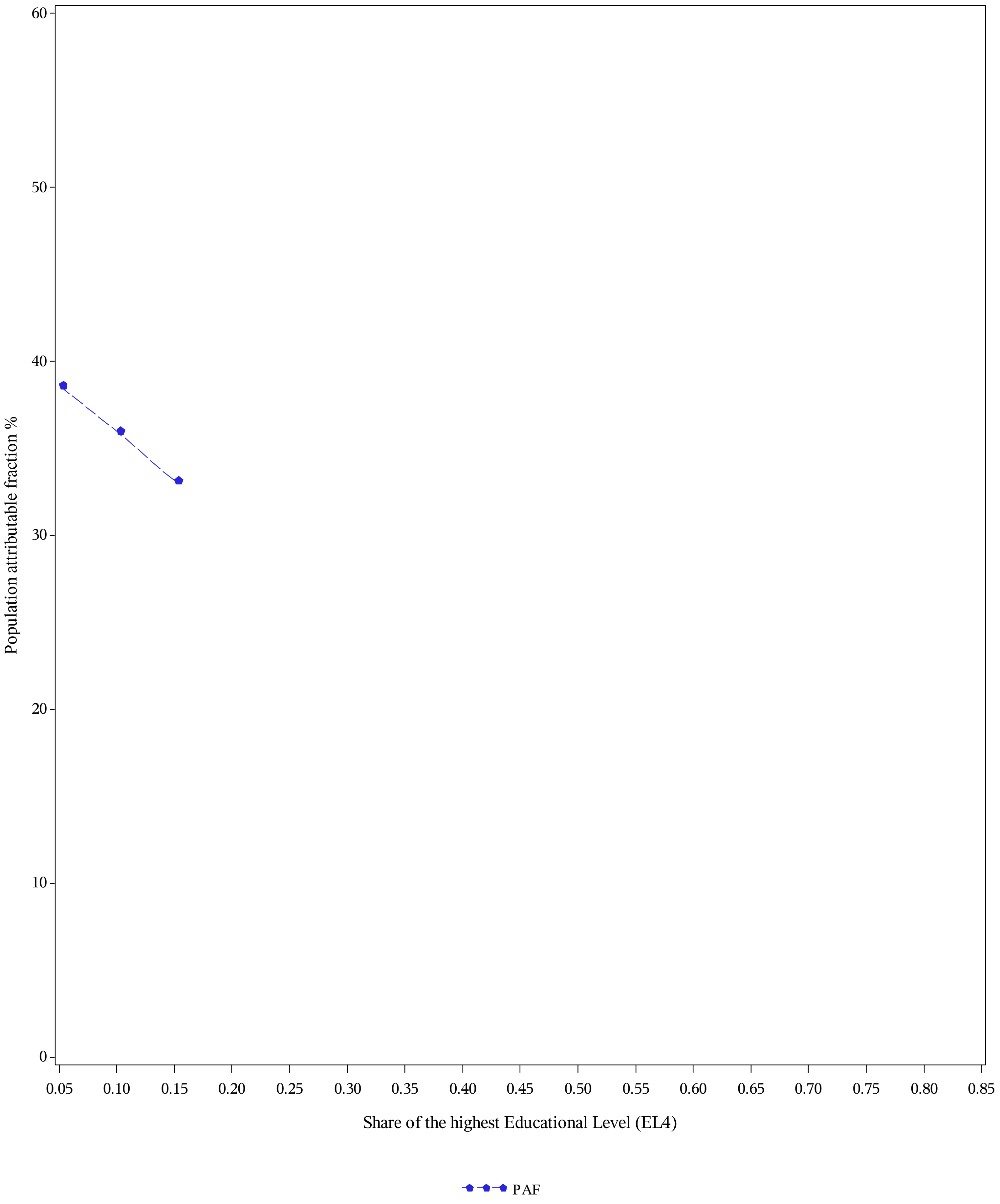

PAF in function of the share of EL4

When EL2 and EL3 are fixed at: EL2=25% ; EL3=60%
EL1 =1- EL4 - EL2 - EL3

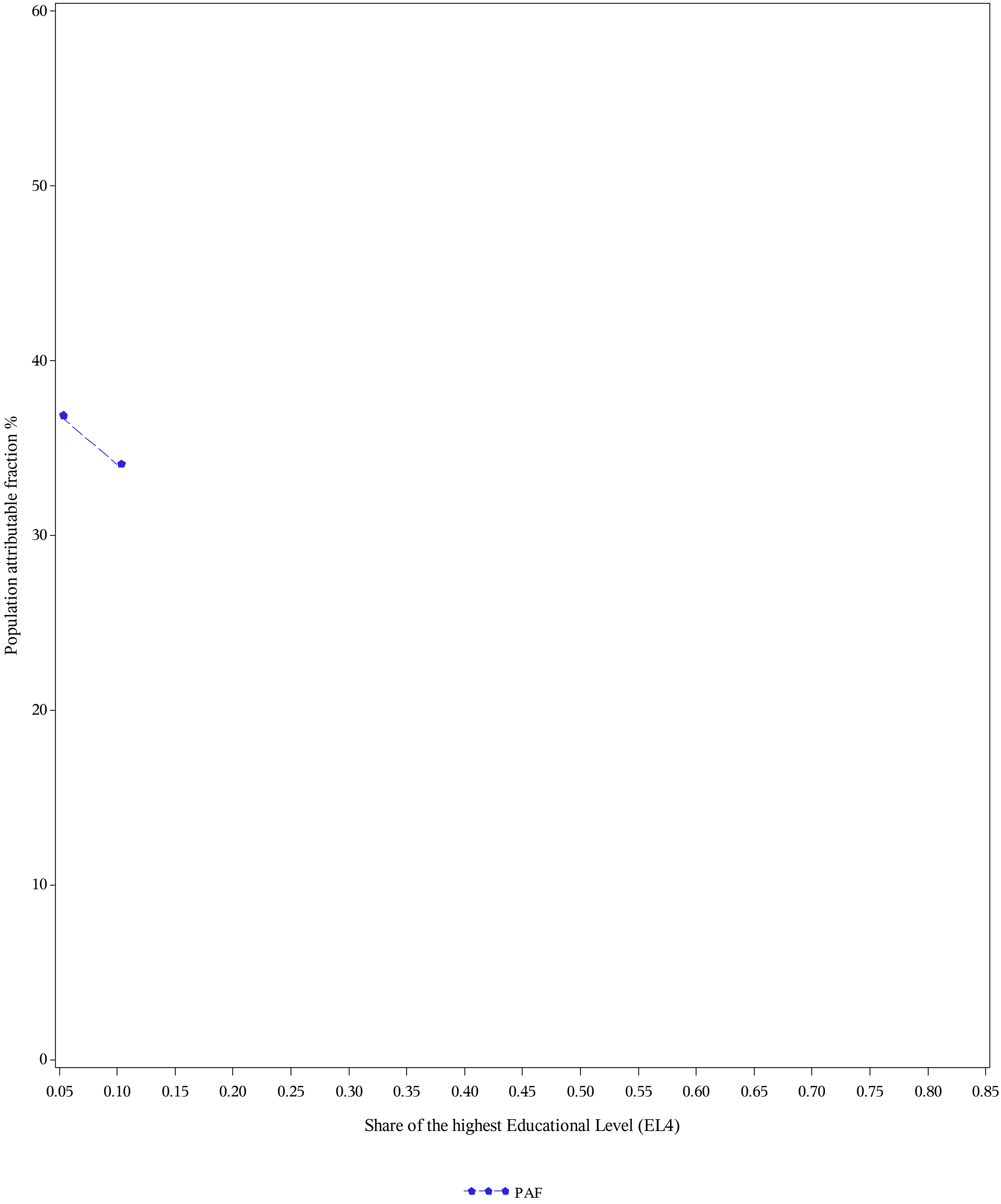

PAF in function of the share of EL4

When EL2 and EL3 are fixed at: EL2=30% ; EL3=5%
EL1 =1- EL4 - EL2 - EL3

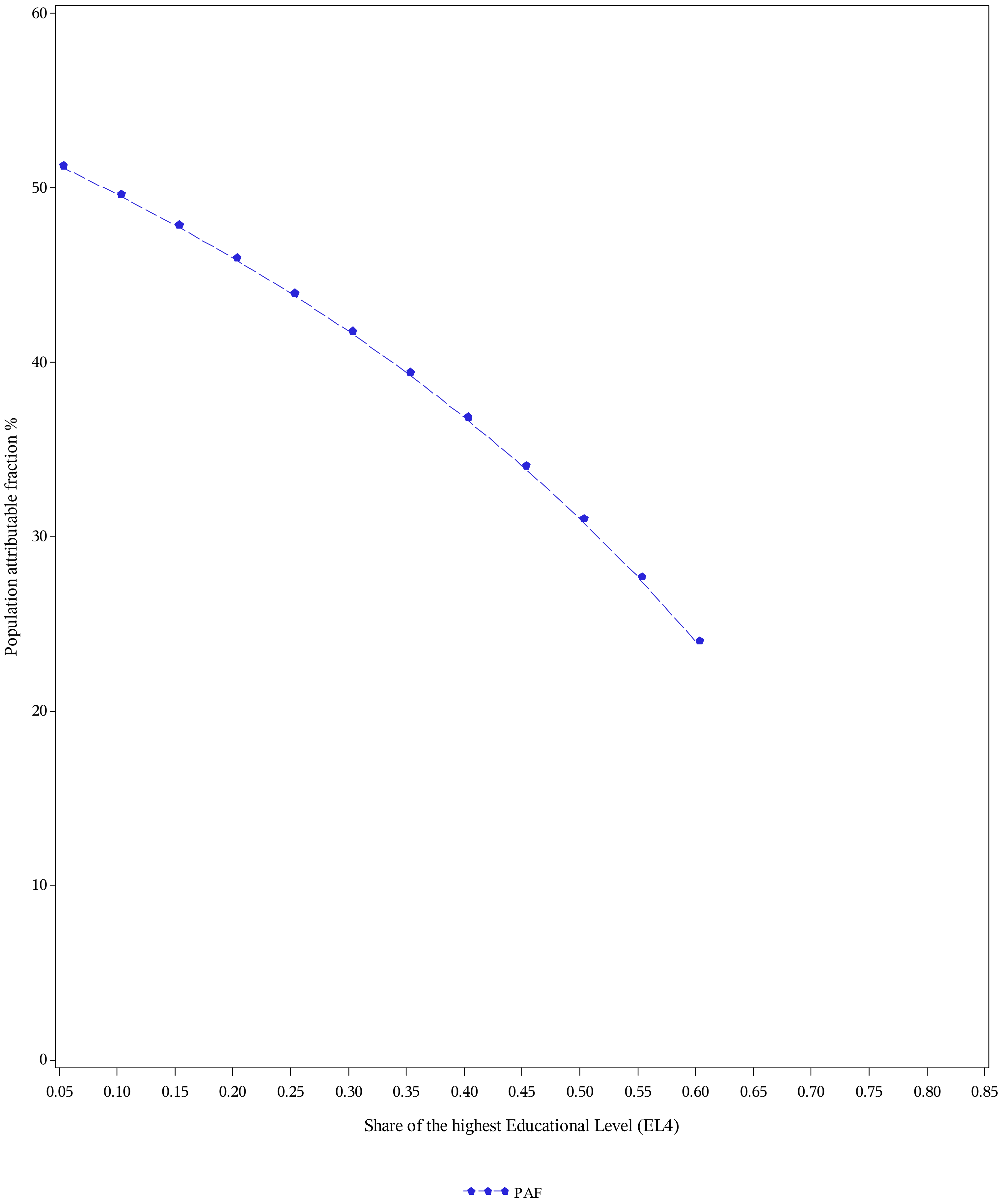

PAF in function of the share of EL4

When EL2 and EL3 are fixed at: EL2=30% ; EL3=10%
EL1 =1- EL4 - EL2 - EL3

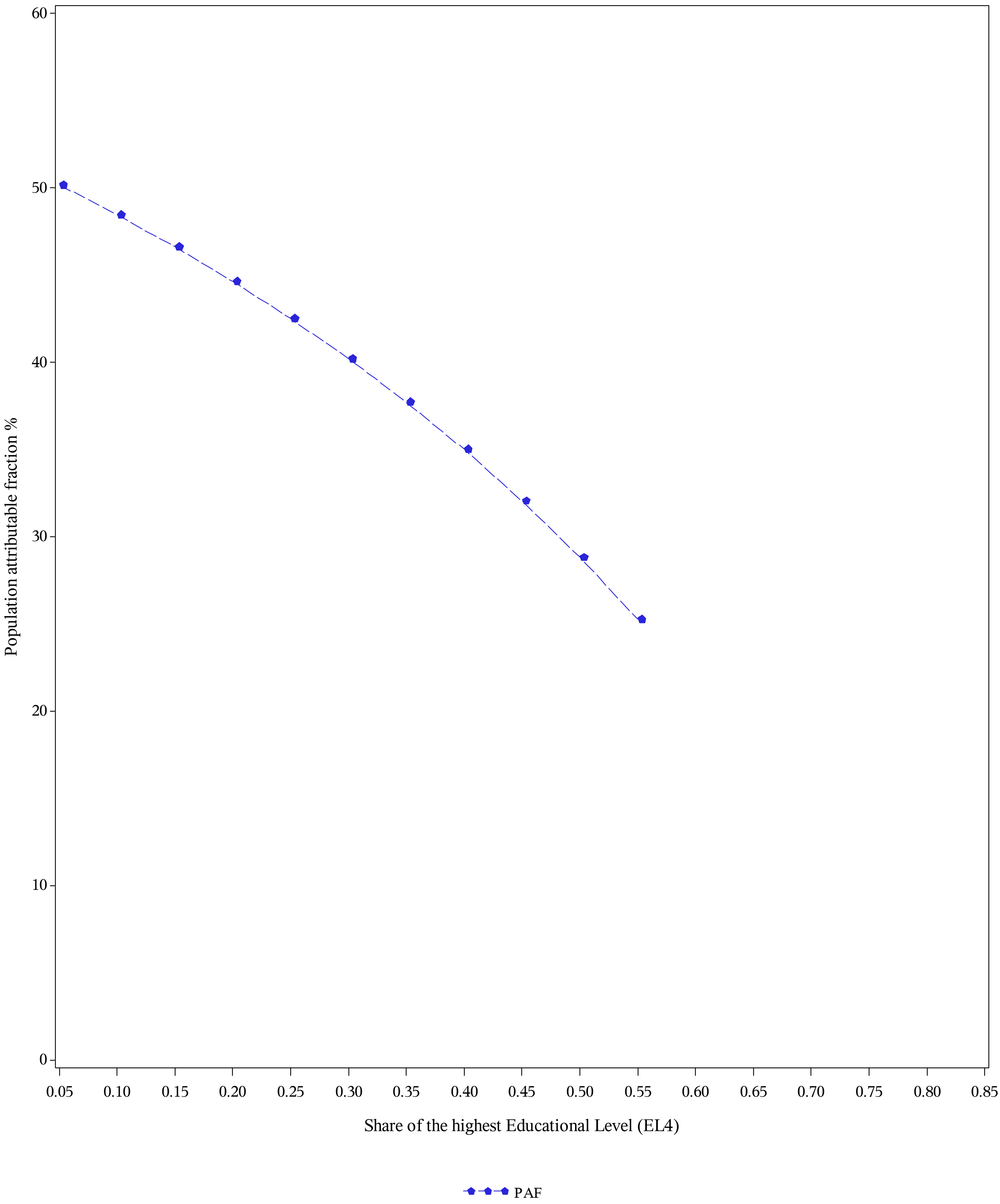

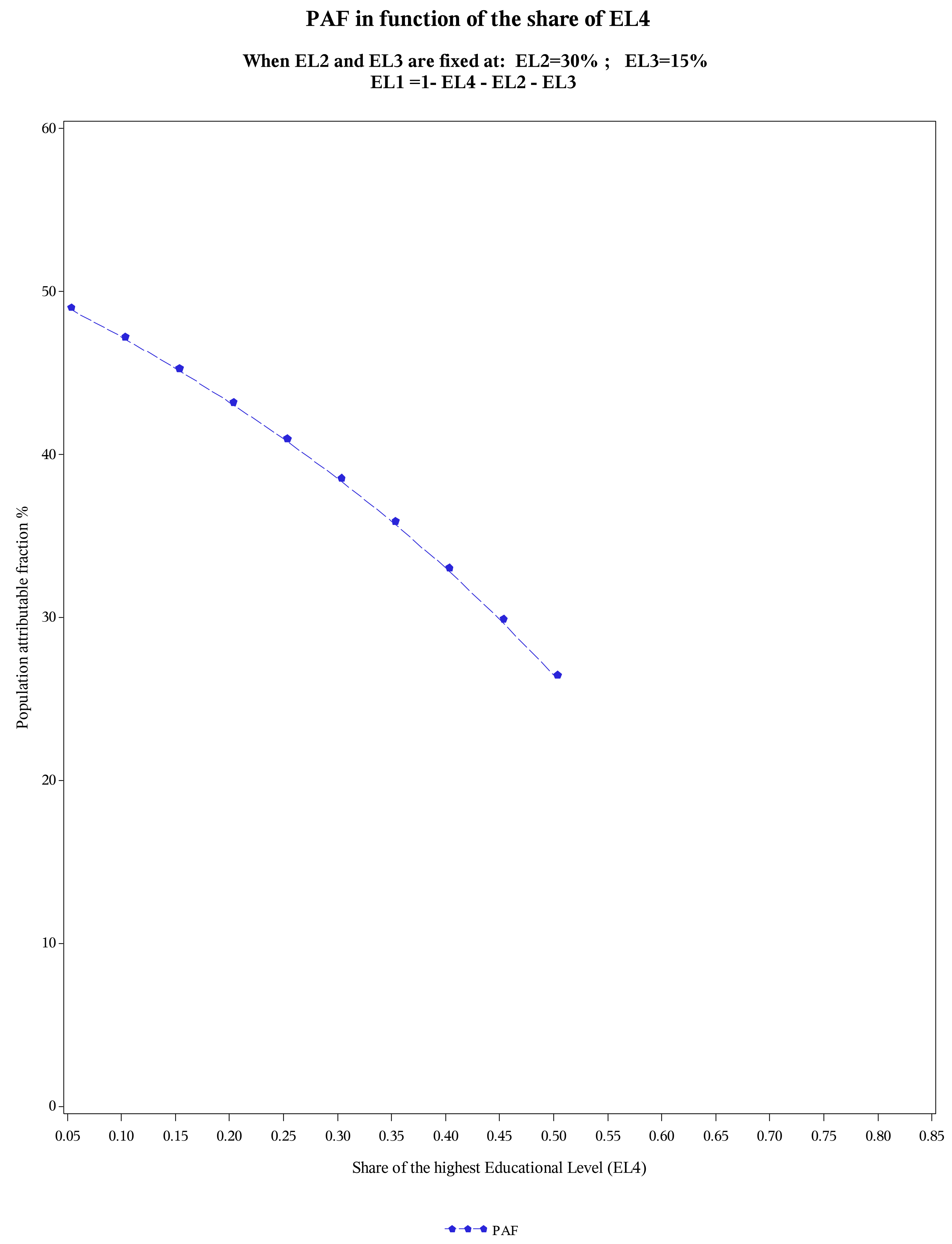
PAF in function of the share of EL4
When EL2 and EL3 are fixed at: EL2=30% ; EL3=15%
EL1 =1- EL4 - EL2 - EL3
Population attributable fraction %
0
10
20
30
40
50
60
0.05
0.10
0.15
0.20
0.25
0.30
0.35
0.40
0.45
0.50
0.55
0.60
0.65
0.70
0.75
0.80
0.85
Share of the highest Educational Level (EL4)
PAF

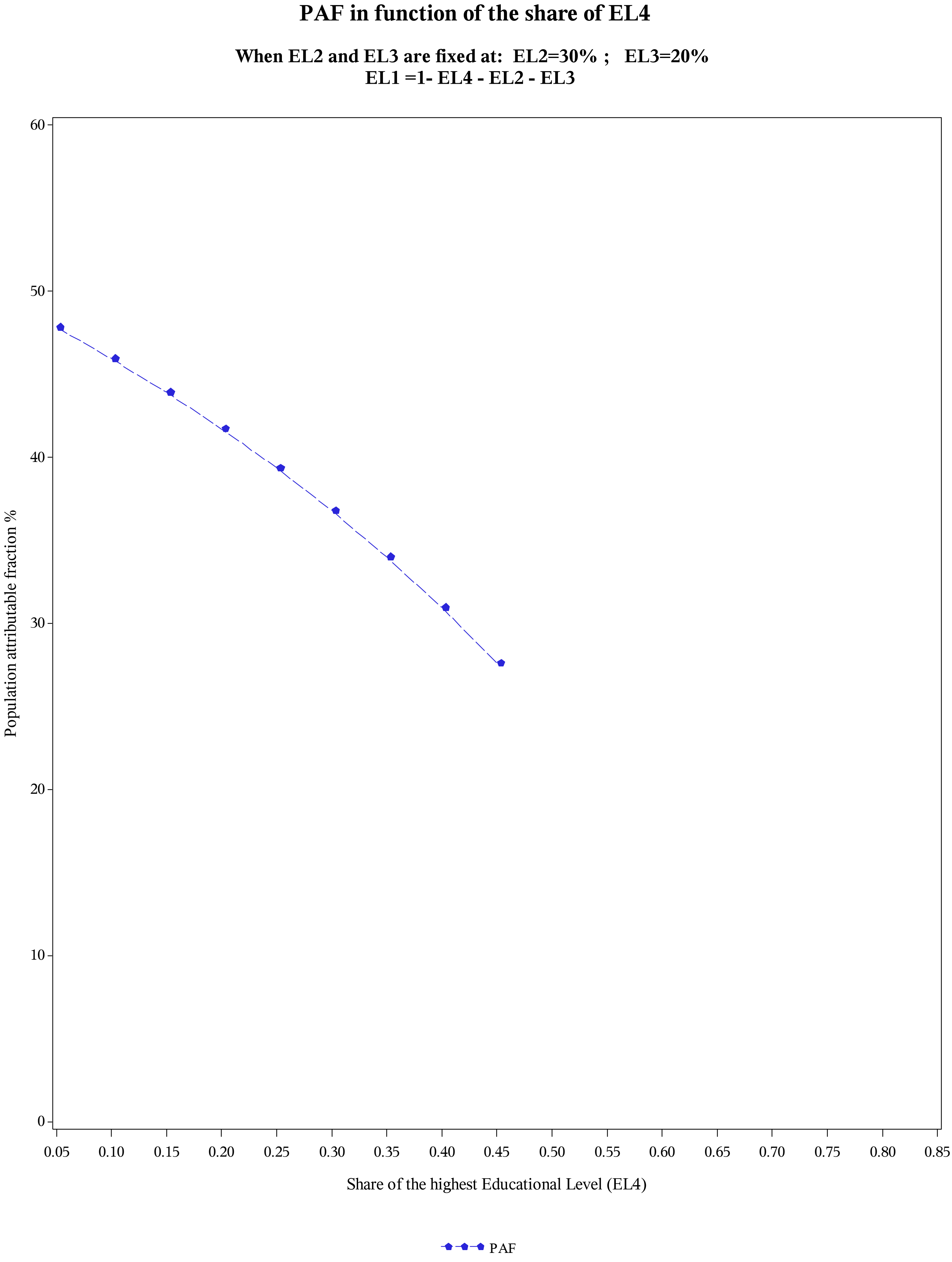

PAF in function of the share of EL4
When EL2 and EL3 are fixed at: EL2=30% ; EL3=20%
EL1 =1- EL4 - EL2 - EL3
Population attributable fraction %
0
10
20
30
40
50
60
0.05
0.10
0.15
0.20
0.25
0.30
0.35
0.40
0.45
0.50
0.55
0.60
0.65
0.70
0.75
0.80
0.85
Share of the highest Educational Level (EL4)
PAF

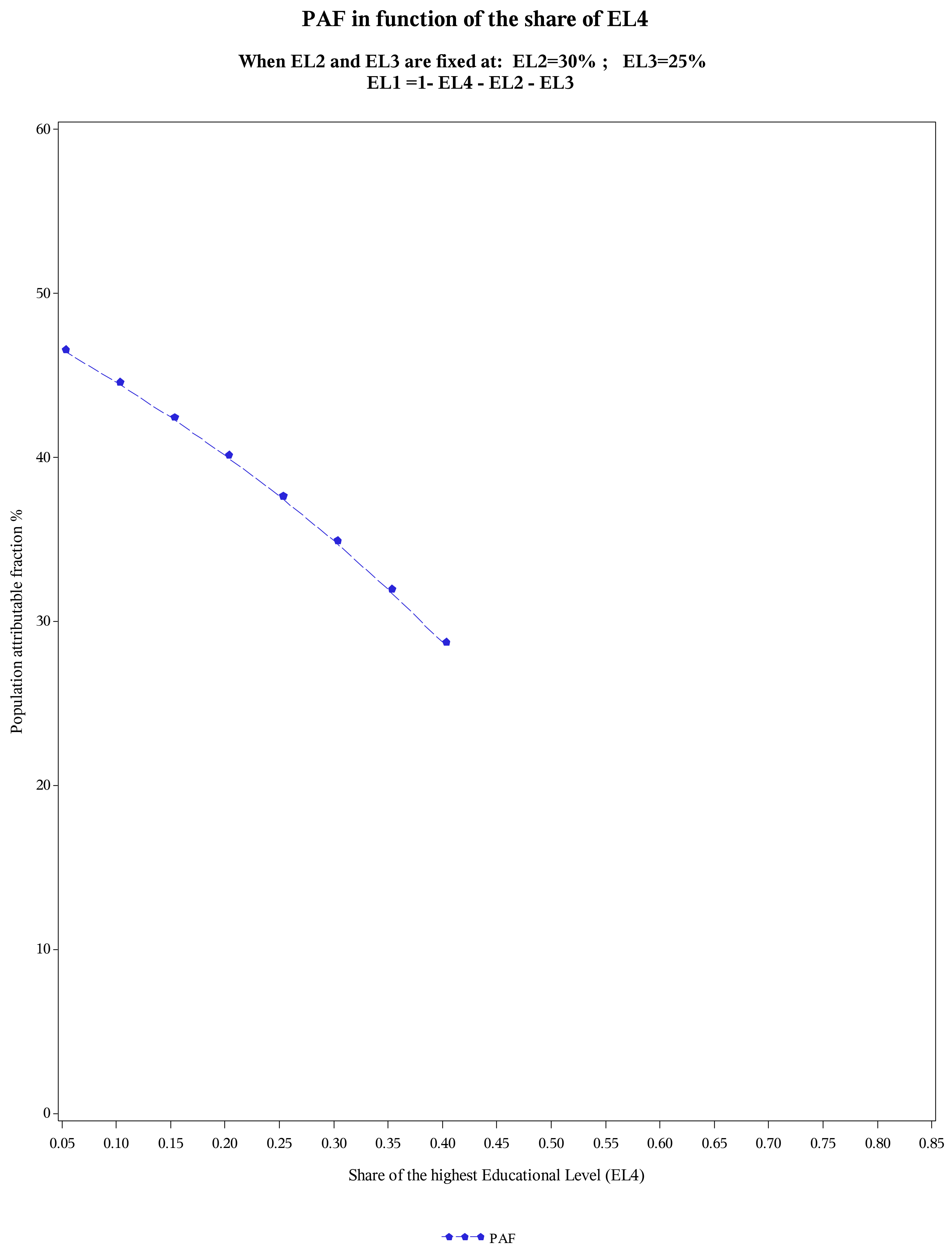
PAF in function of the share of EL4
When EL2 and EL3 are fixed at: EL2=30% ; EL3=25%
EL1 =1- EL4 - EL2 - EL3
Population attributable fraction %
0
10
20
30
40
50
60
0.05
0.10
0.15
0.20
0.25
0.30
0.35
0.40
0.45
0.50
0.55
0.60
0.65
0.70
0.75
0.80
0.85
Share of the highest Educational Level (EL4)
PAF

PAF in function of the share of EL4

When EL2 and EL3 are fixed at: EL2=30% ; EL3=30%
EL1 =1- EL4 - EL2 - EL3

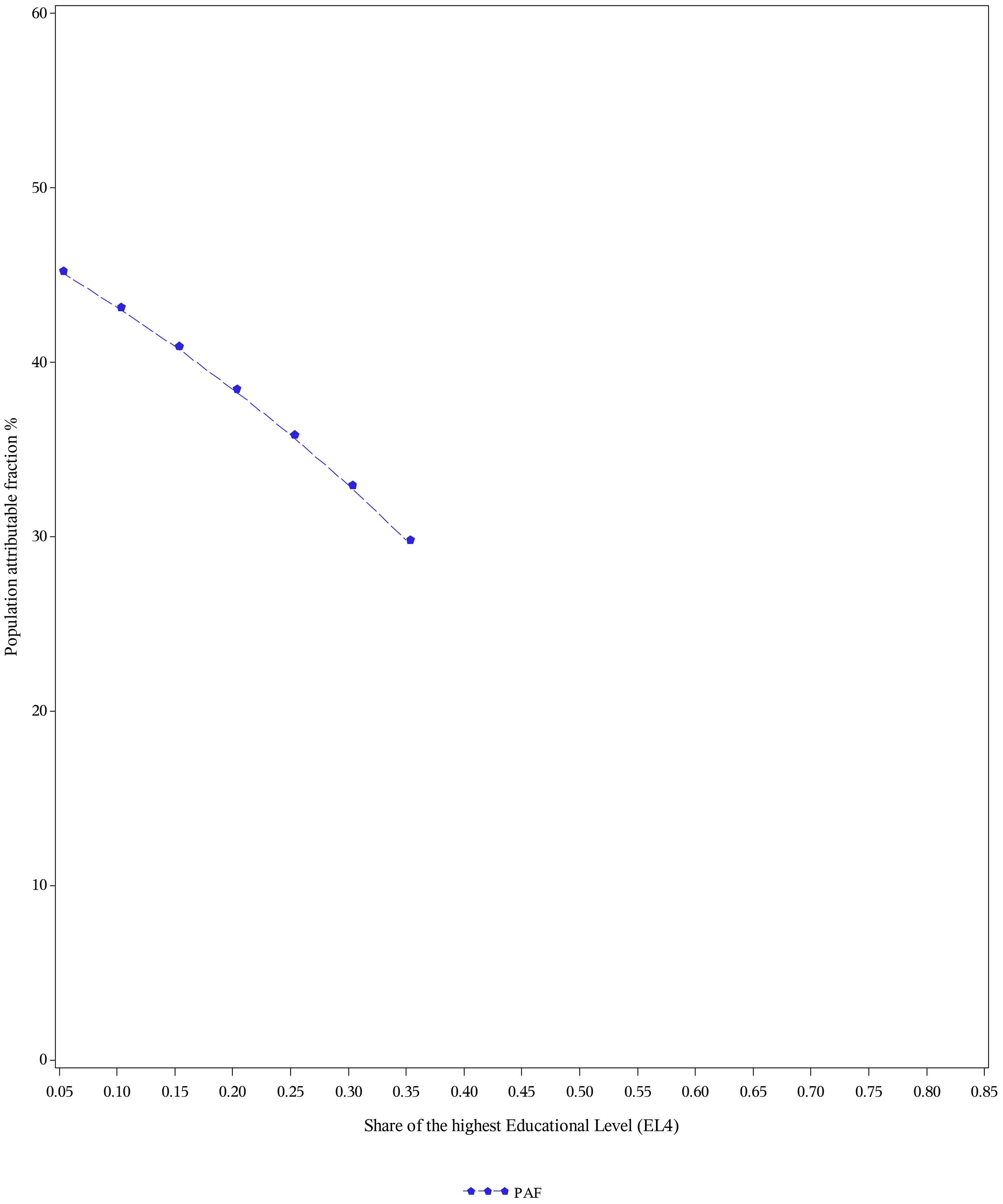

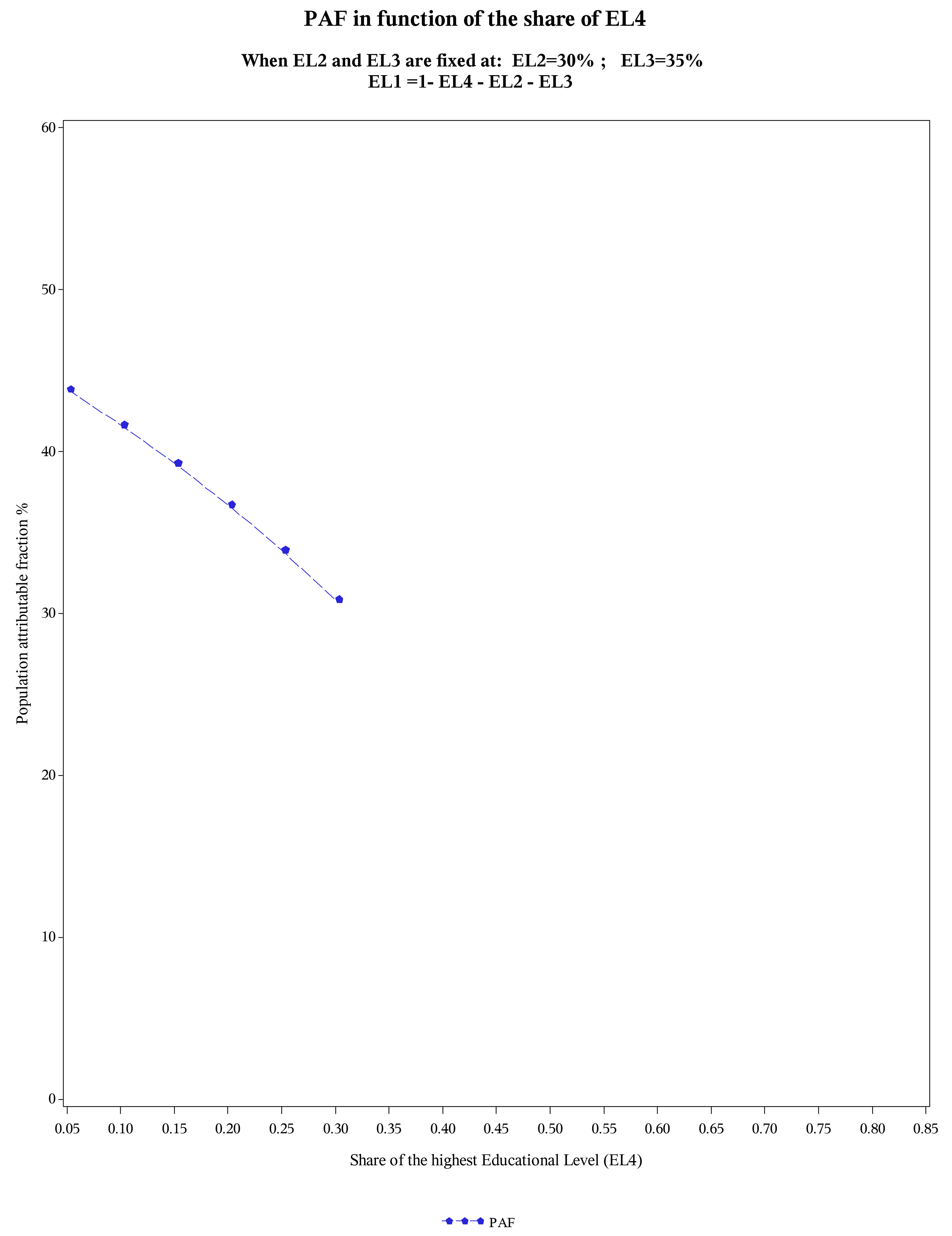

PAF in function of the share of EL4
When EL2 and EL3 are fixed at: EL2=30% ; EL3=35%
EL1 =1- EL4 - EL2 - EL3
Population attributable fraction %
0
10
20
30
40
50
60
0.05
0.10
0.15
0.20
0.25
0.30
0.35
0.40
0.45
0.50
0.55
0.60
0.65
0.70
0.75
0.80
0.85
Share of the highest Educational Level (EL4)
PAF

PAF in function of the share of EL4

When EL2 and EL3 are fixed at: EL2=30% ;   EL3=40%
EL1 =1- EL4 - EL2 - EL3

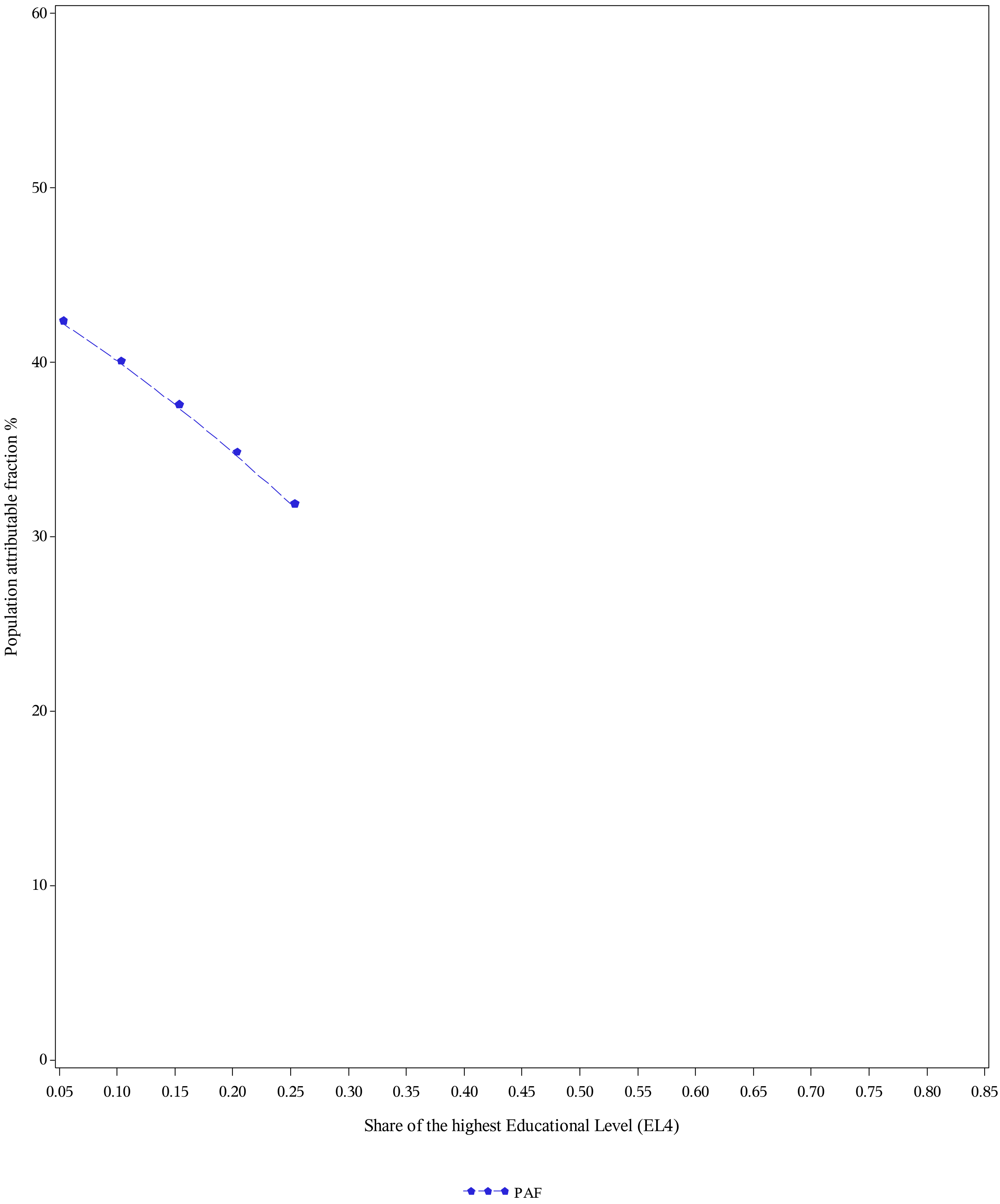

PAF in function of the share of EL4

When EL2 and EL3 are fixed at: EL2=30% ; EL3=45%
EL1 =1- EL4 - EL2 - EL3

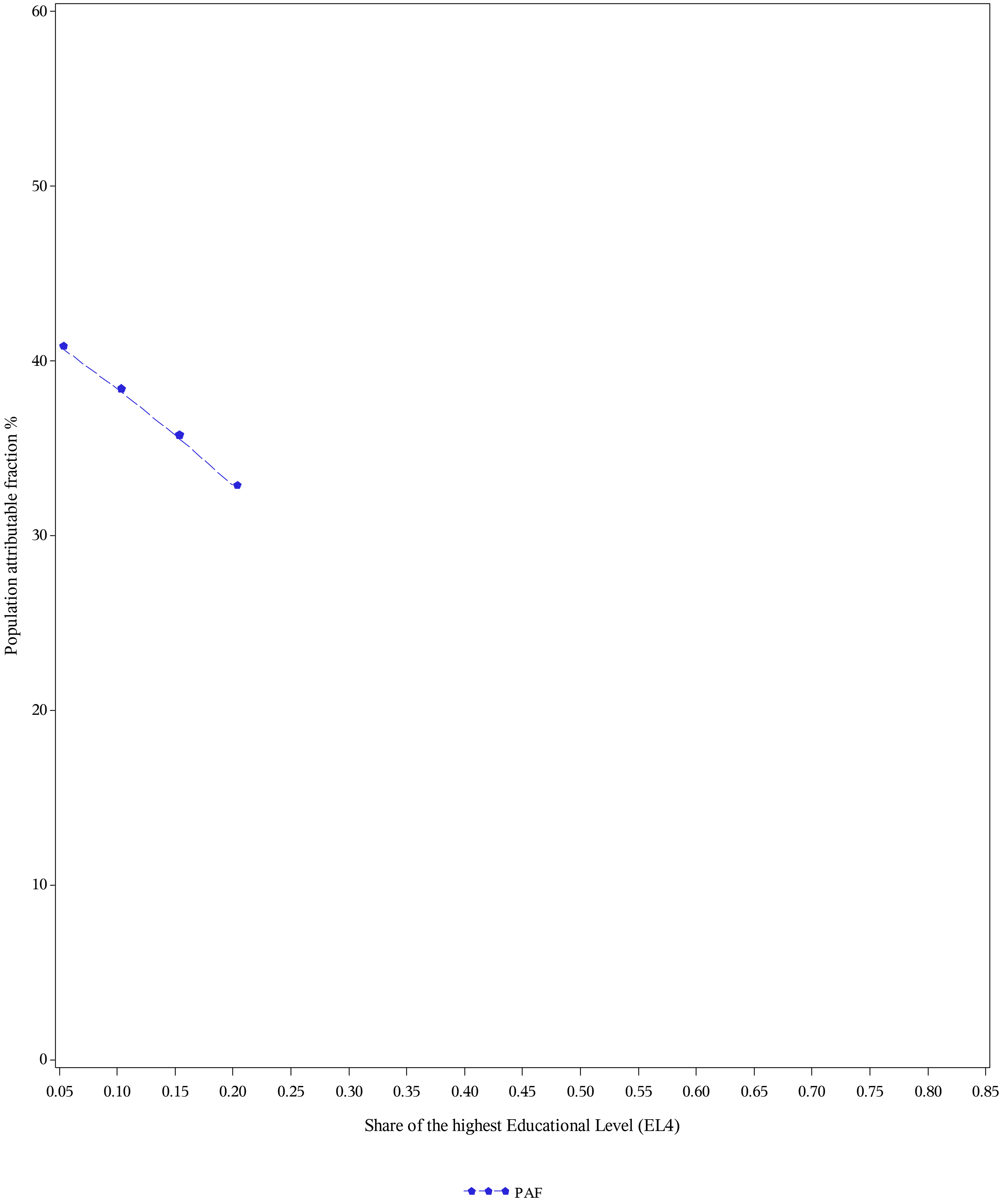

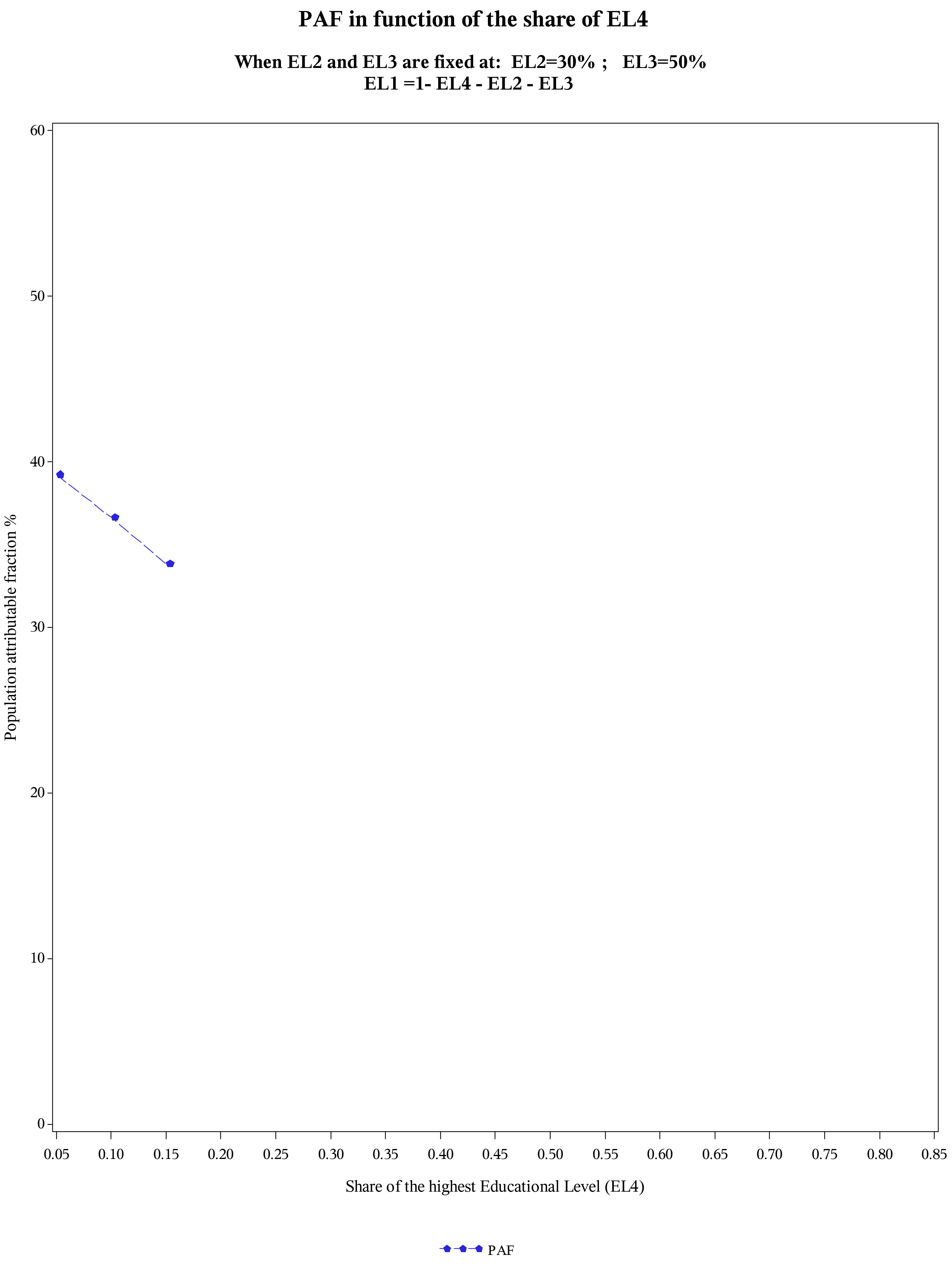
PAF in function of the share of EL4
When EL2 and EL3 are fixed at: EL2=30% ; EL3=50%
EL1 =1- EL4 - EL2 - EL3
Population attributable fraction %
0
10
20
30
40
50
60
0.05
0.10
0.15
0.20
0.25
0.30
0.35
0.40
0.45
0.50
0.55
0.60
0.65
0.70
0.75
0.80
0.85
Share of the highest Educational Level (EL4)
PAF

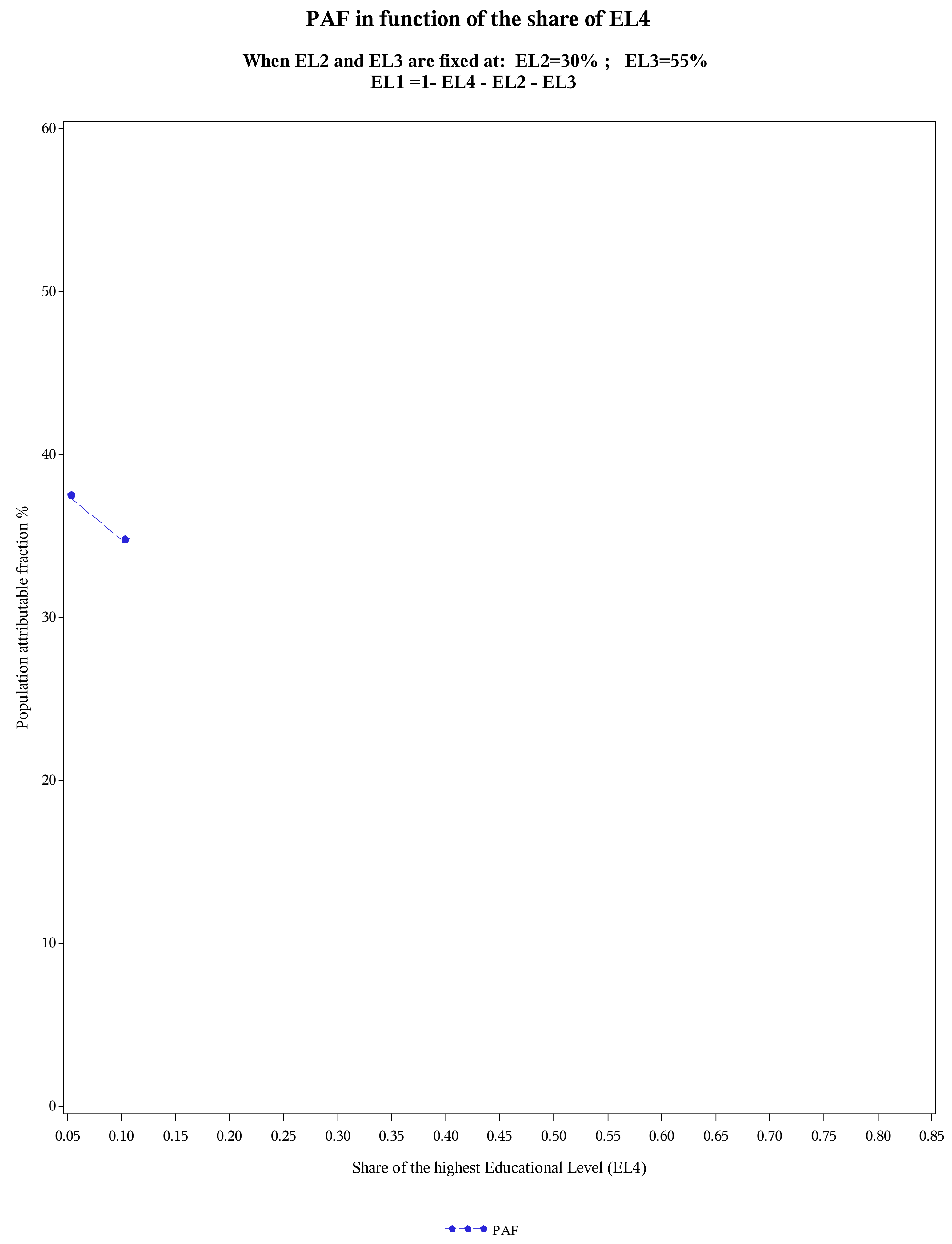

PAF in function of the share of EL4
When EL2 and EL3 are fixed at: EL2=30% ; EL3=55%
EL1 =1- EL4 - EL2 - EL3
Population attributable fraction %
0
10
20
30
40
50
60
0.05
0.10
0.15
0.20
0.25
0.30
0.35
0.40
0.45
0.50
0.55
0.60
0.65
0.70
0.75
0.80
0.85
Share of the highest Educational Level (EL4)
PAF

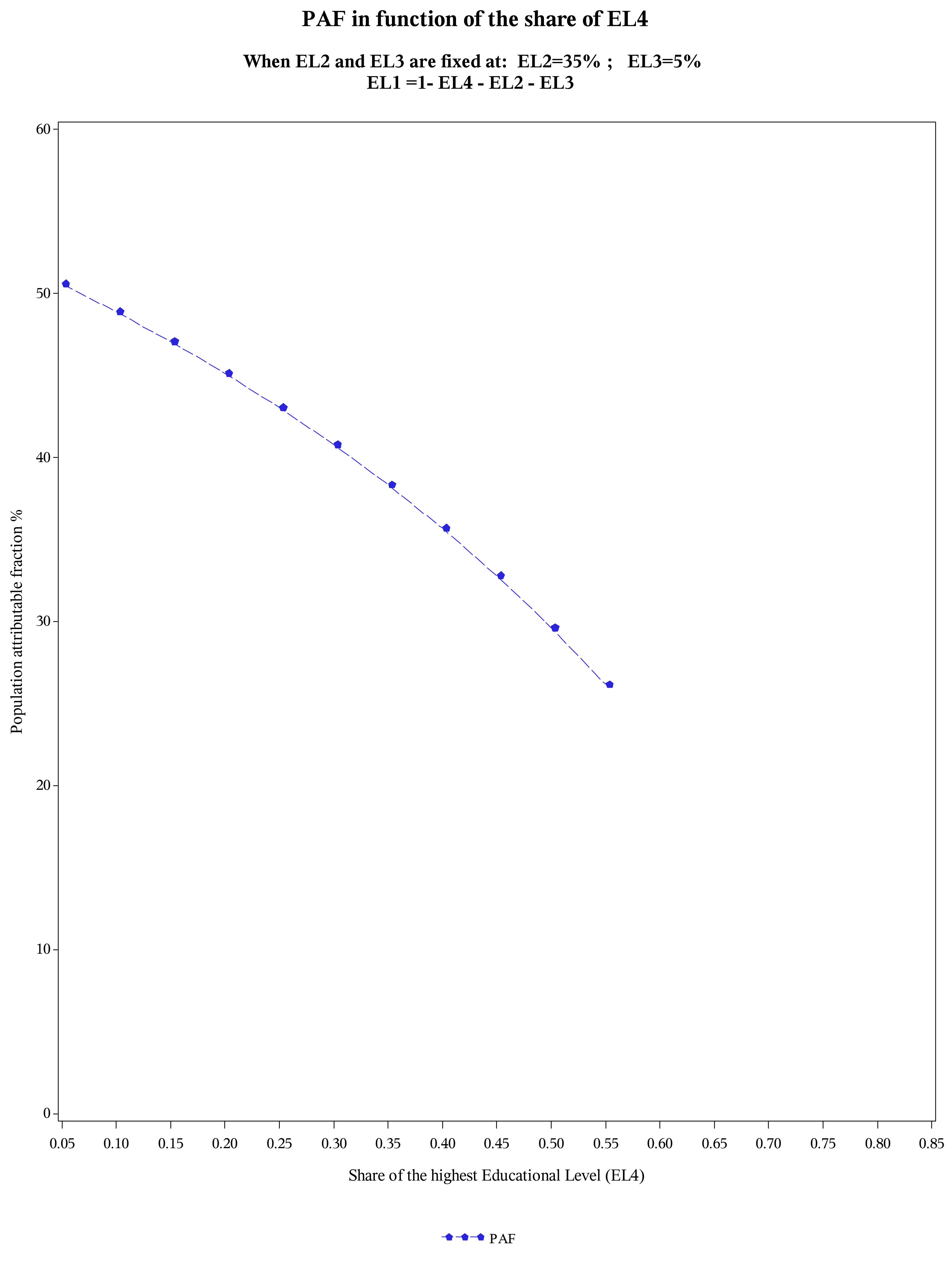
PAF in function of the share of EL4
When EL2 and EL3 are fixed at: EL2=35% ; EL3=5%
EL1 =1- EL4 - EL2 - EL3
Population attributable fraction %
0
10
20
30
40
50
60
0.05
0.10
0.15
0.20
0.25
0.30
0.35
0.40
0.45
0.50
0.55
0.60
0.65
0.70
0.75
0.80
0.85
Share of the highest Educational Level (EL4)
PAF

PAF in function of the share of EL4

When EL2 and EL3 are fixed at: EL2=35% ; EL3=10%
EL1 =1- EL4 - EL2 - EL3

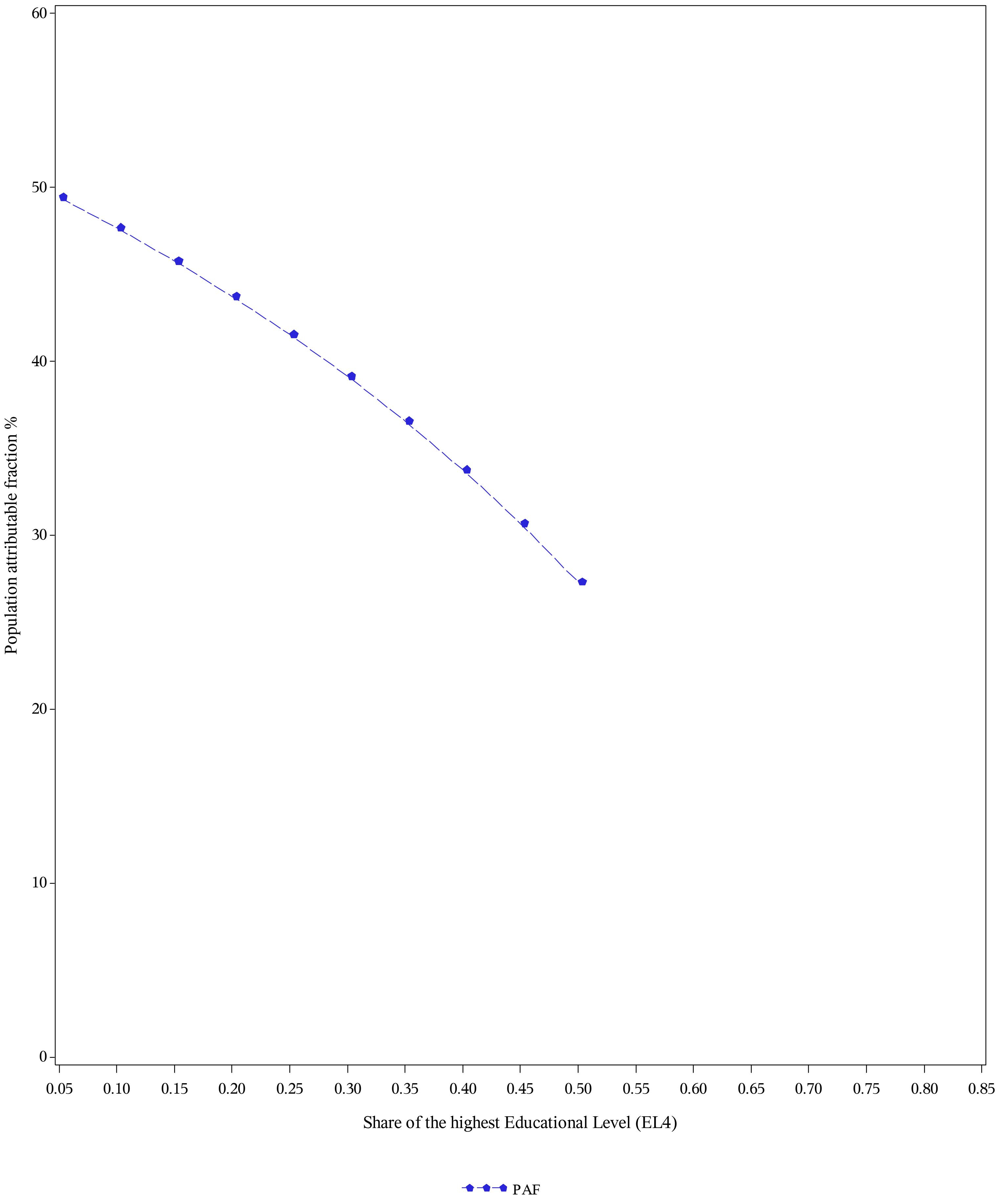

PAF in function of the share of EL4

When EL2 and EL3 are fixed at: EL2=35% ; EL3=15%
EL1 =1- EL4 - EL2 - EL3

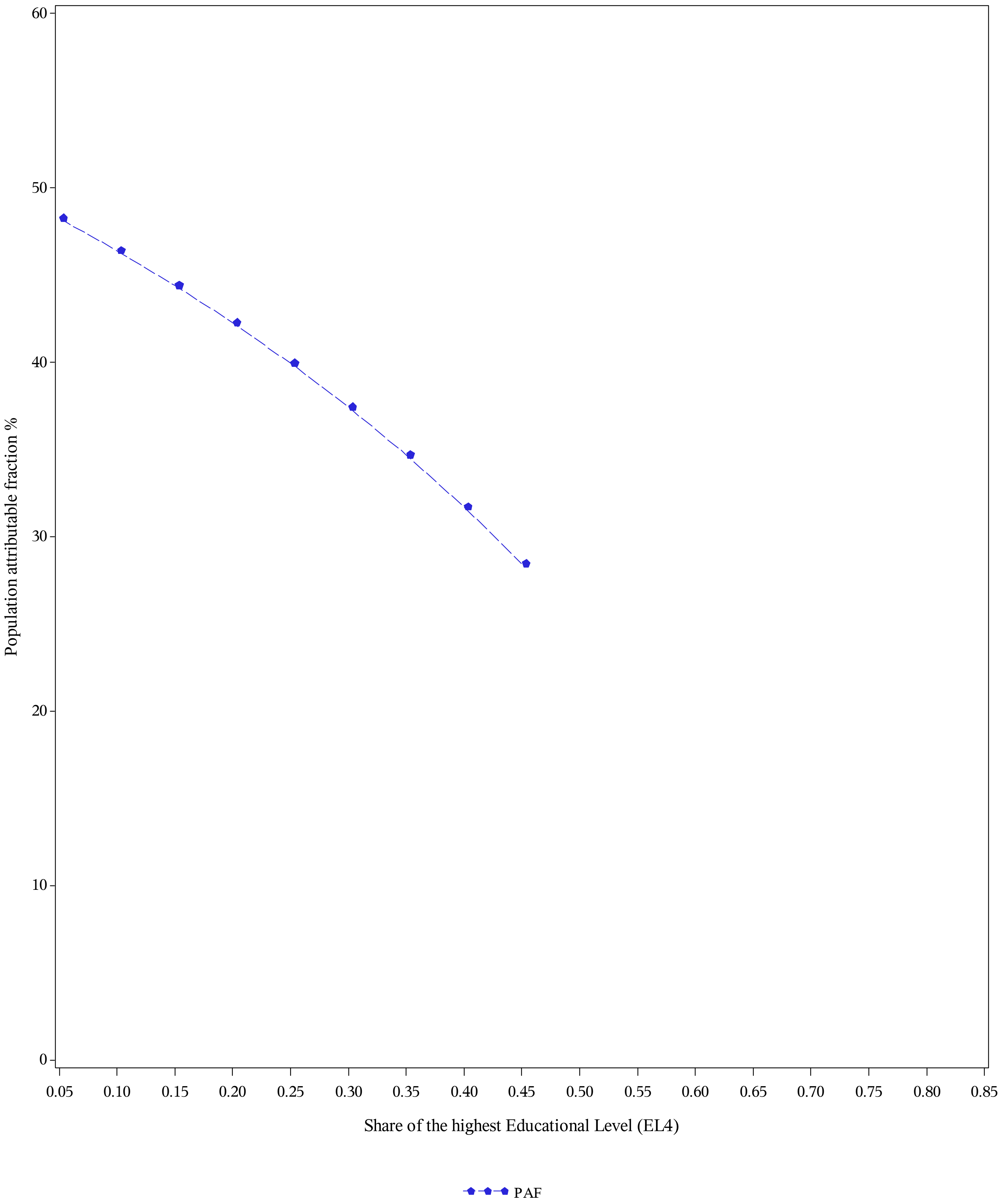

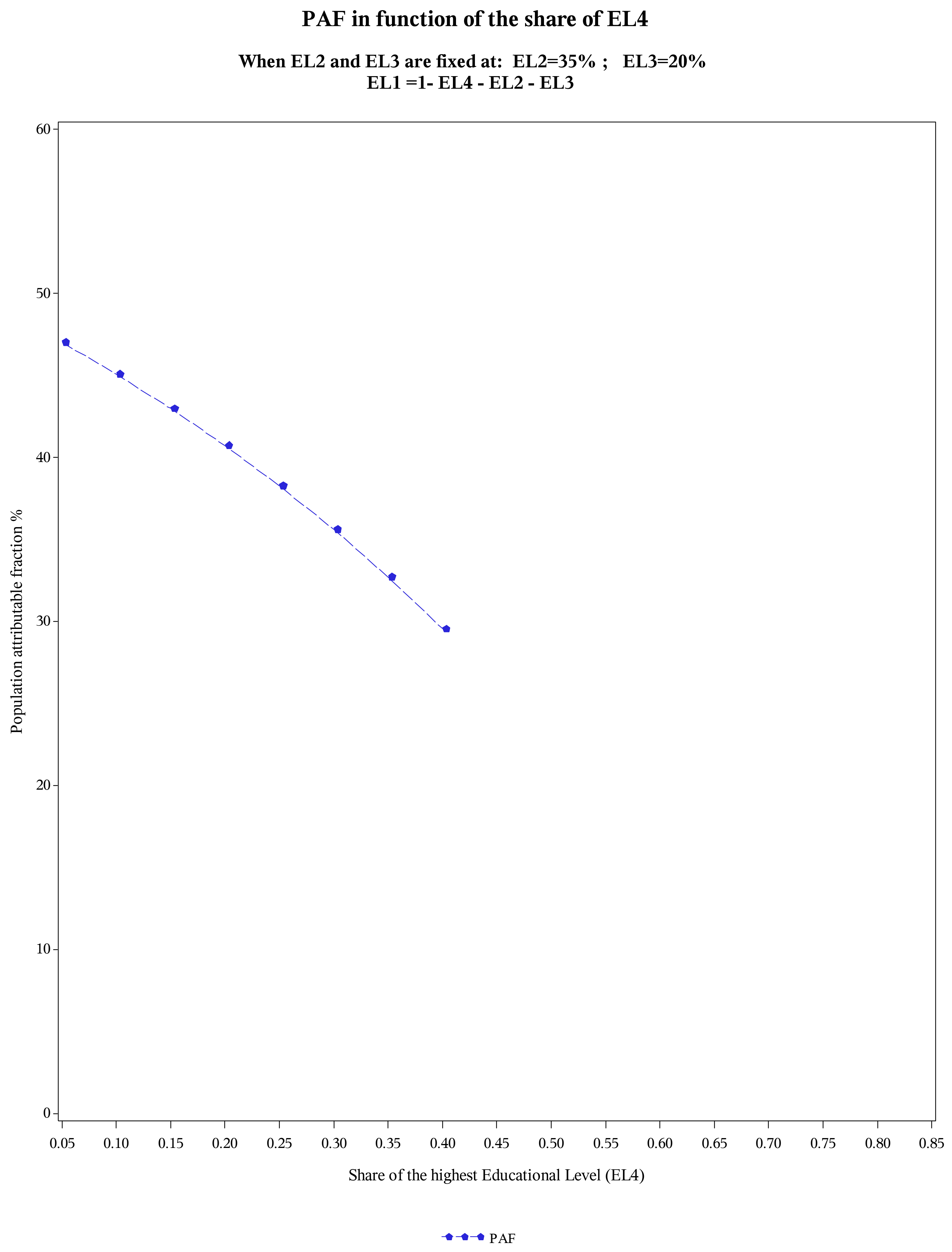
PAF in function of the share of EL4
When EL2 and EL3 are fixed at: EL2=35% ; EL3=20%
EL1 =1- EL4 - EL2 - EL3
Population attributable fraction %
0
10
20
30
40
50
60
0.05
0.10
0.15
0.20
0.25
0.30
0.35
0.40
0.45
0.50
0.55
0.60
0.65
0.70
0.75
0.80
0.85
Share of the highest Educational Level (EL4)
PAF

PAF in function of the share of EL4

When EL2 and EL3 are fixed at: EL2=35% ; EL3=25%
EL1 =1- EL4 - EL2 - EL3

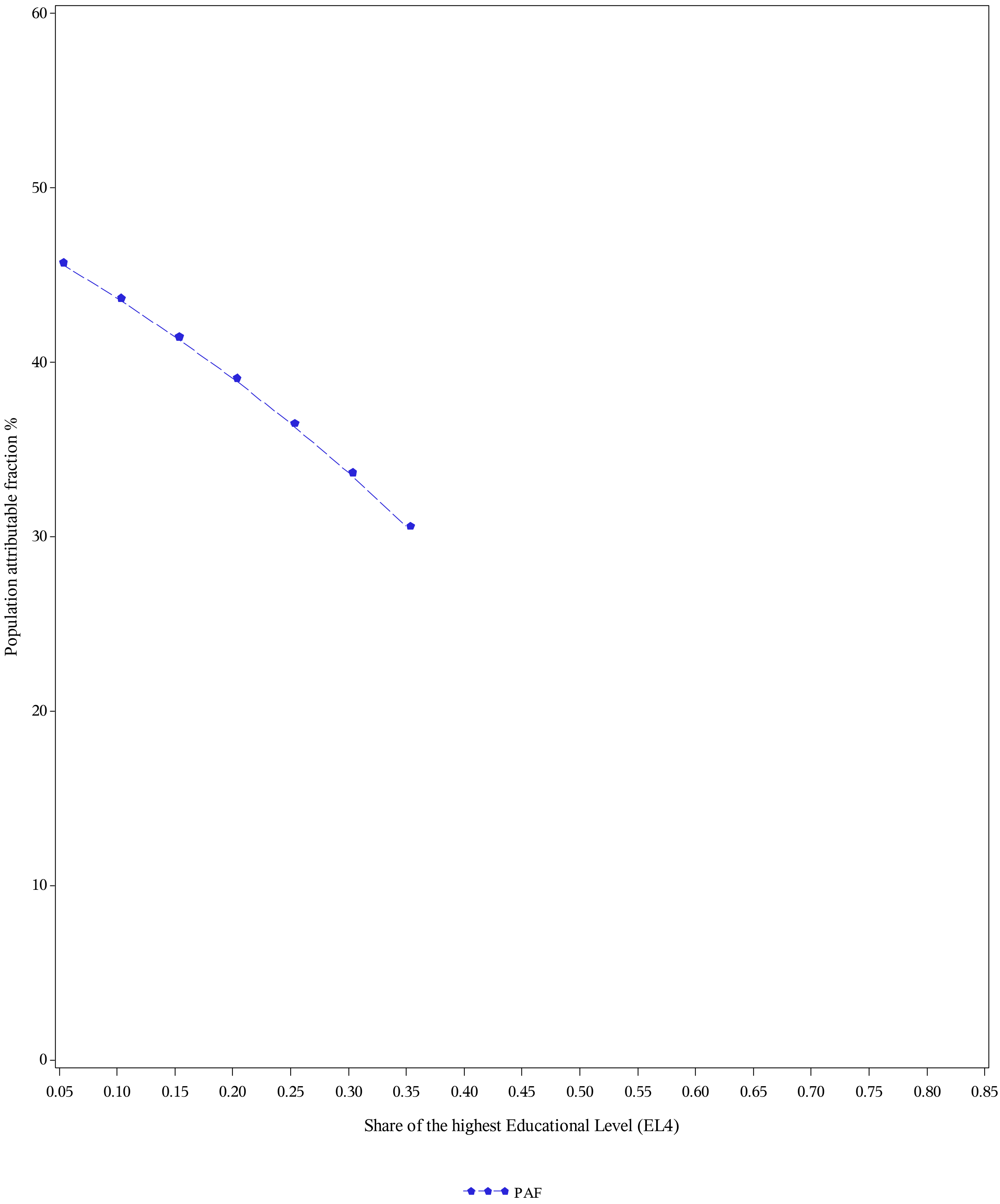

PAF in function of the share of EL4

When EL2 and EL3 are fixed at: EL2=35% ; EL3=30%
EL1 =1- EL4 - EL2 - EL3

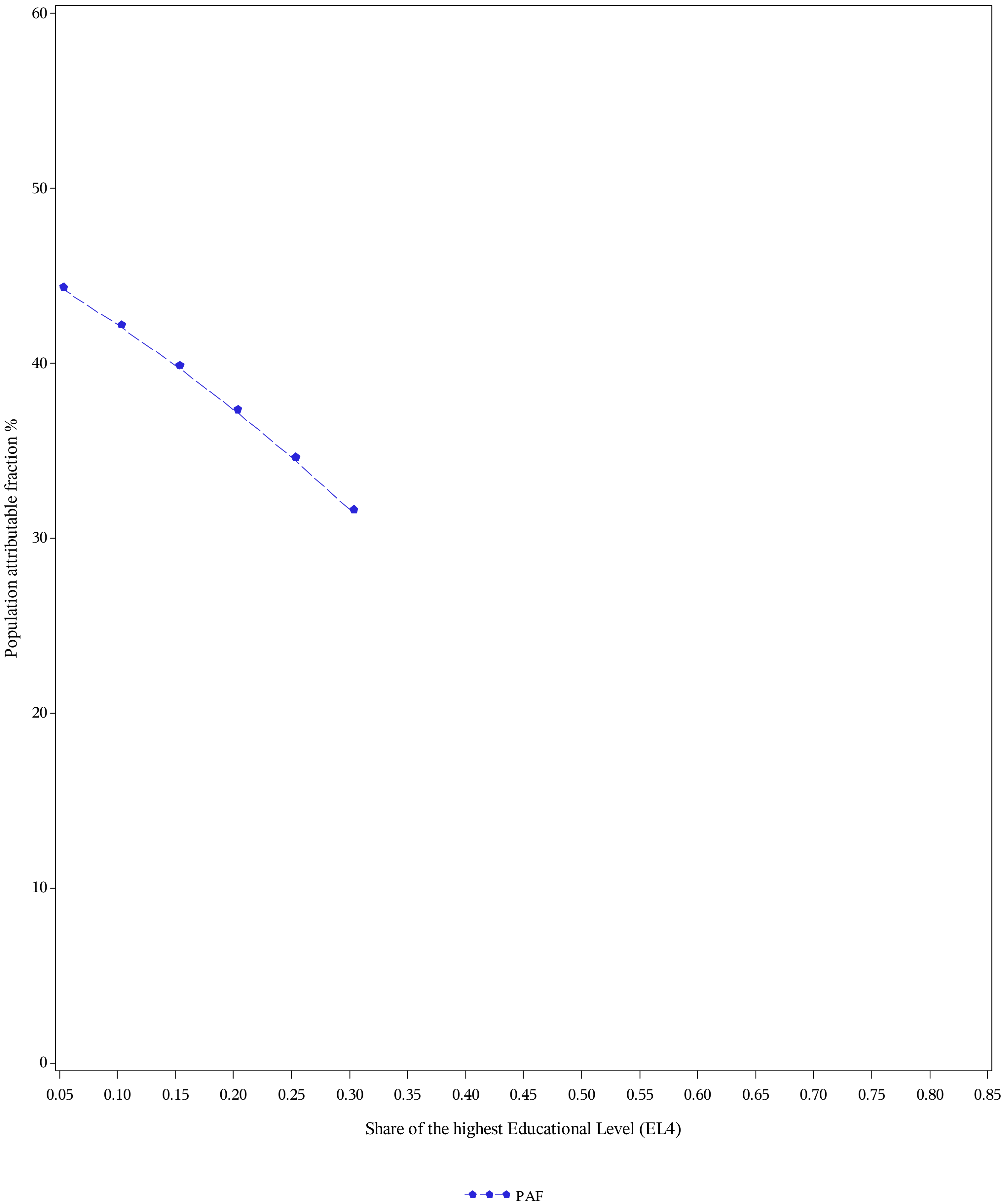

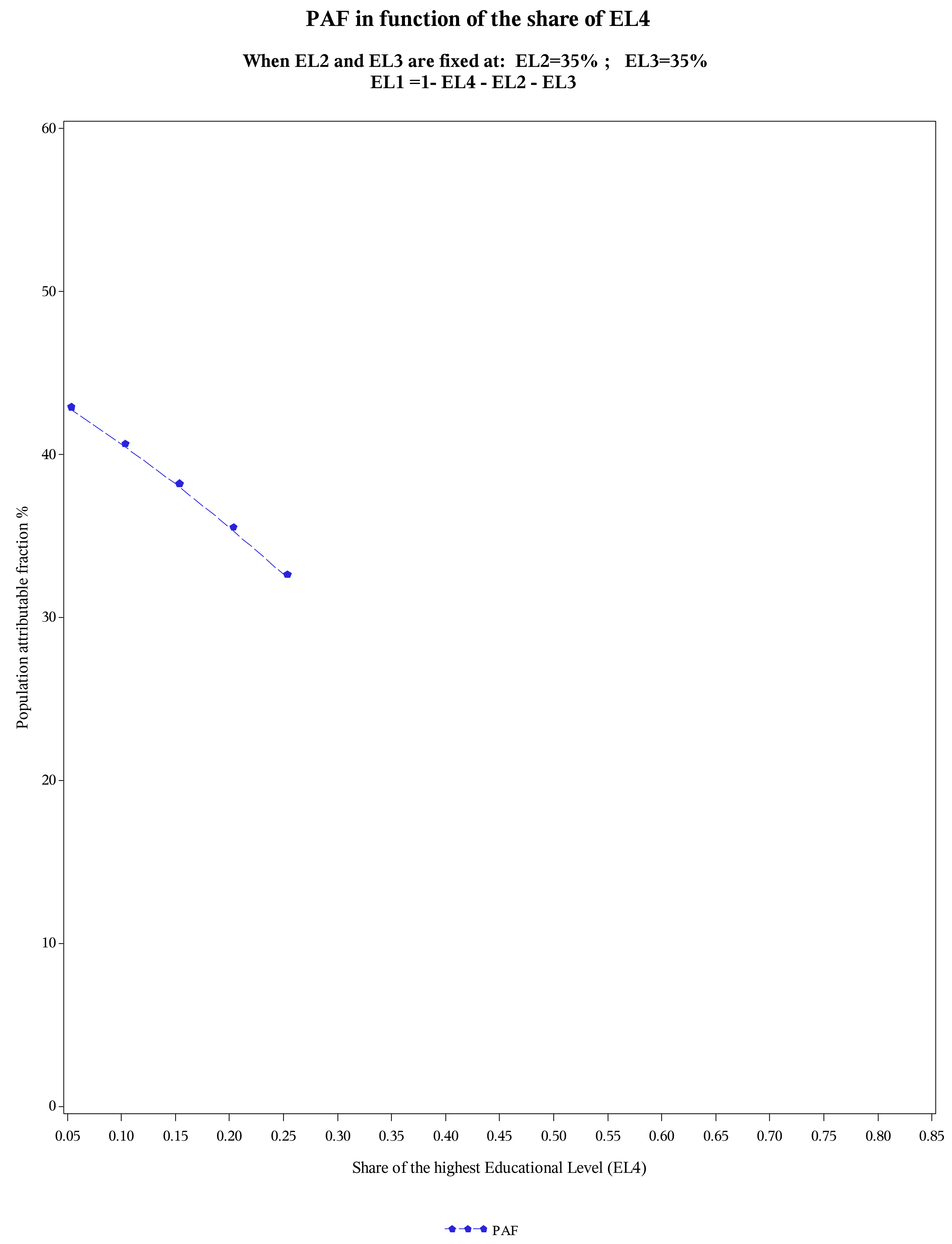
PAF in function of the share of EL4
When EL2 and EL3 are fixed at: EL2=35% ; EL3=35%
EL1 =1- EL4 - EL2 - EL3
Population attributable fraction %
0
10
20
30
40
50
60
0.05
0.10
0.15
0.20
0.25
0.30
0.35
0.40
0.45
0.50
0.55
0.60
0.65
0.70
0.75
0.80
0.85
Share of the highest Educational Level (EL4)
PAF

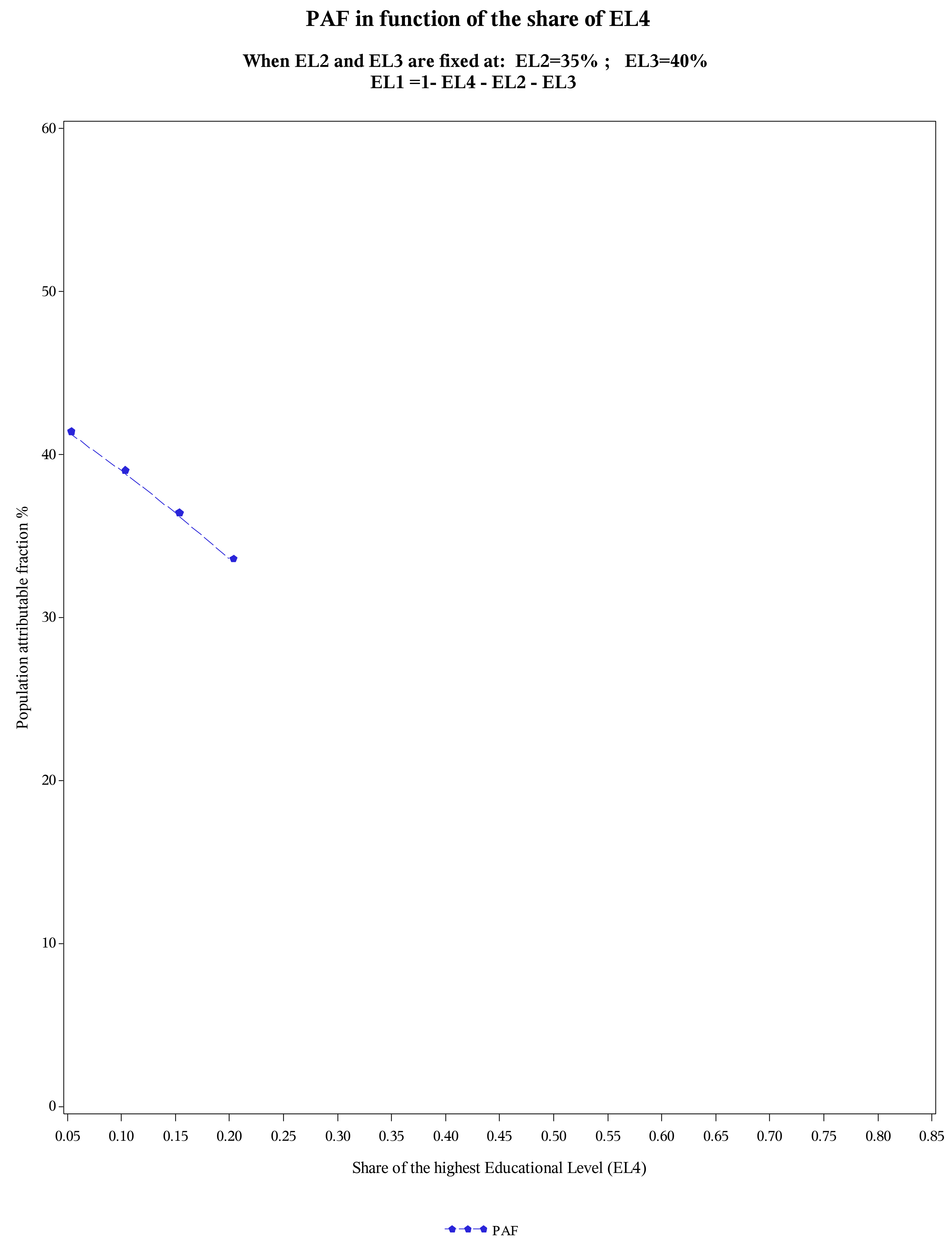

PAF in function of the share of EL4
When EL2 and EL3 are fixed at: EL2=35% ; EL3=40%
EL1 =1- EL4 - EL2 - EL3
Population attributable fraction %
0
10
20
30
40
50
60
0.05
0.10
0.15
0.20
0.25
0.30
0.35
0.40
0.45
0.50
0.55
0.60
0.65
0.70
0.75
0.80
0.85
Share of the highest Educational Level (EL4)
PAF

PAF in function of the share of EL4

When EL2 and EL3 are fixed at: EL2=35% ; EL3=45%
EL1 =1- EL4 - EL2 - EL3

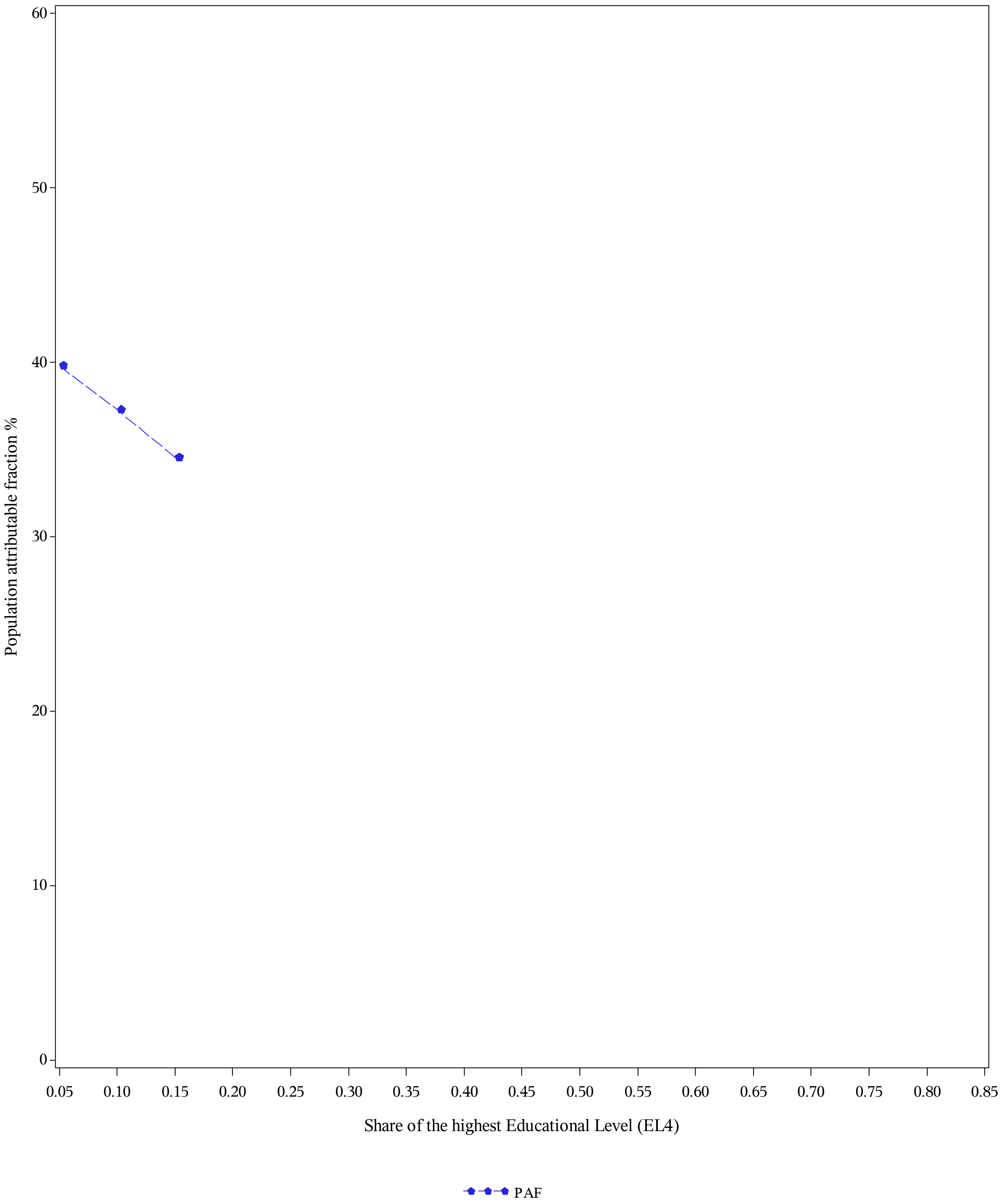

PAF in function of the share of EL4

When EL2 and EL3 are fixed at: EL2=35% ; EL3=50%
EL1 =1- EL4 - EL2 - EL3

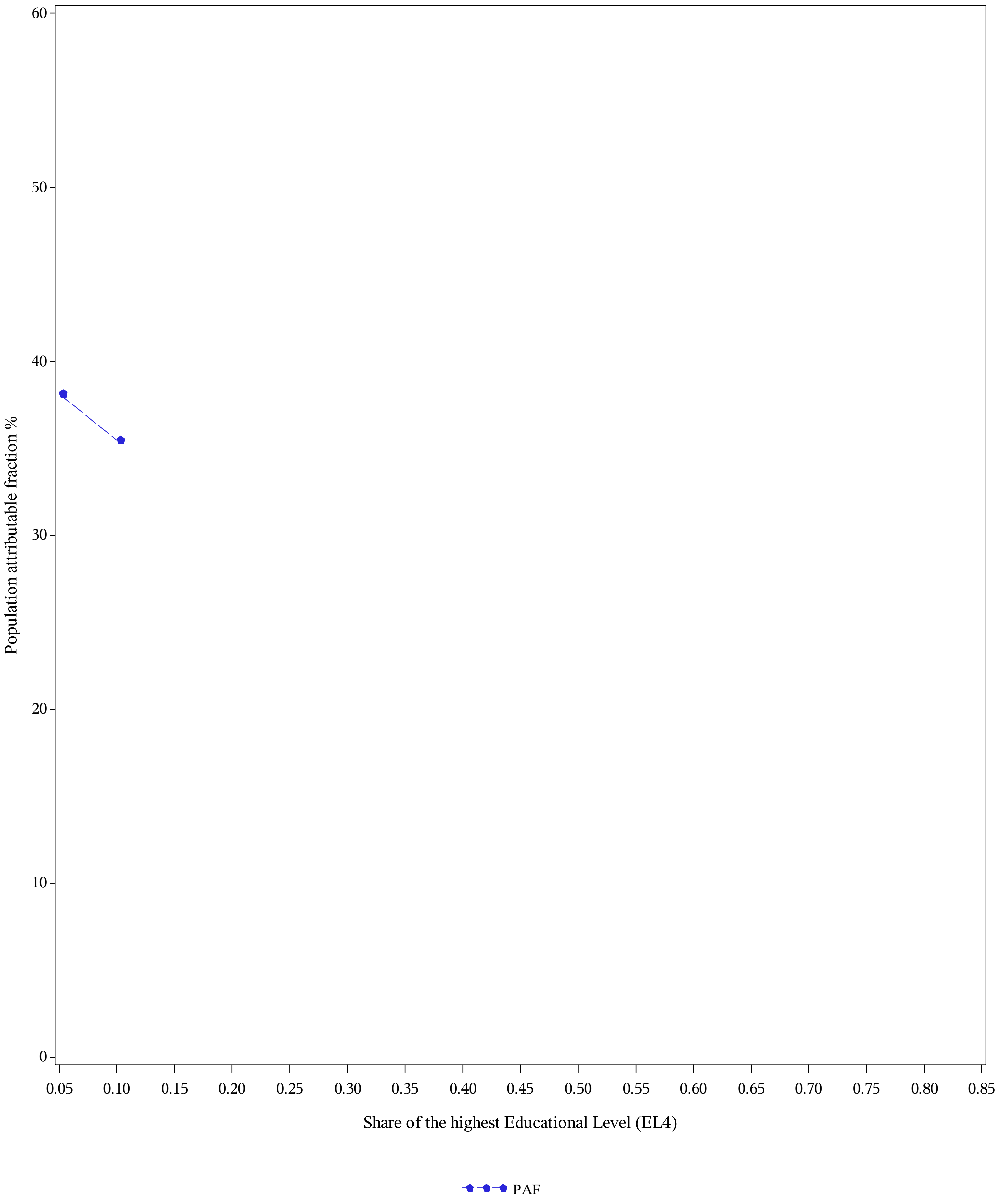

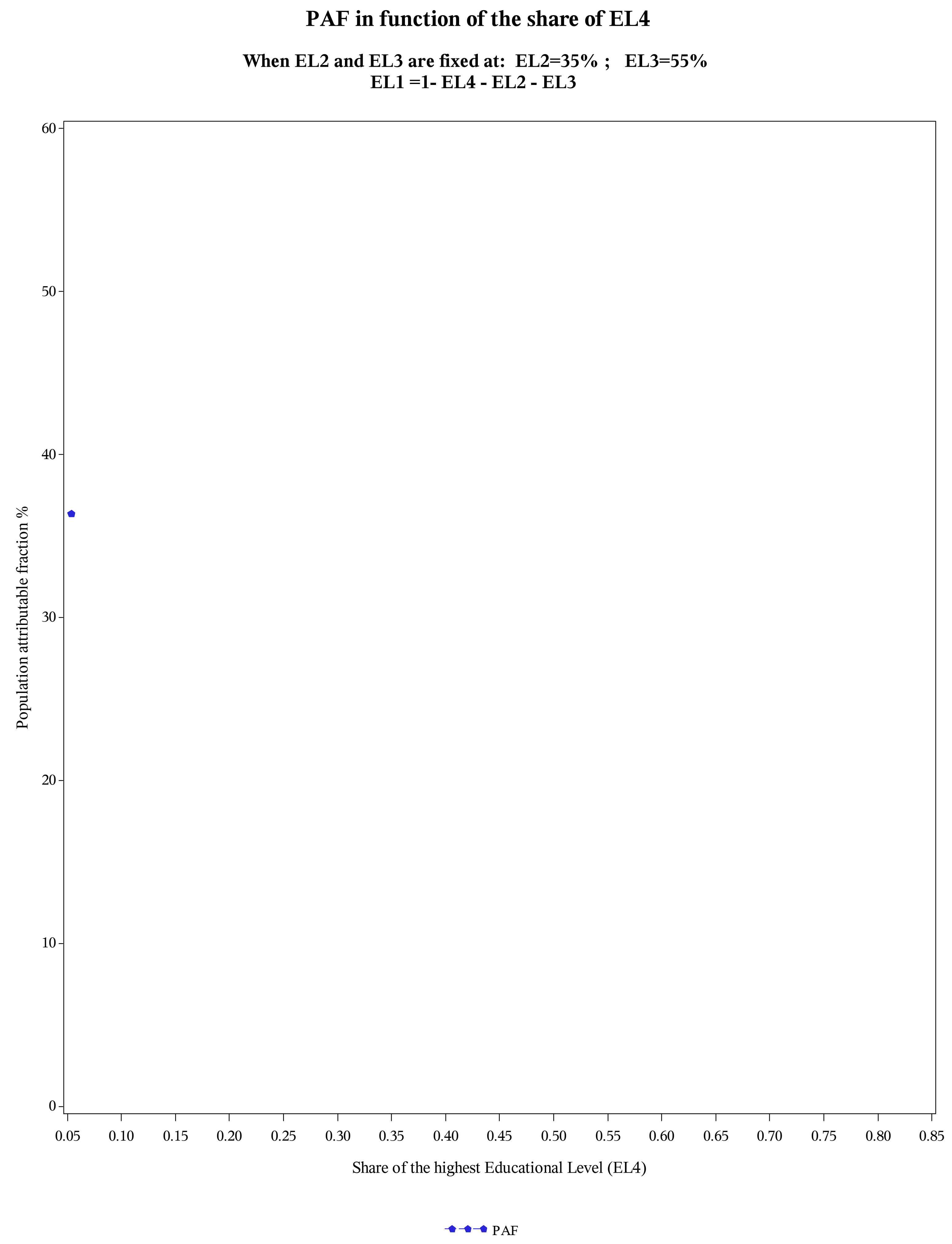
PAF in function of the share of EL4
When EL2 and EL3 are fixed at: EL2=35% ; EL3=55%
EL1 =1- EL4 - EL2 - EL3
Population attributable fraction %
0
10
20
30
40
50
60
0.05
0.10
0.15
0.20
0.25
0.30
0.35
0.40
0.45
0.50
0.55
0.60
0.65
0.70
0.75
0.80
0.85
Share of the highest Educational Level (EL4)
PAF

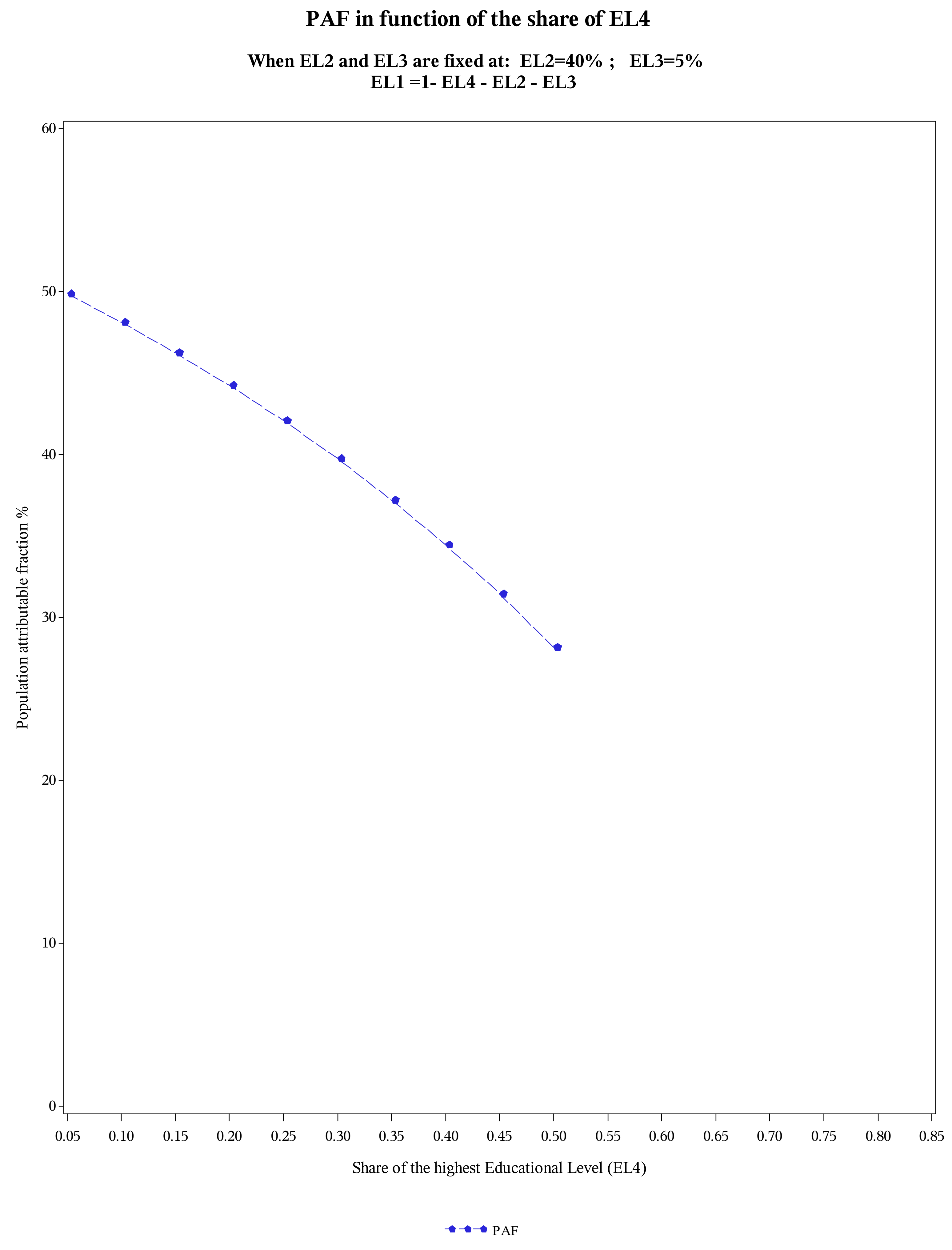
PAF in function of the share of EL4
When EL2 and EL3 are fixed at: EL2=40% ; EL3=5%
EL1 =1- EL4 - EL2 - EL3
Population attributable fraction %
0
10
20
30
40
50
60
0.05
0.10
0.15
0.20
0.25
0.30
0.35
0.40
0.45
0.50
0.55
0.60
0.65
0.70
0.75
0.80
0.85
Share of the highest Educational Level (EL4)
PAF

PAF in function of the share of EL4

When EL2 and EL3 are fixed at: EL2=40% ; EL3=10%
EL1 =1- EL4 - EL2 - EL3

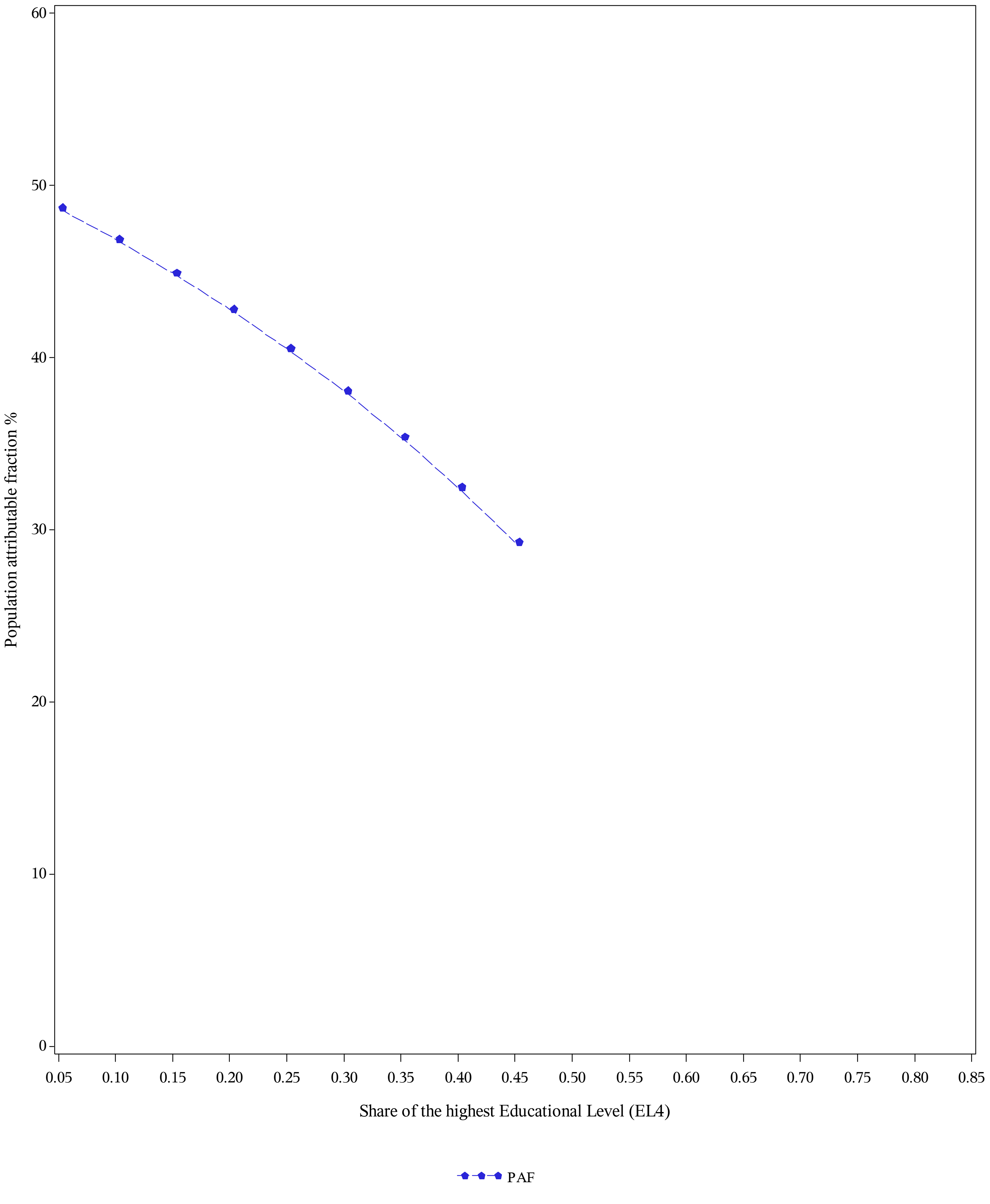

PAF in function of the share of EL4

When EL2 and EL3 are fixed at: EL2=40% ; EL3=15%
EL1 =1- EL4 - EL2 - EL3

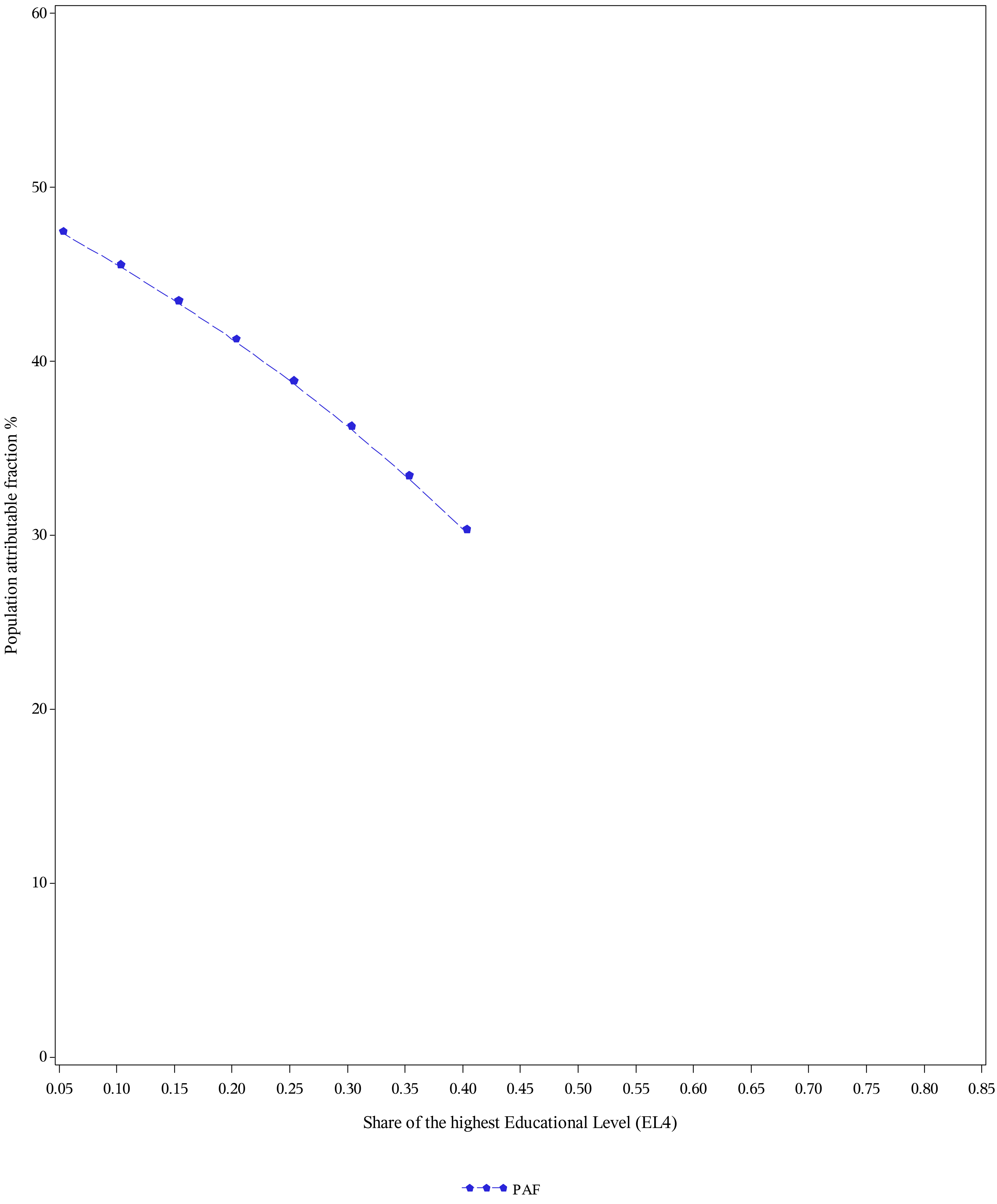

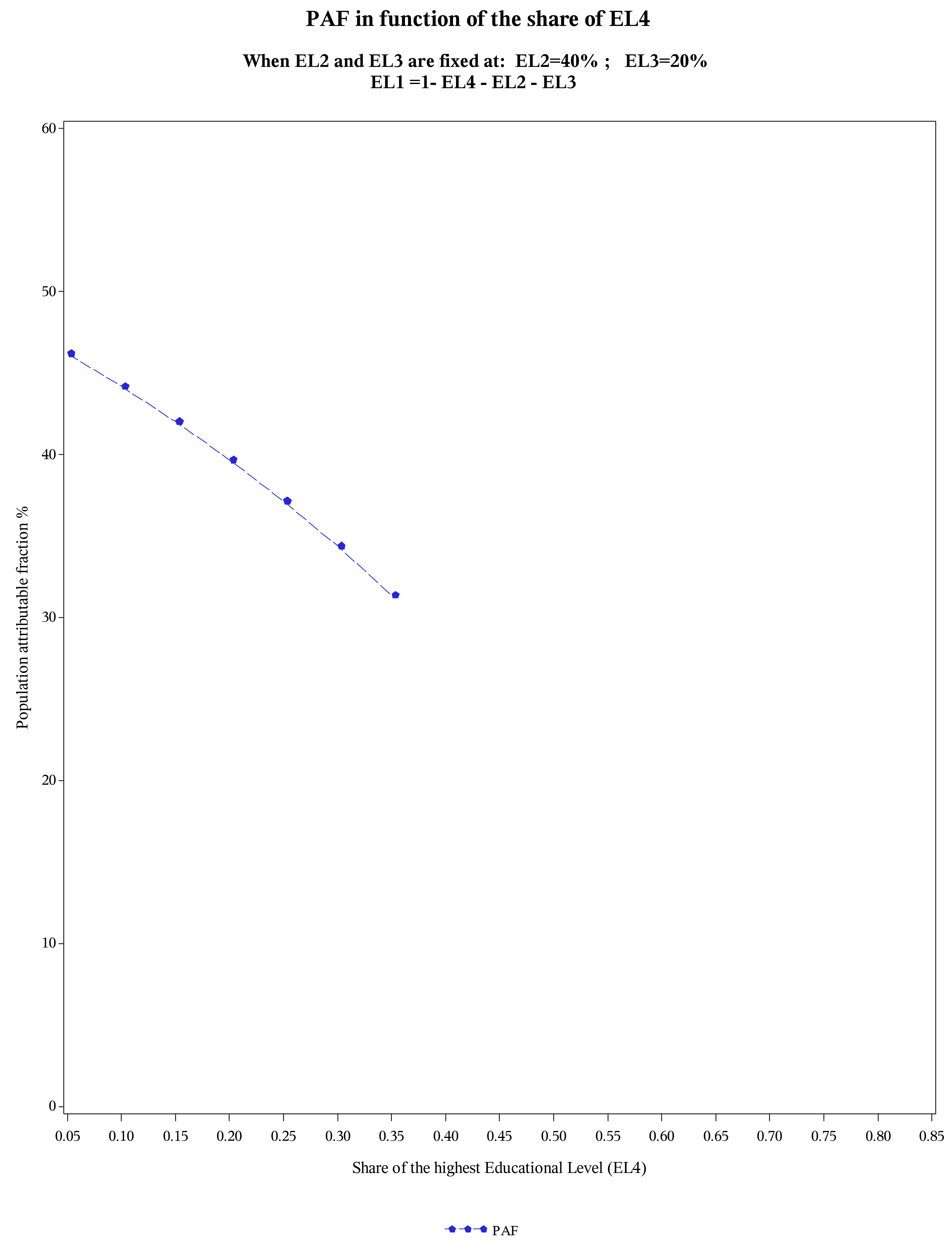
PAF in function of the share of EL4
When EL2 and EL3 are fixed at: EL2=40% ; EL3=20%
EL1 =1- EL4 - EL2 - EL3
Population attributable fraction %
0
10
20
30
40
50
60
0.05
0.10
0.15
0.20
0.25
0.30
0.35
0.40
0.45
0.50
0.55
0.60
0.65
0.70
0.75
0.80
0.85
Share of the highest Educational Level (EL4)
PAF

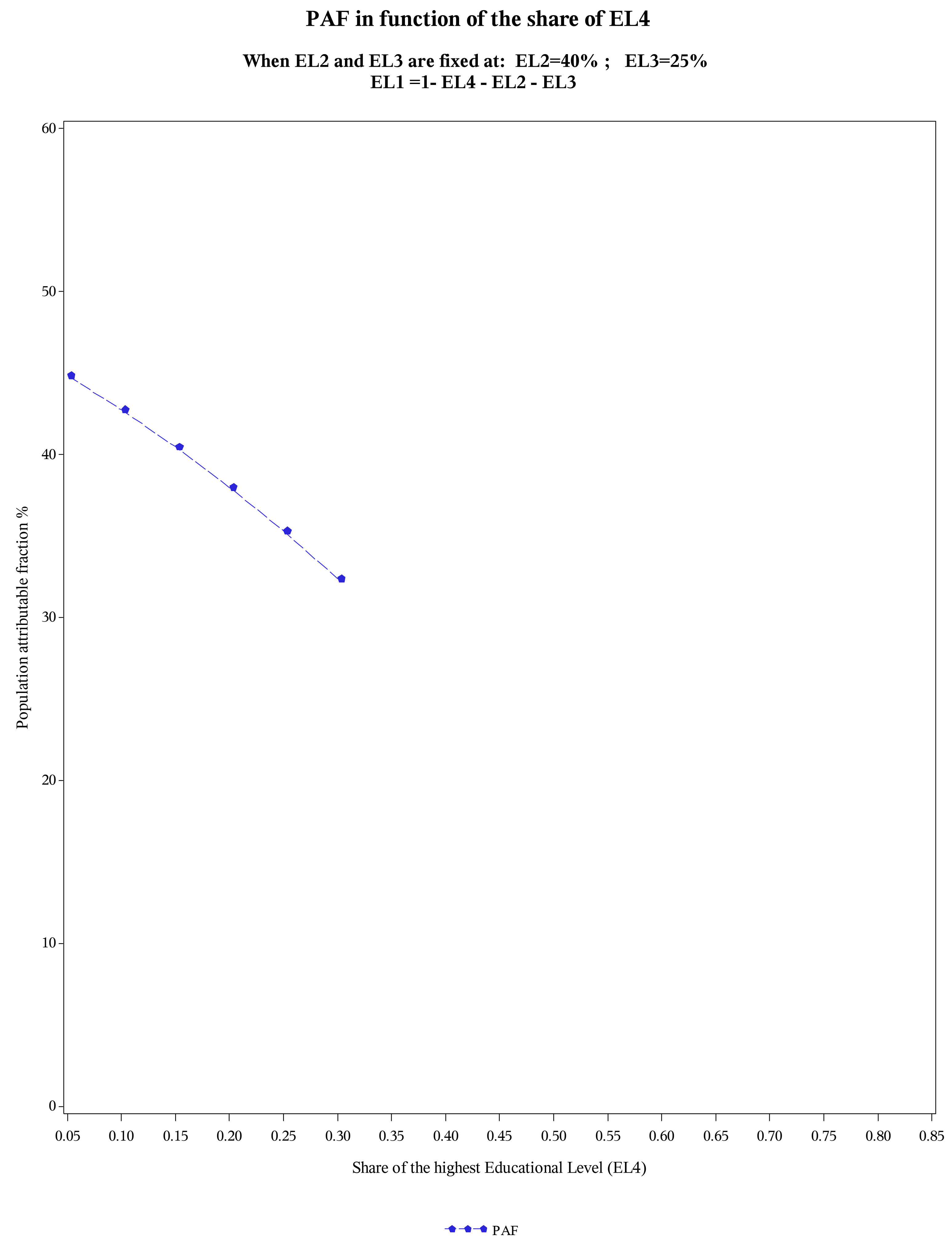
PAF in function of the share of EL4
When EL2 and EL3 are fixed at: EL2=40% ; EL3=25%
EL1 =1- EL4 - EL2 - EL3
Population attributable fraction %
0
10
20
30
40
50
60
0.05
0.10
0.15
0.20
0.25
0.30
0.35
0.40
0.45
0.50
0.55
0.60
0.65
0.70
0.75
0.80
0.85
Share of the highest Educational Level (EL4)
PAF

PAF in function of the share of EL4

When EL2 and EL3 are fixed at: EL2=40% ; EL3=30%
EL1 =1- EL4 - EL2 - EL3

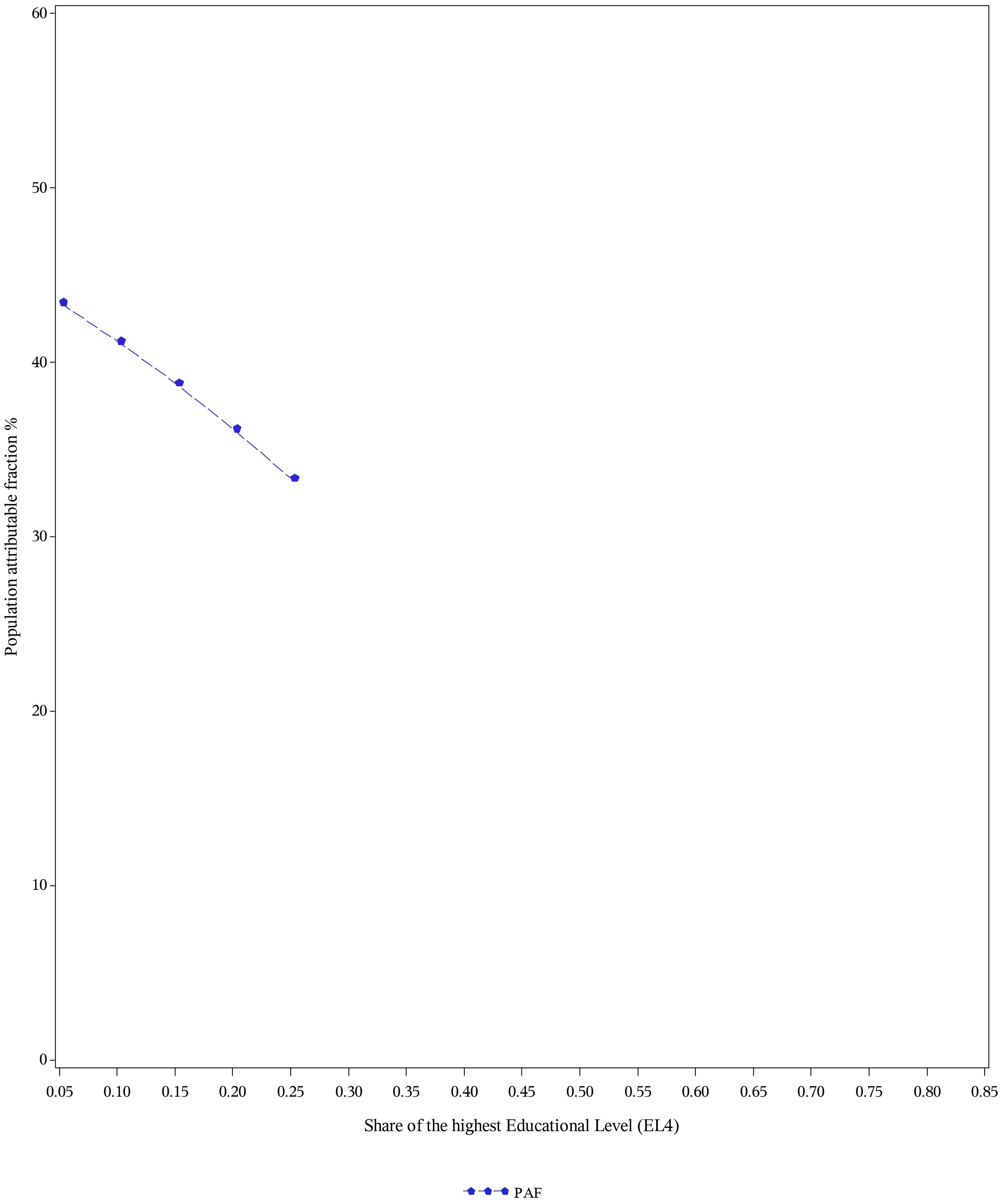

PAF in function of the share of EL4

When EL2 and EL3 are fixed at: EL2=40% ; EL3=35%
EL1 =1- EL4 - EL2 - EL3

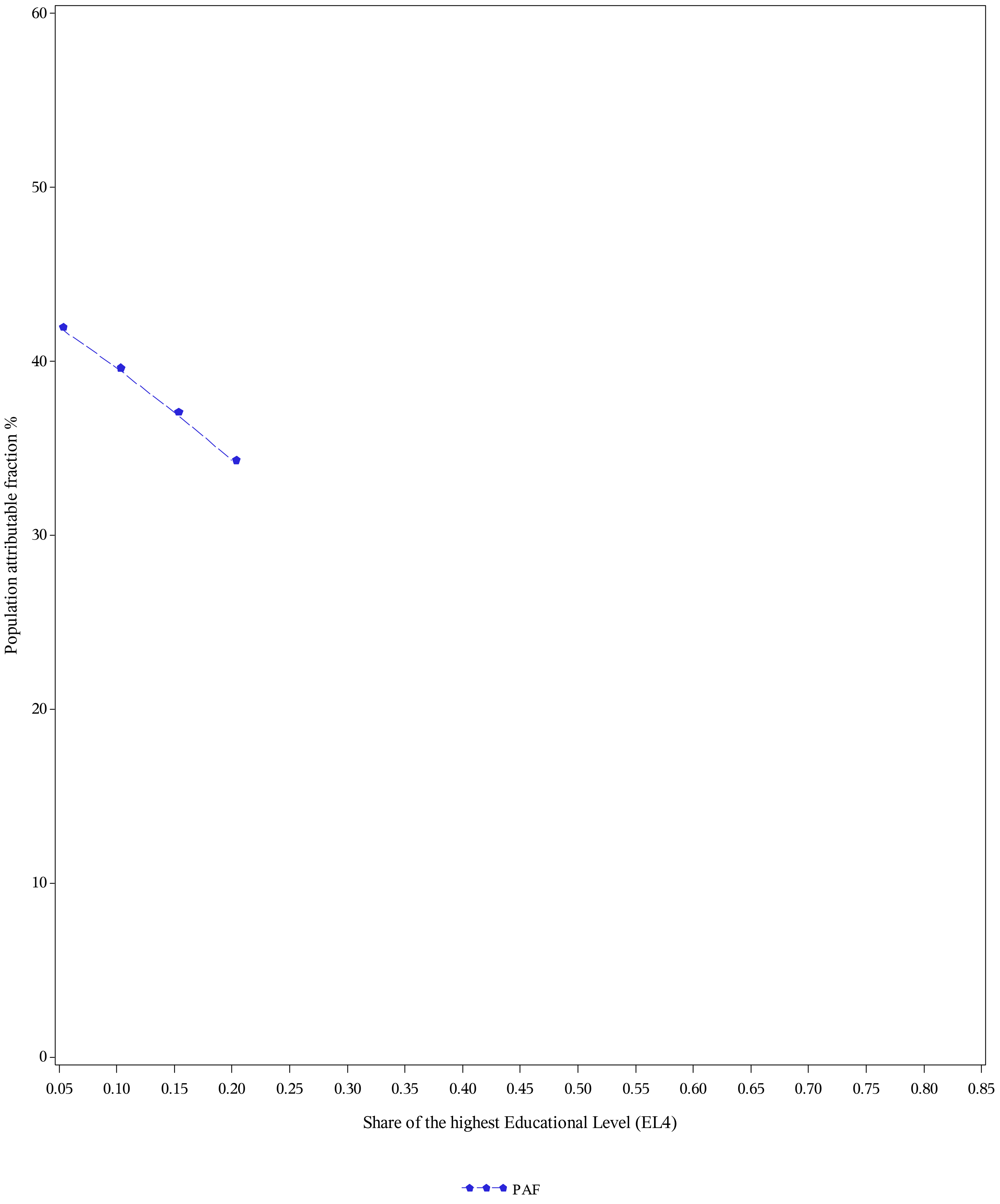

PAF in function of the share of EL4

When EL2 and EL3 are fixed at: EL2=40% ; EL3=40%
EL1 =1- EL4 - EL2 - EL3

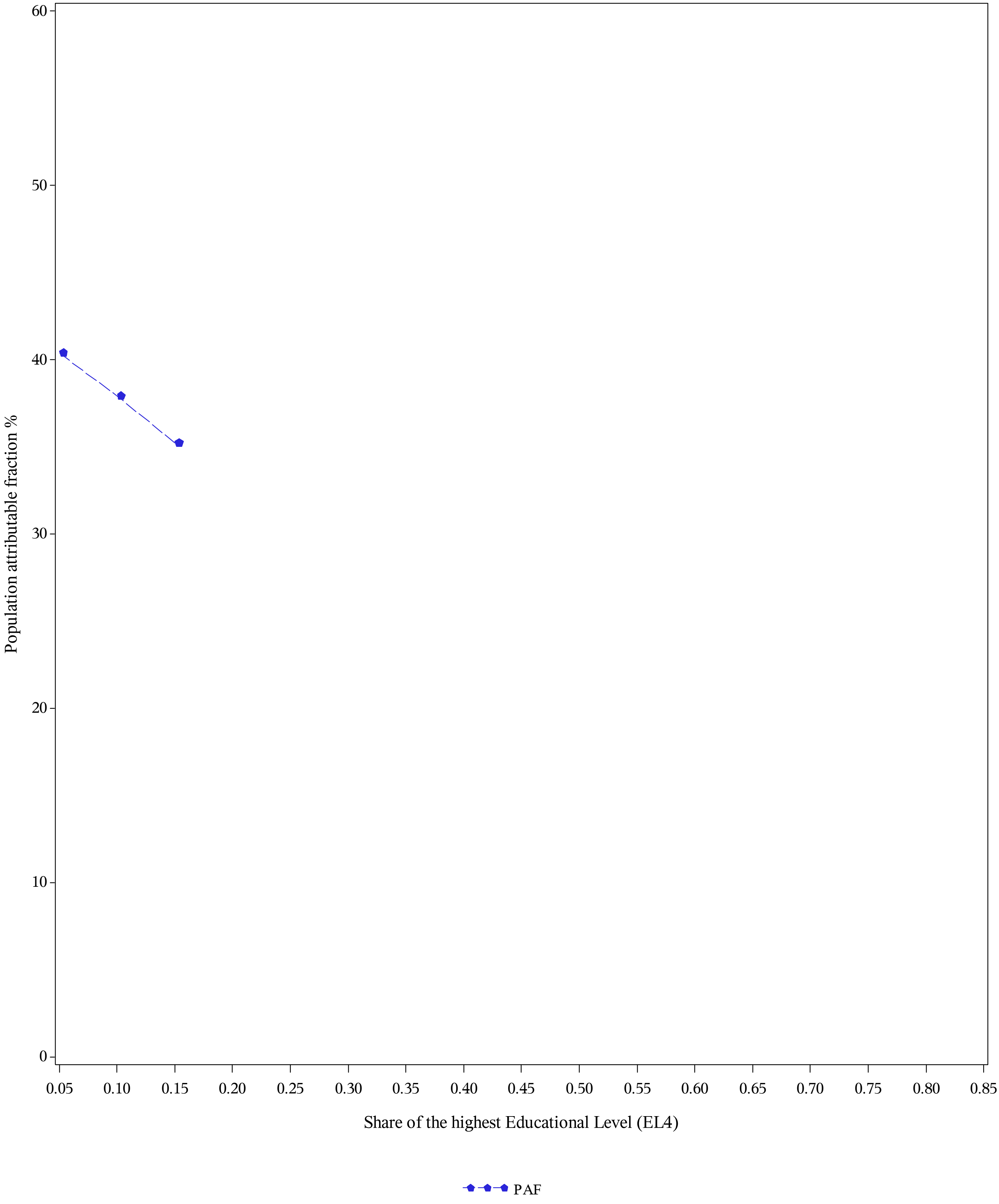

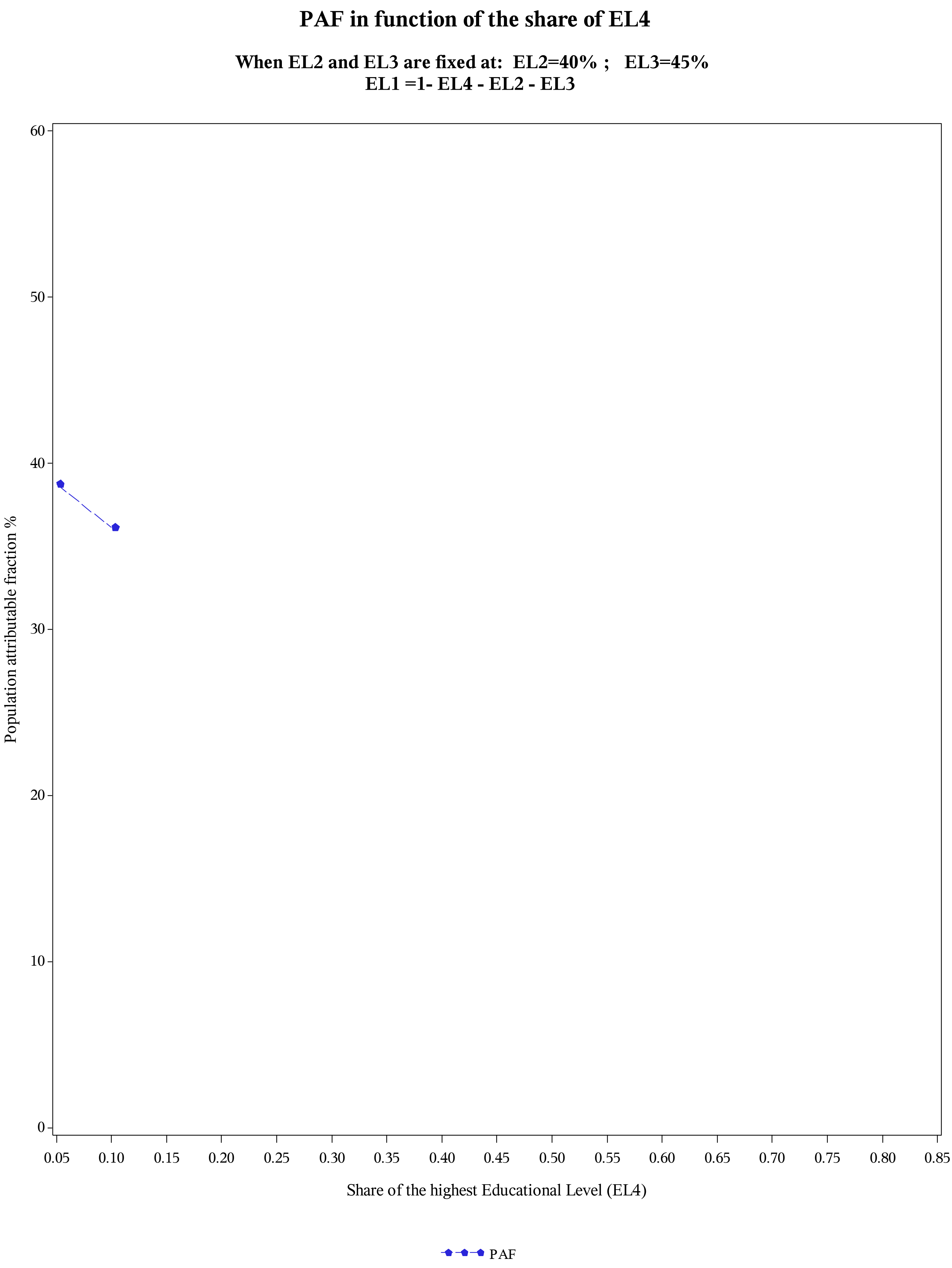
PAF in function of the share of EL4
When EL2 and EL3 are fixed at: EL2=40% ; EL3=45%
EL1 =1- EL4 - EL2 - EL3
Population attributable fraction %
0
10
20
30
40
50
60
0.05
0.10
0.15
0.20
0.25
0.30
0.35
0.40
0.45
0.50
0.55
0.60
0.65
0.70
0.75
0.80
0.85
Share of the highest Educational Level (EL4)
PAF

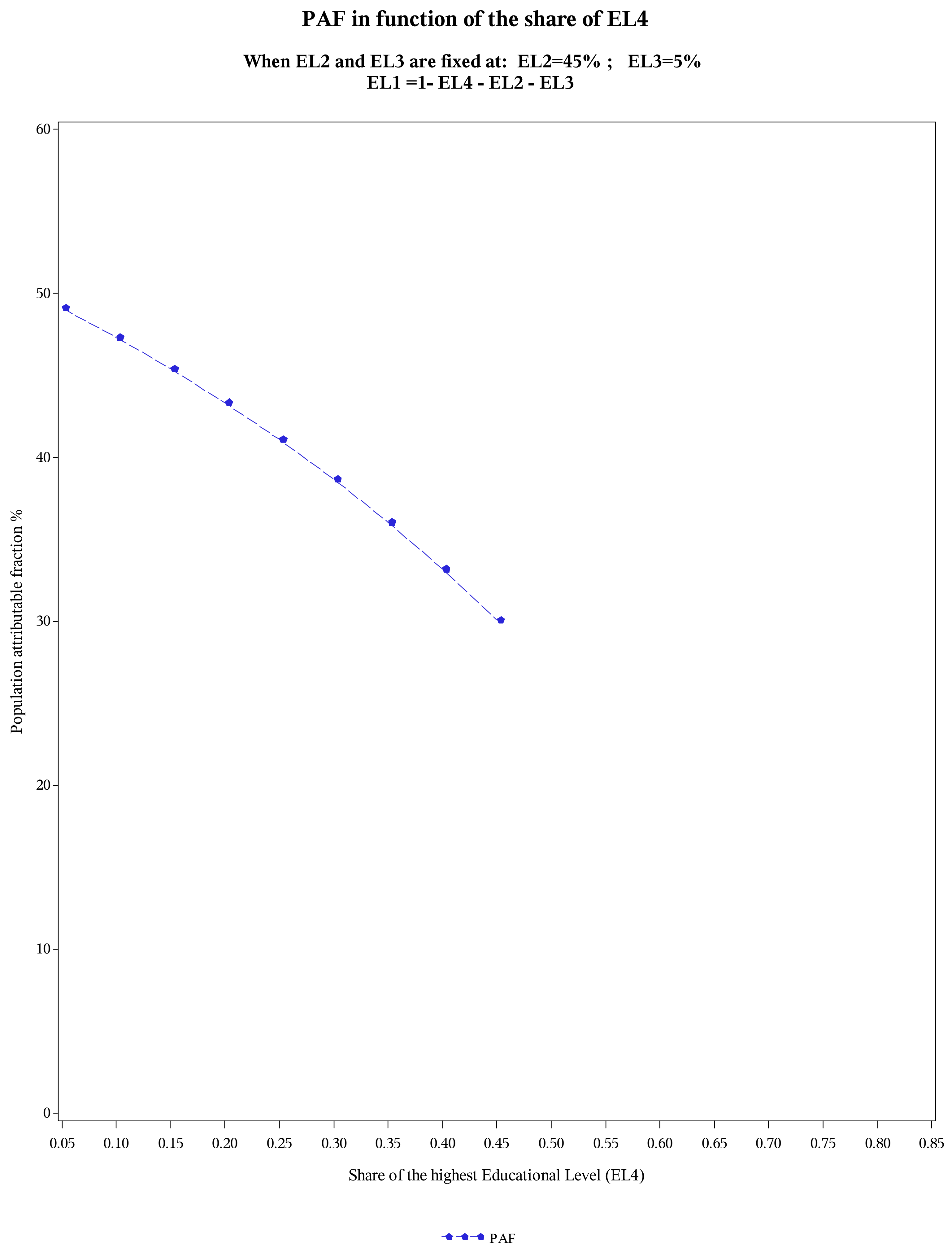

PAF in function of the share of EL4
When EL2 and EL3 are fixed at: EL2=45% ; EL3=5%
EL1 =1- EL4 - EL2 - EL3
Population attributable fraction %
0
10
20
30
40
50
60
0.05
0.10
0.15
0.20
0.25
0.30
0.35
0.40
0.45
0.50
0.55
0.60
0.65
0.70
0.75
0.80
0.85
Share of the highest Educational Level (EL4)
PAF

PAF in function of the share of EL4

When EL2 and EL3 are fixed at: EL2=45% ; EL3=10%
EL1 =1- EL4 - EL2 - EL3

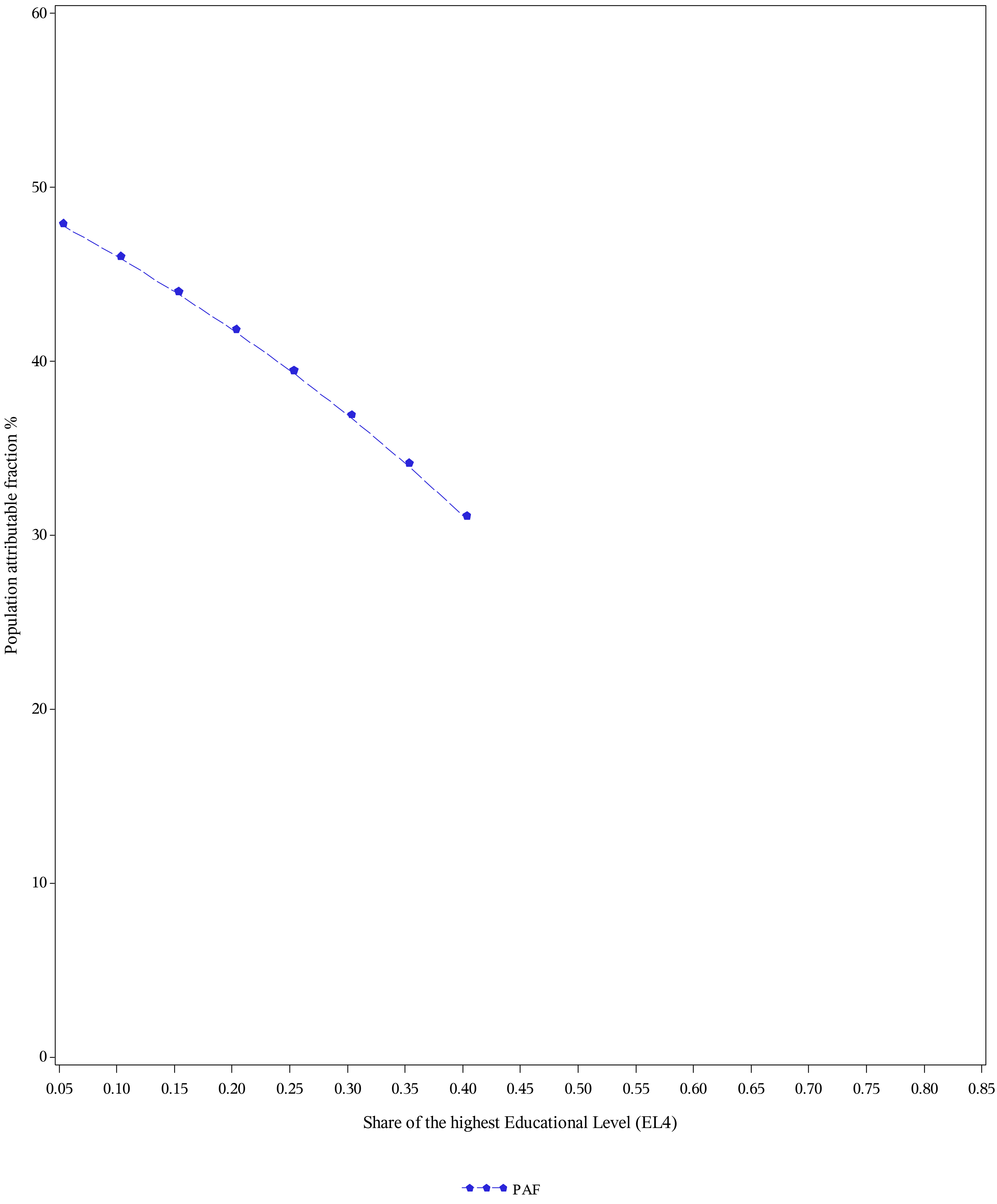

PAF in function of the share of EL4

When EL2 and EL3 are fixed at: EL2=45% ; EL3=15%
EL1 =1- EL4 - EL2 - EL3

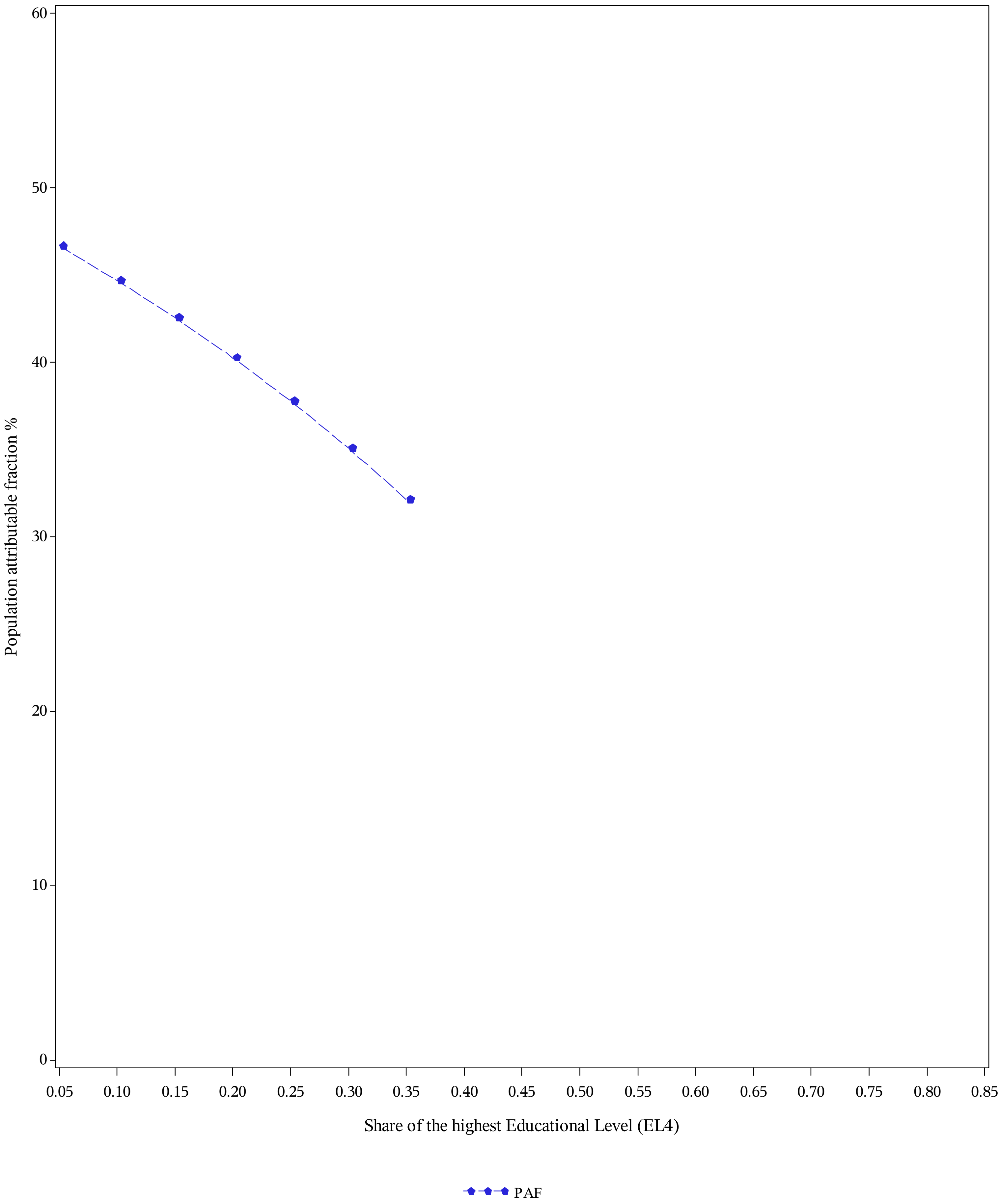

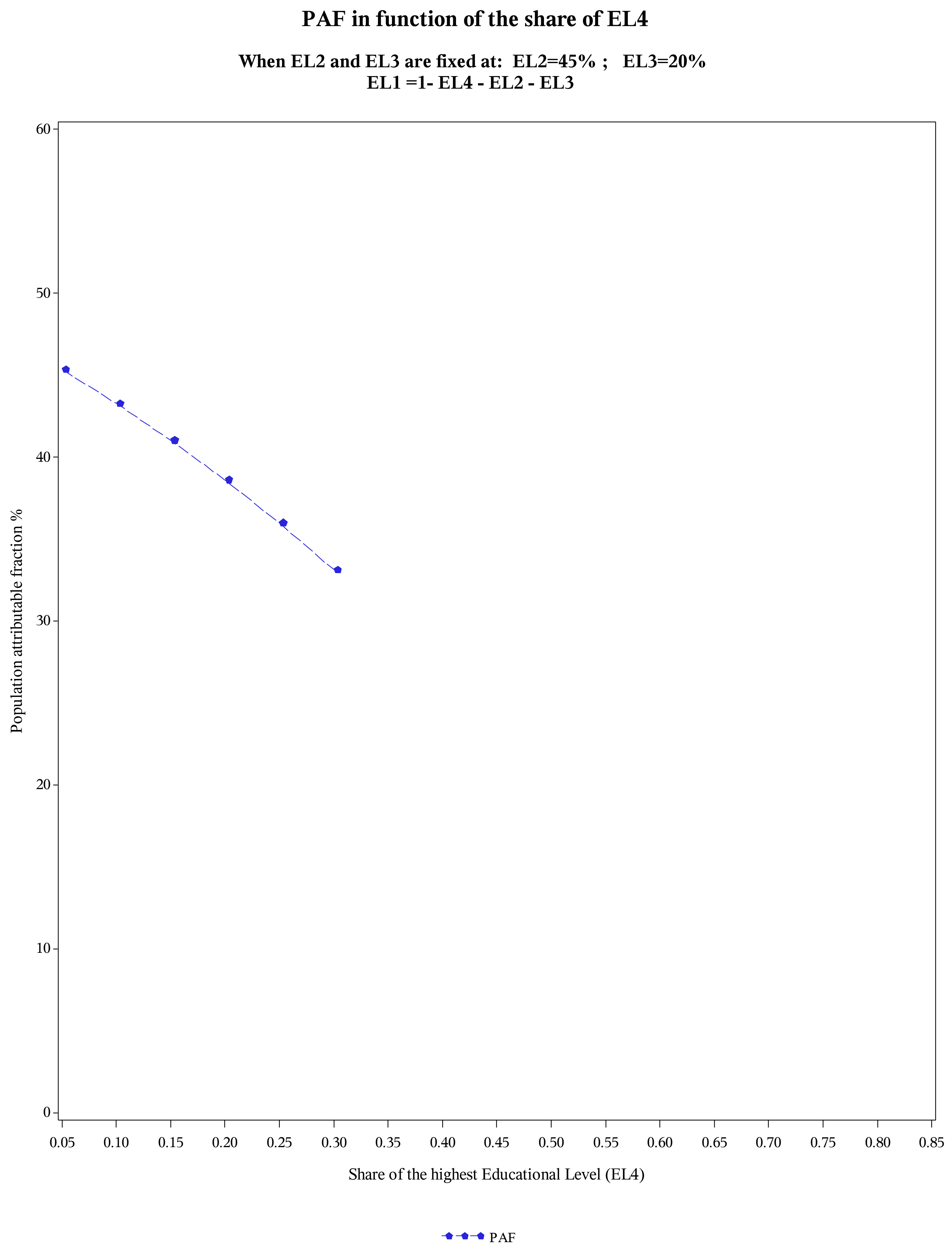
PAF in function of the share of EL4
When EL2 and EL3 are fixed at: EL2=45% ; EL3=20%
EL1 =1- EL4 - EL2 - EL3
Population attributable fraction %
0
10
20
30
40
50
60
0.05
0.10
0.15
0.20
0.25
0.30
0.35
0.40
0.45
0.50
0.55
0.60
0.65
0.70
0.75
0.80
0.85
Share of the highest Educational Level (EL4)
PAF

PAF in function of the share of EL4

When EL2 and EL3 are fixed at: EL2=45% ; EL3=25%
EL1 =1- EL4 - EL2 - EL3

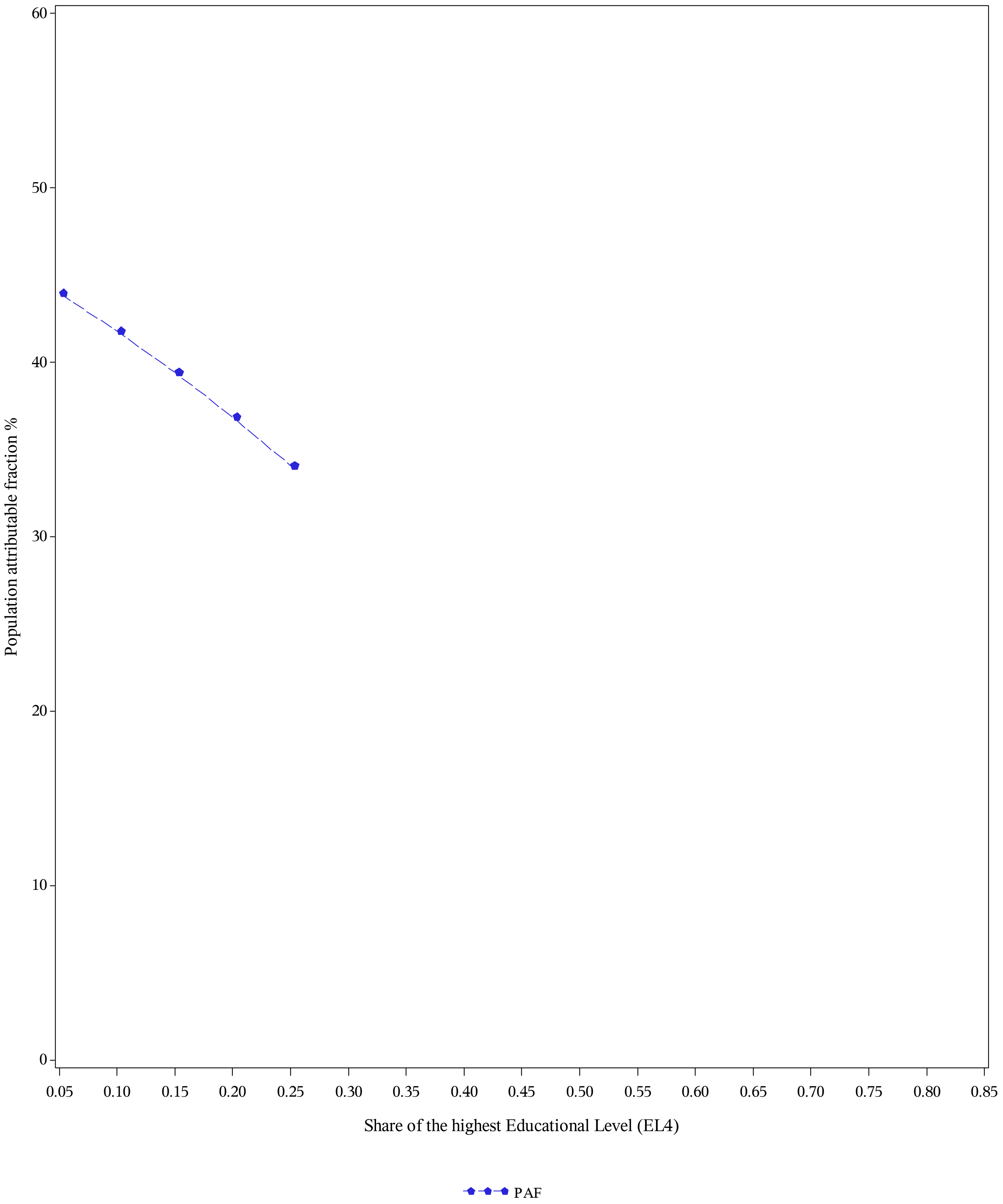

PAF in function of the share of EL4

When EL2 and EL3 are fixed at: EL2=45% ; EL3=30%
EL1 =1- EL4 - EL2 - EL3

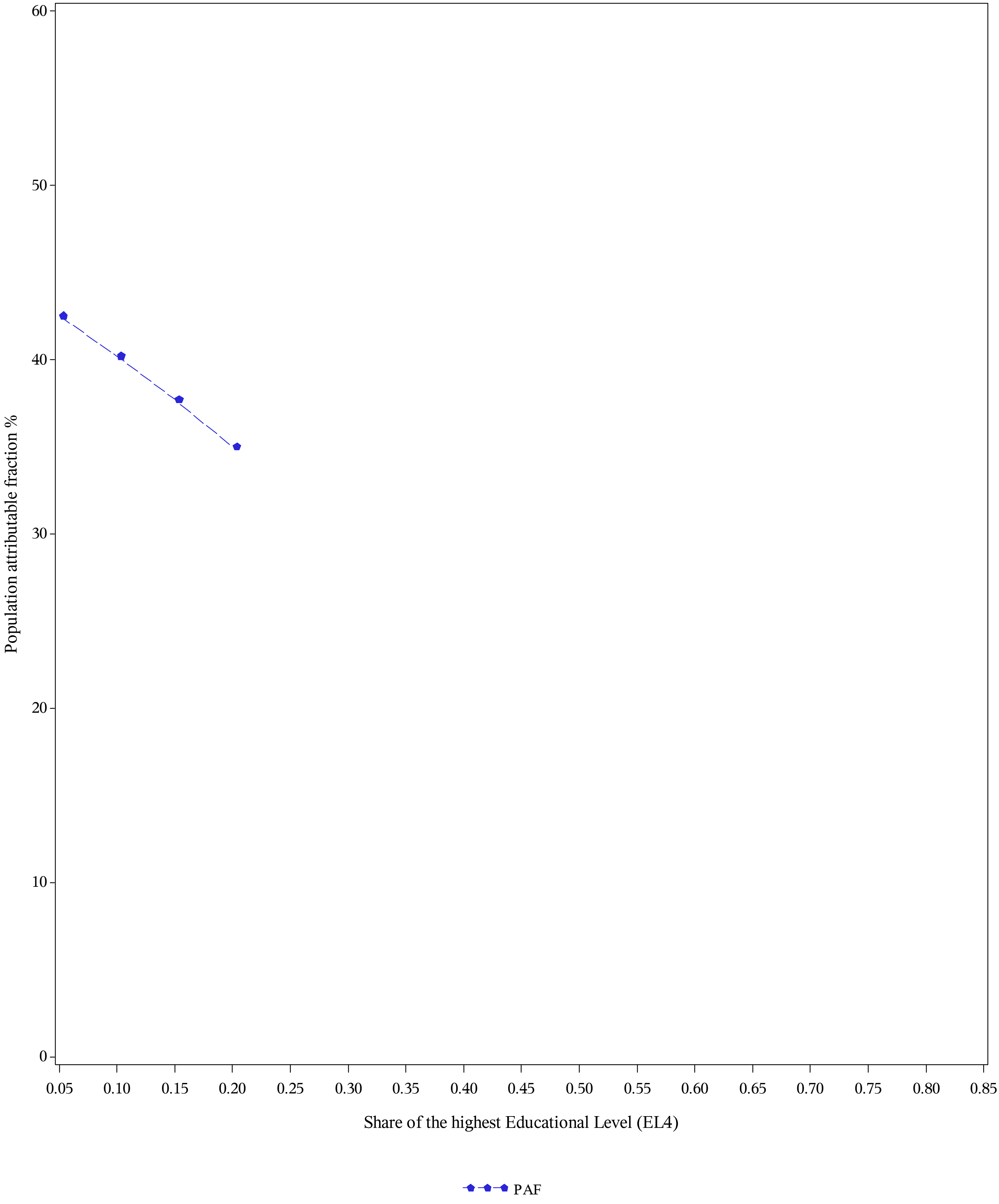

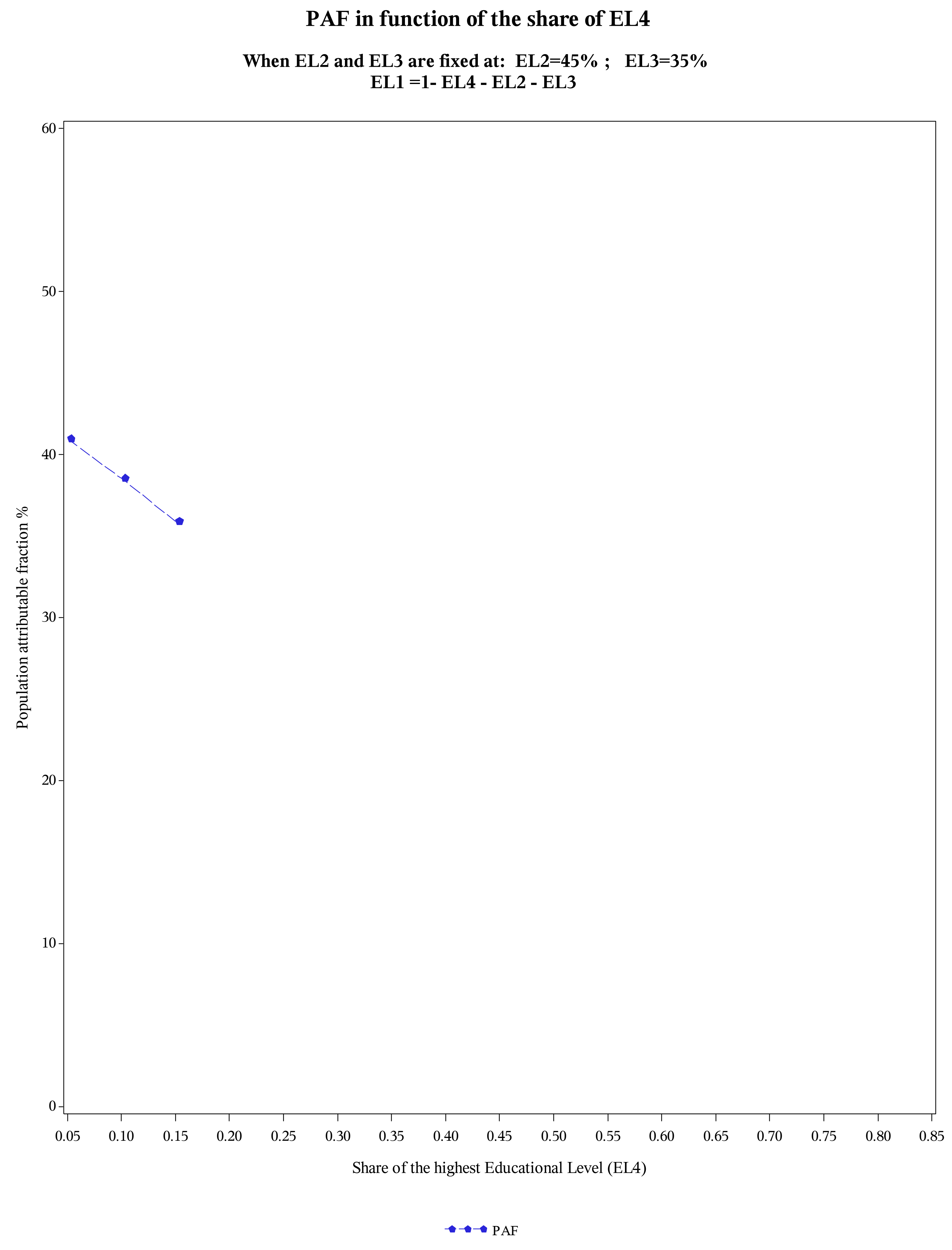
PAF in function of the share of EL4
When EL2 and EL3 are fixed at: EL2=45% ; EL3=35%
EL1 =1- EL4 - EL2 - EL3
Population attributable fraction %
0
10
20
30
40
50
60
0.05
0.10
0.15
0.20
0.25
0.30
0.35
0.40
0.45
0.50
0.55
0.60
0.65
0.70
0.75
0.80
0.85
Share of the highest Educational Level (EL4)
PAF

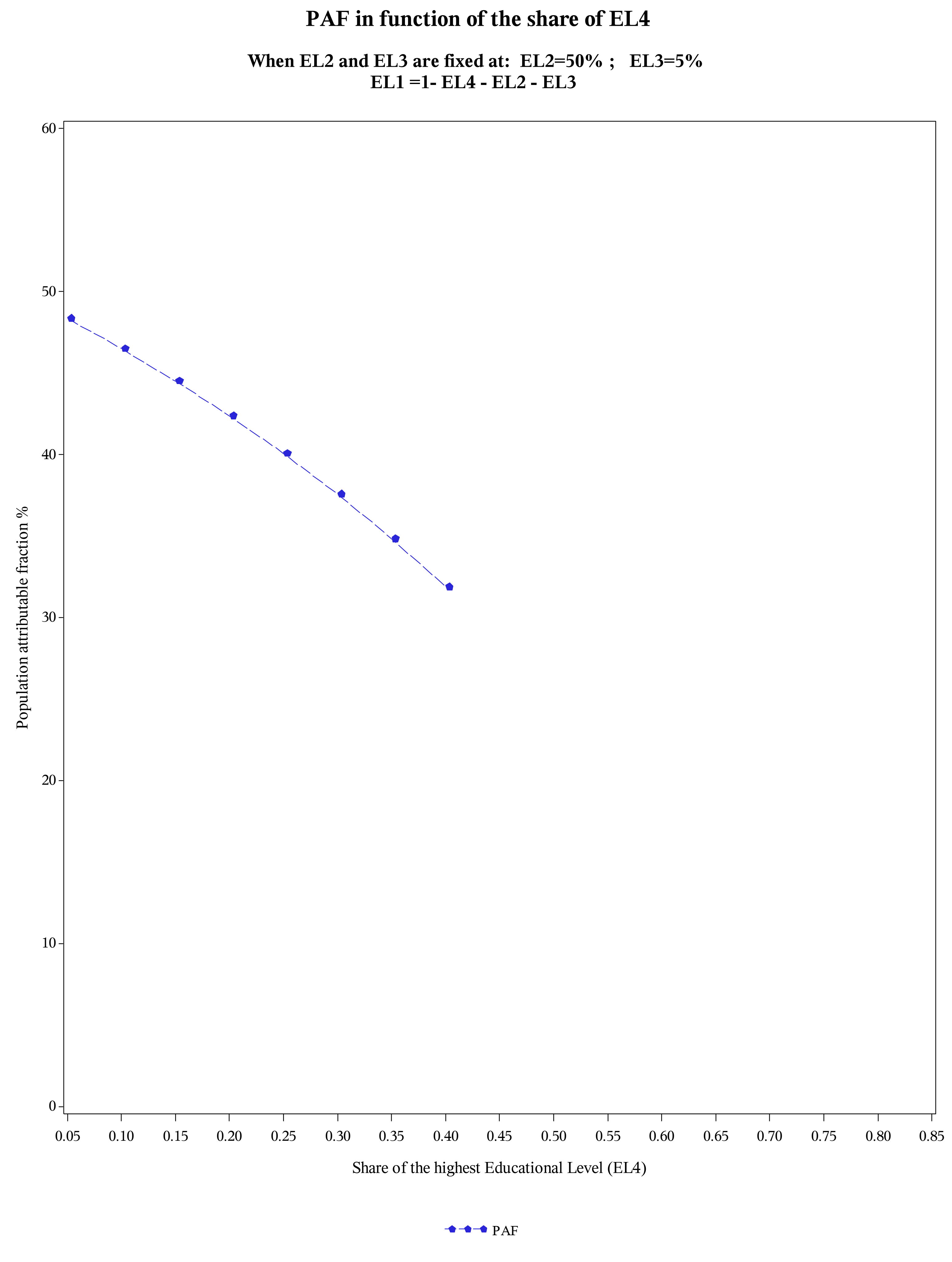
PAF in function of the share of EL4
When EL2 and EL3 are fixed at: EL2=50% ; EL3=5%
EL1 =1- EL4 - EL2 - EL3
Population attributable fraction %
0
10
20
30
40
50
60
0.05
0.10
0.15
0.20
0.25
0.30
0.35
0.40
0.45
0.50
0.55
0.60
0.65
0.70
0.75
0.80
0.85
Share of the highest Educational Level (EL4)
PAF

PAF in function of the share of EL4

When EL2 and EL3 are fixed at: EL2=50% ;   EL3=10%
EL1 =1- EL4 - EL2 - EL3

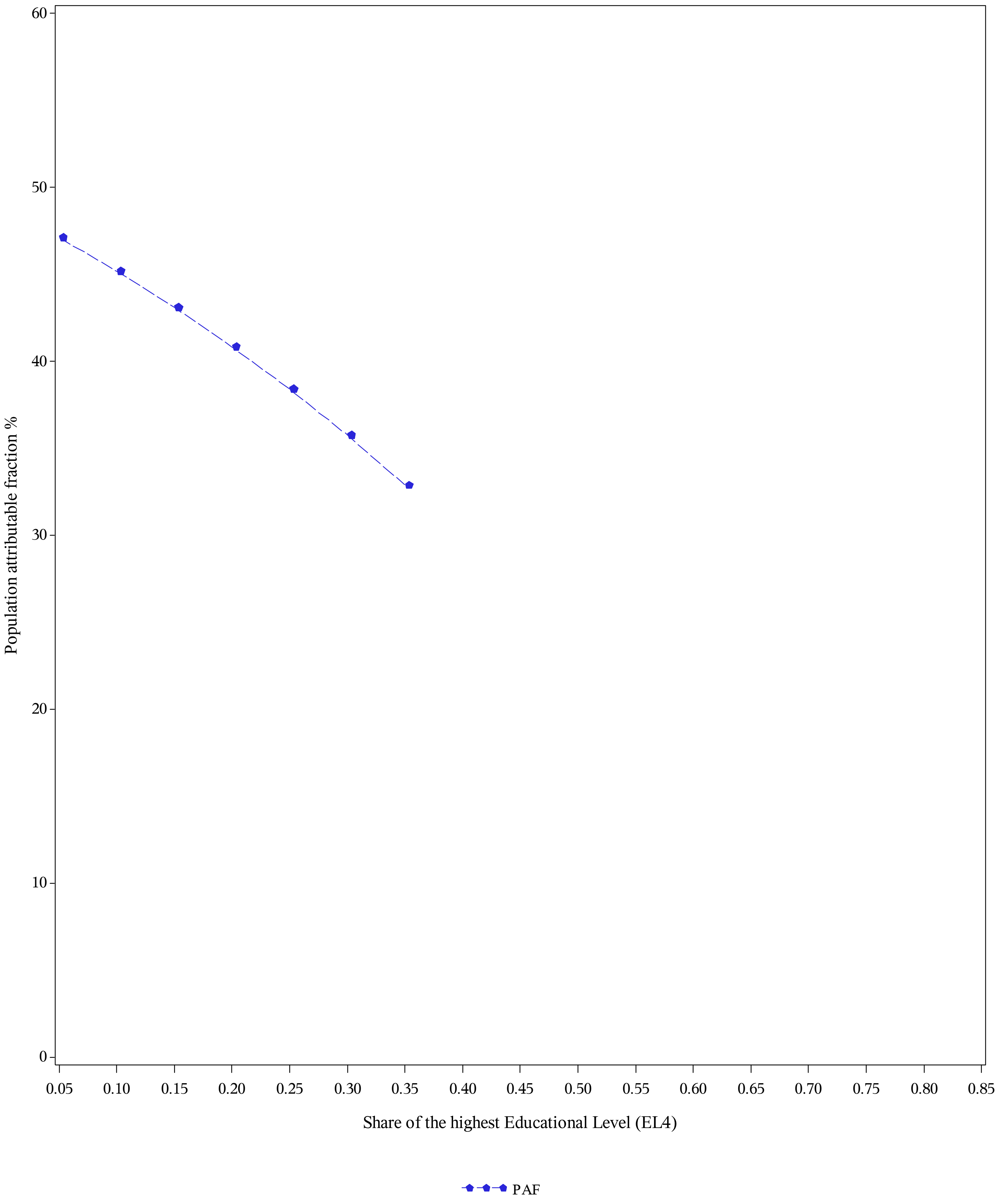

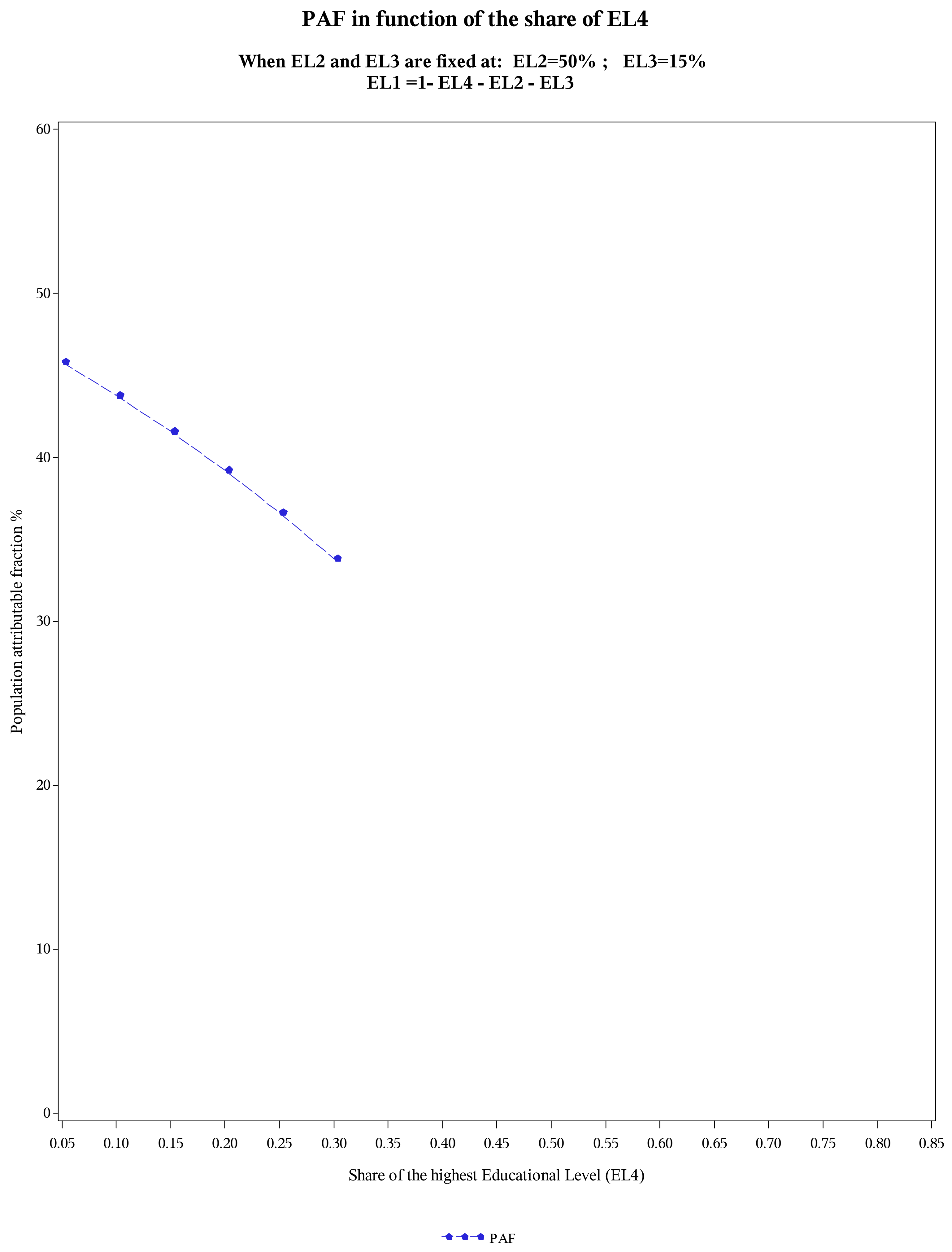

PAF in function of the share of EL4
When EL2 and EL3 are fixed at: EL2=50% ; EL3=15%
EL1 =1- EL4 - EL2 - EL3
Population attributable fraction %
0
10
20
30
40
50
60
0.05
0.10
0.15
0.20
0.25
0.30
0.35
0.40
0.45
0.50
0.55
0.60
0.65
0.70
0.75
0.80
0.85
Share of the highest Educational Level (EL4)
PAF

PAF in function of the share of EL4

When EL2 and EL3 are fixed at: EL2=50% ; EL3=20%
EL1 =1- EL4 - EL2 - EL3

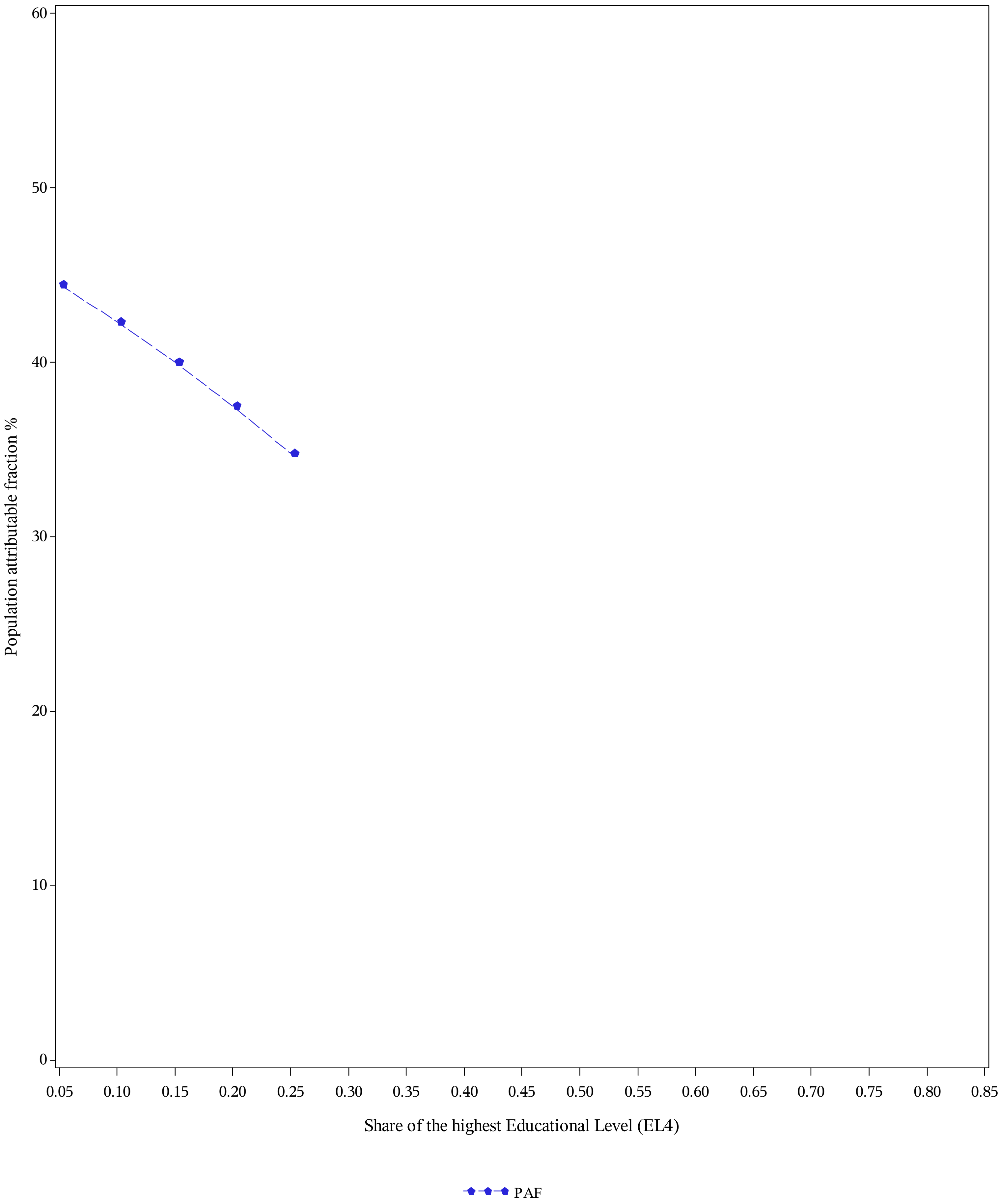

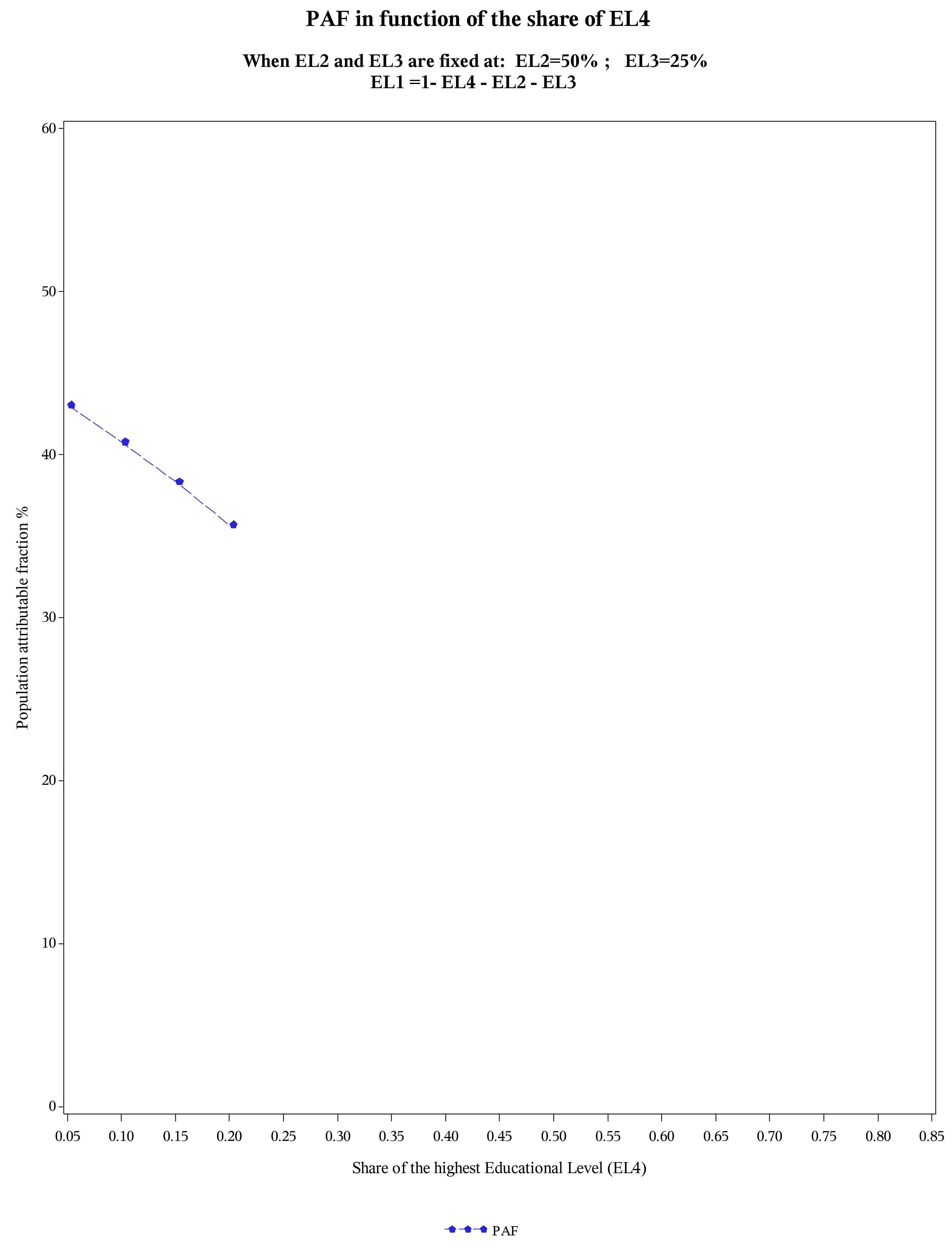

PAF in function of the share of EL4
When EL2 and EL3 are fixed at: EL2=50% ; EL3=25%
EL1 =1- EL4 - EL2 - EL3
Population attributable fraction %
0
10
20
30
40
50
60
0.05
0.10
0.15
0.20
0.25
0.30
0.35
0.40
0.45
0.50
0.55
0.60
0.65
0.70
0.75
0.80
0.85
Share of the highest Educational Level (EL4)
PAF

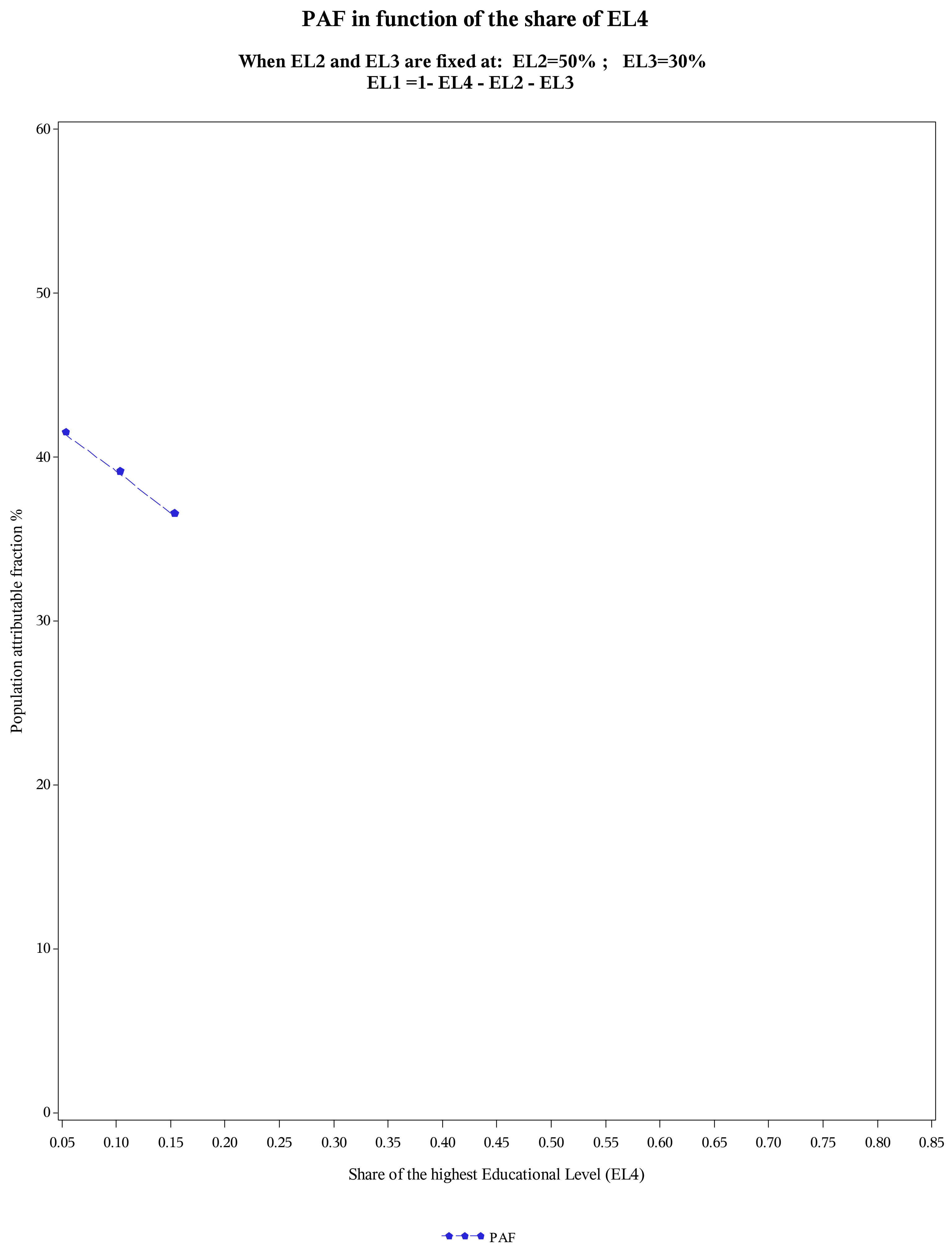
PAF in function of the share of EL4
When EL2 and EL3 are fixed at: EL2=50% ; EL3=30%
EL1 =1- EL4 - EL2 - EL3
Population attributable fraction %
0
10
20
30
40
50
60
0.05
0.10
0.15
0.20
0.25
0.30
0.35
0.40
0.45
0.50
0.55
0.60
0.65
0.70
0.75
0.80
0.85
Share of the highest Educational Level (EL4)
PAF

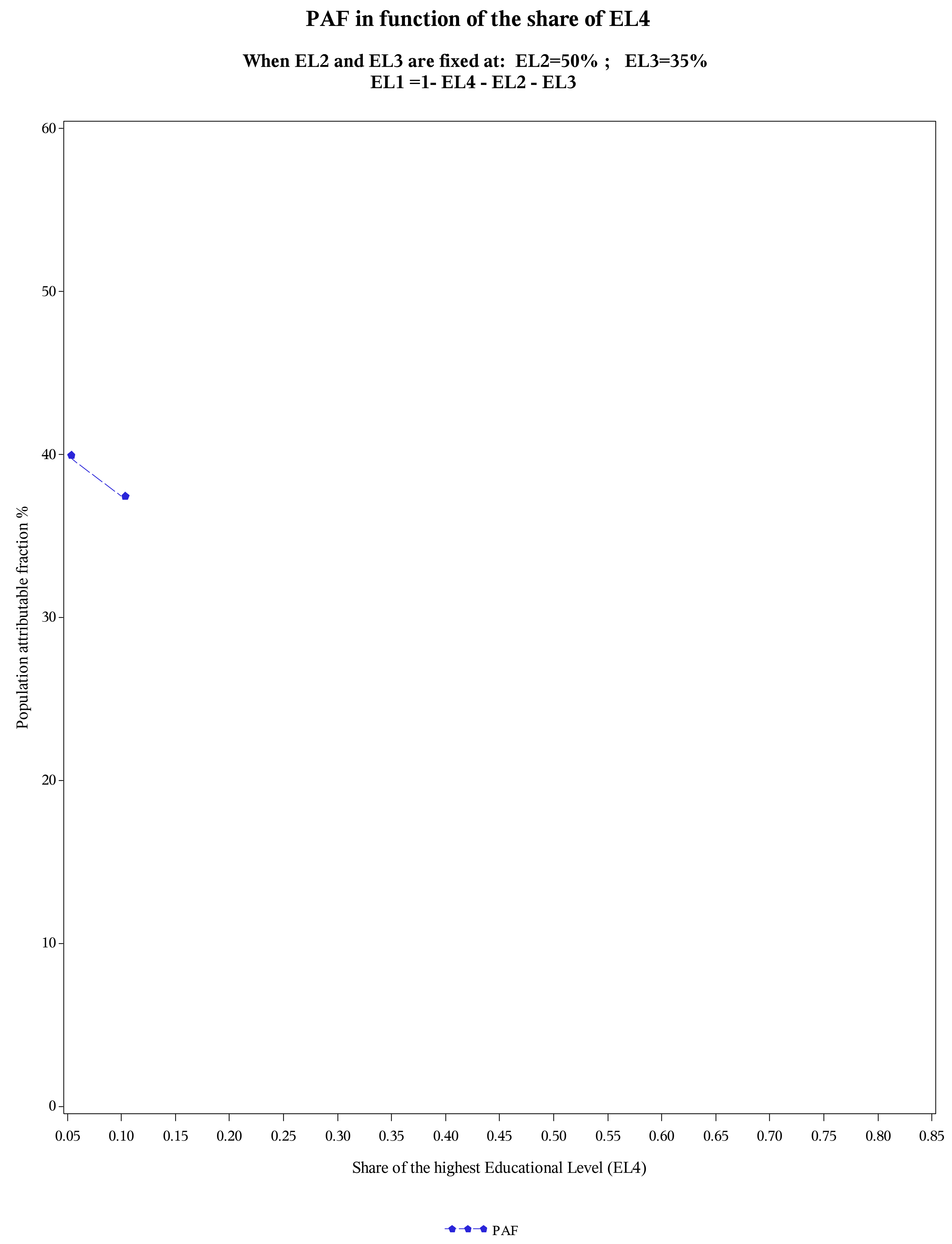
PAF in function of the share of EL4
When EL2 and EL3 are fixed at: EL2=50% ; EL3=35%
EL1 =1- EL4 - EL2 - EL3
Population attributable fraction %
0
10
20
30
40
50
60
0.05
0.10
0.15
0.20
0.25
0.30
0.35
0.40
0.45
0.50
0.55
0.60
0.65
0.70
0.75
0.80
0.85
Share of the highest Educational Level (EL4)
PAF

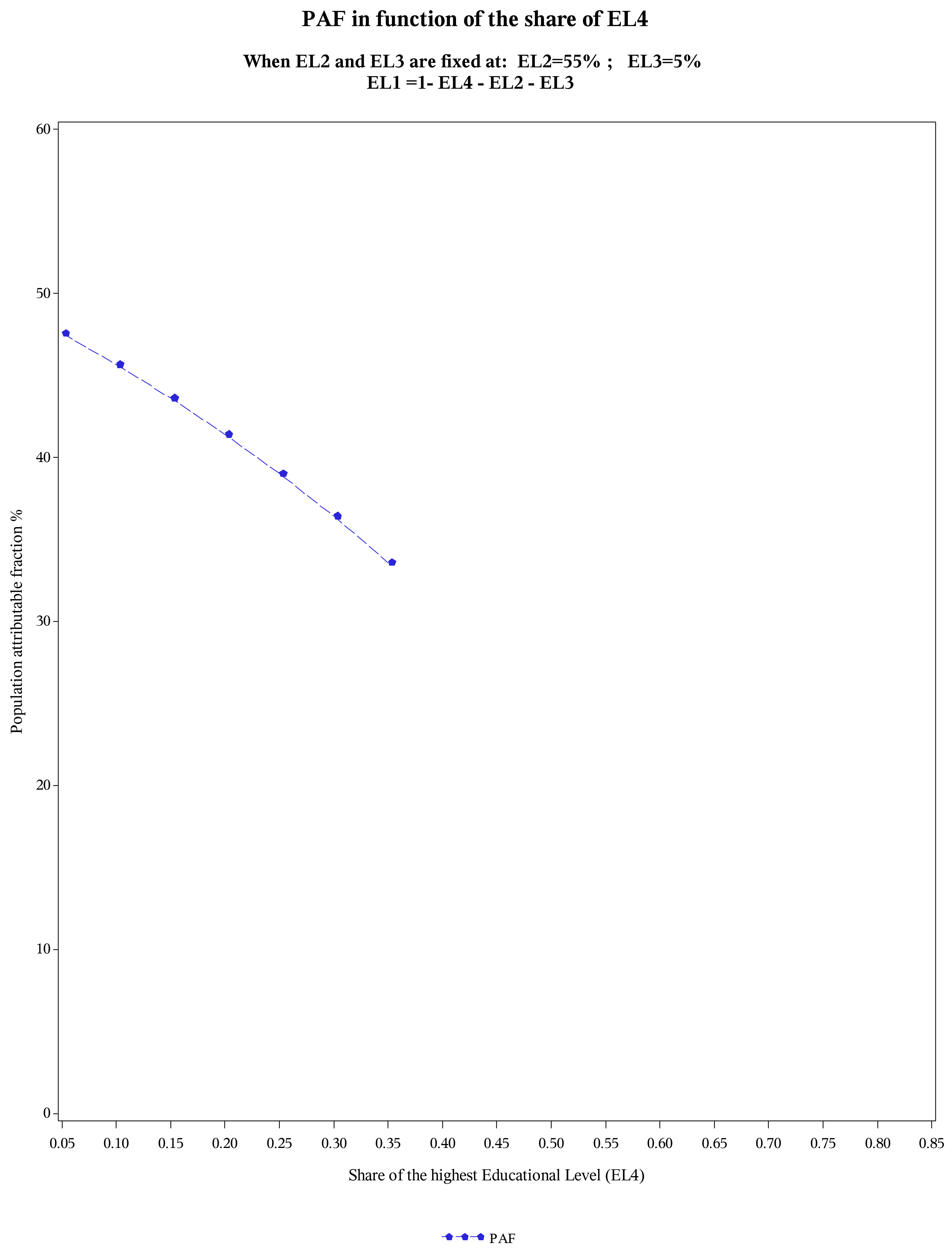

PAF in function of the share of EL4
When EL2 and EL3 are fixed at: EL2=55% ; EL3=5%
EL1 =1- EL4 - EL2 - EL3
Population attributable fraction %
0
10
20
30
40
50
60
0.05
0.10
0.15
0.20
0.25
0.30
0.35
0.40
0.45
0.50
0.55
0.60
0.65
0.70
0.75
0.80
0.85
Share of the highest Educational Level (EL4)
PAF

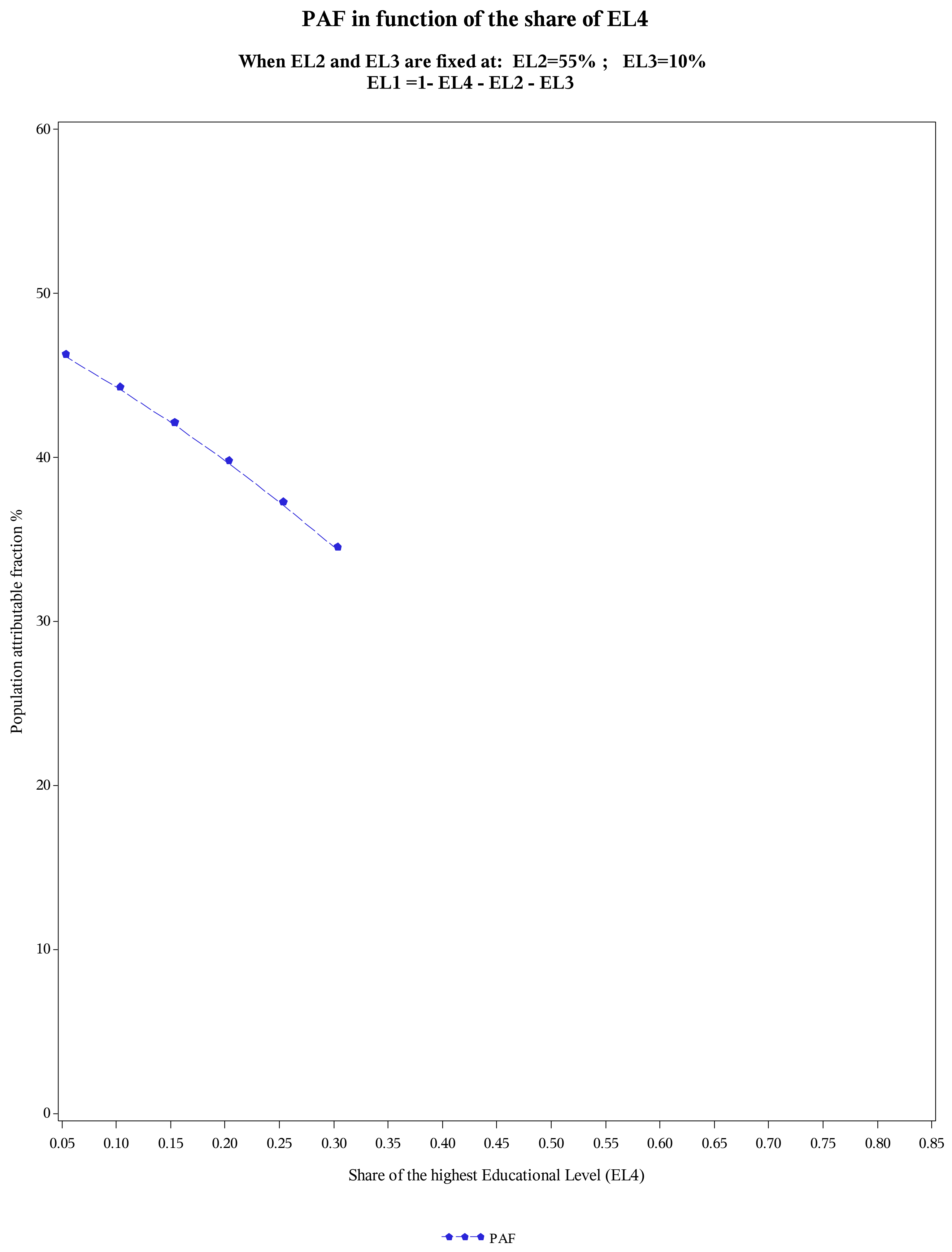

PAF in function of the share of EL4
When EL2 and EL3 are fixed at: EL2=55% ; EL3=10%
EL1 =1- EL4 - EL2 - EL3
Population attributable fraction %
0
10
20
30
40
50
60
0.05
0.10
0.15
0.20
0.25
0.30
0.35
0.40
0.45
0.50
0.55
0.60
0.65
0.70
0.75
0.80
0.85
Share of the highest Educational Level (EL4)
PAF

PAF in function of the share of EL4

When EL2 and EL3 are fixed at: EL2=55% ; EL3=15%
EL1 =1- EL4 - EL2 - EL3

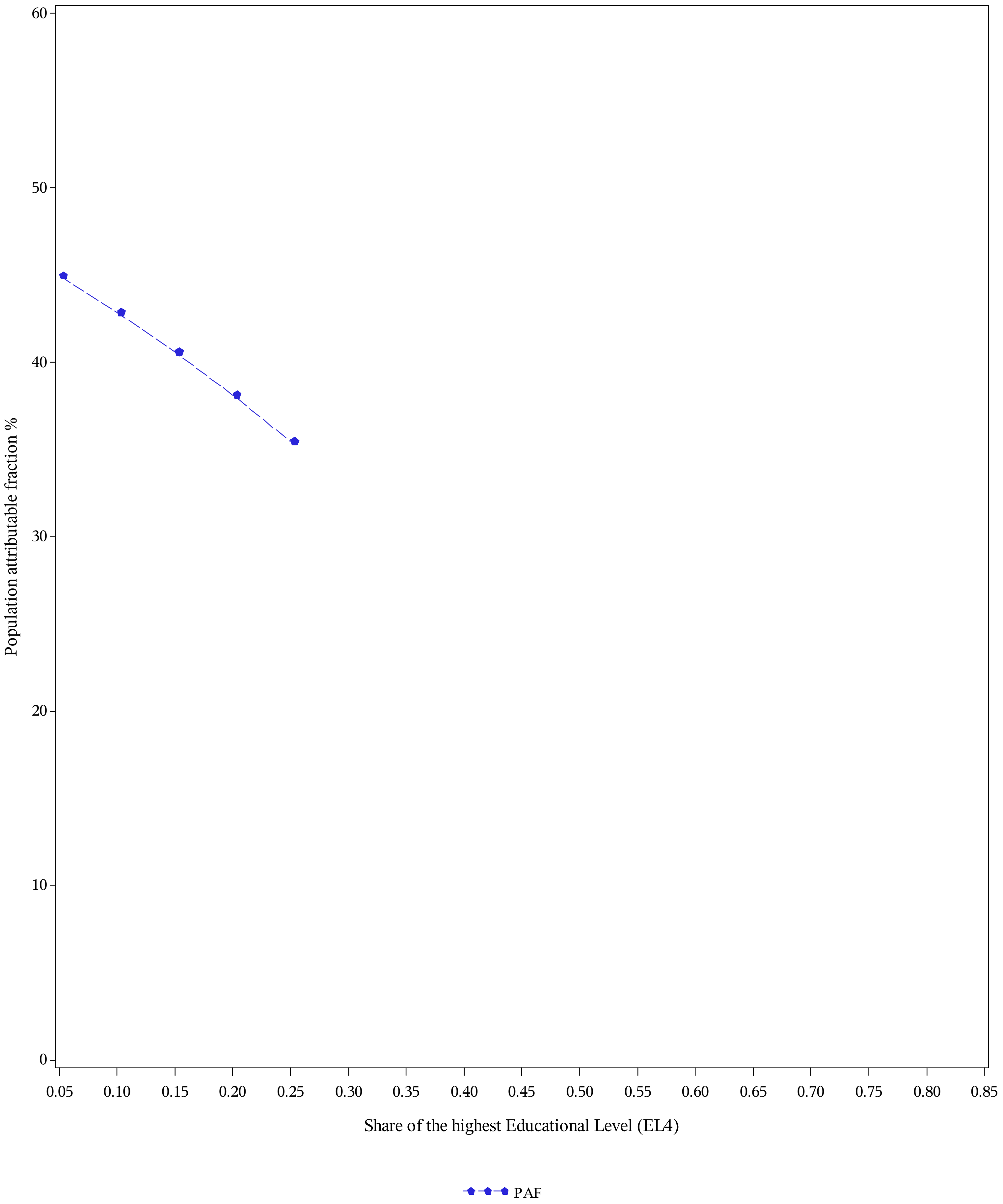

PAF in function of the share of EL4

When EL2 and EL3 are fixed at: EL2=55% ; EL3=20%
EL1 =1- EL4 - EL2 - EL3

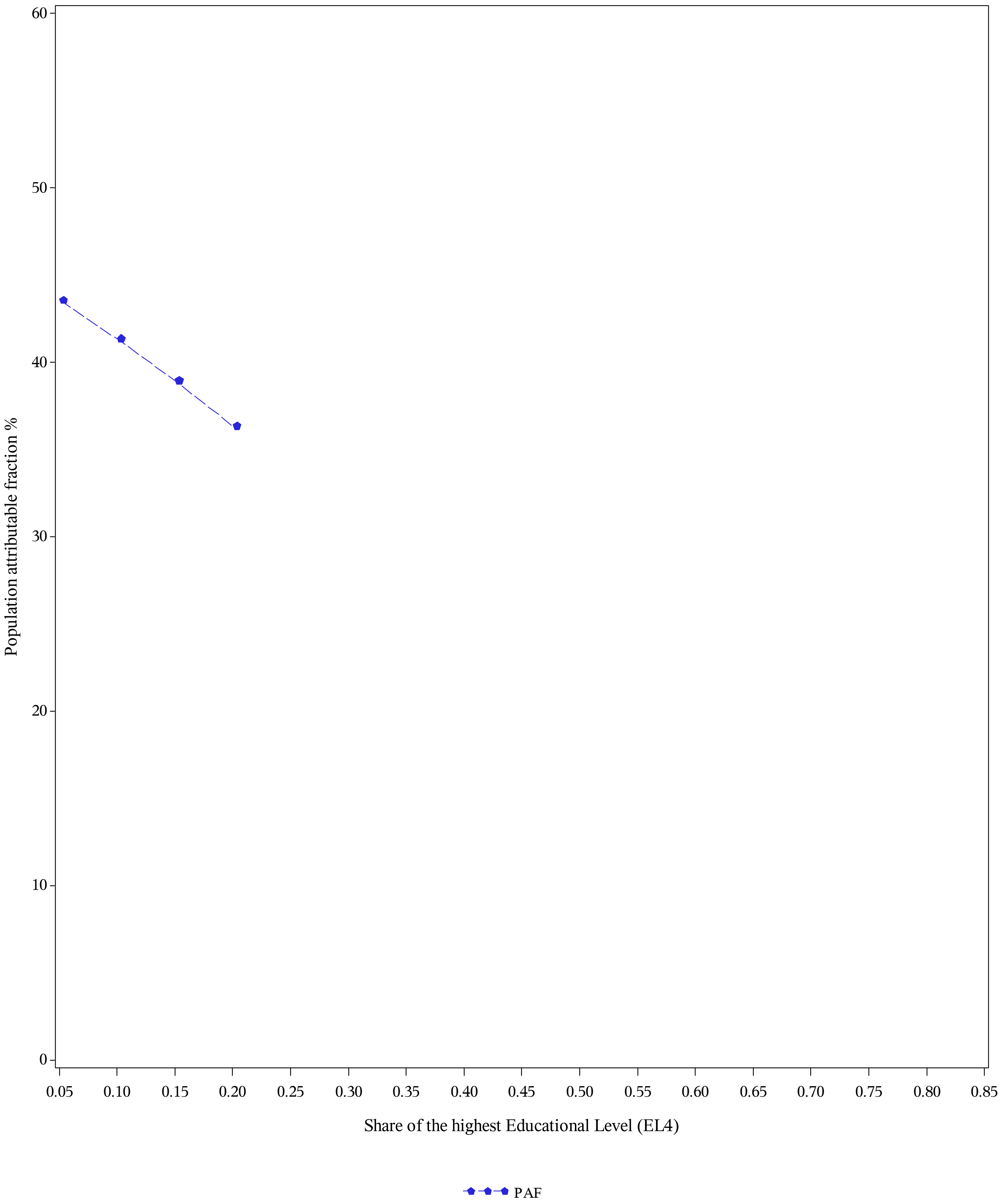

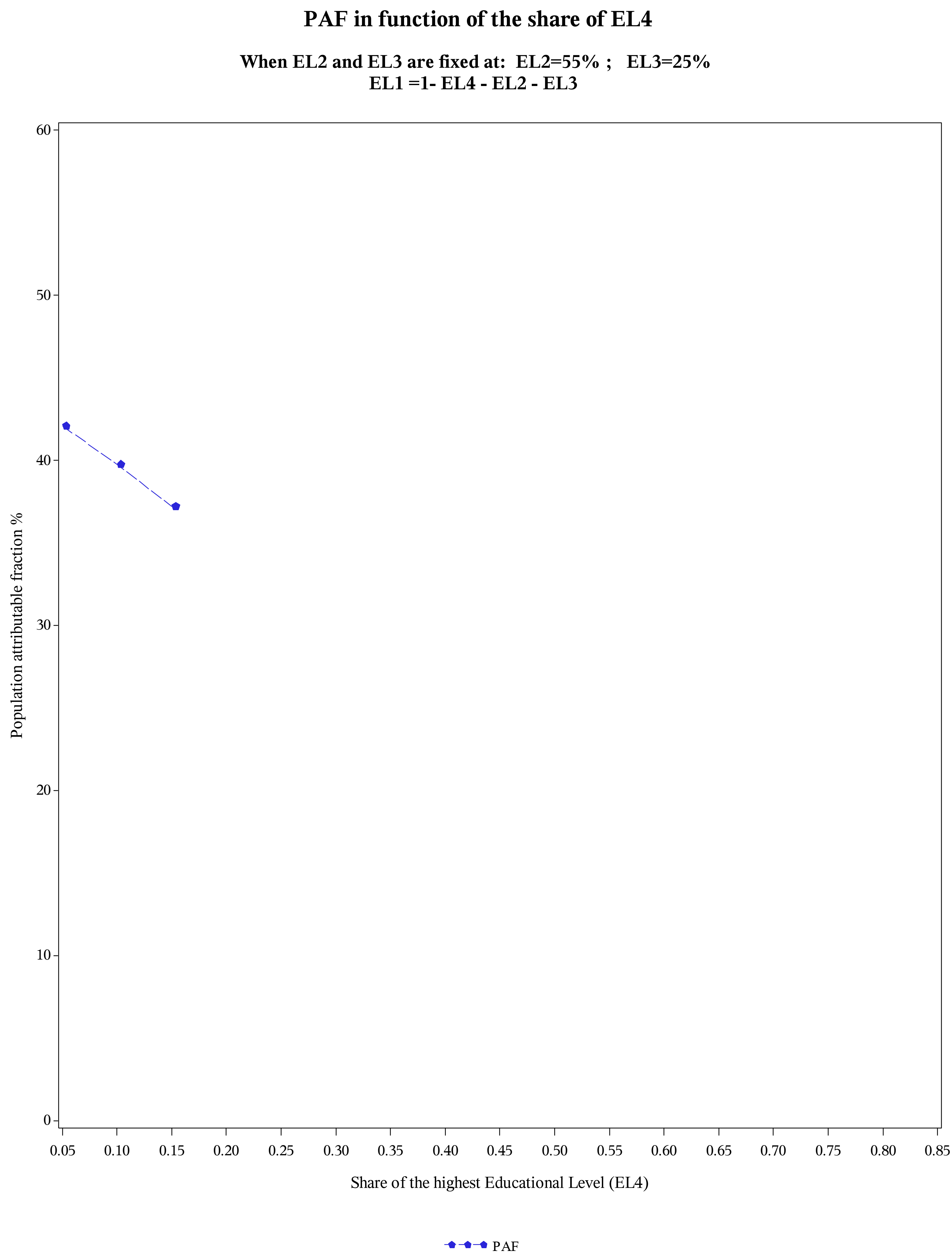
PAF in function of the share of EL4
When EL2 and EL3 are fixed at: EL2=55% ; EL3=25%
EL1 =1- EL4 - EL2 - EL3
Population attributable fraction %
0
10
20
30
40
50
60
0.05
0.10
0.15
0.20
0.25
0.30
0.35
0.40
0.45
0.50
0.55
0.60
0.65
0.70
0.75
0.80
0.85
Share of the highest Educational Level (EL4)
PAF

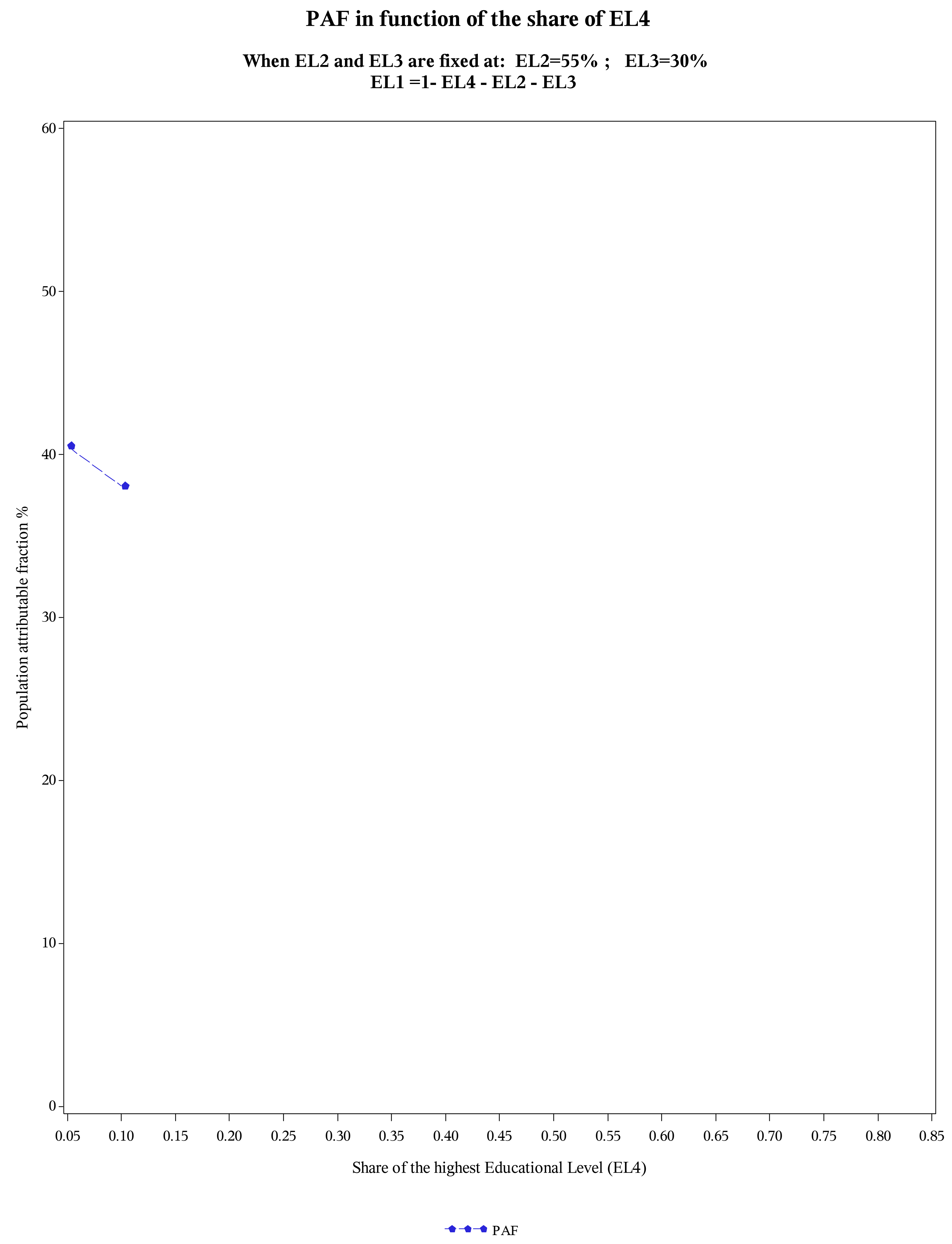

PAF in function of the share of EL4
When EL2 and EL3 are fixed at: EL2=55% ; EL3=30%
EL1 =1- EL4 - EL2 - EL3
Population attributable fraction %
0
10
20
30
40
50
60
0.05
0.10
0.15
0.20
0.25
0.30
0.35
0.40
0.45
0.50
0.55
0.60
0.65
0.70
0.75
0.80
0.85
Share of the highest Educational Level (EL4)
PAF

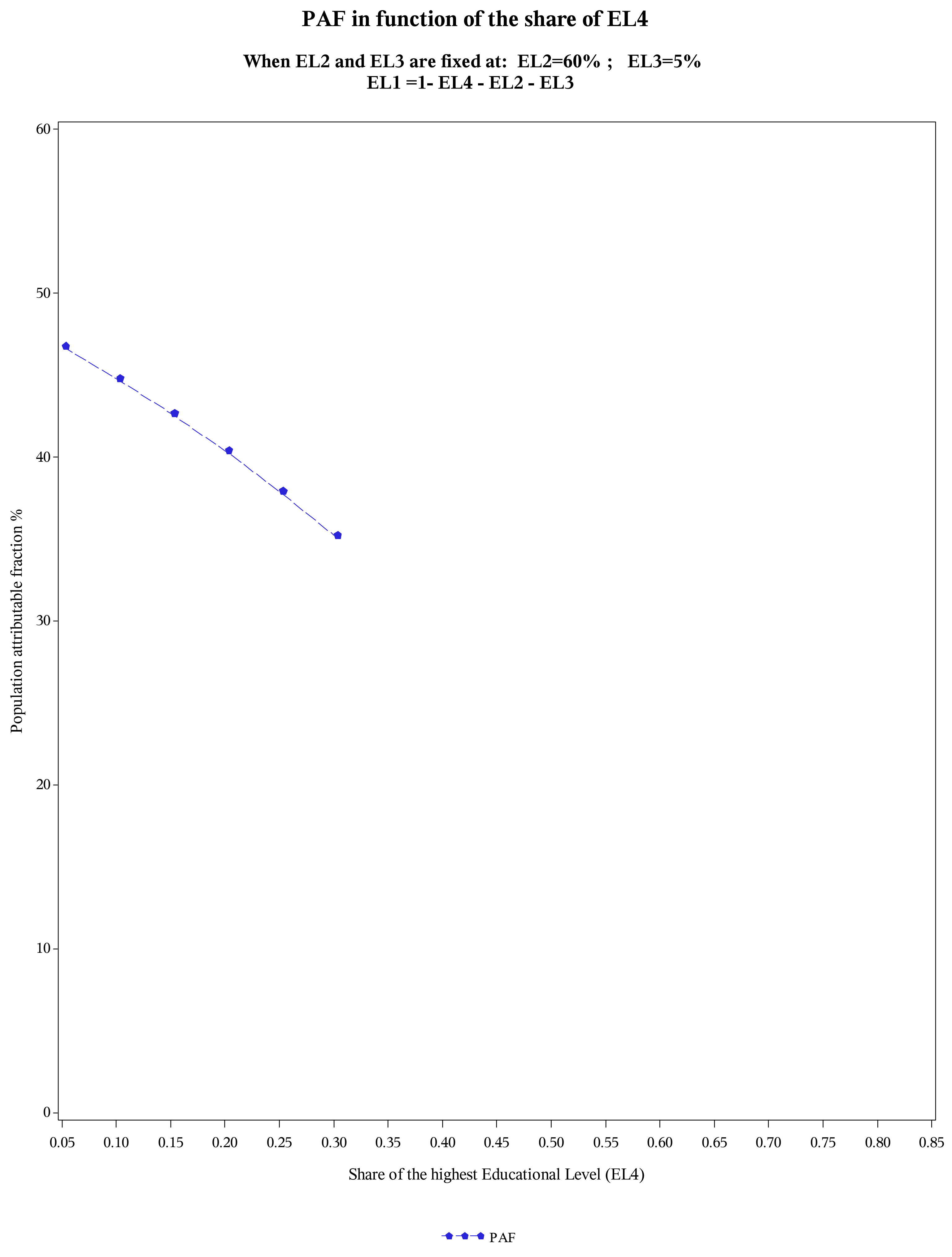
PAF in function of the share of EL4
When EL2 and EL3 are fixed at: EL2=60% ; EL3=5%
EL1 =1- EL4 - EL2 - EL3
Population attributable fraction %
0
10
20
30
40
50
60
0.05
0.10
0.15
0.20
0.25
0.30
0.35
0.40
0.45
0.50
0.55
0.60
0.65
0.70
0.75
0.80
0.85
Share of the highest Educational Level (EL4)
PAF

PAF in function of the share of EL4

When EL2 and EL3 are fixed at: EL2=60% ; EL3=10%
EL1 =1- EL4 - EL2 - EL3

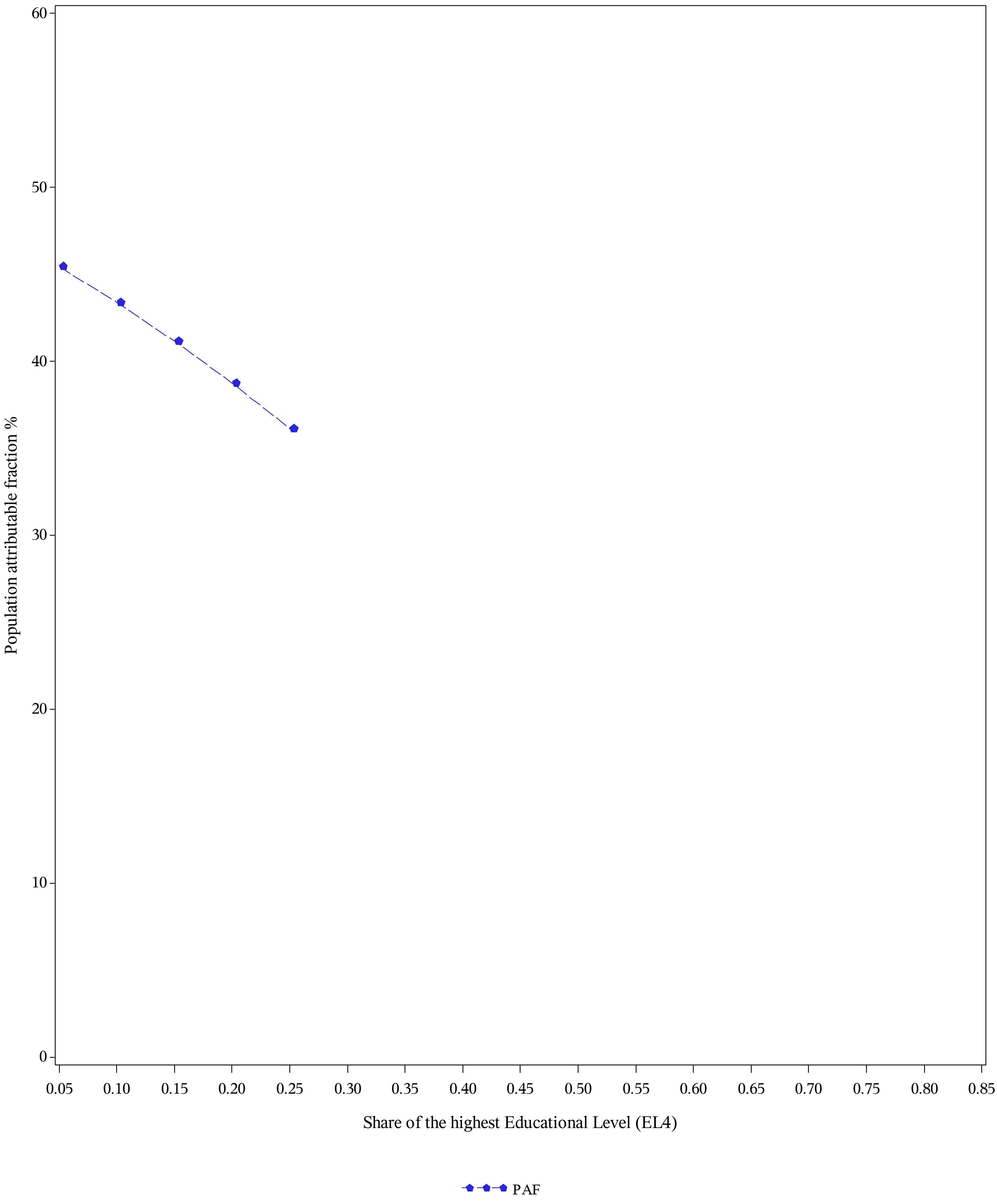

PAF in function of the share of EL4

When EL2 and EL3 are fixed at: EL2=60% ; EL3=15%
EL1 =1- EL4 - EL2 - EL3

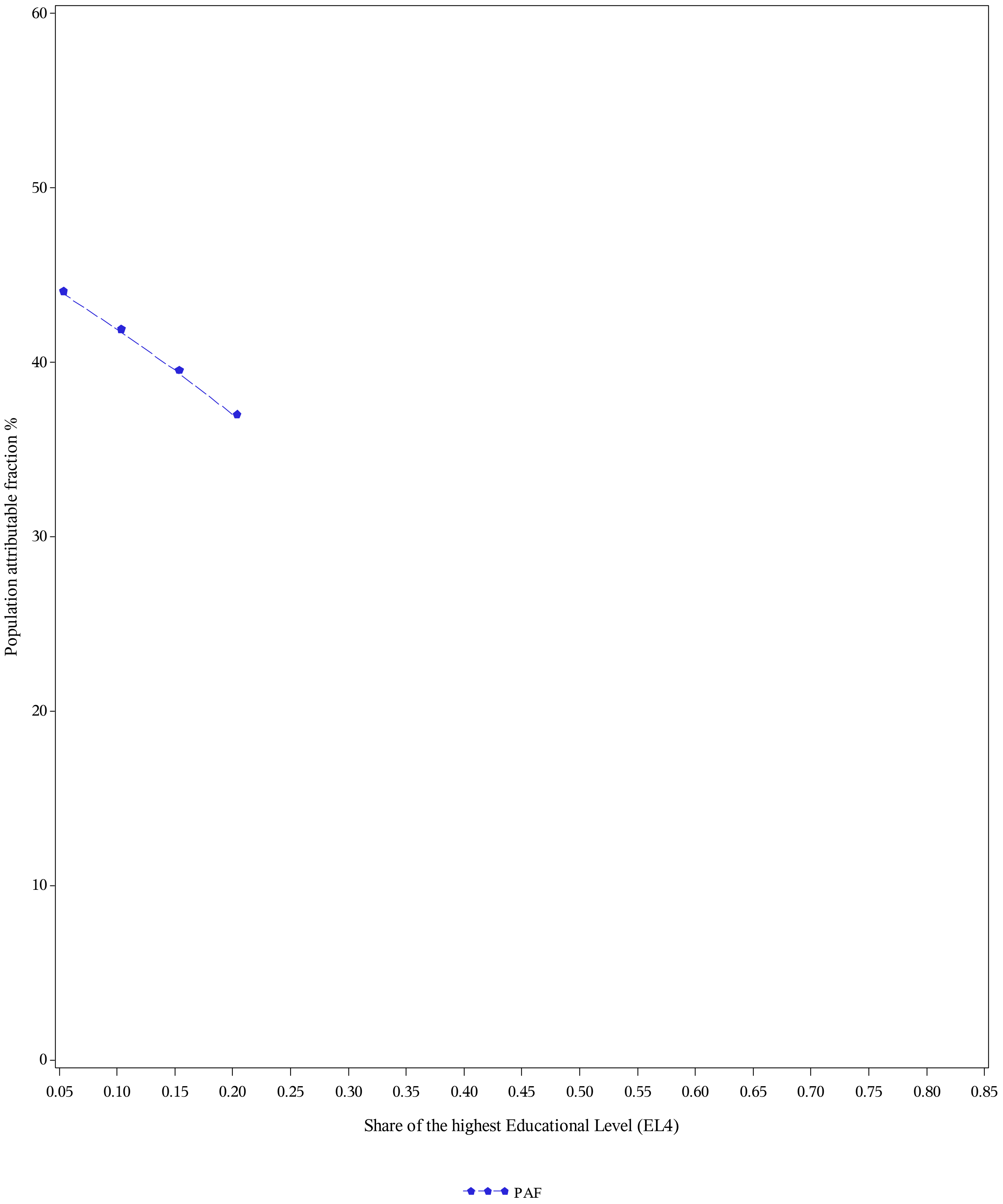

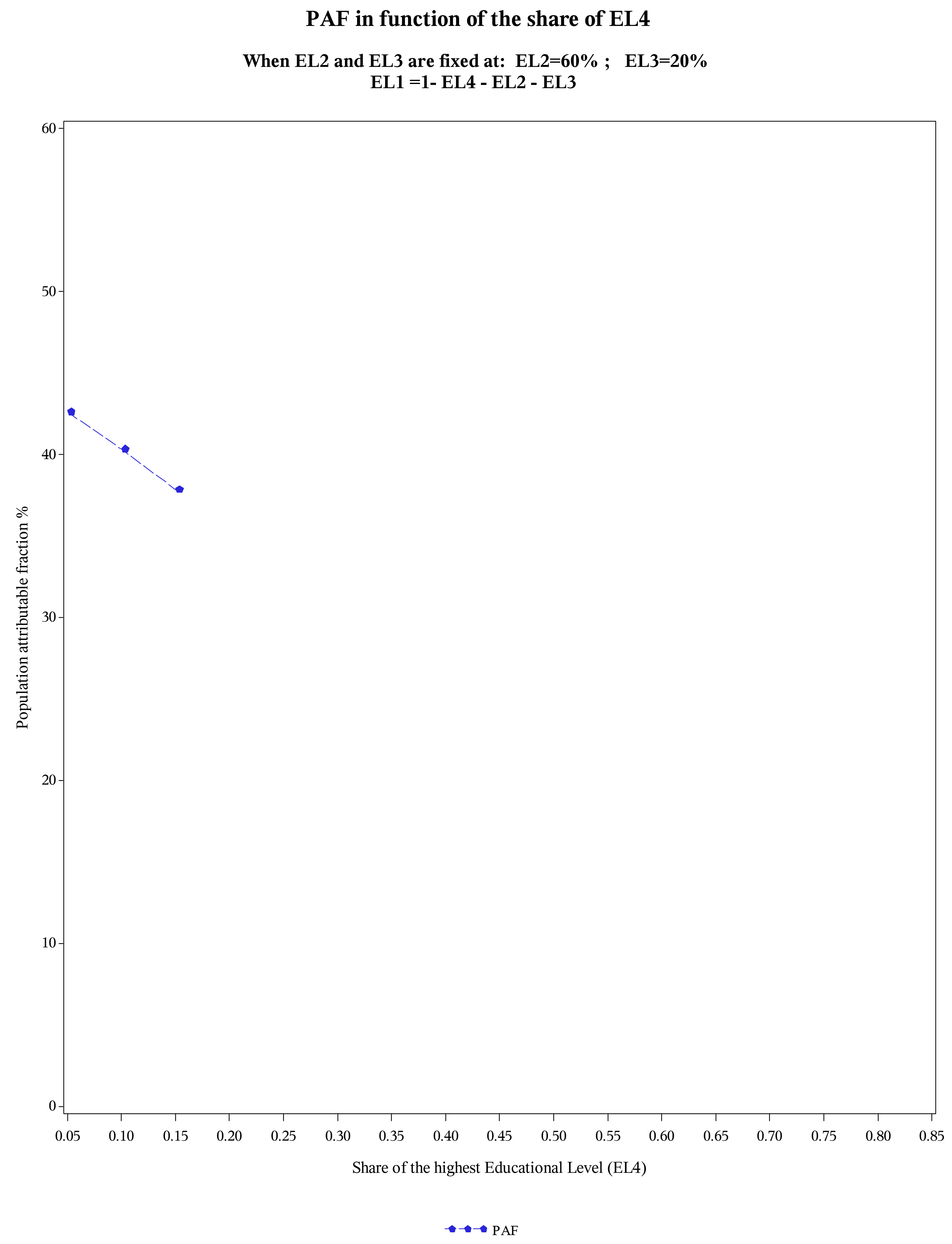
PAF in function of the share of EL4
When EL2 and EL3 are fixed at: EL2=60% ; EL3=20%
EL1 =1- EL4 - EL2 - EL3
Population attributable fraction %
0
10
20
30
40
50
60
0.05
0.10
0.15
0.20
0.25
0.30
0.35
0.40
0.45
0.50
0.55
0.60
0.65
0.70
0.75
0.80
0.85
Share of the highest Educational Level (EL4)
PAF

PAF in function of the share of EL4

When EL2 and EL3 are fixed at: EL2=60% ; EL3=25%
EL1 =1- EL4 - EL2 - EL3

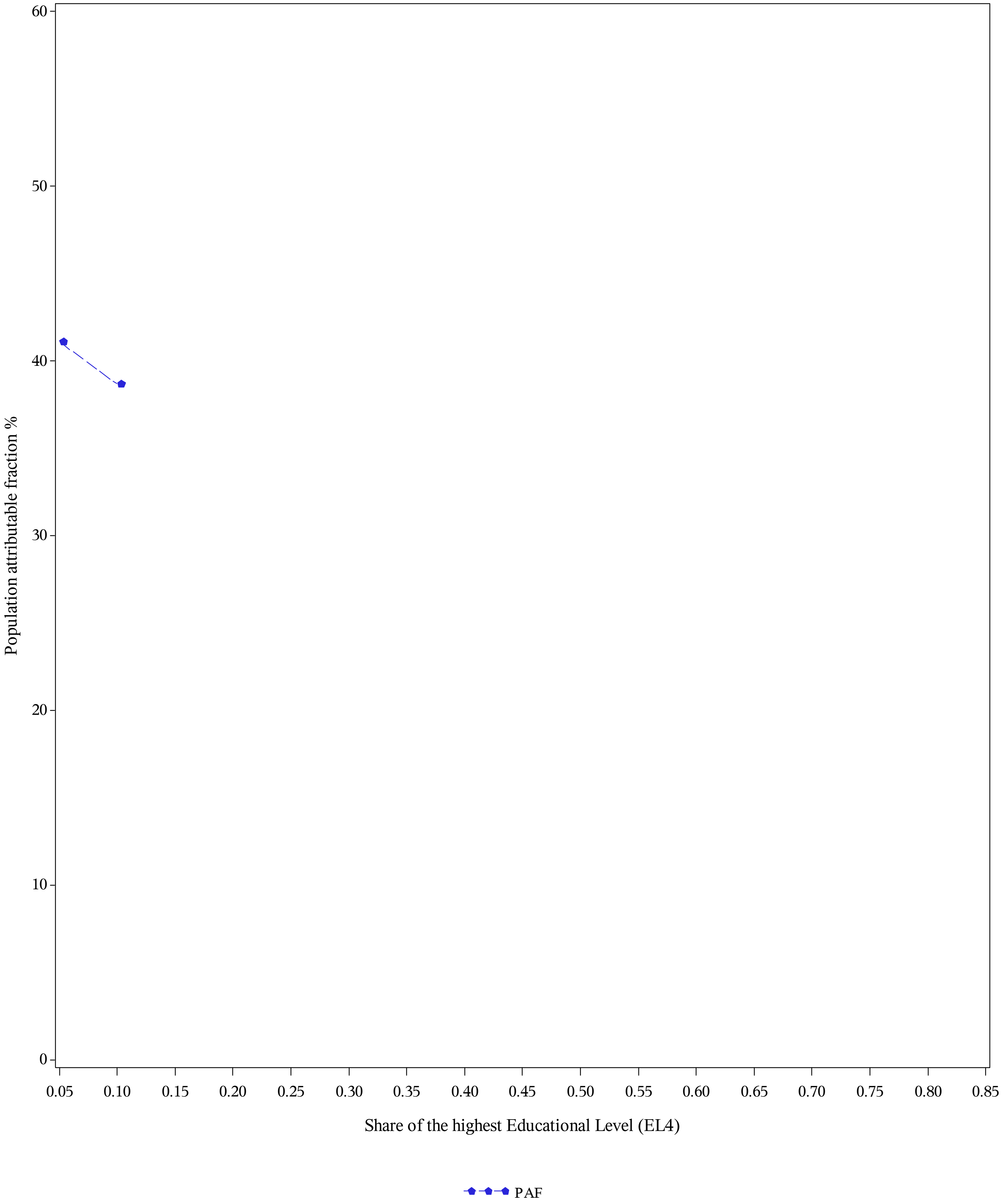

PAF in function of the share of EL4

When EL2 and EL3 are fixed at: EL2=65% ; EL3=5%
EL1 =1- EL4 - EL2 - EL3

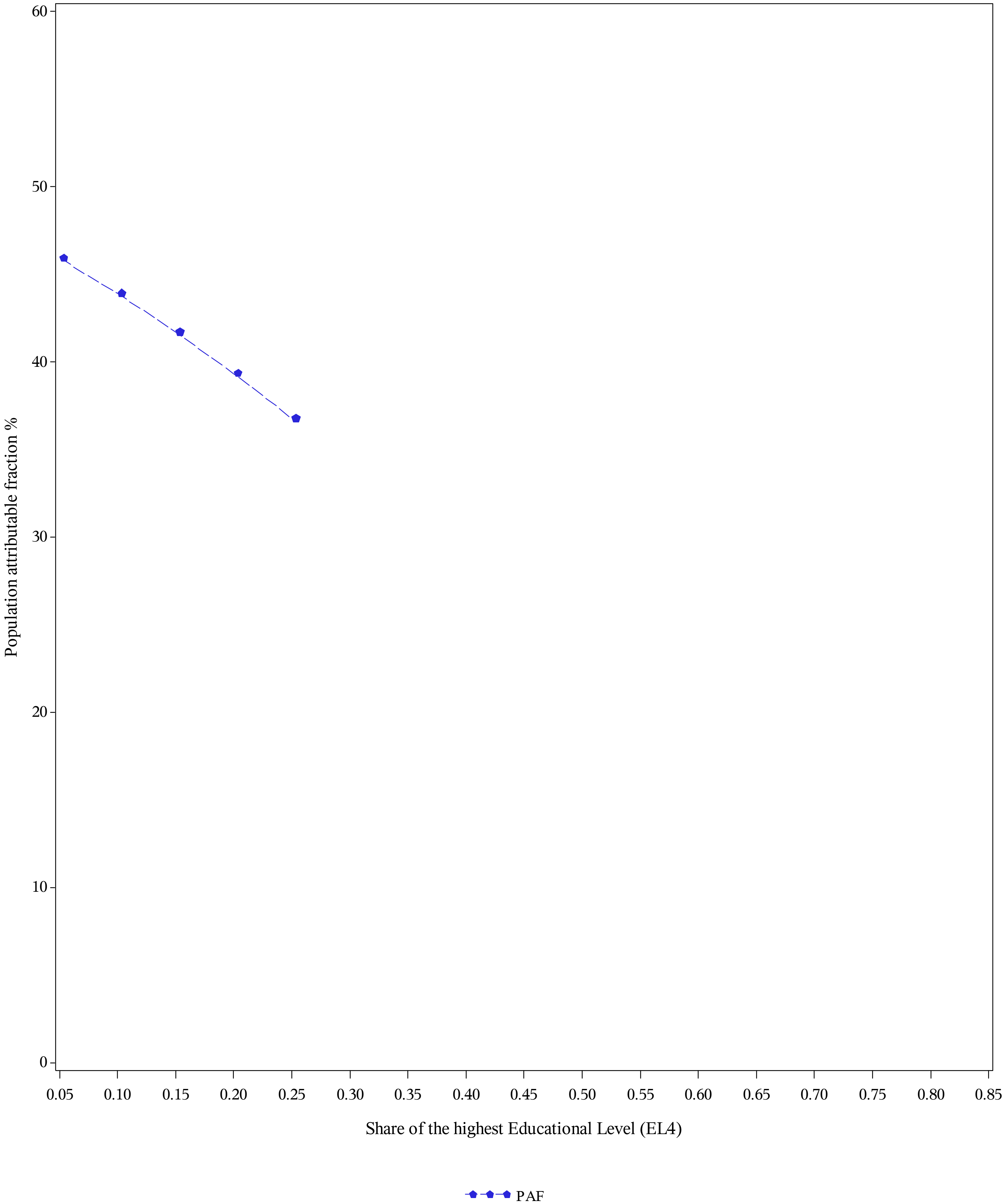

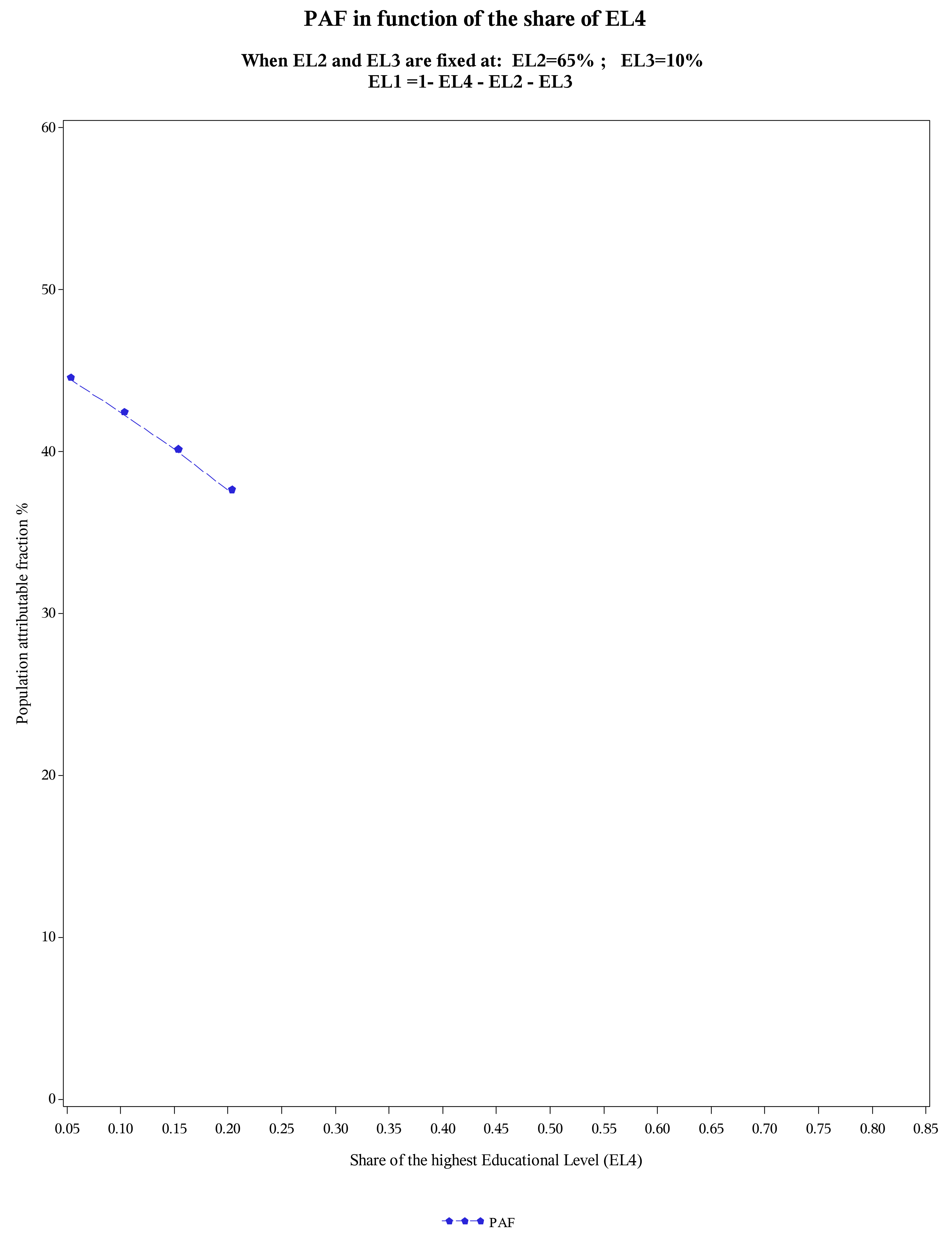
PAF in function of the share of EL4
When EL2 and EL3 are fixed at: EL2=65% ; EL3=10%
EL1 =1- EL4 - EL2 - EL3
Population attributable fraction %
0
10
20
30
40
50
60
0.05
0.10
0.15
0.20
0.25
0.30
0.35
0.40
0.45
0.50
0.55
0.60
0.65
0.70
0.75
0.80
0.85
Share of the highest Educational Level (EL4)
PAF

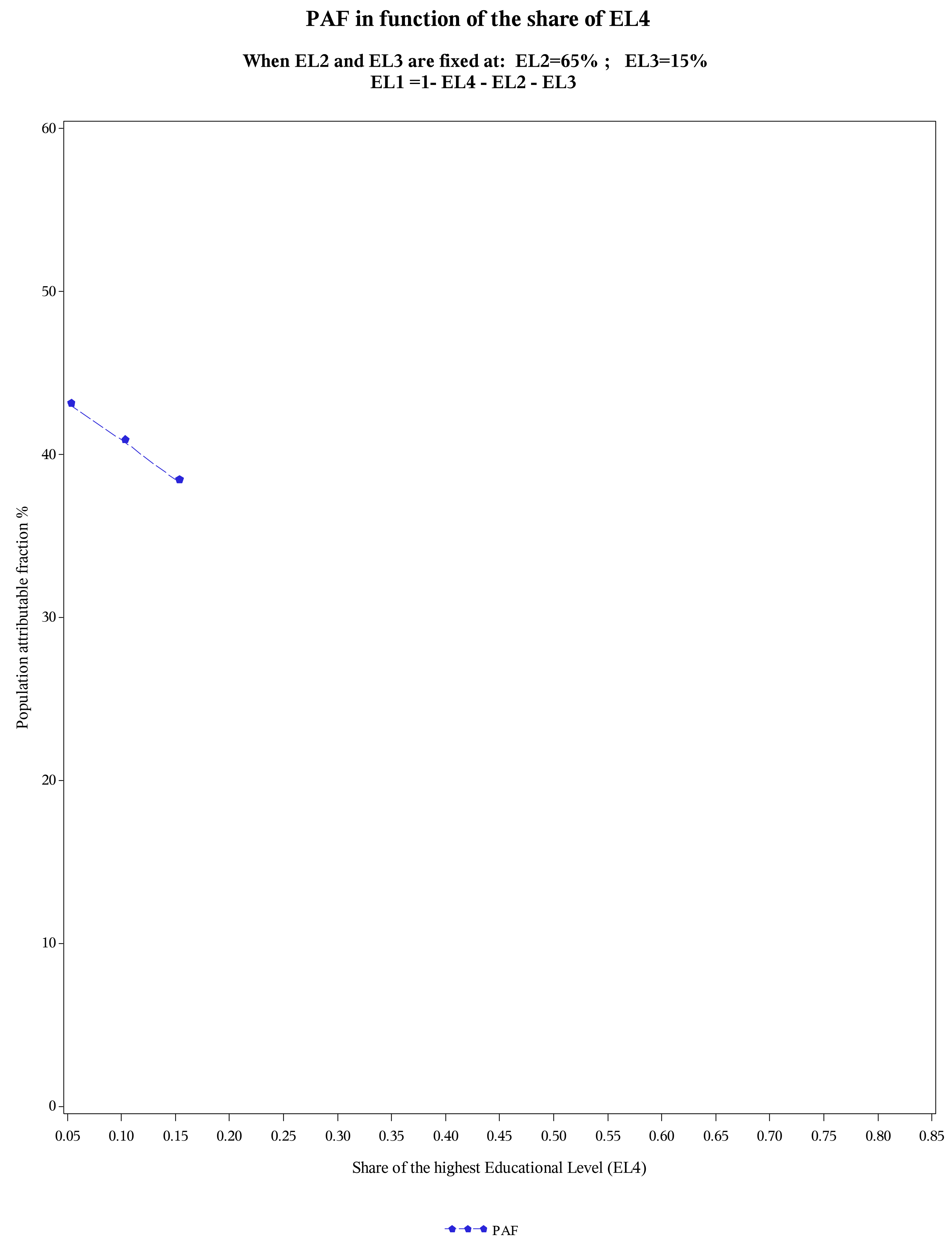
PAF in function of the share of EL4
When EL2 and EL3 are fixed at: EL2=65% ; EL3=15%
EL1 =1- EL4 - EL2 - EL3
Population attributable fraction %
0
10
20
30
40
50
60
0.05
0.10
0.15
0.20
0.25
0.30
0.35
0.40
0.45
0.50
0.55
0.60
0.65
0.70
0.75
0.80
0.85
Share of the highest Educational Level (EL4)
PAF

PAF in function of the share of EL4

When EL2 and EL3 are fixed at: EL2=65% ; EL3=20%
EL1 =1- EL4 - EL2 - EL3

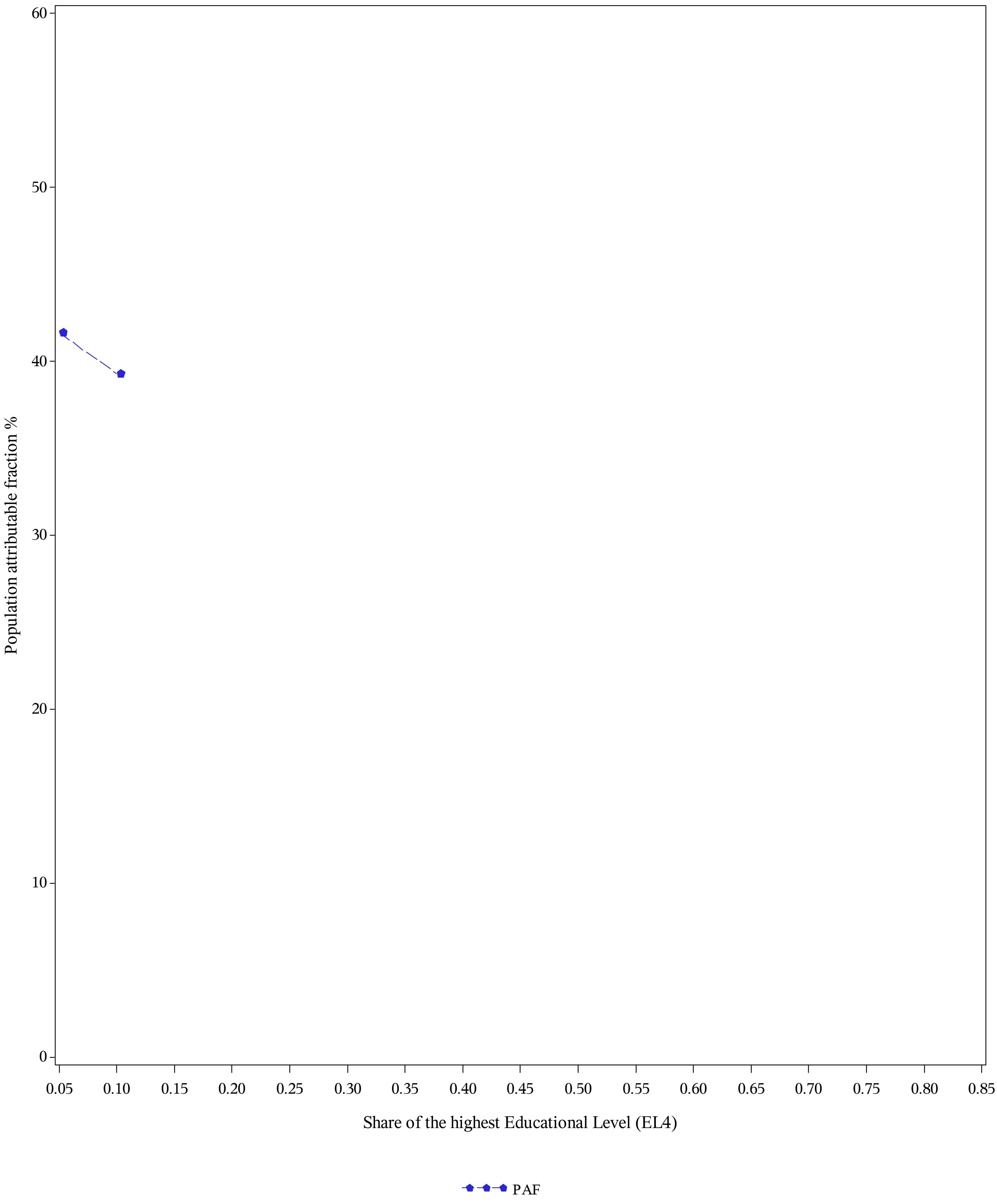

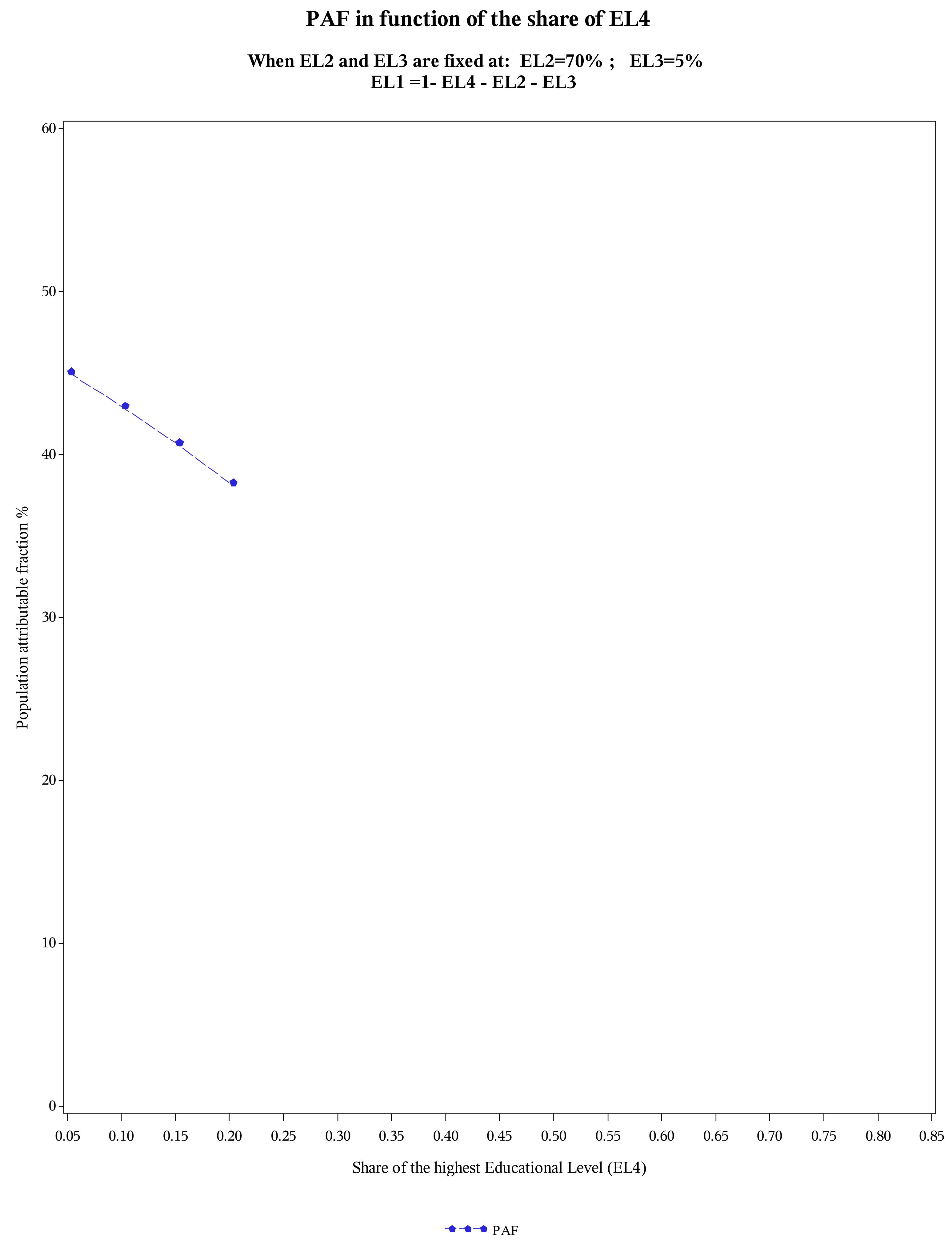
PAF in function of the share of EL4
When EL2 and EL3 are fixed at: EL2=70% ; EL3=5%
EL1 =1- EL4 - EL2 - EL3
Population attributable fraction %
0
10
20
30
40
50
60
0.05
0.10
0.15
0.20
0.25
0.30
0.35
0.40
0.45
0.50
0.55
0.60
0.65
0.70
0.75
0.80
0.85
Share of the highest Educational Level (EL4)
PAF

PAF in function of the share of EL4

When EL2 and EL3 are fixed at: EL2=70% ; EL3=10%
EL1 =1- EL4 - EL2 - EL3

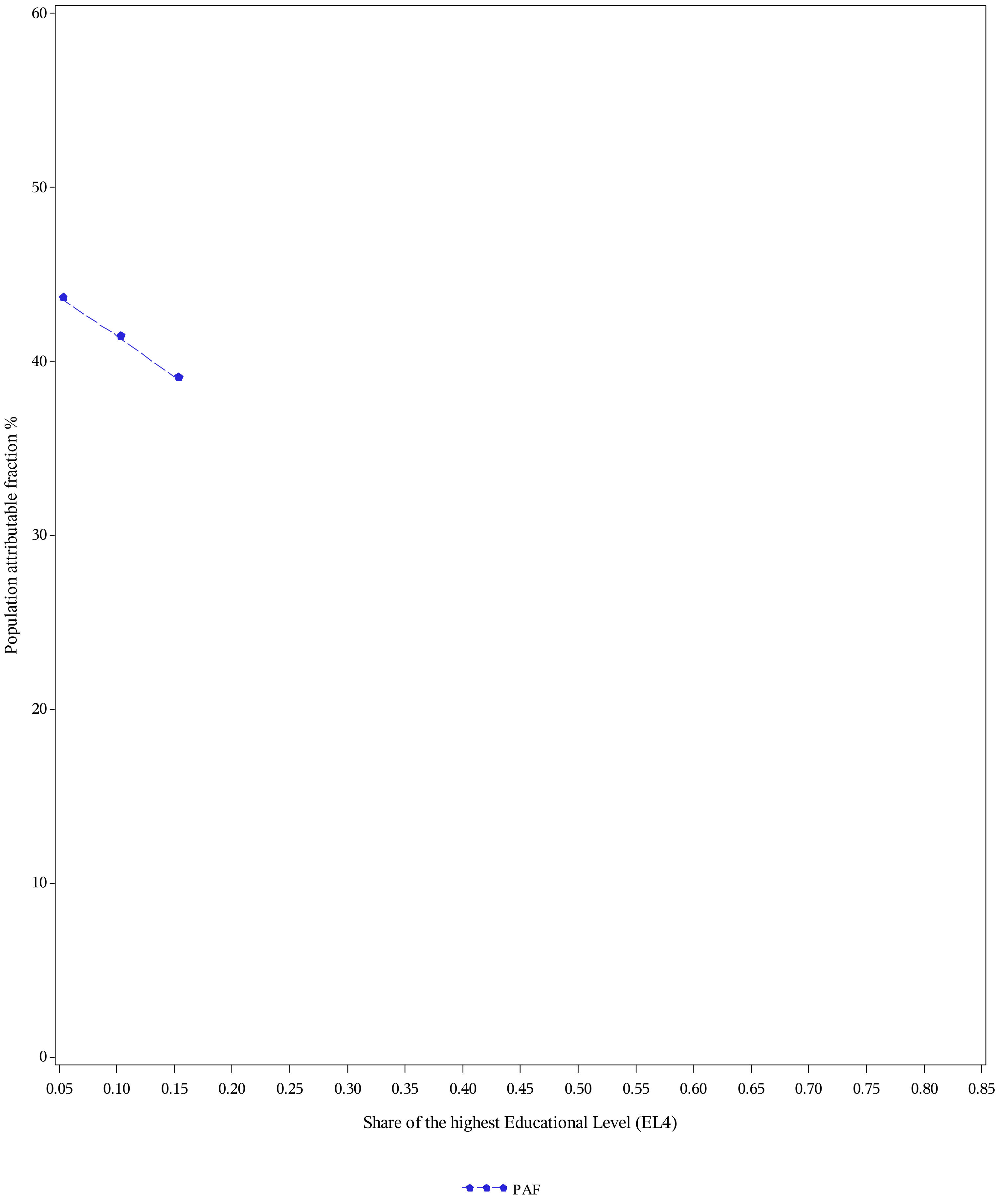

PAF in function of the share of EL4

When EL2 and EL3 are fixed at: EL2=70% ; EL3=15%
EL1 =1- EL4 - EL2 - EL3

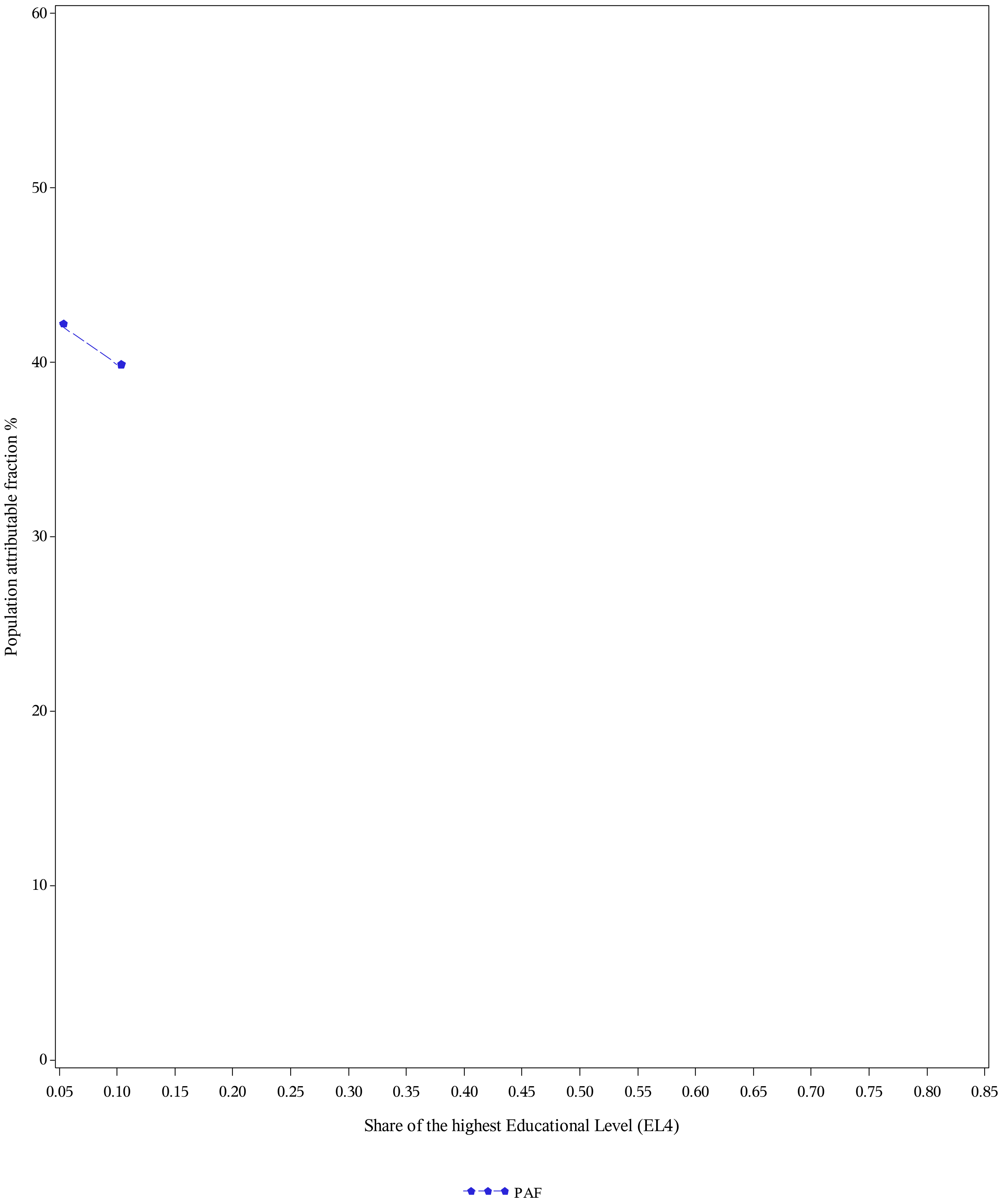

PAF in function of the share of EL4

When EL2 and EL3 are fixed at: EL2=75% ; EL3=5%
EL1 =1- EL4 - EL2 - EL3

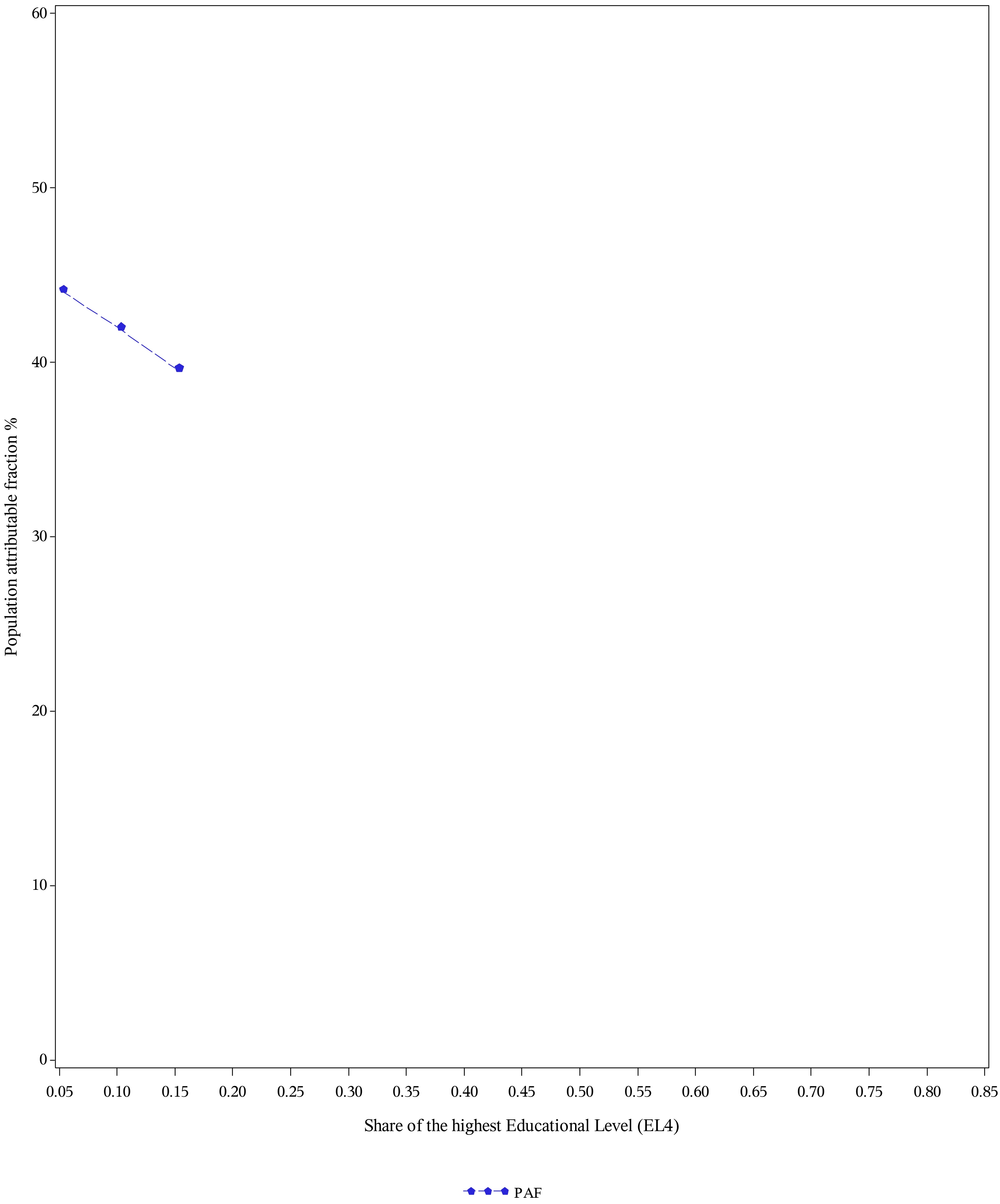

PAF in function of the share of EL4

When EL2 and EL3 are fixed at: EL2=75% ; EL3=10%
EL1 =1- EL4 - EL2 - EL3

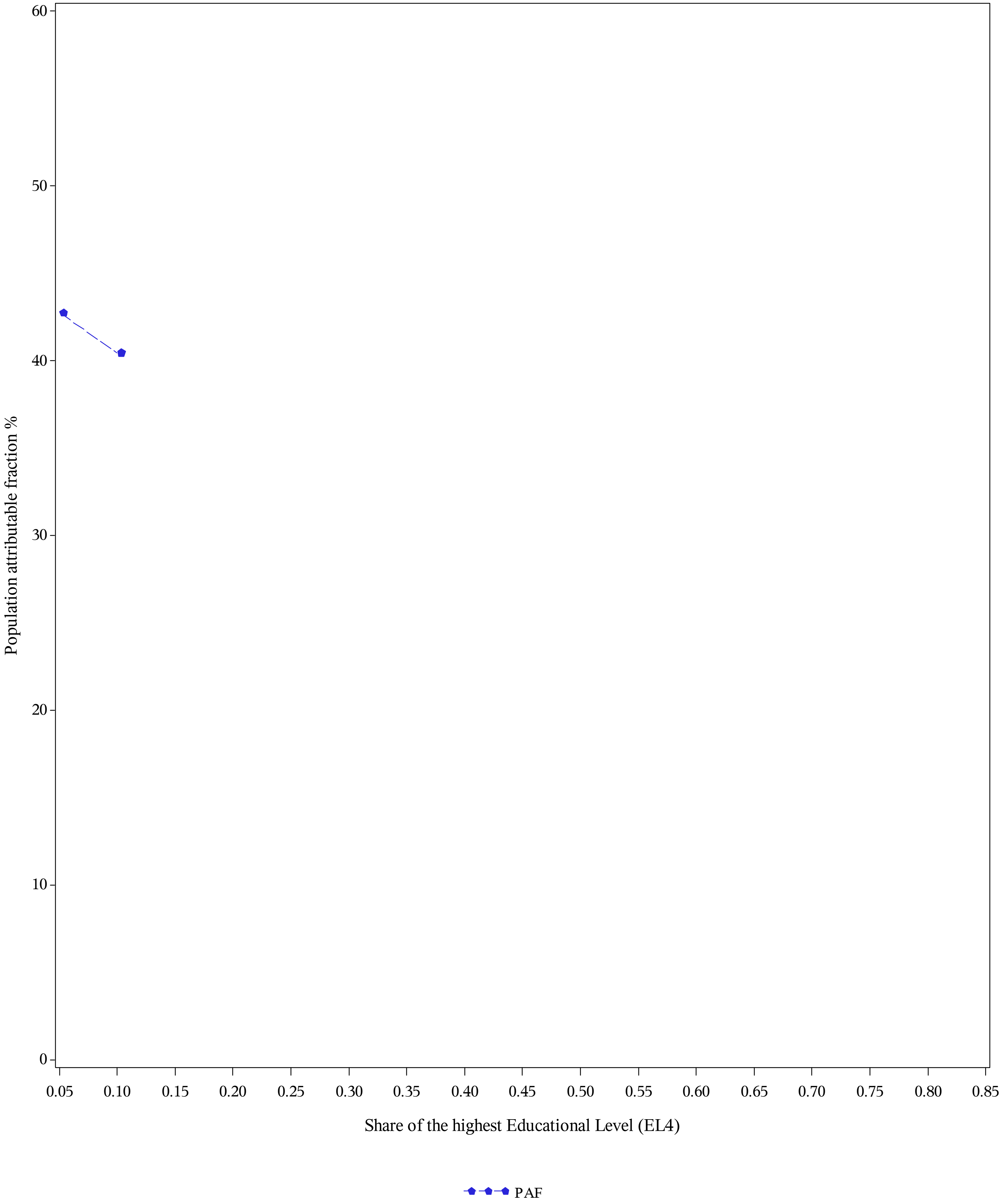

PAF in function of the share of EL4

When EL2 and EL3 are fixed at: EL2=80% ; EL3=5%
EL1 =1- EL4 - EL2 - EL3

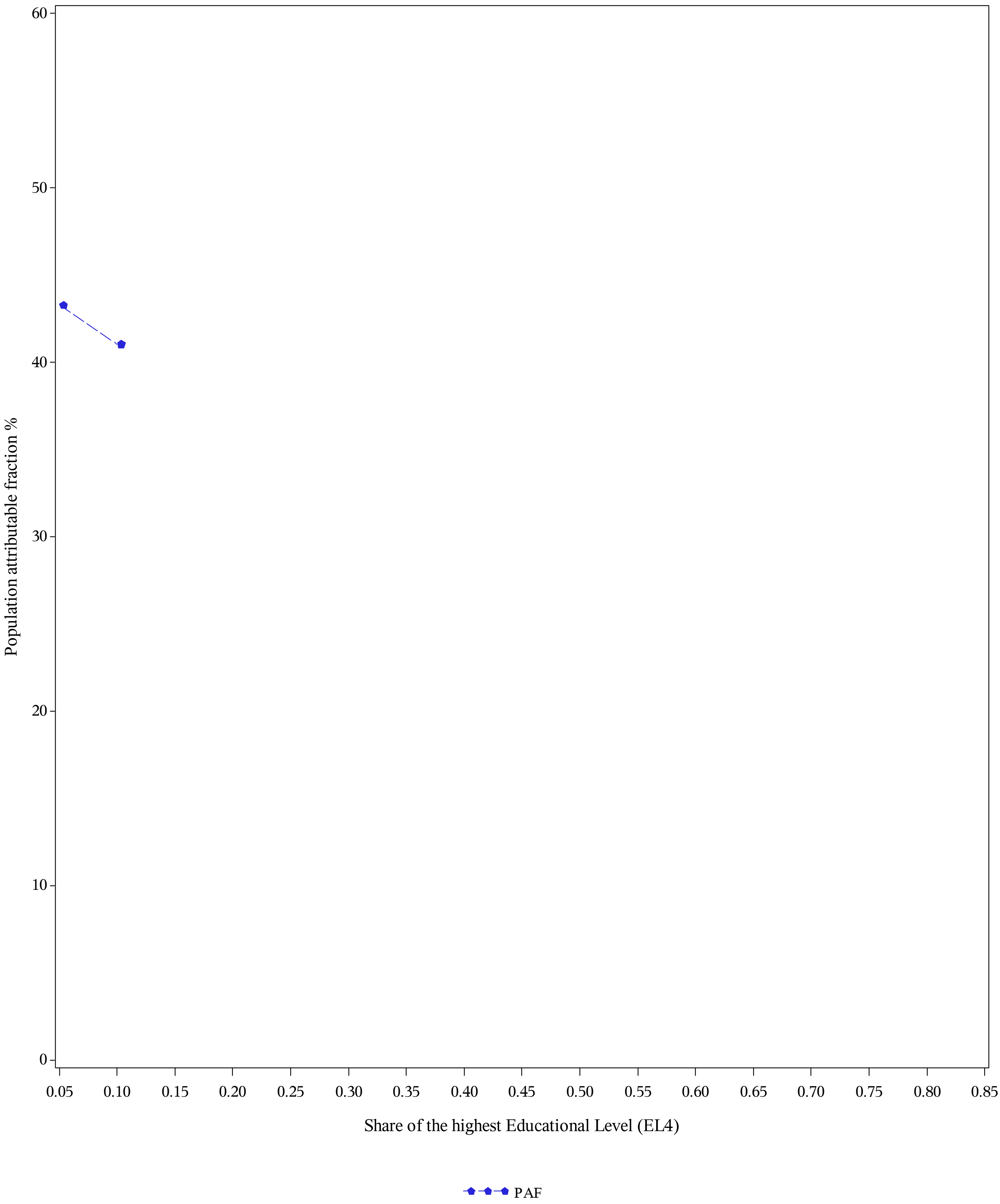

Supplement: Supplementary file 9 — Full set of figures representing the evolution of the PAF in function of P4 at fixed p2 and p3 (PDF 484 kb) [file 12889_2019_6980_MOESM9_ESM.pdf]
